# Total Synthesis of Conjugation-Ready Sulfated Red Algae Carrageenan Oligosaccharides for Sensing Applications

**DOI:** 10.1021/jacs.6c09827

**Published:** 2026-06-10

**Authors:** Yonatan Sukhran, Roey G. Meir, Israel Alshanski, Shlomo Yitzchaik, Mattan Hurevich

**Affiliations:** Institute of Chemistry and Center for Nanoscience and Nanotechnology, The Hebrew University of Jerusalem, Safra Campus, Givat Ram, Jerusalem 9190401, Israel

## Abstract

Carrageenans are versatile sulfated marine galactans that possess attractive modification-dependent bulk properties, making them prime candidates for various cosmetic, drug delivery, and food-related applications. The structural diversity and intrinsic complexity of carrageenans hamper access to homogeneous polysaccharides, limiting the development of many carrageenan-based applications. We devised a synthetic strategy for the acquisition of a panel of γ-carrageenan-derived oligosaccharides with varying sulfation profiles and chain length. To that end, we synthesized a set of specialized building blocks with an elaborate multilevel protecting group hierarchy, tailor-made to specifically accommodate the structural complexity of carrageenans. In doing so, we uncovered interdependent protecting group and reactivity constraints, which we resolved strategically to adapt the synthetic route across the panel of carrageenans and minimize trade-offs. Assembly of di-, tri-, and tetrasaccharides with precise control over monomer connectivity and regiodefined sulfation on a conjugation-ready linker showcased the first total synthesis of homogeneous carrageenan oligogalactans. We demonstrated that the application of the curated panel enabled elucidation of the effect of γ-carrageenan molecular features on IL-8 binding preferences via electrochemical sensing and surface analyses.

## Graphical abstract

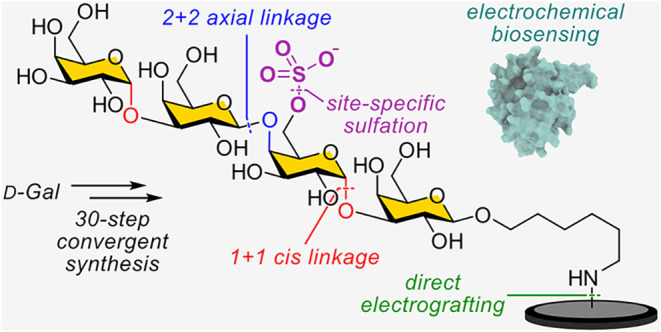

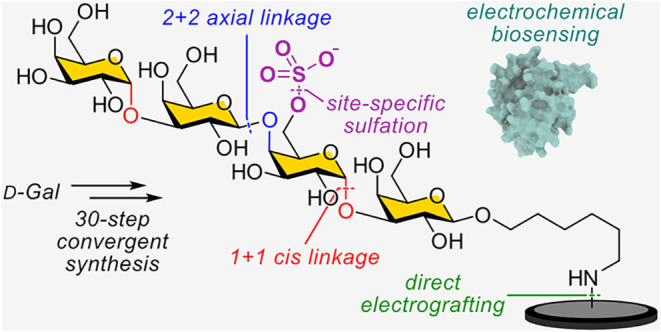

## Introduction

Sulfated algal glycans are valuable biomaterials that constitute a significant portion of marine biomass. As a major cell wall component of macroalgae, sulfated glycans possess vital structural, osmoregulatory, and defensive functions.^1,2^ These diverse sulfated biopolymers display a range of properties as potent stabilizing, gelling, antifungal, and antimicrobial agents.^3–7^ Carrageenans (CGNs), linear galactans found in red algae (Rhodophyta),^8^ show a wide functional diversity that is linked to the degree and the pattern of scaffold modifications.^9^ CGNs consist of α(1→3) linked repeats of a β(1→4) digalactose “carrabiose” unit (Figure 1). To date, over a dozen distinct subclasses of CGNs have been identified and categorized based on the sulfation and anhydration pattern of the core carrabiose disaccharide repeats.^9^ The antipathogenic and rheological properties make CGNs attractive for various cosmetic, drug delivery, and food-related applications.^10^ Research into immunomodulatory and anticancer properties of CGNs, warranted by some structural similarities to mammalian glycosaminoglycans, foreshadows exciting therapeutic applications.^4,11^ However, these efforts are hampered by the poor understanding of the structure–function relationship of CGNs.

**Figure 1:**
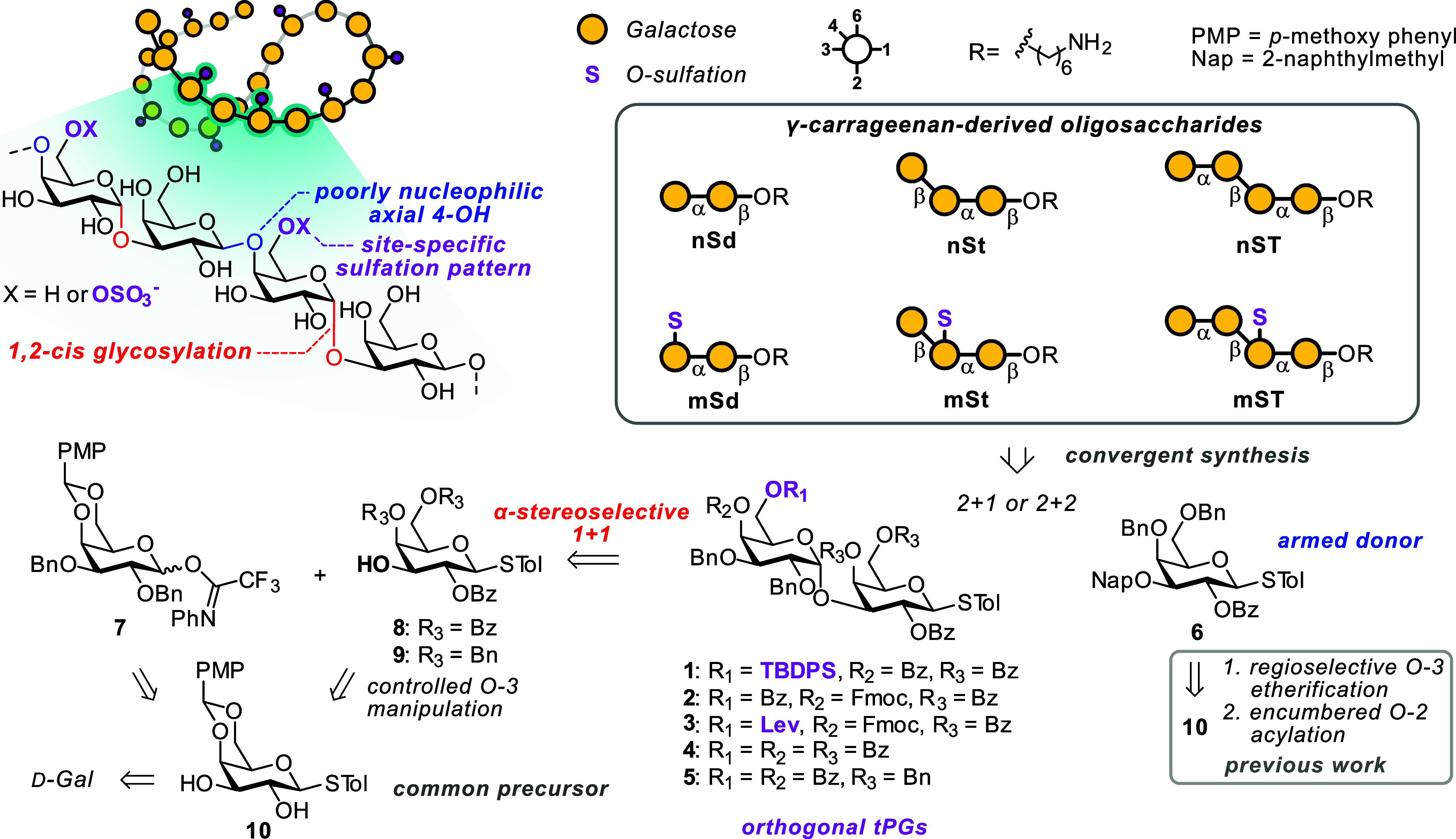
Structure of γ-CGN and derived oligosaccharides. Top left: unique synthetic challenges of CGN synthesis. Bottom: retrosynthesis of monosaccharide and disaccharide BBs for the synthesis of γ-CGNs. Inset: the panel of γ-CGN-derived oligosaccharides.

Natural CGNs exist as heterogeneous polysaccharide mixtures with varying chain lengths and modification patterns.^12^ The ability to manipulate glycan expression and production is extremely limited, since glycans are regulated by nontemplated biochemical pathways and complex chemical cascades.^13,14^ Moreover, glycan structures lack a unified blueprint: dynamic modifications to monosaccharide residues, i.e., sulfation and anhydration, occur even within the same polysaccharide chain, resulting in hybrid CGNs.^15,16^ While CGN extracts from natural sources display a wide range of desirable bulk properties (e.g., antiviral, anticoagulatory),^17,18^ batch-to-batch variability and cross-reactivity impede the understanding of glycobiology on a molecular level.^19^ Inaccessibility of homogeneous, structurally well-defined oligosaccharides hampers the development of many CGN-based applications.

Bottom-up chemical synthesis of CGNs is necessary for the acquisition of much-needed homogeneous constructs.^20–23^ This strategy entails the chemical glycosylation in a regio- and stereodefined manner and site-specific sulfation. Specifically, a strategy for the synthesis of CGNs must accommodate (1) a challenging 1,2-*cis* stereoselective glycosylation,^24,25^ (2) glycosylation of a sterically hindered and poorly nucleophilic axial 4-OH of galactose,^26,27^ and (3) introduction of a multilevel protecting group (PG) hierarchy that enables site-specific post-assembly sulfation.^28^ Some CGN structural motifs have been synthesized, focusing either on scaffold regio- and stereochemistry,^29^ or carrabiose modifications,^30,31^ but not both. Although protected synthetic intermediates have been obtained,^32^ homogeneous sulfated CGNs remain inaccessible due to formidable synthetic challenges. Robust stereocontrol of glycosidic linkages and a multilevel orthogonal PG architecture that accommodates both alternating α(1→3) and β(1→4) linkages and precise site-specific sulfation is crucial for the synthesis of CGNs. Application-compatible CGNs require the addition of a suitable chemical handle for conjugation or surface immobilization.^33–35^ To achieve this, we set out to design tailor-made building blocks (BBs) that capture the uncommon structural features of CGNs with an elaborate PG hierarchy and develop suitable assembly strategies.

We highlighted the monosulfated γ-CGN as a prototypical member of the CGN family. Synthesis of γ-CGN-derived oligosaccharides with the unique galactan scaffold consisting of alternating glycosidic bonds is a necessary stepping stone for accessing CGNs with complex modification patterns. We assessed that early-stage stereoselective installation of the 1,2-*cis* glycosidic linkage is crucial for achieving a well-defined galactan scaffold. For that purpose, α(1→3) digalactose BBs were envisioned. These disaccharide units serve as precursors that embody all the characteristic features necessary for the assembly of γ-CGN oligosaccharides.

## Results and Discussion

### Synthesis of γ-CGN BBs

Thioglycoside BBs **1**–**5** were designed, with distinct PG composition of the nonreducing galactoside subunit. These BBs are derived from key 4′,6′-O-arylidene-protected digalactose precursors obtained through glycosylation of **8** or **9** with **7**. The 4,6-O-arylidene PG of **7** plays a dual role: it promotes stereoselective 1,2-*cis* glycosylation^36,37^ and enables subsequent selective O-4′ and O-6′ manipulation of the disaccharide.^38^

An orthogonally protected O-6′ of **1** and **3** ensures site-specific post-assembly sulfation, which is a defining characteristic of γ-CGN. The 4′-O-Fmoc-protected **2** and **3** enable the unmasking of a glycosyl acceptor for the subsequent oligosaccharide elongation. To compensate for the poor nucleophilicity of the O-4′ acceptor, the use of an armed monosaccharide thiogalactoside **6** was envisioned, which can be obtained according to our previously developed strategy.^39^ The combination of **1**–**5** disaccharide BBs, monosaccharide donor **6**, and an amino-functionalized alkyl linker provides the required flexibility for the synthesis of conjugation-ready γ-CGN-derived di-, tri-, and tetrasaccharides (Figure 1, inset). Finally, all monomeric units **6**–**9** can be obtained from one common precursor **10**. With that in mind, we set out to synthesize γ-CGN-derived oligosaccharides.

Arylidene-protected glycosyl imidate donor **7** and thioglycoside acceptor **8** were synthesized from common precursor **10** (Scheme 1). The O-3 acceptor **8** was obtained by the transient regioselective installation of an Fmoc PG via a stannylene acetal intermediate. To the best of our knowledge, the use of organotin-mediated Fmoc installation was not reported and provides a useful novel O-3 protection strategy of 2,3-diols. Notably, this method was completely regioselective, and disubstituted byproducts were not detected. Arylidene acetal hydrolysis and per-benzoylation afforded thiogalactoside **13**.

**Scheme 1:**
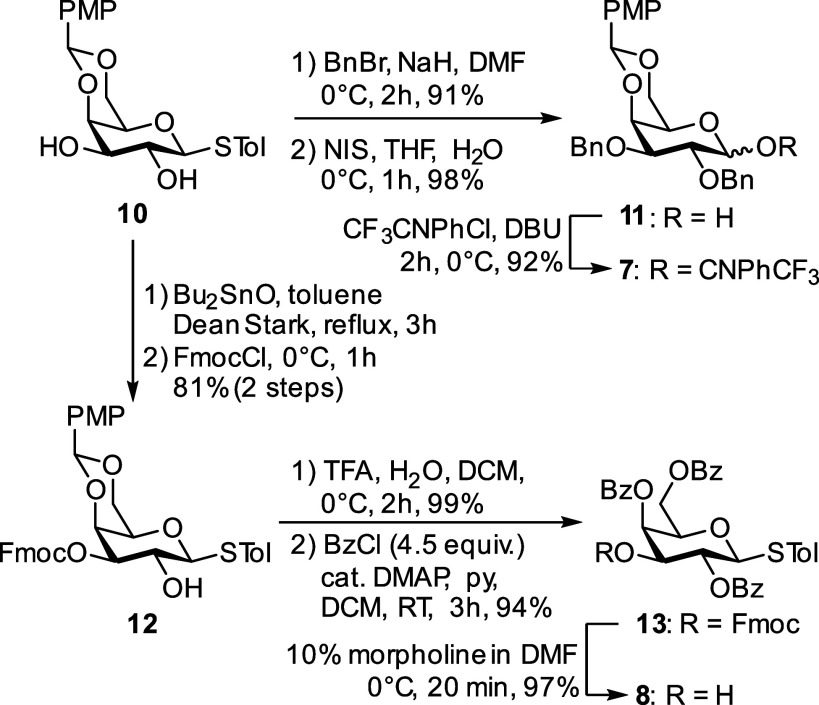
Synthesis of α-Stereodirecting Arylidene-Protected Glycosyl Imidate Donor and Thioglycoside Acceptor by Regioselective Fmoc Protection–Migration-Free Deprotection

Subsequent Fmoc removal using mild conditions (10% morpholine in DMF) produced acceptor **8**, circumventing O-4 and O-2 acyl migrations, which were prominent under standard deprotection conditions using piperidine (Table S1). Notably, benzoyl-protected acceptor **8** was designed with multiple ester-type PGs, since electron-poor acceptors are reported to enhance 1,2-*cis* stereoselectivity.^40^

Acceptor **8** was glycosylated with **7** to obtain disaccharide thioglycoside **14** (Figure 2). The galactosyl imidate donor **7** was activated under catalytically acidic conditions without affecting the orthogonal thioaryl latent leaving group of the O-3 thiogalactoside acceptor **8**.^38^

**Figure 2:**
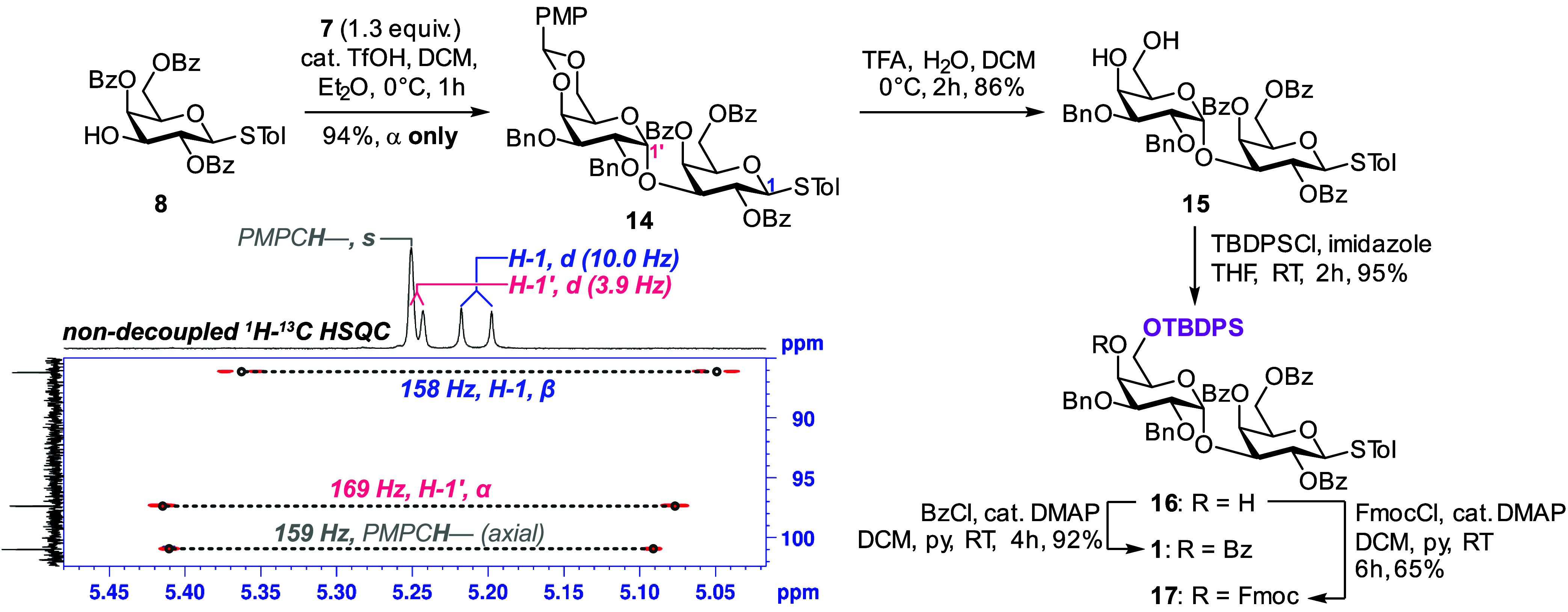
Synthesis of α(1→3)-linked digalactose thioglycoside donor *via* stereoselective 1 + 1 glycosylation. Inset: nondecoupled HSQC of the anomeric region. ^*2*^ *J*_HH_ and ^*1*^ *J*_CH_ values are shown for the reducing (blue) and nonreducing (red) monosaccharides.

Although the installation of an α-linkage without auxiliary assistance is not trivial,^24,41,42^ disaccharide **14** was obtained with complete α-stereoselectivity, favored by the torsion strain-promoted 1,2-*cis* glycosylation of bicyclic arylidene-protected donors.^36,37^ The stereochemistry of the newly formed glycosidic bond was confirmed by the measured ^2^ *J*_H1,H2_ and ^1^ *J*_C1,H1_ coupling constants in ^1^H NMR and ^1^H–^13^C nondecoupled heteronuclear single-quantum coherence (HSQC) NMR spectroscopy, respectively (Figure 2, inset). The ^1^ *J*_C1′,H1′_ of 169 Hz was consistent with a typical coupling constant for α configuration in galactopyranosides, unambiguously higher than the ^1^ *J*_C1,H1_ of 158 Hz of the β anomer at the reducing end.^43,44^ The α-linked disaccharide thioglycoside was further derivatized to obtain donors **1** and **17**, with O-6′ protected with an orthogonal *tert*-butyldiphenylsilyl (TBDPS) PG that would enable site-specific sulfation at a later stage.

### Synthesis of Conjugation-Ready γ-CGNs

A commonly used amine-functionalized alkyl acceptor **18** was glycosylated with disaccharide donor **1**, followed by the 6′-O-TBDPS deprotection to obtain γ-CGN disaccharide precursor **19** (Figure 3). Excess of the simple alkyl acceptor enabled the formation of **19** in excellent yield, over two steps. Above-zero temperatures were required for efficient glycosylation with the disarmed donor **1** in accordance with our previous studies.^45^ Global deprotection of **19** yielded the nonsulfated disaccharide **nSd**. To obtain the monosulfated γ-CGN disaccharide **mSd**, **19** was sulfated providing intermediate **20**, which was followed by global deprotection. Notably, **mSd** was obtained only in fair yield, attributed to some sulfate ester cleavage during methanolysis (see Supporting Information). The regiodefined sulfation of **mSd** was confirmed by an indicative ^13^C downfield shift of the C-6′ methylene, as measured by distortionless enhancement by polarization transfer (DEPT) NMR spectroscopy, compared to the nonsulfated **nSd** (Figure 3, bottom).^46^

**Figure 3:**
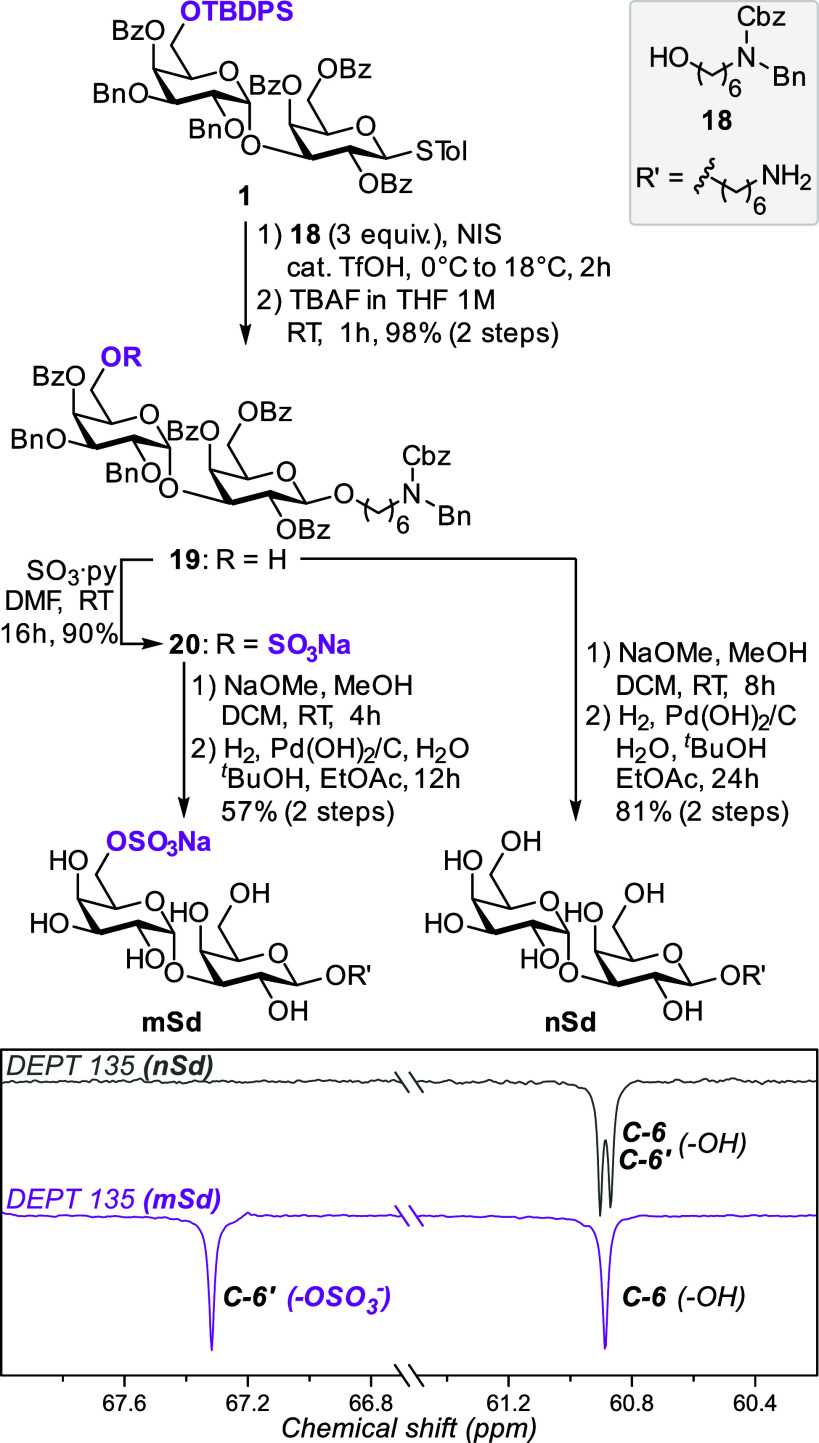
Synthesis of γ-CGN disaccharides **mSd** and **nSd**. NaOMe was quenched with H-form ion-exchanged resin, immediately followed by the addition of Na-form resin to the solution (see Supporting Information). Bottom: DEPT-135 of C-6 methylene region of nonsulfated **nSd** (gray) and 6-O-sulfated **mSd** (magenta).

Disaccharides **nSd** and **mSd** display basic structural motifs of γ-CGN, containing the α(1→3) digalactose unit and the O-6′ sulfation site. Moreover, the amine-functionalized linker of these disaccharides enables bioconjugation or surface immobilization for the study of molecular interactions, immunomodulatory properties, etc.^47^

After successfully establishing a synthetic strategy for the stereoselective 1,2-*cis* glycosylation and the regiodefined sulfation, we set out to tackle the extension of the disaccharide motif to obtain γ-CGN-derived oligosaccharides. The assembly of the γ-CGN trisaccharides was envisioned *via* 2 + 1 glycosylation, which would involve the installation of a β(1→4) linkage on a sterically hindered, poorly nucleophilic disaccharide O-4 axial acceptor (Scheme 2).^27,48^ Galactosyl donor **13** possessed suitable β-stereodirecting properties; however, its strongly disarming PG hierarchy would be disadvantageous in the glycosylation of an encumbered glycosyl acceptor. In order to compensate for the poor reactivity of the O-4 glycosyl acceptor, we resolved to utilize an armed β-stereodirecting thioglycoside donor **6** (Scheme 2, inset). Thiogalactoside **6** was acquired from the common precursor **10** over four steps. The O-2 benzoylation of the conformationally restricted arylidene-protected galactoside **21** proceeded smoothly according to a previously developed method.^39^ Digalactose thioglycoside donor **2** was obtained from diol **15** by regioselective O-6′ acylation,^49^ followed by Fmoc protection of the free 4′-OH (Scheme 2). Glycosylation of alkyl acceptor **18** with donor **2**, followed by Fmoc deprotection, produced disaccharide O-4′ acceptor **24**. Glycosylation of **24** with **6** was optimized to obtain trisaccharide **25** in fair yield (Table S2).

**Scheme 2:**
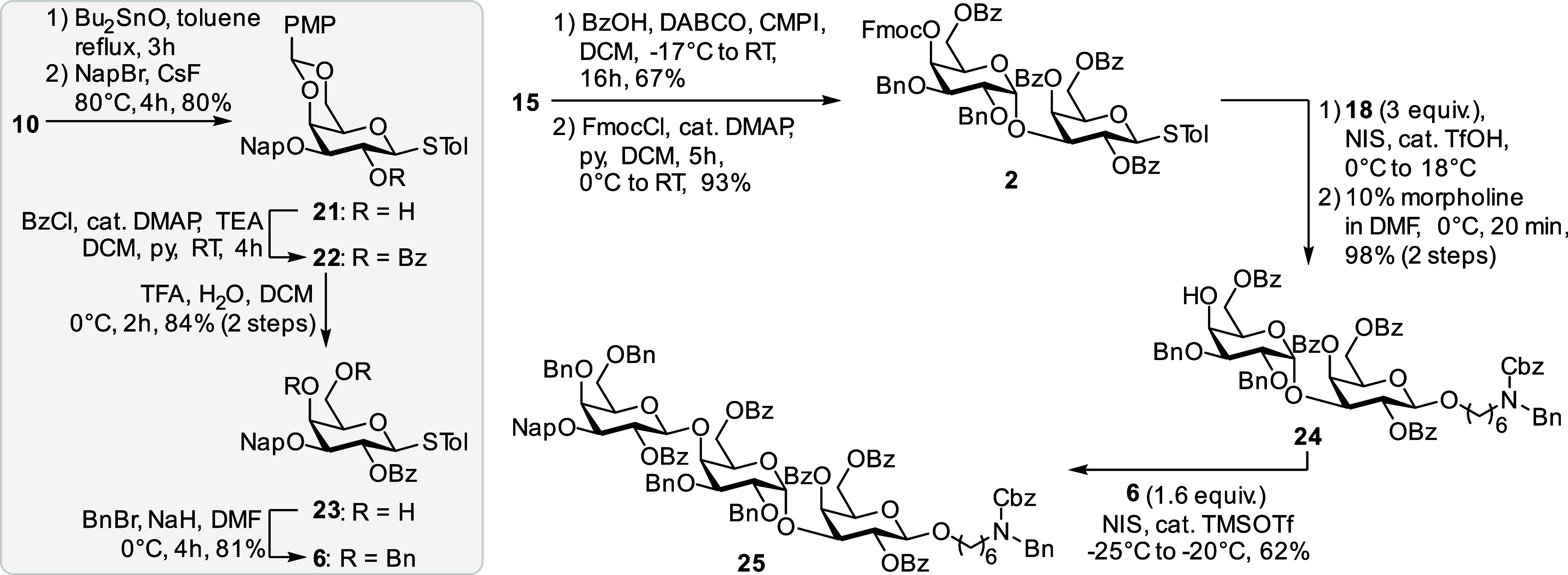
Synthesis of γ-CGN Trisaccharide *via* 2 + 1 Glycosylation with an Armed Thiogalactoside Donor

Surprisingly, the global deprotection of **25** to yield nonsulfated γ-CGN trisaccharide **nSt** proved nontrivial. Methanolysis of **25** resulted in incomplete deprotection, affording a 1:1 mixture of **26a** and **26b**, even after extended reaction time (Figure 4, middle). Structural assignment of **26a** revealed that the ether-adjacent 2″-O-Bz of the terminal galactoside was not removed. Anomalous resistance of ester PGs to methanolysis in 2-O-acyl-3-O-alkyl glycosides was reported previously.^50^ Recently, we reported O-2 steric masking by 3-O-ether substituents.^39^ A similar phenomenon may be involved in the uncharacteristic resistance of the isolated 2″-O-Bz to methanolysis. In order to overcome this issue, we attempted to reverse the common order of global deprotection, with hydrogenolysis preceding hydrolysis.^51,52^ In this manner, the two-step cascade will proceed *via* intermediate **27** with an accessible 2″-O-Bz (Figure 4, left). Hydrogenolysis of **25** produced an inseparable mixture of numerous partially deprotected intermediates and traces of the ether-deprotected trisaccharide **27**, even after prolonged reaction times and with increased catalyst loading (see Supporting Information). This was attributed to the highly contrasting solubility of reactant and products that produced poorly soluble intermediates in tested solvent systems.

**Figure 4:**
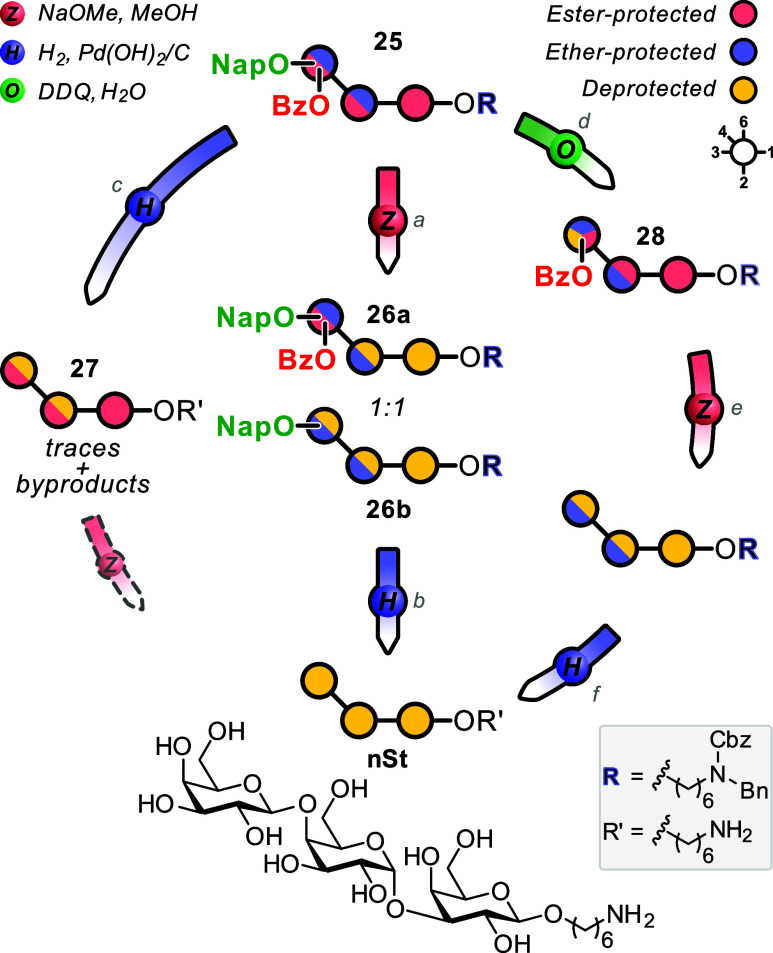
Deprotection strategies of **nSt** precursor. Left route: “reverse” hydrogenolysis-first route; middle route: “classical” methanolysis, followed by hydrogenolysis; right route: selective Nap deprotection, followed by methanolysis and hydrogenolysis. (a) NaOMe, MeOH, DCM, RT, 24h (**26a**, 45%; **26b**, 53%); (b) H_2_, Pd­(OH)_2_/C, THF, H_2_O, 75%; (c) H_2_, Pd­(OH)_2_/C, THF *or* EtOAc, ^*t*^BuOH, traces; (d) DDQ, DCM, H_2_O, 74%; (e) NaOMe, MeOH, DCM; (f) H_2_, Pd­(OH)_2_/C, THF, H_2_O, 97% 2-step yield.

Finally, we resolved to eliminate the problematic 2-O-acyl-3-O-alkyl PG arrangement by selective Nap cleavage to produce **28**. The following two-step global deprotection proceeded smoothly to obtain γ-CGN trisaccharide **nSt** (Figure 4, right). This further reinforced the apparent masking effect produced by the 3-O-Nap substituent.

With nonsulfated γ-CGN trisaccharide **nSt** in hand, we proceeded to synthesize a selectively 6′-O-protected trisaccharide precursor with a latent sulfation site. We envisioned a 2 + 1 glycosylation of an O-4′ disaccharide acceptor with an orthogonally protected O-6′. Glycosylation of alkyl acceptor **18** with digalactose donor **17**, followed by Fmoc deprotection, produced a suitable disaccharide acceptor **29**. However, the glycosylation of **29** with the armed donor **6** afforded trisaccharide **30** in poor yield (Scheme 3, left).

Exceptionally inefficient glycosylation of a 6-O-TBDPS-adjacent glycosyl acceptor was previously reported, attributed to the steric crowding of the proximal bulky PG.^53^ To improve the efficiency of the 2 + 1 glycosylation, we resolved to replace TBDPS with the less sterically hindered levulinic ester (Lev) PG. To that end, 6′-O-Lev protected digalactose thioglycoside donor **3** was obtained from diol **15** employing the same regioselective acylation strategy used to obtain donor **2**.^49^ Glycosylation of **18** with donor **3**, followed by Fmoc deprotection afforded O-4′ digalactose acceptor **31** (Scheme 3, middle). Acceptor **31** was then glycosylated with **6** to obtain trisaccharide **32** in fair yield. Replacing the bulky 6′-O-TBDPS with a Lev PG on the glycosyl acceptor produced a profound impact on the efficiency of the 2 + 1 glycosylation with the same armed donor **6**. This highlighted the significance of acceptor accessibility and reframed the glycosylation of the O-4 axial acceptor as a major bottleneck for longer γ-CGN oligosaccharides.

Selective deprotection of trisaccharide **32** with hydrazine acetate produced **33** with a free 6′-OH. Sulfation on the O-6′ proceeded smoothly to obtain **34**, despite the potential steric crowding of the adjacent glycosylated O-4′. Sulfated trisaccharide **34** is a key precursor of the monosulfated γ-CGN-derived trisaccharide **mSt**. However, basic ester hydrolysis of **34** resulted in incomplete removal of Bz PGs, presumably associated with the presence of a 3″-O-Nap, as observed for **25**. We refrained from directly mirroring the deprotection strategy of **25**, since the acidic hydroquinone byproduct of Nap oxidative removal could promote hydrolysis of the acid-labile 6′-O-sulfate ester. Instead, we resolved to replace the Nap PG, before introducing sulfation (Scheme 3 right).

The masking Nap PG of **32** was replaced by an Ac via oxidative cleavage and acetylation of the liberated 3″-OH. This was followed by Lev deprotection to obtain **35**. The sulfation of the only free 6′-OH ensured formation of γ-CGN trisaccharide precursor **36** as a single product. Methanolysis was avoided to circumvent the desulfation, which was observed for **mSd**. Benzoate ester hydrolysis of trisaccharide **36** with LiOH proceeded smoothly without the loss of a sulfate group.^54^ The presence of an adjacent 3″-O-Ac did not impede Bz deprotection in agreement with previous reports.^50^ Finally, hydrogenolysis afforded 6′-O-sulfated trisaccharide **mSt** in excellent yield.

Encouraged by the successful synthesis of γ-CGN trisaccharides, we set out to obtain longer constructs. Tetrasaccharides are considered benchmarks for biological studies, as they are long enough to replicate key features and interactions of the parent compounds.^55,56^

We moved on to tackle the synthesis of γ-CGN-derived tetrasaccharides containing two challenging α(1→3) glycosidic linkages. In order to ensure correct stereochemistry, we envisioned assembly of tetrasaccharides in a 2 + 2 glycosylation of a sufficiently accessible O-4′ digalactose acceptor (Table 1, top).

**Table 1:**
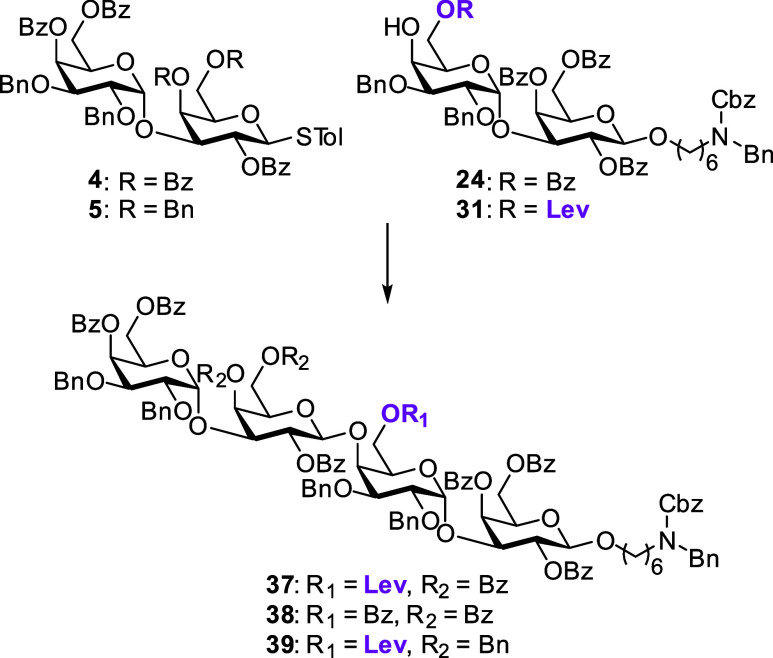
Assembly of γ-CGN Tetrasaccharides

entry	acceptor+ donor	conditions	product	yield (%)^a^
1	**31** + **4**	NIS, TfOH 1 h, 5 °C → 9 °C	**37**	n.d.^b^
2	**31** + **4**	NIS, TfOH, 4Å MS 2 h, 5 °C → 20 °C	**37**	15
3	**24** + **4**	NIS, TfOH, 4 Å MS 1 h, 2 °C → 19 °C	**38**	16
4	**24** + **4**	Ph_2_SO, Tf_2_O, TTBP 3 h, −70 °C → 5 °C	**38**	n.d.^b^
5	**31** + **5**	NIS, TfOH, 4 Å MS 2 h, −14 °C → 2 °C	**39**	31
6	**31** + **5**	NIS, TMSOTf, 4 Å MS 1 h, −35 °C → −20 °C	**39**	44

a Isolated yield.

b Not detected (n.d.) by LCMS analysis of the crude.

Glycosylation of **31** with digalactose donor **4**, which was readily obtained from precursor **15**, proved extremely inefficient even at elevated temperatures (Table 1, entry 1 and 2). Glycosylation of a 6′-O-Bz-protected acceptor **24** with **4** produced **38**, with a similarly poor yield (entry 3). Switching to an alternative activator system did not produce tetrasaccharide **38** (entry 4). The major challenges faced with glycosylation of **24** or **31** with **4**, compared to the glycosylation of the same acceptors with **6** (Scheme 2, Scheme 3), indicated that for this 2 + 2 glycosylation scenario, the poor reactivity of the disarmed digalactose donor **4** was a major drawback.

**Scheme 3:**
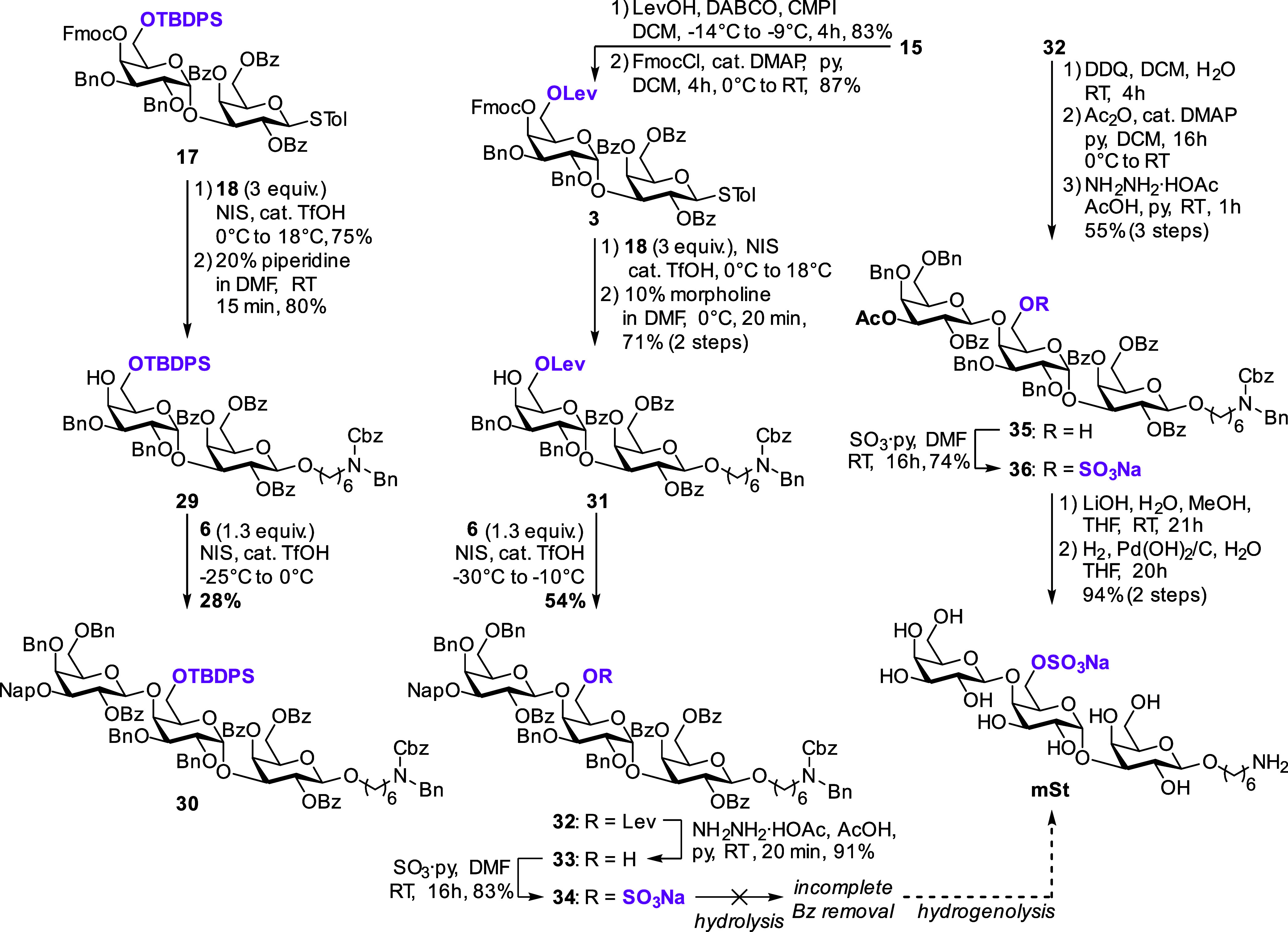
Synthesis of Sulfated γ-CGN Trisaccharides mSt *via* 2 + 1 Glycosylation with a Less Hindered Acceptor and Replacement of the Masking 3-O-Nap Group to Facilitate Global Deprotection

The successful application of the armed donor **6** for the formation of trisaccharides suggested that an analogous armed digalactose thioglycoside donor would benefit the preparation of tetrasaccharides. Donor **5** was synthesized as a replacement for the unreactive donor **4** *via* α-stereoselective 1 + 1 glycosylation of O-3 thioglycoside acceptor **9** in three steps (derived from **6**, see Supporting Information). Notably, aglycon transfer and anomerization byproducts observed en route to **5** reduced its overall yield.^57,58^ This showed that the complete imidate-thioglycoside orthogonality utilized in the formation of the disarmed disaccharide **14** was not preserved in this case. Nevertheless, glycosylation of acceptor **31** with armed digalactoside donor **5** benefited the formation of tetrasaccharide **39** with significantly improved efficiency, compared to **37** (Table 1, entry 5). Glycosylation at lower temperatures further improved the yield (Table 1, entry 6), highlighting that the decomposition of the activated donor **5** and not its reactivity was the limiting factor (unlike the unreactive donor **4**). This strategy entailed a favorable trade-off: the 3-fold gain in glycosylation efficiency with **5** justified the reduced yield of its preparation.

Selective 6′-O-Lev deprotection to obtain **40**, followed by sulfation of the liberated position afforded O-6′ sulfated γ-CGN precursor **41** (Figure 5A). Complete removal of benzoate esters of **41** was achieved with LiOH, albeit with extended reaction time and careful adjustment of solvent ratios (see Supporting Information). Finally, hydrogenolysis afforded monosulfated γ-CGN tetrasaccharide **mST** with excellent yield. The HSQC of **mST** showed four distinct anomeric protons, two α- and two β-linkages, and the 6^*II*^-O-sulfate-adjacent H-5^*II*^ showed a markedly downfield shift, consistent with the shift observed for **mSd** and **mSt** (Figure 5A, inset). Global deprotection of tetrasaccharide **37** afforded nonsulfated CGN counterpart **nST.**

**Figure 5:**
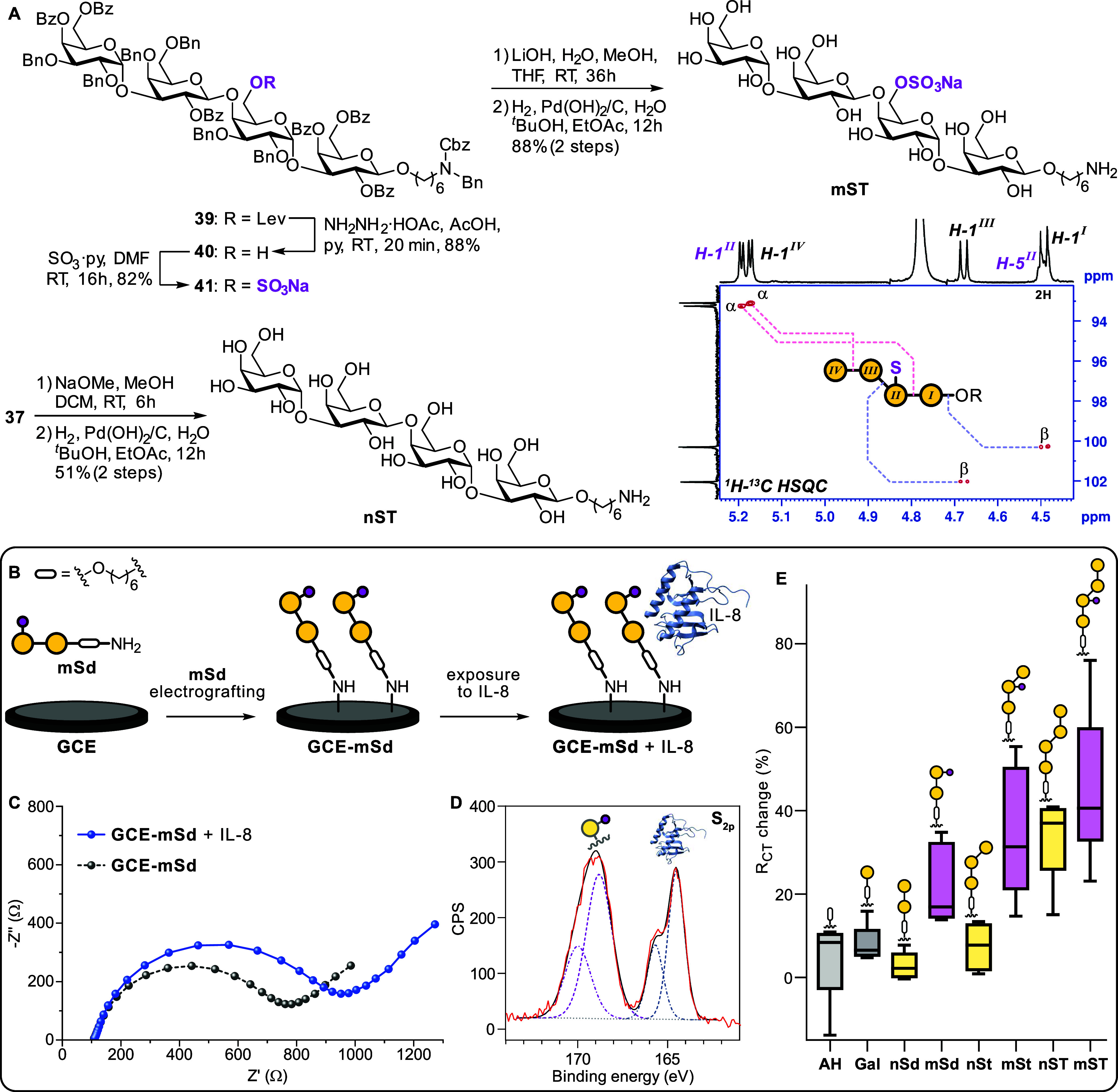
(A) Synthesis of sulfated γ-CGN tetrasaccharide **mST** by Lev deprotection, sulfation, and global deprotection from **39**. Inset: ^1^H–^13^C HSQC of **mST** two β (blue lines) and two α (red lines) anomeric signals. Synthesis of nonsulfated **nST** by global deprotection of **37**. (B) Electrografting of GCE with amine-functionalized-CGN **mSd** under oxidative potential applied by cyclic voltammetry. Exposure of **GCE-mSd** to IL-8. (C) Representative Nyquist plot of one **GCE-mSd** electrode before (black) and after (blue) exposure to IL-8. (D) S_2p_ XPS spectrum of **GCE-mSd** + IL-8. The *Y*-axis offset is zero-corrected, and the gray dotted line indicates background counts per second (CPS). CPS (red line) is fitted with an envelope (black line). S_2p1/2_ (dot-dash) and S_2p3/2_ (dash) deconvolution shown for SO_4_ (purple) and SH (navy) signals. (E) Box plot of *R*_CT_ change (%) in CGN-modified GCEs and controls, at least five electrodes each, in response to IL-8. The *R*_CT_ change (%) is obtained by subtracting the *R*_CT_ value before exposure from the *R*_CT_ after exposure, divided by the *R*_CT_ before exposure, presented in percentages. Each box shows the interquartile range, with the line inside indicating the median and whiskers extending to the min and max values.

The above efforts highlighted that conventional strategies failed to provide γ-CGNs *via* a single standardized route. Only the tailored approach, involving unconventional installation and removal of PGs, and reconciliation of reactivity with the selectivity of glycosylation enabled access to the γ-CGN panel.

In possession of the first synthetic set of conjugation-ready sulfated and nonsulfated γ-CGNs, we set out to explore the structure-dependent bioactivity of these well-defined oligomers.

### CGN-Based Biosensing

Polysaccharides are key components of the extracellular matrix. Sulfated polysaccharides, e.g., heparan sulfate (HS), are known binding partners of chemokines that are involved in crucial intercellular signaling pathways.^59^ Interleukin-8 (IL-8) is a secreted chemokine that is responsible for neutrophil recruitment. The binding of IL-8 by HS depends on the sulfation degree, pattern, and monosaccharide composition; hence, they modulate the immune response in a structure-dependent manner.^60^ CGNs also bind IL-8;^61^ however, the lack of well-defined constructs has hampered the elucidation of structure-dependent interactions. Electrochemical impedance spectroscopy (EIS) combined with surface-analysis methods allows the utilization of small amounts of synthetic glycans to gain label-free insights on the effect of molecular features on binding preferences.^62–65^ In previous works, we used these methods to prove that the sulfation pattern, monosaccharide composition, and sulfation degree of tetrasaccharide HS segments affect IL-8 binding.^66,67^ We set out to utilize these methods to characterize the effect of sulfation and chain length on the binding of the synthesized γ-CGN segments to IL-8.

The functional primary amine was pivotal for exploiting CGNs for a sensing application. CGNs **nSd**, **mSd**, **nSt**, **mSt**, **nST**, and **mST** were immobilized on glassy carbon electrodes (GCEs) by applying oxidative potential *via* cyclic voltammetry (CV), to produce CGN-modified GCEs **GCE-nSd**, **GCE-mSd**, **GCE-nSt**, **GCE-mSt**, **GCE-nST**, and **GCE-mST**, respectively, using a previously developed method (Figure 5B and Figures S1 and S2).^68^ Repeated CV cycles promote the electrografting of the primary amine-functionalized linker onto the GCE, while tolerating chemically labile sulfate moieties. Electrografting and integrity of sulfated CGN on GCEs were supported by X-ray photoelectron spectroscopy (XPS) signal at the binding energy (BE) of S_2p_ (Figure S5). Fitting of the signal with a characteristic 2:1 ratio and 1.2 eV spin–orbit coupling for the S_2p3/2_ and S_2p1/2_ components provided BEs of 168.8 and 170.0 eV, corresponding to the sulfate moiety.

To provide biologically inactive negative controls, 6-aminohexanol (**AH**) and β-O-galactosylated AH (**Gal**) were grafted on GCEs to give **GCE-AH** and **GCE-Gal**, respectively, using the same assembly method.

The set of CGN-modified GCEs and controls was exposed to IL-8 to evaluate the effect of CGN chain length and sulfation profile on binding (Figure 5B,E). Exposure of the controls **GCE-AH** and **GCE-Gal**, as well as **GCE-nSd,** to IL-8 showed no appreciable change in the resistance to charge transfer (*R*_CT_) (Figure 5E and Figure S2). The monosulfated disaccharide-modified **GCE-mSd** showed a modest but unambiguous increase in *R*_CT_ after exposure to IL-8 (Figure 5C,E), indicative of the chemokine binding.^67^ IL-8 adsorption was validated by the appearance of an additional S_2p_ XPS signal at the BEs of 164.5 and 165.7 eV, consistent with cysteine residues (Figure 5D). A 6-fold increase in the N_1s_ XPS signal and a 350% increase in N surface atomic concentration corroborated the protein adsorption (Figure S5 and Table S11).

A discernible increase in *R*_CT_ resulting from surface binding of IL-8 to the O-6′ sulfated **mSd**, compared to **GCE-AH**, showed that IL-8 does not bind nonspecifically to a hydroxyl containing surface. This established a response threshold that showed that even GCE functionalization with short nonsulfated mono- and disaccharides did not lead to IL-8 binding. Together, these observations indicated that (1) binding of IL-8 is modification-dependent, (2) there is no pronounced affinity of the chemokine to galactose residues at the mono- or disaccharide level, and (3) sulfation significantly enhanced IL-8 binding to CGN disaccharides.

The **GCE-nSt** and **GCE-mSt** pair behaved similarly to the disaccharide-duo. **GCE-nSt** showed no appreciable response to IL-8, while exposure of **GCE-mSt** produced a pronounced increase in *R*_CT_, consistent with affinity toward the 6′-O-sulfate. Both tetrasaccharide-modified **GCE-nST** and **GCE-mST** showed IL-8 binding comparable to **GCE-mSt**. The high response of the nonsulfated **nST**-modified GCEs compared to **GCE-nSd** and **GCE-nSt** hints at a possible affinity of IL-8 toward an extended CGN scaffold, rather than being solely based on sulfate-mediated interactions. The monotonic increase in the response of the monosulfated CGN series (**GCE-mSd**, **GCE-mSt**, and **GCE-mST**) cemented this effect of chain length on IL-8 binding.

Comparison of the *R*_CT_ change of **GCE-mST** with that of our recently reported HS-derived tetrasaccharide constructs in an analogous sensing platform showed that both had a similar, ∼50% mean increase in *R*_CT_ following IL-8 binding (Figure 5E and Table S10).^67^ Comparable response of **GCE-mST** to that of GCEs modified with HS, a native IL-8 binding partner,^69^ highlighted the substantial and biorelevant affinity of γ-CGN toward IL-8. Moreover, CGNs interacted with IL-8 in a structure-dependent manner that is affected both by chain length and sulfation. We showed that even disaccharide and trisaccharide γ-CGN-derived constructs displayed biorelevant affinity, which are often considered too short for meaningful interactions.^70^ The ability to evaluate these parameters was enabled by the new capability to synthesize well-defined CGN fragments with a functional handle and properly characterize the interaction properties. Further exploration of structure-dependent CGN bioactivity might elucidate intricate aspects of molecular-level interactions.

## Conclusions

A panel of γ-CGN-derived oligomers was obtained through multistep total synthesis by employing an elaborate PG architecture and assembly of tailor-made BBs. Careful selection of PGs was used to establish stereocontrolled and regiodefined formation of the unique CGN scaffold consisting of alternating α- and β-glycosidic linkages and containing latent sulfation sites. Taking into consideration both the steric constraints of the glycosyl acceptor and the arming effects of the glycosyl donor facilitated the extension of the disaccharide structural motif to obtain CGN oligomers **nSt**, **mSt**, **mST**, and **nST**, overcoming the poor nucleophilicity of the axial O-4′ acceptor. Controlled manipulation of orthogonal PGs ensured site-specific 6′-O-sulfation, which carried over to a well-defined sulfation profile of **mSd**, **mSt**, and **mST** after global deprotection. Previously reported attempts to obtain CGNs provided either nonsulfated fragments or protected precursors.^29,32^ Our endeavor to integrate all CGN features produced interdependent synthetic constraints, which were resolved by nonstandard strategies, like tin-mediated Fmoc installation and an altered cascade of global deprotection.

This marks the first total synthesis of conjugation-ready γ-CGN-derived sulfated oligosaccharides with full control over the stereo- and regiochemistry of glycosidic linkages and site-specific sulfation. The undertaken effort to install a functional amine handle and overcome synthetic, solubility, and purification challenges associated with it was rewarded with the ability to directly apply CGNs to a chemokine binding study. EIS analyses provided clear evidence of the effect of chain length and sulfation of CGNs on IL-8 binding preference. A robust synthetic strategy was established for the future acquisition of an expanded library of well-defined CGN oligosaccharides. Acquisition of homogeneous CGNs paves the way for studies of glycan-protein interactions and the development of therapeutic and sensing applications.

## Supplementary Material





## Notes

General methods, experimental protocols for the preparation and characterization of all compounds, including NMR assignment and HRMS (PDF)
NMR spectra of all compounds (PDF)

## Abbreviations

CGN: Carrageenan
PG: Protecting group
BB: Building block
Bn: Benzyl
Bz: Benzoyl
Cbz: Benzyloxycarbonyl
Fmoc: Fluorenylmethoxycarbonyl
Lev: Levulinoyl
Nap: 2-Naphthylmethyl
PMP: 4-Methoxyphenyl
TBDPS: *tert*-Butyldiphenylsilyl
Tol: 4-Methylphenyl
GCE: Glassy carbon electrode
CV: Cyclic voltammetry
IL-8: Interleukin-8
