# Supplementary material for: Total Synthesis of Conjugation-Ready Sulfated Red Algae Carrageenan Oligosaccharides for Sensing Applications
Source: J Am Chem Soc. 2026 Jun 10;148(27):29430–40. doi: 10.1021/jacs.6c09827 (PMC13383626; doi:10.1021/jacs.6c09827)
Supplement: Supplementary file 1 [file ja6c09827_si_001.pdf]

Supporting Information

Total Synthesis of Conjugation-Ready Sulfated Red Algae Carrageenan Oligosaccharides for Sensing Applications

Yonatan Sukhran, Roey G. Meir, Israel Alshanski, Shlomo Yitzchaik, Mattan Hurevich*.

Institute of Chemistry and Center for Nanoscience and Nanotechnology, The Hebrew University of Jerusalem, Safra Campus, Givat Ram, Jerusalem 9190401, Israel.

E-mail: Mattan.Hurevich@mail.huji.ac.il

Table of Contents

Experimental Section 5

General methods 5

Building block synthesis 6

Scheme S1. Outline of building block synthesis. 6

***p*-Tolyl-1-thio-β-D-galactopyranoside (S1)** 7

***p*-Tolyl 4,6-O-(4-methoxyphenyl)methylene-1-thio-β-D-galactopyranoside (10)** 7

***p*-Tolyl 2,3-di-O-benzyl-4,6-O-(4-methoxyphenyl)methylene-1-thio-β-D-galactopyranoside (S2)** 8

2,3-di-O-benzyl-4,6-O-(4-methoxyphenyl)methylene-1-thio-D-galactopyranose (11) 9

2,3-Di-O-benzyl-4,6-O-(4-methoxyphenyl)methylene-1-O-(2,2,2-trifluoro-N-phenylacetimidoyl)-α/β-D-galactopyranoside (7) 9

***p*-Tolyl 3-O-(9-fluorenylmethyloxycarbonyl)-4,6-O-(4-methoxyphenyl)methylene-1-thio-β-D-galactopyranoside (12)** 10

***p*-Tolyl 3-O-(9-fluorenylmethyloxycarbonyl)-1-thio-β-D-galactopyranoside (S3)** 11

***p*-Tolyl 2,4,6-tri-O-benzoyl-3-O-(9-fluorenylmethyloxycarbonyl)-1-thio-β-D-galactopyranoside (13)** 11

***p*-Tolyl 2,4,6-tri-O-benzoyl-1-thio-β-D-galactopyranoside (8)** 12

Table S1. Acyl migration under Fmoc deprotection conditions 13

***p*-Tolyl 4,6-O-(4-methoxyphenyl)methylene-3-O-(2-naphthalenylmethyl)-1-thio-β-D-galactopyranoside (21)** 14

***p*-Tolyl 2-O-benzoyl-4,6-(4-methoxyphenyl)methylene-3-O-(2-naphthalenylmethyl)-1-thio-β-D-galactopyranoside (22)** 15

***p*-Tolyl 2-O-benzoyl-3-O-(2-naphthalenylmethyl)-1-thio-β-D-galactopyranoside (23)** 15

***p*-Tolyl 2-O-benzoyl-4,6-di-O-benzyl-3-O-(2-naphthalenylmethyl)-1-thio-β-D-galactopyranoside (6)** 16

***p*-Tolyl 2-O-benzoyl-4,6-di-O-benzyl-1-thio-β-D-galactopyranoside (9)** 17

p-Tolyl 2,3-di-O-benzyl-4,6-O-(4-methoxyphenyl)methylene-α-D-galactopyranosyl-(1→3)-2,4,6-tri-O-benzoyl-1-thio-β-D-galactopyranoside (14) 17

p-Tolyl 2,3-di-O-benzyl-α-D-galactopyranosyl-(1→3)-2,4,6-tri-O-benzoyl-1-thio-β-D-galactopyranoside (15) 18

p-Tolyl 2,3-di-O-benzyl-6-O-(tert-butyldiphenylsilyl)-α-D-galactopyranosyl-(1→3)-2,4,6-tri-O-benzoyl-1-thio-β-D-galactopyranoside (16) 19

p-Tolyl 4-O-benzoyl-2,3-di-O-benzyl-6-O-(tert-butyldiphenylsilyl)-α-D-galactopyranosyl-(1→3)-2,4,6-tri-O-benzoyl-1-thio-β-D-galactopyranoside (1) 19

p-Tolyl 2,3-di-O-benzyl-4-O-(9-fluorenylmethyloxycarbonyl)-6-O-(tert-butyldiphenylsilyl)-α-D-galactopyranosyl-(1→3)-2,4,6-tri-O-benzoyl-1-thio-β-D-galactopyranoside (17) 20

p-Tolyl 6-O-benzoyl-2,3-di-O-benzyl-α-D-galactopyranosyl-(1→3)-2,4,6-tri-O-benzoyl-1-thio-β-D-galactopyranoside (S5) 21

p-Tolyl 6-O-benzoyl-2,3-di-O-benzyl-4-O-(9-fluorenylmethyloxycarbonyl)-α-D-galactopyranosyl-(1→3)-2,4,6-tri-O-benzoyl-1-thio-β-D-galactopyranoside (2) 21

p-Tolyl 2,3-di-O-benzyl-6-O-levulinoyl-α-D-galactopyranosyl-(1→3)-2,4,6-tri-O-benzoyl-1-thio-β-D-galactopyranoside (S6) 22

p-Tolyl 2,3-di-O-benzyl-4-O-(9-fluorenylmethyloxycarbonyl)-6-O-levulinoyl-α-D-galactopyranosyl-(1→3)-2,4,6-tri-O-benzoyl-1-thio-β-D-galactopyranoside (3) 23

p-Tolyl 4,6-di-O-benzoyl-2,3-di-O-benzyl-α-D-galactopyranosyl-(1→3)-2,4,6-tri-O-benzoyl-1-thio-β-D-galactopyranoside (4) 24

p-Tolyl 2,3-di-O-benzyl-4,6-O-(4-methoxyphenyl)methylene-α-D-galactopyranosyl-(1→3)-2-O-benzoyl-4,6-di-O-benzyl-1-thio-β-D-galactopyranoside (S7) 24

p-Tolyl 2,3-di-O-benzyl-α-D-galactopyranosyl-(1→3)-2-O-benzoyl-4,6-di-O-benzyl-1-thio-β-D-galactopyranoside (S9) 26

p-Tolyl 4,6-di-O-benzoyl-2,3-di-O-benzyl-α-D-galactopyranosyl-(1→3)-2-O-benzoyl-4,6-di-O-benzyl-1-thio-β-D-galactopyranoside (5) 27

Oligosaccharide synthesis 28

Scheme S2. Outline of the synthesis of mono-, di-, and trisaccharides 28

Scheme S3. Outline of the synthesis of tri- and tetrasaccharides 29

N-benzyl-N-benzyloxycarbonyl-6-aminohexanol (18) 29

N-benzyl-N-benzyloxycarbonyl-6-aminohexyl 2,4,6-tri-O-benzoyl-β-D-galactopyranoside (S10) 30

N-benzyl-N-benzyloxycarbonyl-6-aminohexyl β-D-galactopyranoside (S11) 31

6-Aminohexyl β-D-galactopyranoside (Gal) 31

N-benzyl-N-benzyloxycarbonyl-6-aminohexyl 4-O-benzoyl-2,3-di-O-benzyl-α-D-galactopyranosyl-(1→3)-2,4,6-tri-O-benzoyl-β-D-galactopyranoside (19) 32

6-Aminohexyl α-D-galactopyranosyl-(1→3)-β-D-galactopyranoside (nSd) 33

N-benzyl-N-benzyloxycarbonyl-6-aminohexyl 4-O-benzoyl-2,3-di-O-benzyl-6-O-sulfo-α-D-galactopyranosyl-(1→3)-2,4,6-tri-O-benzoyl-β-D-galactopyranoside (20) 34

6-Aminohexyl 6-O-sulfo-α-D-galactopyranosyl-(1→3)-β-D-galactopyranoside (mSd) 34

N-benzyl-N-benzyloxycarbonyl-6-aminohexyl 2,3-di-O-benzyl-6-O-benzoyl-α-D-galactopyranosyl-(1→3)-2,4,6-tri-O-benzoyl-β-D-galactopyranoside (24) 35

N-benzyl-N-benzyloxycarbonyl-6-aminohexyl 2-O-benzoyl-4,6-di-O-benzyl-3-O-(2-naphthalenylmethyl)-β-D-galactopyranosyl-(1→4)-6-O-benzoyl-2,3-di-O-benzyl-α-D-galactopyranosyl-(1→3)-2,4,6-tri-O-benzoyl-β-D-galactopyranoside (25) 36

Table S2. Synthesis of 25 *via* glycosylation of 24 with 6 with different conditions 37

N-benzyl-N-benzyloxycarbonyl-6-aminohexyl 2-O-R-4,6-di-O-benzyl-3-O-(2-naphthalenylmethyl)-β-D-galactopyranosyl-(1→4)-2,3-di-O-benzyl-α-D-galactopyranosyl-(1→3)-β-D-galactopyranoside (26) 38

6-Aminohexyl 2-O-benzoyl-β-D-galactopyranosyl-(1→4)-6-O-benzoyl-α-D-galactopyranosyl-(1→3)-2,4,6-tri-O-benzoyl-β-D-galactopyranoside (27) 39

N-benzyl-N-benzyloxycarbonyl-6-aminohexyl 2-O-benzoyl-4,6-di-O-benzyl-β-D-galactopyranosyl-(1→4)-6-O-benzoyl-2,3-di-O-benzyl-α-D-galactopyranosyl-(1→3)-2,4,6-tri-O-benzoyl-β-D-galactopyranoside (28) .. 40

6-Aminohexyl β-D-galactopyranosyl-(1→4)-α-D-galactopyranosyl-(1→3)-β-D-galactopyranoside (nSt) 41

N-benzyl-N-benzyloxycarbonyl-6-aminohexyl 2,3-di-O-benzyl-4-O-(9-fluorenylmethyloxycarbonyl)-6-O-(tert-butyldiphenylsilyl)-α-D-galactopyranosyl-(1→3)-2,4,6-tri-O-benzoyl-β-D-galactopyranoside (S12) 42

N-benzyl-N-benzyloxycarbonyl-6-aminohexyl 2,3-di-O-benzyl-6-O-(tert-butyldiphenylsilyl)-α-D-galactopyranosyl-(1→3)-2,4,6-tri-O-benzoyl-β-D-galactopyranoside (29) 43

N-benzyl-N-benzyloxycarbonyl-6-aminohexyl 2-O-benzoyl-4,6-di-O-benzyl-3-O-(2-naphthalenylmethyl)-β-D-galactopyranosyl-(1→4)-2,3-di-O-benzyl-6-O-(tert-butyldiphenylsilyl)-α-D-galactopyranosyl-(1→3)-2,4,6-tri-O-benzoyl-β-D-galactopyranoside (30) 44

N-benzyl-N-benzyloxycarbonyl-6-aminohexyl 2,3-di-O-benzyl-6-O-levulinoyl-α-D-galactopyranosyl-(1→3)-2,4,6-tri-O-benzoyl-β-D-galactopyranoside (31) 45

N-benzyl-N-benzyloxycarbonyl-6-aminohexyl 2-O-benzoyl-4,6-di-O-benzyl-3-O-(2-naphthalenylmethyl)-β-D-galactopyranosyl-(1→4)-2,3-di-O-benzyl-6-O-levulinoyl-α-D-galactopyranosyl-(1→3)-2,4,6-tri-O-benzoyl-β-D-galactopyranoside (32) 46

N-benzyl-N-benzyloxycarbonyl-6-aminohexyl 2-O-benzoyl-4,6-di-O-benzyl-3-O-(2-naphthalenylmethyl)-β-D-galactopyranosyl-(1→4)-2,3-di-O-benzyl-α-D-galactopyranosyl-(1→3)-2,4,6-tri-O-benzoyl-β-D-galactopyranoside (33) 47

N-benzyl-N-benzyloxycarbonyl-6-aminohexyl 2-O-benzoyl-4,6-di-O-benzyl-3-O-(2-naphthalenylmethyl)-β-D-galactopyranosyl-(1→4)-2,3-di-O-benzyl-6-O-sulfo-α-D-galactopyranosyl-(1→3)-2,4,6-tri-O-benzoyl-β-D-galactopyranoside (34) 48

N-benzyl-N-benzyloxycarbonyl-6-aminohexyl 3-O-acetyl-2-O-benzoyl-4,6-di-O-benzyl-β-D-galactopyranosyl-(1→4)-2,3-di-O-benzyl-α-D-galactopyranosyl-(1→3)-2,4,6-tri-O-benzoyl-β-D-galactopyranoside (35) 49

N-benzyl-N-benzyloxycarbonyl-6-aminohexyl 3-O-acetyl-2-O-benzoyl-4,6-di-O-benzyl-β-D-galactopyranosyl-(1→4)-2,3-di-O-benzyl-6-O-sulfo-α-D-galactopyranosyl-(1→3)-2,4,6-tri-O-benzoyl-β-D-galactopyranoside (36) 51

6-Aminohexyl β-D-galactopyranosyl-(1→4)-6-O-sulfo-α-D-galactopyranosyl-(1→3)-β-D-galactopyranoside (mSt) 52

N-benzyl-N-benzyloxycarbonyl-6-aminohexyl 4,6-di-O-benzoyl-2,3-di-O-benzyl-α-D-galactopyranosyl-(1→3)-2,4,6-tri-O-benzyl-β-D-galactopyranosyl-(1→4)-2,3-di-O-benzyl-6-O-levulinoyl-α-D-galactopyranosyl-(1→3)-2,4,6-tri-O-benzoyl-β-D-galactopyranoside (37) 53

N-benzyl-N-benzyloxycarbonyl-6-aminohexyl 4,6-di-O-benzoyl-2,3-di-O-benzyl-α-D-galactopyranosyl-(1→3)-2,4,6-tri-O-benzyl-β-D-galactopyranosyl-(1→4)-6-O-benzoyl-2,3-di-O-benzyl-α-D-galactopyranosyl-(1→3)-2,4,6-tri-O-benzoyl-β-D-galactopyranoside (38) 54

N-benzyl-N-benzyloxycarbonyl-6-aminohexyl 4,6-di-O-benzoyl-2,3-di-O-benzyl-α-D-galactopyranosyl-(1→3)-2-O-benzoyl-4,6-di-O-benzyl-β-D-galactopyranosyl-(1→4)-2,3-di-O-benzyl-6-O-levulinoyl-α-D-galactopyranosyl-(1→3)-2,4,6-tri-O-benzoyl-β-D-galactopyranoside (39) 55

6-Aminohexyl α-D-galactopyranosyl-(1→3)-β-D-galactopyranosyl-(1→4)-α-D-galactopyranosyl-(1→3)-β-D-galactopyranoside (nST) 56

N-benzyl-N-benzyloxycarbonyl-6-aminohexyl 4,6-di-O-benzoyl-2,3-di-O-benzyl-α-D-galactopyranosyl-(1→3)-2-O-benzoyl-4,6-di-O-benzyl-β-D-galactopyranosyl-(1→4)-2,3-di-O-benzyl-α-D-galactopyranosyl-(1→3)-2,4,6-tri-O-benzoyl-β-D-galactopyranoside (40) 57

N-benzyl-N-benzyloxycarbonyl-6-aminohexyl 4,6-di-O-benzoyl-2,3-di-O-benzyl-α-D-galactopyranosyl-(1→3)-2-O-benzoyl-4,6-di-O-benzyl-β-D-galactopyranosyl-(1→4)-2,3-di-O-benzyl-6-O-sulfo-α-D-galactopyranosyl-(1→3)-2,4,6-tri-O-benzoyl-β-D-galactopyranoside (41) 58

6-Aminohexyl α-D-galactopyranosyl-(1→3)-β-D-galactopyranosyl-(1→4)-6-O-sulfo-α-D-galactopyranosyl-(1→3)-β-D-galactopyranoside (mST) 59

Electrochemical Sensing 61

X-ray Photoelectron Spectroscopy Analysis 67

References 68

Experimental Section

General methods

Reagent-grade commercial chemicals were used without purification unless stated otherwise. Solvents were dehydrated if necessary, according to the procedure using molecular sieves (4Å, unless stated otherwise). Reactions were carried out under Ar or N_2 atmosphere. Thin-layer chromatography (TLC) was carried out on aluminum sheets coated with Silica gel 60 F_{254} (TLC, Merck) or Silica gel 60 RP-18 F_{254}S (RP-TLC, Merck). TLC plates were developed by treatment with *p*-anisaldehyde stain. Flash chromatography was performed in silica gel columns with ethyl acetate (EtOAc) hexane gradient, elution is reported for % EtOAc by volume. Preparative HPLC (Prep-HPLC) was performed on Phenomenex Luna 5μm C18 or Phenomenex Luna 5μm C8 columns with acetonitrile (ACN) and triply-distilled water (TDW) gradient with 0.1% (*v*/*v*) trifluoroacetic acid (TFA), elution is reported for % ACN by volume. Analytical HPLC was performed in XTerra 5μm RP-8, 4.6x150mm column with ACN:TDW gradient with 0.1% (*v*/*v*) TFA at 1ml/min flow. Matrix-associated laser desorption/ionization (MALDI) was performed on Bruker autoflex maX on a ground steel plate, using graphite (4B pencil lead) as matrix. Low resolution LC-MS was performed on ExionLC AE (LC), Triple Quad 3500 (AcQuRate pulse-counting channel electron multiplier (QqQ-CEM) MS) (SCIEX). LC-HRMS was performed on Dionex UltiMate 3000 UPLC (LC), Q Exactive Plus (Q-Orbitrap HRMS) (Thermo Fisher Scientific) or on ExionLC AE (LC), X500R Q TOF (HRMS) (SCIEX). NMR spectra were recorded on Bruker Avance-IV-500 (500MHz), Bruker Avance Nanobay (400MHz), Bruker Avance-III (700MHz, CryoProbe), or Bruker Avance Neo (700MHz, Prodigy CryoProbe) spectrometers. The structural assignments of ^{1}H and ^{13}C spectra were made with additional information from gCOSY, gHSQC, and gHMBC. Stereochemistry of glycosidic bonds was assigned with additional information from non-decoupled gHSQC, non-decoupled gHMQC, or non-decoupled unfiltered gHMBC. For unfiltered $^{1}J_{CH}$ non-decoupled HMBC, a modified experiment without low-pass *J*-filter was used (no suppression of one-bond correlation). Chemical shifts are reported in ppm, referenced to solvent residual peak (^{1}H-NMR δ 7.26/^{13}C-NMR δ 77.1 for $CDCl_3$, ^{1}H-NMR δ 2.05/^{13}C-NMR δ 29.8 for d_6-acetone, ^{1}H-NMR δ 3.31/^{13}C-NMR δ 49.0 for d_4-MeOD, ^{1}H-NMR δ 8.03/^{13}C-NMR δ 163.2 for d_7-N,N-dimethylformamide (d_7-DMF), ^{1}H-NMR δ 4.79 for D_2O; ^{13}C-NMR in D_2O was calibrated for acetate δ 21.0 or formate δ 166.3 aminium counterions).[1,2]

Building block synthesis

Scheme S1. Outline of building block synthesis.

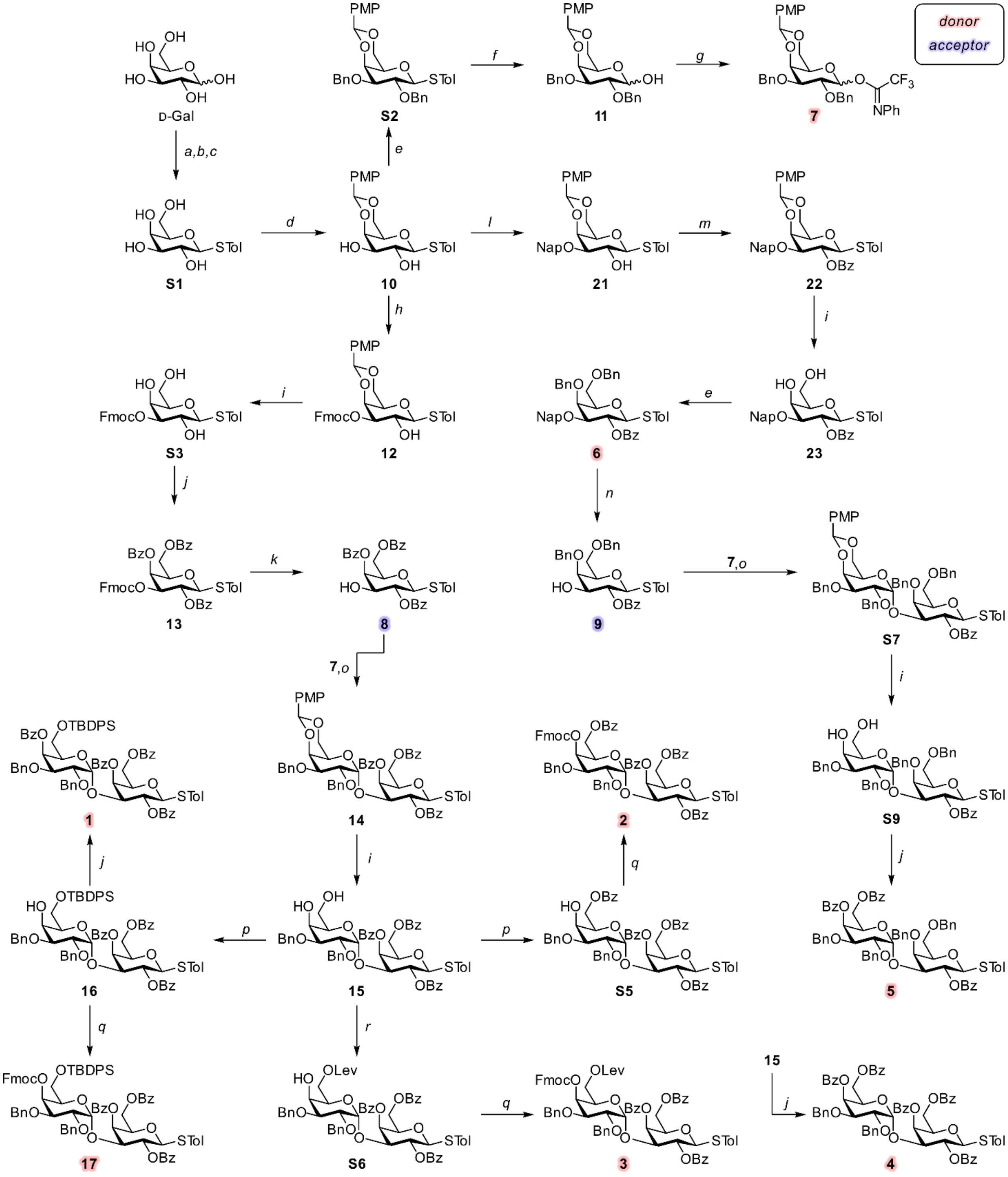

a. Ac_2O, BF_3·Et2O; *b.* TolSH; *c.* NaOMe, MeOH; *d.* $PMPCH(OMe)_2$, CSA, ACN; *e.* BnBr, NaH, DMF; *f.* NIS, H_2O, THF; *g.* $CF_3CNPhCl$, DBU, DCM; *h.* Bu_2SnO, FmocCl, toluene; *i.* TFA, H_2O, DCM; *j.* BzCl, DMAP, pyridine, DCM; *k.* morpholine, DMF; *l.* Bu_2SnO, NapBr, CsF, toluene; *m.* BzCl, TEA, DMAP, pyridine, DCM; *n.* DDQ, DCM; *o.* TfOH, Et_2O; *p.* BzOH, DABCO, CMPI, DCM; *q.* FmocCl, DMAP, pyridine, DCM; *r.* LevOH, DABCO, CMPI, DCM. *Compounds used as glycosyl donor are highlighted in red; compounds used as glycosyl acceptors are highlighted in blue.*

p-Tolyl-1-thio-β-D-galactopyranoside (S1)

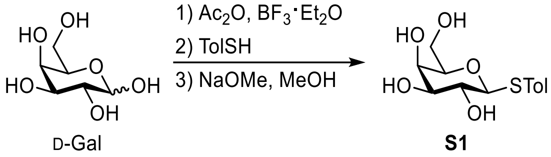

Thiogalactoside **S1** was synthesized according to a previously reported procedure,[3] *via* a sequential one-pot thioglycosylation,[4] followed by deacetylation. D-Galactose (10.1 g, 56.1 mmol) was suspended in acetic anhydride (Ac_2O) (28 ml, 296 mmol) and cooled to 0°C in an ice-water bath. $BF_3{\cdot}Et_2O$ (12 ml, 97 mmol) was added dropwise over 15 min. An exothermic reaction occurred, the reaction mixture was allowed to reach 30°C, slowly over 2h. After 2h the solution appeared light-amber and clear, and the formation of per-acetylated galactose was complete (as indicated by TLC, 40% EtOAc in hexane Rf=0.44). p-Thiocresol (TolSH) (7.6 g, 61 mmol) was added in one portion. The per-acetylated galactose was mostly consumed and acetylated thioglycoside was formed after 24h at 30°C (as indicated by TLC, 40% EtOAc in hexane R_f=0.68). The reaction mixture was diluted with dichloromethane (DCM) (50 ml), then carefully quenched with sat. $NaHCO_3$. The organic layer was separated, washed with sat. $NaHCO_3$, then brine, dried over Na_2SO_4 and filtered. The organic layer was concentrated under reduced pressure to yield the crude p-tolyl 2,3,4,6-O-tetraacetyl-1-thio-β-D-galactopyranoside as a yellow syrup (25.4 g). The crude acetylated thioglycoside was dissolved in anhydrous DCM and anhydrous MeOH (300 ml, 1:1) and cooled to 0°C in an ice-water bath. Sodium methoxide (NaOMe) (1.59 g, 29.4 mmol) was added and the reaction mixture was allowed to reach ambient room temperature (RT). After stirring at RT for 3h the solution was turbid and the reaction was complete (as indicated by TLC, 10% MeOH in DCM, R_f=0.48). Ion exchange beads Amberlight® XAD16N (H-form, pre-washed in DCM and MeOH) were added in small portions until neutral pH was reached and reaction mixture turned clear. The reaction mixture was filtered and concentrated under reduced pressure to produce a yellow syrup. The residue was triturated with n-hexane repeatedly to remove residual TolSH until the decanted solution was colorless. The syrup-like residue was dissolved in a minimal amount of n-butanol (~150 ml) and vacuum filtered (to remove precipitated D-galactose), then the filtrate was concentrated under reduced pressure until almost solvent-free. n-Hexane was added, resulting in the crashing out of an off-white solid. The supernatant solution was decanted and the off-white solid was dried under vacuum to obtain sufficiently pure thioglycoside **S1** (15.03 g, 3-step yield 94%). HRMS (ESI-QTOF) m/z: $[M+H]^+$ Calcd for $C_{13}H_{19}O_5S$ 287.0948; found 287.0940. ^1H NMR (400MHz, d_4-MeOD): δ, ppm 7.45 (m, 2H, o-Tol), 7.11 (m, 2H, m-Tol), 4.50 (d, $J_{1,2}$ = 9.6 Hz, 1H, H-1), 3.89 (dd, $J_{3,4}$ = 3.3 Hz, $J_{4,5}$ = 1.0 Hz, 1H, H-4), 3.75, 3.70 (ABdq, $\Delta\delta_{AB}$ = 0.05, J_{AB} = 11.4 Hz, $J_{5,6A}$ = 6.9 Hz, $J_{5,6B}$ = 5.4 Hz, 2H, H-6), 3.57 (dd, $J_{1,2}$ = $J_{2,3}$ = 9.4 Hz, 1H, H-2), 3.53 (m, 1H, H-5), 3.48 (dd, $J_{2,3}$ = 9.2 Hz, $J_{3,4}$ = 3.3 Hz, 1H, H-3), 2.31 (s, 3H, S-Ph-C***H*****3**). $^{13}C\{^1H\}$ NMR (101MHz, d_4-MeOD): 138.4 (p-Tol), 132.9 (o-Tol), 132.1 (i-Tol), 130.5 (m-Tol), 90.7 (C-1), 80.6 (C-5), 76.4 (C-3), 71.0 (C-2), 70.4 (C-4), 62.6 (C-6), 21.1 (S-Ph-**C**H_3). The ^1H-NMR and ^{13}C-NMR spectra were in agreement with the literature.[5]

p-Tolyl 4,6-O-(4-methoxyphenyl)methylene-1-thio-β-D-galactopyranoside (10)

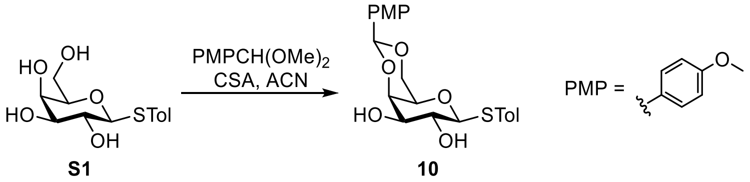

Compound **S1** (12.4 g, 43.2 mmol) was suspended in anhydrous ACN (140 ml) and cooled to 0°C in an ice-water bath. Anisaldehyde dimethyl acetal ($PMPCH(OMe)_2$) (8.75 ml, 52.0 mmol) and camphorsulfonic acid (CSA) (2.00 g, 8.48 mmol) were added. The acidic reaction mixture (pH~1) turned bright purple. The ice-water bath was removed and the

reaction mixture was stirred at RT for 2.5h until a major product was formed (as indicated by TLC, 5% MeOH in DCM, R_f=0.33). The reaction was cooled to 0°C in an ice-water bath, then quenched by the addition of triethylamine (TEA) (~1.3 ml) until a neutral pH was reached, accompanied by a color change from purple to light yellow. The solvent was removed under reduced pressure. The crude product was re-dissolved in DCM (120 ml). The organic layer was washed with TDW (x3), then sat. $NaHCO_3$ (x3) and brine. The organic phase was dried over Na_2SO_4, filtered and evaporated to produce an off-white solid. The crude solid was triturated repeatedly with 10% EtOAc in hexane until the decanted solution was colorless to produce sufficiently pure **10** (15.0 g, yield 86%), which was used later without further purification. HRMS (ESI-Q-Orbitrap) m/z: $[M+H]^+$ Calcd for $C_{21}H_{25}O_6S$ 405.1366; found 405.1367. ^1H NMR (500MHz, $CDCl_3$): δ , ppm 7.58 (m, 2H, *o*-Tol), 7.31 (m, 2H, *o*-PMP), 7.13 (m, 2H, *m*-Tol), 6.87 (m, 2H, *m*-PMP), 5.46 (s, 1H, PMPC***H***OO), 4.45 (d, $J_{1,2}$ = 9.2 Hz, 1H, H-1), 4.36, 4.01 (ABdq, $\Delta\delta_{AB}$ = 0.35, J_{AB} = 12.5 Hz, $J_{5,6}$ = 1.6 Hz, 2H, H-6), 4.21 (dd, $J_{3,4}$ = 3.5 Hz, $J_{4,5}$ = 1.0 Hz, 1H, H-4), 3.82 (s, 3H, ***Me***OPhCHOO), 3.69 (dd, $J_{3,4}$ = 3.4 Hz, $J_{2,3}$ = 9.2 Hz, 1H, H-3), 3.62 (dd, $J_{1,2}$ = $J_{2,3}$ = 9.2 Hz, 1H, H-2), 3.53 (m, 1H, H-5), 2.61-2.40 (br, 2H, 2-O***H***; 3-O***H***), 2.37 (s, 3H, S-Ph-C***H***$_3$), residual anisaldehyde (~1:0.03 ratio; δ 9.89 (s), 7.85 (d), 7.00 (d), 3.90 (s)). $^{13}C\{^1H\}$ NMR (126 MHz, $CDCl_3$): δ , ppm 160.4 (*p*-PMP), 138.6 (*i*-Tol), 134.5 (*o*-Tol), 130.3 (*i*-PMP), 129.8 (*m*-Tol), 128.0 (*o*-PMP), 126.7 (*p*-Tol), 113.6 (*m*-PMP), 101.4 (PMP**C**HOO), 87.1 (C-1), 75.4 (C-4), 73.8 (C-3), 70.1 (C-5), 69.4 (C-6), 68.9 (C-2), 55.4 (***Me***OPhCHOO), 21.4 (S-Ph-**C**H$_3$). The ^1H-NMR and ^{13}C-NMR spectra were in agreement with the literature.[6]

***p*-Tolyl 2,3-di-O-benzyl-4,6-O-(4-methoxyphenyl)methylene-1-thio-β-D-galactopyranoside (S2)**

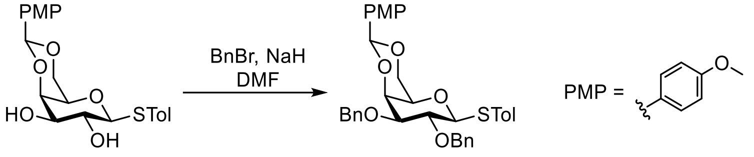

10 **S2**

Compound **10** (9.81 g, 24.3 mmol) was dissolved in anhydrous DMF (200 ml) and cooled to 0°C in an ice-water bath. NaH (60% dispersion in mineral oil, 2.23 g, 55.9 mmol) was added in three portions, followed by the addition of benzyl bromide (BnBr) (6.1 ml, 51 mmol). The reaction was stirred at RT for 3h until completion (as indicated by TLC, 30% EtOAc in hexane, R_f=0.30). The reaction was diluted with DCM and carefully poured onto dilute aqueous HCl (0.01 M). The organic layer was separated and washed several times with TDW until no residual reactant was observed in the organic phase (as indicated by TLC). The organic layer was washed with brine, dried over Na_2SO_4, filtered and the solvent was evaporated. The crude solid was triturated with hexane. Sufficiently pure **S2** (12.9 g, yield 91%) was obtained and used later without further purification. HRMS (ESI-Q-Orbitrap) m/z: $[M+H]^+$ Calcd for $C_{35}H_{37}O_6S$ 585.2305; found 585.2307. ^1H NMR (500MHz, *d*$_6$-acetone): δ , ppm 7.60 (m, 2H, *o*-Tol), 7.46 (m, 2H, *o*-PMP), 7.41 - 7.35 (m, 4H, Bn), 7.33 - 7.24 (m, 6H, Bn), 7.07 (m, 2H, *m*-Tol), 6.97 (m, 2H, *m*-PMP), 5.61 (s, 1H, PMPC***H***OO), 4.77, 4.66 (ABq, $\Delta\delta_{AB}$ = 0.11, J_{AB} = 12.1 Hz, 2H, 3-O-C***H***$_2$-Ph), 4.71, 4.64 (ABq, $\Delta\delta_{AB}$ = 0.07, J_{AB} = 10.7 Hz, 2H, 2-O-C***H***$_2$-Ph), 4.70 (d, $J_{1,2}$ = 9.2 Hz, 1H, H-1), 4.55 (dd, $J_{3,4}$ = 3.3 Hz, $J_{4,5}$ = 1.0 Hz, 1H, H-4), 4.23, 4.11 (ABdq, $\Delta\delta_{AB}$ = 0.12, J_{AB} = 12.3 Hz, $J_{5,6}$ = 1.7 Hz, 2H, H-6), 3.83 (s, 3H, ***Me***OPhCHOO), 3.79 (dd, $J_{2,3}$ = 9.2 Hz, $J_{3,4}$ = 3.3 Hz, 1H, H-3), 3.75 (dd, $J_{1,2}$ = $J_{2,3}$ = 9.2 Hz, 1H, H-2), 3.66 (dd, $J_{3,4}$ = 2.6 Hz, $J_{5,6}$ = 1.5 Hz, 1H, H-5), 2.30 (s, 3H, S-Ph-C***H***$_3$). $^{13}C\{^1H\}$ NMR (126 MHz, , *d*$_6$-acetone): δ , ppm 161.0 (*p*-PMP), 140.1 (Bn), 139.8 (Bn), 137.9 (*i*-Tol), 133.2 (*o*-Tol), 132.4 (Bn), 130.9 (*p*-Tol), 130.4 (*m*-Tol), 129.0 (Bn), 128.9 (*i*-PMP), 128.7 (*o*-PMP), 128.4 (Bn), 128.2 (Bn), 128.2 (Bn), 114.1 (*m*-PMP), 101.4 (PMP**C**HOO), 87.2 (C-1), 82.4 (C-3), 76.6 (C-2), 75.7 (3-O-**C**H$_2$-Ph), 73.8 (2-O-**C**H$_2$-Ph), 71.4 (C-4), 70.6 (C-5), 70.0 (C-6), 55.6 (***Me***OPhCHOO), 21.1 (S-Ph-**C**H$_3$).

2,3-di-O-benzyl-4,6-O-(4-methoxyphenyl)methylene-1-thio-D-galactopyranose (11)

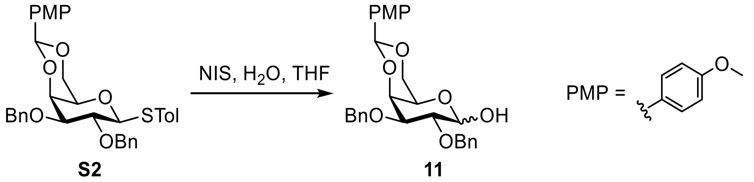

Compound **S2** (1.34 g, 2.29 mmol) was dissolved in tetrahydrofuran (THF) (20 ml) and TDW (5 ml), and cooled to 0°C in an ice-water bath. N-iodosuccinimide (NIS) (1.55 g, 6.89 mmol) was added. The reaction was complete after stirring for 1h at 0°C (as indicated by TLC, 50% EtOAc in hexane, R_f=0.59). The reaction was diluted with DCM and washed with aq. $Na_2S_2O_3$ (10% w/w) and brine, dried over Na_2SO_4, filtered. The solvent was evaporated to produce a crude solid, which was repeatedly triturated with hexane until the decanted solution was clear. The solid product was redissolved and evaporated from toluene to obtain a sufficiently pure off-white, solid compound **11** (1.08 g, yield 98%) as an 3α:2β anomeric mixture and used later without further purification. HRMS (ESI-Q-Orbitrap) m/z: $[M+H]^+$ Calcd for $C_{28}H_{31}O_7$ 479.2064; found 479.2068. ^{1}H NMR (500MHz, d_6-acetone): δ , ppm 7.45 - 7.39 (m, 6H), 7.33 - 7.22 (m, 6H), 6.93 - 6.89 (m, 2H), 5.85 (d, $J_{1\beta,OH}$= 7.4Hz, 1H, 1-O***H***(Galβ)), 5.59 (s, 1H, PMPC***H***OO(Galβ)), 5.57 (s, 1H, PMPC***H***OO(Galα)), 5.39 (dd, $J_{1,2}$= $J_{1\alpha,OH}$ = 4.0 Hz, 1H, H-1(Galα)), 5.34 (dd, $J_{1\alpha,OH}$ = 3.9 Hz, $J_{2,OH}$ = 0.8 Hz, 1H, 1-O***H***(Galα)), 4.95 - 4.70 (m, 3/2-O-C***H***$_2$-Ph(Galα/β)), 4.68 (m, 1H, H-1(Galβ)), 4.51 (dd, $J_{3,4}$ = 3.6 Hz, $J_{4,5}$ = 1.2 Hz, 1H, H-4(Galα)), 4.46 (dd, $J_{3,4}$ = 3.6 Hz, $J_{4,5}$ = 1.1 Hz, 1H, H-4(Galβ)), 4.17-4.05 (m, H-6(Galα); H-6(Galβ); H-3(Galα)), 3.93 (dd, 1H, $J_{2,3}$ = 10.1Hz, $J_{5,6}$ = 3.4Hz, 1H, H-2(Galα)), 3.90 (m, 1H, H-5(Galα)), 3.80 (s, 3H, ***Me***OPhCHOO(Galβ)), 3.79 (s, 3H, ***Me***OPhCHOO(Galα)), 3.69 (dd, $J_{2,3}$ = 9.7Hz, $J_{3,4}$ = 3.6Hz, 1H, H-3(Galβ)), 3.75 (m, 1H, H-2(Galβ)), 3.53 (m, 1H, H-5(Galβ)), 2.30 (s, 3H, S-Ph-C***H***$_3$). ^{13}C{^{1}H} NMR (126 MHz, d_6-acetone): δ , ppm 160.8 (*p*-PMP(Galβ)), 160.8 (*p*-PMP(Galα)), 140.6, 140.4, 140.3, 140.1, 132.5, 132.4, 128.9, 128.9, 128.8, 128.4, 128.4, 128.3, 128.3, 128.3, 128, 127.9, 114.0, 101.2 (PMP**C**HOO(Galβ)), 101.2 (PMP**C**HOO(Galα)), 98.4 (C-1(Galβ)), 92.6 (C-1(Galα)), 81, 80.6, 77.1, 76.6, 75.3, 74.8, 74, 73.3, 71.5, 71.4, 70.1, 70, 67.2, 63.2, 55.5. *Note: The integration of ^{1}H-NMR was calibrated for a total of 1H for the sum of PMPC**H**OO signals of both anomers (vide infra, ^{1}H-NMR spectrum **11**). The integration assignment in ^{1}H-NMR is written for unambiguous cases only.*

2,3-Di-O-benzyl-4,6-O-(4-methoxyphenyl)methylene-1-O-(2,2,2-trifluoro-N-phenylacetimidoyl)-α/β-D-galactopyranoside (7)

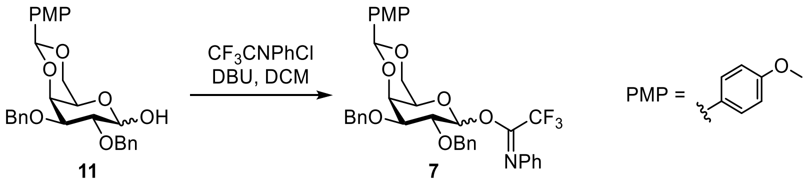

Compound **11** (1.25 g, 2.61 mmol) was dissolved in anhydrous DCM (30 ml) and cooled to 0°C in an ice-water bath. 2,2,2-Trifluoro-N-phenylacetimidoyl chloride (1.25 ml, 8.00 mmol) and 1,8-diazabicyclo[5.4.0]undec-7-ene (DBU) (0.43 ml, 2.9 mmol) were added. The starting material was completely consumed after 1h (as indicated by TLC). The reaction mixture was diluted with DCM (~20 ml). TEA (1 ml) was added, and the solution was evaporated directly on silica gel 60 (~6 g, 40-63 μm). The product was purified by flash chromatography (dry loading method) to obtain **7** (1.58 g, 92% yield, elution at 30%-40% EtOAc in hexane) as two separable anomers (1:2, unassigned). The anomers were combined and used as a mixture in the next steps. MS (MALDI-TOF) m/z: $[M+K]^+$ Calcd for $C_{36}H_{34}F_3KNO_7$ 688.192; found 688.033. Anomer A (major): ^{1}H NMR (500MHz, d_6-acetone): δ , ppm 7.46 (m, 2H, *o*-PMP), 7.41 (m, 2H), 7.38 - 7.22 (m, 11H), 7.18 - 7.11 (m, 2H), 6.94 (m, 2H, *m*-PMP), 6.90 (br, 2H), 5.62 (s, 1H, PMPC***H***OO), 4.82 (ABdq, $\Delta\delta_{AB}$= 0.07, J_{AB}= 11.2 Hz, 2H, 2/3-O-C***H***$_2$-Ph), 4.76 (ABdq, $\Delta\delta_{AB}$= 0.11, J_{AB}= 12.1 Hz, 2H, 2/3-O-C***H***$_2$-Ph), 4.55 (br, 1H), 4.20 - 4.11 (m, 2H, H-6), 3.95 -

3.92 (br, 2H), 3.81 (s, 3H, ***Me***OPhCHOO). $^{13}C\{^1H\}$ NMR (126 MHz, d_6-acetone): δ , ppm 161.1 (*i*-PMP), 144.7, 139.8, 139.8, 132.2, 129.8, 129.6, 129.1, 129.0, 128.6, 128.5, 128.3, 128.3, 125.1, 120.2, 114.2, 101.5 (PMP**C**HOO), 80.5, 78.4, 75.8 (2/3-O-**C**H_2-Ph), 73.5, 71.7 (2/3-O-**C**H_2-Ph), 69.4 (C-6), 68.4, 55.6 (***Me***OPhCHOO). ^{19}F NMR (470 MHz, d_6-acetone): δ -71.40 (C***F***$_3$). Anomer B (minor): ^1H NMR (500 MHz, d_6-acetone): δ , ppm 7.44 - 7.39 (6H, m), 7.36 - 7.27 (8H, m), 7.11 (t, *J* = 7.5 Hz, 1H), 6.91 (d, *J* = 8.8 Hz, 2H), 6.81 - 6.80 (2H, m), 5.63 (s, 1H, PMPC***H***OO), 4.84 (ABdq, $\Delta\delta_{AB}$ = 0.07, J_{AB}= 12.5 Hz, 2H, 2/3-O-C***H***$_2$-Ph), 4.79 (ABdq, $\Delta\delta_{AB}$= 0.08, J_{AB}= 12.0 Hz, 2H, 2/3-O-C***H***$_2$-Ph), 4.68 - 4.66 (1H, br, H-4), 4.19 - 4.12 (4H, m, H-6; H-2; H-3), 3.94 - 3.94 (1H, br, H-5), 3.80 (s, 3H, ***Me***OPhCHOO). $^{13}C\{^1H\}$ NMR (126 MHz, d_6-acetone): δ , ppm 160.9 (*p*-PMP), 144.8, 139.9, 139.8, 132.1, 129.6, 129.0, 129.0, 128.4, 128.3, 128.2, 128.2, 128.1, 125.0, 120.2, 114.0, 101.3 (PMP**C**HOO), 76.6, 75.7, 74.1 (2/3-O-**C**H_2-Ph), 74.0, 71.6 (2/3-O-**C**H_2-Ph), 69.5, 66.4, 55.5.
Note: The anomeric H-1 was not assigned in ^1H-NMR due to broadening of the signal.

p-Tolyl 3-O-(9-fluorenylmethyloxycarbonyl)-4,6-O-(4-methoxyphenyl)methylene-1-thio-β-D-galactopyranoside (12)

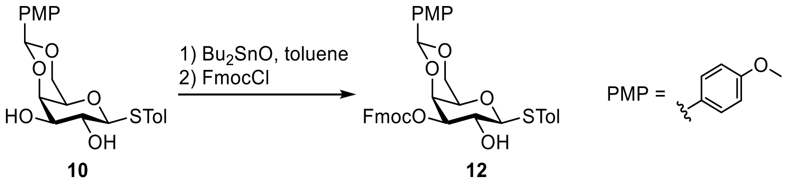

Compound **10** (1.59 g, 3.93 mmol) and dibutyltin oxide (1.03 g, 4.14 mmol) were suspended in anhydrous toluene (70 ml). The reaction mixture was heated in an oil bath and refluxed for 3h in a Dean-Stark apparatus equipped with a drying tube containing $CaCl_2$ until no further water collection was observed in the Dean-Stark trap. The reaction mixture was allowed to cool to 0°C and 9-fluorenylmethoxycarbonyl chloride (FmocCl) (1.32 g, 5.10 mmol) was added. The reaction mixture was stirred for 1h at 0°C until completion (as indicated by TLC, 40% EtOAc in hexane, R_f=0.35). The reaction mixture was evaporated directly on silica gel 60 (~8 g, 40-63 μm) and purified by flash chromatography (dry loading method) to obtain **12** (1.99 g, 81% yield, elution at 50% EtOAc in hexane). HRMS (ESI-Q-Orbitrap) m/z: $[M+H]^+$ Calcd for $C_{36}H_{35}O_8S$ 627.2047; found 627.2049. ^1H NMR (500 MHz, d_6-acetone): δ , ppm 7.86 - 7.82 (m, 2H), 7.63 - 7.61 (m, 2H), 7.59 - 7.57 (m, 2H), 7.42 - 7.38 (m, 2H), 7.36 - 7.33 (m, 2H), 7.30 - 7.25 (m, 2H), 7.12 - 7.10 (m, 2H), 6.93 - 6.91 (m, 2H), 5.53 (s, 1H, PMPC***H***OO), 4.79 (dd, $J_{3,4}$ = 3.5, $J_{2,3}$ = 9.7 Hz, 1H, H-3), 4.71 (d, $J_{1,2}$ = 9.5 Hz, 1H, H-1), 4.63 (d, $J_{2,OH}$ = 5.1 Hz, 1H, 2-O***H***), 4.50, 4.44 (ABq, $\Delta\delta_{AB}$ = 0.05, J_{AB} = 10.6 Hz, $J_{1,9'}$ = 7.0 Hz, 2H, -C***H***$_2$- (Fmoc)), 4.41 (dd, $J_{4,5}$ = 1.0, $J_{3,4}$ = 3.5 Hz, 1H, H-4), 4.28 (t, $J_{1,9'}$ = 6.9 Hz, 1H, -C***H***- (Fmoc)), 4.21, 4.12 (ABq, $\Delta\delta_{AB}$ = 0.09, J_{AB} = 12.3 Hz, $J_{5,6}$ = 1.7 Hz, 2H, H-6), 3.87 (dt, $J_{2,OH}$ = 5.2, $J_{2,3}$ = $J_{1,2}$ = 9.6 Hz, 1H, H-2), 3.83 (s, 3H, ***Me***OPhCHOO), 3.78 - 3.77 (m, 1H, H-5), 2.34 (s, 3H, S-Ph-C***H***$_3$), residual DCM (~1:0.07 ratio, δ 5.62 (s)); H_2O and HOD (δ 2.81 (s); δ 2.78 (t)). $^{13}C\{^1H\}$ NMR (126 MHz, d_6-acetone): δ , ppm 161.0 (*p*-PMP), 155.3 (C=O (Fmoc)), 144.5, 144.3, 142.1, 142.1, 138.3, 134.2, 132, 130.3, 129.4, 128.7, 128.6, 128.0, 126.0, 125.9, 120.9, 120.9, 114.0, 101.3 (PMP**C**HOO), 88.1 (C-1), 79.9 (C-3), 74.4 (C-4), 70.2 (C-5), 70.1 (-**C**H_2- (Fmoc)), 69.7 (C-6), 66.5 (C-2), 55.6 (***Me***OPhCHOO), 47.5 (-**C**H- (Fmoc)), 21.1 (S-Ph-**C**H_3).

p-Tolyl 3-O-(9-fluorenylmethyloxycarbonyl)-1-thio-β-D-galactopyranoside (S3)

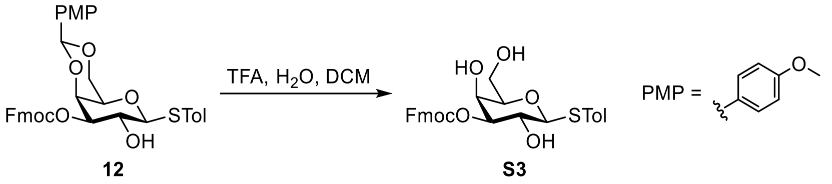

Compound **12** (2.56 g, 4.08 mmol) was dissolved in DCM (70 ml) and cooled to 0°C in an ice-water bath. TDW (0.35 ml) was added, followed by the addition of trifluoroacetic acid (TFA) (7.5 ml) dropwise over 15 min. The reaction mixture acquired a bright maroon color. The reaction was allowed to reach RT slowly over 2.5h, at which point the reaction was complete (as indicated by TLC, 50% EtOAc in hexane, R_f=0.19). The reaction mixture was diluted with DCM (~80 ml), and the organic layer was carefully washed with sat. $NaHCO_3$ until the aqueous layer registered pH~8. The organic layer was washed with brine, dried over Na_2SO_4, filtered and evaporated to yield an off-white solid. The crude product was repeatedly triturated with 10% EtOAc in hexane until the decanted solution was colorless and clear to yield sufficiently pure **S3** as an off-white solid (2.06 g, 99% yield). HRMS (ESI-Q-Orbitrap) m/z: $[M+H]^+$ Calcd for $C_{28}H_{29}O_7S$ 509.1629; found 509.1621. ^{1}H NMR (500 MHz, CDCl3): δ, ppm 7.78 - 7.75 (m, 2H), 7.63 - 7.59 (m, 2H), 7.47 - 7.45 (m, 2H), 7.43 - 7.38 (m, 2H), 7.33 - 7.29 (m, 2H), 7.16 - 7.13 (m, 2H), 4.72 (dd, $J_{3,4}$ = 3.1 Hz, $J_{1,2}$ = 9.6 Hz, 1H, H-3), 4.54 (d, $J_{1,2}$ = 9.6 Hz, 1H, H-1), 4.49, 4.45 (ABq, $\Delta\delta_{AB}$ = 0.05, J_{AB} = 10.5 Hz, $J_{1,9'}$ = 7.2 Hz, 2H, -C***H***$_2$- (Fmoc)), 4.27 (t, $J_{1,9'}$ = 7.1 Hz, 1H, -C***H***- (Fmoc)), 4.20 (dd, $J_{4,5}$ = 0.7, $J_{3,4}$ = 3.0 Hz, 1H, H-4), 3.95 (dd, J_{AB} = 11.9 Hz, $J_{5,6A}$ = 5.7 Hz, 1H, H-6A), 3.91 (t, $J_{1,2}$ = $J_{2,3}$ = 9.5 Hz, 1H, H-2), 3.87 (dd, J_{AB} = 11.9 Hz, $J_{5,6B}$ = 4.3 Hz, 1H, H-6B), 3.62 - 3.59 (m, 1H, H-5), 2.35 (s, 3H, S-Ph-C***H***$_3$), residual anisaldehyde (~1:0.1 ratio; δ 9.89 (s), 7.85 (d), 7.00 (d), 3.89 (s)). ^{13}C{^{1}H} NMR (126 MHz, $CDCl_3$): δ, ppm 154.6 (C=O (Fmoc)), 143.4, 143.2, 141.4, 141.4, 138.9, 133.5, 130.0, 128.0, 127.4, 127.3, 127.3, 125.2, 120.2, 120.2, 89.0 (C-1), 80.1 (C-3), 77.9 (C-5), 70.3 (-**C**H_2- (Fmoc)), 68.5 (C-4), 67.0 (C-2), 63.0 (C-6), 46.8 (-**C**H- (Fmoc)), 21.3 (S-Ph-**C**H_3), residual anisaldehyde (δ 191.0, 132.1, 127.4, 114.4, 113.5, 55.7).

p-Tolyl 2,4,6-tri-O-benzoyl-3-O-(9-fluorenylmethyloxycarbonyl)-1-thio-β-D-galactopyranoside (13)

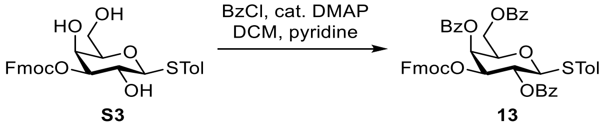

Compound **S3** (2.00 g, 3.54 mmol) was dissolved in anhydrous DCM (90 ml) and pyridine (18 ml). The reaction was cooled to 0°C in an ice-water bath and DMAP (40 mg, 0.33 mmol) and benzoyl chloride (BzCl) (1.85 ml, 15.9 mmol) were added. The reaction mixture was allowed to reach RT and stirred overnight until completion (as indicated by TLC, 30% EtOAc in hexane, R_f=0.34). The reaction mixture was diluted with DCM (100 ml) and washed with aqueous HCl (1 M). Small portions of aqueous HCl (1 M) were added and shaken with the organic phase until the aqueous layer registered pH~3. The organic layer was separated and washed with brine. A small amount of sat. $NaHCO_3$ (~1 ml) was added to neutralize the slightly acidic aqueous layer. The organic layer was separated, dried over Na_2SO_4, filtered and evaporated. Compound **13** was purified by flash chromatography (2.72 g, 94% yield, elution at 30% EtOAc in hexane). HRMS (ESI-Q-Orbitrap) m/z: $[M+H]^+$ Calcd for $C_{49}H_{41}O_{10}S$ 821.2415; found 821.2412. ^{1}H NMR (500 MHz, $CDCl_3$): δ, ppm 8.13 - 8.10 (m, 1H), 8.10 - 8.06 (m, 2H), 8.04 - 8.00 (m, 4H), 7.71 - 7.67 (m, 2H), 7.66 - 7.56 (m, 4H), 7.51 - 7.40 (m, 10H), 7.36 - 7.28 (m, 3H), 7.17 (dt, J = 1.0, 7.5 Hz, 1H), 7.06 (d, J = 7.9 Hz, 2H), 7.00 (dt, J = 1.0, 7.5 Hz, 1H), 5.99 (dd, $J_{4,5}$ = 0.7 Hz, $J_{3,4}$ = 3.4 Hz, 1H, H-4), 5.66 (t, $J_{1,2}$ = $J_{2,3}$ = 10.0 Hz, 1H, H-2), 5.26 (dd, $J_{3,4}$ = 3.3 Hz, $J_{2,3}$ = 10.0 Hz, 1H, H-3), 4.93 (d, $J_{1,2}$ = 9.9 Hz, 1H, H-1), 4.64 (dd, J_{AB} = 11.5 Hz, $J_{5,6A}$ = 7.1 Hz, 1H, H-6A), 4.43 (dd, J_{AB} = 11.5 Hz, $J_{5,6B}$ = 5.6 Hz, 1H, H-6B), 4.40 - 4.34 (m, 1H, -C***H***$_2$- (Fmoc)), 4.30 (m, 1H, H-5), 4.16 - 4.10 (m, 2H, -C***H***$_2$- (Fmoc); -C***H***- (Fmoc)), 2.35 (s, 3H, S-Ph-

C***H***$_3$). ^{13}C{^{1}H} NMR (126 MHz, CDCl$_3$): δ, ppm 166.1 (C=O (6-O-Bz)), 165.7 (C=O (4-O-Bz)), 165.1 (C=O (2-O-Bz)), 154.2 (C=O (Fmoc)), 143.5, 143.1, 141.2, 141.1, 138.7, 134.3, 133.8, 133.7, 133.5, 133.4, 130.3, 130.3, 130.1, 129.9, 129.7, 129.5, 129.5, 128.9, 128.6, 128.6, 128.6, 128.5, 127.8, 127.8, 127.7, 127.2, 127.1, 125.4, 125.3, 119.9, 119.9, 86.4 (C-1), 76.4 (C-3), 75.0 (C-5), 70.7 (-**C**H$_2$- (Fmoc)), 68.0 (C-2), 67.9 (C-4), 62.6 (C-6), 46.5 (-**C**H- (Fmoc)), 21.4 (S-Ph-**C**H$_3$).

***p*-Tolyl 2,4,6-tri-O-benzoyl-1-thio-β-D-galactopyranoside (8)**

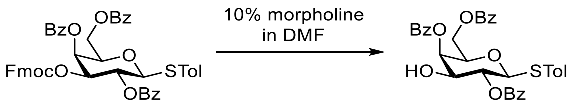

13 → **8**

Compound **13** (580 mg, 0.707 mmol) was dissolved in anhydrous DMF (27 ml) and cooled to 0°C in an ice-water bath. Morpholine (3.0 ml, 35 mmol, 10% in DMF) was added dropwise. The reaction mixture was stirred at 0°C for 15 min, then diluted with DCM (~30 ml) and washed with aqueous HCl (1 M). The layers were separated and the organic layer was washed with brine. A small amount of sat. NaHCO$_3$ (~1 ml) was added to neutralize the slightly acidic aqueous layer. The organic layer was separated, dried over Na$_2$SO$_4$, filtered and evaporated. Compound **8** was purified by flash chromatography (410 mg, 97% yield, elution at 40% EtOAc in hexane). HRMS (ESI-Q-Orbitrap) m/z: [M+H]$^+$ Calcd for C$_{34}$H$_{31}$O$_8$S 599.1734; found 599.1743. ^{1}H NMR (500 MHz, CDCl$_3$): δ, ppm 8.11 - 8.09 (m, 2H), 8.05 - 8.03 (m, 2H), 7.99 - 7.96 (m, 2H), 7.64 - 7.56 (m, 3H), 7.51 - 7.48 (m, 2H), 7.46 - 7.42 (m, 6H), 7.04 - 7.01 (m, 2H), 5.77 (dd, $J_{4,5}$ = 0.8 Hz, $J_{3,4}$ = 3.4 Hz, 1H, H-4), 5.27 (dd, $J_{1,2}$ = $J_{2,3}$ = 9.7 Hz, 1H, H-2), 4.88 (d, $J_{1,2}$ = 9.8 Hz, 1H, H-1), 4.57 (dd, J_{AB} = 11.5 Hz, $J_{5,6A}$ = 7.2 Hz, 1H, H-6A), 4.45 (dd, J_{AB} = 11.5 Hz, $J_{5,6B}$ = 5.4 Hz, 1H, 6-HB), 4.21 - 4.17 (m, 1H, H-5), 4.15 (dd, $J_{3,4}$ = 3.2 Hz, $J_{2,3}$ = 9.5 Hz, 1H, H-3), 2.34 (s, 3H, S-Ph-C***H***$_3$). ^{13}C{^{1}H} NMR (126 MHz, CDCl$_3$): δ, ppm 167.0 (C=O (2-O-Bz)), 166.2 (C=O (Bz)), 166.1 (C=O (Bz)), 138.7, 134.3, 133.7, 133.3, 130.2, 130.2, 130.1, 129.9, 129.7, 129.7, 129.4, 129, 128.6, 128.6, 128.5, 128.5, 127.5, 85.7 (C-1), 75.3 (C-5), 73.2 (C-3), 71.7 (C-2), 70.8 (C-4), 62.9 (C-6), 21.4 (S-Ph-**C**H$_3$). The ^{1}H-NMR spectrum was in agreement with the literature.[7]

Table S1. Acyl migration under Fmoc deprotection conditions

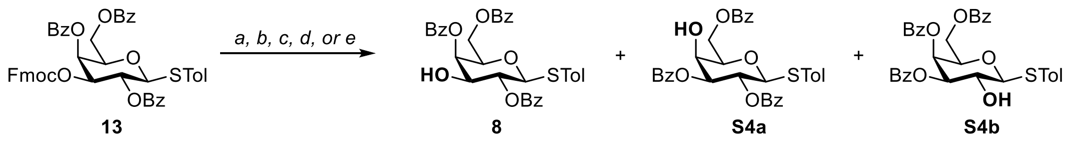

entry	conditions	time (min)	temperature (°C)	yield (%)			
				8	S4a	S4b	13
a	20% piperidine in DMF	20	RT	51[a]	27[b]	5[b]	-
b	10% piperidine in DMF	10	RT	59[a]	20[b]	2[b]	-
c	10% piperidine in DMF	10	0	76[a]	9[b]	<1[b]	-
d	20% triethylamine in DMF	20	RT	89[a c]	-	-	-
e	10% morpholine in DMF	20	0	97[a]	-	-	-

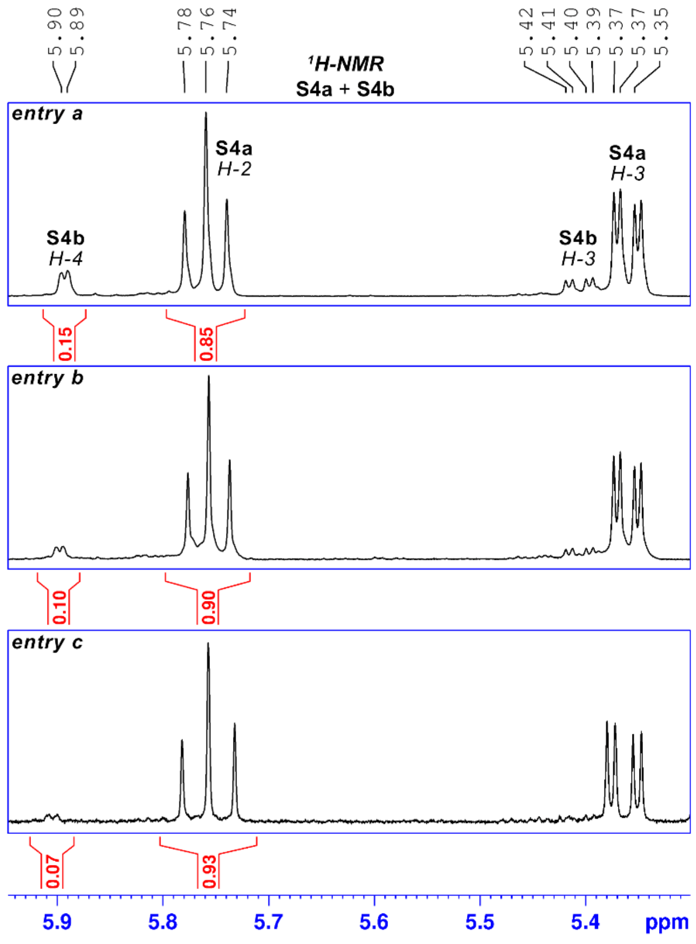

[a] Isolated yield; [b] Acyl migration byproducts **S4a** and **S4b** were obtained as an inseparable mixture by flash chromatography. The yields of **S4a** and **S4b** were calculated based on the combined yield of the mixture, multiplied by the ratio of **S4a** and **S4b**. The ratio of **S4a** and **S4b** was determined from the integration in ¹H-NMR of the mixture (see above), using assigned signals of H-2 and H-4 for **S4a** and **S4b**, respectively. Analytical amount of **S4a** and **S4b** was purified for characterization (see below). [c] Full conversion was confirmed by RP-HPLC. Yields were diminished due to the presence of insoluble byproducts that hampered purification.

Analytical amounts of acyl migration byproducts **S4a** and **S4b** were purified by Prep-HPLC, eluted at 76% and 74% ACN, respectively. 4→3 acyl migration byproduct **S4a**: HRMS (ESI-QTOF) m/z: $[M+H]^+$ Calcd for $C_{34}H_{31}O_8S$ 599.1734; found 599.1751. ^{1}H NMR (500 MHz, $CDCl_3$): δ, ppm 8.05 (dd, *J* = 1.3, 8.4 Hz, 2H), 7.99 (dd, *J* = 1.3, 8.4 Hz, 2H), 7.96 (dd, *J* = 1.3, 8.4 Hz, 2H), 7.63 - 7.58 (m, 1H), 7.55 - 7.50 (m, 4H), 7.49 - 7.44 (m, 4H), 7.41 - 7.34 (m, 6H), 6.98 (d, *J* = 7.9 Hz, 2H), 5.78 (dd, $J_{1,2}$ = $J_{2,3}$ = 10.0 Hz, 1H, H-2), 5.38 (dd, $J_{3,4}$ = 3.1 Hz, $J_{2,3}$ = 9.9 Hz, 1H, H-3), 4.91 (d, $J_{1,2}$ = 10.0 Hz, 1H, H-1(Galβ)), 4.66 (dABq, $\Delta\upsilon_{AB}$ = 21.7 Hz, J_{AB} = 11.6 Hz, $J_{5,6}$ = 6.3 Hz, 2H, H-6), 4.39 (d, $J_{3,4}$ = 2.6 Hz, 1H, H-4), 4.11 (m, 1H), 2.51 - 2.51 (br, 1H, 4-O***H***), 2.29 (s, 3H, S-Ph-C***H***$_3$). ^{13}C{^{1}H} NMR (126 MHz, $CDCl_3$): δ, ppm 166.5 (C=O (6-O-Bz)), 165.8 (C=O (3-O-Bz)), 165.4 (C=O (2-O-Bz)), 138.4, 133.6, 133.4, 133.3, 133.2, 129.9, 129.9, 129.9, 129.7, 129.7, 129.5, 129.0, 128.8, 128.6, 128.5, 128.5, 87.2 (C-1(Galβ)), 76.3 (C-5), 75.3 (C-3), 68.0 (C-2), 67.7 (C-4), 63.3 (C-6), 21.2 (S-Ph-**C**H$_3$). The ^{1}H-NMR and ^{13}C-NMR spectra were in agreement with the literature.[8] 2→3 acyl migration byproduct **S4b**: HRMS (ESI-QTOF) m/z: $[M+NH_4]^+$ Calcd for $C_{34}H_{34}NO_8S$ 616.2000; found 616.1973. ^{1}H NMR (500 MHz, $CDCl_3$): δ, ppm 8.02 (dd, *J* = 1.2, 8.3 Hz, 2H), 7.89 (dd, *J* = 1.2, 8.3 Hz, 2H), 7.82 (dd, *J* = 1.2, 8.3 Hz, 2H), 7.63 - 7.59 (m, 1H), 7.58 - 7.55 (m, 3H), 7.50 - 7.46 (m, 1H), 7.43 (t, *J* = 7.7 Hz, 4H), 7.31 - 7.27 (m, 2H), 7.14 (d, *J* = 7.9 Hz, 2H), 5.90 (dd, $J_{4,5}$ = 0.8 Hz, $J_{3,4}$ = 3.2 Hz, 1H, H-4), 5.41 (dd, $J_{3,4}$ = 3.3 Hz, $J_{2,3}$ = 9.7 Hz, 1H, H-3), 4.71 (d, $J_{1,2}$ = 9.6 Hz, 1H, H-1), 4.62 (dd, J_{AB} = 11.4 Hz, $J_{5,6A}$ = 6.8 Hz, 1H, H-6A), 4.37 (dd, J_{AB} = 11.4 Hz, $J_{5,6B}$ = 6.0 Hz, 1H, H-6B), 4.29 (m, 1H), 4.03 (dd, $J_{1,2}$ = $J_{2,3}$ = 9.6 Hz, 1H, H-2), 2.54 - 2.52 (br, 1H, 2-O***H***), 2.40 (s, 3H, S-Ph-C***H***$_3$), H_2O (δ 1.55 (br)). ^{13}C{^{1}H} NMR (126 MHz, $CDCl_3$): δ, ppm 166.1 (C=O (6-O-Bz)), 166.0 (C=O (Bz)), 165.4 (C=O (Bz)), 138.9, 134.4, 133.6, 133.3, 130.0, 129.9, 129.9, 129.6, 129.3, 129.2, 128.5, 128.5, 128.4, 126.8, 88.5 (C-1(Galβ)), 75.1 (C-5), 74.5 (C-3), 68.6 (C-4), 67.5 (C-2), 62.5 (C-6), 21.4 (S-Ph-**C**H$_3$). The ^{1}H-NMR spectrum was in agreement with the literature.[9]

***p*-Tolyl 4,6-O-(4-methoxyphenyl)methylene-3-O-(2-naphthalenylmethyl)-1-thio-β-D-galactopyranoside (21)**

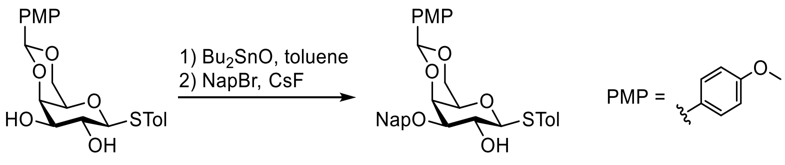

Compound **10** (2.32 g, 5.74 mmol) and dibutyltin oxide (2.14 g, 8.60 mmol) were suspended in anhydrous toluene (40 ml). The reaction mixture was heated in an oil bath and refluxed for 3h in a Dean-Stark apparatus equipped with a drying tube containing $CaCl_2$ until no further water collection was observed in the Dean-Stark trap. The reaction mixture was allowed to cool to 70°C and 2-naphthalenylmethyl bromide (NapBr) (1.91 g, 8.64 mmol) and cesium fluoride (CsF) (1.74 g, 11.5 mmol) were added. The reaction was stirred in an oil bath at 90°C for 5h until a major product had formed (as indicated by TLC, 45% EtOAc in hexane, R_f=0.36). The reaction mixture was filtered through celite, and the celite was washed with DCM (~50 ml). The filtrate was a clear amber solution. The reaction mixture was evaporated directly on silica gel 60 (~12 g, 40-63 μm) and purified by flash chromatography (dry loading method) to obtain **21** (2.24 g, 72% yield, elution at 55% EtOAc in hexane). HRMS (ESI-QTOF) m/z: $[M+H]^+$ Calcd for $C_{32}H_{33}O_6S$ 545.1992; found 545.1986. ^{1}H NMR (500 MHz, *d*$_6$-acetone): δ, ppm 7.88 - 7.85 (m, 2H), 7.84 - 7.78 (m, 2H), 7.56 (d, *J* = 8.1 Hz, 2H), 7.53 (dd, *J* = 1.6, 8.5 Hz, 1H), 7.48 - 7.45 (m, 2H), 7.40 - 7.38 (m, 2H), 7.09 (d, *J* = 7.9 Hz, 2H), 6.93 - 6.91 (m, 2H), 5.55 (s, 1H, MeOPhC***H***OO), 4.91, 4.86 (ABq, $\Delta\delta_{AB}$ = 0.06, J_{AB} = 12.4 Hz, 2H, 3-O-C***H***$_2$-Naph), 4.62 (d, $J_{1,2}$ = 9.5 Hz, 1H, H-1), 4.50 (dd, $J_{4,5}$ = 0.9 Hz, $J_{3,4}$ = 3.4 Hz, 1H, H-4), 4.33 (d, $J_{2,OH}$ = 4.2 Hz, 1H, 2-O***H***), 4.19 (dd, J_{AB} = 12.2 Hz, $J_{5,6A}$ = 1.6 Hz, 1H, H-6A), 4.07 (dd, J_{AB} = 12.3 Hz, $J_{5,6B}$ = 1.7 Hz, 1H, H-6B), 3.86 (ddd, $J_{1,2}$ = $J_{2,3}$ = 9.3 Hz, $J_{2,OH}$ = 4.4 Hz, 1H, H-2), 3.81 (s, 3H, ***Me***OPhCHOO), 3.68 (dd, $J_{3,4}$ = 3.4, $J_{2,3}$ = 9.2 Hz, 1H, H-3), 3.64 - 3.63 (m, 1H, H-5), 2.32 (s, 3H, S-Ph-C***H***$_3$), H_2O and HOD (δ 2.83 (s); δ 2.79 (t)). ^{13}C{^{1}H} NMR (126 MHz, *d*$_6$-acetone): δ, ppm 160.9 (*p*-PMP), 138.0, 137.7, 134.2, 133.8, 133.8, 132.4, 130.2, 130.1, 128.7, 128.6,

128.5, 128.4, 126.8, 126.8, 126.7, 126.5, 113.9, 101.4 (MeOPh**C**HOO), 88.3 (C-1), 82.0 (C-3), 74.0 (C-4), 71.7 (3-O-**C**H_2-Naph), 70.7 (C-5), 70.0 (C-6), 68.3 (C-2), 55.5 (***Me***OPhCHOO), 21.1 (S-Ph-**C**H_3).

***p*-Tolyl 2-O-benzoyl-4,6-(4-methoxyphenyl)methylene-3-O-(2-naphthalenylmethyl)-1-thio-β-D-galactopyranoside (22)**

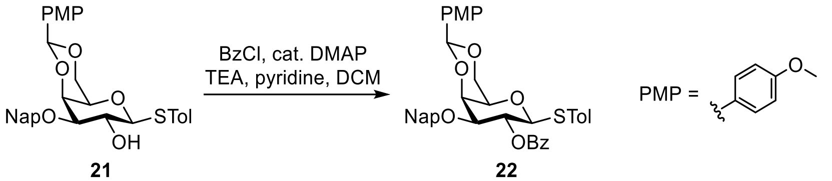

Compound **21** (1.51 g, 2.77 mmol) was dissolved in anhydrous DCM (30 ml) and pyridine (10 ml), then cooled to 0°C in an ice-water bath. BzCl (0.65 ml, 5.6 mmol) was added. DMAP (68 mg, 0.56 mmol) was dissolved in pyridine (3 ml) and added dropwise, followed by the addition of TEA (0.78 ml, 5.6 mmol). The reaction mixture became orange after the addition of TEA. The reaction mixture was stirred at 0°C for 30 min, then for an additional 3h at RT until the reaction was complete (as indicated by RP-TLC, 85% ACN in TDW, R_f=0.10). The reaction mixture was diluted with DCM (~20 ml) and washed with aqueous HCl (1 M, ~30 ml). The aqueous layer registered pH~5 (pyridine buffer region). The organic layer was separated and the aqueous layer was extracted with DCM (5 ml, x2). The combined organic layer was washed with sat. $NaHCO_3$, then brine. The organic layer was dried over Na_2SO_4, filtered and evaporated. The crude product was redissolved in toluene and evaporated again to remove residual pyridine. Crude **22** (2.92 g) was used in the next step without purification. Analytical amount of **22** was purified for characterization HRMS (ESI-QTOF) m/z: $[M+H]^+$ Calcd for $C_{39}H_{37}O_7S$ 649.2255; found 649.2288. ^1H NMR (500 MHz, d_6-acetone): δ , ppm 8.06 - 8.03 (m, 2H), 7.81 - 7.78 (m, 1H), 7.71 - 7.70 (m, 1H), 7.67 - 7.64 (m, 2H), 7.60 - 7.57 (m, 1H), 7.54 - 7.50 (m, 2H), 7.47 - 7.40 (m, 6H), 7.32 (dd, *J* = 1.7, 8.4 Hz, 1H), 7.25 - 7.21 (m, 1H), 7.18 - 7.16 (m, 1H), 7.10 - 7.08 (m, 2H), 6.94 - 6.92 (m, 2H), 5.63 (s, 1H, MeOPhC***H***OO), 5.60 (dd, $J_{1,2}$ = $J_{2,3}$ = 9.8 Hz, 1H, H-2), 5.01 (d, $J_{1,2}$ = 9.9 Hz, 1H, H-1), 4.87, 4.75 (ABq, $\Delta\delta_{AB}$ = 0.11, J_{AB} = 12.7 Hz, 2H, 3-O-C$\boldsymbol{H_2}$-Naph), 4.70 (dd, $J_{4,5}$ = 0.9 Hz, $J_{3,4}$ = 3.5 Hz, 1H, H-4), 4.25 (dd, J_{AB} = 12.2 Hz, $J_{5,6A}$ = 1.6 Hz, 1H, H-6A), 4.14 (dd, J_{AB} = 12.3 Hz, $J_{5,6B}$ = 1.7 Hz, 1H, H-6B), 4.09 (dd, $J_{3,4}$ = 3.5 Hz, $J_{2,3}$ = 9.6 Hz, 1H), 3.82 (s, 3H, ***Me***OPhCHOO), 3.79 - 3.78 (m, 1H, H-5), 2.31 (s, 3H, S-Ph-C$\boldsymbol{H_3}$), H_2O and HOD (δ 2.83 (s); δ 2.79 (t)). $^{13}C\{^1H\}$ NMR (126 MHz, d_6-acetone): δ , ppm 165.6 (C=O (2-O-Bz)), 161.0 (*p*-PMP), 138.3, 136.9, 134.1, 133.9, 133.8, 133.6, 132.1, 131.4, 130.4, 130.3, 130.1, 129.7, 129.4, 129, 128.7, 128.6, 128.5, 128.4, 126.8, 126.8, 126.6, 126.4, 126.1, 114, 101.5 (MeOPh**C**HOO), 86.3 (C-1), 79.5 (C-3), 73.5 (C-4), 70.9 (3-O-**C**H_2-Naph), 70.8 (C-5), 70.1 (C-2), 69.9 (C-6), 55.5 (***Me***OPhCHOO), 21.1 (S-Ph-**C**H_3).

***p*-Tolyl 2-O-benzoyl-3-O-(2-naphthalenylmethyl)-1-thio-β-D-galactopyranoside (23)**

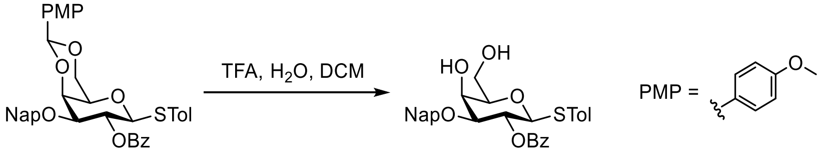

Crude **22** (2.92 g) was dissolved in DCM (27 ml) and cooled to 0°C in an ice-water bath. TDW (0.3 ml) was added, followed by the addition of TFA (3 ml) dropwise over 5 min. The reaction mixture was stirred at 0°C for 30 min, then for an additional 2h at RT until completion (as indicated by TLC, 45% EtOAc in hexane, R_f=0.16). The reaction mixture was diluted with DCM (~50 ml), and the organic layer was washed with sat. $NaHCO_3$. The layers were separated and the aqueous layer was extracted with DCM (5 ml, x4). The combined organic layer was washed again with sat. $NaHCO_3$, then brine. A small amount of MeOH was added to improve layer separation. The organic layer was dried over Na_2SO_4, filtered and evaporated.

The crude product was triturated with 15% EtOAc in hexane, until the decanted solution was colorless (6 times, ~20 ml each). The residual solid was redissolved and evaporated from toluene and MeOH to produce sufficiently pure **23** as a light brown solid (1.24 g, 84% 2-step yield). HRMS (ESI-QTOF) m/z: $[M+H]^+$ Calcd for $C_{31}H_{31}O_6S$ 531.1836; found 531.1858. ^{1}H NMR (500 MHz, d_6-acetone): δ , ppm 8.05 - 8.03 (m, 2H), 7.81 - 7.78 (m, 1H), 7.71 (s, 1H), 7.68 - 7.62 (m, 2H), 7.61 - 7.58 (m, 1H), 7.53 - 7.49 (m, 2H), 7.43 (ddd, *J* = 2.2, 4.7, 7.3 Hz, 2H), 7.37 - 7.35 (m, 2H), 7.32 (dd, *J* = 1.7, 8.4 Hz, 1H), 7.09 - 7.07 (m, 2H), 5.59 (dd, $J_{1,2} = J_{2,3}$ = 9.8 Hz, 1H, H-2), 4.95 (d, $J_{1,2}$ = 10.1 Hz, 1H, H-1), 4.89, 4.71 (ABq, $\Delta\delta_{AB}$ = 0.18, J_{AB} = 12.5 Hz, 2H, 3-O-C***H***$_2$-Naph), 4.43 (m, 1H, H-4), 3.95 (dd, $J_{3,4}$ = 3.1 Hz, $J_{2,3}$ = 9.5 Hz, 1H, H-3), 3.85 - 3.83 (m, 2H, H-6), 3.74 (dt, $J_{4,5}$ = 1.1 Hz, $J_{5,6}$ = 5.9 Hz, 1H, H-5), 2.27 (s, 3H, S-Ph-C***H***$_3$). ^{13}C{^{1}H} NMR (126 MHz, d_6-acetone): δ , ppm 165.9 (C=O (2-O-Bz)), 137.9, 136.9, 134.1, 133.9, 133.8, 132.4, 131.5, 131.4, 130.4, 130.3, 129.4, 128.6, 128.5, 128.4, 126.9, 126.8, 126.6, 126.5, 87.5 (C-1), 81.3 (C-3), 80.1 (C-5), 71.2 (3-O-**C**H$_2$-Naph), 70.9 (C-2), 66.4 (C-4), 62.1 (C-6), 21.0 (S-Ph-**C**H$_3$).

***p*-Tolyl 2-O-benzoyl-4,6-di-O-benzyl-3-O-(2-naphthalenylmethyl)-1-thio-β-D-galactopyranoside (6)**

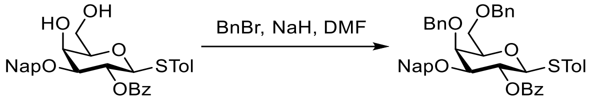

23 **6**

Compound **6** was synthesized using a previously described method.[10] Compound **23** (1.24 g, 2.34 mmol) was dissolved in anhydrous DMF (24 ml) and cooled to 0°C in an ice-water bath. BnBr (0.83 ml, 7.0 mmol) was added dropwise, followed by the addition ofNaH (60% dispersion in mineral oil, 210 mg, 5.25 mmol) in one portion. The solution turned dark purple. The headspace of the flask was purged with Ar and the reaction mixture was stirred at 0°C for 1h until completion (as indicated by TLC, 50% EtOAc in hexane, R_f=0.69). The reaction mixture was diluted with DCM (~20 ml) and quenched by pouring onto aqueous HCl (1 M, ~20 ml). The layers were separated and the aqueous layer was extracted with DCM (10 ml, x2). The combined organic layer was washed with brine. A small amount of sat. $NaHCO_3$ (~1 ml) was added to neutralize the slightly acidic aqueous layer. The layers were separated and the organic layer was dried over Na_2SO_4, filtered and evaporated. Compound **6** was purified by flash chromatography (1.34 g, 81% yield, elution at 30% EtOAc in hexane). HRMS (ESI-Q-Orbitrap) m/z: $[M+NH_4]^+$ Calcd for $C_{45}H_{46}NO_6S$ 728.3040; found 728.3047. ^{1}H NMR (500MHz, $CDCl_3$): δ , ppm 7.98 (dd, $J_{o,m}$ = 8.4 Hz, $J_{o,p}$ = 1.3 Hz, 2H, *o*-Bz), 7.72 (m, 1H, *p*-Bz), 7.59-7.55 (m, 3H, Naph), 7.53 (d, $J_{3,4}$ = 8.5 Hz, 1H, 3-Naph), 7.44 - 7.38 (m, 4H), 7.36 - 7.29 (m, 9H), 7.29 - 7.27 (m, 3H), 7.23 (dd, $J_{3,4}$ = 8.4 Hz, $J_{4,5}$ = 1.7 Hz, 1H, 4-Naph), 6.98 (m, 2H, *m*-Tol), 5.67 (dd, $J_{1,2} = J_{2,3}$ = 9.8 Hz, 1H, H-2), 5.01, 4.64 (ABq, $\Delta\delta_{AB}$ = 0.37, J_{AB} = 11.7 Hz, 2H, 4-O-C***H***$_2$-Ph), 4.78, 4.62 (ABq, $\Delta\delta_{AB}$ = 0.16, J_{AB} = 12.4 Hz, 2H, Naph-C***H***$_2$-O), 4.68 (d, $J_{1,2}$ = 9.9 Hz, 1H, H-1), 4.47, 4.43 (ABq, $\Delta\delta_{AB}$ = 0.04, J_{AB} = 11.6 Hz, 2H, 6-O-C***H***$_2$-Ph), 4.07 (m, 1H, H-4), 3.72 (dd, $J_{2,3}$ = 9.6 Hz, $J_{3,4}$ = 2.7 Hz, 1H, H-3), 3.70 - 3.64 (m, 3H, H-6; H-5), 2.27 (s, 3H, S-Ph-C***H***$_3$). ^{13}C{^{1}H} NMR (126 MHz, $CDCl_3$): δ , ppm 165.3 (**C**=O (Bz)), 138.5, 137.9, 137.6, 135.0, 133.0, 133.0, 132.9 (*o*-Tol), 132.7, 130.2, 129.9 (*o*-Bz), 129.7, 129.5 (*m*-Tol), 128.5, 128.3, 128.2, 128.2, 128.0, 128.0, 127.8, 127.6 (*p*-Bz), 127.5, 126.5, 126.1, 125.9, 125.7, 87.3 (C-1), 81.0 (C-3), 77.7 (C-5), 74.4 (4-O-**C**H$_2$-Ph), 73.6 (6-O-**C**H$_2$-Ph), 72.7 (C-4), 70.4 (Naph-**C**H$_2$-O), 71.8 (C-2), 68.8 (C-6), 21.1 (S-Ph-**C**H$_3$). The ^{1}H-NMR and ^{13}C-NMR spectra were in agreement with the literature.[11]

_p_-Tolyl 2-O-benzoyl-4,6-di-O-benzyl-1-thio-β-D-galactopyranoside (9)

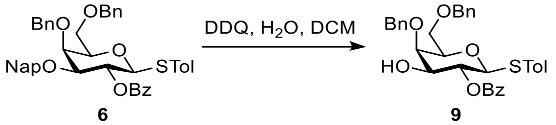

Compound **6** (195 mg, 0.274 mmol) was dissolved in DCM (1.8 ml) and TDW (0.2 ml). 2,3-Dichloro-5,6-dicyano-1,4-benzoquinone (DDQ) (91 mg, 0.40 mmol) was added. The reaction mixture obtained a green-brown color. The reaction mixture was stirred for 1 h and 40 min at RT, covered from light, until completion (as indicated by TLC, 30% EtOAc in hexane, R_f=0.35). The reaction was quenched by the addition of sat. $NaHCO_3$ (~10 ml), then diluted with DCM (~20 ml). The organic layer was washed with additional sat. $NaHCO_3$ (~40 ml). The aqueous layer had an intense red color, the organic phase appeared light yellow. The layers were separated and the aqueous layer was extracted with DCM (5 ml). The combined organic layers were washed with sat. $NaHCO_3$, then and brine. The organic layer was dried over Na_2SO_4, filtered and evaporated to produce a light brown solid. Compound **9** was purified by flash chromatography (149 mg, 95% yield, elution at 35% EtOAc in hexane). HRMS (ESI-QTOF) m/z: $[M+H]^+$ Calcd for $C_{34}H_{35}O_6S$ 571.2149; found 571.2135. ^1H NMR (500 MHz, $CDCl_3$): δ, ppm 8.08 - 8.05 (m, 2H), 7.61 - 7.56 (m, 1H), 7.48 - 7.44 (m, 2H), 7.37 - 7.28 (m, 14H), 7.03 (d, J = 7.9 Hz, 2H), 5.22 (dd, $J_{1,2}$ = $J_{2,3}$ = 9.7 Hz, 1H, H-2), 4.74 (d, $J_{1,2}$ = 9.9 Hz, 1H, H-1), 4.73 (ABq, $\Delta\upsilon_{AB}$ = 14.6 Hz, J_{AB} = 11.8 Hz, 2H, O-C$\boldsymbol{H}_2$-Ph), 4.52 (ABq, $\Delta\upsilon_{AB}$ = 24.5 Hz, J_{AB} = 11.7 Hz, 2H, O-C$\boldsymbol{H}_2$-Ph), 3.98 (d, $J_{3,4}$ = 3.4 Hz, 1H, H-4), 3.80 (dd, $J_{3,4}$ = 3.4 Hz, $J_{2,3}$ = 9.6 Hz, 1H, H-3), 3.78 - 3.71 (m, 3H, H-6; H-5), 2.31 (s, 3H, S-Ph-C$\boldsymbol{H}_3$). $^{13}C\{^1H\}$ NMR (126 MHz, $CDCl_3$): δ, ppm 166.7 (**C**=O (Bz)), 138.2, 138.1, 137.8, 133.3, 133.2, 130, 129.9, 129.8, 129.6, 128.9, 128.6, 128.6, 128.5, 128, 127.9, 127.7, 86.4 (C-1), 77.6 (C-5), 76.8 (C-4), 75.4 (O-CH_2-Ph), 74.4 (C-3), 73.7 (O-**C**H_2-Ph), 72.4 (C-2), 68.4 (C-6), 21.2 (S-Ph-**C**H_3). The ^1H-NMR spectrum was in agreement with the literature.[12]

_p_-Tolyl 2,3-di-O-benzyl-4,6-O-(4-methoxyphenyl)methylene-α-D-galactopyranosyl-(1→3)-2,4,6-tri-O-benzoyl-1-thio-β-D-galactopyranoside (14)

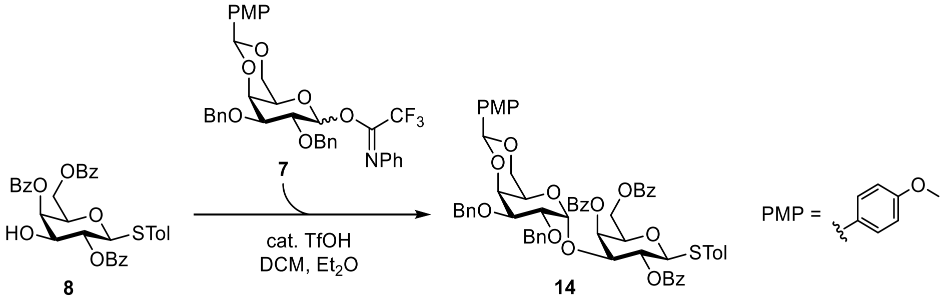

Glycosyl acceptor **8** (498 mg, 0.83 mmol) and glycosyl donor **7** (827 mg, 1.27 mmol) were co-evaporated from toluene and dried overnight under high vacuum. The mixture of glycosyl donor and acceptor was dissolved in anhydrous DCM (8 ml) and anhydrous diethyl ether (Et_2O) (8 ml) and cooled to 0°C in an ice-water bath. The reaction mixture was stirred for 30 min at 0°C, after which trifluoromethanesulfonic acid (TfOH) (10 μl, 0.11 mmol) was added. The reaction mixture turned bright pink, then red. The reaction mixture was stirred at 0°C for 1 h, after which the reaction turned deep blue and the glycosyl acceptor was fully consumed (as indicated by RP-TLC, **8** 75% ACN in TDW, R_f=0.06). The reaction mixture was diluted with DCM (50 ml) and washed with sat. $NaHCO_3$. The layers were separated and the aqueous layer was extracted with DCM (3x 10 ml). The combined organic layer was washed with brine, dried over Na_2SO_4, filtered, and concentrated under reduced pressure. Disaccharide **14** was purified by flash chromatography (770 mg, 87% yield, elution at 40% EtOAc in hexane). HRMS (ESI-Q-Orbitrap) m/z: $[M+Na]^+$ Calcd for $C_{62}H_{58}O_{14}SNa$ 1081.3440; found 1081.3433. ^1H NMR (500MHz, d_6-acetone): δ, ppm 8.12 - 8.10 (m, 2H, Bz), 8.05 - 8.03 (m, 2H, Bz), 7.99 - 7.96 (m, 2H, Bz), 7.74 - 7.65 (m,

3H), 7.62 - 7.59 (m, 2H), 7.53 - 7.47 (m, 6H), 7.27 - 7.15 (m, 12H), 7.08 (m, 2H, *m*-Tol), 6.83 (m, 2H, *m*-PMP), 6.09 (m, 1H, H-4), 5.67 (dd, $J_{1,2} = J_{2,3}$ = 9.7 Hz, 1H, H-2), 5.25 (s, 1H, PMPC***H***OO), 5.25 (d, $J_{1,2}$ = 3.9 Hz, 1H, H-1'(Galα)), 5.21 (d, $J_{1,2}$ = 10.0 Hz, 1H, H-1(Galβ)), 4.58, 4.48 (ABq, $\Delta\delta_{AB}$ = 0.10, J_{AB} = 12.2 Hz, 2H, 3-O-C***H₂***-Ph), 4.57 (dd, $J_{2,3}$ = 9.6 Hz, $J_{3,4}$ = 3.3 Hz, 1H, H-3), 4.53 - 4.49 (m, 3H, H-6; H-5), 4.35, 4.31 (ABq, $\Delta\delta_{AB}$ = 0.04, J_{AB} = 11.7 Hz, 2H, 2-O-C$\boldsymbol{H_2}$-Ph), 3.82 (dd, $J_{3,4}$ = 3.4 Hz, $J_{4,5}$ = 1.1 Hz, 1H, H-4'), 3.80 (dd, $J_{2,3}$ = 10.1Hz, $J_{1,2}$ = 3.3 Hz, 1H, H-2'), 3.75 (s, 3H, ***Me***OPhCHOO), 3.70, 3.54 (ABdq, $\Delta\delta_{AB}$ = 0.16, J_{AB} = 12.5 Hz, $J_{5,6}$ = 1.9 Hz, 2H, H-6'), 3.66 (dd, $J_{2,3}$ = 10.1 Hz, $J_{3,4}$ = 3.4 Hz, 1H, H-3'), 3.66 (m, 1H, H-5'), 2.33 (s, 3H, S-Ph-C$\boldsymbol{H_3}$), H_2O and HOD (δ 2.83 (s); δ 2.79 (t)). $^{13}C\{^1H\}$ NMR (126 MHz, , d_6-acetone): δ , ppm 166.4 (**C**=O (Bz)), 166.4 (**C**=O (Bz)), 165.5 (**C**=O (Bz)), 160.8 (*p*-PMP), 140.0, 139.9, 138.8, 134.4, 134.2, 134.1, 134, 132.1, 130.9, 130.8, 130.7, 130.7, 130.4, 130.4, 130.3, 129.7, 129.4, 129.4, 129.3, 128.8, 128.8, 128.5, 128.3, 128.1, 127.9, 127.8, 113.9 (*m*-PMP), 100.9 (PMPCHOO), 97.3 (C-1'(Galα)), 86.1 (C-1(Galβ)), 77.1 (C-3'), 76.6 (C-3), 76.1 (C-5), 75.3 (C-2'), 74.5 (C-4'), 73.2 (3-O-**C**H_2-Ph), 71.9 (2-O-**C**H_2-Ph), 70.3 (C-2), 69.2 (C-6'), 69.0 (C-4), 64.0 (C-6), 63.9 (C-5'), 55.4 (***Me***OPhCHOO), 21.2 (S-Ph-**C**H_3). *Note: The configuration of the anomeric carbons C-1 and C-1' was assigned with additional information from non-decoupled ^1H-^{13}C HSQC (vide infra, ^1H-^{13}C non-decoupled HSQC spectrum **14**): 101.0 (d, J=159Hz, PMP**C**HOO, axial), 97.4 (d, J=169Hz, C-1' (α)), 86.2 (d, J=158Hz, C-1 (β)).*

p-Tolyl 2,3-di-O-benzyl-α-D-galactopyranosyl-(1→3)-2,4,6-tri-O-benzoyl-1-thio-β-D-galactopyranoside (15)

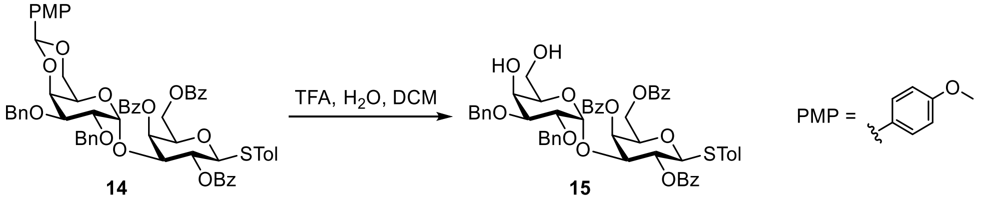

Disaccharide **14** (440 mg, 0.42 mmol) was dissolved in DCM (11 ml) and cooled to 0°C in an ice-water bath. TFA (1.2 ml) was added dropwise, followed by addition of TDW (0.06 ml). The reaction was allowed to reach RT and stirred for 1.5 h. until completion (as indicated by TLC, 50% EtOAc in hexane, R_f=0.21). The reaction mixture was diluted with DCM and washed with sat. $NaHCO_3$, then brine. The organic layer was dried over Na_2SO_4, filtered and evaporated. Disaccharide **15** was purified by flash chromatography (338 mg, 86% yield, elution at 40% EtOAc in hexane). HRMS (ESI-Q-Orbitrap) m/z: $[M+NH_4]^+$ Calcd for $C_{54}H_{56}NO_{13}S$ 958.3467; found 958.3497. ^1H NMR (500 MHz, $CDCl_3$): δ , ppm 8.07 - 8.02 (m, 4H), 7.94 - 7.91 (m, 2H), 7.62 - 7.54 (m, 3H), 7.48 - 7.42 (m, 6H), 7.40 - 7.35 (m, 2H), 7.26 - 7.26 (m, 1H), 7.25 - 7.24 (m, 2H), 7.17 - 7.10 (m, 7H), 7.03 - 7.00 (m, 2H), 5.93 (d, $J_{3,4}$ = 3.1 Hz, 1H, H-4), 5.64 (dd, $J_{1,2} = J_{2,3}$ = 9.8 Hz, 1H, H-2), 5.17 (d, $J_{1,2}$ = 3.4 Hz, 1H, H-1'(Galα)), 4.85 (d, $J_{1,2}$ = 10.0 Hz, 1H, H-1(Galβ)), 4.54 (dd, J_{AB} = 11.6 Hz, $J_{5,6A}$ = 7.2 Hz, 1H, H-6A), 4.46 (dd, J_{AB} = 11.4 Hz, $J_{5,6B}$ = 4.7 Hz, 1H, H-6B), 4.46, 4,28 (ABq, $\Delta\delta_{AB}$ = 0.18, J_{AB} = 11.4 Hz, 2H, O-C$\boldsymbol{H_2}$-Ph), 4.45 (ABq, $\Delta\upsilon_{AB}$ = 31.6 Hz, J_{AB} = 12.3 Hz, 2H, O-C$\boldsymbol{H_2}$-Ph), 4.17 - 4.12 (m, 2H, H-3; H-5), 3.65 (dd, $J_{1,2}$ = 3.3 Hz, $J_{2,3}$ = 9.8 Hz, 1H, H-2'), 3.60 - 3.54 (m, 2H, H-6'A; H-5'), 3.48 (dd, $J_{3,4}$ = 3.5 Hz, $J_{2,3}$ = 9.9 Hz, 1H, H-3'), 3.46 - 3.42 (m, 1H, H-6'B), 3.33 (d, $J_{3,4}$ = 3.3 Hz, 1H, H-4'), 2.33 (s, 3H, S-Ph-C$\boldsymbol{H_3}$). $^{13}C\{^1H\}$ NMR (126 MHz, $CDCl_3$): δ , ppm 166.2 (**C**=O (Bz)), 165.8 (**C**=O (Bz)), 165.0 (**C**=O (Bz)), 138.4, 138.4, 138.0, 134.0, 133.6, 133.4, 133.3, 130.2, 129.9, 129.8, 129.7, 129.6, 129.2, 128.7, 128.5, 128.4, 128.2, 128.1, 127.8, 127.8, 127.7, 127.4, 95.0 (C-1'(Galα)), 86.3 (C-1(Galβ)), 77.0 (C-3'), 75.4 (C-5), 75.1 (C-3), 74.6 (C-2'), 72.9 (O-**C**H_2-Ph), 72.7 (O-**C**H_2-Ph), 69.6 (C-5'), 69.5 (C-2), 69.2 (C-4'), 66.8 (C-4), 63.0 (C-6'), 63.0 (C-6), 21.3 (S-Ph-**C**H_3).

***p*-Tolyl 2,3-di-O-benzyl-6-O-(tert-butyldiphenylsilyl)-α-D-galactopyranosyl-(1→3)-2,4,6-tri-O-benzoyl-1-thio-β-D-galactopyranoside (16)**

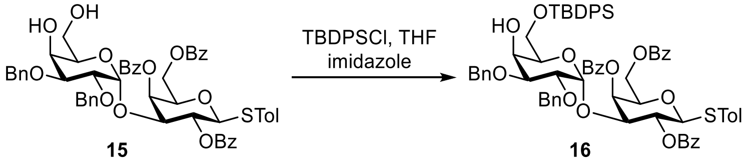

Disaccharide **15** (251 mg, 0.27 mmol) was dissolved in anhydrous THF (4.2 ml). Imidazole (239 mg, 3.51 mmol) and TBDPSCl (0.08 ml, 0.31 mmol) were added. The reaction mixture was stirred for 4 h at RT until completion (as indicated by TLC, 30% EtOAc in hexane, R_f=0.50). The reaction was diluted with THF, then directly evaporated on silica gel 60 (~12 g, 40-63 μm) and purified by flash chromatography (dry loading method) to obtain **16** (303 mg, 95% yield, elution at 35% EtOAc in hexane). HRMS (ESI-QTOF) m/z: $[M+NH_4]^+$ Calcd for $C_{70}H_{74}NO_{13}SSi$ 1196.4645; found 1196.4666. ^{1}H NMR (500MHz, d_6-acetone): δ , ppm 8.11 (m, 2H, Bz), 8.02 (m, 2H, Bz), 7.99 (m, 2H, Bz), 7.77 - 7.75 (m, 4H, Ar), 7.70 - 7.64 (m, 3H, Ar), 7.55 - 7.41 (m, 14H, Ar), 7.25 - 7.09 (m, 17H, Ar), 6.08 (m, 1H, H-4), 5.71 (dd, $J_{1,2}$ = $J_{2,3}$ = 9.9 Hz, 1H, H-2), 5.38 (d, $J_{1,2}$ = 3.4 Hz, 1H, H-1'(Galα)), 5.01 (d, $J_{1,2}$ = 10.0 Hz, 1H, H-1(Galβ)), 4.59 (dd, $J_{2,3}$ = 9.9 Hz, $J_{3,4}$ = 3.1 Hz, 1H, H-3), 4.53, 4.44 (ABdq, $\Delta\delta_{AB}$=0.09, J_{AB} = 11.5 Hz, $J_{5,6A}$ = 7.2 Hz, $J_{5,6B}$ = 5.0 Hz, 2H, H-6), 4.49, 4.39 (ABq, $\Delta\delta_{AB}$ = 0.10, J_{AB} = 12.2 Hz, 2H, 2-O-C***H***$_2$-Ph), 4.27 (s, 2H, 3-O-C***H***$_2$-Ph), 4.21 (ddd, $J_{5,6A}$ = 7.1 Hz, $J_{5,6B}$ = 4.9 Hz, $J_{3,4}$ = 1.0 Hz, 1H, H-5), 3.87 (m, 1H, H-5'), 3.87, 3.70 (ABdq, $\Delta\delta_{AB}$=0.17, J_{AB} = 10.3 Hz, $J_{5,6A}$ = 7.5 Hz, $J_{5,6B}$ = 4.0 Hz, 2H, H-6'), 3.77 (dd, $J_{2,3}$ = 9.9 Hz, $J_{1,2}$ = 3.4 Hz, 1H, H-2'), 3.63 (m, 1H, H-4'), 3.44 (m, 1H, 4'-OH), 3.75 (dd, $J_{2,3}$ = 9.9 Hz, $J_{3,4}$ = 3.1 Hz, 1H, H-3'), 2.35 (s, 3H, S-Ph-C***H***$_3$), 1.10 (s, 9H, SiPh$_2$-C-(C***H***$_3$)$_3$), residual EtOH (1:0.16 ratio; δ 3.57 (dq), 3.36 (t)). ^{13}C{^{1}H} NMR (126 MHz, d_6-acetone): δ , ppm 166.4 (**C**=O (Bz)), 166.3 (**C**=O (Bz)), 165.4 (**C**=O (Bz)), 139.8, 139.8, 139.1, 136.4, 134.4, 134.2, 130.8, 130.5, 130.5, 130.4, 129.8, 129.6, 129.6, 129.5, 129.3, 129.1, 128.9, 128.8, 128.7, 128.7, 128.3, 128.3, 128.0, 127.8, 126.1, 94.0 (C-1'(Galα)), 86.8 (C-1(Galβ)), 78.7 (C-3'), 76.2 (C-5), 75.8 (C-2'), 74.0 (C-3), 72.8 (3-O-**C**H$_2$-Ph), 72.5 (2-O-**C**H$_2$-Ph), 72.4 (C-4'), 70.0 (C-2), 67.8 (C-5'), 67.4 (C-4), 65.1 (C-6'), 63.7 (C-6), 27.5 (SiPh$_2$-C-(**C**H$_3$)$_3$), 21.3 (S-Ph-**C**H$_3$), 20.0 (SiPh$_2$-**C**-(CH$_3$)$_3$), residual EtOH (δ 57.8, 18.9).

***p*-Tolyl 4-O-benzoyl-2,3-di-O-benzyl-6-O-(tert-butyldiphenylsilyl)-α-D-galactopyranosyl-(1→3)-2,4,6-tri-O-benzoyl-1-thio-β-D-galactopyranoside (1)**

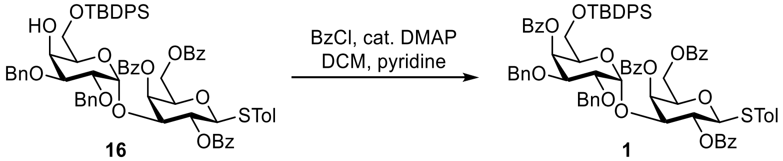

Compound **16** (229 mg, 0.194 mmol) was dissolved in anhydrous DCM (3 ml) and cooled to 0°C in an ice-water bath. BzCl (0.05 ml, 0.39 mmol) was added dropwise. DMAP (5 mg, 0.04 mmol) was dissolved in pyridine (2 ml), and the solution was added to the reaction mixture dropwise, followed by the addition of TEA (0.05 ml, 0.36 mmol). The reaction mixture obtained a faint pink-orange color. The reaction was stirred at 0°C for 1 h, then at RT for an additional 5 h until completion (as indicated by TLC, 20% EtOAc in hexane, R_f=0.24). The reaction mixture was diluted with DCM (~30 ml) and washed with aqueous HCl (0.1 M, 30 ml). The layers were separated and the aqueous layer was extracted with DCM (2x 5 ml). The combined organic layer was washed with sat. $NaHCO_3$, then brine. The organic layer was dried over Na_2SO_4, filtered, and concentrated under reduced pressure. The crude residue was redissolved in toluene and evaporated to remove residual pyridine. Disaccharide **1** was purified by flash chromatography (230 mg, 92% yield, elution at 25% EtOAc in hexane). HRMS (ESI-QTOF) m/z: $[M+H]^+$ Calcd for $C_{77}H_{75}O_{14}SSi$ 1283.4641; found 1283.4691. ^{1}H NMR (500 MHz, $CDCl_3$): δ , ppm 8.09 (dd, J = 1.2, 8.4 Hz, 2H), 8.05 (dd, J = 1.3, 8.4 Hz, 2H), 7.97 (dd, J = 1.3, 8.4 Hz, 2H), 7.77 (dd, J = 1.3, 8.4 Hz,

2H), 7.66 - 7.64 (m, 2H), 7.62 - 7.54 (m, 5H), 7.52 - 7.45 (m, 5H), 7.39 - 7.31 (m, 11H), 7.27 - 7.24 (m, 2H), 7.15 - 7.09 (m, 4H), 7.07 - 7.03 (m, 6H), 6.98 – 6.96 (m, 2H), 5.96 (d, $J_{3,4}$ = 2.6 Hz, 1H, H-4), 5.71 (dd, $J_{1,2}$ = $J_{2,3}$ = 9.9 Hz, 1H, H-2), 5.37 (d, $J_{1,2}$ = 3.4 Hz, 1H, H-1'(Galα)), 5.06 (dd, $J_{4,5}$ = 1.0 Hz, $J_{3,4}$ = 3.1 Hz, 1H, H-4'), 4.83 (d, $J_{1,2}$ = 10.0 Hz, 1H, H-1(Galβ)), 4.55 (dd, $J_{6A,6B}$ = 11.5 Hz, $J_{5,6A}$ = 7.0 Hz, 1H, H-6A), 4.40 (dd, $J_{6A,6B}$ = 11.6 Hz, $J_{5,6B}$ = 5.5 Hz, 1H, H-6B), 4.37 (dd, $J_{3,4}$ = 3.2 Hz, $J_{2,3}$ = 10.0 Hz, 1H, H-3), 4.37 (ABq, $\Delta\upsilon_{AB}$ = 33.6 Hz, J_{AB} = 12.2 Hz, 2H, 2-O-C***H***$_2$-Ph), 4.35, 4.12 (ABq, $\Delta\delta_{AB}$ = 0.23, J_{AB} = 11.0 Hz, 2H, 3-O-C***H***$_2$-Ph), 4.11 (m, 1H, H-5'), 3.95 (t, $J_{5,6}$ = 6.6 Hz, 1H, H-5), 3.74 (dd, $J_{1,2}$ = 3.4 Hz, $J_{2,3}$ = 10.0 Hz, 1H, H-2'), 3.67 (dd, $J_{6A,6B}$ = 10.5 Hz, $J_{5,6A}$ = 7.8 Hz, 1H, H-6'A), 3.57 (dd, $J_{3,4}$ = 3.3 Hz, $J_{2,3}$ = 10.1 Hz, 1H, H-3'), 3.55 (dd, $J_{6A,6B}$ = 10.6 Hz, $J_{5,6B}$ = 4.4 Hz, 1H, H-6'B), 2.37 (s, 3H, S-Ph-C***H***$_3$), 1.06 (s, 9H, SiPh$_2$-C-(C***H***$_3$)$_3$). ^{13}C{^{1}H} NMR (126 MHz, CDCl$_3$): δ, ppm 166.1 (**C**=O (6-O-Bz)), 165.9 (**C**=O (4-O-Bz)), 165.6 (**C**=O (4'-O-Bz)), 164.8 (**C**=O (2-O-Bz)), 138.4, 138.3, 138.1, 135.6, 135.5, 134.0, 133.7, 133.4, 133.3, 133.2, 133.1, 132.9, 130.3, 129.9, 129.9, 129.8, 129.8, 129.8, 129.7, 129.6, 129.1, 128.7, 128.5, 128.5, 128.3, 128.2, 128.1, 127.9, 127.8, 127.7, 127.7, 127.3, 127.0, 93.3 (C-1'(Galα)), 86.4 (C-1(Galβ)), 76.3 (C-3'), 75.3 (C-5), 74.0 (C-2'), 73.2 (C-3), 72.3 (O-CH$_2$-Ph), 71.8 (O-**C**H$_2$-Ph), 70.4 (C-5'), 69.2 (C-2), 68.5 (C-4'), 65.8 (C-4), 63.3 (C-6'), 62.9 (C-6), 26.9 (SiPh$_2$-C-(**C**H$_3$)$_3$), 21.4 (S-Ph-**C**H$_3$), 19.5 (SiPh$_2$-**C**-(CH$_3$)$_3$).

***p*-Tolyl 2,3-di-O-benzyl-4-O-(9-fluorenylmethyloxycarbonyl)-6-O-(tert-butyldiphenylsilyl)-*α*-D-galactopyranosyl-(1→3)-2,4,6-tri-O-benzoyl-1-thio-β-D-galactopyranoside (17)**

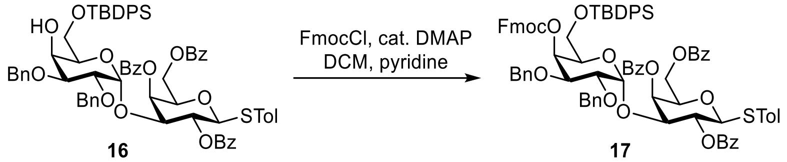

Compound **16** (211 mg, 0.18 mmol) was dissolved in anhydrous DCM (2 ml) and pyridine (0.08 ml), and cooled to 0°C in an ice-water bath. FmocCl (116 mg, 0.45 mmol) and DMAP (~2 mg, 0.02 mmol) were added. The reaction was stirred for 5 h at RT until a major product was formed (as indicated by TLC, 20% EtOAc in hexane, R_f=0.27). The reaction was diluted with DCM then directly evaporated on silica gel 60 (~0.6 g, 40-63 μm) and purified by flash chromatography (dry loading method) to obtain **17** (162 mg, 65% yield, elution at 20% EtOAc in hexane). HRMS (ESI-QTOF) m/z: [M+H]$^+$ Calcd for $C_{85}H_{81}O_{15}SSi$ 1401.5060; found 1401.5112. ^{1}H NMR (500 MHz, CDCl$_3$): δ, ppm 8.07 (dd, J = 1.2, 8.3 Hz, 2H), 8.05 (dd, J = 1.3, 8.3 Hz, 2H), 7.95 (dd, J = 1.2, 8.2 Hz, 2H), 7.71 (d, J = 7.6 Hz, 2H), 7.70 - 7.64 (m, 4H), 7.63 - 7.59 (m, 1H), 7.59 - 7.53 (m, 2H), 7.49 - 7.44 (m, 4H), 7.42 (dd, J = 2.2, 7.6 Hz, 2H), 7.39 - 7.31 (m, 13H), 7.16 (dt, J = 1.0, 7.5 Hz, 1H), 7.11 - 7.02 (m, 14H), 5.96 (d, $J_{3,4}$ = 2.9 Hz, 1H, H-4), 5.70 (dd, $J_{1,2}$ = $J_{2,3}$ = 9.9 Hz, 1H, H-2), 5.37 (d, $J_{1,2}$ = 3.4 Hz, 1H, H-1'(Galα)), 4.82 (d, $J_{1,2}$ = 10.0 Hz, 1H, H-1(Galβ)), 4.68 (d, $J_{3,4}$ = 2.4 Hz, 1H, H-4'), 4.55 (dd, J_{AB} = 11.5 Hz, $J_{5,6A}$ = 7.0 Hz, 1H, H-6A), 4.45 (ABq, $\Delta\upsilon_{AB}$ = 22.5 Hz, J_{AB} = 12.3 Hz, 2H, O-C***H***$_2$-Ph), 4.40 (dd, J_{AB} = 11.6 Hz, $J_{5,6B}$ = 5.4 Hz, 1H, H-6B), 4.34 (dd, $J_{3,4}$ = 3.1 Hz, $J_{2,3}$ = 9.8 Hz, 1H, H-3), 4.30, 4.20 (ABq, $\Delta\delta_{AB}$ = 0.10, J_{AB} = 11.0 Hz, 2H, O-C***H***$_2$-Ph), 4.24 (dd, J_{AB} = 6.9 Hz, $J_{1,9'}$ = 10.0 Hz, 1H, -C***H***$_2$- (Fmoc)), 4.06 (t, $J_{5,6}$ = 7.4 Hz, 1H, H-5'), 4.04 (m, 1H, -C***H***$_2$- (Fmoc)), 4.00 (t, J = 7.4 Hz, 1H, -C***H***- (Fmoc)), 3.94 (t, J = 6.3 Hz, 1H, H-5), 3.79 (dd, J_{AB} = 10.5 Hz, $J_{5,6A}$ = 7.8 Hz, 1H, H-6'A), 3.75 (dd, $J_{1,2}$ = 3.4 Hz, $J_{2,3}$ = 10.1 Hz, 1H, H-2'), 3.58 (dd, J_{AB} = 10.5 Hz, $J_{5,6B}$ = 4.2 Hz, 1H, H-6'B), 3.52 (dd, $J_{3,4}$ = 3.2 Hz, $J_{2,3}$ = 10.0 Hz, 1H, H-3'), 2.36 (s, 3H, S-Ph-C***H***$_3$), 1.08 (s, 9H, SiPh$_2$-C-(C***H***$_3$)$_3$), residual acetone (1:0.03 ratio; δ 2.18 (s)). ^{13}C{^{1}H} NMR (126 MHz, CDCl$_3$): δ, ppm 166.1 (**C**=O (6-O-Bz)), 165.9 (**C**=O (4-O-Bz)), 164.8 (**C**=O (2-O-Bz)), 154.7 (**C**=O (Fmoc)), 143.6, 143.2, 141.2, 141.2, 138.5, 138.4, 138.0, 135.6, 135.6, 134.0, 133.6, 133.4, 133.3, 133.1, 130.3, 130.0, 129.9, 129.8, 129.7, 129.6, 129.1, 128.7, 128.5, 128.5, 128.2, 128.0, 127.9, 127.9, 127.8, 127.8, 127.7, 127.4, 127.3, 127.2, 127.1, 127.1, 125.4, 125.1, 120.0, 119.9, 93.3 (C-1'(Galα)), 86.4 (C-1(Galβ)), 76.0 (C-3'), 75.4 (C-5), 74.7 (C-2'), 73.5 (C-3), 72.6 (O-**C**H$_2$-Ph), 72.4 (C-4'), 72.2 (O-**C**H$_2$-Ph), 70.1 (C-5'; -**C**H$_2$- (Fmoc)), 69.2 (C-2), 65.9 (C-4), 63.2 (C-6'), 62.9 (C-6), 46.5 (-**C**H- (Fmoc)), 27.0 (SiPh$_2$-C-(**C**H$_3$)$_3$), 21.4 (S-Ph-**C**H$_3$), 19.5 (SiPh$_2$-**C**-(CH$_3$)$_3$).

p-Tolyl 6-O-benzoyl-2,3-di-O-benzyl-α-D-galactopyranosyl-(1→3)-2,4,6-tri-O-benzoyl-1-thio-β-D-galactopyranoside (S5)

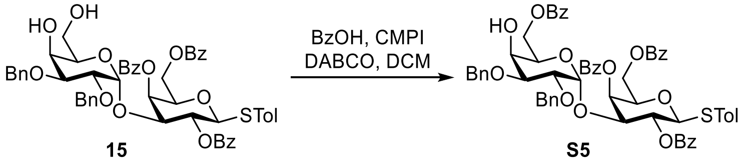

Compound **15** (1.03 g, 1.09 mmol), CMPI (724 mg, 2.83 mmol), and DABCO (501 mg, 4.48 mmol) were dissolved in anhydrous DCM (10 ml) and cooled to -17°C in an ice and salt water bath. The mixture was stirred at -17°C for 10 min, then a solution of benzoic acid (BzOH) (160 mg, 1.31 mmol) in anhydrous DCM (2 ml) was added dropwise. The reaction mixture was stirred overnight reaching 17°C, at which point the reaction was complete (as indicated by TLC, 40% EtOAc in hexane, R_f=0.55). The reaction mixture was diluted with DCM (~60 ml) and washed with sat. $NaHCO_3$. The layers were separated and the aqueous layer was extracted with DCM (2x 10 ml). The combined organic layer was washed with brine, dried over Na_2SO_4, and concentrated under reduced pressure. Disaccharide **S5** was purified by flash chromatography (759 mg, 67% yield, elution at 40% EtOAc in hexane). HRMS (ESI-QTOF) m/z: $[M+H]^+$ Calcd for $C_{61}H_{57}O_{14}S$ 1045.3464; found 1045.3431. ^{1}H NMR (500 MHz, $CDCl_3$): δ, ppm 8.07 - 8.02 (m, 6H), 7.92 (dd, *J* = 1.3, 8.3 Hz, 2H), 7.64 - 7.60 (m, 1H), 7.56 - 7.52 (m, 2H), 7.51 - 7.46 (m, 2H), 7.43 - 7.40 (m, 3H), 7.39 - 7.34 (m, 4H), 7.32 - 7.28 (m, 2H), 7.25 - 7.23 (m, 3H), 7.14 - 7.08 (m, 5H), 7.05 - 7.01 (m, 4H), 5.89 (d, $J_{3,4}$ = 2.6 Hz, 1H, H-4), 5.63 (dd, $J_{1,2}$ = $J_{2,3}$ = 9.9 Hz, 1H, H-2), 5.31 (d, $J_{1,2}$ = 3.4 Hz, 1H, H-1'(Galα)), 4.49 (d, $J_{1,2}$ = 9.9 Hz, 1H, H-1(Galβ)), 4.48 (dd, J_{AB} = 11.6 Hz, $J_{5,6A}$ = 7.3 Hz, 1H, H-6A), 4.45, 4.33 (ABq, $\Delta\delta_{AB}$ = 0.12, J_{AB} = 12.3 Hz, 2H, O-C***H*₂**-Ph), 4.42, 4.19 (ABq, $\Delta\delta_{AB}$ = 0.23, J_{AB} = 11.4 Hz, 2H, O-C***H*₂**-Ph), 4.40 (dd, J_{AB} = 11.6 Hz, $J_{5,6B}$ = 5.2 Hz, 1H, H-6B), 4.37 - 4.32 (m, 2H, H-6'), 4.11 (dd, $J_{3,4}$ = 3.0 Hz, $J_{2,3}$ = 10.0 Hz, 1H, H-3), 3.94 - 3.91 (m, 1H, H-5'), 3.78 - 3.74 (m, 1H, H-5), 3.69 (dd, $J_{1,2}$ = 3.4 Hz, $J_{2,3}$ = 9.8 Hz, 1H, H-2'), 3.38 (dd, $J_{3,4}$ = 3.3 Hz, $J_{2,3}$ = 9.8 Hz, 1H, H-3'), 3.29 (dd, $J_{4,5}$ = 1.3 Hz, $J_{3,4}$ = 3.3 Hz, 1H, H-4'), 2.36 (s, 1H, S-Ph-C$\boldsymbol{H_3}$), residual acetone (~1:0.05 ratio, δ 2.17); H_2O δ 1.58. $^{13}C\{^1H\}$ NMR (126 MHz, $CDCl_3$): δ, ppm 166.1 (**C**=O (6-O-Bz)), 166.0 (**C**=O (6'-O-Bz)), 165.9 (**C**=O (4-O-Bz)), 164.8 (**C**=O (2-O-Bz)), 138.4, 138.2, 138, 134, 133.5, 133.5, 133.4, 130.2, 130.0, 129.9, 129.8, 129.7, 129.6, 129.5, 129, 128.7, 128.7, 128.5, 128.5, 128.4, 128.3, 128.1, 127.8, 127.8, 127.6, 127.3, 92.8 (C-1'(Galα)), 86.6 (C-1(Galβ)), 77.0 (C-3'), 75.4 (C-5), 74.3 (C-2'), 73.3 (C-3), 73.0 (O-**C**H_2-Ph), 72.2 (O-**C**H_2-Ph), 68.8 (C-2), 68.2 (C-5'), 67.5 (C-4'), 65.8 (C-4), 63.8 (C-6'), 63.0 (C-6), 21.4 (S-Ph-**C**H_3).

p-Tolyl 6-O-benzoyl-2,3-di-O-benzyl-4-O-(9-fluorenylmethyloxycarbonyl)-α-D-galactopyranosyl-(1→3)-2,4,6-tri-O-benzoyl-1-thio-β-D-galactopyranoside (2)

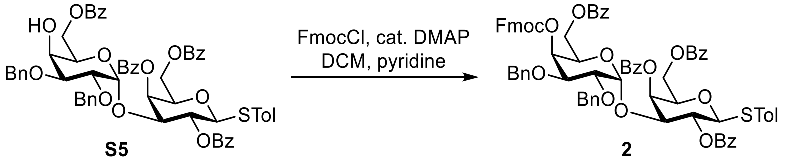

Compound **S5** (739 mg, 0.707 mmol) was dissolved in anhydrous DCM (7 ml) and cooled to 0°C in an ice-water bath. A solution of DMAP (1.7 mg, 0.014 mmol) in pyridine (0.3 ml) was added, followed by the addition of FmocCl (370 mg, 1.43 mmol). The reaction mixture was stirred at 0°C for 1.5 h. The reaction mixture was allowed to reach RT and stirred for an additional 3.5 h until completion (as indicated by TLC, 30% EtOAc in hexane, R_f=0.38). The reaction mixture was diluted with DCM (~10 ml) and toluene (~30 ml) then directly evaporated on silica gel 60 (~2 g, 40-63 μm) and purified by flash chromatography (dry loading method) to obtain **2** as a white foam (836 mg, 93% yield, elution at 40% EtOAc in hexane). HRMS (ESI-QTOF) m/z: $[M+H]^+$ Calcd for $C_{76}H_{67}O_{16}S$ 1267.4144; found 1267.4149. ^{1}H NMR (500 MHz, $CDCl_3$): δ, ppm 8.10 - 8.02 (m, 6H), 7.97 - 7.94 (m, 2H), 7.74 (dd, *J* = 3.0, 7.5 Hz, 2H), 7.65 - 7.62 (m, 1H), 7.57 - 7.54 (m, 1H), 7.52

- 7.47 (m, 5H), 7.46 - 7.43 (m, 3H), 7.40 - 7.32 (m, 8H), 7.26 - 7.20 (m, 2H), 7.15 - 7.06 (m, 10H), 7.03 (d, J = 8.0 Hz, 2H), 5.91 (d, $J_{3,4}$ = 3.0 Hz, 1H, H-4), 5.67 (dd, $J_{1,2}$ = $J_{3,4}$ = 9.9 Hz, 1H, H-2), 5.30 (d, $J_{1,2}$ = 3.4 Hz, 1H, H-1'(Galα)), 4.79 (dd, $J_{4,5}$ = 0.8 Hz, $J_{3,4}$ = 3.1 Hz, 1H, H-4'), 4.61 (d, $J_{1,2}$ = 10.0 Hz, 1H, H-1(Galβ)), 4.52 (dd, J_{AB} = 11.6 Hz, $J_{5,6A}$ = 7.2 Hz, 1H, H-6A), 4.47 (ABq, $\Delta\upsilon_{AB}$ = 32.4 Hz, J_{AB} = 12.5 Hz, 2H, O-C***H*₂**-Ph), 4.45 (dd, J_{AB} = 11.7 Hz, $J_{5,6B}$ = 5.0 Hz, 1H, H-6B), 4.33, 4.22 (ABq, $\Delta\delta_{AB}$ = 0.11, J_{AB} = 11.0 Hz, 2H, O-C***H*₂**-Ph), 4.33 (dd, J_{AB} = 10.3 Hz, $J_{1,9'}$ = 7.1 Hz, 1H, -C***H*₂**- (Fmoc)), 4.29 (dd, J_{AB} = 11.1 Hz, $J_{5,6A}$ = 7.3 Hz, 1H, H-6'A), 4.18 (dd, J_{AB} = 11.2 Hz, $J_{5,6B}$ = 5.4 Hz, 1H, H-6'B), 4.15 (dd, J_{AB} = 10.3 Hz, $J_{1,9'}$ = 7.9 Hz, 1H, -C***H*₂**- (Fmoc)), 4.13 (dd, $J_{3,4}$ = 3.2 Hz, $J_{2,3}$ = 9.8 Hz, 1H, H-3), 4.11 - 4.07 (m, 2H, H-5'; -C***H***- (Fmoc)), 3.90 - 3.86 (m, 1H, H-5), 3.77 (dd, $J_{1,2}$ = 3.4 Hz, $J_{2,3}$ = 10.0 Hz, 1H, H-2'), 3.57 (dd, $J_{3,4}$ = 3.3 Hz, $J_{2,3}$ = 10.0 Hz, 1H), 2.36 (s, 3H, S-Ph-C***H*₃**); H_2O δ 1.62. $^{13}C\{^1H\}$ NMR (126 MHz, $CDCl_3$): δ, ppm 166.1 (**C**=O (6-O-Bz)), 165.8 (**C**=O (4-O-Bz)), 165.8 (**C**=O (6'-O-Bz)), 164.8 (**C**=O (2-O-Bz)), 154.8 (**C**=O (Fmoc)), 143.5, 143.2, 141.3, 141.2, 138.4, 138.3, 137.9, 133.9, 133.7, 133.4, 133.4, 130.2, 129.9, 129.7, 129.7, 129.7, 129.6, 129.4, 129.1, 128.8, 128.6, 128.5, 128.5, 128.3, 128.1, 128.1, 127.9, 127.8, 127.6, 127.6, 127.4, 127.2, 127.2, 125.3, 125.1, 120.0, 120.0, 93.9 (C-1'(Galα)), 86.6 (C-1(Galβ)), 75.8 (C-3'), 75.4 (C-5), 74.1 (C-3), 74.1 (C-2'), 72.8 (O-**C**H₂-Ph), 72.3 (O-**C**H₂-Ph), 72.0 (C-4'), 70.2 (-**C**H₂-(Fmoc)), 68.9 (C-2), 67.2 (C-5'), 66.1 (C-4), 63.0 (C-6), 62.6 (C-6'), 46.6 (-**C**H-(Fmoc)), 21.4 (S-Ph-**C**H₃).

_p_-Tolyl 2,3-di-O-benzyl-6-O-levulinoyl-α-D-galactopyranosyl-(1→3)-2,4,6-tri-O-benzoyl-1-thio-β-D-galactopyranoside (S6)

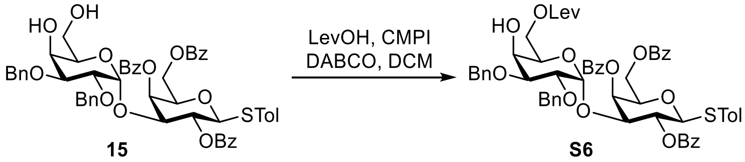

Compound **15** (985 mg, 1.05 mmol), 2-chloro-1-methylpyridinium iodide (CMPI) (697 mg, 2.73 mmol), and 1,4-diazabicyclooctane (DABCO) (483 mg, 4.31 mmol) were dissolved in anhydrous DCM (11 ml) and cooled to -14°C in an ice and salt water bath. The mixture was stirred at -14°C for 10 min, then a solution of levulinic acid (LevOH) (138 mg, 1.19 mmol) in anhydrous DCM (0.6 ml) was added dropwise. The reaction mixture was allowed to reach -9°C over 3.5 h, at which point the reaction was complete (as indicated by TLC, 50% EtOAc in hexane, R_f=0.32). The reaction mixture was diluted with DCM (~65 ml) and washed with sat. $NaHCO_3$. The layers were separated and the aqueous layer was extracted with DCM (2x 5 ml). The combined organic layer was washed with brine, dried over Na_2SO_4, and concentrated under reduced pressure. The crude product was subjected to flash chromatography to obtain sufficiently pure **S6** (910 mg, 83% yield, elution at 60% EtOAc in hexane). HRMS (ESI-QTOF) m/z: $[M+H]^+$ Calcd for $C_{59}H_{59}O_{15}S$ 1039.3569; found 1039.3561. ^1H NMR (500 MHz, $CDCl_3$): δ, ppm 8.08 - 8.04 (m, 4H), 7.92 (dd, J = 1.3, 8.3 Hz, 2H), 7.62 - 7.57 (m, 2H), 7.55 - 7.52 (m, 1H), 7.50 - 7.43 (m, 6H), 7.38 - 7.33 (m, 3H), 7.26 - 7.23 (m, 3H), 7.14 - 7.07 (m, 5H), 7.06 - 7.03 (m, 4H), 6.02 (d, $J_{3,4}$ = 2.8 Hz, 1H, H-4), 5.68 (dd, $J_{1,2}$ = $J_{2,3}$ = 9.8 Hz, 1H, H-2), 5.30 (d, $J_{1,2}$ = 3.3 Hz, 1H, H-1'(Galα)), 5.08 (d, $J_{1,2}$ = 10.0 Hz, 1H, H-1(Galβ)), 4.58 (dd, J_{AB} = 11.5 Hz, $J_{5,6A}$ = 7.1 Hz, 1H, H-6A), 4.46, 4.32 (ABq, $\Delta\delta_{AB}$ = 0.14, J_{AB} = 12.4 Hz, 2H, O-C***H*₂**-Ph), 4.44 (dd, J_{AB} = 11.5 Hz, $J_{5,6B}$ = 5.6 Hz, 1H, H-6B), 4.43, 4.21 (ABq, $\Delta\delta_{AB}$ = 0.20, J_{AB} = 11.4 Hz, 2H, O-C***H*₂**-Ph), 4.37 - 4.33 (m, 2H, H-3; H-5), 4.16 - 4.10 (m, 2H, H-6'A; H-5'), 4.03 (dd, J_{AB} = 11.7 Hz, $J_{5,6B}$ = 3.9 Hz, 1H, H-6'B), 3.83 (ddd, $J_{4,5}$ = 1.4 Hz, $J_{4,OH}$ = 8.2 Hz, $J_{3,4}$ = 3.9 Hz, 1H, H-4'), 3.64 (dd, $J_{1,2}$ = 3.3 Hz, $J_{2,3}$ = 9.8 Hz, 1H, H-2'), 3.44 (dd, $J_{3,4}$ = 3.4 Hz, $J_{2,3}$ = 9.8 Hz, 1H, H-3'), 3.14 (m, 1H, 4'-O***H***), 2.82 - 2.67 (m, 2H, -C***H*₂**- (Lev)), 2.65 - 2.52 (m, 2H, -C***H*₂**- (Lev)), 2.34 (s, 3H, S-Ph-C***H*₃**), 2.11 (s, 3H, -C***H*₃** (Lev)), impurity (~1:0.17 ratio; δ 6.59 (d), 4.81 (dd), 4.73 (dd), 2.74 (m), 2.58 (m), 2.11 (s)). $^{13}C\{^1H\}$ NMR (126 MHz, $CDCl_3$): δ, ppm 206.6 (R**C**OR (Lev)), 172.4 (R**C**OOR (Lev)), 166.2 (**C**=O (6-O-Bz)), 165.9 (**C**=O (4-O-Bz)), 164.8 (**C**=O (2-O-Bz)), 138.4, 138.2, 138.0, 134.0, 133.5, 133.3, 133.3, 130.2, 130.0, 129.9, 129.7, 129.6, 129.6, 129.1, 128.7, 128.6, 128.6, 128.5, 128.4, 128.2, 128.1, 128.0, 128.0, 127.8, 127.8, 127.5, 127.2, 93.2 (C-

1'(Galα)), 85.8 (C-1(Galβ)), 76.8 (C-3'), 75.1 (C-3), 74.3 (C-2'), 73.4 (C-5), 72.9 (O-**C**H_2-Ph), 72.3 (O-**C**H_2-Ph), 69.2 (C-2), 68.1 (C-4'), 67.4 (C-5'), 66.0 (C-4), 63.7 (C-6'), 62.9 (C-6), 37.9 (-**C**H_2- (Lev)), 29.8 (-**C**H_3 (Lev)), 28.0 (-**C**H_2- (Lev)), 21.4 (S-Ph-**C**H_3), impurity (206.6, 172.6, 91.1, 74.7, 73.3, 70.3, 67.3, 63.2, 38.0). *Note: A hard-to-separate impurity was eluted with **S5** (~1:0.17 ratio). The impurity is suspected as a DABCO·LevOH salt that did not appear to interfere with the next reaction step.*

p-Tolyl 2,3-di-O-benzyl-4-O-(9-fluorenylmethyloxycarbonyl)-6-O-levulinoyl-α-D-galactopyranosyl-(1→3)-2,4,6-tri-O-benzoyl-1-thio-β-D-galactopyranoside (3)

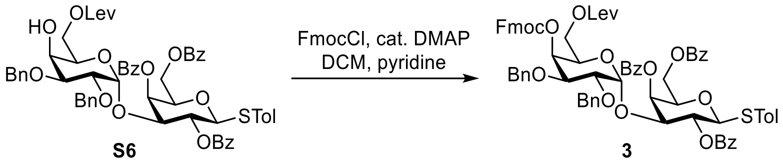

S6 **3**

Compound **S6** (880 mg, 0.847 mmol) was dissolved in anhydrous DCM (8 ml) and cooled to 0°C in an ice-water bath. A solution of DMAP (1.8 mg, 0.015 mmol) in pyridine (0.3 ml) was added, followed by the addition of FmocCl (444 mg, 1.72 mmol). The reaction mixture was stirred at 0°C for 1 h. The solution was initially clear and yellow in color and became gradually turbid and light pink. The reaction mixture was allowed to reach RT and stirred for an additional 3 h until completion (as indicated by TLC, 50% EtOAc in hexane, R_f=0.48). The reaction mixture was diluted with DCM (~40 ml) then directly evaporated on silica gel 60 (~2 g, 40-63 μm) and purified by flash chromatography (dry loading method) to obtain **3** as a white foam (930 mg, 87% yield, elution at 50% EtOAc in hexane). HRMS (ESI-QTOF) m/z: $[M+H]^+$ Calcd for $C_{74}H_{69}O_{17}S$ 1261.4250; found 1261.4258. ^{1}H NMR (500 MHz, $CDCl_3$): δ , ppm 8.10 - 8.06 (m, 4H), 7.96 - 7.93 (m, 2H), 7.75 - 7.72 (m, 2H), 7.67 - 7.63 (m, 1H), 7.62 - 7.58 (m, 1H), 7.58 - 7.53 (m, 1H), 7.53 - 7.44 (m, 8H), 7.40 - 7.34 (m, 4H), 7.25 - 7.20 (m, 2H), 7.16 - 7.05 (m, 10H), 7.03 (d, J = 7.9 Hz, 2H), 6.00 (d, $J_{3,4}$ = 3.1 Hz, 1H, H-4), 5.69 (dd, $J_{1,2}$ = $J_{2,3}$ = 9.8 Hz, 1H, H-2), 5.25 (d, $J_{1,2}$ = 3.3 Hz, 1H, H-1'(Galα)), 5.02 (d, $J_{1,2}$ = 10.0 Hz, 1H, H-1(Galβ)), 4.64 (d, $J_{3,4}$ = 2.7 Hz, 1H, H-4'), 4.58 (dd, $J_{6A,6B}$ = 11.5 Hz, $J_{5,6A}$ = 7.2 Hz, 1H, H-6A), 4.49, 4.42 (ABq, $\Delta\delta_{AB}$ = 0.07, J_{AB} = 12.2 Hz, 2H, 2-O-C***H*****2**-Ph), 4.48 (dd, $J_{6A,6B}$ = 11.5 Hz, $J_{5,6B}$ = 5.4 Hz, 1H, H-6B), 4.34 – 4.28 (m, 3H, H-3; H-5; -C***H*****2**- (Fmoc)), 4.33, 4.24 (ABq, $\Delta\delta_{AB}$ = 0.09, J_{AB} = 11.0 Hz, 2H, 3-O-C***H*****2**-Ph), 4.13 (dd, J_{AB} = 10.1 Hz, $J_{1,9'}$ = 8.0 Hz, 1H, -C***H*****2**- (Fmoc)), 4.10 - 4.05 (m, 1H, -C***H***- (Fmoc)), 4.02 - 3.97 (m, 1H, H-5'), 3.99 (dd, $J_{6A,6B}$ = 14.1 Hz, $J_{5,6A}$ = 7.4 Hz, 2H, H-6'A), 3.91 (dd, $J_{6A,6B}$ = 14.1 Hz, $J_{5,6B}$ = 8.3 Hz, 2H, H-6'B), 3.72 (dd, $J_{1,2}$ = 3.3, $J_{2,3}$ = 10.0 Hz, 1H, H-2'), 3.60 (dd, $J_{3,4}$ = 3.3, $J_{2,3}$ = 10.0 Hz, 1H, H-3'), 2.77 - 2.66 (m, 2H, -C***H*****2**- (Lev)), 2.55 - 2.51 (m, 2H, -C***H*****2**- (Lev)), δ 2.34 (s, 3H, S-Ph-C***H*****3**), δ 2.10 (s, 2H, -C***H*****3**- (Lev)). $^{13}C\{^1H\}$ NMR (126 MHz, $CDCl_3$): δ , ppm 206.5 (R**C**OR (Lev)), 172.1 (R**C**OOR (Lev)), 166.2 (**C**=O (Bz)), 165.9 (**C**=O (Bz)), 164.9 (**C**=O (Bz)), 154.8 (**C**=O (Fmoc)), 143.6, 143.2, 141.3, 141.2, 138.4, 138.3, 137.9, 133.9, 133.8, 133.3, 133.3, 130.2, 130.0, 129.8, 129.7, 129.6, 129.6, 129.2, 128.8, 128.5, 128.5, 128.2, 128.1, 128.0, 127.9, 127.8, 127.7, 127.6, 127.4, 127.2, 127.2, 125.4, 125.1, 120.0, 120.0, 94.4 (C-1'(Galα)), 86.0 (C-1(Galβ)), 75.7 (C-3'), 75.2 (C-5), 74.5 (C-3), 74.0 (C-2'), 72.9 (2-O-**C**H_2-Ph), 72.2 (3-O-**C**H_2-Ph), 72.1 (C-4'), 70.2 (-**C**H_2- (Fmoc)), 69.3 (C-2), 67.2 (C-5'), 66.4 (C-4), 63.0 (C-6), 62.3 (C-6'), 46.5 (-**C**H- (Fmoc)), 37.8 (-**C**H_2- (Lev)), 29.8 (-**C**H_3 (Lev)), 27.9 (-**C**H_2- (Lev)), 21.4 (S-Ph-**C**H_3).

p-Tolyl 4,6-di-O-benzoyl-2,3-di-O-benzyl-α-D-galactopyranosyl-(1→3)-2,4,6-tri-O-benzoyl-1-thio-β-D-galactopyranoside (4)

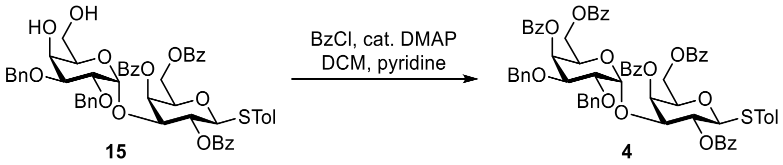

Compound **15** (130 mg, 0.138 mmol) was dissolved in anhydrous DCM (2.6 ml) and cooled to 0°C in an ice-water bath. A solution of DMAP (1.3 mg, 0.011 mmol) in pyridine (0.5 ml) was added, followed by the addition of BzCl (0.03 ml, 0.258 mmol). The reaction mixture was stirred at 0°C for 1 h, then at RT for an additional 5 h until completion (as indicated by TLC, 30% EtOAc in hexane, R_f=0.43). The reaction mixture was diluted with DCM (30 ml) and washed with aqueous HCl (0.1 M, ~20 ml). Small portions of aqueous HCl (0.1 M) were added until pH~4 was reached. The layers were separated and the aqueous layer was acidified to pH~1 and extracted with DCM (5 ml). The combined organic layer was washed with brine, then dried over Na_2SO_4, and concentrated under reduced pressure. Disaccharide **4** was purified by flash chromatography (137 mg, 86% yield, elution at 40% EtOAc in hexane). HRMS (ESI-QTOF) m/z: $[M+H]^+$ Calcd for $C_{68}H_{61}O_{15}S$ 1149.3726; found 1149.3726. ^{1}H NMR (500 MHz, $CDCl_3$): δ, ppm 8.09 - 8.05 (m, 4H), 8.01 (dd, J = 1.1, 8.2 Hz, 2H), 7.97 (dd, J = 1.1, 8.3 Hz, 2H), 7.85 (dd, J = 1.1, 8.3 Hz, 2H), 7.65 - 7.61 (m, 1H), 7.58 - 7.47 (m, 5H), 7.46 - 7.42 (m, 3H), 7.41 - 7.35 (m, 7H), 7.32 - 7.28 (m, 2H), 7.17 - 7.12 (m, 4H), 7.10 - 7.00 (m, 8H), 5.90 (d, $J_{3,4}$ = 3.0 Hz, 1H, H-4), 5.67 (dd, $J_{1,2}$ = $J_{2,3}$ = 9.9 Hz, 1H, H-2), 5.32 (d, $J_{1,2}$ = 3.5 Hz, 1H, H-1(Galα)), 5.15 (d, $J_{3,4}$ = 2.7 Hz, 1H, H-4'), 4.58 (d, $J_{1,2}$ = 10.0 Hz, 1H, H-1(Galβ)), 4.50 (dd, J_{AB} = 11.6 Hz, $J_{5,6A}$ = 7.2 Hz, 1H, H-6A), 4.43 (dd, J_{AB} = 11.5 Hz, $J_{5,6B}$ = 5.1 Hz, 1H, H-6B), 4.43, 4.34 (ABq, $\Delta\delta_{AB}$ = 0.09, J_{AB} = 12.1 Hz, 2H, O-C***H***$_2$-Ph), 4.36, 4.17 (ABq, $\Delta\delta_{AB}$ = 0.19, J_{AB} = 11.1 Hz, 2H), 4.26 (dd, J_{AB} = 12.4 Hz, $J_{5,6A}$ = 9.0 Hz, 1H, H-6'A), 4.15 - 4.11 (m, 4H, H-3; H-6'B; H-5'), 3.82 - 3.77 (m, 2H, H-2'; H-5), 3.61 (dd, $J_{3,4}$ = 3.2 Hz, $J_{2,3}$ = 10.0 Hz, 1H, H-3'), 2.37 (s, 3H, S-Ph-C***H***$_3$). ^{13}C{^{1}H} NMR (126 MHz, $CDCl_3$): δ, ppm 166.1 (**C**=O (6-O-Bz)), 165.9 (**C**=O (4-O-Bz)), 165.8 (**C**=O (6'-O-Bz)), 165.7 (**C**=O (4'-O-Bz)), 164.9 (**C**=O (2-O-Bz)), 138.4, 138.1, 137.9, 133.9, 133.8, 133.5, 133.4, 133.4, 133.2, 130.3, 129.9, 129.9, 129.7, 129.6, 129.6, 129.4, 129.1, 128.8, 128.6, 128.5, 128.5, 128.4, 128.3, 128.1, 128.0, 127.7, 127.4, 127.3, 93.7 (C-1'(Galα)), 86.6 (C-1(Galβ)), 76.0 (C-3'), 75.4 (C-5), 73.8 (C-3), 73.3 (C-2'), 72.5 (O-**C**H$_2$-Ph), 72.0 (O-**C**H$_2$-Ph), 68.9 (C-2), 68.3 (C-4'), 67.7 (C-5), 65.8 (C-4), 63.0 (C-6), 62.9 (C-6'), 21.4 (S-Ph-**C**H$_3$).

p-Tolyl 2,3-di-O-benzyl-4,6-O-(4-methoxyphenyl)methylene-α-D-galactopyranosyl-(1→3)-2-O-benzoyl-4,6-di-O-benzyl-1-thio-β-D-galactopyranoside (S7)

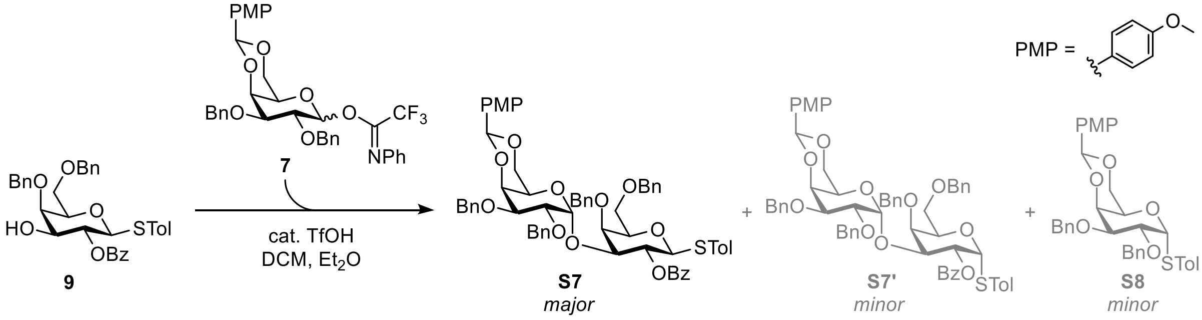

Glycosyl acceptor **9** (178 mg, 0.312 mmol) and glycosyl donor **7** (299 mg, 0.460 mmol) were co-evaporated from anhydrous toluene and dried overnight under high vacuum. The donor, acceptor, and freshly microwave (MW)-activated and crushed molecular sieves (4Å, ~1.5 g) were suspended in anhydrous DCM (3.3 ml) and anhydrous diethyl ether (Et_2O) (2.5 ml). The mixture was stirred at RT for 50 min, then cooled to -10°C in an ice and salt water bath. The reaction mixture was stirred

for 15 min at -10°C, after which TfOH (10 µl, 0.11 mmol) was added. The reaction mixture turned red. The reaction mixture was allowed to reach 0°C over 1 h, at which point the solution was colorless. An additional portion of TfOH (5 µl, 0.06 mmol) was added, and the reaction mixture was stirred at 0°C for 1 h, after which the glycosyl acceptor was fully consumed (as indicated by TLC, **9** 30% EtOAc in hexane, R_f=0.39). The reaction mixture was filtered through celite, directly onto sat. $NaHCO_3$ (~10 ml). The celite was washed with DCM (~50 ml) and the combined filtrate was washed with sat. $NaHCO_3$. The layers were separated and the aqueous layer was extracted with DCM (10 ml). The combined organic layer was washed with brine, dried over Na_2SO_4, filtered, and concentrated under reduced pressure. Compound **S7** was purified by flash chromatography (146 mg, 45% yield, elution at 35% EtOAc in hexane). A minor byproduct was eluted at 30% EtOAc in hexane, and further purified by Prep-HPLC to obtain **S7'** for characterization (~4:1 ratio of **S7** to **S7'**). An additional byproduct **S8** was isolated (14% conversion from donor **7**, elution at 30% EtOAc in hexane). Epimer **S7** (major): HRMS (ESI-QTOF) m/z: $[M+H]^+$ Calcd for $C_{62}H_{63}O_{12}S$ 1031.4035; found 1031.4019. ^{1}H NMR (500 MHz, d_6-acetone): δ, ppm 8.08 (dd, J = 1.3, 8.3 Hz, 2H), 7.68 (tt, J = 1.4, 7.4 Hz, 1H), 7.56 (t, J = 7.8 Hz, 2H), 7.39 - 7.29 (m, 13H), 7.28 - 7.21 (m, 9H), 7.17 - 7.14 (m, 2H), 7.04 (d, J = 7.9 Hz, 2H), 6.86 (d, J = 8.9 Hz, 2H), 5.62 (m, 1H, H-2), 5.34 (s, 1H, PMPC***H***OO), 5.30 (d, $J_{1,2}$ = 3.2 Hz, 1H, C-1'(Galα)), 5.08, 4.40 (ABq, $\Delta\delta_{AB}$ = 0.68, J_{AB} = 11.9 Hz, 1H, O-C***H₂***-Ph), 4.97 (d, $J_{1,2}$ = 9.8 Hz, 1H, H-1(Galβ)), 4.79 (ABq, $\Delta\upsilon_{AB}$ = 50.5 Hz, J_{AB} = 11.2, 2H, O-C***H₂***-Ph), 4.58 (ABq, $\Delta\upsilon_{AB}$ = 22.4 Hz, J_{AB} = 11.7 Hz, 2H, O-C***H₂***-Ph), 4.50 (ABq, $\Delta\upsilon_{AB}$ = 28.2 Hz, J_{AB} = 11.9 Hz, 2H, O-C***H₂***-Ph), 4.25 (d, $J_{2,3}$ = 8.9 Hz, 1H, H-3), 4.17 (d, $J_{3,4}$ = 2.3 Hz, 1H, H-4), 4.15 (dd, $J_{4,5}$ = 1.0 Hz, $J_{3,4}$ = 3.2 Hz, 1H, H-4'), 3.97 (dd, $J_{1,2}$ = 3.2 Hz, $J_{2,3}$ = 10.2 Hz, 1H, H-2'), 3.95 (t, $J_{5,6}$ = 6.1 Hz, 1H, H-5), 3.92 (dd, $J_{3,4}$ = 3.2 Hz, $J_{2,3}$ = 10.2 Hz, 1H, H-3'), 3.76 (s, 3H, ***Me***OPhCHOO), 3.62 (ddd, $\Delta\upsilon_{AB}$ = 18.3 Hz, J_{AB} = 9.6 Hz, $J_{5,6}$ = 6.2 Hz, 2H, H-6), 3.53 (d, $J_{5,6A}$ = 0.9 Hz, 1H, H-5'), 3.50 (dd, J_{AB} = 12.4 Hz, $J_{5,6A}$ = 1.6 Hz, 1H, H-6'A), 3.40 (d, J_{AB} = 12.4 Hz, 1H, H-6'B), 2.27 (s, 3H, S-Ph-C***H₃***), H_2O and HOD (δ 2.83 (s); δ 2.80 (t)). ^{13}C{^{1}H} NMR (126 MHz, d_6-acetone): δ, ppm 165.8 (**C**=O(Bz)), 160.8 (*p*-PMP), 140.5, 140.0, 139.7, 139.5, 138.1, 134.2, 132.8, 132.2, 131.2, 130.8, 130.5, 130.3, 129.5, 129.1, 129, 128.9, 128.8, 128.5, 128.3, 128.3, 128.2, 128.1, 128, 127.7, 113.9, 100.9 (PMP**C**HOO), 100.8 (C-1'(Galα)), 87.1 (C-1(Galβ)), 83.2 (C-3), 78.3 (C-5), 77.1 (C-3'), 76.9 (C-2'), 76.2 (C-4), 75.4 (O-**C**H₂-Ph), 74.7 (O-**C**H₂-Ph), 74.4 (C-4'), 73.7 (O-**C**H₂-Ph), 71.5 (O-**C**H₂-Ph), 71.0 (C-2), 69.8 (C-6), 69.3 (C-6'), 64.1 (C-5'), 55.5 (***Me***OPhCHOO), 21.0 (S-Ph-**C**H₃). Epimer **S7'** (minor): HRMS (ESI-QTOF) m/z: $[M+H]^+$ Calcd for $C_{62}H_{63}O_{12}S$ 1031.4035; found 1031.4042. ^{1}H NMR (500 MHz, d_6-acetone): δ, ppm 8.10 (dd, J = 1.3, 8.2 Hz, 2H), 7.71 (tt, J = 1.5, 7.4 Hz, 1H), 7.61 (t, J = 7.8 Hz, 2H), 7.42 - 7.32 (m, 13H), 7.26 - 7.17 (m, 11H), 7.02 (d, J = 8.3 Hz, 2H), 6.89 (d, J = 8.8 Hz, 2H), 5.82 (d, $J_{1,2}$ = 5.6 Hz, 1H, H-1(Galα)), 5.76 (dd, $J_{1,2}$ = 5.6 Hz, $J_{2,3}$ = 10.5 Hz, 1H, H-2), 5.45 (d, $J_{1,2}$ = 3.3 Hz, 1H, H-1'(Galα)), 5.41 (s, 1H, PMPC***H***OO), 5.12, 4.41 (ABq, $\Delta\delta_{AB}$ = 0.71, J_{AB} = 11.6 Hz, 2H, O-C***H₂***-Ph), 4.83 (ABq, $\Delta\upsilon_{AB}$ = 35.0 Hz, J_{AB} = 11.2 Hz, 2H, O-C***H₂***-Ph), 4.61 (t, $J_{5,6}$ = 6.3 Hz, 1H, H-5), 4.60 (ABq, $\Delta\upsilon_{AB}$ = 28.3 Hz, J_{AB} = 12.2 Hz, 2H, O-C***H₂***-Ph), 4.48 (ABq, $\Delta\upsilon_{AB}$ = 17.2 Hz, J = 11.9 Hz, 2H, O-C***H₂***-Ph), 4.35 (dd, $J_{3,4}$ = 2.8 Hz, $J_{2,3}$ = 10.5 Hz, 1H, H-3), 4.27 (d, $J_{3,4}$ = 1.9 Hz, 1H, H-4), 4.25 (dd, $J_{4,5}$ = 0.8 Hz, $J_{3,4}$ = 3.2 Hz, 1H, H-4'), 4.05 (dd, $J_{1,2}$ = 3.3 Hz, $J_{2,3}$ = 10.2 Hz, 1H, H-2'), 3.99 (dd, $J_{3,4}$ = 3.3, $J_{2,3}$ = 10.2 Hz, 1H, H-3'), 3.78 (s, 3H, ***Me***OPhCHOO), 3.70 (dd, J_{AB} = 12.4 Hz, $J_{5,6A}$ = 1.1 Hz, 1H, H-6'A), 3.70 (1H, m, 1H, H-5'), 3.66 (dd, J_{AB} = 9.7 Hz, $J_{5,6A}$ = 6.0 Hz, 1H, H-6A), 3.61 (dd, J_{AB} = 12.6 Hz, $J_{5,6B}$ = 2.0 Hz, 1H, H-6'B), 3.57 (dd, J_{AB} = 9.7 Hz, $J_{5,6B}$ = 6.4 Hz, 1H, H-6B), 2.24 (s, 3H, S-Ph-C***H₃***), H_2O and HOD (δ 2.83 (s); δ 2.80 (br)). ^{13}C{^{1}H} NMR (126 MHz, d_6-acetone): δ, ppm 166.2 (**C**=O(Bz)), 160.8 (*p*-PMP), 140.4, 140, 139.7, 139.5, 138.4, 134.4, 133.8, 132.2, 130.9, 130.6, 130.6, 130.5, 129.6, 129.1, 129.0, 128.9, 128.8, 128.4, 128.4, 128.3, 128.2, 128.1, 128.0, 127.8, 114.0, 101.0 (PMP**C**HOO), 100.2 (C-1'(Galα)), 87.7 (C-1(Galα)), 78.4 (C-3), 77.0 (C-3'; C-2'), 76.7 (C-4), 75.6 (O-**C**H₂-Ph), 74.8 (O-**C**H₂-Ph), 74.5 (C-4'), 73.7 (O-**C**H₂-Ph), 71.7 (C-5), 71.4 (O-**C**H₂-Ph), 71.1 (C-2), 69.7 (C-6), 69.5 (C-6'), 64.1 (C-5'), 55.5 (***Me***OPhCHOO), 21.0 (S-Ph-**C**H₃). Byproduct **S8**: HRMS (ESI-QTOF) m/z: $[M+H]^+$ Calcd for $C_{35}H_{37}O_6S$ 585.2305; found 585.2298. ^{1}H NMR (500 MHz, d_6-acetone): δ, ppm 7.47 - 7.45 (m, 2H), 7.43 - 7.40 (m, 6H), 7.35 - 7.30 (m, 4H), 7.29 - 7.25 (m, 2H), 7.17 - 7.14 (m, 2H), 6.91 - 6.89 (m, 2H), 5.90 (d, $J_{1,2}$ = 5.3 Hz, 1H, H-1(Galα)), 5.61 (s, 1H, PMPC***H***OO), 4.85, 4.75 (ABq, $\Delta\delta_{AB}$ = 0.10, J_{AB} = 11.4 Hz, 2H, 2-O-C***H₂***-Ph), 4.80, 4.75 (ABq, $\Delta\delta_{AB}$ = 0.05, J_{AB} = 12.0 Hz,

2H, 3-O-C$\boldsymbol{H}_2$-Ph), 4.59 (dd, $J_{4,5}$ = 1.0 Hz, $J_{3,4}$ = 3.6 Hz, 1H, H-4), 4.32 (dd, $J_{1,2}$ = 5.3 Hz, $J_{2,3}$ = 10.1 Hz, 1H, H-2), 4.18 (m, 1H, H-5), 4.14 (dd, $J_{5,6A}$ = 1.8 Hz, J_{AB} = 12.5 Hz, 1H, H-6A), 4.02 (dd, $J_{5,6B}$ = 1.5 Hz, J_{AB} = 12.4 Hz, 1H, H-6B), 3.92 (dd, $J_{3,4}$ = 3.6 Hz, $J_{2,3}$ = 10.1 Hz, 1H, H-3), 3.78 (s, 3H, ***Me***OPhCHOO), 2.31 (s, 3H, S-Ph-C$\boldsymbol{H}_3$), H_2O and HOD (δ 2.82 (s); δ 2.79 (t)). $^{13}C\{^1H\}$ NMR (126 MHz, , d_6-acetone): δ, ppm 160.8 (*p*-PMP), 140.0, 139.8, 137.8, 133.0, 132.2, 131.8, 130.5, 129.0, 129.0, 128.5, 128.4, 128.3, 128.2, 128.1, 114.0 (*m*-PMP), 101.3 (PMP**C**HOO), 88.7 (C-1), 77.4 (C-3), 76.4 (C-2), 74.5 (C-4), 72.8 (2-O-**C**H_2-Ph), 71.7 (3-O-**C**H_2-Ph), 69.7 (C-6), 64.5 (C-5), 55.5 (***Me***OPhCHOO), 21.0 (S-Ph-**C**H_3). *Note: Two epimers were formed via anomerization of the reducing thiogalactopyranoside.*[13] *The configuration of the anomeric carbons C-1 and C-1' was assigned with additional information from non-decoupled ^1H-^{13}C HSQC (vide infra, ^1H-^{13}C non-decoupled HSQC spectra of* ***S7*** *and* ***S7'****);* ***S7****: 100.9 (d, J=159Hz, PMP**C**HOO, axial), 100.8 (d, J=169Hz, C-1' (α)), 87.1 (d, J=157Hz, C-1 (β)).* ***S7'****: 101.0 (d, J=160Hz, PMP**C**HOO, axial), 100.2 (d, J=169Hz, C-1' (α)), 87.7 (d, J=170Hz, C-1 (α)). Note 2: Untreated MS were used (not acid washed), which appeared to quench the reaction over time. An additional portion of TfOH was added to push the reaction to completion.*

p-Tolyl 2,3-di-O-benzyl-α-D-galactopyranosyl-(1→3)-2-O-benzoyl-4,6-di-O-benzyl-1-thio-β-D-galactopyranoside (S9)

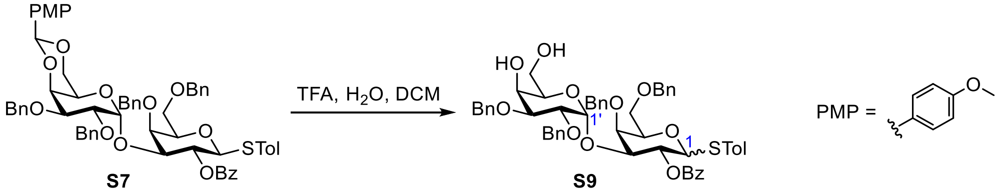

Compound **S7** (120 mg, 0.116 mmol) was dissolved in DCM (9 ml). TDW (0.1 ml) was added and the reaction mixture was cooled to 0°C. TFA (1 ml) was added dropwise. The reaction mixture was stirred at 0°C for 30 min, until completion (as indicated by TLC, 50% EtOAc in hexane, R_f=0.25). The reaction mixture was diluted with DCM (20 ml) and washed with sat. $NaHCO_3$. The layers were separated and the aqueous layer was extracted with DCM (5 ml). The combined organic layer was washed with brine, dried over Na_2SO_4, filtered and concentrated under reduced pressure. Disaccharide **S9** was purified by flash chromatography (81 mg, 76% yield, elution at 70% EtOAc in hexane), obtained as an inseparable diastereomeric mixture of C-1 (reducing) anomers (β:α, 4:1), and used as a mixture for the next step. HRMS (ESI-QTOF) m/z: $[M+NH_4]^+$ Calcd for $C_{54}H_{60}NO_{11}S$ 930.3882; found 930.3843. ^1H NMR (400 MHz, $CDCl_3$): δ, ppm 8.02 (dd, J = 1.2, 8.3 Hz, 2H), 7.62 - 7.57 (m, 1H), 7.48 - 7.43 (m, 3H), 7.36 - 7.27 (m, 14H), 7.26 - 7.24 (m, 10H), 7.17 - 7.09 (m, 3H), 6.99 (d, J = 7.9 Hz, 2H), 5.64 (dd, $J_{1,2}$ = $J_{2,3}$ = 9.8 Hz, 1H, H-2), 5.00 (d, $J_{1,2}$ = 3.4 Hz, 1H, H-1'(Galα)), 4.97, 4.41 (ABq, $\Delta\delta_{AB}$ = 0.56, J_{AB} = 11.8 Hz, 2H, O-C$\boldsymbol{H}_2$-Ph), 4.81, 4.59 (ABq, J_{AB} = 11.6 Hz, 2H, O-C$\boldsymbol{H}_2$-Ph), 4.68 (d, $J_{1,2}$ = 9.8 Hz, 1H, H-1(Galβ)), 4.55 (ABq, $\Delta\upsilon_{AB}$ = 37.7 Hz, J_{AB} = 11.5 Hz, 2H, O-C$\boldsymbol{H}_2$-Ph), 4.44 (ABq, $\Delta\upsilon_{AB}$ = 25.6 Hz, J_{AB} = 11.5 Hz, 2H, O-C$\boldsymbol{H}_2$-Ph), 4.02 (d, $J_{3,4}$ = 2.6 Hz, 1H, H-4), 3.84 - 3.78 (m, 2H, H-3; H-2'), 3.72 - 3.54 (m, 7H, H-5; H-5'; H-6'; H-3'; H-4'), 3.41 (dABq, $\Delta\upsilon$ = 23.7 Hz, J_{AB} = 12.0 Hz, $J_{5,6}$ = 4.5 Hz, 2H, H-6), 2.29 (s, 3H, S-Ph-C$\boldsymbol{H}_3$), minor epimer (1:0.25 ratio, δ 8.05 (dd), 7.62 - 7.57 (m), 7.48 - 7.43 (m), 7.36 - 7.27 (m), 7.26 - 7.24 (m), 7.17 - 7.09 (m), 6.37 (d, H-1), 5.78 (dd, H-2), 5.10 (d, H-1'), 4.85 (d), 4.61 (d), 4.53 (d), 4.31 (dd), 4.28 (t), 4.10 (d), 3.89 (dd), 3.84 - 3.78 (m), 3.74 (dd), 3.72 - 3.54 (m), 3.51 (dd), 2.32 (s). $^{13}C\{^1H\}$ NMR (126 MHz, $CDCl_3$): δ, ppm 165.5 (**C**=O(Bz)), 138.9, 138.3, 138.1, 137.8, 137.8, 133.4, 132.9, 130.1, 129.9, 129.9, 129.8, 129.6, 129.4, 128.7, 128.6, 128.6, 128.6, 128.5, 128.5, 128.1, 128.1, 128.0, 127.9, 127.9, 127.8, 127.6, 127.2, 98.6 (C-1'(Galα)), 87.0 (C-1(Galβ)), 81.6, 77.7, 77.3, 75.7, 74.7, 74.3, 74.1 (C-4), 73.6, 72.4, 70.4 (C-2), 69.9, 69.3, 68.8, 63.1, 21.2, minor epimer (δ 165.7 (**C**=O(Bz)), 138.5, 138.1, 137.8, 137.5, 133.7, 129.4, 128.6, 128.3, 128.2, 128.0, 127.6, 98.2 (C-1'), 93.3 (C-1), 75.2, 74.6, 73.6, 72.7, 72.4, 70.1, 69.8, 69.1, 67.9, 63.0 (C-6), 21.1 (S-Ph-**C**H_3). *Note: Two epimers were formed via anomerization of the reducing thiogalactopyranoside. The configuration of the anomeric carbons C-1 and*

C-1′ was assigned with additional information from non-decoupled ^{1}H-^{13}C HSQC (vide infra, ^{1}H-^{13}C non-decoupled HSQC spectrum of ***S7****); major: 98.6 (d, J=168Hz, C-1′ (α)), 87.0 (d, J=156Hz, C-1 (β)). minor: 98.2 (d, J=168Hz, C-1′ (α)), 93.4 (d, J=184Hz, C-1 (α)). Note 2: Integration was normalized for the major epimer.*

p-Tolyl 4,6-di-O-benzoyl-2,3-di-O-benzyl-α-D-galactopyranosyl-(1→3)-2-O-benzoyl-4,6-di-O-benzyl-1-thio-β-D-galactopyranoside (5)

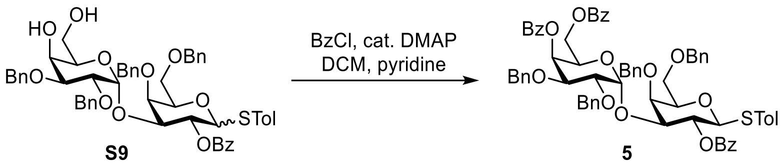

Compound **S9** (34.9 mg, 38.2 μmol) was dissolved in anhydrous DCM (0.8 ml) and cooled to 0°C in an ice-water bath. BzCl (0.014 ml, 0.082 mmol) was added dropwise, followed by the addition of DMAP (1 mg, 8 μmol) in pyridine (0.2 ml) solution. The reaction mixture was stirred at RT overnight, until completion (as indicated by TLC, 40% EtOAc in hexane, R_f=0.63). The reaction mixture was diluted with DCM (20 ml) and washed with aqueous HCl (1 M, 20 ml). The layers were separated and the aqueous phase was extracted with DCM (2x 5 ml). The combined organic phase was washed with sat. $NaHCO_3$ (2x 40 ml), then brine, dried over Na_2SO_4, and concentrated under reduced pressure. Compound **5** was purified by flash chromatography (33.2 mg, 77% yield, elution at 35% EtOAc in hexane). HRMS (ESI-QTOF) m/z: $[M+H]^+$ Calcd for $C_{68}H_{65}O_{13}S$ 1121.4140; found 1121.4137. ^{1}H NMR (500 MHz, $CDCl_3$): δ , ppm 8.06 (dd, *J* = 1.2, 8.2 Hz, 2H), 7.99 - 7.94 (m, 4H), 7.58 - 7.55 (m, 1H), 7.53 - 7.47 (m, 2H), 7.44 - 7.39 (m, 3H), 7.38 - 7.27 (m, 14H), 7.23 - 7.16 (m, 12H), 7.02 (d, *J* = 7.9 Hz, 2H), 5.74 (dd, $J_{1,2}$ = $J_{2,3}$ = 9.8 Hz, 1H, H-2), 5.36 (m, 1H, H-4'), 5.18 (d, $J_{1,2}$ = 2.8 Hz, 1H, H-1'(Galα)), 5.08, 4.42 (ABq, ΔδAB = 0.66, J_{AB} = 11.6 Hz, 2H, O-C***H***$_2$-Ph), 4.78, 4.59 (ABq, $\Delta\delta_{AB}$ = 0.18, J_{AB} = 11.6 Hz, 2H, O-C***H***$_2$-Ph), 4.65 (d, $J_{1,2}$ = 9.8 Hz, 1H, H-1(Galβ)), 4.61, 4.37 (ABq, $\Delta\delta_{AB}$ = 0.24, J_{AB} = 11.1 Hz, 2H, O-C***H***$_2$-Ph), 4.45 (ABq, $\Delta\upsilon_{AB}$ = 26.9 Hz, J_{AB} = 11.7 Hz, 2H, O-C***H***$_2$-Ph), 4.25 - 4.20 (m, 1H, H-5'), 4.13 (dd, J_{AB} = 11.1 Hz, $J_{5,6A}$ = 7.0 Hz, 1H, H-6'A), 4.06 (d, $J_{3,4}$ = 2.1 Hz, 1H, H-4), 4.03 - 3.90 (4H, m, H-6'B; H-2'; H-3'; H-3), 3.66 (dd, J_{AB} = 9.1 Hz, $J_{5,6A}$ = 5.7 Hz, 1H, H-6A), 3.63 - 3.54 (m, 2H, H-6B; H-5), 2.32 (s, 3H, S-Ph-C***H***$_3$), minor epimer (1:0.1 ratio, δ 6.41 (d, H-1), 5.87 (dd, H-2), 2.34 (s)). ^{13}C{^{1}H} NMR (126 MHz, $CDCl_3$): δ , ppm 165.7 (**C**=O(6'-O-Bz)), 165.6 (**C**=O(4'-O-Bz)), 165.1 (**C**=O(2-O-Bz)), 138.8, 138.0, 138.0, 137.9, 137.8, 133.5, 133.2, 132.9, 129.9, 129.8, 129.7, 129.7, 129.7, 129.7, 129.6, 129.4, 128.6, 128.5, 128.5, 128.4, 128.3, 128.3, 128.1, 128.0, 127.9, 127.8, 127.8, 127.6, 127.3, 96.5 (C-1'(Galα)), 87.2 (C-1(Galβ)), 79.3 (C-3), 77.7 (C-5), 76.5 (C-3'), 74.5 (C-2'; O-**C**H$_2$-Ph), 74.3 (O-**C**H$_2$-Ph), 73.6 (O-**C**H$_2$-Ph), 72.6 (C-4), 71.8 (O-**C**H$_2$-Ph), 69.6 (C-2), 68.7 (C-6), 68.1 (C-4'), 67.6 (C-5'), 62.5 (C-6'), 29.8, 21.2 (S-Ph-**C**H$_3$). *Note: Compound* ***5*** *was obtained as a hard-to-separate 9:1(β:α) diastereomeric mixture of C-1 epimers carried over from the previous step (see preparation of* ***S9*** *and notes).*

Oligosaccharide synthesis

Scheme S2. Outline of the synthesis of mono-, di-, and trisaccharides.

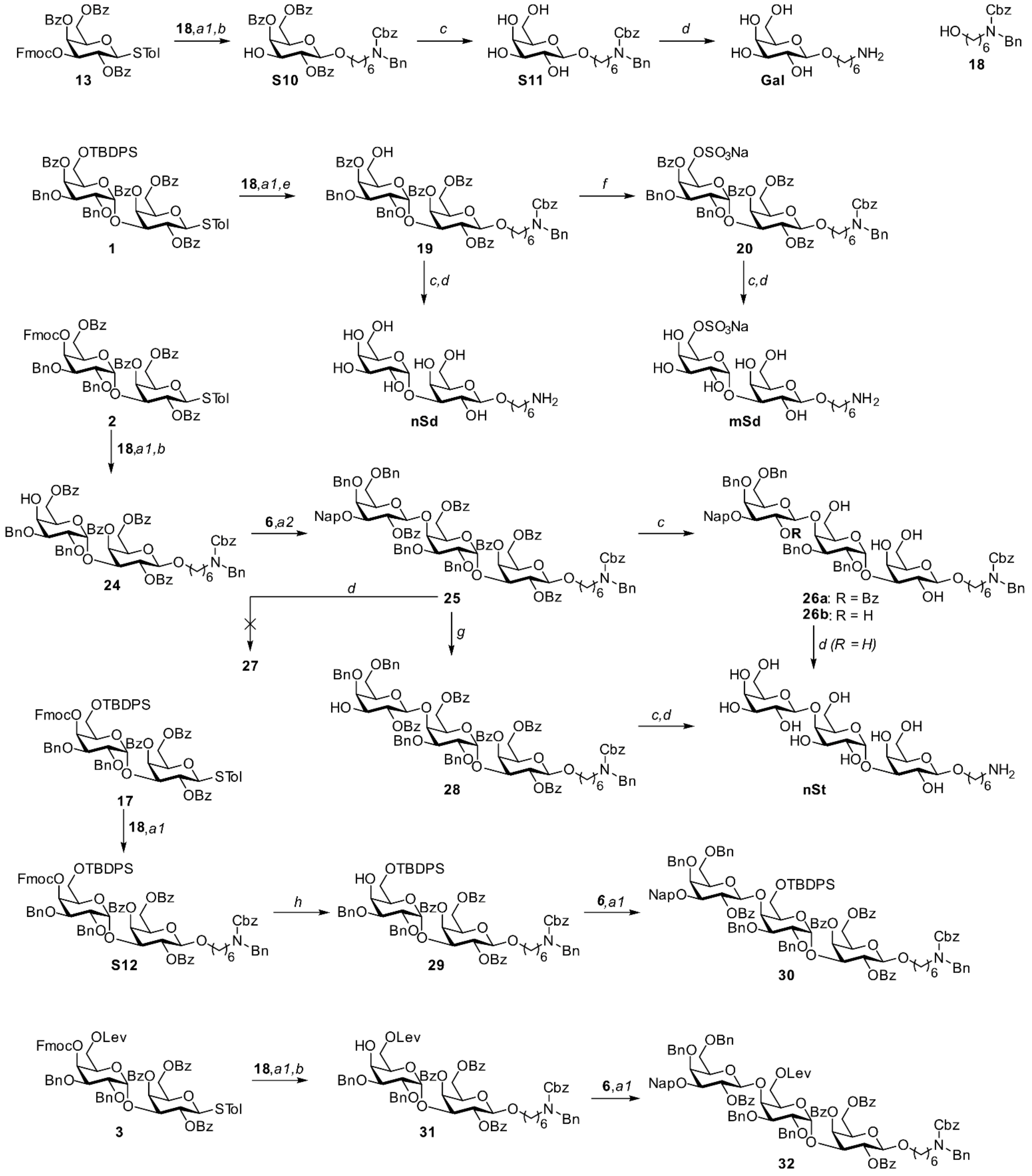

a1. NIS, TfOH, DCM, 1,4-dioxane; a2. NIS, TMSOTf, DCM, 1,4-dioxane; b. morpholine, DMF; c. NaOMe, MeOH, DCM; d. H_2, $Pd(OH)_2/C$; e. TBAF, THF; f. SO_3 ·py, DMF; g. DDQ, DCM, H_2O; h. piperidine, DMF.

Scheme S3. Outline of the synthesis of tri- and tetrasaccharides.

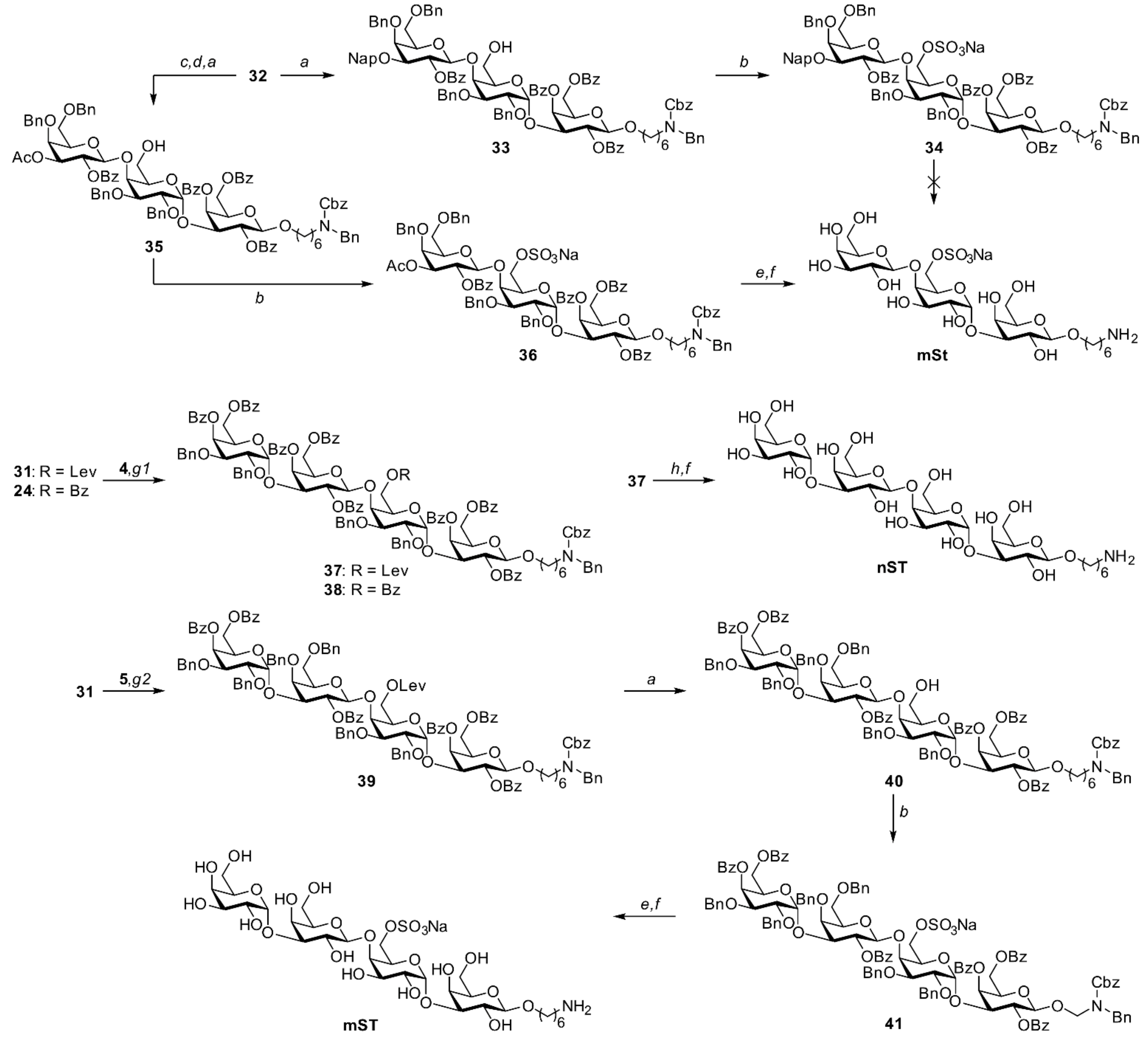

a. $NH_2NH_2{\cdot}HOAc$*, pyridine, AcOH; b.* $SO_3{\cdot}py$*, DMF; c. DDQ, DCM,* H_2O*; d.* Ac_2O*, cat. DMAP, DCM, pyridine; e. LiOH, MeOH,* H_2O*; f.* H_2*,* $Pd(OH)_2/C$*; g1. NIS, TfOH, DCM, 1,4-dioxane; g2. NIS, TMSOTf, DCM, 1,4-dioxane; h. NaOMe, MeOH, DCM.*

N-benzyl-N-benzyloxycarbonyl-6-aminohexanol (18)

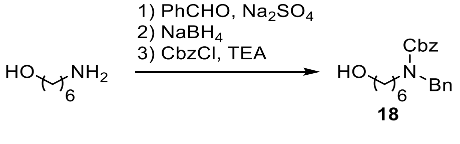

Compound **18** was synthesized from 6-aminohexanol according to a previously reported procedure in three steps.[14]

N-benzyl-N-benzyloxycarbonyl-6-aminohexyl 2,4,6-tri-O-benzoyl-β-D-galactopyranoside (S10)

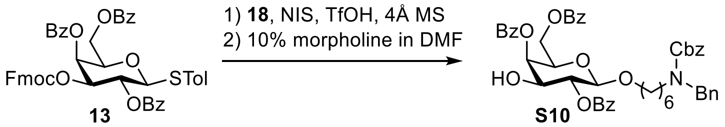

Glycosyl donor **13** (58 mg, 0.071 mmol) and acceptor **18** (72 mg, 0.21 mmol) were co-evaporated from anhydrous toluene and dried overnight under high vacuum. The donor, acceptor, and freshly MW-activated and crushed molecular sieves (4Å, ~0.5 g) were suspended in anhydrous DCM (2.2 ml) and stirred at RT for 30 min, then cooled to 0°C in an ice-water bath. Activator solution (0.82 ml, anhydrous DCM and anhydrous 1,4-dioxane (4:1)) containing recrystallized NIS (32 mg, 0.14 mmol) and TfOH (2.5 µl, 28 µmol) was added dropwise. The reaction mixture was stirred in a cold water bath at 5°C, and allowed to reach 16°C over 1 h. Another portion of the activator solution (0.41 ml, anhydrous DCM and anhydrous 1,4-dioxane (4:1)) containing recrystallized NIS (16 mg, 0.071 mmol) and TfOH (1.3 µl, 14 µmol) was added dropwise. The reaction mixture was stirred for 30 min, reaching 18°C. The reaction mixture was diluted with DCM (~20 ml) and filtered through celite, directly onto aqueous $Na_2S_2O_3$ (10% w/w, ~5 ml) and sat. $NaHCO_3$ (~5 ml). The celite was washed with DCM (~10 ml) and the combined filtrate was washed with an additional portion of aqueous $Na_2S_2O_3$ (10% w/w, ~5 ml) and sat. $NaHCO_3$ (~5 ml). The layers were separated and the aqueous phase was extracted with DCM (1x 5ml). The organic phase was washed with brine, dried over Na_2SO_4, then filtered and concentrated under reduced pressure. The crude product was used directly in the next step without purification. HRMS (ESI-QTOF) m/z: $[M+H]^+$ Calcd for $C_{63}H_{60}NO_{13}$ 1038.4059; found 1038.4035. The crude product was dissolved in DMF (3.6 ml) and cooled to 0°C in an ice-water bath. Morpholine (0.4 ml, 4.6 mmol, 10% in DMF) was added dropwise. The reaction mixture was stirred at 0°C for 20 min. The reaction mixture was diluted with DCM (~20 ml) and poured onto aqueous HCl (1 M, ~15 ml). The layers were separated and the aqueous phase was extracted with DCM (2x 5 ml). The combined organic phase was washed with brine. A small amount of sat. $NaHCO_3$ (~0.5 ml) was added to neutralize the slightly acidic aqueous layer. The organic phase was dried over Na_2SO_4, filtered and evaporated. Compound **S10** was purified by flash chromatography (50 mg, 87% 2-step yield, elution at 45% EtOAc in hexane). HRMS (ESI-QTOF) m/z: $[M+H]^+$ Calcd for $C_{48}H_{50}NO_{11}$ 816.3378; found 816.3341. ^{1}H NMR (700 MHz, $CDCl_3$): δ, ppm 8.16 (dd, J = 1.2, 8.3 Hz, 2H), 8.05 - 8.03 (m, 4H), 7.63 - 7.60 (m, 1H), 7.57 - 7.52 (m, 2H), 7.50 - 7.47 (m, 2H), 7.45 - 7.40 (m, 4H), 7.36 - 7.29 (m, 6H), 7.22 - 7.20 (m, 1H), 7.14 - 7.12 (m, 1H), 5.78 (dd, $J_{4,5}$ = 1.0 Hz, $J_{3,4}$ = 3.6 Hz, 1H, H-4), 5.35 (dd, $J_{1,2}$ = 7.9 Hz, $J_{2,3}$ = 10.0 Hz, 1H, H-2), 5.17 - 5.14 (2H, br, -C***H***$_2$-(Cbz)), 4.68 (1H, br, H-1(Galβ)), 4.61 (dd, $J_{5,6A}$ = 6.8 Hz, J_{AB} = 11.3 Hz, 1H, H-6A), 4.44 - 4.39 (3H, m, H-6B; -C***H***$_2$-(NBn)), 4.16 - 4.11 (m, 2H, H-3; H-5), 3.93 - 3.90 (br, 1H, -OC***H***$_2$(CH_2)$_5$N-), 3.53 - 3.47 (br, 1H, -OC***H***$_2$(CH_2)$_5$N-), 3.17 - 3.05 (br, 2Hr, -O(CH_2)$_5$C***H***$_2$N-), 2.66 - 2.66 (m, 1H, 3-O***H***), 1.56 - 1.50 (m, 2H, -(C***H***$_2$)$_4$-), 1.34 - 1.28 (m, 2H, -(C***H***$_2$)$_4$-), 1.22 - 1.06 (m, 4H, -(C***H***$_2$)$_4$-). ^{13}C{^{1}H} NMR (176 MHz, $CDCl_3$): δ, ppm 166.9 (**C**=O(2-O-Bz)), 166.4 (**C**=O(4-O-Bz)), 166.2 (**C**=O(6-O-Bz)), 156.8 (**C**=O(Cbz)), 156.2 (**C**=O(Cbz)), 138.0, 136.9, 133.7, 133.5, 133.4, 130.2, 129.9, 129.8, 129.6, 129.6, 129.1, 128.7, 128.6, 128.5, 128.0, 127.9, 127.2, 101.4 (C-1(Galβ)), 73.9 (C-2), 72.1 (C-5), 71.4 (C-3), 70.5 (C-4), 70.4 (-O**C**H$_2$(CH_2)$_5$N-), 67.2 (-**C**H$_2$-(Cbz)), 62.4 (C-6), 50.4 (-**C**H$_2$-(NBn)), 50.1 (-**C**H$_2$-(NBn)), 47.1 (-O(CH_2)$_5$**C**H$_2$N-), 46.1 (-O(CH_2)$_5$**C**H$_2$N-), 29.4 (-(**C**H$_2$)$_4$-), 27.9 (-(**C**H$_2$)$_4$-), 27.6 (-(**C**H$_2$)$_4$-), 26.5 (-(**C**H$_2$)$_4$-), 25.7 (-(**C**H$_2$)$_4$-). *Note: Cis-trans isomerism of the Cbz carbamate moiety results in split ^{13}C signal for N-**C**H$_2$-Ph; N-**C**H$_2$(CH$_5$)O-; **C**=O (Cbz);* (**C**H$_2$)$_4$. *Broadening of corresponding -C**H**$_2$- groups in ^{1}H-NMR.*

N-benzyl-N-benzyloxycarbonyl-6-aminohexyl β-D-galactopyranoside (S11)

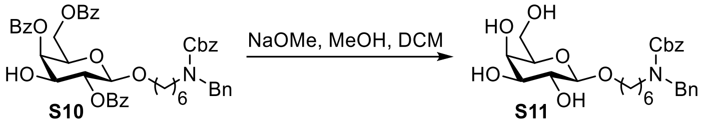

Compound **S10** (5.1 mg, 6.3 μmol) was dissolved in anhydrous DCM (0.4 ml) and cooled to 0°C in an ice-water bath. NaOMe in anhydrous MeOH solution (0.5 M, 0.5 ml) was added dropwise. The reaction mixture was stirred at RT for 1.5 h until completion (as indicated by TLC, 10% MeOH in DCM, R_f=0.26). Ion exchange beads Amberlight® XAD16N (H-form, pre-washed in DCM and MeOH) were added in small portions until a neutral pH was reached and reaction mixture turned clear. The reaction mixture was filtered, and the filtrate was concentrated under reduced pressure. Compound **S11** was purified by Prep-HPLC (2.7 mg, 86% yield, elution at 46% ACN). HRMS (ESI-QTOF) m/z: $[M+H]^+$ Calcd for $C_{27}H_{37}NO_8$ 504.2592; found 504.2588. ^{1}H NMR (700 MHz, d_4-MeOD): δ , ppm 7.40 - 7.18 (m, 10H), 5.18 - 5.14 (br, 2H, -C***H*₂**-(Cbz)), 4.51 (s, 2H, -C***H*₂**-(NBn)), 4.19 - 4.18 (br, 1H, H-1(Galβ)), 3.87 - 3.87 (br, 1H, -OC***H*₂**$(CH_2)_5$N-), 3.83 (dd, $J_{4,5}$ = 0.8 Hz, $J_{3,4}$ = 3.3 Hz, 1H, H-4), 3.74 (dd, J_{AB} = 11.1 Hz, $J_{5,6A}$ = 6.4 Hz, 1H, H-6A), 3.72 (dd, J_{AB} = 11.1 Hz, $J_{5,6B}$ = 5.5 Hz, 1H, H-6B), 3.52 - 3.52 (br, 1H, -OC***H*₂**$(CH_2)_5$N-), 3.51 - 3.47 (m, 2H, H-2; H-5), 3.45 (dd, $J_{3,4}$ = 3.3 Hz, $J_{2,3}$ = 9.7 Hz, 1H, H-3), 3.28 - 3.23 (be, 2H, -O$(CH_2)_5$C***H*₂**N-), 1.59 - 1.48 (m, 4H, -(C***H*₂**)$_4$-), 1.38 - 1.21 (m, 4H, -(C***H*₂**)$_4$-), H_2O (δ 4.85 (s)). ^{13}C{^{1}H} NMR (176 MHz, d_4-MeOD): δ , ppm 129.6, 129.5, 129.1, 129.0, 128.7, 128.4, 128.4, 105.0 (C-1(Galβ)), 76.6 (C-5), 75.1 (C-3), 72.6 (C-2), 70.6 (-O**C**H$_2$$(CH_2)_5$N-), 70.3 (C-4), 68.5 (-**C**H$_2$-(Cbz)), 68.4 (-**C**H$_2$-(Cbz)), 62.5 (C-6), 51.6 (-**C**H$_2$-(NBn)), 51.3 (-**C**H$_2$-(NBn)), 48.5 (-O$(CH_2)_5$**C**H$_2$N-), 47.7 (-O$(CH_2)_5$**C**H$_2$N-), 30.7 (-(**C**H$_2$)$_4$-), 29.2 (-(**C**H$_2$)$_4$-), 28.7 (-(**C**H$_2$)$_4$-), 27.6 (-(**C**H$_2$)$_4$-), 26.7 (-(**C**H$_2$)$_4$-). *Note: Cis-trans isomerism of the Cbz carbamate moiety results in split ^{13}C signal for N-**C**H$_2$-Ph; N-**C**H$_2$(CH$_5$)O-; (**C**H$_2$)$_4$. Broadening of corresponding -C**H₂**- groups in ^{1}H-NMR. The ^{13}C signal of **C**=O (Cbz) (δ 158.3) did not appear in ^{13}C-NMR and was confirmed with additional information from ^{1}H-^{13}C HMBC.*

6-Aminohexyl β-D-galactopyranoside (Gal)

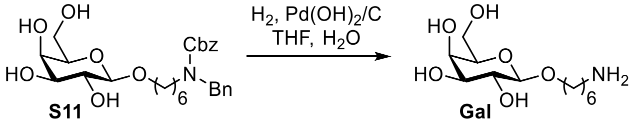

Compound **S11** (3.2 mg, 6.4 μmol) and $Pd(OH)_2$/C (10-20% wt. loading, 3.4 mg) were suspended in THF and TDW (1:1 1 ml). The mixture was stirred in a pressurized reaction vessel under 4 bar of H_2 at RT for 6 h. The reaction mixture was filtered through a Whatman 42 filter paper, then the filter paper was washed with more TDW, and the filtrate concentrated under reduced pressure. The crude product was dissolved in a minimal amount of TDW (~50 μl) and passed through an Orbilica pre-packed solid phase extraction (SPE) cartridge (200 mg/3 ml, C18). The cartridge was conditioned in 85% ACN in TDW, then equilibrated in TDW. The crude product was loaded onto the SPE cartridge. Compound **Gal** was eluted with TDW (~1.5 ml) and lyophilized (1.8 mg, 100% yield). HRMS (ESI-QTOF) m/z: $[M+H]^+$ Calcd for $C_{12}H_{37}NO_6$ 280.1755; found 280.1752. ^{1}H NMR (500 MHz, D_2O): δ , ppm 4.40 (d, $J_{1,2}$ = 8.0 Hz, 1H, H-1(Galβ)), 3.97 - 3.91 (m, 1H, -OC***H*₂**$(CH_2)_5NH_2$), 3.93 (d, $J_{3,4}$ = 2.9 Hz, 1H, H-4), 3.79 (dd, J_{AB} = 11.7 Hz, $J_{5,6A}$ = 7.7 Hz, 1H, H-6A), 3.75 (dd, J_{AB} = 11.7 Hz, $J_{5,6B}$ = 4.7 Hz, 1H, H-6B), 3.71 - 3.66 (m, 1H, H-5; -OC***H*₂**$(CH_2)_5NH_2$), 3.64 (dd, $J_{3,4}$ = 3.4 Hz, $J_{2,3}$ = 9.9 Hz, 1H, H-3), 3.50 (dd, $J_{1,2}$ = 8.0 Hz, $J_{2,3}$ = 9.7 Hz, 1H, H-2), 2.98 (t, J = 7.5 Hz, 2H, -O$(CH_2)_5$C***H*₂**NH_2), 1.69 - 1.63 (m, 4H, -OCH$_2$C***H*₂**$(CH_2)_2$C***H*₂**CH$_2$NH$_2$), 1.43 - 1.40 (m, 4H, -O$(CH_2)_2$ (C***H*₂**)$_2$$(CH_2)_2NH_2$), formate (δ 8.46 (s)). ^{13}C{^{1}H} NMR (126 MHz, D_2O): δ , ppm 98.0 (C-1(Galβ)), 70.4 (C-5), 68.1 (C-3), 66.0 (C-2), 65.6 (-O**C**H$_2$$(CH_2)_5NH_2$), 63.9 (C-4), 56.2 (C-6), 34.7 (-O$(CH_2)_5$**C**H$_2$NH$_2$), 23.7 (-(**C**H$_2$)$_4$-), 22.1 (-(**C**H$_2$)$_4$-), 20.5 (-(**C**H$_2$)$_4$-), 19.8 (-(**C**H$_2$)$_4$-), formate (δ 166.3).

N-benzyl-N-benzyloxycarbonyl-6-aminohexyl 4-O-benzoyl-2,3-di-O-benzyl-α-D-galactopyranosyl-(1→3)-2,4,6-tri-O-benzoyl-β-D-galactopyranoside (19)

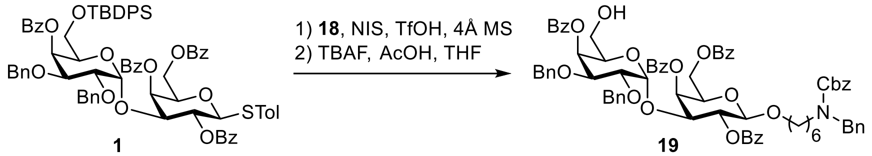

Glycosyl donor **1** (111 mg, 86.5 μmol) and acceptor **18** (88.2 mg, 258 μmol) were co-evaporated from anhydrous toluene and dried overnight under high vacuum. The donor, acceptor, and freshly MW-activated and crushed molecular sieves (4Å, ~1.5 g) were suspended in anhydrous DCM (2.6 ml) and stirred at RT for 1 h, then cooled to -5°C in an ice-salt-water bath. Activator solution (1.73 ml, anhydrous DCM and anhydrous 1,4-dioxane (4:1)) containing recrystallized NIS (58 mg, 0.26 mmol) and TfOH (4.6 μl, 0.052 mmol) was added dropwise. The reaction mixture was stirred at -5°C for 5 min, after which the reaction mixture was stirred at 5°C for an additional 1 h. Another portion of the activator solution (0.6 ml, anhydrous DCM and anhydrous 1,4-dioxane (4:1)) containing recrystallized NIS (20 mg, 0.090 mmol) and TfOH (1.6 μl, 0.018 mmol) was added and the reaction mixture was allowed to reach 14°C over 50 min. The reaction mixture was filtered through celite, directly onto aqueous $Na_2S_2O_3$ (10% w/w, ~2 ml) and sat. $NaHCO_3$ (~2 ml). The celite was washed with DCM (~50 ml) and the combined filtrate was washed with an additional portion of aqueous $Na_2S_2O_3$ (10% w/w, ~5 ml) and sat. $NaHCO_3$ (~5 ml). The layers were separated and the aqueous layer was extracted with DCM (2x 5 ml). The combined organic phase was washed with brine, dried over Na_2SO_4, then filtered and concentrated under reduced pressure. The crude product was subjected to flash chromatography (elution at 30% EtOAc in hexane) and used directly in the next step. HRMS (ESI-QTOF) m/z: $[M+H]^+$ Calcd for $C_{91}H_{94}NO_{17}Si$ 1500.6286; found 1500.6269. The disaccharide was dissolved in anhydrous THF (4 ml) and cooled to 0°C in an ice-water bath. Acetic acid (AcOH) (0.03 ml, 0.6 mmol) was added, followed by the addition of tetrabutylammonium fluoride (TBAF) (1 M in THF, 0.46 ml, 0.46 mmol). The reaction mixture was stirred at RT overnight until completion (as indicated by TLC, 40% EtOAc in hexane, R_f=0.26). The reaction mixture was diluted with DCM (~30 ml) and washed with sat. $NaHCO_3$. The layers were separated and the aqueous phase was extracted with DCM (2x 5 ml). The combined organic layer was washed with brine, dried over Na_2SO_4, then filtered and concentrated under reduced pressure to produce a yellow syrup. Compound **19** was purified by flash chromatography (105 mg, 97% 2-step yield, elution at 45% EtOAc in hexane). HRMS (ESI-QTOF) m/z: $[M+H]^+$ Calcd for $C_{75}H_{76}NO_{17}$ 1262.5108; found 1262.5111. ^{1}H NMR (500 MHz, $CDCl_3$): δ, ppm 8.16 - 8.13 (m, 2H), 8.09 - 8.05 (m, 4H), 7.84 - 7.81 (m, 2H), 7.60 - 7.52 (m, 4H), 7.47 - 7.40 (m, 6H), 7.40 - 7.27 (m, 8H), 7.16 - 7.11 (m, 9H), 7.04 - 7.01 (m, 2H), 5.95 (d, $J_{3,4}$ = 2.6 Hz, 1H, H-4), 5.71 (dd, $J_{1,2}$ = 8.0 Hz, $J_{2,3}$ = 9.9 Hz, 1H, H-2), 5.22 (d, $J_{1,2}$ = 3.2 Hz, 1H, H-1'(Galα))), 5.16 (s, 2H, -C***H***$_2$-(Cbz)), 5.05 (d, $J_{3,4}$ = 2.1 Hz, 1H, H-4'), 4.67 (br, 1H, H-1(Galβ))), 4.61 (dd, J_{AB} = 11.3 Hz, $J_{5,6A}$ = 6.8 Hz, 1H, H-6A), 4.45 (ABq, $\Delta\upsilon_{AB}$ = 32.9 Hz, J_{AB} = 12.2 Hz, 2H, O-C***H***$_2$-Ph), 4.47 - 4.42 (m, 3H, H-6B; -C***H***$_2$-(NBn)), 4.29 (ABq, $\Delta\upsilon_{AB}$ = 29.6 Hz, J = 11.3 Hz, 2H, O-C***H***$_2$-Ph), 4.19 (d, $J_{2,3}$ = 9.4 Hz, 1H, H-3), 4.14 (t, $J_{5,6}$ = 6.4 Hz, 1H, H-5), 3.95 (t, $J_{5,6}$ = 6.3 Hz, 1H, H-5'), 3.91 (br, 1H, -OC***H***$_2$(CH$_2$)$_5$N-), 3.80 (dd, $J_{1,2}$ = 3.3 Hz, $J_{2,3}$ = 10.0 Hz, 1H, H-2'), 3.71 (dd, $J_{3,4}$ = 3.1 Hz, $J_{2,3}$ = 10.0 Hz, 1H, H-3'), 3.51 (br, 1H, -OC***H***$_2$(CH$_2$)$_5$N-), 3.45 (dd, J_{AB} = 11.7 Hz, $J_{5,6A}$ = 6.8 Hz, 1H, H-6A), 3.28 (dd, J_{AB} = 11.5 Hz, $J_{5,6B}$ = 6.3 Hz, 1H, H-6B), 3.08 (br, 2H, -O(CH$_2$)$_5$C***H***$_2$N-), 1.49 - 1.45 (m, 2H, -(**C**H$_2$)$_4$-), 1.37 - 1.22 (m, 2H, -(**C**H$_2$)$_4$-), 1.20 - 1.00 (m, 4H, -(**C**H$_2$)$_4$-). ^{13}C{^{1}H} NMR (126 MHz, $CDCl_3$): δ, ppm 166.8 (**C**=O(4'-O-Bz)), 166.3 (**C**=O(6-O-Bz)), 166.0 (**C**=O(4-O-Bz)), 165.1 (**C**=O(2-O-Bz)), 156.8 (**C**=O(Cbz)), 156.2 (**C**=O(Cbz)), 138.3, 138.0, 137.0, 133.6, 133.4, 133.4, 133.3, 130.3, 130.0, 129.9, 129.8, 129.7, 129.6, 129.4, 129.3, 128.7, 128.6, 128.5, 128.4, 128.1, 128.1, 128.0, 127.9, 127.6, 127.4, 127.3, 127.2, 101.8 (C-1(Galα)), 95.1 (C-1'(Galβ)), 75.9 (C-3'), 73.7 (C-3; C-2'), 72.5 (O-**C**H$_2$-Ph), 71.9 (O-**C**H$_2$-Ph), 71.6 (C-5'), 71.2 (C-2), 70.2 (-O**C**H$_2$(CH$_2$)$_5$N-), 69.9 (C-5), 69.2 (C-4'), 67.2 (-**C**H$_2$-(Cbz)), 66.4 (C-4), 63.4, 62.6 (C-6'), 61.1 (C-6), 50.4 (-**C**H$_2$-(NBn)), 50.1 (-**C**H$_2$-(NBn)), 47.1 (-O(CH$_2$)$_5$**C**H$_2$N-), 46.1 (-O(CH$_2$)$_5$**C**H$_2$N-), 29.8, 29.4,

27.9, 27.5, 26.4, 25.6, 24.6, 19.7, 14.2, 13.8. Note: Cis-trans isomerism of the Cbz carbamate moiety results in split ^{13}*C signal for N-**C**H_2-Ph; N-**C**H_2(CH_5)O-; **C**=O (Cbz); (**C**H_2)$_4$. Broadening of corresponding -C**H_2**- groups in* ^{1}*H-NMR. Note 2: Aliphatic impurity attributed to tetrabutylammonium salts is present; This impurity did not appear to affect subsequent steps.*

6-Aminohexyl α-D-galactopyranosyl-(1→3)-β-D-galactopyranoside (nSd)

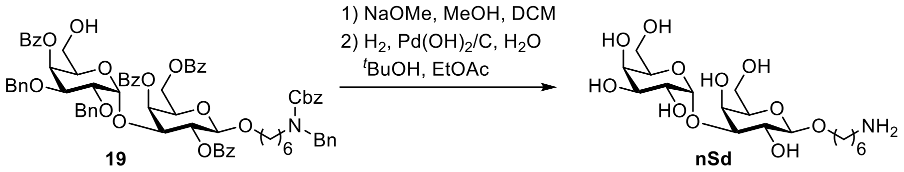

Compound **19** (20.7 mg, 16.4 μmol) was dissolved in anhydrous DCM (1 ml) and cooled to 0°C in an ice-water bath. NaOMe in anhydrous MeOH solution (0.5 M, 1 ml) was added dropwise. The reaction mixture was stirred at RT for 7 h until completion (as indicated by TLC, 5% MeOH in DCM, R_f=0.22). Ion exchange beads Amberlight® XAD16N (H-form, pre-washed in DCM and MeOH) were added in small portions until a neutral pH was reached, and reaction mixture turned clear. The reaction mixture was filtered and the filtrate was concentrated under reduced pressure, then dried under high vacuum. The crude product and $Pd(OH)_2/C$ (10-20% wt. loading, 15 mg) were suspended in EtOAc, ^{t}BuOH, and TDW (0.5:1:0.5, 2 ml). The mixture was stirred in a pressurized reaction vessel under 4 bar of H_2 at RT for 24 h. The reaction mixture was filtered through celite, and the celite was washed with MeOH and MeOH/TDW (1:1). The combined filtrate was concentrated under reduced pressure to produce a colorless glassy residue. The crude product was dissolved in a minimal amount of ACN and TDW (35% ACN, ~50 μl) and passed through an a Orbilica pre-packed SPE cartridge (200 mg/3 ml, C18). The cartridge was conditioned in 85% ACN in TDW, then equilibrated in 5% ACN in TDW. The crude product was loaded onto the SPE cartridge and eluted with 35% ACN in TDW (~2 ml). The collected fraction containing **nSd** was lyophilized. The lyophilized fraction was dissolved in an ammonium formate buffer (pH = 4.5, 1.75 M, 100 μl) and incubated at RT for 2 h. Compound **nSd** was purified by size exclusion chromatography (SEC) on Sephadex LH-20 size exclusion resin (160 mg, 140-550 mesh, swelled in TDW for 2.5 h). Disaccharide **nSd** was eluted with TDW within the first 1.5 column void volume (5.9 mg, 81% 2-step yield). HRMS (ESI-QTOF) m/z: $[M+H]^+$ Calcd for $C_{18}H_{36}NO_{11}$ 442.2283; found 442.2270. ^{1}H NMR (500 MHz, D_2O): δ, ppm 5.16 (d, $J_{1,2}$ = 3.9 Hz, 1H, H-1(Galα)), 4.46 (d, $J_{1,2}$ = 8.0 Hz, 1H, H-1(Galβ)), 4.21 - 4.17 (m, 2H, H-4; H-5'), 4.02 (dd, $J_{4,5}$ = 1.0 Hz, $J_{3,4}$ = 3.3 Hz, 1H, H-4'), 3.98 - 3.92 (m, 2H, H-3'; -OC**H_2**(CH_2)$_5$$NH_2$), 3.87 (dd, $J_{1,2}$ = 3.9 Hz, $J_{2,3}$ = 10.3 Hz, 1H, H-2'), 3.81 (dd, J_{AB} = 11.7 Hz, $J_{5,6A}$ = 7.7 Hz, 1H, H-6A), 3.78 - 3.72 (m, 4H, H-6B; H-6'; H-3), 3.72 - 3.66 (m, 2H, H-5, -OC**H_2**(CH_2)$_5$$NH_2$), 3.64 (dd, $J_{1,2}$ = 8.0 Hz, $J_{2,3}$ = 9.8 Hz, 1H, H-2), 3.00 (t, J = 7.6 Hz, 2H, -O(CH_2)$_5$C**H_2**NH_2), 1.70 - 1.63 (m, 4H, -OCH_2C**H_2**(CH_2)$_2$C**H_2**CH_2NH_2), 1.44 - 1.39 (m, 4H, -O(CH_2)$_2$ (C**H_2**)$_2$(CH_2)$_2$$NH_2$), formate (δ 8.45 (s)). ^{13}C{^{1}H} NMR (126 MHz, D_2O): δ, ppm 97.8 (C-1(Galβ)), 90.5 (C-1'(Galα)), 72.6 (C-3), 70.1 (C-5), 66.1 (C-5'), 65.6 (-O**C**H_2(CH_2)$_5$$NH_2$), 64.5 (C-3'), 64.5 (C-2), 64.4 (C-4'), 63.5 (C-2'), 60.0 (C-4), 56.2 (C-6'), 56.2 (C-6), 34.7 (-O(CH_2)$_5$**C**$H_2$$NH_2$), 23.7 (-(**C**$H_2$)$_4$-), 21.8 (-(**C**$H_2$)$_4$-), 20.5 (-(**C**$H_2$)$_4$-), 19.8 (-(**C**$H_2$)$_4$-), formate (δ 166.3). *Note: The ammonium formate buffer is used in excess (~10 equiv.) to produce a uniform aminium formate salt form and remove anionic contaminants (e.g. benzoate).*

N-benzyl-N-benzyloxycarbonyl-6-aminohexyl 4-O-benzoyl-2,3-di-O-benzyl-6-O-sulfo-α-D-galactopyranosyl-(1→3)-2,4,6-tri-O-benzoyl-β-D-galactopyranoside (20)

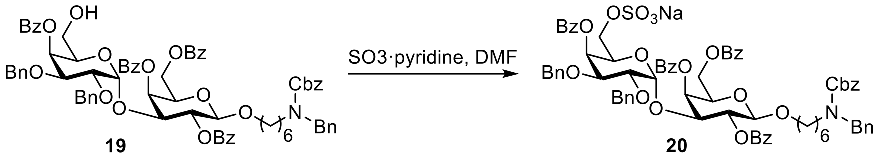

Compound **19** (14.5 mg, 11.5 μmol) and SO_3·pyridine (17.6 mg, 111 μmol) were dried under high vacuum overnight. The mixture was dissolved in anhydrous DMF (0.9 ml). The reaction mixture was stirred at RT for 20 h until completion (as indicated by TLC, 10% MeOH in DCM, R_f=0.29). The reaction was quenched by the addition of MeOH (~3 ml) and concentrated under reduced pressure. Disaccharide **20** was purified by flash chromatography (elution at 10% MeOH in DCM) and Dowex 50W X8 ion exchange resin (Na-form) was added directly to the eluted fraction. The fraction was filtered and the filtrate evaporated under reduced pressure to obtain **20** (14.1 mg, 90% yield). HRMS (ESI-QTOF) m/z: $[M-Na+2H]^+$ Calcd for $C_{75}H_{76}NO_{20}S$ 1342.4676; found 1342.4692. ^1H NMR (500 MHz, d_7-DMF): δ , ppm 8.38 (dd, J = 1.9, 7.8 Hz, 2H), 8.13 (dd, J = 1.3, 8.4 Hz, 2H), 8.09 (dd, J = 1.3, 8.4 Hz, 2H), 7.94 (dd, J = 1.2, 8.3 Hz, 2H), 7.73 - 7.63 (m, 6H), 7.61 - 7.48 (m, 6H), 7.43 - 7.25 (m, 10H), 7.18 - 7.16 (m, 4H), 7.11 - 7.08 (m, 4H), 7.05 - 7.03 (m, 2H), 6.18 (d, $J_{3,4}$ = 3.0 Hz, 1H, H-4), 5.66 (dd, $J_{1,2}$ = 8.0 Hz, $J_{2,3}$ = 10.4 Hz, 1H, H-2), 5.53 (d, $J_{1,2}$ = 3.3 Hz, 1H, H-1(Galα)), 5.20 - 5.16 (m, 3H, H-4'; -C***H***$_2$-(Cbz)), 5.12 (d, $J_{1,2}$ = 8.0 Hz, 1H, H-1(Galβ)), 4.83 (dd, $J_{3,4}$ = 3.1 Hz, $J_{2,3}$ = 10.4 Hz, 1H, H-3), 4.63 - 4.57 (m, 2H, H-6A; H-5), 4.55 (t, $J_{5,6}$ = 6.5 Hz, 1H, H-5'), 4.53 - 4.48 (m, 3H, H-6B; -C***H***$_2$-(NBn)), 4.48, 4.40 (ABq, $\Delta\delta_{AB}$ = 0.08, J_{AB} = 11.9 Hz, 1H, 2-O-C***H***$_2$-Ph), 4.22 (d, J_{AB} = 11.0 Hz, 1H, 3-O-C***H***$_A$H$_B$-Ph), 4.00 - 3.92 (m, 3H, H-6'; 3-O-CH$_A$***H***$_B$-Ph), 3.89 (dd, $J_{1,2}$ = 3.3 Hz, $J_{2,3}$ = 10.1 Hz, 1H, H-2'), 3.86 - 3.85 (br, 1H, -OC***H***$_2$(CH$_2$)$_5$N-), 3.59 - 3.58 (br, 1H, -OC***H***$_2$(CH$_2$)$_5$N-), 3.54 (dd, $J_{3,4}$ = 3.2 Hz, $J_{2,3}$ = 10.1 Hz, 1H, H-3'), 3.13 - 3.11 (br, 2H, -O(CH$_2$)$_5$C***H***$_2$N-), 1.47 - 1.46 (m, 2H, -(C***H***$_2$)$_4$-), 1.32 - 1.32 (m, 2H, -(C***H***$_2$)$_4$-), 1.19 - 1.06 (m, 4H, -(C***H***$_2$)$_4$-), H_2O (δ 3.49). $^{13}C\{^1H\}$ NMR (126 MHz, d_7-DMF): δ , ppm 166.8 (**C**=O(4-O-Bz)), 166.8 (**C**=O(6-O-Bz)), 166.3 (**C**=O(4'-O-Bz)), 166.2 (**C**=O(2-O-Bz)), 157.4 (**C**=O(Cbz)), 156.8 (**C**=O(Cbz)), 139.9, 139.7, 139.4, 138.6, 134.8, 134.6, 134.5, 134.4, 131.5, 131.0, 130.8, 130.7, 130.7, 130.6, 130.3, 130.0, 129.8, 129.6, 129.1, 129.0, 128.9, 128.7, 128.7, 128.4, 128.2, 102.4 (C-1(Galβ)), 93.6 (C-1'(Galα)), 77.3 (C-3'), 76.1 (C-2'), 73.2 (2-O-**C**H$_2$-Ph), 72.4 (3-O-**C**H$_2$-Ph), 72.4 (C-5), 71.9 (C-3), 71.9 (C-2), 70.6 (-O**C**H$_2$(CH$_2$)$_5$N-), 69.7 (C-4'), 69.1 (C-5'), 67.7 (C-4), 67.6 (-**C**H$_2$-(Cbz)), 66.0 (C-6'), 63.7 (C-6), 51.2 (-**C**H$_2$-(NBn)), 50.9 (-**C**H$_2$-(NBn)), 48.2 (-O(CH$_2$)$_5$**C**H$_2$N-), 47.4 (-O(CH$_2$)$_5$**C**H$_2$N-), 30.6 (), 29.1 (-(**C**H$_2$)$_4$-), 28.6 (-(**C**H$_2$)$_4$-), 27.3 (-(**C**H$_2$)$_4$-), 26.6 (-(**C**H$_2$)$_4$-), residual EtOH (δ 55.9, 14.7). *Note: Cis-trans isomerism of the Cbz carbamate moiety results in split* ^{13}C *signals for N-**C**H$_2$-Ph; N-**C**H$_2$(CH$_5$)O-; **C**=O (Cbz);* (**C**H$_2$)$_4$. *Broadening of corresponding -C**H**$_2$- groups in* ^1H*-NMR.*

6-Aminohexyl 6-O-sulfo-α-D-galactopyranosyl-(1→3)-β-D-galactopyranoside (mSd)

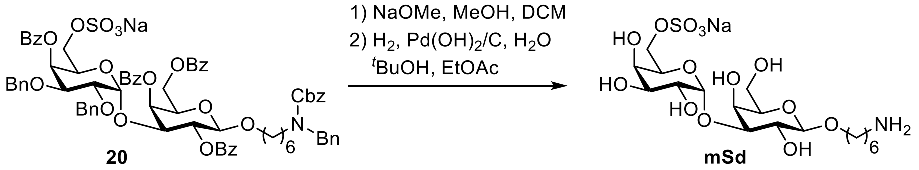

Compound **20** (14.1 mg, 10.3 μmol) was dissolved in anhydrous DCM (0.5 ml) and cooled to 0°C in an ice-water bath. NaOMe in anhydrous MeOH solution (0.5 M, 1 ml) was added dropwise. The reaction mixture was stirred at RT for 4 h until completion (as indicated by RP-TLC, 50% ACN in TDW, R_f=0.66). Formation of some desulfation byproduct was observed (as indicated by TLC, 5% MeOH in DCM, R_f=0.22; *synthesis of **nSd** used for reference*). The reaction mixture was diluted with MeOH (~5 ml) and ion exchange beads Amberlight® XAD16N (H-form, pre-washed in DCM and MeOH)

were added in small portions until neutral pH was reached and reaction mixture turned clear. The reaction mixture was filtered and Dowex 50W X8 ion exchange resin (Na-form) was added to the filtrate. The mixture was filtered again, and the filtrate was concentrated under reduced pressure, then dried under high vacuum. The crude product was dissolved in a minimal amount of ACN and TDW (50% ACN, ~0.1 ml) and passed through Orbilica pre-packed SPE cartridges (200 mg/3 ml, C18). The cartridge was conditioned in 85% ACN in TDW, then equilibrated in 5% ACN in TDW. The crude product was distributed equally between three SPE cartridges and washed with 10% ACN, then dried with Ar. The cartridges were extracted with 35% ACN (~2 ml) and the combined eluted fraction was lyophilized and used directly in the next step. LC-MS (ESI-QqQ-CEM) m/z: $[M-Na]^-$ Calcd for $C_{47}H_{58}NO_{16}S$ 924.3; found 924.5. Fractions eluted with 50% ACN contained desulfated byproduct as indicated by MS (MALDI-TOF) m/z: $[M+Na]^+$ Calcd for $C_{47}H_{59}NO_{13}Na$ 868.388; found 868.102. The lyophilized fraction (eluted with 35% ACN) and $Pd(OH)_2/C$ (10-20% wt. loading, 15 mg) were suspended in EtOAc, ^{t}BuOH and TDW (0.5:1:0.5, 2 ml). The mixture was stirred in a pressurized reaction vessel under 4 bar of H_2 at RT for 15 h. The reaction mixture was filtered through celite, and the celite was washed with MeOH and MeOH/TDW (1:1). The combined filtrate was concentrated under reduced pressure to produce a colorless glassy residue. The crude product was dissolved in a minimal amount of ACN and TDW (5% ACN, ~50 µl) and purified by SPE on a Orbilica pre-packed cartridge (200 mg/3 ml, C18). The SPE cartridge was conditioned in 85% ACN, then equilibrated in TDW. The crude product was loaded onto the SPE cartridge and disaccharide **mSd** was eluted with TDW and lyophilized (3.1 mg, 57% 2-step yield). HRMS (ESI-QTOF) m/z: $[M-Na+2H]^+$ Calcd for $C_{18}H_{36}NO_{14}S$ 522.1851; found 522.1857. ^{1}H NMR (700 MHz, D_2O): δ, ppm 5.17 (d, $J_{1,2}$ = 3.8 Hz, 1H, H-1(Galα)), 4.50 (d, $J_{1,2}$ = 8.0 Hz, 1H, H-1(Galβ)), 4.44 (dd, $J_{5,6A}$ = 5.4 Hz, $J_{5,6B}$ = 7.0 Hz, 1H, H-5'), 4.23 - 4.16 (m, 3H, H-6'; H-4), 4.08 (d, $J_{3,4}$ = 2.9 Hz, 1H, H-4'), 4.00 (dd, $J_{3,4}$ = 3.3 Hz, $J_{2,3}$ = 10.4 Hz, 1H, H-3'), 3.97 (td, J_{AB} = 10.7 Hz, J = 6.8 Hz, 1H, $-OC\boldsymbol{H_2}(CH_2)_5NH_2$), 3.89 (dd, $J_{1,2}$ = 3.9 Hz, $J_{2,3}$ = 10.4 Hz, 1H, H-2'), 3.84 - 3.77 (m, 3H, H-6; H-3), 3.75 - 3.68 (m, 2H, H-5; $-OC\boldsymbol{H_2}(CH_2)_5NH_2$), 3.63 (dd, $J_{1,2}$ = 8.1 Hz, $J_{2,3}$ = 9.7 Hz, 1H, H-2), 3.02 (t, J = 7.6 Hz, 2H, $-OC\boldsymbol{H_2}(CH_2)_5NH_2$), 1.72 - 1.66 (m, 4H, $-OCH_2C\boldsymbol{H_2}(CH_2)_2C\boldsymbol{H_2}CH_2NH_2$), 1.47 - 1.41 (m, 4H, $-O(CH_2)_2(C\boldsymbol{H_2})_2(CH_2)_2NH_2$), formate (δ 8.48 (s)), acetate (~1:0.1 ratio; δ 1.93 (s)). ^{13}C{^{1}H} NMR (176 MHz, D_2O): δ, ppm 97.8 (C-1(Galβ)), 90.8 (C-1'(Galα)), 72.9 (C-3), 70.1 (C-5), 65.5 ($-O\boldsymbol{C}H_2(CH_2)_5NH_2$), 64.6 (C-2), 64.3 (C-3'), 64.2 (C-4'), 64.1 (C-5'), 63.4 (C-2'), 62.6 (C-6'), 60.2 (C-4), 56.2 (C-6), 34.7 (($-O(CH_2)_5\boldsymbol{C}H_2NH_2$), 23.6 ($-(\boldsymbol{C}H_2)_4-$), 21.8 ($-(\boldsymbol{C}H_2)_4-$), 20.5 ($-(\boldsymbol{C}H_2)_4-$), 19.8 ($-(\boldsymbol{C}H_2)_4-$), formate (δ 166.3).

N-benzyl-N-benzyloxycarbonyl-6-aminohexyl 2,3-di-O-benzyl-6-O-benzoyl-α-D-galactopyranosyl-(1→3)-2,4,6-tri-O-benzoyl-β-D-galactopyranoside (24)

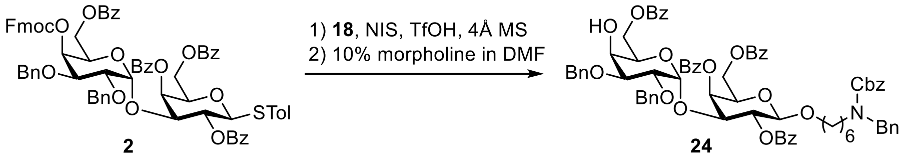

Glycosyl donor **2** (157 mg, 0.124 mmol) and acceptor **18** (126 mg, 0.369 mmol) were co-evaporated from anhydrous toluene and dried overnight under high vacuum. The donor, acceptor, and freshly MW-activated and crushed molecular sieves (4Å, ~1.8 g) were suspended in anhydrous DCM (5 ml) and stirred at RT for 1 h, then cooled to 0°C in an ice-water bath. Activator solution (1.6 ml, anhydrous DCM and anhydrous 1,4-dioxane (4:1)) containing recrystallized NIS (53 mg, 0.24 mmol) and TfOH (4.3 µl, 0.049 mmol) was added dropwise. The reaction mixture was stirred at 0°C for 10 min, after which the reaction mixture was allowed to reach 12°C over 2 h. Another portion of the activator solution (0.7 ml, anhydrous DCM and anhydrous 1,4-dioxane (2:1)) containing recrystallized NIS (23 mg, 0.11 mmol) and TfOH (1.9 µl, 21 µmol) was added and the reaction mixture was allowed to reach 18°C over an additional 20 min. The reaction mixture was diluted with DCM (~30 ml) and filtered through celite, directly onto aqueous $Na_2S_2O_3$ (10% w/w, ~10 ml) and sat. $NaHCO_3$ (~10 ml). The celite was washed with DCM (~20 ml) and the combined filtrate was washed with an additional portion of aqueous

$Na_2S_2O_3$ (10% w/w, ~5 ml) and sat. $NaHCO_3$ (~5 ml). The layers were separated and the organic phase was washed with brine, dried over Na_2SO_4, then filtered and concentrated under reduced pressure. The crude product was used directly in the next step without purification. The crude product was dissolved in DMF (13.5 ml) and cooled to 0°C in an ice-water bath. Morpholine (1.5 ml, 17.4 mmol, 10% in DMF) was added dropwise. The reaction mixture was stirred at 0°C for 20 min. The reaction mixture was diluted with DCM (~20 ml) and washed with aqueous HCl (0.1 M, ~20 ml). Small portions of aqueous HCl (1 M) were added until the aqueous phase registered pH~2. The layers were separated and the aqueous phase was extracted with DCM (10 ml). The combined organic phase was washed with brine, dried over Na_2SO_4, filtered and evaporated. Compound **24** was purified by flash chromatography (154 mg, 98% 2-step yield, elution at 40% EtOAc in hexane). HRMS (ESI-QTOF) m/z: $[M+H]^+$ Calcd for $C_{75}H_{76}NO_{17}$ 1264.5108; found 1262.5091. ^{1}H NMR (500 MHz, $CDCl_3$): δ, ppm 8.10 - 8.05 (m, 6H), 7.98 (d, J = 7.6 Hz, 2H), 7.61 - 7.51 (m, 3H), 7.49 - 7.41 (m, 5H), 7.37 (t, J = 7.8 Hz, 2H), 7.34 - 7.29 (m, 9H), 7.25 - 7.21 (m, 5H), 7.14 - 7.05 (m, 8H), 5.90 (d, $J_{3,4}$ = 2.9 Hz, 1H, H-4), 5.65 (dd, $J_{1,2}$ = 8.0 Hz, $J_{2,3}$ = 10.3 Hz, 1H, H-2), 5.34 (d, $J_{1,2}$ = 3.4 Hz, 1H, H-1'(Galα)), 5.15 (s, 1H, -C***H*₂**-(Cbz)), 4.53 (dd, J_{AB} = 11.3 Hz, $J_{5,6}$ = 6.6 Hz, 1H, H-6A), 4.49, 4.35 (ABq, $\Delta\delta_{AB}$ = 0.14, J_{AB} = 12.0 Hz, 2H, O-C$\boldsymbol{H_2}$-Ph), 4.43, 4.21 (ABq, ΔδAB = 0.22, J_{AB} = 11.3 Hz, 2H, O-C$\boldsymbol{H_2}$-Ph), 4.40 (br, 2H, -C$\boldsymbol{H_2}$-(NBn)), 4.47 - 4.31 (m, 3H, H-6B; H-6'), 4.27 (d, $J_{1,2}$ = 8.0 Hz, 1H, H-1(Galβ)), 4.13 (dd, $J_{3,4}$ = 3.2, $J_{2,3}$ = 10.4 Hz, 1H, H-3), 3.97 (dd, $J_{5,6A}$ = 4.9 Hz, $J_{5,6B}$ = 6.8 Hz, 1H, H-5'), 3.82 (t, $J_{5,6}$ = 6.4 Hz, 1H, H-5), 3.80 - 3.80 (br, 1H, -OC$\boldsymbol{H_2}$$(CH_2)_5$N-), 3.71 (dd, $J_{1,2}$ = 3.4 Hz, $J_{2,3}$ = 9.8 Hz, 1H, H-2'), 3.44 (dd, $J_{3,4}$ = 3.3 Hz, $J_{2,3}$ = 9.7 Hz, 1H, H-3'), 3.36 (d, $J_{3,4}$ = 2.1 Hz, 1H, H-4'), 3.34 - 3.34 (br, 1H, -OC$\boldsymbol{H_2}$$(CH_2)_5$N-), 3.11 - 3.03 (br, 2H, -O$(CH_2)_5$C$\boldsymbol{H_2}$N-), 1.42 - 1.42 (br, 2H, -(C$\boldsymbol{H_2}$)$_4$-), 1.29 - 1.26 (br, 2H, -(C$\boldsymbol{H_2}$)$_4$-), 1.14 - 1.02 (br, 4H, -(C$\boldsymbol{H_2}$)$_4$-), residual acetone (~1:0.06 ratio, δ 2.17). ^{13}C{^{1}H} NMR (126 MHz, $CDCl_3$): δ, ppm 166.1 (**C**=O (6/6'-O-Bz)), 166.0 (**C**=O (6/6'-O-Bz)), 166.0 (**C**=O (4-O-Bz)), 164.8 (**C**=O (2-O-Bz)), 138.3, 138, 136.9, 133.4, 133.4, 130.2, 130.1, 129.9, 129.8, 129.7, 129.6, 129.6, 129.1, 128.7, 128.6, 128.6, 128.6, 128.4, 128.1, 128.0, 127.9, 127.8, 127.8, 127.6, 127.2, 101.8 (C-1(Galβ)), 93.1 (C-1'(Galα)), 77.0 (C-3'), 74.4 (C-2'), 73.0 (O-**C**H$_2$-Ph), 72.3 (O-**C**H$_2$-Ph), 72.1 (C-3), 71.6 (C-5), 70.6 (C-2), 70.3 (-O**C**H$_2$$(CH_2)_5$N-), 68.1 (C-5'), 67.5 (C-4'), 67.2 (-**C**H$_2$-(Cbz)), 65.7 (C-4), 63.7 (C-6'), 62.4 (C-6), 50.4 (-**C**H$_2$-(NBn)), 50.1 (-**C**H$_2$-(NBn)), 47.1 (-O$(CH_2)_5$**C**H$_2$N-), 46.1 (-O$(CH_2)_5$**C**H$_2$N-), 29.4 (-(**C**H$_2$)$_4$-), 27.9 (-(**C**H$_2$)$_4$-), 27.5 (-(**C**H$_2$)$_4$-), 26.4 (-(**C**H$_2$)$_4$-), 25.6 (-(**C**H$_2$)$_4$-), residual acetone (δ 31.0). *Note: Cis-trans isomerism of the Cbz carbamate moiety results in split ^{13}C signal for N-**C**H$_2$-Ph; N-**C**H$_2$(CH$_5$)O-;* (**C**H$_2$)$_4$. *Broadening of corresponding -C**H**$_2$- groups in ^{1}H-NMR. The ^{13}C signal of **C**=O (Cbz) (δ 156.4) did not appear in ^{13}C-NMR and was confirmed with additional information from ^{1}H-^{13}C HMBC.*

N-benzyl-N-benzyloxycarbonyl-6-aminohexyl 2-O-benzoyl-4,6-di-O-benzyl-3-O-(2-naphthalenylmethyl)-β-D-galactopyranosyl-(1→4)-6-O-benzoyl-2,3-di-O-benzyl-α-D-galactopyranosyl-(1→3)-2,4,6-tri-O-benzoyl-β-D-galactopyranoside (25)

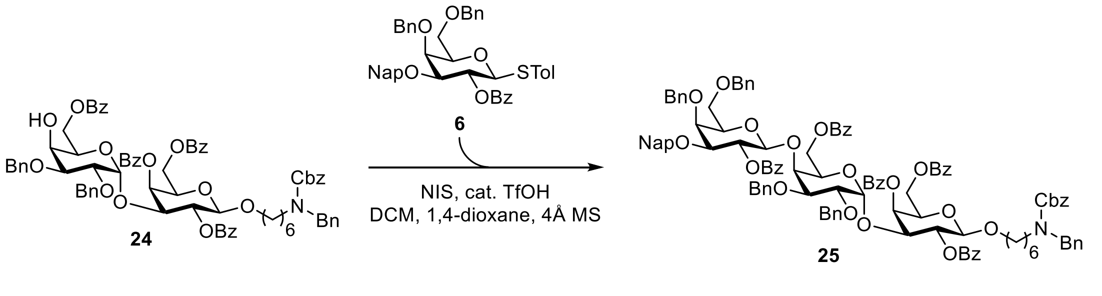

Trisaccharide **25** was prepared using several different conditions summarized in Table S2. The procedure that afforded the highest yield follows (entry d). Glycosyl acceptor **24** (19.5 mg, 15.4 μmol) and glycosyl donor **6** (18.1 mg, 25.4 μmol) were co-evaporated from anhydrous toluene and dried overnight under high vacuum. The donor, acceptor, and freshly MW-activated and crushed molecular sieves (4Å, ~0.3 g) were suspended in anhydrous DCM (0.9 ml) and stirred at RT for 15 min, then cooled to -25°C in an acetone bath with an immersion cooler and stirred for an additional 30 min. Activator

solution (0.21 ml, anhydrous DCM and anhydrous 1,4-dioxane (4:1)) containing recrystallized NIS (8.6 mg, 0.038 mmol) and TMSOTf (0.92 µl, 5.1 µmol) was added dropwise. The reaction was stirred for 20 min, gradually reaching -20°C. The reaction mixture was diluted with DCM + ~0.1% TEA (~10 ml), and filtered through celite, directly onto aqueous $Na_2S_2O_3$ (10% w/w, ~5 ml) and sat. $NaHCO_3$ (~5 ml). The celite was washed with DCM (~20 ml) and the combined filtrate was washed with an additional portion of aqueous $Na_2S_2O_3$ (10% w/w, ~3 ml) and sat. $NaHCO_3$ (~5 ml). The layers were separated, the organic phase was washed with brine, dried over Na_2SO_4, then filtered and concentrated under reduced pressure. Compound **25** was purified by Prep-HPLC (17.6 mg, 62% yield, elution at 100% ACN). HRMS (ESI-QTOF) m/z: $[M+H]^+$ Calcd for $C_{113}H_{110}NO_{23}$ 1848.7463; found 1848.7467. ^1H NMR (500 MHz, $CDCl_3$): δ , ppm 8.10 (d, *J* = 7.6 Hz, 2H), 8.04 (dd, *J* = 1.1, 8.2 Hz, 2H), 7.95 (d, *J* = 7.7 Hz, 2H), 7.91 - 7.88 (m, 2H), 7.79 (dd, *J* = 1.1, 8.2 Hz, 2H), 7.73 (d, *J* = 7.9 Hz, 1H), 7.60 - 7.49 (m, 5H), 7.49 - 7.39 (m, 9H), 7.38 - 7.27 (m, 18H), 7.25 - 7.18 (m, 6H), 7.13 - 7.11 (m, 1H), 7.08 (t, *J* = 7.8 Hz, 2H), 7.03 - 6.97 (m, 2H), 6.93 (d, *J* = 4.4 Hz, 4H), 6.87 (t, *J* = 7.6 Hz, 2H), 6.52 (d, *J* = 7.4 Hz, 2H), 5.80 (d, $J_{3,4}$ = 2.7 Hz, 1H, H-4), 5.56 (dd, $J_{1,2}$ = 7.9 Hz, $J_{2,3}$ = 10.1 Hz, 1H, H-2''), 5.53 (dd, $J_{1,2}$ = 8.0 Hz, $J_{2,3}$ = 10.4 Hz, 1H, H-2), 5.19 (d, $J_{1,2}$ = 3.4 Hz, 1H, H-1'(Galα)), 5.14 (br, 2H, -C***H*₂**-(Cbz)), 4.98, 4.63 (ABq, $\Delta\delta_{AB}$ = 0.35, J_{AB} = 11.8 Hz, 2H, O-C$\boldsymbol{H_2}$-Ar), 4.73, 4.56 (ABq, $\Delta\delta_{AB}$ = 0.17, J_{AB} = 12.8 Hz, 2H, O-C$\boldsymbol{H_2}$-Ar), 4.59 (dd, J_{AB} = 11.9 Hz, $J_{5,6}$ = 4.3 Hz, 1H, H-6'A), 4.54 (d, $J_{1,2}$ = 7.9 Hz, 1H, H-1''(Galβ)), 4.46 (dd, J_{AB} = 11.3 Hz, $J_{5,6}$ = 6.6 Hz, 1H, H-6A), 4.40 - 4.37 (br, 2H, -C$\boldsymbol{H_2}$-(NBn)), 4.34 (s, 2H, O-C$\boldsymbol{H_2}$-Ar), 4.31 - 4.23 (m, 2H, H-6B; H-6'B), 4.20, 4.00 (ABq, $\Delta\delta_{AB}$ = 0.20, J_{AB} = 11.6 Hz, 2H, O-C$\boldsymbol{H_2}$-Ar), 4.06 (d, $J_{1,2}$ = 8.0 Hz, 1H, H-1(Galβ)), 4.01 (dd, $J_{3,4}$ = 3.6 Hz, $J_{2,3}$ = 10.1 Hz, 1H, H-3), 3.96 (d, $J_{3,4}$ = 2.7 Hz, 1H, H-4''), 3.93, 3.49 (ABq, $\Delta\delta_{AB}$ = 0.44, J_{AB} = 11.4 Hz, 2H, O-C$\boldsymbol{H_2}$-Ar), 3.89 (dd, $J_{5,6A}$ = 4.4, $J_{5,6B}$ = 7.8 Hz, 1H, H-5'), 3.72 - 3.72 (br, 1H, -OC$\boldsymbol{H_2}$(CH_2)$_5$N-), 3.69 (t, $J_{5,6}$ = 6.6 Hz, 1H, H-5), 3.60 - 3.55 (m, 1H, H-6''A), 3.53 - 3.49 (m, 2H, H-3''; H-5''), 3.46 (dd, $J_{1,2}$ = 3.4 Hz, $J_{2,3}$ = 10.0 Hz, 1H, H-2'), 3.41 - 3.37 (m, 2H, H-6''B; H-4'), 3.29 (dd, $J_{3,4}$ = 2.8, $J_{2,3}$ = 10.0 Hz, 1H, H-3'), 3.22 - 3.22 (br, 1H, -OC$\boldsymbol{H_2}$(CH_2)$_5$N-), 3.09 - 3.00 (br, 2H, -O(CH_2)$_5$C$\boldsymbol{H_2}$N-), 1.34 - 1.21 (m, 4H, -(C$\boldsymbol{H_2}$)$_4$-), 1.07 - 1.02 (m, 4H, -(C$\boldsymbol{H_2}$)$_4$-), grease (δ 1.50 - 1.00), H_2O (δ 1.72). $^{13}C\{^1H\}$ NMR (126 MHz, $CDCl_3$): δ , ppm 166.1 (**C**=O (Bz)), 165.8 (**C**=O (Bz)), 165.8 (**C**=O (Bz)), 165.4 (**C**=O (Bz)), 164.9 (**C**=O (Bz)), 138.7, 138.6, 138.5, 138.0, 135.2, 133.3, 133.2, 133.2, 133.1, 133.1, 133.0, 132.7, 130.5, 130.2, 130.1, 130.1, 129.8, 129.8, 129.7, 129.6, 129.1, 128.7, 128.6, 128.5, 128.4, 128.3, 128.2, 128.2, 128.1, 128.0, 127.9, 127.9, 127.9, 127.7, 127.6, 127.6, 127.5, 127.3, 126.8, 126.5, 126.5, 126.1, 125.9, 125.8, 101.9 (C-1(Galβ)), 101.8 (C-1''(Galβ)), 93.3 (C-1'(Galα)), 79.4 (C-5''), 77.5 (C-3'), 76.4 (C-2'), 74.5 (O-**C**H_2-Ar), 74.3 (C-3''), 73.6 (O-**C**H_2-Ar), 73.4 (C-4'), 73.4 (O-**C**H_2-Ar), 72.6 (C-6''), 72.5 (C-4''), 71.9 (C-3), 71.8 (C-2''), 71.6 (O-**C**H_2-Ar), 71.5 (C-5), 70.5 (C-2), 70.2 (-O**C**H_2(CH_2)$_5$N-), 68.8 (C-5'), 68.7 (C-6''), 67.2 (-**C**H_2-(Cbz)), 65.7 (C-4), 64.4 (C-6'), 62.3 (C-6), 50.4 (-**C**H_2-(NBn)), 50.1 (-**C**H_2-(NBn)), 47.1 (-O(CH_2)$_5$**C**H_2N-), 46.1 (-O(CH_2)$_5$**C**H_2N-), 29.8 (-(**C**H_2)$_4$-), 29.3 (-(**C**H_2)$_4$-), 27.9 (-(**C**H_2)$_4$-), 27.5 (-(**C**H_2)$_4$-), 26.4 (-(**C**H_2)$_4$-), 25.6 (-(**C**H_2)$_4$-). *Note: Cis-trans isomerism of the Cbz carbamate moiety results in split ^{13}C signal for N-**C**H_2-Ph; N-**C**H_2(CH_5)O-; (**C**H_2)$_4$. Broadening of corresponding -C$\boldsymbol{H_2}$-groups in ^1H-NMR. The ^{13}C signal of **C**=O (Cbz) (δ 156.4) did not appear in ^{13}C-NMR and was confirmed with additional information from ^1H-^{13}C HMBC. Note 2: The configuration of the anomeric carbons C-1, C-1'and C-1'' was assigned with additional information from non-decoupled ^1H-^{13}C HSQC (vide infra, ^1H-^{13}C non-decoupled HSQC spectrum **25**): 101.9 (d, J=159Hz, C-1 (β)), 101.8 (d, J=163Hz, C-1'' (β)), 93.3 (d, J=172Hz, C-1' (α)).*

Table S2. Synthesis of 25 *via* glycosylation of 24 with 6 with different conditions

entry	donor equiv.	TMSOTf equiv.	temperature (°C)	time (min)	yield (%)
a	1.3	0.1	-40 → -20	40	-
b	1.3	0.3	-40 → -20	40	22
c	1.6	0.2	-30 → -25	20	50
d	1.6	0.2	-25 → -20	20	62
e	1.6	0.2	-20 → -15	20	48

N-benzyl-N-benzyloxycarbonyl-6-aminohexyl 2-O-R-4,6-di-O-benzyl-3-O-(2-naphthalenylmethyl)-β-D-galactopyranosyl-(1→4)-2,3-di-O-benzyl-α-D-galactopyranosyl-(1→3)-β-D-galactopyranoside (26)

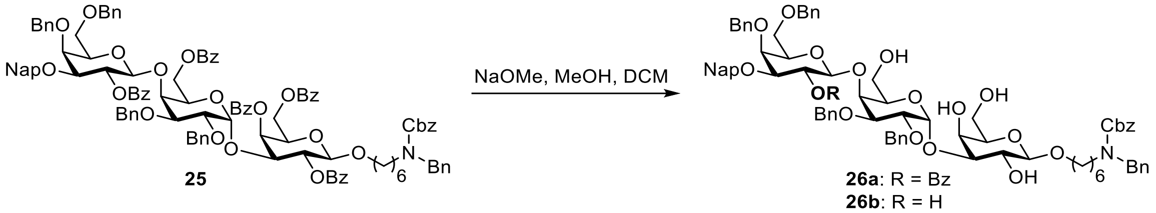

Compound **25** (12.4 mg, 6.71 μmol) was dissolved in anhydrous DCM (0.3 ml) and cooled to 0°C in an ice-water bath. NaOMe in anhydrous MeOH solution (0.5 M, 0.4 ml) was added dropwise. The reaction mixture was stirred at RT. After 1 h a single product was formed (as indicated by TLC, 5% MeOH in DCM, R_f=0.40), which contained one remaining benzoate ester LC-MS (ESI-QqQ-CEM) m/z: $[M+H]^+$ Calcd for $C_{85}H_{94}NO_{19}$ 1432.6; found 1432.6. The reaction mixture was stirred for an additional 23 h. Ion exchange beads Amberlight® XAD16N (H-form, pre-washed in DCM and MeOH) were added in small portions until a neutral pH was reached and the reaction mixture turned clear. The reaction mixture was filtered, and the filtrate was concentrated under reduced pressure. The crude product was purified by Prep-HPLC to obtain **26a** (4.3 mg, 45% yield, elution at 91% ACN) and **26b** (4.7 mg, 53% yield, elution at 87% ACN). Compound **26a**: HRMS (ESI-QTOF) m/z: $[M+H]^+$ Calcd for $C_{85}H_{94}NO_{19}$ 1432.6415; found 1432.6355. ^{1}H NMR (700 MHz, $CDCl_3$): δ, ppm 7.85 (d, *J* = 7.6 Hz, 2H), 7.77 (d, *J* = 7.9 Hz, 1H), 7.62 - 7.58 (m, 2H), 7.53 (d, *J* = 8.4 Hz, 1H), 7.49 - 7.44 (m, 2H), 7.41 (t, *J* = 7.4 Hz, 1H), 7.37 - 7.27 (m, 16H), 7.25 - 7.20 (m, 6H), 7.19 - 7.11 (m, 6H), 7.11 - 7.07 (m, 2H), 6.92 - 6.90 (m, 2H), 5.70 (dd, $J_{1,2}$ = $J_{2,3}$ = 9.0 Hz, 1H, H-2"), 5.16 (br d, J_{AB} = 25.8 Hz, 2H, -C***H*₂**-(Cbz)), 5.02, 4.63 (ABq, $\Delta\delta_{AB}$ = 0.39, J_{AB} = 11.8 Hz, 2H, O-C***H*₂**-Ar), 4.80, 4.64 (ABq, $\Delta\delta_{AB}$ = 0.16, J_{AB} = 12.6 Hz, 2H, O-C***H*₂**-Ar), 4.67 (d, $J_{1,2}$ = 7.8 Hz, 1H, H-1"(Galβ)), 4.59 (d, $J_{1,2}$ = 3.6 Hz, 1H, H-1'(Galα)), 4.56, 4.37 (ABq, $\Delta\delta_{AB}$ = 0.19, J_{AB} = 11.8 Hz, 2H, O-C***H*₂**-Ar), 4.54 - 4.45 (m, 4H, -C***H*₂**-(NBn); O-C***H*₂**-Ar), 4.19 - 4.13 (br, 2H, H-1(Galβ); H-5), 4.12 - 4.08 (br, 1H, H-2), 4.05, 3.69 (ABq, $\Delta\delta_{AB}$ = 0.36, J_{AB} = 11.4 Hz, 2H, O-C***H*₂**-Ar), 3.90 - 3.87 (m, 4H, H-6A; H-6'A; H-4"; -OC***H*₂**(CH₂)₅N-), 3.80 (dd, $J_{3,4}$ = 2.7 Hz, $J_{2,3}$ = 10.0 Hz, 1H, H-3'), 3.72 (dd, J_{AB} = 11.7 Hz, $J_{5,6}$ = 4.2 Hz, 1H, H-6'B), 3.71 - 3.62 (m, 2H, H-6"A; H-3"), 3.62 - 3.52 (m, 5H, H-4'; H-4; H-5"; H-2'; H-6B), 3.48 - 3.37 (m, 4H, H-3; H-5'; H-6"B; -OC***H*₂**(CH₂)₅N-), 3.28 - 3.15 (br, 2H, -O(CH₂)₅C***H*₂**N-), 1.59 - 1.43 (m, 4H, -(C***H*₂**)₄-), 1.37 - 1.16 (m, 4H, -(C***H*₂**)₄-), H_2O/R-O***H*** exchange (δ 2.18). ^{13}C{^{1}H} NMR (176 MHz, $CDCl_3$): δ, ppm 165.5 (**C**=O (2"-O-Bz)), 138.4, 138.2, 137.9, 137.9, 137.4, 135.0, 133.1, 133.1, 133.0, 130.2, 130.1, 128.6, 128.5, 128.5, 128.4, 128.4, 128.3, 128.3, 128.1, 128.1, 128.0, 127.9, 127.9, 127.9, 127.8, 127.3, 126.8, 126.3, 126.1, 125.9, 103.1 (C-1(Galβ)), 102.9 (C-1"(Galβ)), 95.8 (C-1'(Galα)), 79.4 (C-3"), 78.7 (C-3), 77.9 (C-3'), 76.7 (C-5"), 74.7 (C-5), 74.6 (O-**C**H₂-Ar), 74.4 (O-**C**H₂-Ar), 73.9 (C-2'), 73.8 (O-**C**H₂-Ar; C-5'), 73.0 (O-**C**H₂-Ar), 72.6 (C-4"), 72.0 (O-**C**H₂-Ar), 71.7 (C-2"), 70.0 (C-2), 69.9 (C-4), 69.8 (-O**C**H₂(CH₂)₅N-), 69.2 (C-6"), 67.3 (-**C**H₂-(Cbz)), 66.4 (C-4'), 63.0 (C-6'), 59.7 (C-6), 50.5 (-**C**H₂-(NBn)), 50.1 (-**C**H₂-(NBn)), 47.0 (-O(CH₂)₅**C**H₂N-), 46.2 (-O(CH₂)₅**C**H₂N-), 29.4 (-(**C**H₂)₄-), 28.0 (-(**C**H₂)₄-), 27.5 (-(**C**H₂)₄-), 26.6 (-(**C**H₂)₄-), 26.4 (-(**C**H₂)₄-), 25.7 (-(**C**H₂)₄-), residual acetone (δ 207.2; 31.0). *Note: Cis-trans isomerism of the Cbz carbamate moiety results in split ^{13}C signal for N-**C**H₂-Ph; N-**C**H₂(CH₅)O-;* (**C**H₂)₄. *Broadening of corresponding -C**H₂**- groups in ^{1}H-NMR. The ^{13}C signal of **C**=O (Cbz) (δ 156.6) did not appear in ^{13}C-NMR and was confirmed with additional information from ^{1}H-^{13}C HMBC. Note 2: Assignment was done with additional information from ^{1}H-^{13}C HMBC using the H-1↔OCH₂(CH)₅N- correlation to assign the reducing H-1.* Compound **26b**: HRMS (ESI-QTOF) m/z: $[M+H]^+$ Calcd for $C_{78}H_{90}NO_{18}$ 1328.6152; found 1328.6086. ^{1}H NMR (700 MHz, $CDCl_3$): δ, ppm 7.86 - 7.83 (m, 3H), 7.82 - 7.79 (m, 1H), 7.54 - 7.52 (m, 1H), 7.50 - 7.47 (m, 2H), 7.39 - 7.27 (m, 23H), 7.25 - 7.23 (m, 4H), 7.19 - 7.14 (m, 1H), 5.17 (br d, *J* = 27.2 Hz, 2H, -C***H*₂**-(Cbz)), 5.01, 4.88 (ABq, $\Delta\delta_{AB}$ = 0.13, J_{AB} = 12.4 Hz, 2H, O-C***H*₂**-Ar), 4.93, 4.57 (ABq, $\Delta\delta_{AB}$ = 0.36, J_{AB} = 11.6 Hz, 2H, O-C***H*₂**-Ar), 4.84, 4.60 (ABq, $\Delta\delta_{AB}$ = 0.24, J_{AB} = 11.7 Hz, 2H, O-C***H*₂**-Ar), 4.83 - 4.77 (m, 1H, O-C***H*ₐ**H_B-Ar), 4.75 (d, $J_{1,2}$ = 3.7 Hz, 1H, H-1'(Galα)), 4.76 - 4.69 (m, 1H, O-CHₐ***H*_B**-Ar), 4.52 - 4.47

(br, 2H, -C$\boldsymbol{H_2}$-(NBn)), 4.48, 4.36 (ABq, $\Delta\delta_{AB}$ = 0.12, J_{AB} = 11.9 Hz, 1H), 4.22 (d, $J_{1,2}$ = 6.9 Hz, 1H, H-1''(Galβ)), 4.20 - 4.14 (m, 2H, H-1(Galβ); H-2), 4.13 - 4.08 (m, 2H, H-2''; H-5), 4.01 (dd, $J_{3,4}$ = 3.1 Hz, $J_{2,3}$ = 9.9 Hz, 1H, H-3'), 3.97 (dd, J_{AB} = 11.5 Hz, $J_{5,6}$ = 6.5 Hz, 1H, H-6'A), 3.92 (dd, $J_{1,2}$ = 3.5 Hz, $J_{2,3}$ = 9.9 Hz, 1H, H-2'), 3.89 - 3.82 (m, 2H, H-6A; -OC$\boldsymbol{H_2}$(CH_2)$_5$N-), 3.80 (dd, J_{AB} = 11.9 Hz, $J_{5,6}$ = 4.5 Hz, 1H, H-6'B), 3.78 (d, $J_{3,4}$ = 3.3 Hz, 1H, H-4'), 3.67 (d, $J_{3,4}$ = 2.6 Hz, 1H, H-4/H-4''), 3.68 - 3.64 (m, 1H, H-4/H-4''), 3.61 (m, 1H, H-6''A), 3.57 (dd, J_{AB} = 10.3 Hz, $J_{5,6}$ = 5.2 Hz, 1H, H-6B), 3.53 (d, $J_{2,3}$ = 7.6 Hz, 1H, H-3), 3.50 - 3.44 (m, 3H, -OC$\boldsymbol{H_2}$(CH_2)$_5$N-; H-5'; H-5''), 3.42 (d, $J_{2,3}$ = 9.1 Hz, 1H, H-3''), 3.28 - 3.16 (m, 3H, H-6''B; -O(CH_2)$_5$C$\boldsymbol{H_2}$N-), 1.62 - 1.44 (m, 4H, -(C$\boldsymbol{H_2}$)$_4$-), 1.36 - 1.18 (m, 4H, -(C$\boldsymbol{H_2}$)$_4$-), H_2O/R-O$\boldsymbol{H}$ exchange (δ 2.36). $^{13}C\{^1H\}$ NMR (176 MHz, $CDCl_3$): δ , ppm 138.4, 137.9, 137.6, 137.5, 137.3, 136.1, 133.4, 133.1, 128.8, 128.6, 128.5, 128.5, 128.4, 128.3, 128.3, 128.3, 128.2, 128.2, 128.1, 128.0, 127.9, 127.8, 127.8, 127.3, 126.5, 126.2, 126.0, 125.8, 106.6 (C-1''(Galβ)), 103.1 (C-1(Galβ)), 95.6 (C-1'(Galα)), 82.0 (C-3), 78.7 (C-3), 78.4 (C-5), 77.9 (C-3''), 76.3 (C-2'), 74.8 (O-**C**H_2-Ar), 74.5 (O-**C**H_2-Ar), 74.4 (C-5'/C-5''), 73.9 (C-5'/C-5''), 73.8 (C-4/C-4''), 73.6 (O-**C**H_2-Ar), 73.6 (O-**C**H_2-Ar), 73.0 (O-**C**H_2-Ar), 72.4 (C-2''), 69.9 (C-4/C-4''), 69.9 (-O**C**H_2(CH_2)$_5$N-), 69.6 (C-2), 69.4 (C-6''), 67.3 (-**C**H_2-(Cbz)), 66.4 (C-4'), 62.9 (C-6'), 59.7 (C-6), 50.5 (-**C**H_2-(NBn)), 50.1 (-**C**H_2-(NBn)), 47.0 (-O(CH_2)$_5$**C**H_2N-), 46.2 (-O(CH_2)$_5$**C**H_2N-), 29.8 (-(**C**H_2)$_4$-), 29.4 (-(**C**H_2)$_4$-), 28.0 (-(**C**H_2)$_4$-), 27.5 (-(**C**H_2)$_4$-), 26.6 (-(**C**H_2)$_4$-), 26.4 (-(**C**H_2)$_4$-), 25.7 (-(**C**H_2)$_4$-), 25.7 (-(**C**H_2)$_4$-). *Note: Cis-trans isomerism of the Cbz carbamate moiety results in split ^{13}C signal for N-**C**H_2-Ph; N-**C**H_2(CH_5)O-; (**C**H_2)$_4$. Broadening of corresponding -C$\boldsymbol{H_2}$- groups in ^1H-NMR. The ^{13}C signal of **C**=O (Cbz) (δ 156.6) did not appear in ^{13}C-NMR and was confirmed with additional information from ^1H-^{13}C HMBC. Note 2: Assignment was done with additional information from ^1H-^{13}C HMBC using the H-1↔OCH_2(CH)$_5$N- correlation to assign the reducing H-1.*

6-Aminohexyl 2-O-benzoyl-β-D-galactopyranosyl-(1→4)-6-O-benzoyl-α-D-galactopyranosyl-(1→3)-2,4,6-tri-O-benzoyl-β-D-galactopyranoside (27)

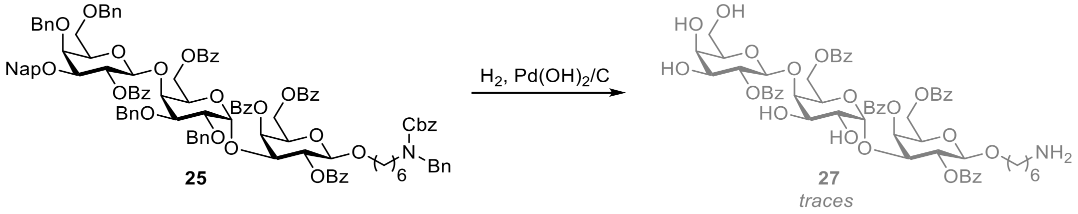

Method 1: Compound **25** (18 mg, 9.7 μmol) and $Pd(OH)_2/C$ (10-20% wt. loading, 20 mg) were suspended in EtOAc and ^{t}BuOH (1:1, 2 ml). The mixture was stirred in a pressurized reaction vessel under 4 bar of H_2 at RT. The progression of the reaction was monitored periodically by LC-MS (ESI-QqQ-CEM). After 5 days, aliquot of the reaction mixture contained byproducts with two (m/z: $[M+H]^+$ Calcd for $C_{73}H_{78}NO_{21}$ 1304.5; found 1304.5), three (m/z: $[M+H]^+$ Calcd for $C_{80}H_{84}NO_{21}$ 1394.6; found 1394.4), or four (m/z: $[M+H]^+$ Calcd for $C_{87}H_{90}NO_{21}$ 1484.6; found 1484.6) remaining benzyl ethers. Another portion $Pd(OH)_2/C$ (10-20% wt. loading, 20 mg) was added and the mixture was stirred in a pressurized reaction vessel under 4 bar of H_2 at RT. After 9 days, aliquot of the reaction mixture contained traces of the deprotected product (m/z: $[M+H]^+$ Calcd for $C_{59}H_{66}NO_{21}$ 1124.4; found 1124.4) and byproducts with one (m/z: $[M+H]^+$ Calcd for $C_{66}H_{72}NO_{21}$ 1214.5; found 1214.3), two or three remaining benzyl ethers. Further extension of the reaction time resulted in accumulation of hydrogenation byproducts.

Method 2: Compound **25** (9.8 mg, 5.3 μmol) and $Pd(OH)_2/C$ (10-20% wt. loading, 20 mg) were suspended in THF (1 ml). The mixture was stirred in a pressurized reaction vessel under 4 bar of H_2 at RT. The progression of the reaction was monitored periodically by LC-MS (ESI-QqQ-CEM). After 16 h, aliquot of the reaction mixture contained the deprotected product (m/z: $[M+H]^+$ Calcd for $C_{59}H_{66}NO_{21}$ 1124.4; found 1124.5) and byproducts with one (m/z: $[M+H]^+$ Calcd for $C_{66}H_{72}NO_{21}$ 1214.5; found 1214.4), two (m/z: $[M+H]^+$ Calcd for $C_{73}H_{78}NO_{21}$ 1304.5; found 1304.5), three (m/z: $[M+H]^+$ Calcd for $C_{80}H_{84}NO_{21}$ 1394.6; found 1394.5), or four (m/z: $[M+H]^+$ Calcd for $C_{87}H_{90}NO_{21}$ 1484.6; found 1484.5)

remaining benzyl ethers. After 40 h accumulation of new byproducts was observed. The reaction mixture was filtered through celite, and the celite was washed with THF and THF/TDW (2:1). The combined filtrate was concentrated under reduced pressure to produce a colorless glassy residue. The complex mixture was subjected to Prep-HPLC purification. Fraction eluted at 48% ACN contained compound **27** in sub-milligram amount. HRMS (ESI-QTOF) m/z: $[M+H]^+$ Calcd for $C_{59}H_{66}NO_{21}$ 1124.4122; found 1124.4097.

N-benzyl-N-benzyloxycarbonyl-6-aminohexyl 2-O-benzoyl-4,6-di-O-benzyl-β-D-galactopyranosyl-(1→4)-6-O-benzoyl-2,3-di-O-benzyl-α-D-galactopyranosyl-(1→3)-2,4,6-tri-O-benzoyl-β-D-galactopyranoside (28)

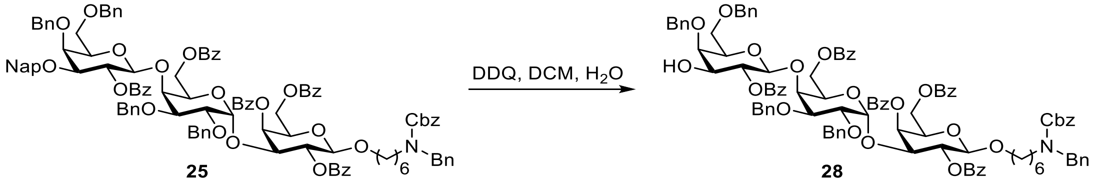

Compound **25** (15.3 mg, 8.27 μmol) was dissolved in DCM (0.9 ml) and TDW (0.1 ml). DDQ (3.1 mg, 0.014 mmol) was added. The reaction mixture obtained a green-yellow color. The reaction mixture was stirred for 2 h at RT, covered from light. A pink-red precipitate formed. Another portion of DDQ (4.3 mg, 0.019 mmol) was added. The reaction mixture was stirred for 40 min until the starting material was fully consumed and a major product was formed (as indicated by TLC, 40% EtOAc in hexane, R_f=0.47). The reaction was quenched by the addition of sat. $NaHCO_3$ (~5 ml), then diluted with DCM (~10 ml). The organic layer was washed with additional sat. $NaHCO_3$ (~20 ml). The aqueous layer had an intense red color, the organic phase was colorless. The layers were separated and the aqueous phase was extracted with DCM (1x 5 ml). The combined organic layer was washed with brine, dried over Na_2SO_4, filtered and evaporated to produce a yellowish syrup. Compound **28** was purified by Prep-HPLC (10.5 mg, 74% yield, elution at 95% ACN). HRMS (ESI-QTOF) m/z: $[M+H]^+$ Calcd for $C_{102}H_{102}NO_{23}$ 1708.6837; found 1708.6822. ^1H NMR (700 MHz, $CDCl_3$): δ, ppm 8.11 - 8.08 (br, 2H), 8.03 (d, *J* = 7.4 Hz, 2H), 8.00 - 7.96 (br, 2H), 7.91 (d, *J* = 7.4 Hz, 4H), 7.58 (t, *J* = 7.4 Hz, 1H), 7.56 - 7.52 (br, 1H), 7.48 - 7.42 (m, 5H), 7.37 - 7.27 (m, 20H), 7.26 - 7.23 (m, 4H), 7.23 - 7.18 (m, 4H), 7.15 - 7.11 (br, 1H), 7.11 - 7.08 (m, 4H), 7.02 (t, *J* = 7.4 Hz, 1H), 6.91 (t, *J* = 7.6 Hz, 2H), 6.62 (d, *J* = 7.5 Hz, 2H), 5.80 (d, $J_{3,4}$ = 2.7 Hz, 1H, H-4), 5.57 (dd, $J_{1,2}$ = 8.2 Hz, $J_{2,3}$ = 10.1 Hz, 1H, H-2), 5.19 (d, $J_{1,2}$ = 3.3 Hz, 1H, H-1'(Galα)), 5.15 (dd, $J_{1,2}$ = 7.9 Hz, $J_{2,3}$ = 9.8 Hz, 1H, H-2"), 5.16 - 5.14 (br, 2H, -C***H***$_2$-(Cbz)), 4.68 (ABq, $\Delta\upsilon_{AB}$ = 12.4 Hz, *J* = 12.4 Hz, 2H, O-C***H***$_2$-Ph), 4.62 (d, $J_{1,2}$ = 7.9 Hz, 1H, H-1"(Galβ)), 4.56 (dd, J_{AB} = 11.6 Hz, $J_{5,6}$ = 4.4 Hz, 1H, H-6'A), 4.47 (dd, J_{AB} = 10.6 Hz, $J_{5,6}$ = 6.9 Hz, 1H, H-6A), 4.42 - 4.38 (br, 2H, -C***H***$_2$-(NBn)), 4.38, 4.14 (ABq, $\Delta\delta_{AB}$ = 0.24, J_{AB} = 11.6 Hz, 2H, O-C***H***$_2$-Ph), 4.36 (s, 2H, O-C***H***$_2$-Ph), 4.29 - 4.23 (m, 2H, H-6'B; H-6B), 4.11 (d, $J_{1,2}$ = 7.9 Hz, 1H, H-1(Galβ)), 4.03, 3.66 (ABq, $\Delta\delta_{AB}$ = 0.37, J_{AB} = 11.3 Hz, 2H, O-C***H***$_2$-Ph), 4.02 (dd, $J_{3,4}$ = 3.3 Hz, $J_{2,3}$ = 10.1 Hz, 1H, H-3), 3.94 (dd, $J_{5,6A}$ = 5.0 Hz, $J_{5,6B}$ = 6.9 Hz, 1H, H-5'), 3.85 (d, $J_{3,4}$ = 3.3 Hz, 1H, H-4"), 3.77 - 3.72 (br, 1H, -OC***H***$_2$(CH_2)$_5$N-), 3.70 (t, $J_{5,6}$ = 6.6 Hz, 1H, H-5), 3.62 (dd, $J_{3,4}$ = 3.5 Hz, $J_{2,3}$ = 10.0 Hz, 1H, H-3"), 3.58 - 3.54 (m, 2H, H-4'; H-6"A), 3.52 (dd, $J_{1,2}$ = 3.4 Hz, $J_{2,3}$ = 10.0 Hz, 1H, H-2'), 3.47 (t, $J_{5,6}$ = 6.6 Hz, 1H, H-5"), 3.39 (dd, $J_{3,4}$ = 2.7 Hz, $J_{2,3}$ = 9.9 Hz, 1H, H-2'), 3.41 - 3.37 (m, 1H, H-6"B), 3.29 - 3.21 (br, 1H, -OC***H***$_2$(CH_2)$_5$N-), 3.10 - 2.98 (br, 2H, -O(CH_2)$_5$C***H***$_2$N-), 1.41 - 1.17 (m, 4H, -(C***H***$_2$)$_4$-), 1.12 - 1.03 (m, 4H, -(C***H***$_2$)$_4$-), H_2O/3"-O***H*** exchange (δ 2.18). $^{13}C\{^1H\}$ NMR (176 MHz, $CDCl_3$): δ, ppm 166.8 (**C**=O (Bz)), 166.1 (**C**=O (Bz)), 165.8 (**C**=O (Bz)), 164.9 (**C**=O (Bz)), 156.8 (**C**=O(Cbz)), 156.2 (**C**=O(Cbz)), 138.8, 138.5, 138.2, 138.0, 137.8, 136.9, 133.3, 133.3, 133.2, 133.0, 130.4, 130.1, 129.8, 129.8, 129.8, 129.7, 129.7, 129.6, 129.0, 128.7, 128.6, 128.6, 128.6, 128.4, 128.3, 128.2, 128.0, 128.0, 127.9, 127.9, 127.7, 127.7, 127.6, 127.4, 127.3, 127.2, 126.7, 126.7, 101.8 (C-1(Galβ)), 101.1 (C-1"(Galβ)), 93.4 (C-1'(Galα)), 77.7 (C-3'), 76.5 (C-4"), 76.3 (C-2'), 75.4 (O-**C**H_2-Ph), 74.3 (C-4'), 74.1 (C-2"), 73.8 (O-**C**H_2-Ph), 73.5 (O-**C**H_2-Ph), 73.3 (C-3"), 73.2 (C-5"), 72.6 (O-**C**H_2-Ph), 72.1 (C-3), 71.5 (C-5), 70.6 (C-2), 70.3 (-O**C**H_2(CH_2)$_5$N-), 70.2 (-O**C**H_2(CH_2)$_5$N-), 68.8 (C-5'), 68.3 (C-6"), 67.2 (-**C**H_2-(Cbz)), 65.8 (C-4), 64.3 (C-6'), 62.3 (C-6), 50.4 (-**C**H_2-(NBn)), 50.1 (-**C**H_2-(NBn)), 47.1

(-O(CH_2)$_5$**C**H_2N-), 46.1 (-O(CH_2)$_5$**C**H_2N-), 29.3 (-(**C**H_2)$_4$-), 27.9 (-(**C**H_2)$_4$-), 27.5 (-(**C**H_2)$_4$-), 26.4 (-(**C**H_2)$_4$-), 25.6 (-(**C**H_2)$_4$-). *Note: Cis-trans isomerism of the Cbz carbamate moiety results in split* ^{13}*C signal for N-***C***H_2-Ph; N-***C***H_2(CH_5)O-;* **C***=O (Cbz);* (**C**H_2)$_4$*. Broadening of corresponding -C***H_2***- groups in* ^{1}*H-NMR.*

6-Aminohexyl β-D-galactopyranosyl-(1→4)-α-D-galactopyranosyl-(1→3)-β-D-galactopyranoside (nSt)

Method 1 (from ***26b****):*

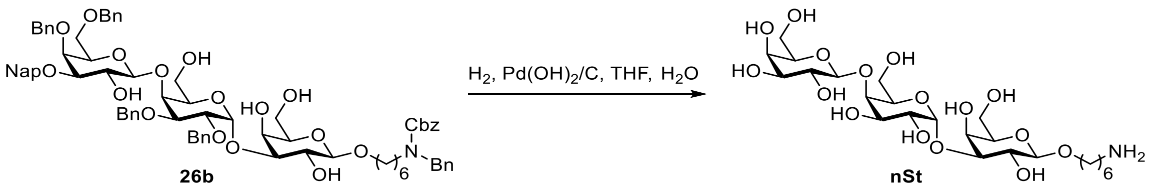

Compound **26b** (4.7 mg, 3.5 μmol) and $Pd(OH)_2$/C (10-20% wt. loading, 7.7 mg) were suspended in THF and TDW (1:1, 1 ml). The mixture was stirred in a pressurized reaction vessel under 4 bar of H_2 at RT. The progression of the reaction was monitored periodically by LC-MS (ESI-QqQ-CEM). After 16 h aliquot of the reaction contained byproducts with one (m/z: $[M+H]^+$ Calcd for $C_{31}H_{52}NO_{16}$ 694.3; found 694.3), two (m/z: $[M+H]^+$ Calcd for $C_{38}H_{58}NO_{16}$ 784.4; found 784.4), or three (m/z: $[M+H]^+$ Calcd for $C_{45}H_{64}NO_{16}$ 874.4; found 874.5) remaining benzyl ethers. Another portion $Pd(OH)_2$/C (10-20% wt. loading, 6.6 mg) was added, and the mixture was stirred in a pressurized reaction vessel under 4 bar of H_2 at RT for 24 h. LC-MS indicated completion of the reaction. The reaction mixture was filtered through a Whatman 42 filter paper, then the filter paper was washed with more TDW, and the filtrate concentrated under reduced pressure. The crude product was dissolved in a minimal amount of TDW (~50 μl) and passed through an a Orbilica pre-packed SPE cartridge (200 mg/3 ml, C18). The cartridge was conditioned in 85% ACN in TDW, then equilibrated in TDW. The crude product was loaded onto the SPE cartridge. Compound **nSt** was eluted with TDW (~1.5 ml) and lyophilized (1.6 mg, 75% yield).

Method 2 (from ***28****):*

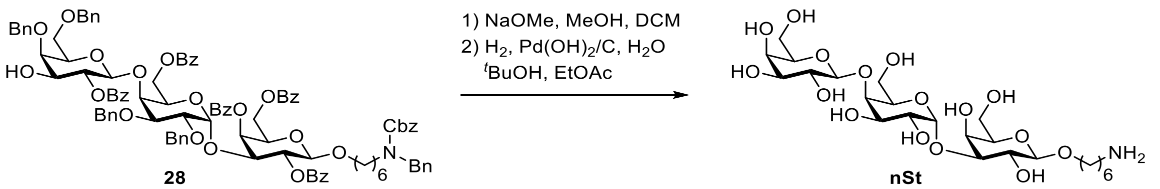

Compound **28** (10.5 mg, 6.14 μmol) was dissolved in anhydrous DCM (0.4 ml) and cooled to 0°C in an ice-water bath. NaOMe in anhydrous MeOH solution (0.5 M, 0.5 ml) was added dropwise. The reaction mixture was stirred at RT for 2 h until completion (as indicated by TLC, 5% MeOH in DCM, R_f=0.33). Ion exchange beads Amberlight® XAD16N (H-form, pre-washed in DCM and MeOH) were added in small portions until a neutral pH was reached and reaction mixture turned clear. The reaction mixture was filtered and the filtrate was concentrated under reduced pressure, to obtain the crude product as a white solid (7.5 mg). HRMS (ESI-QTOF) m/z: $[M+H]^+$ Calcd for $C_{67}H_{82}NO_{18}$ 1188.5526; found 1188.5519. The crude product and $Pd(OH)_2$/C (10-20% wt. loading, 13.4 mg) were suspended in EtOAc, ^{t}BuOH, and TDW (0.5:1:0.5, 2 ml). The mixture was stirred in a pressurized reaction vessel under 4 bar of H_2 at RT for 20 h. The reaction mixture was filtered through celite, and the celite was washed with ^{t}BuOH/TDW (1:1). The combined filtrate was concentrated under reduced pressure to produce a colorless glassy residue. Compound **nSt** was purified by SEC. Sephadex LH-20 size exclusion resin (120 mg, 140-550 mesh) was swelled in TDW for 4 h. The crude product was dissolved in an ammonium formate buffer (pH = 4.5, 1.75 M, 100 μl) and loaded on the SEC column. Trisaccharide **nSt** was eluted with TDW within the first 1.5 column void volume (3.6 mg, 97% 2-step yield).

Compound **nSt**: HRMS (ESI-QTOF) m/z: $[M+H]^+$ Calcd for $C_{24}H_{46}NO_{16}$ 604.2811; found 604.2796. ^{1}H NMR (700 MHz, D_2O): δ, ppm 5.17 (d, $J_{1,2}$ = 3.9 Hz, 1H, H-1'(Galα)), 4.61 (d, $J_{1,2}$ = 7.9 Hz, 1H, H-1"(Galβ)), 4.46 (d, $J_{1,2}$ = 8.0 Hz, 1H, H-1(Galβ)), 4.28 (d, $J_{3,4}$ = 3.0 Hz, 1H, H-4'), 4.26 (t, $J_{5,6}$ = 6.6 Hz, 1H, H-5'), 4.18 (d, $J_{3,4}$ = 3.1 Hz, 1H, H-4), 4.07 (dd, $J_{3,4}$ = 3.1 Hz, $J_{2,3}$ = 10.4 Hz, 1H, H-3'), 3.97 (dd, $J_{1,2}$ = 3.9, $J_{2,3}$ = 10.3 Hz, 1H, H-2'), 3.95 (m, 1H, -OC***H***$_2$(CH_2)$_5$$NH_2$), 3.92 (d, $J_{3,4}$ = 3.4 Hz, 1H, H-4"), 3.86 (dd, J_{AB} = 11.7 Hz, $J_{5,6}$ = 6.4 Hz, 1H, H-6'A), 3.83 - 3.76 (m, 4H, H-6; H-6"), 3.76 - 3.74 (m, 1H, H-3), 3.72 (dd, $J_{5,6}$ = 5.1 Hz, J_{AB} = 12.4 Hz, 1H, H-6'B), 3.72 - 3.69 (m, 3H, H-5; H-5"; -OC***H***$_2$(CH_2)$_5$$NH_2$), 3.69 - 3.67 (m, 1H, H-3"), 3.64 (dd, $J_{1,2}$ = 8.5 Hz, $J_{2,3}$ = 9.3 Hz, 1H, H-2), 3.60 (dd, $J_{1,2}$ = 8.1 Hz, $J_{2,3}$ = 9.7 Hz, 1H, H-2"), 3.01 (t, J = 7.6 Hz, 2H, -O(CH_2)$_5$C***H***$_2$$NH_2$), 1.70 - 1.64 (m, 4H, -OCH_2C***H***$_2$(CH_2)$_2$C***H***$_2$$CH_2$$NH_2$), 1.45 - 1.41 (m, 4H, -O(CH_2)$_2$ (C***H***$_2$)$_2$(CH_2)$_2$$NH_2$). ^{13}C{^{1}H} NMR (176 MHz, D_2O): δ, ppm 99.9 (C-1"(Galβ)), 98.1 (C-1(Galβ)), 91.0 (C-1'(Galα)), 73.7 (C-4'), 73.1 (C-3), 70.6 (C-5/C-5"), 70.3 (C-5/C-5"), 68.3 (C-3"), 66.9 (C-2"), 65.8 (-O**C**H_2(CH_2)$_5$$NH_2$), 65.6 (C-5'), 65.2 (C-3'), 64.7 (C-2), 64.1 (C-2'), 64.1 (C-4"), 60.4 (C-4), 56.5 (C-6/C-6"), 56.4 (C-6/C-6"), 55.9 (C-6'), 34.9 (-O(CH_2)$_5$**C**$H_2$$NH_2$), 23.9 (-(**C**$H_2$)$_4$-), 22.1 (-(**C**$H_2$)$_4$-), 20.7 (-(**C**$H_2$)$_4$-), 20.0 (-(**C**$H_2$)$_4$-). *Note: Assignment of the reducing H-1 was done with additional information from ^{1}H-^{13}C HMBC using the H-1↔OCH$_2$(CH)$_5$N- correlation. Note 2: The configuration of the anomeric carbons C-1, C-1', and C-1" was assigned with additional information from non-decoupled ^{1}H-^{13}C HSQC (vide infra, ^{1}H-^{13}C non-decoupled HSQC spectrum* ***nSt****): 99.9 (d, J=162Hz, C-1" (β)), 98.1 (d, J=160Hz, C-1 (β)), 91.0 (d, J=171Hz, C-1' (α)).*

N-benzyl-N-benzyloxycarbonyl-6-aminohexyl 2,3-di-O-benzyl-4-O-(9-fluorenylmethyloxycarbonyl)-6-O-(tert-butyldiphenylsilyl)-α-D-galactopyranosyl-(1→3)-2,4,6-tri-O-benzoyl-β-D-galactopyranoside (S12)

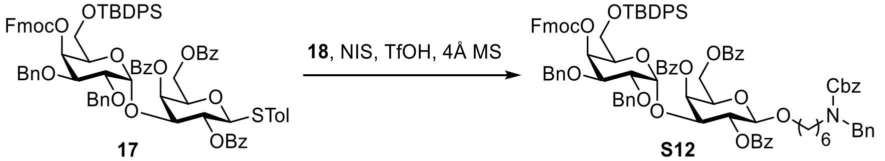

Glycosyl donor **17** (40 mg, 0.029 mmol) and acceptor **18** (30 mg, 0.089 mmol) were co-evaporated from anhydrous toluene and dried overnight under high vacuum. The donor, acceptor, and freshly MW-activated and crushed molecular sieves (4Å, ~1 g) were suspended in anhydrous DCM (1.5 ml) and stirred at RT for 1.5 h, then cooled to 0°C in an ice-water bath. Activator solution (1.0 ml, anhydrous DCM and anhydrous 1,4-dioxane (2:1)) containing recrystallized NIS (33 mg, 0.15 mmol) and TfOH (2.0 µl, 0.023 mmol) was added dropwise. The reaction mixture was stirred at 0°C for 5 min, after which the reaction mixture was allowed to reach 13°C over 1 h and 15 min. Another portion of the activator solution (0.3 ml, anhydrous DCM and anhydrous 1,4-dioxane (2:1)) containing recrystallized NIS (9.9 mg, 0.045 mmol) and TfOH (0.60 µl, 6.9 µmol) was added and the reaction mixture was allowed to reach 17°C over an additional 45 min. The reaction mixture was filtered through celite, directly onto aqueous $Na_2S_2O_3$ (10% w/w, ~2 ml) and sat. $NaHCO_3$ (~2 ml). The celite was washed with DCM (~30 ml) and the combined filtrate was washed with an additional portion of aqueous $Na_2S_2O_3$ (10% w/w, ~5 ml) and sat. $NaHCO_3$ (~5 ml). The layers were separated and the aqueous layer was extracted with DCM (3x 5 ml). The combined organic phase was washed with brine, dried over Na_2SO_4, then filtered and concentrated under reduced pressure. Compound **S12** was purified by Prep-HPLC (34.7 mg, 75% yield, elution at 100% ACN). HRMS (ESI-QTOF) m/z: $[M+H]^+$ Calcd for $C_{99}H_{100}NO_{18}Si$ 1618.6704; found 1618.6685. ^{1}H NMR (500 MHz, $CDCl_3$): δ, ppm 8.14 - 8.11 (m, 2H), 8.09 - 8.05 (m, 4H), 7.75 - 7.71 (m, 4H), 7.68 (dd, J = 1.5, 7.9 Hz, 2H), 7.61 - 7.57 (m, 1H), 7.57 - 7.50 (m, 2H), 7.50 - 7.27 (m, 24H), 7.25 - 7.20 (m, 1H), 7.19 - 7.04 (m, 13H), 5.99 (d, $J_{3,4}$ = 2.8 Hz, 1H, H-4), 5.74 (dd, $J_{1,2}$ = 8.0, $J_{2,3}$ = 10.2 Hz, 1H, H-2), 5.43 (d, $J_{1,2}$ = 3.4 Hz, 1H, H-1'(Galα)), 5.17 (s, 2H, -C***H***$_2$-(Cbz)), 4.74 (d, $J_{3,4}$ = 2.4 Hz, 1H, H-4'), 4.66 (d, $J_{1,2}$ = 8.1 Hz, 1H, H-1(Galβ)), 4.61 (dd, J_{AB} = 11.3 Hz, $J_{5,6A}$ = 6.6, 1H, H-6A), 4.53, 4.46 (ABq, $\Delta\delta_{AB}$ = 0.07, J_{AB} = 12.2 Hz, 2H, 2-O-C***H***$_2$-Ph), 4.43 (br, 2H, 2H, -C***H***$_2$-(NBn)), 4.40 - 4.35 (m, 2H, H-6B; H-3), 4.33 (d, J_{AB} = 11.0 Hz, 1H, 3-O-C***H***$_A$$H_B$-Ph),

4.27 - 4.22 (m, 2H, 3-O-CH$_A$***H$_B$***-Ph; -C***H***$_2$-(Fmoc)), 4.15 (dd, $J_{3,4}$ = 3.7, $J_{5,6}$ = 7.7 Hz, 1H, H-5'), 4.06 (dd, J_{AB} = 10.1 Hz, $J_{1,9'}$ = 8.0 Hz, 1H, -C***H***$_2$-(Fmoc)), 4.01 (t, J = 7.5 Hz, 1H, -C***H***-(Fmoc)), 3.95 (t, $J_{5,6}$ = 6.6 Hz, 1H, H-5), 3.92 - 3.87 (m, 1H, -OC***H***$_2$(CH$_2$)$_5$N-), 3.82 (dd, J_{AB} = 10.3 Hz, $J_{5,6A}$ = 7.6 Hz, 1H, H-6'A), 3.80 (dd, $J_{1,2}$ = 3.6 Hz, $J_{2,3}$ = 9.8 Hz, 1H, H-2'), 3.63 (dd, J_{AB} = 10.6 Hz, $J_{5,6B}$ = 4.0 Hz, 1H, H-6'B), 3.59 (dd, $J_{3,4}$ = 3.2 Hz, $J_{2,3}$ = 10.0 Hz, 1H, H-3'), 3.49 - 3.48 (m, 1H, -OC***H***$_2$(CH$_2$)$_5$N-), 3.15 - 3.05 (m, 2H, -O(CH$_2$)$_5$C***H***$_2$N-), 1.52 - 1.51 (br, 2H, -(C***H***$_2$)$_4$-), 1.31 - 1.21 (br, 4H, -(C***H***$_2$)$_4$-), 1.15 (s, 9H, SiPh$_2$-C(C***H***$_3$)$_3$), 1.09 - 1.05 (br, 2H, -(C***H***$_2$)$_4$-). ^{13}C{^{1}H} NMR (126 MHz, CDCl$_3$): δ, ppm 166.1 (**C**=O(4-O-Bz), 166.1 (**C**=O(6-O-Bz)), 164.8 (**C**=O(2-O-Bz)), 156.9 (**C**=O(Cbz)), 156.3 (**C**=O(Cbz)), 154.7 (**C**=O(Fmoc)), 143.6, 143.2, 141.2, 141.1, 138.3, 137.9, 137.8, 136.7, 135.7, 135.6, 134.9, 133.5, 133.4, 133.3, 133.2, 133.1, 133.1, 130.3, 130, 129.9, 129.9, 129.7, 129.6, 129.1, 128.6, 128.6, 128.6, 128.5, 128.0, 128.0, 127.9, 127.9, 127.8, 127.8, 127.7, 127.7, 127.4, 127.3, 127.2, 127.2, 127.1, 125.3, 125.1, 119.9, 119.9, 101.8 (C-1(Galβ)), 93.4 (C-1'(Galα)), 75.9 (C-3'), 74.7 (C-2'), 72.8 (O-**C**H$_2$-Ph), 72.5 (C-4'), 72.2 (C-3), 72.1 (O-**C**H$_2$-Ph), 71.6 (C-5), 70.9 (C-2), 70.2 (-O**C**H$_2$(CH$_2$)$_5$N-), 70.1 (C-5'; -**C**H$_2$-(Fmoc)), 67.3 (-**C**H$_2$-(Cbz)), 65.7 (C-5), 63.4 (C-6'), 62.4 (C-6), 50.5 (-**C**H$_2$-(NBn)), 50.2 (-**C**H$_2$-(NBn)), 47.1 (-O(CH$_2$)$_5$**C**H$_2$N-), 46.5 (-**C**H-(Fmoc)), 46.2 (-O(CH$_2$)$_5$**C**H$_2$N-), 29.4 (-(**C**H$_2$)$_4$-), 27.9 (-(**C**H$_2$)$_4$-), 27.5 (-(**C**H$_2$)$_4$-), 27.0 (SiPh$_2$-C-(**C**H$_3$)$_3$), 26.9 (-(**C**H$_2$)$_4$-), 26.6 (-(**C**H$_2$)$_4$-), 26.5 (-(**C**H$_2$)$_4$-), 25.7 (-(**C**H$_2$)$_4$-), 19.5 (SiPh$_2$-**C**-(CH$_3$)$_3$). *Note: Cis-trans isomerism of the Cbz carbamate moiety results in split ^{13}C signal for N-**C**H$_2$-Ph; N-**C**H$_2$(CH$_5$)O-; **C**=O (Cbz);* (**C**H$_2$)$_4$. *Broadening of corresponding -C**H**$_2$- groups in ^{1}H-NMR.*

N-benzyl-N-benzyloxycarbonyl-6-aminohexyl 2,3-di-O-benzyl-6-O-(tert-butyldiphenylsilyl)-α-D-galactopyranosyl-(1→3)-2,4,6-tri-O-benzoyl-β-D-galactopyranoside (29)

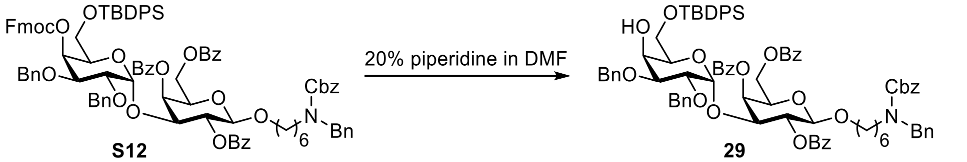

Compound **S12** (47.7 mg, 29.5 µmol) was dissolved in DMF (1.6 ml). Piperidine (0.4 ml, 4.0 mmol, 20% in DMF) was added dropwise. The reaction mixture was stirred at RT for 15 min. The reaction mixture was diluted with DCM (~30 ml) and washed with aqueous HCl (0.5 M). Small portions of aqueous HCl (0.5 M) were added until the aqueous phase registered pH~2 (~20 ml). The layers were separated and the aqueous phase was extracted with DCM (2x 5 ml). The combined organic phase was washed with brine. A small amount of sat. NaHCO$_3$ (~1 ml) was added to neutralize the slightly acidic aqueous layer. The organic layer was separated, dried over Na$_2$SO$_4$, filtered and evaporated. Compound **29** was purified by Prep-HPLC (32.8 mg, 80% yield, elution at 100% ACN). HRMS (ESI-QTOF) m/z: [M+H]$^+$ Calcd for C$_{84}$H$_{90}$NO$_{16}$Si 1396.6024; found 1396.5978. ^{1}H NMR (500 MHz, CDCl$_3$): δ, ppm 8.11 (dd, J = 1.1, 8.2 Hz, 2H), 8.06 (dd, J = 1.1, 8.3 Hz, 2H), 8.03 (d, J = 7.6 Hz, 2H), 7.73 - 7.68 (m, 4H), 7.60 - 7.53 (m, 2H), 7.49 - 7.42 (m, 4H), 7.41 - 7.37 (m, 7H), 7.36 - 7.26 (m, 9H), 7.22 - 7.20 (m, 4H), 7.15 - 7.05 (m, 8H), 5.96 (d, $J_{3,4}$ = 3.0 Hz, 1H, H-4), 5.71 (dd, $J_{1,2}$ = 8.0 Hz, $J_{2,3}$ = 10.2 Hz, 1H, H-2), 5.39 (d, J = 3.4 Hz, 1H, H-1'(Galα)), 5.16 (s, 2H, -C***H***$_2$-(Cbz)), 4.63 (d, $J_{1,2}$ = 8.0 Hz, 1H, H-1(Galβ)), 4.59 (dd, J_{AB} = 11.3 Hz, $J_{5,6A}$ = 6.6 Hz, 1H, H-6A), 4.49 (d, J_{AB} = 12.2 Hz, 1H, 2-O-C***H$_A$***H$_B$-Ph), 4.44 - 4.34 (m, 6H, H-6B; -C***H***$_2$-(NBn); 2-O-CH$_A$***H$_B$***-Ph; 3-O-C***H$_A$***H$_B$-Ph; H-3), 4.20 (d, J_{AB} = 11.5 Hz, 1H, 3-O-CH$_A$***H$_B$***-Ph;), 3.95 - 3.84 (m, 4H, H-5; H-5'; H-6'A; -OC***H***$_2$(CH$_2$)$_5$N-), 3.73 (dd, $J_{1,2}$ = 3.4 Hz, $J_{2,3}$ = 9.8 Hz, 1H, H-2'), 3.66 (dd, $J_{5,6B}$ = 3.0 Hz, J_{AB} = 10.6 Hz, 1H, H-6'B), 3.47 - 3.45 (m, 1H, -OC***H***$_2$(CH$_2$)$_5$N-), 3.43 (dd, $J_{3,4}$ = 3.2 Hz, $J_{2,3}$ = 9.8 Hz, 1H), 3.28 (d, $J_{3,4}$ = 2.5 Hz, 1H, H-4'), 3.14 - 3.04 (br, 2H, -O(CH$_2$)$_5$C***H***$_2$N-), 1.50 - 1.49 (br, 2H, -(C***H***$_2$)$_4$-), 1.33 - 1.18 (br, 4H, -(C***H***$_2$)$_4$-), 1.14 (s, 9H, SiPh$_2$-C(C***H***$_3$)$_3$), 1.09 - 1.03 (br, 2H, -(C***H***$_2$)$_4$-). ^{13}C{^{1}H} NMR (126 MHz, CDCl$_3$): δ, ppm 166.1 (**C**=O(4-O-Bz); **C**=O(6-O-Bz)), 164.8 (**C**=O(2-O-Bz)), 156.9 (**C**=O(Cbz)), 156.3 (**C**=O(Cbz)), 138.3, 138.1, 137.8, 136.8, 135.7, 135.7, 133.4, 133.3, 130.3, 129.9, 129.9, 129.7, 129.7, 129.1, 128.6, 128.5, 128.3, 128.0, 127.9, 127.8, 127.8, 127.8, 127.7, 127.6, 127.3,

127.2, 101.8 (C-1(Galβ)), 92.9 (C-1'(Galα)), 77.2 (C-3'), 74.7 (C-2'), 72.7 (O-**C**H_2-Ph), 72.3 (O-**C**H_2-Ph), 71.8 (C-3), 71.6 (C-5), 70.9 (C-2), 70.7 (C-5'), 70.2 (-O**C**$H_2(CH_2)_5$N-), 68.1 (C-4'), 67.3 (-**C**H_2-(Cbz)), 65.7 (C-4), 64.5 (C-6'), 62.4 (C-6), 50.5 (-**C**H_2-(NBn)), 50.2 (-**C**H_2-(NBn)), 47.1 (-O$(CH_2)_5$**C**H_2N-), 46.2 (-O$(CH_2)_5$**C**H_2N-), 29.4 (-(**C**H_2)$_4$-), 27.9 (-(**C**H_2)$_4$-), 27.5 (-(**C**H_2)$_4$-), 27.1 ($SiPh_2$-C-(**C**H_3)$_3$), 26.5 (-(**C**H_2)$_4$-), 25.6 (-(**C**H_2)$_4$-), 19.5 ($SiPh_2$-**C**-$(CH_3)_3$). *Note: Cis-trans isomerism of the Cbz carbamate moiety results in split* ^{13}C *signal for N-**C**H_2-Ph; N-**C**$H_2(CH_5)$O-; **C**=O (Cbz);* (**C**H_2)$_4$. *Broadening of corresponding -C**H_2**- groups in* ^1H*-NMR.*

N-benzyl-N-benzyloxycarbonyl-6-aminohexyl 2-O-benzoyl-4,6-di-O-benzyl-3-O-(2-naphthalenylmethyl)-β-D-galactopyranosyl-(1→4)-2,3-di-O-benzyl-6-O-(tert-butyldiphenylsilyl)-α-D-galactopyranosyl-(1→3)-2,4,6-tri-O-benzoyl-β-D-galactopyranoside (30)

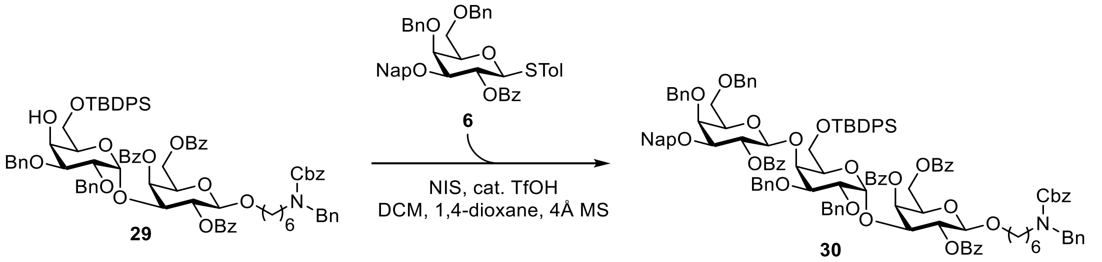

Glycosyl acceptor **29** (38 mg, 0.027 mmol) and glycosyl donor **6** (25 mg, 0.035 mmol) were co-evaporated from anhydrous toluene and dried overnight under high vacuum. The donor, acceptor, and freshly MW-activated and crushed molecular sieves (4Å, ~1.2 g) were suspended in anhydrous DCM (1 ml) and stirred at RT for 45 min, then cooled to -25°C in an acetone bath with an immersion cooler. Activator solution (1.1 ml, anhydrous DCM and anhydrous 1,4-dioxane (2:1)) containing recrystallized NIS (36 mg, 0.17 mmol) and TfOH (2.2 µl, 0.025 mmol) was added dropwise. The reaction mixture was stirred at -25°C for 5 min, after which the reaction mixture was allowed to reach -10°C over 40 min. Another portion of the activator solution (0.35 ml, anhydrous DCM and anhydrous 1,4-dioxane (2:1)) containing recrystallized NIS (12 mg, 0.052 mmol) and TfOH (0.70 µl, 8.0 µmol) was added and the reaction mixture was stirred at 0°C for an additional 1 h. The reaction mixture was filtered through celite, directly onto aqueous $Na_2S_2O_3$ (10% w/w, ~2 ml) and sat. $NaHCO_3$ (~2 ml). The celite was washed with DCM (~30 ml) and the combined filtrate was washed with an additional portion of aqueous $Na_2S_2O_3$ (10% w/w, ~10 ml) and sat. $NaHCO_3$ (~15 ml). The layers were separated and the aqueous layer was extracted with DCM (3x 5 ml). The combined organic phase was washed with brine, dried over Na_2SO_4, then filtered and concentrated under reduced pressure. Compound **30** was purified by Prep-HPLC (15.4 mg, 28% yield, elution at 100% ACN). HRMS (ESI-QTOF) m/z: $[M+H]^+$ Calcd for $C_{122}H_{124}NO_{22}Si$ 1982.8379; found 1982.8304. ^1H NMR (500 MHz, $CDCl_3$): δ, ppm 8.05 - 8.01 (m, 4H), 7.94 (d, J = 7.3 Hz, 2H), 7.76 - 7.68 (m, 7H), 7.60 - 7.52 (m, 3H), 7.49 - 7.40 (m, 7H), 7.36 - 7.27 (m, 24H), 7.26 - 7.18 (m, 9H), 7.13 - 7.13 (m, 1H), 7.06 (t, J = 7.7 Hz, 2H), 7.00 - 6.96 (m, 2H), 6.92 - 6.90 (m, 4H), 6.84 (t, J = 7.6 Hz, 2H), 6.46 (d, J = 7.4 Hz, 2H), 5.88 (d, $J_{3,4}$ = 2.5 Hz, 1H, H-4), 5.62 (dd, $J_{1,2}$ = 8.1 Hz, $J_{2,3}$ = 10.2 Hz, 1H, H-2), 5.43 (dd, $J_{1,2}$ = 8.0 Hz, $J_{2,3}$ = 10.0 Hz, 1H, H-2"), 5.30 (d, $J_{1,2}$ = 3.5 Hz, 1H, H-1'(Galα)), 5.15 (s, 2H, -C**H_2**-(Cbz)), 5.00, 4.56 (ABq, $\Delta\delta_{AB}$ = 0.44, J_{AB} = 11.7 Hz, 2H, -C**H_2**-Ar), 4.73, 4.55 (ABq, $\Delta\delta_{AB}$ = 0.18, J_{AB} = 12.7 Hz, 2H, -C**H_2**-Ar), 4.53 - 4.51 (m, 1H, H-6A), 4.49 (d, $J_{1,2}$ = 8.1 Hz, 1H, H-1(Galβ)), 4.41 (d, $J_{1,2}$ = 7.9 Hz, 1H, H-1"(Galβ)), 4.43 - 4.35 (m, 3H, H-3; -C**H_2**-(NBn)), 4.29 (ABq, $\Delta\upsilon_{AB}$ = 13.5 Hz, J_{AB} = 12.1 Hz, 2H, -C**H_2**-Ar), 4.24 - 4.19 (m, 1H, H-6B), 4.21, 3.98 (ABq, $\Delta\delta_{AB}$ = 0.23, J_{AB} = 11.5 Hz, 2H, -C**H_2**-Ar), 4.03 (dd, J_{AB} = 11.6 Hz, $J_{5,6A}$ = 8.2, 1H, H-6'A), 3.97, 3.48 (ABq, ΔδAB = 0.49, J_{AB} = 11.5 Hz, 2H, -C**H_2**-Ar), 3.95 - 3.89 (m, 2H, H-4"; H-5'), 3.81 (br, 1H, -OC**H_2**$(CH_2)_5$N-), 3.75 (t, $J_{5,6}$ = 6.5 Hz, 1H, H-5), 3.68 (dd, J_{AB} = 11.4 Hz, $J_{5,6B}$ = 2.0, 1H, H-6'B), 3.48 - 3.41 (m, 3H, H-3"; H-2'; H-6"A), 3.37 - 3.36 (br, 1H, -OC**H_2**$(CH_2)_5$N-), 3.32 - 3.30 (m, 1H, H-5"), 3.28 - 3.25 (m, 2H, H-4'; H-3'), 3.18 (dd, J_{AB} = 8.8 Hz, $J_{5,6B}$ = 5.1 Hz, 1H, H-6"B), 3.11 - 3.03 (m, 2H, -O$(CH_2)_5$C**H_2**N-), 1.47 - 1.42 (br, 2H, -(C**H_2**)$_4$-), 1.30 - 1.26 (br, 4H, -(C**H_2**)$_4$-), 1.14 (s, 9H, $SiPh_2$-C(C**H_3**)$_3$), 1.08

- 1.01 (br, 4H, -(C**H_2**)$_4$-). $^{13}C\{^1H\}$ NMR (126 MHz, $CDCl_3$): δ, ppm 166.0 (**C**=O(6-O-Bz)), 165.8 (**C**=O(4-O-Bz)), 165.4 (**C**=O(2"-O-Bz)), 164.8 (**C**=O(2-O-Bz)), 156.8 (**C**=O(Cbz)), 138.9, 138.8, 138.4, 137.9, 136.8, 135.6, 135.2, 134.1, 133.8, 133.3, 133.1, 133.1, 133.0, 132.6, 130.2, 130.2, 130.1, 129.8, 129.8, 129.7, 129.7, 129.1, 128.6, 128.5, 128.4, 128.4, 128.3, 128.2, 128.1, 128.0, 128.0, 127.9, 127.8, 127.8, 127.7, 127.5, 127.2, 126.8, 126.5, 126.5, 126.1, 125.9, 125.8, 101.9 (C-1(Galβ)), 101.6 (C-1"(Galβ)), 92.3 (C-1'(Galα)), 79.4 (C-3"), 77.7 (C-3'), 76.7 (C-2'), 74.9 (C-4'), 74.6 (O-**C**H_2-Ar), 73.5 (O-**C**H_2-Ar), 73.3 (O-**C**H_2-Ar), 73.1 (C-5"), 72.8 (C-4"), 72.7 (O-**C**H_2-Ar), 71.8 (C-5'), 71.8 (C-2"), 71.5 (C-5), 71.5 (O-**C**H_2-Ar), 70.9 (C-2), 70.9 (C-3), 70.1 (-O**C**$H_2(CH_2)_5$N-), 68.6 (C-6"), 67.3 (-**C**H_2-(Cbz)), 65.5 (C-6'), 65.4 (C-4), 62.1 (C-6), 50.4 (-**C**H_2-(NBn)), 50.1 (-**C**H_2-(NBn)), 47.1 (-O$(CH_2)_5$**C**H_2N-), 46.2 (-O$(CH_2)_5$**C**H_2N-), 29.4 (-(**C**H_2)$_4$-), 27.9 (-(**C**H_2)$_4$-), 27.5 (-(**C**H_2)$_4$-), 27.2 ($SiPh_2$-C-(**C**H_3)$_3$), 26.5 (-(**C**H_2)$_4$-), 25.6 (-(**C**H_2)$_4$-), 19.6 ($SiPh_2$-**C**-$(CH_3)_3$). *Note: Cis-trans isomerism of the Cbz carbamate moiety results in split ^{13}C signal for N-**C**H_2-Ph; N-**C**$H_2(CH_5)$O-; (**C**H_2)$_4$. Broadening of corresponding -C**H_2**- groups in ^{1}H-NMR. Note 2: Assignment was done with additional information from ^1H-^{13}C HMBC using the H-1'↔C-5' correlation to assign H-6'. The reducing H-1 was assigned using the H-1↔O$CH_2(CH)_5$N- correlation. Note 3: The configuration of the anomeric carbons C-1, C-1'and C-1" was assigned with additional information from non-decoupled ^1H-^{13}C HSQC (vide infra, ^1H-^{13}C non-decoupled HSQC spectrum **30**): 101.9 (d, J=158Hz, C-1 (β)), 101.6 (d, J=162Hz, C-1" (β)), 92.3 (d, J=171Hz, C-1' (α)).*

N-benzyl-N-benzyloxycarbonyl-6-aminohexyl 2,3-di-O-benzyl-6-O-levulinoyl-α-D-galactopyranosyl-(1→3)-2,4,6-tri-O-benzoyl-β-D-galactopyranoside (31)

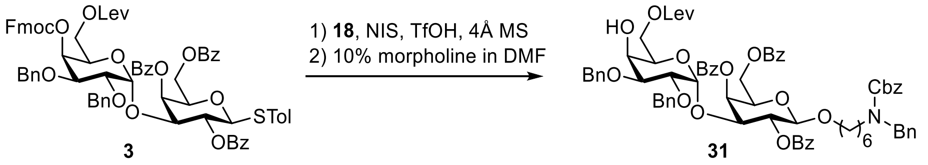

Glycosyl donor **3** (152 mg, 0.121 mmol) and acceptor **18** (122 mg, 0.357 mmol) were co-evaporated from anhydrous toluene and dried overnight under high vacuum. The donor, acceptor, and freshly MW-activated and crushed molecular sieves (4Å, ~1.8 g) were suspended in anhydrous DCM (5 ml) and stirred at RT for 1 h, then cooled to 0°C in an ice-water bath. Activator solution (1.6 ml, anhydrous DCM and anhydrous 1,4-dioxane (4:1)) containing recrystallized NIS (53 mg, 0.24 mmol) and TfOH (4.3 µl, 0.049 mmol) was added dropwise. The reaction mixture was stirred at 0°C for 10 min, after which the reaction mixture was allowed to reach 16°C over 2 h. The reaction mixture was diluted with DCM (~40 ml) and filtered through celite, directly onto aqueous $Na_2S_2O_3$ (10% w/w, ~4 ml) and sat. $NaHCO_3$ (~4 ml). The celite was washed with DCM (~10 ml) and the combined filtrate was washed with an additional portion of aqueous $Na_2S_2O_3$ (10% w/w, ~10 ml) and sat. $NaHCO_3$ (~10 ml). The layers were separated and the aqueous layer was extracted with DCM (5 ml). The combined organic phase was washed with brine, dried over Na_2SO_4, then filtered and concentrated under reduced pressure. The crude product was used directly in the next step without purification. The crude product was dissolved in DMF (13.5 ml) and cooled to 0°C in an ice-water bath. Morpholine (1.5 ml, 17.4 mmol, 10% in DMF) was added dropwise. The reaction mixture was stirred at 0°C for 20 min. The reaction mixture was diluted with DCM (~20 ml) and washed with aqueous HCl (0.1 M, ~20 ml). Small portions of aqueous HCl (0.5 M) were added until the aqueous phase registered pH~1. The layers were separated and the aqueous phase was extracted with DCM (2x 5 ml). The combined organic phase was washed with brine. A small amount of sat. $NaHCO_3$ (~2 ml) was added to neutralize the slightly acidic aqueous layer. The organic layer was separated, dried over Na_2SO_4, filtered and evaporated. The crude product was redissolved in toluene and evaporated to remove residual DMF. Compound **31** was purified by flash chromatography (107 mg, 71% 2-step yield, elution at 55% EtOAc in hexane). HRMS (ESI-QTOF) m/z: $[M+H]^+$ Calcd for $C_{73}H_{78}NO_{18}$ 1256.5213; found 1256.5229. ^1H NMR (500 MHz, $CDCl_3$): δ, ppm 8.08 (dd, J = 1.3, 8.4 Hz, 2H), 8.06 (dd, J = 1.3, 8.4 Hz, 2H), 8.02 (d, J = 7.6 Hz, 2H), 7.58 - 7.51 (m, 3H), 7.46 - 7.29 (m, 12H), 7.25 - 7.22 (m, 5H), 7.14 - 7.05 (m, 8H), 5.99 (d, $J_{3,4}$ = 2.7 Hz, 1H, H-4), 5.69 (dd, $J_{1,2}$ = 8.0, $J_{2,3}$

= 10.2 Hz, 1H, H-2), 5.30 (d, $J_{1,2}$ = 3.3 Hz, 1H, H-1'(Galα)), 5.14 (s, 1H, -C$\boldsymbol{H_2}$-(Cbz)), 4.80 (d, $J_{1,2}$ = 7.9 Hz, 1H, H-1(Galβ)), 4.61 (dd, J_{AB} = 11.3 Hz, $J_{5,6}$ = 6.5 Hz, 1H, H-6A), 4.49, 4.33 (ABq, $\Delta\delta_{AB}$ =0.16, J_{AB} = 12.3 Hz, 2H, O-C$\boldsymbol{H_2}$-Ph), 4.43, 4.23 (ABq, $\Delta\delta_{AB}$ = 0.20, J = 11.4 Hz, 2H, O-C$\boldsymbol{H_2}$-Ph), 4.40 (2H, br, -C$\boldsymbol{H_2}$-(NBn)), 4.38 (dd, J_{AB} = 11.4 Hz, $J_{5,6}$ = 6.6, 1H, H-6B), 4.29 (dd, $J_{3,4}$ = 3.3 Hz, $J_{2,3}$ = 10.2 Hz, 1H, H-3), 4.25 (dt, $J_{3,4}$ = 0.9 Hz, $J_{5,6}$ = 6.5 Hz, 1H, H-5), 4.15 (dd, J_{AB} = 11.6 Hz, $J_{5,6A}$ = 8.4 Hz, 1H, H-6'A), 4.05 (dd, J_{AB} = 11.6 Hz, $J_{5,6B}$ = 4.1 Hz, 1H, H-6'B), 3.91 - 3.90 (br, 1H, -OC$\boldsymbol{H_2}$(CH_2)$_5$N-), 3.88 (ddd, $J_{3,4}$ = 1.3 Hz, $J_{5,6B}$ = 4.1 Hz, $J_{5,6A}$ = 8.1 Hz, 1H, H-5'), 3.66 (dd, $J_{1,2}$ = 3.3 Hz, $J_{2,3}$ = 9.8 Hz, 1H, H-2'), 3.53 - 3.51 (br, 1H, -OC$\boldsymbol{H_2}$(CH_2)$_5$N-), 3.48 (dd, $J_{3,4}$ = 3.3 Hz, $J_{2,3}$ = 9.8 Hz, 1H, H-3'), 3.20 (dd, $J_{4,5}$ = 1.5 Hz, $J_{3,4}$ = 3.3 Hz, 1H, H-4'), 3.12 - 3.04 (br, 2H, -O(CH_2)$_5$C$\boldsymbol{H_2}$N-), 2.77 (t, J = 6.1 Hz, 2H, -C$\boldsymbol{H_2}$-(Lev)), 2.62 - 2.58 (m, 2H, -C$\boldsymbol{H_2}$-(Lev)), 2.15 (s, 3H, -C$\boldsymbol{H_3}$ (Lev)), 1.50 - 1.50 (br, 2H, -(C$\boldsymbol{H_2}$)$_4$-), 1.31 - 1.31 (br, 2H, -(C$\boldsymbol{H_2}$)$_4$-), 1.20 - 1.07 (br, 4H, -(C$\boldsymbol{H_2}$)$_4$-), H_2O/4'-O***H*** exchange (δ 2.02). ^{13}C{^{1}H} NMR (126 MHz, $CDCl_3$): δ , ppm 206.6 (R**C**OR (Lev)), 172.4 (R**C**OOR (Lev)), 166.2 (**C**=O (6-O-Bz)), 166.1 (**C**=O (4-O-Bz)), 164.9 (**C**=O (2-O-Bz)), 138.4, 138, 133.4, 133.3, 133.3, 130.3, 129.9, 129.8, 129.7, 129.3, 128.6, 128.6, 128.5, 128.4, 128.1, 128, 127.9, 127.8, 127.6, 127.2, 101.7 (C-1(Galβ)), 93.4 (C-1'(Galα)), 76.8 (C-3'), 74.4 (C-2'), 72.8 (O-**C**H_2-Ph), 72.4 (O-**C**H_2-Ph), 72.3 (C-3), 71.4 (C-5), 71.1 (C-2), 70.2 (-O**C**H_2(CH_2)$_5$N-), 68.0 (C-5'), 67.4 (C-4'), 67.2 (-**C**H_2-(Cbz)), 65.9 (C-4), 63.6 (C-6'), 62.4 (C-6), 50.5 (-**C**H_2-(NBn)), 50.2 (-**C**H_2-(NBn)), 47.1 (-O(CH_2)$_5$**C**H_2N-), 46.2 (-O(CH_2)$_5$**C**H_2N-), 37.8 (-**C**H_2- (Lev)), 29.9 (-**C**H_3 (Lev)), 29.5 (-(**C**H_2)$_4$-), 28.0 (-**C**H_2- (Lev)), 26.5 (-(**C**H_2)$_4$-), 25.7 (-(**C**H_2)$_4$-). *Note: Cis-trans isomerism of the Cbz carbamate moiety results in split ^{13}C signal for N-**C**H_2-Ph; N-**C**H_2(CH_5)O-; (**C**H_2)$_4$. Broadening of corresponding -C**H**$_2$- groups in ^{1}H-NMR. The ^{13}C signal of **C**=O (Cbz) (δ 156.5) did not appear in ^{13}C-NMR and was confirmed with additional information from ^{1}H-^{13}C HMBC.*

N-benzyl-N-benzyloxycarbonyl-6-aminohexyl 2-O-benzoyl-4,6-di-O-benzyl-3-O-(2-naphthalenylmethyl)-β-D-galactopyranosyl-(1→4)-2,3-di-O-benzyl-6-O-levulinoyl-α-D-galactopyranosyl-(1→3)-2,4,6-tri-O-benzoyl-β-D-galactopyranoside (32)

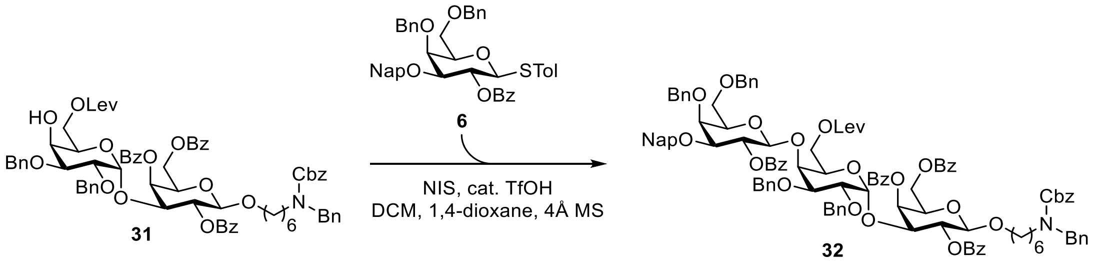

Glycosyl acceptor **31** (21 mg, 0.017 mmol) and glycosyl donor **6** (16 mg, 0.023 mmol) were co-evaporated from anhydrous toluene and dried overnight under high vacuum. The donor, acceptor, and freshly MW-activated and crushed molecular sieves (4Å, ~0.3 g) were suspended in anhydrous DCM (0.7 ml) and stirred at RT for 40 min, then cooled to -30°C in an acetone bath with an immersion cooler. Activator solution (0.22 ml, anhydrous DCM and anhydrous 1,4-dioxane (4:1)) containing recrystallized NIS (7.3 mg, 0.032 mmol) and TfOH (0.59 µl, 6.6 µmol) was added dropwise. The reaction mixture was stirred at -30°C for 15 min, after which the reaction mixture was allowed to reach -20°C over 1 h. Another portion of the activator solution (0.11 ml, anhydrous DCM and anhydrous 1,4-dioxane (4:1)) containing recrystallized NIS (3.7 mg, 0.016 mmol) and TfOH (0.30 µl, 3.3 µmol) was added and the reaction mixture was stirred for an additional 40 min at -10°C. The reaction mixture was filtered through celite, directly onto aqueous $Na_2S_2O_3$ (10% w/w, ~5 ml) and sat. $NaHCO_3$ (~5 ml). The celite was washed with DCM (~20 ml) and the combined filtrate was washed with an additional portion of aqueous $Na_2S_2O_3$ (10% w/w, ~3 ml) and sat. $NaHCO_3$ (~5 ml). The layers were separated and the aqueous layer was extracted with DCM (5 ml). The combined organic phase was washed with brine, dried over Na_2SO_4, then filtered and concentrated under reduced pressure. Compound **32** was purified by Prep-HPLC (16.7 mg, 54% yield, elution at 100% ACN). HRMS (ESI-QTOF) m/z: $[M+H]^+$ Calcd for $C_{111}H_{112}NO_{24}$ 1842.7569; found 1842.7655. ^{1}H NMR (500 MHz,

$CDCl_3$): δ, ppm 8.03 (dd, J = 1.1, 8.2 Hz, 2H), 7.98 (d, J = 7.3 Hz, 2H), 7.91 (dd, J = 1.1, 8.2 Hz, 2H), 7.77 - 7.71 (m, 3H), 7.57 - 7.53 (m, 3H), 7.50 - 7.27 (m, 26H), 7.25 - 7.17 (m, 5H), 7.13 - 7.13 (m, 1H), 7.06 (t, J = 7.8 Hz, 2H), 7.01 - 6.97 (m, 2H), 6.95 - 6.92 (m, 4H), 6.88 (t, J = 7.6 Hz, 2H), 6.54 (d, J = 7.3 Hz, 2H), 5.89 (d, $J_{3,4}$ = 2.6 Hz, 1H, H-4), 5.59 (dd, $J_{1,2}$ = 8.0 Hz, $J_{2,3}$ = 10.1 Hz, 1H, H-2), 5.49 (dd, $J_{1,2}$ = 8.0 Hz, $J_{2,3}$ = 10.0 Hz, 1H, H-2"), 5.16 (d, $J_{1,2}$ = 3.3 Hz, 1H, H-1'(Galα)), 5.15 - 5.13 (m, 2H, -C***H***$_2$-(Cbz)), 4.98, 4.64 (ABq, $\Delta\delta_{AB}$ = 0.34, J_{AB} = 11.7 Hz, 1H, O-C***H***$_2$-Ph), 4.75 (d, $J_{1,2}$ = 8.2 Hz, 1H, H-1(Galβ)), 4.72, 4.55 (ABq, ΔδAB = 0.17, J_{AB} = 12.6 Hz, 2H, O-C***H***$_2$-Ph), 4.59 - 4.55 (m, 1H, H-6A), 4.49 (d, $J_{1,2}$ = 7.9 Hz, 1H, H-1"(Galβ)), 4.45 (s, 2H, O-C***H***$_2$-Ar), 4.43 - 4.40 (br, 2H, -C***H***$_2$-(NBn)), 4.28 (dd, J_{AB} = 11.2 Hz, $J_{5,6}$ = 6.6 Hz, 1H, H-6B), 4.25, 4.06 (ABq, $\Delta\delta_{AB}$ = 0.19, J_{AB} = 11.5 Hz, 2H, O-C***H***$_2$-Ar), 4.22 - 4.17 (m, 2H, H-3; H-5), 4.13 (d, $J_{5,6}$ = 5.3 Hz, 2H, H-6'), 3.96 - 3.91 (m, 2H, H-4"; O-C***H***$_A$H$_B$-Ar), 3.88 - 3.87 (br, 1H, -OC***H***$_2$(CH$_2$)$_5$N-), 3.81 (t, $J_{5,6}$ = 5.4 Hz, 1H, H-5'), 3.60 (dd, J_{AB} = 8.9 Hz, $J_{5,6}$ = 7.5 Hz, 1H, H-6"A), 3.53 - 3.45 (m, 4H, O-CH$_A$***H***$_B$-Ar; -OC***H***$_2$(CH$_2$)$_5$N-; H-6"B; H-3"), 3.41 (dd, $J_{1,2}$ = 3.1 Hz, $J_{2,3}$ = 9.5 Hz, 1H, H-2'), 3.39 - 3.31 (m, 3H, H-3'; H-5"; H-4'), 3.14 – 2.99 (br, 2H, -O(CH$_2$)$_5$C***H***$_2$N-), 2.72 (br, 2H, -C***H***$_2$-(Lev)), 2.61 (br, 2H, -C***H***$_2$-(Lev)), 2.13 (s, 3H, -C***H***$_3$ (Lev)), 1.47 - 1.45 (br, 2H, -(C***H***$_2$)$_4$-), 1.29 - 1.28 (br, 2H, -(C***H***$_2$)$_4$-), 1.16 - 1.04 (br, 4H, -(C***H***$_2$)$_4$-). $^{13}C\{^1H\}$ NMR (126 MHz, $CDCl_3$): δ, ppm 206.8 (R**C**OR (Lev)), 172.3 (R**C**OR (Lev)), 166.2 (**C**=O (6-O-Bz)), 165.8 (**C**=O (4-O-Bz)), 165.4 (**C**=O (2"-O-Bz)), 165.0 (**C**=O (2-O-Bz)), 156.9 (**C**=O (Cbz)), 156.3 (**C**=O (Cbz)), 138.7, 138.6, 138.5, 138.0, 136.8, 135.1, 133.2, 133.2, 133.1, 133.0, 132.7, 130.1, 130.1, 129.9, 129.8, 129.7, 129.1, 128.6, 128.5, 128.3, 128.2, 128.1, 128.0, 128.0, 127.9, 127.7, 127.7, 127.6, 127.3, 126.7, 126.6, 126.5, 126.1, 125.9, 125.8, 101.8 (C-1(Galβ)), 101.7 (C-1"(Galβ)), 93.8 (C-1'(Galα)), 79.4 (C-3"), 77.4, 76.5 (C-2), 74.6 (O-**C**H$_2$-Ar), 74.4, 73.6 (O-**C**H$_2$-Ar), 73.4, 73.3 (O-**C**H$_2$-Ar), 72.8 (O-**C**H$_2$-Ar), 72.6 (C-4"), 72.3, 71.7 (C-2"), 71.6 (O-**C**H$_2$-Ar), 71.3, 71.1 (C-2), 70.1 (-O**C**H$_2$(CH$_2$)$_5$N-), 68.8 (C-5'; H-6"), 67.3 (-**C**H$_2$-(Cbz)), 66.1 (C-4), 64.8 (C-6'), 62.2 (C-6), 50.4 (-**C**H$_2$-(NBn)), 50.1 (-**C**H$_2$-(NBn)), 47.1 (-O(CH$_2$)$_5$CH$_2$N-), 46.2 (-O(CH$_2$)$_5$**C**H$_2$N-), 37.8 (-**C**H$_2$- (Lev)), 30.0 (-**C**H$_3$ (Lev)), 29.4 (-(**C**H$_2$)$_4$-), 28.1 (-**C**H$_2$- (Lev)), 27.9 (-(**C**H$_2$)$_4$-), 27.5 (-(**C**H$_2$)$_4$-), 26.5 (-(**C**H$_2$)$_4$-), 25.6 (-(**C**H$_2$)$_4$-). *Note: Cis-trans isomerism of the Cbz carbamate moiety results in split ^{13}C signal for **C**=O (Cbz); N-CH$_2$-Ph; N-**C**H$_2$(CH$_5$)O-; (**C**H$_2$)$_4$. Broadening of corresponding -C**H$_2$**- groups in ^1H-NMR. Note 2: The configuration of the anomeric carbons C-1, C-1' and C-1" was assigned with additional information from non-decoupled ^1H-^{13}C HSQC (vide infra, ^1H-^{13}C non-decoupled HSQC spectrum **32**): 101.8 (d, J=160Hz, C-1 (β)), 101.7 (d, J=162Hz, C-1" (β)), 93.8 (d, J=172Hz, C-1' (α)).*

N-benzyl-N-benzyloxycarbonyl-6-aminohexyl 2-O-benzoyl-4,6-di-O-benzyl-3-O-(2-naphthalenylmethyl)-β-D-galactopyranosyl-(1→4)-2,3-di-O-benzyl-α-D-galactopyranosyl-(1→3)-2,4,6-tri-O-benzoyl-β-D-galactopyranoside (33)

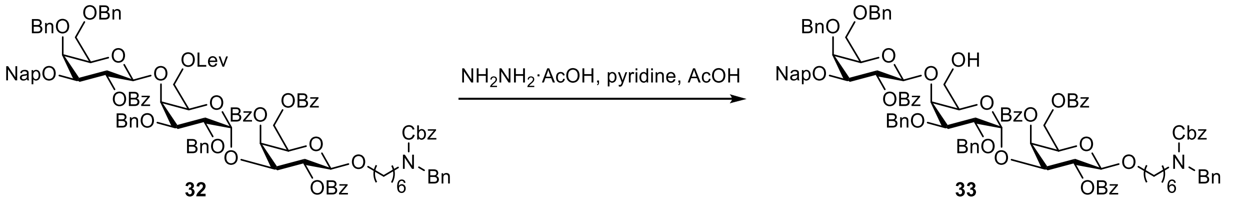

Compound **32** (18.5 mg, 10.0 μmol) was dissolved in a pyridine/AcOH buffer (4:1, 0.5 ml). Hydrazine acetate (NH_2NH_2·AcOH) (7.1 mg, 77 μmol) was separately dissolved in pyridine/AcOH (4:1, 0.5 ml), two drops of TDW and sonication was needed to completely dissolve the solids. The solution of NH_2NH_2·AcOH in pyridine/AcOH was added dropwise to **32**. The reaction mixture was stirred at RT for 20 min. The reaction mixture was diluted with DCM (~20 ml) and washed with aqueous HCl (0.1 M, ~30 ml). The layers were separated and the aqueous phase was acidified to pH~1, then extracted with DCM (10 ml). The combined organic phase was washed with sat. $NaHCO_3$, then brine. The organic phase was dried over Na_2SO_4, filtered, and concentrated under reduced pressure. Compound **33** was purified by Prep-HPLC (16.0 mg, 91% yield, elution at 95% ACN). HRMS (ESI-QTOF) m/z: $[M+H]^+$ Calcd for $C_{106}H_{106}NO_{22}$ 1744.7201; found

1744.7124. ^{1}H NMR (500 MHz, $CDCl_3$): δ, ppm 8.04 - 7.94 (m, 6H), 7.75 (d, J = 8.2 Hz, 3H), 7.58 - 7.40 (m, 11H), 7.35 - 7.27 (m, 23H), 7.26 - 7.19 (m, 4H), 7.13 - 7.13 (m, 1H), 7.08 - 7.02 (m, 4H), 6.96 - 6.93 (m, 6H), 6.63 (d, J = 7.5 Hz, 2H), 5.81 (d, $J_{3,4}$ = 1.9 Hz, 1H, H-4), 5.62 - 5.56 (m, 2H, H-2; H-2"), 5.14 (s, 2H, -C***H***$_2$-(Cbz)), 4.99, 4.60 (ABq, $\Delta\delta_{AB}$ = 0.39, J_{AB} = 11.8 Hz, 2H, O-C***H***$_2$-Ar), 4.96 (br, 1H, H-1'(Galα)), 4.74, 4.59 (ABq, $\Delta\delta_{AB}$ = 0.15, J_{AB} = 12.6 Hz, 2H, O-C***H***$_2$-Ar), 4.56 - 4.53 (m ,2H, H-1(Galβ), H-6A), 4.52, 4.35 (ABq, $\Delta\delta_{AB}$ = 0.17, J_{AB} = 11.8 Hz, 2H, O-C***H***$_2$-Ar), 4.48 (d, $J_{1,2}$ = 8.0 Hz, 1H, H-1"(Galβ)), 4.42 - 4.38 (br, 2H, -C***H***$_2$-(NBn)), 4.32 (dd, J_{AB} = 11.2 Hz, $J_{5,6B}$ = 5.9 Hz, 1H, H-6B), 4.20, 4.05 (ABq, $\Delta\delta_{AB}$ = 0.15, J_{AB} = 11.4 Hz, 2H, O-C***H***$_2$-Ar), 4.03 - 3.96 (m, 2H, H-5; H-3), 3.85 - 3.85 (br, 1H, -OC***H***$_2$(CH_2)$_5$N-), 3.82 (d, $J_{3,4}$ = 2.4 Hz, 1H, H-4"), 3.82, 3.63 (ABq, $\Delta\delta_{AB}$ = 0.19, J_{AB} = 11.4 Hz, 2H, O-C***H***$_2$-Ar), 3.73 - 3.70 (m, 2H, H-5'; H-6'A), 3.67 – 3.58 (m, 2H, H-6"A; H-4'), 3.52 (dd, $J_{3,4}$ = 2.6, $J_{2,3}$ = 10.1 Hz, 1H, H-3"), 3.48 - 3.44 (m, 1H, H-5"), 3.42 - 3.41 (br, 1H, -OC***H***$_2$(CH_2)$_5$N-), 3.41 - 3.36 (m, 4H, H-6'B; H-6"B; H-3'; H-2'), 3.10 - 3.01 (br, 2H, -O(CH_2)$_5$C***H***$_2$N-), 1.45 - 1.44 (m, 2H, -(C***H***$_2$)$_4$-), 1.27 - 1.24 (m, 2H, -(C***H***$_2$)$_4$-), 1.14 - 1.00 (m, 4H, -(C***H***$_2$)$_4$-), H_2O/6'-OH exchange (δ 3.25), residual acetone (~1:0.02, δ 2.18). ^{13}C{^{1}H} NMR (126 MHz, $CDCl_3$): δ, ppm 166.2 (**C**=O (6-O-Bz)), 165.7 (**C**=O (4-O-Bz)), 165.4 (**C**=O (2/2'-O-Bz)), 165.1 (**C**=O (2/2'-O-Bz)), 156.8 (**C**=O (Cbz)), 156.2 (**C**=O (Cbz)), 138.8, 138.7, 138.2, 137.9, 137.5, 136.9, 134.9, 133.2, 133.2, 133.1, 133.1, 132.8, 130.1, 130.0, 129.8, 129.8, 129.7, 129.7, 129.3, 128.6, 128.5, 128.4, 128.4, 128.3, 128.3, 128.2, 128.1, 128.1, 128.0, 127.9, 127.8, 127.7, 127.7, 127.6, 127.3, 127.0, 126.7, 126.7, 126.2, 126.1, 125.8, 102.5 (C-1"(Galβ)), 101.9 (C-1(Galβ)), 96.1 (C-1'(Galα)), 79.3 (C-3"), 77.4 (C-2'/3'), 76.8 (C-2'/3'), 74.5 (O-**C**H_2-Ar), 74.5 (C-4'), 74.3 (C-3), 73.8 (C-5"), 73.7 (O-**C**H_2-Ar), 73.4 (O-**C**H_2-Ar), 73.0 (O-**C**H_2-Ar), 72.6 (C-4"), 72.0 (O-**C**H_2-Ar), 71.6 (C-5), 71.4 (C-2/2"), 71.2 (C-2/2"), 70.2 (-O**C**H_2(CH_2)$_5$N-), 69.9 (C-5'), 69.2 (C-6"), 67.2 (-**C**H_2-(Cbz)), 67.0 (C-4), 62.5 (C-6), 59.5 (C-6'), 50.4 (-**C**H_2-(NBn)), 50.1 (-**C**H_2-(NBn)), 47.1 (-O(CH_2)$_5$**C**H_2N-), 46.1 (-O(CH_2)$_5$**C**H_2N-), 29.3 (-(**C**H_2)$_4$-), 27.9 (-(**C**H_2)$_4$-), 27.5 (-(**C**H_2)$_4$-), 26.4 (-(**C**H_2)$_4$-), 25.6 (-(**C**H_2)$_4$-). *Note: Cis-trans isomerism of the Cbz carbamate moiety results in split ^{13}C signal for **C**=O (Cbz); N-**C**H_2-Ph; N-**C**H_2(CH_5)O-; (**C**H_2)$_4$. Broadening of corresponding -C**H**$_2$- groups in ^{1}H-NMR. Note 2: The configuration of the anomeric carbons C-1, C-1'and C-1" was assigned with additional information from non-decoupled ^{1}H-^{13}C HSQC (vide infra, ^{1}H-^{13}C non-decoupled HSQC spectrum **33**): 102.5 (d, J=162Hz, C-1" (β)), 101.9 (d, J=159Hz, C-1 (β)), 96.1 (d, J=171Hz, C-1' (α)).*

N-benzyl-N-benzyloxycarbonyl-6-aminohexyl 2-O-benzoyl-4,6-di-O-benzyl-3-O-(2-naphthalenylmethyl)-β-D-galactopyranosyl-(1→4)-2,3-di-O-benzyl-6-O-sulfo-α-D-galactopyranosyl-(1→3)-2,4,6-tri-O-benzoyl-β-D-galactopyranoside (34)

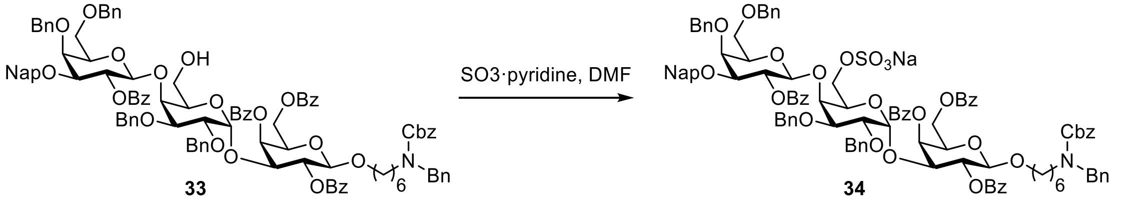

Compound **33** (29.2 mg, 16.7 µmol) and SO_3·pyridine (25.7 mg, 161 µmol) were dried under high vacuum overnight. The mixture was dissolved in anhydrous DMF (1.2 ml). The reaction mixture was stirred overnight at RT, at which point the reaction was complete (as indicated by TLC, 5% MeOH in DCM, R_f=0.28). The reaction was quenched by the addition of MeOH (~3 ml) and concentrated under reduced pressure. The crude product was purified by flash chromatography (elution at 5% MeOH in DCM) and Dowex 50W X8 ion exchange resin (Na-form) was added directly to the eluted fraction. The fraction was filtered and the filtrate evaporated under reduced pressure to obtain a yellowish residue. The residue was mostly dissolved in 5% MeOH in DCM and filtered through a cotton plug. The filtrate was evaporated to obtain pure **34** as a white solid (25.8 mg, 83% yield). HRMS (ESI-QTOF) m/z: $[M-Na]^-$ Calcd for $C_{106}H_{104}NO_{25}S$ 1822.6624; found 1822.6534. ^{1}H NMR (500 MHz, d_7-DMF): δ, ppm 8.36 (dd, J = 3.0, 6.7 Hz, 2H), 8.03 (t, J = 4.5 Hz, 2H), 8.02 - 7.99 (m,

2H), 7.86 (d, J = 7.8 Hz, 1H), 7.78 (dd, J = 1.0, 8.1 Hz, 2H), 7.73 (s, 1H), 7.69 - 7.63 (m, 3H), 7.62 - 7.58 (m, 1H), 7.55 - 7.47 (m, 10H), 7.46 - 7.39 (m, 8H), 7.38 - 7.30 (m, 11H), 7.29 - 7.24 (m, 3H), 7.20 (t, J = 7.8 Hz, 2H), 7.16 - 7.10 (m, 5H), 7.05 (t, J = 7.4 Hz, 1H), 6.91 (t, J = 7.6 Hz, 2H), 6.53 (d, J = 7.3 Hz, 2H), 6.04 (d, $J_{3,4}$ = 2.8 Hz, 1H, H-4), 5.54 (dd, $J_{1,2}$ = 7.9 Hz, $J_{2,3}$ = 10.4 Hz, 1H, H-2), 5.46 (dd, $J_{1,2}$ = 8.0 Hz, $J_{2,3}$ = 10.2 Hz, 1H, H-2”), 5.29 (d, $J_{1,2}$ = 3.4 Hz, 1H, H-1’(Galα)), 5.17 (s, 2H, -C***H***2-Cbz), 5.13 (d, $J_{1,2}$ = 8.0 Hz, 1H, H-1(Galβ)), 4.97 (d, J_{AB} = 11.4 Hz, 1H, O-C***H***2-Ar), 4.90 (d, J_{AB} = 12.6 Hz, 1H, O-C***H***2-Ar), 4.89 (dd, $J_{3,4}$ = 3.0 Hz, $J_{2,3}$ = 10.5 Hz, 1H, H-3), 4.75 (d, $J_{1,2}$ = 8.0 Hz, 1H, H-1”(Galβ)), 4.74 (d, J_{AB} = 13.1 Hz, 1H, O-C***H***2-Ar), 4.72 (d, J_{AB} = 11.6 Hz, 1H, O-C***H***2-Ar), 4.65 (ABq, $\Delta\upsilon_{AB}$ = 38.9 Hz, J = 11.8 Hz, 2H, O-C***H***2-Ar), 4.53 (dd, $J_{5,6A}$ = 6.6 Hz, J_{AB} = 10.4 Hz, 1H, H-6A), 4.48 - 4.45 (m, 3H, -C***H***2-(NBn); H-5), 4.41 (dd, $J_{5,6B}$ = 5.5 Hz, J_{AB} = 10.5 Hz, 1H, H-6B), 4.35 (m, 1H, H-5’), 4.28 (dd, $J_{5,6}$ = 3.0 Hz, J_{AB} = 12.0 Hz, 1H, H-6’A), 4.28 - 4.27 (m, 1H, H-4”), 4.06 (dd, $J_{5,6}$ = 8.2 Hz, J_{AB} = 12.1 Hz, 1H, H-6’B), 3.97 (ABq, $\Delta\upsilon_{AB}$ = 40.3 Hz, J_{AB} = 11.3 Hz, 2H, O-C***H***2-Ar), 3.90 (dd, $J_{3,4}$ = 3.0 Hz, $J_{2,3}$ = 10.2 Hz, 1H, H-3”), 3.89 (d, J_{AB} = 11.0 Hz, 1H, O-C***H***2-Ar), 3.84 - 3.76 (m, 3H, H-6”A; H-5”; -OC***H***2(CH2)5N-), 3.73 (dd, $J_{5,6}$ = 4.7 Hz, J_{AB} = 8.3 Hz, 1H. H-6”B), 3.63 (s, 1H, H-4’), 3.55 (d, J_{AB} = 11.2 Hz, 1H, O-C***H***2-Ar), 3.51 (br, 1H, -OC***H***2(CH2)5N-), 3.43 (dd, $J_{1,2}$ = 3.3 Hz, $J_{2,3}$ = 10.0 Hz, 1H, H-2’), 3.22 (dd, $J_{3,4}$ = 2.6 Hz, $J_{2,3}$ = 10.0 Hz, 1H, H-3’), 3.10 - 3.09 (br, 2H, -O(CH2)5C***H***2N-), 1.43 (m, 2H, -(C***H***2)4-), 1.30 (m, 2H, -(C***H***2)4-), 1.14 - 1.14 (m, 4H, -(C***H***2)4-), grease (δ 1.50 - 1.00), H_2O (δ 3.51). $^{13}C\{^1H\}$ NMR (126 MHz, *d*7-DMF): δ , ppm 166.8 (**C**=O (Bz)), 166.6 (**C**=O (Bz)), 166.4 (**C**=O (Bz)), 166.2 (**C**=O (Bz)), 157.4 (**C**=O (Cbz)), 140.3, 140.1, 139.8, 139.7, 139.7, 138.6, 137.3, 134.6, 134.4, 134.3, 134.2, 134.0, 133.9, 131.5, 131.5, 130.9, 130.9, 130.8, 130.7, 130.6, 130.1, 129.9, 129.8, 129.6, 129.5, 129.5, 129.3, 129.2, 129.1, 129.0, 129.0, 128.9, 128.8, 128.7, 128.6, 128.6, 128.5, 128.4, 128.2, 127.8, 127.2, 127.2, 127.1, 127.0, 126.9, 102.6 (C-1”(Galβ)), 102.4 (C-1(Galβ)), 93.0 (C-1’(Galα)), 80.9 (C-3”), 79.3 (C-3’), 77.8 (C-2’), 75.7 (O-**C**H2-Ar), 75.2 (C-4’), 74.9 (C-4”), 74.5 (C-5”), 74.2 (O-**C**H2-Ar), 73.6 (O-**C**H2-Ar), 73.3 (O-**C**H2-Ar), 73.1 (C-2”), 72.3 (O-**C**H2-Ar; C-5), 71.7 (C-2), 71.2 (C-3), 71.1 (C-5), 70.5 (-O**C**H2(CH2)5N-), 70.4 (C-5’), 70.1 (C-6”), 68.0 (C-6’), 67.8, 67.6 (-**C**H2-(Cbz)), 63.8 (C-6), 51.1 (-**C**H2-(NBn)), 50.9 (-**C**H2-(NBn)), 50, 48.1 (-O(CH2)5**C**H2N-), 47.3 (-O(CH2)5**C**H2N-), 29.1, 28.6, 27.3, 26.5, 23.6, 14.7. *Note: The ^{1}H-NMR signals at δ 3.53 - 3.48 were masked by the H_2O signal (δ 3.51) and were assigned with additional information from ^{1}H-^{13}C HSQC. Note 2: Cis-trans isomerism of the Cbz carbamate moiety results in split ^{13}C signal for N-**C**H2-Ph; N-**C**H2(CH5)O-;* (**C**H2)4*. Broadening of corresponding -C**H**2- groups in ^{1}H-NMR. Note 3: The configuration of the anomeric carbons C-1, C-1', and C-1" was assigned with additional information from non-decoupled ^{1}H-^{13}C HSQC (vide infra, ^{1}H-^{13}C non-decoupled HSQC spectrum **34**): 102.6 (d, J=164Hz, C-1" (β)), 102.4 (d, J=163Hz, C-1 (β)), 93.0 (d, J=172Hz, C-1' (a)).*

N-benzyl-N-benzyloxycarbonyl-6-aminohexyl 3-O-acetyl-2-O-benzoyl-4,6-di-O-benzyl-β-D-galactopyranosyl-(1→4)-2,3-di-O-benzyl-α-D-galactopyranosyl-(1→3)-2,4,6-tri-O-benzoyl-β-D-galactopyranoside (35)

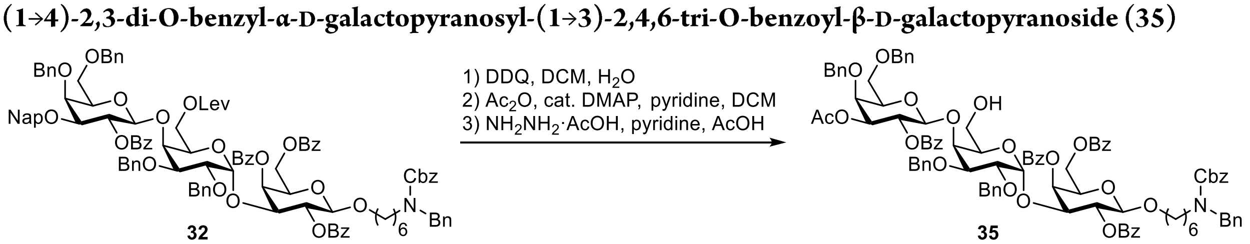

Compound **32** (12.0 mg, 6.51 μmol) was dissolved in DCM (0.9 ml) and TDW (0.1 ml). DDQ (3.0 mg, 0.013 mmol) was added. The reaction mixture obtained a green-yellow color. The reaction mixture was stirred for 3.5 h at RT, covered from light, until a major product was formed (as indicated by TLC, 40% EtOAc in hexane, R_f=0.35). The reaction was diluted with DCM (~20 ml) and washed with sat. $NaHCO_3$ (~60 ml). The aqueous layer had an intense red color, the organic phase was colorless. The aqueous layer was diluted with more sat. $NaHCO_3$ and extracted with DCM (5 ml). The combined organic layer was washed with brine, dried over Na_2SO_4, filtered and evaporated to produce the crude product as a yellowish

syrup. HRMS (ESI-QTOF) m/z: $[M+H]^+$ Calcd for $C_{100}H_{104}NO_{24}$ 1702.6943; found 1702.6935. The crude product was evaporated from anhydrous toluene and dried under high vacuum overnight. The crude product was dissolved in anhydrous DCM (0.8 ml) and cooled to 0°C in an ice-water bath. Acetic anhydride (Ac_2O) (6.9 mg, 0.068 mmol) was dissolved in anhydrous DCM (0.2 ml) and added dropwise to the reaction mixture. DMAP (~2 crystals) were dissolved in pyridine (0.5 ml) and added dropwise. The reaction mixture was stirred for 15 h at RT. The reaction was quenched by the addition of MeOH (~0.05 ml) and stirred for 1 h. The reaction mixture was diluted with DCM (~20 ml) and washed with aqueous HCl (0.2 M, ~20 ml). Small portions of aqueous HCl (0.2 M) were added until the aqueous phase registered pH~4. The layers were separated and the aqueous phase was acidified with aqueous HCl (1 M) and extracted with DCM (2x 5 ml). The combined organic layer was washed with sat. $NaHCO_3$ (~20 ml). The layers were separated and the aqueous phase was extracted with DCM (2x 5 ml). The combined organic layer was washed with brine, then dried over Na_2SO_4, filtered, and concentrated under reduced pressure to obtain the crude product. HRMS (ESI-QTOF) m/z: $[M+NH_4]^+$ Calcd for $C_{102}H_{109}N_2O_{25}$ 1761.7314; found 1761.7320. The crude product was dissolved in a pyridine/AcOH buffer (4:1, 0.5 ml). Hydrazine acetate (NH_2NH_2·AcOH) (6.0 mg, 0.065 mmol) was separately dissolved in pyridine/AcOH (4:1, 0.5 ml), a drop of TDW and sonication was needed to completely dissolve the solids. The solution of NH_2NH_2·AcOH in pyridine/AcOH was added dropwise. The reaction mixture was stirred at RT for 1 h. The reaction mixture was diluted with DCM (~30 ml) and washed with aqueous HCl (0.1 M, ~50 ml). Small portions of aqueous HCl (1 M) were added until the aqueous phase registered pH~3. The layers were separated and the aqueous phase was acidified to pH~1, then extracted with DCM (5 ml). The combined organic phase was washed with sat. $NaHCO_3$, then brine. The organic phase was dried over Na_2SO_4, filtered, and concentrated under reduced pressure. The crude product was evaporated from toluene to remove residual pyridine. Compound **35** was purified by Prep-HPLC (5.9 mg, 55% 3-step yield, elution at 91% ACN). HRMS (ESI-QTOF) m/z: $[M+Na]^+$ Calcd for $C_{97}H_{99}NO_{23}Na$ 1668.6500; found 1668.6499. ^{1}H NMR (700 MHz, $CDCl_3$): δ, ppm 8.03 (d, J = 7.3 Hz, 2H), 8.00 (t, J = 7.3 Hz, 2H), 7.95 (d, J = 7.3 Hz, 2H), 7.80 (d, J = 7.3 Hz, 2H), 7.55 (t, J = 7.4 Hz, 1H), 7.47 (t, J = 7.4 Hz, 1H), 7.45 - 7.40 (m, 3H), 7.38 - 7.27 (m, 21H), 7.26 - 7.22 (m, 6H), 7.20 (br d, J = 7.2 Hz, 1H), 7.13 (br d, J = 6.8 Hz, 1H), 7.08 (t, J = 7.8 Hz, 2H), 7.06 - 7.02 (m, 3H), 6.95 (t, J = 7.5 Hz, 2H), 6.65 (d, J = 7.4 Hz, 2H), 5.82 (d, $J_{3,4}$ = 1.9 Hz, 1H, H-4), 5.60 (dd, $J_{1,2}$ = 8.1 Hz, $J_{2,3}$ = 10.0 Hz, 1H, H-2), 5.55 (dd, $J_{1,2}$ = 8.0 Hz, $J_{2,3}$ = 10.5 Hz, 1H, H-2"), 5.14 (br d, J = 8.1 Hz, 2H, -C***H*₂**-(Cbz)), 4.97 (d, J = 2.4 Hz, 1H, H-1'(Galα)), 4.97 (dd, $J_{3,4}$ = 3.1 Hz, $J_{2,3}$ = 10.3 Hz, 1H, H-3"), 4.67, 4.41 (ABq, $\Delta\delta_{AB}$ = 0.26, J_{AB} = 11.8 Hz, 2H, O-C***H*₂**-Ph), 4.58 (d, J = 8.2 Hz, 1H, H-1"(Galβ)), 4.55 (m, 1H, H-1(Galβ)), 4.53, 4.35 (ABq, $\Delta\delta_{AB}$ = 0.18, J_{AB} = 11.8 Hz, 2H, O-C***H*₂**-Ph), 4.56 - 4.50 (m, 1H, H-6A), 4.39 (br d, J = 14.4 Hz, 2H, -C***H*₂**-(NBn)), 4.32 (dd, J_{AB} = 11.4 Hz, $J_{5,6}$ = 6.2 Hz, 1H, H-6B), 4.21, 4.10 (ABq, $\Delta\delta_{AB}$ = 0.11, J_{AB} = 11.5 Hz, 2H, O-C***H*₂**-Ph), 4.01 (t, $J_{5,6}$ = 6.7 Hz, 1H, H-5), 3.98 (dd, $J_{3,4}$ = 3.2 Hz, $J_{2,3}$ = 10.2 Hz, 1H, H-3), 3.87, 3.66 (ABq, $\Delta\delta_{AB}$ = 0.21, J_{AB} = 11.8 Hz, 2H, O-C***H*₂**-Ph), 3.88 - 3.81 (m, 2H, H-4"; -OC***H*₂**$(CH_2)_5$N-), 3.75 - 3.68 (m, 2H, H-5'; H-6'A), 3.66 - 3.64 (m, 1H, H-4'), 3.62 (dd, $J_{5,6A}$ = 4.8 Hz, $J_{5,6B}$ = 7.5 Hz, 1H, H-5"), 3.58 (dd, $J_{5,6}$ = 7.8 Hz, J_{AB} = 9.2 Hz, 1H, H-6"A), 3.45 - 3.42 (m, 1H, -OC***H*₂**$(CH_2)_5$N-), 3.44 - 3.40 (m, 1H, H-3"), 3.40 (dd, $J_{1,2}$ = 2.7 Hz, $J_{2,3}$ = 10.2 Hz, 1H, H-2"), 3.38 - 3.33 (m, 2H, H-6'B; H-6"B), 3.13 - 2.98 (br m, 2H, -O$(CH_2)_5$C***H*₂**N-), 1.87 (s, 3H, -C***H*₃**(Ac)), 1.49 - 1.36 (m, 2H, -(C***H*₂**)₄-), 1.29 - 1.22 (m, 2H, -(C***H*₂**)₄-), 1.15 - 0.96 (m, 4H, -(C***H*₂**)₄-), H_2O/6'-OH exchange (δ 1.96), grease (δ 1.32 - 1.19). $^{13}C\{^1H\}$ NMR (176 MHz, $CDCl_3$): δ, ppm 170.6 (**C**=O (3"-O-Ac)), 166.2 (**C**=O (6-O-Bz), 165.8, (**C**=O (4-O-Bz) 165.3 (**C**=O (2"-O-Bz), 165.1 (**C**=O (2-O-Bz), 156.8 (**C**=O (Cbz)), 156.2 (**C**=O (Cbz)), 138.8, 138.6, 137.9, 137.6, 137.4, 136.9, 133.3, 133.2, 133.0, 130.1, 130.0, 129.8, 129.8, 129.7, 129.6, 129.5, 129.2, 128.6, 128.6, 128.5, 128.4, 128.3, 128.2, 128.2, 128.1, 128.1, 128.1, 128.0, 127.9, 127.9, 127.8, 127.7, 127.5, 127.4, 127.2, 126.9, 126.7, 102.4 (C-1"(Galβ)), 101.9 (C-1(Galβ)), 96.0 (C-1'(Galα)), 77.4 (C-2'/C-3'), 76.8 (C-2'/C-3'), 75.1 (C-4'), 75.0 (O-**C**H_2-Ph), 74.2 (C-3), 74.0 (C-4), 73.7 (C-3"), 73.6 (O-**C**H_2-Ph; O-**C**H_2-Ph), 73.5 (C-5"), 73.0 (O-**C**H_2-Ph), 71.5 (C-5), 71.1 (C-2), 70.2 (-O**C**$H_2(CH_2)_5$N-), 70.0 (C-5'), 69.7 (C-2"), 68.7 (C-6"), 67.2 (-**C**H_2-(Cbz)), 67.1 (-**C**H_2-(Cbz)), 66.9 (C-4), 62.5 (C-6), 59.6 (C-6'), 50.4 (-**C**H_2-(NBn)), 50.0 (-**C**H_2-(NBn)), 47.0 (-O$(CH_2)_5$**C**H_2N-), 46.1 (-O$(CH_2)_5$**C**H_2N-), 29.8 (-(**C**H_2)₄-), 29.3 (-(**C**H_2)₄-), 27.9 (-(**C**H_2)₄-), 27.5

(-(CH_2)$_4$-), 26.4 (-(CH_2)$_4$-), 25.6 (-(CH_2)$_4$-), 20.9 (-CH_3(Ac)). *Note: Cis-trans isomerism of the Cbz carbamate moiety results in split ^{13}C signal for **C**=O (Cbz); O-**C**H_2-Ph (Cbz); N-**C**H_2-Ph; N-**C**H_2(CH_5)O-; (**C**H_2)$_4$. Broadening of corresponding -C**H**$_2$- groups in ^{1}H-NMR. Note 2: The configuration of the anomeric carbons C-1, C-1'and C-1" was assigned with additional information from non-decoupled ^{1}H-^{13}C HSQC (vide infra, ^{1}H-^{13}C non-decoupled HSQC spectrum **35**): 102.4 (d, J=163Hz, C-1" (β)), 101.9 (d, J=159Hz, C-1 (β)), 96.0 (d, J=172Hz, C-1' (α)).*

N-benzyl-N-benzyloxycarbonyl-6-aminohexyl 3-O-acetyl-2-O-benzoyl-4,6-di-O-benzyl-β-D-galactopyranosyl-(1→4)-2,3-di-O-benzyl-6-O-sulfo-α-D-galactopyranosyl-(1→3)-2,4,6-tri-O-benzoyl-β-D-galactopyranoside (36)

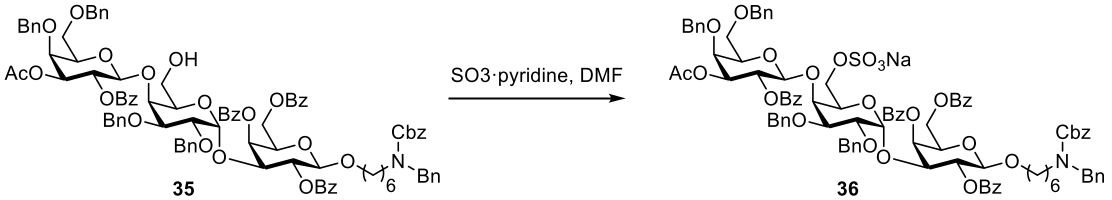

Compound **35** (8.1 mg, 4.9 μmol) and SO_3·pyridine (7.8 mg, 0.049 mmol) were dried under high vacuum overnight. The mixture was dissolved in anhydrous DMF (0.9 ml). The reaction mixture was stirred overnight at RT, at which point the reaction was complete (as indicated by TLC, 5% MeOH in DCM, R_f=0.22). The reaction was quenched by the addition of MeOH (~3 ml) and concentrated under reduced pressure. The crude product was purified by flash chromatography (elution at 6% MeOH in DCM) and Dowex 50W X8 ion exchange resin (Na-form) was added directly to the eluted fraction. The fraction was filtered and the filtrate evaporated under reduced pressure to obtain a yellowish residue. The residue was mostly dissolved in 7% MeOH in DCM and filtered through a cotton plug. The filtrate was evaporated to obtain pure **36** as a white solid (6.4 mg, 74% yield). HRMS (ESI-QTOF) m/z: [M-Na]$^-$ Calcd for $C_{97}H_{98}NO_{26}S$ 1724.6103; found 1724.6041. ^{1}H NMR (700 MHz, DMF): δ, ppm 8.36 (dd, *J* = 3.0, 6.6 Hz, 2H), 8.04 (dd, *J* = 1.3, 8.4 Hz, 2H), 8.00 (dd, *J* = 1.2, 8.2 Hz, 2H), 7.79 (dd, *J* = 1.2, 8.3 Hz, 2H), 7.68 - 7.64 (m, 2H), 7.54 - 7.49 (m, 9H), 7.44 - 7.37 (m, 9H), 7.35 - 7.24 (m, 13H), 7.21 - 7.17 (m, 4H), 7.05 (t, *J* = 7.4 Hz, 1H), 6.92 (t, *J* = 7.7 Hz, 2H), 6.56 (d, *J* = 7.5 Hz, 2H), 6.04 (d, $J_{3,4}$ = 2.8 Hz, 1H, H-4), 5.54 (dd, $J_{1,2}$ = 7.9 Hz, $J_{2,3}$ = 10.5 Hz, 1H, H-2), 5.42 (dd, $J_{1,2}$ = 7.9 Hz, $J_{2,3}$ = 10.5 Hz, 1H, H-2"), 5.30 (d, $J_{1,2}$ = 3.4 Hz, 1H, H-1'(Galα)), 5.18 (dd, $J_{3,4}$ = 3.2 Hz, $J_{2,3}$ = 10.6 Hz, 1H, H-3"), 5.17 - 5.15 (br, 2H, -C***H***$_2$-Cbz), 5.13 (d, $J_{1,2}$ = 7.9 Hz, 1H, H-1(Galβ)), 4.89 (d, $J_{1,2}$ = 8.0 Hz, 1H, H-1"(Galβ)), 4.88 (dd, $J_{3,4}$ = 3.2 Hz, $J_{2,3}$ = 10.7 Hz, 1H, H-3), 4.74, 4.63 (ABq, $\Delta\delta_{AB}$ = 0.11, J_{AB} = 11.4 Hz, 2H, O-C***H***$_2$-Ph), 4.68, 4.61 (ABq, $\Delta\delta_{AB}$ = 0.07, J_{AB} = 11.8 Hz, 2H, O-C***H***$_2$-Ph), 4.53 (dd, J_{AB} = 10.7 Hz, $J_{5,6}$ = 6.8 Hz, 1H, H-6A), 4.50 - 4.45 (m, 3H, H-5; -C***H***$_2$-(NBn)), 4.41 (dd, J_{AB} = 10.7 Hz, $J_{5,6}$ = 5.6 Hz, 1H, H-6B), 4.35 (dd, $J_{5,6A}$ = 2.4 Hz, $J_{5,6B}$ = 8.2 Hz, 1H, H-5'), 4.27 (dd, $J_{5,6}$ = 3.1 Hz, J_{AB} = 11.9 Hz, 1H, H-6'A), 4.10, 3.95 (ABq, $\Delta\delta_{AB}$ = 0.15, J_{AB} = 11.5 Hz, 2H, O-C***H***$_2$-Ph), 4.08 - 4.03 (m, 2H, H-4"; H-6'B), 3.95 - 3.92 (m, 1H, H-5"), 3.92, 3.56 (ABq, $\Delta\delta_{AB}$ = 0.36, J_{AB} = 11.0 Hz, 2H, O-C***H***$_2$-Ph), 3.81 (dd, $J_{5,6}$ = 7.4 Hz, J_{AB} = 9.4 Hz, 1H, H-6"A), 3.79 - 3.79 (br, 1H, -OC***H***$_2$(CH_2)$_5$N-), 3.73 (dd, $J_{5,6}$ = 5.6 Hz, J_{AB} = 9.4 Hz, 1H, H-6"B), 3.68 (d, $J_{3,4}$ = 1.8 Hz, 1H, H-4'), 3.56 - 3.54 (br, 1H, -OC***H***$_2$(CH_2)$_5$N-), 3.42 (dd, $J_{1,2}$ = 3.4 Hz, $J_{2,3}$ = 10.0 Hz, 1H, H-2'), 3.24 (dd, $J_{3,4}$ = 2.6 Hz, $J_{2,3}$ = 10.0 Hz, 1H, H-3'), 3.13 - 3.09 (br, 2H, -O(CH_2)$_5$C***H***$_2$N-), 1.89 (s, 3H, -C***H***$_3$(Ac)), 1.43 - 1.42 (m, 2H, -(C***H***$_2$)$_4$-), 1.34 - 1.27 (m, 2H, -(C***H***$_2$)$_4$-), 1.17 - 1.12 (m, 2H, -(C***H***$_2$)$_4$-), 1.08 - 1.03 (m, 2H, -(C***H***$_2$)$_4$-), grease (δ 1.50 - 1.00), H_2O (δ 3.52). ^{13}C{^{1}H} NMR (176 MHz, *d*$_7$-DMF): δ, ppm 171.0 (**C**=O (3"-O-Ac)), 166.8 (**C**=O (6-O-Bz), 166.6 (**C**=O (4-O-Bz), 166.3 (**C**=O (2"-O-Bz), 166.2 (**C**=O (2-O-Bz), 157.4 (**C**=O (Cbz)), 156.7 (**C**=O (Cbz)), 139.9, 139.9, 139.8, 139.8, 139.7, 138.6, 134.6, 134.4, 134.3, 134.1, 131.5, 131.0, 130.9, 130.7, 130.6, 130.1, 130.0, 129.9, 129.8, 129.6, 129.5, 129.4, 129.3, 129.3, 129.3, 129.1, 129.0, 128.9, 128.9, 128.8, 128.8, 128.6, 128.5, 128.2, 127.8, 127.1, 102.4 (C-1(Galβ)), 102.4 (C-1"(Galβ)), 93.0 (C-1'(Galα)), 79.3 (C-3'), 77.7 (C-2'), 76.0 (O-**C**H_2-Ph), 76.0 (C-4"), 75.6 (C-4'), 74.5 (C-3"), 74.2 (O-**C**H_2-Ph), 74.1 (C-5"), 73.5 (O-**C**H_2-Ph), 73.3 (O-**C**H_2-Ph), 72.4 (C-5), 71.7 (C-2), 71.7 (C-2"), 71.1 (C-3), 70.5 (-O**C**H_2(CH_2)$_5$N-), 70.3 (C-5'), 69.4 (C-6"), 67.9 (C-6'), 67.8 (C-4), 67.6 (-**C**H_2-

(Cbz)), 63.8 (C-6), 51.2 (-**C**H_2-(NBn)), 50.9 (-**C**H_2-(NBn)), 48.1 (-O(CH_2)$_5$**C**H_2N-), 47.4 (-O(CH_2)$_5$**C**H_2N-), 32.9, 29.1, 28.6, 27.3, 26.7, 26.5, 25.9, 23.6, 21.1 (-**C**H_3(Ac)), 14.7. *Note: The ^{1}H-NMR signals at δ 3.60 - 3.50 were masked by the H_2O signal (δ 3.52) and were assigned with additional information from ^{1}H-^{13}C HSQC. Note 2: Cis-trans isomerism of the Cbz carbamate moiety results in split ^{13}C signal for **C**=O (Cbz); O-**C**H_2-Ph (Cbz); N-**C**H_2-Ph; N-**C**H_2(CH_5)O-; (**C**H_2)$_4$. Broadening of corresponding -C**H_2**- groups in ^{1}H-NMR. Note 3: The configuration of the anomeric carbons C-1, C-1'and C-1" was assigned with additional information from non-decoupled ^{1}H-^{13}C HSQC (vide infra, ^{1}H-^{13}C non-decoupled HSQC spectrum **36**): 102.4 (d, J=162Hz, C-1 (β)), 102.4 (d, J=162Hz, C-1" (β)), 93.0 (d, J=172Hz, C-1' (α)).*

6-Aminohexyl β-D-galactopyranosyl-(1→4)-6-O-sulfo-α-D-galactopyranosyl-(1→3)-β-D-galactopyranoside (mSt)

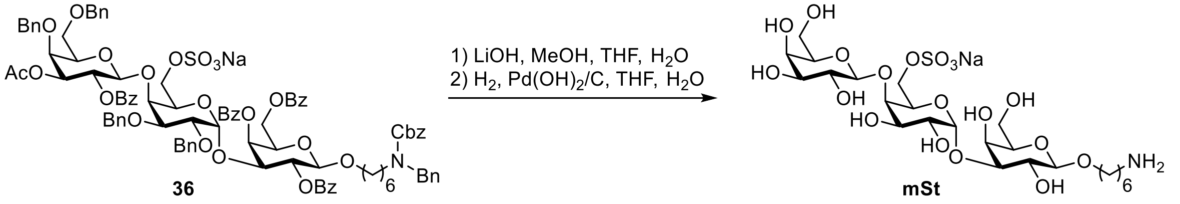

Compound **36** (5.8 mg, 3.3 μmol) was dissolved in THF (0.3 ml) and MeOH (0.3 ml), and cooled to 0°C in an ice-water bath. Aqueous lithium hydroxide (LiOH) (2 M, 0.8 ml) was added dropwise. The reaction mixture was stirred at RT for 21 h until completion (as indicated by TLC, 10% MeOH in DCM, R_f=0.24). Ion exchange beads Amberlight® XAD16N (H-form, pre-washed in DCM and MeOH) were added in small portions until neutral pH was reached and reaction mixture turned clear. The reaction mixture was filtered and Dowex 50W X8 ion exchange resin (Na-form) was added to the filtrate. The mixture was filtered again, and the filtrate was concentrated under reduced pressure. The residue was mostly dissolved in 15% MeOH in DCM and filtered through a cotton plug. The filtrate was evaporated to obtain a white solid. The crude product was dissolved in a minimal amount of ACN and TDW (50% ACN, ~0.1 ml) and passed through Hypersep pre-packed SPE cartridge (500 mg/2.8 ml, C18). The cartridge was conditioned in 85% ACN in TDW, then equilibrated in 10% ACN in TDW. The crude product was loaded on the SPE cartridge and washed with 10% ACN (2.5 ml), then dried with Ar. The cartridge was extracted with 65% ACN (~2.5 ml), and the fraction evaporated to obtain the deacylation product. HRMS (ESI-QTOF) m/z: $[M+H]^+$ Calcd for $C_{67}H_{82}NO_{21}S$ 1268.5095; found 1268.5090. The extracted fraction and $Pd(OH)_2$/C (10-20% wt. loading, 8.8 mg) were suspended in THF and TDW (1:1, 1.2 ml). The mixture was stirred in a pressurized reaction vessel under 4 bar of H_2 at RT for 20 h. LC-MS indicated completion of the reaction. The reaction mixture was filtered through a Whatman 42 filter paper, then the filter paper was washed with more TDW, and the filtrate concentrated under reduced pressure. The crude product was dissolved in a minimal amount of TDW (~50 μl) and passed through Hypersep pre-packed SPE cartridge (500 mg/2.8 ml, C18). The cartridge was conditioned in 85% ACN in TDW, then equilibrated in TDW. The crude product was loaded on the SPE cartridge and washed with TDW (1.2 ml, 4x column void volume). Compound **mSt** was eluted with additional TDW (~2 ml) and lyophilized (2.2 mg, 94% 2-step yield). HRMS (ESI-QTOF) m/z: $[M-Na]^-$ Calcd for $C_{24}H_{44}NO_{19}S$ 682.2234; found 682.2223. ^{1}H NMR (700 MHz, D_2O): δ, ppm 5.18 (d, $J_{1,2}$ = 3.8 Hz, 1H, H-1'(Galα)), 4.60 (d, $J_{1,2}$ = 7.8 Hz, 1H, H-1"(Galβ)), 4.48 (d, $J_{1,2}$ = 7.9 Hz, 1H, H-1(Galβ)), 4.48 (t, $J_{5,6}$ = 6.3 Hz, 1H, H-5'), 4.32 (dd, J_{AB} = 11.2 Hz, $J_{5,6}$ = 5.0 Hz, 1H, H-6'A), 4.31 (d, $J_{3,4}$ = 3.4 Hz, 1H, H-4'), 4.20 (dd, J_{AB} = 10.7 Hz, $J_{5,6}$ = 7.5 Hz, 1H, H-6'B), 4.19 (d, $J_{3,4}$ = 2.8 Hz, 1H, H-4), 4.08 (dd, $J_{3,4}$ = 3.1 Hz, $J_{2,3}$ = 10.4 Hz, 1H, H-3'), 3.98 (dd, $J_{1,2}$ = 4.3 Hz, $J_{2,3}$ = 10.5 Hz, 1H, H-2'), 3.98 - 3.94 (m, 1H, -OC**H_2**(CH_2)$_5$$NH_2$), 3.93 (d, $J_{3,4}$ = 3.4 Hz, 1H, H-4"), 3.84 (dd, J_{AB} = 11.8 Hz, $J_{5,6}$ = 7.6 Hz, 1H, H-6"A), 3.81 (dd, J_{AB} = 11.6 Hz, $J_{5,6}$ = 7.6 Hz, 1H, H-6A), 3.79 (dd, $J_{3,4}$ = 4.1 Hz, $J_{2,3}$ = 9.9 Hz, 1H, H-3), 3.77 (dd, J_{AB} = 11.9 Hz, $J_{5,6}$ = 4.4 Hz, 1H, H-6B), 3.74 (dd, J_{AB} = 11.8 Hz, $J_{5,6}$ = 4.6 Hz, 1H, H-6"B), 3.73 - 3.66 (m, 4H, -OC**H_2**(CH_2)$_5$$NH_2$; H-5; H-5"; H-3"), 3.62 (dd, $J_{1,2}$ = 8.6 Hz, $J_{2,3}$ = 9.4 Hz, 1H, H-2), 3.60 (dd, $J_{1,2}$ = 8.2 Hz, $J_{2,3}$ = 9.6 Hz, 1H, H-2"), 3.00 (t, J = 7.5 Hz, 2H, -O(CH_2)$_5$C**H_2**NH_2), 1.71 - 1.63 (m, 4H, -OCH_2C**H_2**(CH_2)$_2$C**H_2**$CH_2$$NH_2$), 1.46 - 1.41 (m, 4H, -O(CH_2)$_2$ (C**H_2**)$_2$(CH_2)$_2$$NH_2$). ^{13}C{^{1}H} NMR (176 MHz, D_2O): δ, ppm 102.1 (C-1"(Galβ)), 100.3 (C-1(Galβ)),

93.2 (C-1'(Galα)), 76.1 (C-4'), 75.5 (C-3), 72.9 (C-5"), 72.6 (C-5), 70.6 (C-3"), 69.4 (C-2"), 68.0 (-O**C**$H_2(CH_2)_5NH_2$), 67.4 (C-3'), 67.0 (C-2), 66.4 (C-4"), 66.3 (C-2'/C-5'), 66.3 (C-2'/C-5'), 65.6 (C-6'), 62.7 (C-4), 58.7 (C-6; C-6"), 37.2 (-O$(CH_2)_5$**C**H_2NH_2), 26.1 (-(**C**$H_2)_4$-), 24.5 (-(**C**$H_2)_4$-), 23.0 (-(**C**$H_2)_4$-), 22.2 (-(**C**$H_2)_4$-). *Note: Assignment of the reducing H-1 was done with additional information from ^1H-^{13}C HMBC using the H-1↔$OCH_2(CH)_5N$- correlation. Note 2: The configuration of the anomeric carbons C-1, C-1', and C-1" was assigned with additional information from non-decoupled ^1H-^{13}C HSQC (vide infra, ^1H-^{13}C non-decoupled HSQC spectrum **mSt**): 102.1 (d, J=163Hz, C-1" (β)), 100.3 (d, J=161Hz, C-1 (β)), 93.2 (d, J=171Hz, C-1' (α)).*

N-benzyl-N-benzyloxycarbonyl-6-aminohexyl 4,6-di-O-benzoyl-2,3-di-O-benzyl-α-D-galactopyranosyl-(1→3)-2,4,6-tri-O-benzyl-β-D-galactopyranosyl-(1→4)-2,3-di-O-benzyl-6-O-levulinoyl-α-D-galactopyranosyl-(1→3)-2,4,6-tri-O-benzoyl-β-D-galactopyranoside (37)

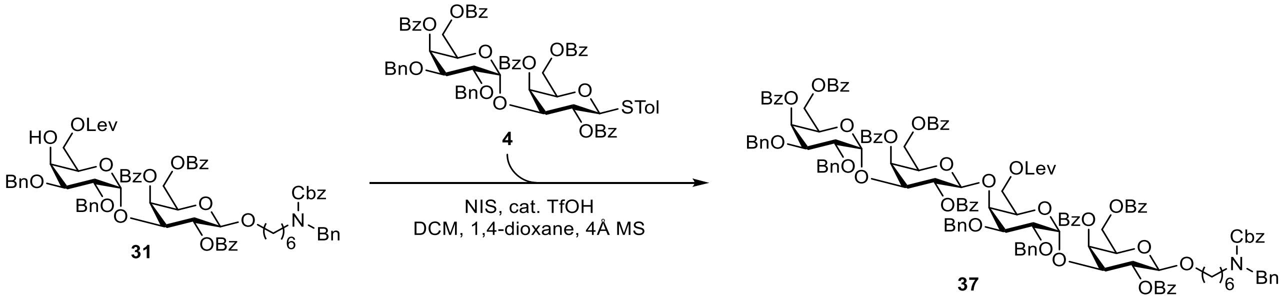

Glycosyl acceptor **31** (25.7 mg, 20.5 μmol) and glycosyl donor **4** (34.6 mg, 30.1 μmol) were co-evaporated from anhydrous toluene and dried overnight under high vacuum. The donor, acceptor, and freshly MW-activated and crushed molecular sieves (4Å, ~0.35 g) were suspended in anhydrous DCM (2 ml) and stirred at RT for 2 h, then cooled to 5°C in water bath. Activator solution (0.4 ml, anhydrous DCM and anhydrous 1,4-dioxane (2:1)) containing recrystallized NIS (13 mg, 59 μmol) and TfOH (0.53 μl, 6.0 μmol) was added dropwise. The reaction mixture was allowed to reach 20°C over 2 h. The reaction mixture was filtered through celite, directly onto aqueous $Na_2S_2O_3$ (10% w/w, ~2 ml) and sat. $NaHCO_3$ (~2 ml). The celite was washed with DCM (~30 ml) and the combined filtrate was washed with an additional portion of aqueous $Na_2S_2O_3$ (10% w/w, ~10 ml) and sat. $NaHCO_3$ (~10 ml). The layers were separated and the aqueous layer was extracted with DCM (3x 10 ml). The combined organic phase was washed with brine, dried over Na_2SO_4, then filtered and concentrated under reduced pressure. Compound **37** was purified by Prep-HPLC (7.0 mg, 15% yield, elution at 100% ACN). HRMS (ESI-QTOF) m/z: $[M+H]^+$ Calcd for $C_{134}H_{130}NO_{33}$ 2280.8520; found 2280.8505. ^1H NMR (500 MHz, $CDCl_3$): δ, ppm 8.09 - 8.01 (m, 6H), 7.99 (d, J = 7.5 Hz, 2H), 7.95 - 7.92 (m, 2H), 7.80 - 7.75 (m, 5H), 7.61 (t, J = 7.6 Hz, 1H), 7.57 - 7.53 (m, 2H), 7.51 - 7.40 (m, 11H), 7.37 - 7.28 (m, 15H), 7.17 - 7.11 (m, 10H), 7.10 - 7.03 (m, 7H), 7.02 - 6.95 (m, 5H), 6.88 - 6.84 (m, 2H), 6.51 (d, J = 7.5 Hz, 2H), 5.91 (d, $J_{3,4}$ = 3.1 Hz, 1H, H-4), 5.83 (d, $J_{3,4}$ = 3.3 Hz, 1H, H-4"), 5.62 (dd, $J_{1,2}$ = 7.9 Hz, $J_{2,3}$ = 10.2 Hz, 1H, H-2), 5.56 (dd, $J_{1,2}$ = 8.1 Hz, $J_{2,3}$ = 10.1 Hz, 1H, H-2"), 5.24 (d, $J_{3,4}$ = 1.7 Hz, 1H, H-4'''), 5.22 (d, $J_{1,2}$ = 3.4 Hz, 1H, H-1'(Galα)), 5.19 (d, $J_{1,2}$ = 3.4 Hz, 1H, H-1'''(Galα)), 5.15 (s, 2H, -C***H*₂**-(Cbz)), 4.76 (d, $J_{1,2}$ = 7.8 Hz, 1H, H-1(Galβ)), 4.69 (d, $J_{1,2}$ = 7.9 Hz, 1H, H-1"(Galβ)), 4.57 (dd, $J_{5,6}$ = 6.9 Hz, J_{AB} = 11.2 Hz, 1H, H-6A), 4.44 (dd, $J_{5,6}$ = 6.1 Hz, J_{AB} = 11.2 Hz, 1H, H-6'A), 4.42 - 4.42 (br, 2H, -C$\boldsymbol{H_2}$-(NBn)), 4.41 (d, J_{AB} = 12.2 Hz, 1H, O-C$\boldsymbol{H_2}$-Bn), 4.40 (d, J_{AB} = 11.0 Hz, 1H, O-C$\boldsymbol{H_2}$-Bn), 4.32 (d, J_{AB} = 12.1 Hz, 1H, O-C$\boldsymbol{H_2}$-Bn), 4.24 (d, J_{AB} = 11.7 Hz, 1H, O-C$\boldsymbol{H_2}$-Bn), 4.30 - 4.12 (m, 7H, H-6B; H-6'B; H-3; H-5; H-6"; H-6'''A), 4.18 (d, J_{AB} = 11.1 Hz, 1H, O-C$\boldsymbol{H_2}$-Bn), 4.12 (d, J_{AB} = 12.0 Hz, 1H, O-C$\boldsymbol{H_2}$-Bn), 4.01 - 4.00 (m, 2H, H-3"; H-5"), 4.00 (d, J_{AB} = 11.5 Hz, 1H, O-C$\boldsymbol{H_2}$-Bn), 3.95 (dd, $J_{5,6}$ = 7.3 Hz, J_{AB} = 10.5 Hz, 1H, H-6'''B), 3.89 - 3.86 (m, 2H, H-5'''; -OC$\boldsymbol{H_2}$$(CH_2)_5N$-), 3.75 (dd, $J_{1,2}$ = 3.3 Hz, $J_{2,3}$ = 10.0 Hz, 1H, H-2'''), 3.74 (t, $J_{5,6}$ = 6.6 Hz, 1H, H-5'), 3.68 (dd, $J_{3,4}$ = 3.1 Hz, $J_{2,3}$ = 10.1 Hz, 1H, H-3'''), 3.63 (d, J_{AB} = 11.6 Hz, 1H, O-C$\boldsymbol{H_2}$-Bn), 3.52 (dd, $J_{1,2}$ = 3.3 Hz, $J_{2,3}$ = 9.3 Hz, 1H, H-2'), 3.49 - 3.47 (br, 1H, -OC$\boldsymbol{H_2}$$(CH_2)_5N$-), 3.44 - 3.40 (m, 2H, H-3'; H-4'), 3.11 - 3.03 (br,

2H, -O(CH_2)$_5$CH_2N-), 2.74 - 2.73 (br, 2H, -CH_2-(Lev)), 2.65 - 2.64 (br, 2H, -CH_2-(Lev)), 2.12 (s, 3H, -CH_3(Lev)), 1.47 - 1.46 (br, 2H, -(CH_2)$_4$-), 1.30 - 1.30 (br, 2H, -(CH_2)$_4$-), 1.16 - 1.06 (br, 4H, -(CH_2)$_4$-), residual acetone (~1.3:1 ratio, δ 2.18), impurity (δ 2.62). ^{13}C{^{1}H} NMR (126 MHz, $CDCl_3$): δ , ppm 206.6 (R**C**OR (Lev)), 172.1 (R**C**OR (Lev)), 166.2 (**C**=O (Bz)), 166.0 (**C**=O (Bz)), 165.9 (**C**=O (Bz)), 165.7 (**C**=O (Bz)), 165.0 (**C**=O (Bz)), 164.9 (**C**=O (Bz)), 138.6, 138.5, 138.3, 138.0, 133.4, 133.3, 133.3, 133.1, 130.3, 130.1, 130.0, 129.9, 129.8, 129.8, 129.6, 129.5, 129.4, 129.2, 128.6, 128.5, 128.4, 128.3, 128.1, 128.1, 128.0, 127.9, 127.7, 127.6, 127.3, 127.2, 126.7, 126.5, 101.8 (C-1(Galβ)), 101.6 (C-1''(Galβ)), 94.8 (C-1'''(Galα)), 93.9 (C-1'(Galα)), 77.4, 76.5, 75.9, 75.5, 73.7, 73.4, 73.1, 72.9, 72.5, 71.9, 71.4, 71.1, 71, 70.2, 68.7, 68.1, 67.2, 65.9, 64.4, 62.2, 62, 37.8, 30, 29.8, 29.5, 28, 27.5, 26.5, 25.6, acetone (δ 207.1, 31.0). *Note: Cis-trans isomerism of the Cbz carbamate moiety results in broadening ^{13}C signals of* ***C****=O (Cbz); N-****C****H_2-Ph; N-****C****H_2(CH_5)O-, which did not appear ^{13}C-NMR. Note 2: The configuration of the anomeric carbons C-1, C-1', C-1'' and C-1''' was assigned with additional information from non-decoupled ^{1}H-^{13}C HSQC (vide infra, ^{1}H-^{13}C non-decoupled HSQC spectrum* ***37****): 101.8 (d, J=158Hz, C-1 (β)), 101.6 (d, J=162Hz, C-1'' (β)), 94.8 (d, J=172Hz, C-1''' (α)), 93.9 (d, J=173Hz, C-1' (α)).*

N-benzyl-N-benzyloxycarbonyl-6-aminohexyl 4,6-di-O-benzoyl-2,3-di-O-benzyl-α-D-galactopyranosyl-(1→3)-2,4,6-tri-O-benzyl-β-D-galactopyranosyl-(1→4)-6-O-benzoyl-2,3-di-O-benzyl-α-D-galactopyranosyl-(1→3)-2,4,6-tri-O-benzoyl-β-D-galactopyranoside (38)

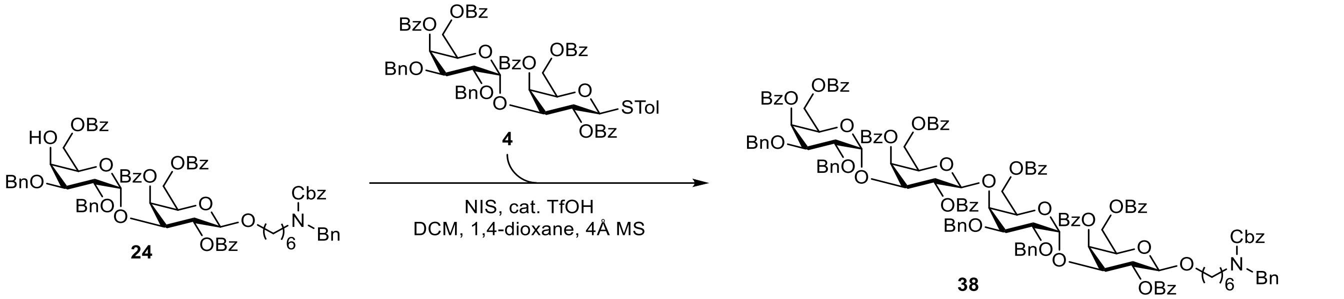

Glycosyl acceptor **24** (24.0 mg, 19.0 μmol) and glycosyl donor **4** (32.2 mg, 28.0 μmol) were co-evaporated from anhydrous toluene and dried overnight under high vacuum. The donor, acceptor, and freshly MW-activated and crushed molecular sieves (4Å, ~0.5 g) were suspended in anhydrous DCM (1 ml) and stirred at RT for 1.5 h, then cooled to 5°C in water bath. Activator solution (0.94 ml, anhydrous DCM and anhydrous 1,4-dioxane (2:1)) containing recrystallized NIS (31 mg, 0.14 mmol) and TfOH (1.2 μl, 14 μmol) was added dropwise. The reaction mixture was allowed to reach 19°C over 2 h. The reaction mixture was filtered through celite, directly onto aqueous $Na_2S_2O_3$ (10% w/w, ~4 ml) and sat. $NaHCO_3$ (~4 ml). The celite was washed with DCM (~30 ml) and the combined filtrate was washed with an additional portion of aqueous $Na_2S_2O_3$ (10% w/w, ~10 ml) and sat. $NaHCO_3$ (~10 ml). The layers were separated and the organic phase was washed with brine, dried over Na_2SO_4, then filtered and concentrated under reduced pressure. Compound **38** was purified by Prep-HPLC (6.9 mg, 16% yield, elution at 100% ACN). HRMS (ESI-QTOF) m/z: $[M+H]^+$ Calcd for $C_{136}H_{128}NO_{32}$ 2286.8414; found 2286.8336. ^{1}H NMR (500 MHz, $CDCl_3$): δ , ppm 8.11 - 8.06 (m, 6H), 8.05 (q, J = 3.1 Hz, 2H), 7.97 (d, J = 7.8 Hz, 2H), 7.93 - 7.91 (m, 2H), 7.81 - 7.79 (m, 6H), 7.61 - 7.56 (m, 2H), 7.56 - 7.41 (m, 13H), 7.37 - 7.26 (m, 12H), 7.25 - 7.22 (m, 5H), 7.16 - 7.11 (m, 9H), 7.10 - 7.03 (m, 6H), 7.01 - 6.96 (m, 5H), 6.84 (t, J = 7.7 Hz, 2H), 6.49 (d, J = 7.4 Hz, 2H), 5.83 (d, $J_{3,4}$ = 3.1 Hz, 1H, H-4), 5.81 (d, $J_{3,4}$ = 2.8 Hz, 1H, H-4''), 5.61 (dd, $J_{1,2}$ = 8.0 Hz, $J_{2,3}$ = 10.2 Hz, 1H, H-2), 5.56 (dd, $J_{1,2}$ = 8.0 Hz, $J_{2,3}$ = 10.4 Hz, 1H, H-2''), 5.28 - 5.26 (m, 2H, H-1'(Galα); H-4'''), 5.19 (d, $J_{1,2}$ = 3.2 Hz, 1H, H-1'''(Galα)), 5.15 (s, 2H, -CH_2-(Cbz)), 4.75 (d, $J_{1,2}$ = 7.9 Hz, 1H, H-1), 4.65 (dd, $J_{5,6}$ = 3.9 Hz, J_{AB} = 11.7 Hz, 1H, H-6A), 4.47 (dd, $J_{5,6}$ = 6.3 Hz, J_{AB} = 11.2 Hz, 1H, H-6'A), 4.42 (d, J_{AB} = 12.3 Hz, 1H, O-CH_2-Ph), 4.41 (d, J_{AB} = 11.1 Hz, 1H, O-CH_2-Ph), 4.40 - 4.34 (m, 4H, -CH_2-(NBn); H-6B; H-6''A), 4.33 (d, J_{AB} = 12.2 Hz, 1H, O-CH_2-Ph), 4.30 - 4.30 (m, 1H, H-6''B), 4.26 (dd, $J_{5,6}$ = 6.3 Hz, J_{AB} = 11.2 Hz, 1H, H-6'B), 4.19 (d, J_{AB} = 11.6 Hz, 2H, O-CH_2-Ph), 4.13 (dd, $J_{5,6}$ = 6.0 Hz, J_{AB} = 10.7 Hz, 1H, H-6'''A),

4.05 (d, J_{AB} = 11.7 Hz, 1H, O-C***H*₂**-Ph), 4.05 - 4.02 (m, 3H, H-1''(Galβ); H-3; H-3''), 4.02 - 3.98 (m, 2H, H-5; H-5''), 4.00 (d, J_{AB} = 11.3 Hz, 1H, O-C***H₂***-Ph), 3.96 (dd, $J_{5,6}$ = 7.0 Hz, J_{AB} = 10.8 Hz, 1H, H-6'''B), 3.78 - 3.74 (m, 2H, H-2'''; H-5'''), 3.73 - 3.73 (m, 1H, -OC***H₂***(CH_2)$_5$N-), 3.71 (dd, $J_{3,4}$ = 3.0 Hz, J_{AB} = 10.0 Hz, 1H, H-3'''), 3.66 (t, $J_{5,6}$ = 6.7 Hz, 1H, H-5'), 3.60 (d, J_{AB} = 11.3 Hz, 1H, O-C***H₂***-Ph), 3.57 - 3.56 (m, 1H, H-4'), 3.55 (dd, $J_{1,2}$ = 3.3 Hz, $J_{2,3}$ = 10.0 Hz, 1H, H-2'), 3.35 (dd, $J_{3,4}$ = 2.6 Hz, *J2,3* = 9.9 Hz, 1H, H-3'), 3.24 - 3.23 (br, 1H, -OC***H₂***(CH_2)$_5$N-), 3.10 - 3.01 (m, 2H, -O(CH_2)$_5$C***H₂***N-), 1.77 - 1.61 (m, 2H, -(C***H₂***)$_4$-), 1.38 - 1.33 (m, 2H, -(C***H₂***)$_4$-), 1.05 - 1.03 (m, 4H, -(C***H₂***)$_4$-), grease (δ 1.50 - 1.12). $^{13}C\{^1H\}$ NMR (126 MHz, $CDCl_3$): δ, ppm 166.1 (**C**=O (Bz)), 166.1 (**C**=O (Bz)), 166.0 (**C**=O (Bz)), 165.8 (**C**=O (Bz)), 165.7 (**C**=O (Bz)), 165.5 (**C**=O (Bz)), 164.9 (**C**=O (Bz)), 164.8 (**C**=O (Bz)), 138.5, 138.3, 138.3, 138.0, 133.4, 133.3, 133.1, 130.2, 130.1, 130.0, 129.9, 129.8, 129.8, 129.8, 129.7, 129.6, 129.6, 129.5, 129.4, 129.0, 128.7, 128.6, 128.6, 128.5, 128.4, 128.4, 128.3, 128.1, 128.1, 128.1, 127.9, 127.8, 127.8, 127.7, 127.6, 127.3, 127.2, 126.8, 126.6, 101.9 (C-1''(Galβ)), 101.6 (C-1(Galβ)), 94.8 (C-1'''(Galα)), 93.2 (C-1'(Galα)), 77.3, 76.3, 75.9, 75.1, 73.8, 73.3, 73.2, 72.8, 72.5, 71.9, 71.5, 71.2, 71.0, 70.5, 70.2, 68.7, 68.1, 67.1, 66.0, 65.7, 65.6, 64.3, 62.3, 62.0, 50.5 (-**C**H₂-(NBn)), 50.1 (-**C**H₂-(NBn)), 47.1 (-O(CH_2)$_5$**C**H₂N-), 46.1 (-O(CH_2)$_5$**C**H₂N-), 34.5, 29.8, 29.6, 29.5, 29.4, 29.3, 29.1, 26.8, 26.4, 25.6, 22.7. *Note: Cis-trans isomerism of the Cbz carbamate moiety results in split ^{13}C signal for N-**C**H₂-Ph; N-**C**H₂(CH₅)O-;* (**C**H₂)₄. *Broadening of corresponding -C**H₂**- groups in ^1H-NMR. Note 2: The configuration of the anomeric carbons C-1, C-1', C-1'' and C-1''' was assigned with additional information from non-decoupled ^1H-^{13}C HSQC (vide infra, ^1H-^{13}C non-decoupled HSQC spectrum **38**): 101.9 (d, J=160Hz, C-1 (β)), 101.6 (d, J=162Hz, C-1'' (β)), 94.8 (d, J=171Hz, C-1''' (α)), 93.2 (d, J=173Hz, C-1' (α)).*

N-benzyl-N-benzyloxycarbonyl-6-aminohexyl 4,6-di-O-benzoyl-2,3-di-O-benzyl-α-D-galactopyranosyl-(1→3)-2-O-benzoyl-4,6-di-O-benzyl-β-D-galactopyranosyl-(1→4)-2,3-di-O-benzyl-6-O-levulinoyl-α-D-galactopyranosyl-(1→3)-2,4,6-tri-O-benzoyl-β-D-galactopyranoside (39)

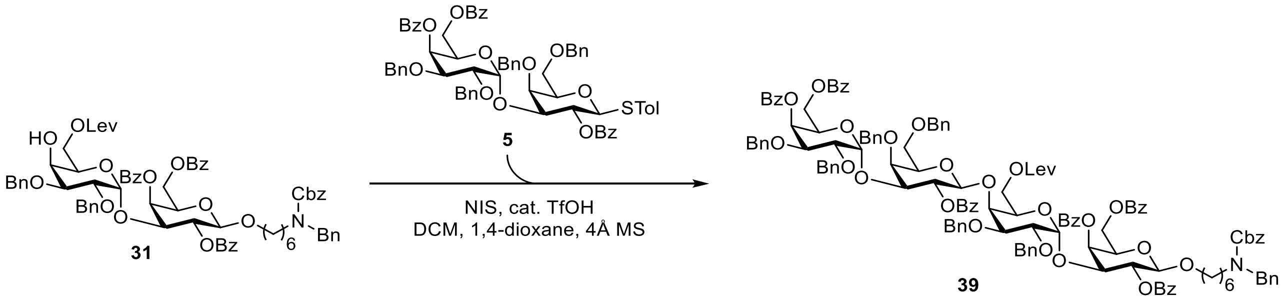

Glycosyl acceptor 31 (22.0 mg, 17.5 µmol) and glycosyl donor **5** (27.2 mg, 24.3 µmol) were co-evaporated from anhydrous toluene and dried overnight under high vacuum. The donor, acceptor, and freshly MW-activated and crushed molecular sieves (4Å, ~0.3 g) were suspended in anhydrous DCM (1 ml) and stirred at RT for 30 min, then cooled to -35°C in an acetone bath with an immersion cooler. Activator solution (0.19 ml, anhydrous DCM and anhydrous 1,4-dioxane (4:1)) containing recrystallized NIS (8.4 mg, 0.037 mmol) and trifluoromethanesulfonic acid (TMSOTf) (1.1 µl, 6.3 µmol) was added dropwise. The reaction mixture was allowed to reach -20°C over 1 h. The reaction mixture was filtered through celite, directly onto aqueous $Na_2S_2O_3$ (10% w/w, ~5 ml) and sat. $NaHCO_3$ (~5 ml). The celite was washed with DCM (~20 ml) and the combined filtrate was washed with an additional portion of aqueous $Na_2S_2O_3$ (10% w/w, ~10 ml) and sat. $NaHCO_3$ (~10 ml). The layers were separated and the aqueous layer was extracted with DCM (10 ml). The combined organic phase was washed with brine, dried over Na_2SO_4, then filtered and concentrated under reduced pressure. Compound **39** was purified by Prep-HPLC (17.3 mg, 44% yield, elution at 100% ACN). HRMS (ESI-QTOF) m/z: $[M+H]^+$ Calcd for $C_{134}H_{134}NO_{31}$ 2252.8934; found 2252.8846. ^1H NMR (500 MHz, $CDCl_3$): δ, ppm 8.02 (d, *J* = 7.6 Hz, 2H), 7.98 - 7.95 (m, 2H), 7.91 - 7.86 (m, 4H), 7.81 - 7.77 (m, 4H), 7.57 - 7.47 (m, 3H), 7.46 - 7.26 (m, 23H), 7.25 - 7.14 (m, 22H), 7.09 - 7.04 (m, 3H), 7.03 - 6.97 (m, 2H), 6.94 (t, *J* = 7.4 Hz, 2H), 6.88 (t, *J* = 7.4 Hz, 2H), 6.56 (d, *J* = 7.6 Hz, 2H), 5.88 (br, 1H, H-4),

5.59 (dd, $J_{1,2} = J_{2,3}$ = 8.5 Hz, 1H, H-2), 5.52 (dd, $J_{1,2} = J_{2,3}$ = 8.9 Hz, 1H, H-2'), 5.48 (d, $J_{3,4}$ = 1.4 Hz, 1H, H-4'''), 5.17 - 5.13 (m, 3H, -C***H***$_2$-(Cbz); H-1'(Galα)), 5.03 (d, $J_{1,2}$ = 3.2 Hz, 1H, H-1'''(Galα)), 4.98 (d, J_{AB} = 11.8 Hz, 1H, O-C***H***$_A$H$_B$-Bn), 4.75, 4.57 (ABq, $\Delta\delta_{AB}$ = 0.18, J_{AB} = 11.6 Hz, 2H, O-C***H***$_2$-Bn), 4.74 - 4.73 (m, 1H, H-1(Galβ)), 4.62, 4.36 (ABq, $\Delta\delta_{AB}$ = 0.26, J_{AB} = 11.3 Hz, 2H, O-C***H***$_2$-Bn), 4.59 - 4.56 (m, 2H, H-1''(Galβ); H-6A), 4.44 - 4.39 (m, 5H, O-CH$_A$***H***$_B$-Bn; -C***H***$_2$-(NBn); O-C***H***$_2$-Bn), 4.28, 4.14 (ABq, $\Delta\delta_{AB}$ = 0.14, J_{AB} = 11.5 Hz, 2H, O-C***H***$_2$-Bn), 4.27 - 4.27 (m, 1H, H-6B), 4.18 - 4.16 (m, 2H, H-3; H-5), 4.12 - 4.10 (m, 3H, H-4'; H-6'), 3.96 - 3.84 (m, 7H, H-3'''; H-4''; H-6'''; H-2'''; -OC***H***$_2$(CH$_2$)$_5$N-; O-C***H***$_A$H$_B$-Bn), 3.82 - 3.80 (m, 1H, H-5'), 3.75 - 3.73 (m, 1H, H-3''), 3.52 - 3.43 (m, 4H, H-6''; -OC***H***$_2$(CH$_2$)$_5$N-; O-CH$_A$***H***$_B$-Bn), 3.39 - 3.37 (m, 4H, H-5''; H-5'''; H-2'; H-3'), 3.11 - 3.02 (br, 2H, -O(CH$_2$)$_5$C***H***$_2$N-), 2.70 - 2.70 (br, 2H, -C***H***$_2$-(Lev)), 2.59 - 2.58 (br, 2H, -C***H***$_2$-(Lev)), 2.13 (s, 3H, -C***H***$_3$ (Lev)), 1.47 - 1.41 (br, 2H, -(C***H***$_2$)$_4$-), 1.28 - 1.25 (br, 2H, -(C***H***$_2$)$_4$-), 1.16 - 1.08 (br, 4H, -(C***H***$_2$)$_4$-), H_2O exchange (δ 3.00). $^{13}C\{^1H\}$ NMR (126 MHz, $CDCl_3$): δ , ppm 206.8 (R**C**OR (Lev)), 172.3 (R**C**OR (Lev)), 166.1 (**C**=O (Bz)), 165.8 (**C**=O (Bz)), 165.6 (**C**=O (Bz)), 165.5 (**C**=O (Bz)), 165.3 (**C**=O (Bz)), 165.0 (**C**=O (Bz)), 139.0, 138.7, 138.4, 138.1, 138.1, 138.0, 136.8, 133.2, 133.2, 133.1, 133.0, 130.1, 129.9, 129.8, 129.7, 129.7, 129.6, 129.1, 128.6, 128.6, 128.5, 128.4, 128.4, 128.3, 128.3, 128.3, 128.2, 128.2, 127.9, 127.9, 127.9, 127.8, 127.8, 127.7, 127.6, 127.5, 127.3, 126.7, 126.5, 101.8 (C-1(Galβ)), 101.6 (C-1''(Galβ)), 98.2 (C-1'''(Galα)), 93.8 (C-1'(Galα)), 79.7 (C-3''), 77.3, 76.5, 76.2, 74.8, 74.8 (O-**C**H$_2$-Bn), 74.5, 74.3 (O-**C**H$_2$-Bn), 73.6, 73.4 (O-**C**H$_2$-Bn; O-**C**H$_2$-Bn), 72.7 (O-**C**H$_2$-Bn), 72.4 (C-3), 71.7 (O-**C**H$_2$-Bn), 71.5 (C-2''), 71.4 (C-5), 71.1 (C-2), 70.2 (-O**C**H$_2$(CH$_2$)$_5$N-), 68.7 (C-5'; C-6''), 67.9 (C-4'''), 67.3 (-**C**H$_2$-(Cbz)), 67.2 (C-4'), 66.1 (C-4), 64.7 (C-6'), 62.2 (C-6), 61.6 (C-6'''), 50.4 (-**C**H$_2$-(NBn)), 50.1 (-**C**H$_2$-(NBn)), 47.1 (-O(CH$_2$)$_5$**C**H$_2$N-), 46.2 (-O(CH$_2$)$_5$**C**H$_2$N-), 37.9 (-**C**H$_2$- (Lev)), 30.0 (-**C**H$_3$ (Lev)), 29.8 (-(**C**H$_2$)$_4$-), 29.4 (-(**C**H$_2$)$_4$-), 28.2 (-**C**H$_2$- (Lev)), 27.9 (-(**C**H$_2$)$_4$-), 27.5 (-(**C**H$_2$)$_4$-), 26.5 (-(**C**H$_2$)$_4$-), 25.6 (-(**C**H$_2$)$_4$-). *Note: Cis-trans isomerism of the Cbz carbamate moiety results in split ^{13}C signal for N-**C**H$_2$-Ph; N-**C**H$_2$(CH$_5$)O-; (**C**H$_2$)$_4$. Broadening of corresponding -C**H$_2$**- groups in ^1H-NMR. The ^{13}C signal of **C**=O (Cbz) (δ 156.6) did not appear in ^{13}C-NMR and was confirmed with additional information from ^1H-^{13}C HMBC. Note 2: Assignment of the reducing H-1 was done with additional information from ^1H-^{13}C HMBC using the H-1↔OCH$_2$(CH)$_5$N- correlation. Note 3: The configuration of the anomeric carbons C-1, C-1', C-1'' and C-1''' was assigned with additional information from non-decoupled ^1H-^{13}C HSQC (vide infra, ^1H-^{13}C non-decoupled HSQC spectrum* ***39****): 101.8 (d, J=159Hz, C-1 (β)), 101.6 (d, J=162Hz, C-1'' (β)), 98.2 (d, J=169Hz, C-1''' (α)), 93.8 (d, J=171Hz, C-1' (α)).*

6-Aminohexyl α-D-galactopyranosyl-(1→3)-β-D-galactopyranosyl-(1→4)-α-D-galactopyranosyl-(1→3)-β-D-galactopyranoside (nST)

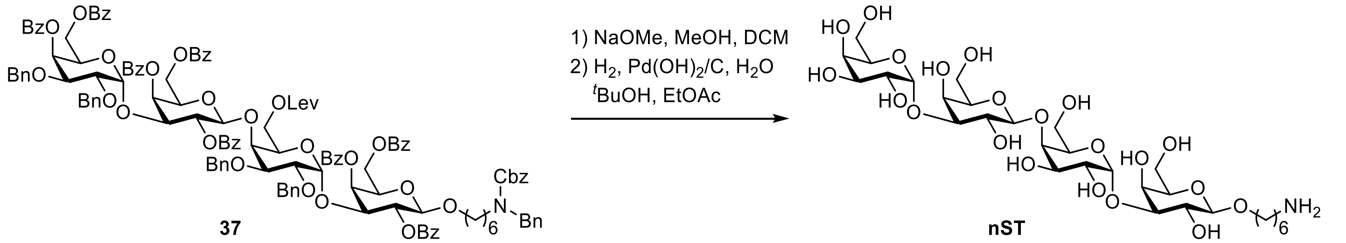

Compound **37** (7.0 mg, 3.1 μmol) was dissolved in anhydrous DCM (0.5 ml). NaOMe in anhydrous MeOH (0.5 M, 1 ml) was added dropwise. The reaction mixture was stirred at RT for 7 h, until completion (as indicated by TLC, 5% MeOH in DCM, R_f=0.11). The reaction mixture was diluted with MeOH (~5 ml) and ion exchange beads Amberlight® XAD16N (H-form, pre-washed in DCM and MeOH) were added in small portions until neutral pH was reached and reaction mixture turned clear. The reaction mixture was filtered and the filtrate was concentrated under reduced pressure, then dried under high vacuum. The crude product was passed through RP-HPLC in several runs and partially deprotected tetrasaccharide was collected (elution at 43% ACN in TDW), lyophilized, and used directly in the next step. The lyophilized fraction and

$Pd(OH)_2/C$ (10-20% wt. loading, 1.6 mg) were suspended in EtOAc, ^{t}BuOH, and TDW (0.5:1:0.5, 2 ml). The mixture was stirred in a pressurized reaction vessel under 4 bar of H_2 at RT for 12 h. The reaction mixture was filtered through celite, and the celite was washed with MeOH and MeOH/TDW (1:1). The combined filtrate was concentrated under reduced pressure to produce a colorless glassy residue. Compound **nST** was purified by SEC on Sephadex LH-20 size exclusion resin (100 mg, 140-550 mesh, swelled in 90% MeOH in TDW). Tetrasaccharide **nST** was eluted with 90% MeOH in TDW within the first 1.5 column void volume (1.2 mg, 51% 2-step yield). HRMS (ESI-QTOF) m/z: $[M+H]^+$ Calcd for $C_{30}H_{56}NO_{21}$ 766.3339; found 766.3319. ^1H NMR (700 MHz, D_2O): δ, ppm 5.18 (d, $J_{1,2}$ = 4.0 Hz, 1H, H-1^{II}), 5.17 (d, $J_{1,2}$ = 4.0 Hz, 1H, H-1^{IV}), 4.70 (d, $J_{1,2}$ = 8.0 Hz, 1H, H-1^{III}), 4.47 (d, $J_{1,2}$ = 7.9 Hz, 1H, H-1^{I}), 4.31 (d, $J_{3,4}$ = 2.6 Hz, 1H, H-4^{II}), 4.28 (t, $J_{5,6}$ = 6.6 Hz, 1H, H-5^{II}), 4.23 (t, $J_{5,6}$ = 6.3 Hz, 1H, H-5^{IV}), 4.19 (m, 2H, H-4^{I}; H-4^{III}), 4.07 (dd, $J_{3,4}$ = 3.1 Hz, $J_{2,3}$ = 10.3 Hz, 1H, H-3^{II}), 4.04 (d, $J_{3,4}$ = 3.4 Hz, 1H, H-4^{IV}), 3.99 - 3.95 (m, 3H, H-2^{II}; H-3^{IV}; -OC$\boldsymbol{H_2}$(CH$_2$)$_5$NH$_2$), 3.90 - 3.86 (m, 2H, H-2^{VI}; H-6^{II}), 3.84 - 3.74 (m, 10H, H-6^{I}; H-6^{II}; H-6^{III}; H-6^{IV}; H-2^{III}; H-5^{I}; H-5^{III}), 3.74 - 3.69 (m, 3H; H-3^{I}; H-3^{III}; -OC$\boldsymbol{H_2}$(CH$_2$)$_5$NH$_2$), 3.65 (dd, $J_{1,2}$ = 8.1 Hz, $J_{2,3}$ = 9.7 Hz, 1H), 2.98 (t, J = 7.5 Hz, 2H, -O(CH$_2$)$_5$C$\boldsymbol{H_2}$NH$_2$), 1.68 - 1.66 (m, 4H, -OCH$_2$C$\boldsymbol{H_2}$(CH$_2$)$_2$C$\boldsymbol{H_2}$CH$_2$NH$_2$), 1.44 - 1.42 (m, 4H, -O(CH$_2$)$_2$ (C$\boldsymbol{H_2}$)$_2$(CH$_2$)$_2$NH$_2$), formate (δ 8.47 (s)), acetate (~1:1.3 ratio; δ 1.93 (s)). $^{13}C\{^1H\}$ NMR (176 MHz, D_2O): δ, ppm 102.1 (C-1^{III}), 100.4 (C-1^{I}), 93.2 (C-1^{II}), 93.1 (C-1^{IV}), 76.4 (C-4^{II}), 75.4 (C-5$^{I/III}$), 75.1 (C-5$^{I/III}$), 72.7 (C-3$^{I/III}$), 72.6 (C-3$^{I/III}$), 68.7 (C-5^{IV}), 68.1 (-O**C**H$_2$(CH$_2$)$_5$NH$_2$), 67.9 (C-5^{II}), 67.8, 67.6, 67.1, 67.0, 66.9, 66.5, 66.0 (C-2^{II}), 62.7 (C-4$^{I/III}$), 62.6 (C-4$^{I/III}$), 58.8 (C-6$^{I/III/IV}$), 58.7 (C-6$^{I/III/IV}$), 58.7 (C-6$^{I/III/IV}$), 58.2 (C-6^{II}), 37.3 (-O(CH$_2$)$_5$CH$_2$NH$_2$), 26.2 (-(**C**H$_2$)$_4$-), 24.8 (-(**C**H$_2$)$_4$-), 23.1 (-(**C**H$_2$)$_4$-), 22.3 (-(**C**H$_2$)$_4$-), acetate (δ 179.3, 21.0), formate (δ 168.8).

N-benzyl-N-benzyloxycarbonyl-6-aminohexyl 4,6-di-O-benzoyl-2,3-di-O-benzyl-α-D-galactopyranosyl-(1→3)-2-O-benzoyl-4,6-di-O-benzyl-β-D-galactopyranosyl-(1→4)-2,3-di-O-benzyl-α-D-galactopyranosyl-(1→3)-2,4,6-tri-O-benzoyl-β-D-galactopyranoside (40)

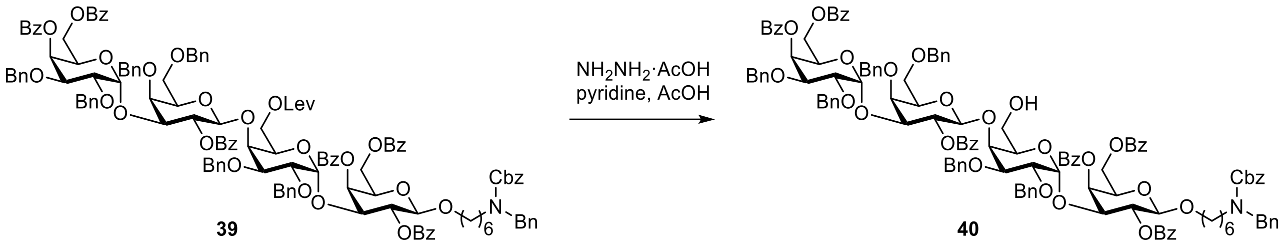

Compound **39** (28.5 mg, 12.6 μmol) was dissolved in a pyridine/AcOH buffer (4:1, 0.5 ml). $NH_2NH_2 \cdot AcOH$ (6.8 mg, 74 μmol) was separately dissolved in pyridine/AcOH (4:1, 0.5 ml), two drops of TDW and sonication was needed to completely dissolve the solids. The solution of $NH_2NH_2 \cdot AcOH$ in pyridine/AcOH was added dropwise to **33**. The reaction mixture was stirred at RT for 40 min. The reaction mixture was diluted with DCM (~10 ml) and was washed with aqueous HCl (0.1 M, ~10 ml). The layers were separated and the aqueous phase was acidified to pH~2, then extracted with DCM (5 ml). The combined organic phase was washed with sat. $NaHCO_3$, then brine. The organic phase was dried over Na_2SO_4, filtered, and evaporated to obtain pure **40** (24.0 mg, 88% yield) without further purification. HRMS (ESI-QTOF) m/z: $[M+H]^+$ Calcd for $C_{129}H_{128}NO_{29}$ 2154.8567; found 2154.8593. ^1H NMR (500 MHz, $CDCl_3$): δ, ppm 8.04 - 7.98 (m, 4H), 7.94 (dd, J = 1.1, 8.2 Hz, 2H), 7.89 (dd, J = 1.1, 8.3 Hz, 2H), 7.80 (dt, J = 1.1, 8.2 Hz, 4H), 7.57 - 7.50 (m, 3H), 7.48 - 7.39 (m, 24H), 7.39 - 7.26 (m, 24H), 7.25 - 7.14 (m, 20H), 7.10 (t, J = 7.4 Hz, 1H), 7.04 (t, J = 7.4 Hz, 3H), 6.95 (dt, J = 1.9, 7.7 Hz, 4H), 6.65 (d, J = 7.3 Hz, 2H), 5.81 (d, $J_{3,4}$ = 3.0 Hz, 1H, H-4), 5.64 - 5.58 (m, 1H, H-2”), 5.60 (dd, $J_{1,2}$ = 8.0 Hz, $J_{2,3}$ = 10.2 Hz, 1H, H-2), 5.50 (d, $J_{3,4}$ = 1.2 Hz, 1H, H-4”’), 5.15 (s, 2H, -C$\boldsymbol{H_2}$-(Cbz)), 5.02 (d, $J_{1,2}$ = 3.3 Hz, 1H, H-1”’(Galα)), 4.94 (d, J_{AB} = 11.7 Hz, 1H, O-C$\boldsymbol{H_2}$-Bn), 4.94 (d, $J_{1,2}$ = 3.3 Hz, 1H, H-1’(Galα)), 4.78 (d, J_{AB} = 11.6 Hz, 1H, O-C$\boldsymbol{H_2}$-Bn), 4.64 (d, J_{AB} = 11.4 Hz, 1H, O-C$\boldsymbol{H_2}$-Bn), 4.57 (d, J_{AB} = 11.5 Hz, 1H, O-C$\boldsymbol{H_2}$-Bn), 4.60 - 4.50 (m, 2H, H-1(Galβ); H-1”(Galβ); H-6A), 4.51 (d, J_{AB} = 11.8 Hz, 1H, O-C$\boldsymbol{H_2}$-Bn), 4.41 - 4.40 (br, 2H, -C$\boldsymbol{H_2}$-(NBn)), 4.38 (d, J_{AB} = 12.0 Hz, 1H, O-C$\boldsymbol{H_2}$-Bn),

4.37 (d, J_{AB} = 11.4 Hz, 1H, O-C***H***$_2$-Bn), 4.32 (dd, J_{AB} = 11.6 Hz, $J_{5,6}$ = 5.0 Hz, 1H, H-6B), 4.32 (d, J_{AB} = 11.9 Hz, 1H, O-C***H***$_2$-Bn), 4.24 (d, J_{AB} = 11.6 Hz, 1H, O-C***H***$_2$-Bn), 4.16 - 4.11 (m, 2H, H-5'''; O-C***H***$_2$-Bn), 4.00 (t, $J_{5,6}$ = 7.0 Hz, 1H, H-5), 3.97 (dd, $J_{3,4}$ = 3.1 Hz, $J_{2,3}$ = 10.9 Hz, 1H, H-3), 3.94 (dd, $J_{3,4}$ = 2.9 Hz, $J_{2,3}$ = 10.5 Hz, 1H, H-3'''), 3.92 - 3.86 (m, 4H, H-6'''; H-2'''; -OC***H***$_2$(CH$_2$)$_5$N-), 3.82 - 3.78 (m, 2H, H-4'', O-C***H***$_2$-Bn), 3.77 - 3.66 (m, 4H, H-3''; H-4'; H-5'; H-6'A), 3.59 (d, J_{AB} = 11.7 Hz, 1H, O-C***H***$_2$-Bn), 3.54 (dd, J_{AB} = 9.2 Hz, $J_{5,6A}$ = 7.5, 1H, H-6''A), 3.49 - 3.42 (m, 3H, H-5''; H-3'; -OC***H***$_2$(CH$_2$)$_5$N-), 3.39 (dd, $J_{1,2}$ = 3.2 Hz, $J_{2,3}$ = 10.1 Hz, 1H, H-2'), 3.34 (dd, J_{AB} = 10.2 Hz, $J_{5,6}$ = 4.2 Hz, 1H, H-6'B), 3.29 (dd, J_{AB} = 9.3 Hz, $J_{5,6}$ = 4.6 Hz, 1H, H-6''B), 3.11 - 3.01 (m, 2H, -O(CH$_2$)$_5$C***H***$_2$N-), 1.45 - 1.44 (br, 2H, -(C***H***$_2$)$_4$-), 1.31 - 1.25 (br, 2H, -(C***H***$_2$)$_4$-), 1.15 - 1.07 (br, 4H, -(C***H***$_2$)$_4$-), residual acetone (δ 2.05). ^{13}C{^{1}H} NMR (126 MHz, CDCl$_3$): δ , ppm 166.2 (**C**=O(Bz)), 165.7 (**C**=O(Bz)), 165.6 (**C**=O(Bz)), 165.5 (**C**=O(Bz)), 165.3 (**C**=O(Bz)), 165.1 (**C**=O(Bz)), 156.7 (**C**=O(Cbz)), 156.2 (**C**=O(Cbz)), 138.9, 138.7, 138.6, 138.1, 138.0, 137.9, 137.6, 136.9, 133.2, 133.1, 133.1, 133.1, 133.0, 130.1, 129.8, 129.8, 129.7, 129.7, 129.6, 129.2, 128.6, 128.5, 128.4, 128.4, 128.3, 128.3, 128.2, 128.2, 128.0, 127.9, 127.9, 127.9, 127.7, 127.6, 127.5, 127.5, 127.3, 126.9, 126.7, 102.4 (C-1(Galβ)), 101.9 (C-1''(Galβ)), 98.5 (C-1'''(Galα)), 96.2 (C-1'(Galα)), 79.8 (C-3''), 77.4 (C-3'), 77.1 (C-2'), 76.2 (C-3'''), 74.9 (C-2'''), 74.8 (C-5'), 74.6 (O-**C**H$_2$-Bn), 74.5 (C-3), 74.4 (O-**C**H$_2$-Bn), 73.6, 73.6, 73.5, 72.9 (O-**C**H$_2$-Bn), 71.7 (O-**C**H$_2$-Bn), 71.6 (C-5), 71.2 (C-2; C-2''), 70.2 (-O**C**H$_2$(CH$_2$)$_5$N-; C-4'), 69.1 (C-6''), 67.8 (C-4''), 67.3 (C-5'''), 67.2 (C-4; -**C**H$_2$-(Cbz)), 62.5 (C-6), 61.6 (C-6'''), 59.5 (C-6'), 50.4 (-**C**H$_2$-(NBn)), 50.1 (-**C**H$_2$-(NBn)), 47.0 (-O(CH$_2$)$_5$**C**H$_2$N-), 46.1 (-O(CH$_2$)$_5$**C**H$_2$N-), 29.8 (-(**C**H$_2$)$_4$-), 29.4 (-(**C**H$_2$)$_4$-), 29.3 (-(**C**H$_2$)$_4$-), 27.9 (-(**C**H$_2$)$_4$-), 27.5 (-(**C**H$_2$)$_4$-), 26.4 (-(**C**H$_2$)$_4$-), 25.6 (-(**C**H$_2$)$_4$-), 22.8 (-(**C**H$_2$)$_4$-), residual acetone (δ 31.0); EtOH (δ 14.2). *Note: Cis-trans isomerism of the Cbz carbamate moiety results in split ^{13}C signal for N-**C**H$_2$-Ph; N-**C**H$_2$(CH$_5$)O-;* (**C**H$_2$)$_4$. *Broadening of corresponding -C**H**$_2$- groups in ^{1}H-NMR. Note 2: The configuration of the anomeric carbons C-1, C-1', C-1'' and C-1''' was assigned with additional information from non-decoupled ^{1}H-^{13}C HSQC (vide infra, ^{1}H-^{13}C non-decoupled HSQC spectrum **40**): 102.4 (d, J=162Hz, C-1 (β)), 101.9 (d, J=158Hz, C-1'' (β)), 98.5 (d, J=169Hz, C-1''' (α)), 96.2 (d, J=169Hz, C-1' (α)).*

N-benzyl-N-benzyloxycarbonyl-6-aminohexyl 4,6-di-O-benzoyl-2,3-di-O-benzyl-α-D-galactopyranosyl-(1→3)-2-O-benzoyl-4,6-di-O-benzyl-β-D-galactopyranosyl-(1→4)-2,3-di-O-benzyl-6-O-sulfo-α-D-galactopyranosyl-(1→3)-2,4,6-tri-O-benzoyl-β-D-galactopyranoside (41)

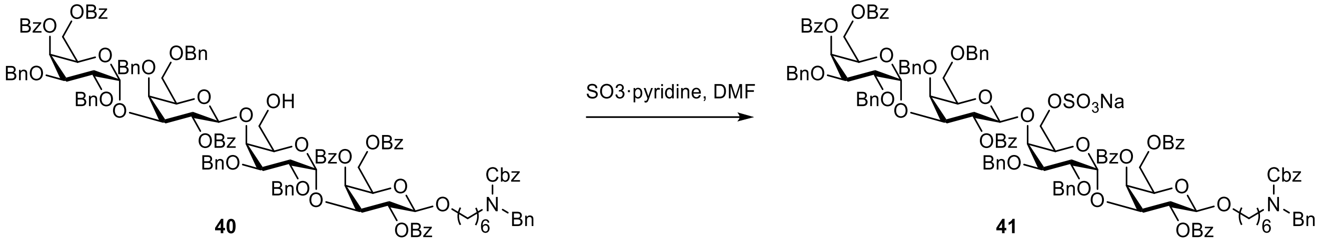

Compound **40** (24.0 mg, 11.1 μmol) and SO$_3$·pyridine (17.7 mg, 111 μmol) were dried under high vacuum overnight. The mixture was dissolved in anhydrous DMF (1 ml). The reaction mixture was stirred at RT for 21 h until completion (as indicated by TLC, 5% MeOH in DCM, R_f=0.29). The reaction was quenched by the addition of MeOH (~3 ml) and concentrated under reduced pressure. The crude product was purified by flash chromatography (elution at 5% MeOH in DCM) and Dowex 50W X8 ion exchange resin (Na-form) was added directly to the eluted fraction. The fraction was filtered and the filtrate evaporated under reduced pressure to obtain a yellowish residue. The residue was mostly dissolved in 5% MeOH in DCM and filtered through a cotton plug. The filtrate was evaporated to obtain pure **41** as a white solid (20.6 mg, 82% yield). HRMS (ESI-QTOF) m/z: [M+Na]$^+$ Calcd for $C_{129}H_{128}NNa_2O_{32}S$ 2280.7930; found 2280.7866. ^{1}H NMR (500 MHz, d_7-DMF): δ , ppm 8.38 - 8.34 (m, 2H), 8.01 - 7.97 (m, 2H), 7.91 - 7.88 (m, 2H), 7.87 - 7.83 (m, 4H), 7.69 - 7.62 (m, 4H), 7.55 - 7.42 (m, 16H), 7.37 - 7.23 (m, 24H), 7.21 - 7.15 (m, 6H), 7.08 - 7.02 (m, 3H), 6.92 (t, J = 7.6 Hz, 2H), 6.55 (d,

J = 7.2 Hz, 2H), 6.02 (d, $J_{3,4}$ = 2.7 Hz, 1H, H-4), 5.66 (m, 1H, H-4'''), 5.56 - 5.50 (m, 3H, H-2''; H-1'''(Galα); H-2), 5.28 (d, $J_{1,2}$ = 3.3 Hz, 1H, H-1'(Galα)), 5.19 - 5.15 (m, 2H, -C***H***$_2$-(Cbz)), 5.14 (d, $J_{1,2}$ = 7.9 Hz, 1H, H-1 (Galβ)), 5.10 (d, J_{AB} = 11.7 Hz, 1H, O-C***H***$_2$-Ar), 4.89 (dd, $J_{3,4}$ = 2.9 Hz, $J_{2,3}$ = 10.5 Hz, 1H, H-3), 4.83 (d, $J_{1,2}$ = 7.9 Hz, 1H, H-1''(Galβ)), 4.80 (s, 2H, O-C***H***$_2$-Ar), 4.68 (d, J_{AB} = 12.0 Hz, 1H, O-C***H***$_2$-Ar), 4.62 (d, J_{AB} = 11.5 Hz, 1H, O-C***H***$_2$-Ar), 4.58 (d, J_{AB} = 12.0 Hz, 1H, O-C***H***$_2$-Ar), 4.56 (d, J_{AB} = 11.7 Hz, 1H, O-C***H***$_2$-Ar), 4.52 (dd, J_{AB} = 10.4 Hz, $J_{5,6A}$ = 6.7 Hz, 1H, H-6A), 4.50 - 4.46 (m, 3H, -C***H***$_2$-(NBn); H-5), 4.45 (d, J_{AB} = 11.5 Hz, 1H, O-C***H***$_2$-Ar), 4.41 (dd, J_{AB} = 10.4 Hz, $J_{5,6B}$ = 5.5 Hz, 1H, H-6B), 4.37 - 4.33 (m, 2H, H-5'; H-5'''), 4.30 (dd, J_{AB} = 12.3 Hz, $J_{5,6}$ = 3.0 Hz, 1H, H-6'A), 4.23 (d, $J_{3,4}$ = 2.3 Hz, 1H, H-4''), 4.20 (dd, $J_{3,4}$ = 2.6 Hz, $J_{2,3}$ = 10.2 Hz, 1H, H-3''), 4.10 - 4.04 (m, 4H, O-C***H***$_2$-Ar; H-2'''; H-6'B; H-3'''), 4.01 (dd, J_{AB} = 10.7 Hz, $J_{5,6A}$ = 8.5 Hz, 1H, H-6'''A), 3.93 (d, J_{AB} = 11.5 Hz, 1H, O-C***H***$_2$-Ar), 3.89 - 3.85 (m, 2H, H-6'''B; -OC***H***$_2$(CH$_2$)$_5$N-), 3.82 - 3.68 (m, 5H, O-C***H***$_2$-Ar; H-5''; H-6''; H-4'), 3.53 - 3.48 (m, 2H, O-C***H***$_2$-Ar; -OC***H***$_2$(CH$_2$)$_5$N-), 3.41 (dd, $J_{1,2}$ = 3.3 Hz, $J_{2,3}$ = 10.0 Hz, 1H, H-2'), 3.24 (dd, $J_{3,4}$ = 2.5 Hz, $J_{2,3}$ = 10.0 Hz, 1H, H-3'), 3.12 - 3.09 (br, 2H, -O(CH$_2$)$_5$C***H***$_2$N-), 1.43 - 1.39 (m, 2H, -(C***H***$_2$)$_4$-), 1.33 - 1.27 (m, 2H, -(C***H***$_2$)$_4$-), 1.14 - 1.05 (m, 4H, -(C***H***$_2$)$_4$-), grease (δ 1.50 – 1.00), H_2O (δ 3.51). ^{13}C{^{1}H} NMR (126 MHz, d_7-DMF): δ, ppm 166.8 (**C**=O(Bz)), 166.6 (**C**=O(Bz)), 166.4 (**C**=O(Bz)), 166.4 (**C**=O(Bz); **C**=O(Bz)), 166.2 (**C**=O(Bz)), 157.4 (**C**=O(Cbz)), 140.6, 140.1, 139.9, 139.8, 139.7, 139.7, 139.6, 138.6, 134.6, 134.5, 134.2, 134.0, 131.5, 131.3, 131.0, 131.0, 130.9, 130.9, 130.7, 130.6, 130.5, 130.0, 129.9, 129.8, 129.8, 129.7, 129.6, 129.5, 129.5, 129.4, 129.3, 129.2, 129.1, 129.0, 128.9, 128.9, 128.7, 128.6, 128.6, 128.5, 128.2, 128.2, 127.8, 102.6 (C-1''(Galβ)), 102.4 (C-1(Galβ)), 98.7 (C-1'''(Galα)), 92.9 (C-1'(Galα)), 80.4 (C-3''), 79.4 (C-3'), 77.7 (C-2'), 77.2 (C-2'''/3'''), 77.0 (C-2'''/3'''), 75.8, 75.7, 75.4, 74.7, 74.4, 74.2, 73.4, 72.9, 72.6, 72.4, 71.7, 71, 70.5, 70.4 (-O**C**H$_2$(CH$_2$)$_5$N-), 70.0 (C-6''), 69.4 (C-4'''), 68.0 (C-6'), 67.9, 67.8 (C-4), 67.6 (-**C**H$_2$-(Cbz)), 63.8 (C-6), 62.6 (C-6'''), 51.2 (-**C**H$_2$-(NBn)), 50.9 (-**C**H$_2$-(NBn)), 48.1 (-O(CH$_2$)$_5$**C**H$_2$N-), 47.3 (-O(CH$_2$)$_5$**C**H$_2$N-), 29.1, 28.6, 28.1, 27.3, 26.5, 23.6, 14.7, 0.5. *Note: The ^{1}H-NMR signals at δ 3.53 - 3.48 were masked by the H_2O signal (δ 3.51) and were assigned with additional information from ^{1}H-^{13}C HSQC. Note: Cis-trans isomerism of the Cbz carbamate moiety results in split ^{13}C signal for N-**C**H$_2$-Ph; N-**C**H$_2$(CH$_5$)O-; (**C**H$_2$)$_4$. Broadening of corresponding -C**H$_2$**-groups in ^{1}H-NMR. Note 2: The configuration of the anomeric carbons C-1, C-1', C-1'' and C-1''' was assigned with additional information from non-decoupled ^{1}H-^{13}C HSQC (vide infra, ^{1}H-^{13}C non-decoupled HSQC spectrum **41**): 102.6 (d, J=160Hz, C-1'' (β)), 102.4 (d, J=161Hz, C-1 (β)), 98.7 (d, J=170Hz, C-1''' (α)), 92.9 (d, J=172Hz, C-1' (α)).*

6-Aminohexyl α-D-galactopyranosyl-(1→3)-β-D-galactopyranosyl-(1→4)-6-O-sulfo-α-D-galactopyranosyl-(1→3)-β-D-galactopyranoside (mST)

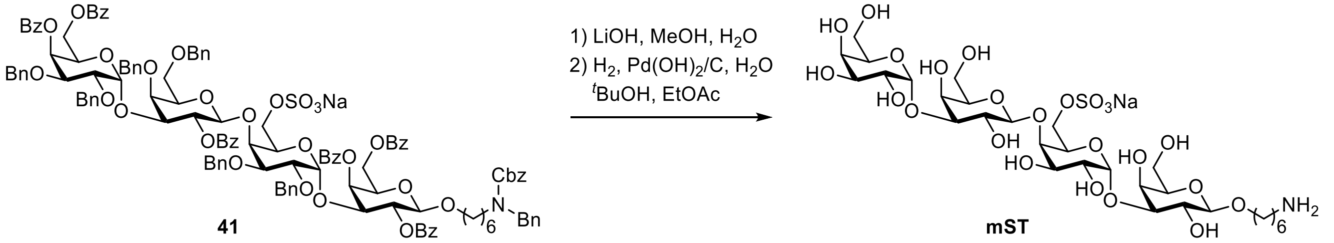

Compound **41** (18.6 mg, 8.24 µmol) was dissolved in THF (0.4 ml) and MeOH (0.2 ml), and cooled to 0°C in an ice-water bath. Aqueous lithium hydroxide (LiOH) (2 M, 0.6 ml) was added dropwise. The reaction mixture was stirred at RT for 16 h. The reaction mixture was turbid with visibly insoluble matter. MeOH (0.1 ml) and aqueous LiOH (1 M, 0.1 ml) were added, resulting in dissolution of the insoluble residue. The reaction mixture was stirred at RT for 5 h, then another portion of aqueous LiOH (2 M, 0.2 ml) was added. The reaction mixture was stirred overnight at RT (42 h total) until completion (as indicated by TLC, 10% MeOH in DCM, R_f=0.39). Ion exchange beads Amberlight® XAD16N (H-form, pre-washed in DCM and MeOH) were added in small portions until neutral pH was reached and reaction mixture turned clear. The reaction mixture was filtered and Dowex 50W X8 ion exchange resin (Na-form) was added to the filtrate. The mixture was

filtered again, and the filtrate was concentrated under reduced pressure, then dried under high vacuum. The crude product was dissolved in a minimal amount of ACN and TDW (50% ACN, ~0.1 ml) and passed through Orbilica pre-packed SPE cartridges (200 mg/3 ml, C18). The cartridge was conditioned in 85% ACN in TDW, then equilibrated in 10% ACN in TDW. The crude product was distributed equally between three SPE cartridges and washed with 10% ACN, then dried with Ar. The cartridges were extracted with 85% ACN (~3 ml) and the combined eluted fraction evaporated. The crude product was re-dissolved in ^{t}BuOH, EtOAc and TDW (~10:1:1) and filtered through a cotton plug to remove a yellowish insoluble residue. The filtrate was evaporated to produce a colorless glassy solid that was used directly in the next step. HRMS (ESI-QTOF) m/z: $[M\text{-}Na+2H]^+$ Calcd for $C_{87}H_{103}NO_{26}S$ 1610.6562; found 1610.6498. The crude product and $Pd(OH)_2/C$ (10-20% wt. loading, 24 mg) were suspended in EtOAc, ^{t}BuOH and TDW (0.5:1:0.5, 2 ml). The mixture was stirred in a pressurized reaction vessel under 4 bar of H_2 at RT for 19 h. The reaction mixture was filtered through celite, and the celite was washed with ^{t}BuOH and TDW (1:1), then TDW. The combined filtrate was concentrated under reduced pressure. The crude product was dissolved in a minimal amount of ACN and TDW (5% ACN, ~50 μl) and passed through an a Orbilica pre-packed SPE cartridge (200 mg/3 ml, C18). The cartridge was conditioned in 85% ACN in TDW, then equilibrated in TDW. The crude product was loaded onto the SPE cartridge and eluted with TDW (~2 ml). The collected fraction containing **mST** was lyophilized. The lyophilized fraction was dissolved in a sodium acetate buffer (pH = 6.0, 2 M, 50 μl) and incubated at RT for 2 h. Compound **mST** was purified by SEC on Sephadex G-10 size exclusion resin (160 mg, 80-270 mesh, swelled in TDW for 3 h). Tetrasaccharide **mST** was eluted with TDW within the first 1.5 column void volume (6.7 mg, 88% 2-step yield). HRMS (ESI-QTOF) m/z: $[M\text{-}Na+2H]^+$ Calcd for $C_{30}H_{56}NO_{24}S$ 846.2908; found 846.2932. ^{1}H NMR (700 MHz, D_2O): δ, ppm 5.18 (d, $J_{1,2}$ = 3.8 Hz, 1H, H-1^{II}(Galα)), 5.16 (d, $J_{1,2}$ = 3.8 Hz, 1H, H-1^{IV}(Galα)), 4.67 (d, $J_{1,2}$ = 7.9 Hz, 1H, H-1^{III}(Galβ)), 4.50 - 4.47 (m, 1H, H-5^{II}), 4.48 (d, $J_{1,2}$ = 7.9 Hz, 1H, H-1^{I}(Galβ)), 4.33 (dd, J_{AB} = 10.6 Hz, $J_{5,6}$ = 4.4 Hz, 1H, H-6^{II}A), 4.33 - 4.31 (m, 1H, H-4^{II}), 4.23 - 4.20 (m, 2H, H-6^{II}B; H-5^{IV}), 4.20 - 4.18 (m, 2H, H-4^{I}; H-4^{III}), 4.07 (dd, $J_{3,4}$ = 3.0 Hz, $J_{2,3}$ = 10.3 Hz, 1H, H-3^{II}), 4.03 (d, $J_{3,4}$ = 3.0 Hz, 1H, H-4^{IV}), 3.98 - 3.93 (m, 3H, H-2^{II}; H-3^{IV}; -OC***H*₂**(CH₂)₅NH₂), 3.89 - 3.84 (m, 2H, H-2^{IV}; H-6^{I}A), 3.83 - 3.67 (m, 11H, H-6^{I}B; H-6^{III}; H-6^{IV}; H-3^{I}; H-3^{III}; H-2^{III}; -OC***H*₂**(CH₂)₅NH₂; H-5^{III}; H-5^{I}), 3.62 (dd, $J_{1,2}$ = 8.2 Hz, $J_{2,3}$ = 9.7 Hz, 1H, H-2^{I}), 3.01 (t, J = 7.6 Hz, 2H, -O(CH₂)₅C***H*₂**NH₂), 1.92 (s, 8H), 1.71 - 1.64 (m, 4H, OCH₂C***H*₂**(CH₂)₂C***H*₂**CH₂NH₂), 1.46 - 1.40 (m, 4H, -O(CH₂)₂ (C***H*₂**)₂(CH₂)₂NH₂), acetate (~1:2.6 ratio, δ 1.92 (s)). ^{13}C{^{1}H} NMR (176 MHz, D_2O): δ, ppm 102.1 (C-1^{III}), 100.3 (C-1^{I}), 93.3 (C-1^{II}), 93.1 (C-1^{IV}), 76.5 (C-4^{II}), 75.5 (C-3$^{I/III}$), 75.2 (C-3$^{I/III}$), 72.7 (C-5$^{I/III}$), 72.6 (C-5$^{I/III}$), 68.7 (C-5^{IV}), 68.0 (-O**C**H₂(CH₂)₅NH₂), 67.9 (C-2^{III}), 67.5 (C-3^{II}), 67.1 (C-2^{I}/C-3^{IV}), 67.1 (C-2^{I}/C-3^{IV}), 67.0 (C-4^{IV}), 66.4 (C-2^{II}), 66.3 (C-5^{II}), 66.0 (C-2^{IV}), 65.5 (C-6^{II}), 62.8 (C-4$^{I/III}$), 62.6 (C-4$^{I/III}$), 58.8 (C-6$^{I/III/IV}$), 58.7 (C-6$^{I/III/IV}$), 58.7 (C-6$^{I/III/IV}$), 37.2 (-O(CH₂)₅**C**H₂NH₂), 26.1 (-(**C**H₂)₄-), 24.4 (-(**C**H₂)₄-), 23.0 (-(**C**H₂)₄-), 22.3 (-(**C**H₂)₄-), acetate (δ 179.3, 21.0). *Note: The sodium acetate buffer is used in excess (~10 equiv.) to produce a uniform aminium acetate and sodium sulfonate salt forms and remove anionic contaminants (e.g. benzoate). Note 2: The configuration of the anomeric carbons C-1^{I}, C-1^{II}, C-1^{III} and C-1^{IV} was assigned with additional information from non-decoupled ^{1}H-^{13}C HSQC (vide infra, ^{1}H-^{13}C non-decoupled HSQC spectrum **mST**): 102.1 (d, J=161Hz, C-1^{III} (β)), 100.3 (d, J=160Hz, C-1^{I} (β)), 93.3 (d, J=171Hz, C-1^{II} (α)), 93.1 (d, J=171Hz, C-1^{IV} (α)). Note 3: Compound **mST** was desalted by passing the product again through SEC Sephadex G-10 size exclusion resin (93% recovery).*

Electrochemical Sensing

Electrochemical analyses were performed on BioLogic SP-300 potentiostat in a standard three electrode electrochemical cell comprised of: 1) Ag/AgCl (in 3 M KCl) reference electrode (RE), 2) Pt wire counter electrode (CE), and 3) glassy carbon electrode (GCE), 3 mm diameter, as the working electrode (WE). GCEs were manually polished on a micro-cloth pad (Buehler) with de-agglomerated alumina slurry (particle size: 0.05 μm, Buehler) and washed with TDW. Controls 6-aminohexanol (**AH**) and **Gal**, and carrageenans (CGNs) **nSd**, **mSd**, **nSt**, **mSt**, **nST**, and **mST** were electrografted on freshly polished GCEs *via* their alkylamine handle by applying oxidative potential *via* five cycles of cyclic voltammetry (CV) to GCEs immersed in 1.3 μM solution of **AH**, **Gal**, and each CGN in aqueous 0.1 M KCl to obtain **GCE-AH**, **GCE-Gal**, **GCE-nSd**, **GCE-mSd**, **GCE-nSt**, **GCE-mSt**, **GCE-nST**, and **GCE-mST**, respectively. CV was applied between 0.6 V to 1.2 V against the RE at a scan rate of 10 mV/s. The CV plots of representative **GCE-AH**, **GCE-Gal**, **GCE-nSd**, **GCE-mSd**, **GCE-nSt**, **GCE-mSt**, **GCE-nST**, and **GCE-mST** electrodes are presented in Figure S1 and Figure S2.

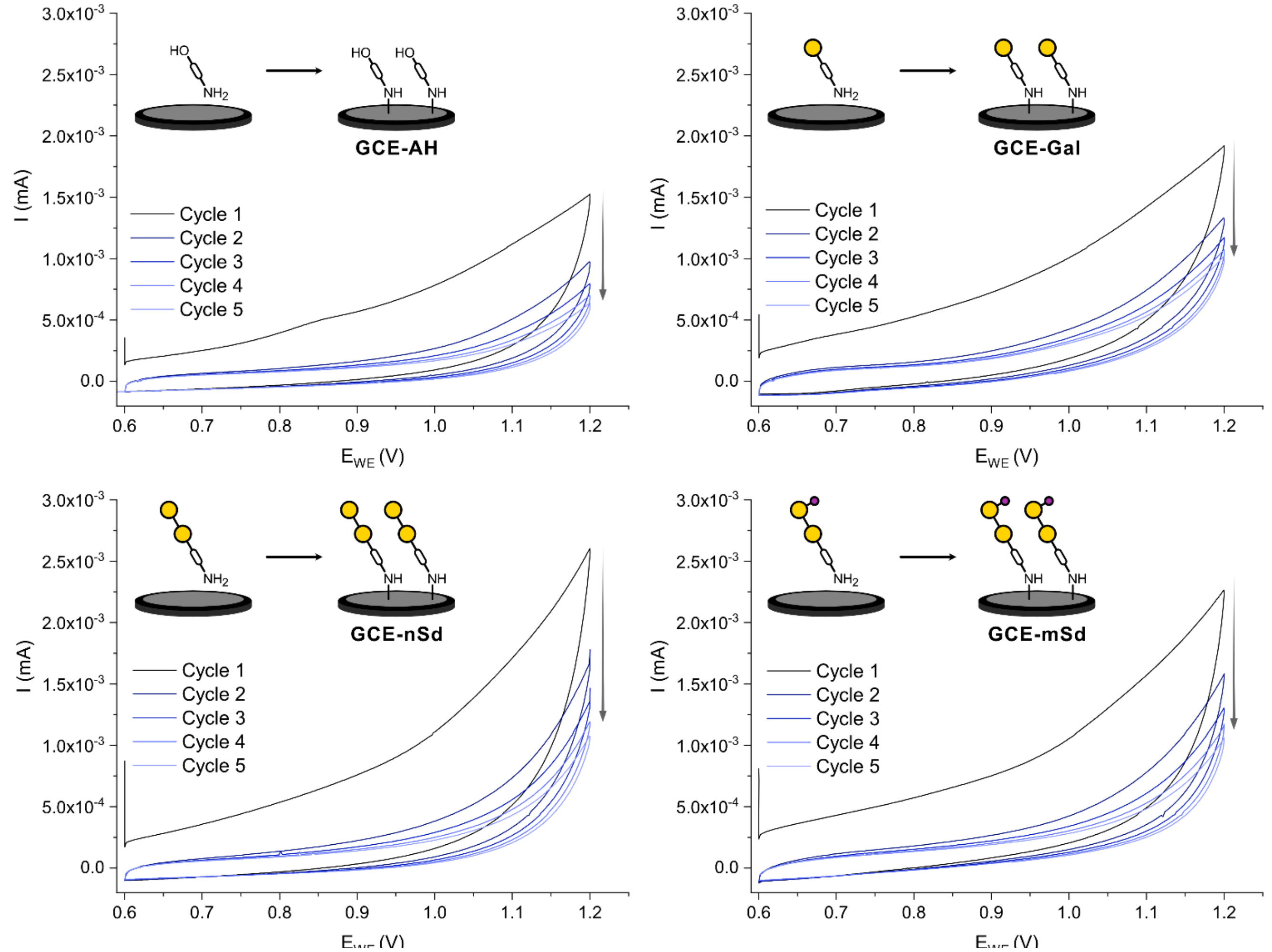

Figure S1. Representative CV plots of CGN electrografting onto GCEs to produce CGN-modified GCEs by applied potential from 0.6 V to 1.2 V conducted in 5 cycles. Top-left: **GCE-AH**. Top-right: **GCE-Gal**. Bottom-left: **GCE-nSd**. Bottom-right: **GCE-mSd**.

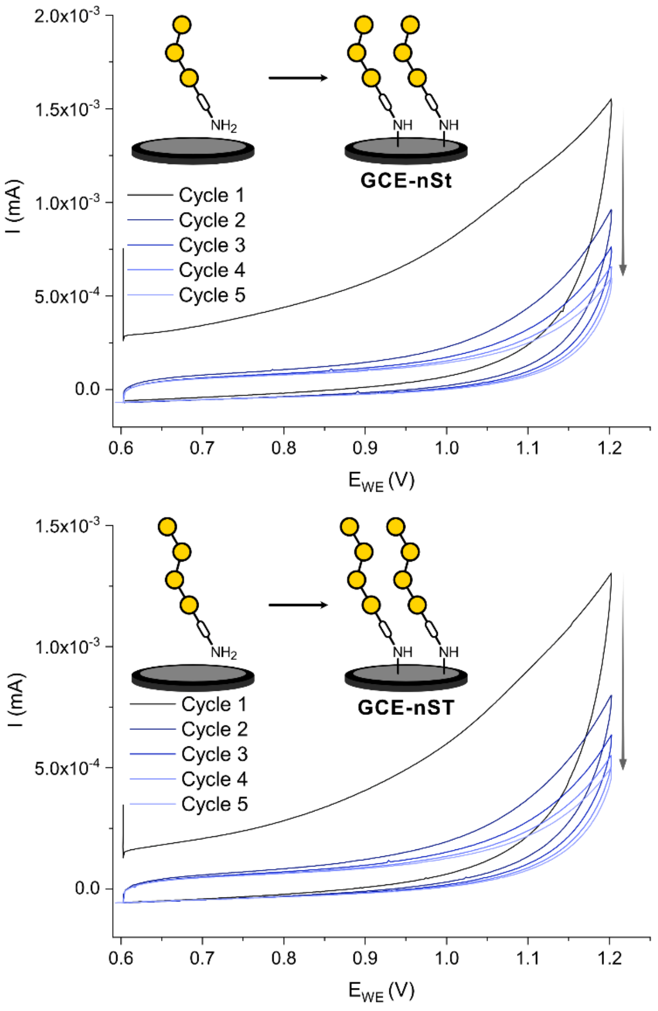

Figure S2. Representative CV plots of CGN electrografting onto GCEs to produce CGN-modified GCEs by applied potential from 0.6 V to 1.2 V conducted in 5 cycles. Top-left: **GCE-nSt**. Top-right: **GCE-mSt**. Bottom-left: **GCE-nST**. Bottom-right: **GCE-mST**.

After electrografting, CGN-modified GCEs were washed and kept in ammonium acetate (AA) buffer (50 mM in 0.1 M KCl, pH 6.7) for 60 min at 25°C. Electrochemical impedance spectroscopy (EIS) was measured in a solution of $[Fe(CN)_6]^{3-}/[Fe(CN)_6]^{4-}$ redox-active couple (1 mM/1 mM in AA buffer), by applying a single sine AC potential with 10 mV amplitude super-imposed with 0.21 V DC potential and scanning over the frequency range of 100 kHz to 0.1 Hz.

Recombinant human IL-8 (CXCL8) (77 aa) was purchased from PeproTech (Thermo Fisher Scientific), or prepared according to a previously reported work.[15] or The EIS response of CGN-modified GCEs to IL-8 was performed by: 1) incubating the electrodes in a 1 μM solution of IL-8 in AA buffer (50 mM in 0.1 M KCl, pH 6.7) for 60 min at 25°C, and 2) EIS measurement in a solution of $[Fe(CN)_6]^{3-}/[Fe(CN)_6]^{4-}$ redox-active couple (1 mM/1 mM in AA buffer), by applying a single sine AC potential with 10 mV amplitude super-imposed with 0.21 V DC potential and scanning over the frequency range of 100 kHz to 0.1 Hz. Each experiment was performed with five replicates. The Nyquist plots of the EIS response of representative **GCE-AH**, **GCE-Gal**, **GCE-nSd**, **GCE-mSd**, **GCE-nSt**, **GCE-mSt**, **GCE-nST**, and **GCE-mST** electrodes before and after exposure to IL-8 are presented in Figure S3 and Figure S4.

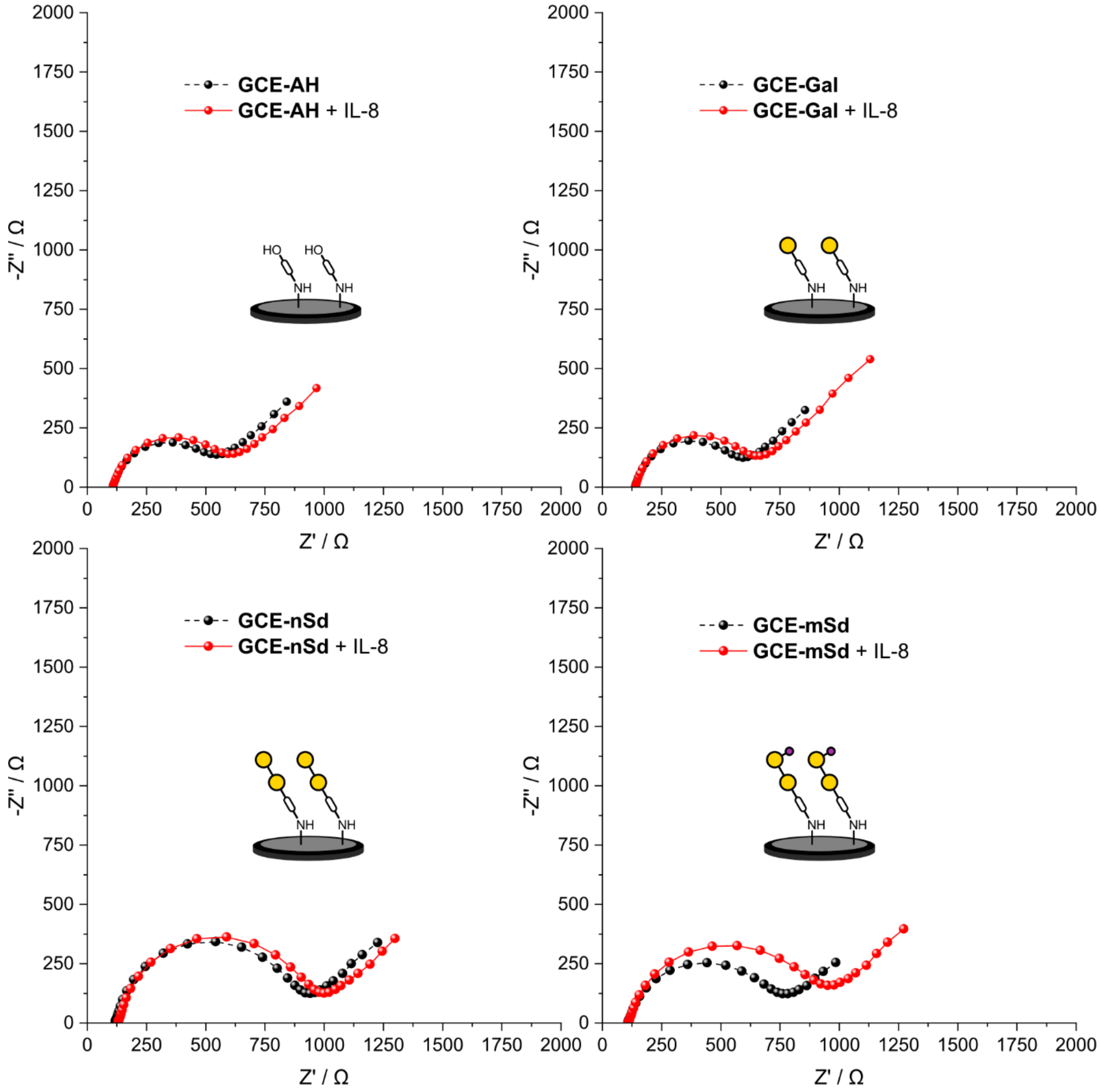

Figure S3. Representative Nyquist plots of the EIS response of CGN-modified GCEs prior (black, dashed) and after (red, solid) exposure to IL-8. Top-left: **GCE-AH**. Top-right: **GCE-Gal**. Bottom-left: **GCE-nSd**. Bottom-right: **GCE-mSd**.

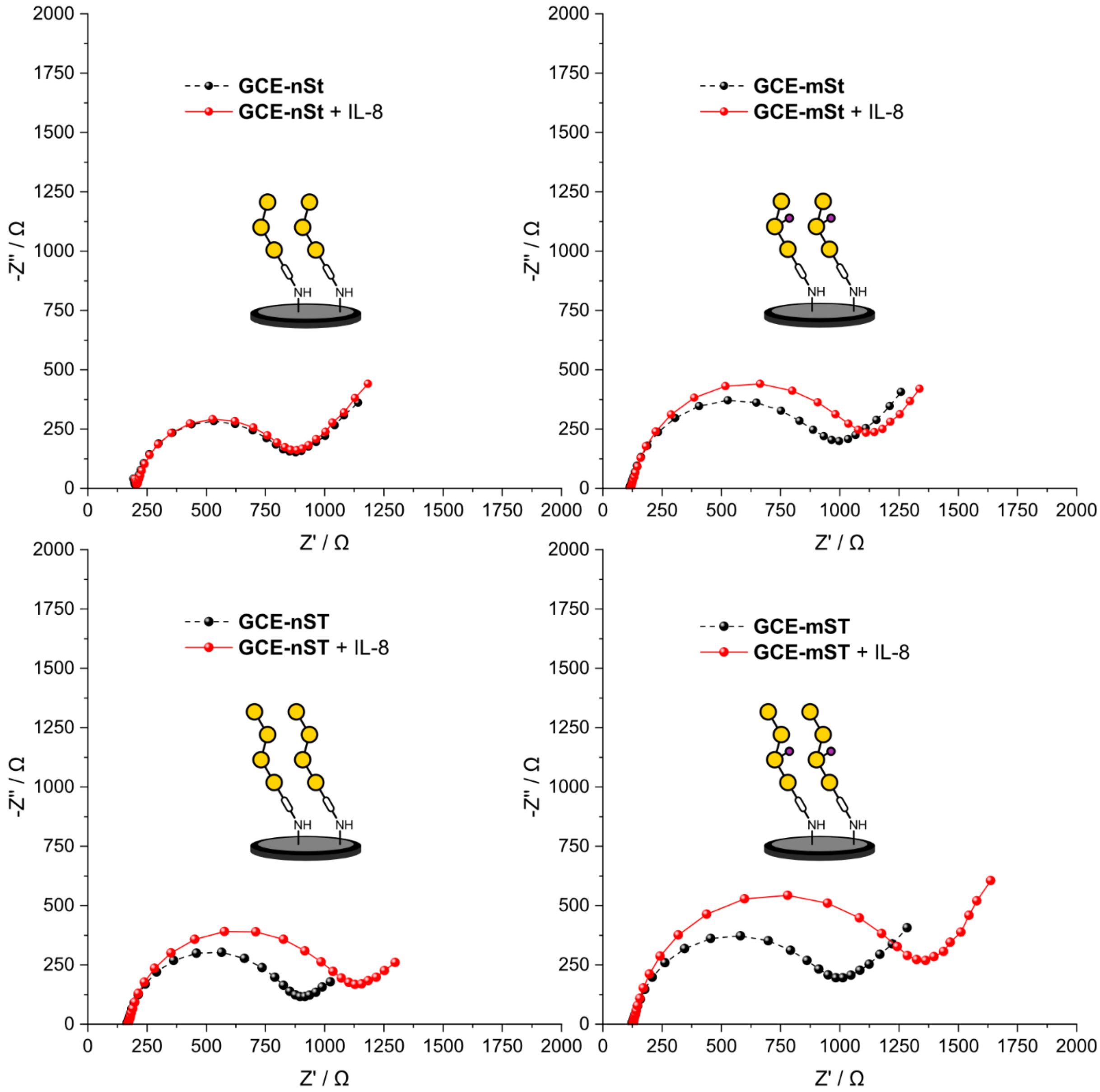

Figure S4. Representative Nyquist plots of the EIS response of CGN-modified GCEs prior (black, dashed) and after (red, solid) exposure to IL-8. Top-left: **GCE-nSt**. Top-right: **GCE-mSt**. Bottom-left: **GCE-nST**. Bottom-right: **GCE-mST**.

Nyquist plots of EIS measurements were fitted to the equivalent circuit of R_S [Q|(R_{CT} W)], where R_S is the solution resistance, R_{CT} is the interfacial resistance to charge transfer, W is the Warburg diffusion element, and Q is the constant phase element of nonideal capacitance. The R_{CT} values measured for **GCE-AH**, **GCE-Gal**, **GCE-nSd**, **GCE-mSd**, **GCE-nSt**, **GCE-mSt**, **GCE-nST** (five replicates of each), and **GCE-mST** (five replicates + four replicated after additional desalting) are summarized in Tables S3-S10, respectively.

Table S3. Measured R_{CT} values for GCE-HA electrodes before and after exposure to IL-8.

electrode	R_{CT} **GCE-HA** (Ω)	R_{CT} **GCE-HA** + IL-8 (Ω)	R_{CT} change (%)
1	550	474	-14
2	633	683	8
3	427	463	8
4	381	421	10
5	413	458	11
average	481 ± 95	500 ± 93	5 ± 9

Table S4. Measured R_{CT} values for GCE-Gal electrodes before and after exposure to IL-8.

electrode	R_{CT} **GCE-Gal** (Ω)	R_{CT} **GCE-Gal** + IL-8 (Ω)	R_{CT} change (%)
1	617	715	16
2	398	419	5
3	483	506	5
4	441	470	7
5	374	402	7
average	463 ± 86	500 ± 110	8 ± 4

Table S5. Measured R_{CT} values for GCE-nSd electrodes before and after exposure to IL-8.

electrode	R_{CT} **GCE-nSd** (Ω)	R_{CT} **GCE-nSd** + IL-8 (Ω)	R_{CT} change (%)
1	1180	1176	0
2	1613	1680	4
3	1528	1529	0
4	1542	1576	2
5	1092	1177	8
average	1390 ± 210	1430 ± 210	3 ± 3

Table S6. Measured R_{CT} values for GCE-mSd electrodes before and after exposure to IL-8.

electrode	R_{CT} **GCE-mSd** (Ω)	R_{CT} **GCE-mSd** + IL-8 (Ω)	R_{CT} change (%)
1	976	1316	35
2	540	618	14
3	701	798	14
4	591	691	17
5	611	795	30
average	680 ± 160	840 ± 250	22 ± 9

Table S7. Measured R_{CT} values for GCE-nSt electrodes before and after exposure to IL-8.

electrode	R_{CT} **GCE-nSt** (Ω)	R_{CT} **GCE-nSt** + IL-8 (Ω)	R_{CT} change (%)
1	770	873	13
2	787	794	1
3	613	626	2
4	396	446	13
5	411	443	8
average	600 ± 170	640 ± 180	7 ± 5

Table S8. Measured R_{CT} values for GCE-mSt electrodes before and after exposure to IL-8.

electrode	R_{CT} **GCE-mSt** (Ω)	R_{CT} **GCE-mSt** + IL-8 (Ω)	R_{CT} change (%)
1	1142	1310	15
2	870	1267	46
3	999	1552	55
4	815	1036	27
5	399	524	31
average	850 ± 250	1140 ± 350	35 ± 14

Table S9. Measured R_{CT} values for GCE-nST electrodes before and after exposure to IL-8.

electrode	R_{CT} **GCE-nST** (Ω)	R_{CT} **GCE-nST** + IL-8 (Ω)	R_{CT} change (%)
1	864	1184	37
2	665	933	40
3	770	886	15
4	734	1034	41
5	639	870	36
average	730 ± 80	980 ± 120	34 ± 10

Table S10. Measured R_{CT} values for GCE-mST electrodes before and after exposure to IL-8.

electrode	R_{CT} **GCE-mST** (Ω)	R_{CT} **GCE-mST** + IL-8 (Ω)	R_{CT} change (%)
1	948	1290	36
2	865	1143	32
3	680	905	33
4	471	720	53
5	646	1080	67
6	699	988	41
7	447	787	76
8	815	1146	41
9	601	740	23
average	690 ± 160	980 ± 190	45 ± 16

X-ray Photoelectron Spectroscopy Analysis

GCEs with a diameter of 7 mm were manually polished on a micro-cloth pad (Buehler) with de-agglomerated alumina slurry (particle size: 0.05 μm, Buehler) and washed with TDW. The 7 mm **GCE-mSd** electrodes were prepared by electrografting in a solution of **mSd** by applying oxidative potential *via* five cycles of CV (as detailed earlier, see previous section). CGN-modified electrodes were exposed to IL-8 to produce **GCE-mSd** + IL-8 7 mm GCEs (as detailed earlier, see previous section). X-ray photoelectron spectroscopy (XPS) measurements were performed on the CGN-modified electrodes **GCE-mSd** + IL-8 and **GCE-mSd** (with and without exposure to IL-8, respectively, three replicates each) by utilizing Axis Supra+ spectrometer (Kratos Analytical Ltd., Manchester, U.K.) with Al Kα monochromatic x-ray source (1486.7 eV). The XPS spectra were obtained with a takeoff angle of 90° (normal to analyzer) at vacuum 1.9 nTorr in the chamber. High-resolution XPS spectra were acquired with a pass energy of 20 eV and step size of 0.1 eV. The binding energies (BEs) were calibrated according to the C_{1s} peak position (285.0 eV). Data were collected and analyzed by using Casa XPS (Casa Software Ltd.) and Vision data processing program (Kratos Analytical Ltd.). The average atomic concentrations calculated for three replicates and the percentage change with and without exposure to IL-8 are presented in Table S11.

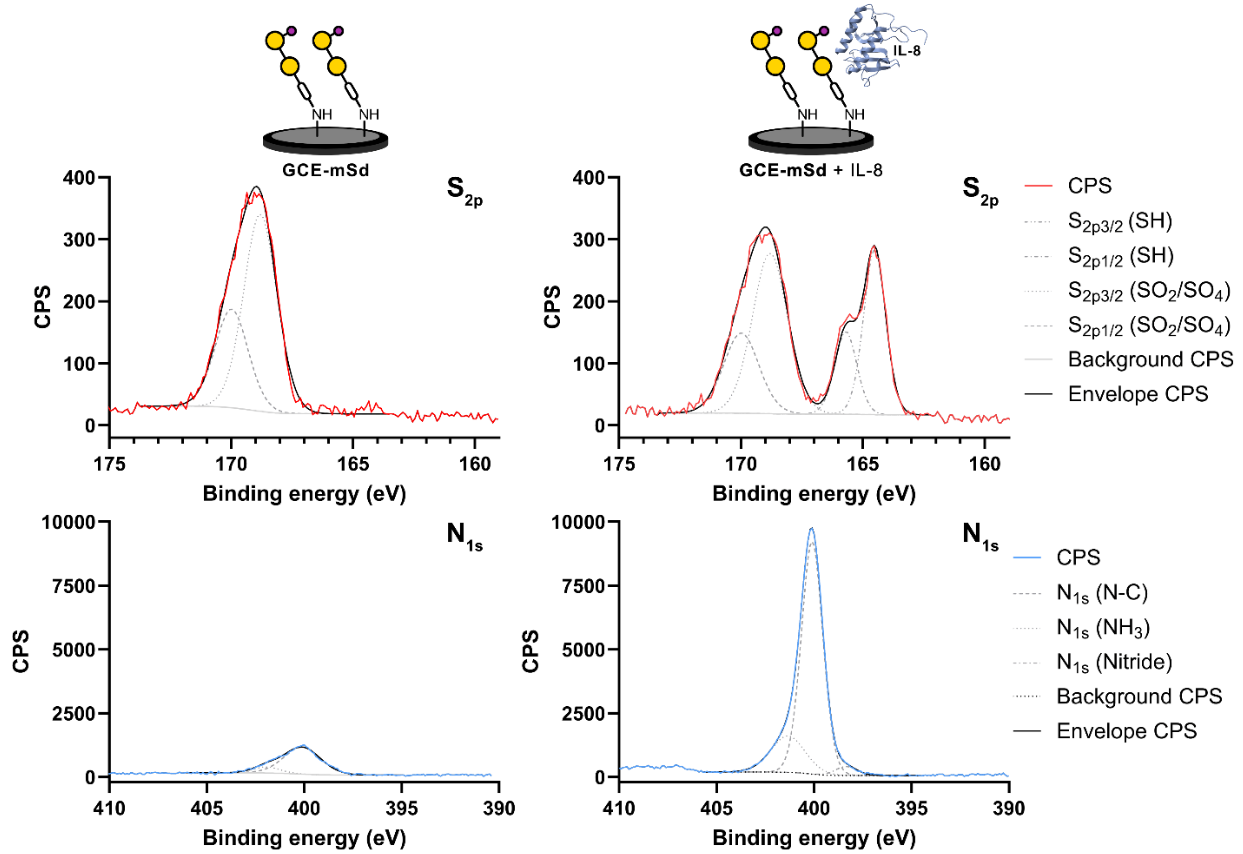

Figure S5. Representative XPS spectra of **GCE-mSd** (left panels) and **GCE-mSd** + IL-8 (right panels), in the S_{2p} BEs (top panels) and the N_{1s} BEs (bottom panels). The Y-axis offset of the spectra was zero-corrected. For S_{2p} the spectra signals at characteristic BEs of SH and SO_4 species were fitted with two components (2p3/2 and 2p1/2) with a 2:1 ratio and 1.2 eV spin-orbit splitting. For N_{1s} the spectra were fitted with characteristic BEs of N-C, NH_3, and Nitride.

Table S11. Surface atomic concentration quantified for S_{2p}, O_{1s}, N_{1s}, and C_{1s}

electrode	S (%)	N (%)	O (%)	C (%)
GCE-mSd'	0.31 ± 0.02	1.46 ± 0.08	9.8 ± 0.1	88.4 ± 0.2
GCE-mSd'+IL-8	0.5 ± 0.1	6.6 ± 0.4	16 ± 4	76 ± 4
change (%)	61	352	63	-14

References

1. Babij, N. R. *et al.* NMR Chemical Shifts of Trace Impurities: Industrially Preferred Solvents Used in Process and Green Chemistry. *Org. Process Res. Dev.* **20**, 661–667 (2016).

2. O'Neil, M. J. & Co., M. &. *The Merck Index : An Encyclopedia of Chemicals, Drugs, and Biologicals*. (Merck, Whitehouse Station, N.J, 2006).

3. Sukhran, Y. *et al.* Unexpected Nucleophile Masking in Acyl Transfer to Sterically Crowded and Conformationally Restricted Galactosides. *J. Org. Chem.* **88**, 9313–9320 (2023).

4. Das, S. K. & Roy, N. An improved method for the preparation of some ethyl 1-thioglycosides. *Carbohydr. Res.* **296**, 275–277 (1996).

5. Vargas-Berenguel, A., Meldal, M., Paulsen, H., Jensena, K. J. & Bock, K. Synthesis of glycosyltyrosine building blocks for solid-phase glycopeptide assembly: use of aryl tert-butyl ethers as glycosyl acceptors in aromatic glycosylations. *J. Chem. Soc. Perkin Trans. 1* 3287–3294 (1994) doi:10.1039/P19940003287.

6. Budhadev, D. & Mukhopadhyay, B. Chemical synthesis of the pentasaccharide related to the repeating unit of the O-antigen of Enterobacter cloacae G2277. *Tetrahedron* **71**, 6155–6163 (2015).

7. Zhang, Z. *et al.* Programmable one-pot oligosaccharide synthesis. *J. Am. Chem. Soc.* **121**, 734–753 (1999).

8. Liang, X.-Y. *et al.* TsOH-catalyzed acyl migration reaction of the Bz-group: innovative assembly of various building blocks for the synthesis of saccharides. *Org. Biomol. Chem.* **21**, 1537–1548 (2023).

9. Li, T. *et al.* Catalytic Regioselective Benzoylation of 1,2-trans-Diols in Carbohydrates with Benzoyl Cyanide: The Axial Oxy Group Effect and the Action of Achiral and Chiral Amine Catalysts. *ACS Catal.* **10**, 11406–11416 (2020).

10. Shitrit, A. *et al.* Monosaccharide-Derived Enantioselectivity in SWCNT Chemoresistive VOC Sensing. *Chem. – A Eur. J.* **31**, e02553 (2025).

11. Verma, N. *et al.* Threshold of Thioglycoside Reactivity Difference Is Critical for Efficient Synthesis of Type i Oligosaccharides by Chemoselective Glycosylation. *J. Org. Chem.* **86**, 892–916 (2021).

12. Bartetzko, M. P., Schuhmacher, F., Hahm, H. S., Seeberger, P. H. & Pfrengle, F. Automated Glycan Assembly of Oligosaccharides Related to Arabinogalactan Proteins. *Org. Lett.* **17**, 4344–4347 (2015).

13. Li, Z. & Gildersleeve, J. C. Mechanistic Studies and Methods To Prevent Aglycon Transfer of Thioglycosides. *J. Am. Chem. Soc.* **128**, 11612–11619 (2006).

14. Bakhatan, Y. *et al.* Translating solution to solid phase glycosylation conditions. *Chem. Commun.* **58**, 11256–11259 (2022).

15. Pichert, A. *et al.* Characterization of the interaction of interleukin-8 with hyaluronan, chondroitin sulfate, dermatan sulfate and their sulfated derivatives by spectroscopy and molecular modeling. *Glycobiology* **22**, 134–145 (2012).
