# Supplementary material for: Total Synthesis of Conjugation-Ready Sulfated Red Algae Carrageenan Oligosaccharides for Sensing Applications
Source: J Am Chem Soc. 2026 Jun 10;148(27):29430–40. doi: 10.1021/jacs.6c09827 (PMC13383626; doi:10.1021/jacs.6c09827)
Supplement: Supplementary file 2 [file ja6c09827_si_002.pdf]

Supporting Information

Total Synthesis of Conjugation-Ready Sulfated Red Algae Carrageenan Oligosaccharides for Sensing Applications

Yonatan Sukhran, Roey G. Meir, Israel Alshanski, Shlomo Yitzchaik, Mattan Hurevich*.

Institute of Chemistry and Center for Nanoscience and Nanotechnology, The Hebrew University of Jerusalem, Safra Campus, Givat Ram, Jerusalem 9190401, Israel.

E-mail: Mattan.Hurevich@mail.huji.ac.il

Table of Contents

Spectroscopic data 4

Compound **Gal** 4

Compound **nSd** 8

Compound **mSd** 15

Compound **nSt** 23

Compound **mSt** 31

Compound **nST** 39

Compound **mST** 47

Compound **1** 55

Compound **2** 60

Compound **3** 66

Compound **4** 72

Compound **5** 77

Compound **6** 83

Compound **7** 87

Compound **8** 96

Compound **9** 101

Compound **10** 106

Compound **11** 110

Compound **12** 114

Compound **13** 118

Compound **14** 123

Compound **15** 129

Compound **16** ... 134
Compound **17** ... 138
Compound **19** ... 143
Compound **20** ... 148
Compound **21** ... 154
Compound **22** ... 159
Compound **23** ... 164
Compound **24** ... 169
Compound **25** ... 175
Compound **26a** ... 181
Compound **26b** ... 186
Compound **28** ... 191
Compound **29** ... 196
Compound **30** ... 202
Compound **31** ... 208
Compound **32** ... 213
Compound **33** ... 219
Compound **34** ... 225
Compound **35** ... 231
Compound **36** ... 237
Compound **37** ... 243
Compound **38** ... 248
Compound **39** ... 253
Compound **40** ... 259
Compound **41** ... 265
Compound **S1** ... 271
Compound **S2** ... 275
Compound **S3** ... 280
Compound **S4a** ... 284
Compound **S4b** ... 289
Compound **S5** ... 294
Compound **S6** ... 299

Compound **S7** 304
Compound **S7’** 310
Compound **S8** 316
Compound **S9** 321
Compound **S10** 326
Compound **S11** 331
Compound **S12** 336

Spectroscopic data

Compound **Gal**

^{1}H-NMR

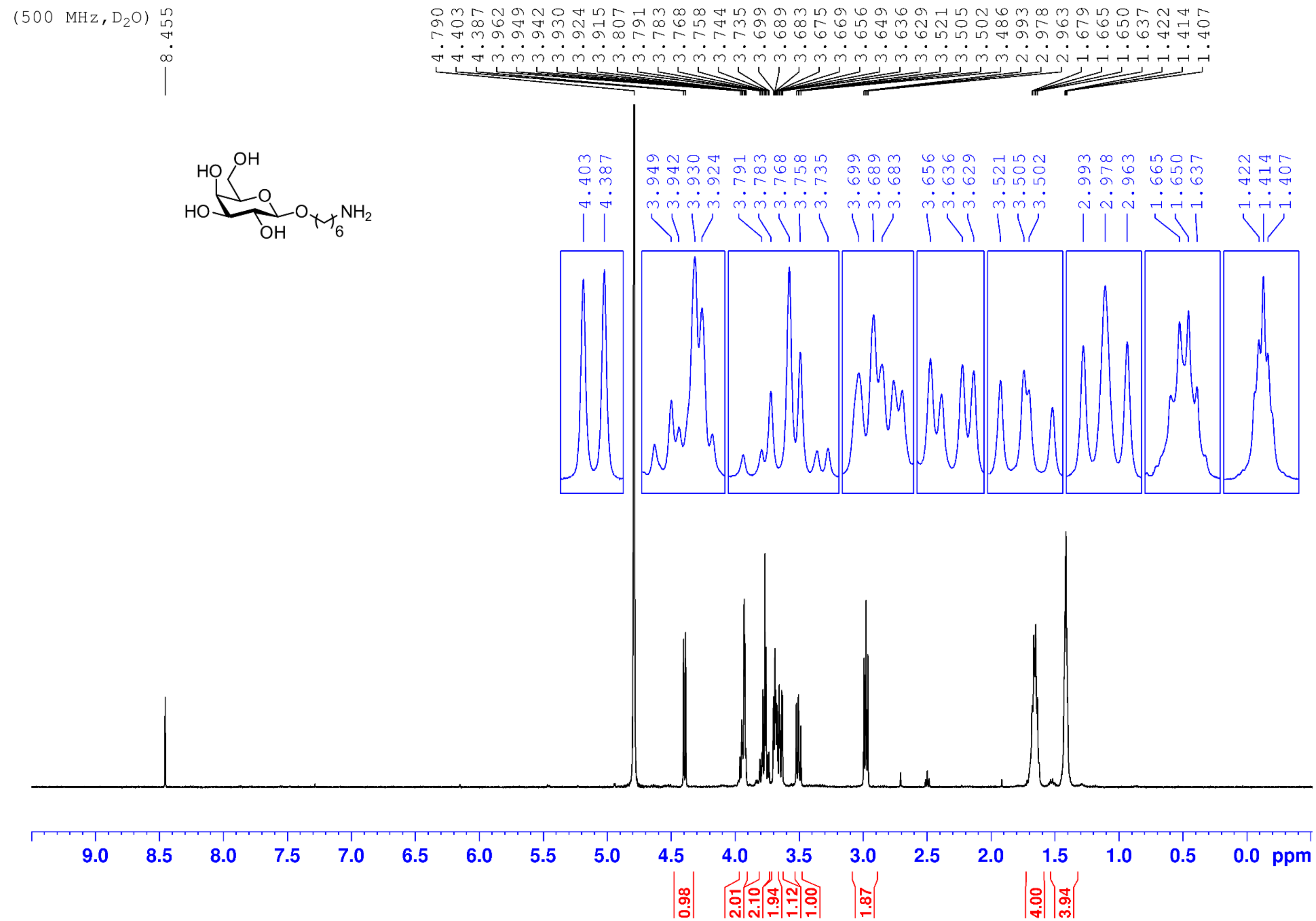

^{1}H-^{1}H COSY

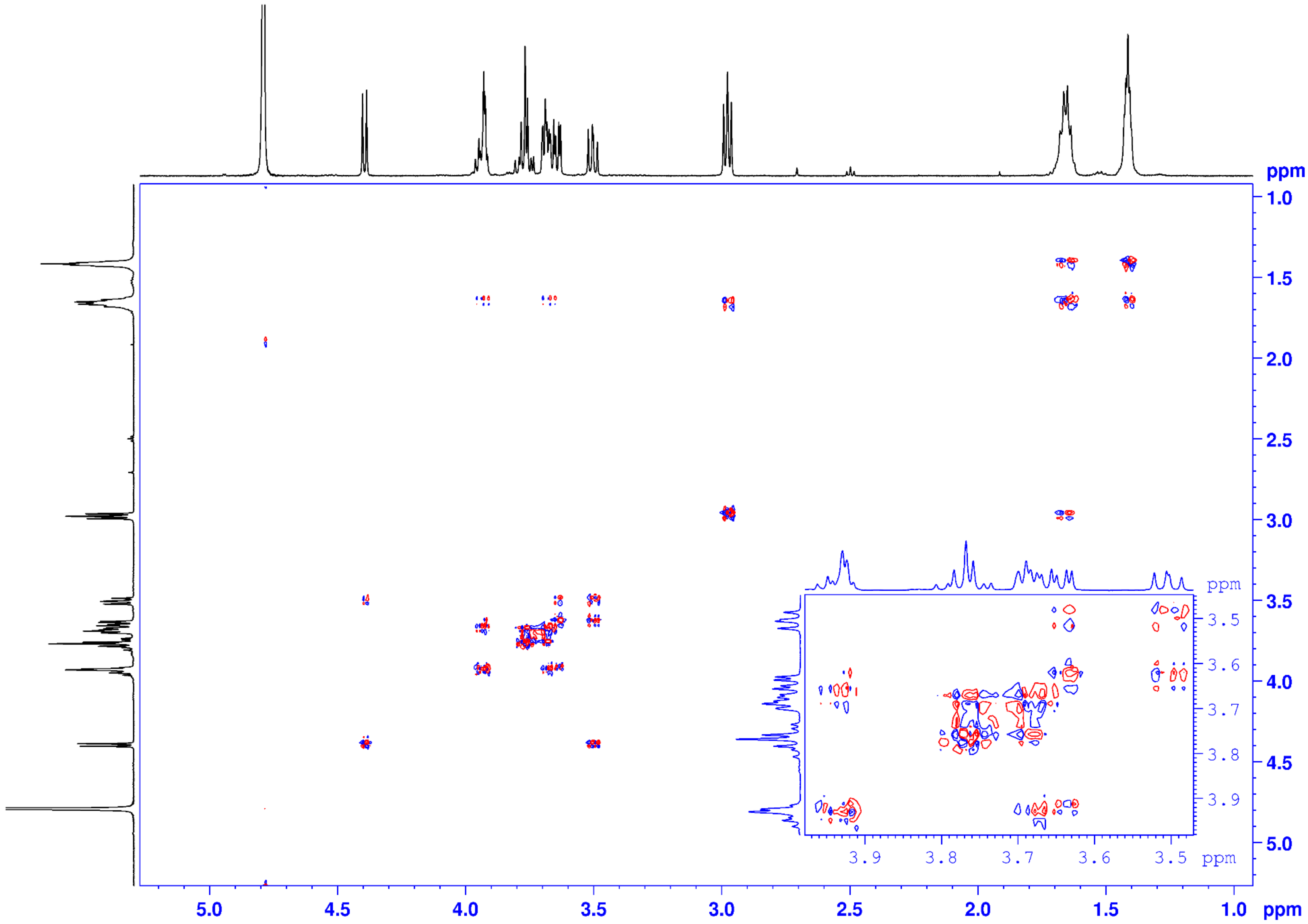

ppm
1.0
1.5
2.0
2.5
3.0
3.5
4.0
4.5
5.0
5.0
4.5
4.0
3.5
3.0
2.5
2.0
1.5
1.0
ppm
3.5
3.6
3.7
3.8
3.9
3.9
3.8
3.7
3.6
3.5
ppm

^1H-^{13}C HSQC

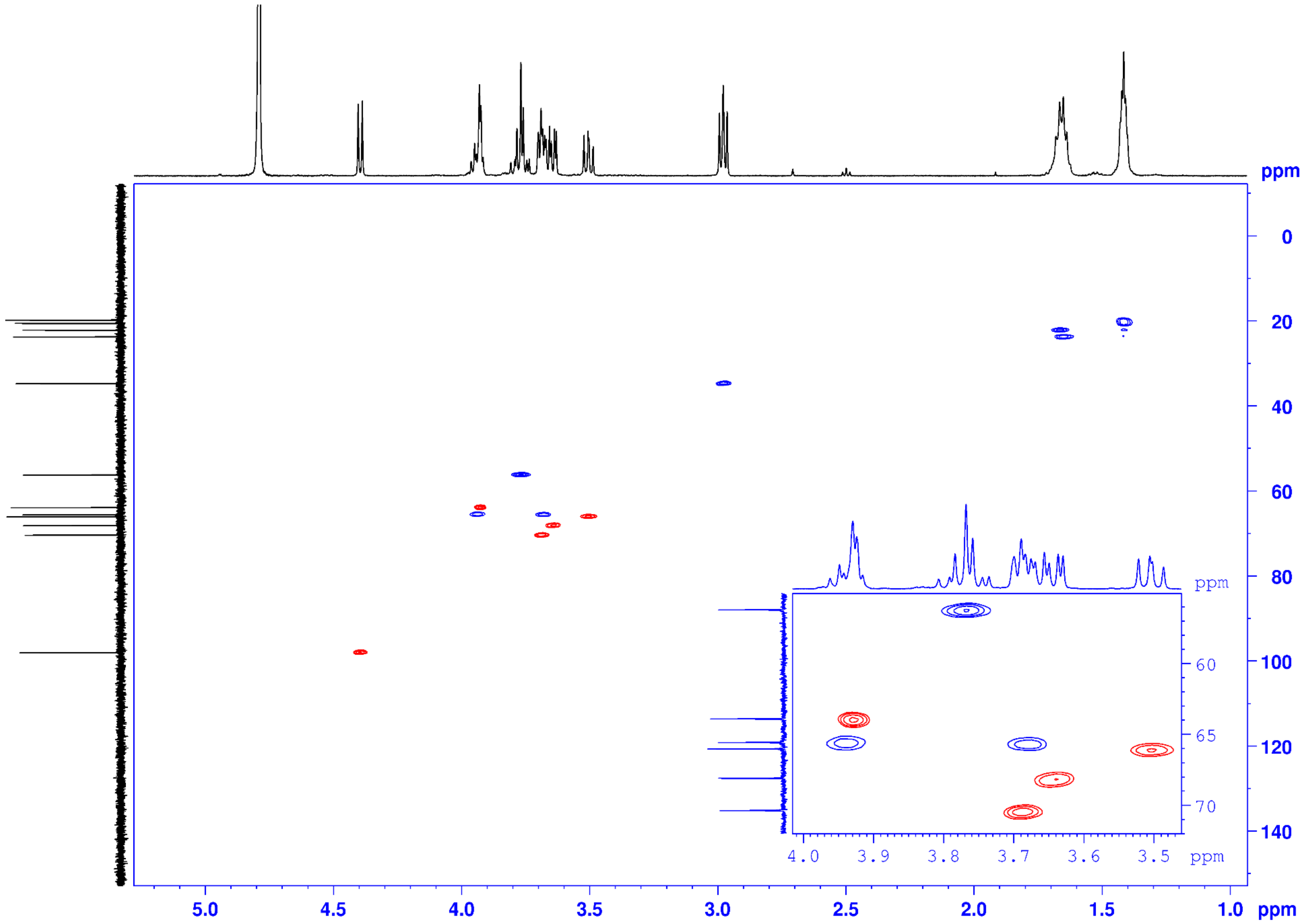

ppm
0
20
40
60
80
100
120
140
5.0
4.5
4.0
3.5
3.0
2.5
2.0
1.5
1.0
ppm
60
65
70
4.0
3.9
3.8
3.7
3.6
3.5
ppm

$^{13}C\{^1H\}$ NMR

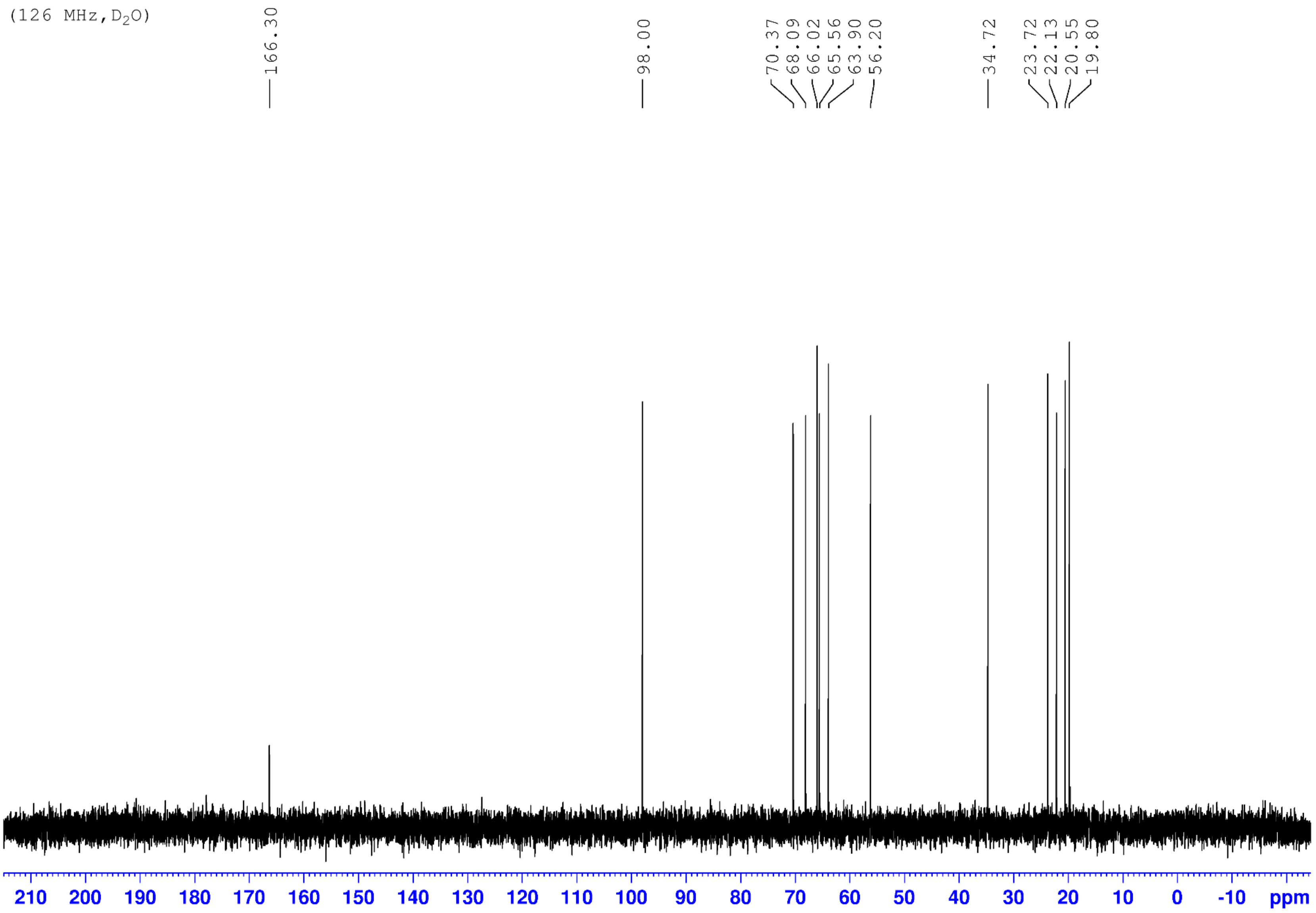
(126 MHz,D2O)
166.30
98.00
70.37
68.09
66.02
65.56
63.90
56.20
34.72
23.72
22.13
20.55
19.80
210 200 190 180 170 160 150 140 130 120 110 100 90 80 70 60 50 40 30 20 10 0 -10 ppm

Compound **nSd**

^{1}H-NMR

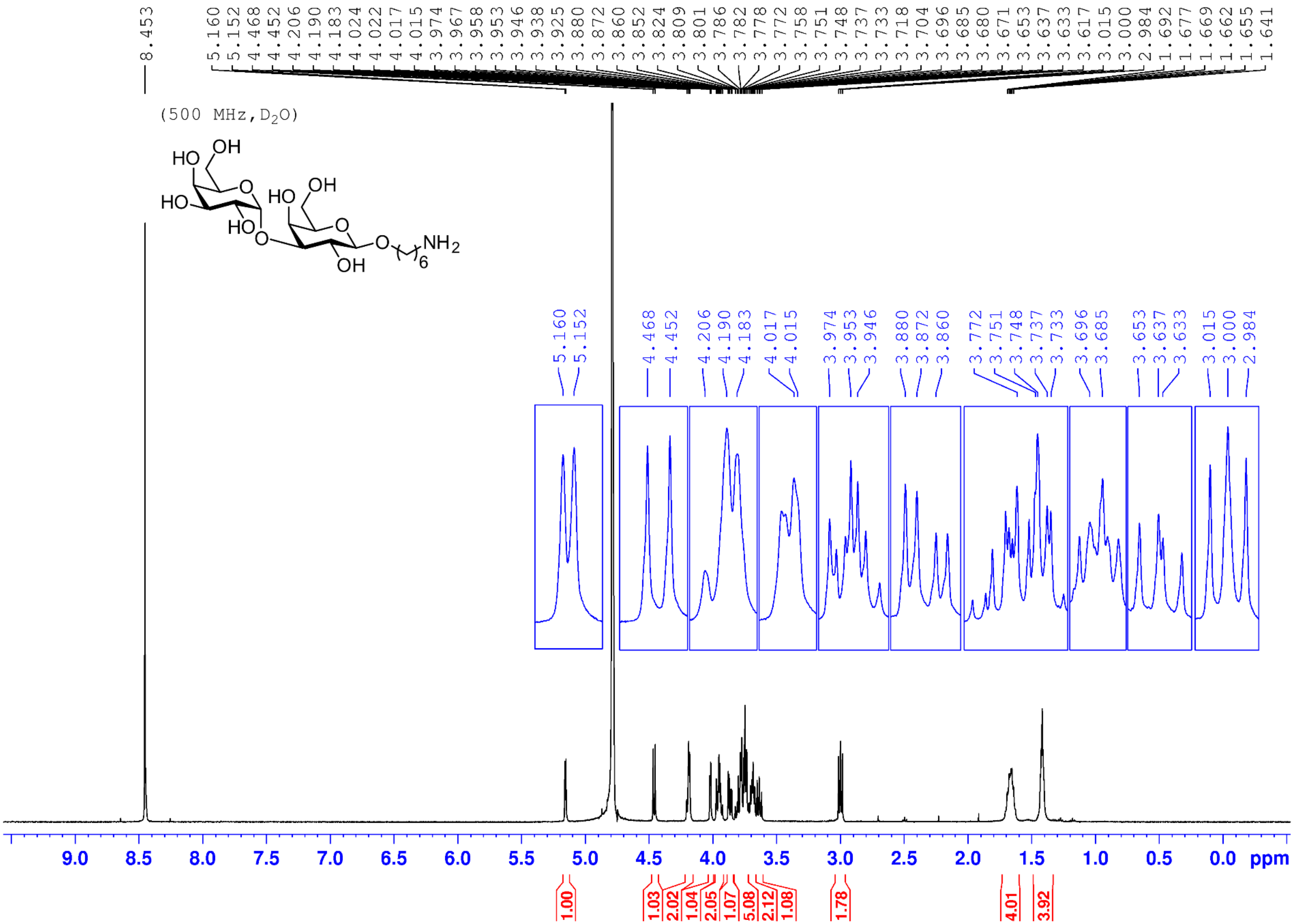

^{1}H-^{1}H COSY

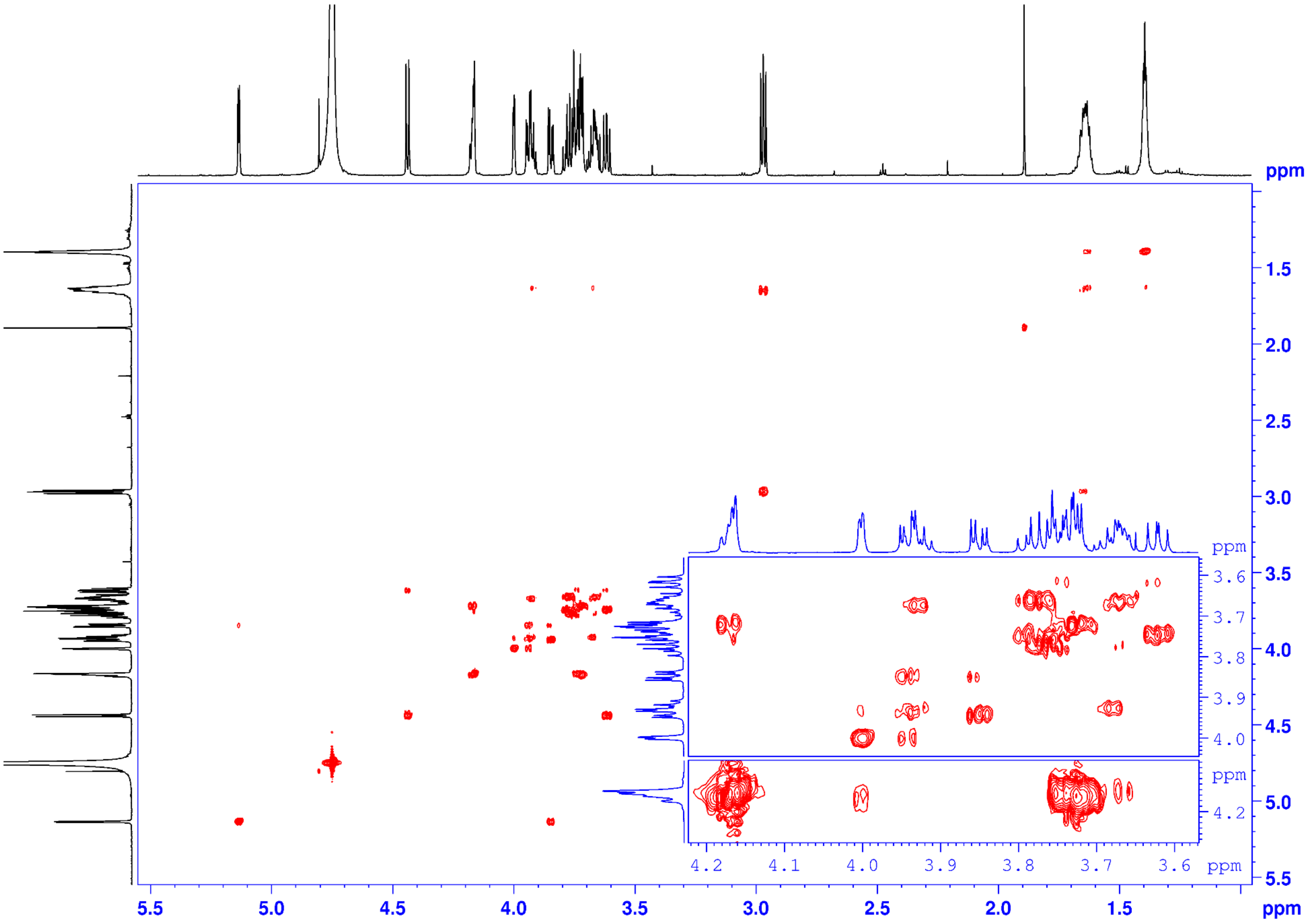

ppm
1.5
2.0
2.5
3.0
3.5
4.0
4.5
5.0
5.5
5.5
5.0
4.5
4.0
3.5
3.0
2.5
2.0
1.5
ppm
3.6
3.7
3.8
3.9
4.0
4.2
4.2
4.1
4.0
3.9
3.8
3.7
3.6
ppm

[1]H-^{1}H TOCSY

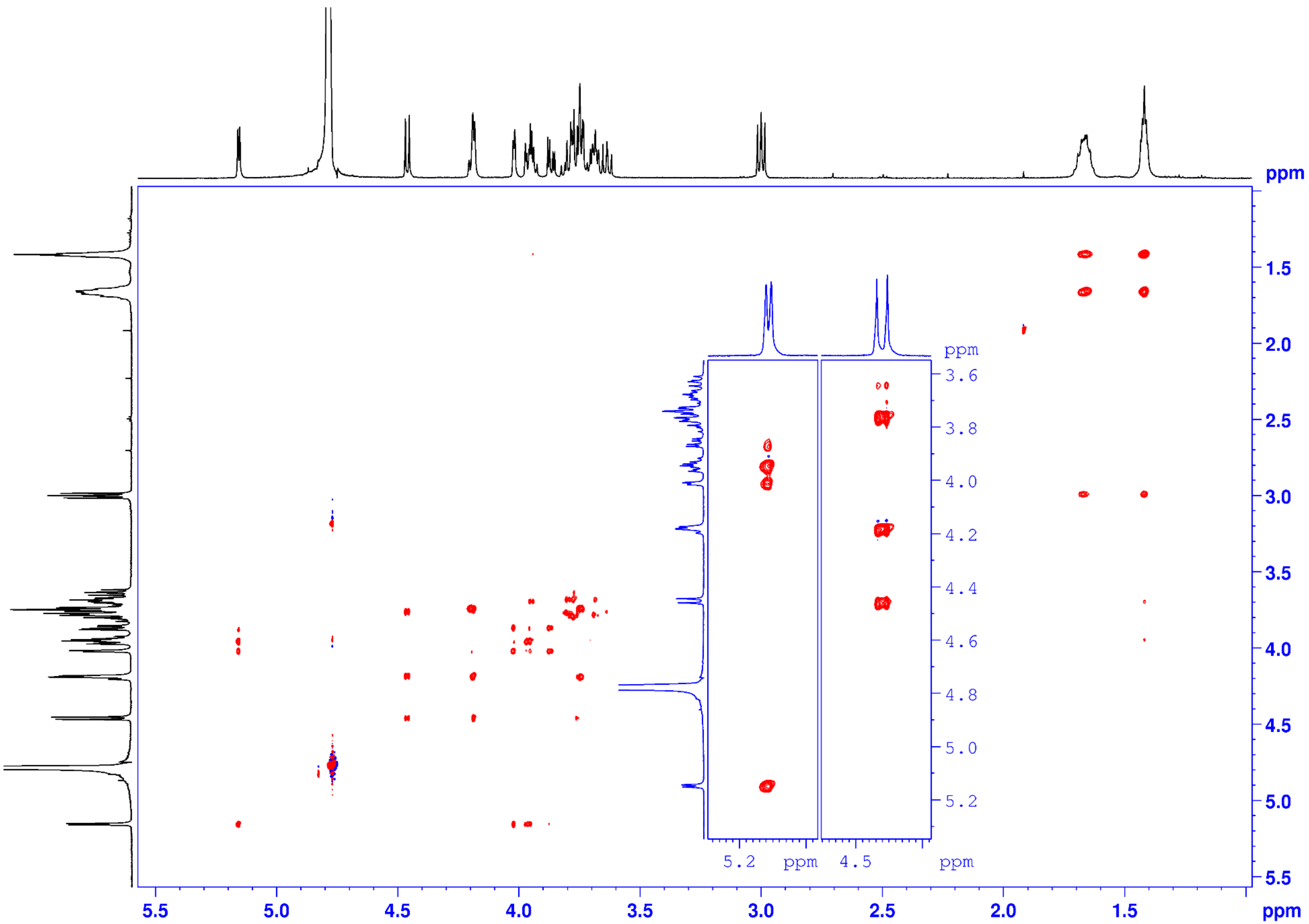

ppm
1.5
2.0
2.5
3.0
3.5
4.0
4.5
5.0
5.5
5.5
5.0
4.5
4.0
3.5
3.0
2.5
2.0
1.5
ppm
3.6
3.8
4.0
4.2
4.4
4.6
4.8
5.0
5.2
5.2
ppm
4.5
ppm

^{1}H-^{13}C HSQC

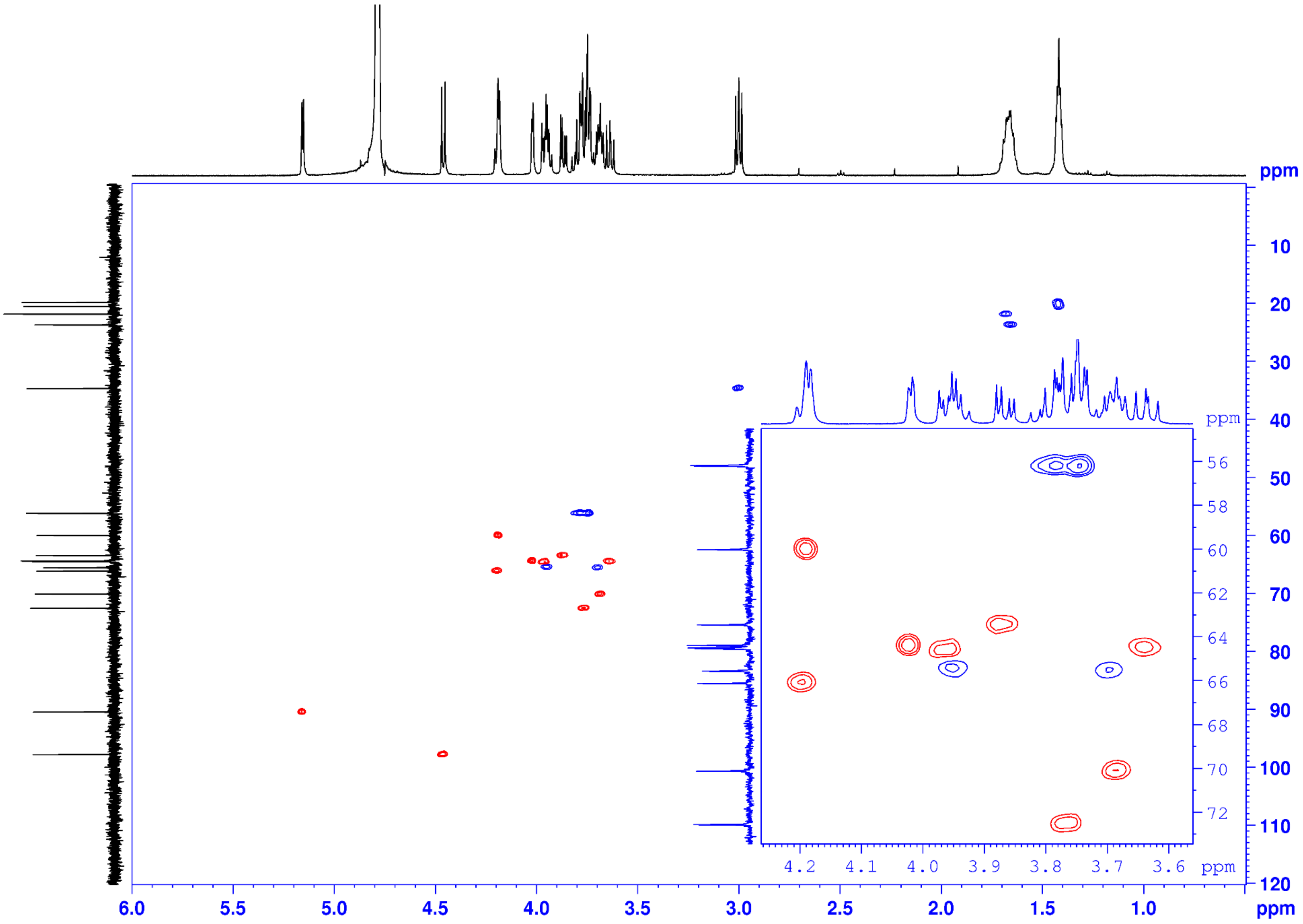

ppm
10
20
30
40
50
60
70
80
90
100
110
120
ppm
6.0
5.5
5.0
4.5
4.0
3.5
3.0
2.5
2.0
1.5
1.0
ppm
56
58
60
62
64
66
68
70
72
ppm
4.2
4.1
4.0
3.9
3.8
3.7
3.6
ppm

^{1}H-^{13}C non-decoupled HSQC

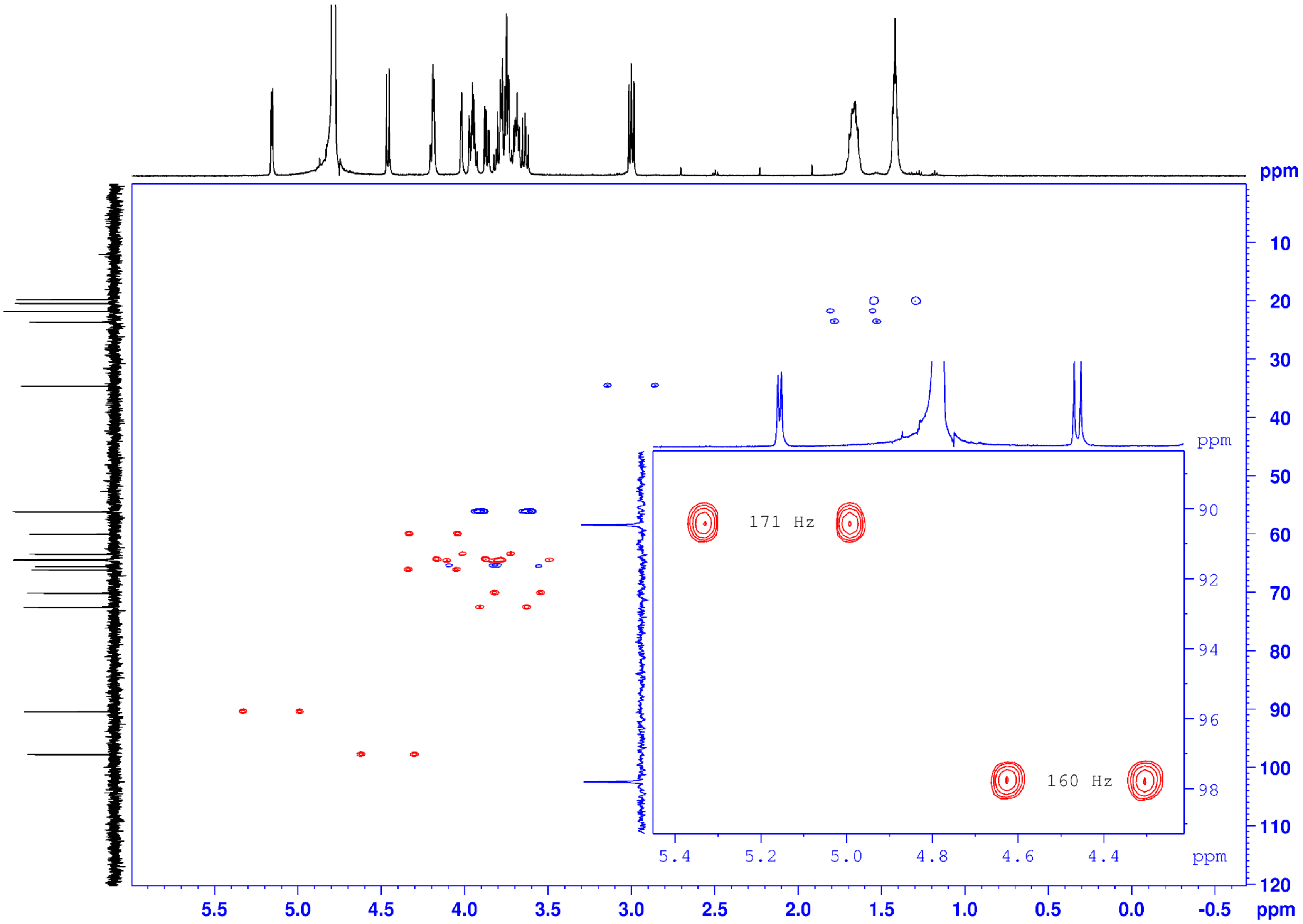

$^{13}C\{^{1}H\}$ NMR

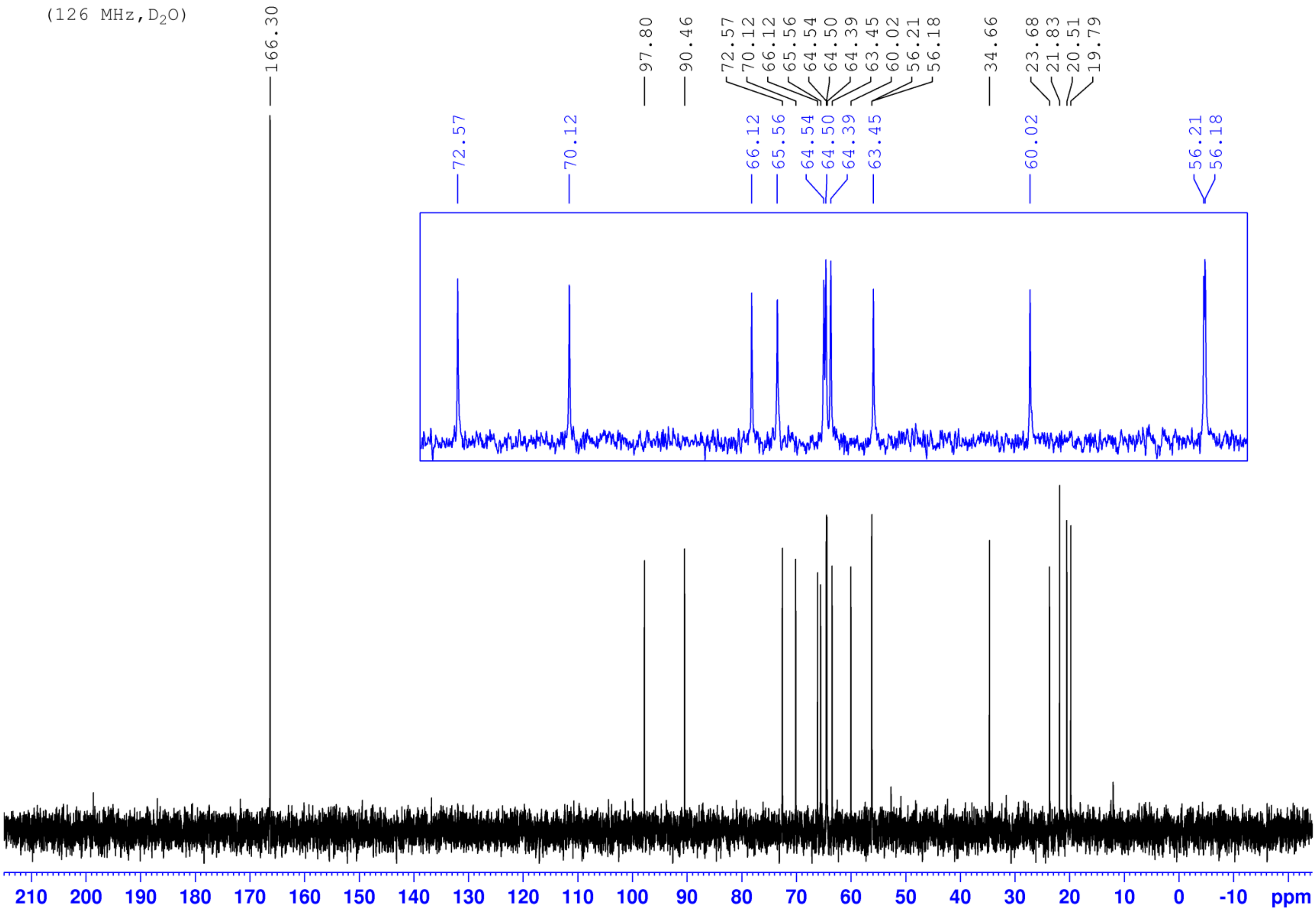

(126 MHz, D2O)
166.30
97.80
90.46
72.57
70.12
66.12
65.56
64.54
64.50
64.39
63.45
60.02
56.21
56.18
34.66
23.68
21.83
20.51
19.79
210 200 190 180 170 160 150 140 130 120 110 100 90 80 70 60 50 40 30 20 10 0 -10 ppm

^{13}C DEPT-135

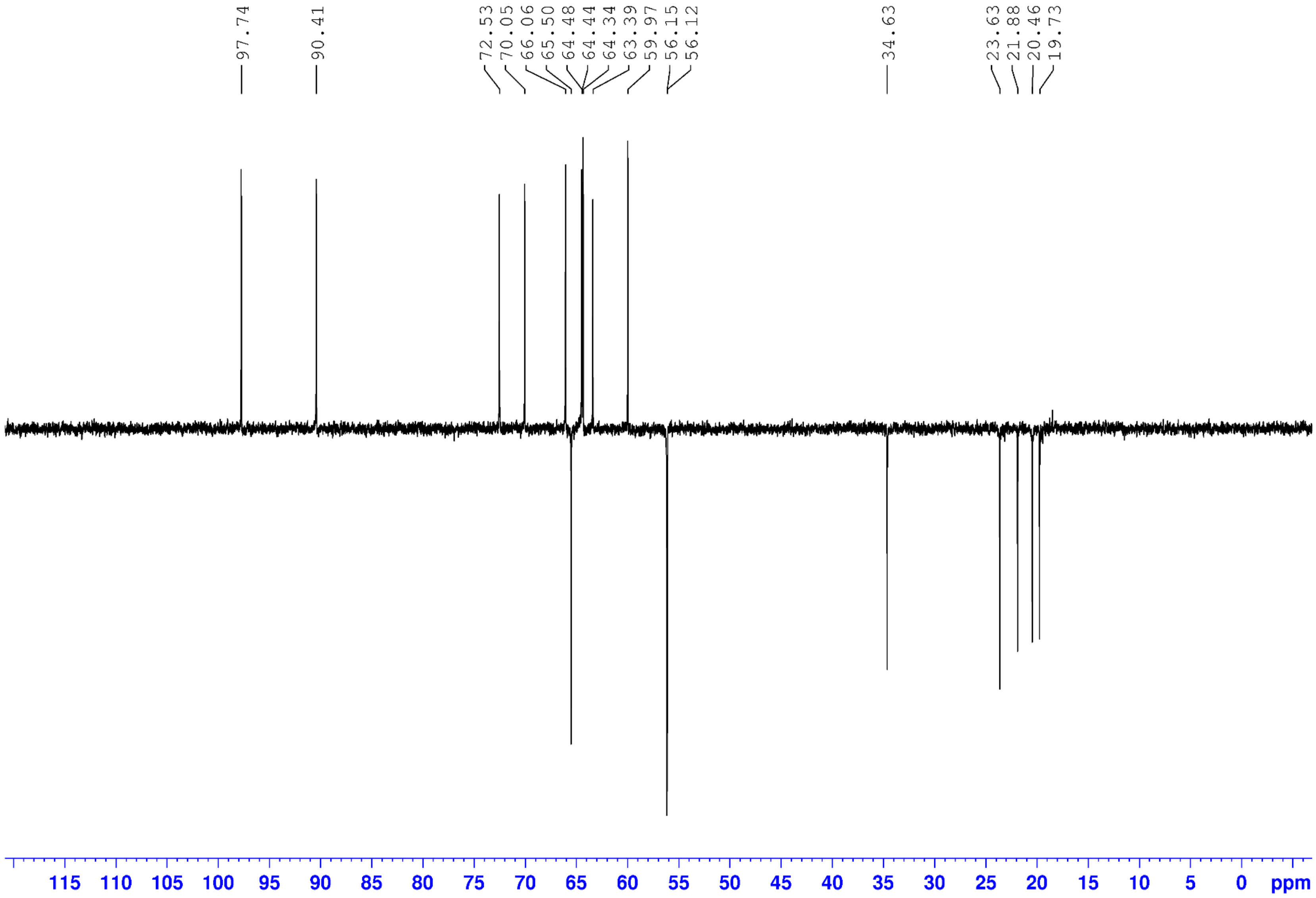
97.74
90.41
72.53
70.05
66.06
65.50
64.48
64.44
64.34
63.39
59.97
56.15
56.12
34.63
23.63
21.88
20.46
19.73
115 110 105 100 95 90 85 80 75 70 65 60 55 50 45 40 35 30 25 20 15 10 5 0 ppm

Compound **mSd**

^{1}H-NMR

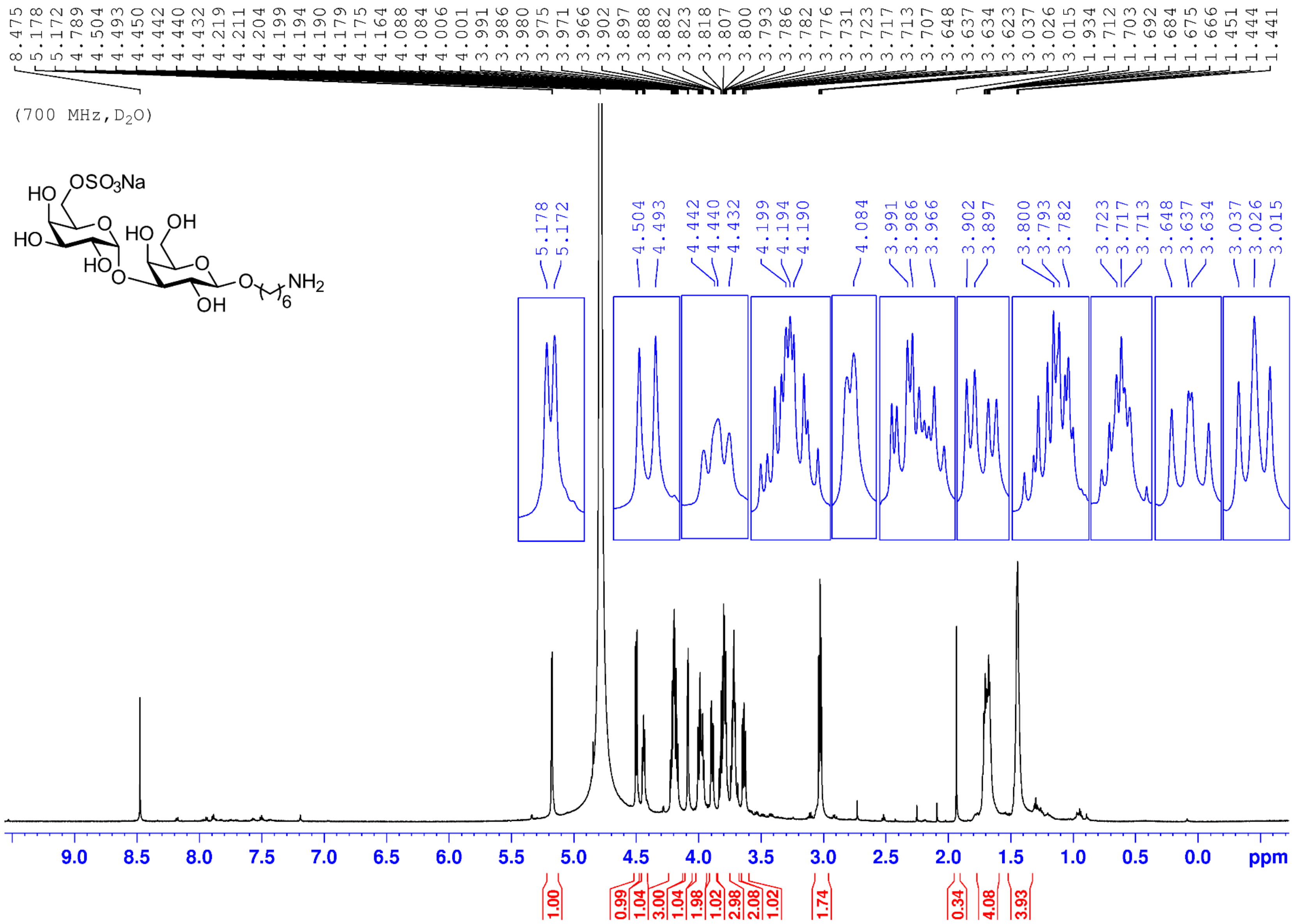

^{1}H-^{1}H COSY

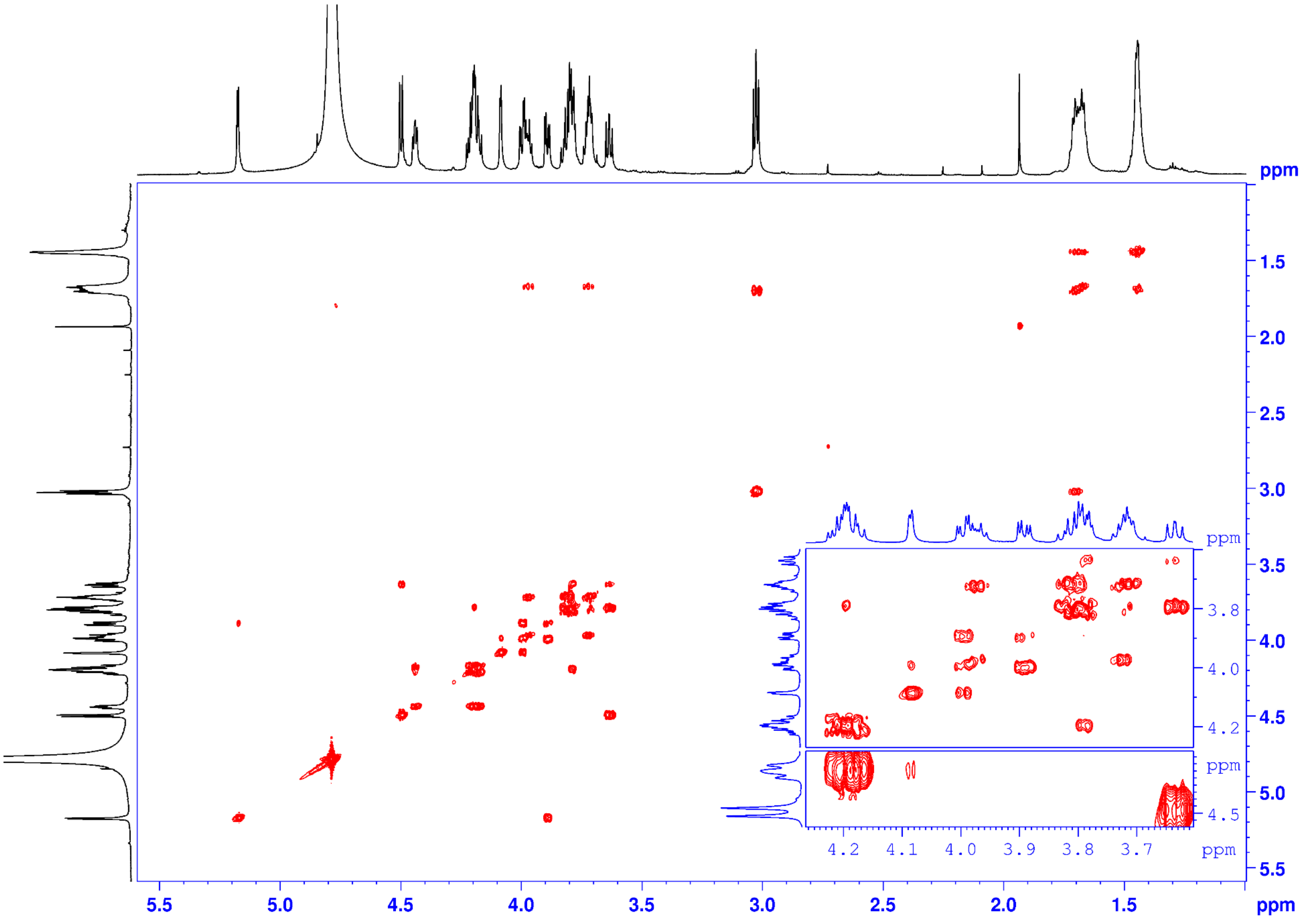
ppm
1.5
2.0
2.5
3.0
3.5
4.0
4.5
5.0
5.5
5.5
5.0
4.5
4.0
3.5
3.0
2.5
2.0
1.5
ppm
3.8
4.0
4.2
4.5
4.2
4.1
4.0
3.9
3.8
3.7

[1]H-^{1}H TOCSY

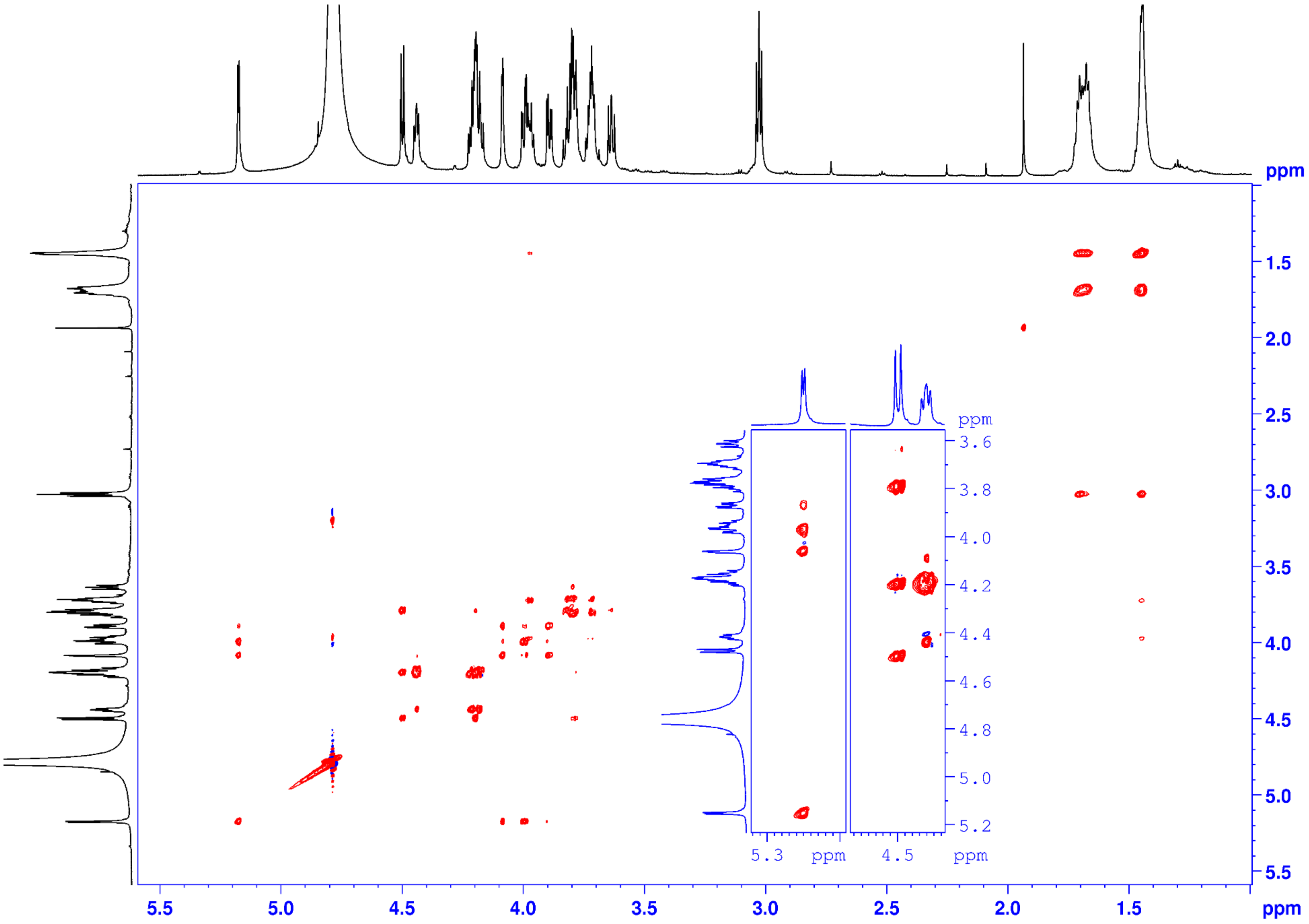

ppm
1.5
2.0
2.5
3.0
3.5
4.0
4.5
5.0
5.5
5.5
5.0
4.5
4.0
3.5
3.0
2.5
2.0
1.5
ppm
3.6
3.8
4.0
4.2
4.4
4.6
4.8
5.0
5.2
5.3
ppm
4.5
ppm

^{1}H-^{13}C HMQC

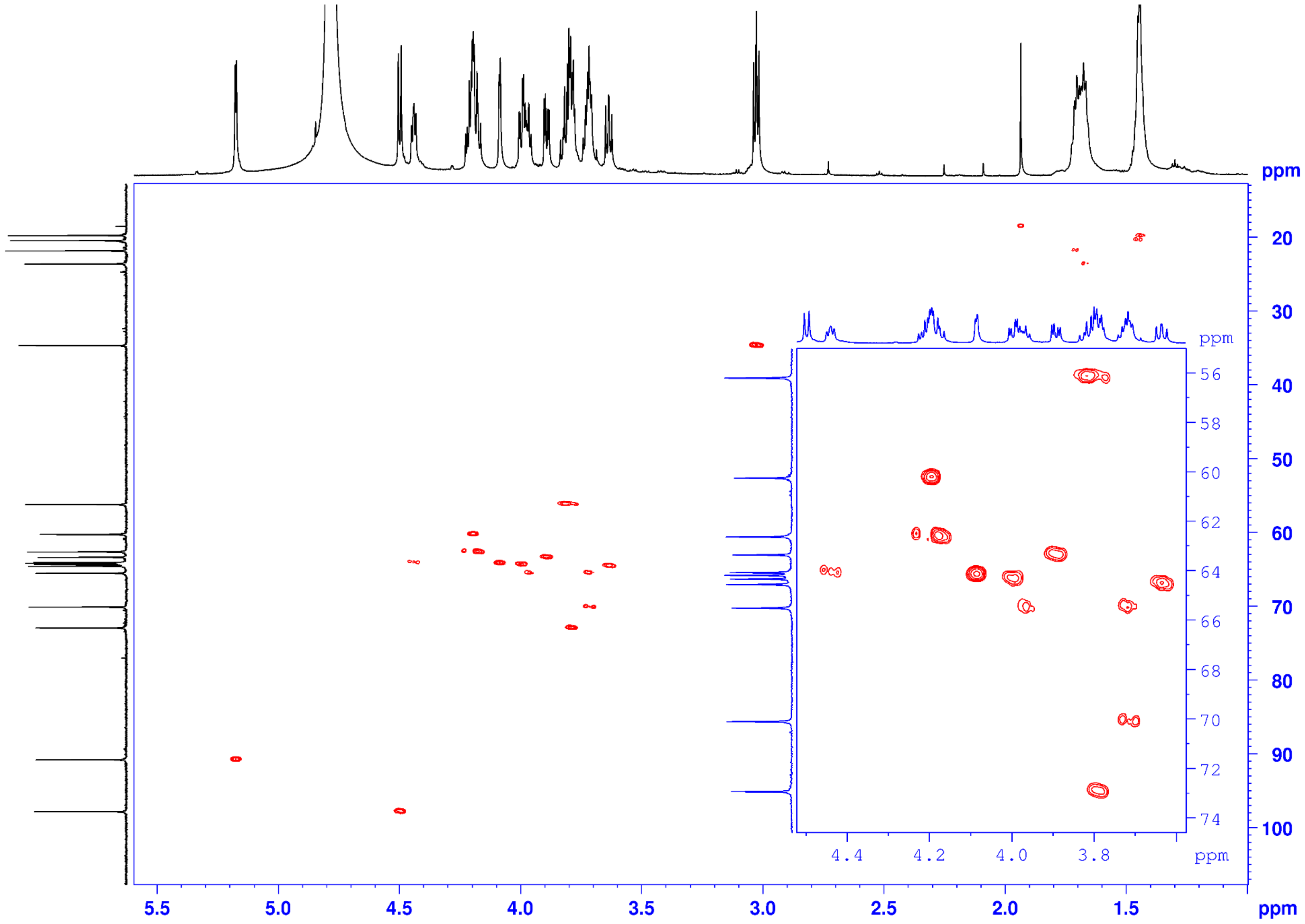
ppm
20
30
40
50
60
70
80
90
100
5.5
5.0
4.5
4.0
3.5
3.0
2.5
2.0
1.5
ppm
56
58
60
62
64
66
68
70
72
74
4.4
4.2
4.0
3.8
ppm

^{1}H-^{13}C non-decoupled HMQC

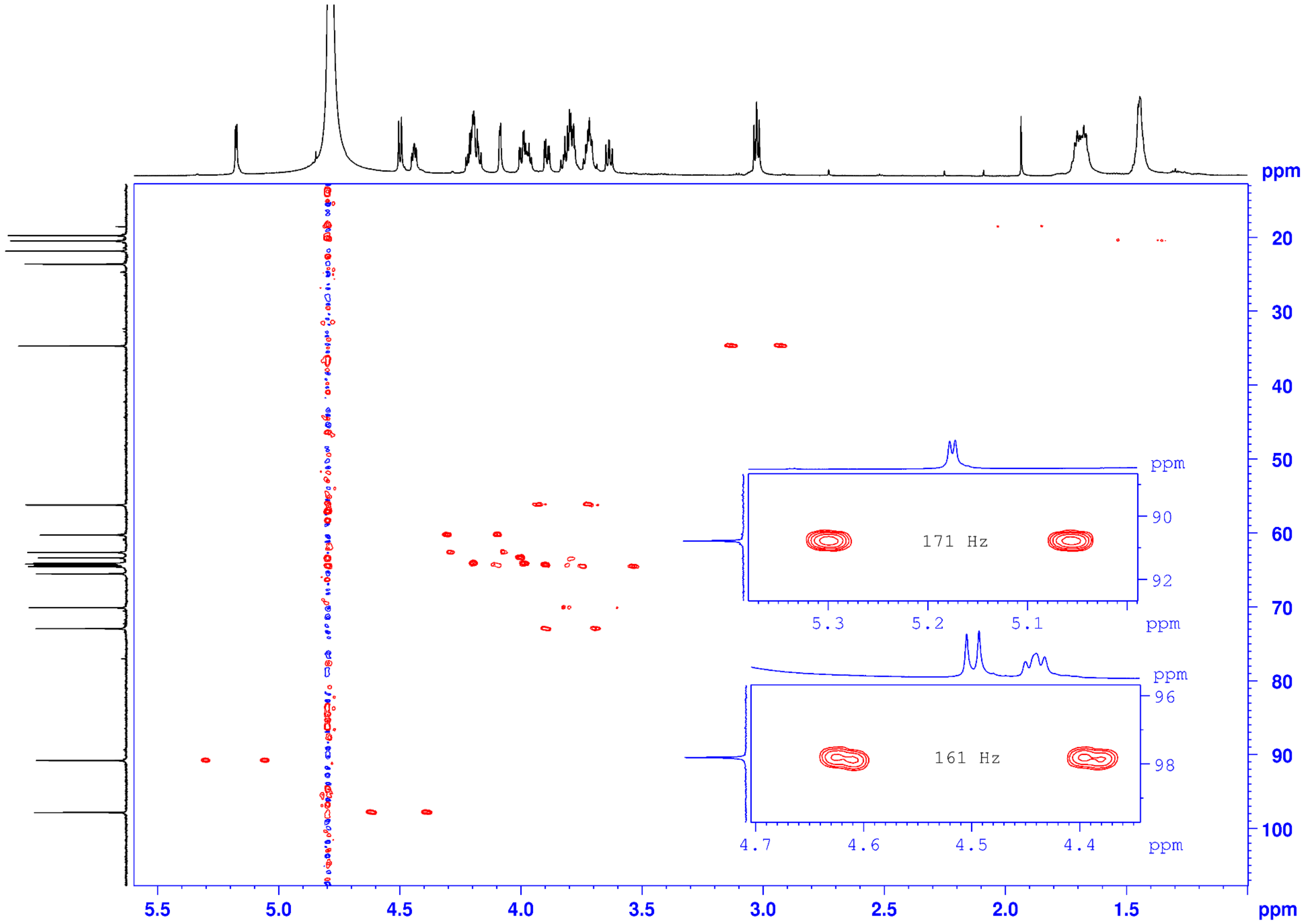

^{1}H-^{13}C unfiltered $^{1}J_{CH}$ non-decoupled HMBC

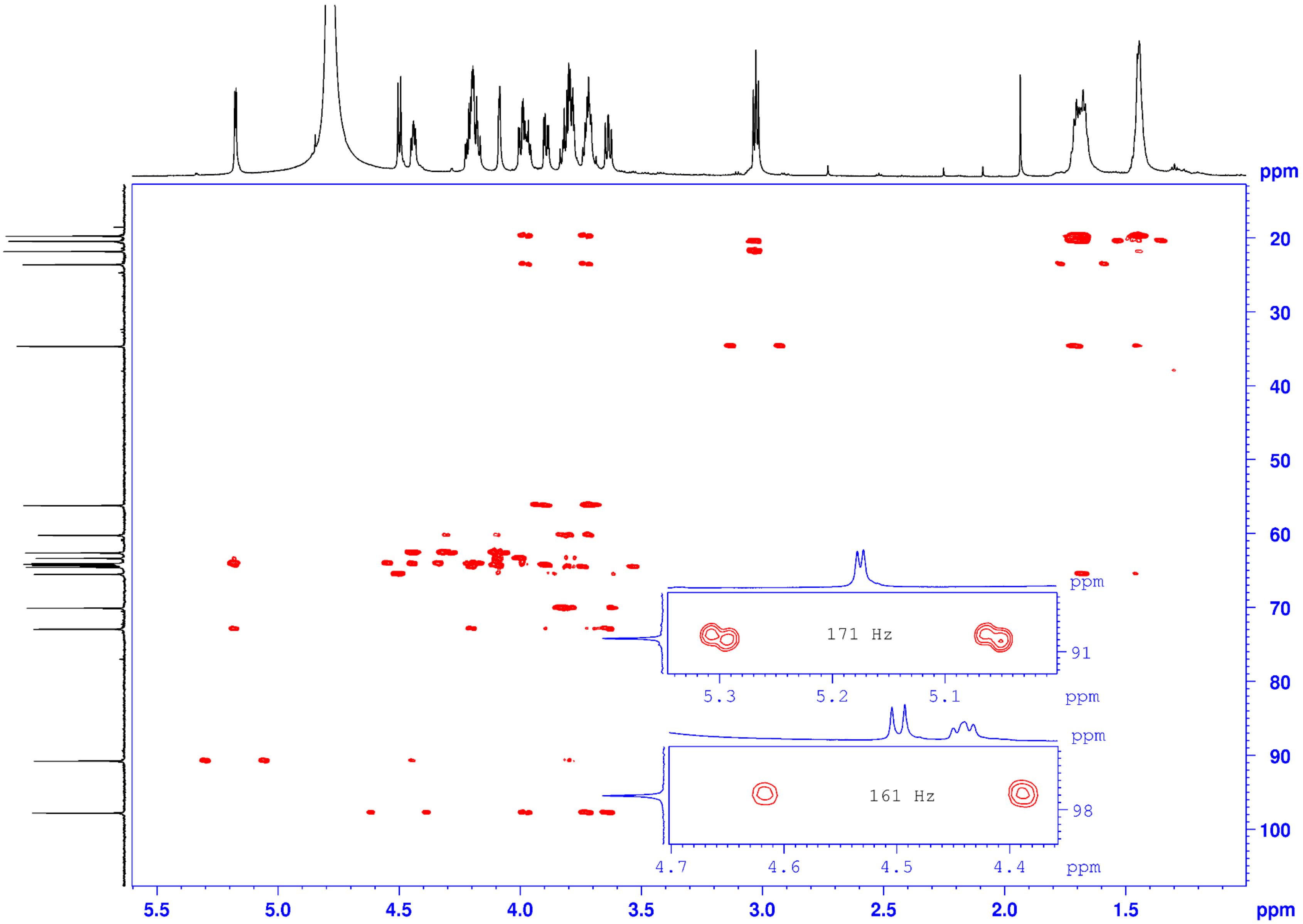

$^{13}C\{^{1}H\}$ NMR

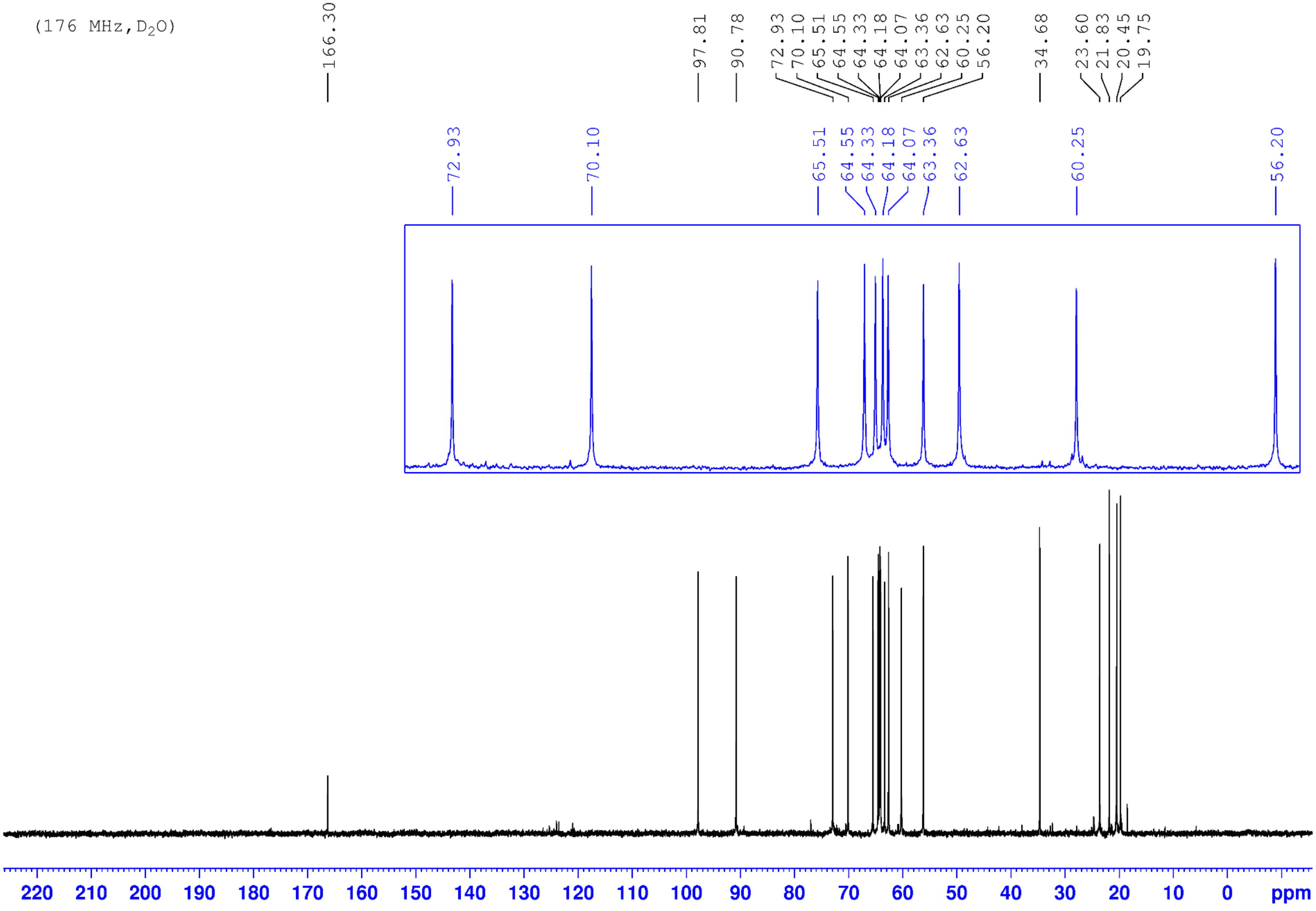

(176 MHz, D2O)
166.30
97.81
90.78
72.93
70.10
65.51
64.55
64.33
64.18
64.07
63.36
62.63
60.25
56.20
34.68
23.60
21.83
20.45
19.75
220 210 200 190 180 170 160 150 140 130 120 110 100 90 80 70 60 50 40 30 20 10 0 ppm

^{13}C DEPT-135

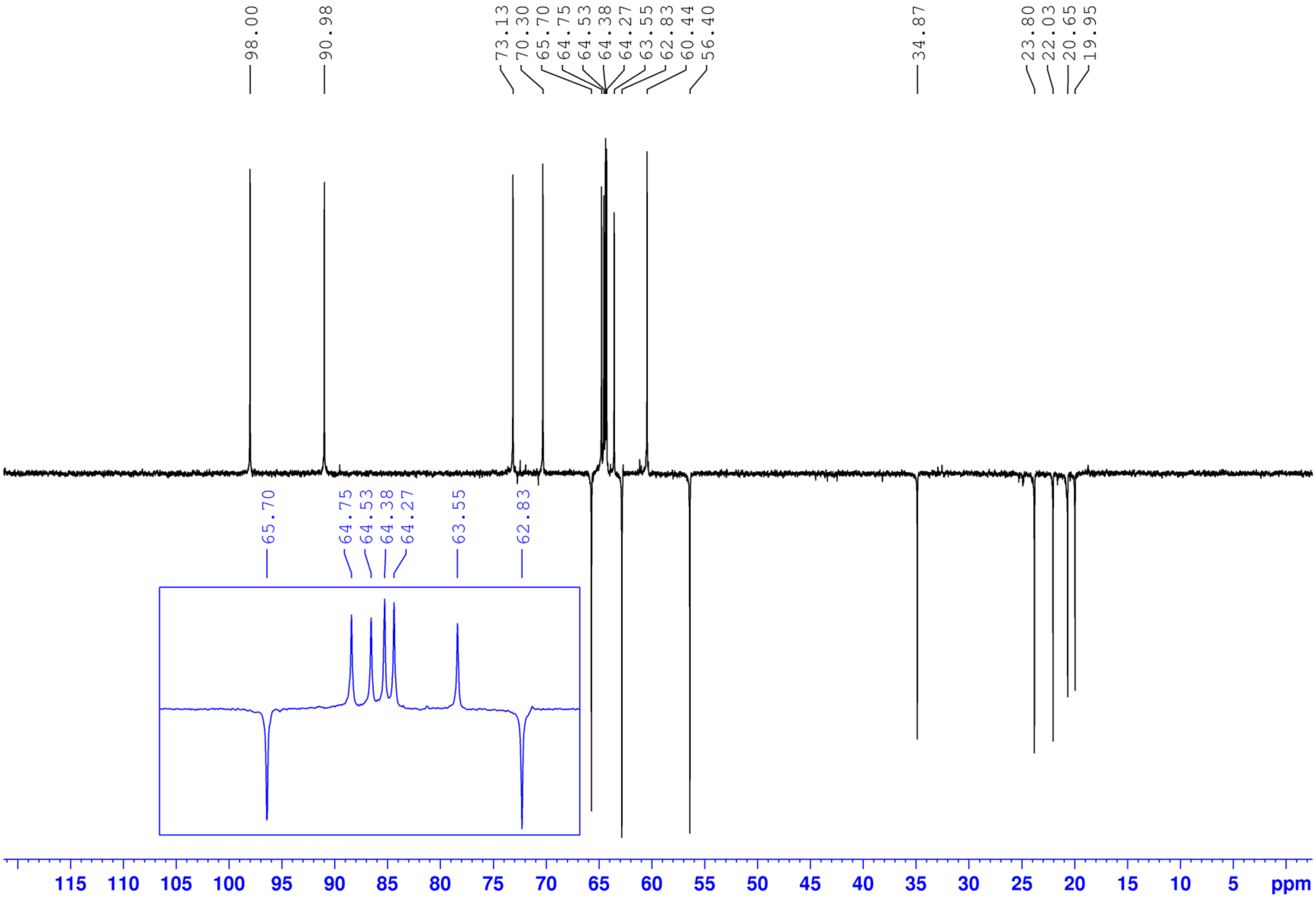

Compound **nSt**

^{1}H-NMR

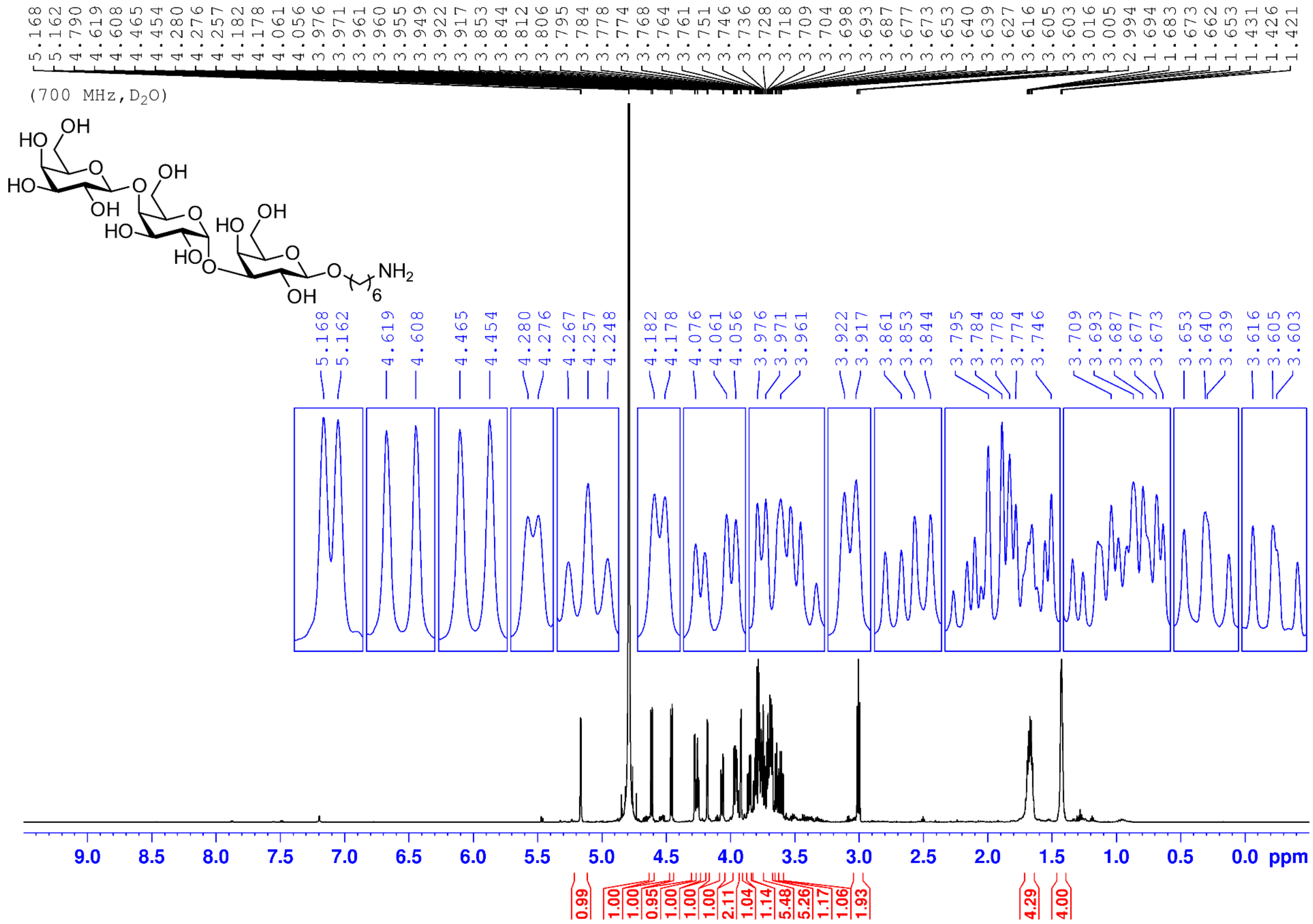

^{1}H-^{1}H COSY

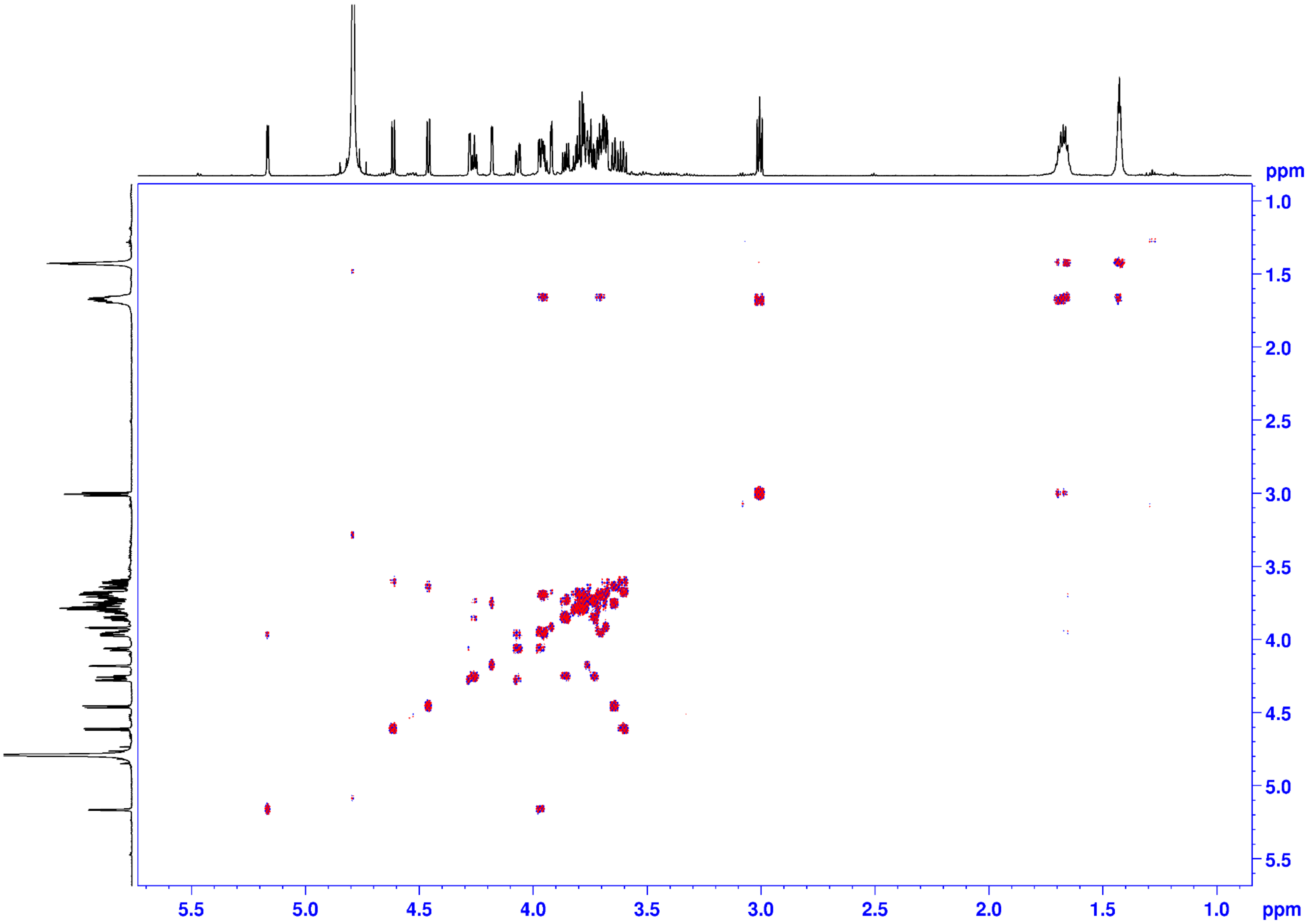

^{1}H-^{1}H COSY (enlarged section)

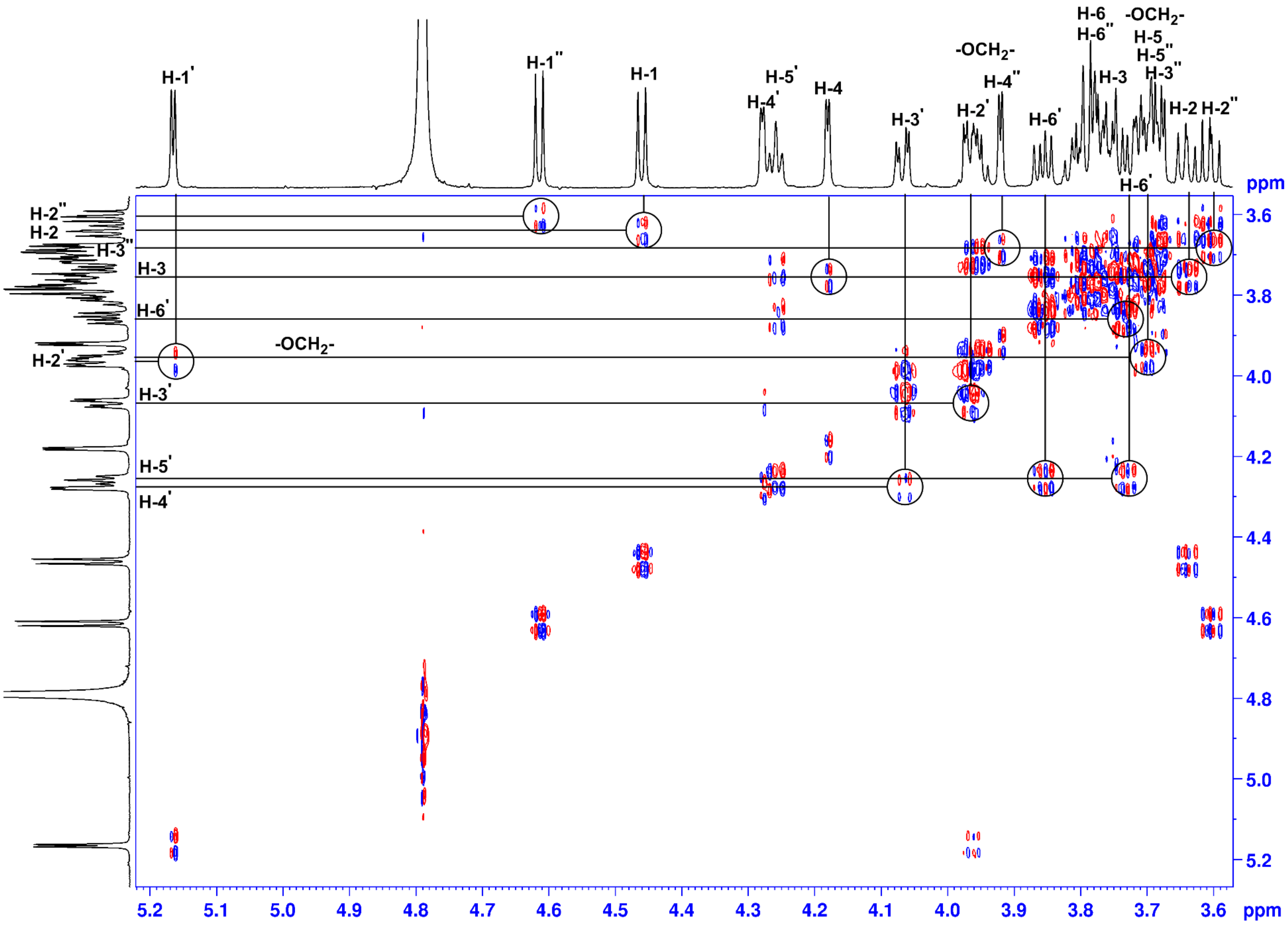

^{1}H-^{13}C HSQC

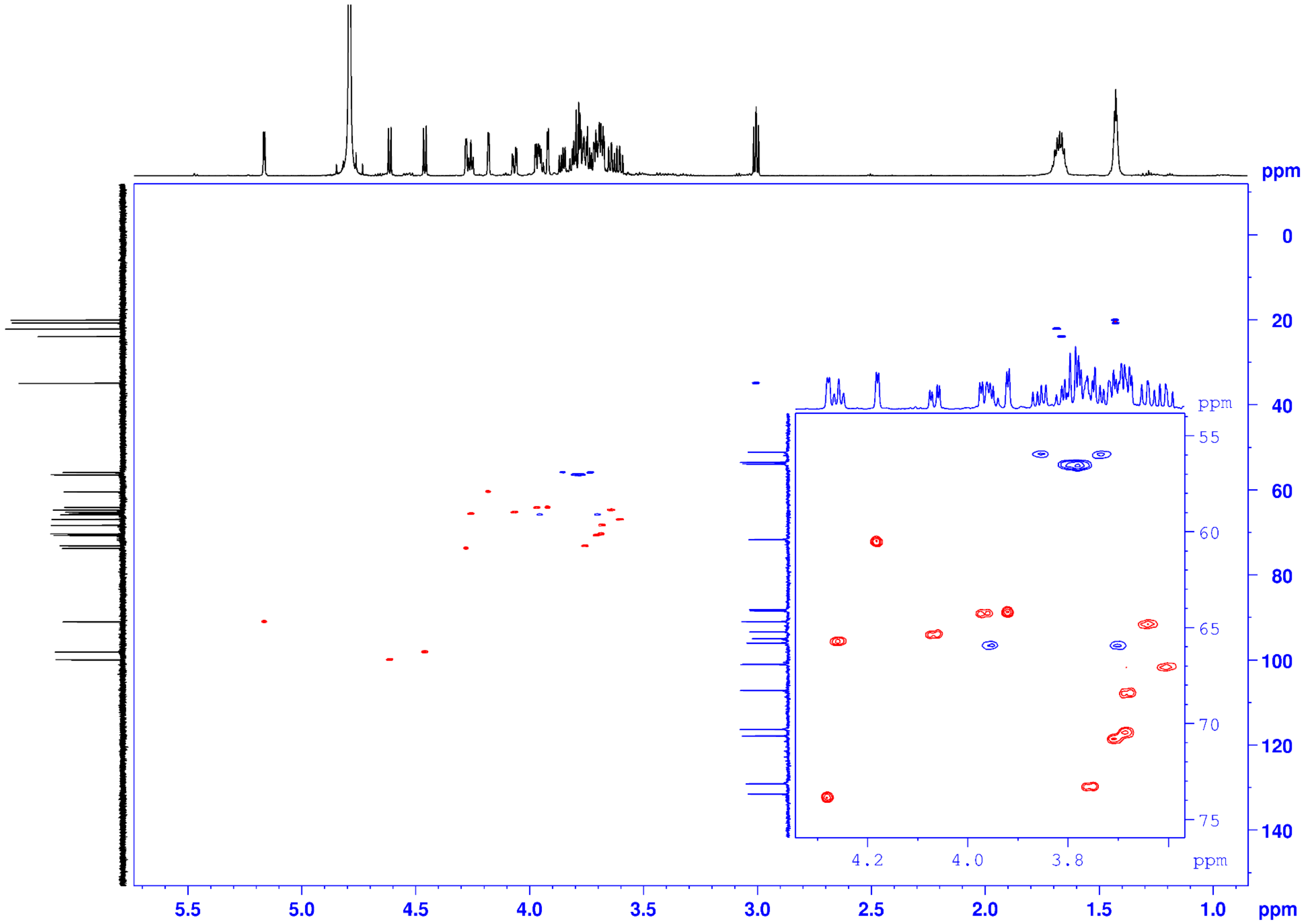

ppm
0
20
40
60
80
100
120
140
5.5
5.0
4.5
4.0
3.5
3.0
2.5
2.0
1.5
1.0
ppm
55
60
65
70
75
4.2
4.0
3.8

^{1}H-^{13}C non-decoupled HSQC

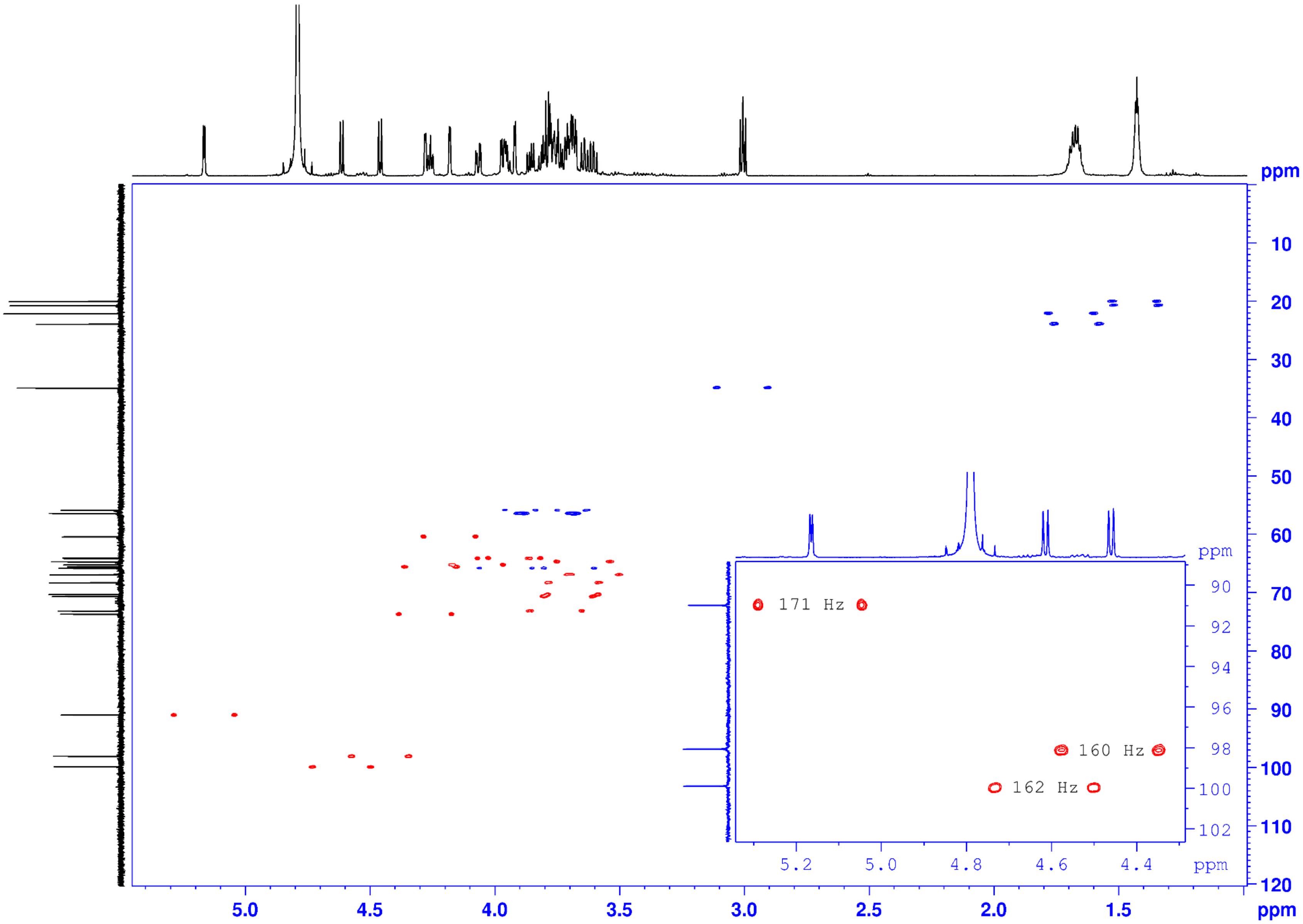

^{1}H-^{13}C HMBC

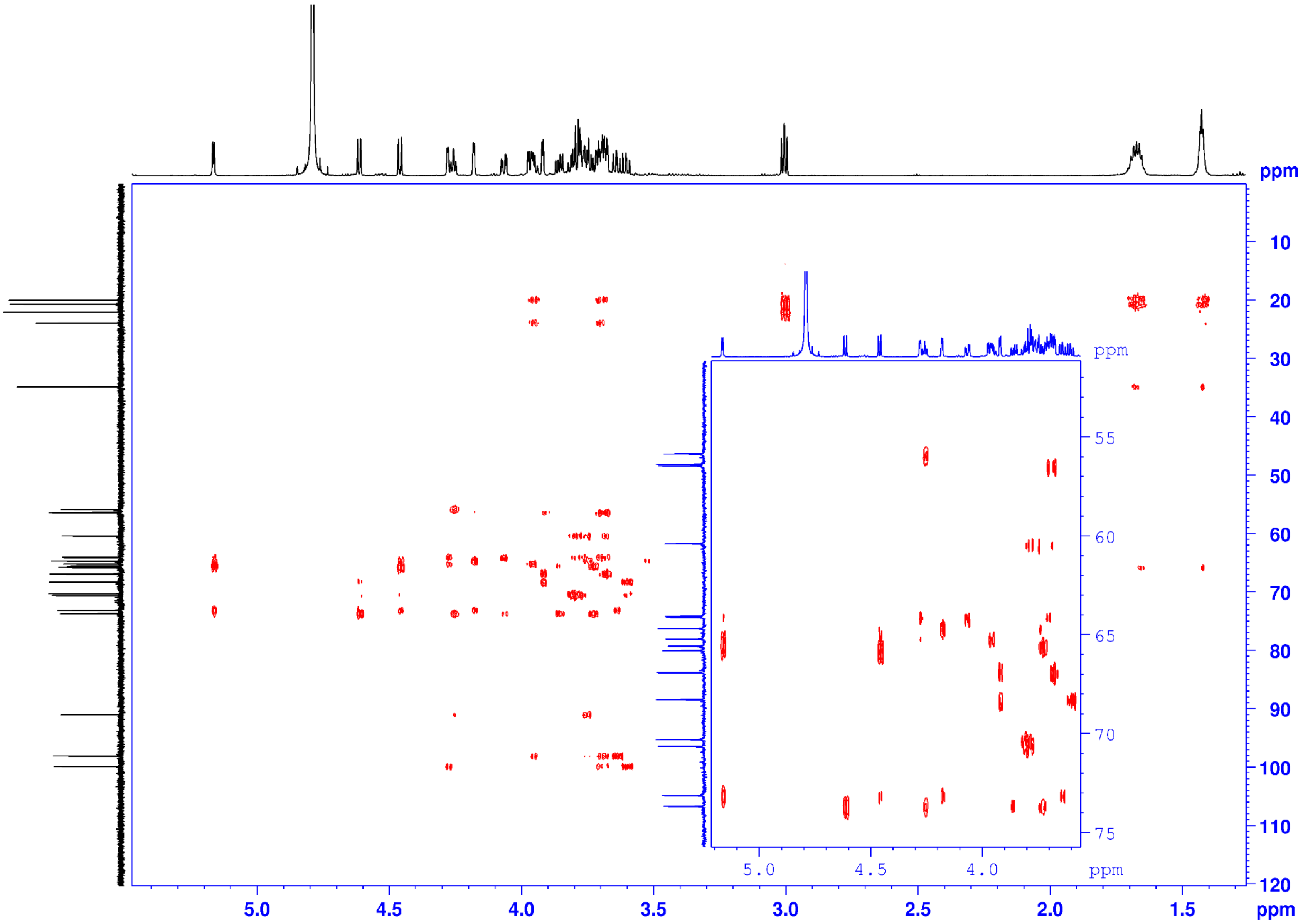

ppm
10
20
30
40
50
60
70
80
90
100
110
120
ppm
5.0
4.5
4.0
3.5
3.0
2.5
2.0
1.5
ppm
ppm
55
60
65
70
75
5.0
4.5
4.0
ppm

$^{13}C\{^1H\}$ NMR

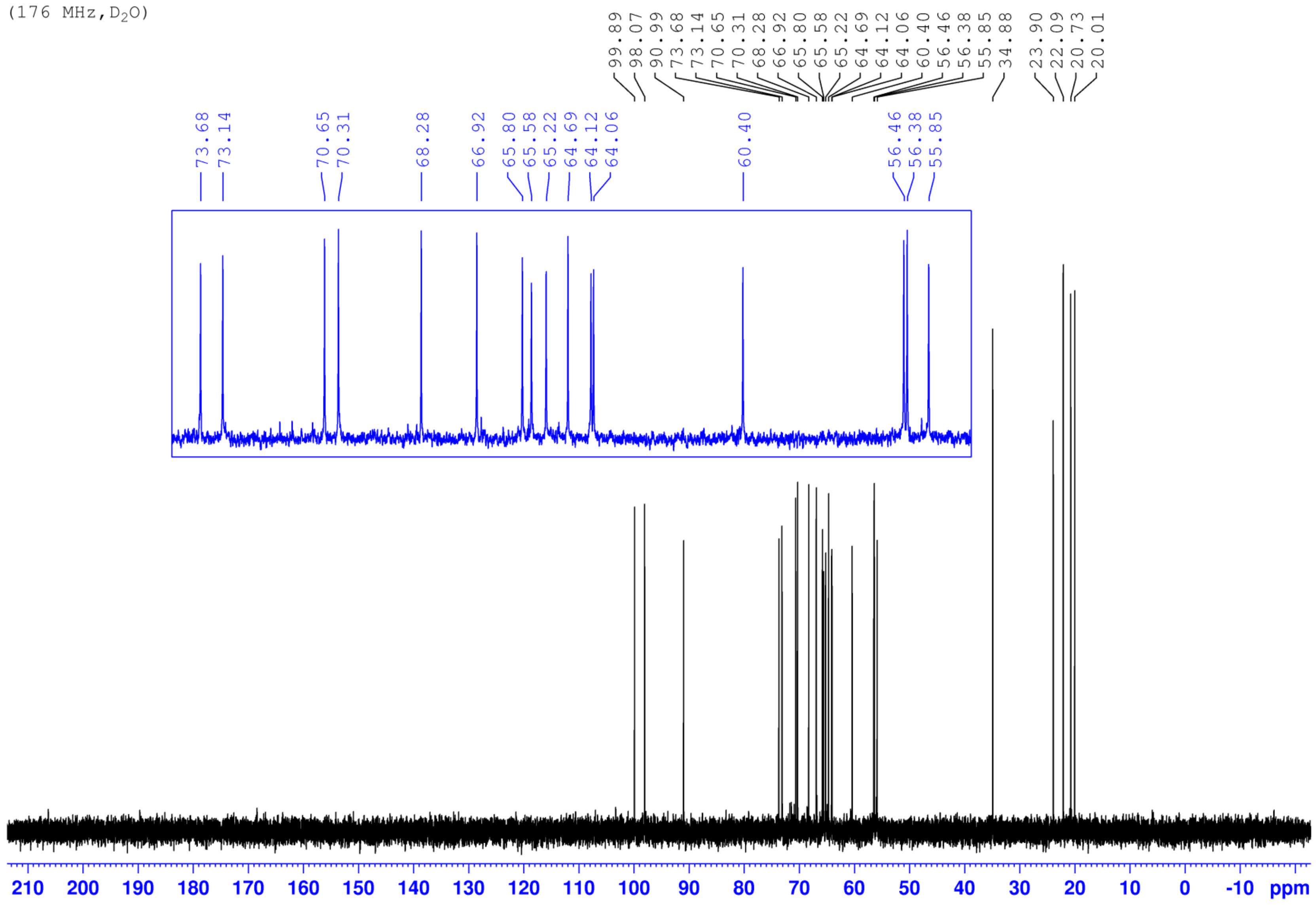
(176 MHz, D2O)
99.89
98.07
90.99
73.68
73.14
70.65
70.31
68.28
66.92
65.80
65.58
65.22
64.69
64.12
64.06
60.40
56.46
56.38
55.85
34.88
23.90
22.09
20.73
20.01
210 200 190 180 170 160 150 140 130 120 110 100 90 80 70 60 50 40 30 20 10 0 -10 ppm

^{13}C DEPT-135

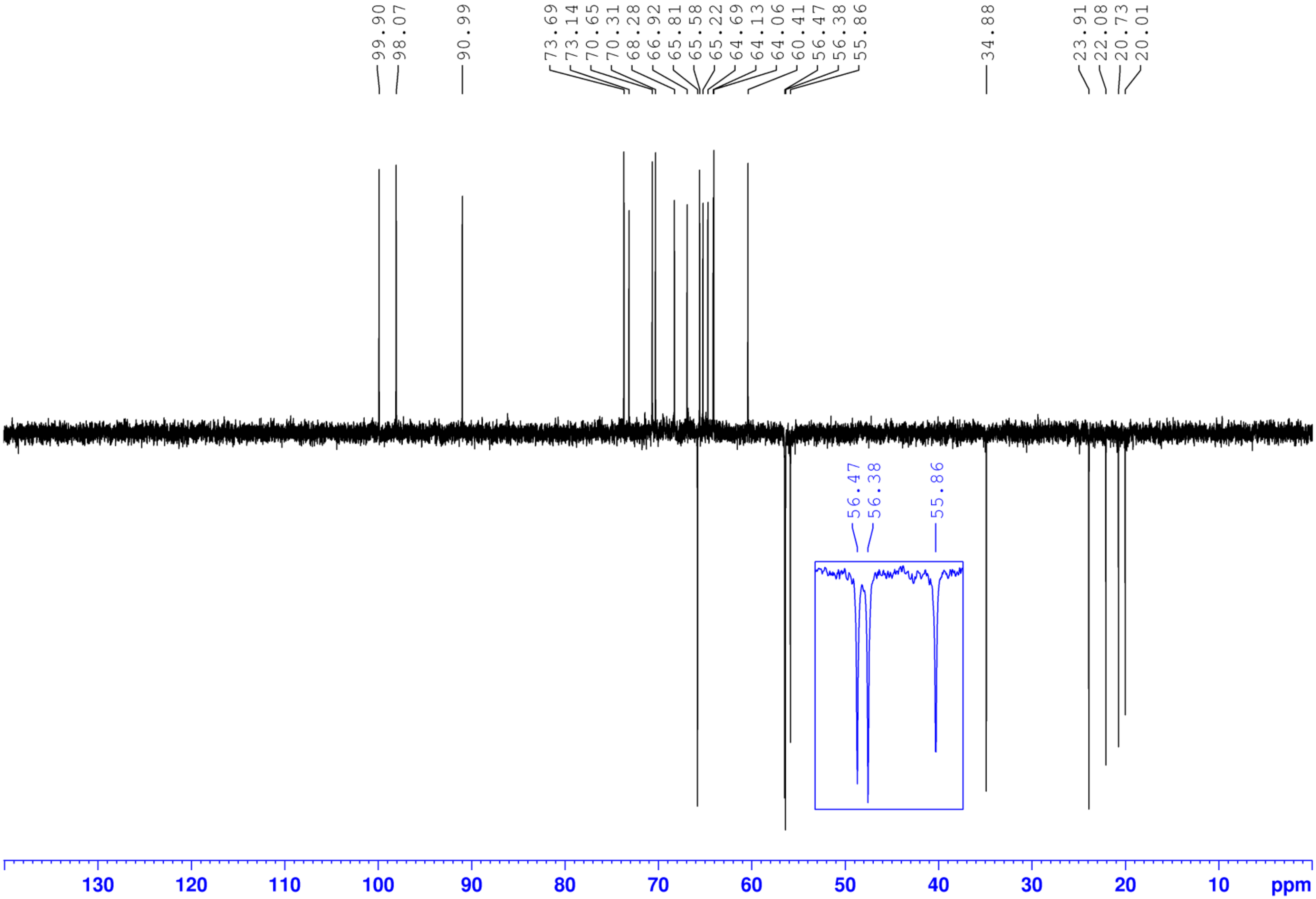

99.90
98.07
90.99
73.69
73.14
70.65
70.31
68.28
66.92
65.81
65.58
65.22
64.69
64.13
64.06
60.41
56.47
56.38
55.86
34.88
23.91
22.08
20.73
20.01
56.47
56.38
55.86
130
120
110
100
90
80
70
60
50
40
30
20
10
ppm

Compound **mSt**

^{1}H-NMR

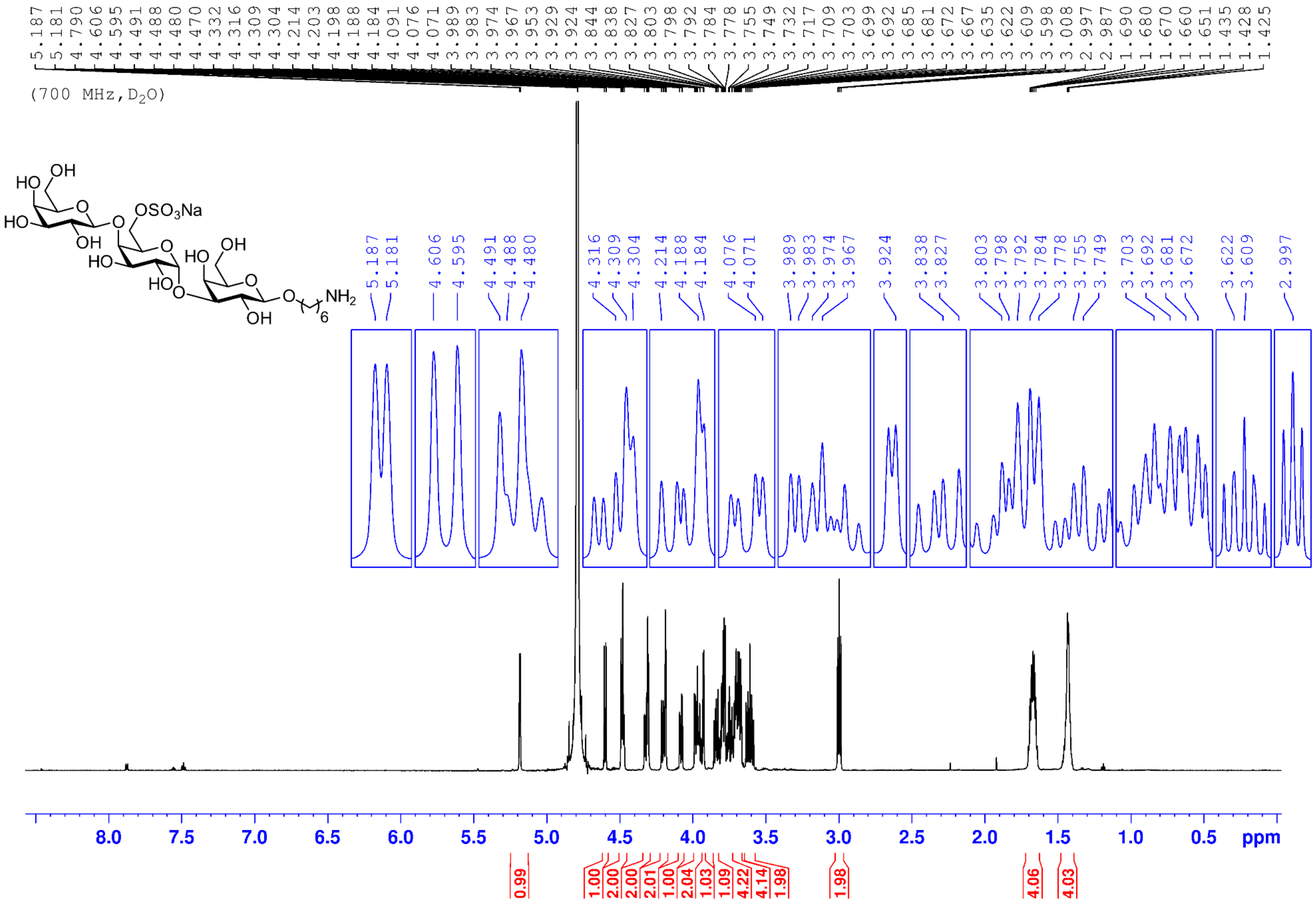

^{1}H-^{1}H COSY

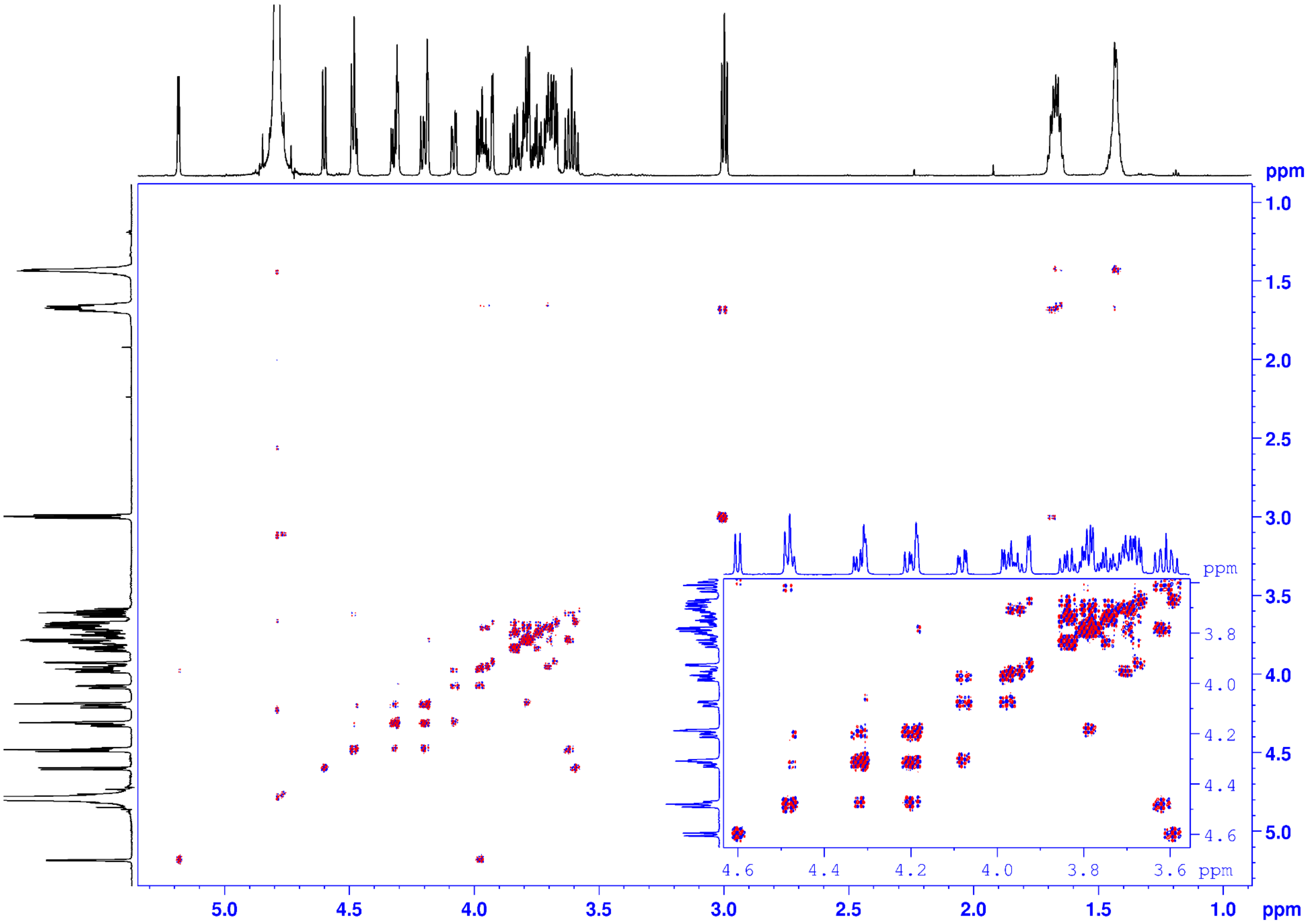
ppm
1.0
1.5
2.0
2.5
3.0
3.5
4.0
4.5
5.0
5.0
4.5
4.0
3.5
3.0
2.5
2.0
1.5
1.0
ppm
4.6
4.4
4.2
4.0
3.8
3.6
ppm

^1H-^1H COSY (enlarged section)

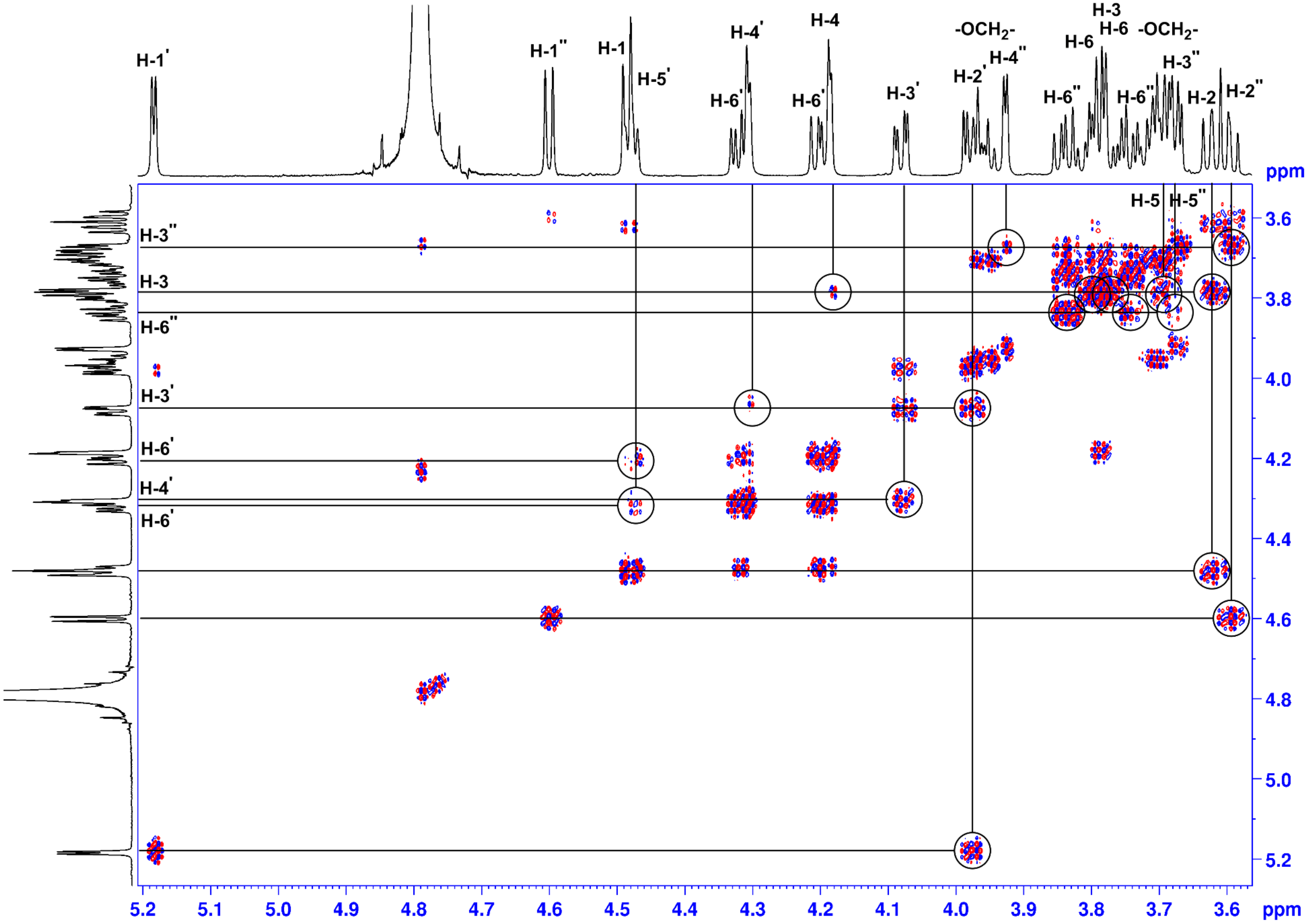

^1H-^{13}C HSQC

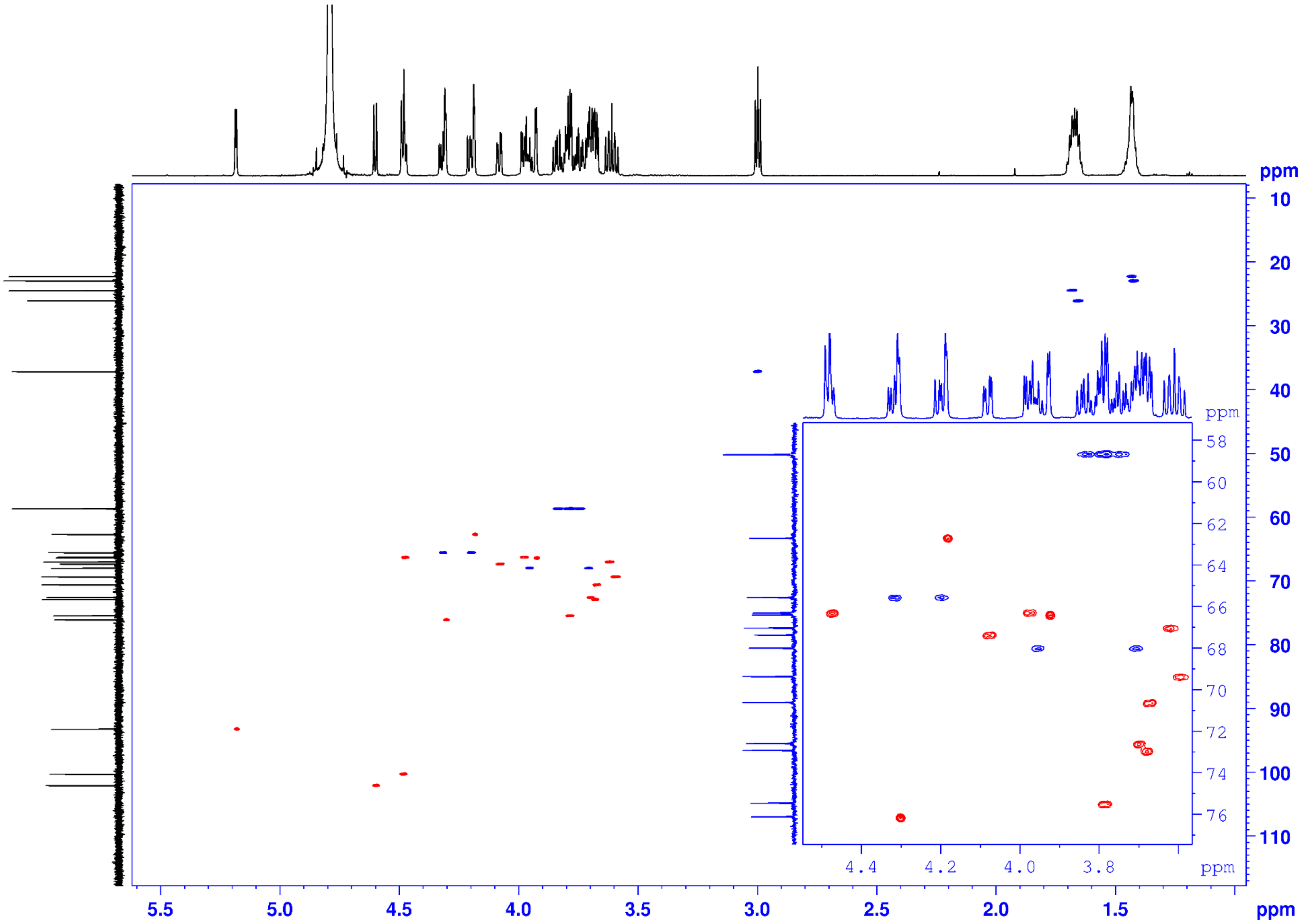

ppm
10
20
30
40
50
60
70
80
90
100
110
5.5
5.0
4.5
4.0
3.5
3.0
2.5
2.0
1.5
ppm
58
60
62
64
66
68
70
72
74
76
4.4
4.2
4.0
3.8

^{1}H-^{13}C non-decoupled HSQC

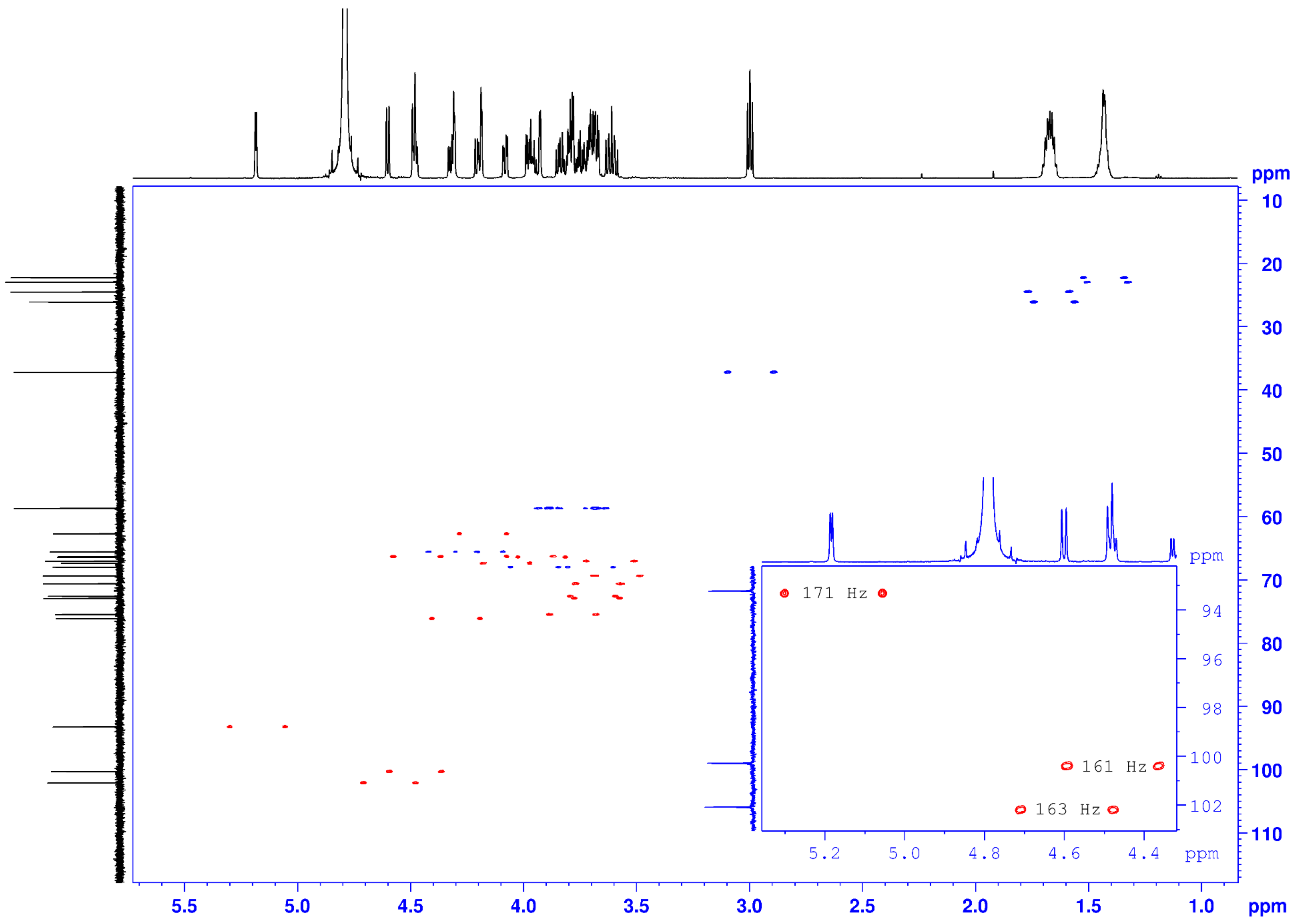

^{1}H-^{13}C HMBC

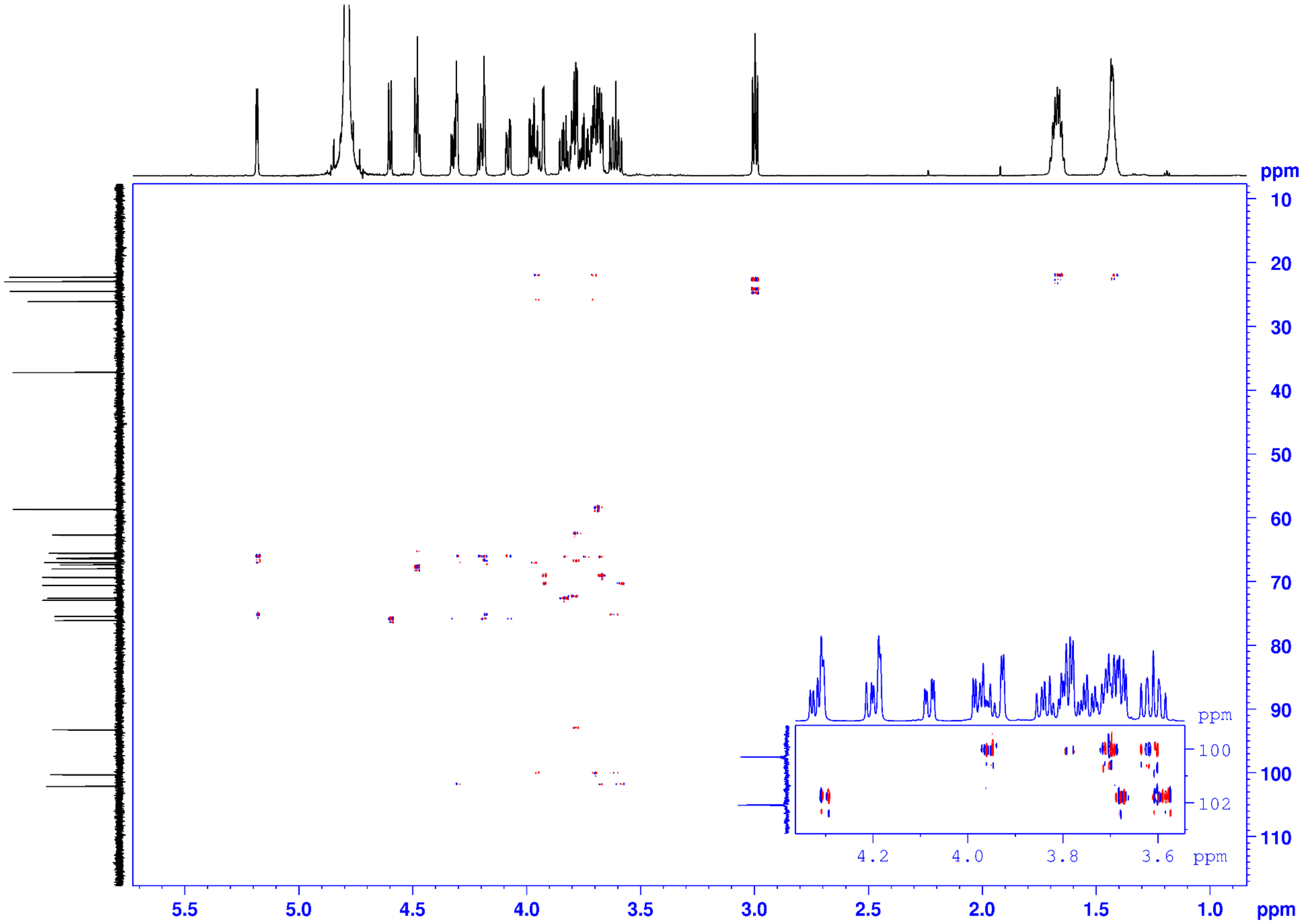

ppm
10
20
30
40
50
60
70
80
90
100
110
5.5
5.0
4.5
4.0
3.5
3.0
2.5
2.0
1.5
1.0
ppm
4.2
4.0
3.8
3.6
ppm
100
102

$^{13}C\{^{1}H\}$ NMR

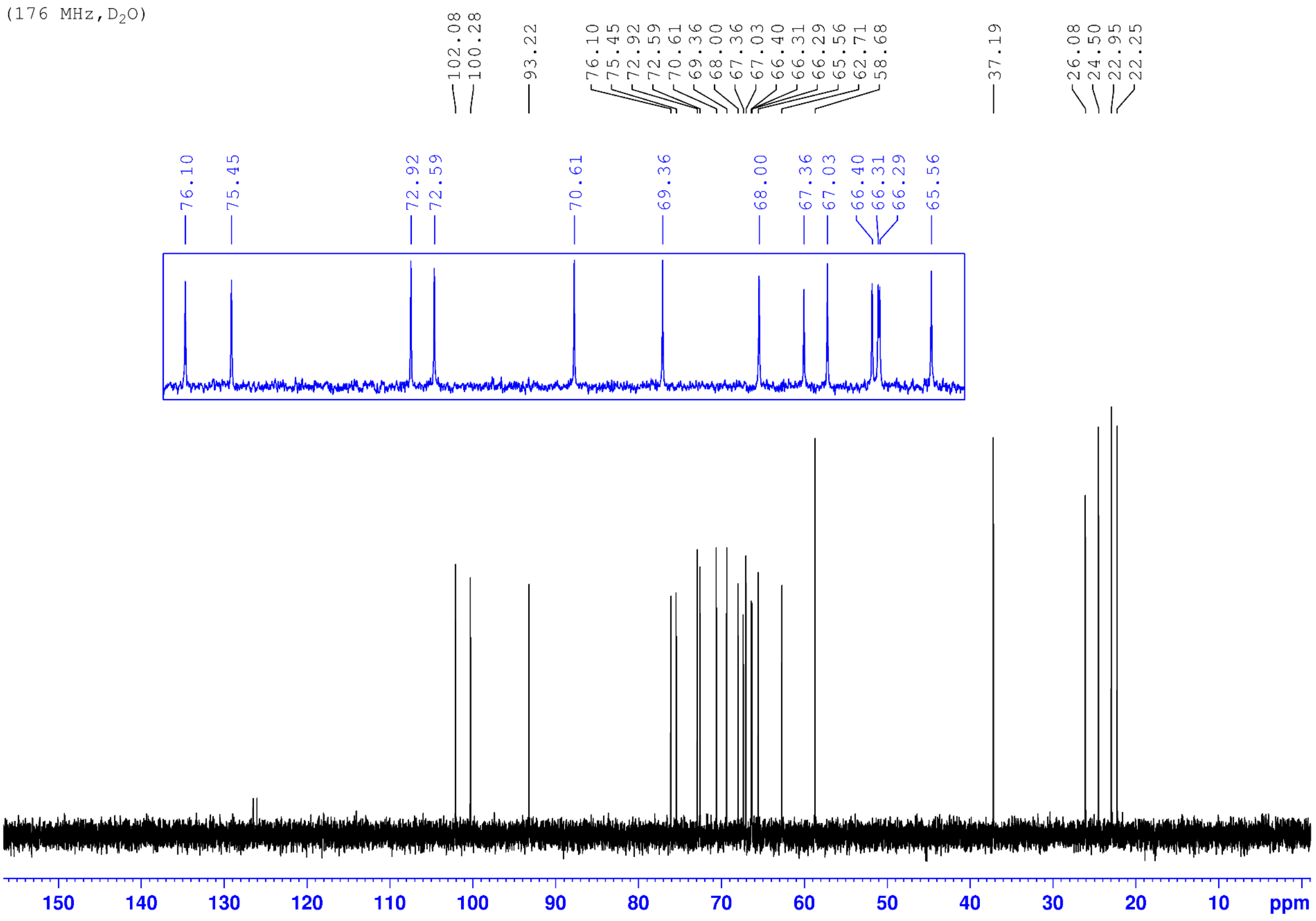

(176 MHz, D2O)
102.08
100.28
93.22
76.10
75.45
72.92
72.59
70.61
69.36
68.00
67.36
67.03
66.40
66.31
66.29
65.56
62.71
58.68
37.19
26.08
24.50
22.95
22.25
150
140
130
120
110
100
90
80
70
60
50
40
30
20
10
ppm

^{13}C DEPT-135

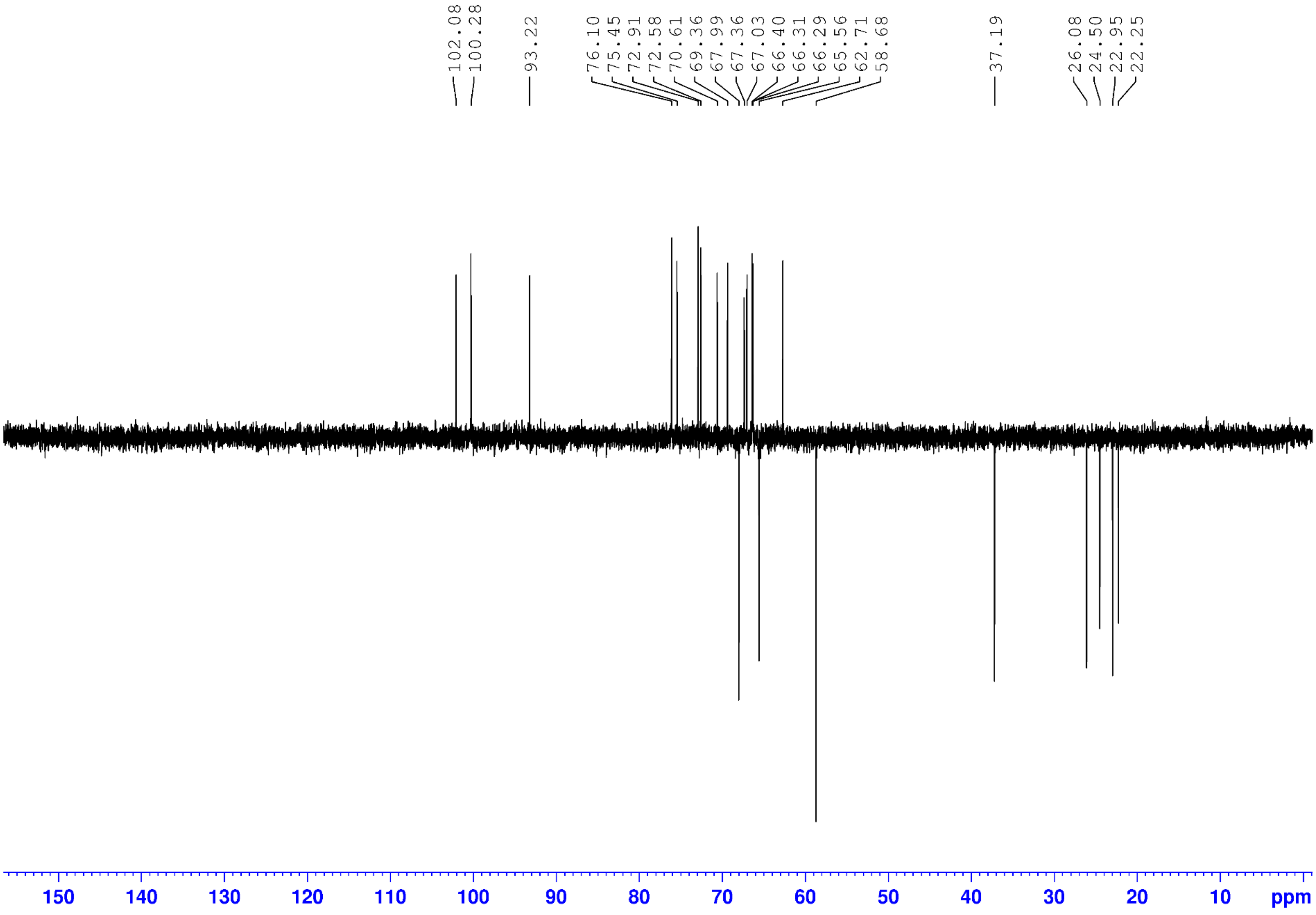

102.08
100.28
93.22
76.10
75.45
72.91
72.58
70.61
69.36
67.99
67.36
67.03
66.40
66.31
66.29
65.56
62.71
58.68
37.19
26.08
24.50
22.95
22.25
150
140
130
120
110
100
90
80
70
60
50
40
30
20
10
ppm

Compound **nST**

^{1}H-NMR

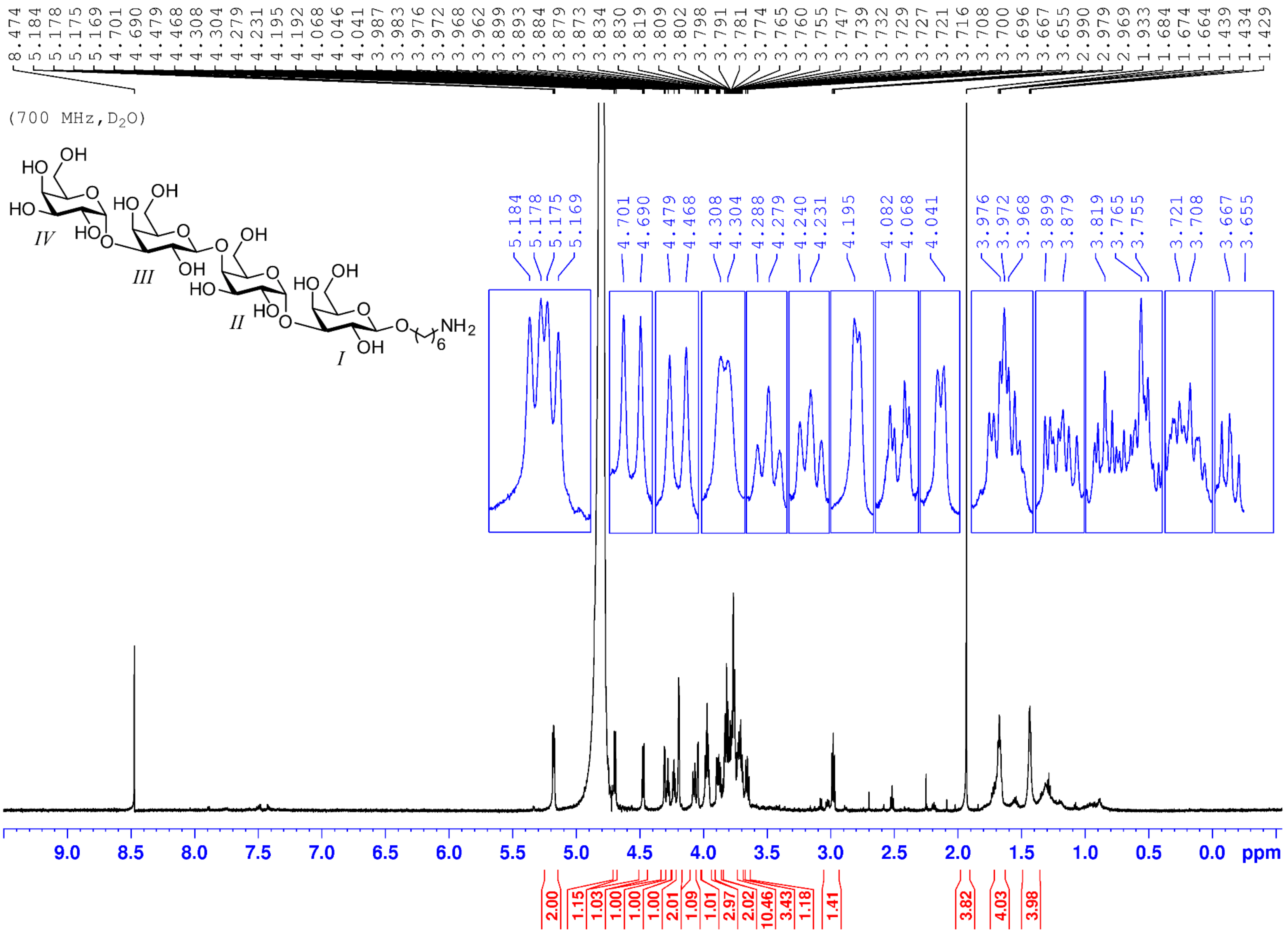

^{1}H-^{1}H COSY

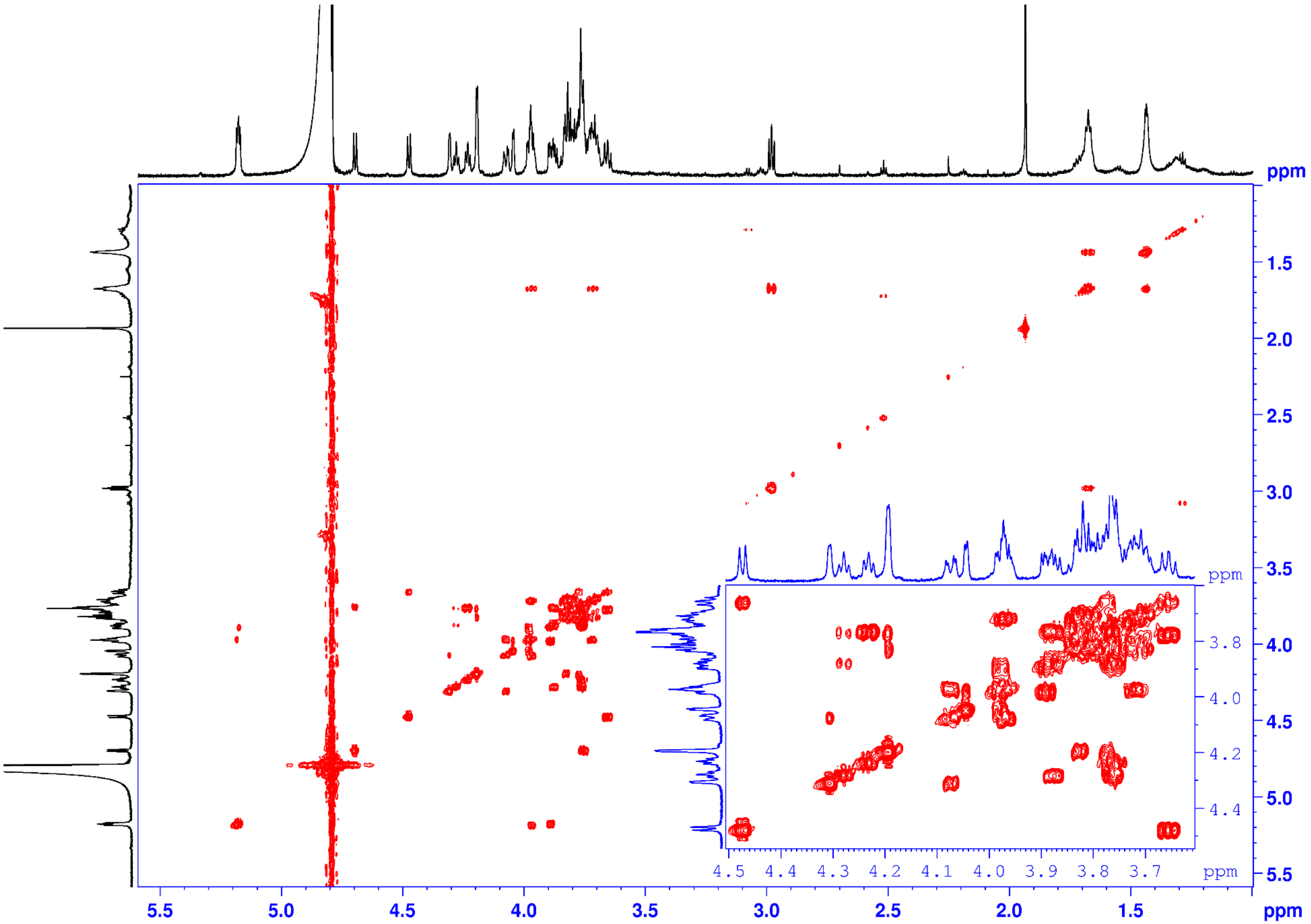

ppm
1.5
2.0
2.5
3.0
3.5
4.0
4.5
5.0
5.5
5.5
5.0
4.5
4.0
3.5
3.0
2.5
2.0
1.5
ppm
4.5 4.4 4.3 4.2 4.1 4.0 3.9 3.8 3.7 ppm
3.8
4.0
4.2
4.4

^{1}H-^{1}H TOCSY

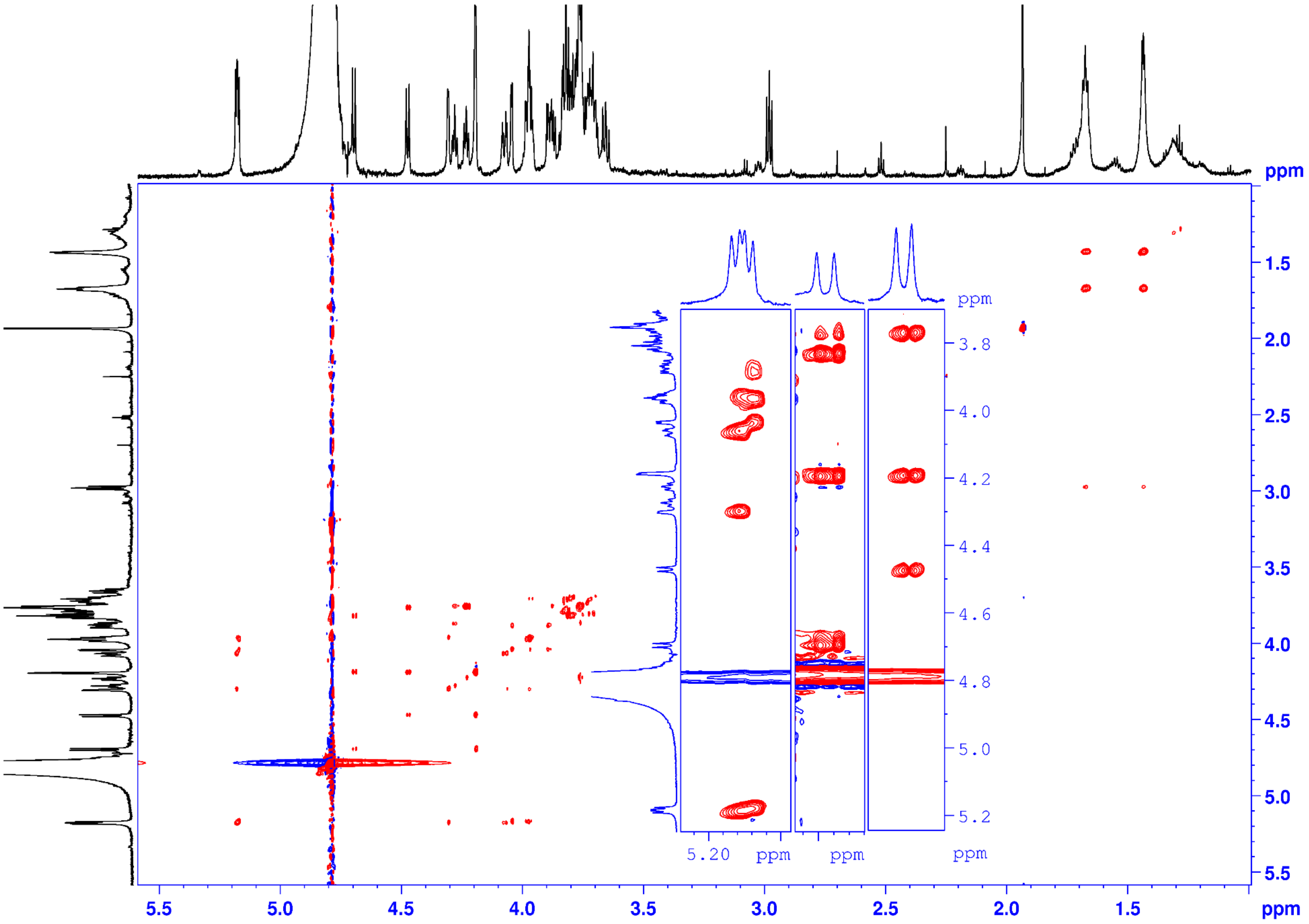

ppm
1.5
2.0
2.5
3.0
3.5
4.0
4.5
5.0
5.5
5.5 5.0 4.5 4.0 3.5 3.0 2.5 2.0 1.5 ppm
3.8
4.0
4.2
4.4
4.6
4.8
5.0
5.2
5.20 ppm

^1H-^1H COSY (enlarged section)

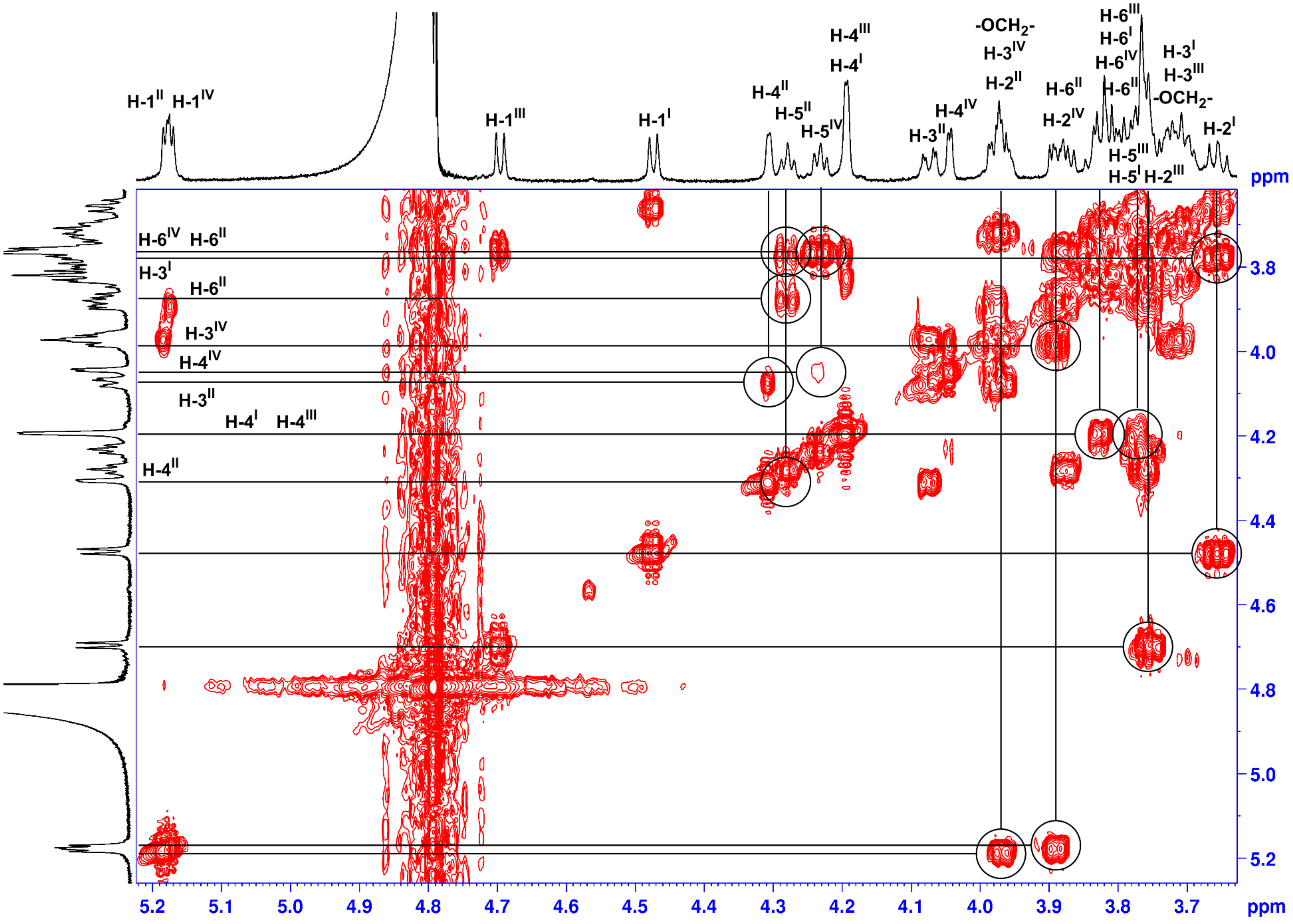

^{1}H-^{13}C HMQC

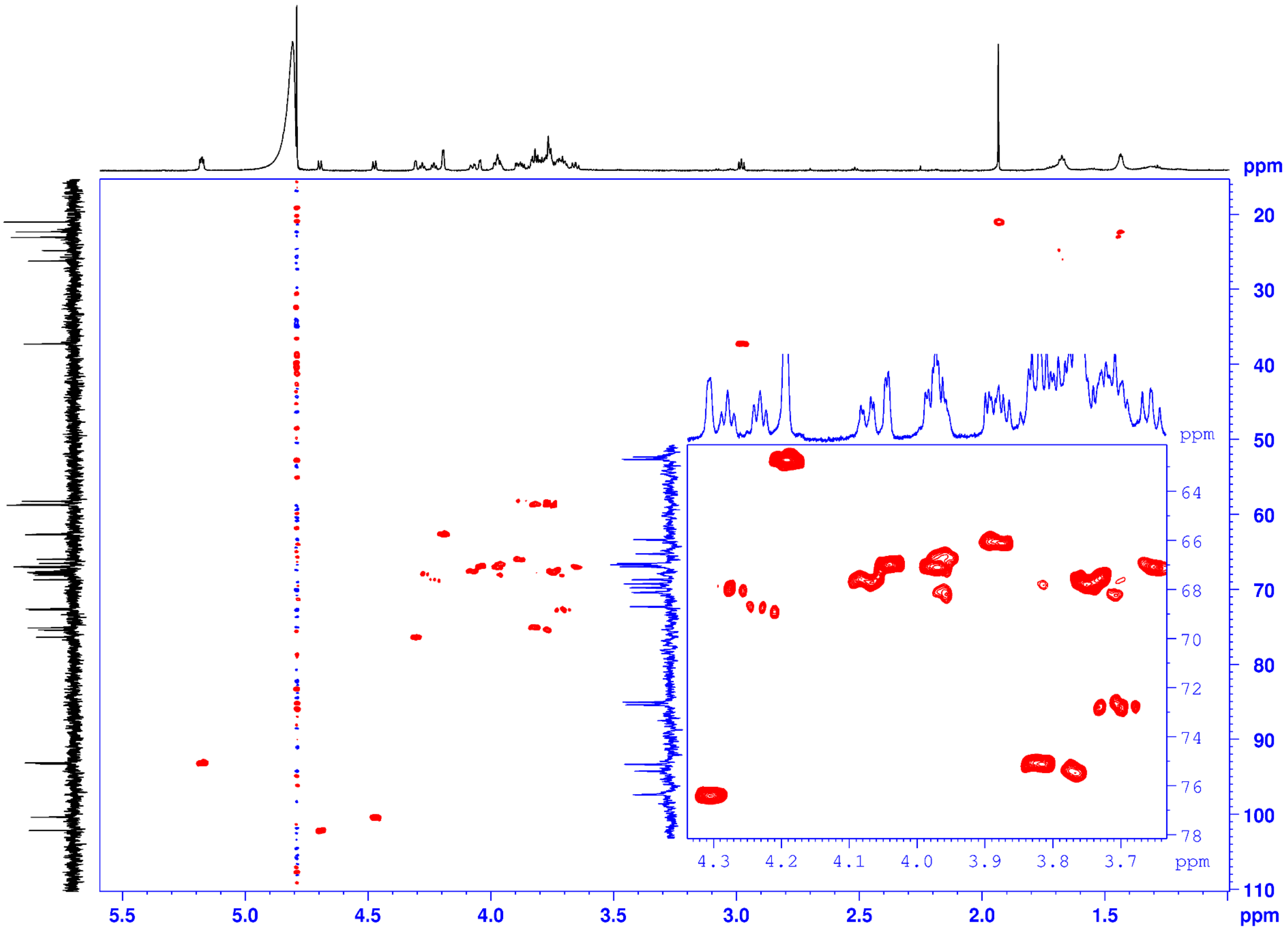

ppm
20
30
40
50
60
70
80
90
100
110
ppm
5.5
5.0
4.5
4.0
3.5
3.0
2.5
2.0
1.5
ppm
64
66
68
70
72
74
76
78
ppm
4.3
4.2
4.1
4.0
3.9
3.8
3.7
ppm

^{1}H-^{13}C unfiltered $^{1}J_{CH}$ non-decoupled HMBC

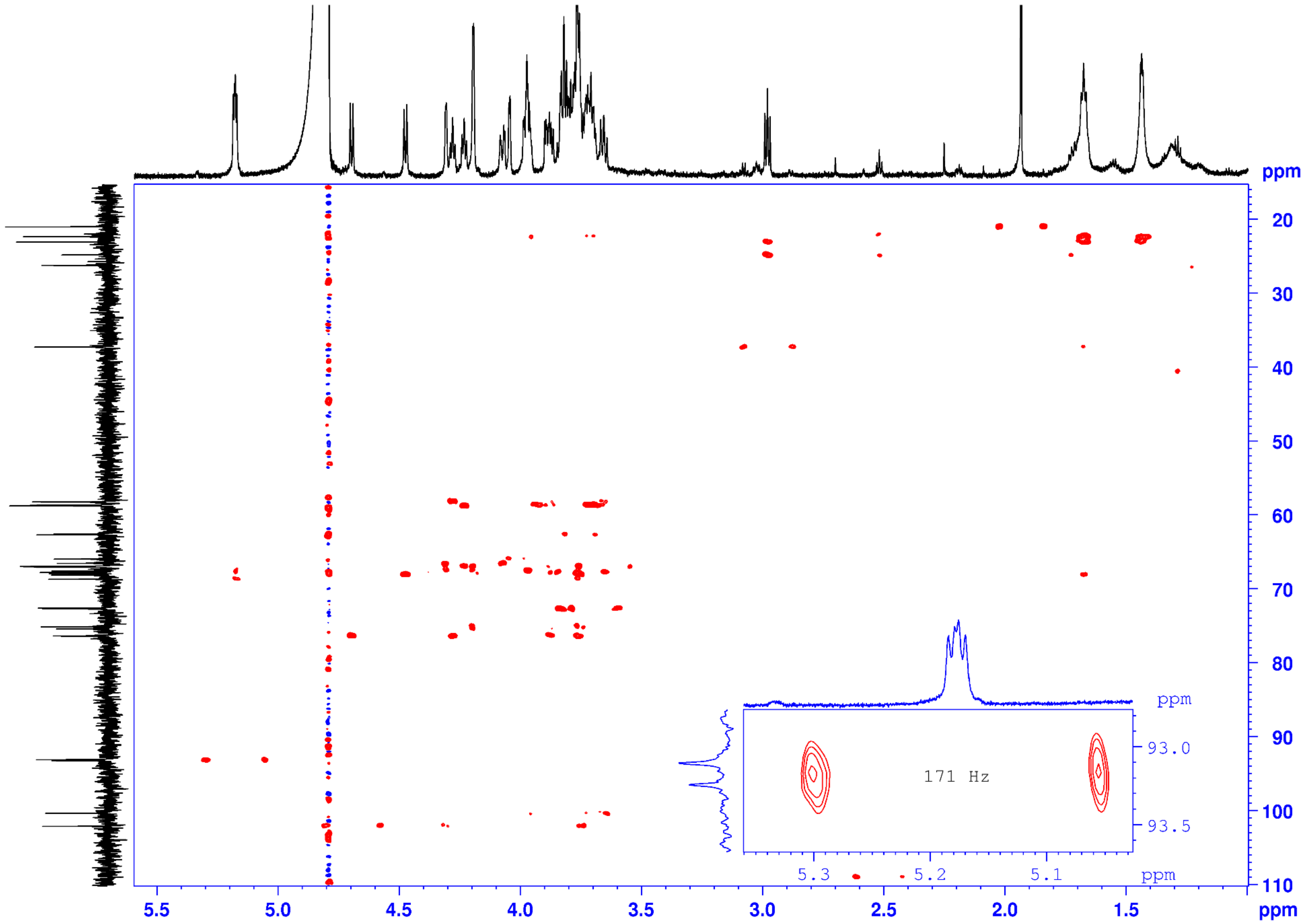

$^{13}C\{^{1}H\}$ NMR

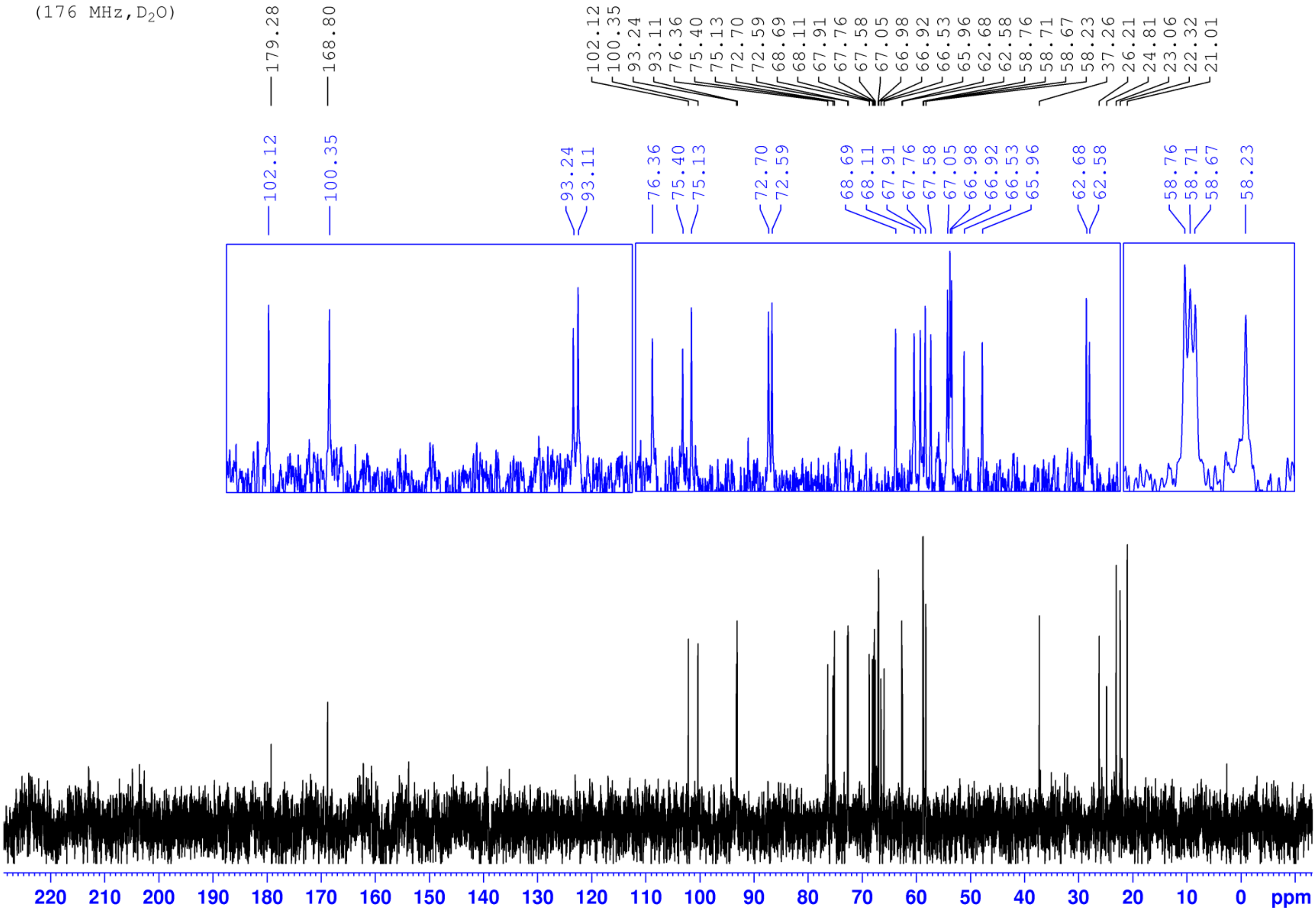

(176 MHz, D2O)
179.28
168.80
102.12
100.35
93.24
93.11
76.36
75.40
75.13
72.70
72.59
68.69
68.11
67.91
67.76
67.58
67.05
66.98
66.92
66.53
65.96
62.68
62.58
58.76
58.71
58.67
58.23
37.26
26.21
24.81
23.06
22.32
21.01
ppm

^{13}C DEPT-135

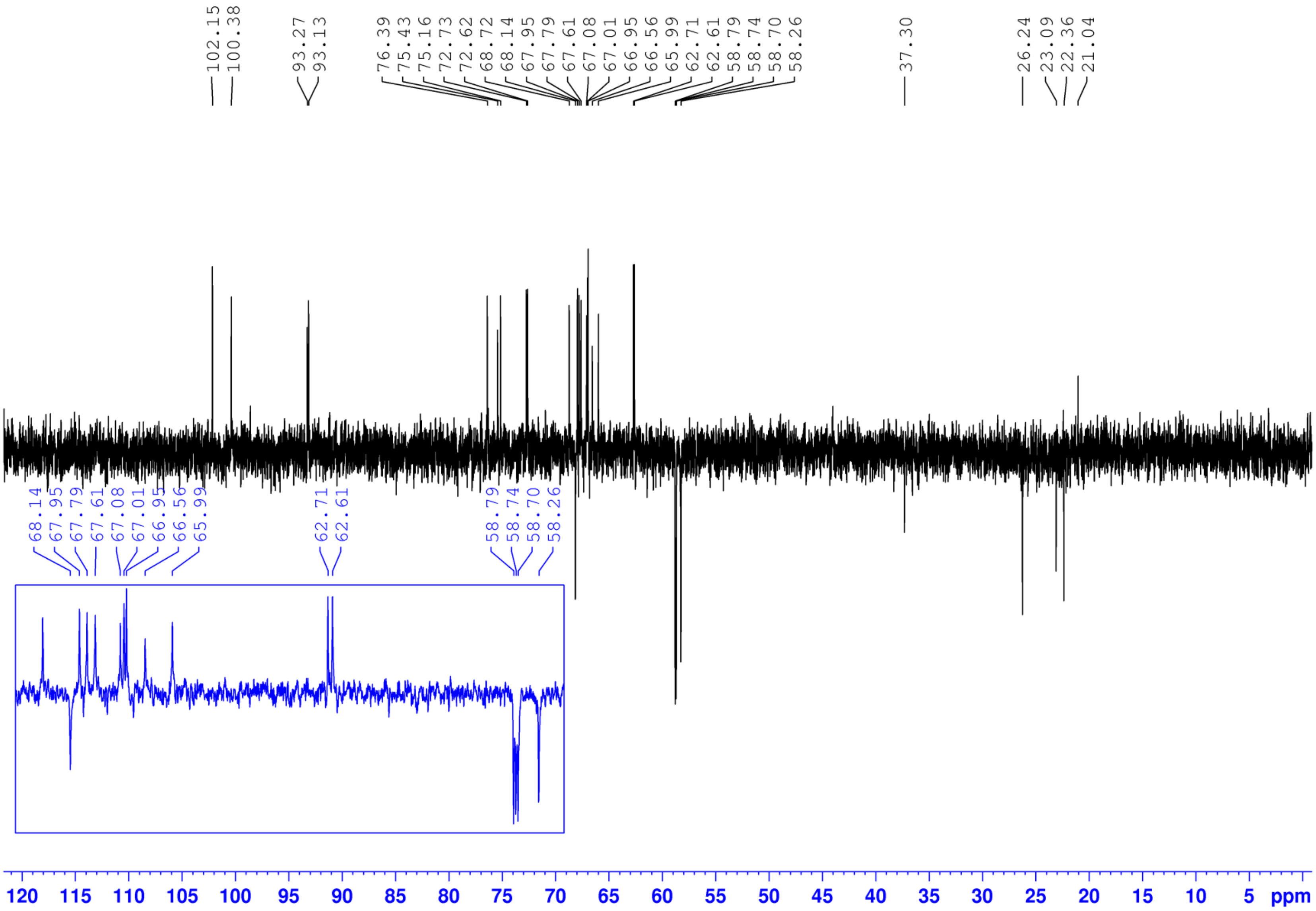
102.15
100.38
93.27
93.13
76.39
75.43
75.16
72.73
72.62
68.72
68.14
67.95
67.79
67.61
67.08
67.01
66.95
66.56
65.99
62.71
62.61
58.79
58.74
58.70
58.26
37.30
26.24
23.09
22.36
21.04
ppm

Compound **mST**

^{1}H-NMR

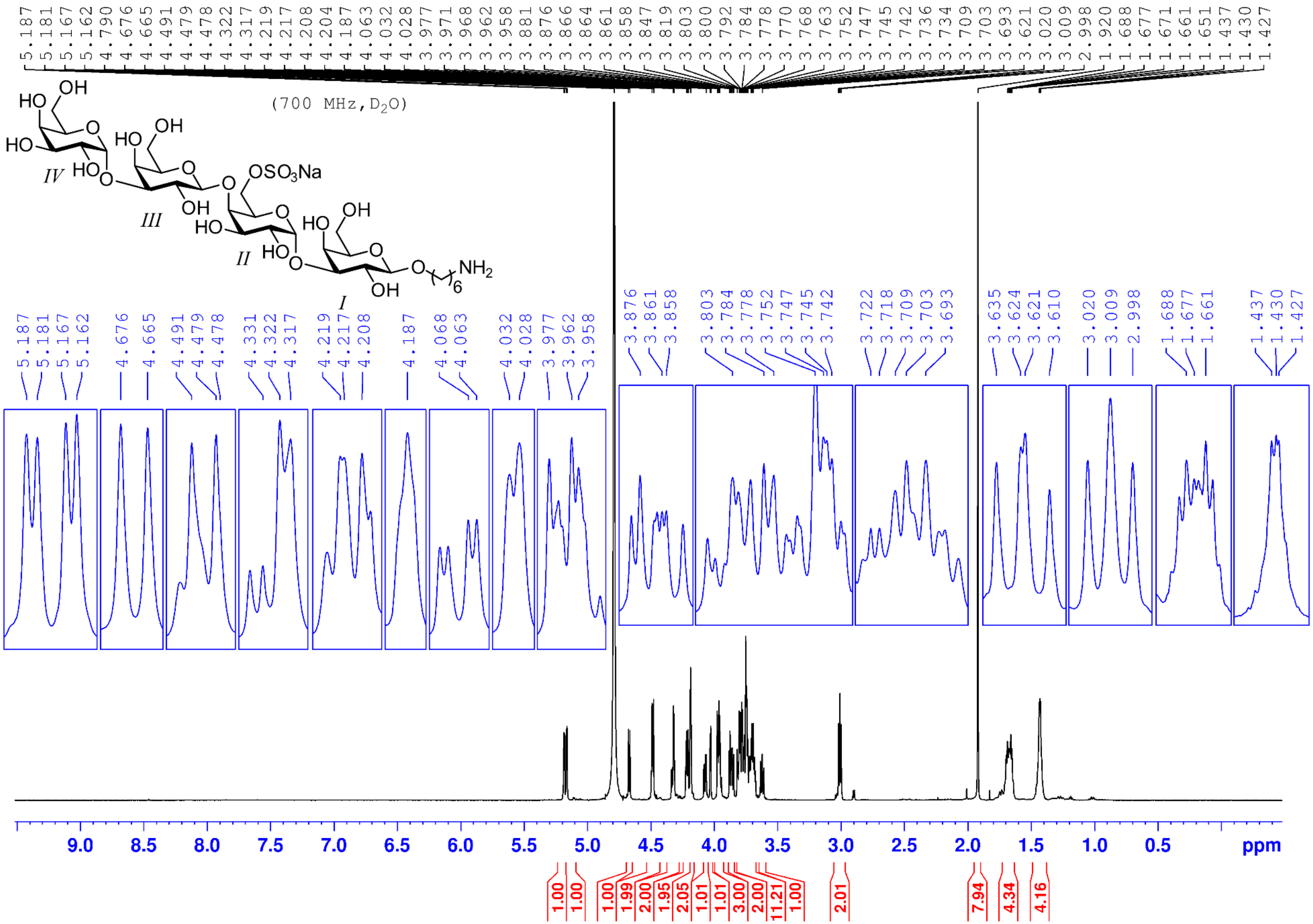

(700 MHz, D_2O)

^{1}H-^{1}H COSY

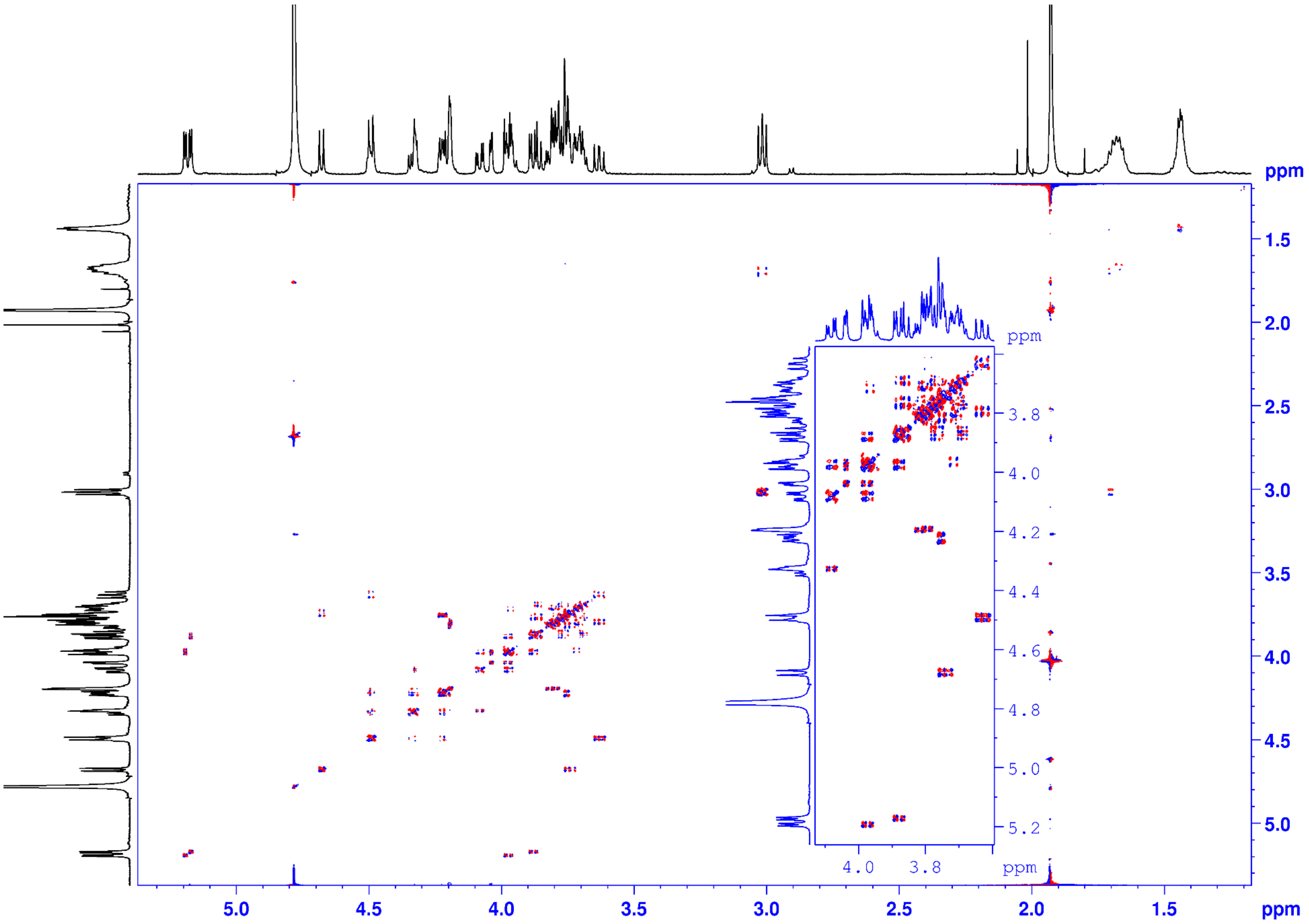

ppm
5.0
4.5
4.0
3.5
3.0
2.5
2.0
1.5
ppm
1.5
2.0
2.5
3.0
3.5
4.0
4.5
5.0
ppm
4.0
3.8
ppm
3.8
4.0
4.2
4.4
4.6
4.8
5.0
5.2

^1H-^1H COSY (enlarged section)

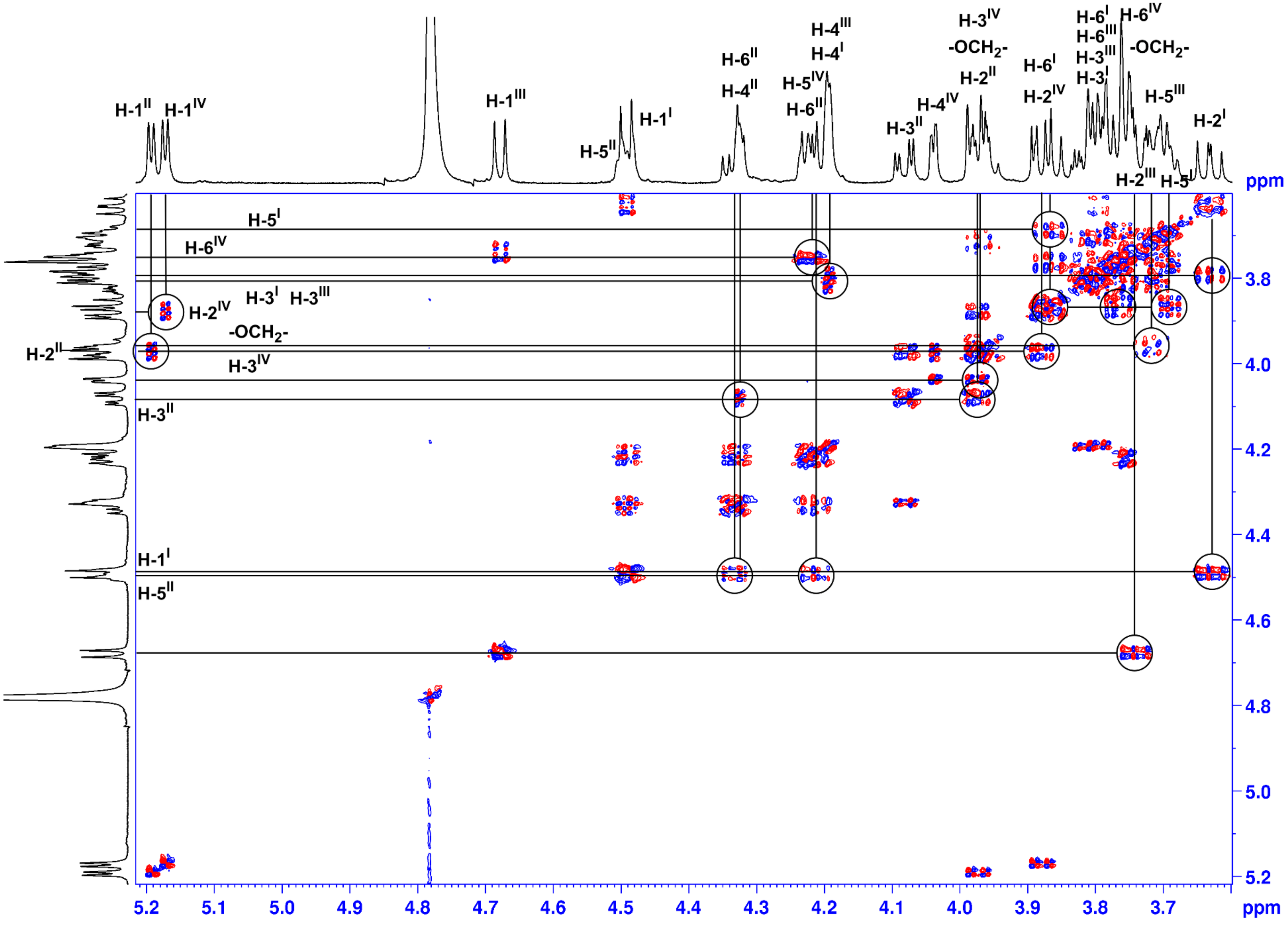

[1]H-^{1}H TOCSY

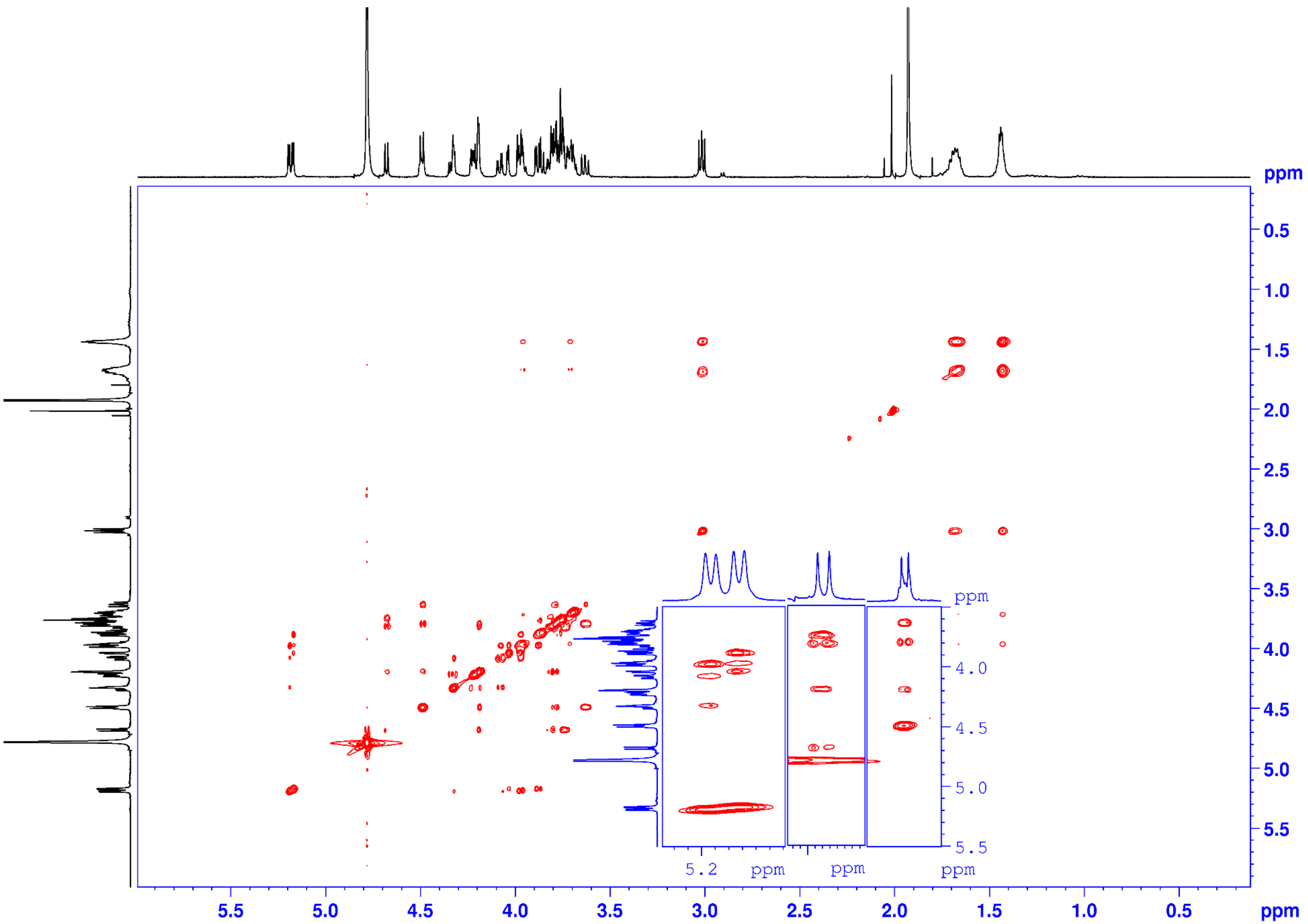

ppm
0.5
1.0
1.5
2.0
2.5
3.0
3.5
4.0
4.5
5.0
5.5
5.5
5.0
4.5
4.0
3.5
3.0
2.5
2.0
1.5
1.0
0.5
ppm

$^{1}H-^{13}C$ HSQC

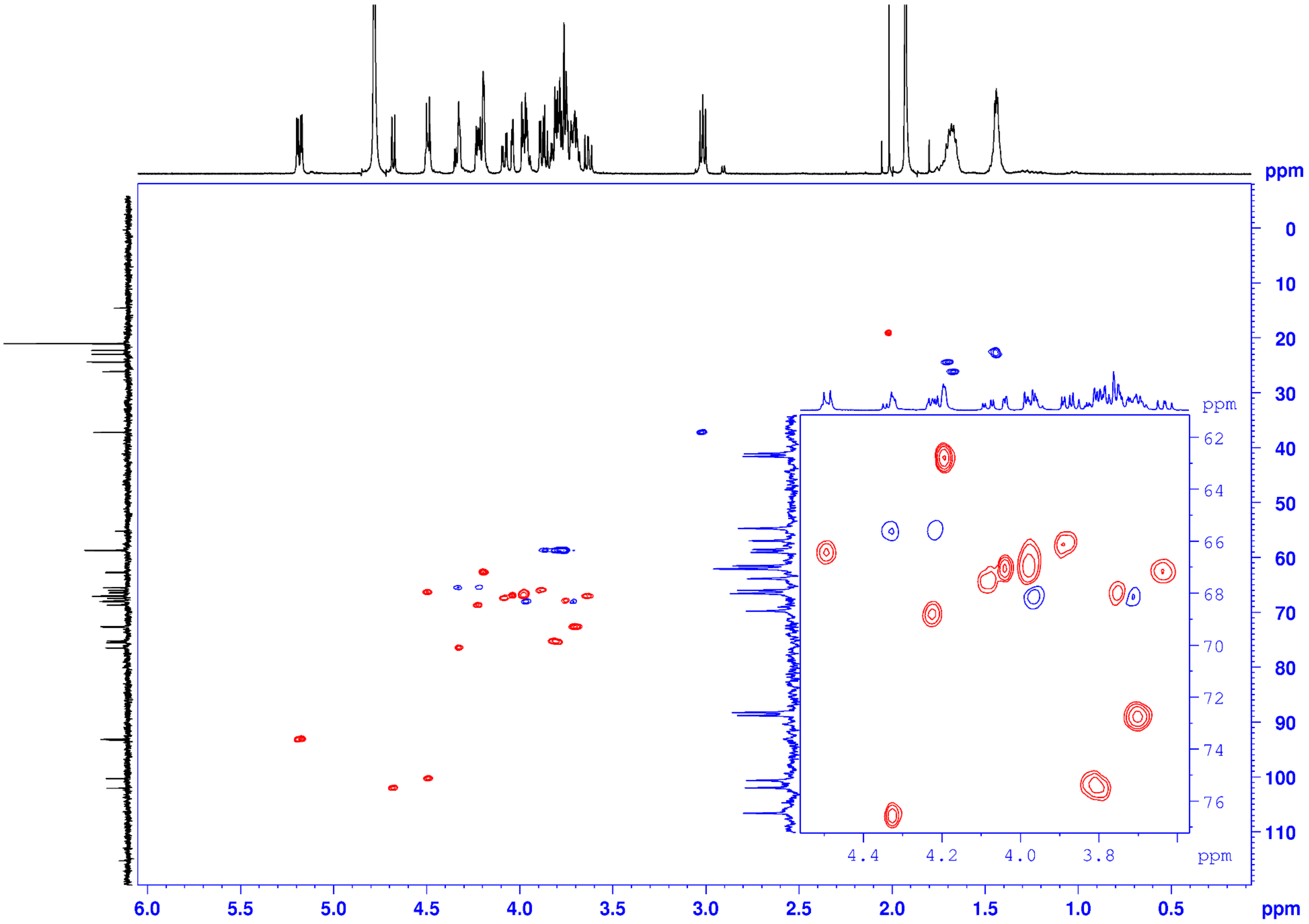
ppm
0
10
20
30
40
50
60
70
80
90
100
110
6.0
5.5
5.0
4.5
4.0
3.5
3.0
2.5
2.0
1.5
1.0
0.5
ppm
62
64
66
68
70
72
74
76
ppm
4.4
4.2
4.0
3.8
ppm

^{1}H-^{13}C non-decoupled HSQC

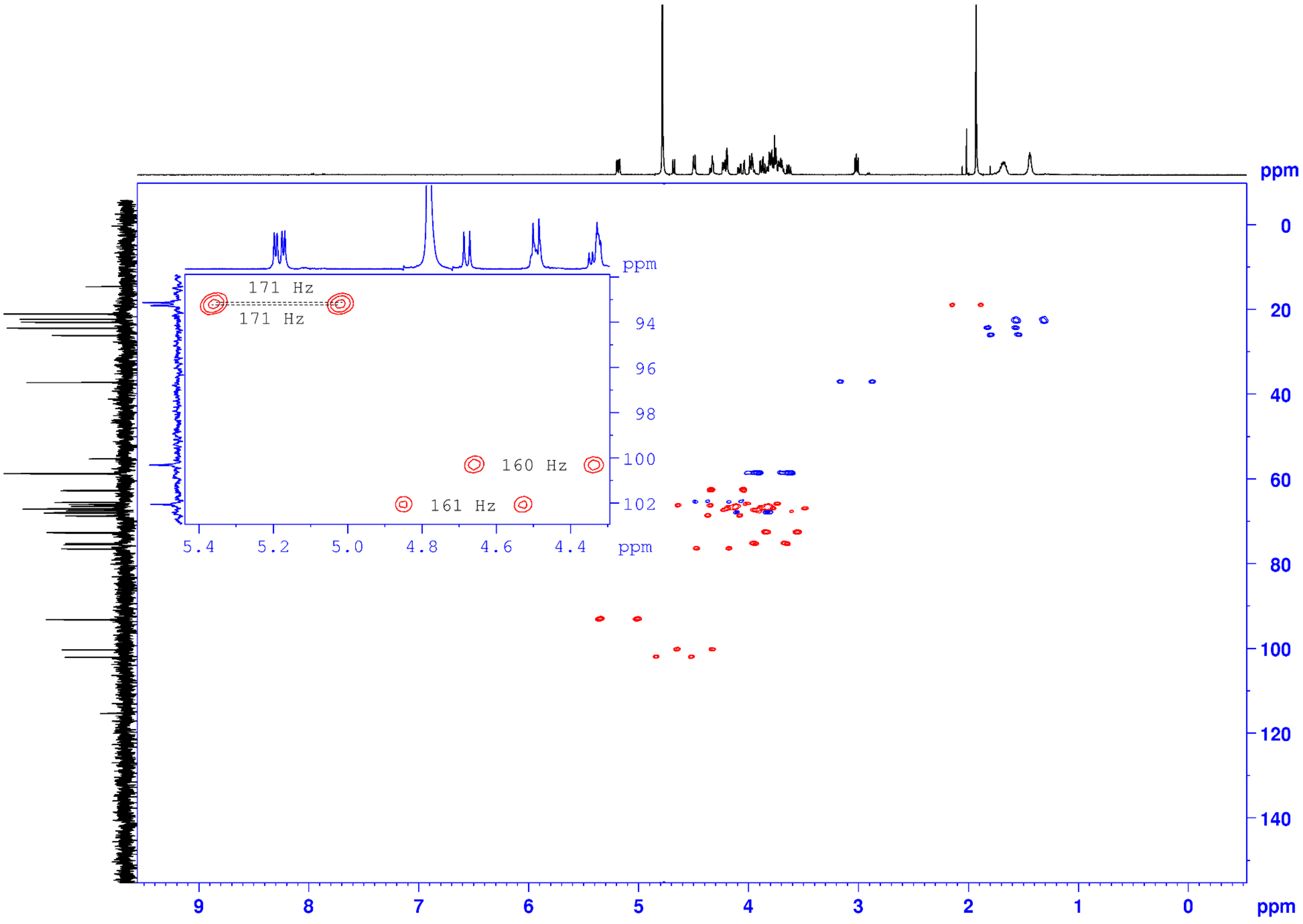

$^{13}C\{^1H\}$ NMR

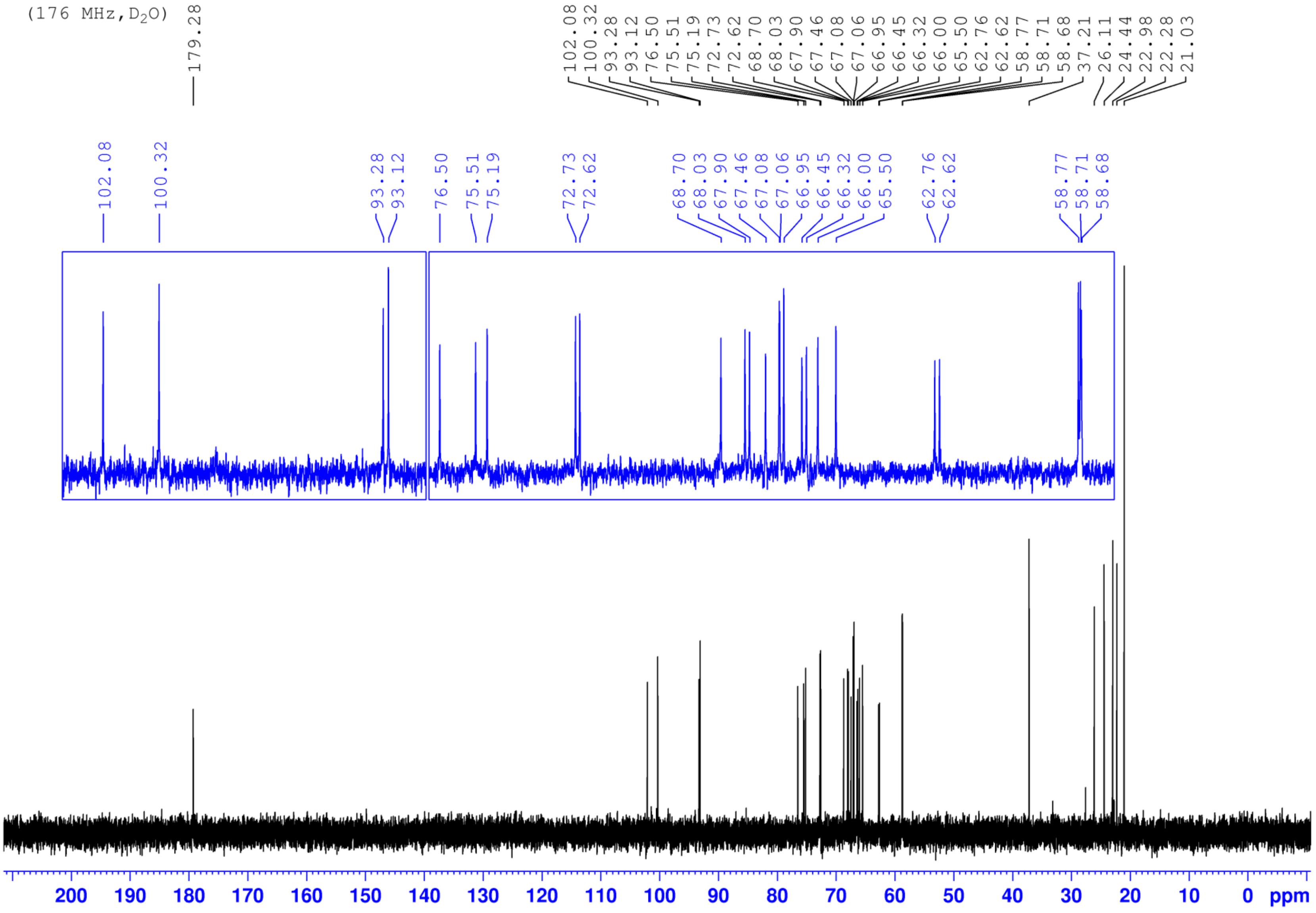
(176 MHz, D_2O)
179.28
102.08
100.32
93.28
93.12
76.50
75.51
75.19
72.73
72.62
68.70
68.03
67.90
67.46
67.08
67.06
66.95
66.45
66.32
66.00
65.50
62.76
62.62
58.77
58.71
58.68
37.21
26.11
24.44
22.98
22.28
21.03
200
190
180
170
160
150
140
130
120
110
100
90
80
70
60
50
40
30
20
10
0
ppm

^{13}C DEPT-135

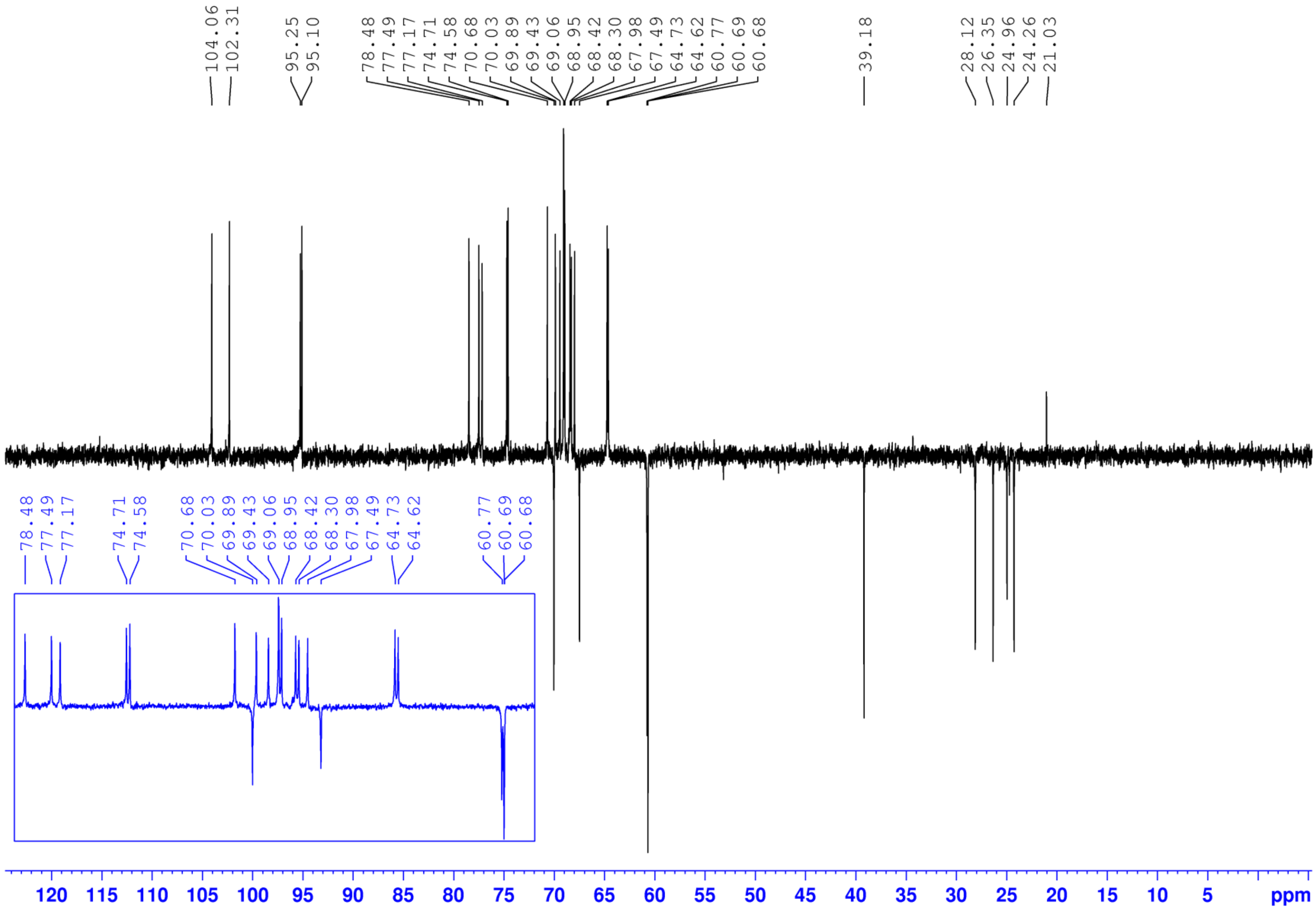

104.06
102.31
95.25
95.10
78.48
77.49
77.17
74.71
74.58
70.68
70.03
69.89
69.43
69.06
68.95
68.42
68.30
67.98
67.49
64.73
64.62
60.77
60.69
60.68
39.18
28.12
26.35
24.96
24.26
21.03
120
115
110
105
100
95
90
85
80
75
70
65
60
55
50
45
40
35
30
25
20
15
10
5
ppm

Compound **1**

^{1}H-NMR

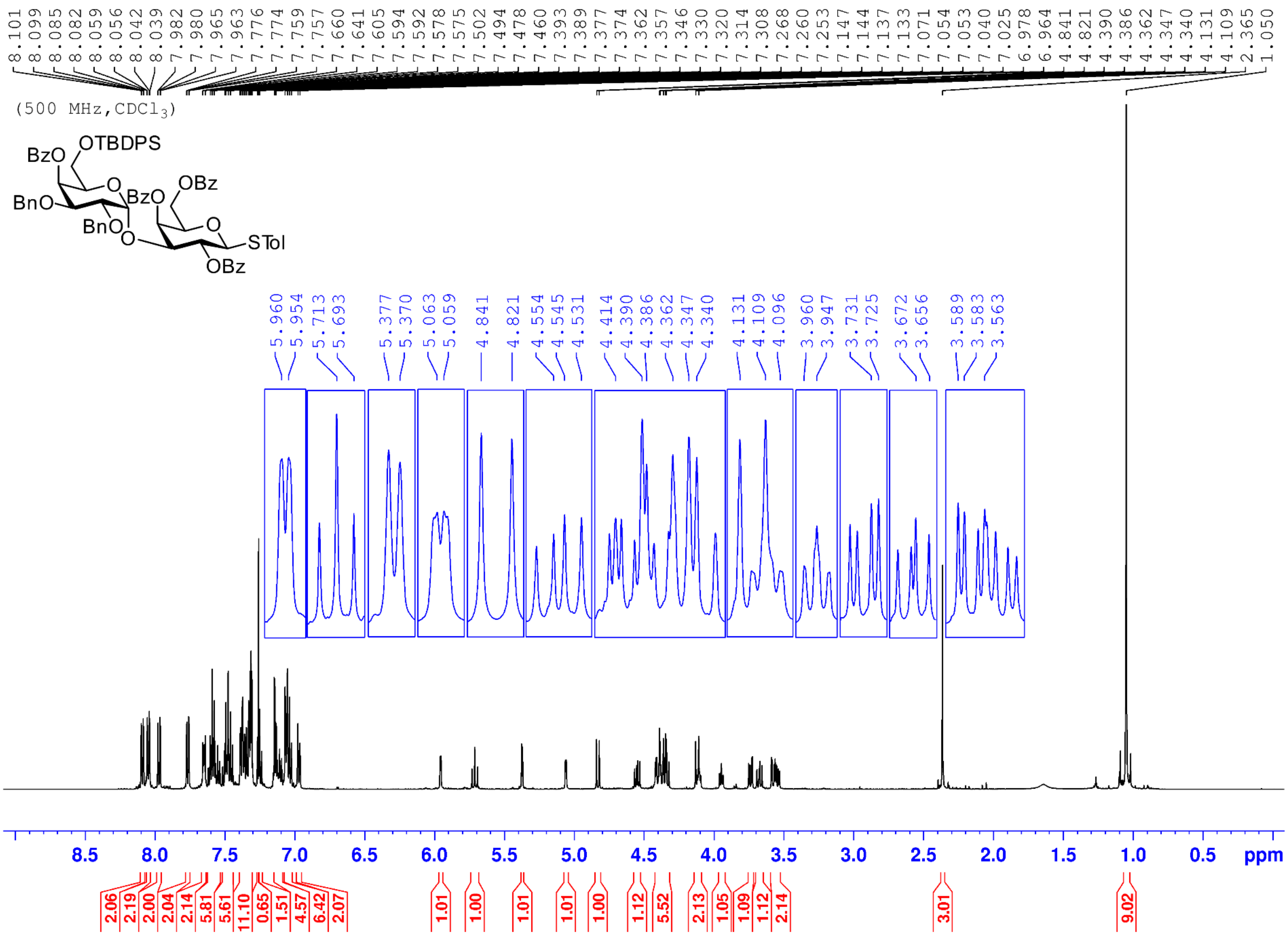

^{1}H-^{1}H COSY

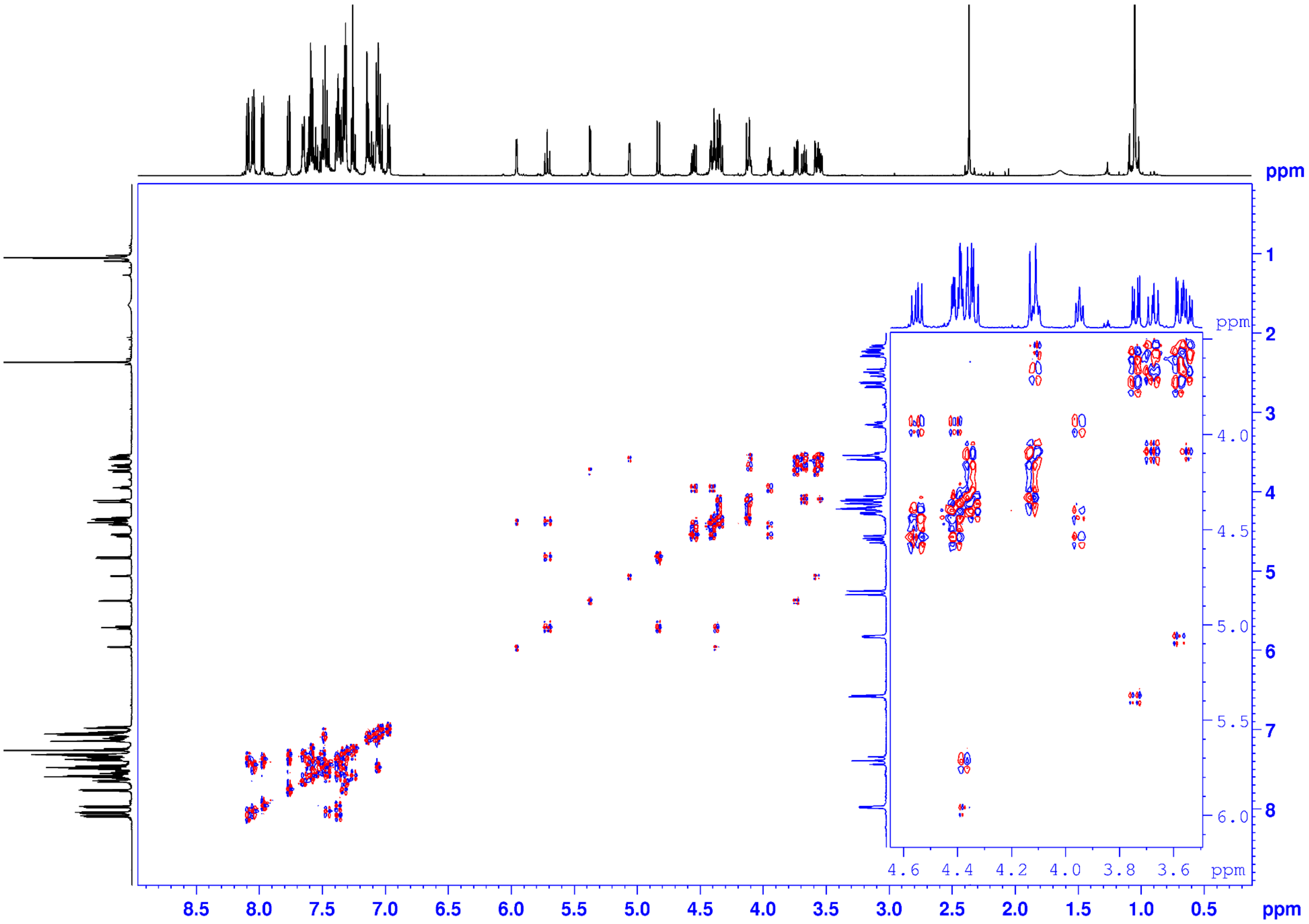

ppm
8.5
8.0
7.5
7.0
6.5
6.0
5.5
5.0
4.5
4.0
3.5
3.0
2.5
2.0
1.5
1.0
0.5
ppm
1
2
3
4
5
6
7
8
4.6
4.4
4.2
4.0
3.8
3.6
ppm
4.0
4.5
5.0
5.5
6.0

^{1}H-^{13}C HSQC

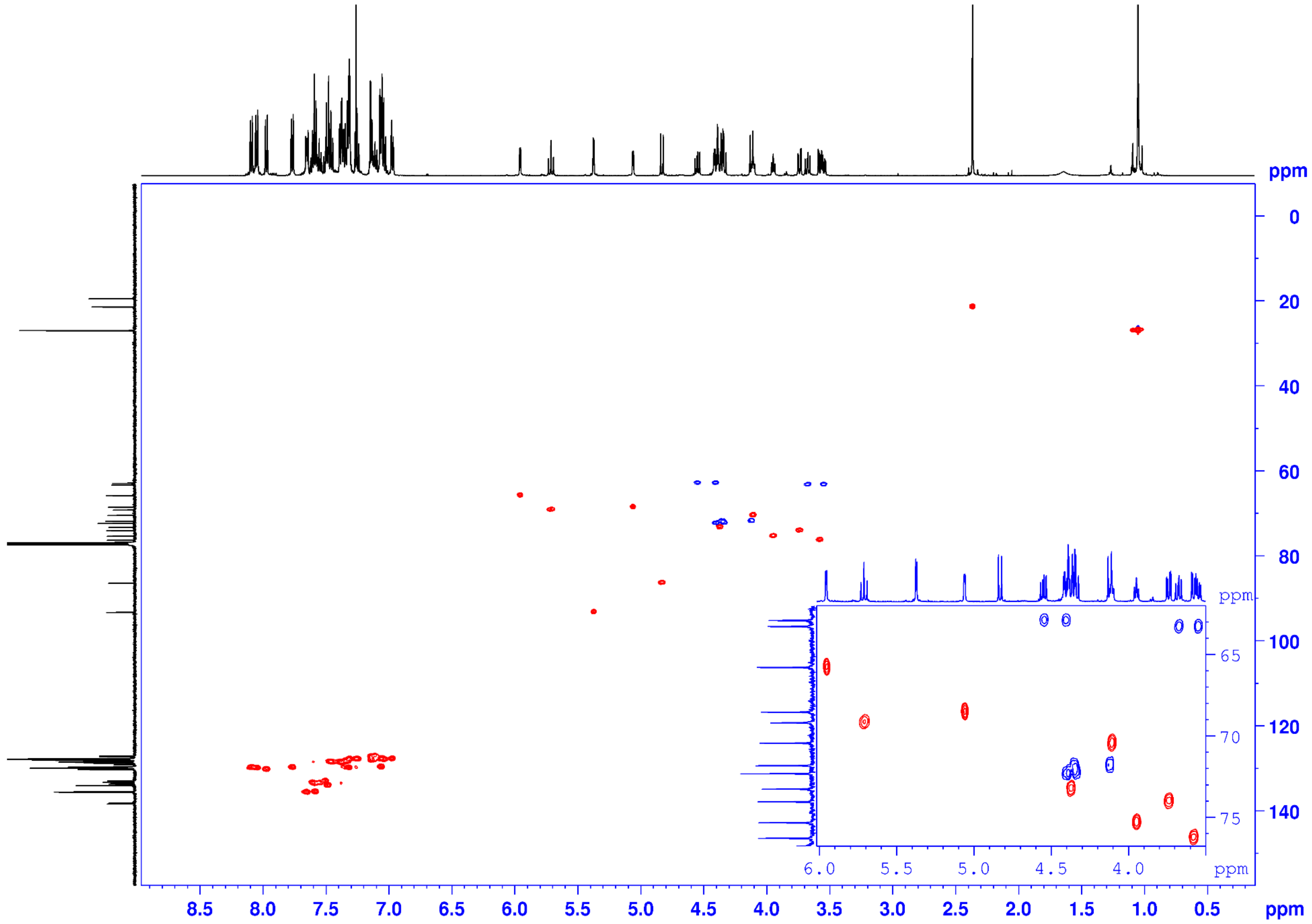

ppm
0
20
40
60
80
100
120
140
8.5
8.0
7.5
7.0
6.5
6.0
5.5
5.0
4.5
4.0
3.5
3.0
2.5
2.0
1.5
1.0
0.5
ppm
65
70
75
ppm
6.0
5.5
5.0
4.5
4.0
ppm

^{1}H-^{13}C HMBC

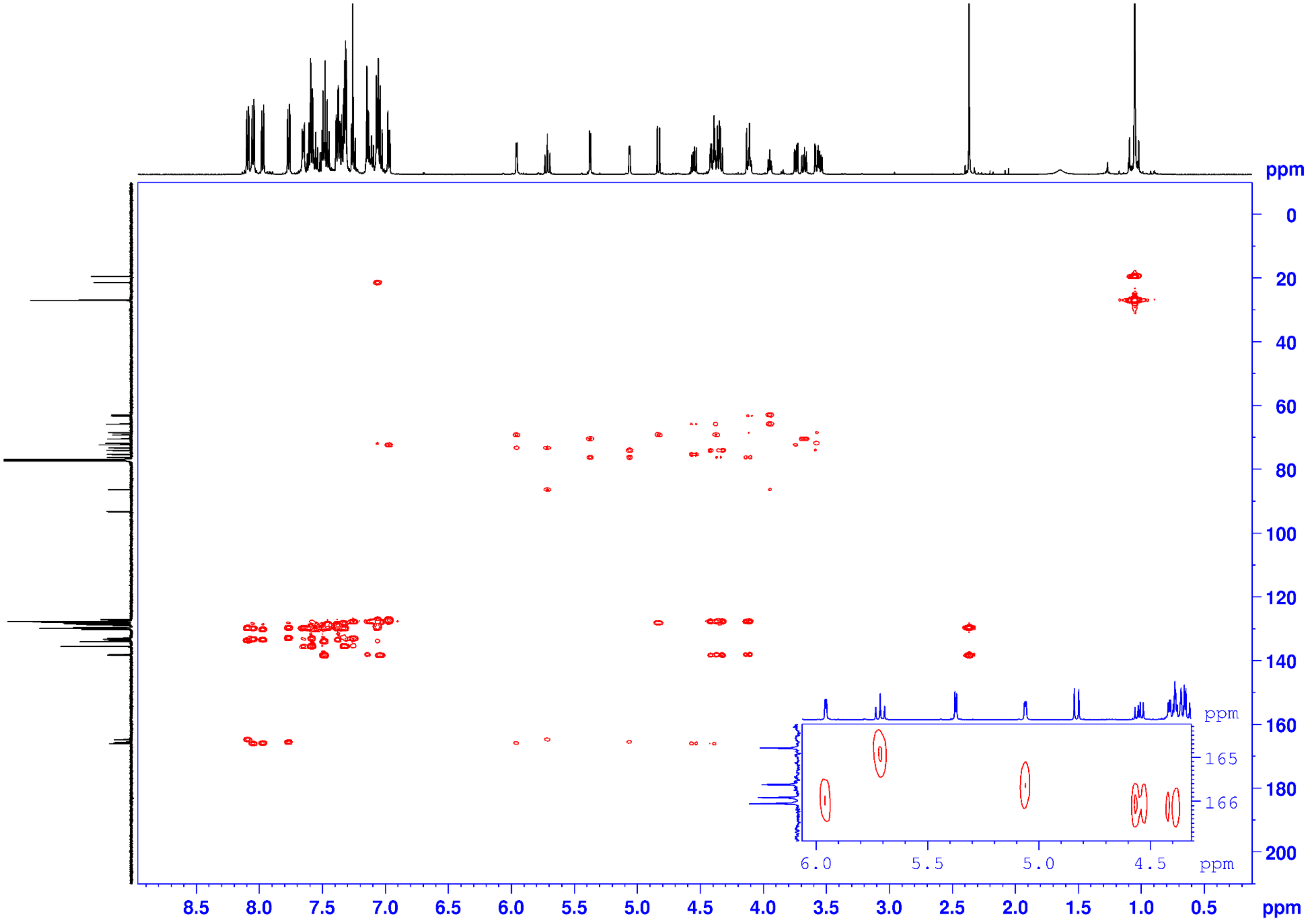
ppm
0
20
40
60
80
100
120
140
160
180
200
8.5
8.0
7.5
7.0
6.5
6.0
5.5
5.0
4.5
4.0
3.5
3.0
2.5
2.0
1.5
1.0
0.5
ppm
165
166
6.0
5.5
5.0
4.5
ppm

$^{13}C\{^1H\}$ NMR

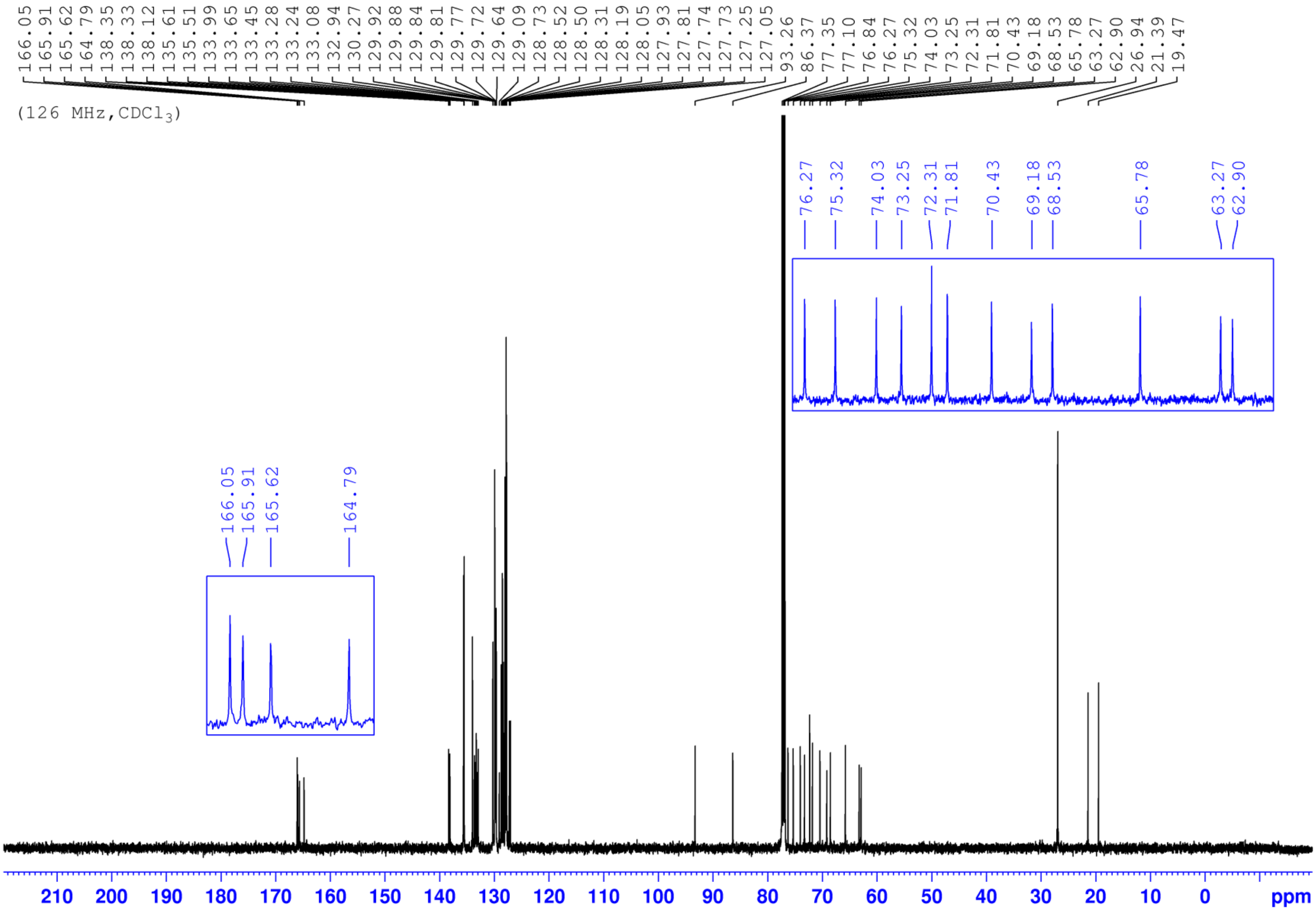

166.05
165.91
165.62
164.79
138.35
138.33
138.12
135.61
135.51
133.99
133.65
133.45
133.28
133.24
133.08
132.94
130.27
129.92
129.88
129.84
129.81
129.77
129.72
129.64
129.09
128.73
128.52
128.50
128.31
128.19
128.05
127.93
127.81
127.74
127.73
127.25
127.05
93.26
86.37
77.35
77.10
76.84
76.27
75.32
74.03
73.25
72.31
71.81
70.43
69.18
68.53
65.78
63.27
62.90
26.94
21.39
19.47
(126 MHz,CDCl3)
ppm

Compound **2**

^{1}H-NMR

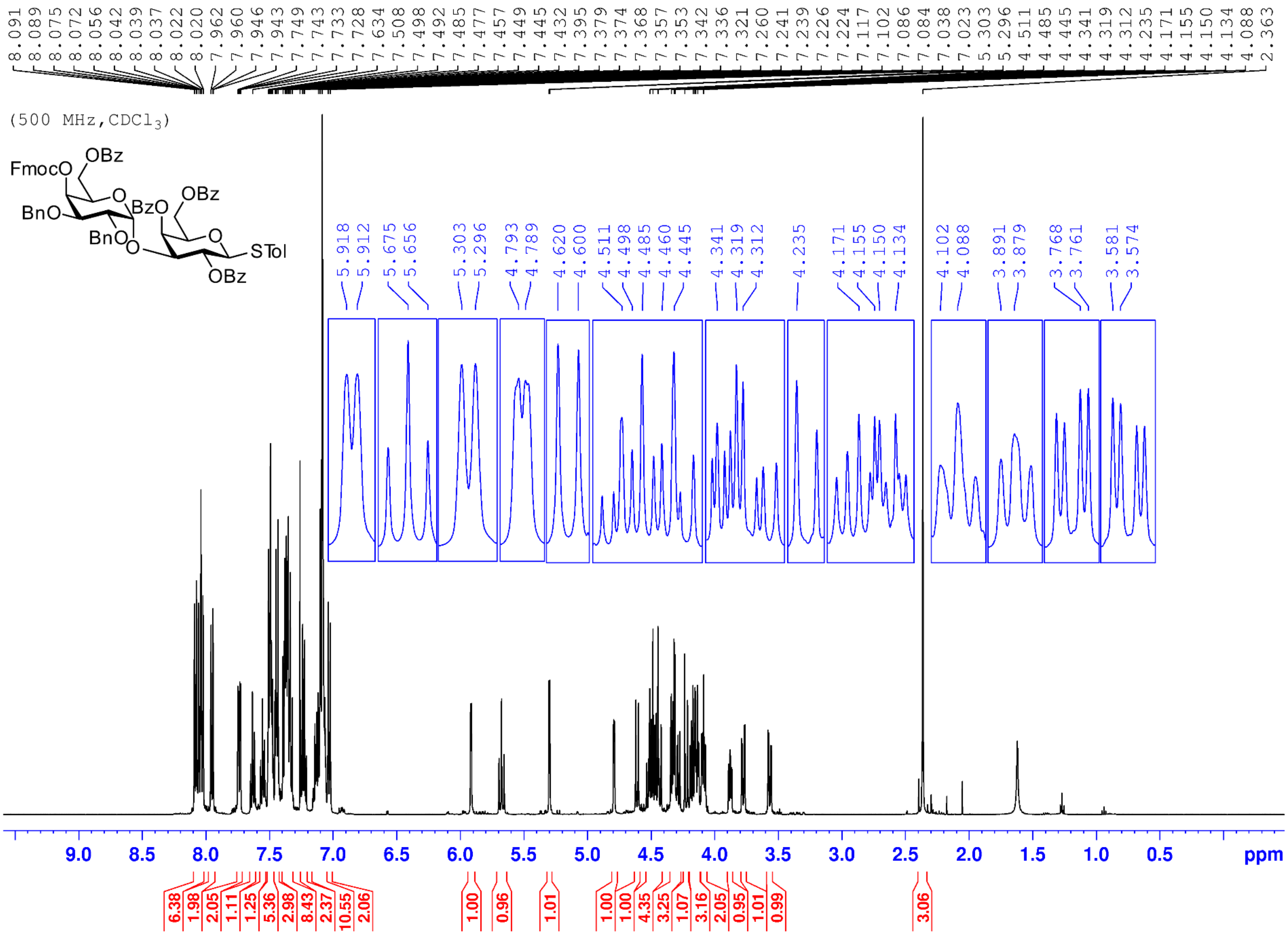

^{1}H-^{1}H COSY

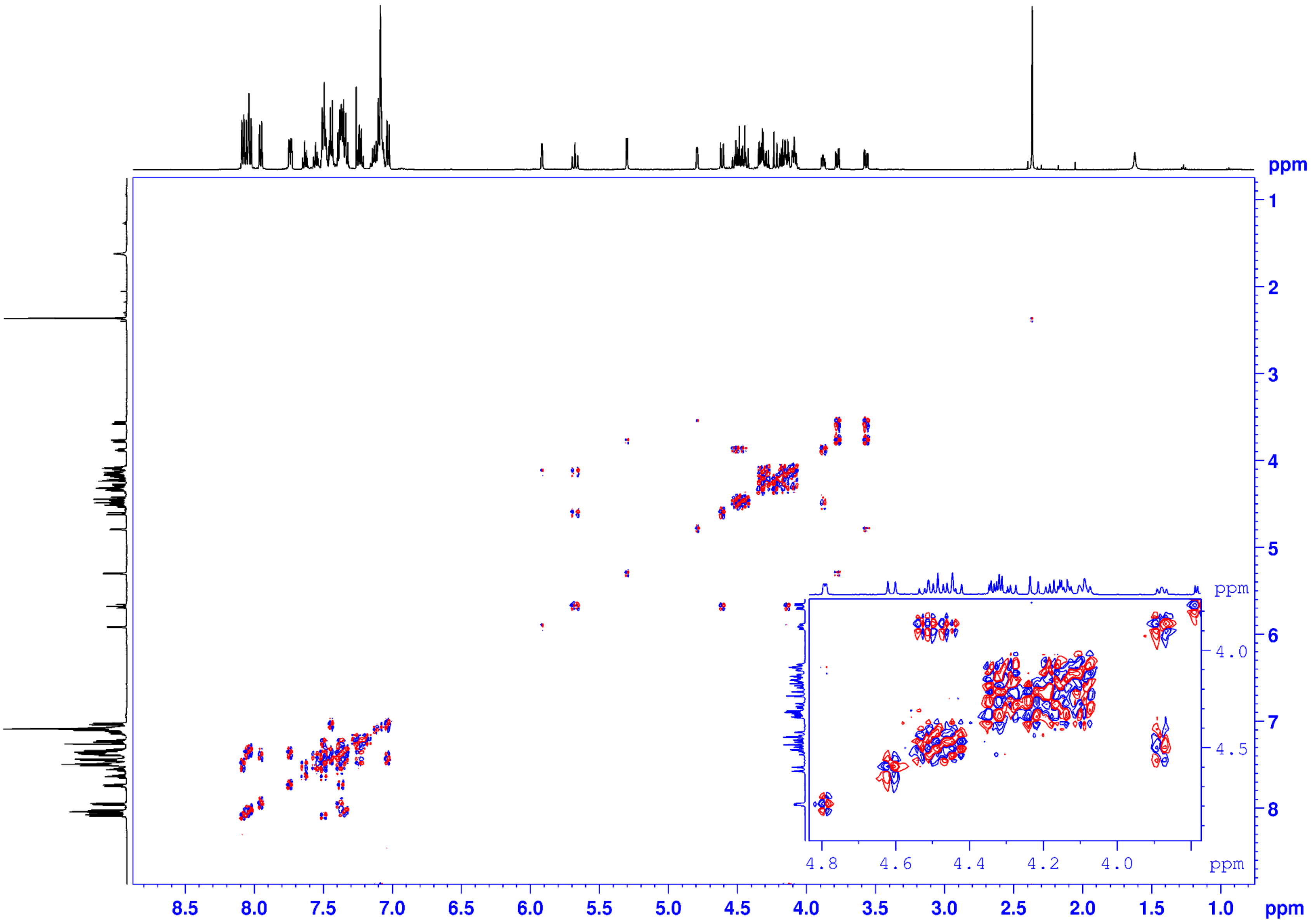
ppm
1
2
3
4
5
6
7
8
8.5
8.0
7.5
7.0
6.5
6.0
5.5
5.0
4.5
4.0
3.5
3.0
2.5
2.0
1.5
1.0
ppm
4.0
4.5
4.8
4.6
4.4
4.2
4.0
ppm

^{1}H-^{13}C HSQC

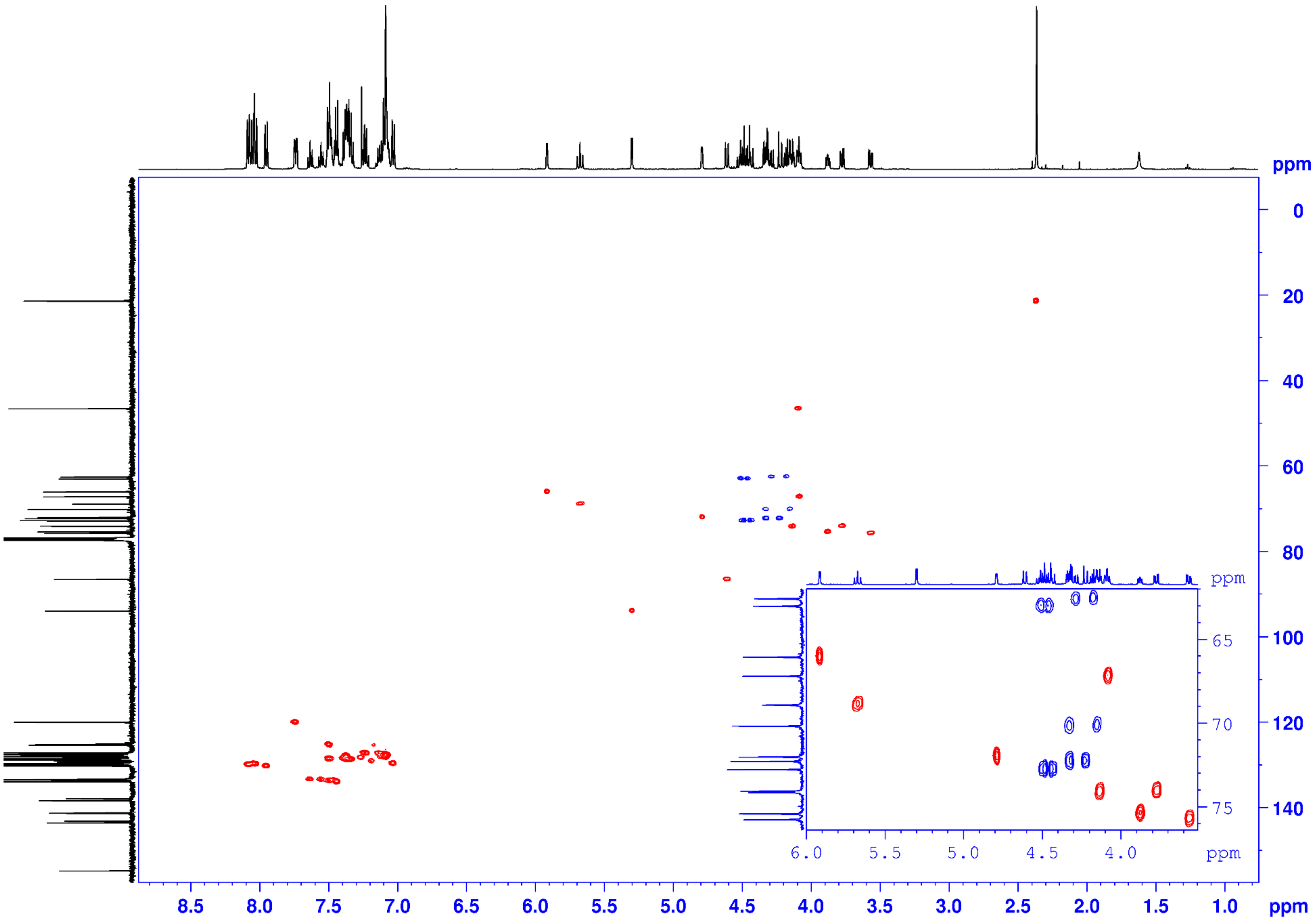

ppm
0
20
40
60
80
100
120
140
8.5
8.0
7.5
7.0
6.5
6.0
5.5
5.0
4.5
4.0
3.5
3.0
2.5
2.0
1.5
1.0
ppm
65
70
75
6.0
5.5
5.0
4.5
4.0
ppm

^{1}H-^{13}C non-decoupled HSQC

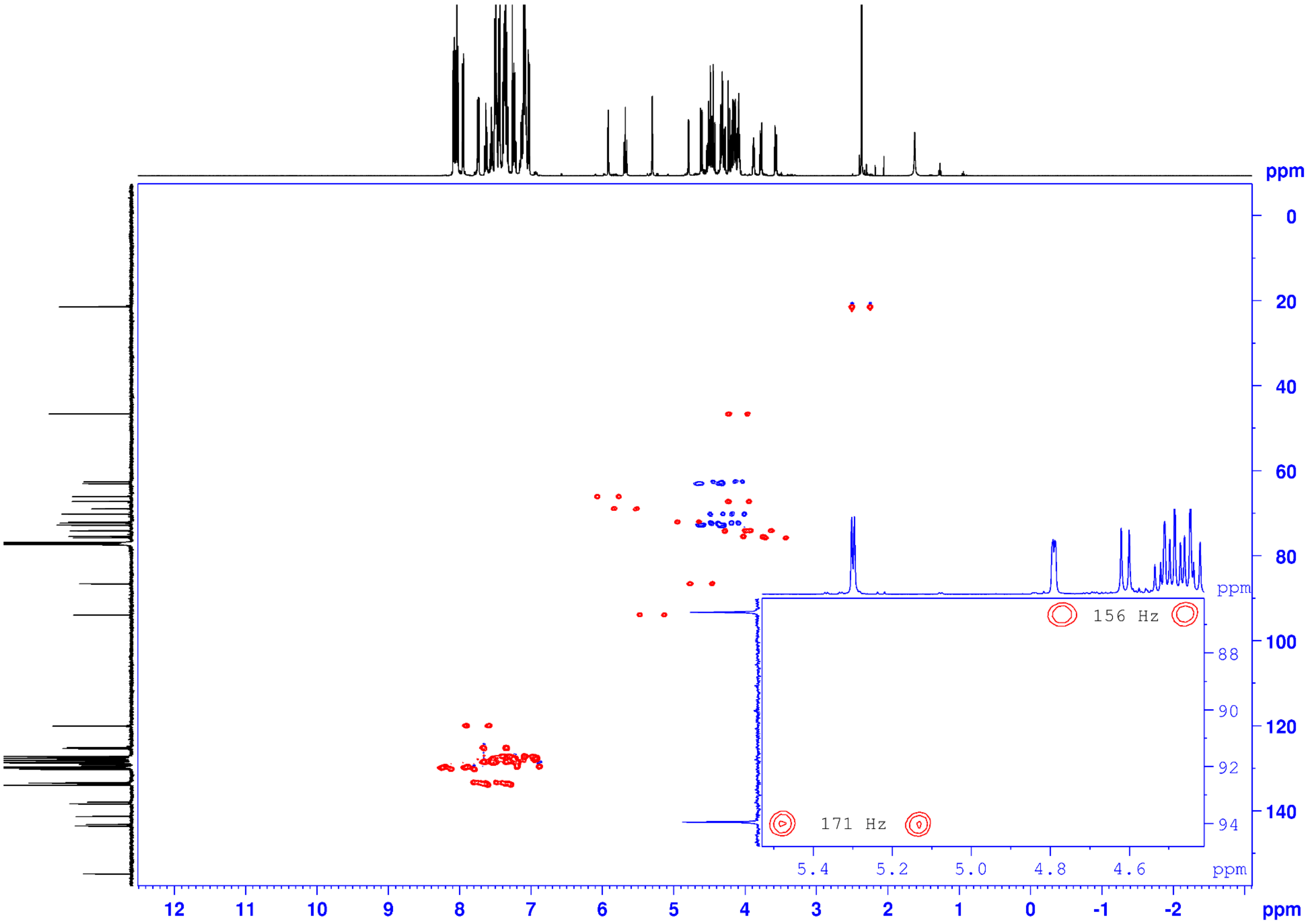

^{1}H-^{13}C HMBC

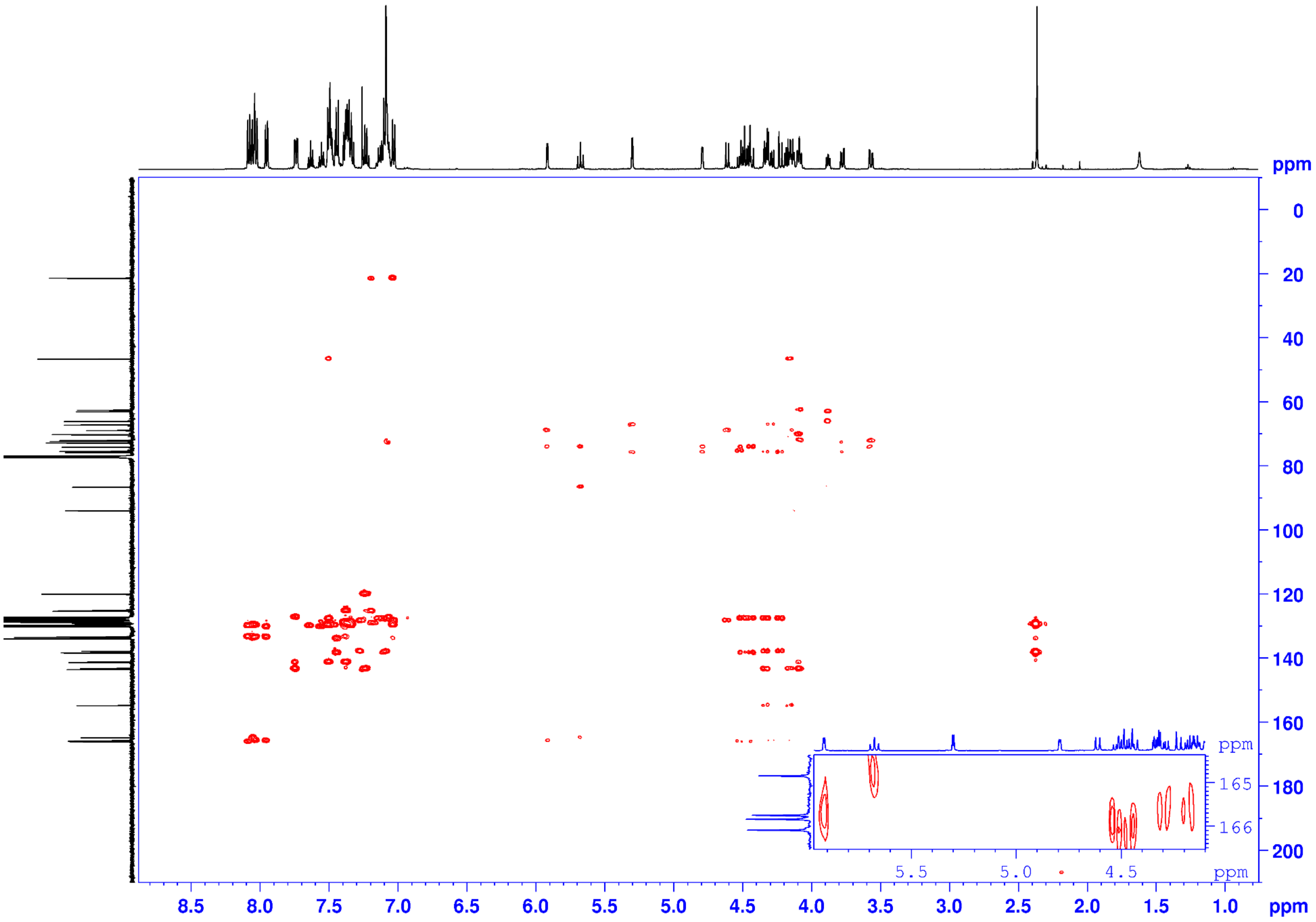

ppm
0
20
40
60
80
100
120
140
160
180
200
8.5
8.0
7.5
7.0
6.5
6.0
5.5
5.0
4.5
4.0
3.5
3.0
2.5
2.0
1.5
1.0
ppm
165
166
5.5
5.0
4.5

$^{13}C\{^{1}H\}$ NMR

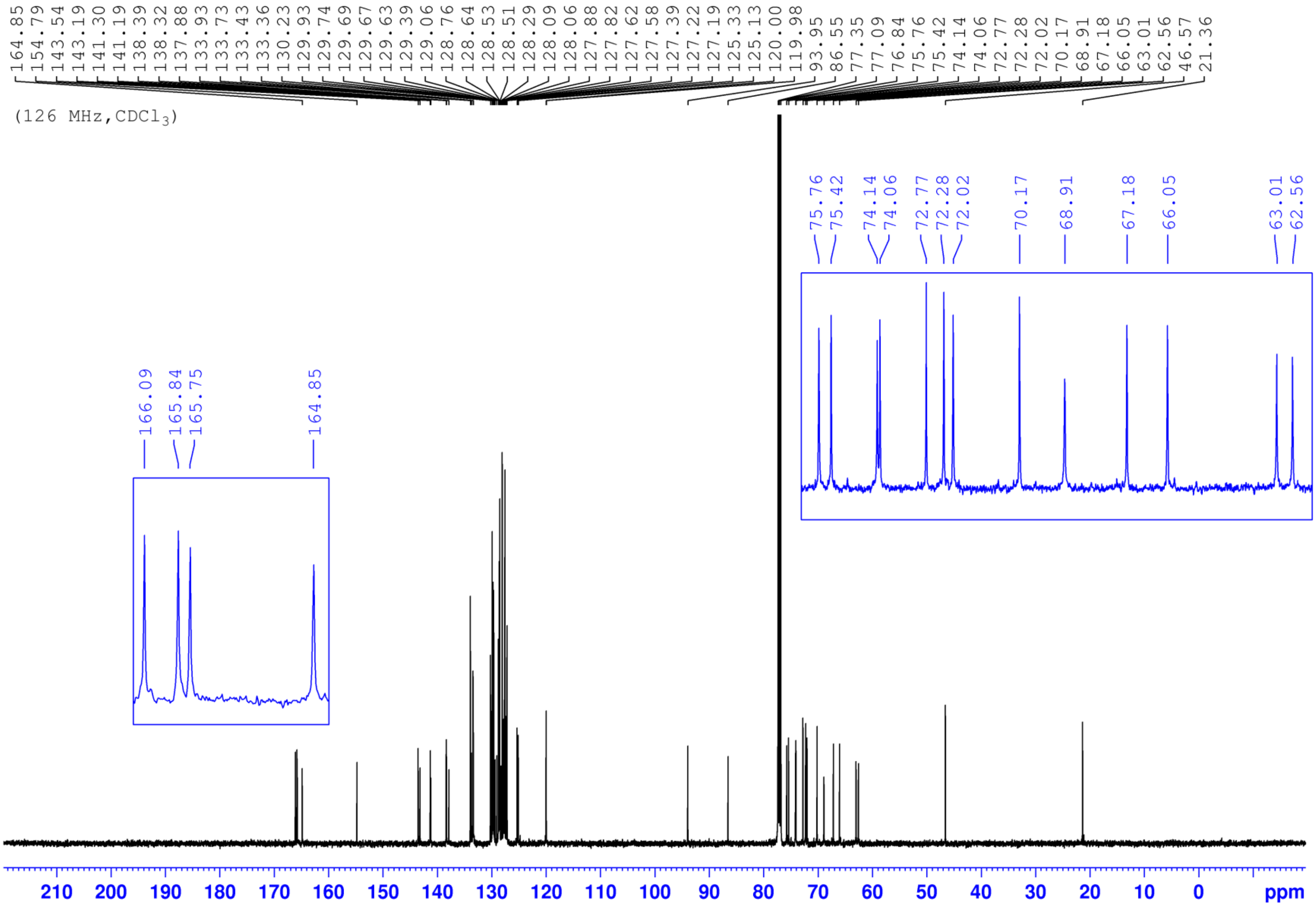
164.85
154.79
143.54
143.19
141.30
141.19
138.39
138.32
137.88
133.93
133.73
133.43
133.36
130.23
129.93
129.74
129.69
129.67
129.63
129.39
129.06
128.76
128.64
128.53
128.51
128.29
128.09
128.06
127.88
127.82
127.62
127.58
127.39
127.22
127.19
125.33
125.13
120.00
119.98
93.95
86.55
77.35
77.09
76.84
75.76
75.42
74.14
74.06
72.77
72.28
72.02
70.17
68.91
67.18
66.05
63.01
62.56
46.57
21.36
(126 MHz, CDCl3)
166.09
165.84
165.75
164.85
75.76
75.42
74.14
74.06
72.77
72.28
72.02
70.17
68.91
67.18
66.05
63.01
62.56
210 200 190 180 170 160 150 140 130 120 110 100 90 80 70 60 50 40 30 20 10 0 ppm

Compound **3**

^{1}H-NMR

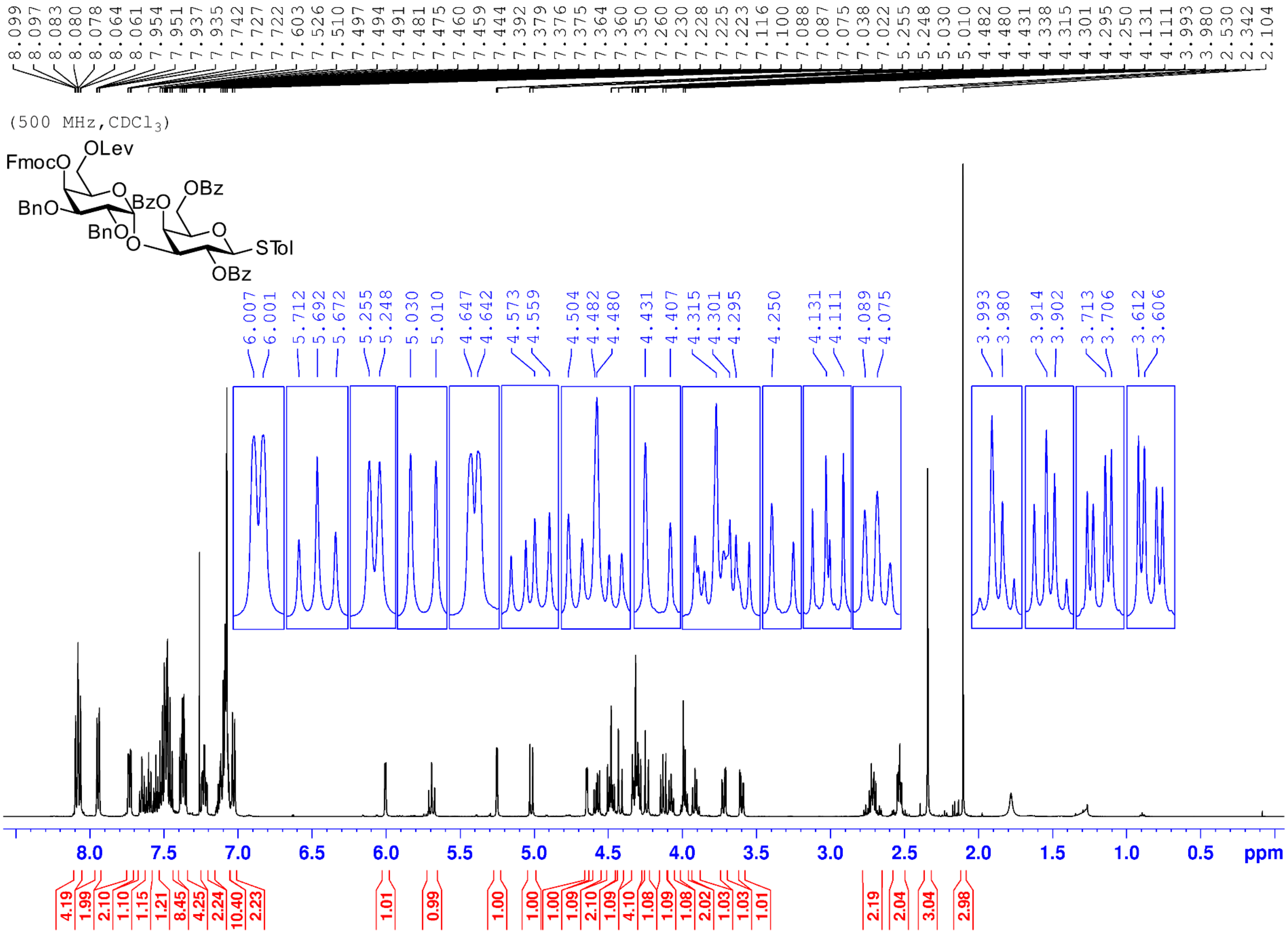

^{1}H-^{1}H COSY

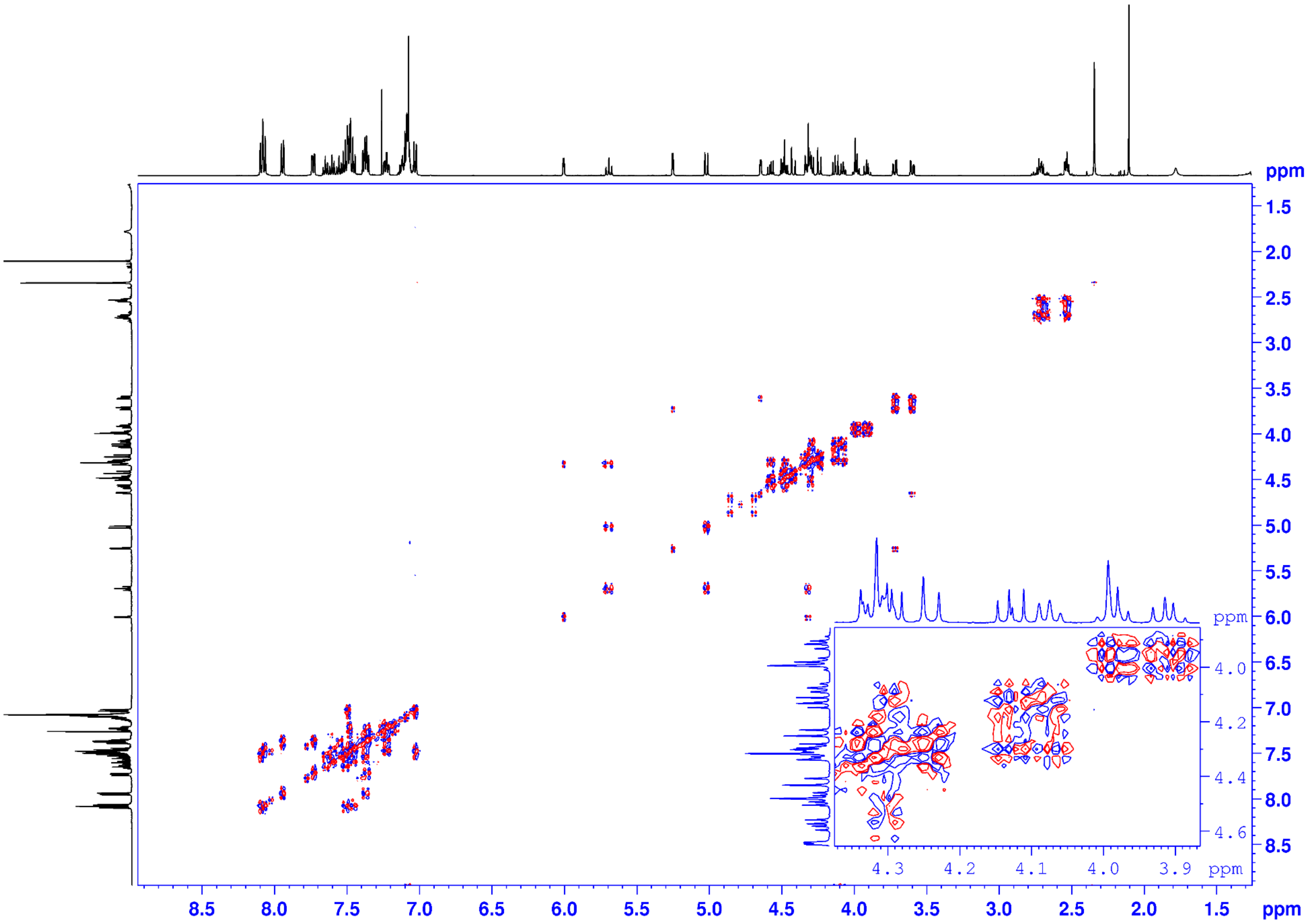

ppm
1.5
2.0
2.5
3.0
3.5
4.0
4.5
5.0
5.5
6.0
6.5
7.0
7.5
8.0
8.5
8.5 8.0 7.5 7.0 6.5 6.0 5.5 5.0 4.5 4.0 3.5 3.0 2.5 2.0 1.5 ppm
4.3 4.2 4.1 4.0 3.9 ppm
4.0
4.2
4.4
4.6

^{1}H-^{13}C HSQC

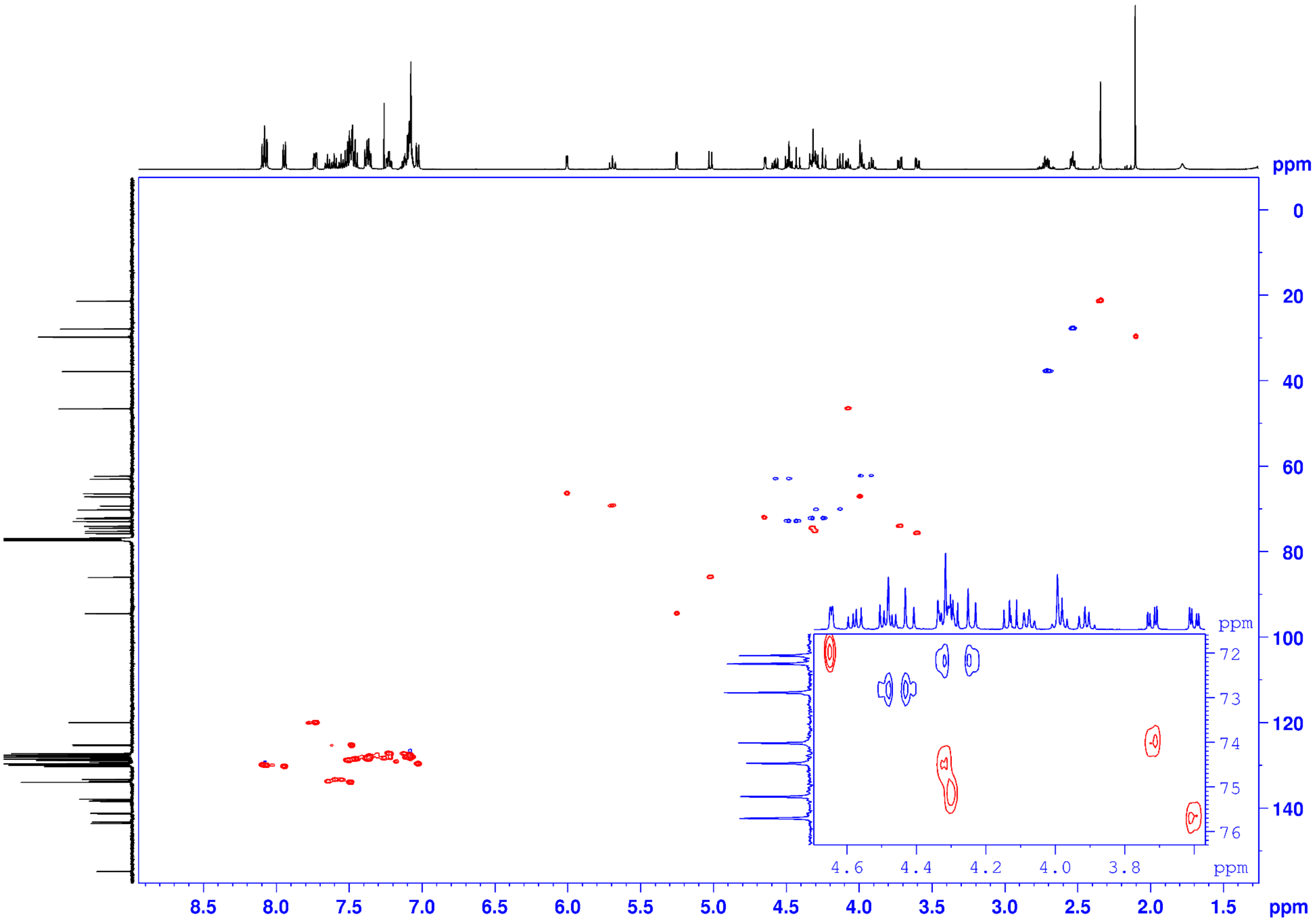

ppm
0
20
40
60
80
100
120
140
8.5
8.0
7.5
7.0
6.5
6.0
5.5
5.0
4.5
4.0
3.5
3.0
2.5
2.0
1.5
ppm
72
73
74
75
76
4.6
4.4
4.2
4.0
3.8

^{1}H-^{13}C non-decoupled HSQC

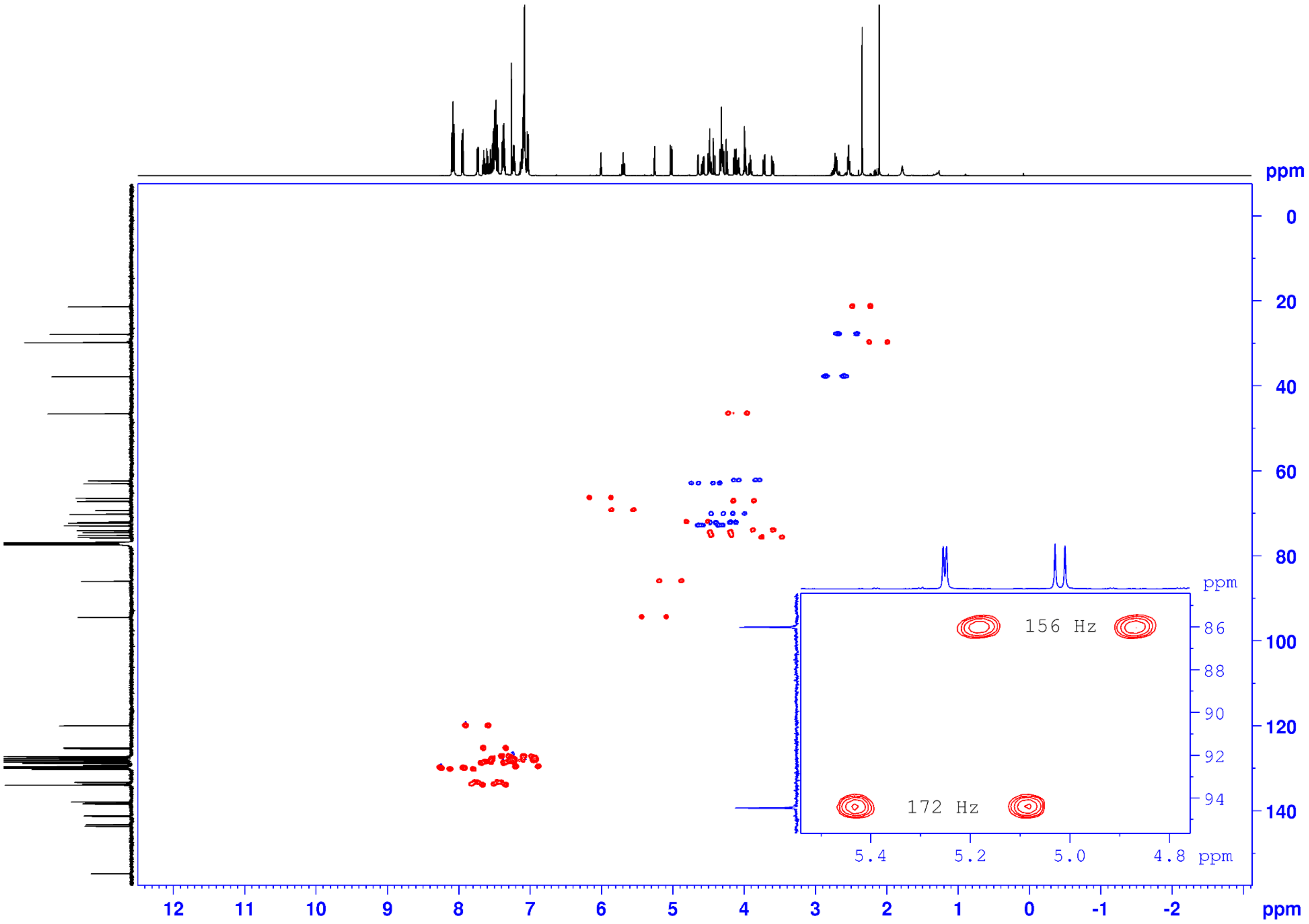

^{1}H-^{13}C HMBC

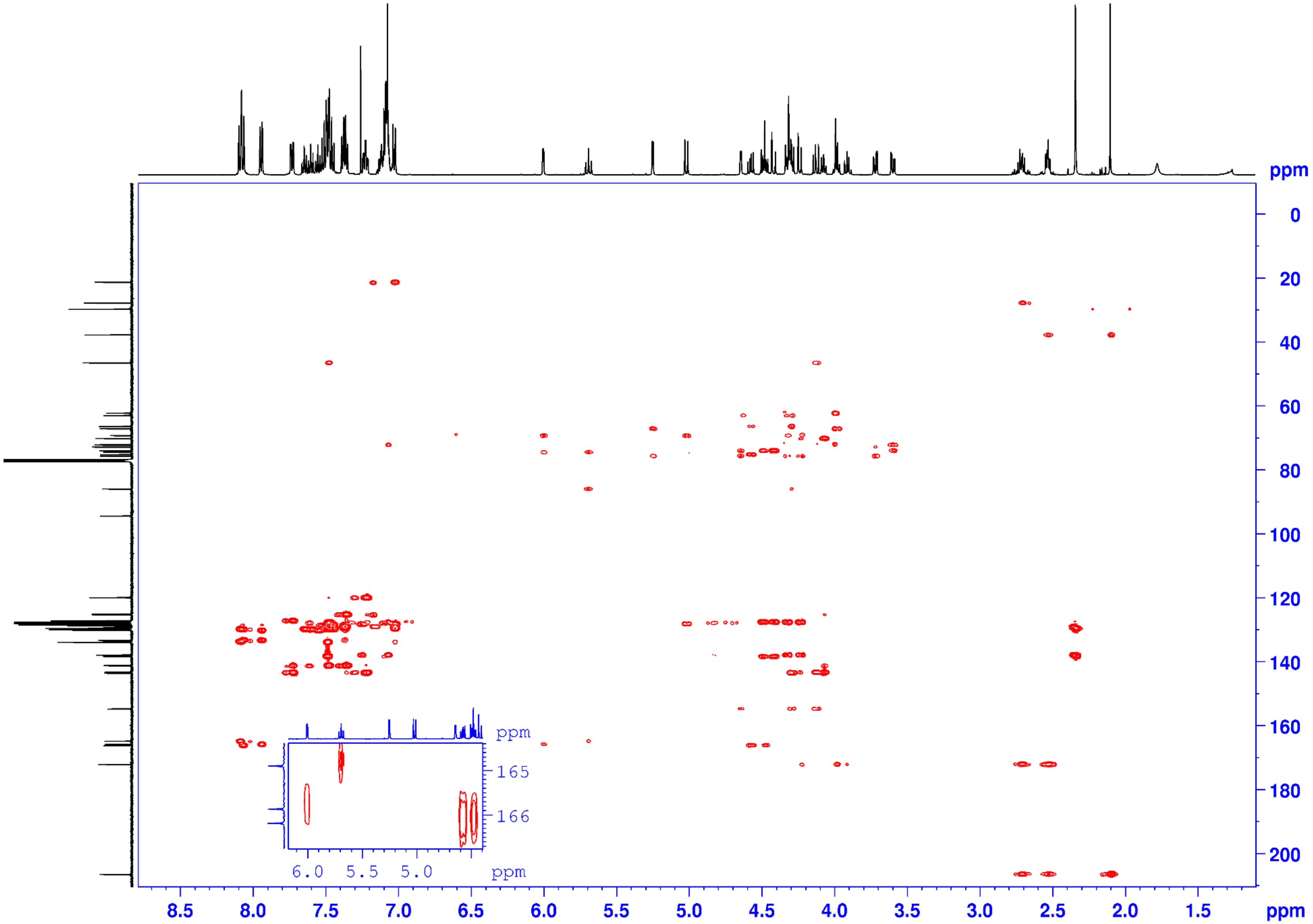

ppm
0
20
40
60
80
100
120
140
160
180
200
8.5
8.0
7.5
7.0
6.5
6.0
5.5
5.0
4.5
4.0
3.5
3.0
2.5
2.0
1.5
ppm
165
166
6.0
5.5
5.0

$^{13}C\{^{1}H\}$ NMR

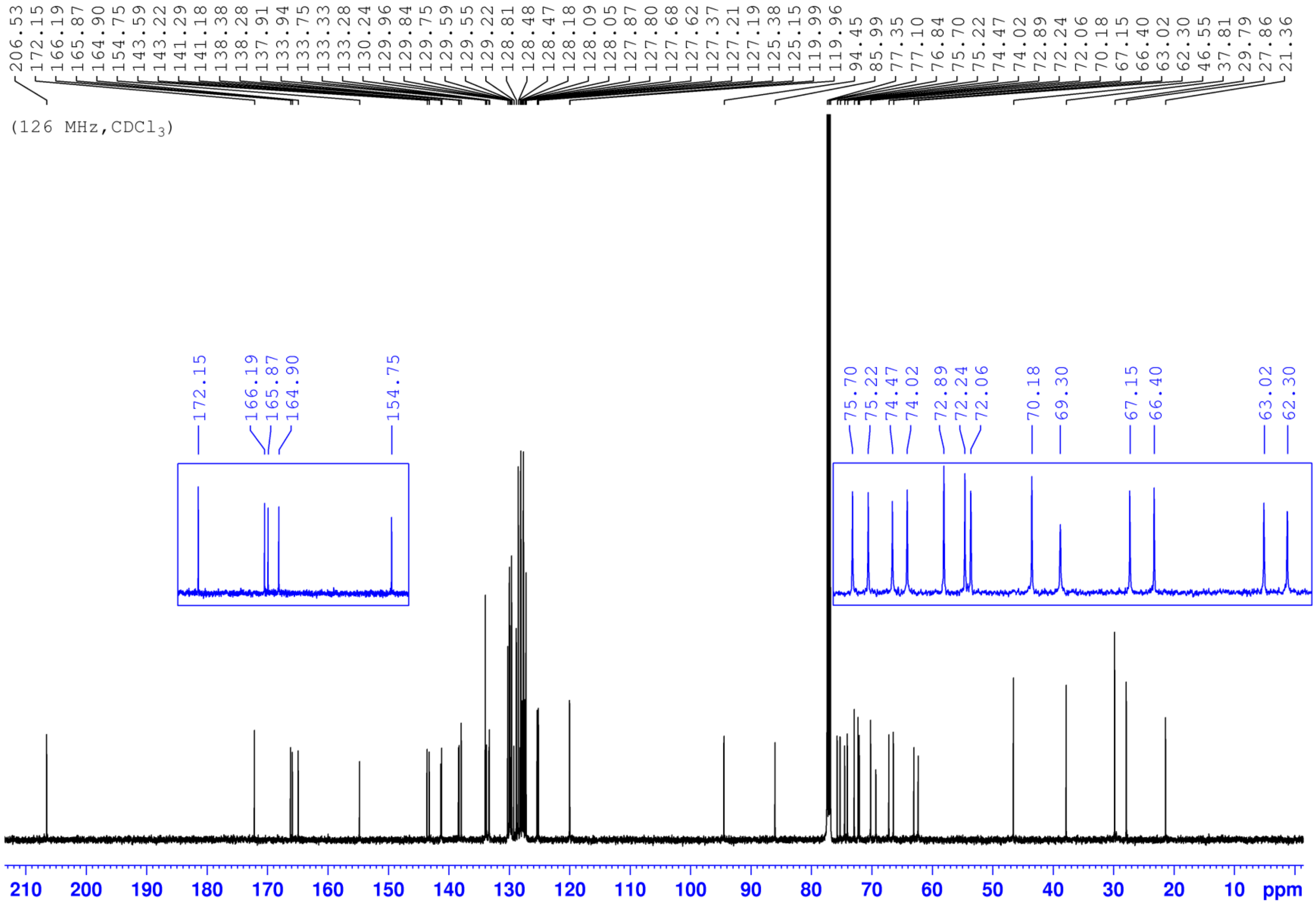
(126 MHz, CDCl3)
206.53
172.15
166.19
165.87
164.90
154.75
143.59
143.22
141.29
141.18
138.38
138.28
137.91
133.94
133.75
133.33
133.28
130.24
129.96
129.84
129.75
129.59
129.55
129.22
128.81
128.48
128.47
128.18
128.09
128.05
127.87
127.80
127.68
127.62
127.37
127.21
127.19
125.38
125.15
119.99
119.96
94.45
85.99
77.35
77.10
76.84
75.70
75.22
74.47
74.02
72.89
72.24
72.06
70.18
67.15
66.40
63.02
62.30
46.55
37.81
29.79
27.86
21.36
69.30
ppm

Compound **4**

^{1}H-NMR

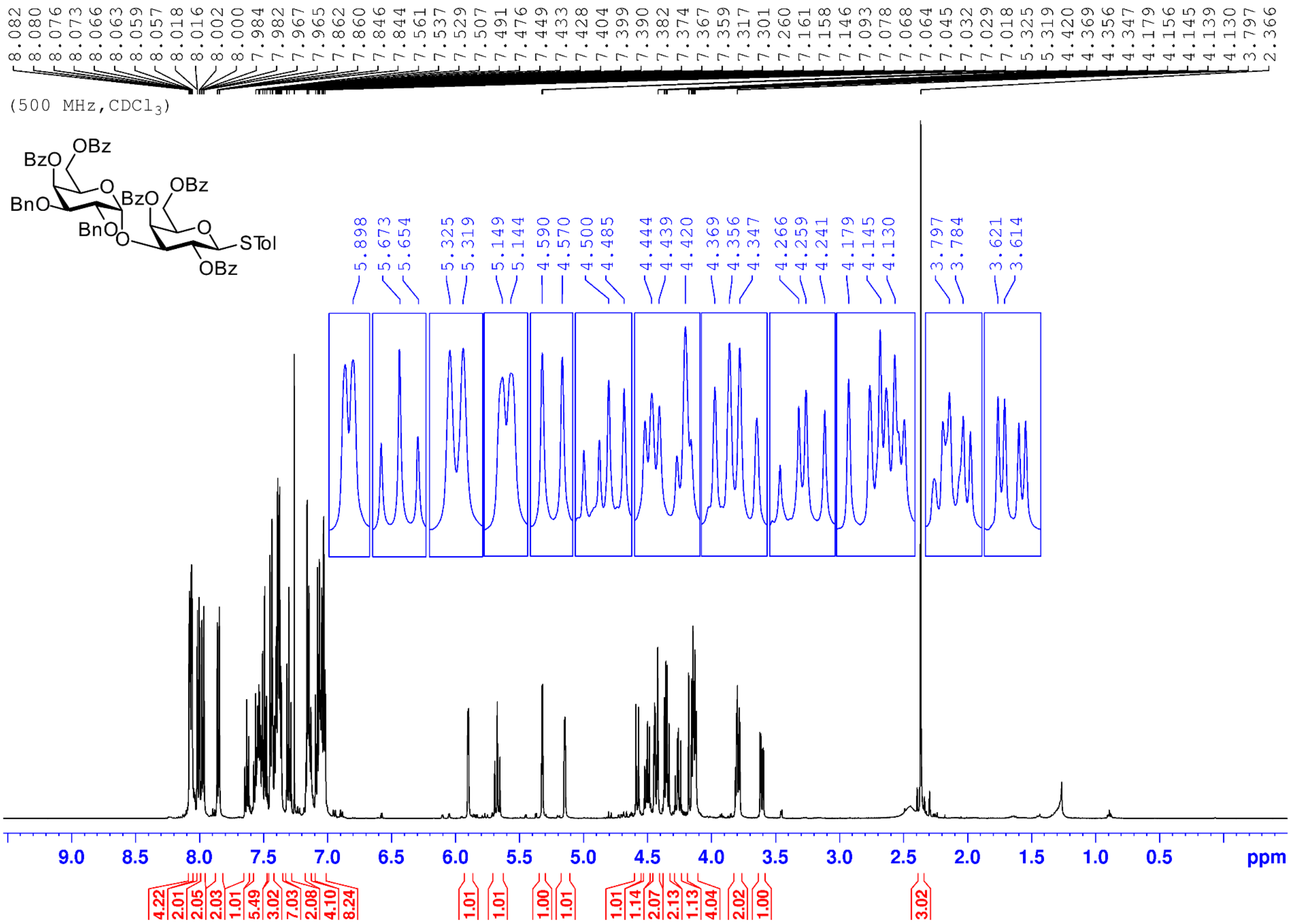

^{1}H-^{1}H COSY

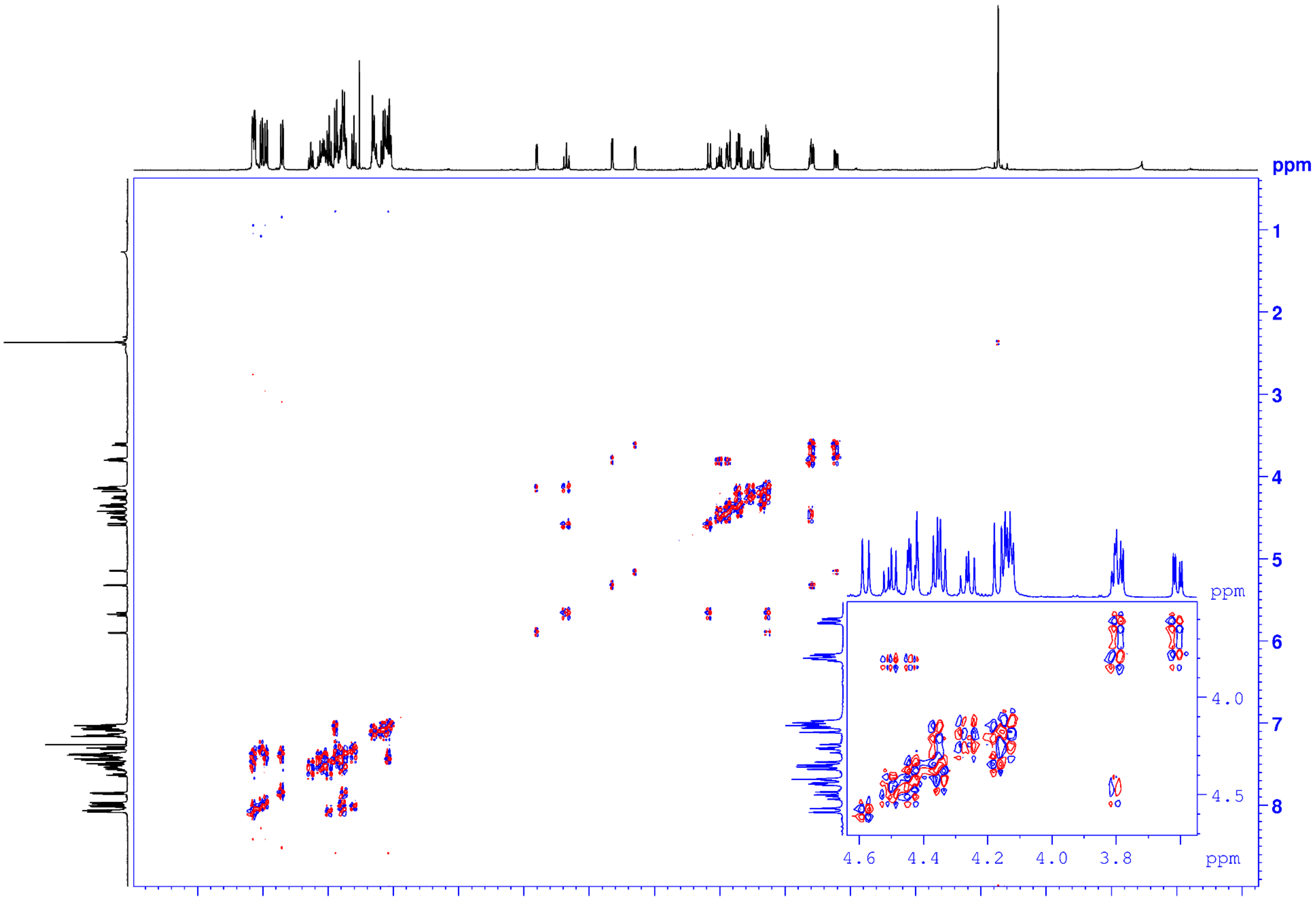

ppm
1
2
3
4
5
6
7
8
8.5
8.0
7.5
7.0
6.5
6.0
5.5
5.0
4.5
4.0
3.5
3.0
2.5
2.0
1.5
1.0
0.5
ppm
4.6
4.4
4.2
4.0
3.8
ppm
4.0
4.5

^{1}H-^{13}C HSQC

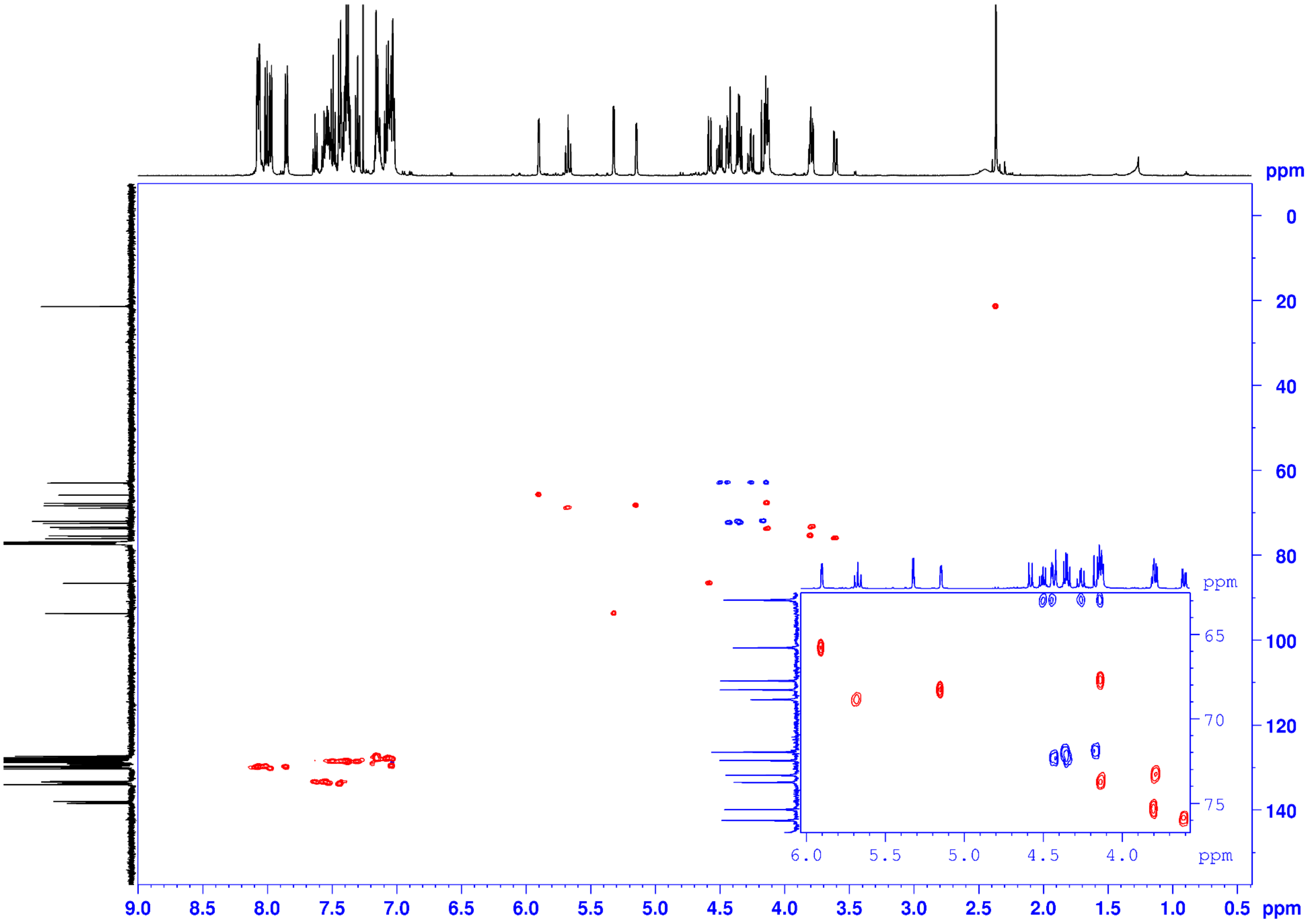

ppm
0
20
40
60
80
100
120
140
9.0 8.5 8.0 7.5 7.0 6.5 6.0 5.5 5.0 4.5 4.0 3.5 3.0 2.5 2.0 1.5 1.0 0.5 ppm
65
70
75
ppm
6.0 5.5 5.0 4.5 4.0 ppm

^{1}H-^{13}C HMBC

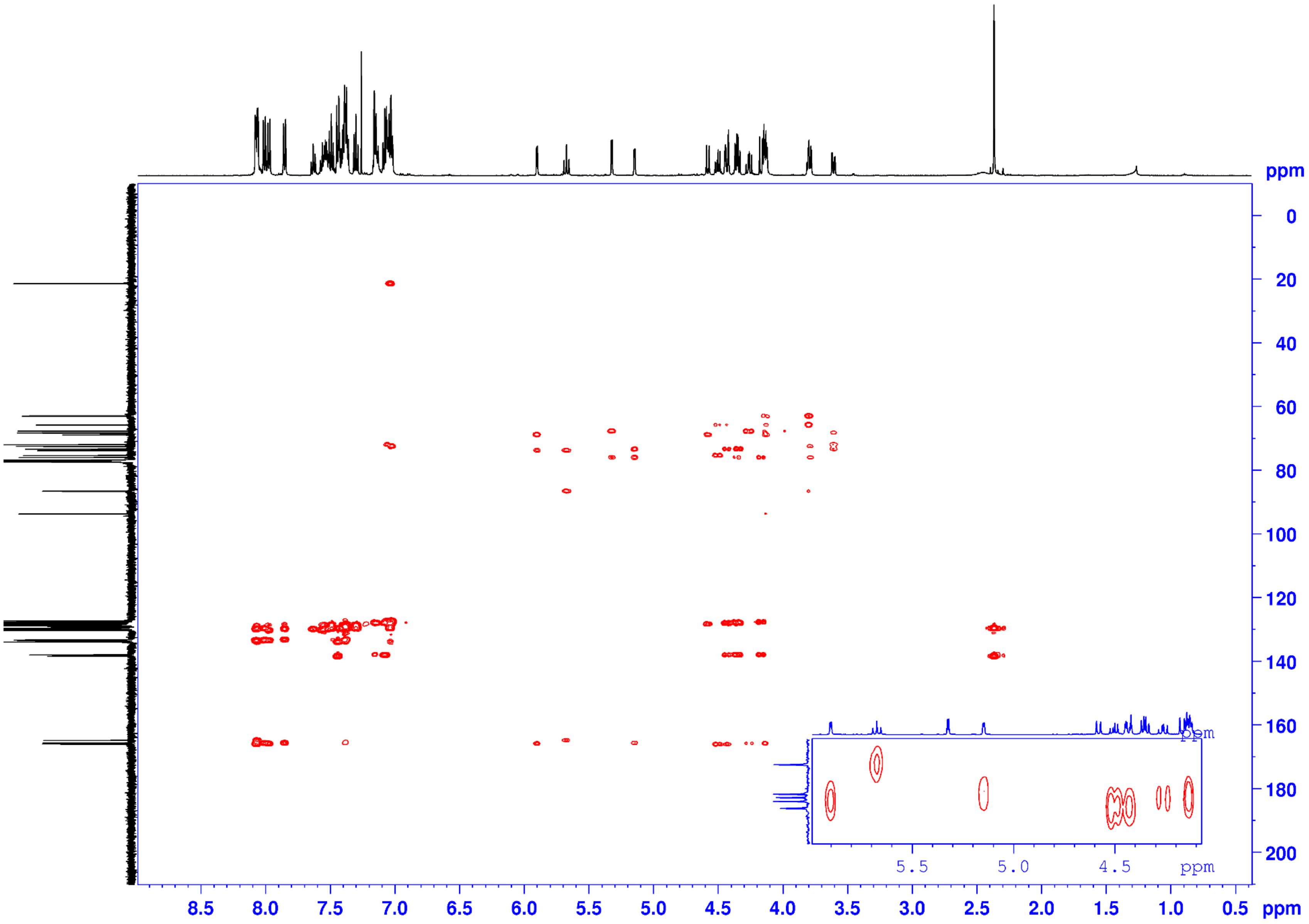
ppm
0
20
40
60
80
100
120
140
160
180
200
8.5
8.0
7.5
7.0
6.5
6.0
5.5
5.0
4.5
4.0
3.5
3.0
2.5
2.0
1.5
1.0
0.5
ppm
5.5
5.0
4.5
ppm

$^{13}C\{^1H\}$ NMR

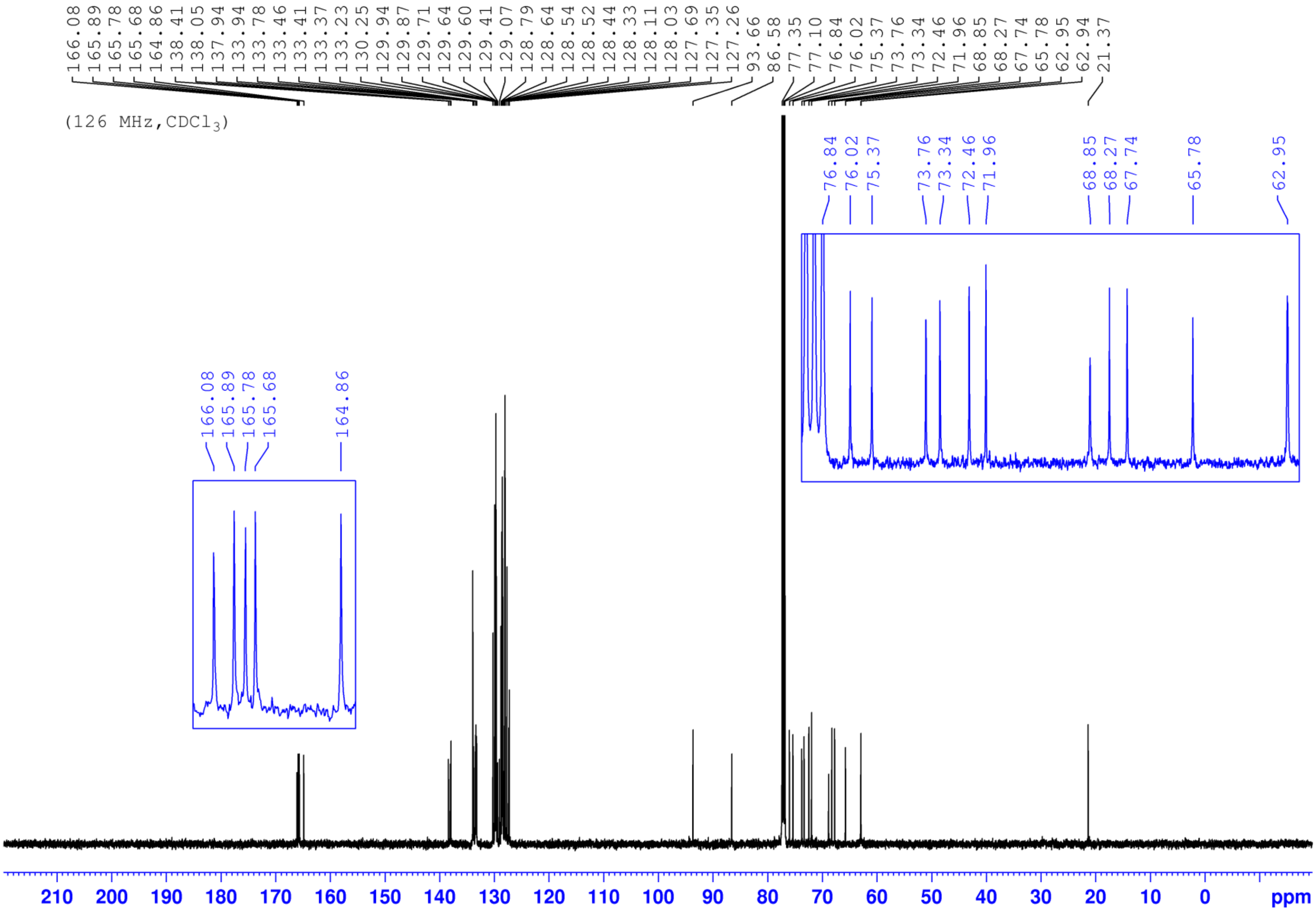
166.08
165.89
165.78
165.68
164.86
138.41
138.05
137.94
133.94
133.78
133.46
133.41
133.37
133.23
130.25
129.94
129.87
129.71
129.64
129.60
129.41
129.07
128.79
128.64
128.54
128.52
128.44
128.33
128.11
128.03
127.69
127.35
127.26
93.66
86.58
77.35
77.10
76.84
76.02
75.37
73.76
73.34
72.46
71.96
68.85
68.27
67.74
65.78
62.95
62.94
21.37
(126 MHz, CDCl3)
ppm

Compound **5**

^{1}H-NMR

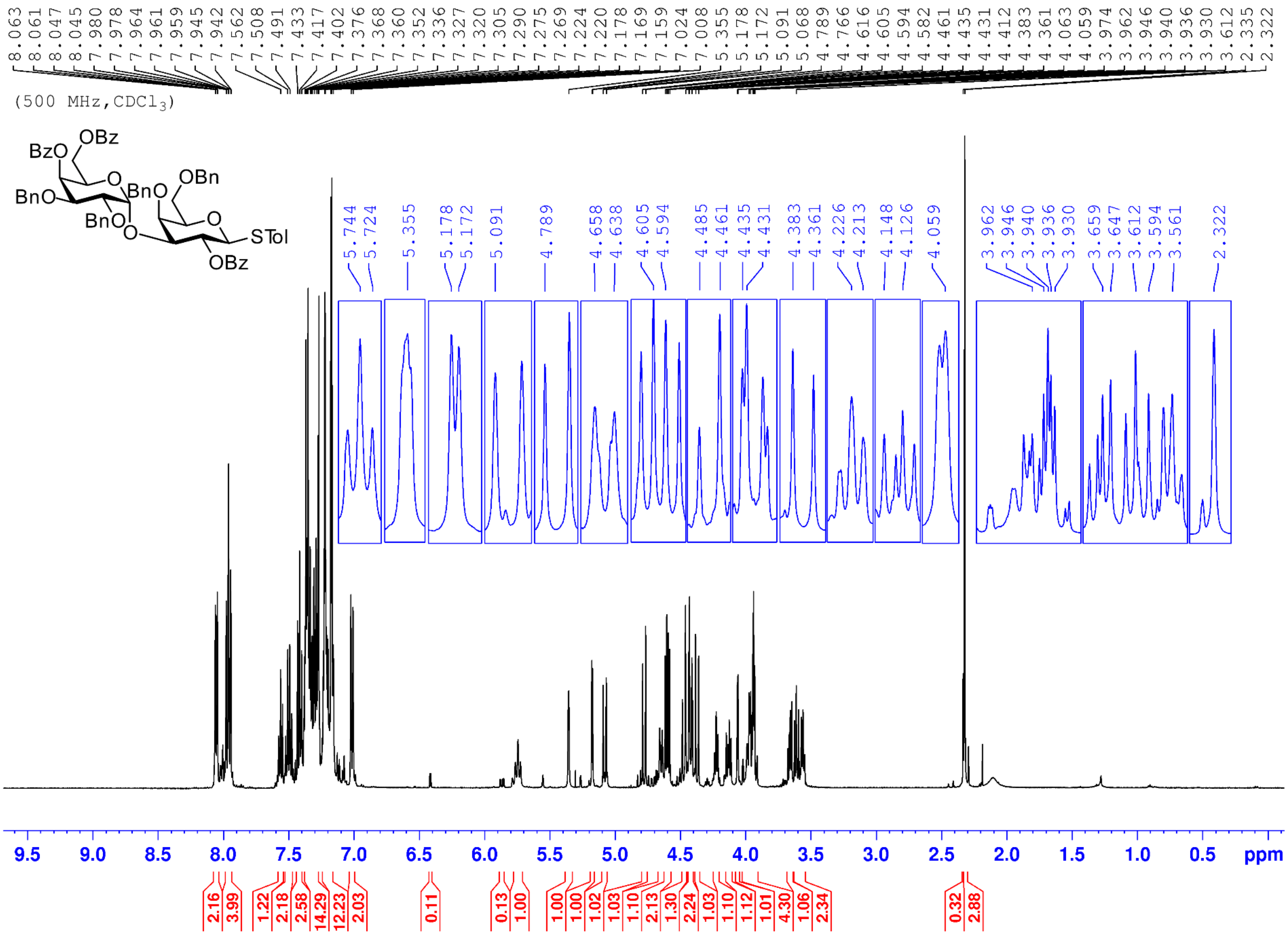

^{1}H-^{1}H COSY

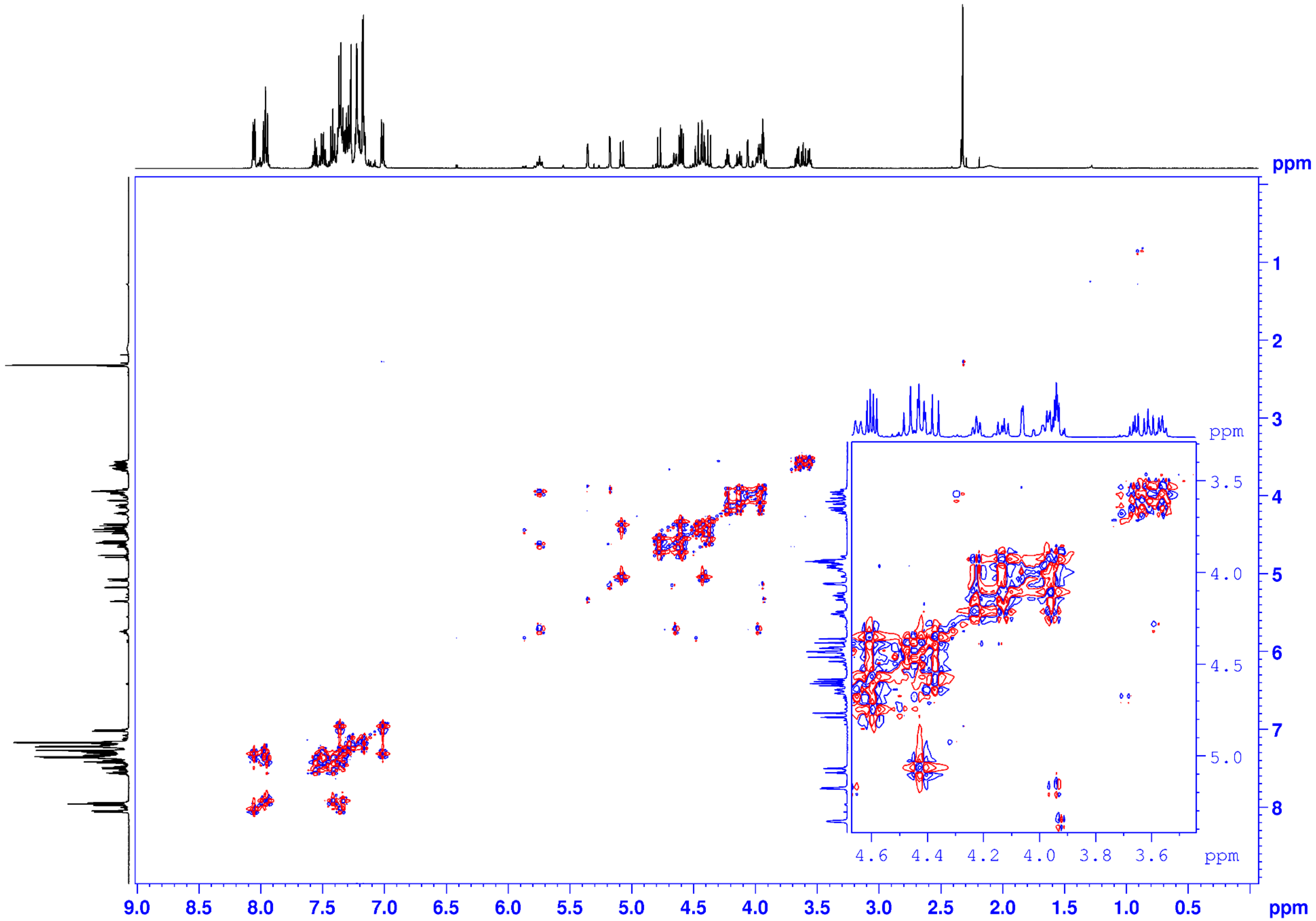

ppm
1
2
3
4
5
6
7
8
9.0
8.5
8.0
7.5
7.0
6.5
6.0
5.5
5.0
4.5
4.0
3.5
3.0
2.5
2.0
1.5
1.0
0.5
ppm
3.5
4.0
4.5
5.0
4.6
4.4
4.2
4.0
3.8
3.6
ppm

^{1}H-^{13}C HSQC

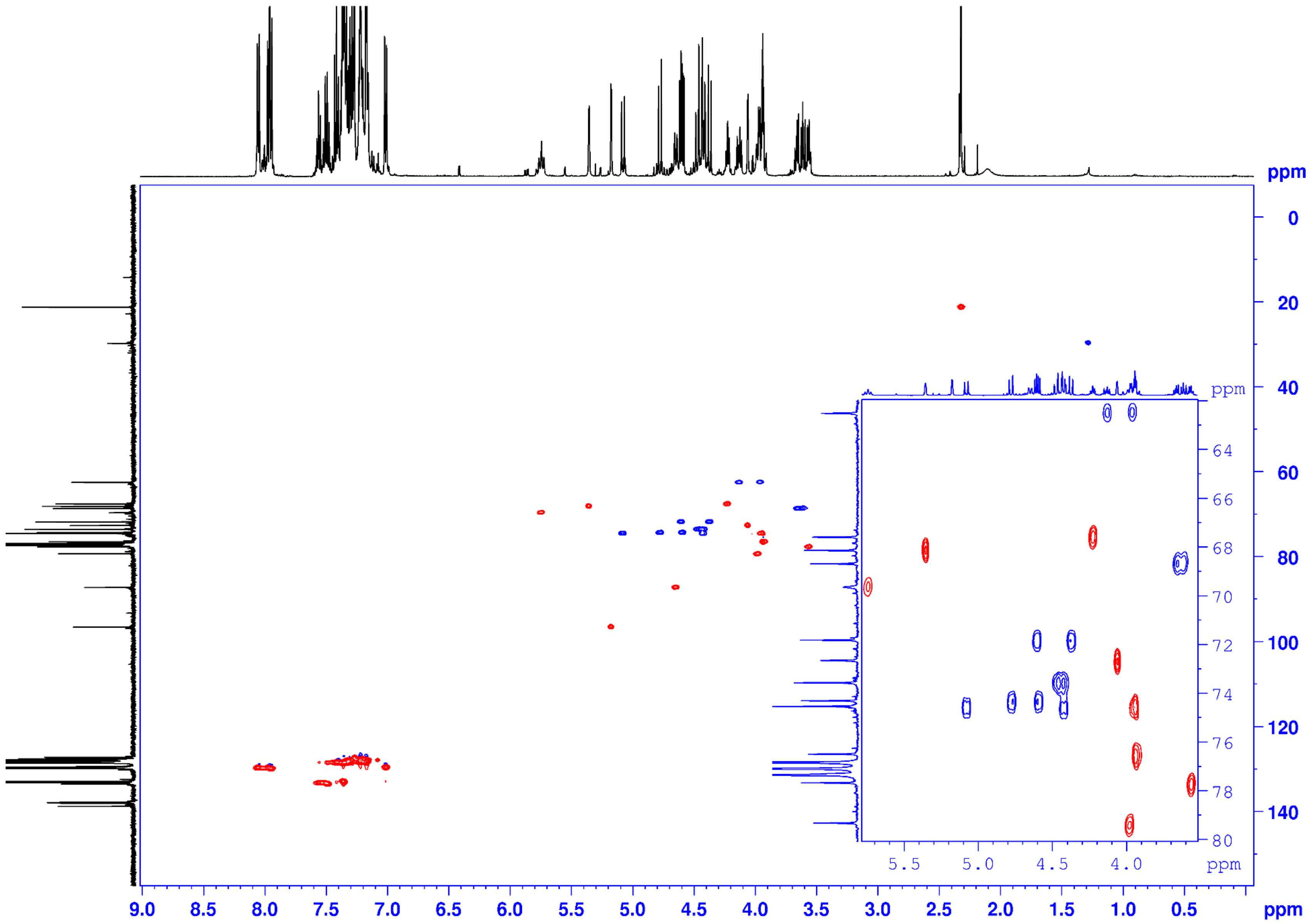

ppm
0
20
40
60
80
100
120
140
9.0
8.5
8.0
7.5
7.0
6.5
6.0
5.5
5.0
4.5
4.0
3.5
3.0
2.5
2.0
1.5
1.0
0.5
ppm
64
66
68
70
72
74
76
78
80
5.5
5.0
4.5
4.0

^{1}H-^{13}C non-decoupled HSQC

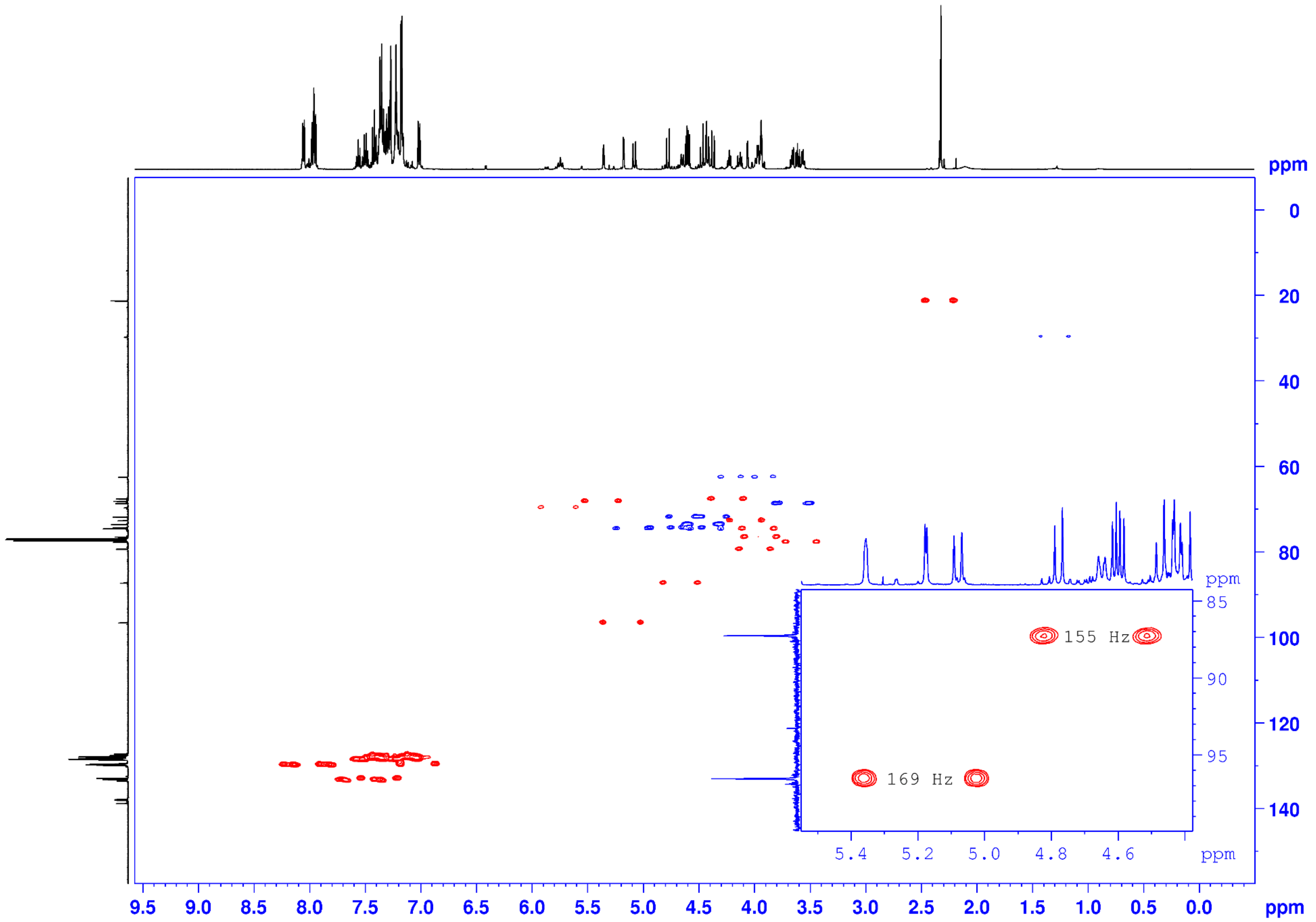

¹H-¹³C HMBC

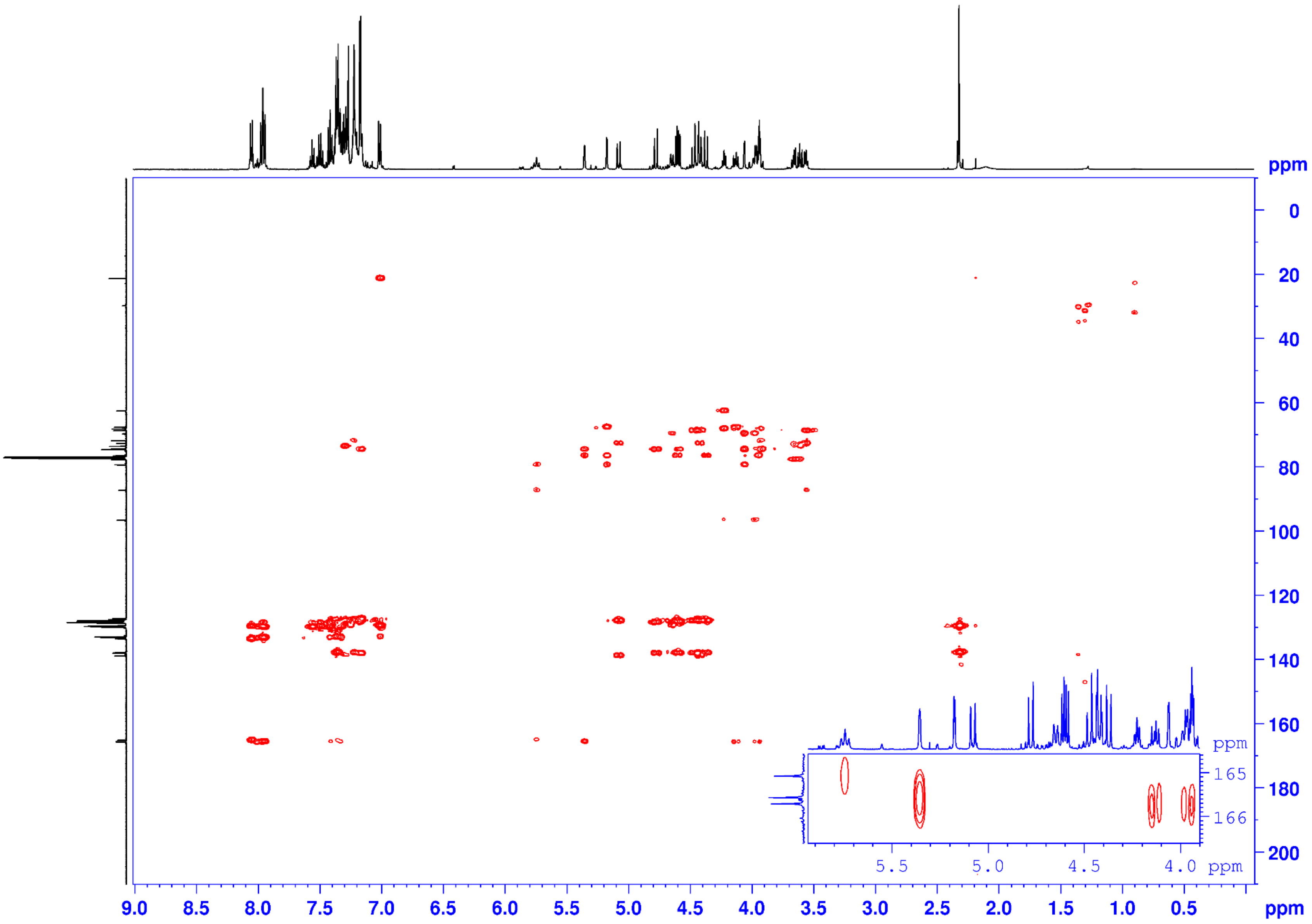
ppm
0
20
40
60
80
100
120
140
160
180
200
ppm
165
166
5.5
5.0
4.5
4.0 ppm
9.0
8.5
8.0
7.5
7.0
6.5
6.0
5.5
5.0
4.5
4.0
3.5
3.0
2.5
2.0
1.5
1.0
0.5
ppm

$^{13}C\{^{1}H\}$ NMR

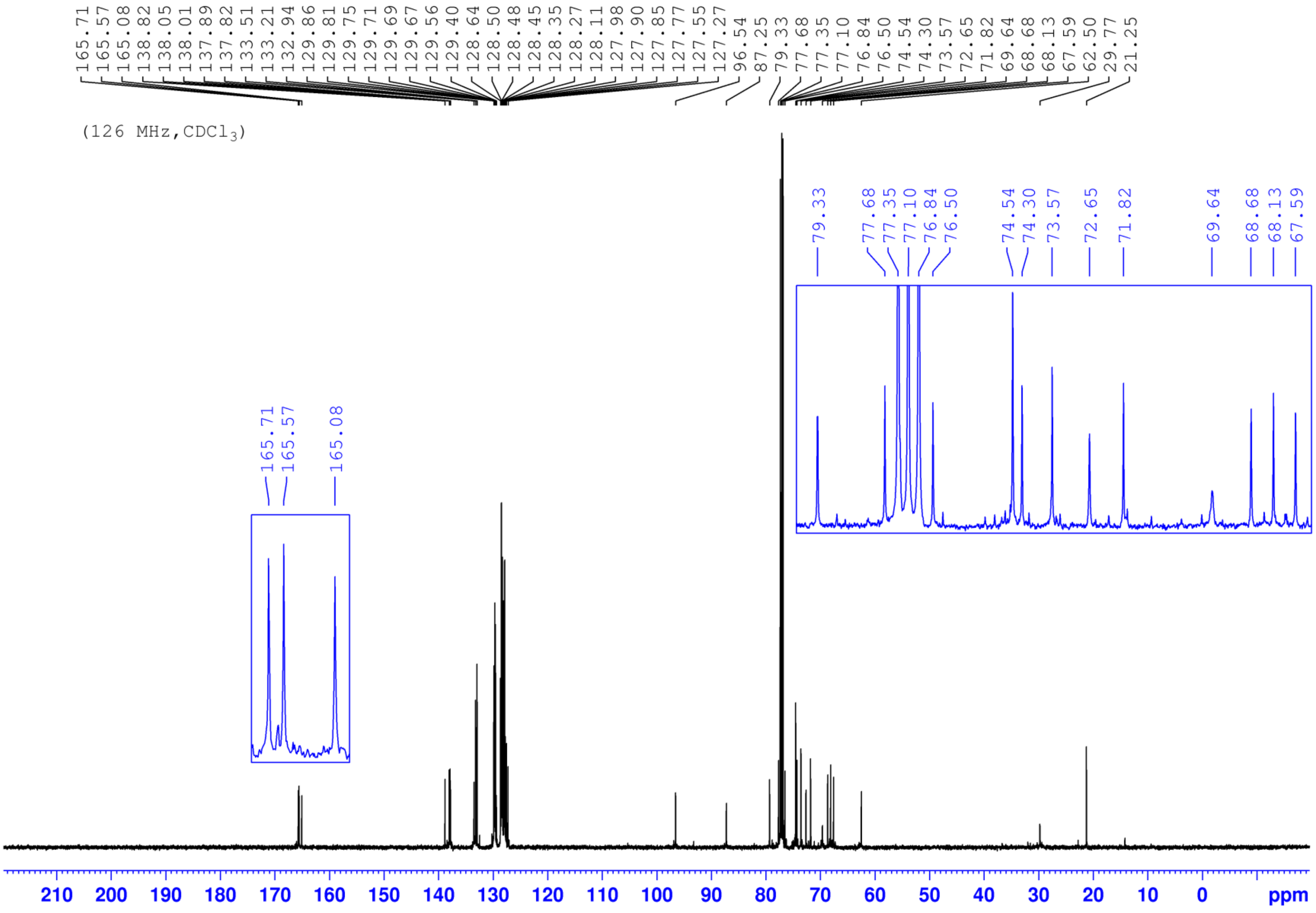
(126 MHz,CDCl3)
165.71
165.57
165.08
138.82
138.05
138.01
137.89
137.82
133.51
133.21
132.94
129.86
129.81
129.75
129.71
129.69
129.67
129.56
129.40
128.64
128.50
128.48
128.45
128.35
128.27
128.11
127.98
127.90
127.85
127.77
127.55
127.27
96.54
87.25
79.33
77.68
77.35
77.10
76.84
76.50
74.54
74.30
73.57
72.65
71.82
69.64
68.68
68.13
67.59
62.50
29.77
21.25
ppm

Compound **6**

^{1}H-NMR

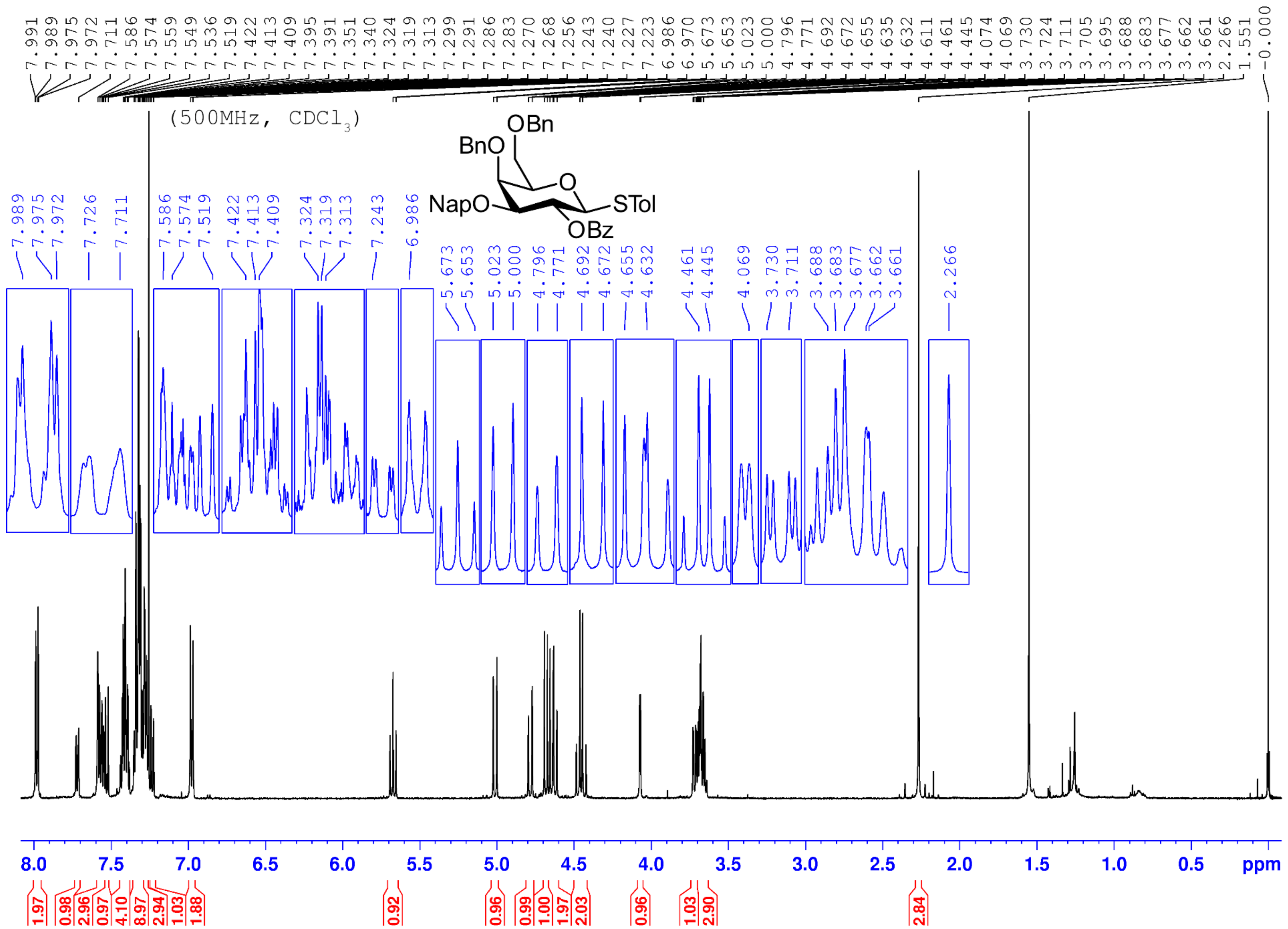

^1H-^1H COSY

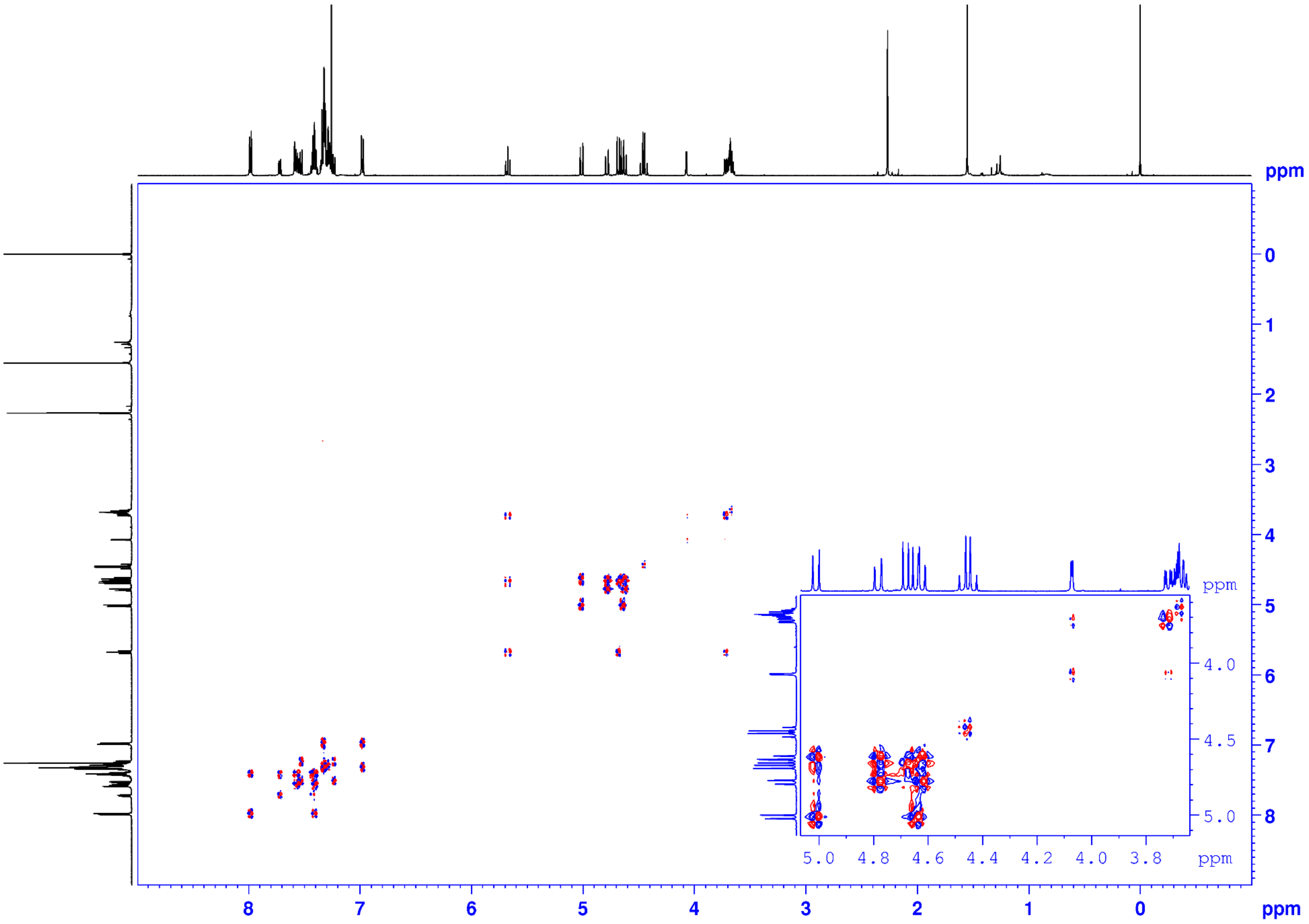
ppm
0
1
2
3
4
5
6
7
8
8
7
6
5
4
3
2
1
0
ppm
ppm
4.0
4.5
5.0
5.0
4.8
4.6
4.4
4.2
4.0
3.8
ppm

^{1}H-^{13}C HSQC

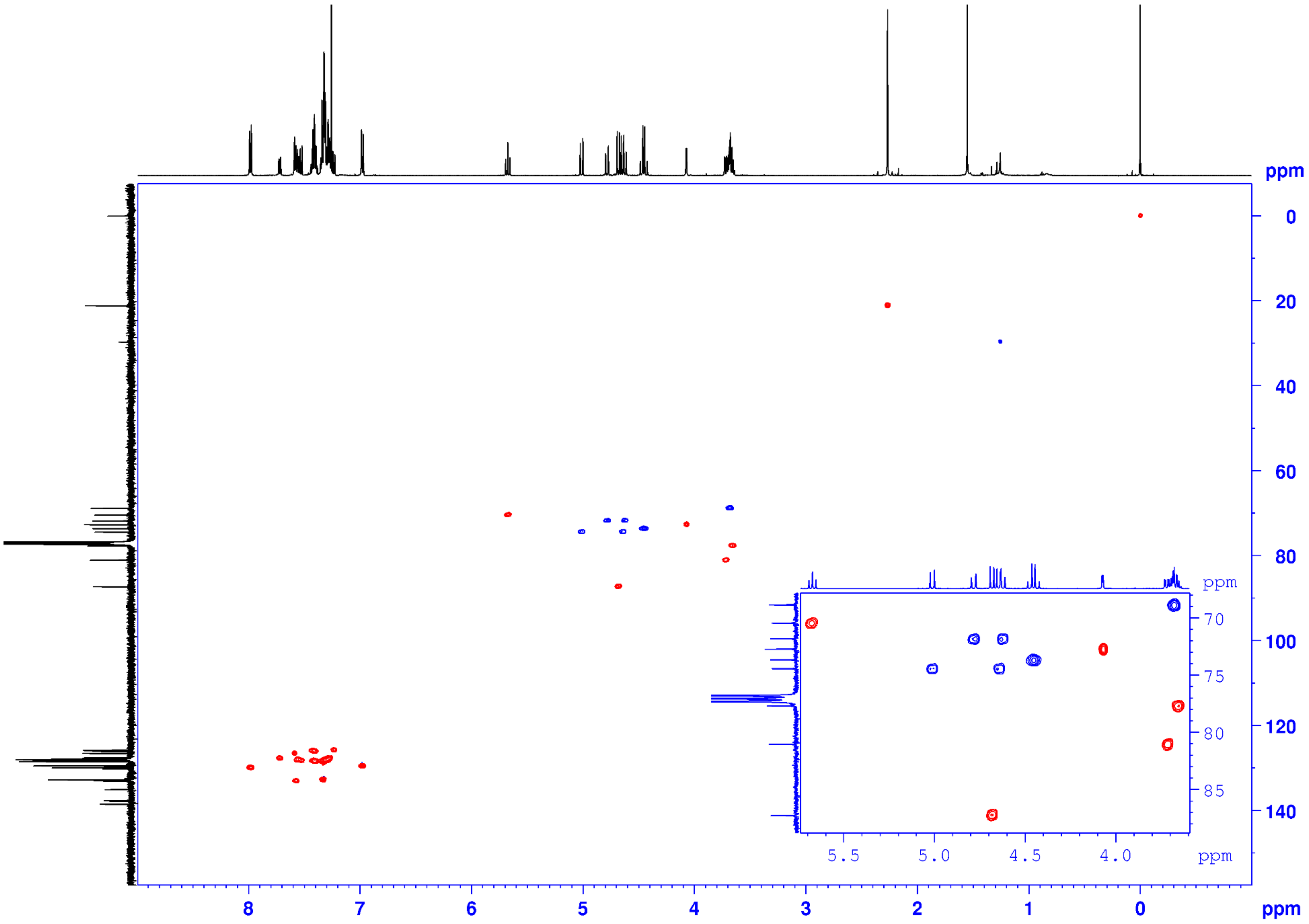
ppm
0
20
40
60
80
100
120
140
8
7
6
5
4
3
2
1
0
ppm
70
75
80
85
5.5
5.0
4.5
4.0
ppm

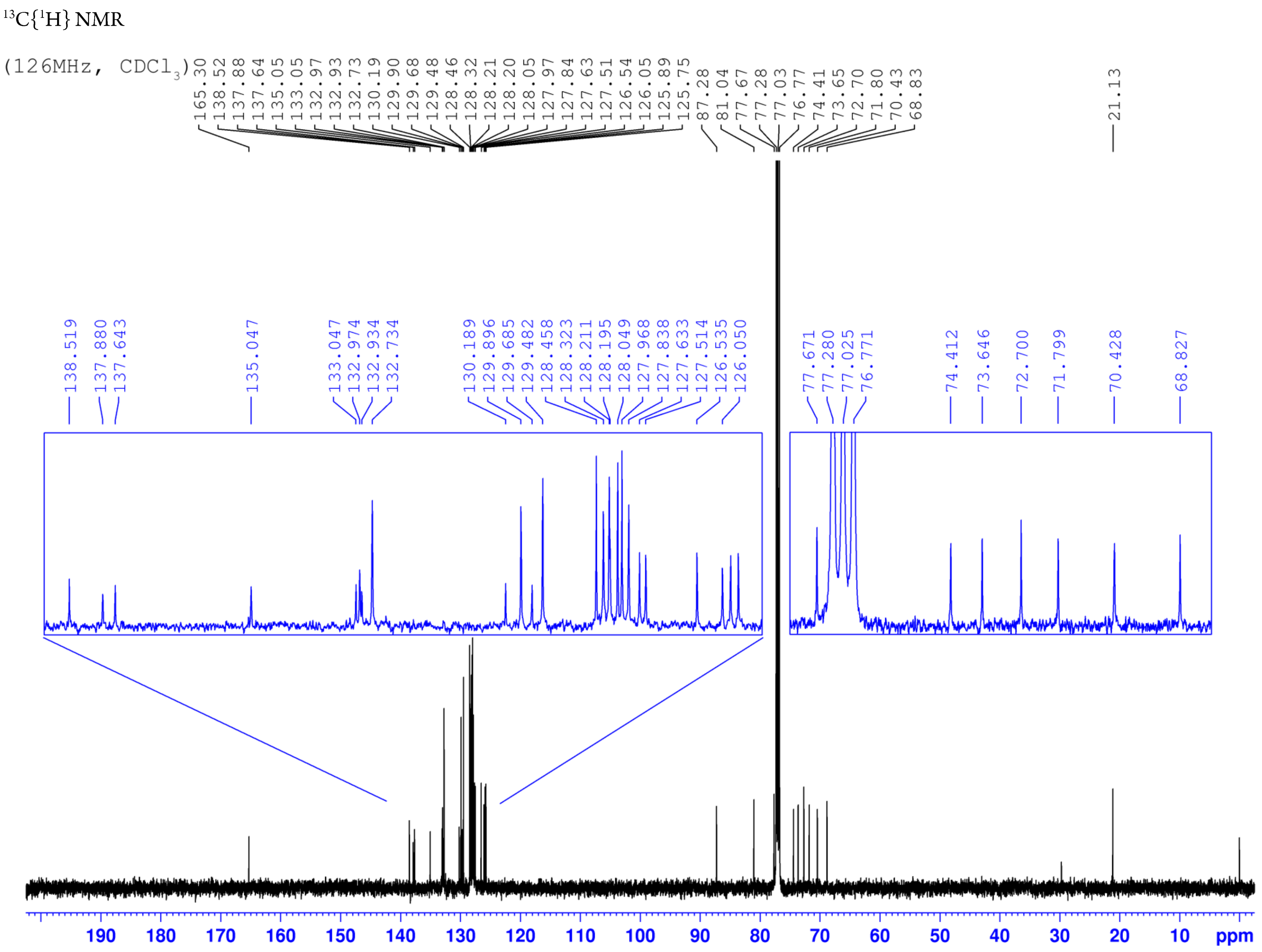
$^{13}C\{^{1}H\}$ NMR
(126MHz, $CDCl_3$)
165.30 138.52 137.88 137.64 135.05 133.05 132.97 132.93 132.73 130.19 129.90 129.68 129.48 128.46 128.32 128.21 128.20 128.05 127.97 127.84 127.63 127.51 126.54 126.05 125.89 125.75 87.28 81.04 77.67 77.28 77.03 76.77 74.41 73.65 72.70 71.80 70.43 68.83
21.13
138.519 137.880 137.643 135.047 133.047 132.974 132.934 132.734 130.189 129.896 129.685 129.482 128.458 128.323 128.211 128.195 128.049 127.968 127.838 127.633 127.514 126.535 126.050
77.671 77.280 77.025 76.771 74.412 73.646 72.700 71.799 70.428 68.827
190 180 170 160 150 140 130 120 110 100 90 80 70 60 50 40 30 20 10 ppm

Compound **7**

^{1}H-NMR (anomer A)

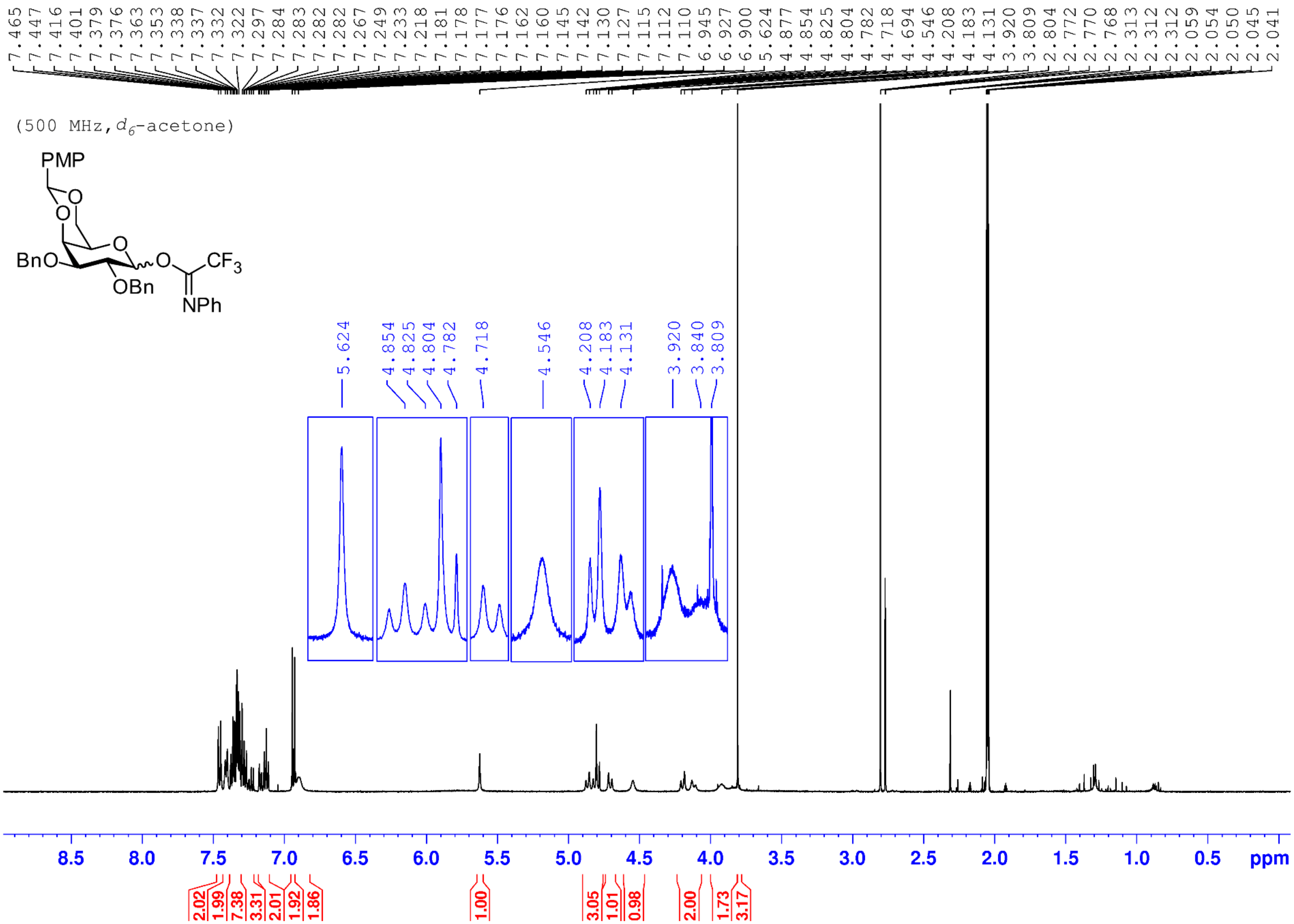

^{1}H-^{1}H COSY (anomer A)

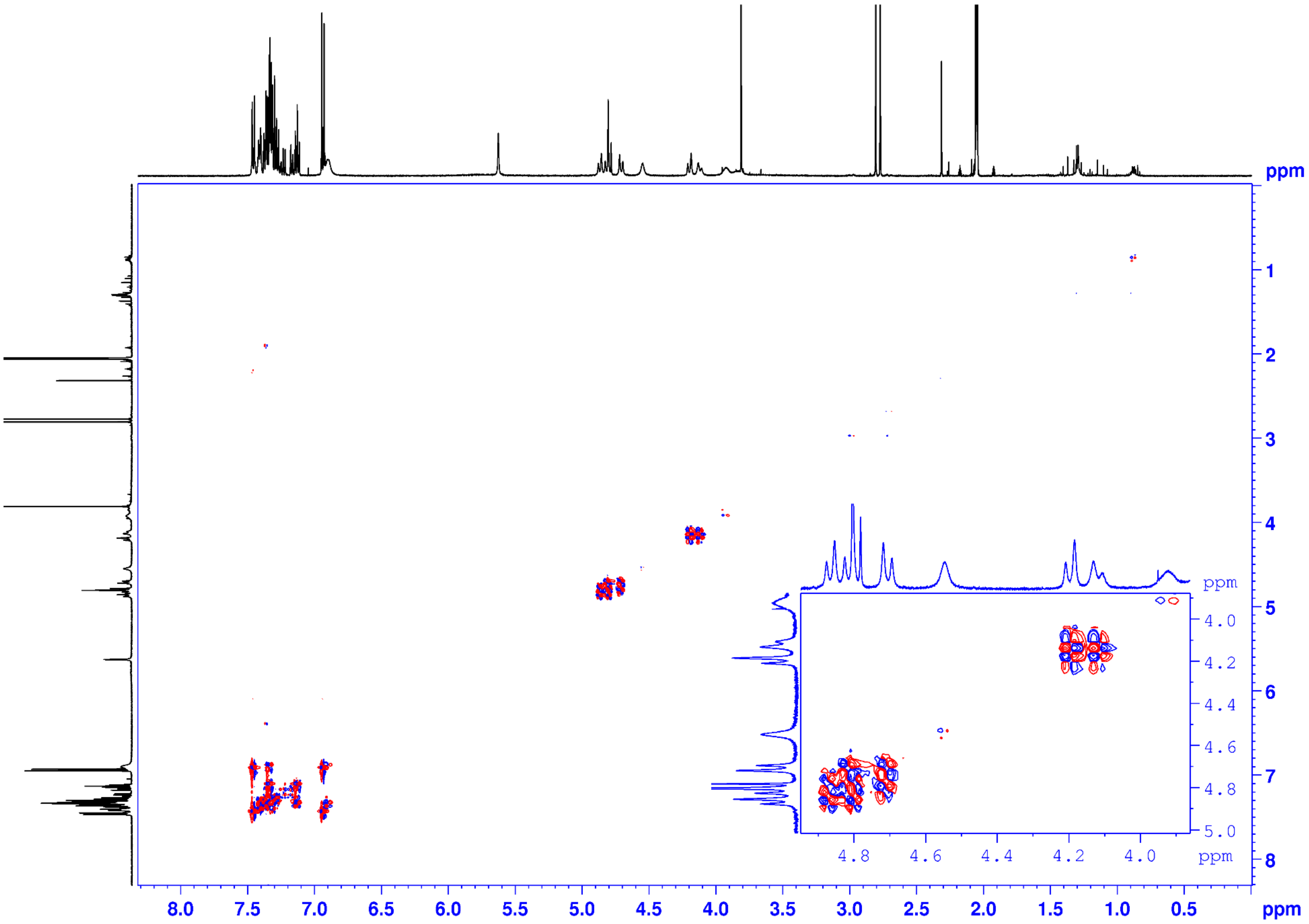

^{1}H-^{13}C HSQC (anomer A)

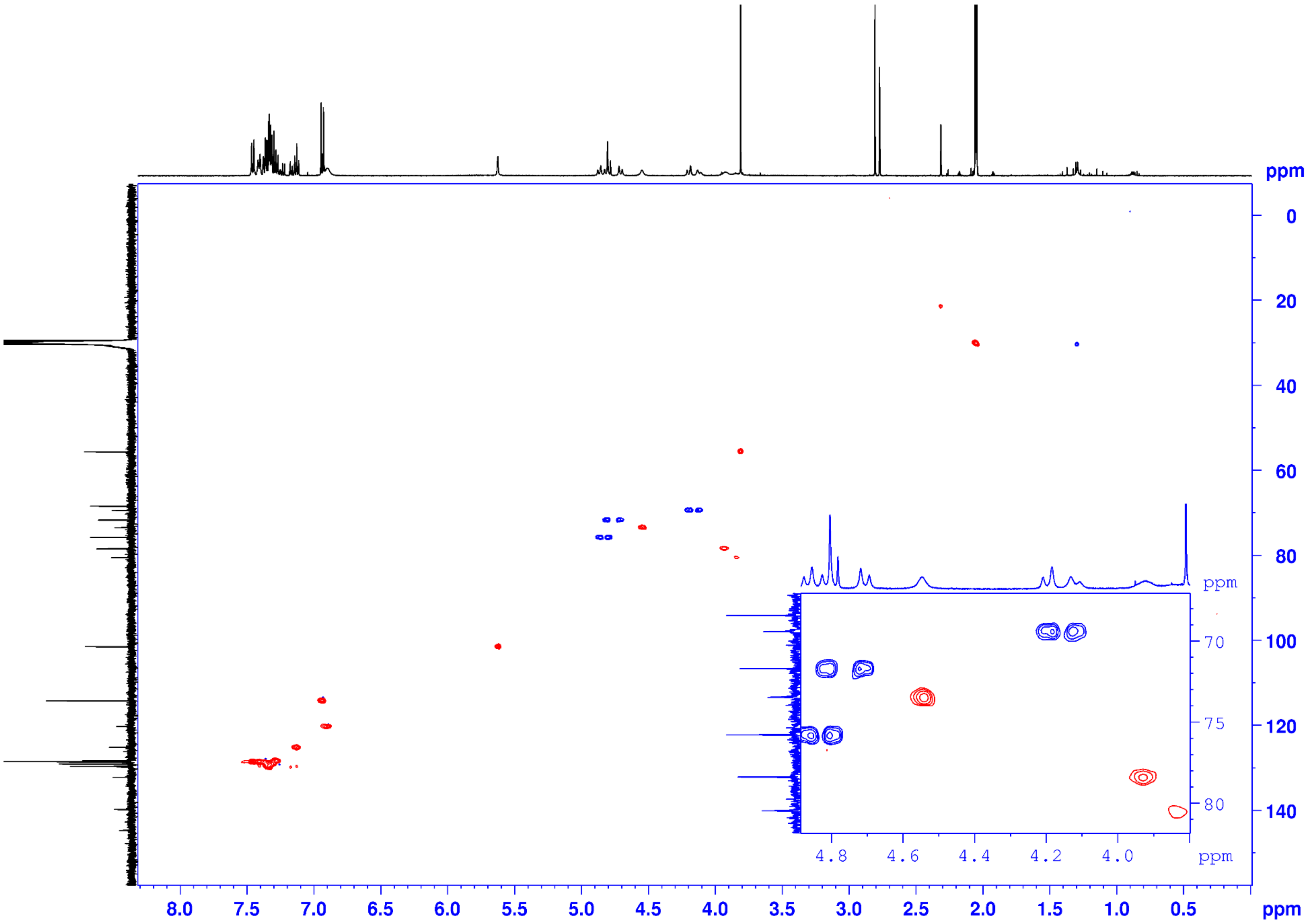

$^{13}C\{^1H\}$ NMR (anomer A)

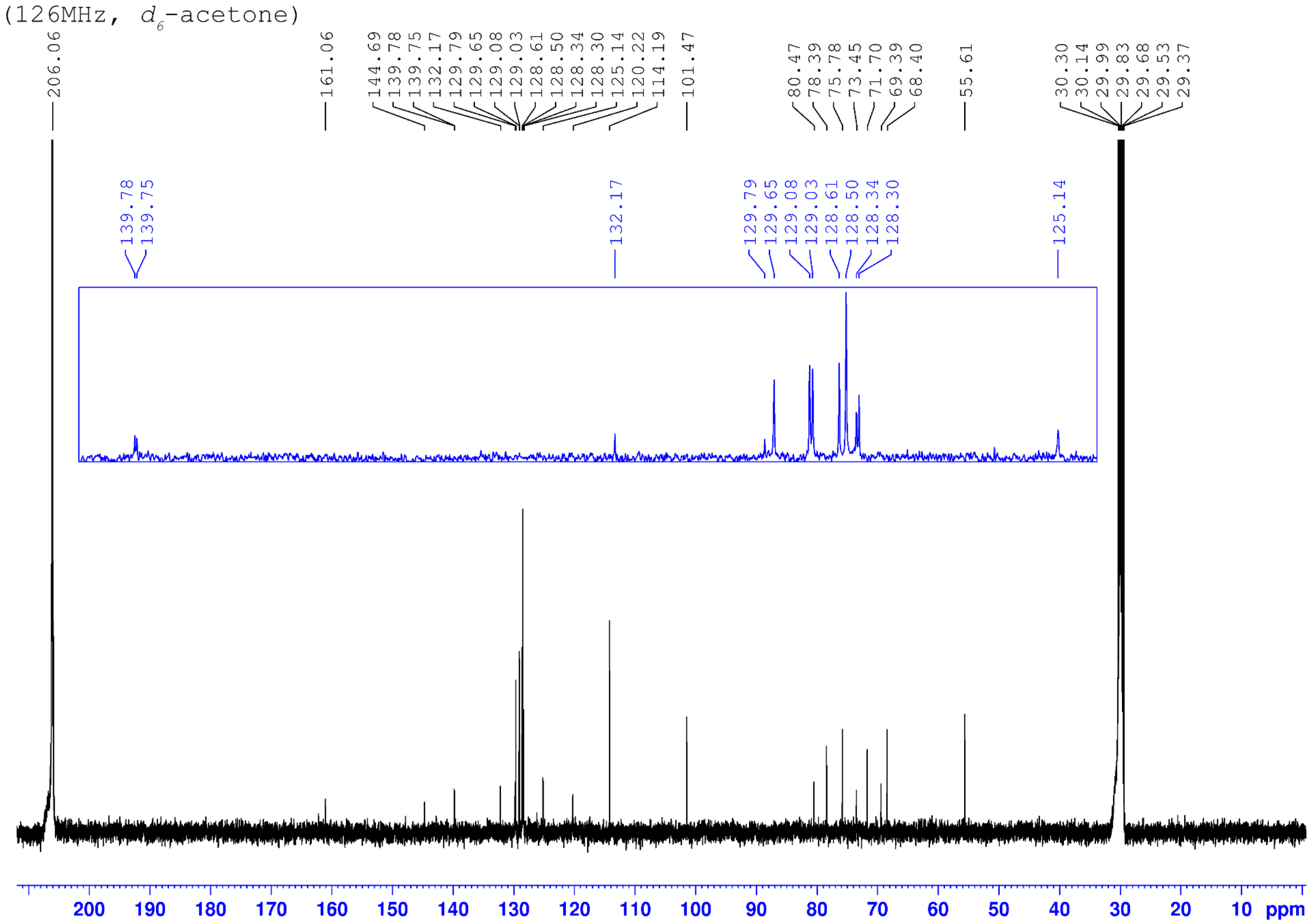

^{19}F-NMR (anomer A)

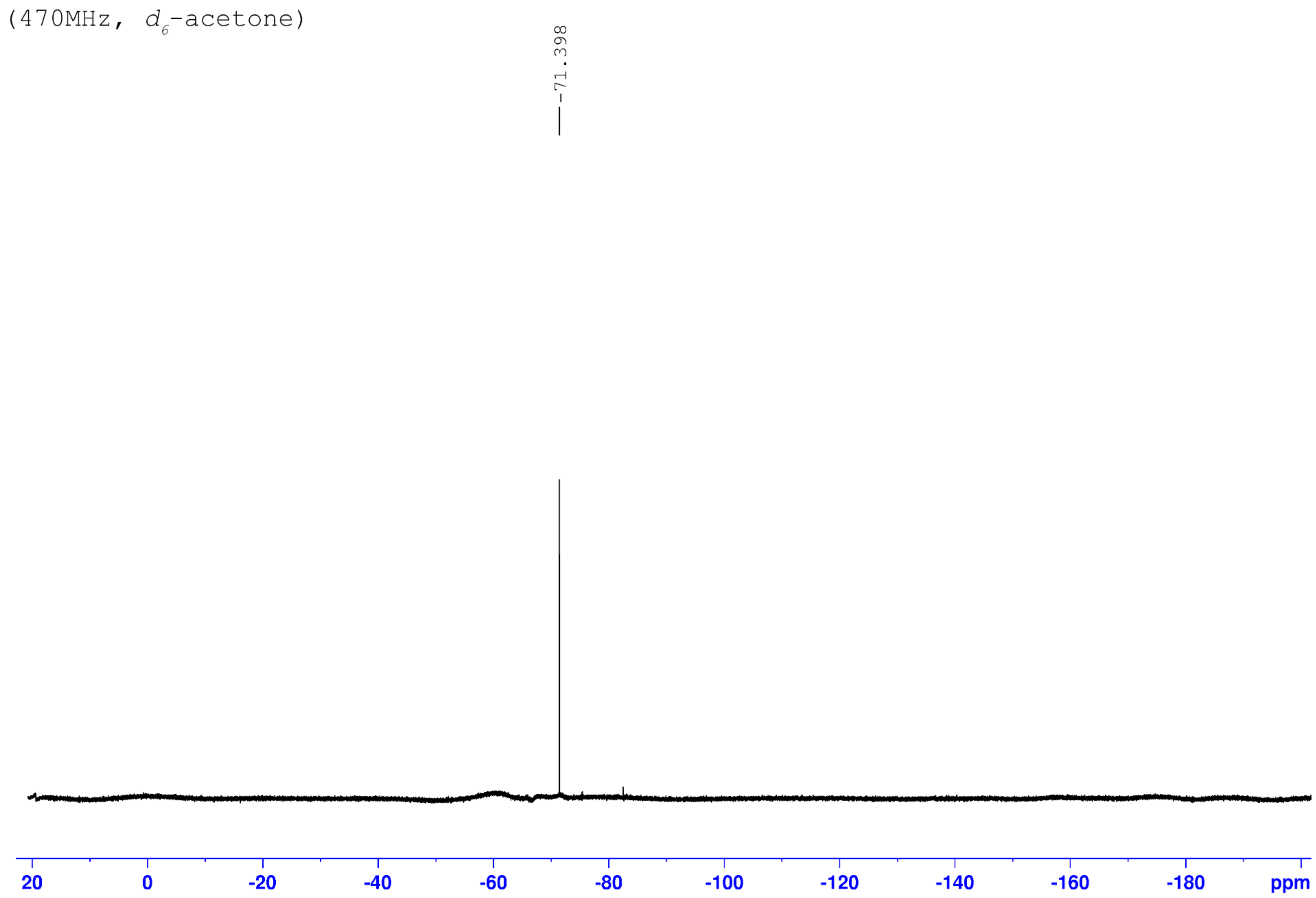

^{1}H-NMR (anomer B)

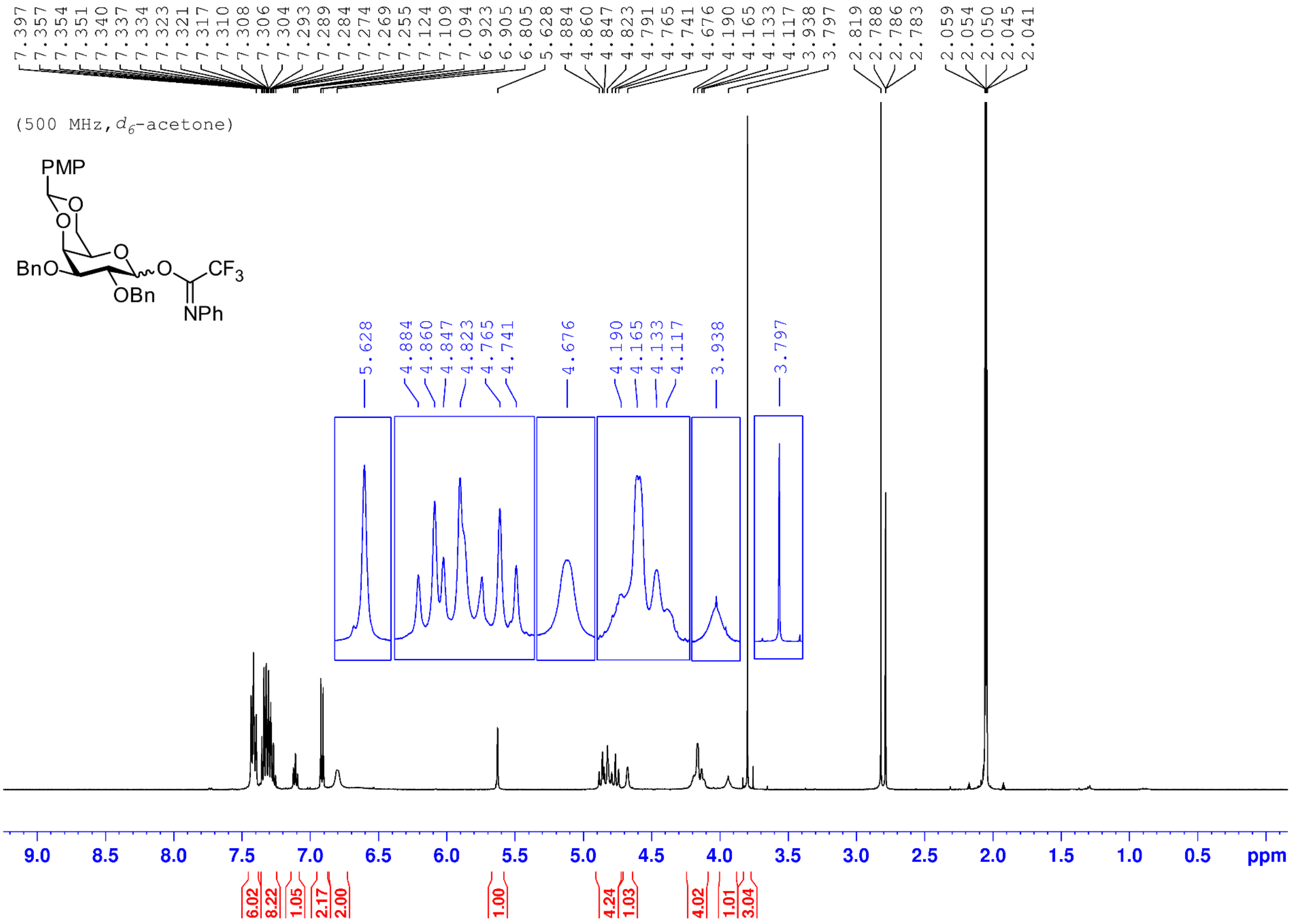

^1H-^1H COSY (anomer B)

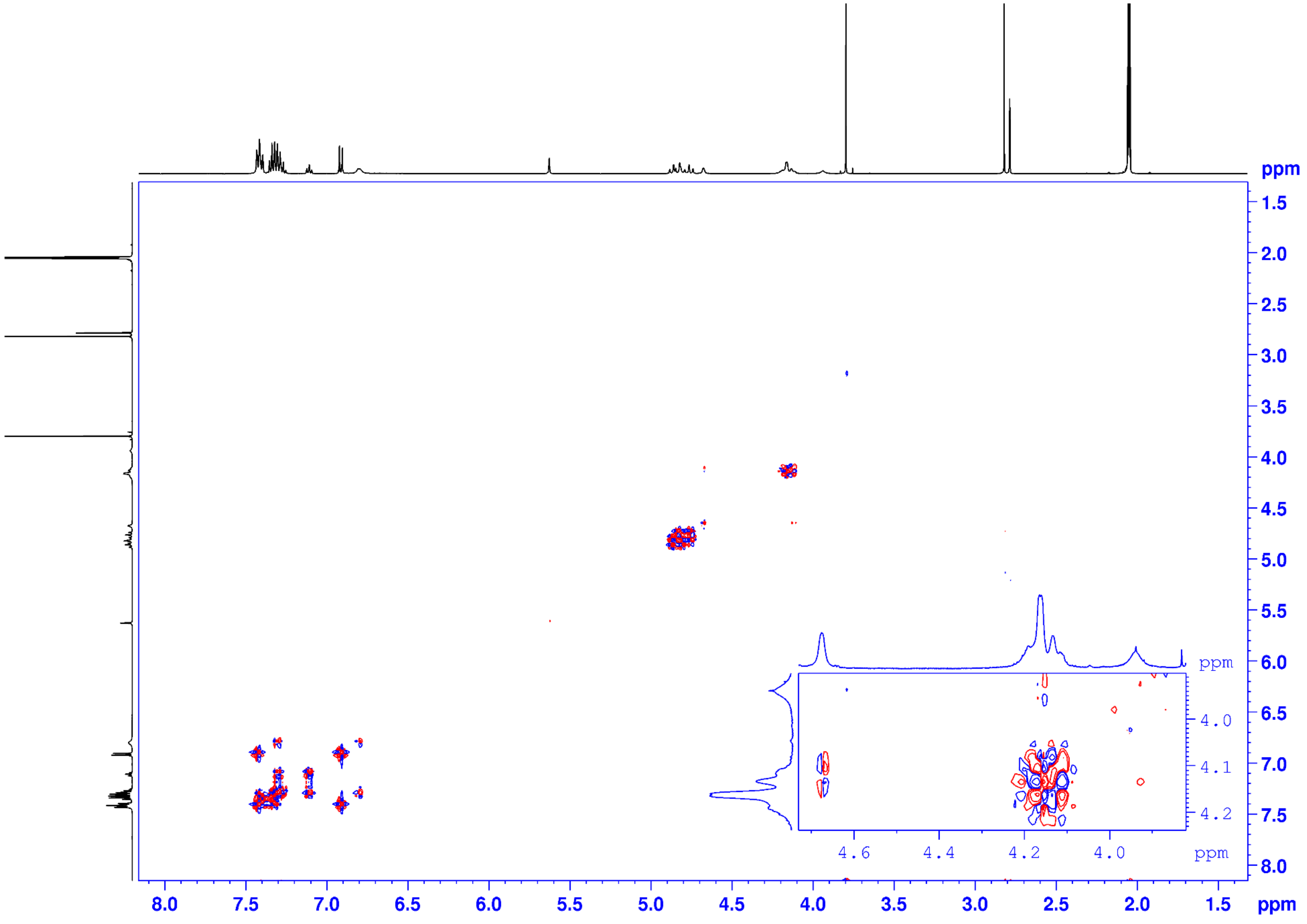

^{1}H-^{13}C HSQC (anomer B)

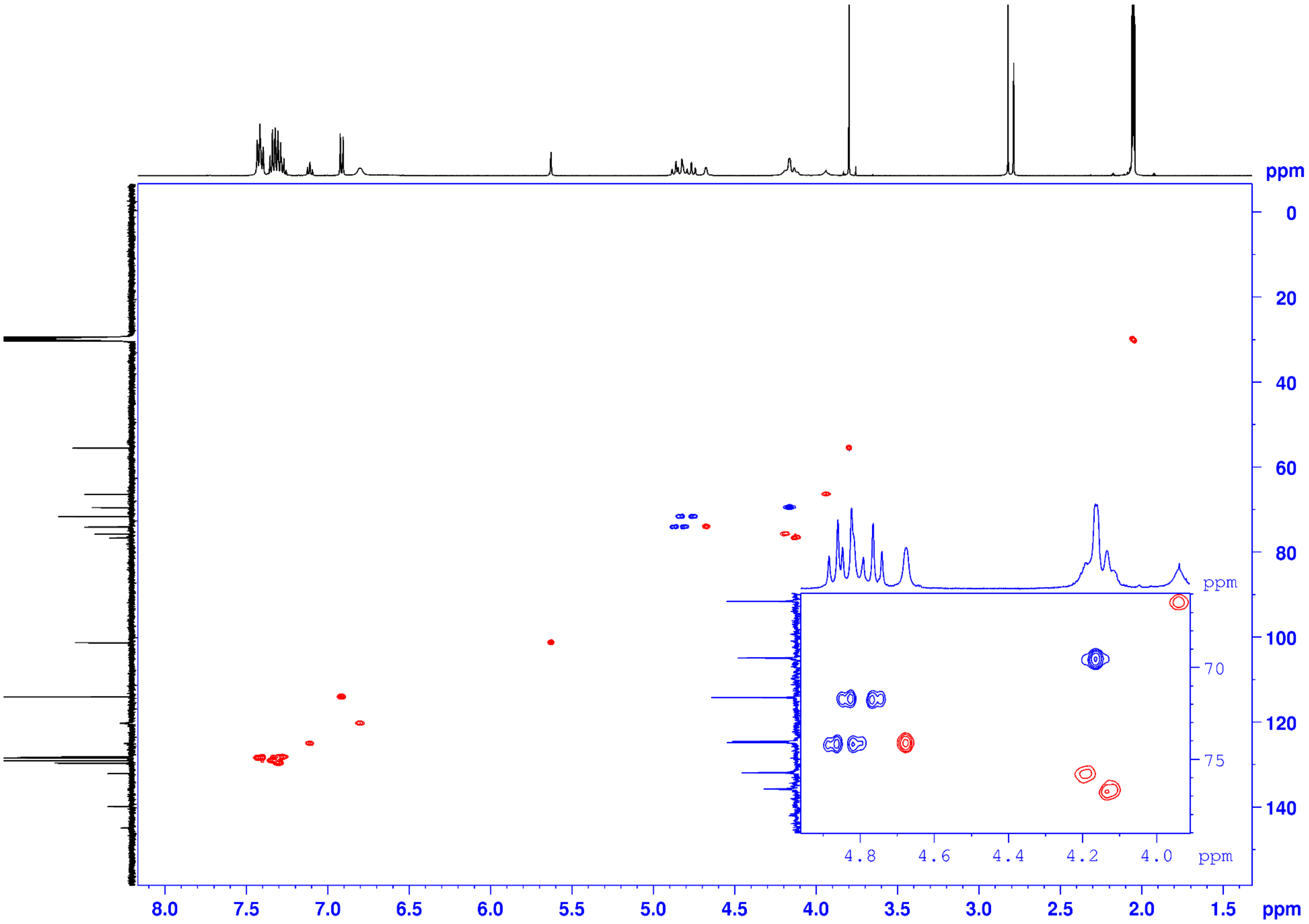

$^{13}C\{^{1}H\}$ NMR (anomer B)

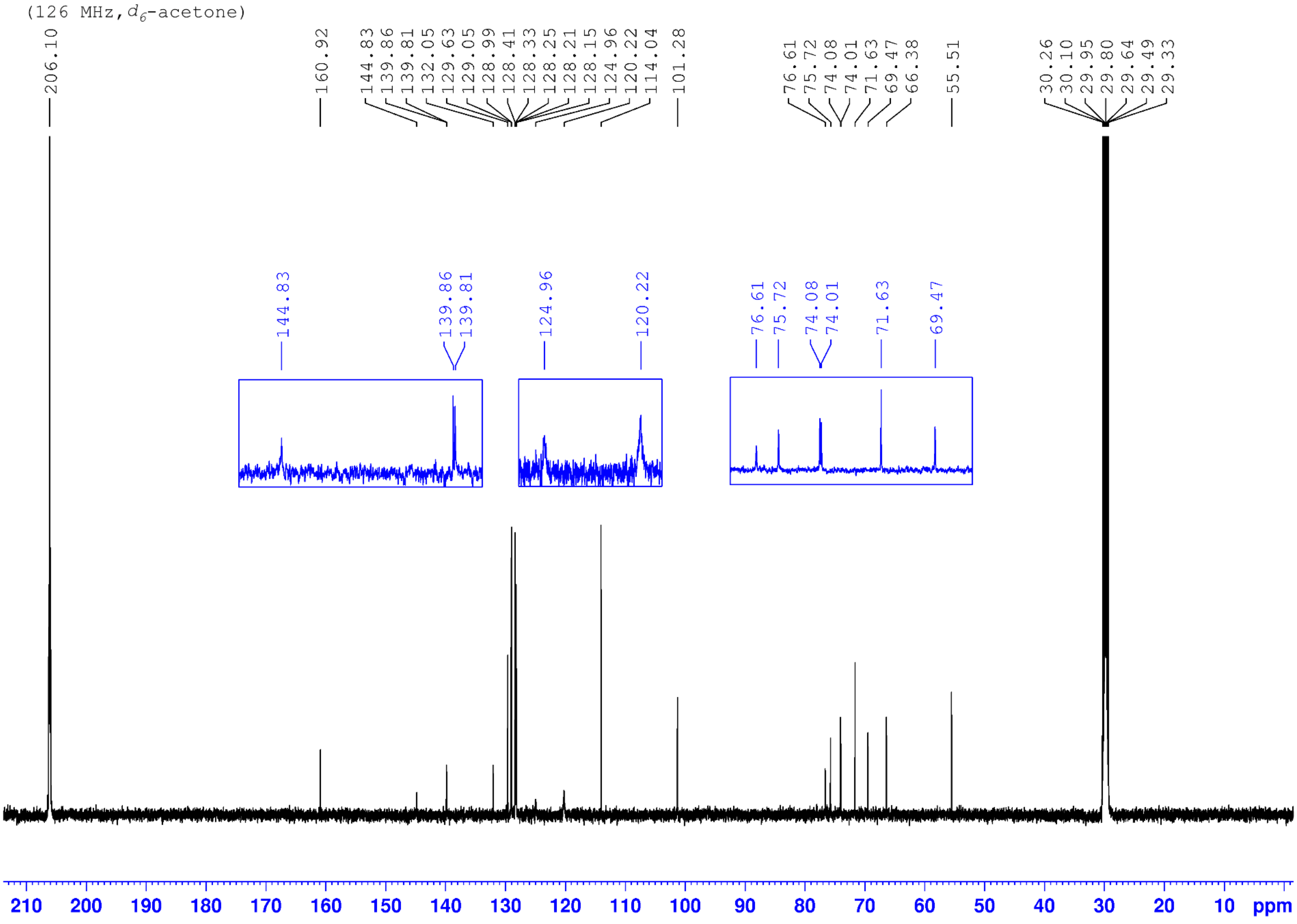

Compound **8**

^{1}H-NMR

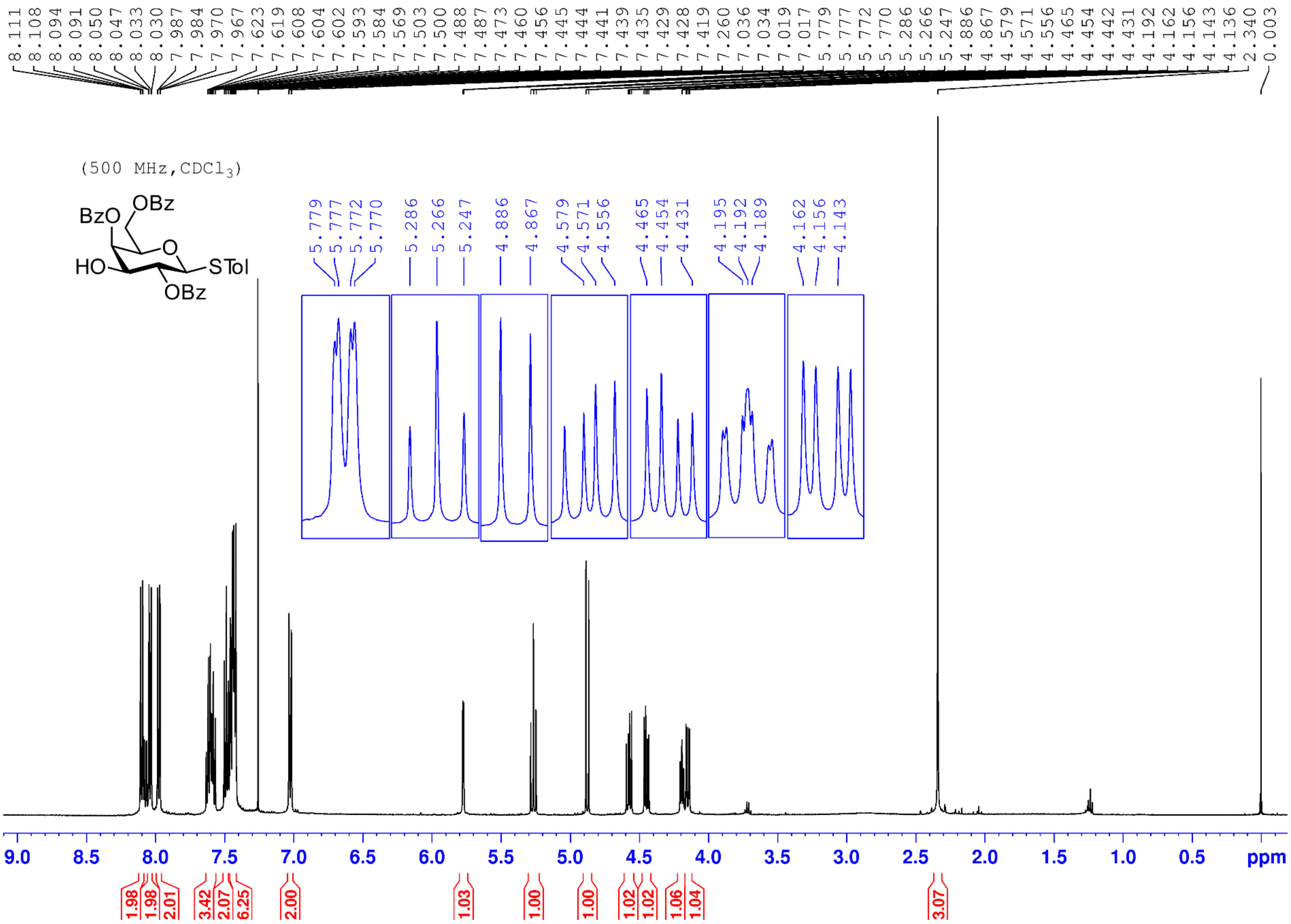

^{1}H-^{1}H COSY

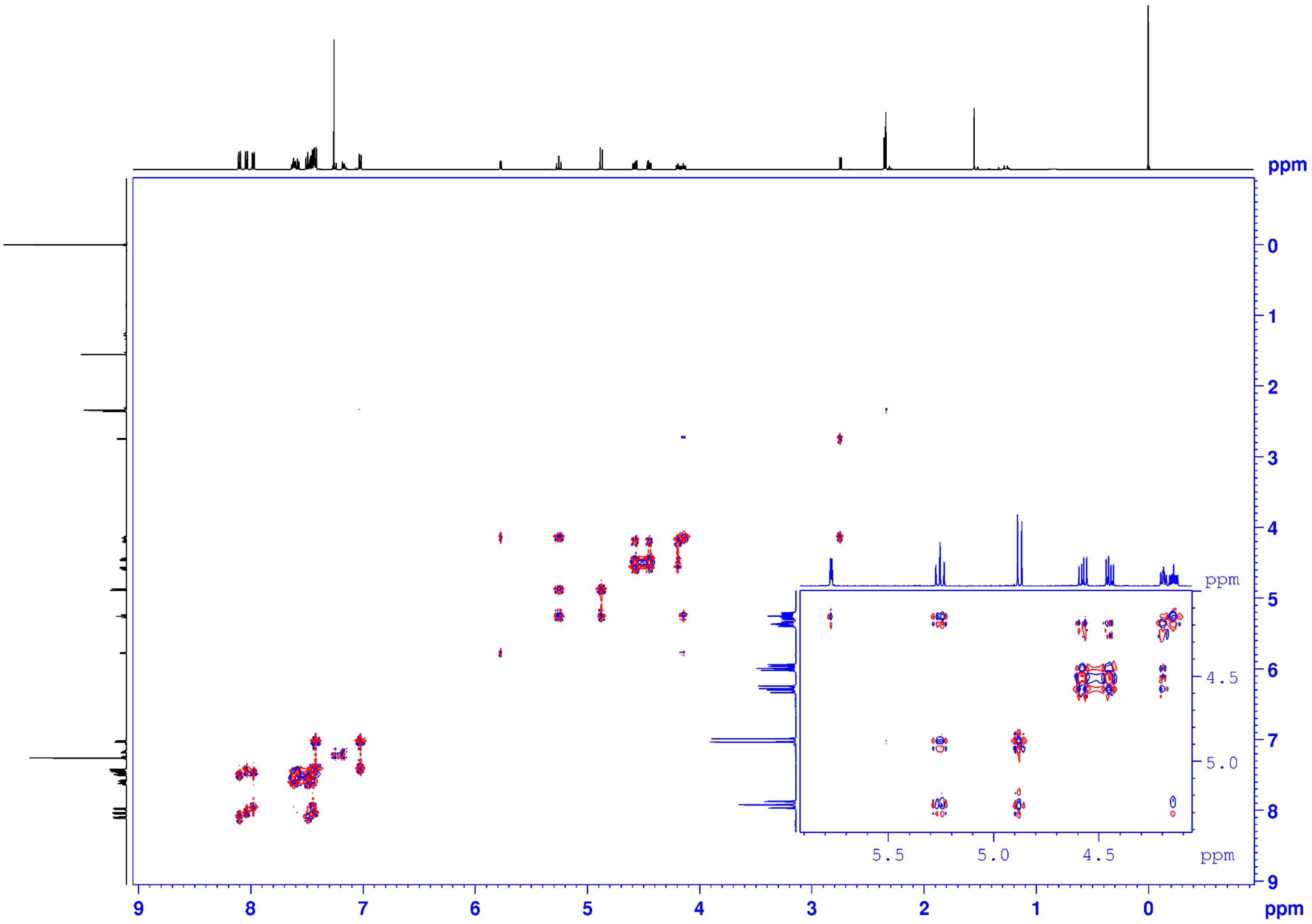

^{1}H-^{13}C HSQC

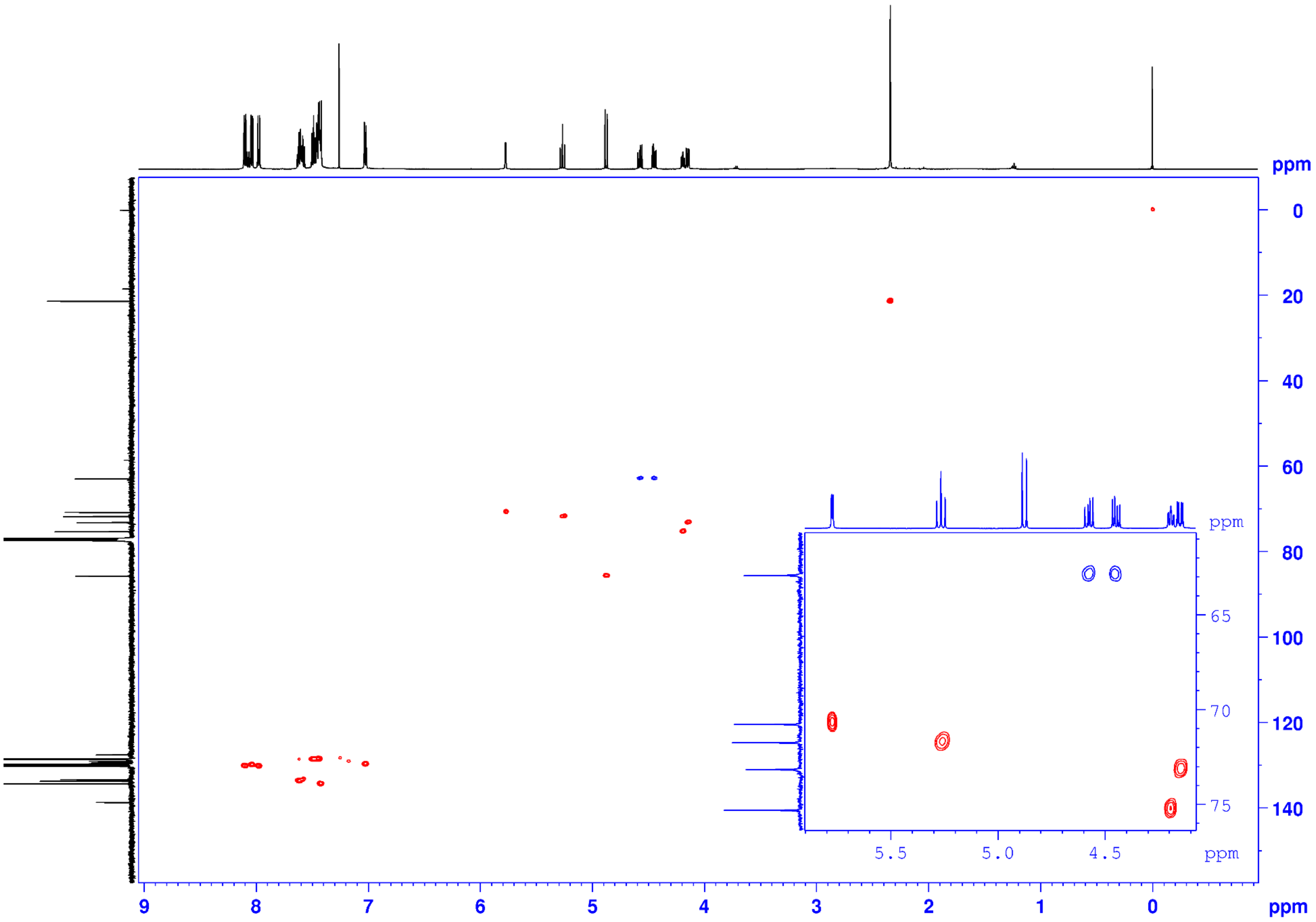

$^{1}H-^{13}C$ HMBC

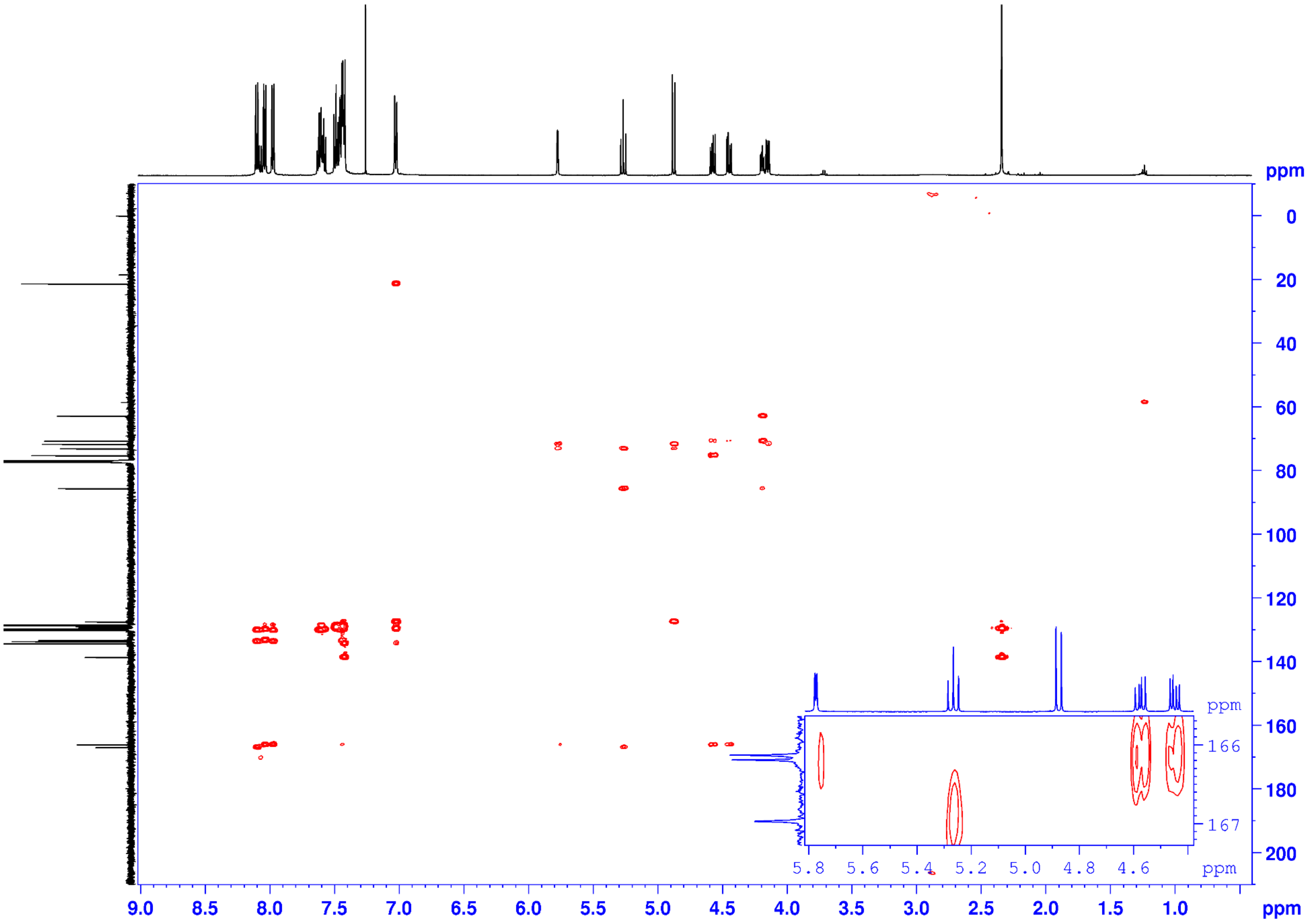

ppm
0
20
40
60
80
100
120
140
160
180
200
9.0
8.5
8.0
7.5
7.0
6.5
6.0
5.5
5.0
4.5
4.0
3.5
3.0
2.5
2.0
1.5
1.0
ppm
166
167
5.8
5.6
5.4
5.2
5.0
4.8
4.6

$^{13}C\{^{1}H\}$ NMR

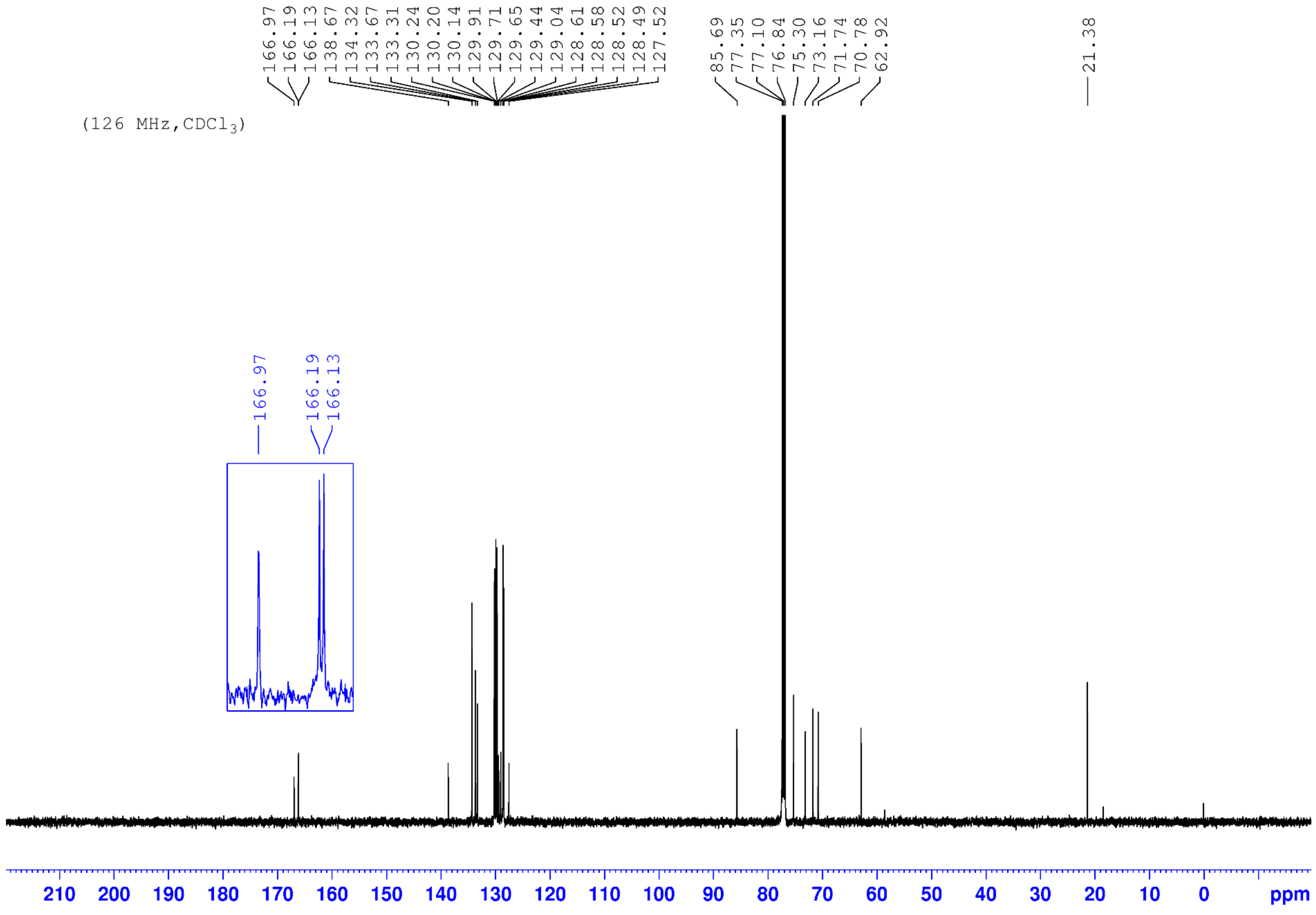
(126 MHz,CDCl3)
166.97
166.19
166.13
138.67
134.32
133.67
133.31
130.24
130.20
130.14
129.91
129.71
129.65
129.44
129.04
128.61
128.58
128.52
128.49
127.52
85.69
77.35
77.10
76.84
75.30
73.16
71.74
70.78
62.92
21.38
166.97
166.19
166.13
210 200 190 180 170 160 150 140 130 120 110 100 90 80 70 60 50 40 30 20 10 0 ppm

Compound **9**

^{1}H-NMR

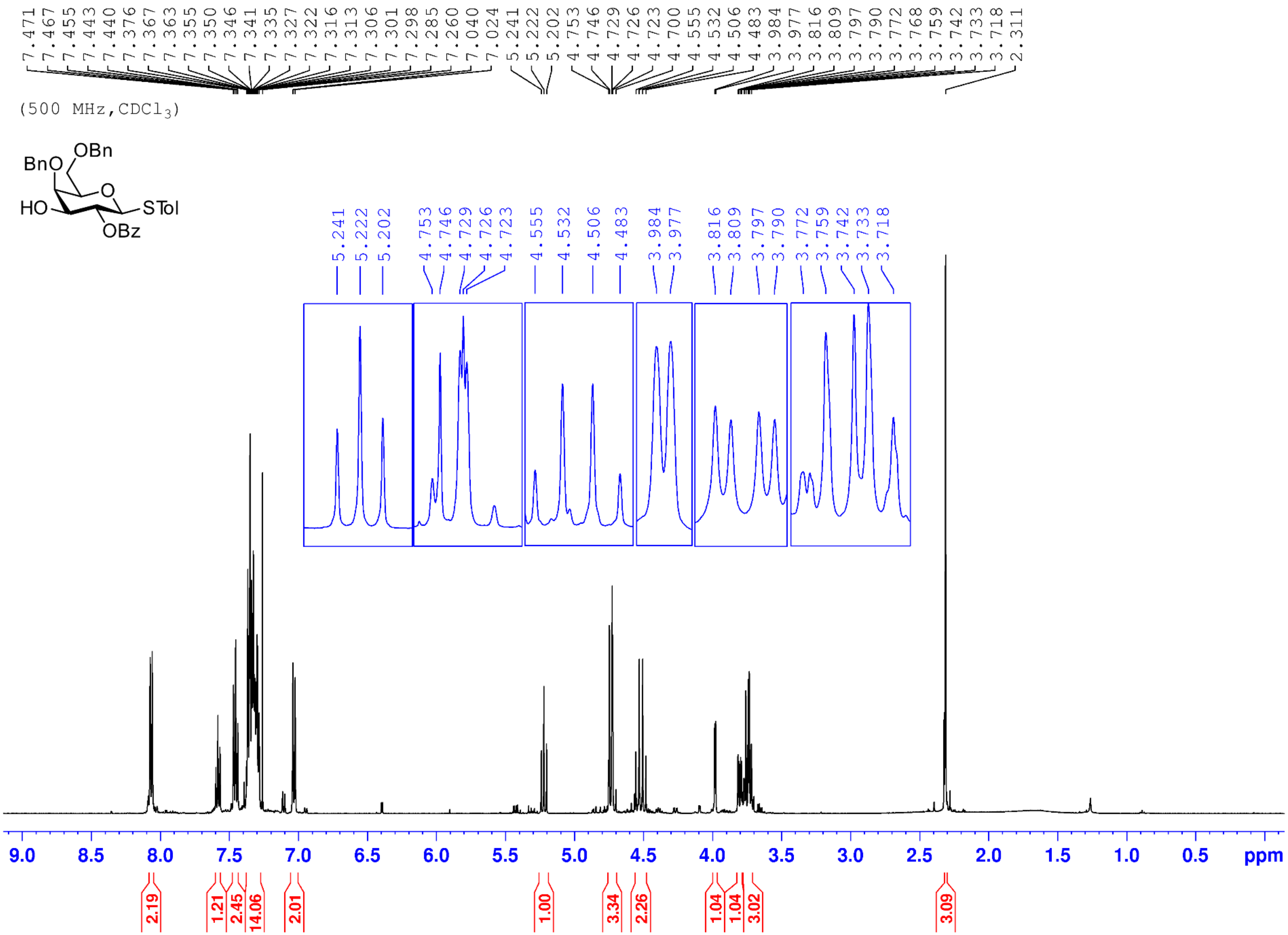

^{1}H-^{1}H COSY

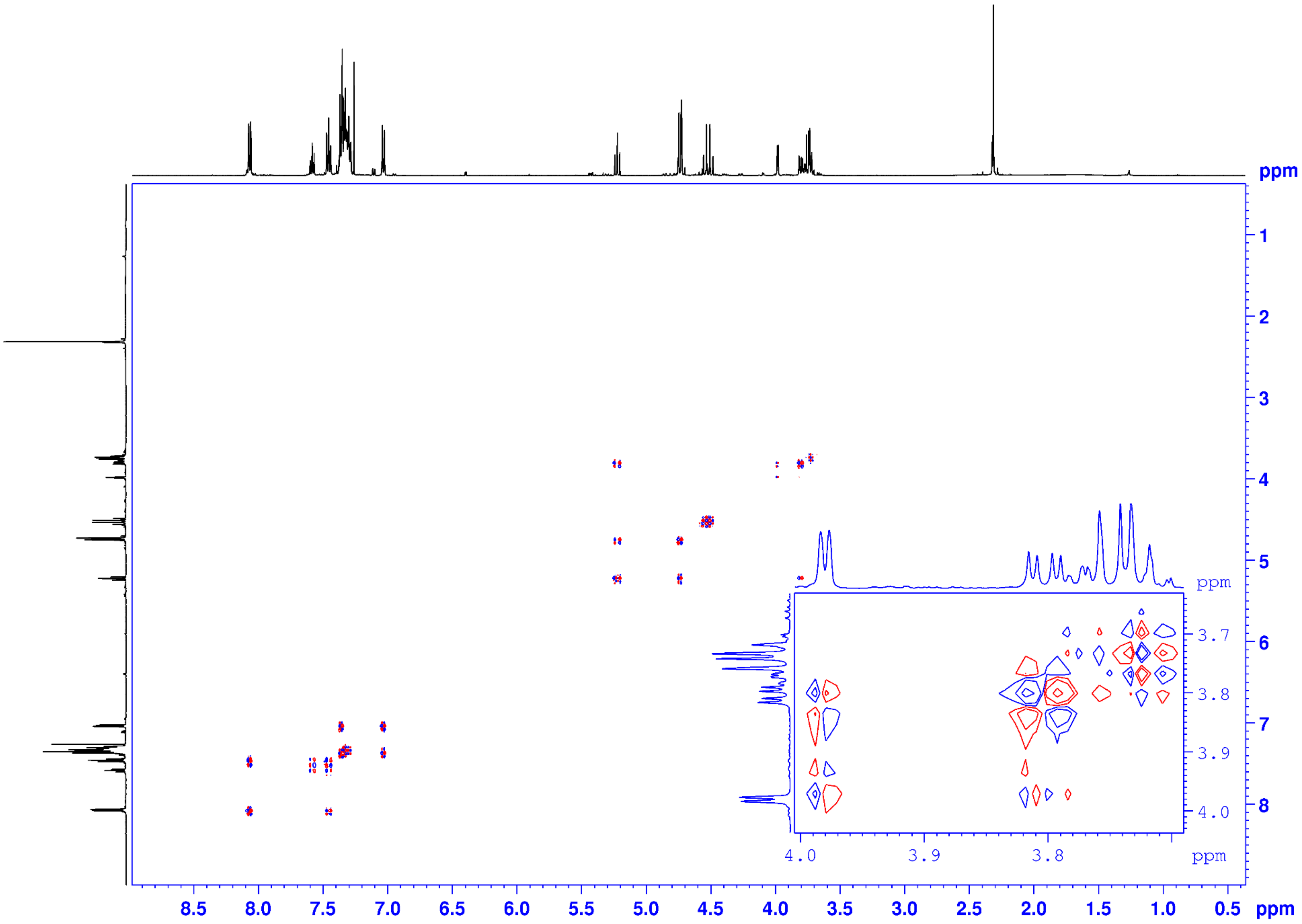

ppm
1
2
3
4
5
6
7
8
8.5
8.0
7.5
7.0
6.5
6.0
5.5
5.0
4.5
4.0
3.5
3.0
2.5
2.0
1.5
1.0
0.5
ppm
3.7
3.8
3.9
4.0
4.0
3.9
3.8
ppm
ppm

^{1}H-^{13}C HSQC

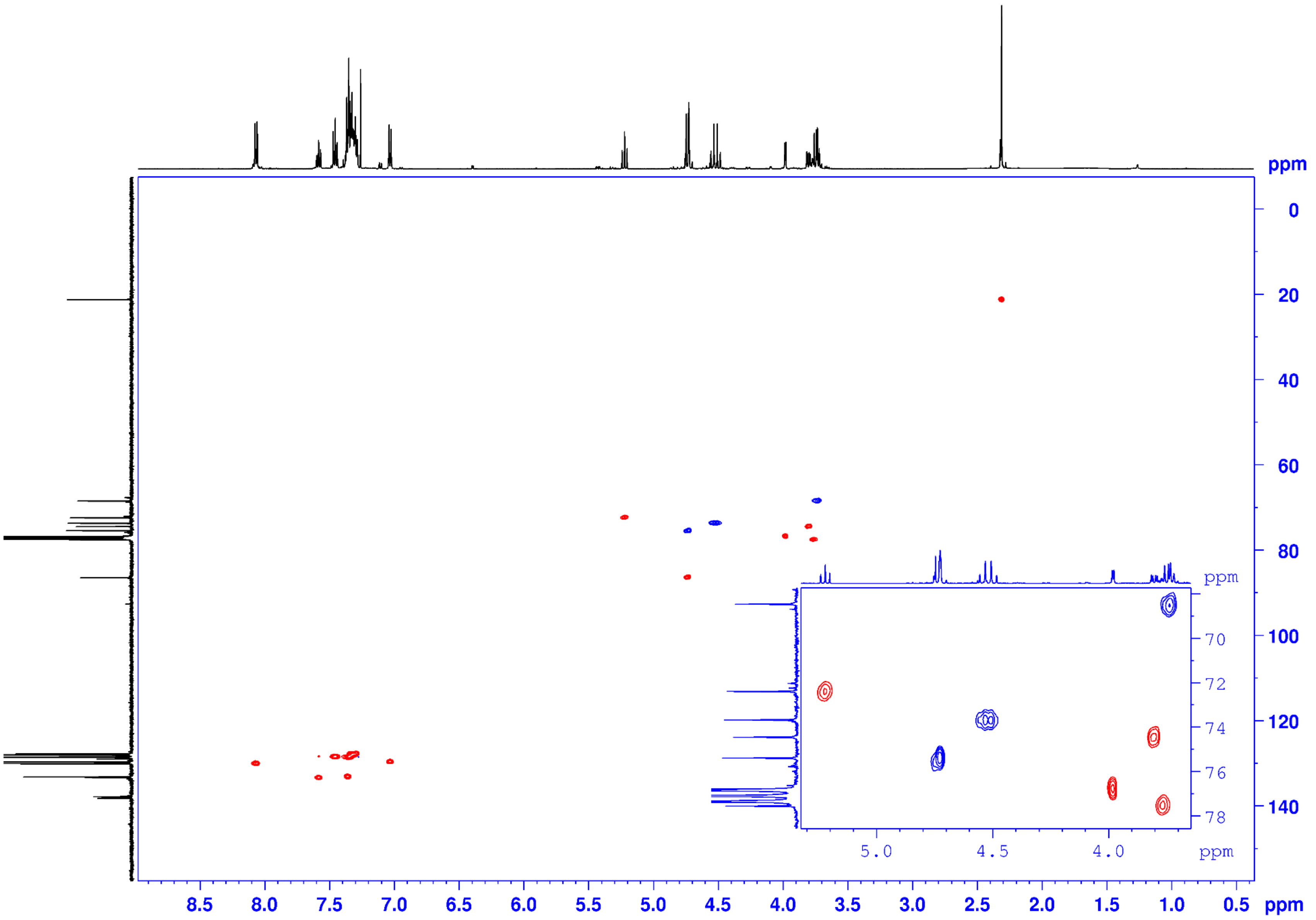

ppm
0
20
40
60
80
100
120
140
8.5
8.0
7.5
7.0
6.5
6.0
5.5
5.0
4.5
4.0
3.5
3.0
2.5
2.0
1.5
1.0
0.5
ppm
70
72
74
76
78
5.0
4.5
4.0
ppm

^{1}H-^{13}C HSQC

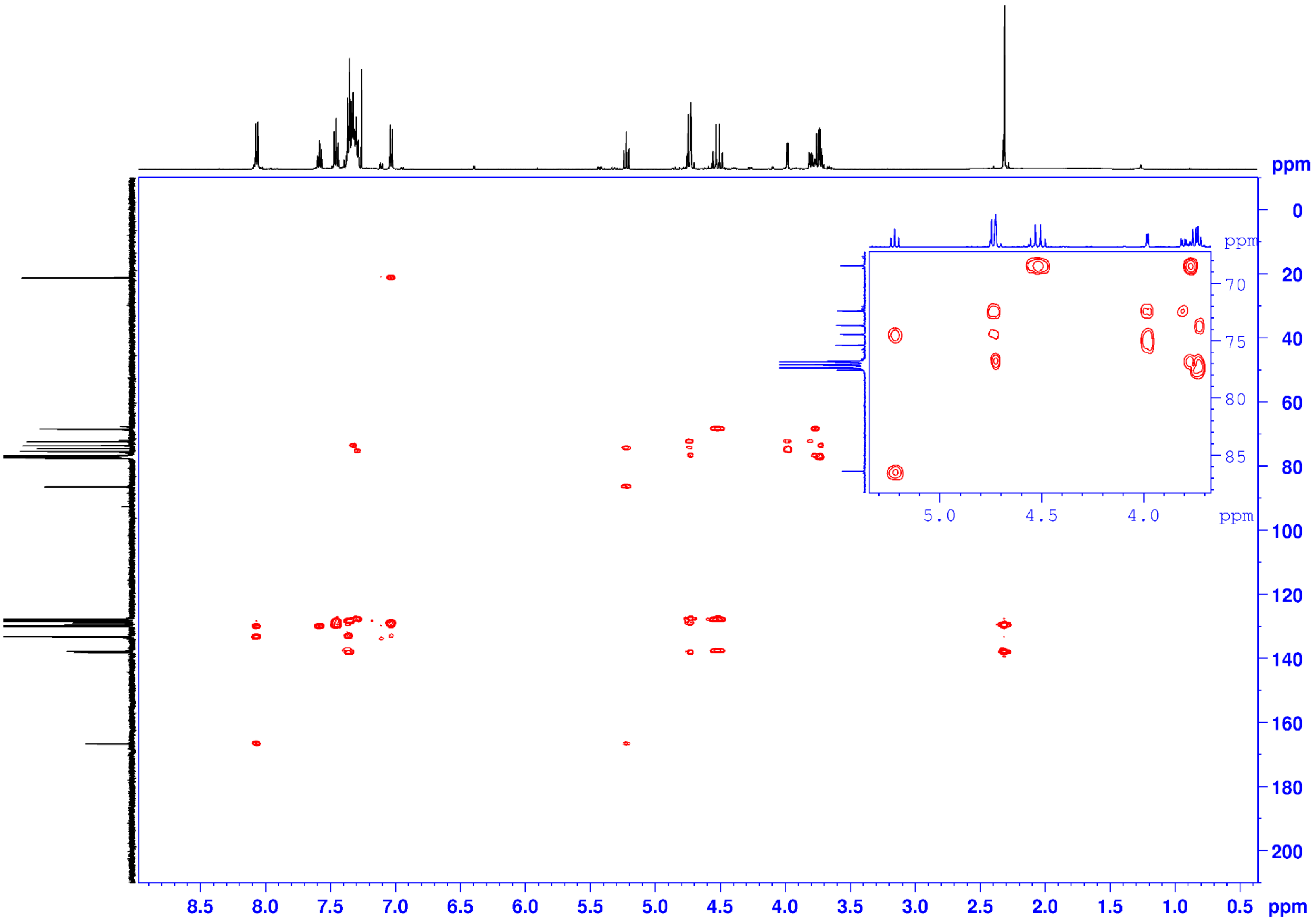

ppm
0
20
40
60
80
100
120
140
160
180
200
8.5
8.0
7.5
7.0
6.5
6.0
5.5
5.0
4.5
4.0
3.5
3.0
2.5
2.0
1.5
1.0
0.5
ppm
70
75
80
85
5.0
4.5
4.0

$^{13}C\{^{1}H\}$ NMR

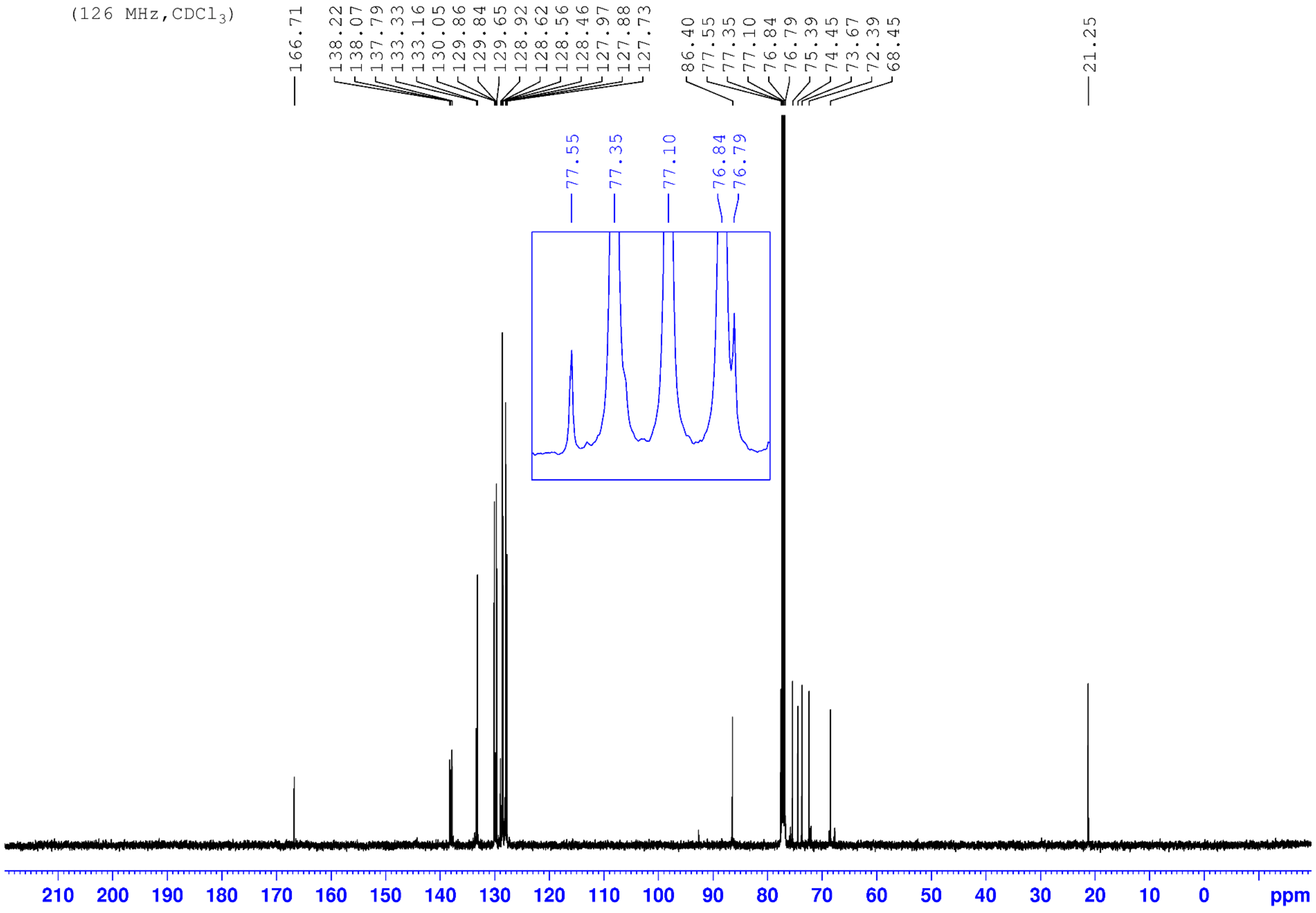
(126 MHz,CDCl3)
166.71
138.22
138.07
137.79
133.33
133.16
130.05
129.86
129.84
129.65
128.92
128.62
128.56
128.46
127.97
127.88
127.73
86.40
77.55
77.35
77.10
76.84
76.79
75.39
74.45
73.67
72.39
68.45
21.25
77.55
77.35
77.10
76.84
76.79
210
200
190
180
170
160
150
140
130
120
110
100
90
80
70
60
50
40
30
20
10
0
ppm

Compound **10**

^{1}H-NMR

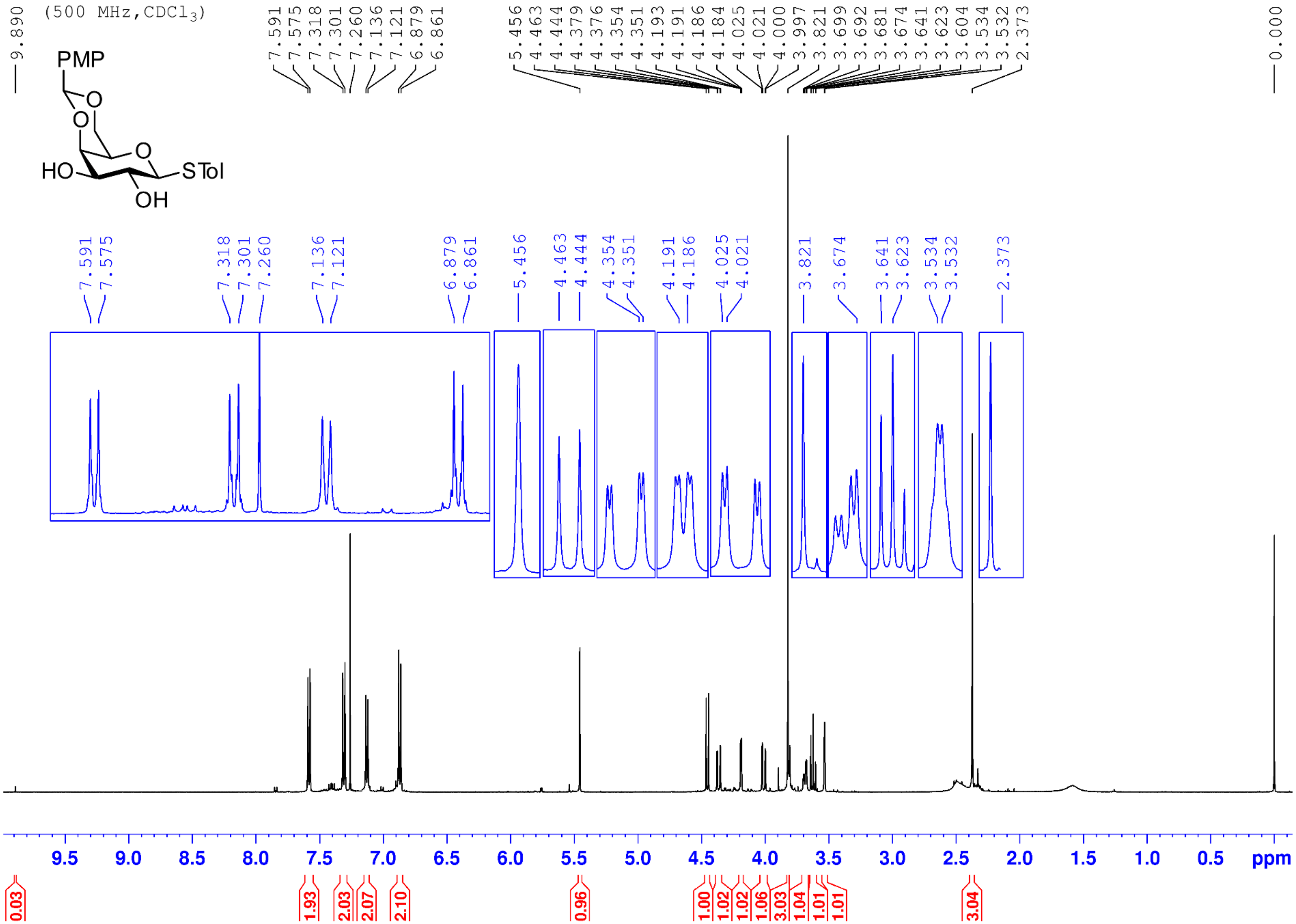

^{1}H-^{1}H COSY

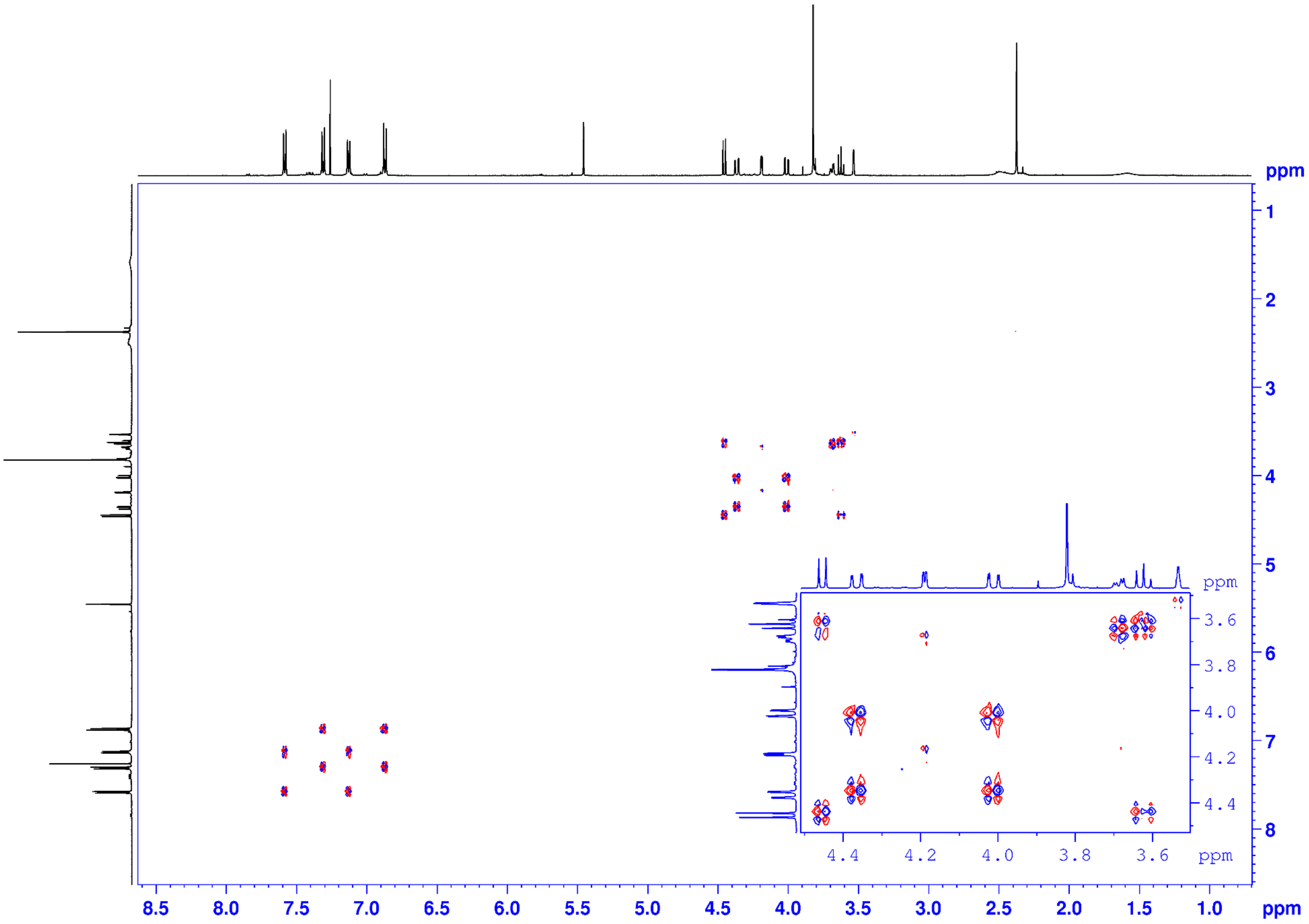

ppm
1
2
3
4
5
6
7
8
8.5
8.0
7.5
7.0
6.5
6.0
5.5
5.0
4.5
4.0
3.5
3.0
2.5
2.0
1.5
1.0
ppm
3.6
3.8
4.0
4.2
4.4
4.4
4.2
4.0
3.8
3.6
ppm

^{1}H-^{13}C HSQC

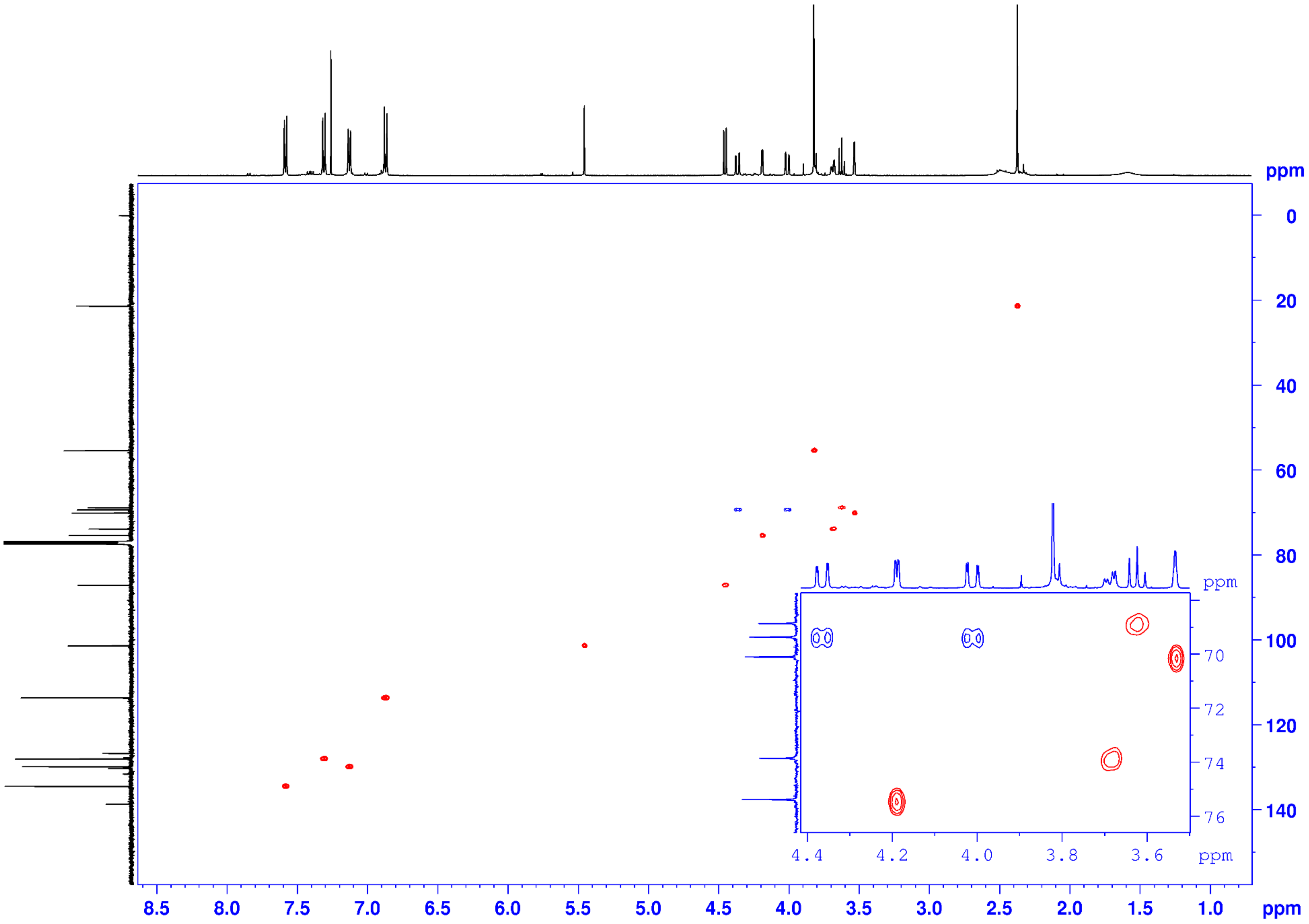

ppm
0
20
40
60
80
100
120
140
8.5
8.0
7.5
7.0
6.5
6.0
5.5
5.0
4.5
4.0
3.5
3.0
2.5
2.0
1.5
1.0
ppm
4.4
4.2
4.0
3.8
3.6
ppm
70
72
74
76
ppm

$^{13}C\{^1H\}$ NMR

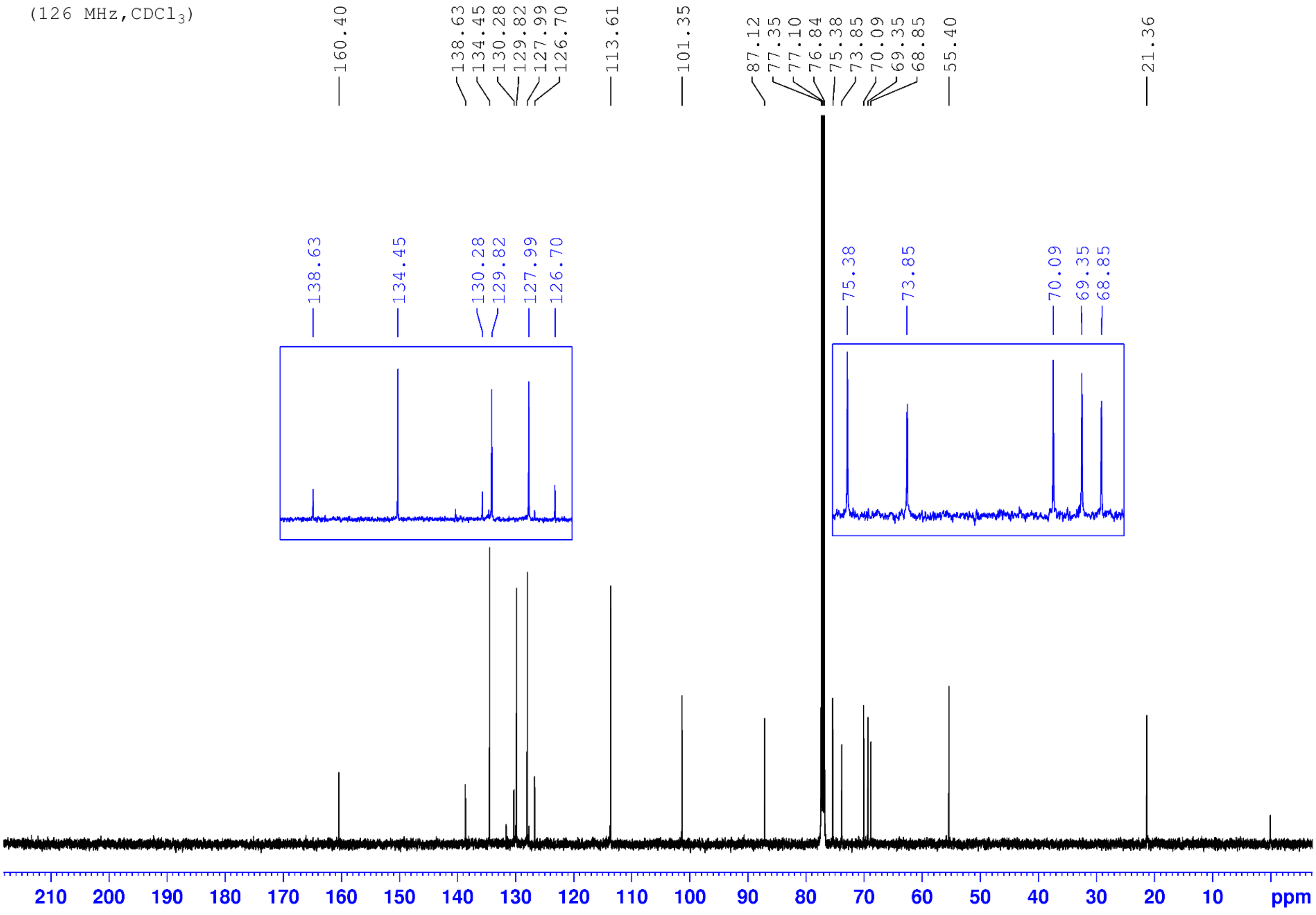

(126 MHz, CDCl3)
160.40
138.63
134.45
130.28
129.82
127.99
126.70
113.61
101.35
87.12
77.35
77.10
76.84
75.38
73.85
70.09
69.35
68.85
55.40
21.36
210 200 190 180 170 160 150 140 130 120 110 100 90 80 70 60 50 40 30 20 10 ppm

Compound **11**

^{1}H-NMR

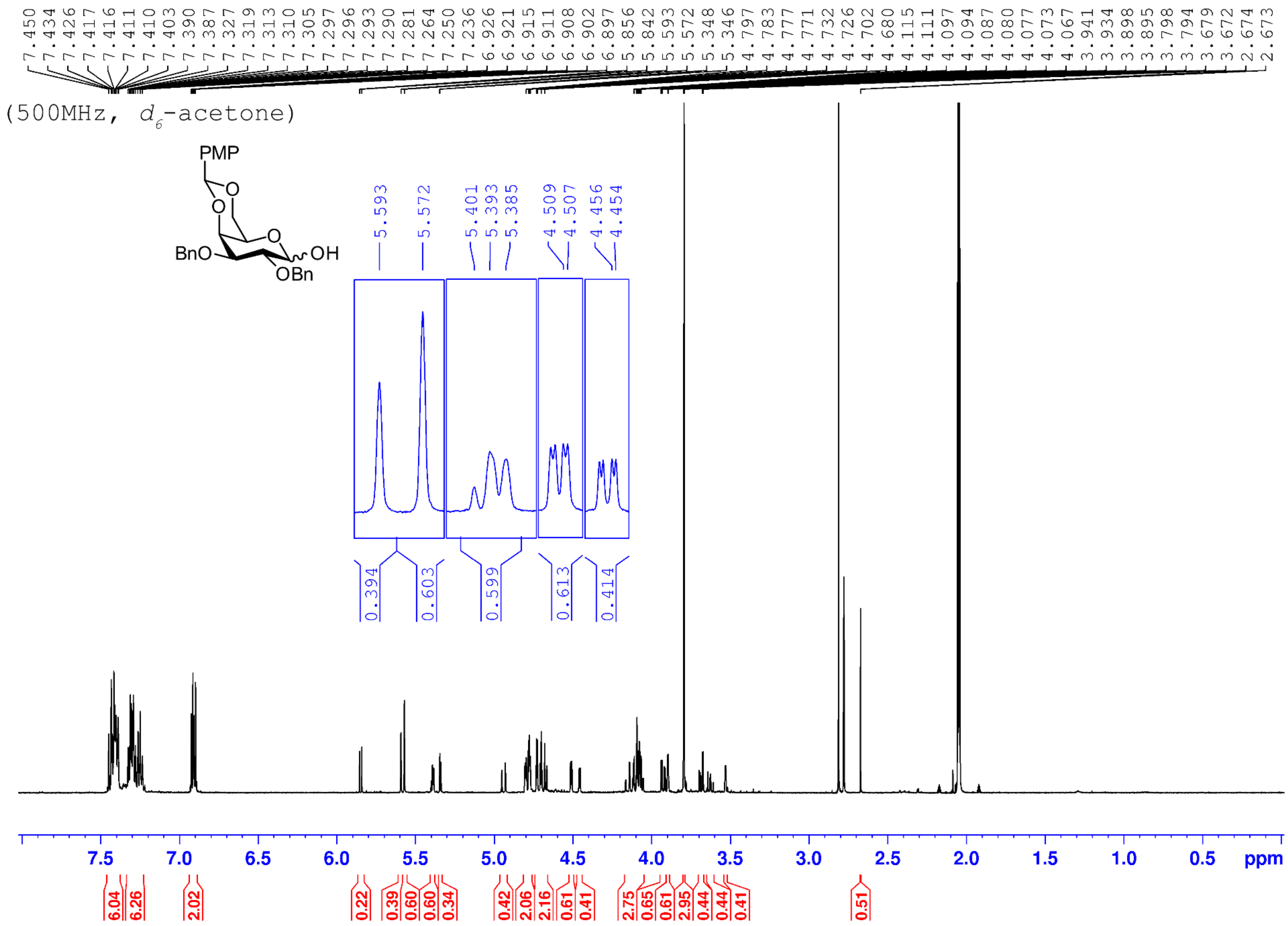

^1H-^1H COSY

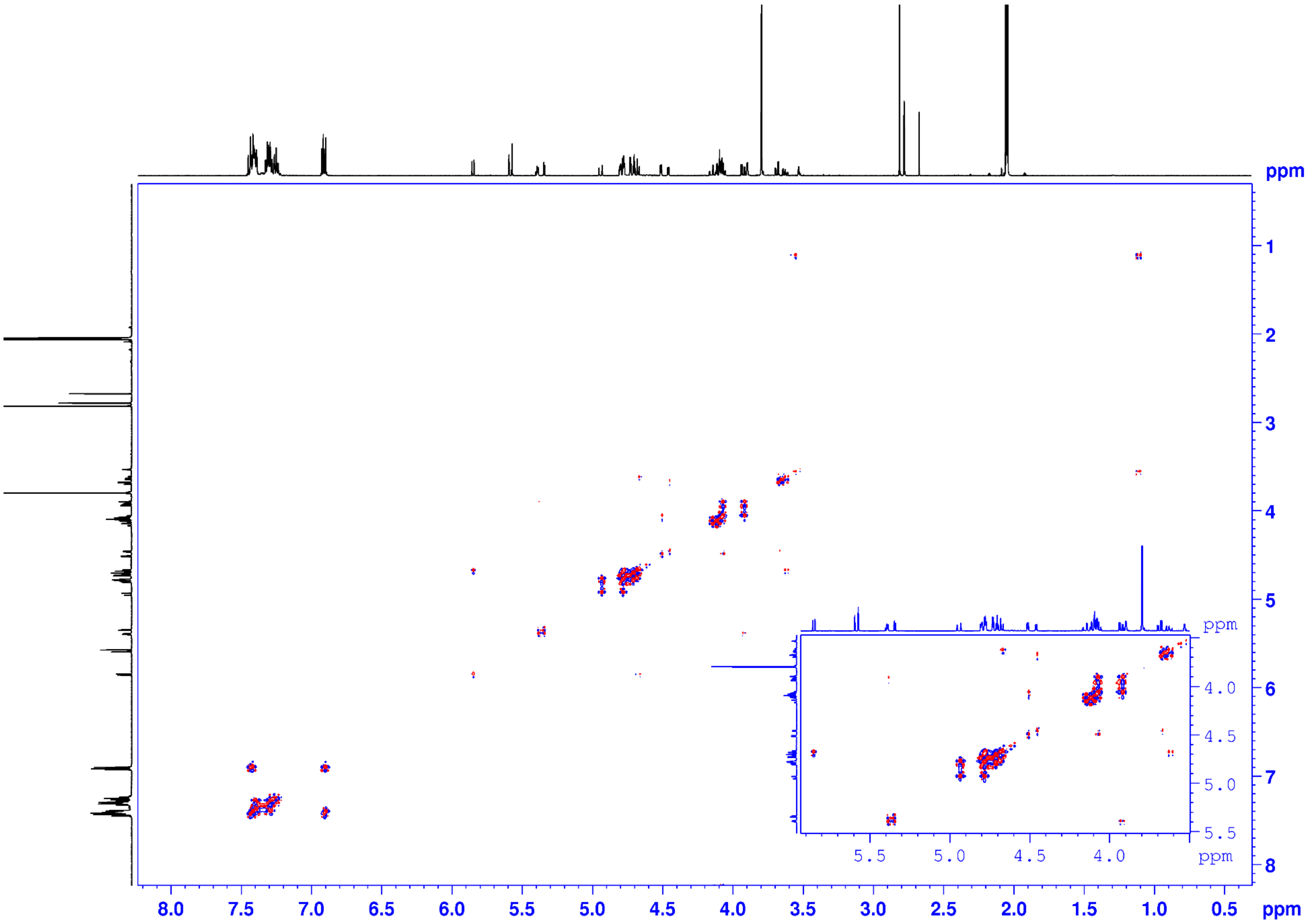
ppm
1
2
3
4
5
6
7
8
8.0
7.5
7.0
6.5
6.0
5.5
5.0
4.5
4.0
3.5
3.0
2.5
2.0
1.5
1.0
0.5
ppm
4.0
4.5
5.0
5.5
5.5
5.0
4.5
4.0
ppm

^{1}H-^{13}C HSQC

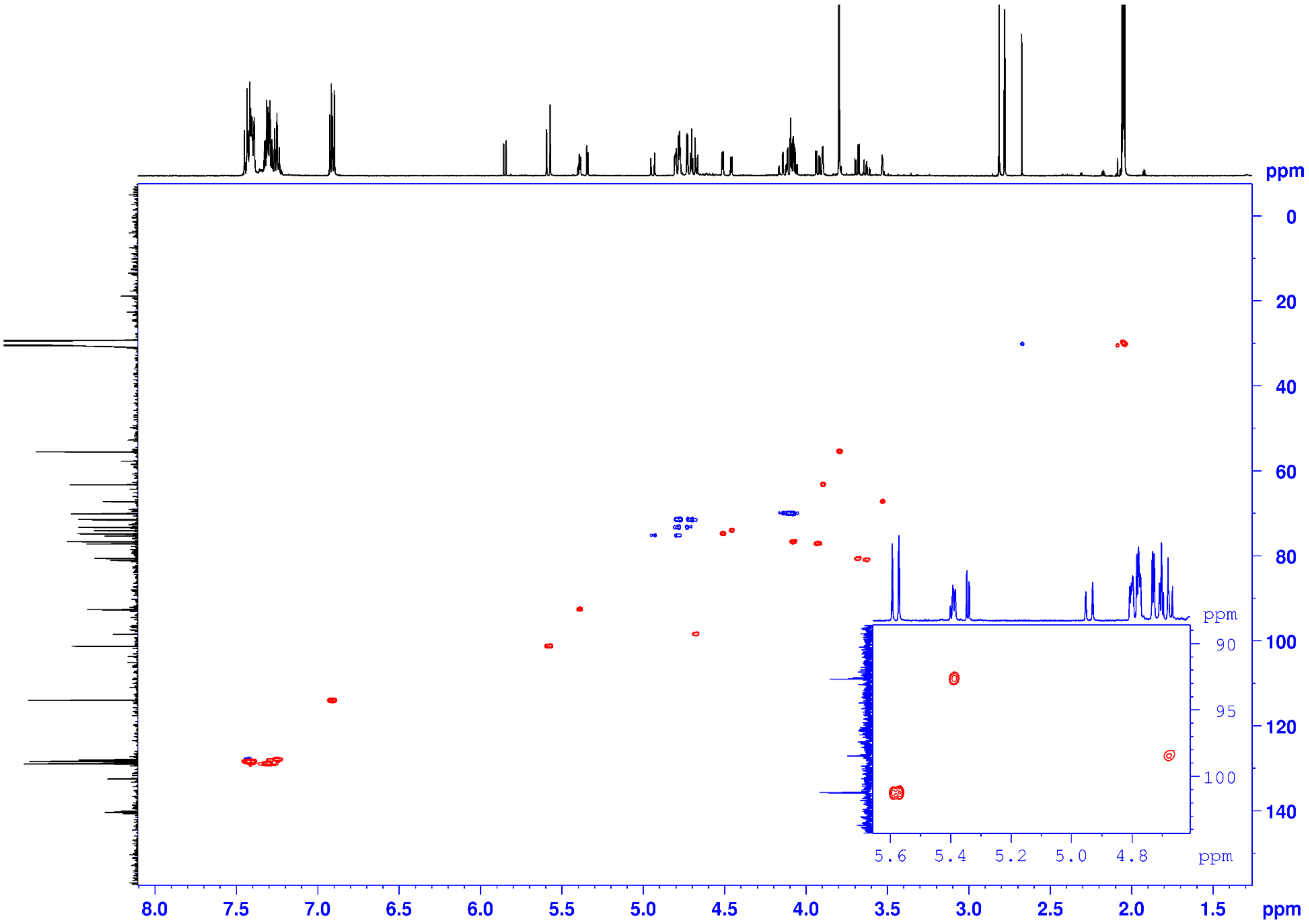

ppm
0
20
40
60
80
100
120
140
8.0
7.5
7.0
6.5
6.0
5.5
5.0
4.5
4.0
3.5
3.0
2.5
2.0
1.5
ppm
90
95
100
5.6
5.4
5.2
5.0
4.8

$^{13}C\{^1H\}$ NMR

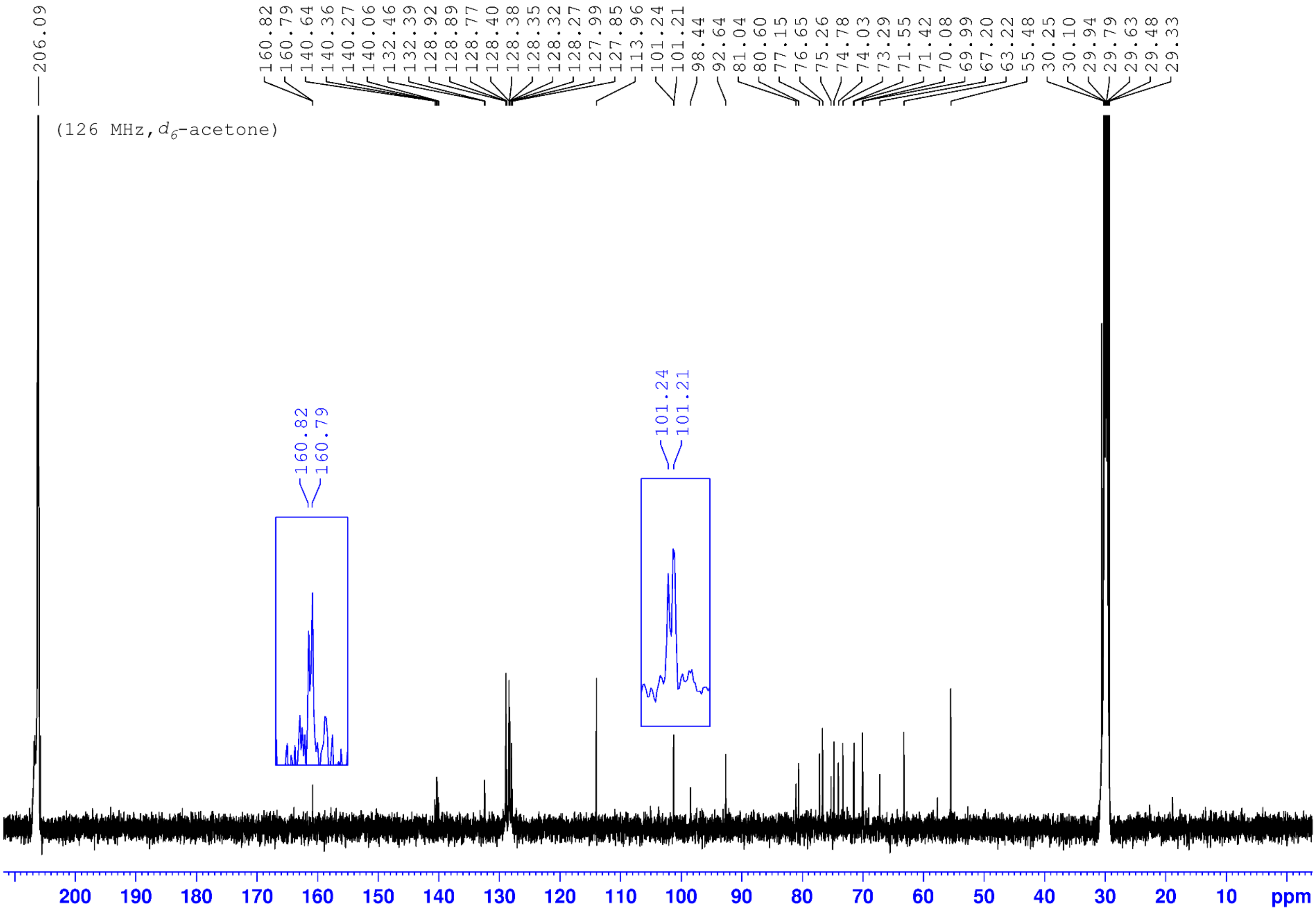

(126 MHz, d_6-acetone)
206.09
160.82
160.79
140.64
140.36
140.27
140.06
132.46
132.39
128.92
128.89
128.77
128.40
128.38
128.35
128.32
128.27
127.99
127.85
113.96
101.24
101.21
98.44
92.64
81.04
80.60
77.15
76.65
75.26
74.78
74.03
73.29
71.55
71.42
70.08
69.99
67.20
63.22
55.48
30.25
30.10
29.94
29.79
29.63
29.48
29.33
200
190
180
170
160
150
140
130
120
110
100
90
80
70
60
50
40
30
20
10
ppm

Compound **12**

^{1}H-NMR

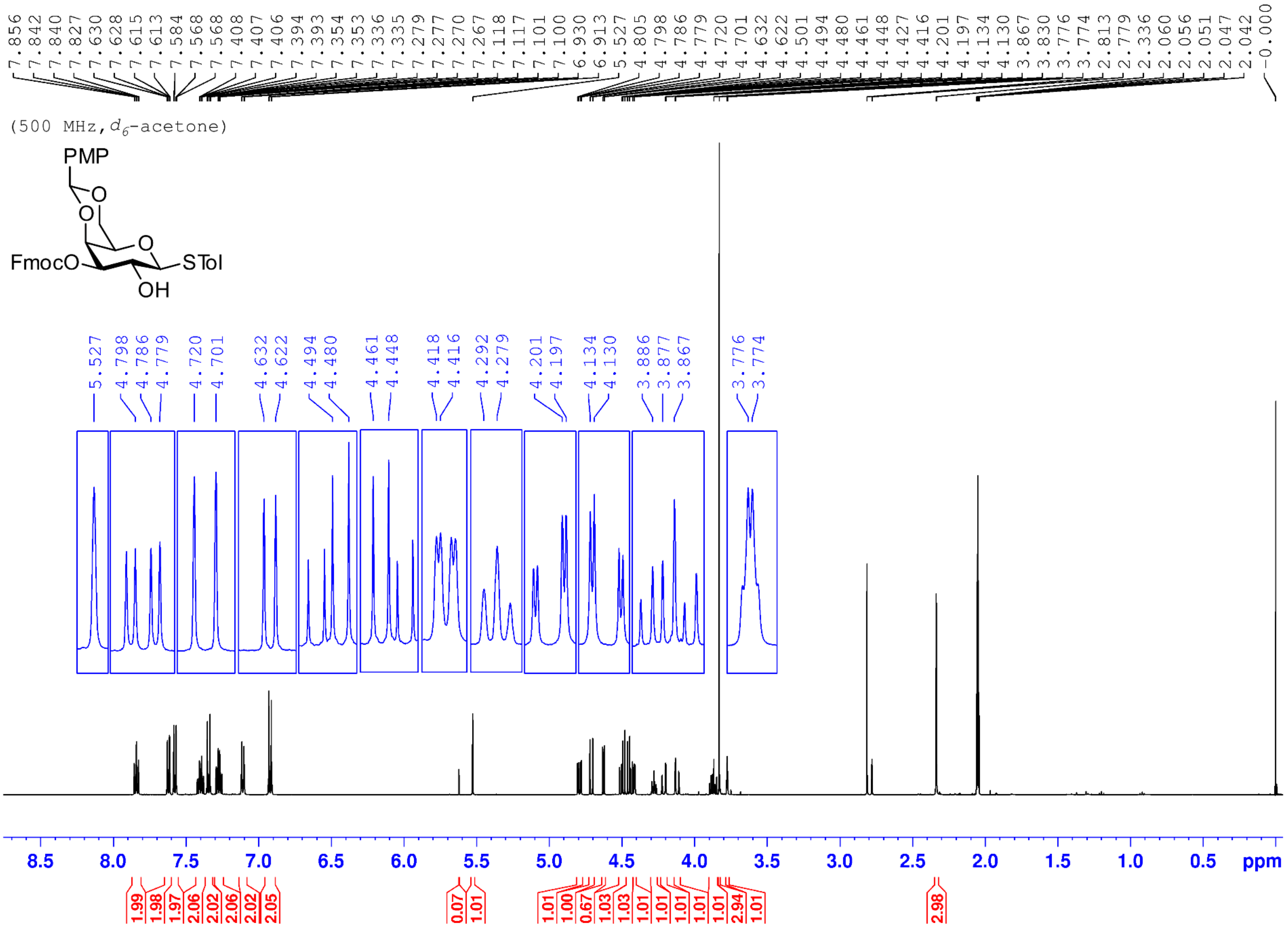

^1H-^1H COSY

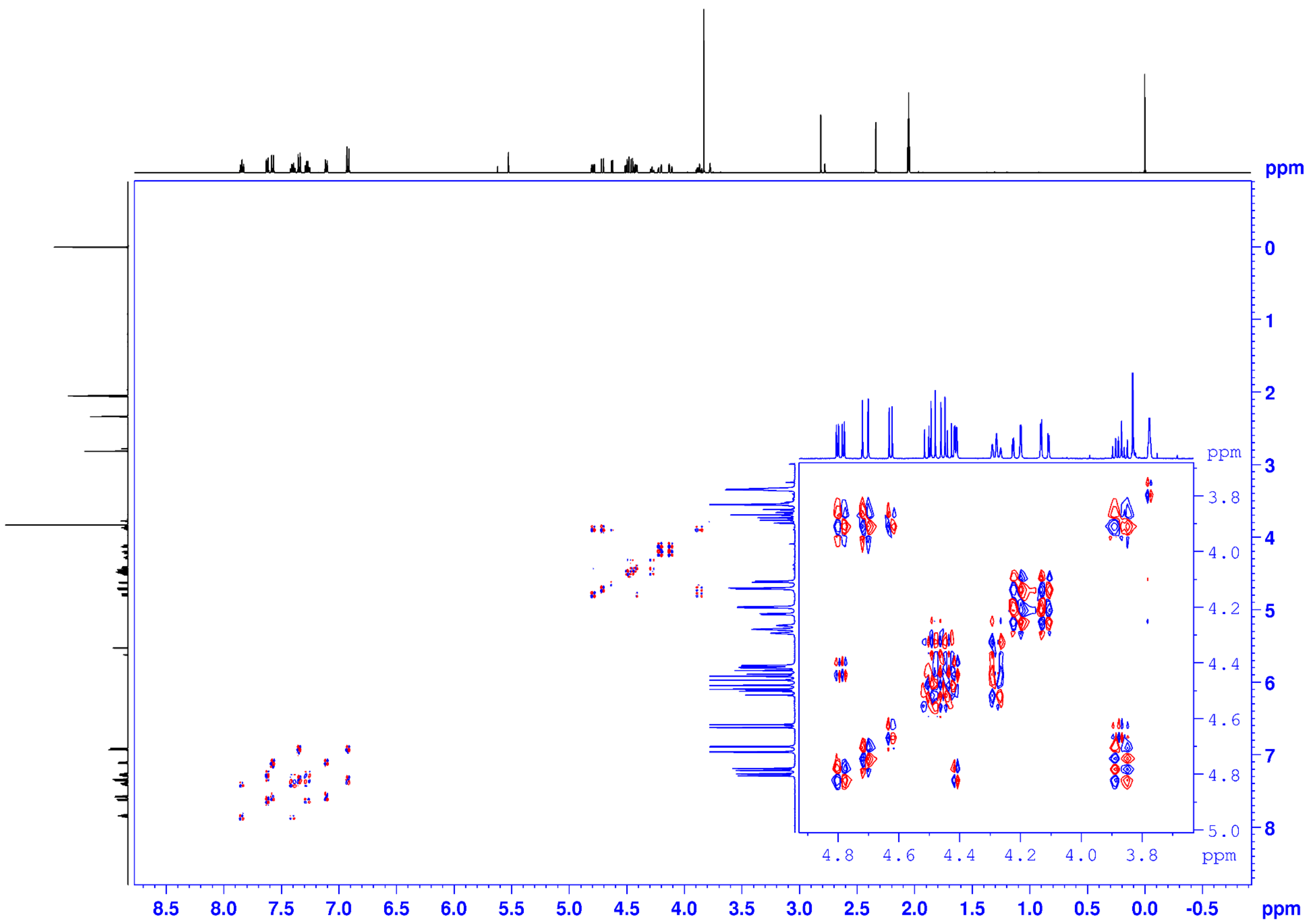

ppm
0
1
2
3
4
5
6
7
8
8.5
8.0
7.5
7.0
6.5
6.0
5.5
5.0
4.5
4.0
3.5
3.0
2.5
2.0
1.5
1.0
0.5
0.0
-0.5
ppm
3.8
4.0
4.2
4.4
4.6
4.8
5.0
4.8
4.6
4.4
4.2
4.0
3.8
ppm

^1H-^{13}C HSQC

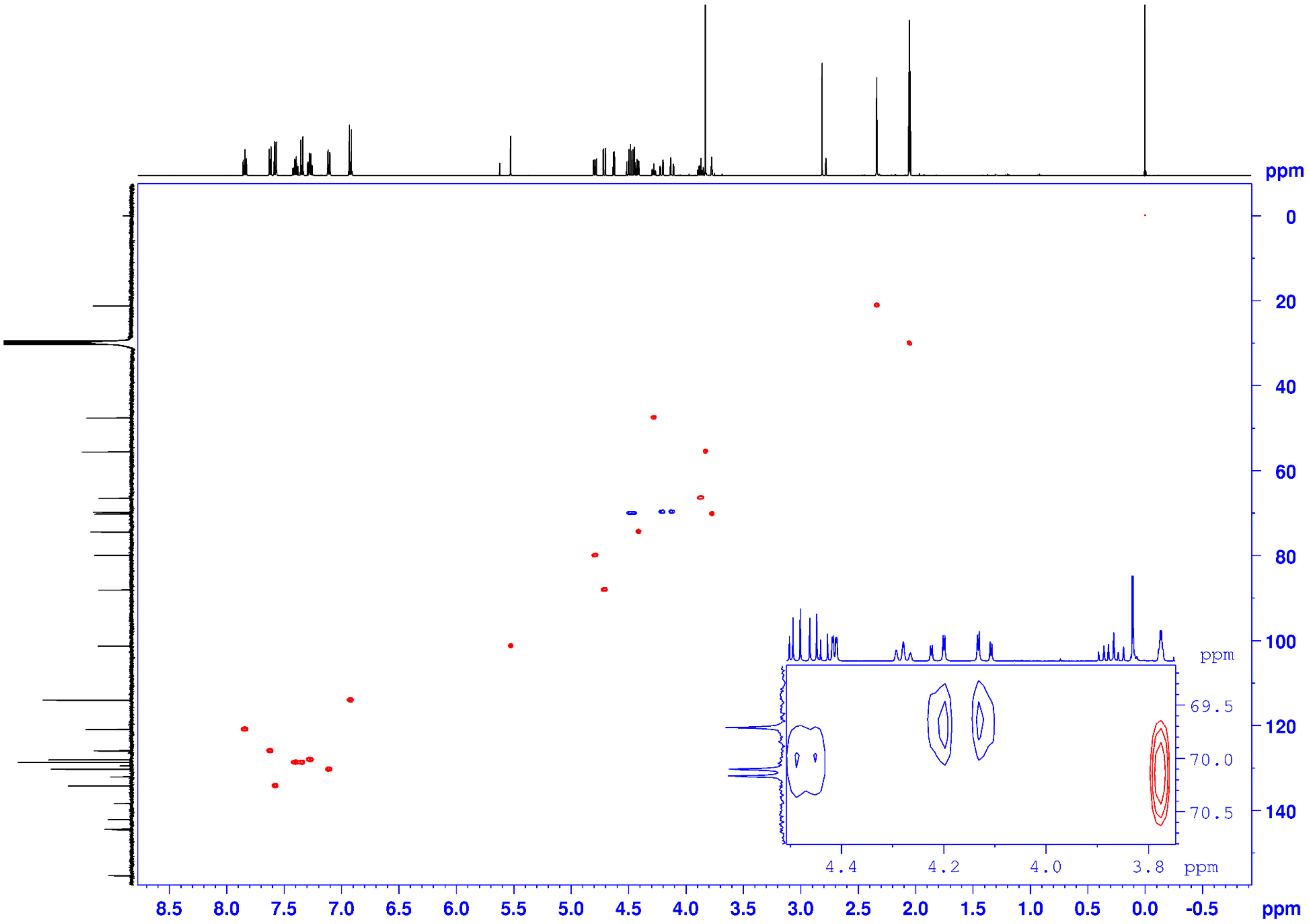
ppm
0
20
40
60
80
100
120
140
8.5
8.0
7.5
7.0
6.5
6.0
5.5
5.0
4.5
4.0
3.5
3.0
2.5
2.0
1.5
1.0
0.5
0.0
-0.5
ppm
69.5
70.0
70.5
4.4
4.2
4.0
3.8
ppm

$^{13}C\{^{1}H\}$ NMR

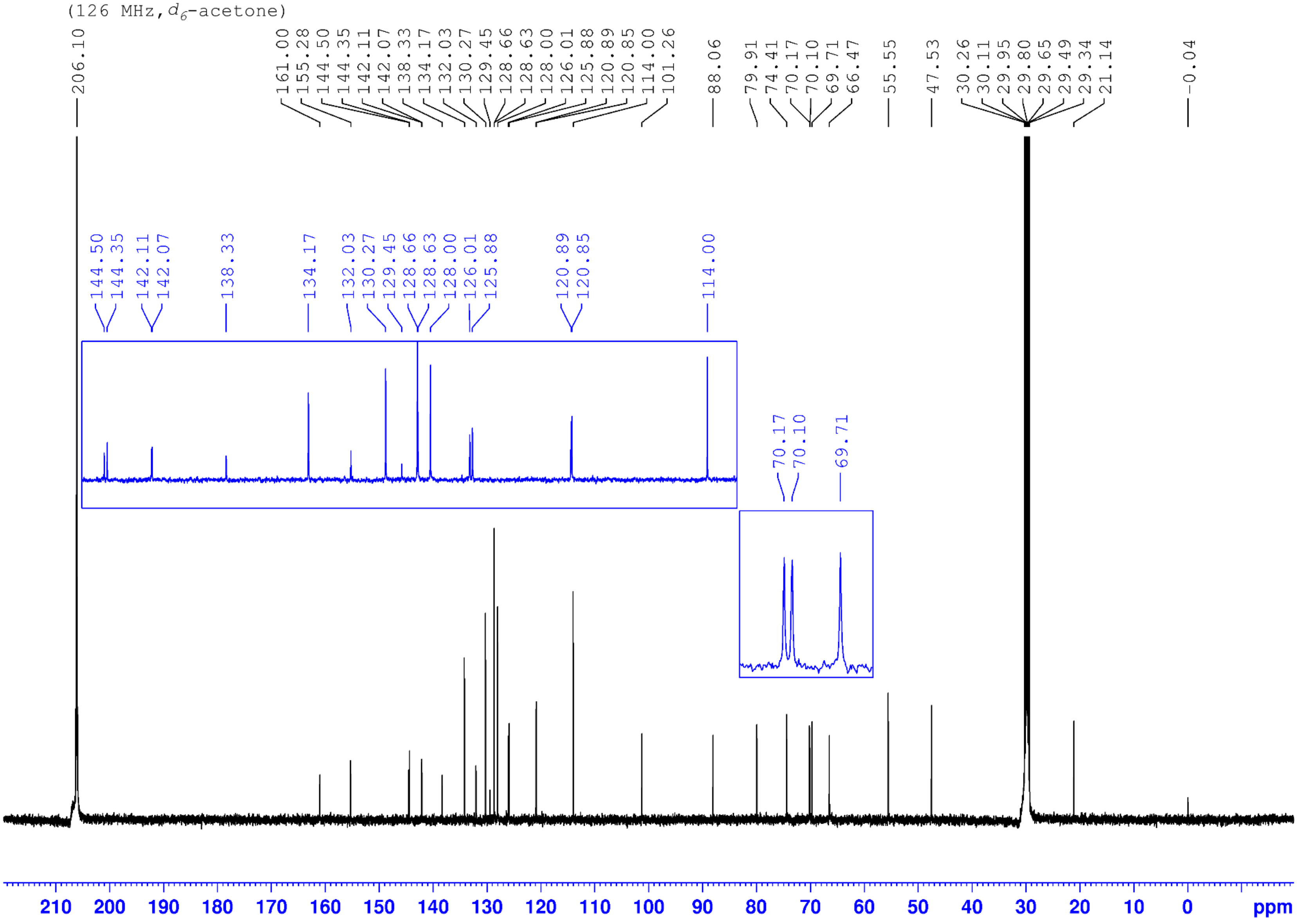
(126 MHz, d_6-acetone)
206.10
161.00
155.28
144.50
144.35
142.11
142.07
138.33
134.17
132.03
130.27
129.45
128.66
128.63
128.00
126.01
125.88
120.89
120.85
114.00
101.26
88.06
79.91
74.41
70.17
70.10
69.71
66.47
55.55
47.53
30.26
30.11
29.95
29.80
29.65
29.49
29.34
21.14
-0.04
ppm

Compound **13**

^{1}H-NMR

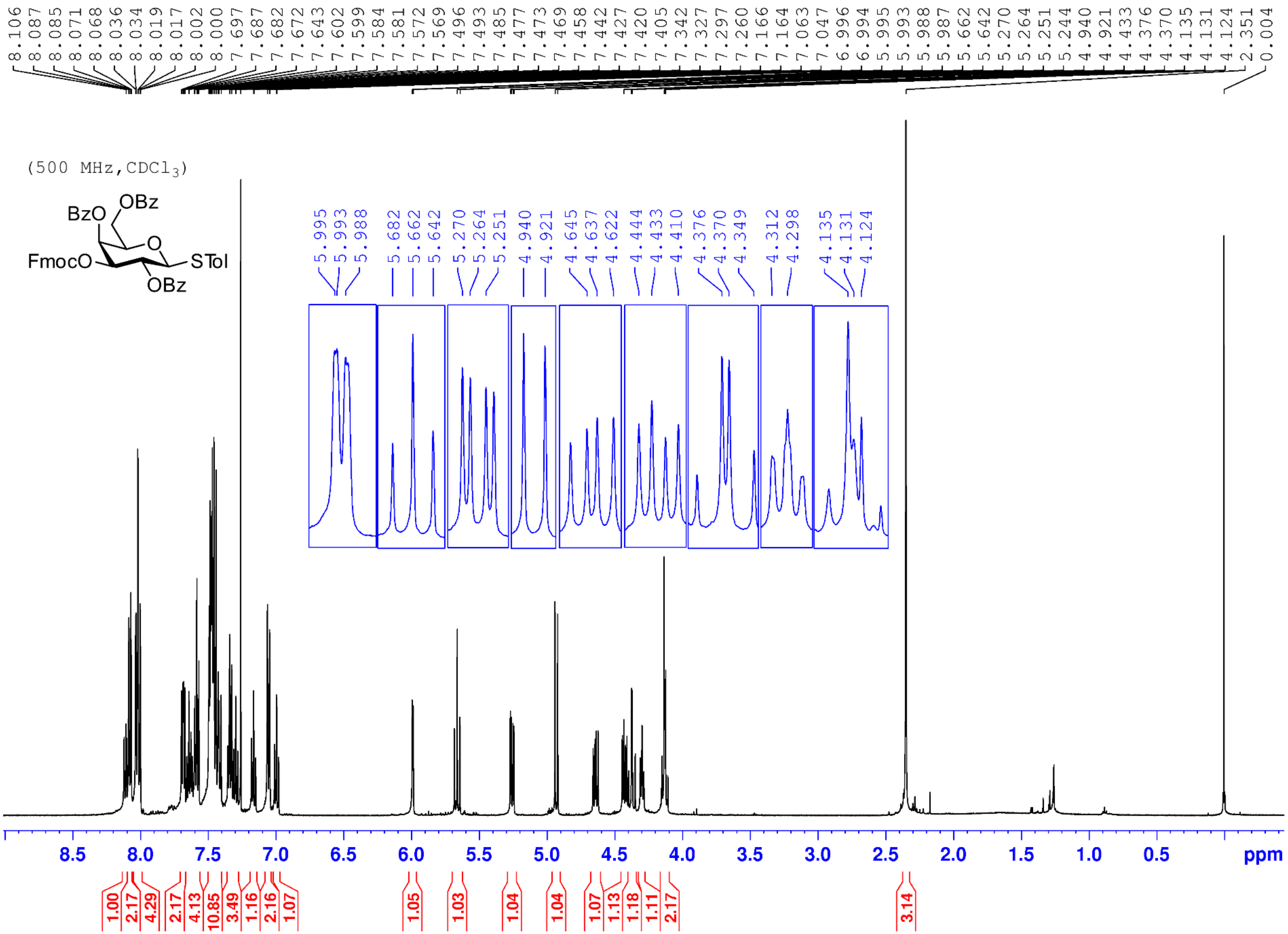

^1H-^1H COSY

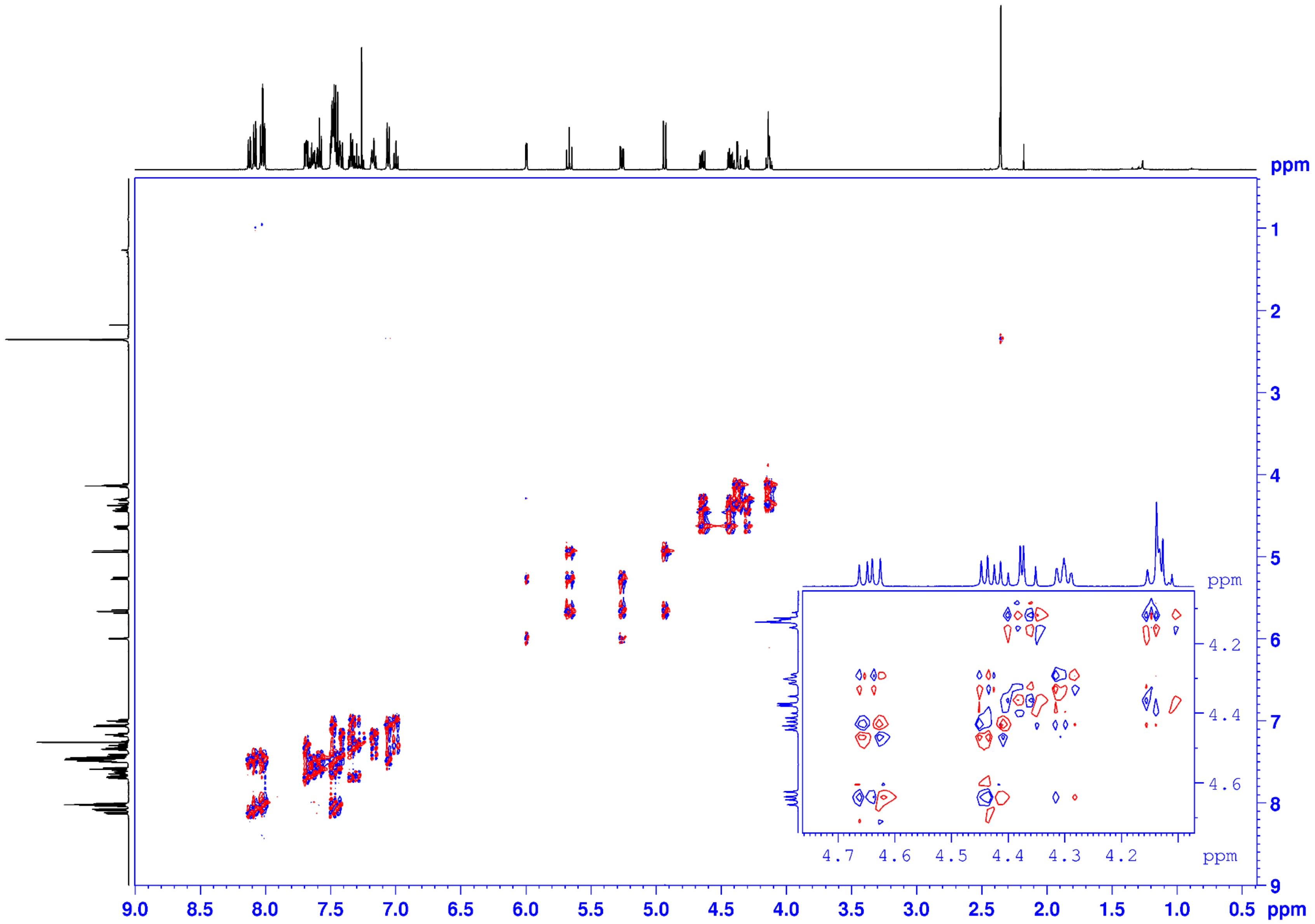
ppm
1
2
3
4
5
6
7
8
9
9.0 8.5 8.0 7.5 7.0 6.5 6.0 5.5 5.0 4.5 4.0 3.5 3.0 2.5 2.0 1.5 1.0 0.5 ppm
4.2
4.4
4.6
4.7 4.6 4.5 4.4 4.3 4.2
ppm

^{1}H-^{13}C HSQC

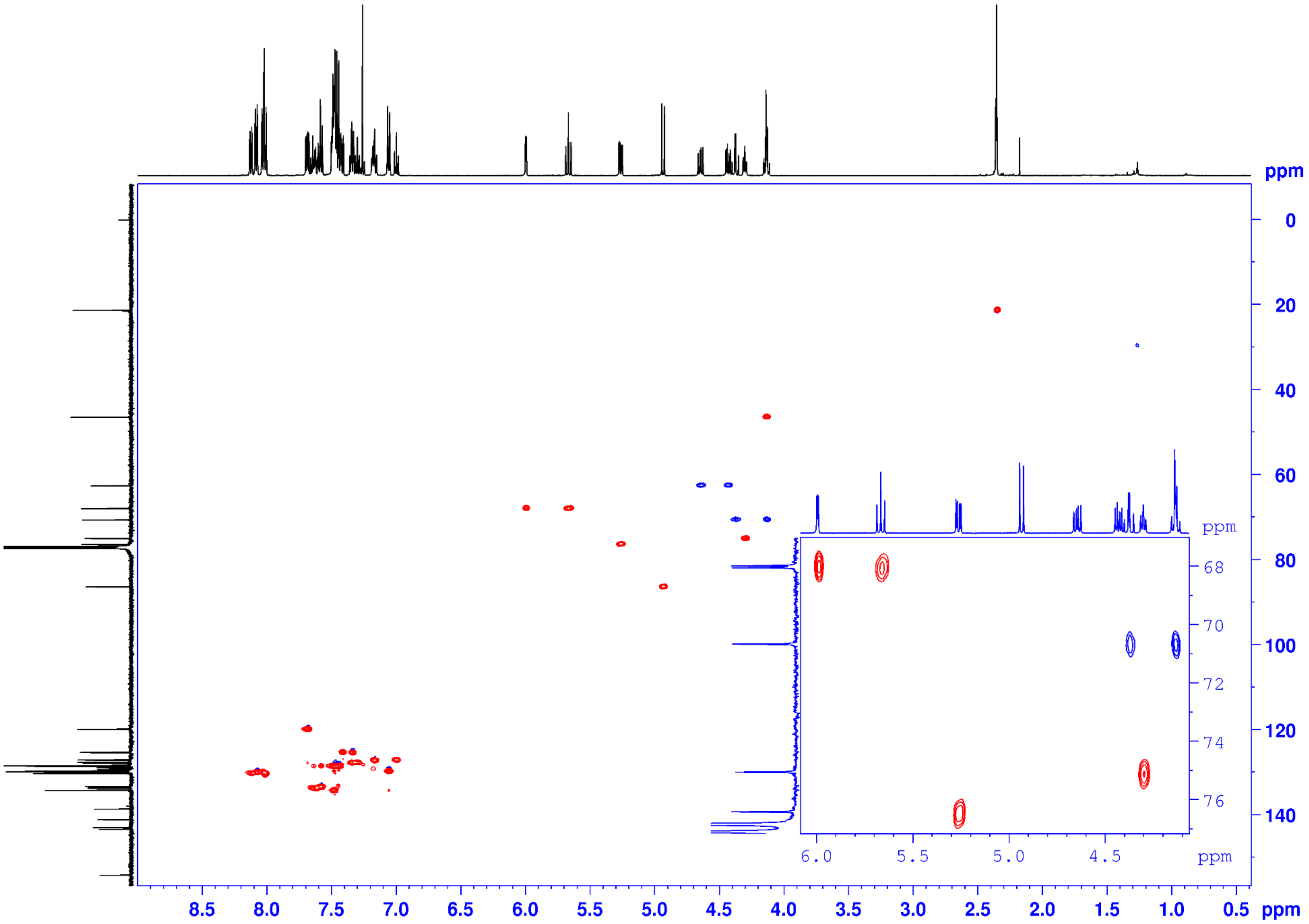

ppm
0
20
40
60
80
100
120
140
8.5
8.0
7.5
7.0
6.5
6.0
5.5
5.0
4.5
4.0
3.5
3.0
2.5
2.0
1.5
1.0
0.5
ppm
68
70
72
74
76
6.0
5.5
5.0
4.5

^{1}H-^{13}C HMBC

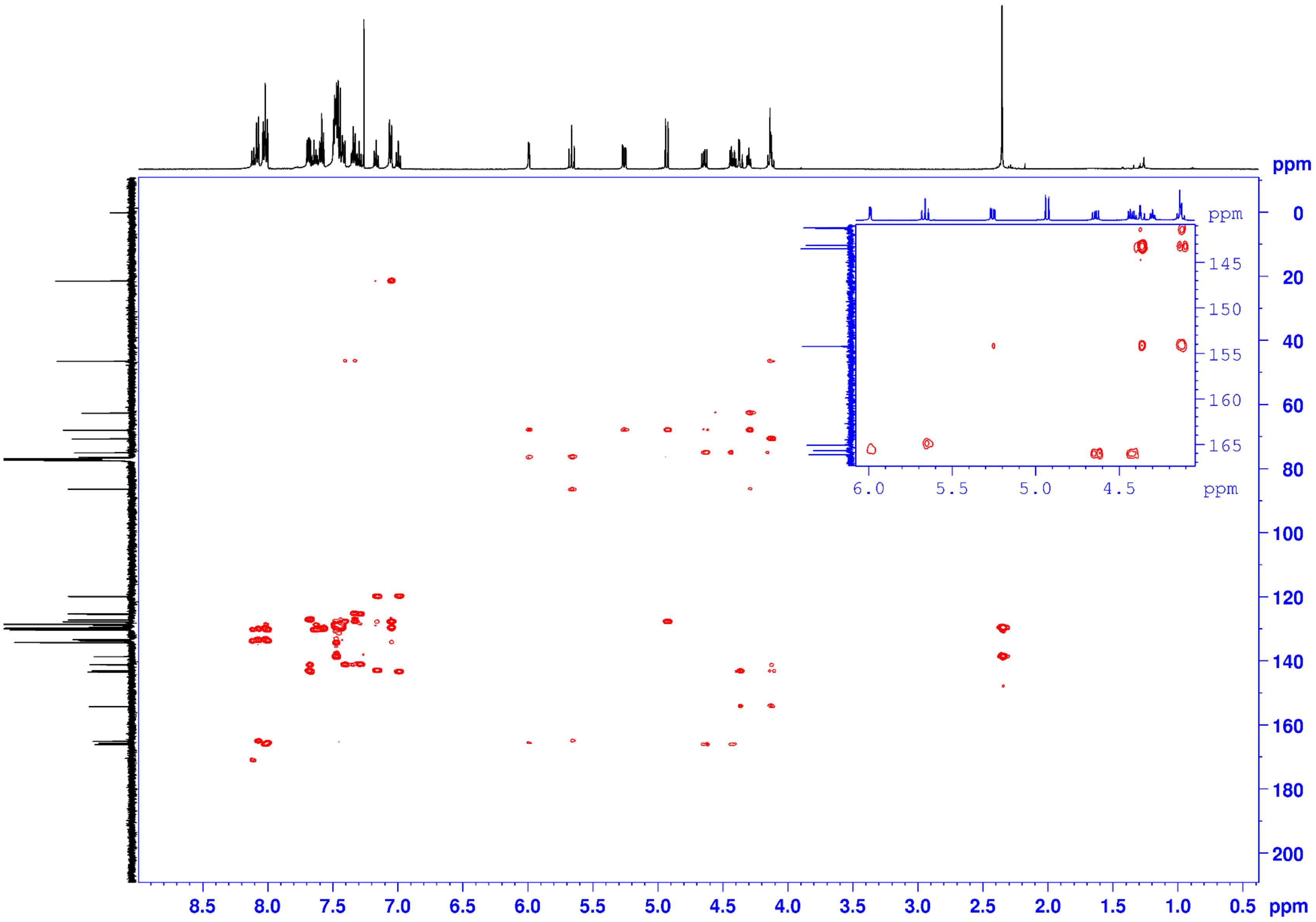
ppm
0
20
40
60
80
100
120
140
160
180
200
8.5
8.0
7.5
7.0
6.5
6.0
5.5
5.0
4.5
4.0
3.5
3.0
2.5
2.0
1.5
1.0
0.5
ppm
145
150
155
160
165
6.0
5.5
5.0
4.5
ppm

$^{13}C\{^1H\}$ NMR

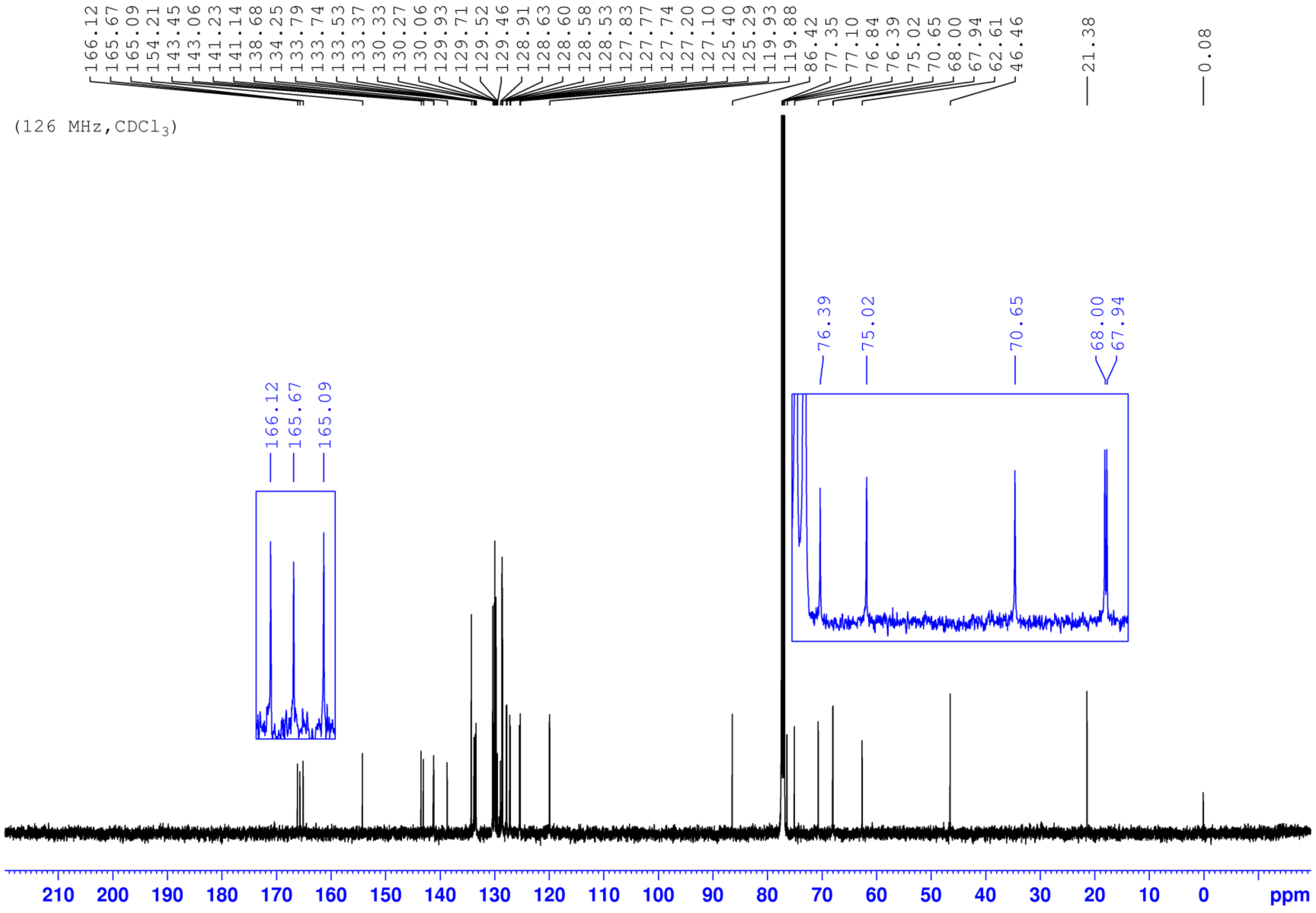
(126 MHz,CDCl3)
166.12
165.67
165.09
154.21
143.45
143.06
141.23
141.14
138.68
134.25
133.79
133.74
133.53
133.37
130.33
130.27
130.06
129.93
129.71
129.52
129.46
128.91
128.63
128.60
128.58
128.53
127.83
127.77
127.74
127.20
127.10
125.40
125.29
119.93
119.88
86.42
77.35
77.10
76.84
76.39
75.02
70.65
68.00
67.94
62.61
46.46
21.38
0.08
166.12
165.67
165.09
76.39
75.02
70.65
68.00
67.94
210 200 190 180 170 160 150 140 130 120 110 100 90 80 70 60 50 40 30 20 10 0 ppm

Compound **14**

^{1}H-NMR

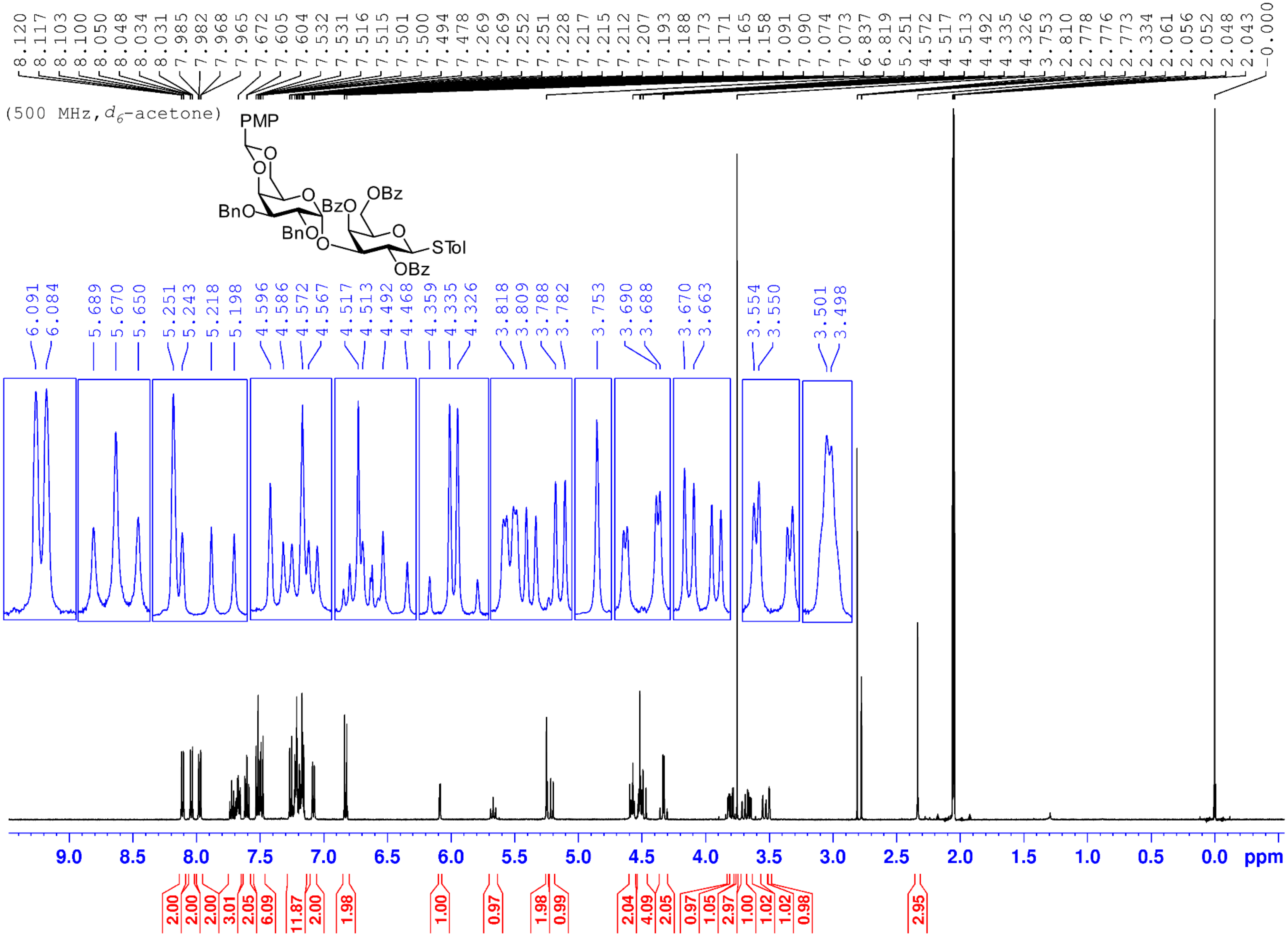

^{1}H-^{1}H COSY

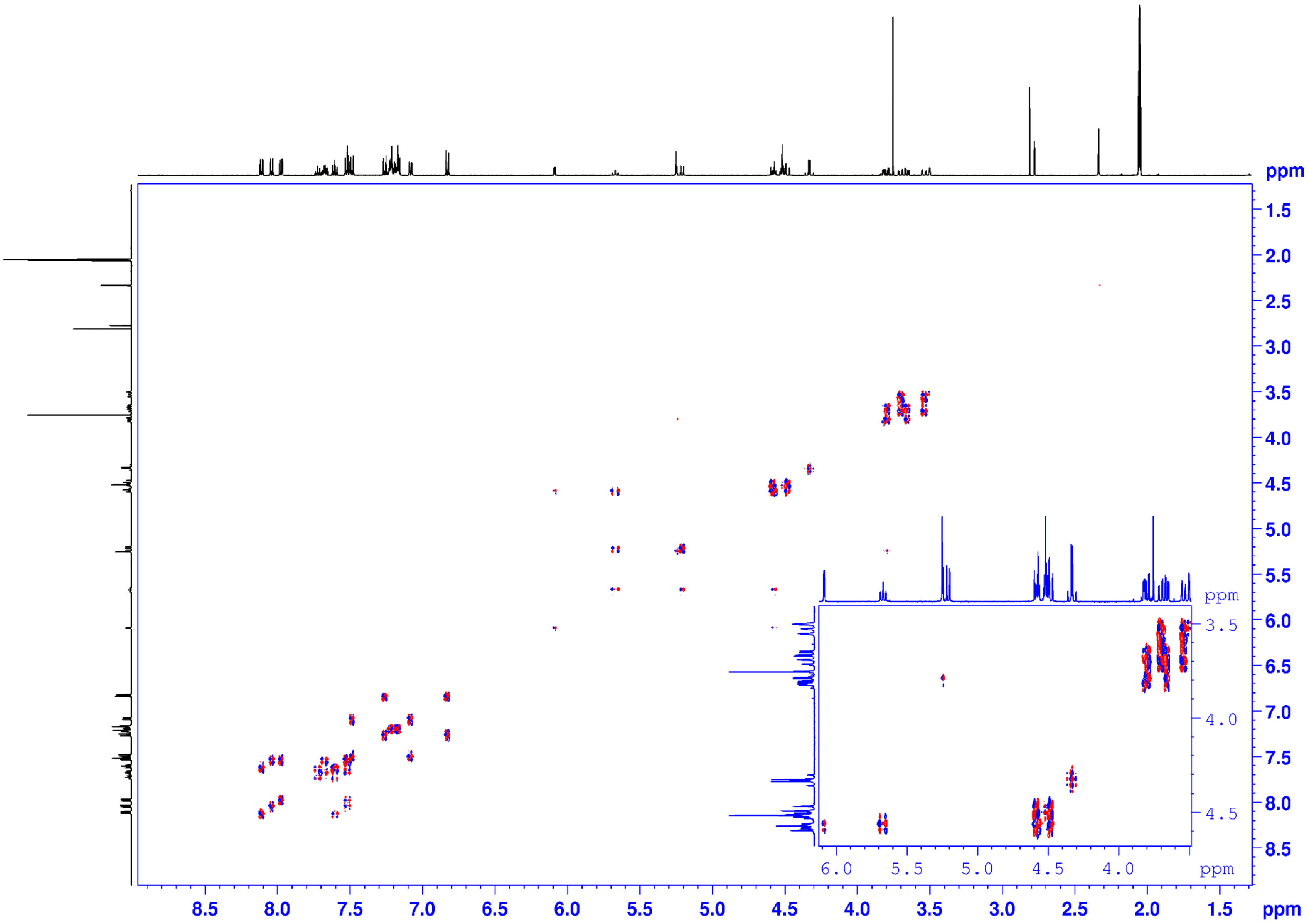

ppm
1.5
2.0
2.5
3.0
3.5
4.0
4.5
5.0
5.5
6.0
6.5
7.0
7.5
8.0
8.5
8.5 8.0 7.5 7.0 6.5 6.0 5.5 5.0 4.5 4.0 3.5 3.0 2.5 2.0 1.5 ppm
6.0 5.5 5.0 4.5 4.0 ppm
3.5 4.0 4.5 ppm

^{1}H-^{13}C HSQC

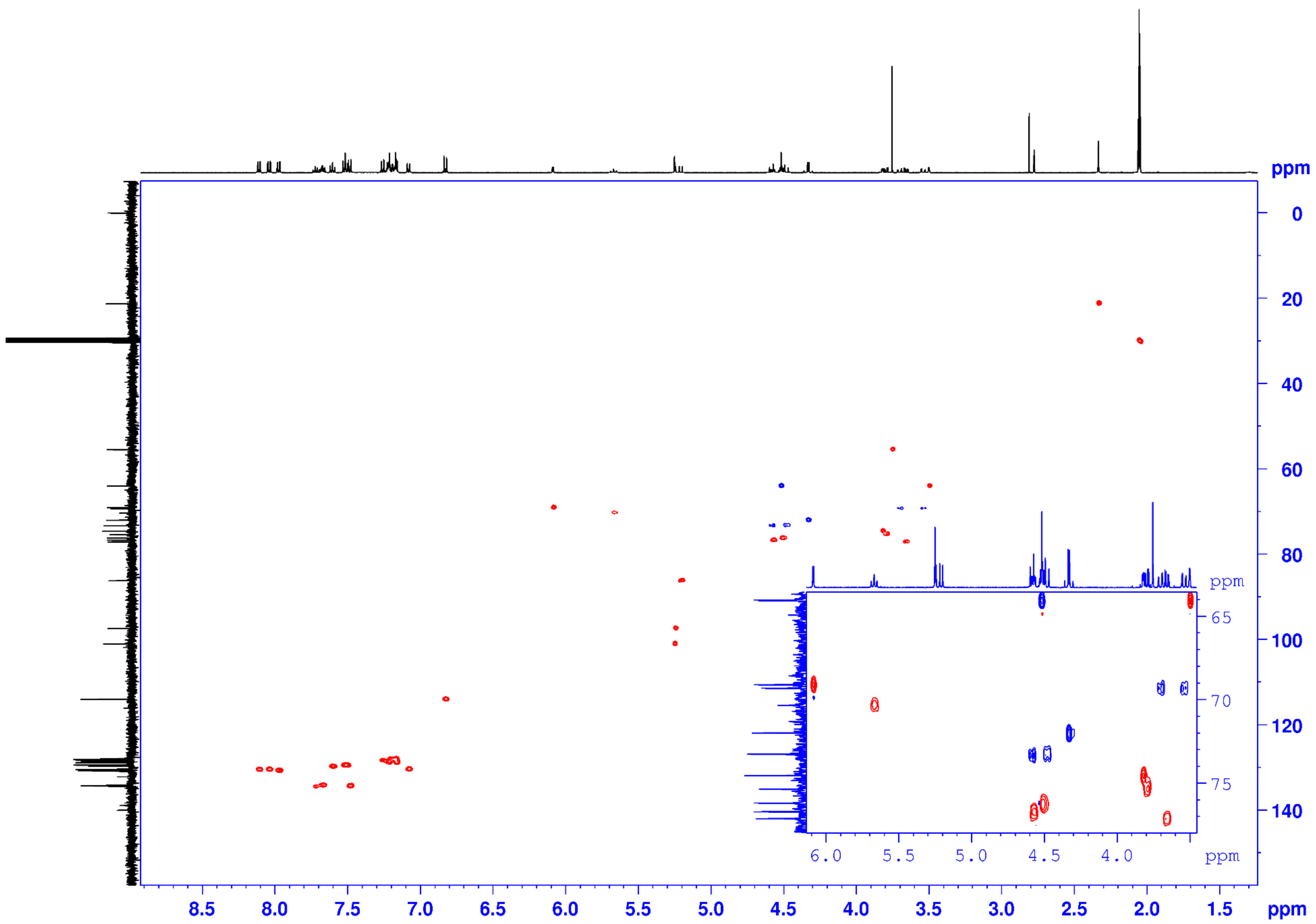

ppm
0
20
40
60
80
100
120
140
8.5
8.0
7.5
7.0
6.5
6.0
5.5
5.0
4.5
4.0
3.5
3.0
2.5
2.0
1.5
ppm
65
70
75
6.0
5.5
5.0
4.5
4.0

^{1}H-^{13}C non-decoupled HSQC

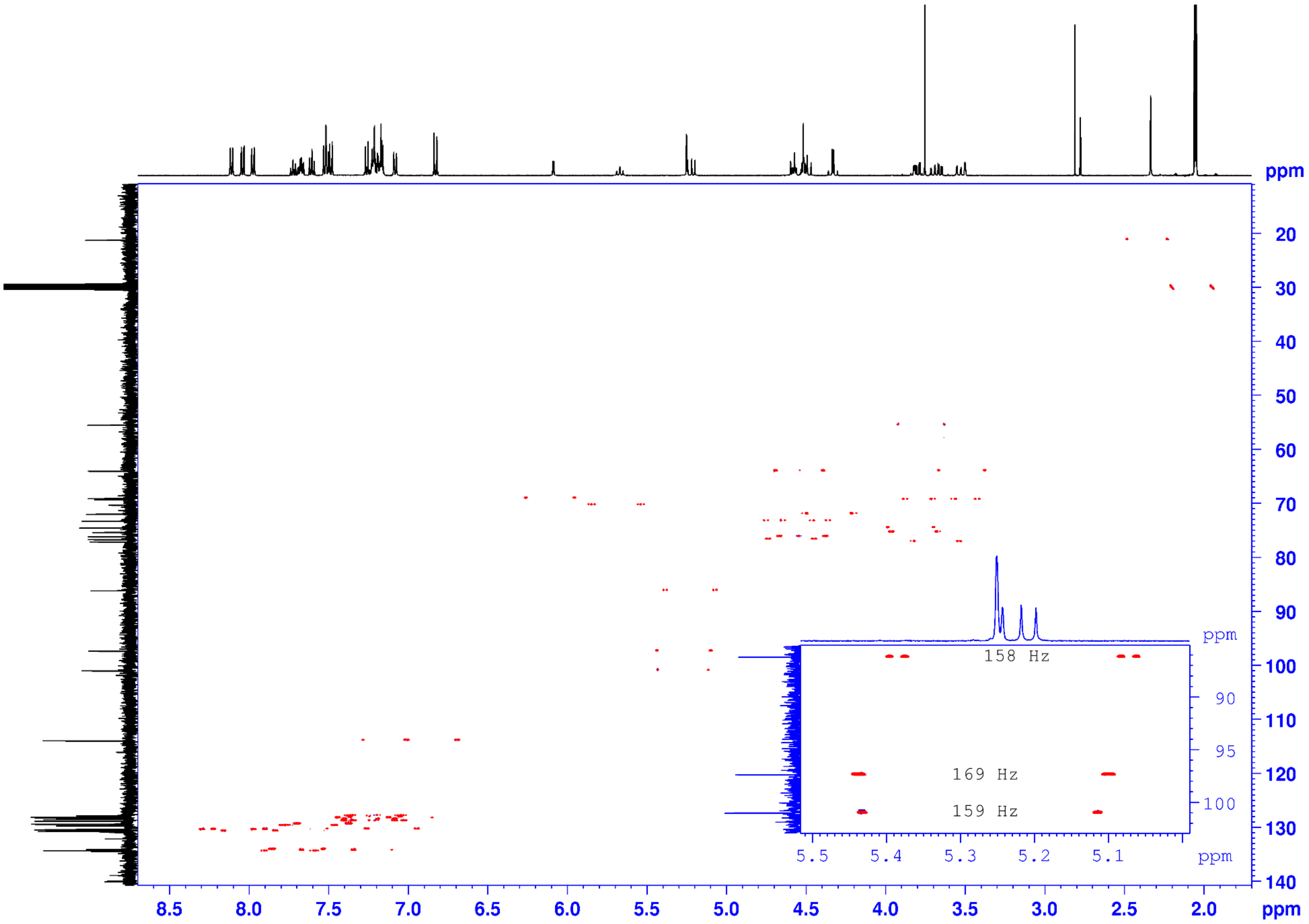

^{1}H-^{13}C HMBC

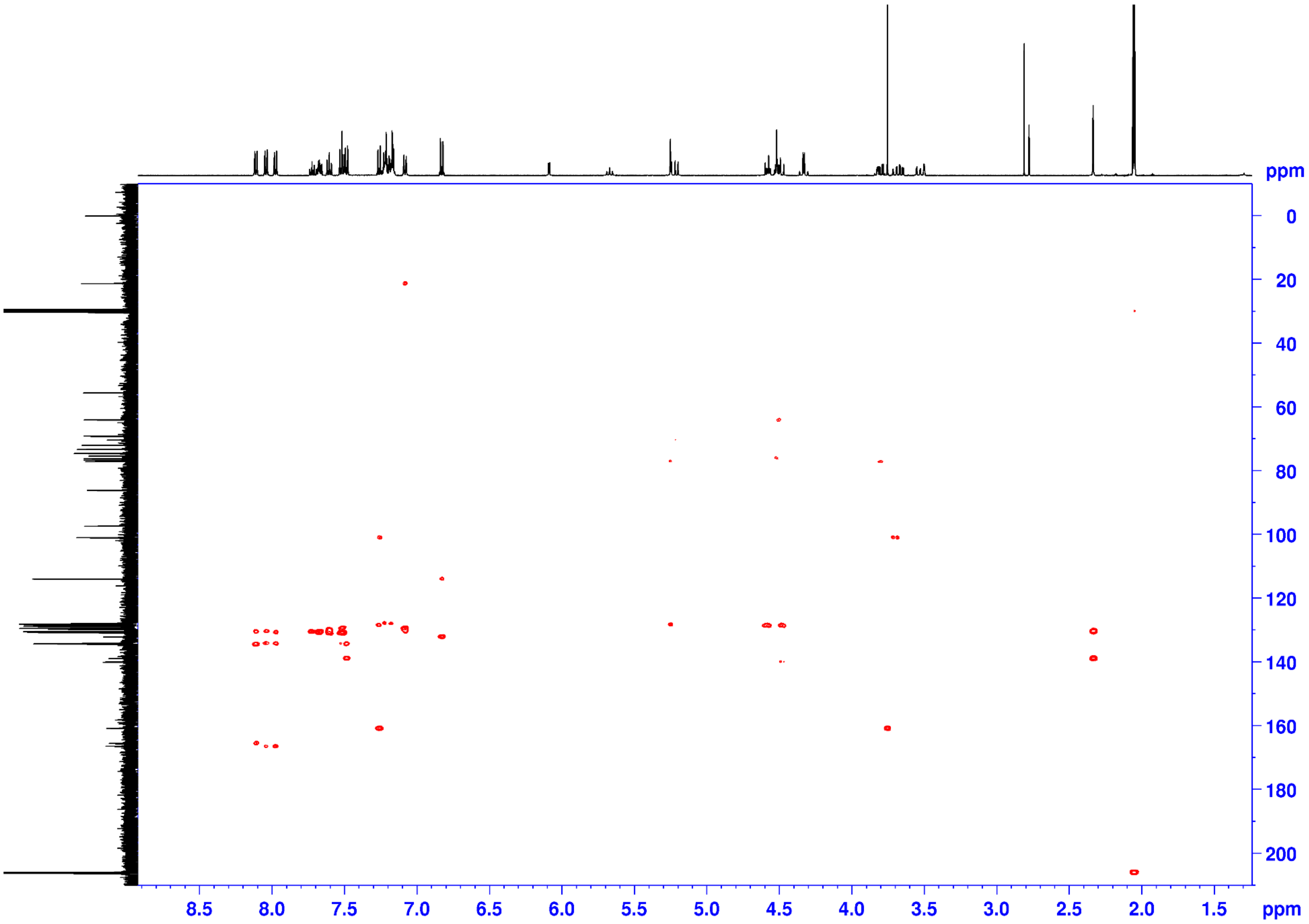

ppm
0
20
40
60
80
100
120
140
160
180
200
8.5
8.0
7.5
7.0
6.5
6.0
5.5
5.0
4.5
4.0
3.5
3.0
2.5
2.0
1.5
ppm

$^{13}C\{^{1}H\}$ NMR

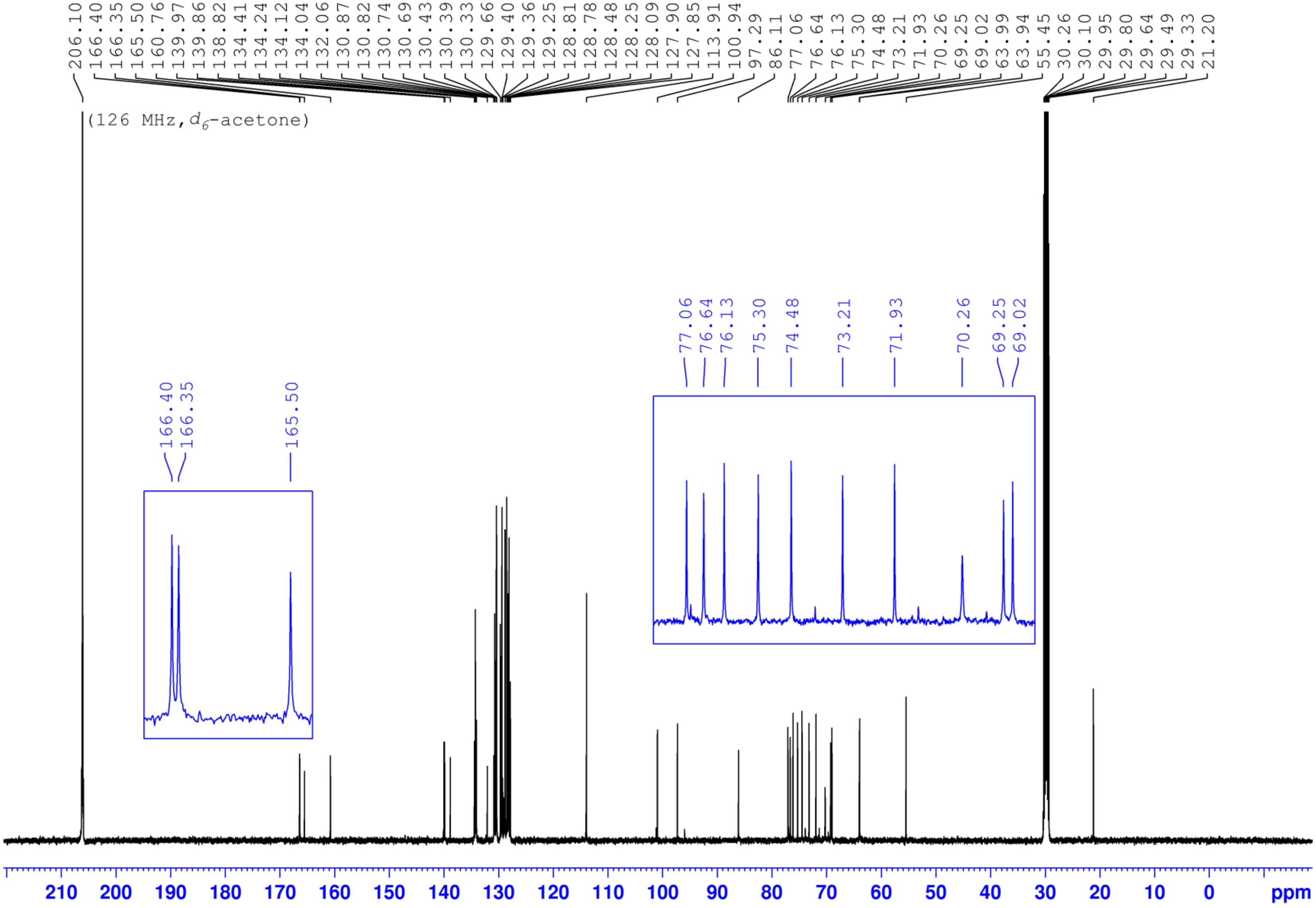
206.10
166.40
166.35
165.50
160.76
139.97
139.86
138.82
134.41
134.24
134.12
134.04
132.06
130.87
130.82
130.74
130.69
130.43
130.39
130.33
129.66
129.40
129.36
129.25
128.81
128.78
128.48
128.25
128.09
127.90
127.85
113.91
100.94
97.29
86.11
77.06
76.64
76.13
75.30
74.48
73.21
71.93
70.26
69.25
69.02
63.99
63.94
55.45
30.26
30.10
29.95
29.80
29.64
29.49
29.33
21.20
(126 MHz, d6-acetone)
210 200 190 180 170 160 150 140 130 120 110 100 90 80 70 60 50 40 30 20 10 0 ppm

Compound **15**

^{1}H-NMR

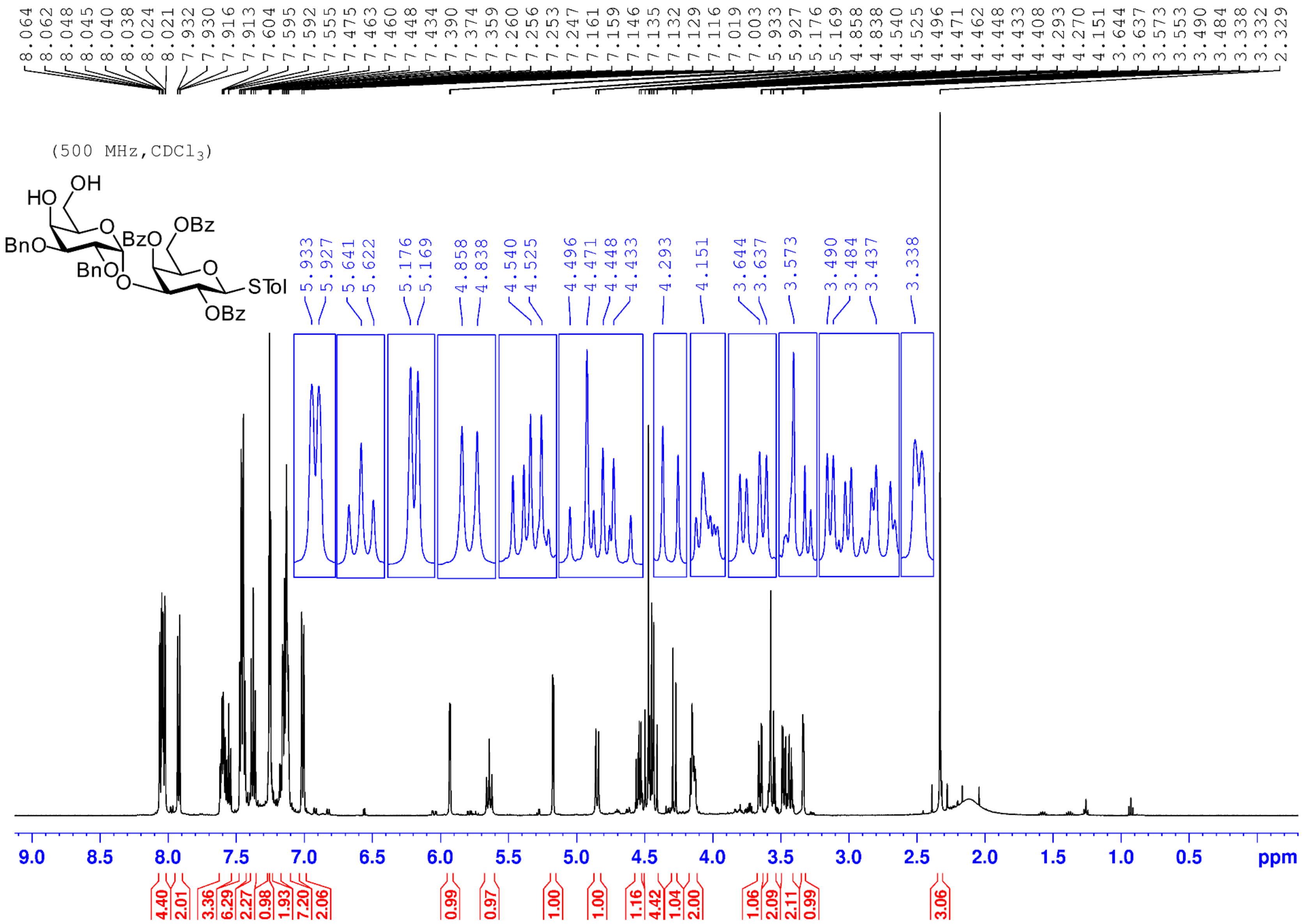

^{1}H-^{1}H COSY

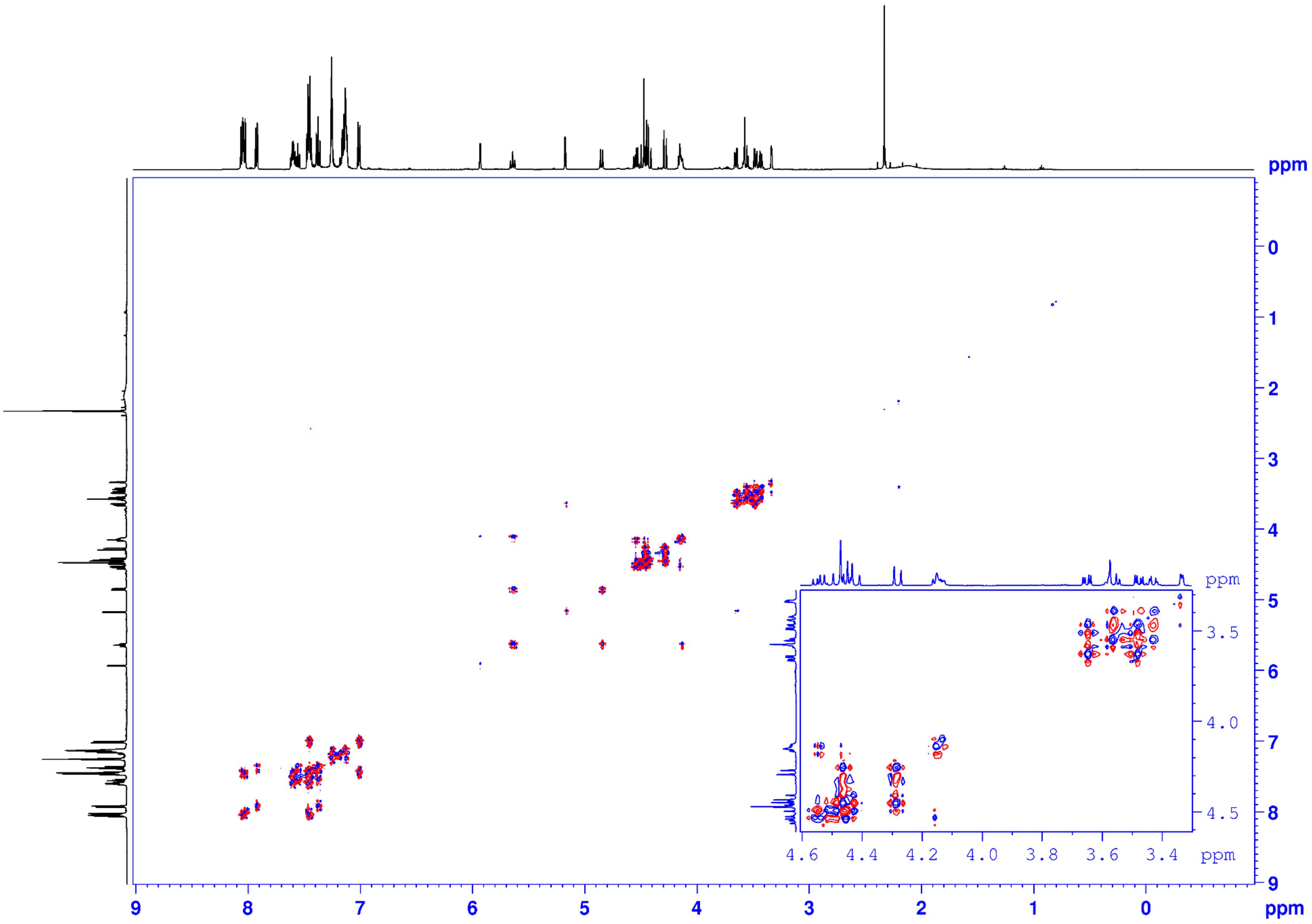

ppm
0
1
2
3
4
5
6
7
8
9
ppm
9
8
7
6
5
4
3
2
1
0
ppm
ppm
3.5
4.0
4.5
4.6
4.4
4.2
4.0
3.8
3.6
3.4
ppm

^{1}H-^{13}C HSQC

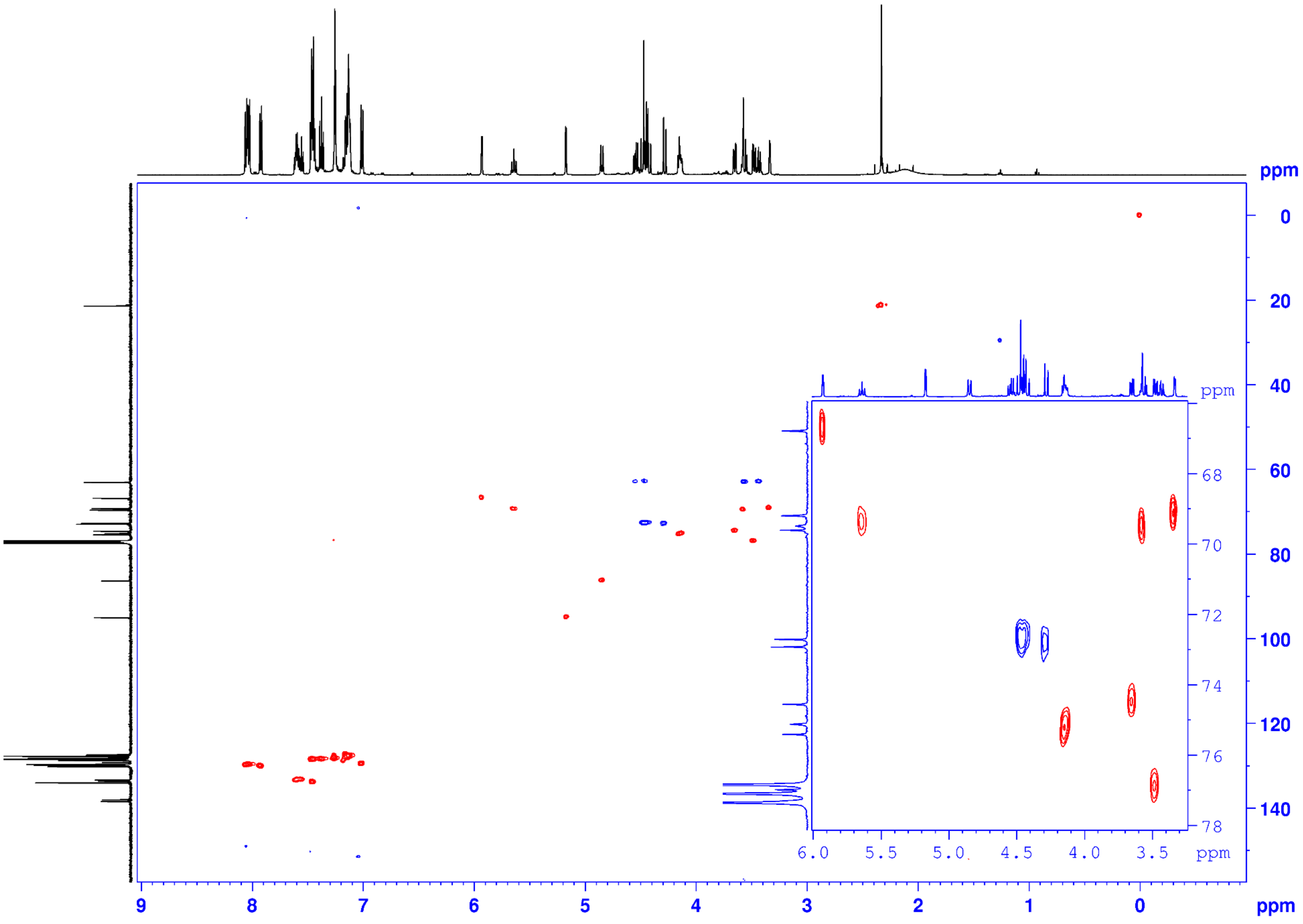

ppm
0
20
40
60
80
100
120
140
9
8
7
6
5
4
3
2
1
0
ppm
6.0
5.5
5.0
4.5
4.0
3.5
ppm
68
70
72
74
76
78

$^{1}H-^{13}C$ HMBC

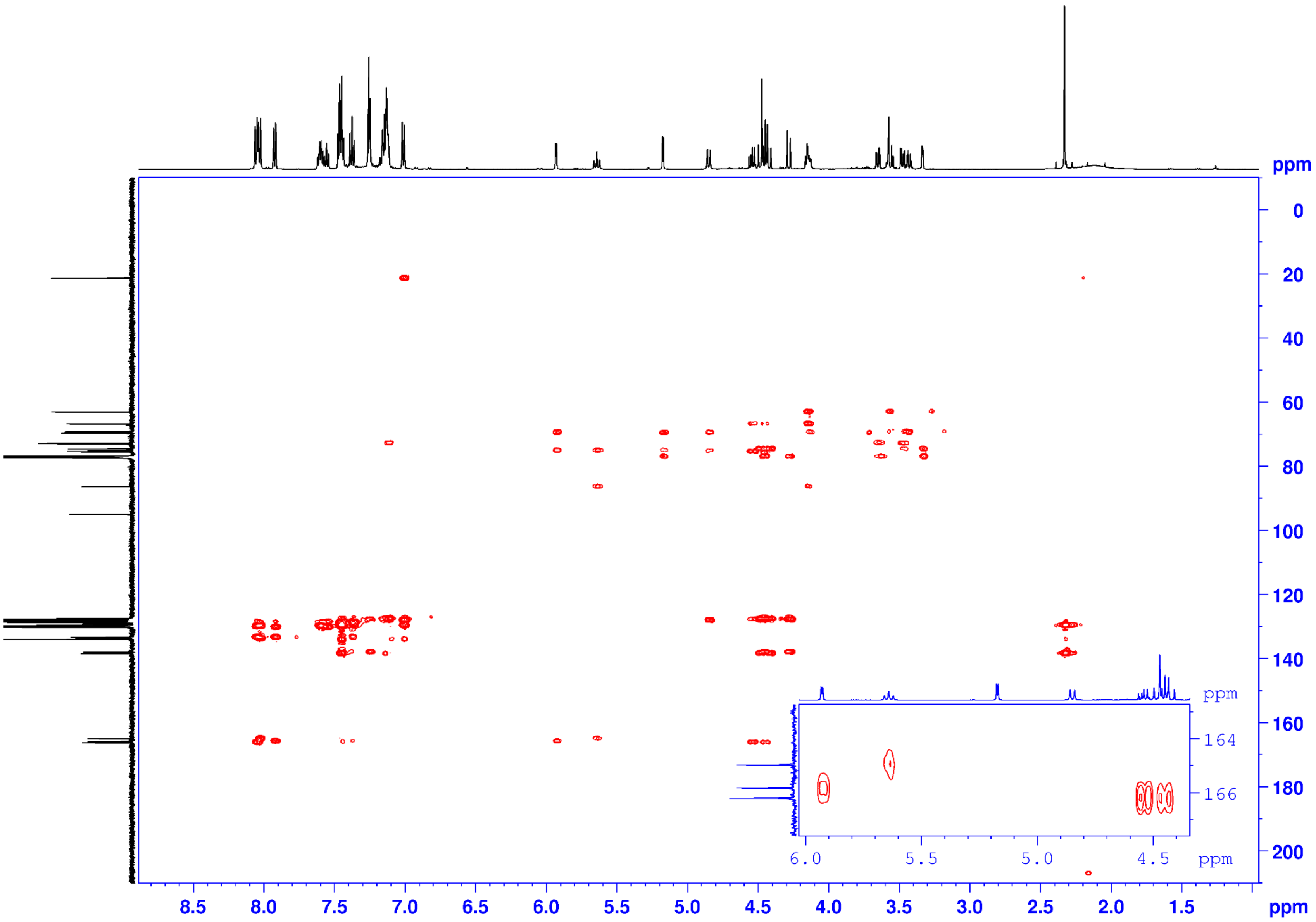

ppm
0
20
40
60
80
100
120
140
160
180
200
8.5
8.0
7.5
7.0
6.5
6.0
5.5
5.0
4.5
4.0
3.5
3.0
2.5
2.0
1.5
ppm
164
166
6.0
5.5
5.0
4.5

$^{13}C\{^1H\}$ NMR

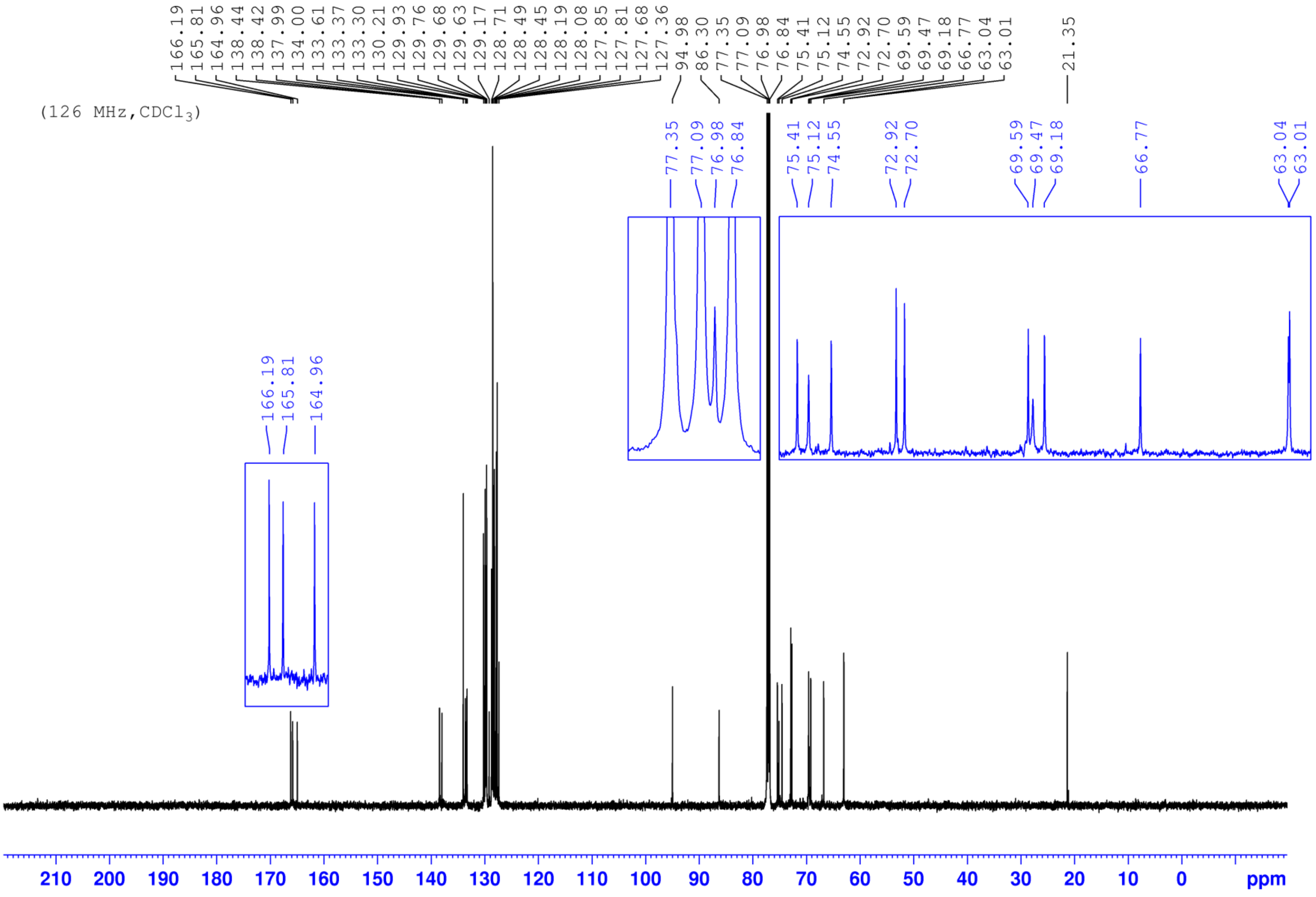

(126 MHz, CDCl3)
166.19
165.81
164.96
138.44
138.42
137.99
134.00
133.61
133.37
133.30
130.21
129.93
129.76
129.68
129.63
129.17
128.71
128.49
128.45
128.19
128.08
127.85
127.81
127.68
127.36
94.98
86.30
77.35
77.09
76.98
76.84
75.41
75.12
74.55
72.92
72.70
69.59
69.47
69.18
66.77
63.04
63.01
21.35
210 200 190 180 170 160 150 140 130 120 110 100 90 80 70 60 50 40 30 20 10 0 ppm

Compound **16**

^{1}H-NMR

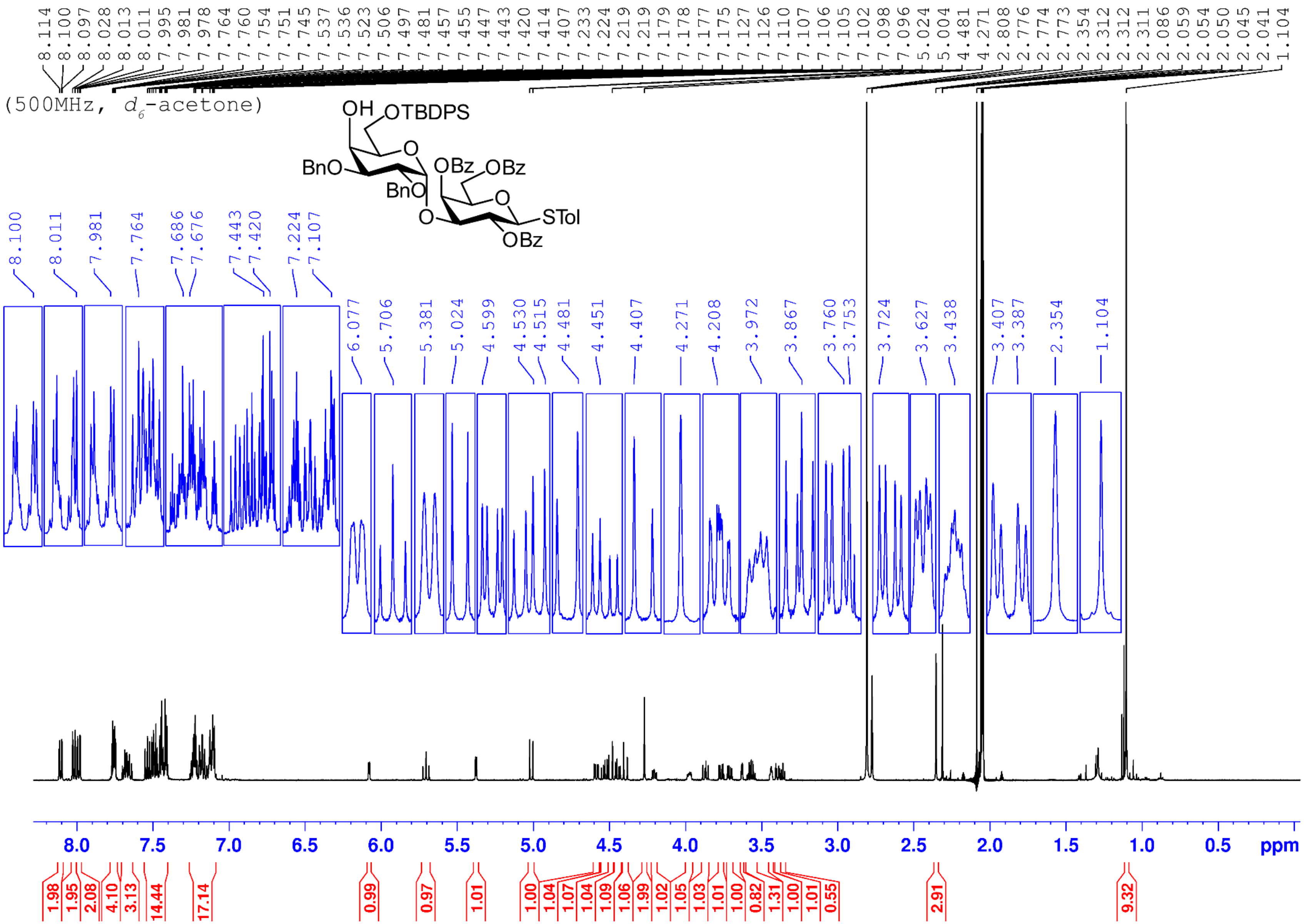

^{1}H-^{1}H COSY

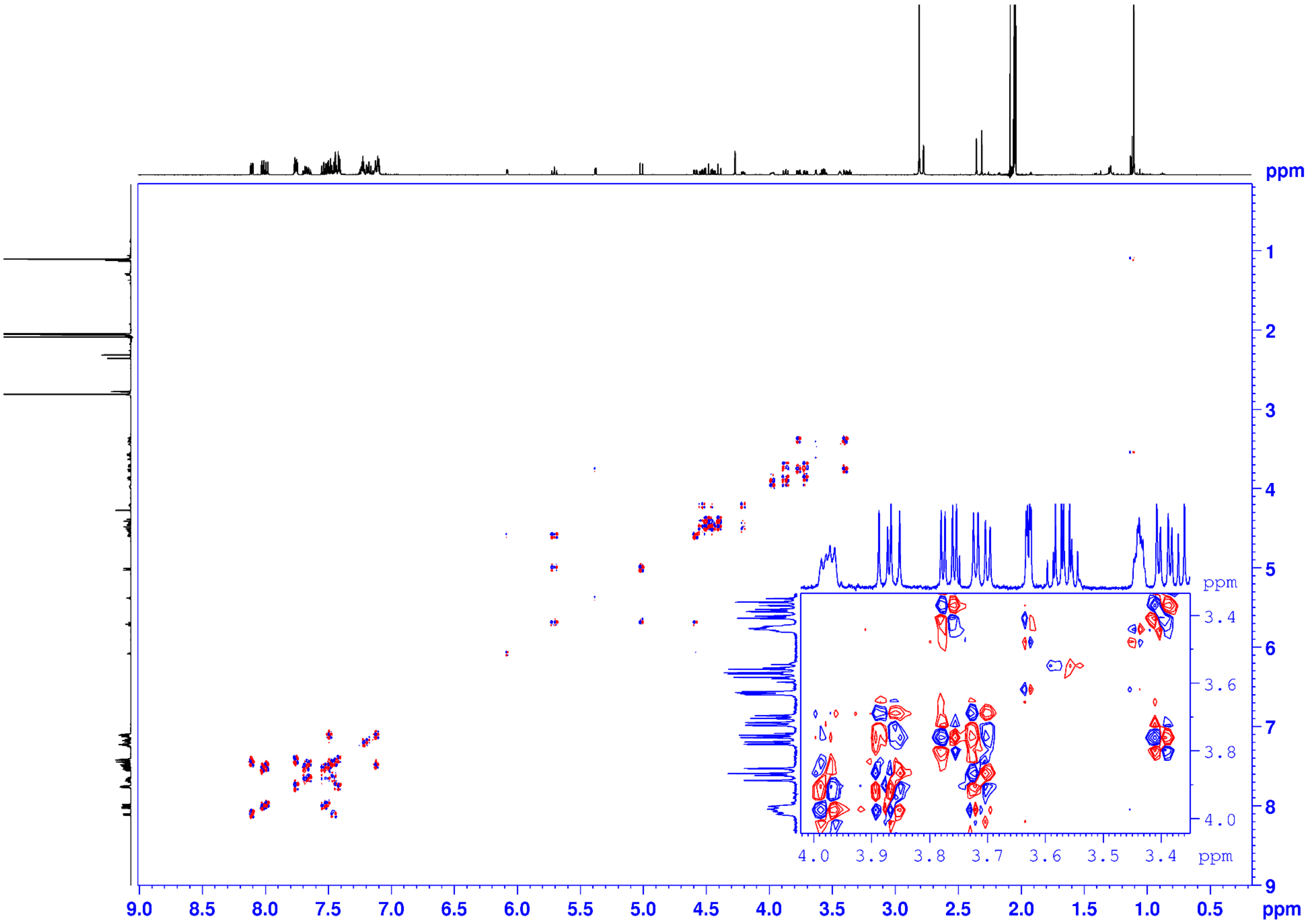
ppm
1
2
3
4
5
6
7
8
9
9.0
8.5
8.0
7.5
7.0
6.5
6.0
5.5
5.0
4.5
4.0
3.5
3.0
2.5
2.0
1.5
1.0
0.5
ppm
3.4
3.6
3.8
4.0
4.0
3.9
3.8
3.7
3.6
3.5
3.4
ppm

^{1}H-^{13}C HSQC

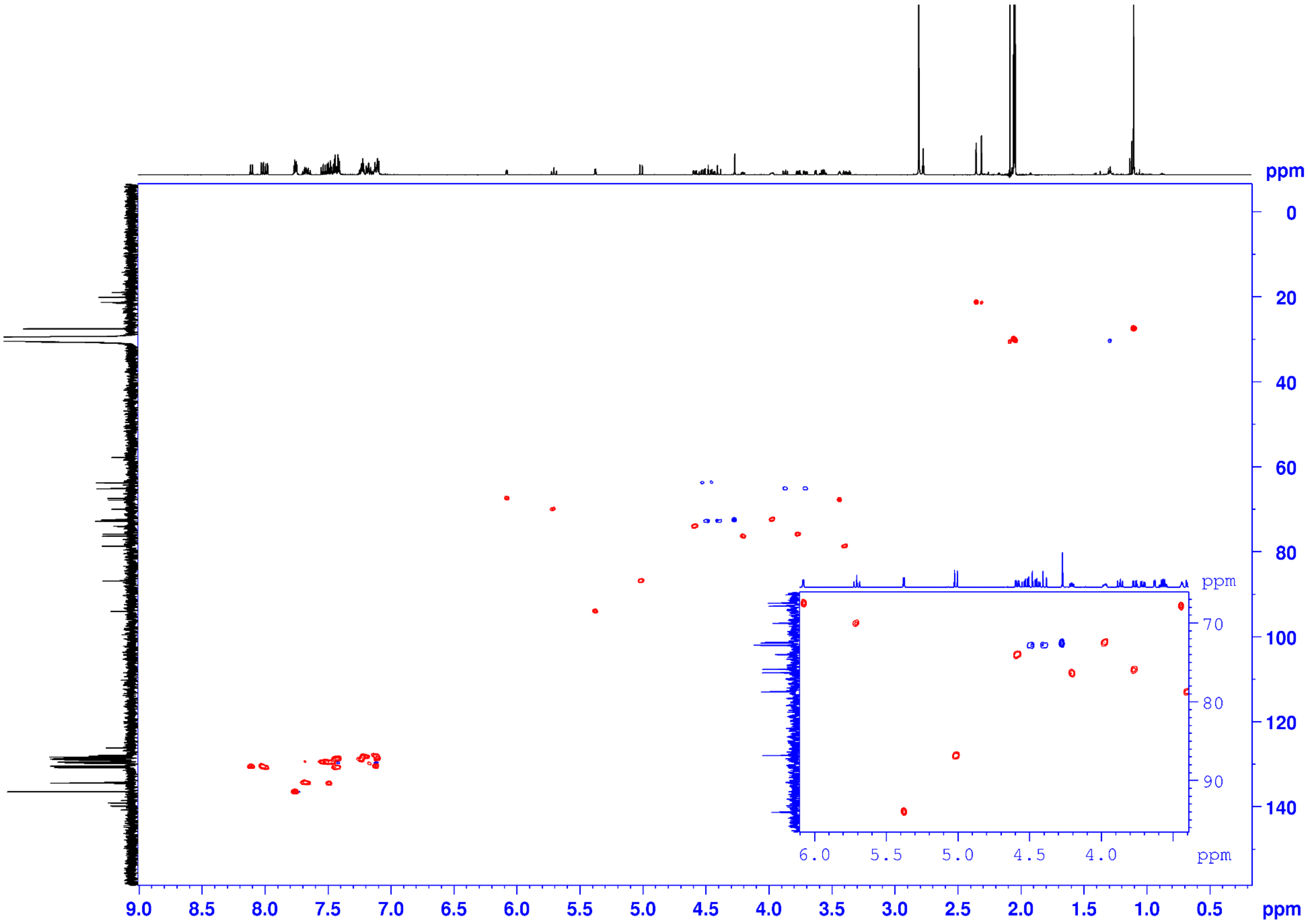

ppm
0
20
40
60
80
100
120
140
9.0
8.5
8.0
7.5
7.0
6.5
6.0
5.5
5.0
4.5
4.0
3.5
3.0
2.5
2.0
1.5
1.0
0.5
ppm
70
80
90
6.0
5.5
5.0
4.5
4.0
ppm

$^{13}C\{^{1}H\}$ NMR

(126MHz, d_6-acetone)

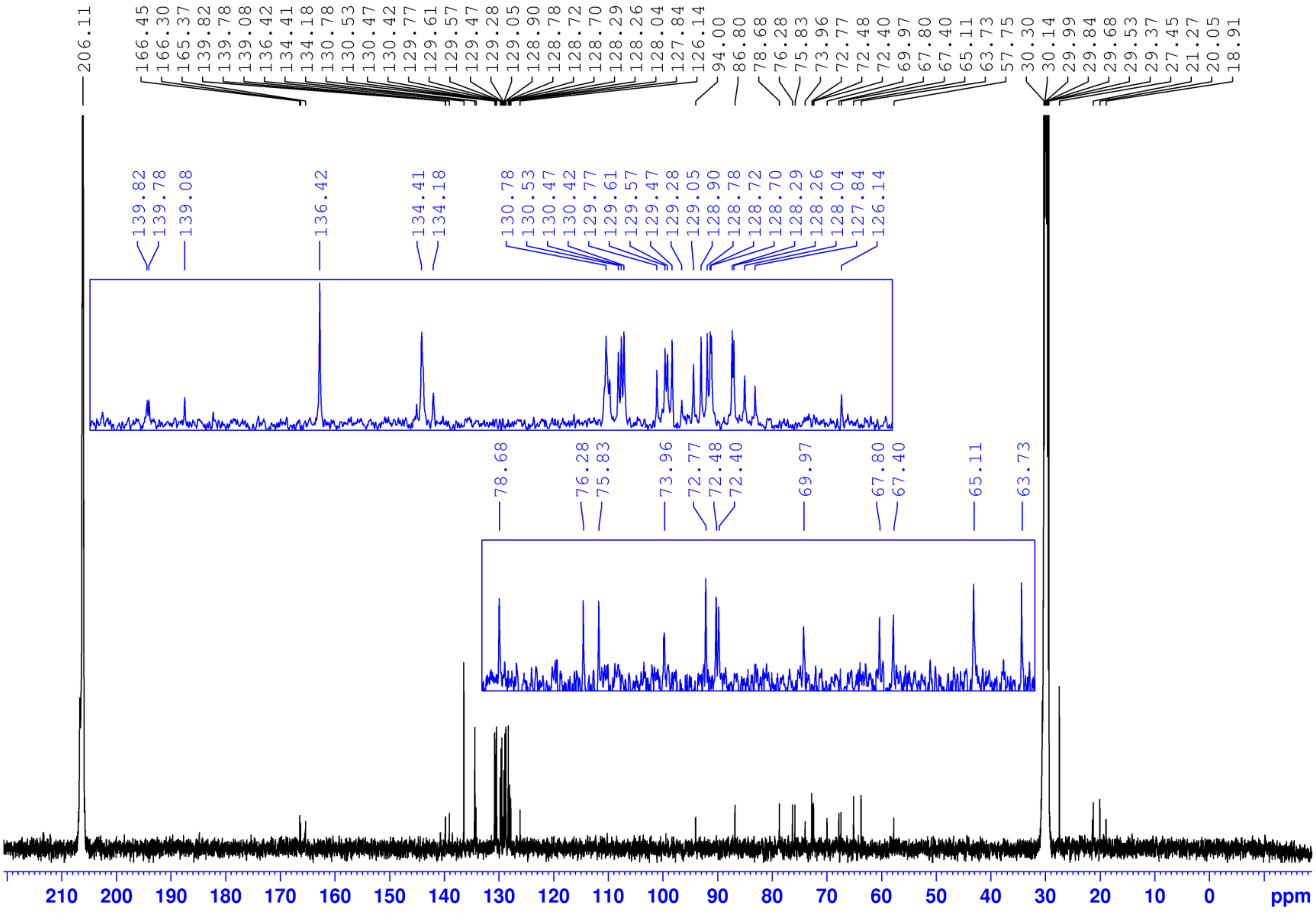

Compound **17**

^{1}H-NMR

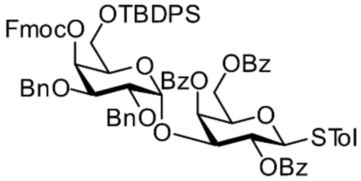

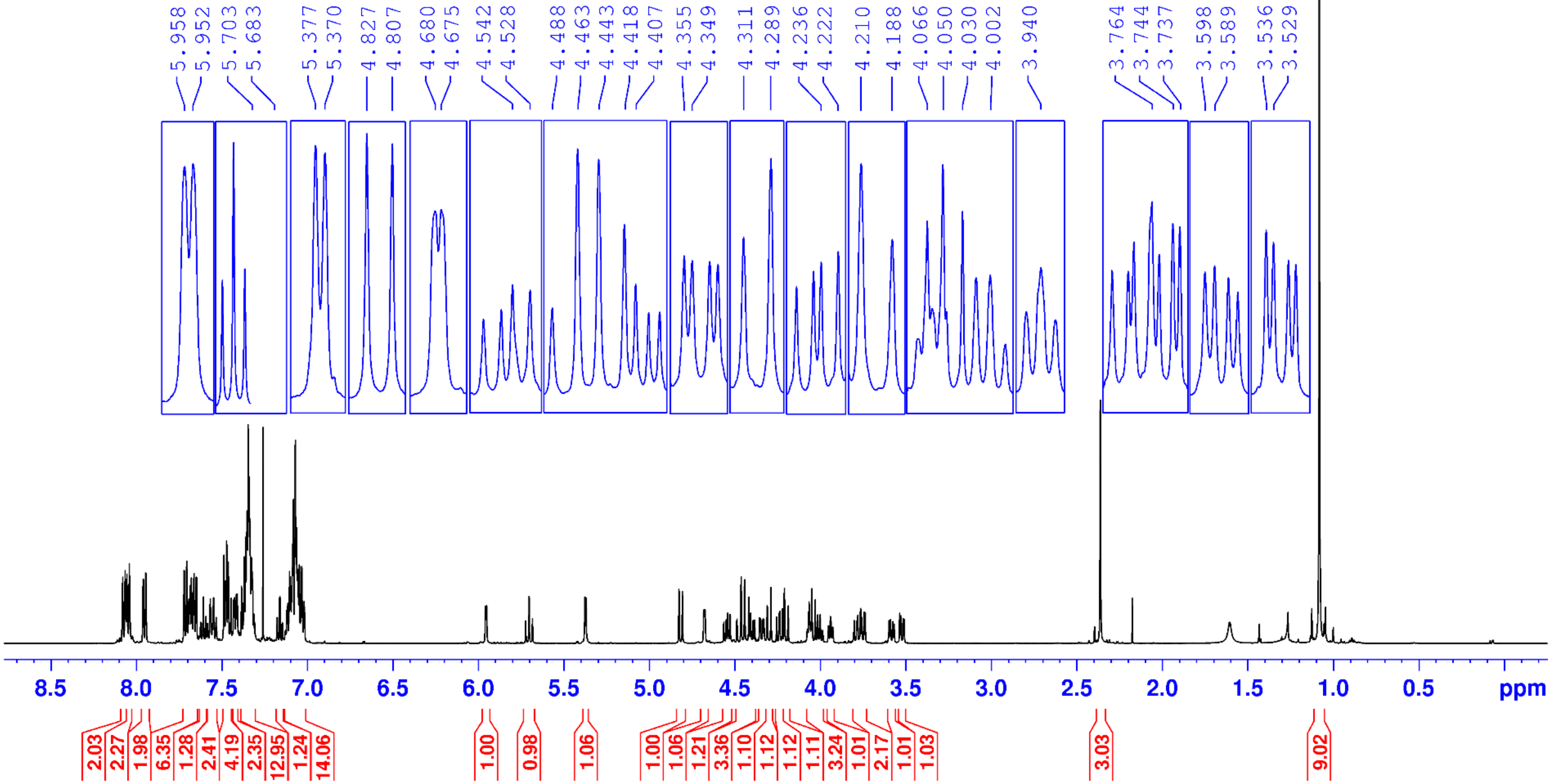

^{1}H-^{1}H COSY

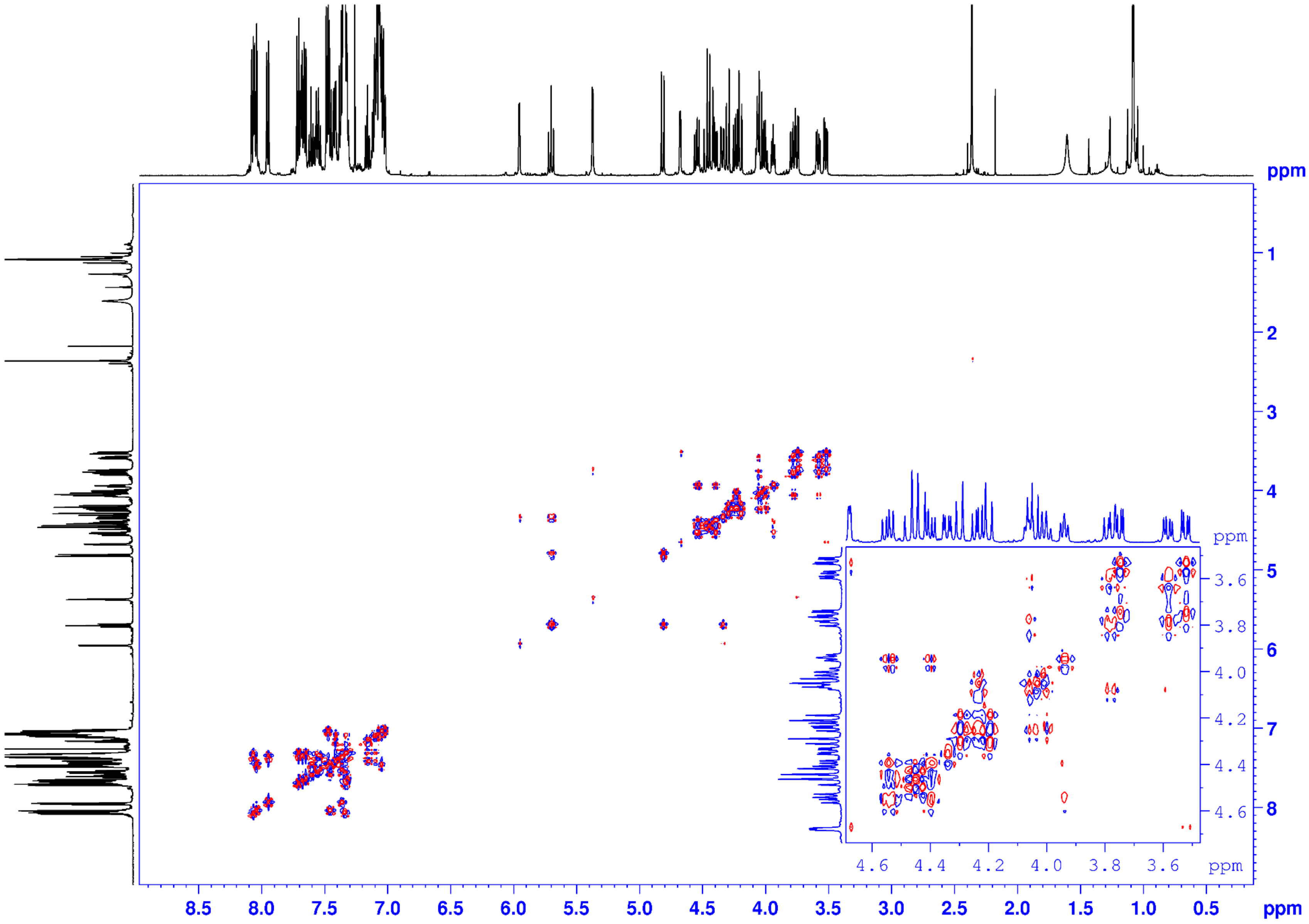
ppm
1
2
3
4
5
6
7
8
8.5
8.0
7.5
7.0
6.5
6.0
5.5
5.0
4.5
4.0
3.5
3.0
2.5
2.0
1.5
1.0
0.5
ppm
4.6
4.4
4.2
4.0
3.8
3.6
ppm

^1H-^{13}C HSQC

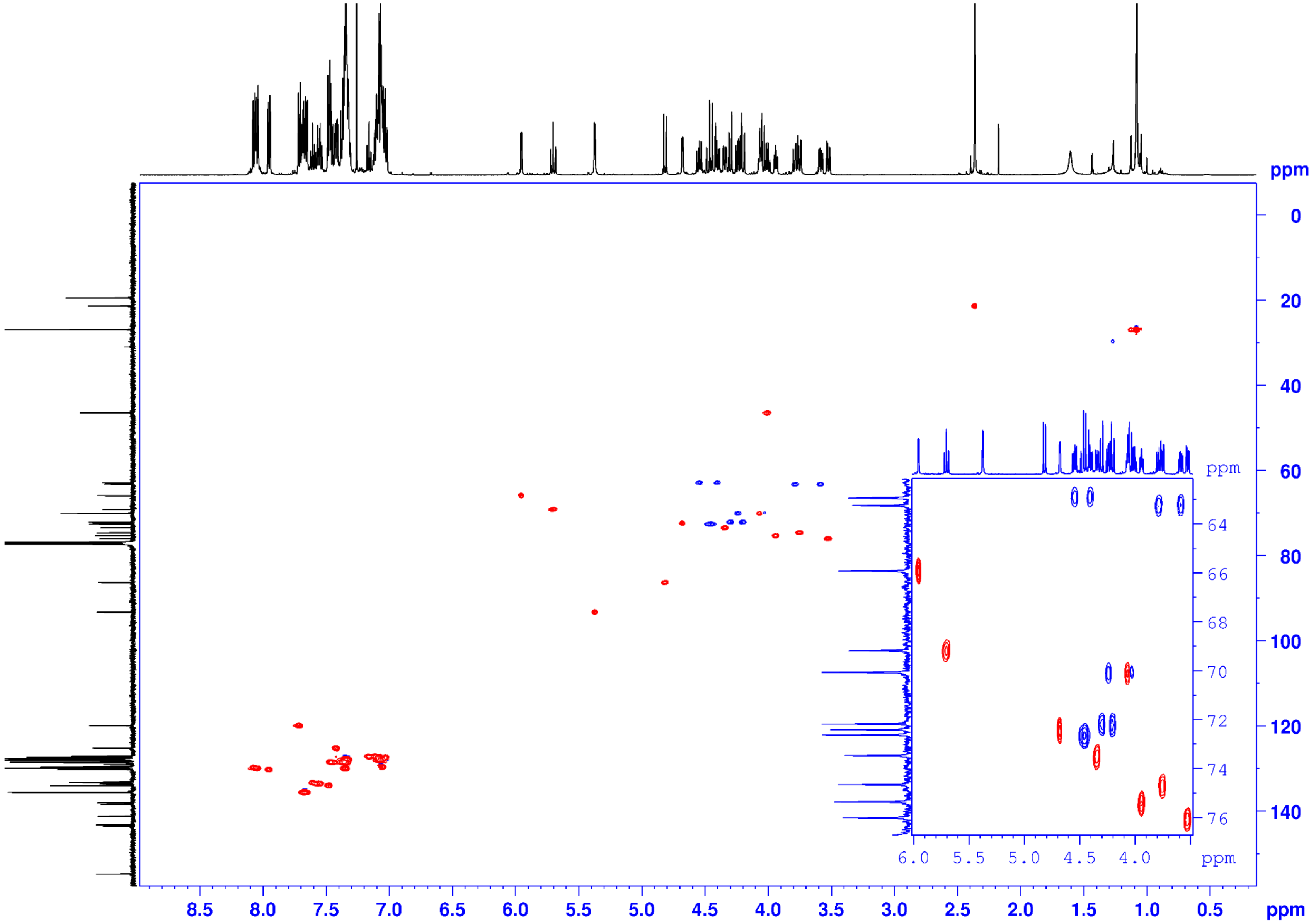

ppm
0
20
40
60
80
100
120
140
8.5 8.0 7.5 7.0 6.5 6.0 5.5 5.0 4.5 4.0 3.5 3.0 2.5 2.0 1.5 1.0 0.5
ppm
64
66
68
70
72
74
76
6.0 5.5 5.0 4.5 4.0

^{1}H-^{13}C HMBC

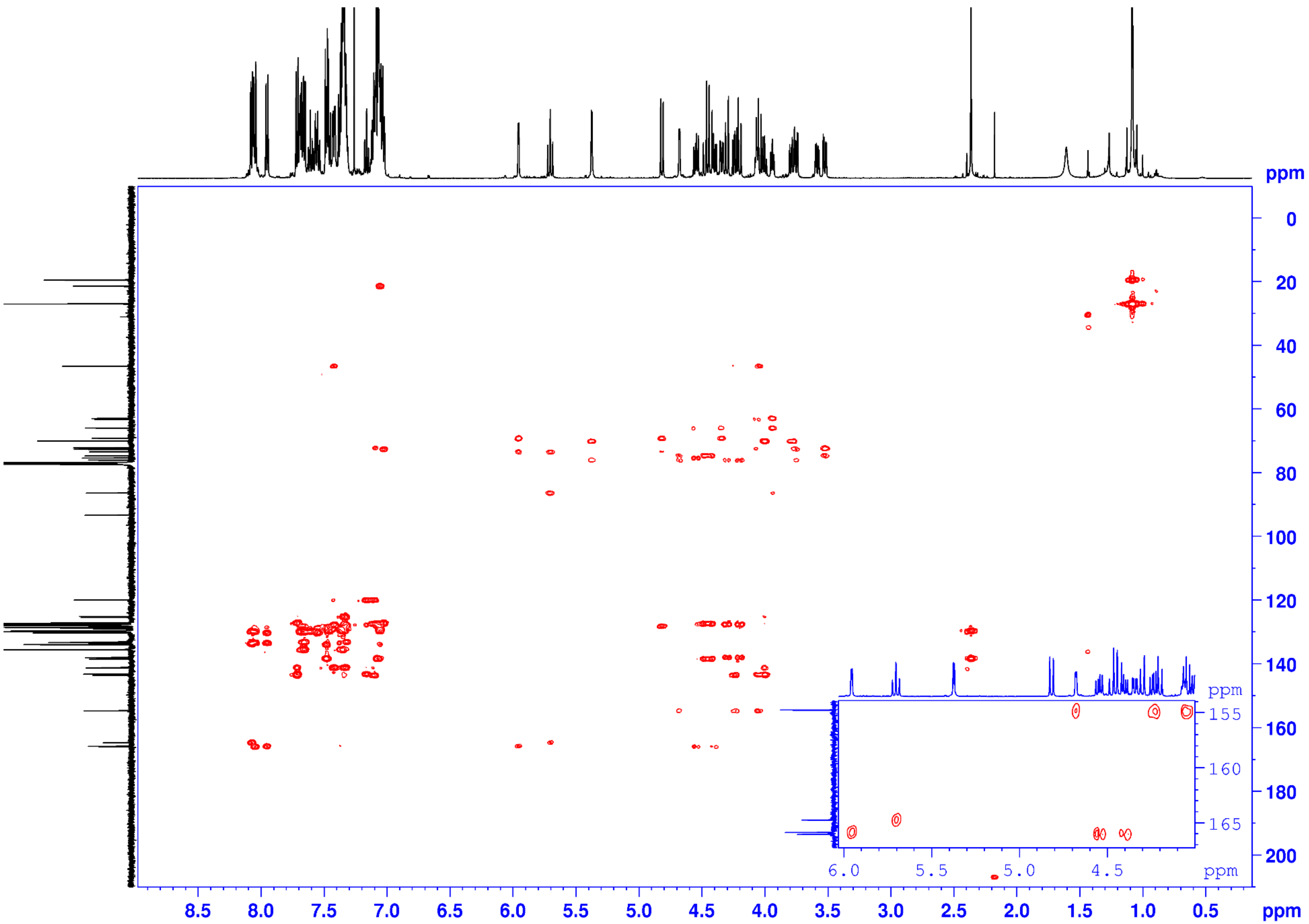
ppm
0
20
40
60
80
100
120
140
160
180
200
ppm
8.5
8.0
7.5
7.0
6.5
6.0
5.5
5.0
4.5
4.0
3.5
3.0
2.5
2.0
1.5
1.0
0.5
ppm
155
160
165
ppm
6.0
5.5
5.0
4.5
ppm

$^{13}C\{^1H\}$ NMR

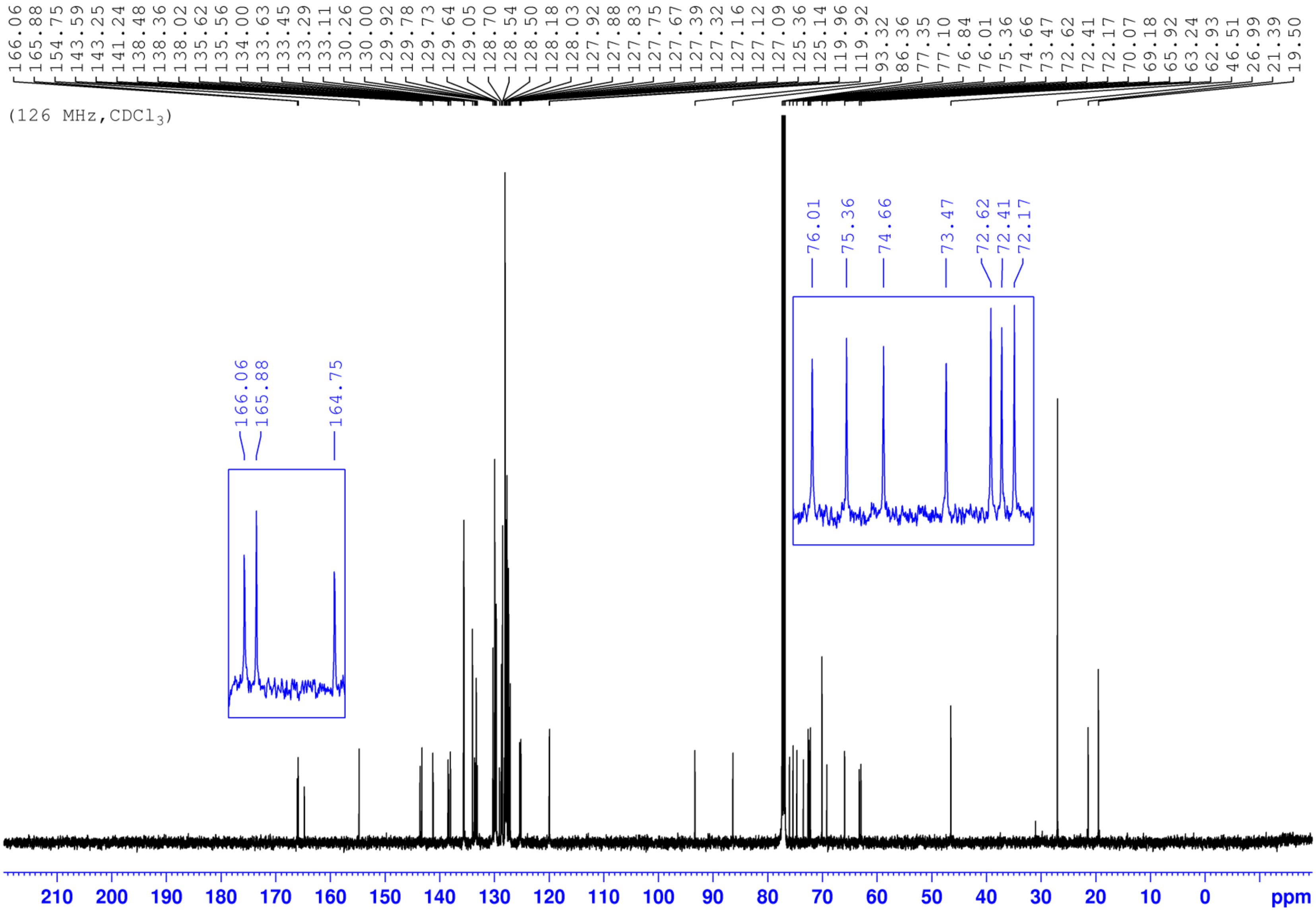
166.06
165.88
154.75
143.59
143.25
141.24
138.48
138.36
138.02
135.62
135.56
134.00
133.63
133.45
133.29
133.11
130.26
130.00
129.92
129.78
129.73
129.64
129.05
128.70
128.54
128.50
128.18
128.03
127.92
127.88
127.83
127.75
127.67
127.39
127.32
127.16
127.12
127.09
125.36
125.14
119.96
119.92
93.32
86.36
77.35
77.10
76.84
76.01
75.36
74.66
73.47
72.62
72.41
72.17
70.07
69.18
65.92
63.24
62.93
46.51
26.99
21.39
19.50
(126 MHz,CDCl3)
166.06
165.88
164.75
76.01
75.36
74.66
73.47
72.62
72.41
72.17
210 200 190 180 170 160 150 140 130 120 110 100 90 80 70 60 50 40 30 20 10 0 ppm

Compound **19**

^{1}H-NMR

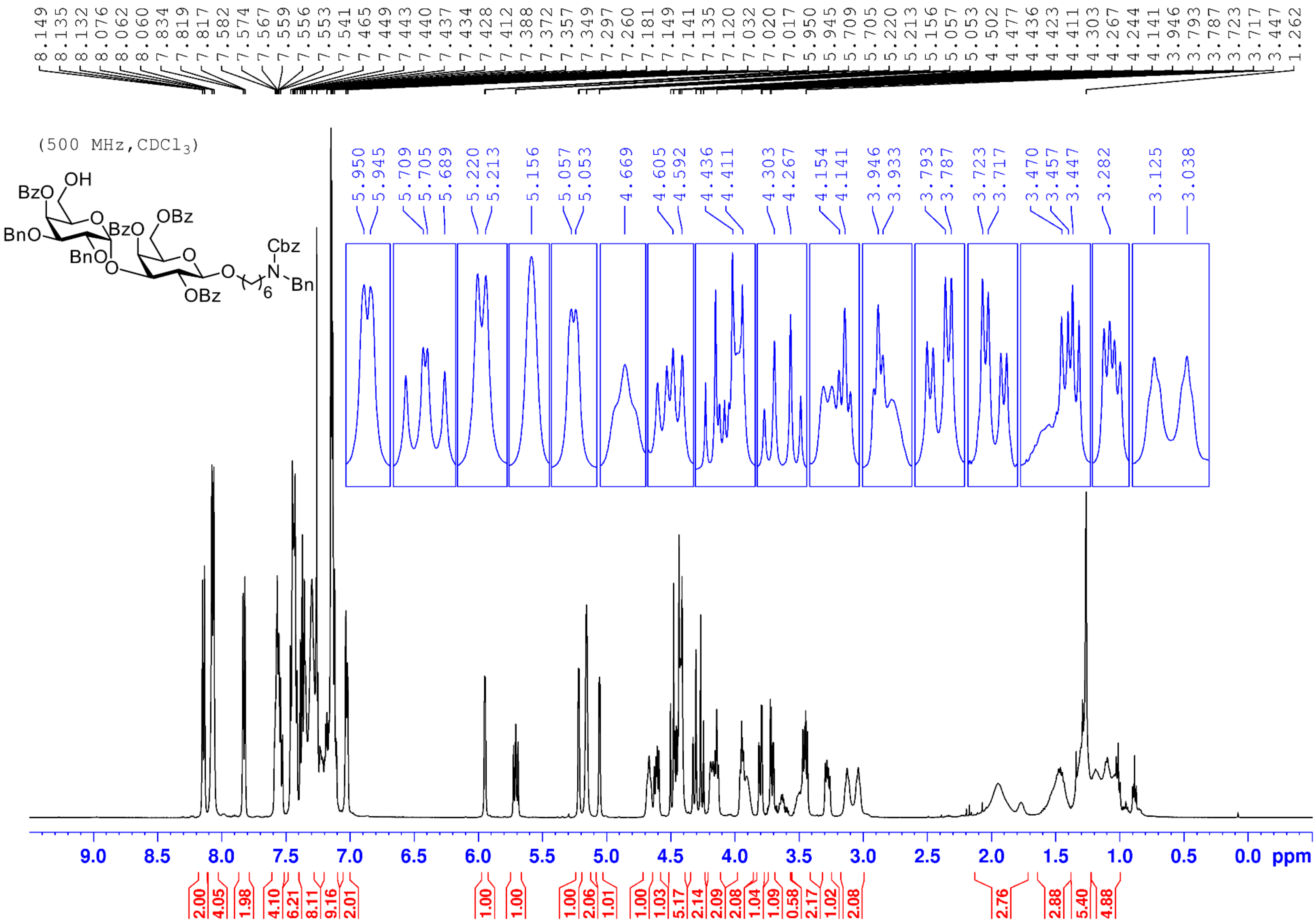

^{1}H-^{1}H COSY

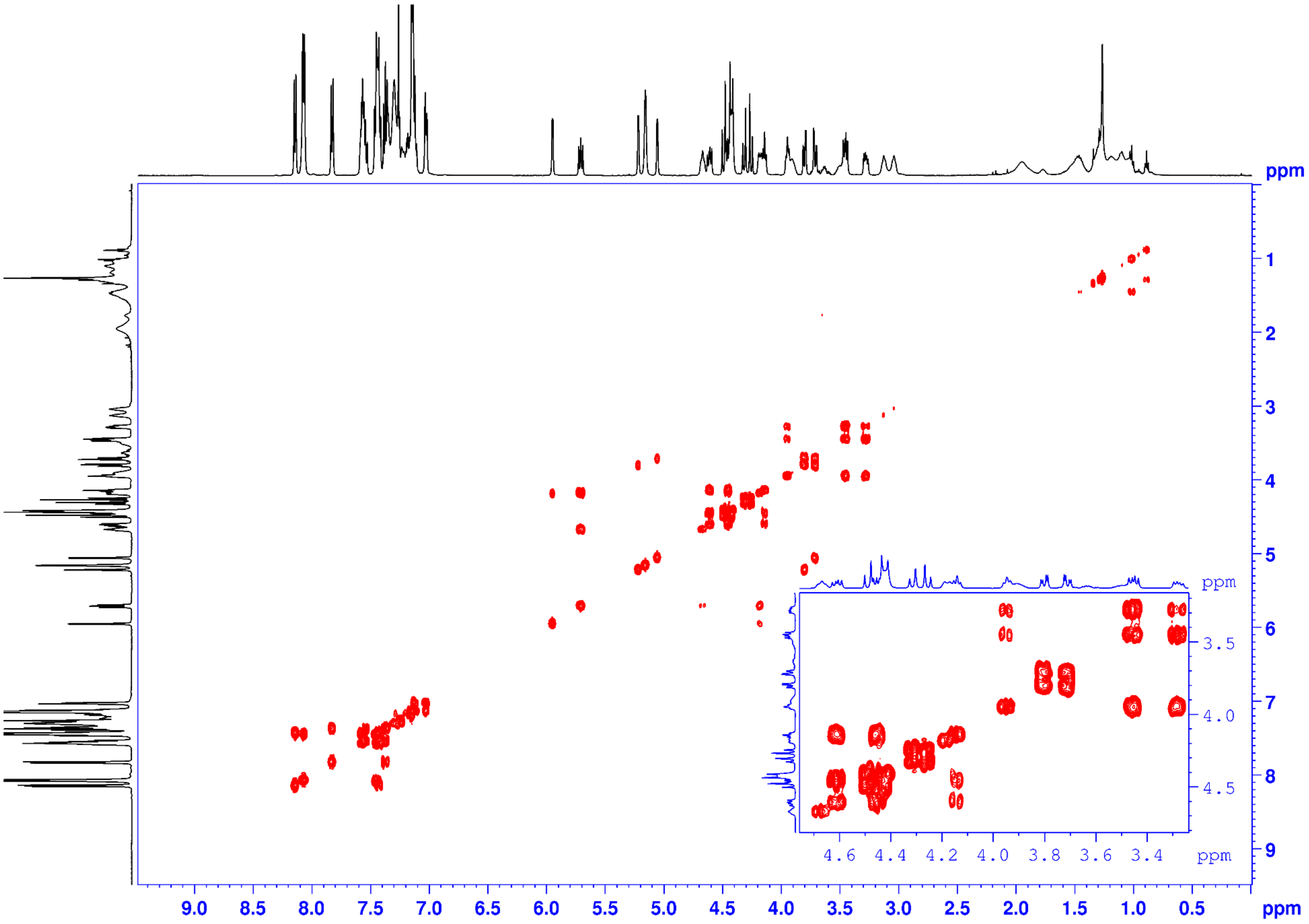

ppm
9.0
8.5
8.0
7.5
7.0
6.5
6.0
5.5
5.0
4.5
4.0
3.5
3.0
2.5
2.0
1.5
1.0
0.5
ppm
1
2
3
4
5
6
7
8
9
4.6
4.4
4.2
4.0
3.8
3.6
3.4
3.5
4.0
4.5

^1H-^{13}C HSQC

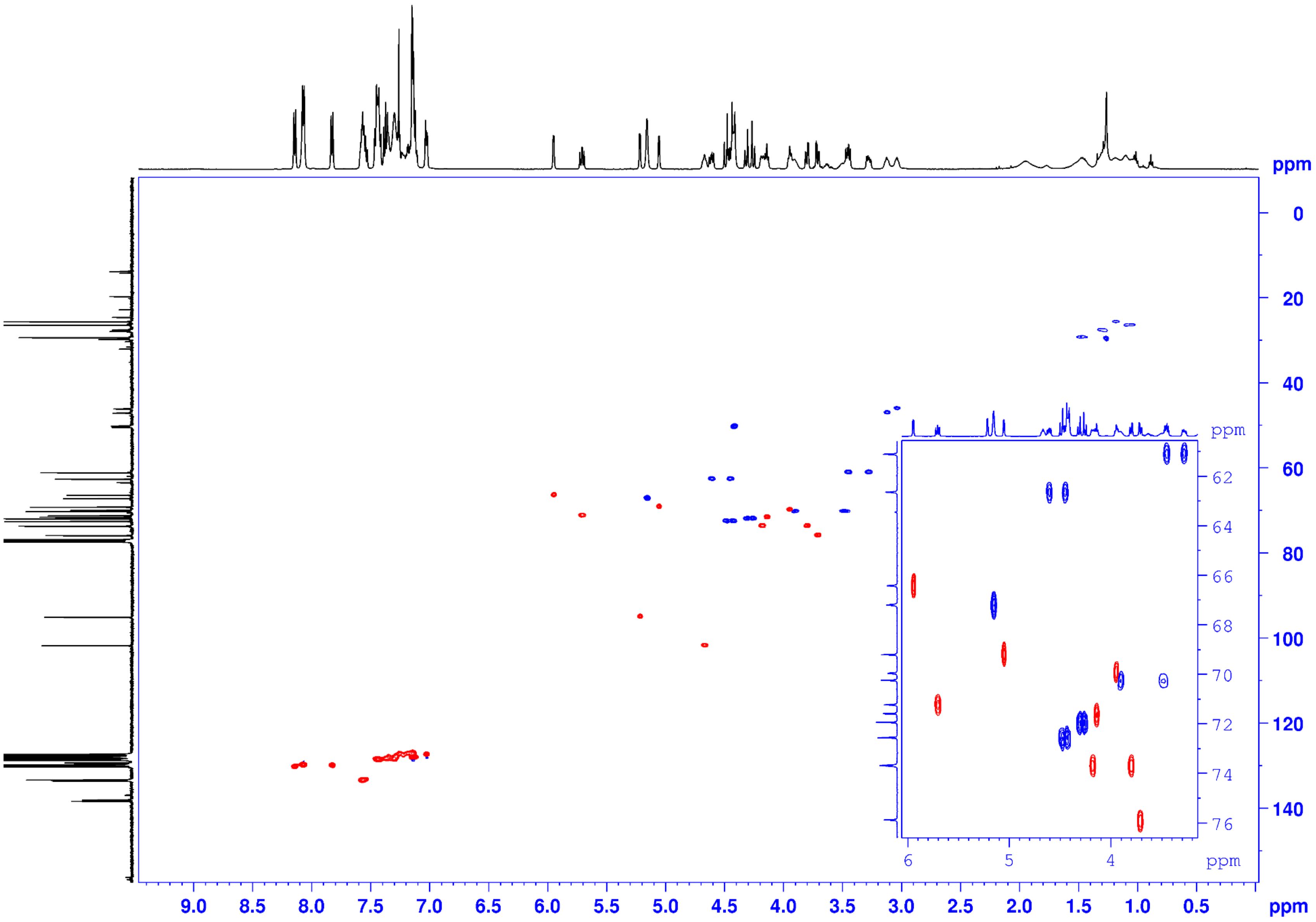

ppm
0
20
40
60
80
100
120
140
9.0
8.5
8.0
7.5
7.0
6.5
6.0
5.5
5.0
4.5
4.0
3.5
3.0
2.5
2.0
1.5
1.0
0.5
ppm
62
64
66
68
70
72
74
76
6
5
4

^{1}H-^{13}C HMBC

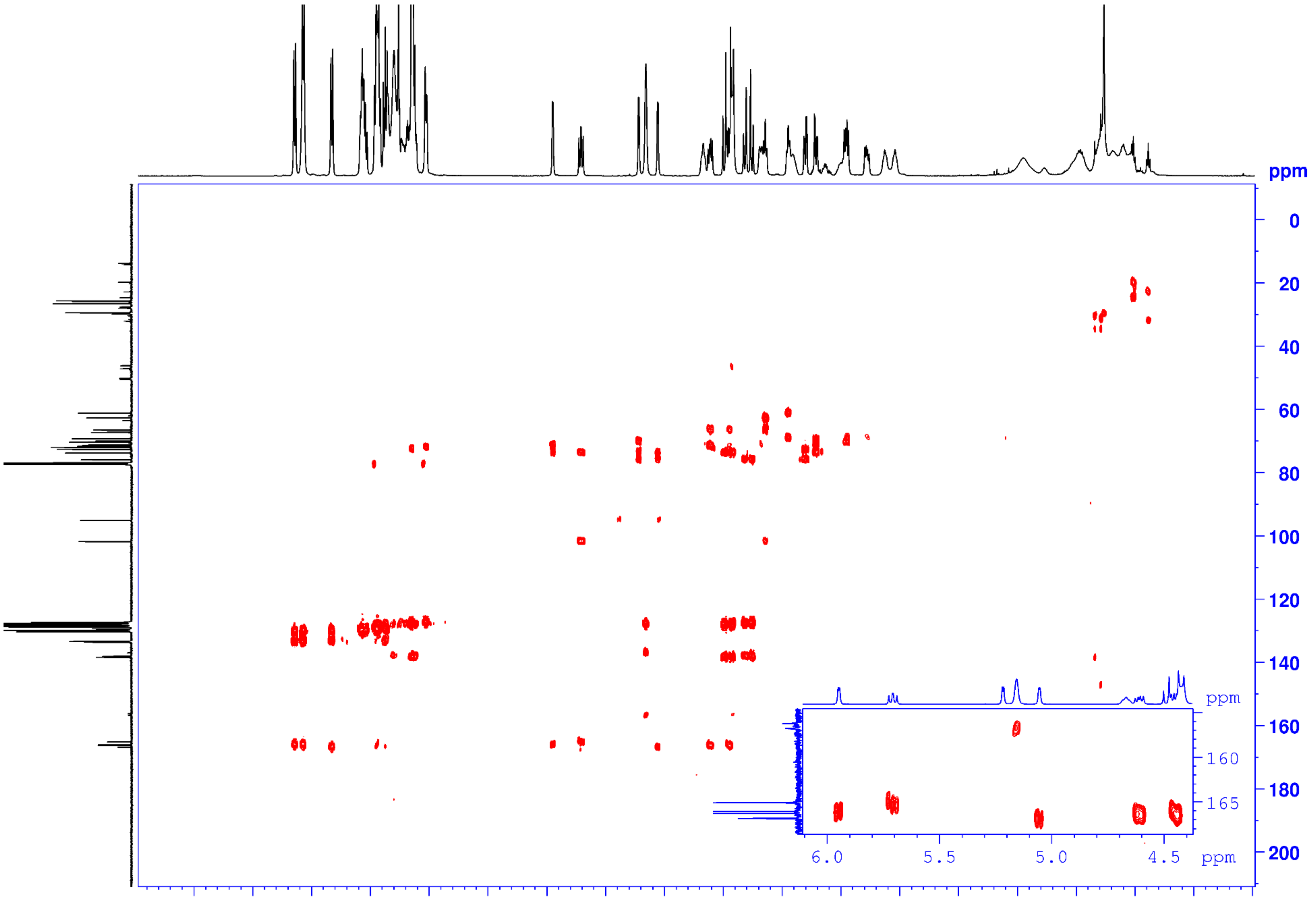

ppm
0
20
40
60
80
100
120
140
160
180
200
9.0
8.5
8.0
7.5
7.0
6.5
6.0
5.5
5.0
4.5
4.0
3.5
3.0
2.5
2.0
1.5
1.0
0.5
ppm
160
165
6.0
5.5
5.0
4.5
ppm

$^{13}C\{^{1}H\}$ NMR

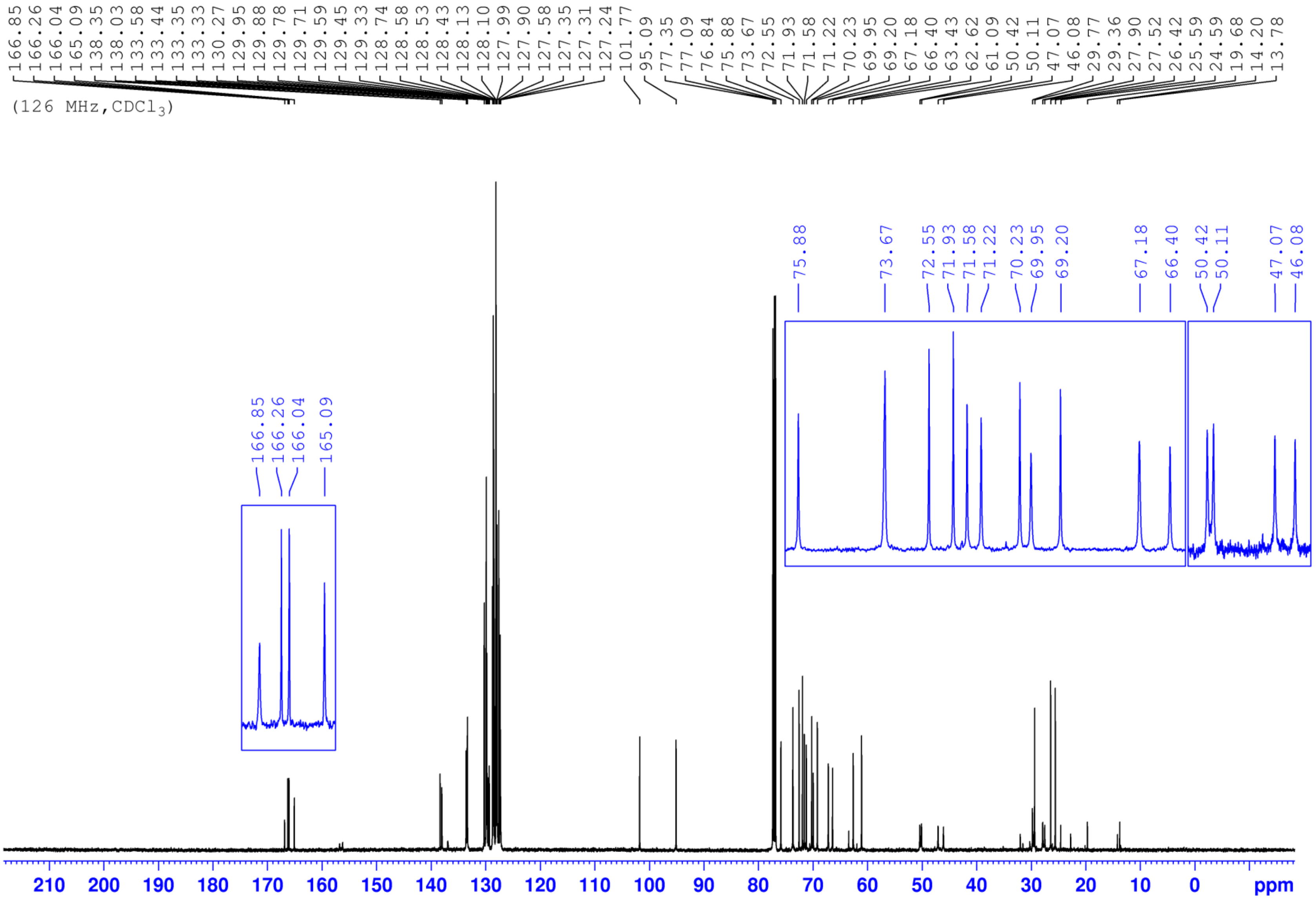
166.85
166.26
166.04
165.09
138.35
138.03
133.58
133.44
133.35
133.33
130.27
129.95
129.88
129.78
129.71
129.59
129.45
129.33
128.74
128.58
128.53
128.43
128.13
128.10
127.99
127.90
127.58
127.35
127.31
127.24
101.77
95.09
77.35
77.09
76.84
75.88
73.67
72.55
71.93
71.58
71.22
70.23
69.95
69.20
67.18
66.40
63.43
62.62
61.09
50.42
50.11
47.07
46.08
29.77
29.36
27.90
27.52
26.42
25.59
24.59
19.68
14.20
13.78
(126 MHz, CDCl3)
210
200
190
180
170
160
150
140
130
120
110
100
90
80
70
60
50
40
30
20
10
0
ppm

Compound **20**

^{1}H-NMR

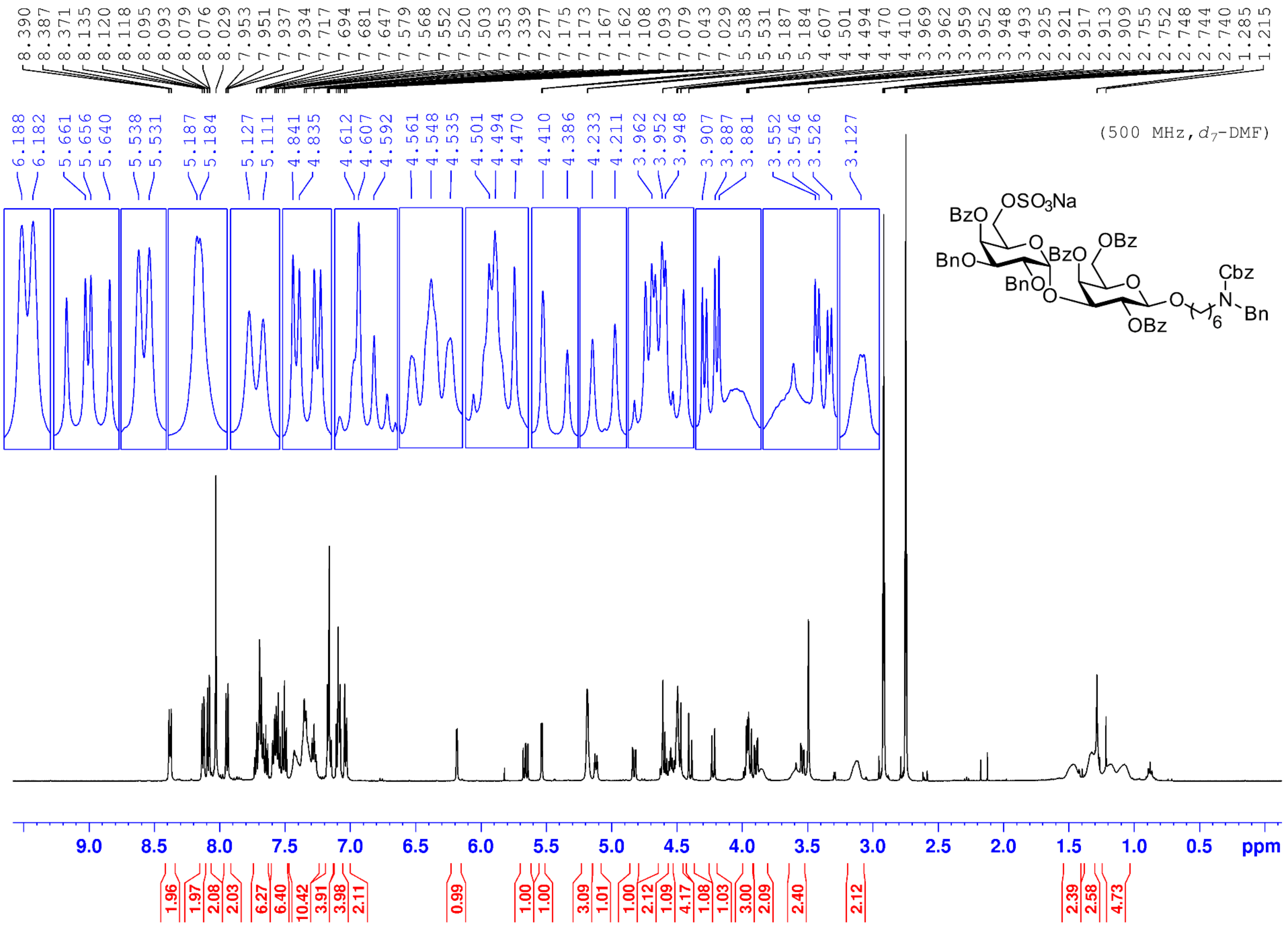

^1H-^1H COSY

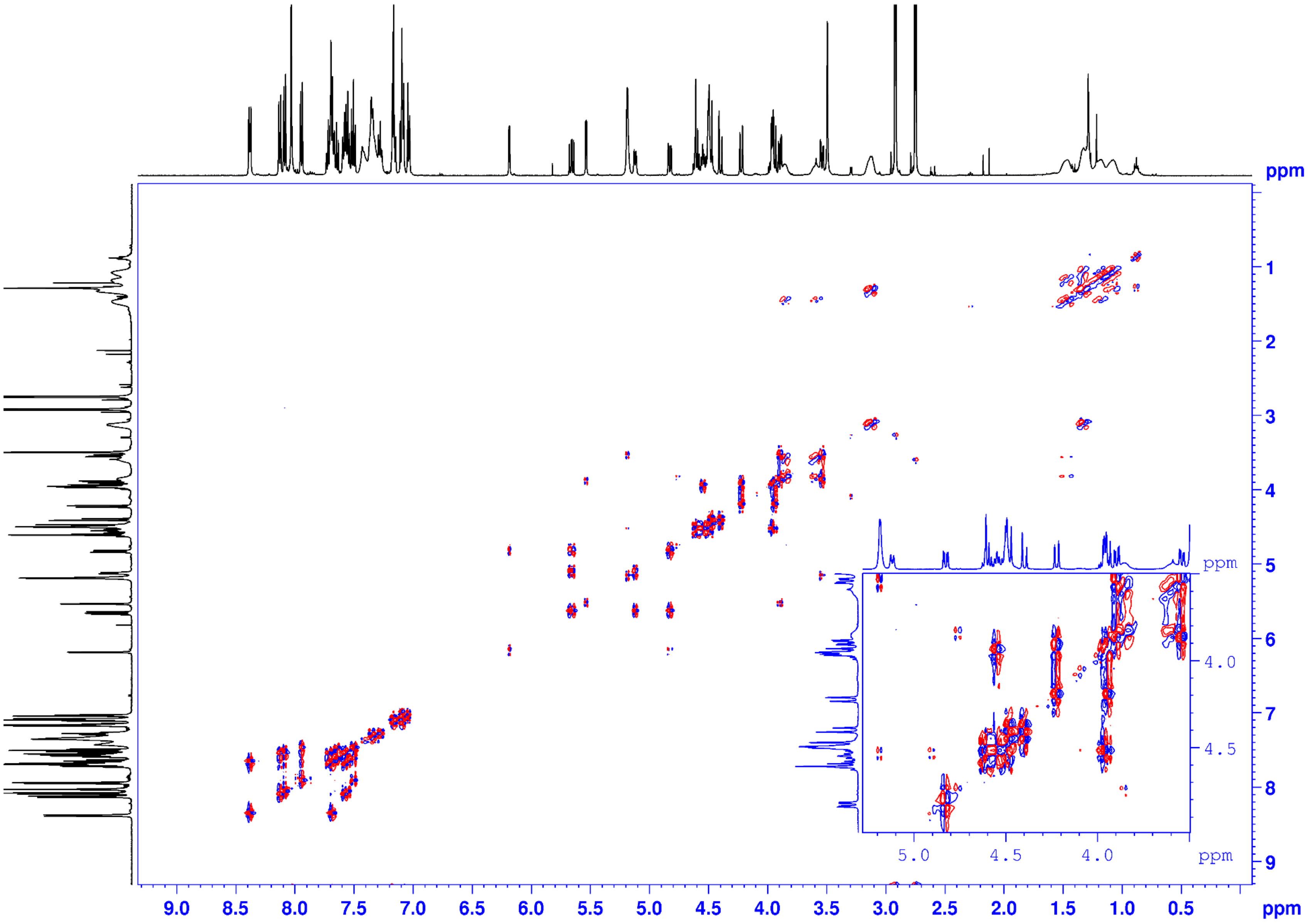
ppm
1
2
3
4
5
6
7
8
9
9.0
8.5
8.0
7.5
7.0
6.5
6.0
5.5
5.0
4.5
4.0
3.5
3.0
2.5
2.0
1.5
1.0
0.5
ppm
4.0
4.5
5.0
4.5
4.0
ppm

^{1}H-^{13}C HSQC

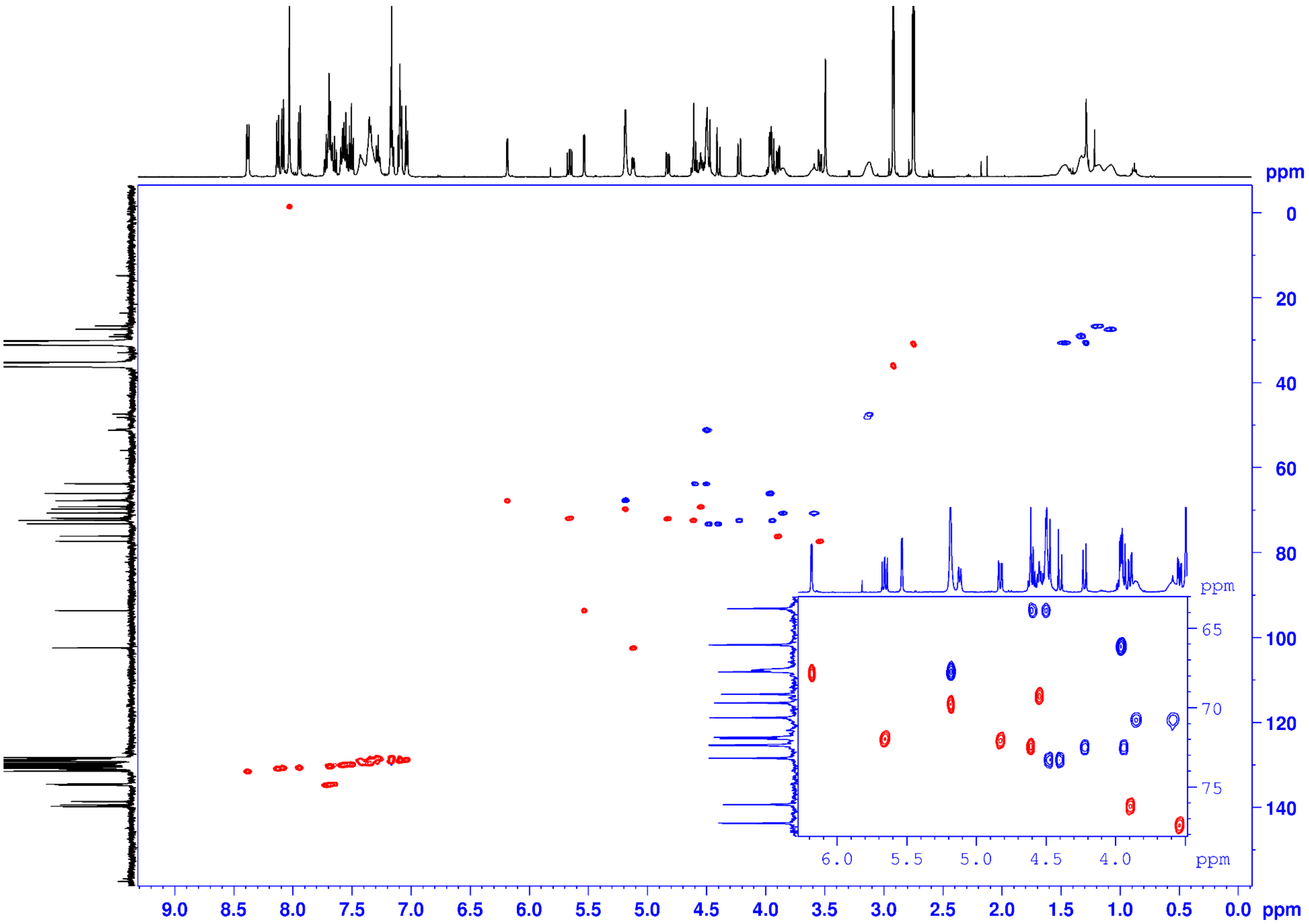
ppm
0
20
40
60
80
100
120
140
9.0
8.5
8.0
7.5
7.0
6.5
6.0
5.5
5.0
4.5
4.0
3.5
3.0
2.5
2.0
1.5
1.0
0.5
0.0
ppm
65
70
75
6.0
5.5
5.0
4.5
4.0
ppm

^{1}H-^{13}C non-decoupled HSQC

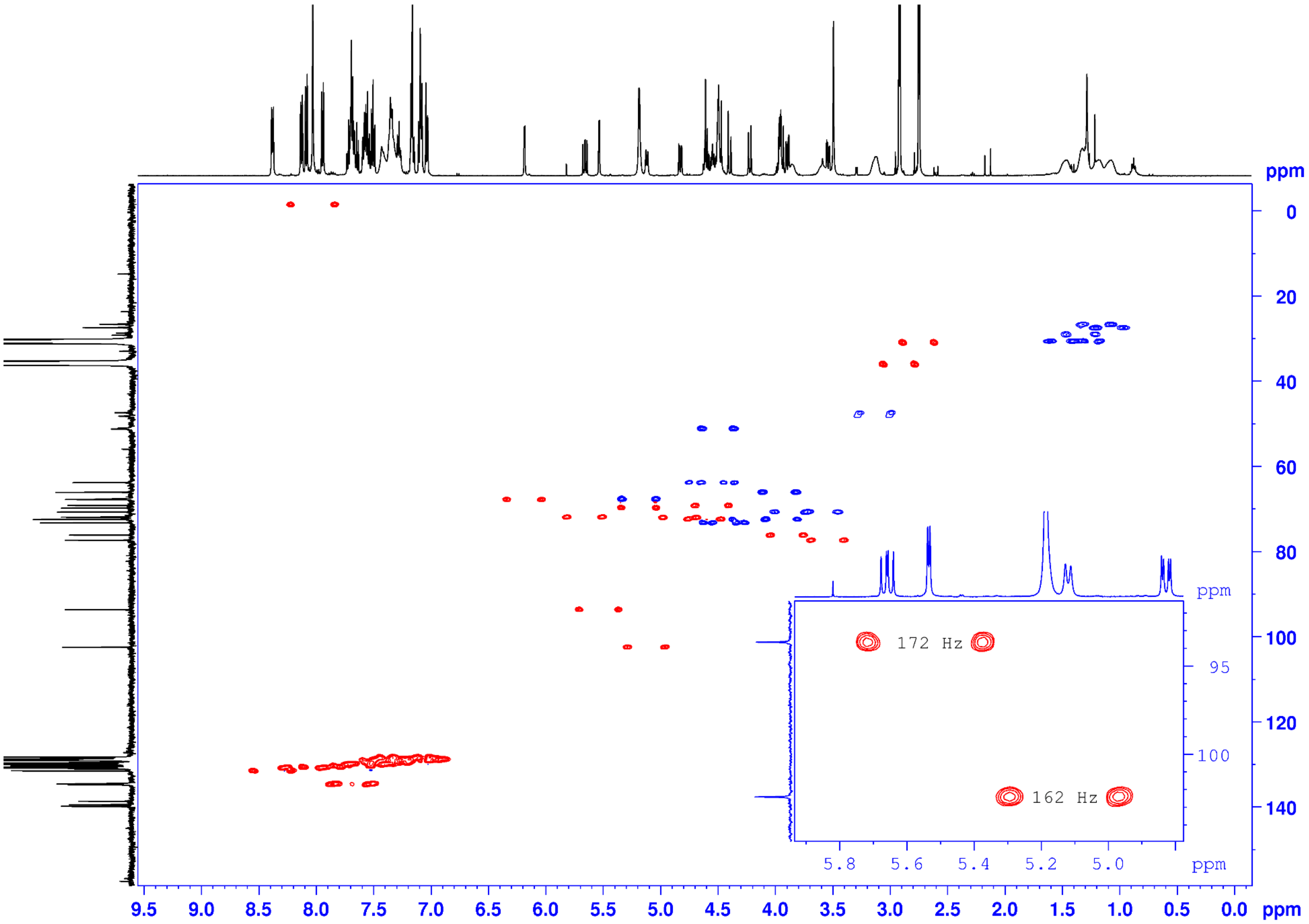

^1H-^{13}C HMBC

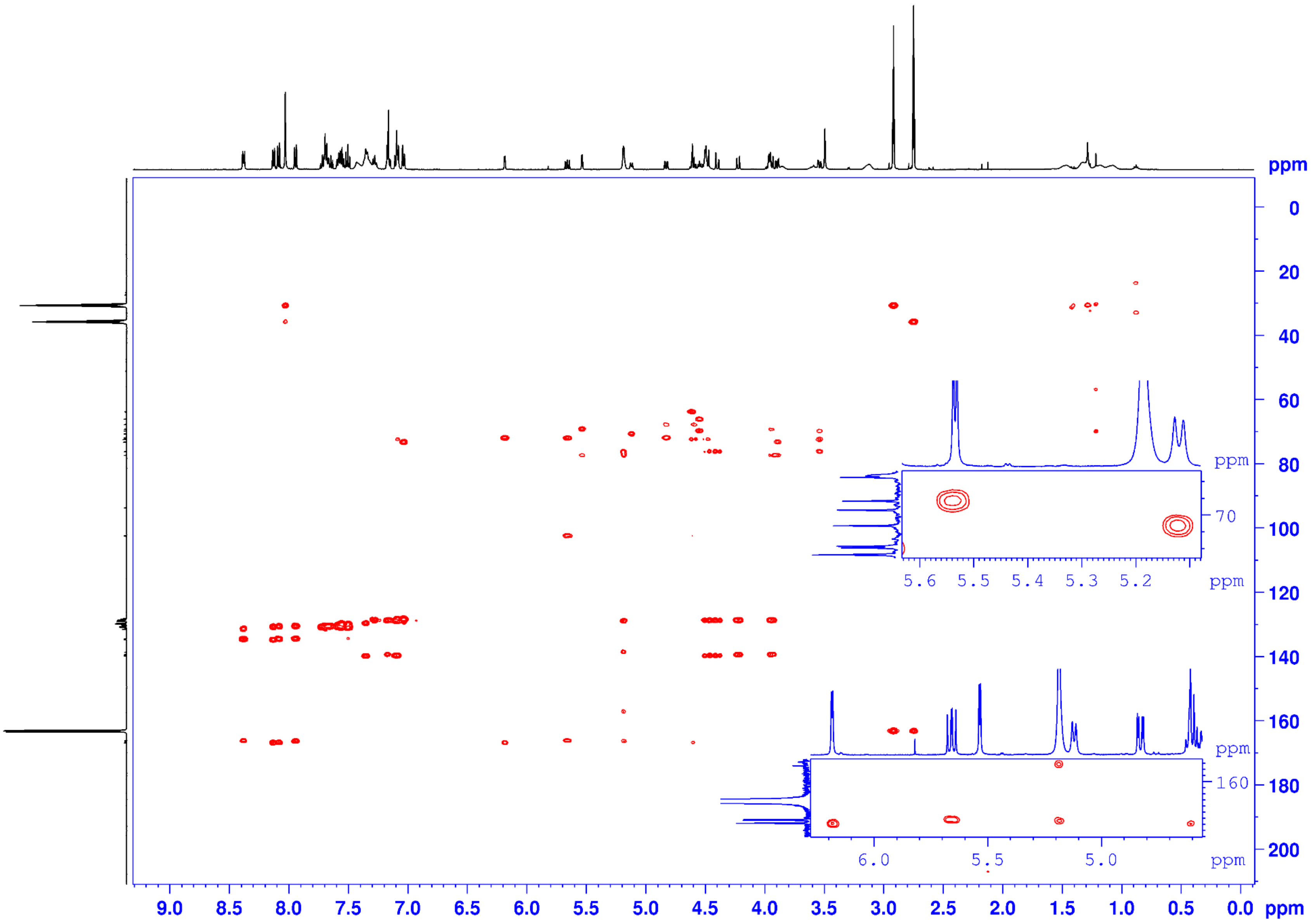

ppm
0
20
40
60
80
100
120
140
160
180
200
9.0
8.5
8.0
7.5
7.0
6.5
6.0
5.5
5.0
4.5
4.0
3.5
3.0
2.5
2.0
1.5
1.0
0.5
0.0
ppm
ppm
70
5.6
5.5
5.4
5.3
5.2
ppm
ppm
160
6.0
5.5
5.0
ppm

$^{13}C\{^1H\}$ NMR

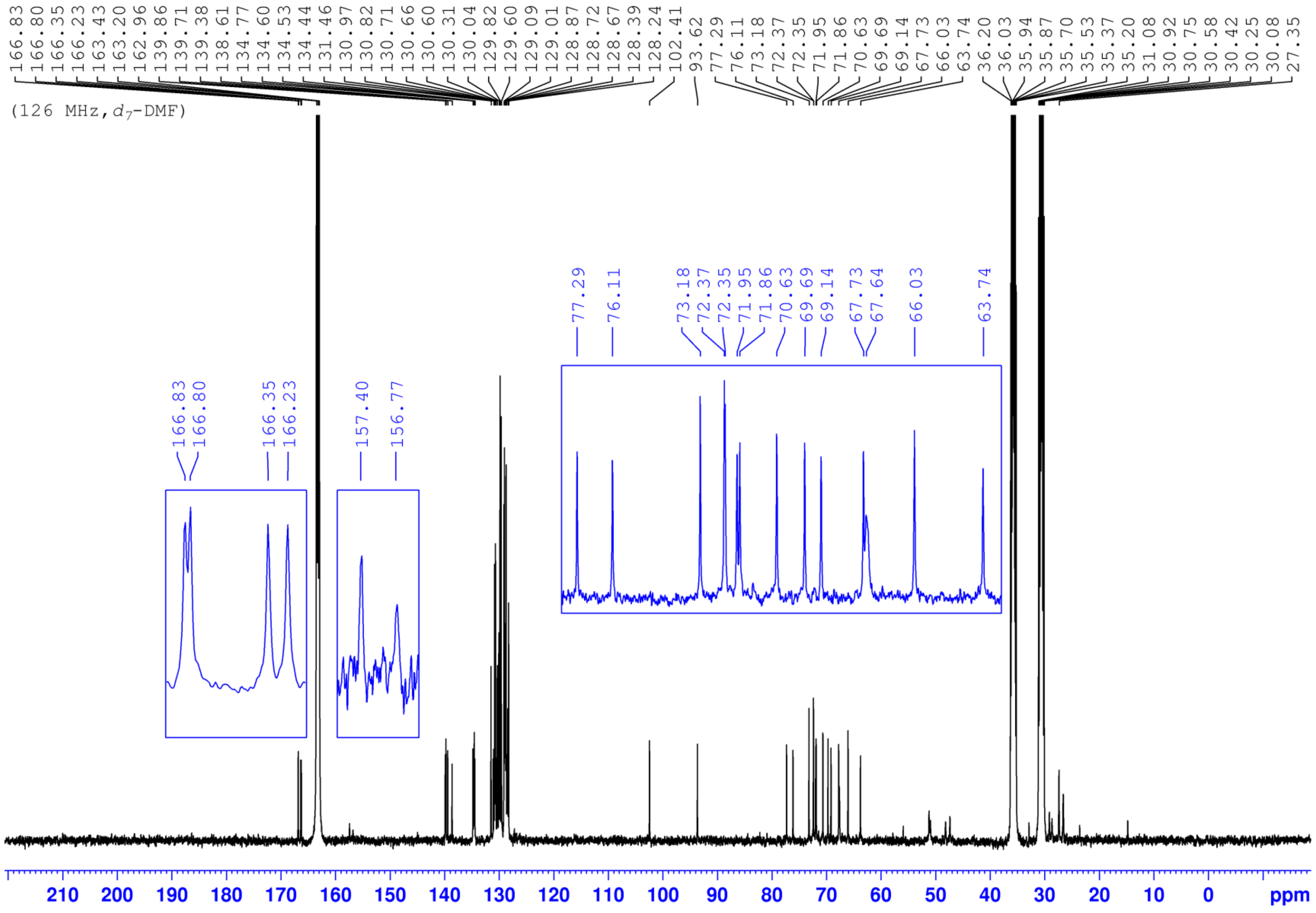
166.83
166.80
166.35
166.23
163.43
163.20
162.96
139.86
139.71
139.38
138.61
134.77
134.60
134.53
134.44
131.46
130.97
130.82
130.71
130.66
130.60
130.31
130.04
129.82
129.60
129.09
129.01
128.87
128.72
128.67
128.39
128.24
102.41
93.62
77.29
76.11
73.18
72.37
72.35
71.95
71.86
70.63
69.69
69.14
67.73
66.03
63.74
36.20
36.03
35.94
35.87
35.70
35.53
35.37
35.20
31.08
30.92
30.75
30.58
30.42
30.25
30.08
27.35
(126 MHz, d7-DMF)
166.83
166.80
166.35
166.23
157.40
156.77
77.29
76.11
73.18
72.37
72.35
71.95
71.86
70.63
69.69
69.14
67.73
67.64
66.03
63.74
210
200
190
180
170
160
150
140
130
120
110
100
90
80
70
60
50
40
30
20
10
0
ppm

Compound **21**

^{1}H-NMR

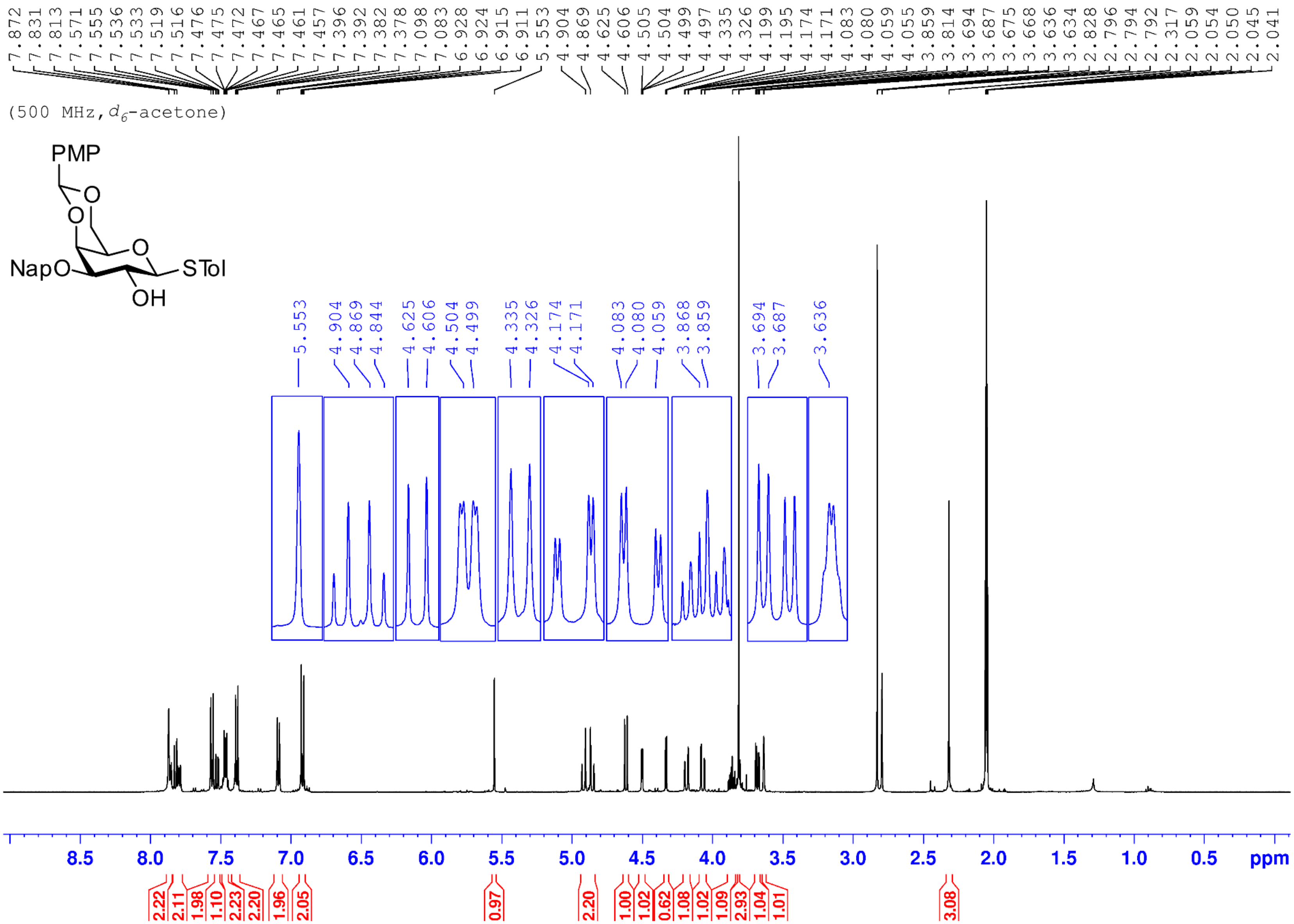

^{1}H-^{1}H COSY

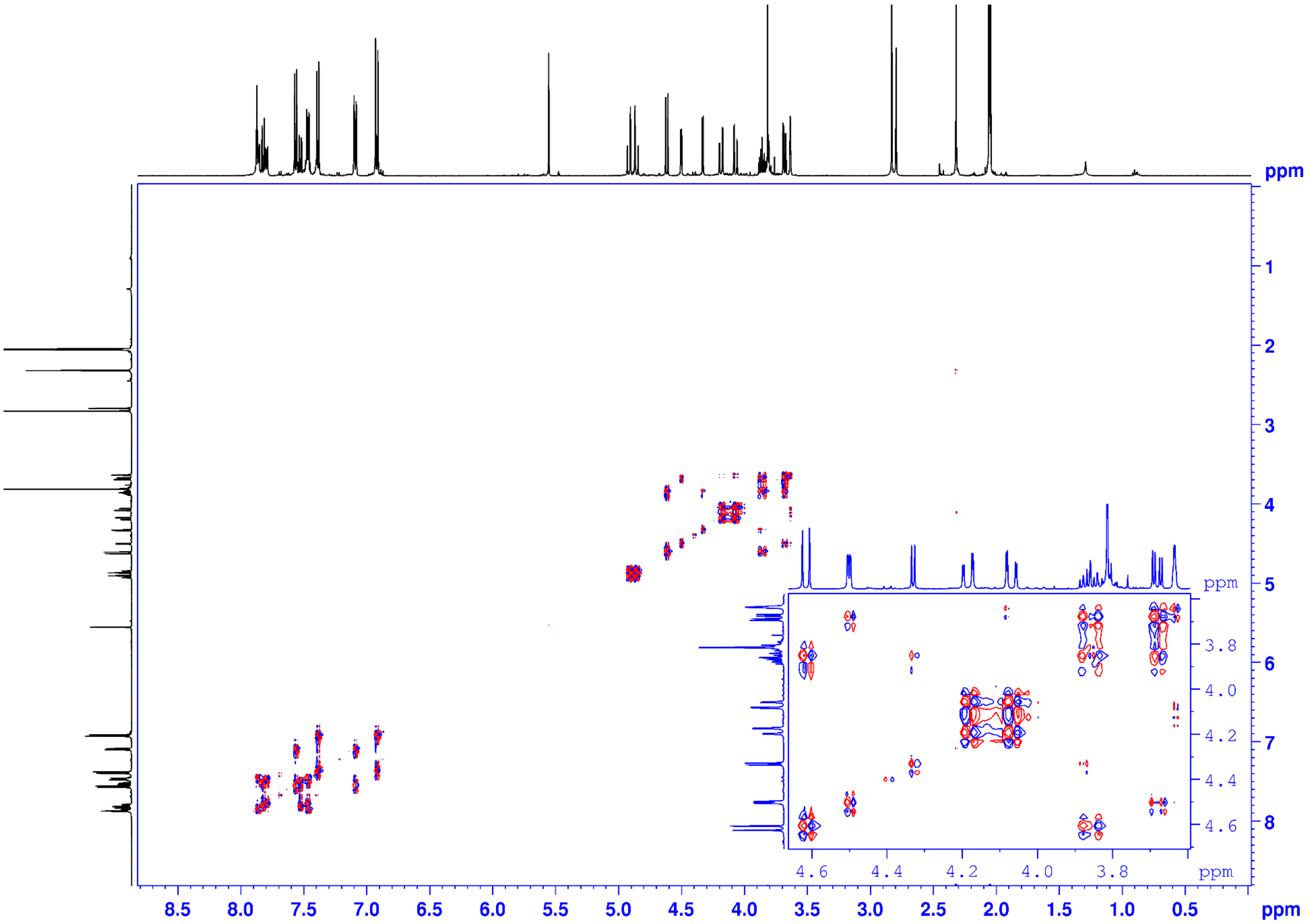

ppm
1
2
3
4
5
6
7
8
8.5
8.0
7.5
7.0
6.5
6.0
5.5
5.0
4.5
4.0
3.5
3.0
2.5
2.0
1.5
1.0
0.5
ppm
4.6
4.4
4.2
4.0
3.8
ppm
3.8
4.0
4.2
4.4
4.6
ppm

^{1}H-^{13}C HSQC

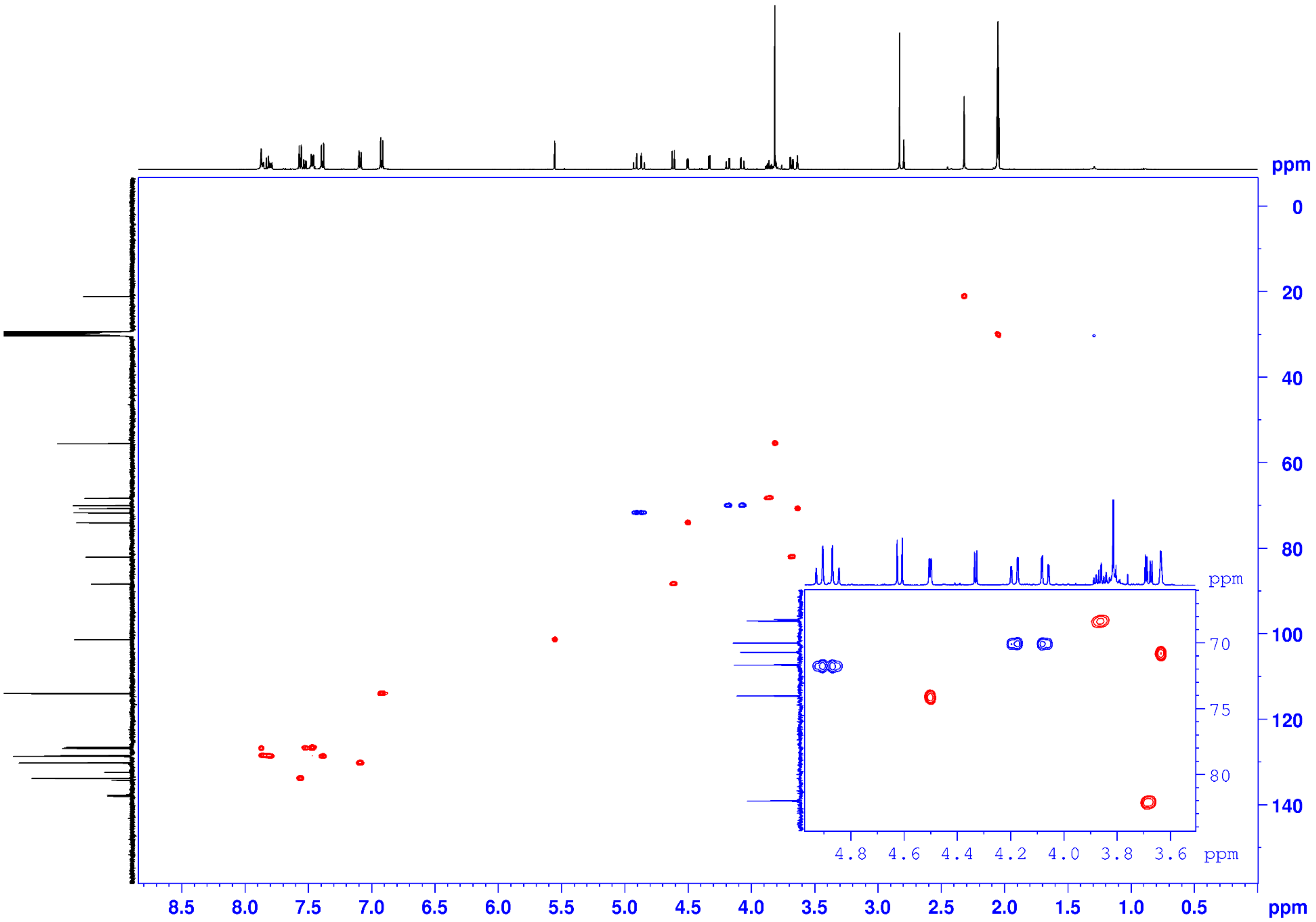
ppm
0
20
40
60
80
100
120
140
ppm
70
75
80
4.8 4.6 4.4 4.2 4.0 3.8 3.6 ppm
8.5 8.0 7.5 7.0 6.5 6.0 5.5 5.0 4.5 4.0 3.5 3.0 2.5 2.0 1.5 1.0 0.5
ppm

^{1}H-^{13}C HMBC

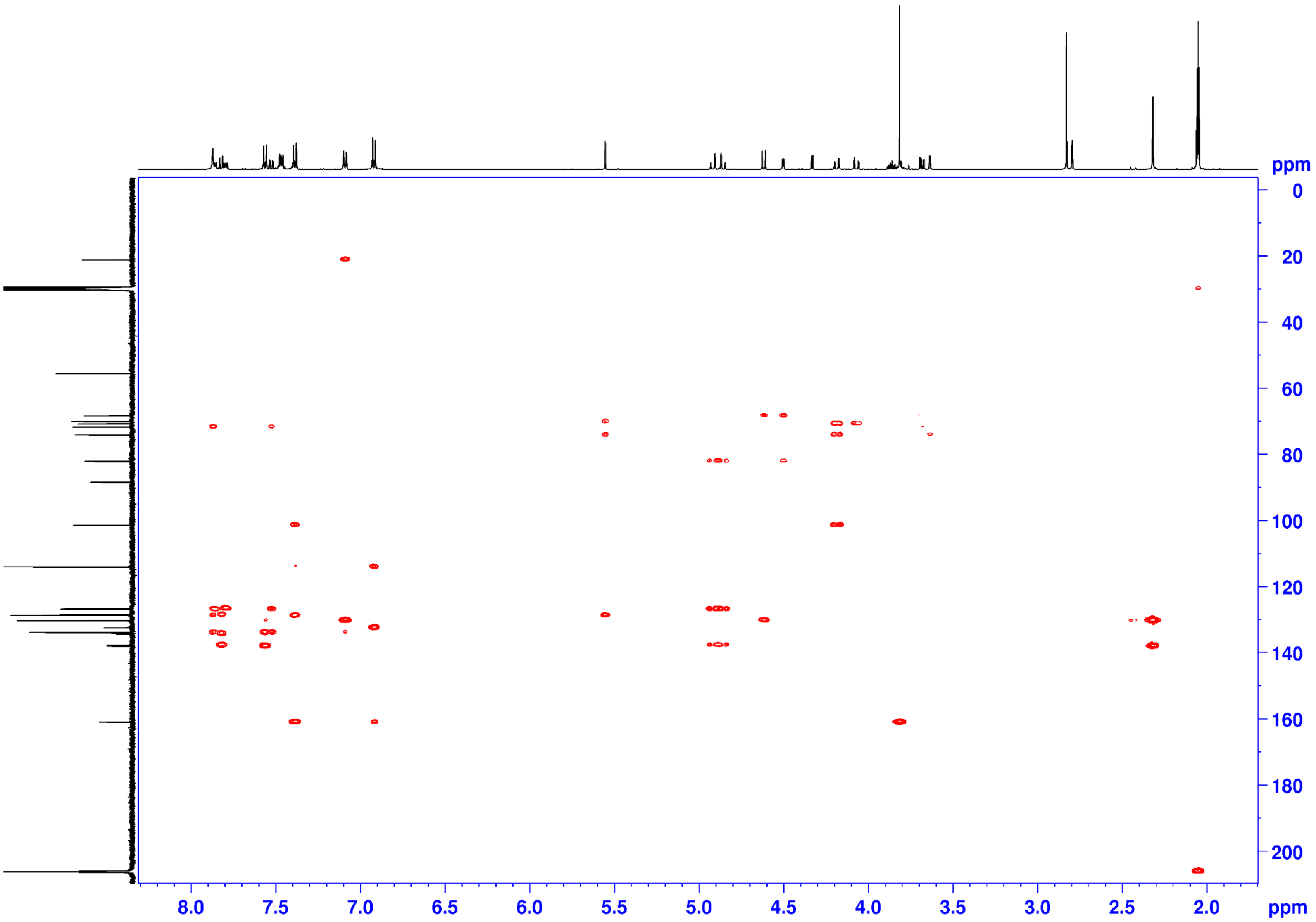

ppm
0
20
40
60
80
100
120
140
160
180
200
8.0
7.5
7.0
6.5
6.0
5.5
5.0
4.5
4.0
3.5
3.0
2.5
2.0
ppm

$^{13}C\{^1H\}$ NMR

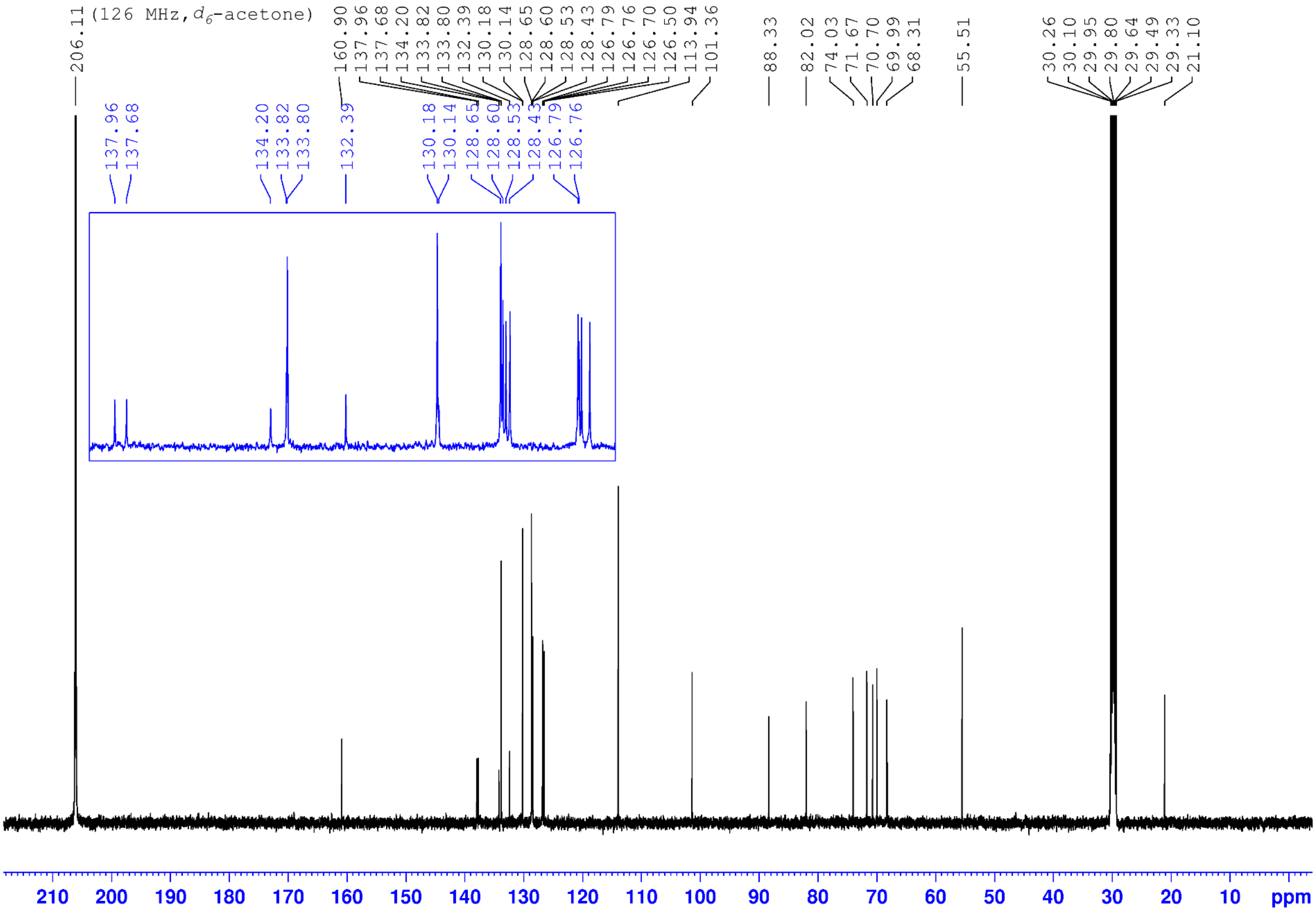
(126 MHz, d6-acetone)
206.11
160.90
137.96
137.68
134.20
133.82
133.80
132.39
130.18
130.14
128.65
128.60
128.53
128.43
126.79
126.76
126.70
126.50
113.94
101.36
88.33
82.02
74.03
71.67
70.70
69.99
68.31
55.51
30.26
30.10
29.95
29.80
29.64
29.49
29.33
21.10
210
200
190
180
170
160
150
140
130
120
110
100
90
80
70
60
50
40
30
20
10
ppm

Compound **22**

^{1}H-NMR

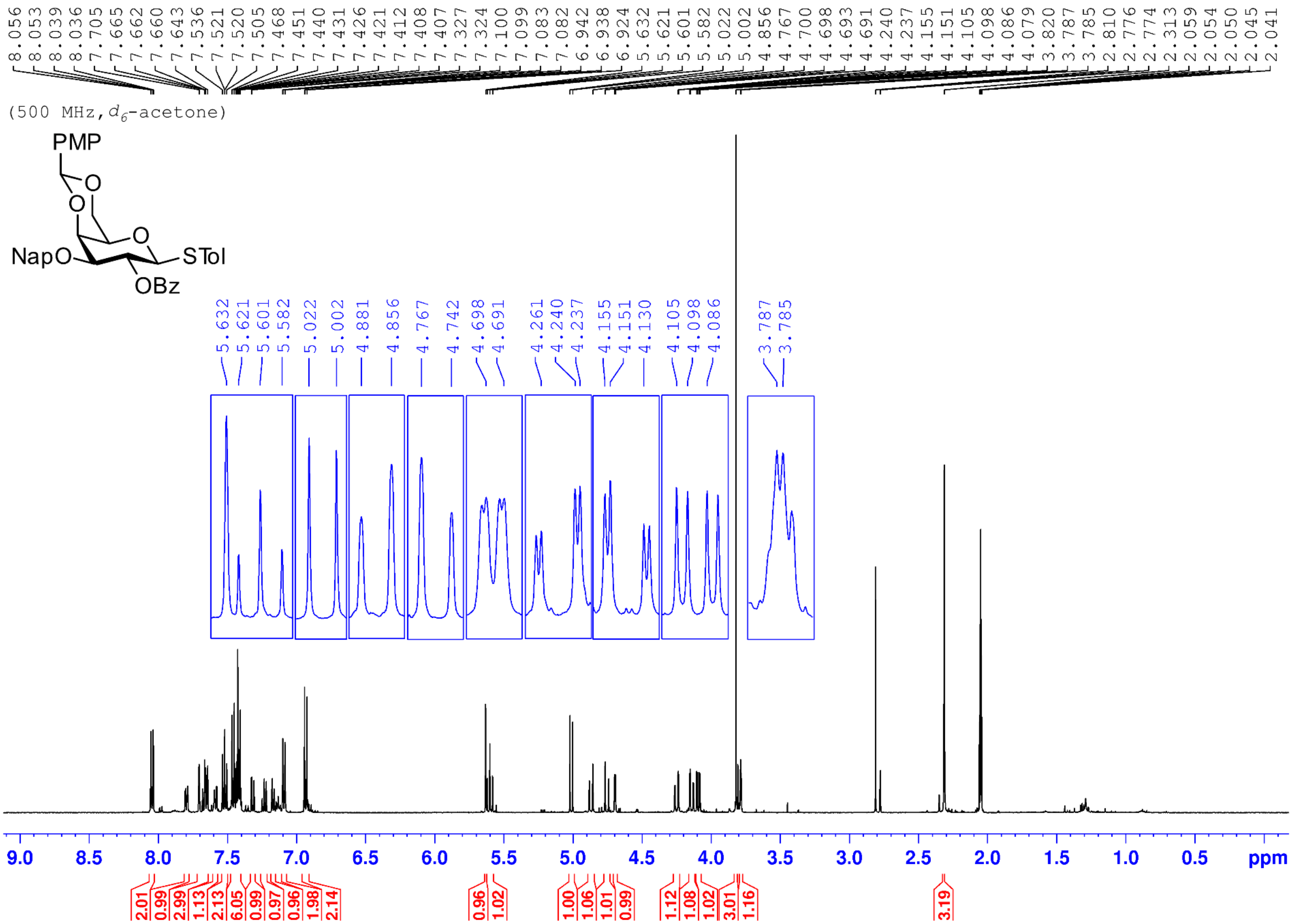

^{1}H-^{1}H COSY

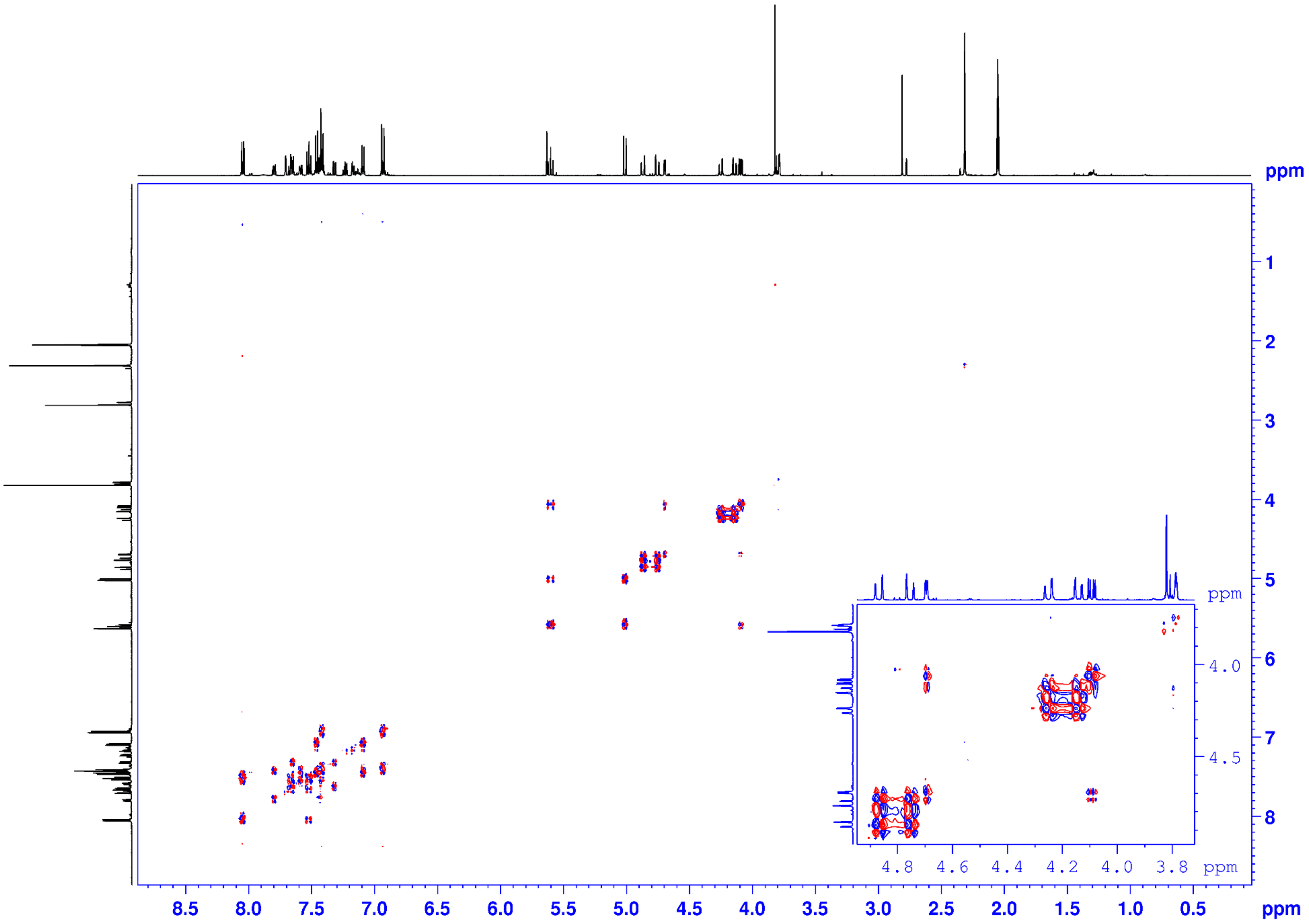

ppm
1
2
3
4
5
6
7
8
8.5
8.0
7.5
7.0
6.5
6.0
5.5
5.0
4.5
4.0
3.5
3.0
2.5
2.0
1.5
1.0
0.5
ppm
4.0
4.5
4.8
4.6
4.4
4.2
4.0
3.8
ppm

$^{1}H-^{13}C$ HSQC

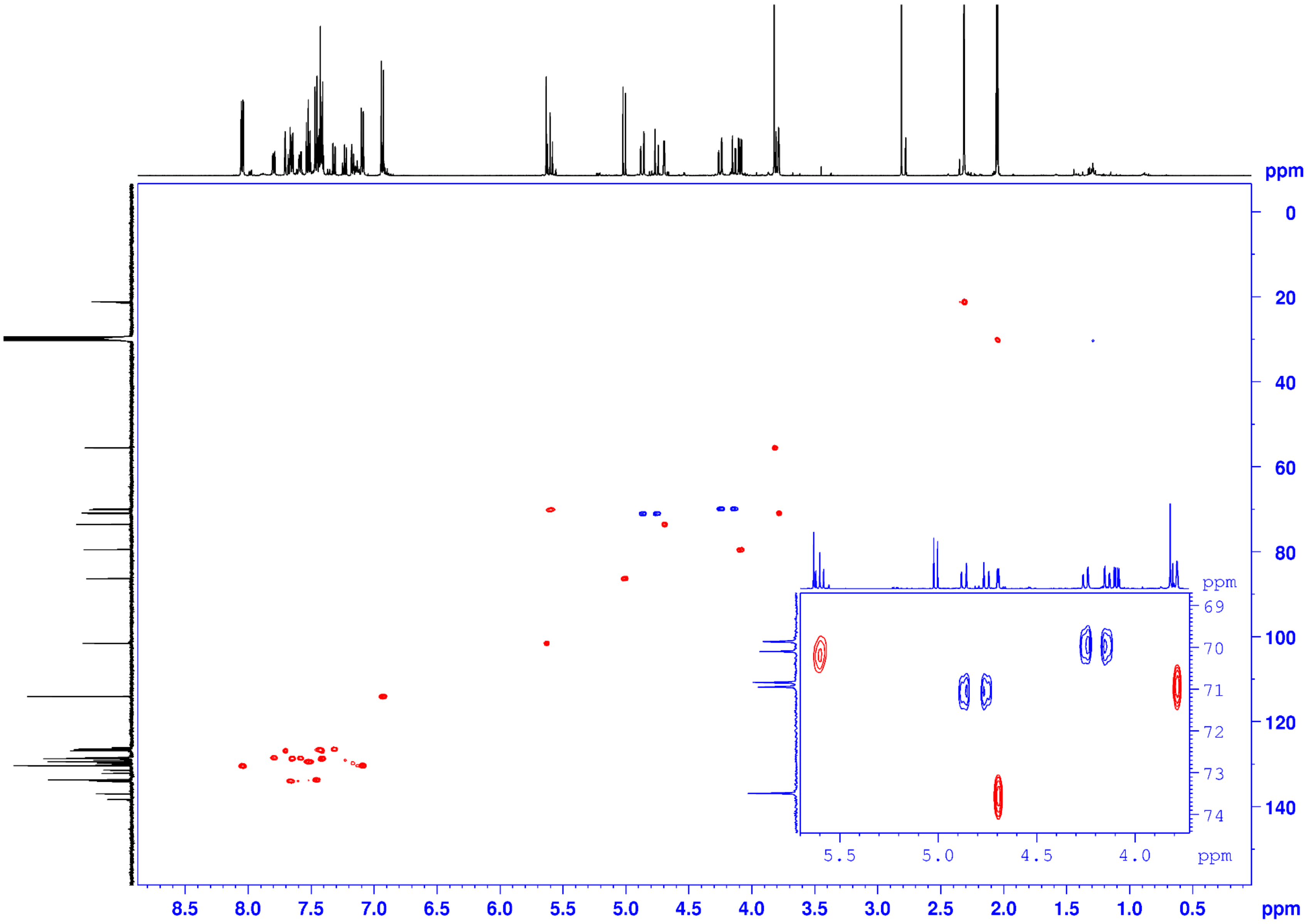

ppm
0
20
40
60
80
100
120
140
8.5
8.0
7.5
7.0
6.5
6.0
5.5
5.0
4.5
4.0
3.5
3.0
2.5
2.0
1.5
1.0
0.5
ppm
69
70
71
72
73
74
5.5
5.0
4.5
4.0

^{1}H-^{13}C HMBC

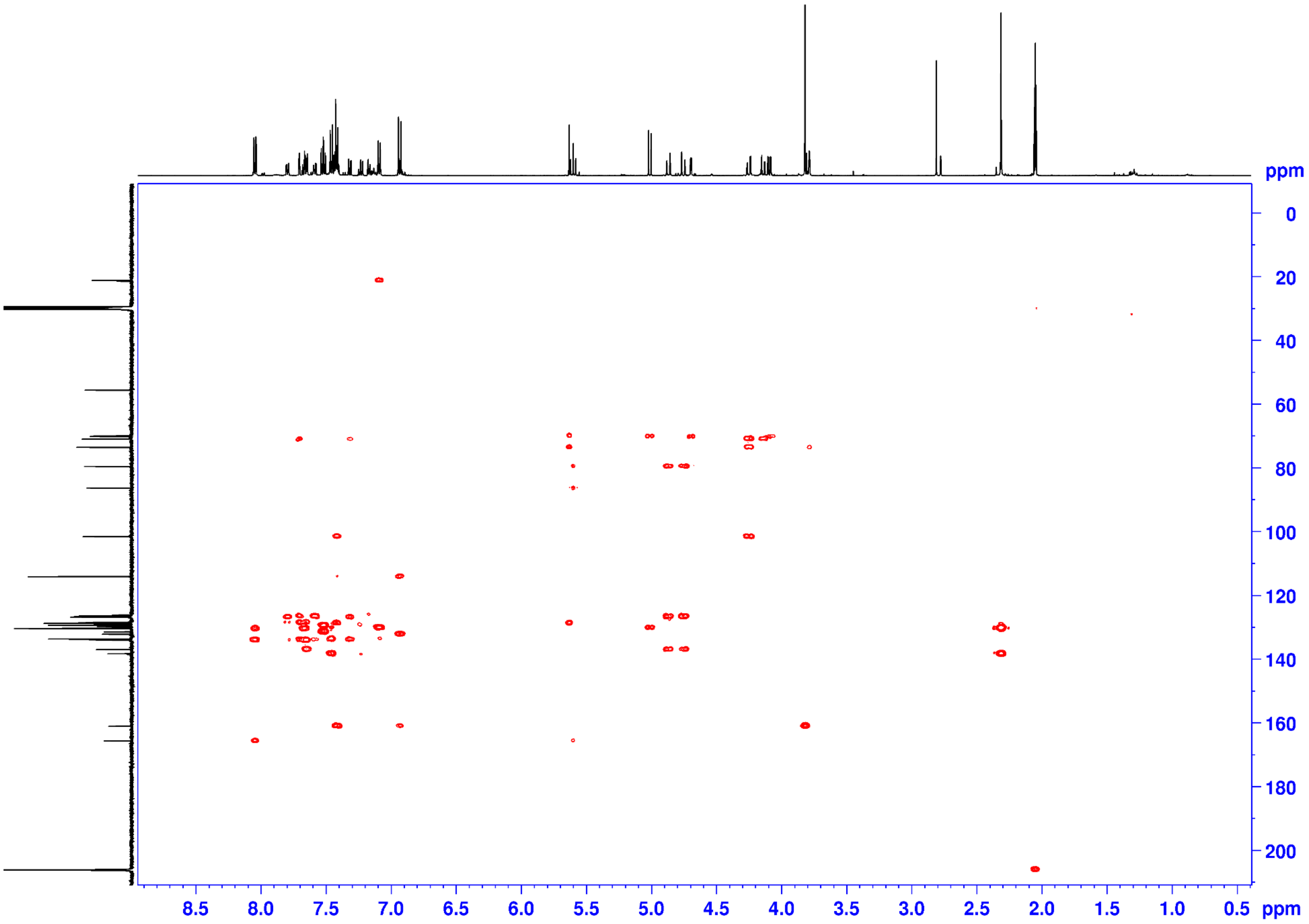
ppm
0
20
40
60
80
100
120
140
160
180
200
8.5
8.0
7.5
7.0
6.5
6.0
5.5
5.0
4.5
4.0
3.5
3.0
2.5
2.0
1.5
1.0
0.5
ppm

$^{13}C\{^1H\}$ NMR

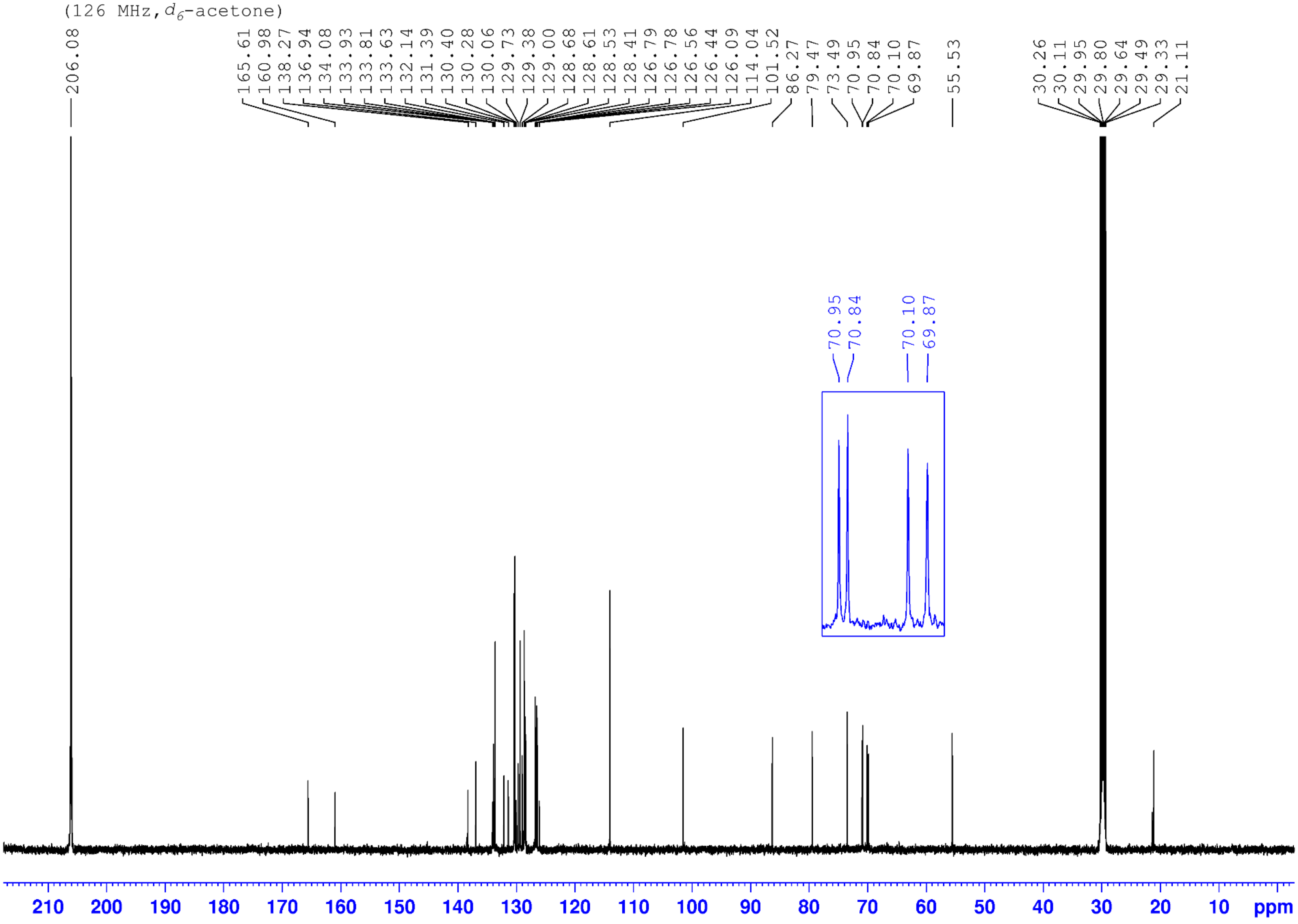

(126 MHz, d_6-acetone)
206.08
165.61
160.98
138.27
136.94
134.08
133.93
133.81
133.63
132.14
131.39
130.40
130.28
130.06
129.73
129.38
129.00
128.68
128.61
128.53
128.41
126.79
126.78
126.56
126.44
126.09
114.04
101.52
86.27
79.47
73.49
70.95
70.84
70.10
69.87
55.53
30.26
30.11
29.95
29.80
29.64
29.49
29.33
21.11
70.95
70.84
70.10
69.87
210 200 190 180 170 160 150 140 130 120 110 100 90 80 70 60 50 40 30 20 10 ppm

Compound **23**

^{1}H-NMR

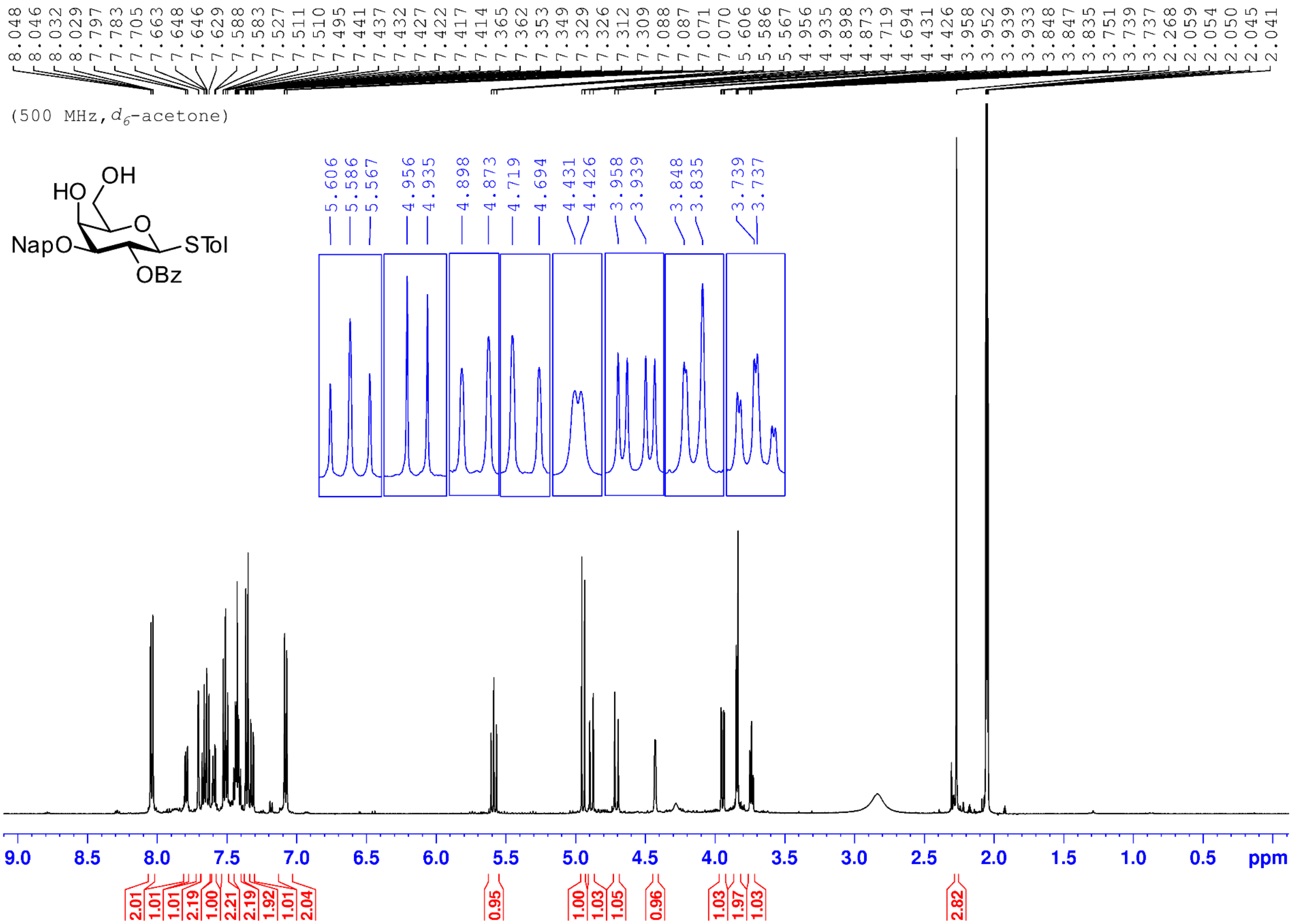

^1H-^1H COSY

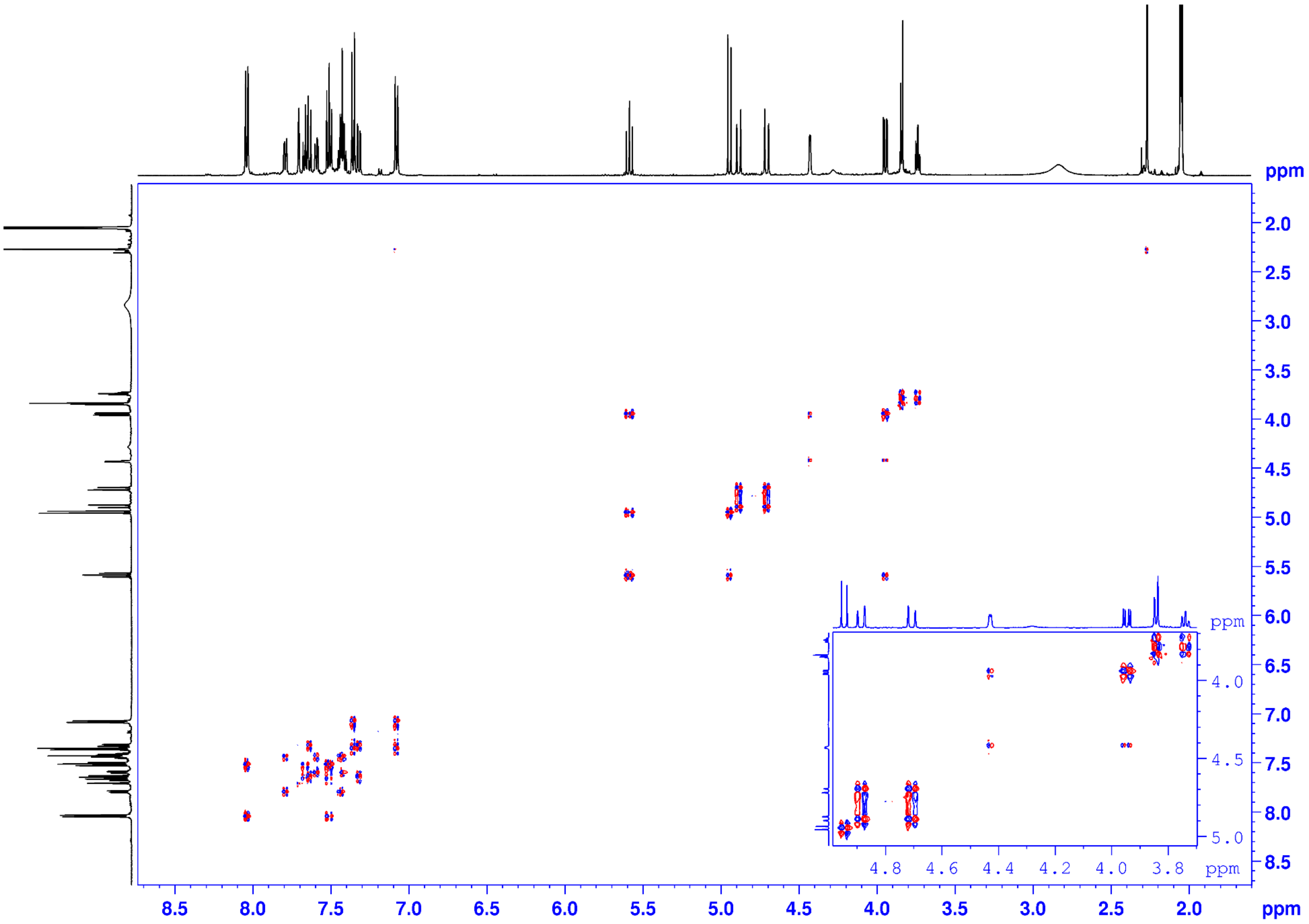

ppm
2.0
2.5
3.0
3.5
4.0
4.5
5.0
5.5
6.0
6.5
7.0
7.5
8.0
8.5
8.5
8.0
7.5
7.0
6.5
6.0
5.5
5.0
4.5
4.0
3.5
3.0
2.5
2.0
ppm
ppm
4.0
4.5
5.0
4.8
4.6
4.4
4.2
4.0
3.8
ppm

^{1}H-^{13}C HSQC

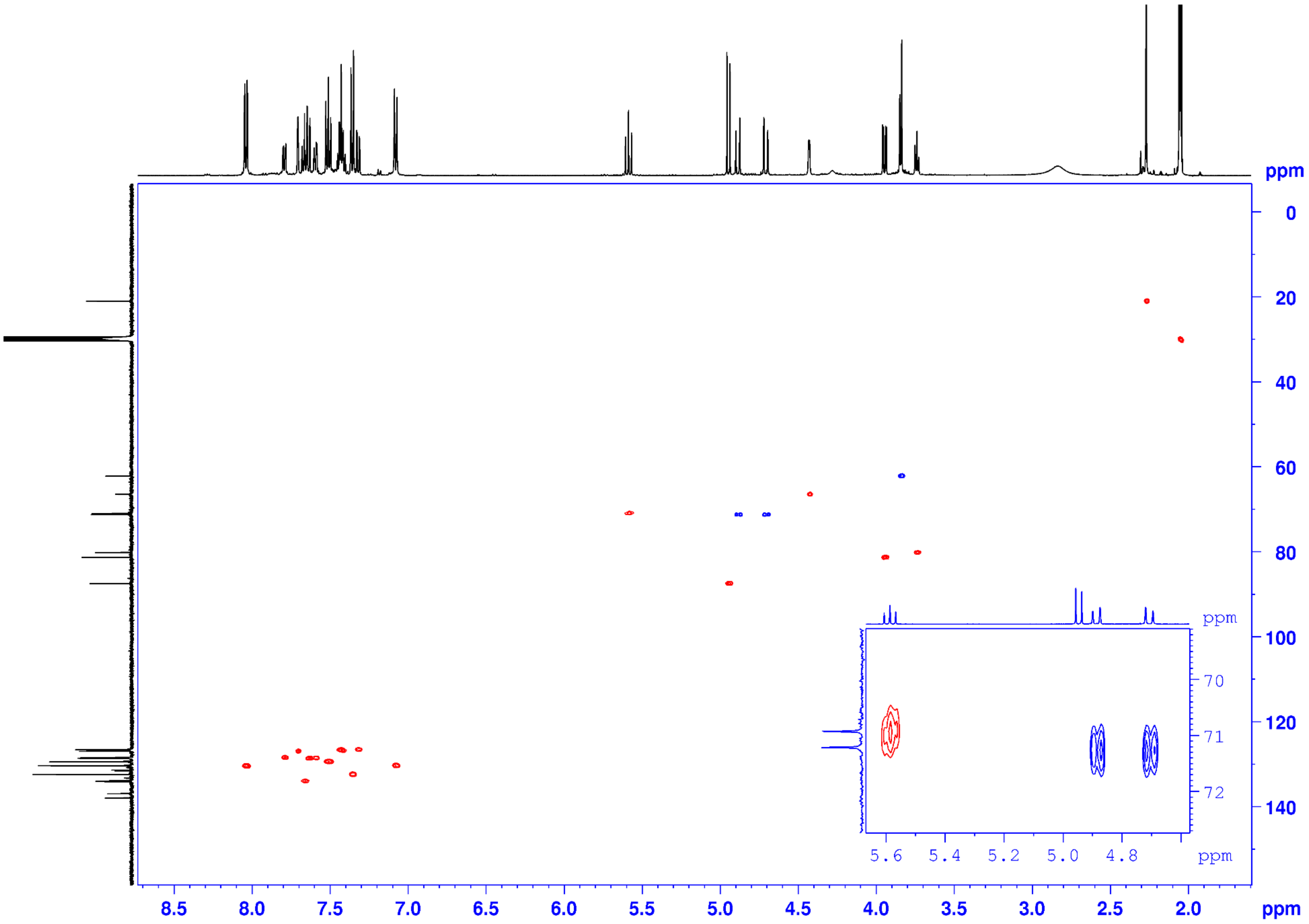

ppm
0
20
40
60
80
100
120
140
8.5
8.0
7.5
7.0
6.5
6.0
5.5
5.0
4.5
4.0
3.5
3.0
2.5
2.0
ppm
70
71
72
5.6
5.4
5.2
5.0
4.8

$^{1}H-^{13}C$ HMBC

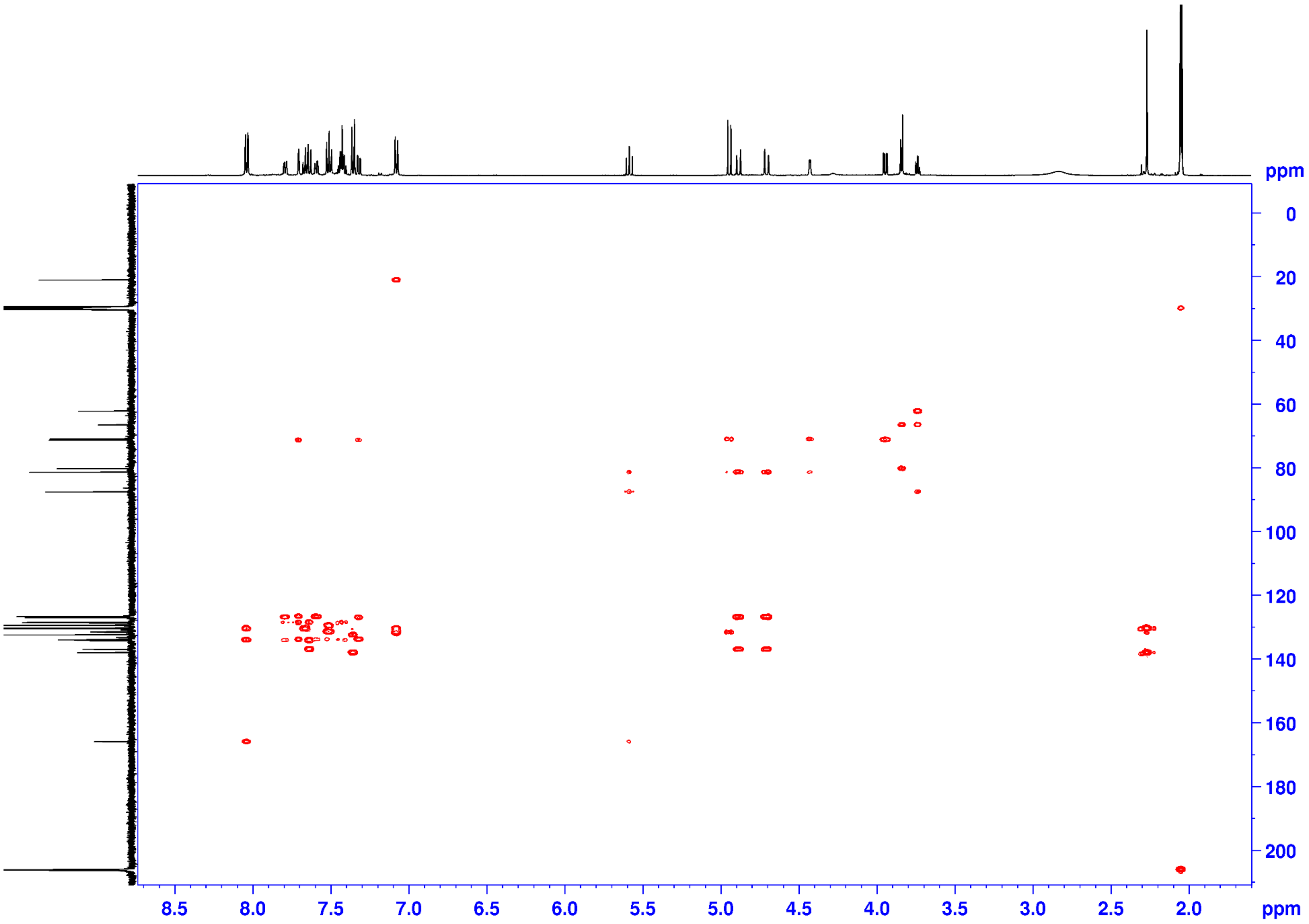

ppm
0
20
40
60
80
100
120
140
160
180
200
8.5
8.0
7.5
7.0
6.5
6.0
5.5
5.0
4.5
4.0
3.5
3.0
2.5
2.0
ppm

$^{13}C\{^{1}H\}$ NMR

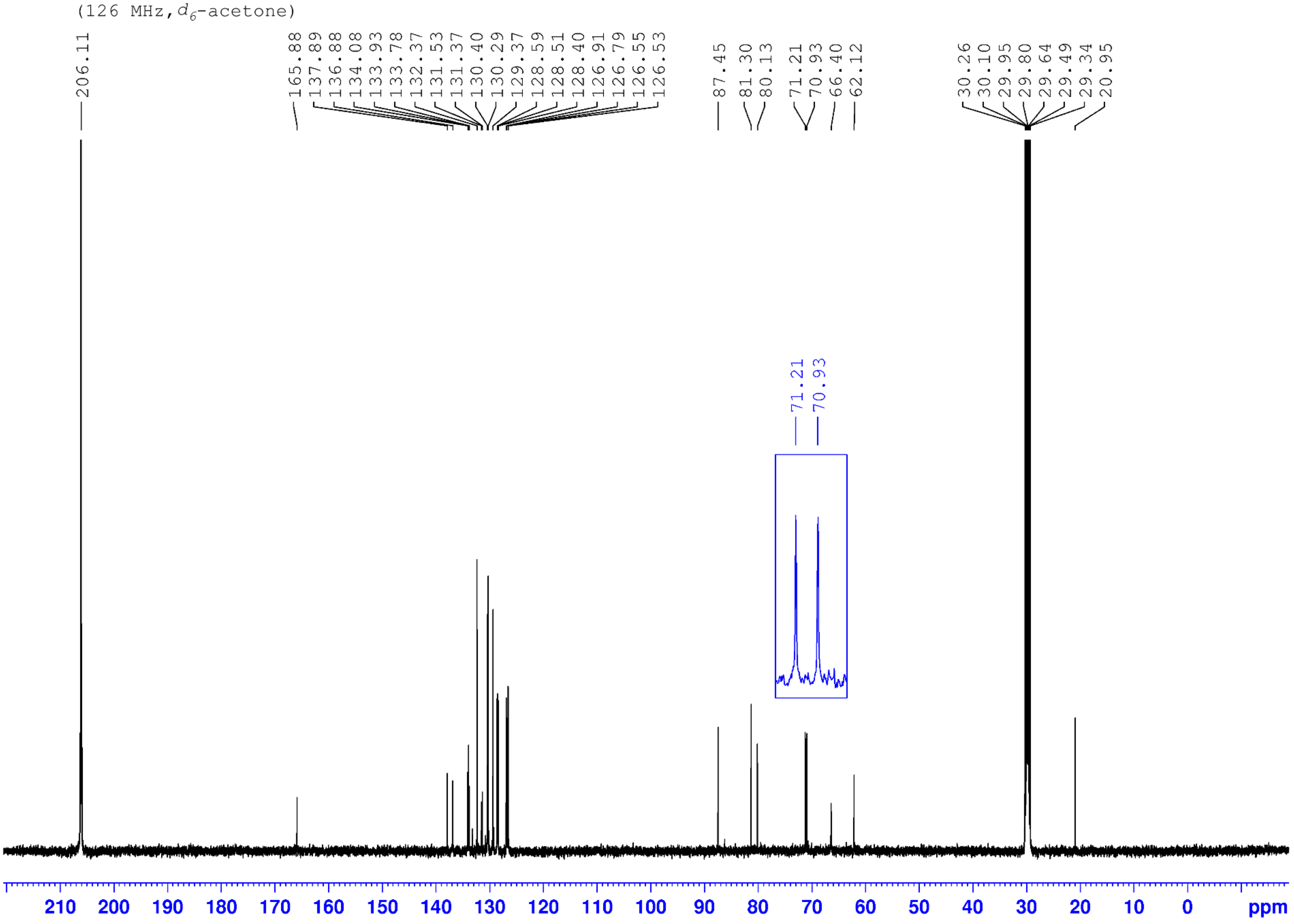

(126 MHz, d6-acetone)
206.11
165.88
137.89
136.88
134.08
133.93
133.78
132.37
131.53
131.37
130.40
130.29
129.37
128.59
128.51
128.40
126.91
126.79
126.55
126.53
87.45
81.30
80.13
71.21
70.93
66.40
62.12
30.26
30.10
29.95
29.80
29.64
29.49
29.34
20.95
71.21
70.93
210 200 190 180 170 160 150 140 130 120 110 100 90 80 70 60 50 40 30 20 10 0 ppm

Compound **24**

^{1}H-NMR

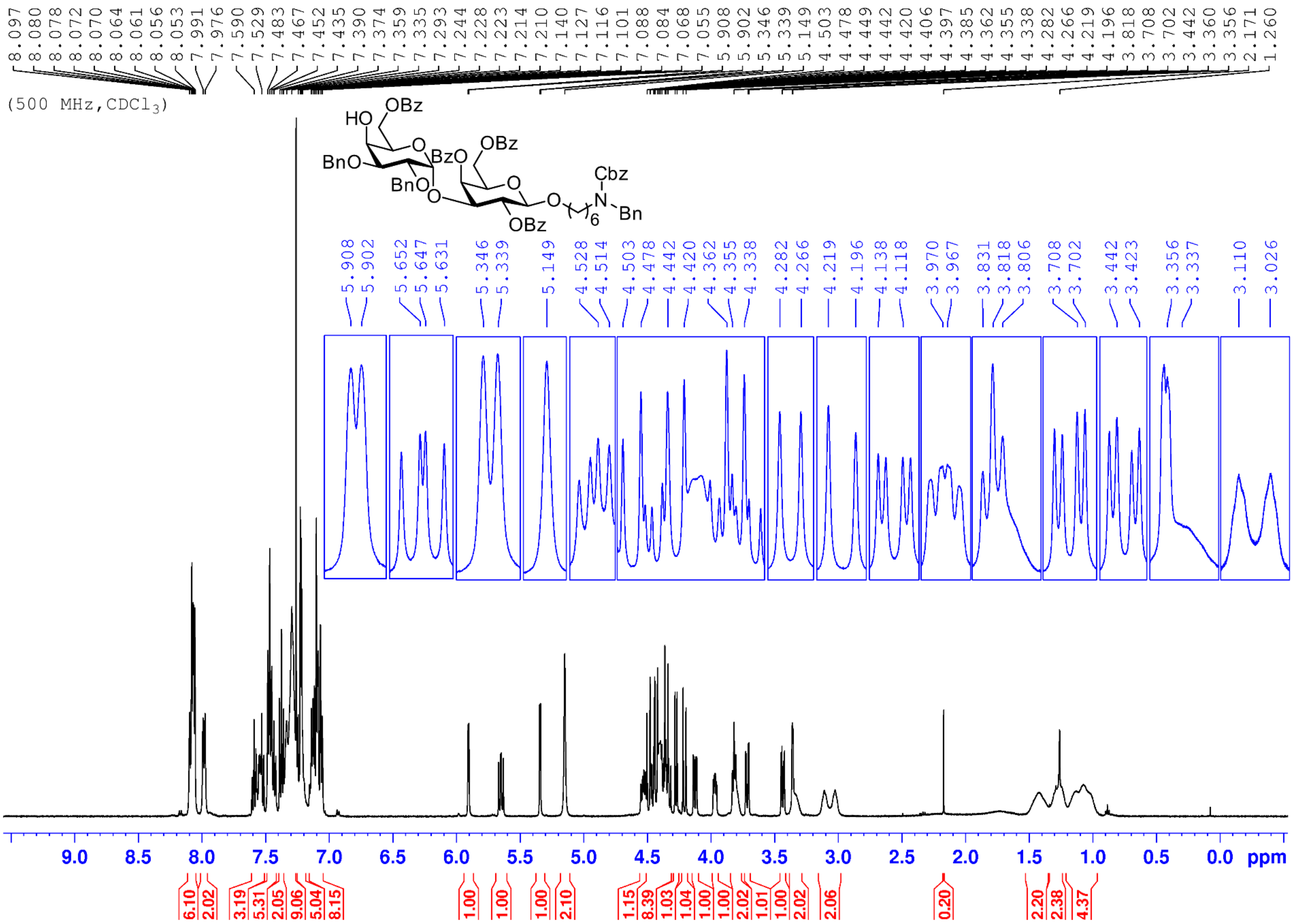

^1H-^1H COSY

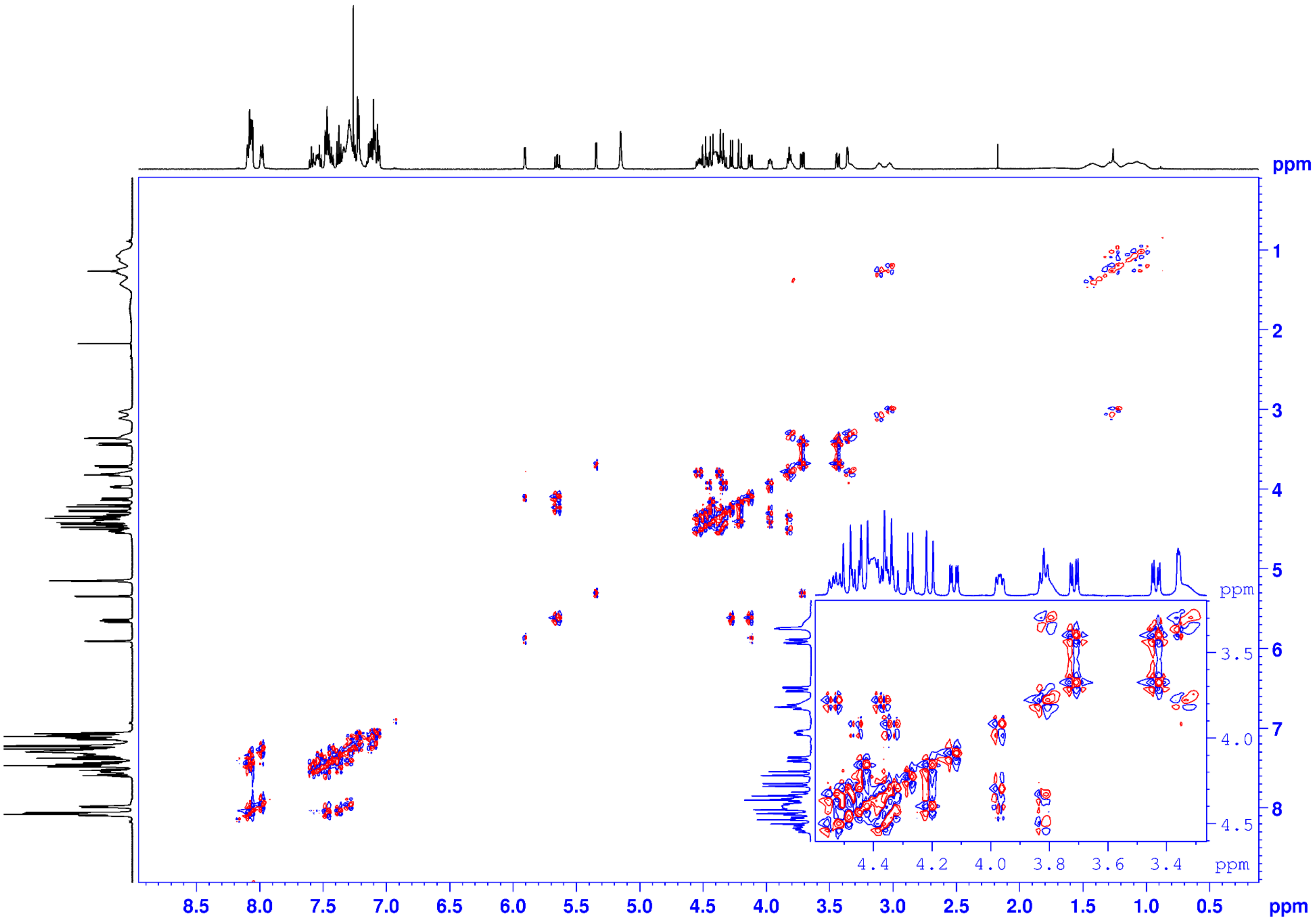

ppm
1
2
3
4
5
6
7
8
8.5
8.0
7.5
7.0
6.5
6.0
5.5
5.0
4.5
4.0
3.5
3.0
2.5
2.0
1.5
1.0
0.5
ppm
4.4
4.2
4.0
3.8
3.6
3.4
ppm
3.5
4.0
4.5
ppm

^{1}H-^{13}C HSQC

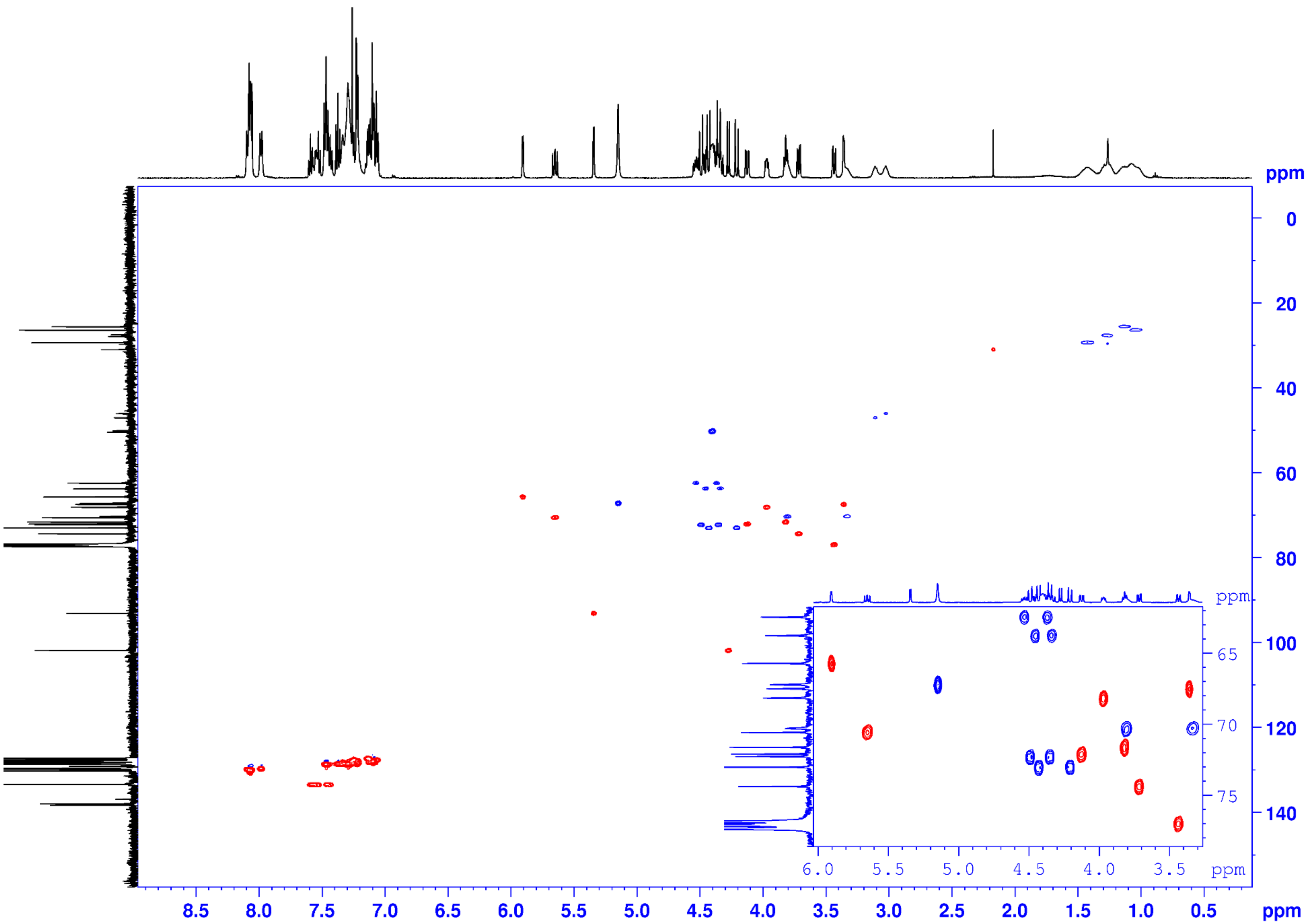

ppm
0
20
40
60
80
100
120
140
8.5
8.0
7.5
7.0
6.5
6.0
5.5
5.0
4.5
4.0
3.5
3.0
2.5
2.0
1.5
1.0
0.5
ppm
65
70
75
6.0
5.5
5.0
4.5
4.0
3.5
ppm

^{1}H-^{13}C non-decoupled HSQC

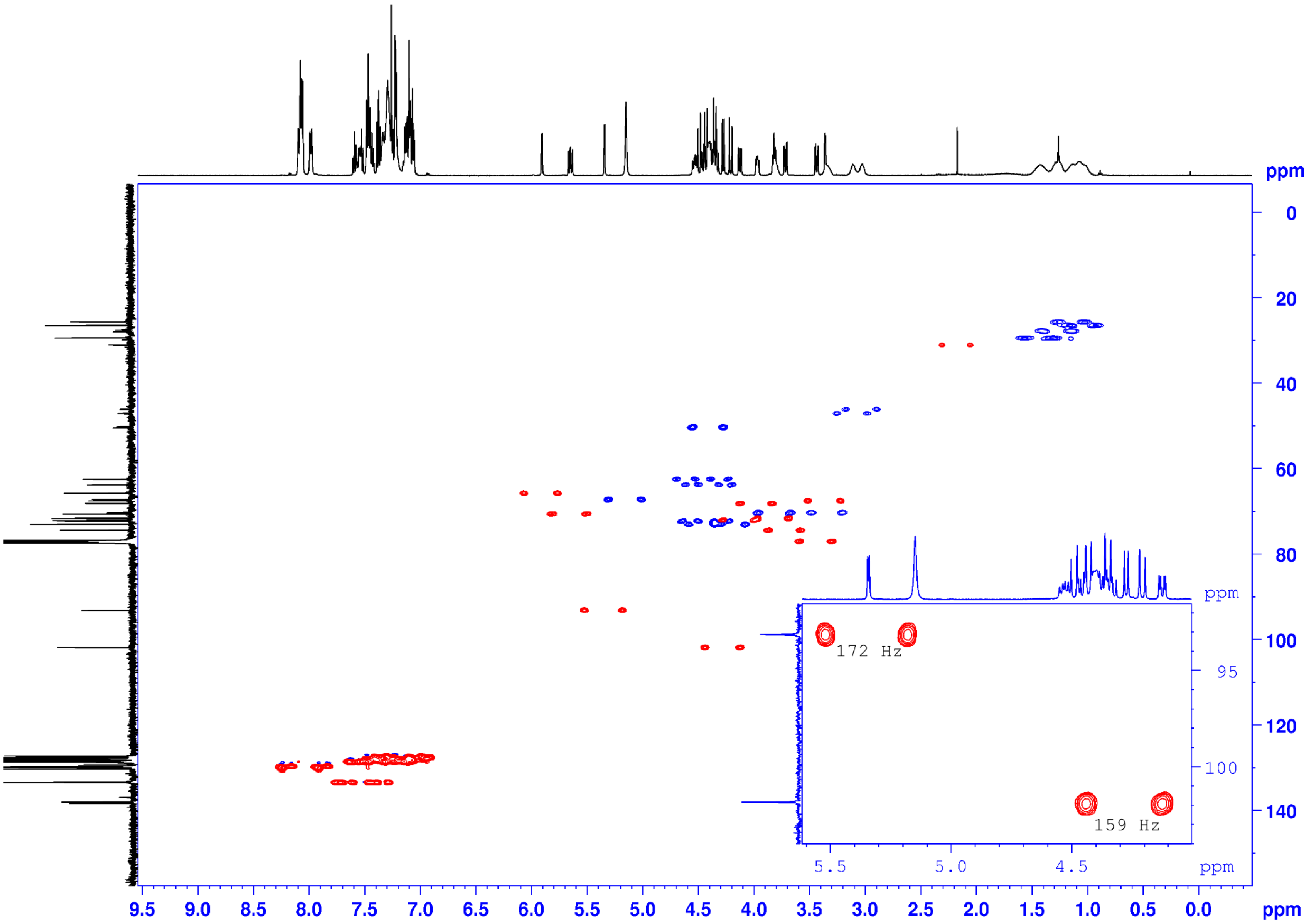

^{1}H-^{13}C HMBC

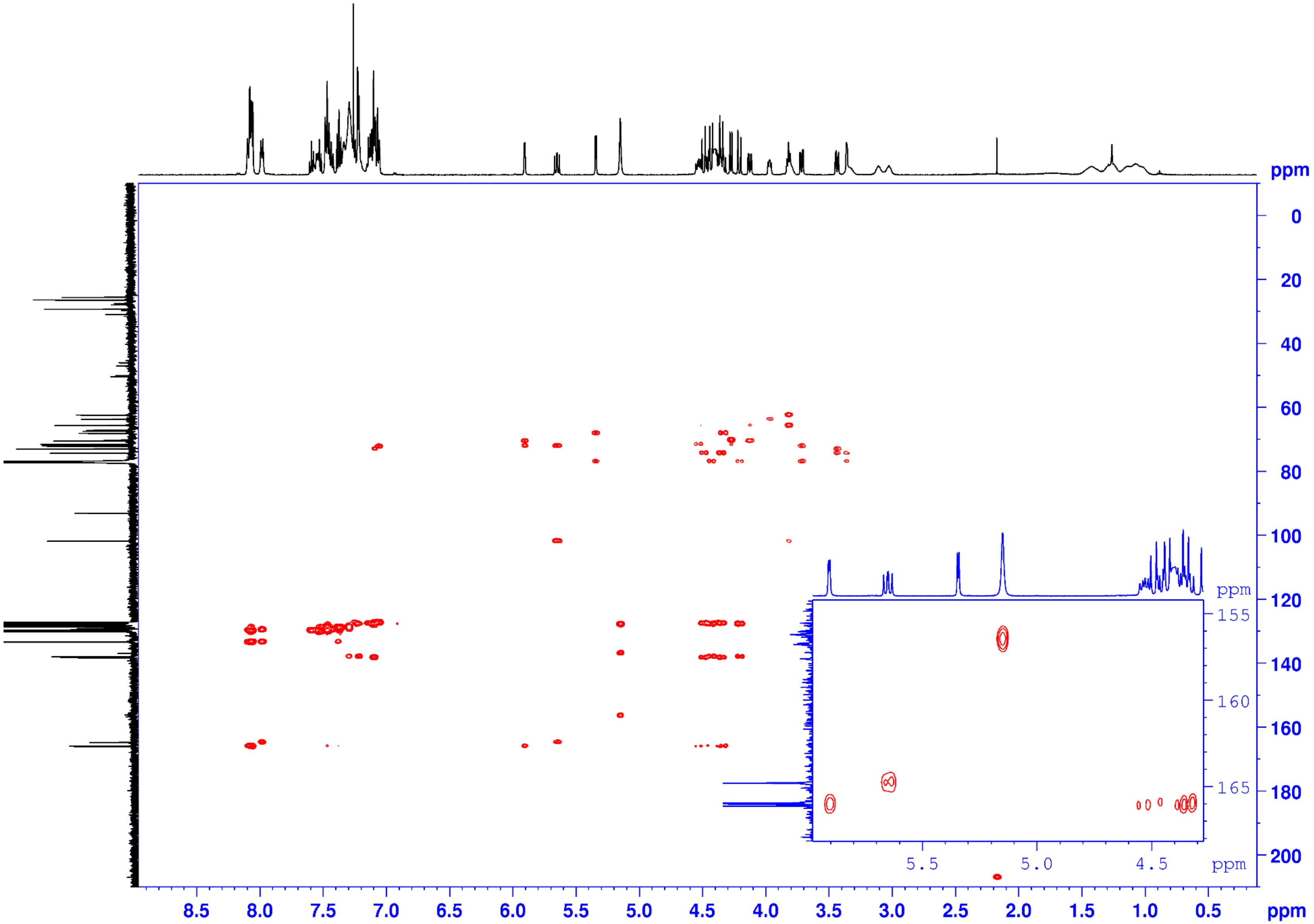

ppm
0
20
40
60
80
100
120
140
160
180
200
8.5
8.0
7.5
7.0
6.5
6.0
5.5
5.0
4.5
4.0
3.5
3.0
2.5
2.0
1.5
1.0
0.5
ppm
155
160
165
5.5
5.0
4.5

$^{13}C\{^{1}H\}$ NMR

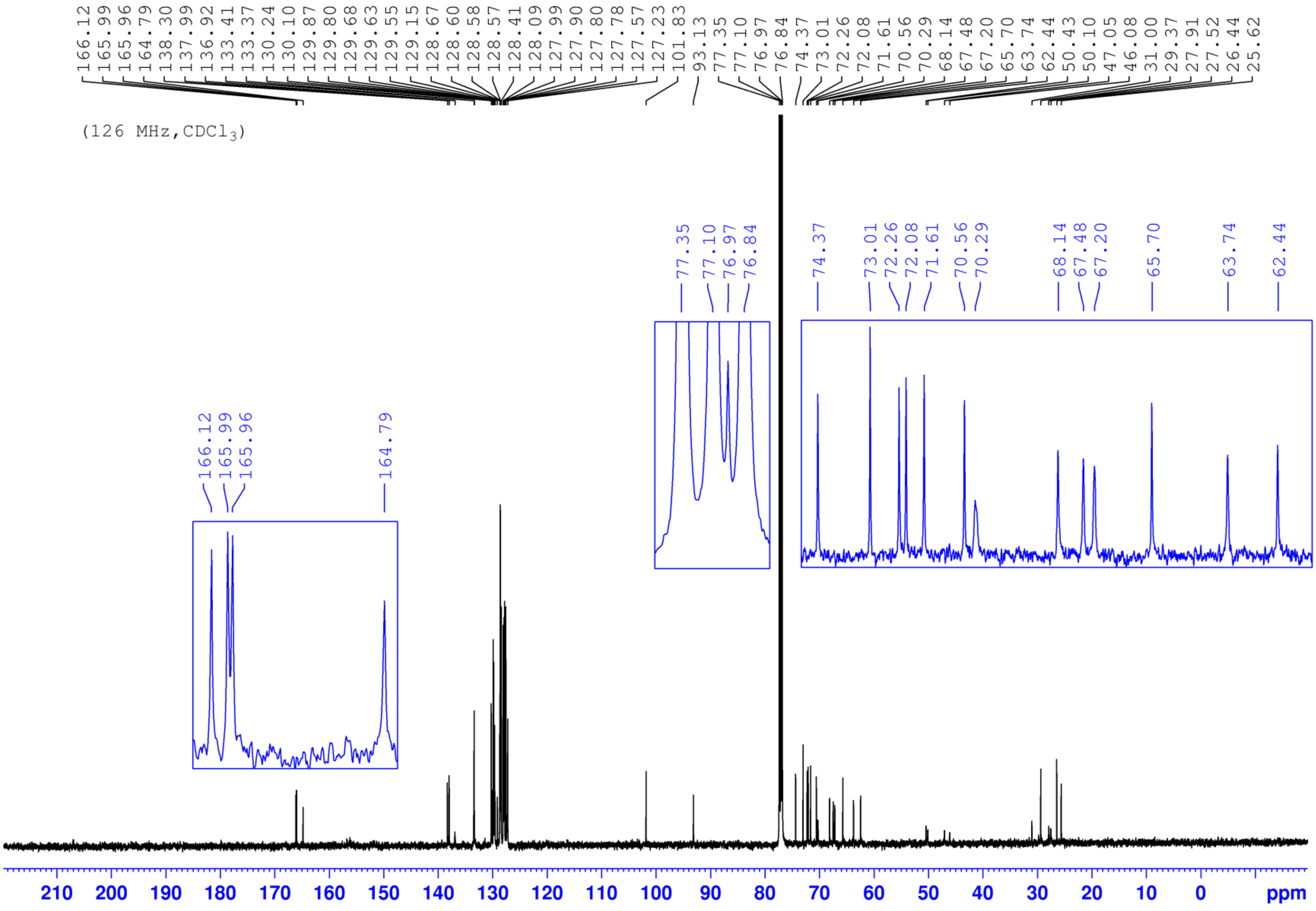

166.12
165.99
165.96
164.79
138.30
137.99
136.92
133.41
133.37
130.24
130.10
129.87
129.80
129.68
129.63
129.55
129.15
128.67
128.60
128.58
128.57
128.41
128.09
127.99
127.90
127.80
127.78
127.57
127.23
101.83
93.13
77.35
77.10
76.97
76.84
74.37
73.01
72.26
72.08
71.61
70.56
70.29
68.14
67.48
67.20
65.70
63.74
62.44
50.43
50.10
47.05
46.08
31.00
29.37
27.91
27.52
26.44
25.62
(126 MHz, CDCl3)
210 200 190 180 170 160 150 140 130 120 110 100 90 80 70 60 50 40 30 20 10 0 ppm

Compound **25**

^{1}H-NMR

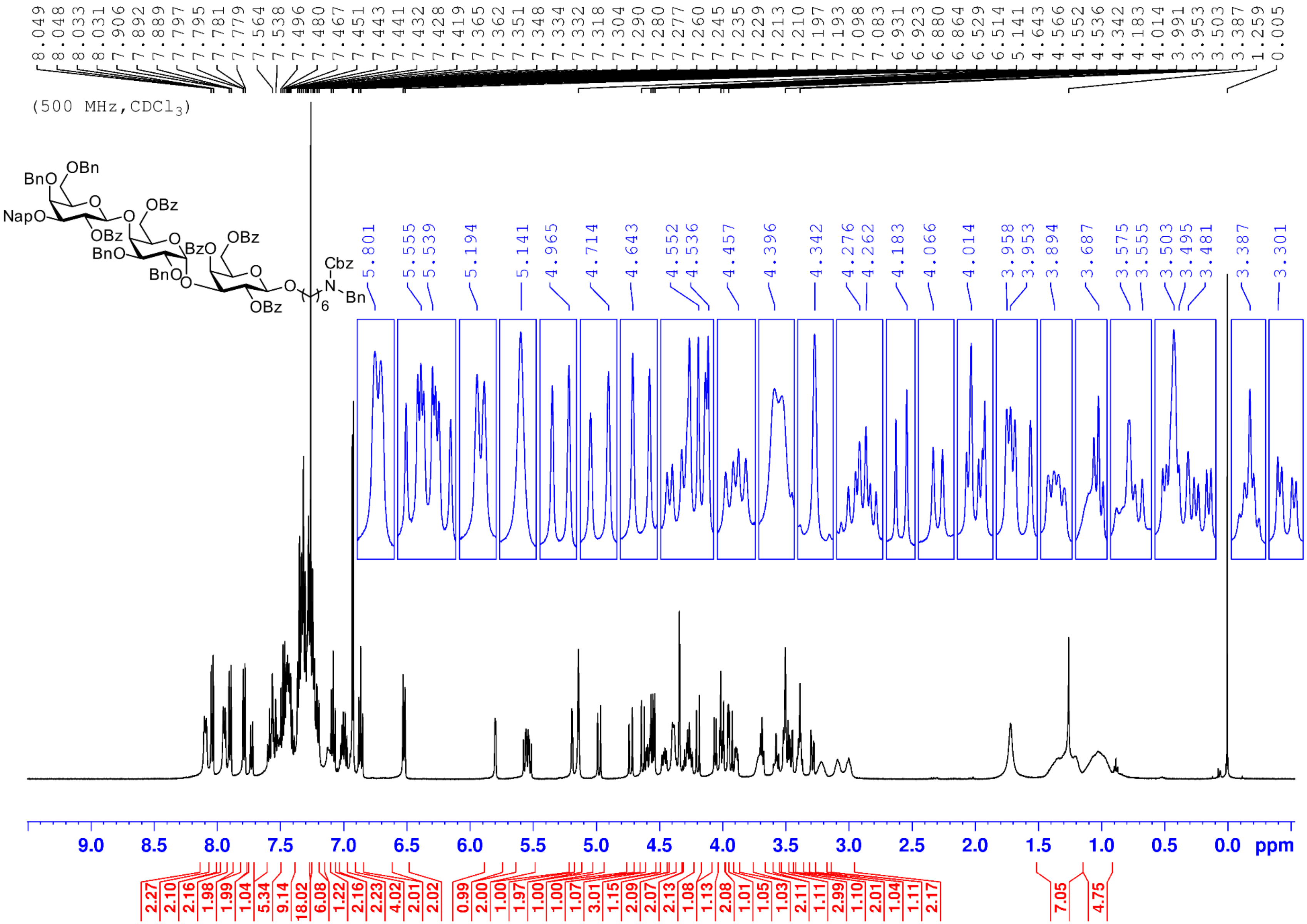

^{1}H-^{1}H COSY

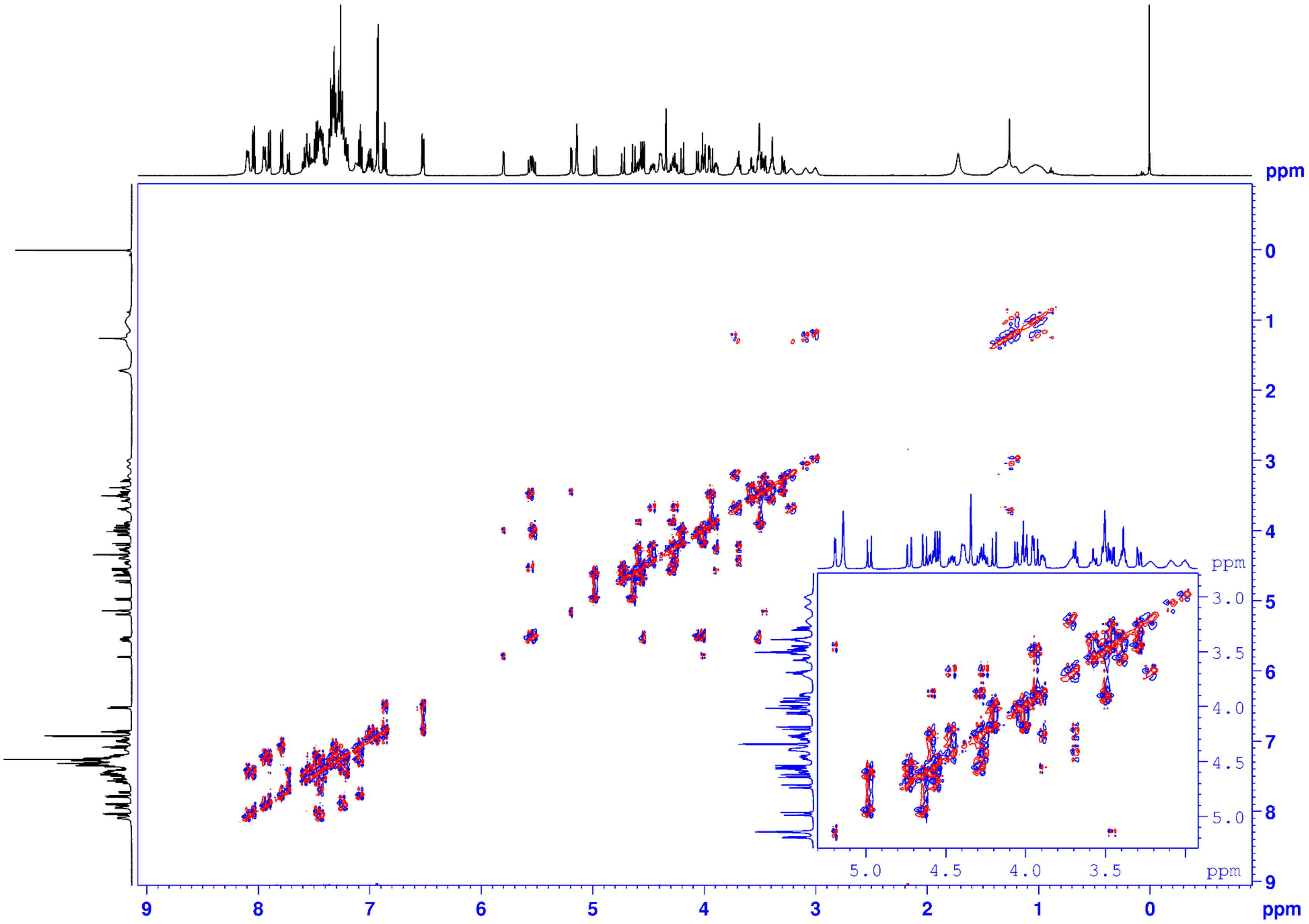

ppm
0
1
2
3
4
5
6
7
8
9
9
8
7
6
5
4
3
2
1
0
ppm
ppm
3.0
3.5
4.0
4.5
5.0
5.0
4.5
4.0
3.5
ppm

^{1}H-^{13}C HSQC

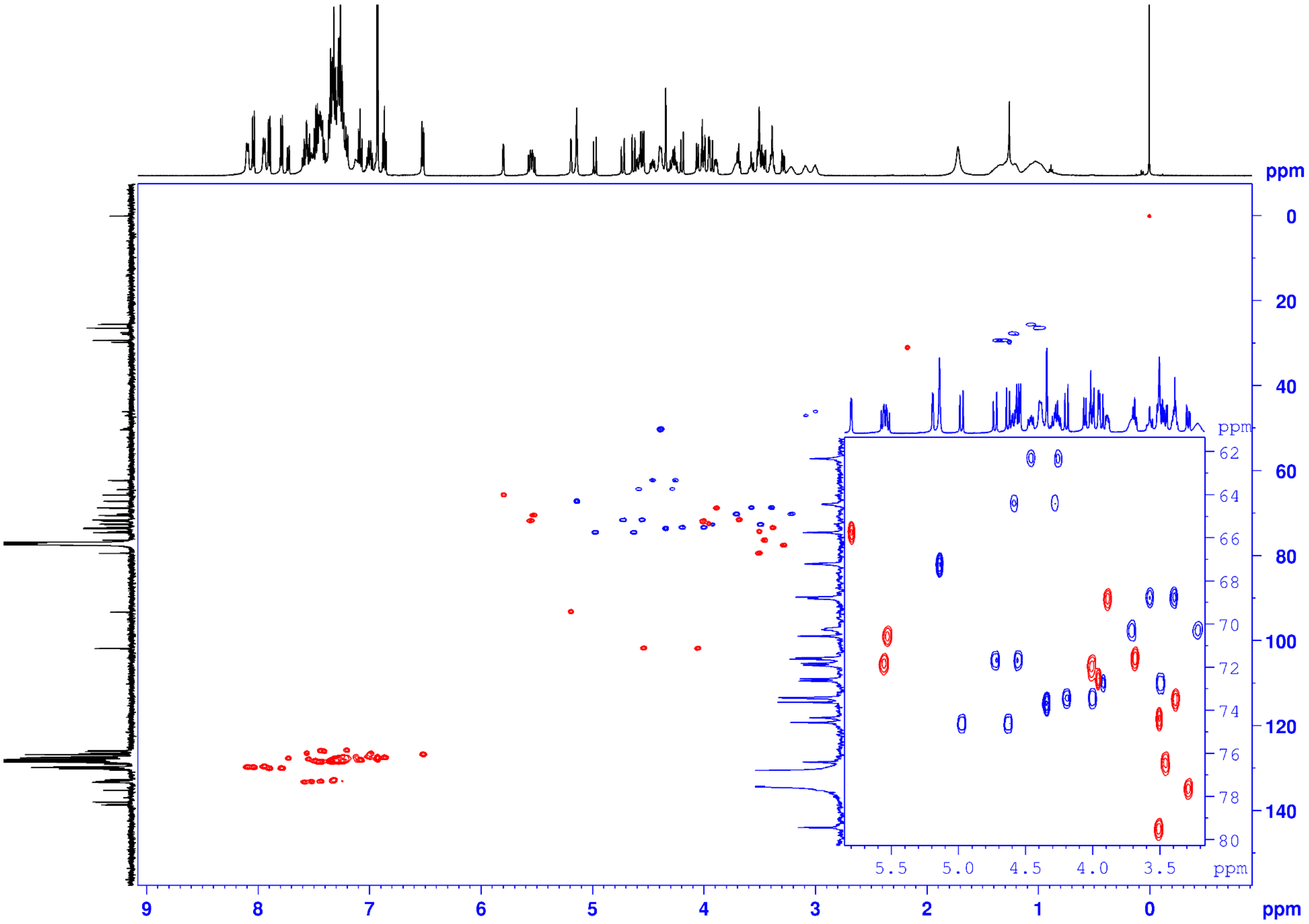
ppm
0
20
40
60
80
100
120
140
9
8
7
6
5
4
3
2
1
0
ppm
62
64
66
68
70
72
74
76
78
80
5.5
5.0
4.5
4.0
3.5
ppm

^{1}H-^{13}C non-decoupled HSQC

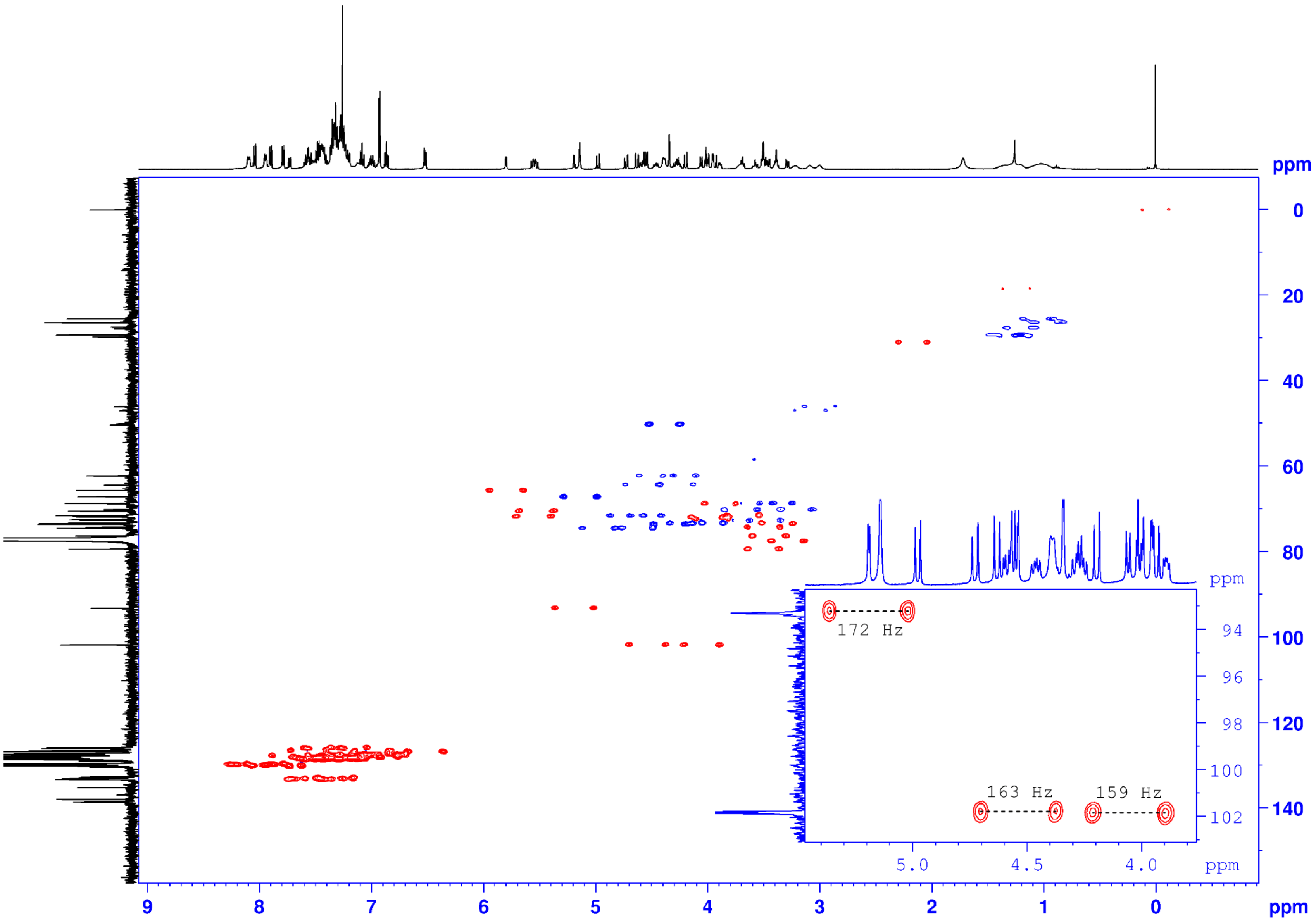

^{1}H-^{13}C HMBC

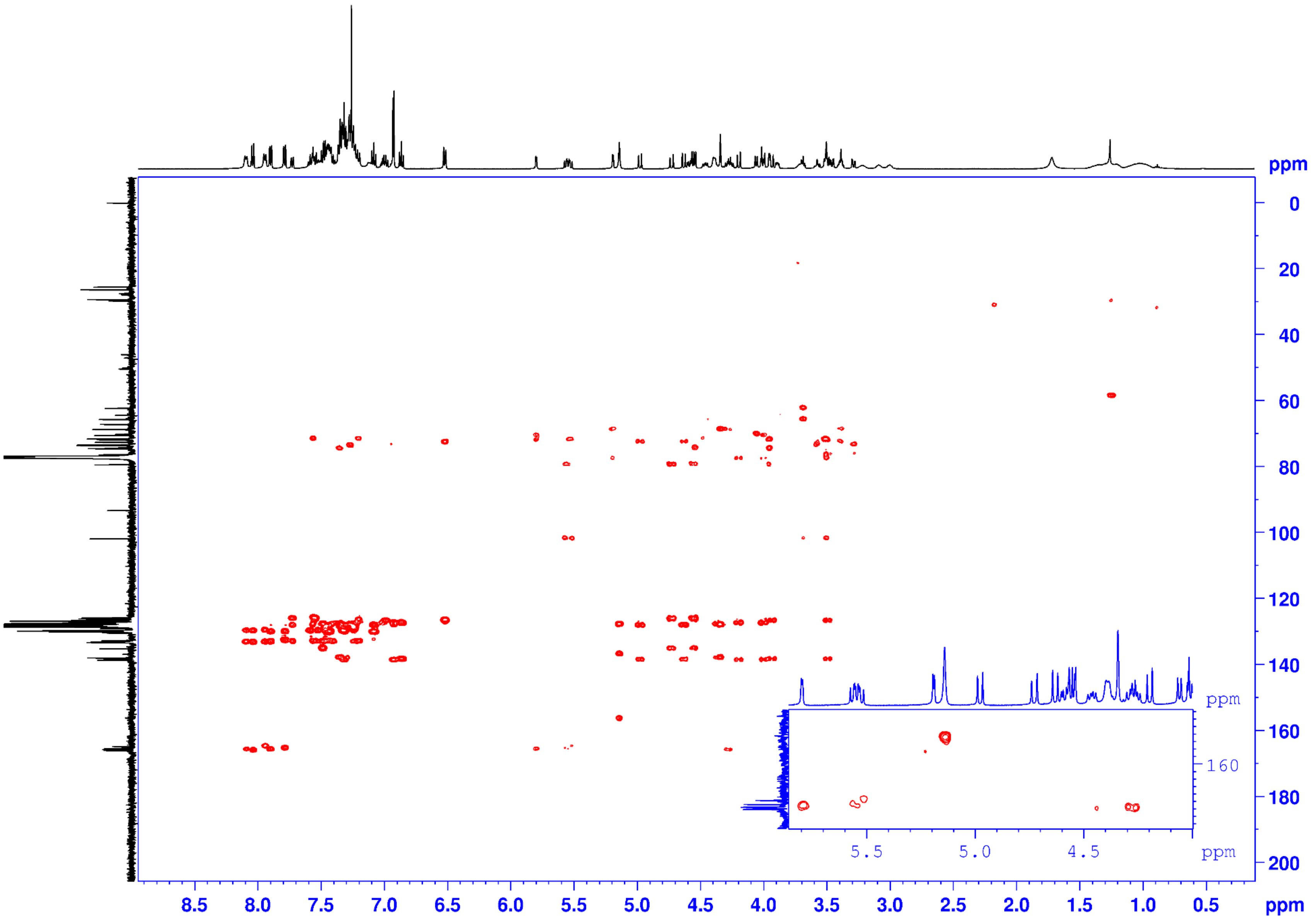

ppm
0
20
40
60
80
100
120
140
160
180
200
ppm
8.5
8.0
7.5
7.0
6.5
6.0
5.5
5.0
4.5
4.0
3.5
3.0
2.5
2.0
1.5
1.0
0.5
ppm
ppm
160
5.5
5.0
4.5
ppm

$^{13}C\{^1H\}$ NMR

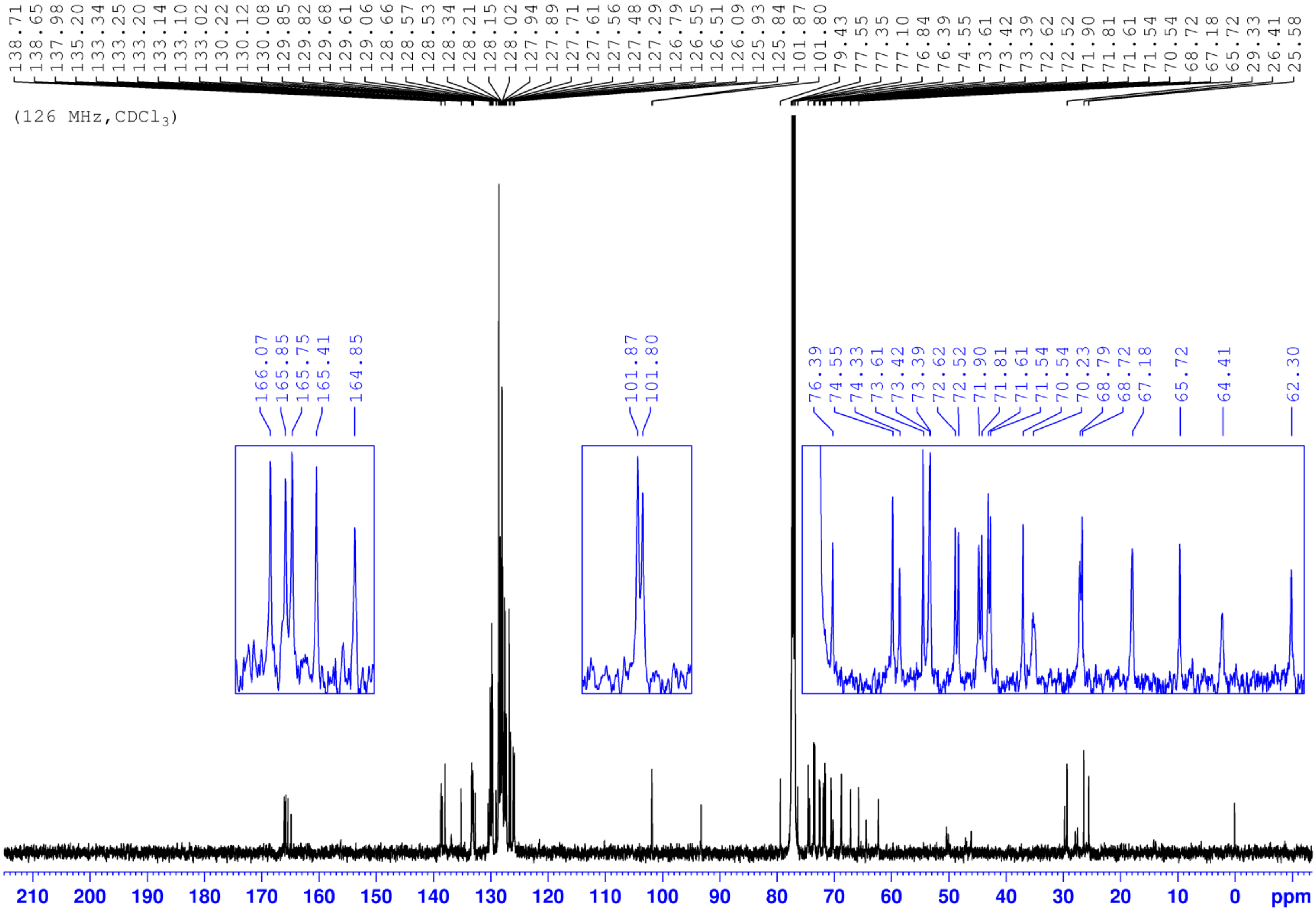

138.71
138.65
137.98
135.20
133.34
133.25
133.20
133.14
133.10
133.02
130.22
130.12
130.08
129.85
129.82
129.68
129.61
129.06
128.66
128.57
128.53
128.34
128.21
128.15
128.02
127.94
127.89
127.71
127.61
127.56
127.48
127.29
126.79
126.55
126.51
126.09
125.93
125.84
101.87
101.80
79.43
77.55
77.35
77.10
76.84
76.39
74.55
73.61
73.42
73.39
72.62
72.52
71.90
71.81
71.61
71.54
70.54
68.72
67.18
65.72
29.33
26.41
25.58
(126 MHz, CDCl3)
166.07
165.85
165.75
165.41
164.85
101.87
101.80
76.39
74.55
74.33
73.61
73.42
73.39
72.62
72.52
71.90
71.81
71.61
71.54
70.54
70.23
68.79
68.72
67.18
65.72
64.41
62.30
210
200
190
180
170
160
150
140
130
120
110
100
90
80
70
60
50
40
30
20
10
0
ppm

Compound **26a**

^{1}H-NMR

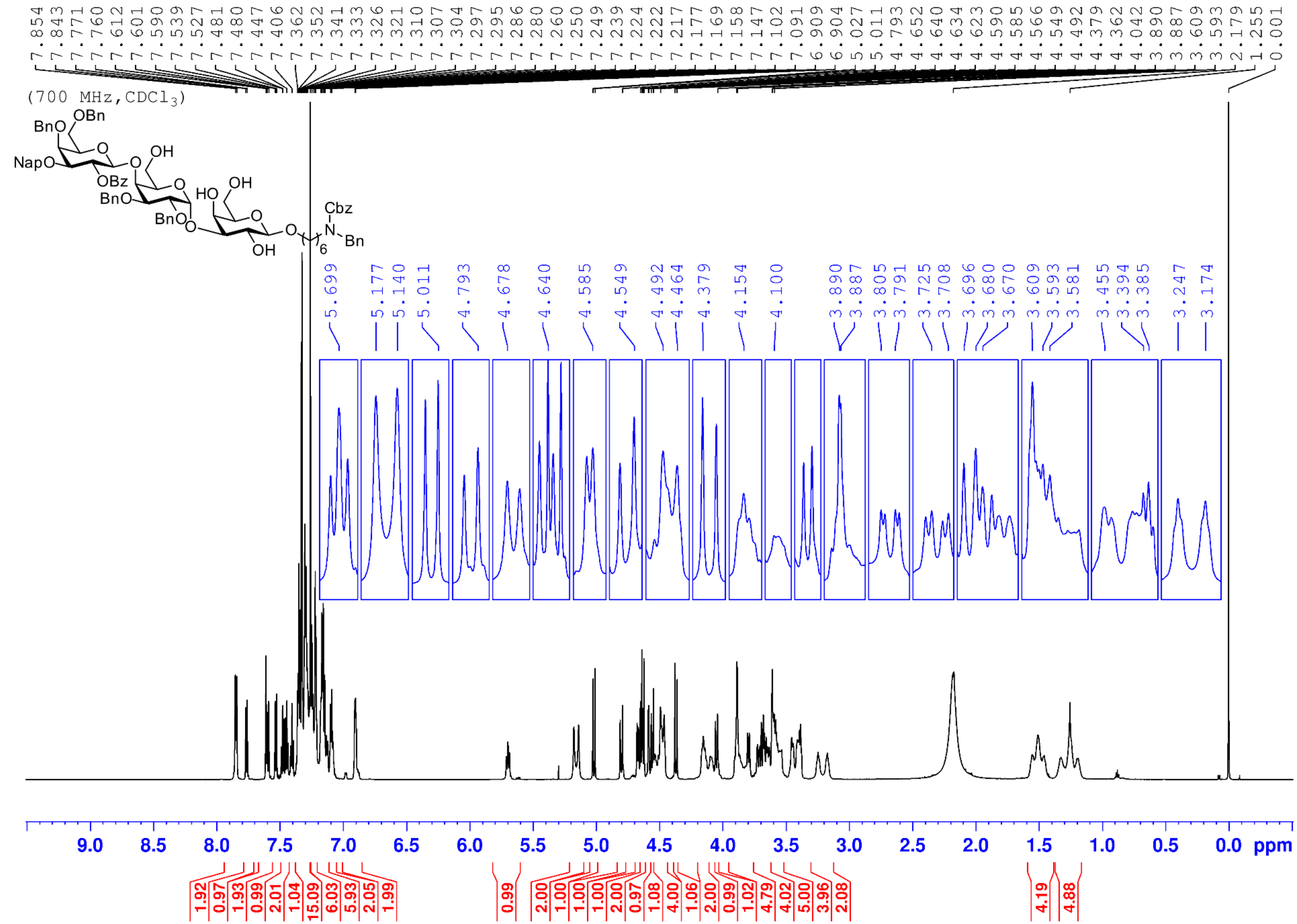

^{1}H-^{1}H COSY

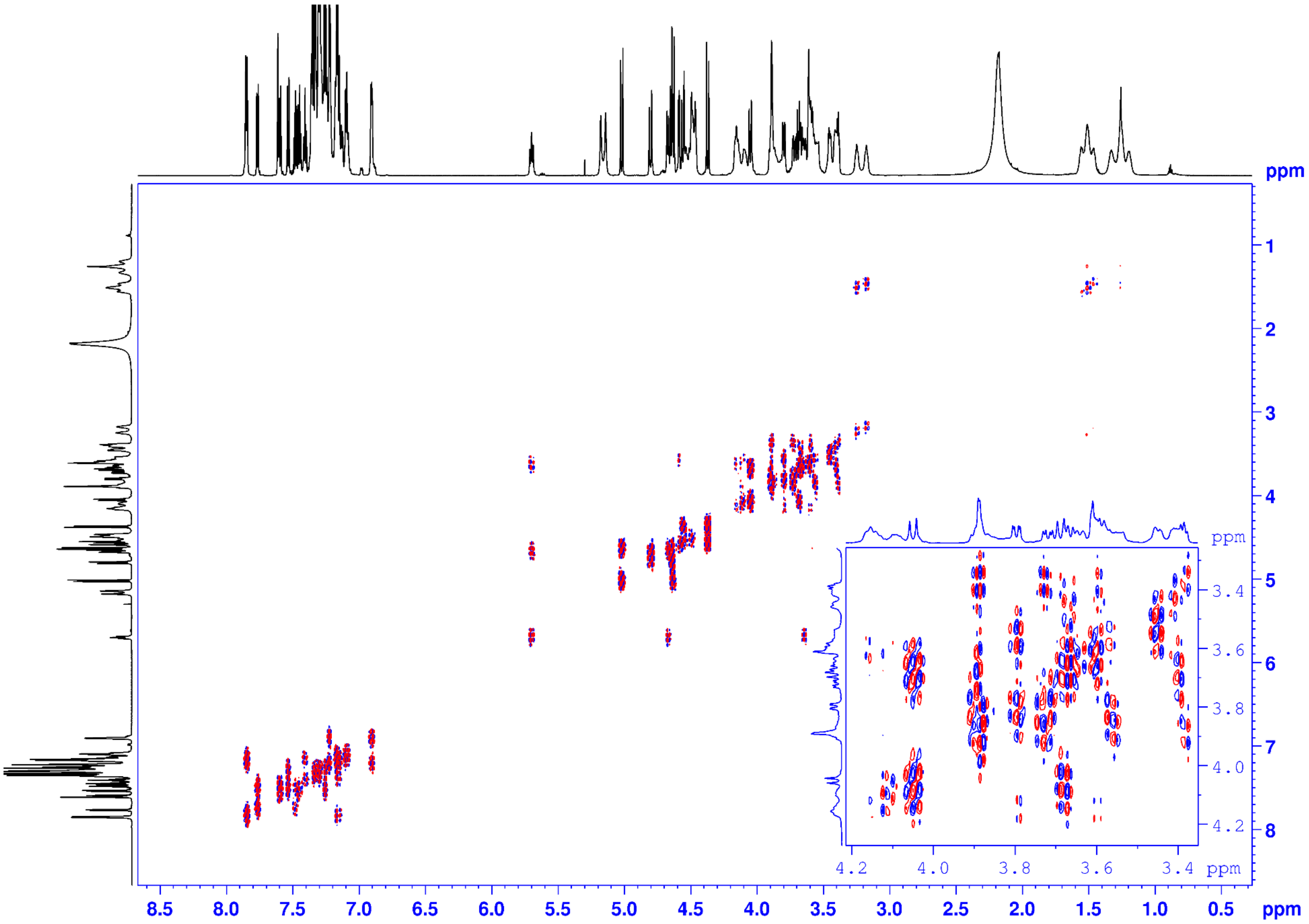
ppm
1
2
3
4
5
6
7
8
8.5 8.0 7.5 7.0 6.5 6.0 5.5 5.0 4.5 4.0 3.5 3.0 2.5 2.0 1.5 1.0 0.5 ppm
ppm
3.4
3.6
3.8
4.0
4.2
4.2 4.0 3.8 3.6 3.4 ppm

^{1}H-^{13}C HSQC

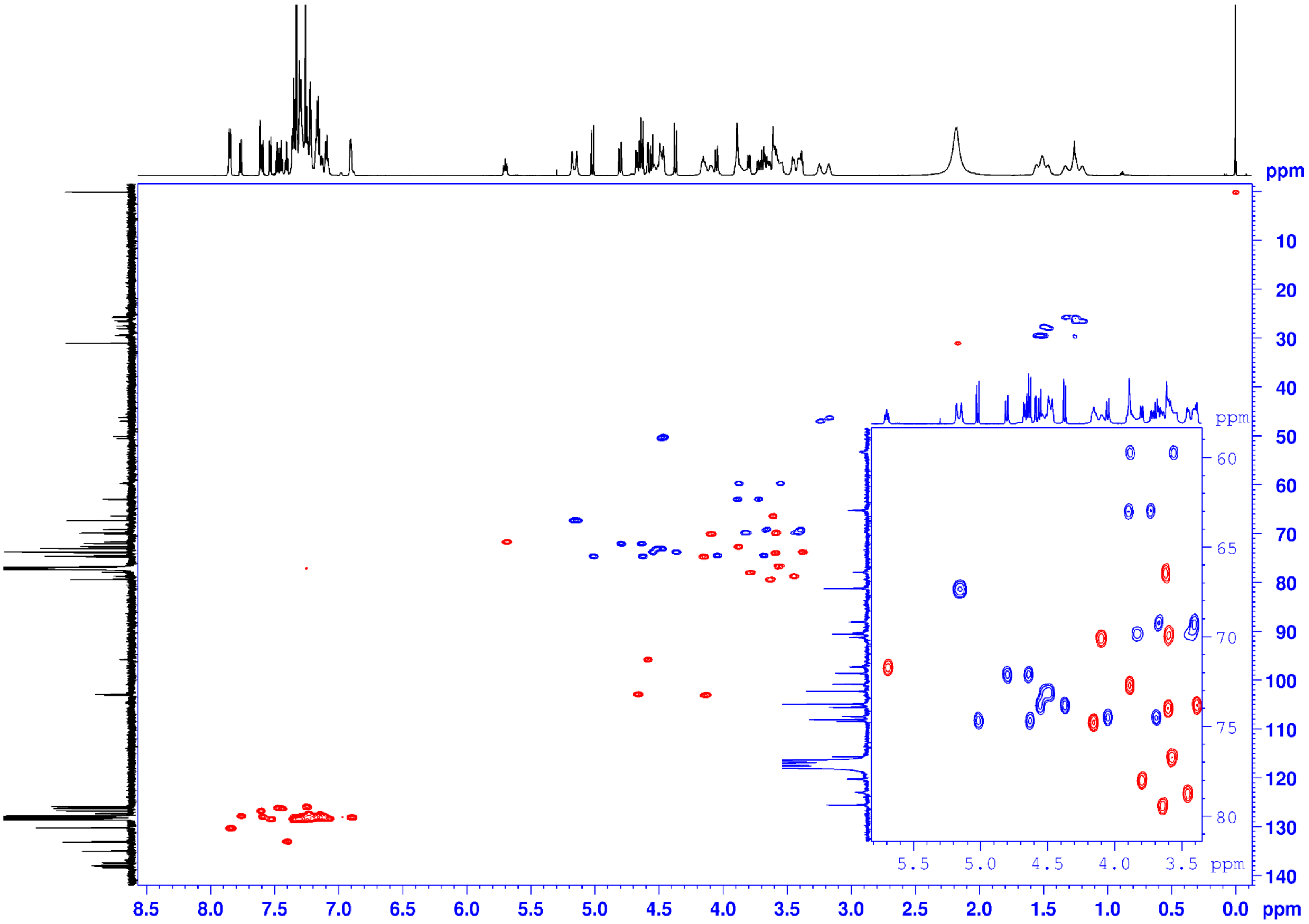
ppm
10
20
30
40
50
60
70
80
90
100
110
120
130
140
8.5
8.0
7.5
7.0
6.5
6.0
5.5
5.0
4.5
4.0
3.5
3.0
2.5
2.0
1.5
1.0
0.5
0.0
ppm
5.5
5.0
4.5
4.0
3.5
ppm
60
65
70
75
80
ppm

^{1}H-^{13}C HMBC

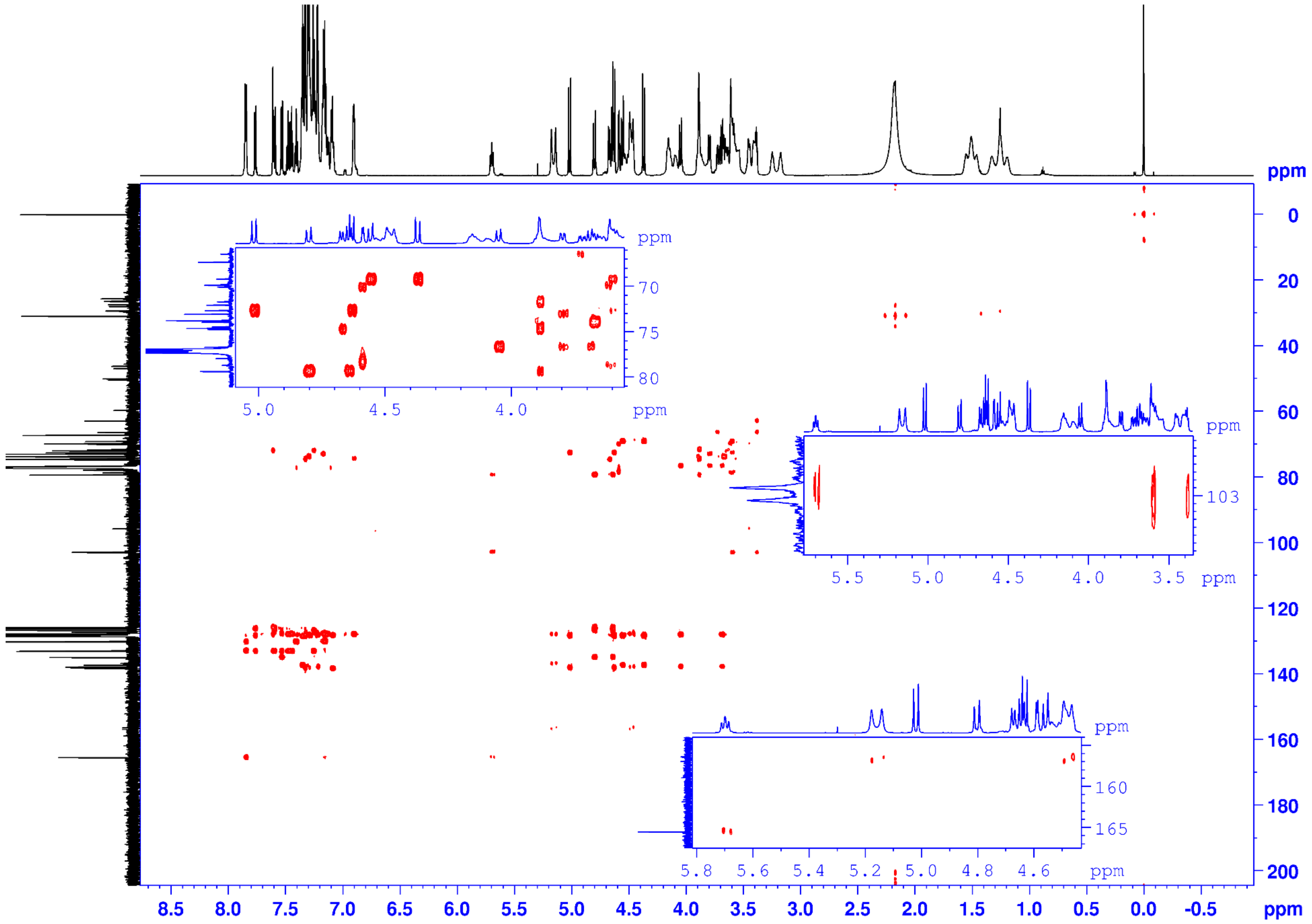
ppm
0
20
40
60
80
100
120
140
160
180
200
8.5
8.0
7.5
7.0
6.5
6.0
5.5
5.0
4.5
4.0
3.5
3.0
2.5
2.0
1.5
1.0
0.5
0.0
-0.5
ppm

$^{13}C\{^1H\}$ NMR

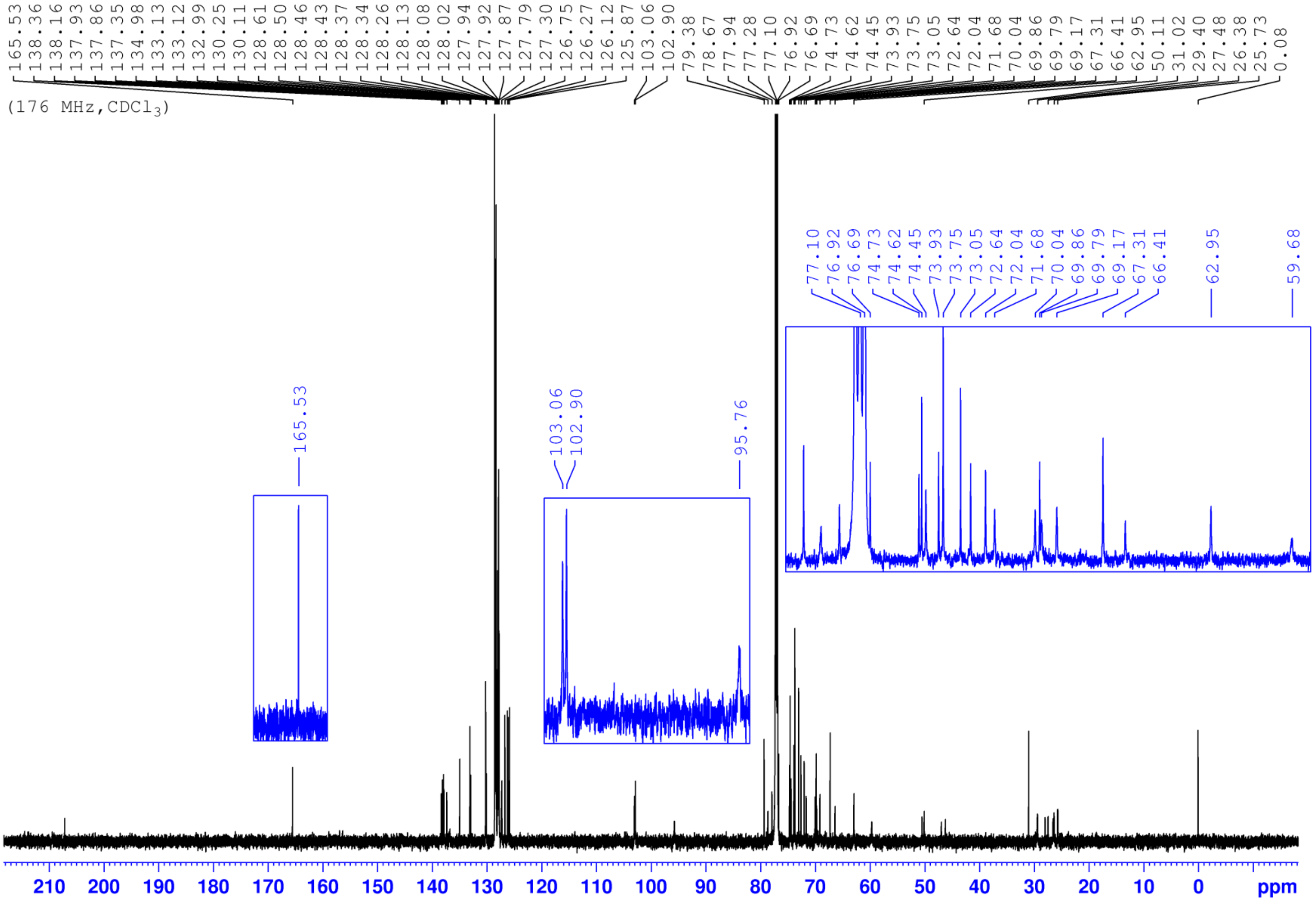
165.53
138.36
138.16
137.93
137.86
137.35
134.98
133.13
133.12
132.99
130.25
130.11
128.61
128.50
128.46
128.43
128.37
128.34
128.26
128.13
128.08
128.02
127.94
127.92
127.87
127.79
127.30
126.75
126.27
126.12
125.87
103.06
102.90
79.38
78.67
77.94
77.28
77.10
76.92
76.69
74.73
74.62
74.45
73.93
73.75
73.05
72.64
72.04
71.68
70.04
69.86
69.79
69.17
67.31
66.41
62.95
50.11
31.02
29.40
27.48
26.38
25.73
0.08
(176 MHz,CDCl3)
165.53
103.06
102.90
95.76
77.10
76.92
76.69
74.73
74.62
74.45
73.93
73.75
73.05
72.64
72.04
71.68
70.04
69.86
69.79
69.17
67.31
66.41
62.95
59.68
210
200
190
180
170
160
150
140
130
120
110
100
90
80
70
60
50
40
30
20
10
0
ppm

Compound **26b**

^{1}H-NMR

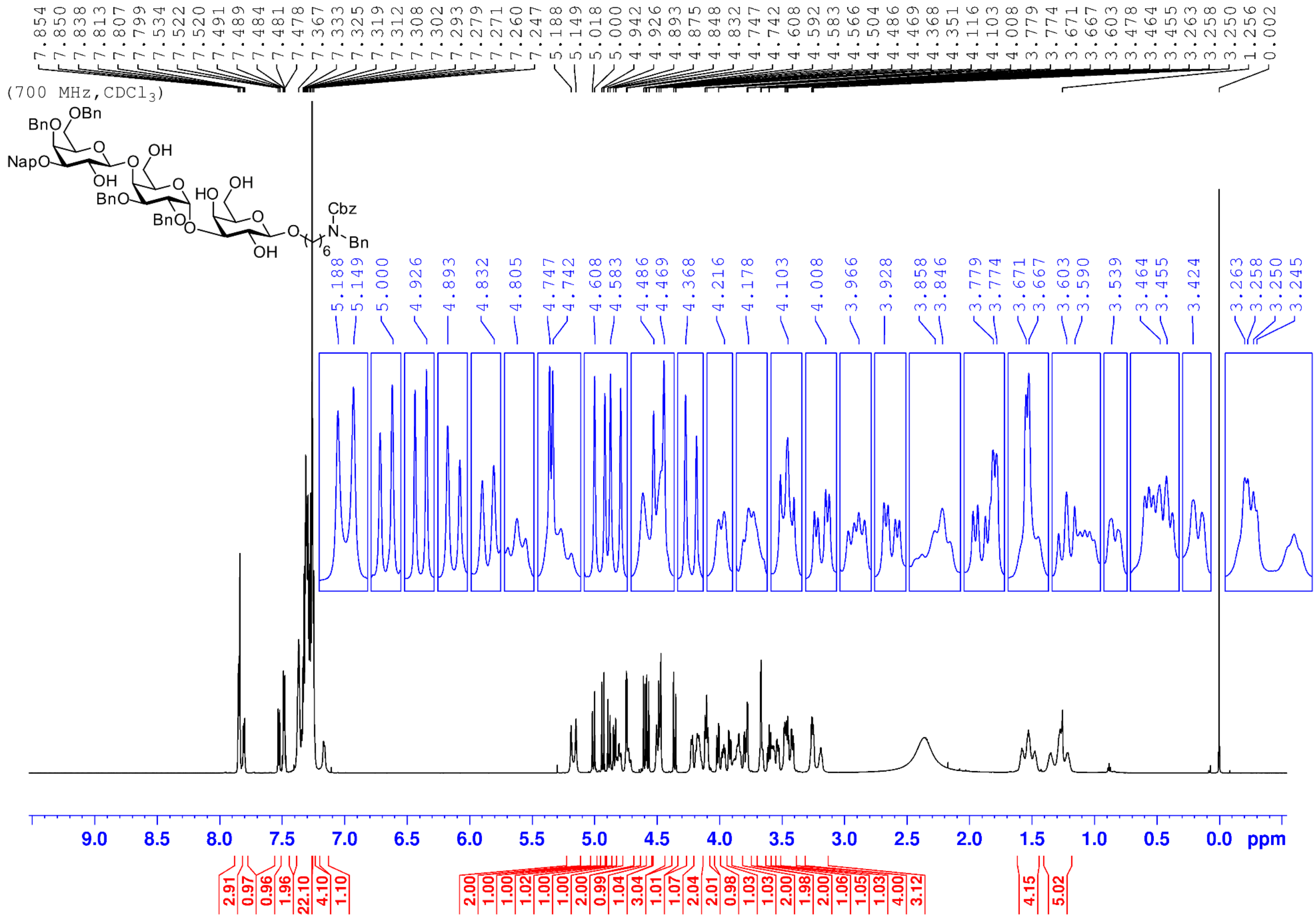

^1H-^1H COSY

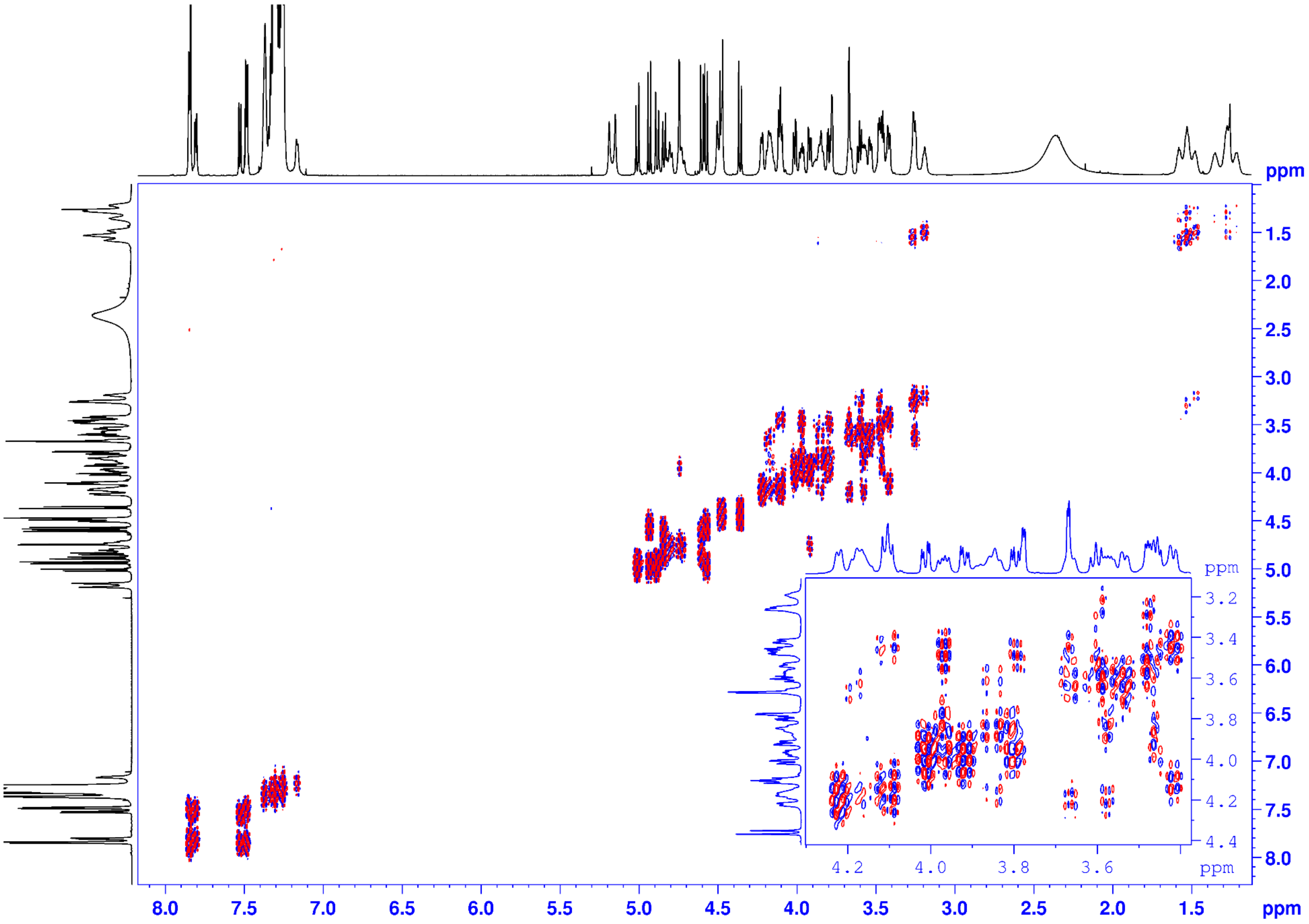

ppm
1.5
2.0
2.5
3.0
3.5
4.0
4.5
5.0
5.5
6.0
6.5
7.0
7.5
8.0
8.0 7.5 7.0 6.5 6.0 5.5 5.0 4.5 4.0 3.5 3.0 2.5 2.0 1.5 ppm
3.2
3.4
3.6
3.8
4.0
4.2
4.4
4.2 4.0 3.8 3.6 ppm

^1H-^{13}C HSQC

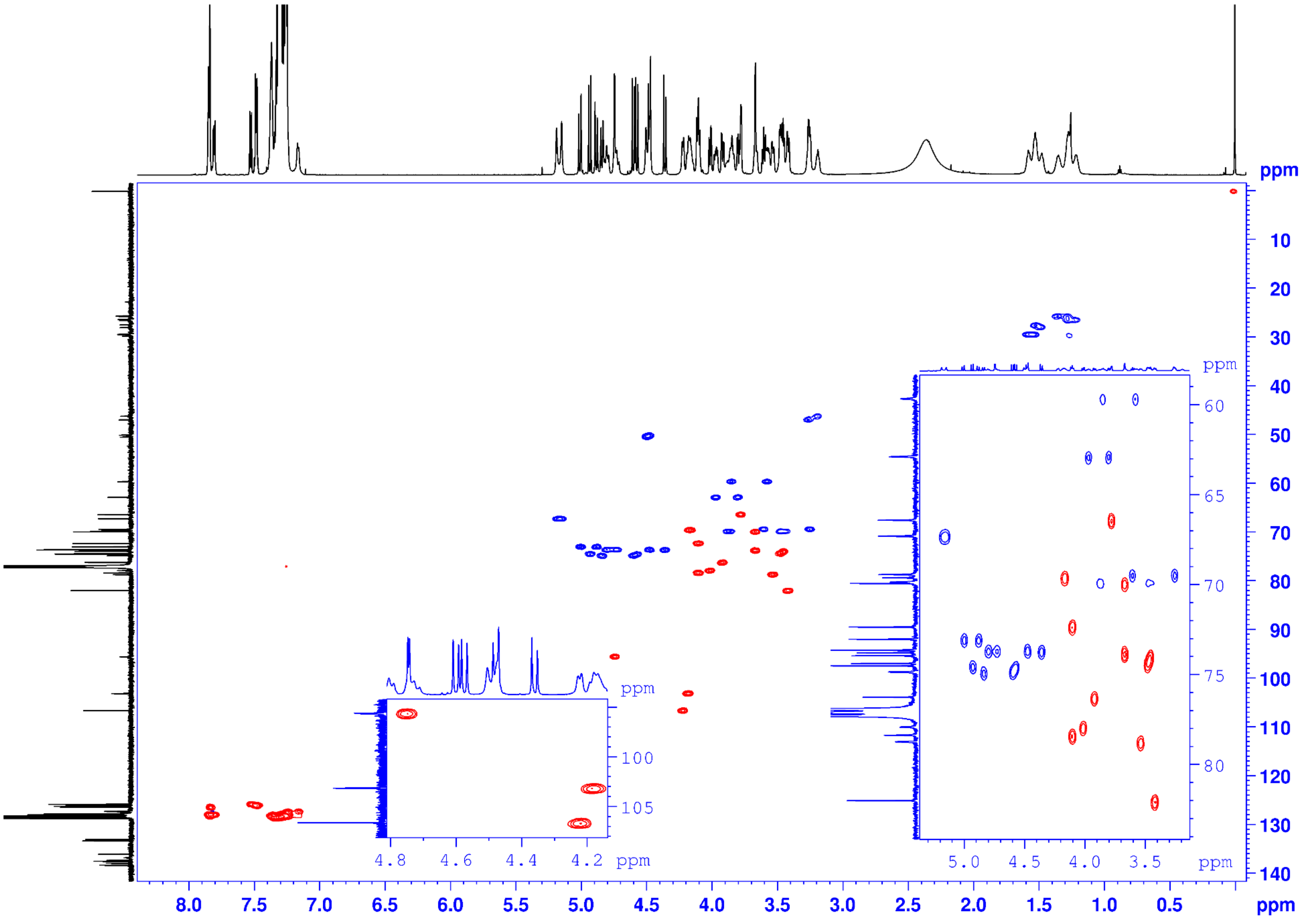

ppm
10
20
30
40
50
60
70
80
90
100
110
120
130
140
ppm
8.0
7.5
7.0
6.5
6.0
5.5
5.0
4.5
4.0
3.5
3.0
2.5
2.0
1.5
1.0
0.5
ppm
ppm
100
105
4.8
4.6
4.4
4.2
ppm
ppm
60
65
70
75
80
5.0
4.5
4.0
3.5
ppm

^{1}H-^{13}C HMBC

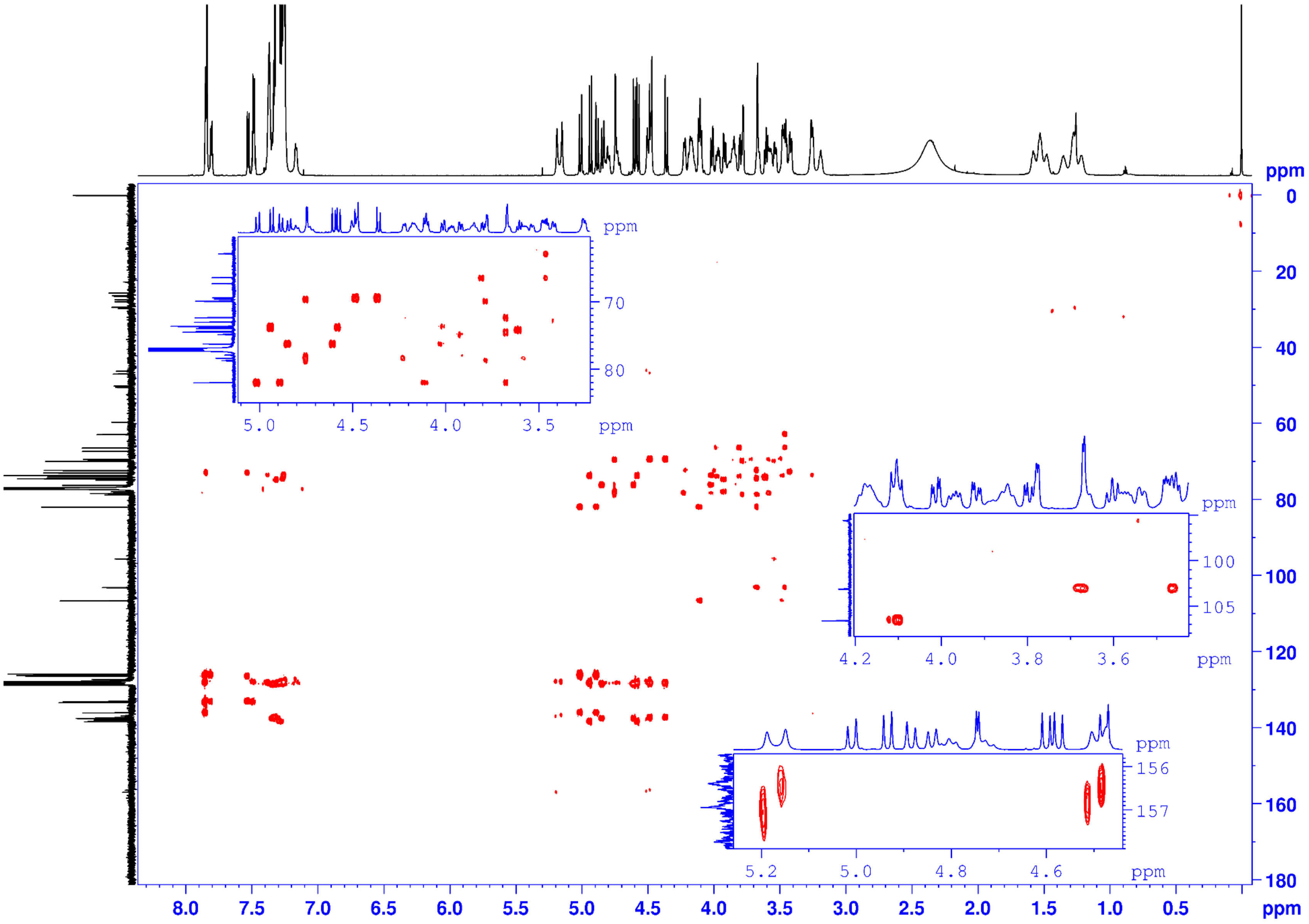

ppm
0
20
40
60
80
100
120
140
160
180
8.0
7.5
7.0
6.5
6.0
5.5
5.0
4.5
4.0
3.5
3.0
2.5
2.0
1.5
1.0
0.5
ppm
70
80
5.0
4.5
4.0
3.5
100
105
4.2
4.0
3.8
3.6
156
157
5.2
5.0
4.8
4.6

$^{13}C\{^1H\}$ NMR

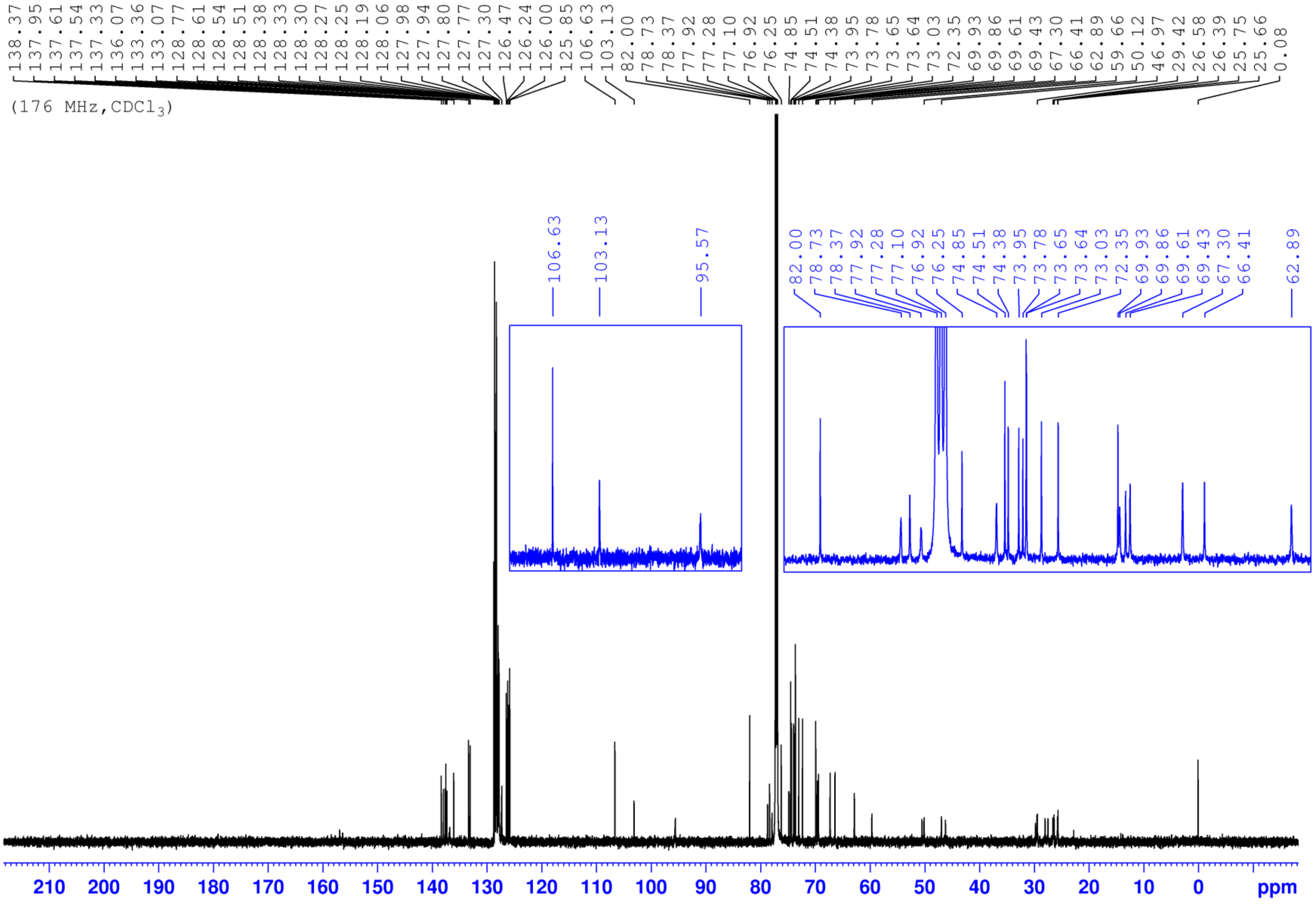

Compound **28**

^{1}H-NMR

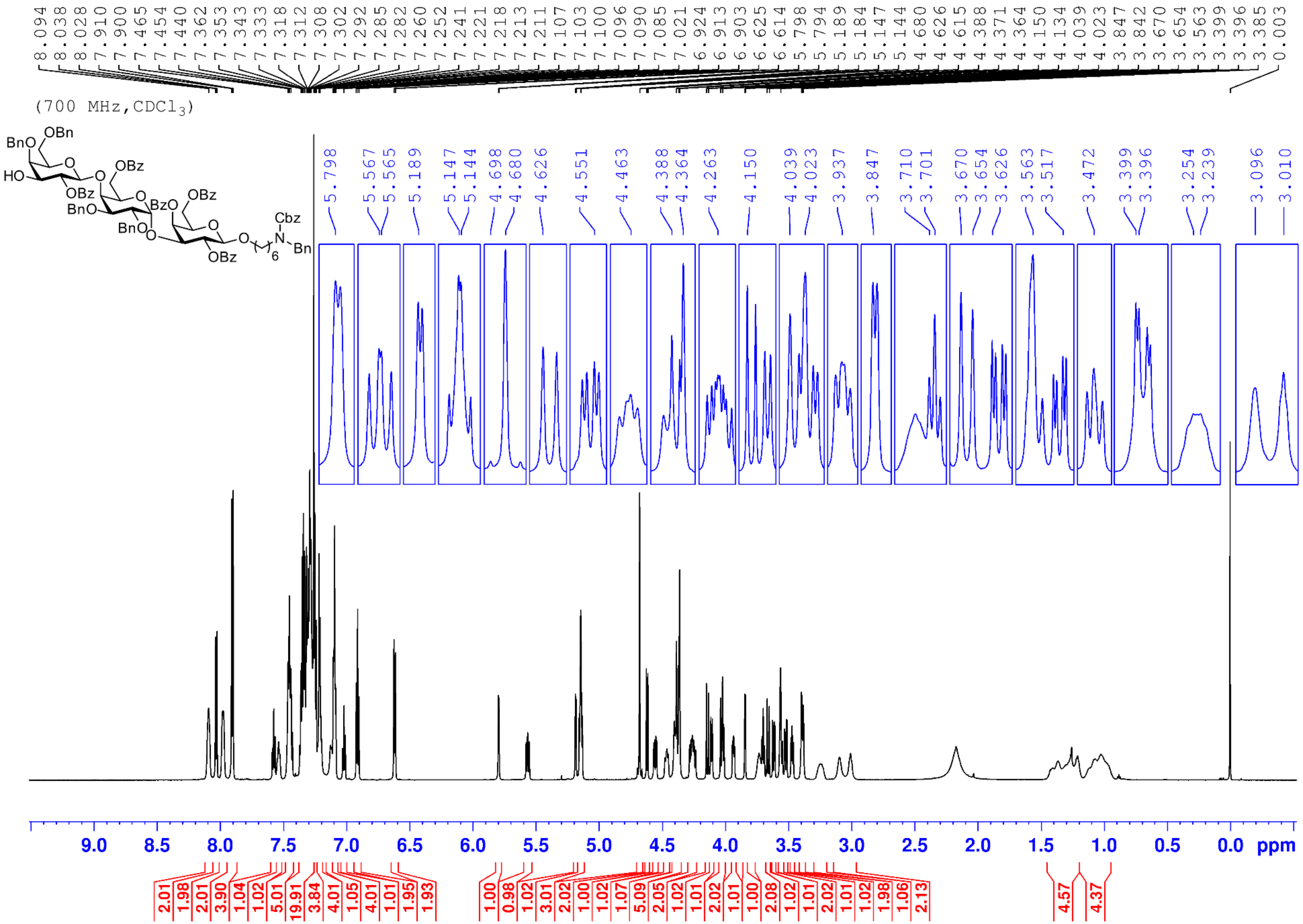

¹H-¹H COSY

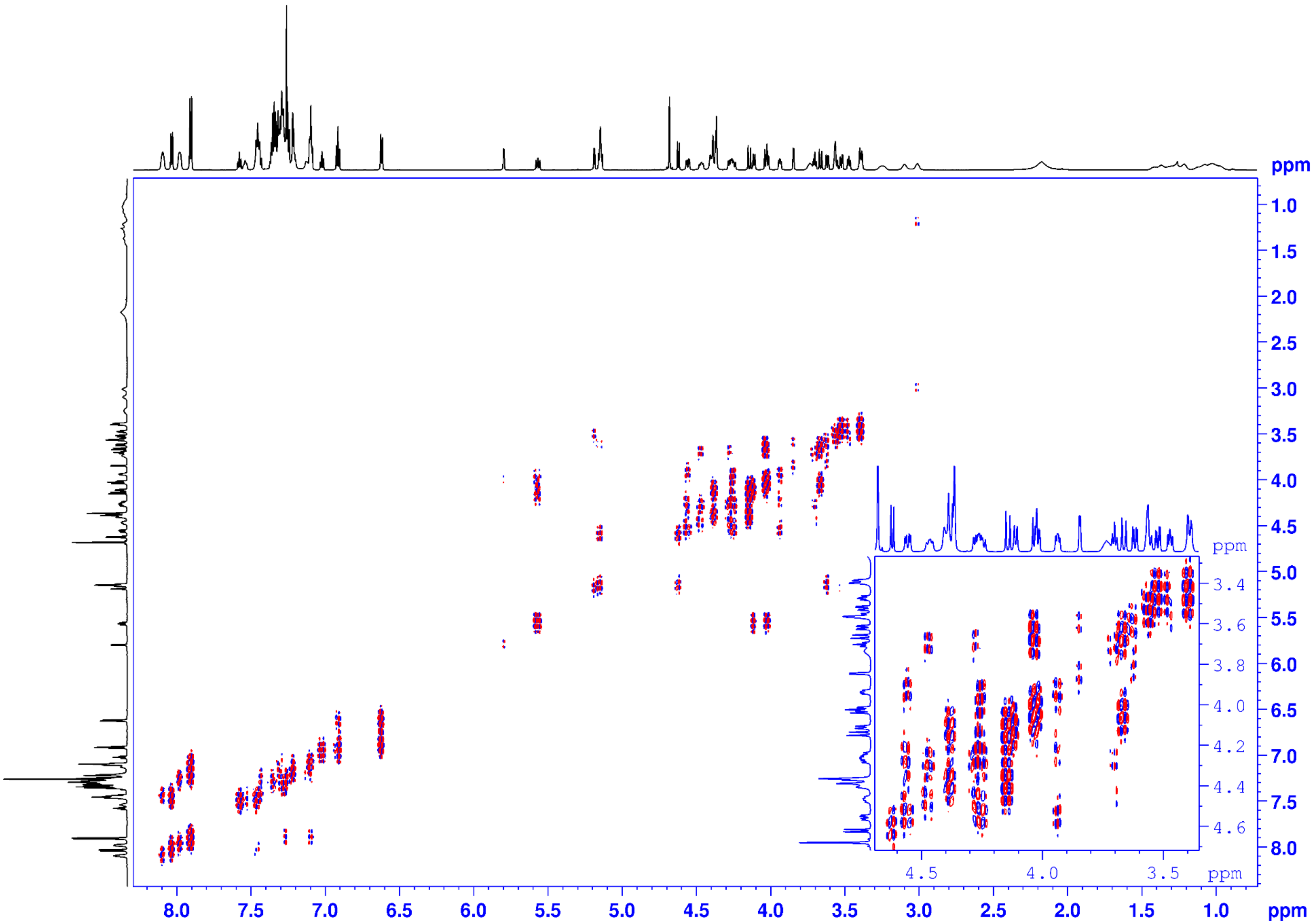
ppm
1.0
1.5
2.0
2.5
3.0
3.5
4.0
4.5
5.0
5.5
6.0
6.5
7.0
7.5
8.0
8.0 7.5 7.0 6.5 6.0 5.5 5.0 4.5 4.0 3.5 3.0 2.5 2.0 1.5 1.0
ppm
3.4
3.6
3.8
4.0
4.2
4.4
4.6
4.5 4.0 3.5

^{1}H-^{13}C HSQC

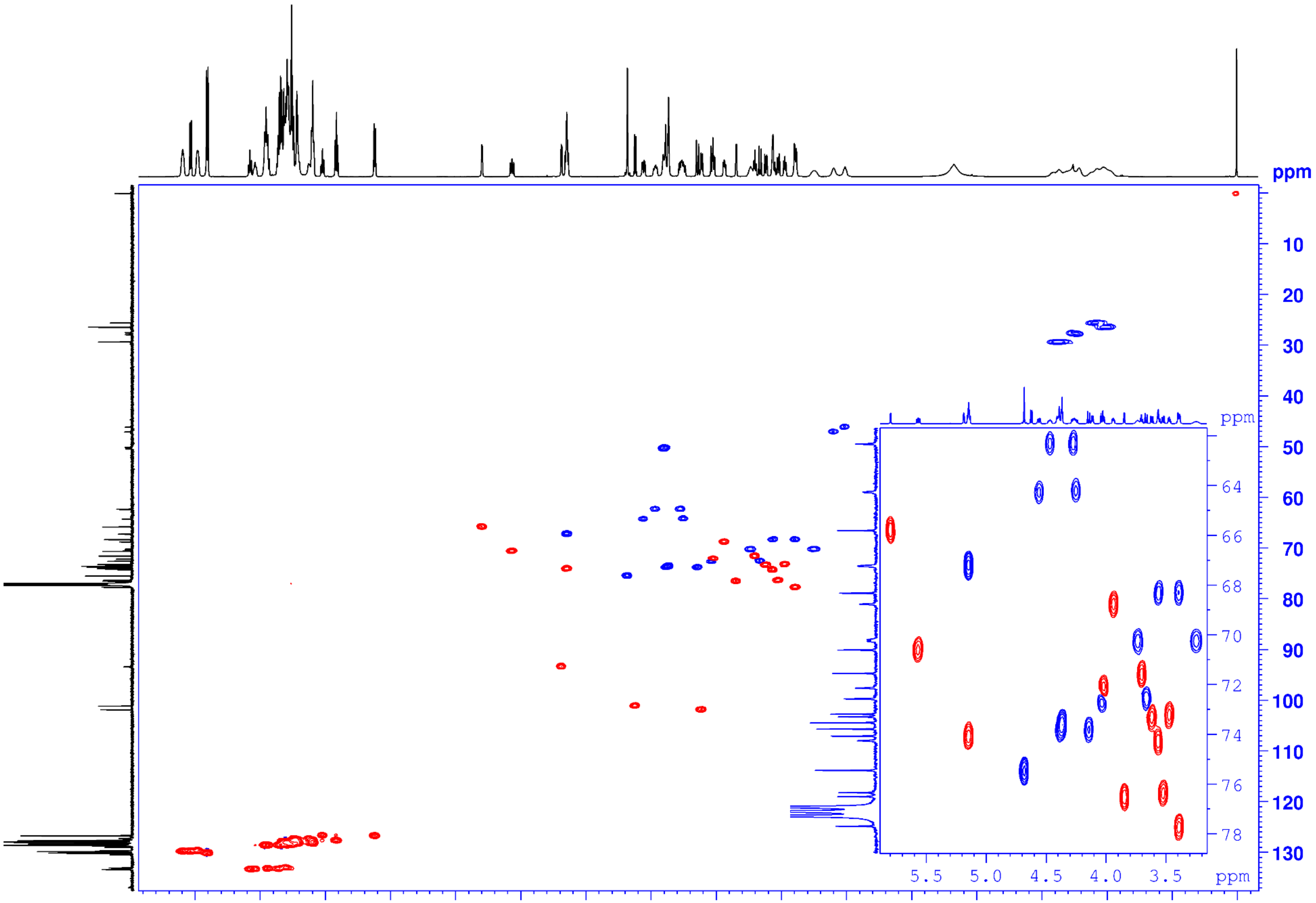

ppm
8.0 7.5 7.0 6.5 6.0 5.5 5.0 4.5 4.0 3.5 3.0 2.5 2.0 1.5 1.0 0.5 0.0 ppm
10 20 30 40 50 60 70 80 90 100 110 120 130
5.5 5.0 4.5 4.0 3.5 ppm
64 66 68 70 72 74 76 78

^1H-^{13}C HMBC

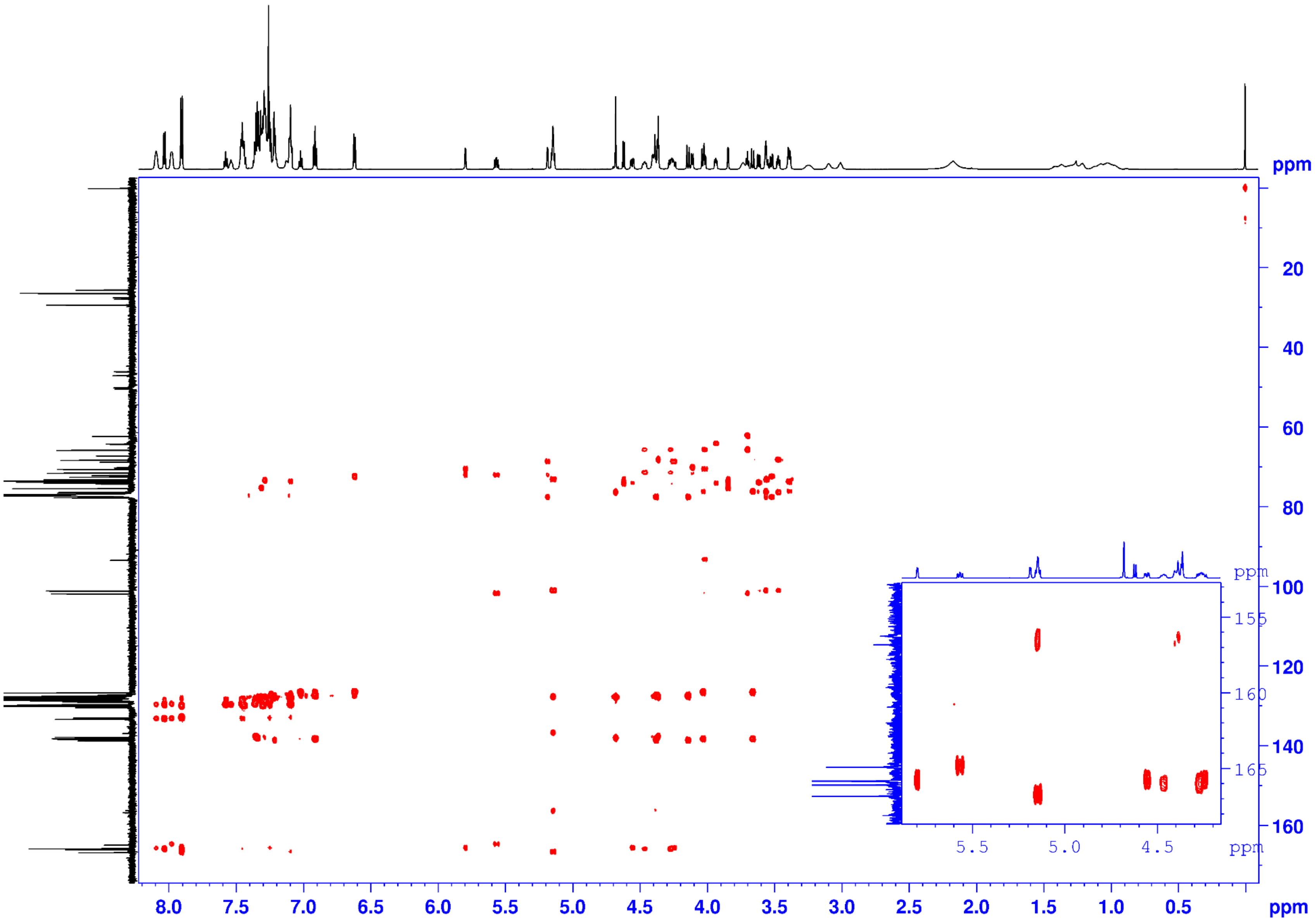

ppm
20
40
60
80
100
120
140
160
8.0
7.5
7.0
6.5
6.0
5.5
5.0
4.5
4.0
3.5
3.0
2.5
2.0
1.5
1.0
0.5
ppm
155
160
165
5.5
5.0
4.5
ppm

$^{13}C\{^{1}H\}$ NMR

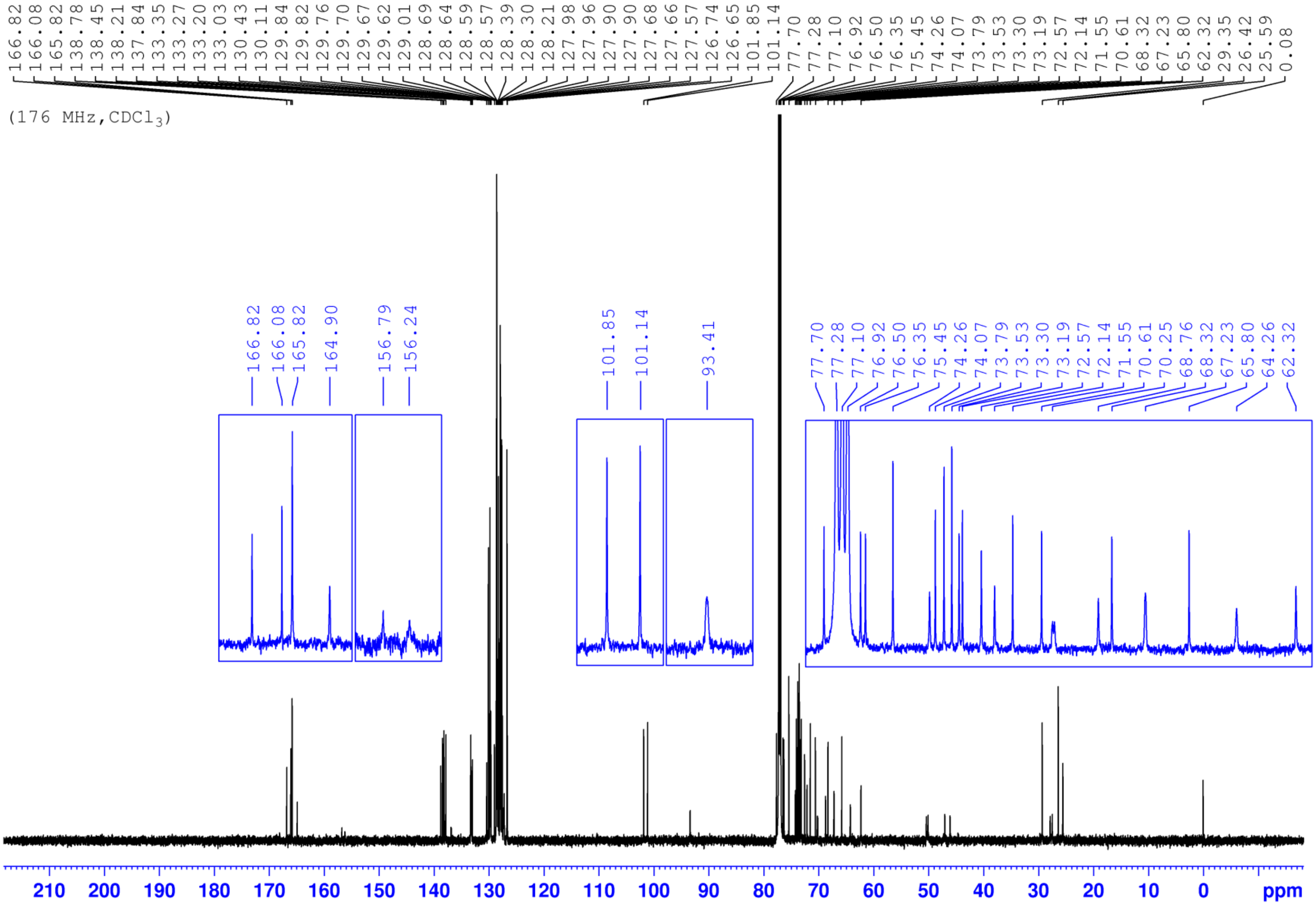

Compound **29**

^{1}H-NMR

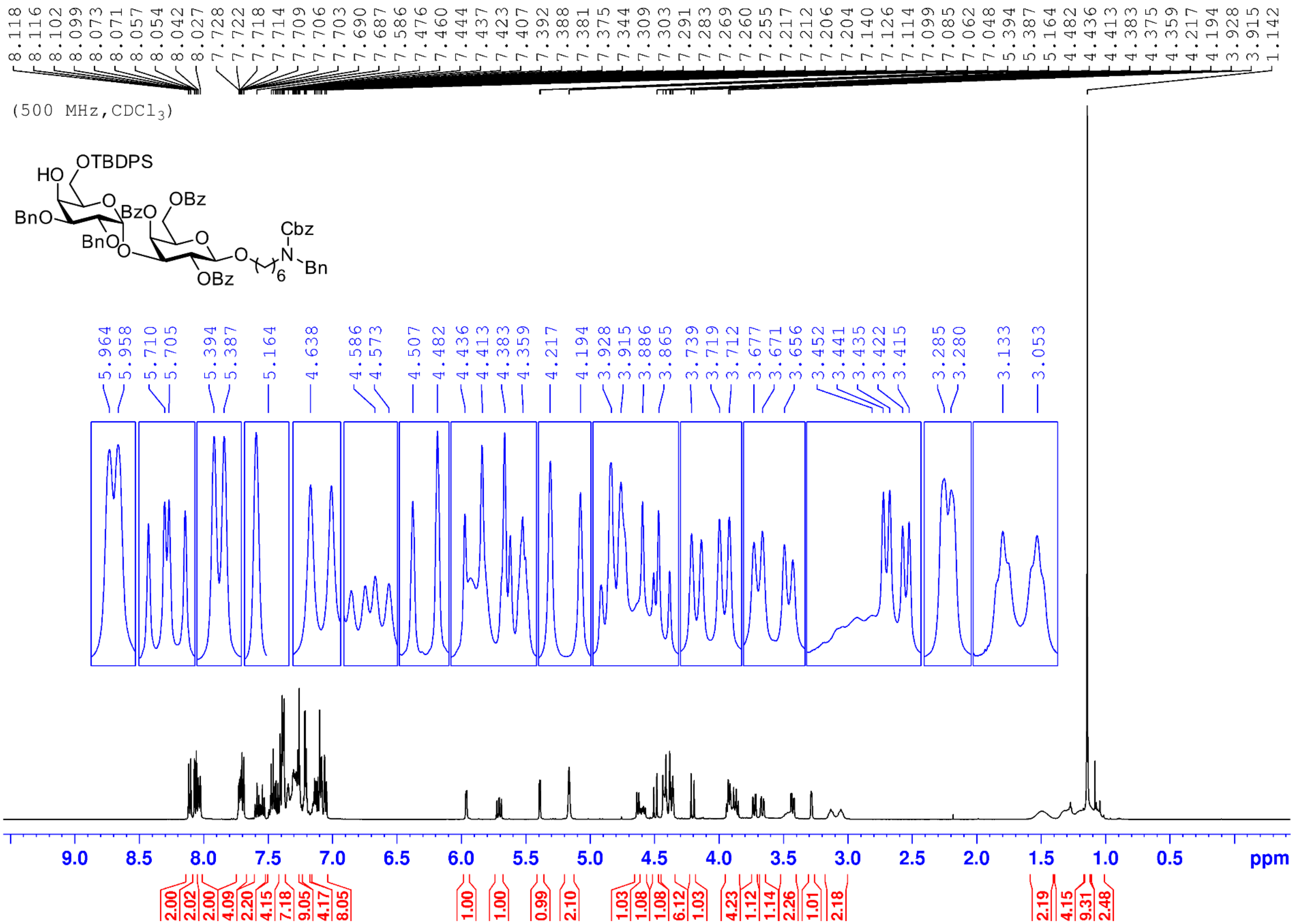

^{1}H-^{1}H COSY

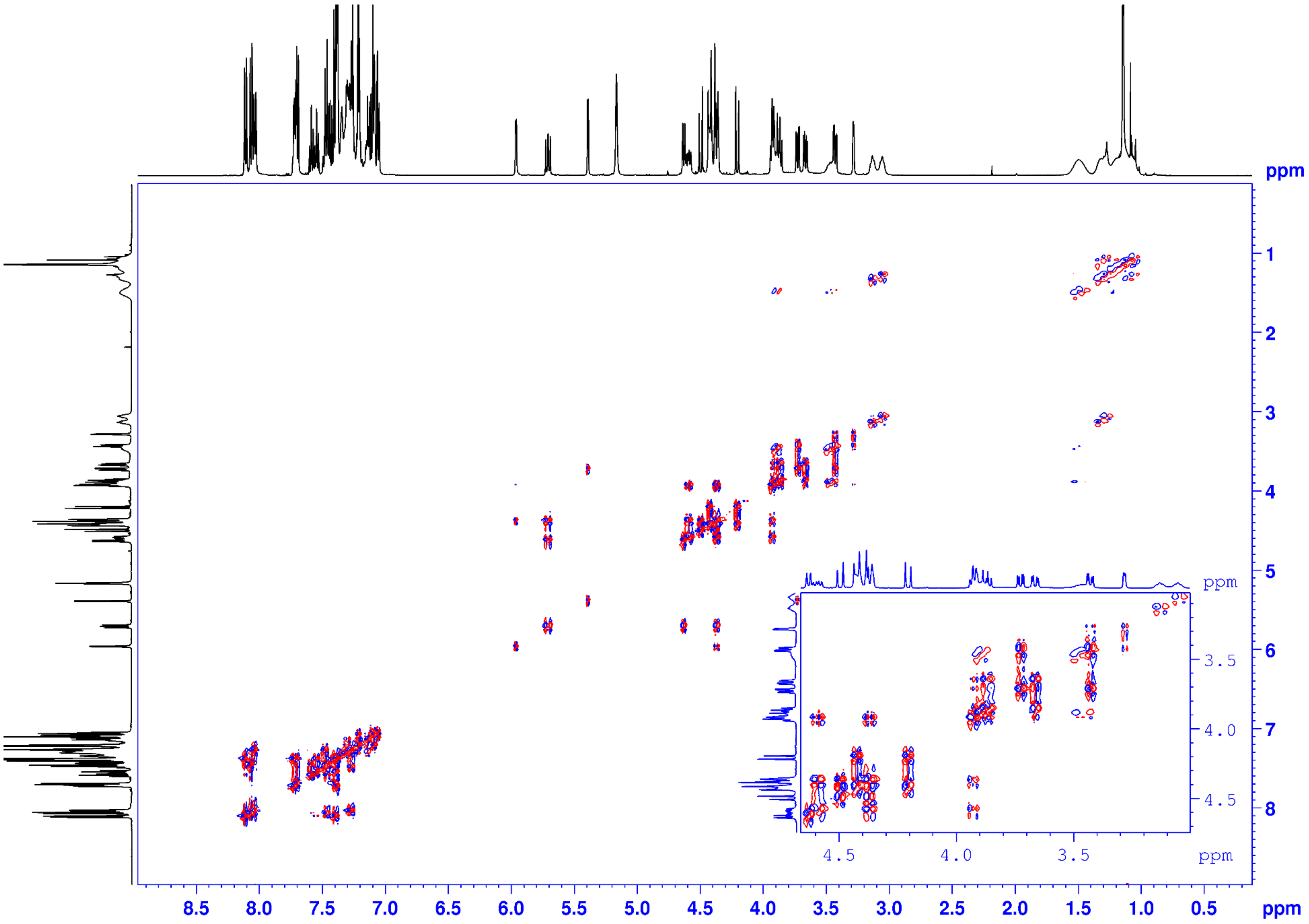

ppm
8.5
8.0
7.5
7.0
6.5
6.0
5.5
5.0
4.5
4.0
3.5
3.0
2.5
2.0
1.5
1.0
0.5
ppm
1
2
3
4
5
6
7
8
4.5
4.0
3.5
ppm
3.5
4.0
4.5
ppm

^{1}H-^{13}C HSQC

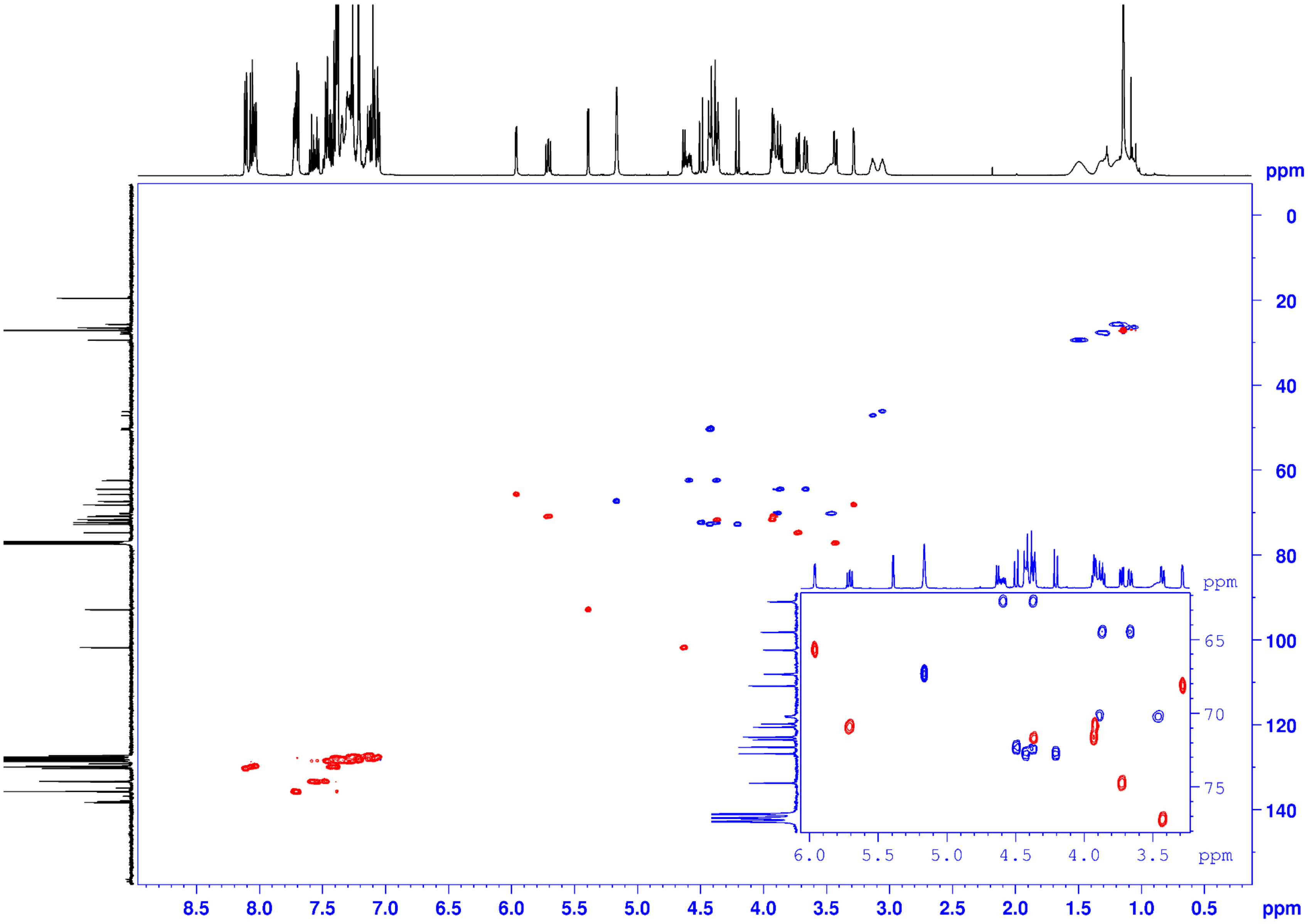
ppm
0
20
40
60
80
100
120
140
ppm
65
70
75
6.0
5.5
5.0
4.5
4.0
3.5
ppm
8.5
8.0
7.5
7.0
6.5
6.0
5.5
5.0
4.5
4.0
3.5
3.0
2.5
2.0
1.5
1.0
0.5
ppm

^{1}H-^{13}C non-decoupled HSQC

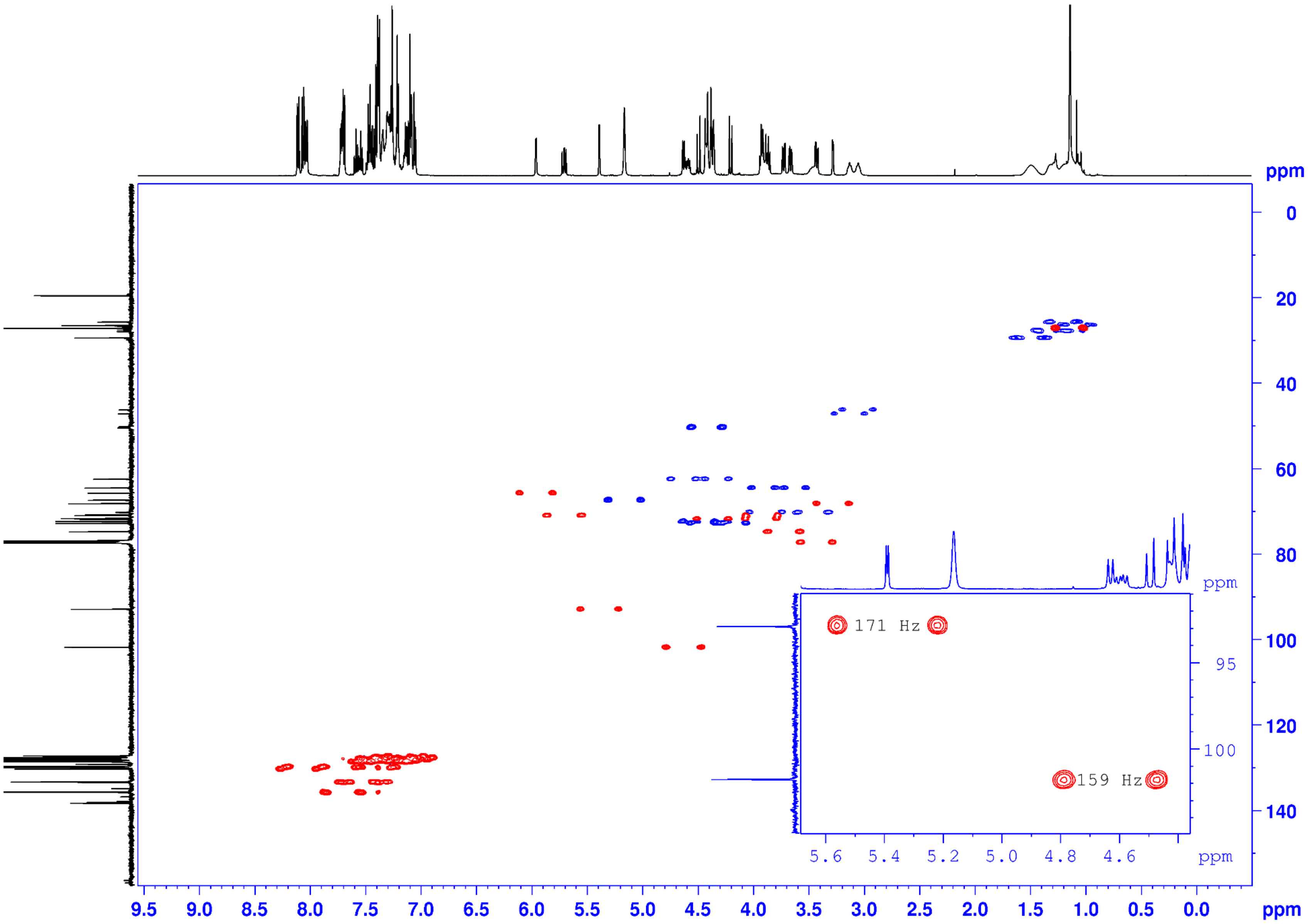

^{1}H-^{13}C HMBC

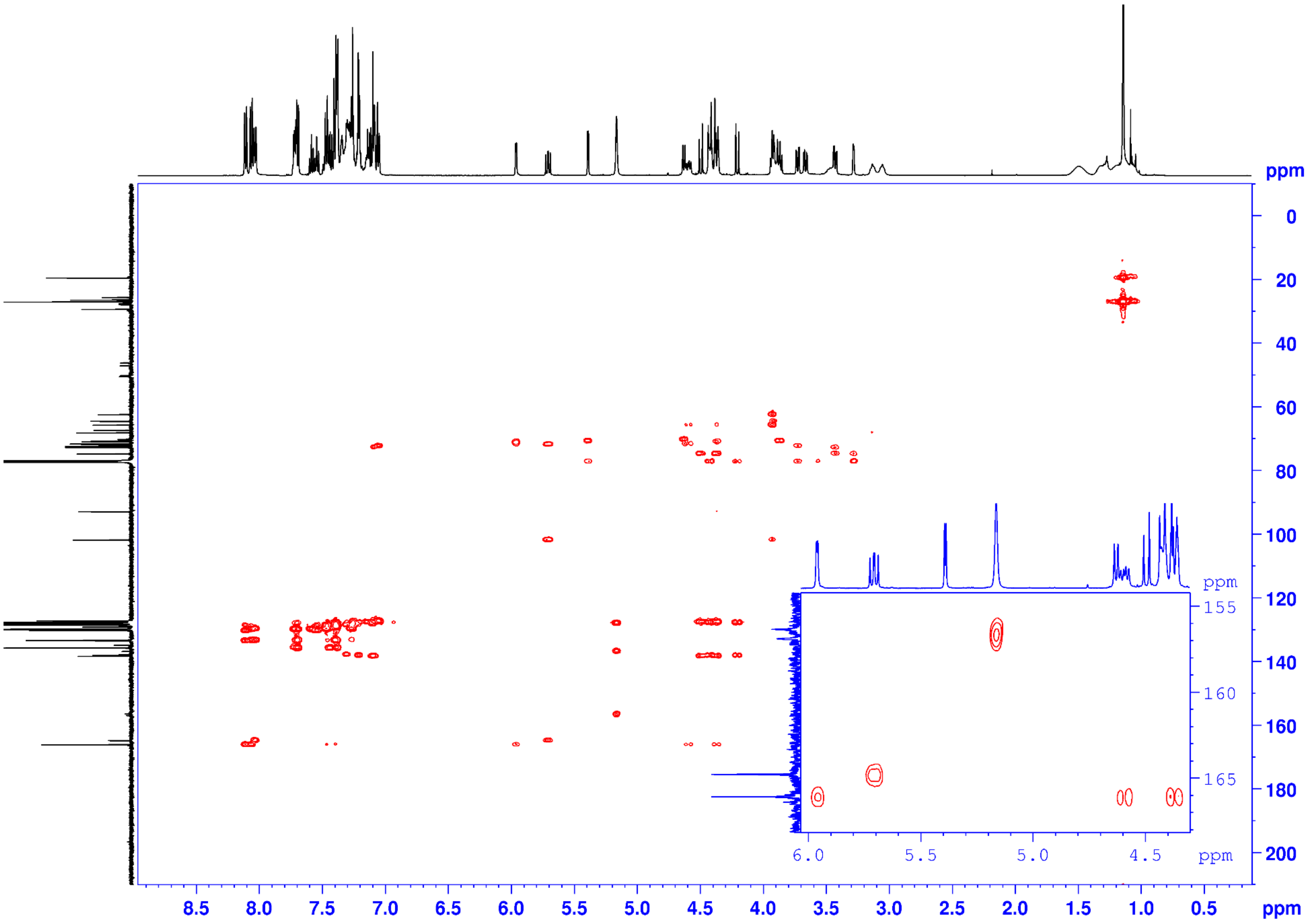

ppm
0
20
40
60
80
100
120
140
160
180
200
8.5
8.0
7.5
7.0
6.5
6.0
5.5
5.0
4.5
4.0
3.5
3.0
2.5
2.0
1.5
1.0
0.5
ppm
155
160
165
6.0
5.5
5.0
4.5
ppm

$^{13}C\{^{1}H\}$ NMR

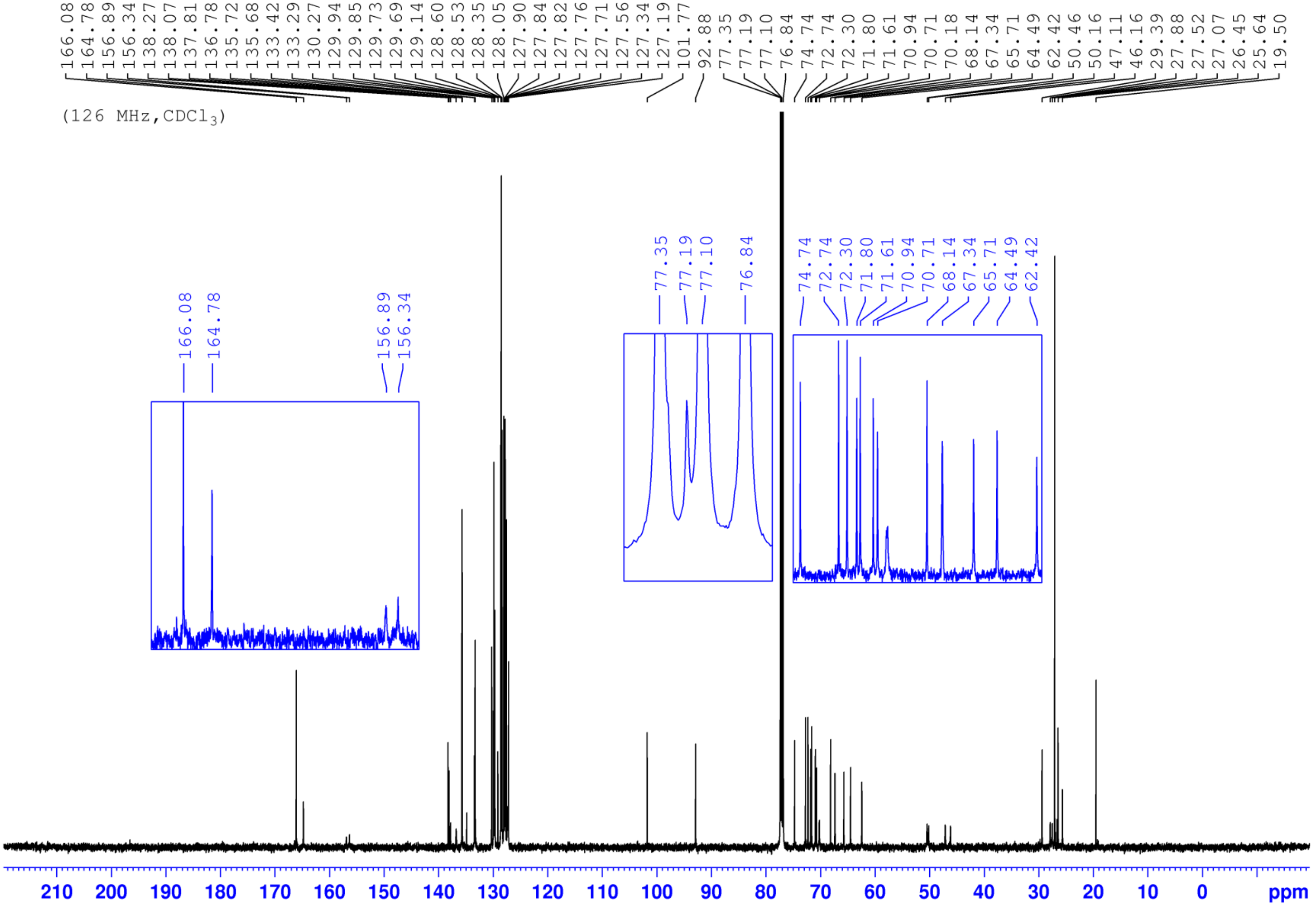

166.08
164.78
156.89
156.34
138.27
138.07
137.81
136.78
135.72
135.68
133.42
133.29
130.27
129.94
129.85
129.73
129.69
129.14
128.60
128.53
128.35
128.05
127.90
127.84
127.82
127.76
127.71
127.56
127.34
127.19
101.77
92.88
77.35
77.19
77.10
76.84
74.74
72.74
72.30
71.80
71.61
70.94
70.71
70.18
68.14
67.34
65.71
64.49
62.42
50.46
50.16
47.11
46.16
29.39
27.88
27.52
27.07
26.45
25.64
19.50
(126 MHz, $CDCl_3$)
210 200 190 180 170 160 150 140 130 120 110 100 90 80 70 60 50 40 30 20 10 0 ppm

Compound **30**

^{1}H-NMR

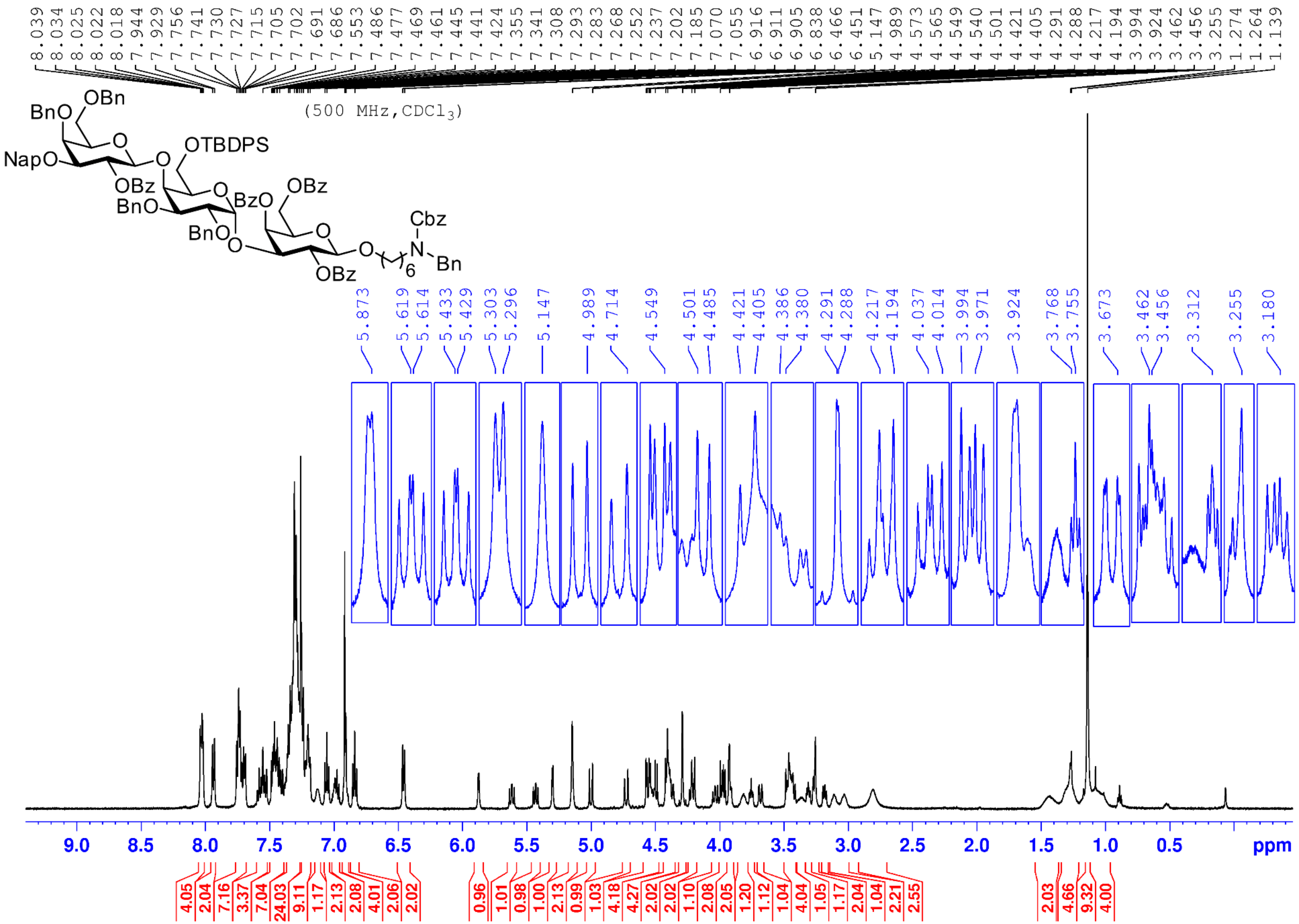

^{1}H-^{1}H COSY

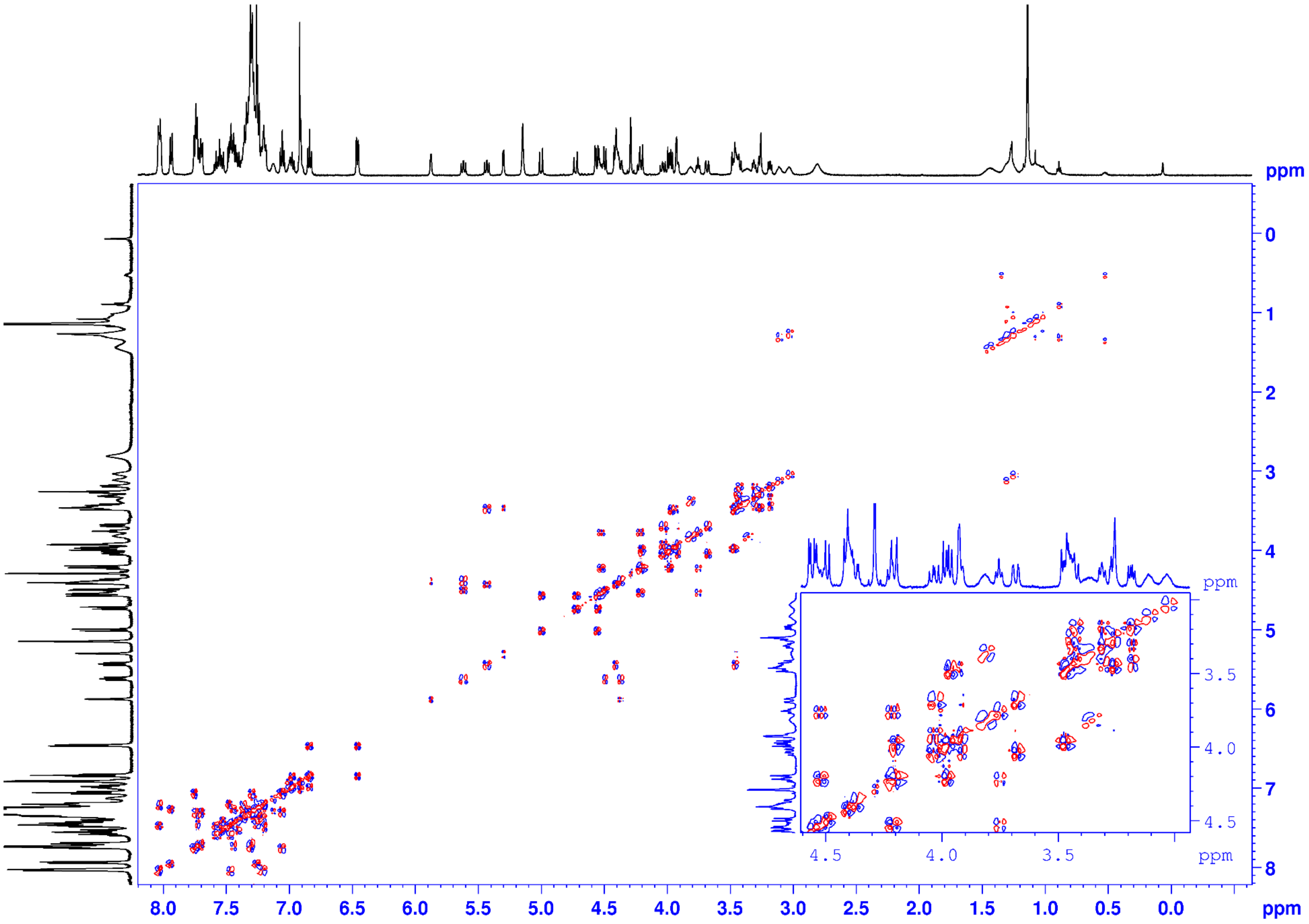
ppm
0
1
2
3
4
5
6
7
8
8.0
7.5
7.0
6.5
6.0
5.5
5.0
4.5
4.0
3.5
3.0
2.5
2.0
1.5
1.0
0.5
0.0
ppm
3.5
4.0
4.5
4.5
4.0
3.5
ppm

^{1}H-^{13}C HSQC

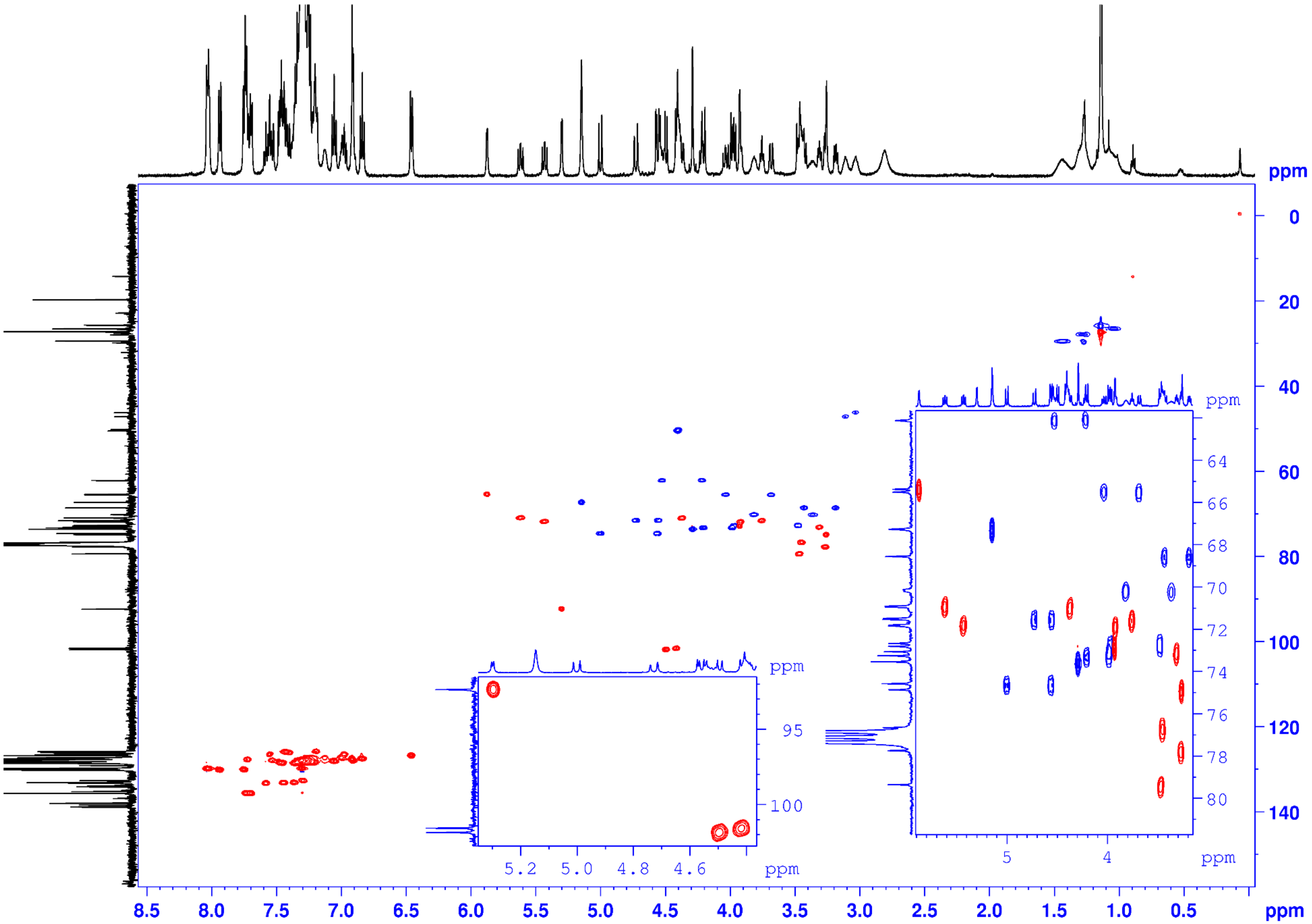

ppm
0
20
40
60
80
100
120
140
ppm
8.5
8.0
7.5
7.0
6.5
6.0
5.5
5.0
4.5
4.0
3.5
3.0
2.5
2.0
1.5
1.0
0.5
ppm
95
100
5.2
5.0
4.8
4.6
ppm
ppm
64
66
68
70
72
74
76
78
80
5
4
ppm

^{1}H-^{13}C non-decoupled HSQC

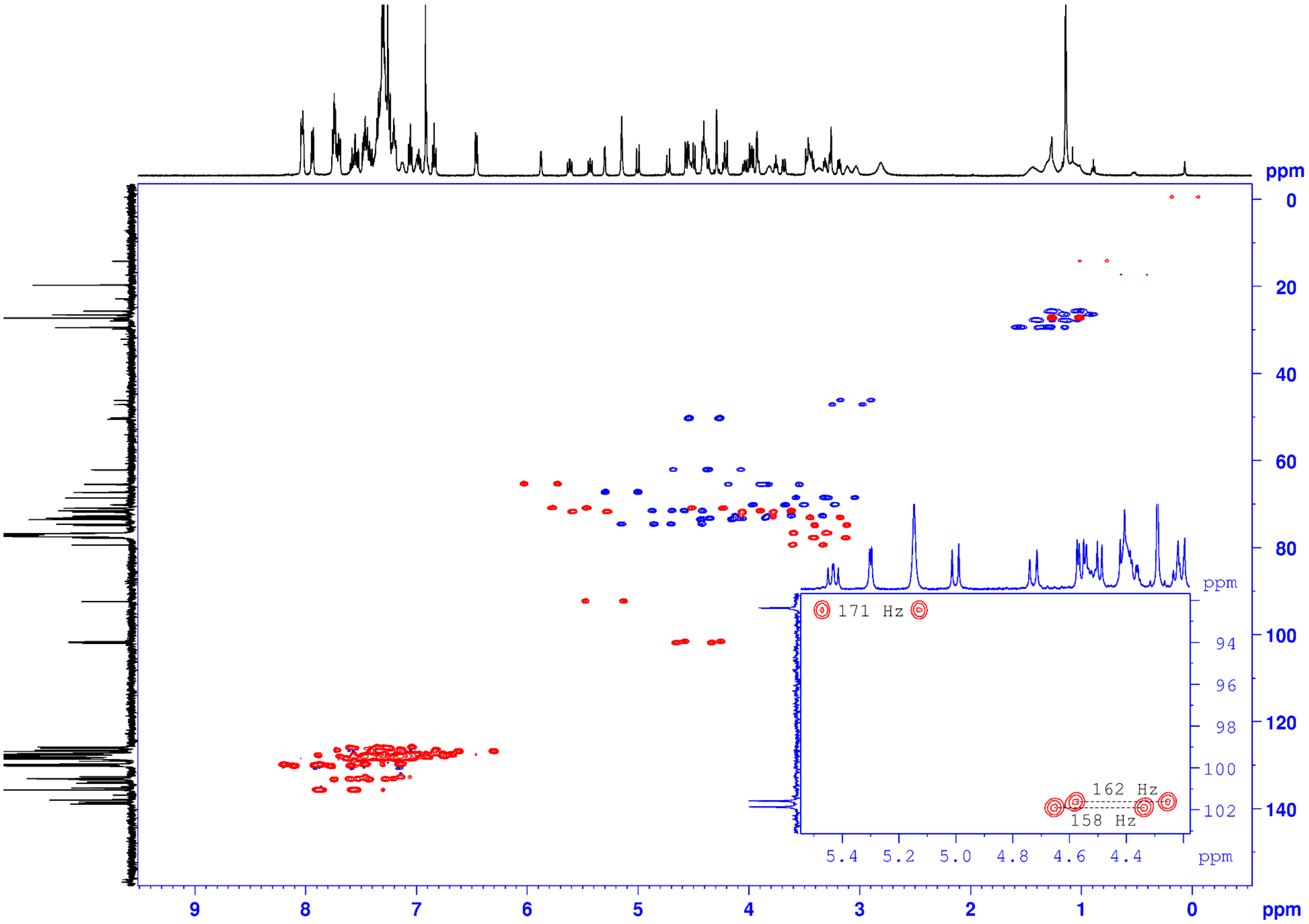

^{1}H-^{13}C HMBC

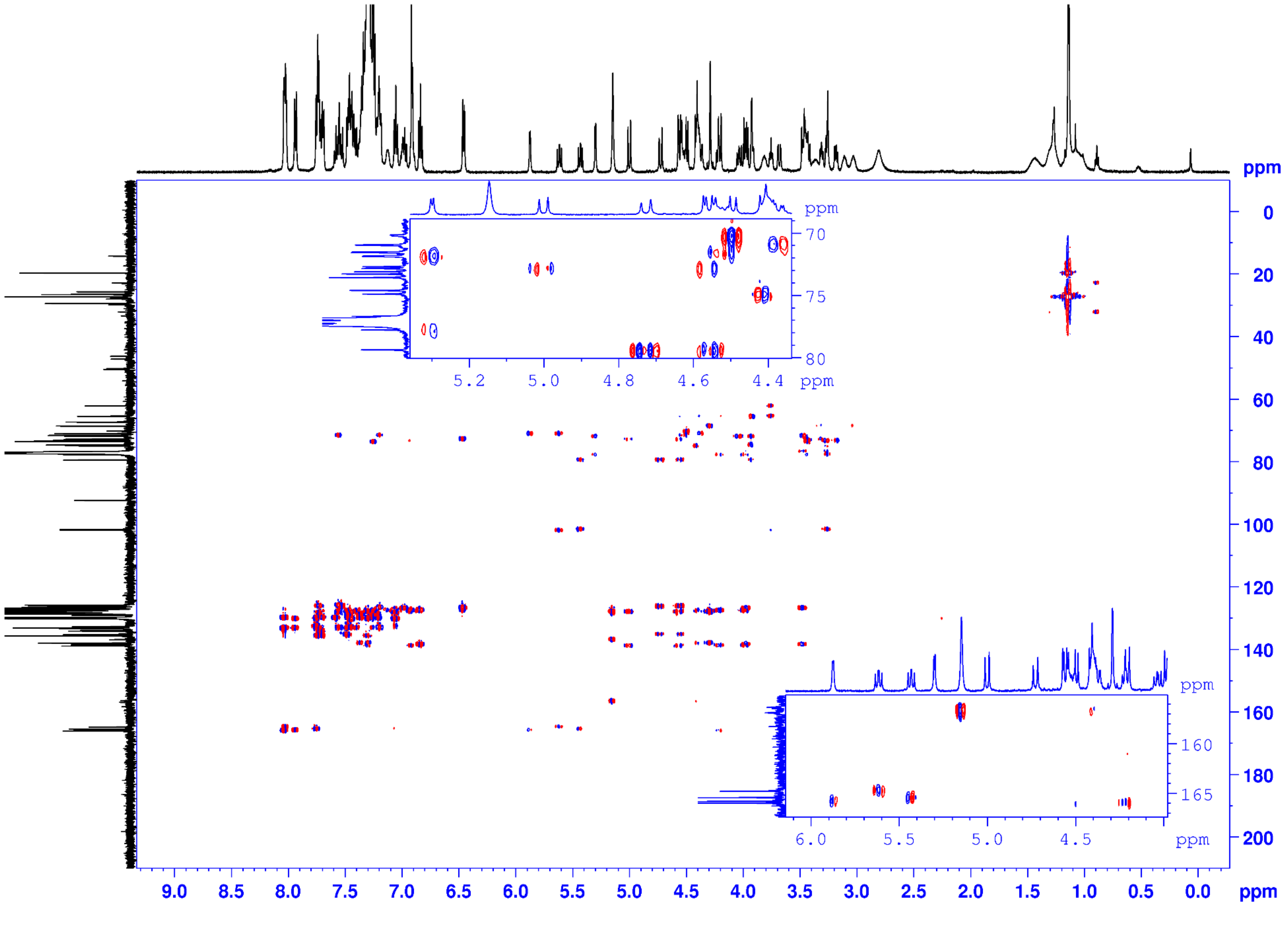

ppm
0
20
40
60
80
100
120
140
160
180
200
9.0 8.5 8.0 7.5 7.0 6.5 6.0 5.5 5.0 4.5 4.0 3.5 3.0 2.5 2.0 1.5 1.0 0.5 0.0
ppm
70
75
80
5.2 5.0 4.8 4.6 4.4
ppm
160
165
6.0 5.5 5.0 4.5

$^{13}C\{^{1}H\}$ NMR

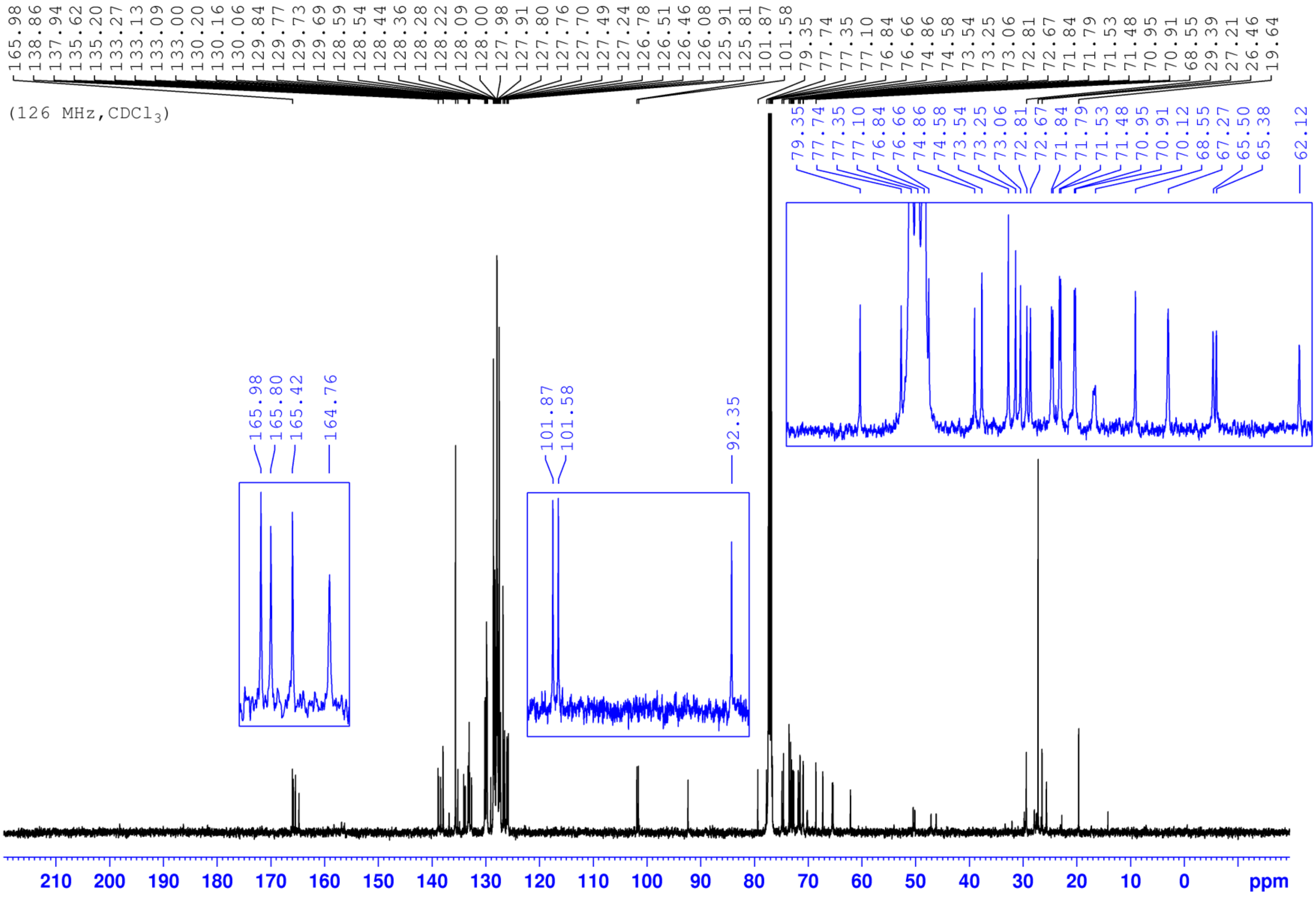

165.98 138.86 137.94 135.62 135.20 133.27 133.13 133.09 133.00 130.20 130.16 130.06 129.84 129.77 129.73 129.69 128.59 128.54 128.44 128.36 128.28 128.22 128.09 128.00 127.98 127.91 127.80 127.76 127.70 127.49 127.24 126.78 126.51 126.46 126.08 125.91 125.81 101.87 101.58 79.35 77.74 77.35 77.10 76.84 76.66 74.86 74.58 73.54 73.25 73.06 72.81 72.67 71.84 71.79 71.53 71.48 70.95 70.91 68.55 29.39 27.21 26.46 19.64
(126 MHz, CDCl3)
165.98 165.80 165.42 164.76
101.87 101.58 92.35
79.35 77.74 77.35 77.10 76.84 76.66 74.86 74.58 73.54 73.25 73.06 72.81 72.67 71.84 71.79 71.53 71.48 70.95 70.91 70.12 68.55 67.27 65.50 65.38 62.12
210 200 190 180 170 160 150 140 130 120 110 100 90 80 70 60 50 40 30 20 10 0 ppm

Compound **31**

^{1}H-NMR

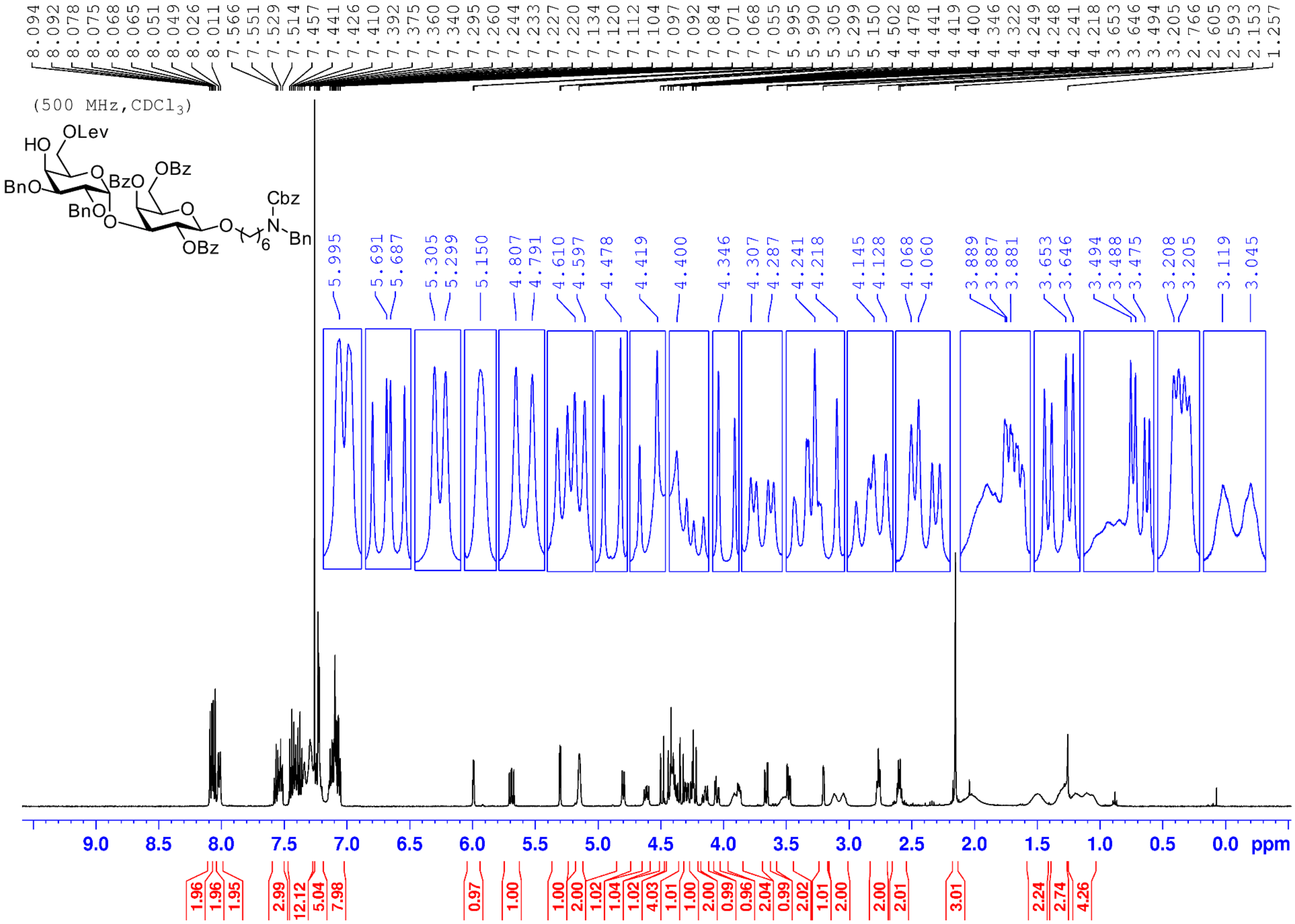

^{1}H-^{1}H COSY

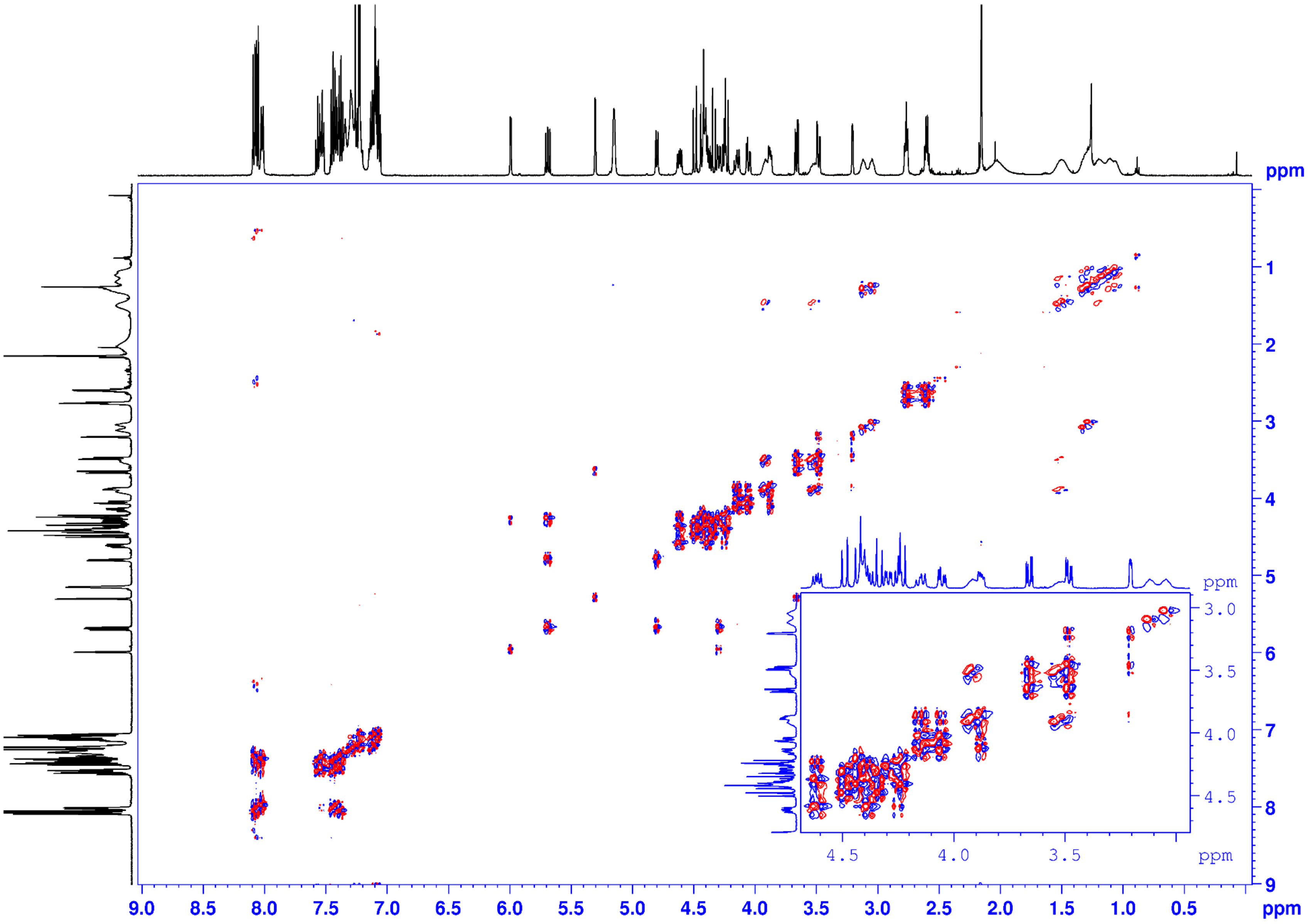

ppm
9.0 8.5 8.0 7.5 7.0 6.5 6.0 5.5 5.0 4.5 4.0 3.5 3.0 2.5 2.0 1.5 1.0 0.5
1 2 3 4 5 6 7 8 9
4.5 4.0 3.5
3.0 3.5 4.0 4.5

^{1}H-^{13}C HSQC

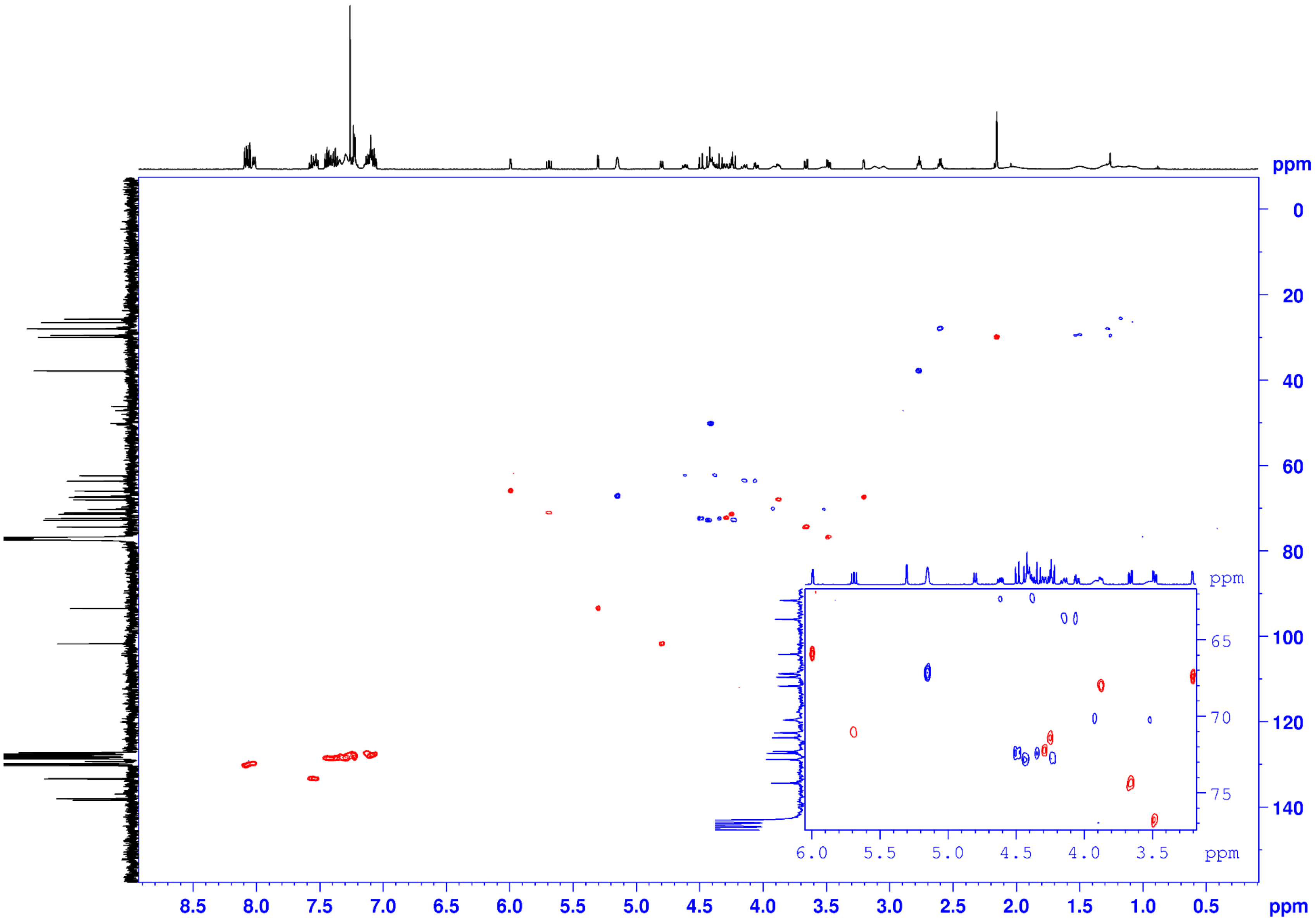

ppm
0
20
40
60
80
100
120
140
8.5
8.0
7.5
7.0
6.5
6.0
5.5
5.0
4.5
4.0
3.5
3.0
2.5
2.0
1.5
1.0
0.5
ppm
65
70
75
6.0
5.5
5.0
4.5
4.0
3.5
ppm

^{1}H-^{13}C HMBC

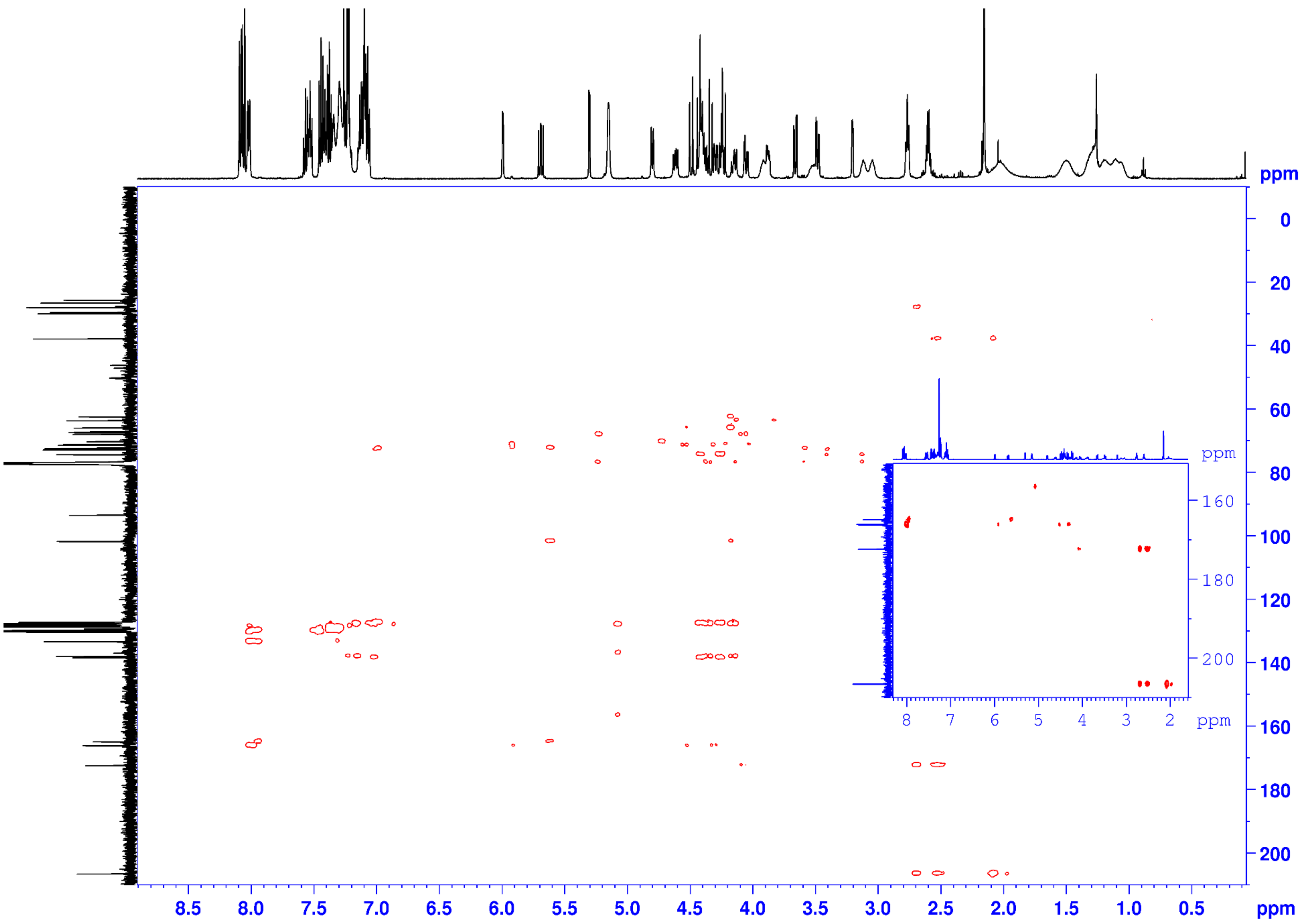
ppm
0
20
40
60
80
100
120
140
160
180
200
8.5
8.0
7.5
7.0
6.5
6.0
5.5
5.0
4.5
4.0
3.5
3.0
2.5
2.0
1.5
1.0
0.5
ppm
160
180
200
8
7
6
5
4
3
2

$^{13}C\{^1H\}$ NMR

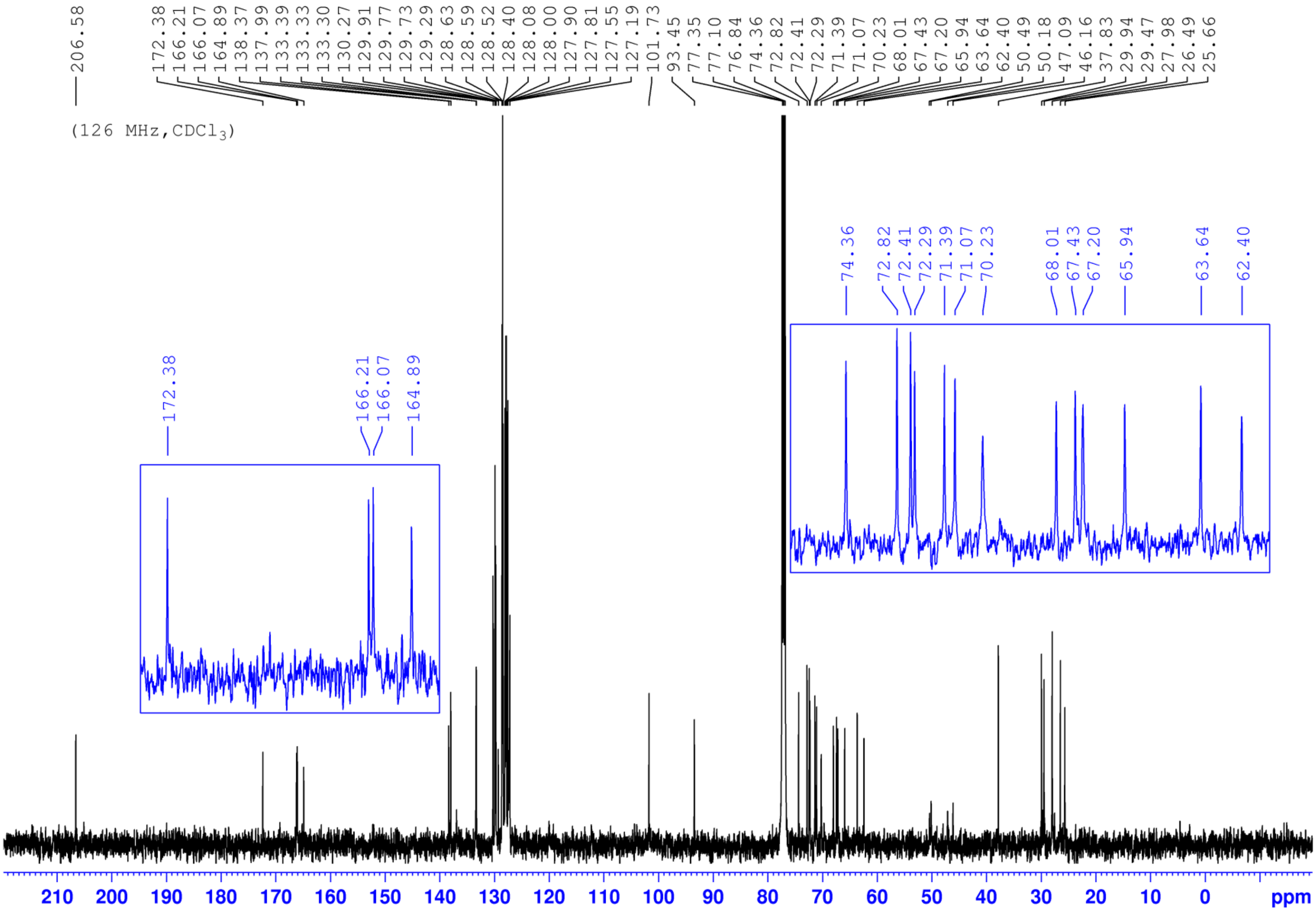
206.58
172.38
166.21
166.07
164.89
138.37
137.99
133.39
133.33
133.30
130.27
129.91
129.77
129.73
129.29
128.63
128.59
128.52
128.40
128.08
128.00
127.90
127.81
127.55
127.19
101.73
93.45
77.35
77.10
76.84
74.36
72.82
72.41
72.29
71.39
71.07
70.23
68.01
67.43
67.20
65.94
63.64
62.40
50.49
50.18
47.09
46.16
37.83
29.94
29.47
27.98
26.49
25.66
(126 MHz, CDCl3)
ppm

Compound **32**

^{1}H-NMR

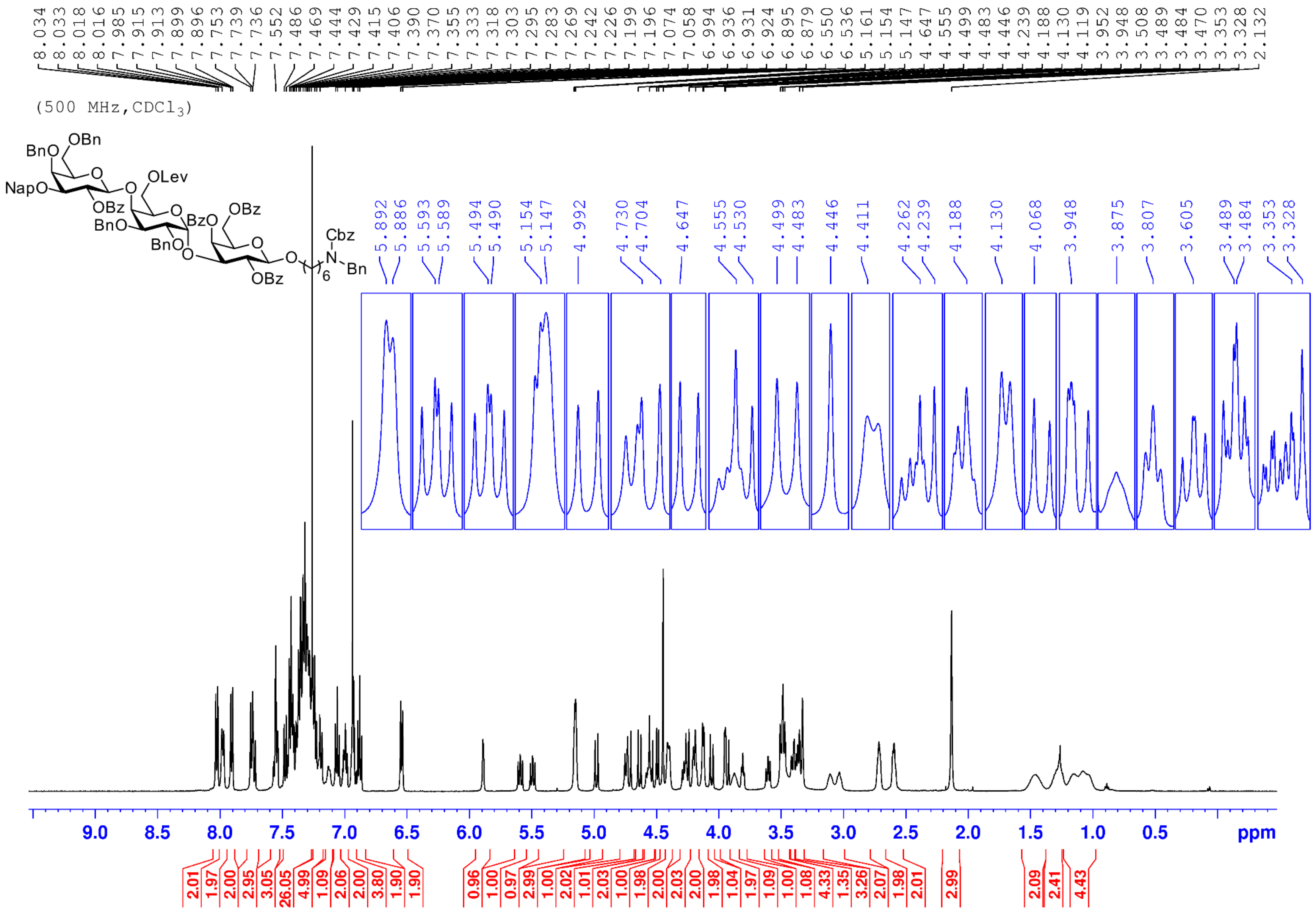

^{1}H-^{1}H COSY

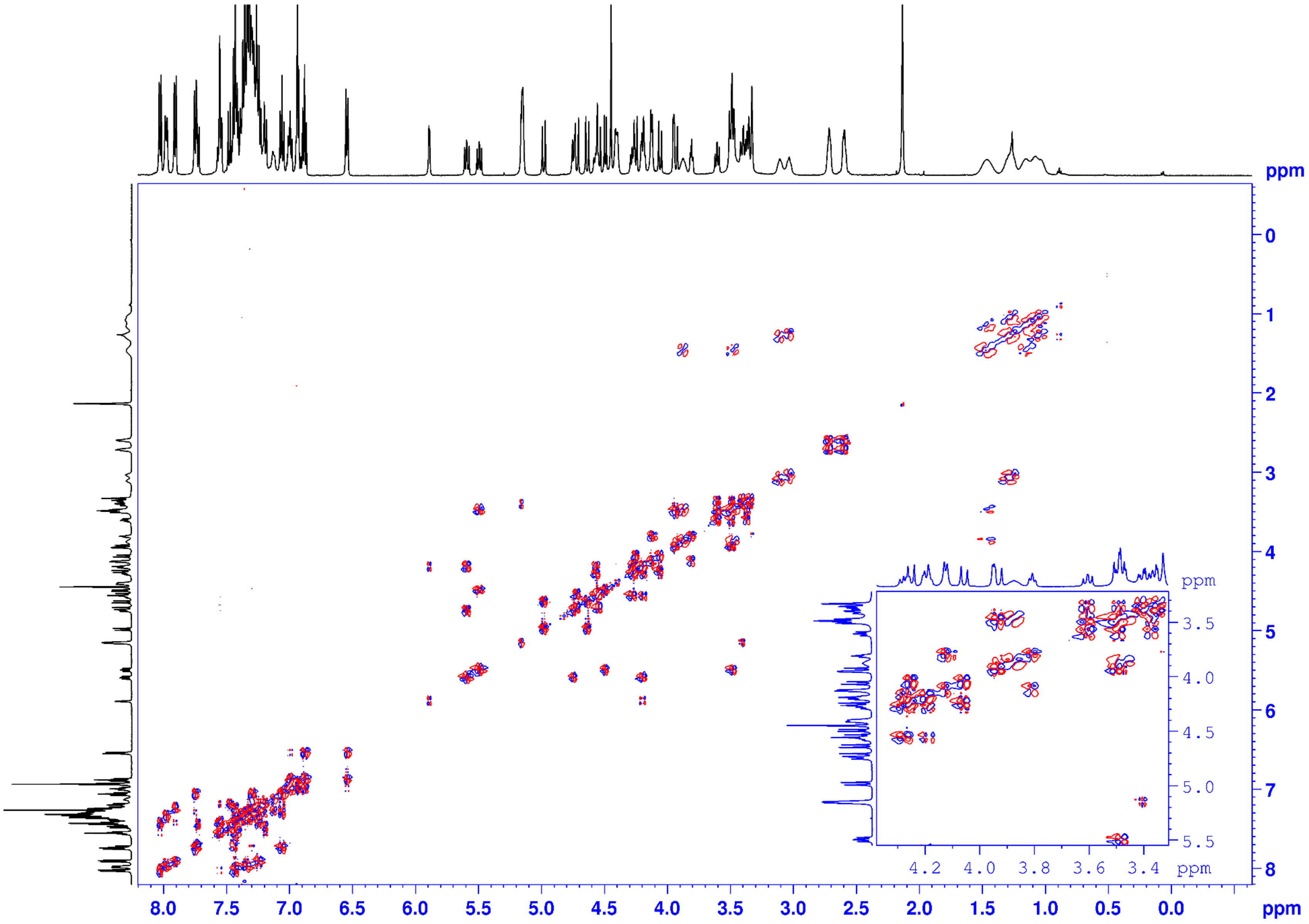

ppm
0
1
2
3
4
5
6
7
8
8.0 7.5 7.0 6.5 6.0 5.5 5.0 4.5 4.0 3.5 3.0 2.5 2.0 1.5 1.0 0.5 0.0
ppm
3.5
4.0
4.5
5.0
5.5
4.2 4.0 3.8 3.6 3.4 ppm

^{1}H-^{13}C HSQC

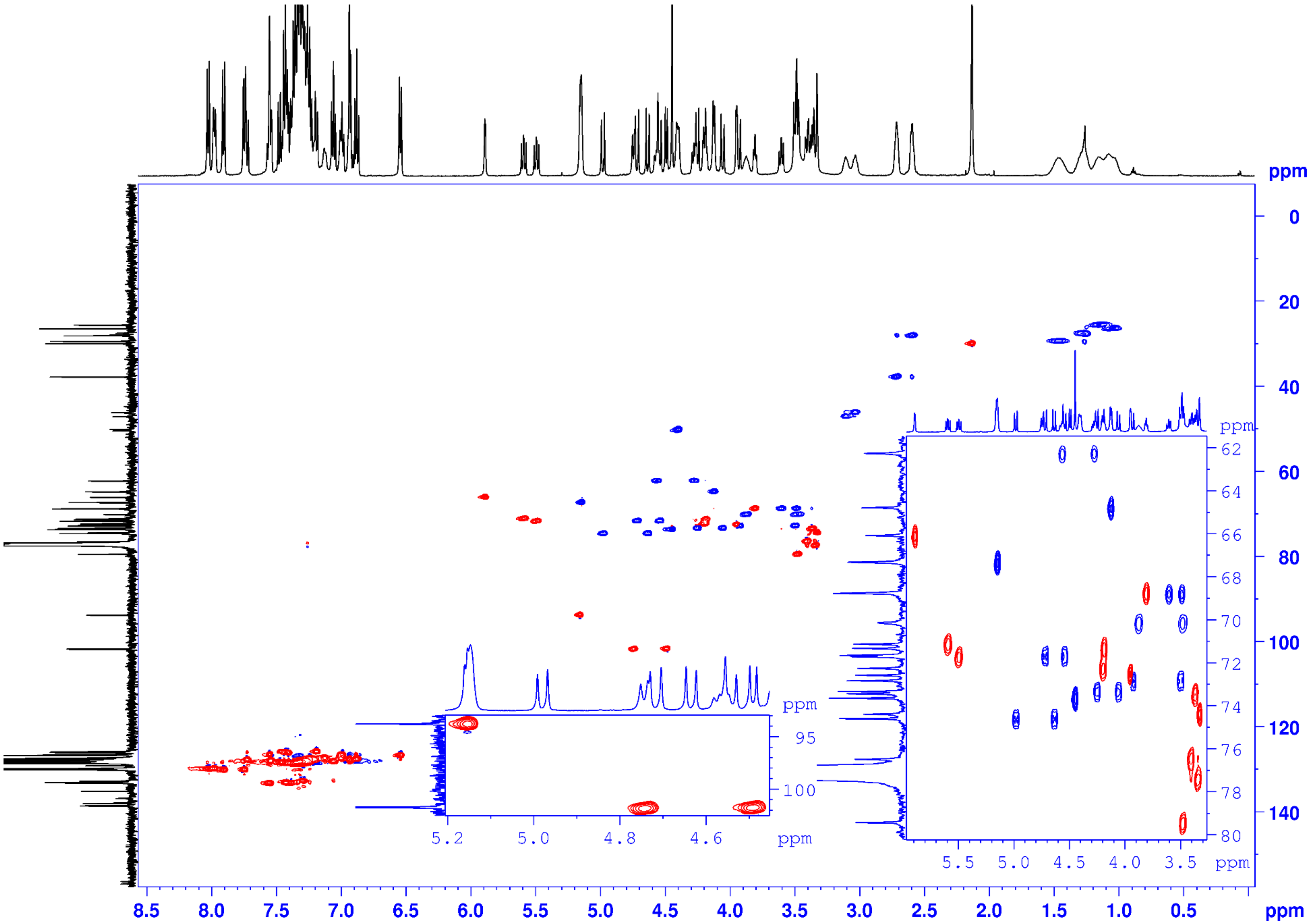
ppm
0
20
40
60
80
100
120
140
8.5
8.0
7.5
7.0
6.5
6.0
5.5
5.0
4.5
4.0
3.5
3.0
2.5
2.0
1.5
1.0
0.5
ppm
95
100
5.2
5.0
4.8
4.6
ppm
62
64
66
68
70
72
74
76
78
80
5.5
5.0
4.5
4.0
3.5
ppm

^{1}H-^{13}C non-decoupled HSQC

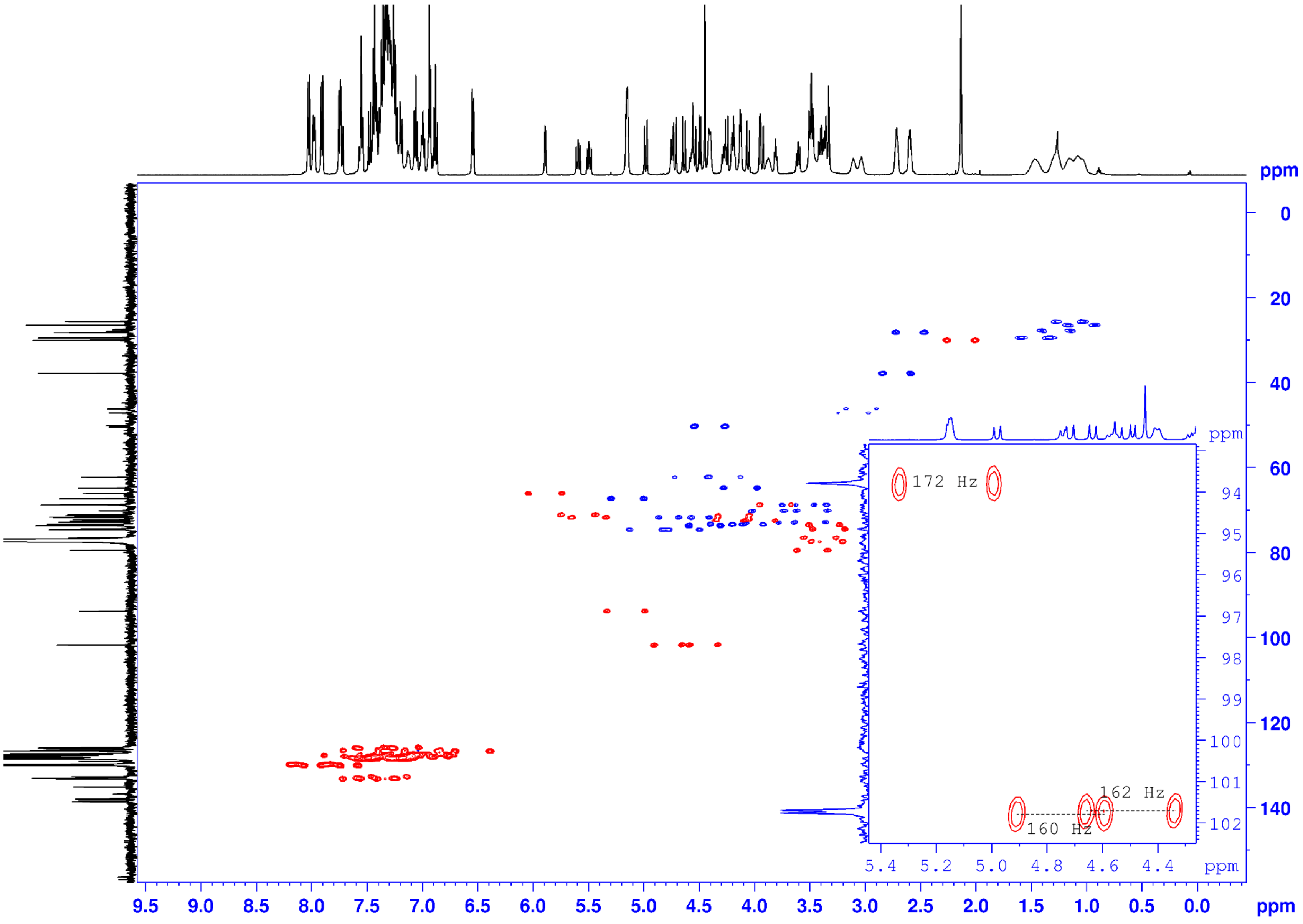

^{1}H-^{13}C HMBC

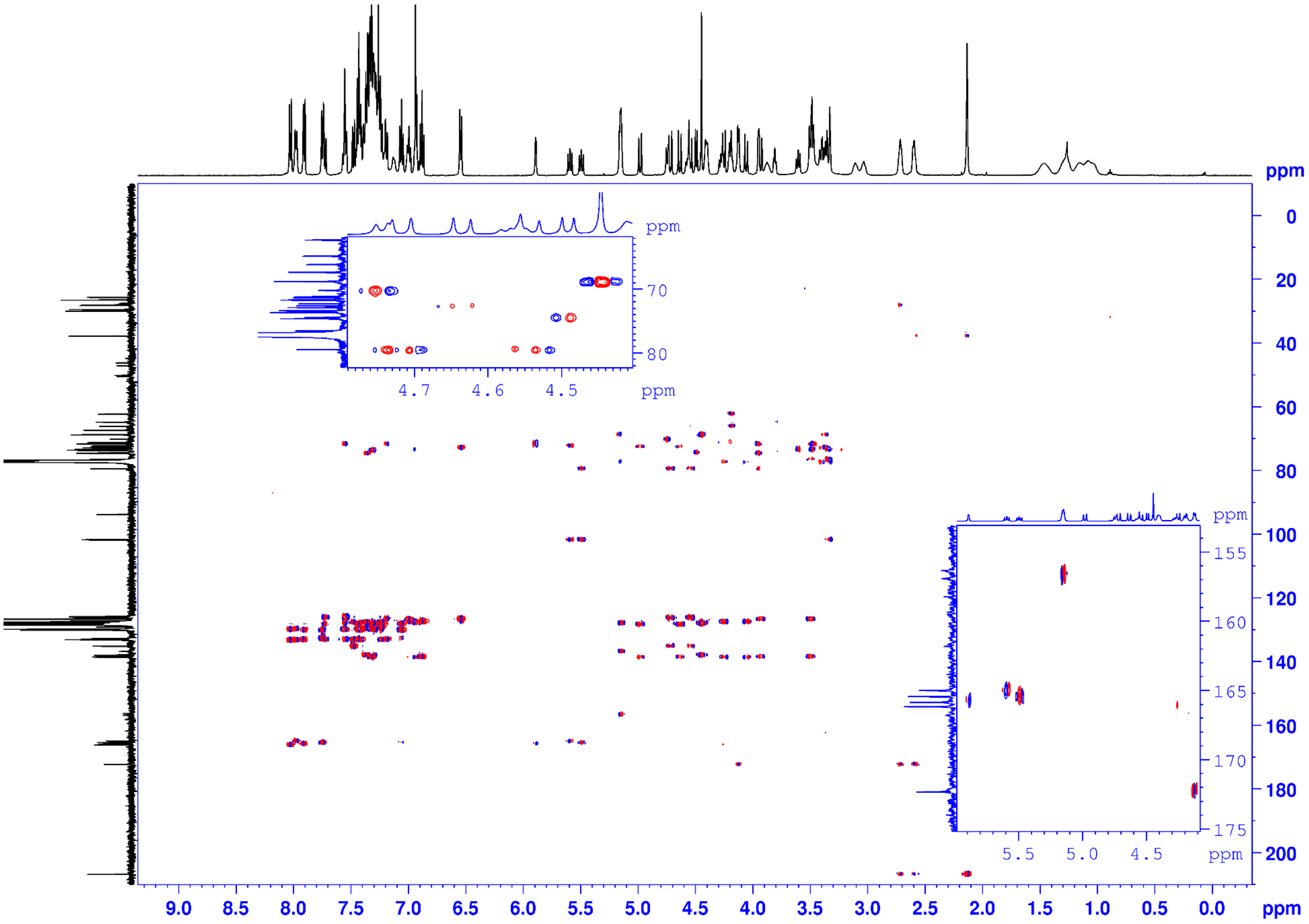

ppm
0
20
40
60
80
100
120
140
160
180
200
9.0
8.5
8.0
7.5
7.0
6.5
6.0
5.5
5.0
4.5
4.0
3.5
3.0
2.5
2.0
1.5
1.0
0.5
0.0
ppm
4.7
4.6
4.5
70
80
155
160
165
170
175
5.5
5.0
4.5

$^{13}C\{^1H\}$ NMR

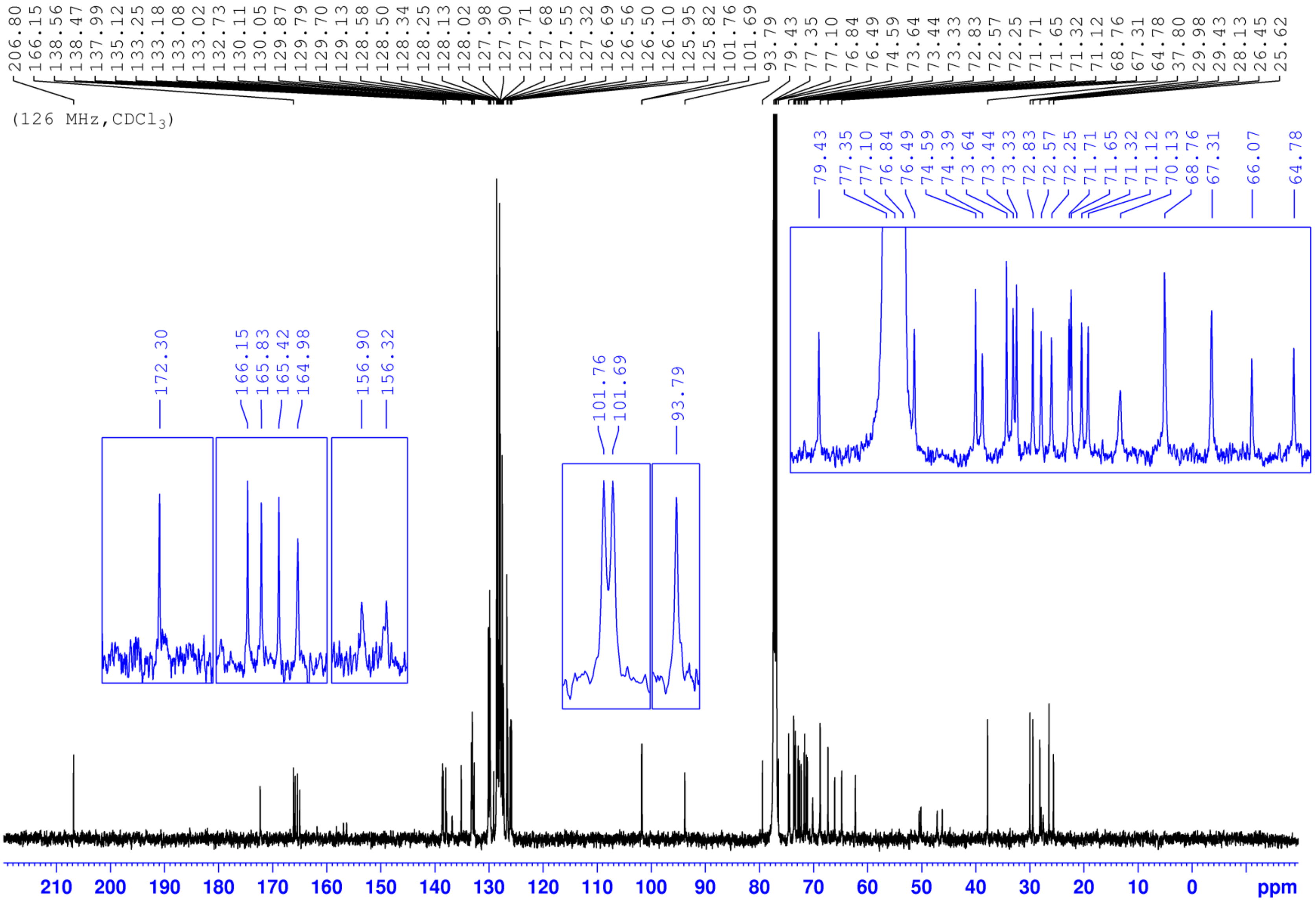

Compound **33**

^{1}H-NMR

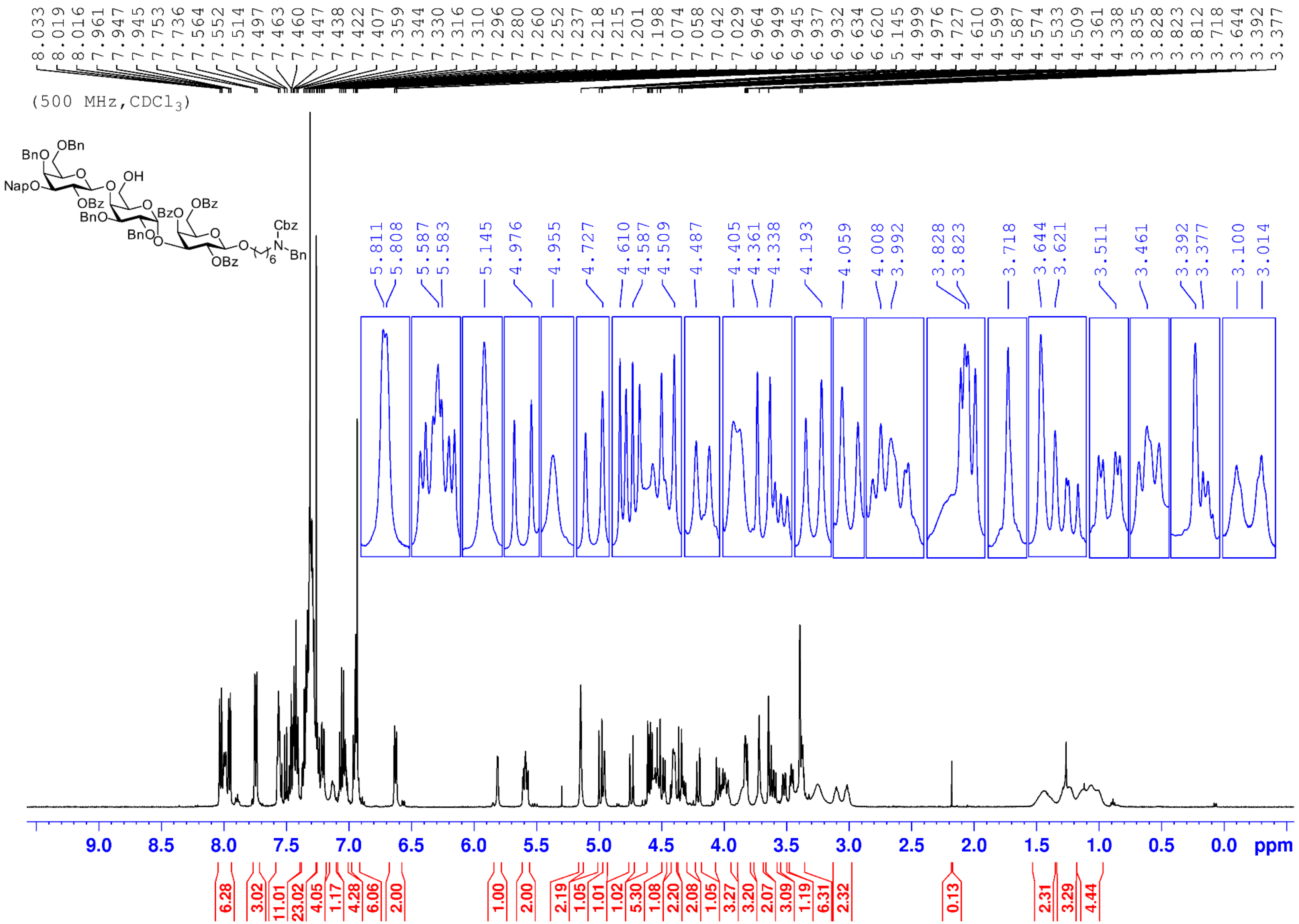

$^{1}H-^{1}H$ COSY

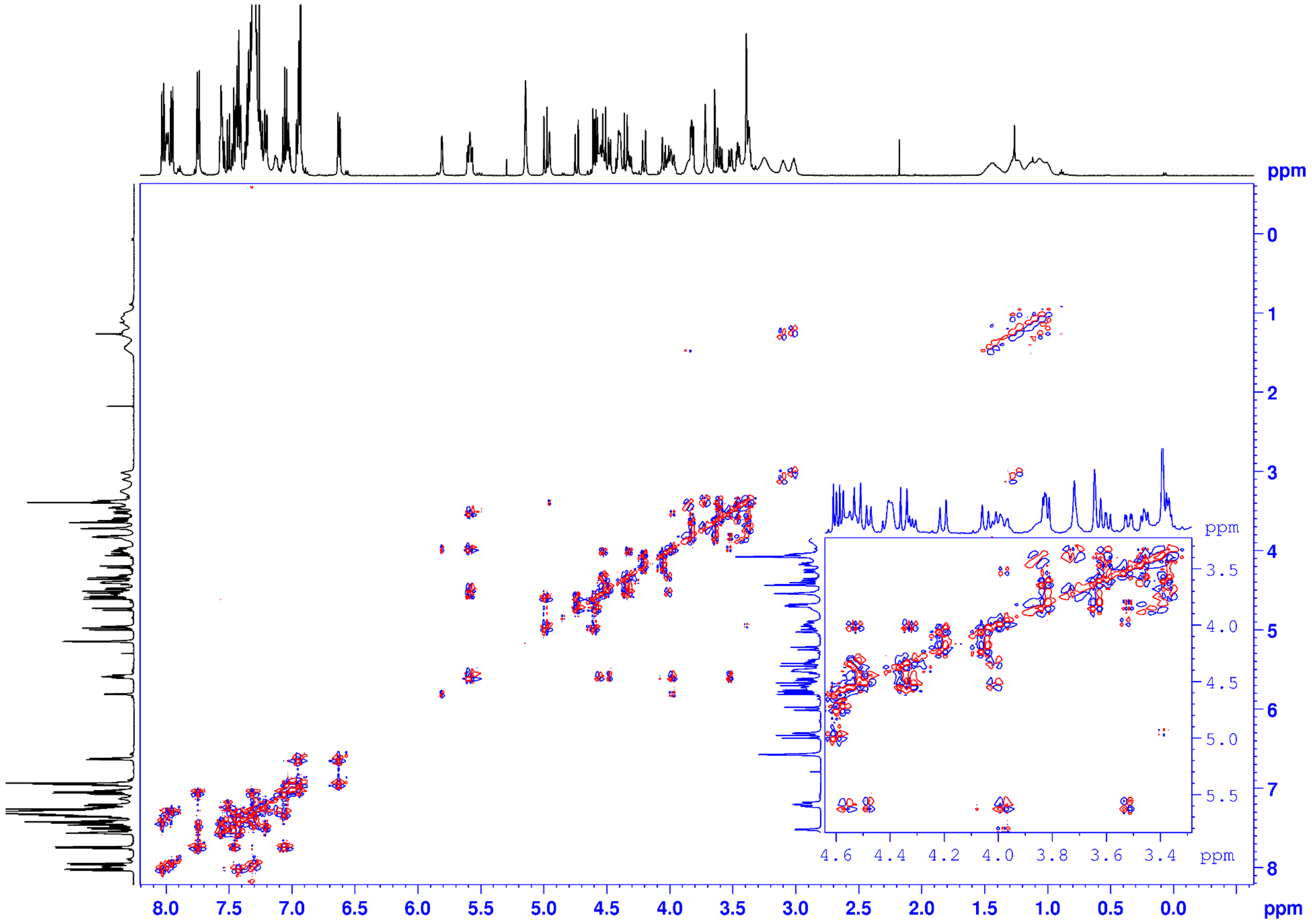

ppm
0
1
2
3
4
5
6
7
8
8.0
7.5
7.0
6.5
6.0
5.5
5.0
4.5
4.0
3.5
3.0
2.5
2.0
1.5
1.0
0.5
0.0
ppm
3.5
4.0
4.5
5.0
5.5
4.6
4.4
4.2
4.0
3.8
3.6
3.4
ppm

^{1}H-^{13}C HSQC

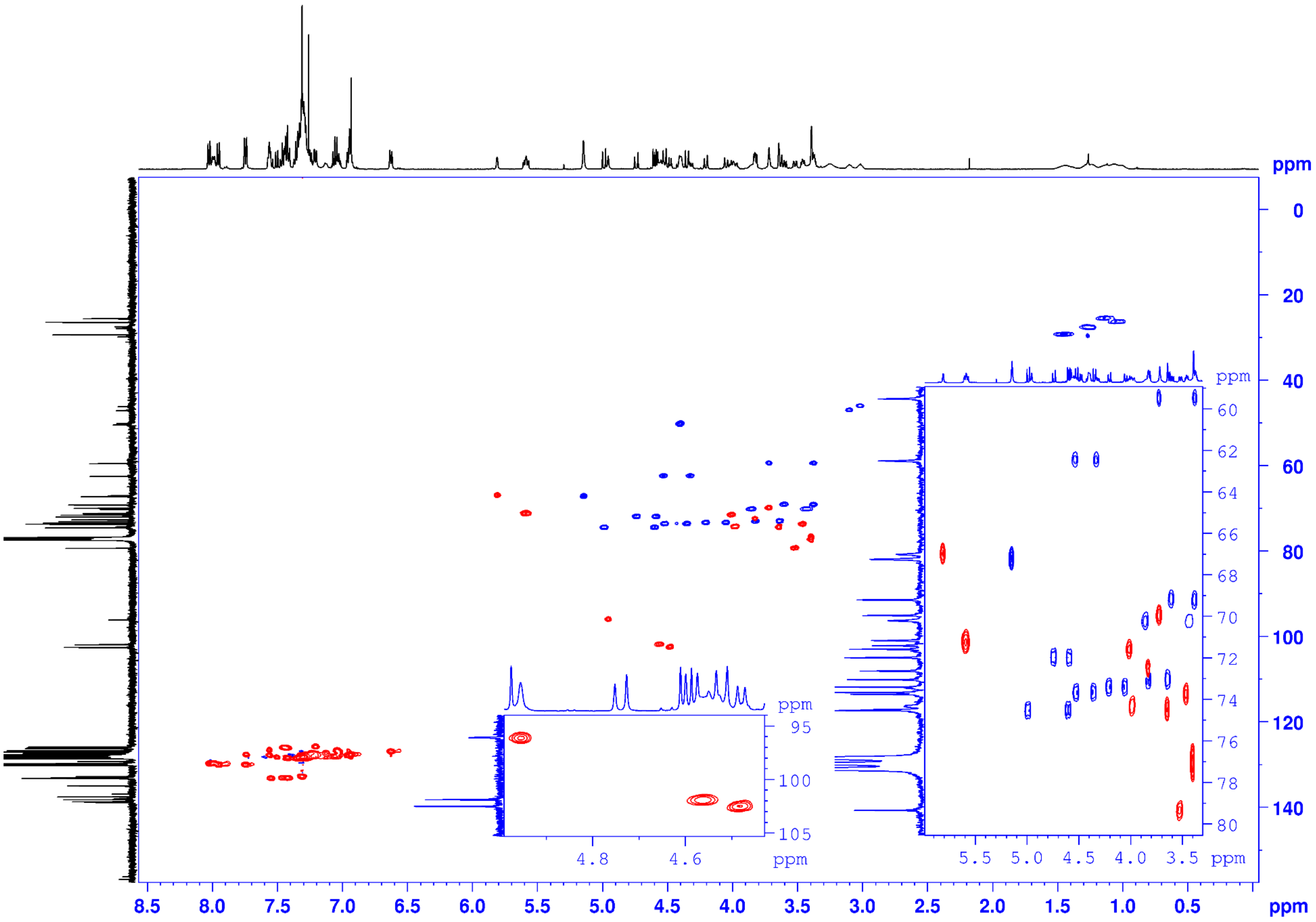
ppm
0
20
40
60
80
100
120
140
8.5
8.0
7.5
7.0
6.5
6.0
5.5
5.0
4.5
4.0
3.5
3.0
2.5
2.0
1.5
1.0
0.5
ppm
95
100
105
4.8
4.6
ppm
60
62
64
66
68
70
72
74
76
78
80
5.5
5.0
4.5
4.0
3.5
ppm

^{1}H-^{13}C non-decoupled HSQC

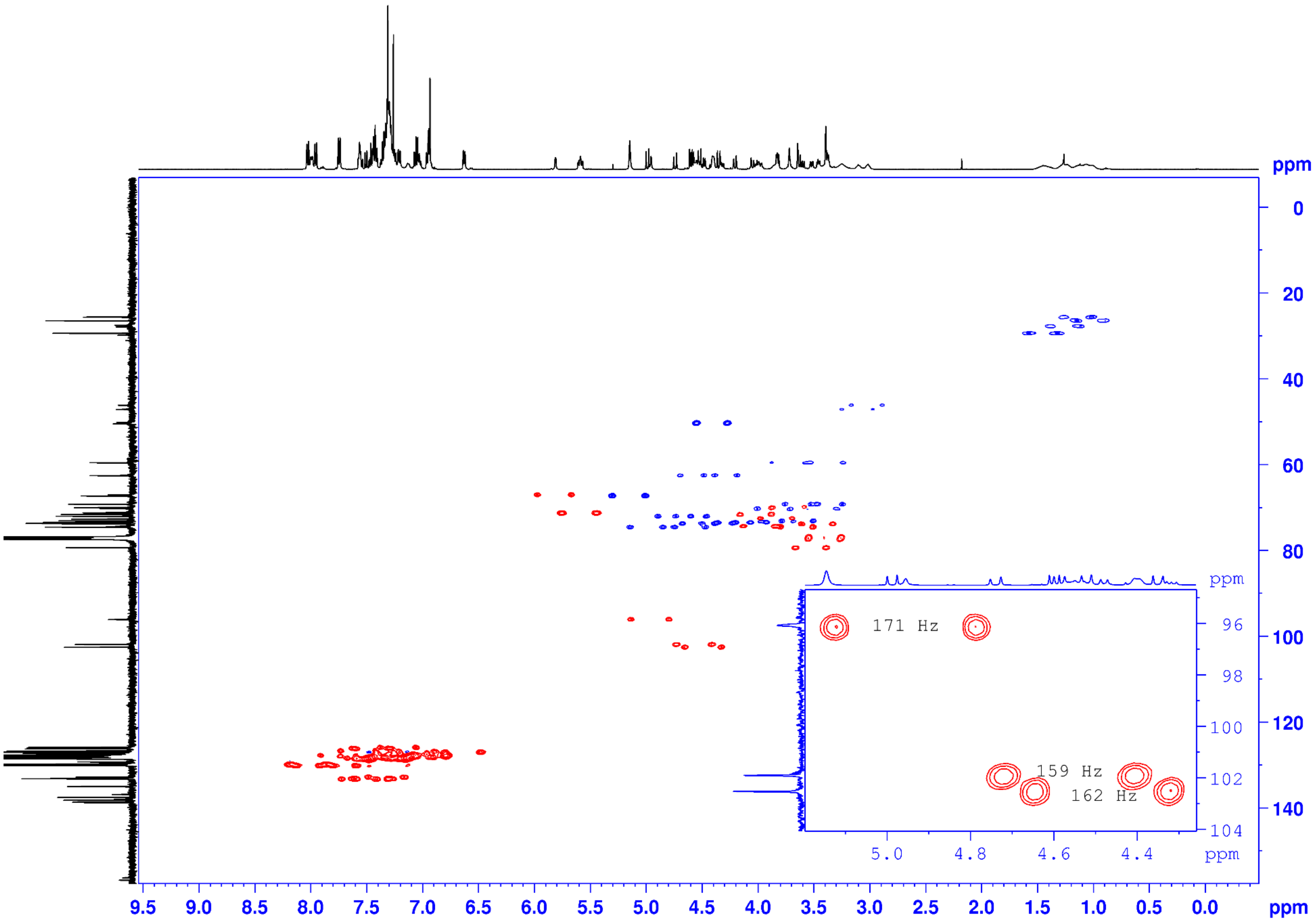

^{1}H-^{13}C HMBC

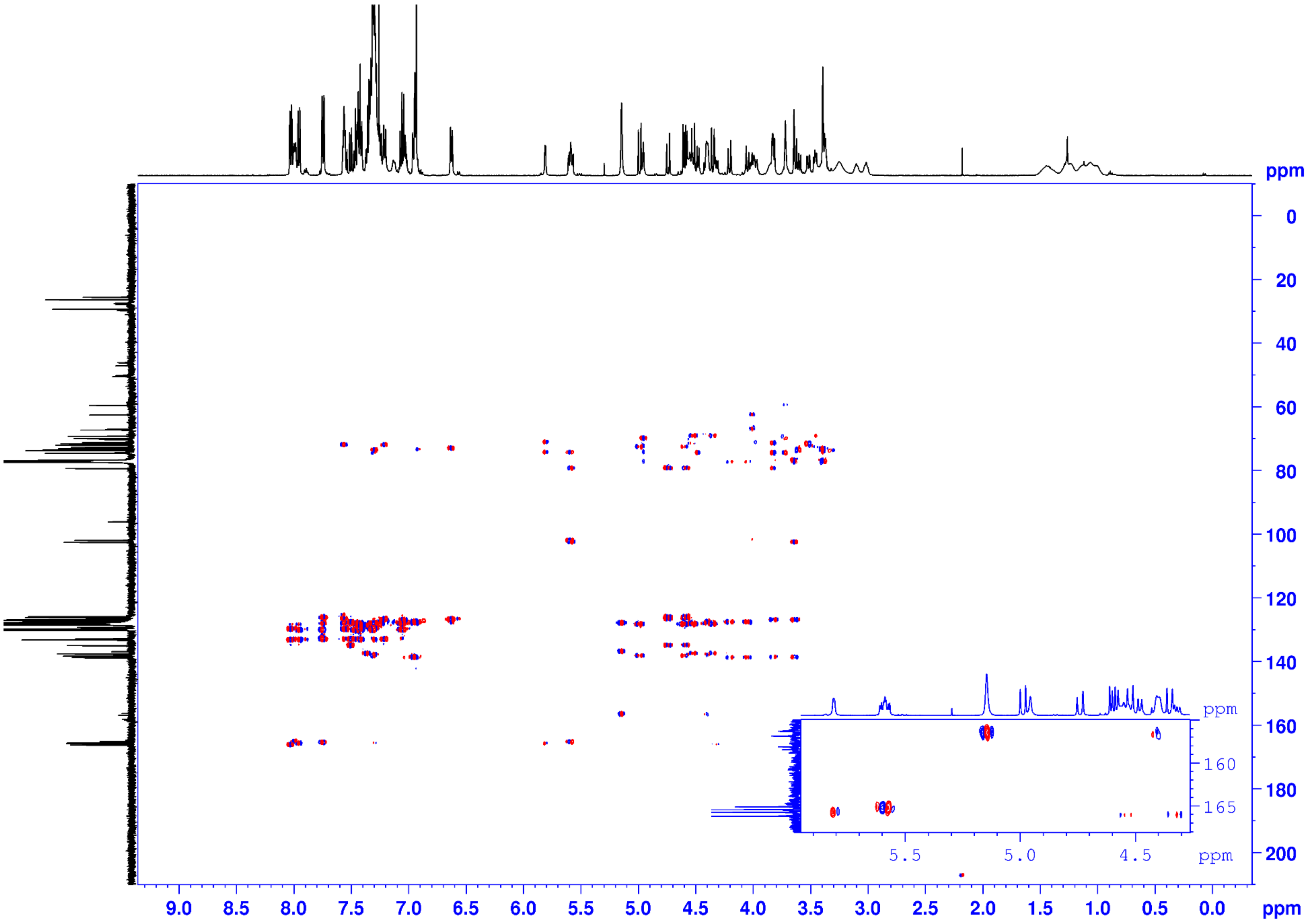
ppm
0
20
40
60
80
100
120
140
160
180
200
9.0 8.5 8.0 7.5 7.0 6.5 6.0 5.5 5.0 4.5 4.0 3.5 3.0 2.5 2.0 1.5 1.0 0.5 0.0
ppm
160
165
5.5 5.0 4.5
ppm

$^{13}C\{^1H\}$ NMR

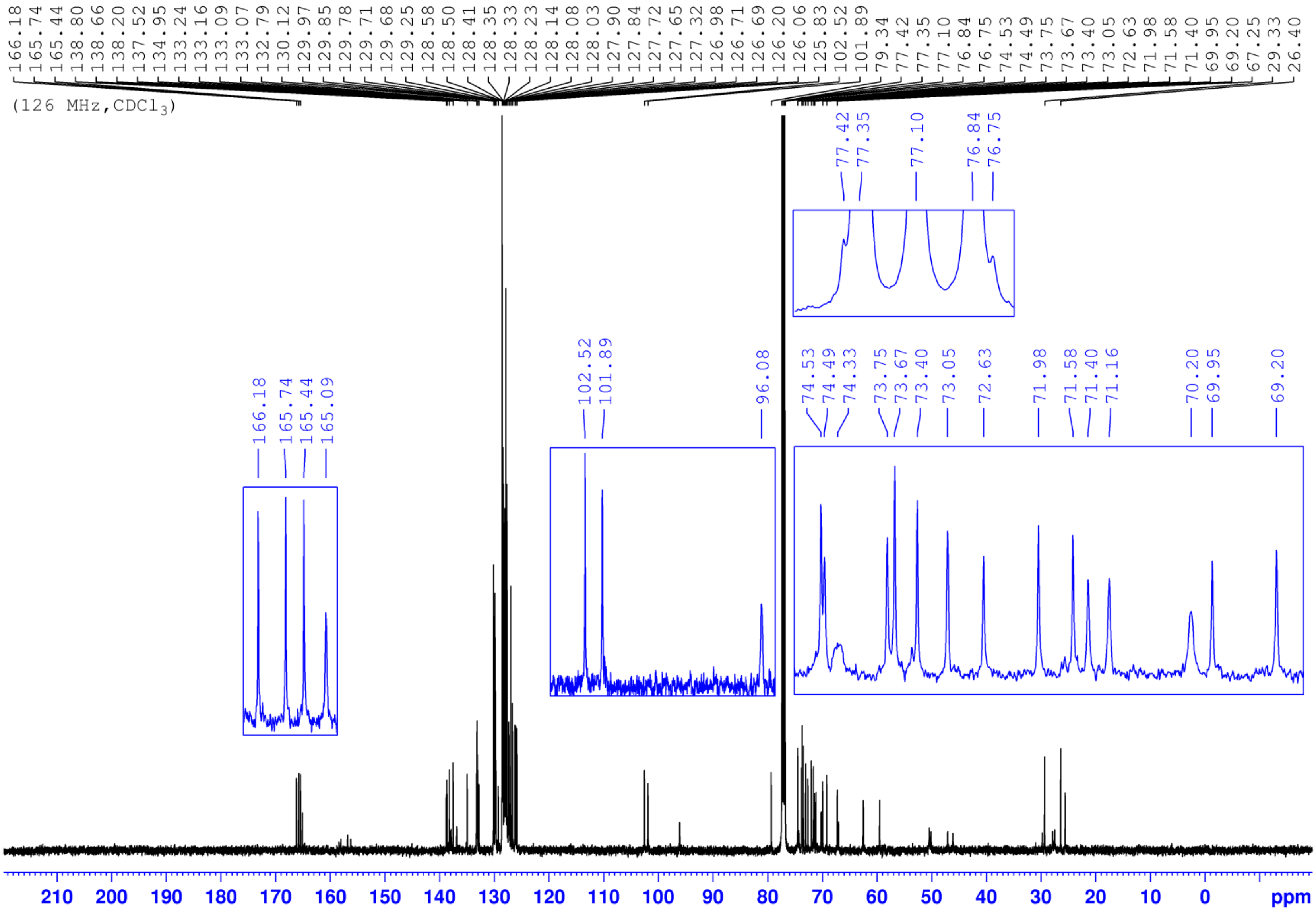

166.18
165.74
165.44
138.80
138.66
138.20
137.52
134.95
133.24
133.16
133.09
133.07
132.79
130.12
129.97
129.85
129.78
129.71
129.68
129.25
128.58
128.50
128.41
128.35
128.33
128.23
128.14
128.08
128.03
127.90
127.84
127.72
127.65
127.32
126.98
126.71
126.69
126.20
126.06
125.83
102.52
101.89
79.34
77.42
77.35
77.10
76.84
76.75
74.53
74.49
73.75
73.67
73.40
73.05
72.63
71.98
71.58
71.40
69.95
69.20
67.25
29.33
26.40
(126 MHz,CDCl3)
166.18
165.74
165.44
165.09
102.52
101.89
96.08
74.53
74.49
74.33
73.75
73.67
73.40
73.05
72.63
71.98
71.58
71.40
71.16
70.20
69.95
69.20
ppm

Compound **34**

^{1}H-NMR

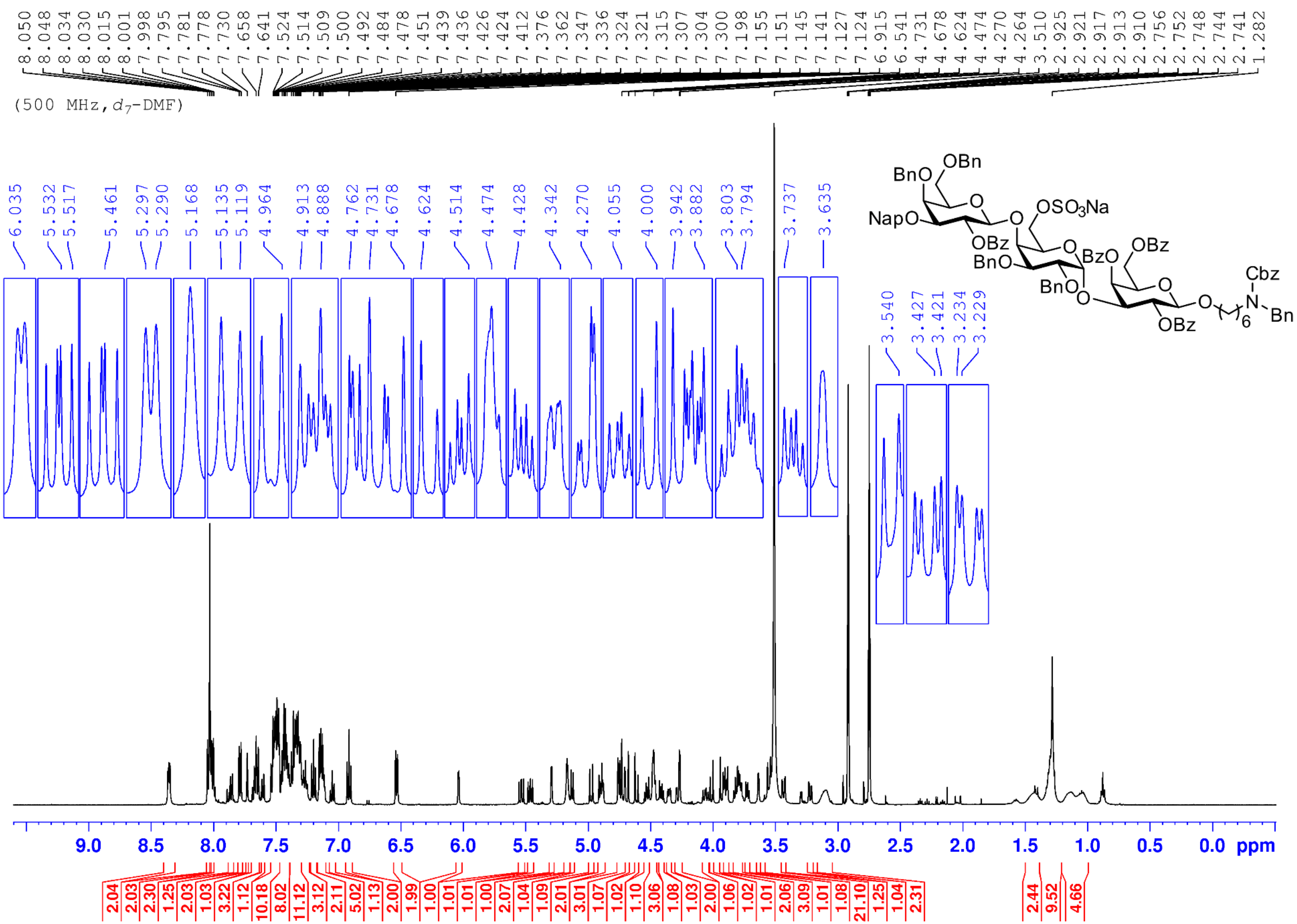

^1H-^1H COSY

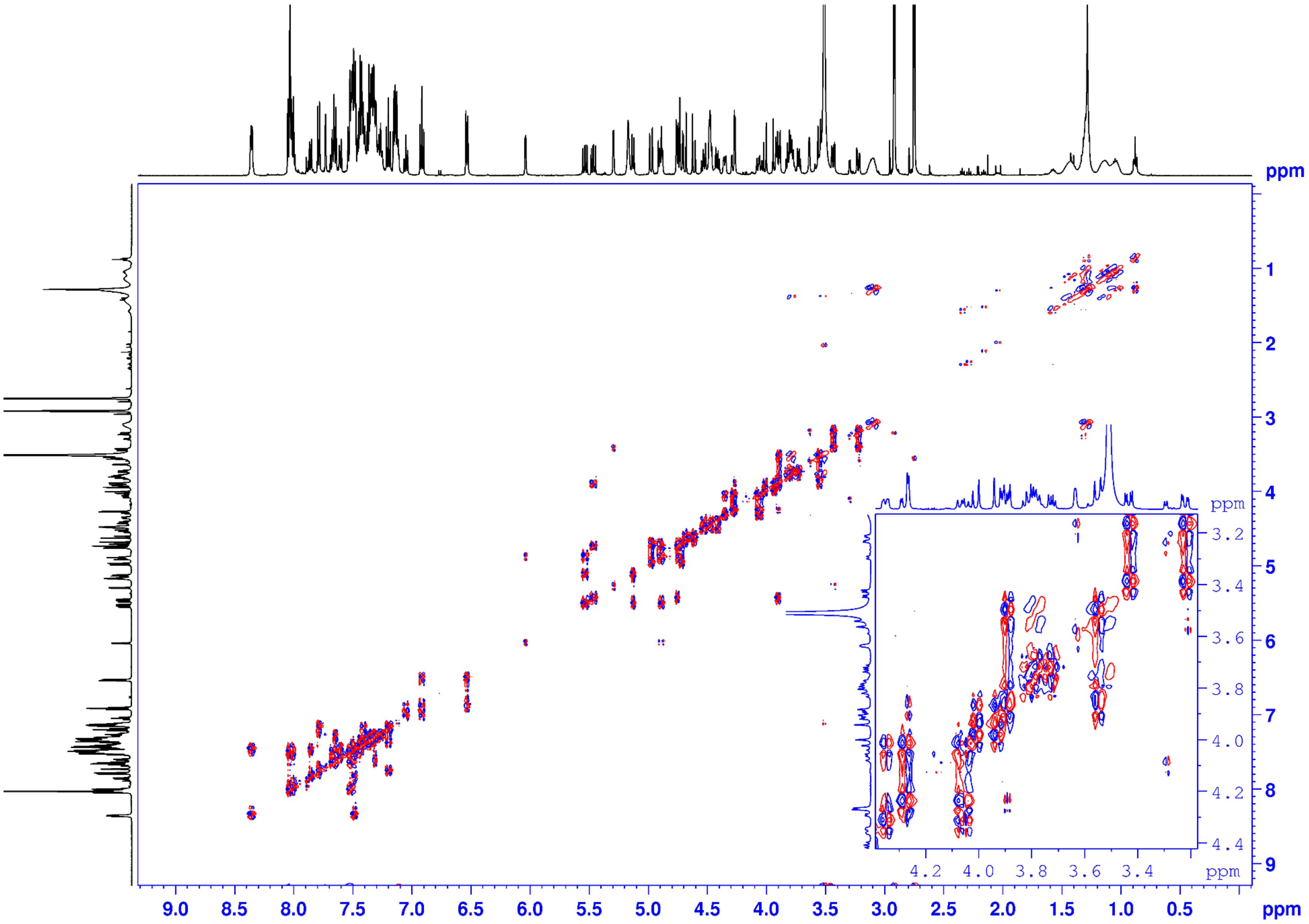
ppm
9.0
8.5
8.0
7.5
7.0
6.5
6.0
5.5
5.0
4.5
4.0
3.5
3.0
2.5
2.0
1.5
1.0
0.5
ppm
1
2
3
4
5
6
7
8
9
4.2
4.0
3.8
3.6
3.4
ppm
3.2
3.4
3.6
3.8
4.0
4.2
4.4
ppm

^{1}H-^{13}C HSQC

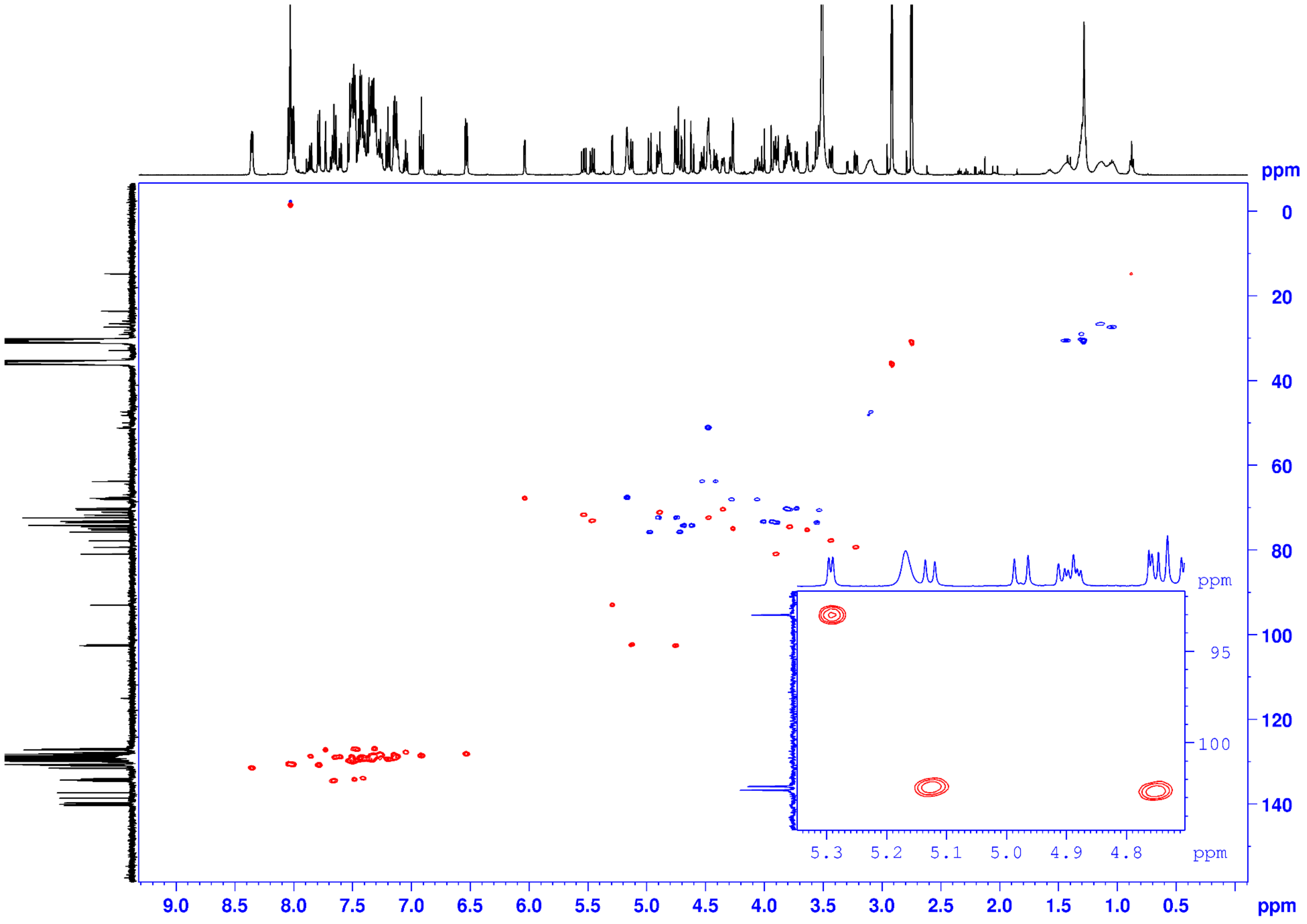
ppm
0
20
40
60
80
100
120
140
9.0
8.5
8.0
7.5
7.0
6.5
6.0
5.5
5.0
4.5
4.0
3.5
3.0
2.5
2.0
1.5
1.0
0.5
ppm
95
100
5.3
5.2
5.1
5.0
4.9
4.8
ppm

^{1}H-^{13}C non-decoupled HSQC

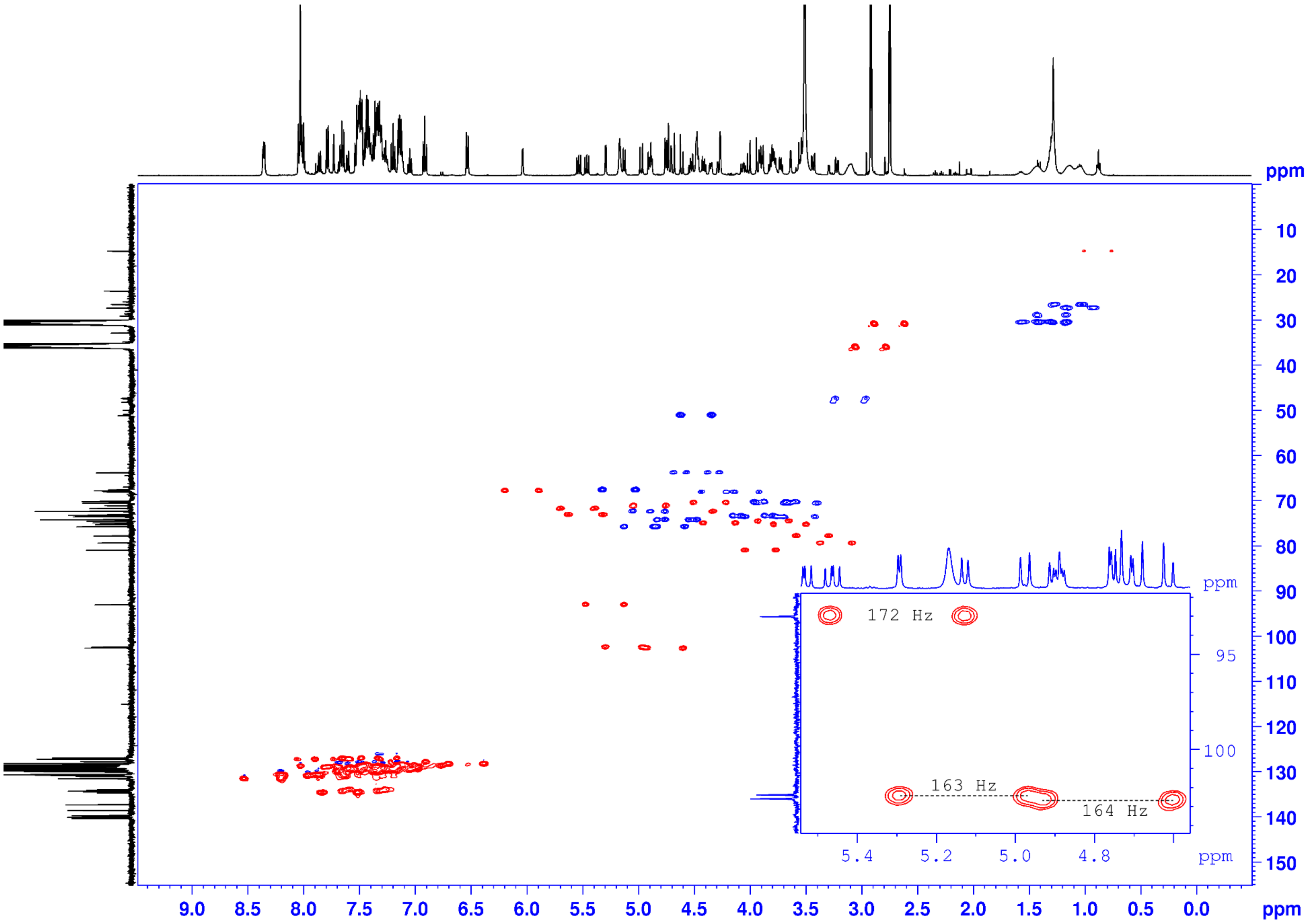

^{1}H-^{13}C HMBC

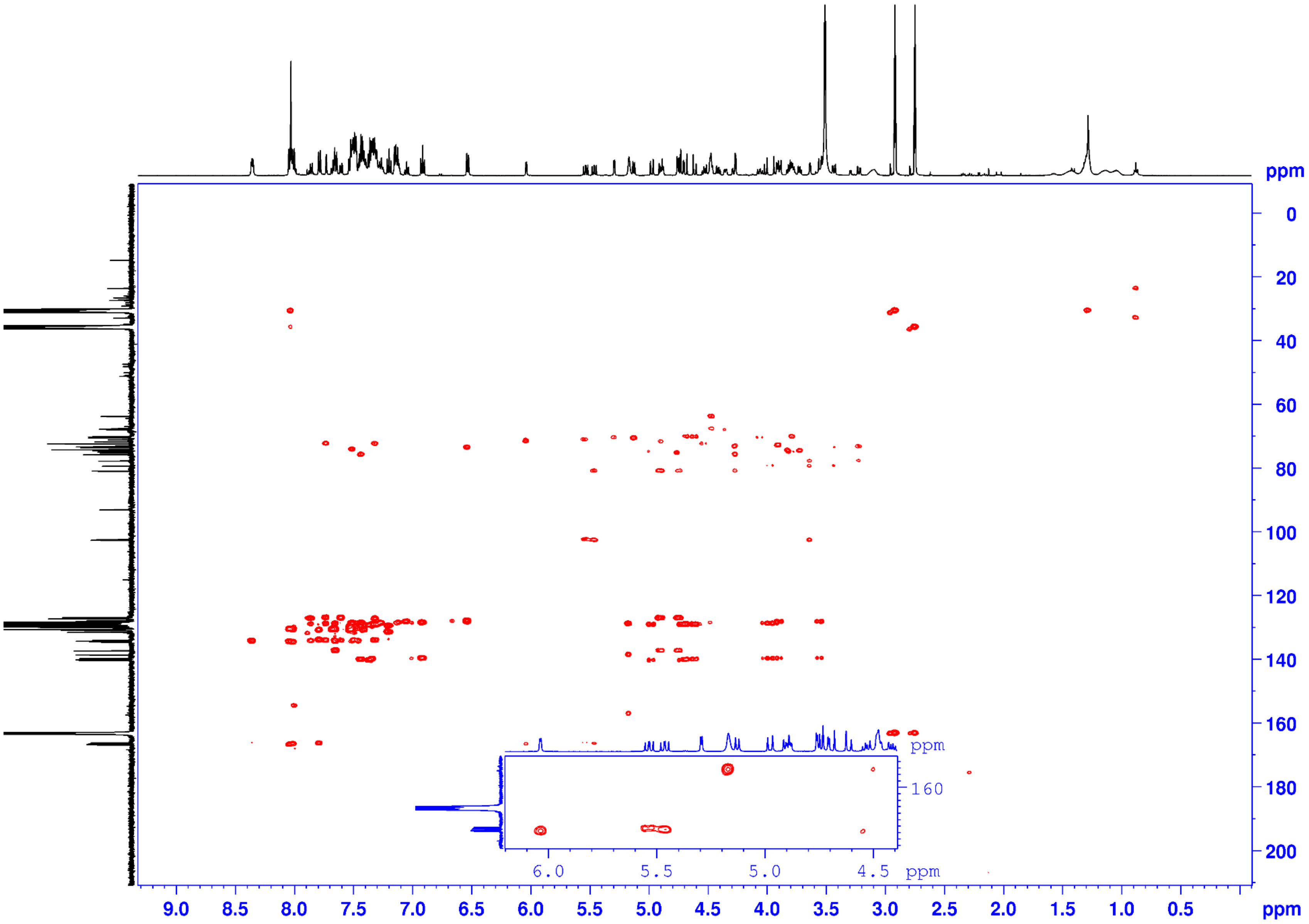
ppm
0
20
40
60
80
100
120
140
160
180
200
9.0
8.5
8.0
7.5
7.0
6.5
6.0
5.5
5.0
4.5
4.0
3.5
3.0
2.5
2.0
1.5
1.0
0.5
ppm
160
6.0
5.5
5.0
4.5
ppm

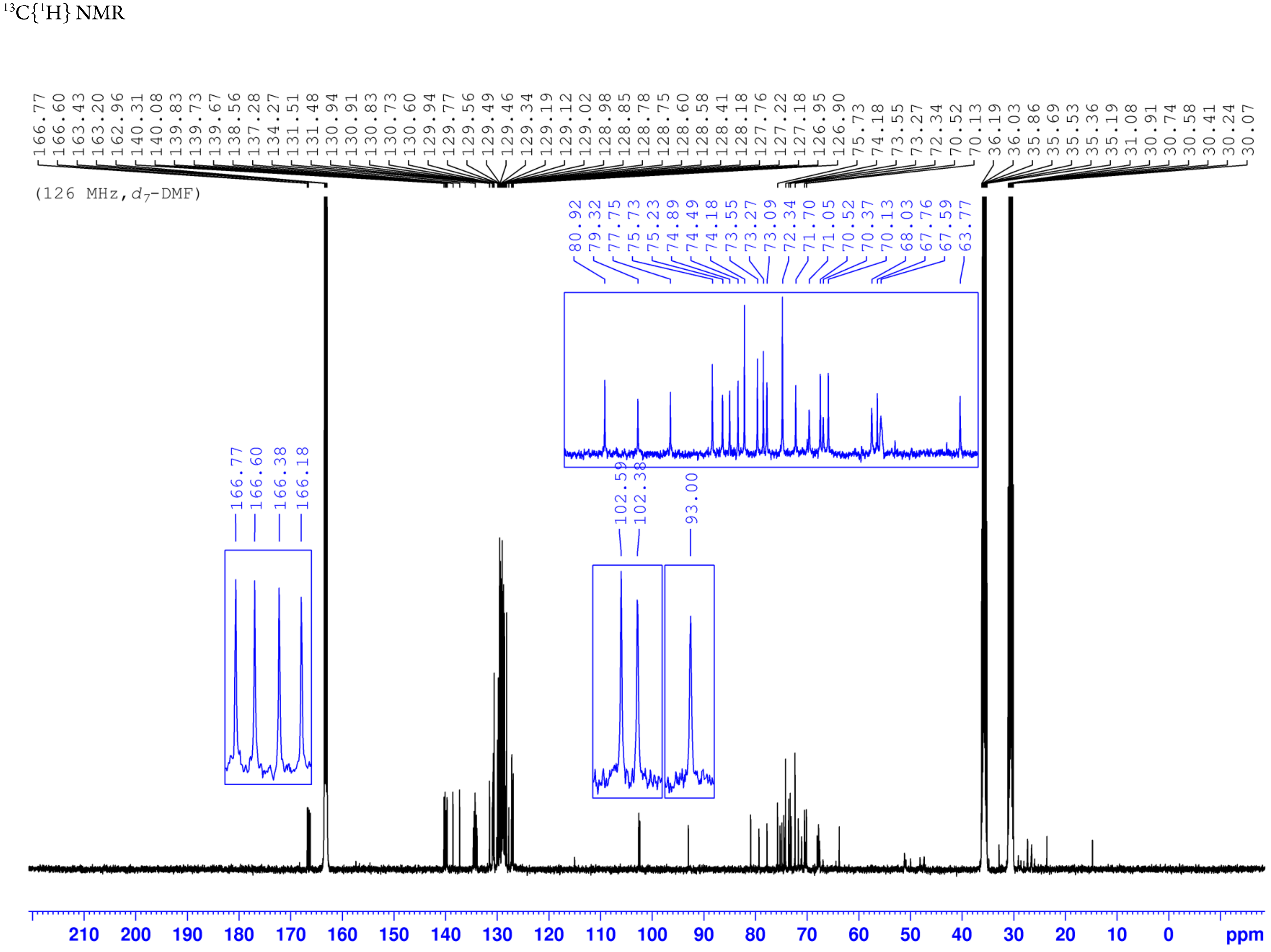
13C{1H} NMR
(126 MHz, d7-DMF)
166.77 166.60 163.43 163.20 162.96 140.31 140.08 139.83 139.73 139.67 138.56 137.28 134.27 131.51 131.48 130.94 130.91 130.83 130.73 130.60 129.94 129.77 129.56 129.49 129.46 129.34 129.19 129.12 129.02 128.98 128.85 128.78 128.75 128.60 128.58 128.41 128.18 127.76 127.22 127.18 126.95 126.90 75.73 74.18 73.55 73.27 72.34 70.52 70.13 36.19 36.03 35.86 35.69 35.53 35.36 35.19 31.08 30.91 30.74 30.58 30.41 30.24 30.07
80.92 79.32 77.75 75.73 75.23 74.89 74.49 74.18 73.55 73.27 73.09 72.34 71.70 71.05 70.52 70.37 70.13 68.03 67.76 67.59 63.77
166.77 166.60 166.38 166.18
102.59 102.38
93.00
210 200 190 180 170 160 150 140 130 120 110 100 90 80 70 60 50 40 30 20 10 0 ppm

Compound **35**

^{1}H-NMR

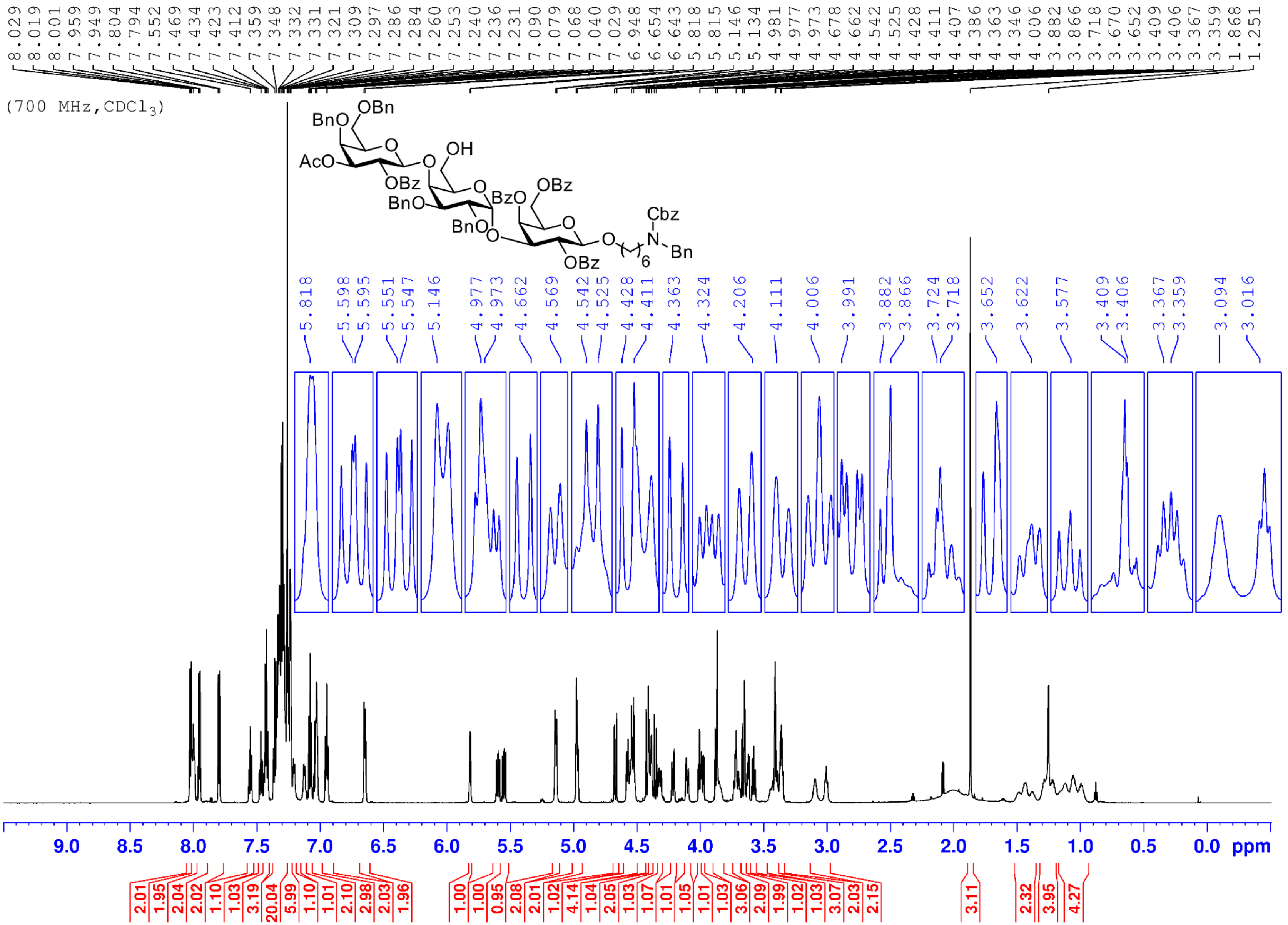

^{1}H-^{1}H COSY

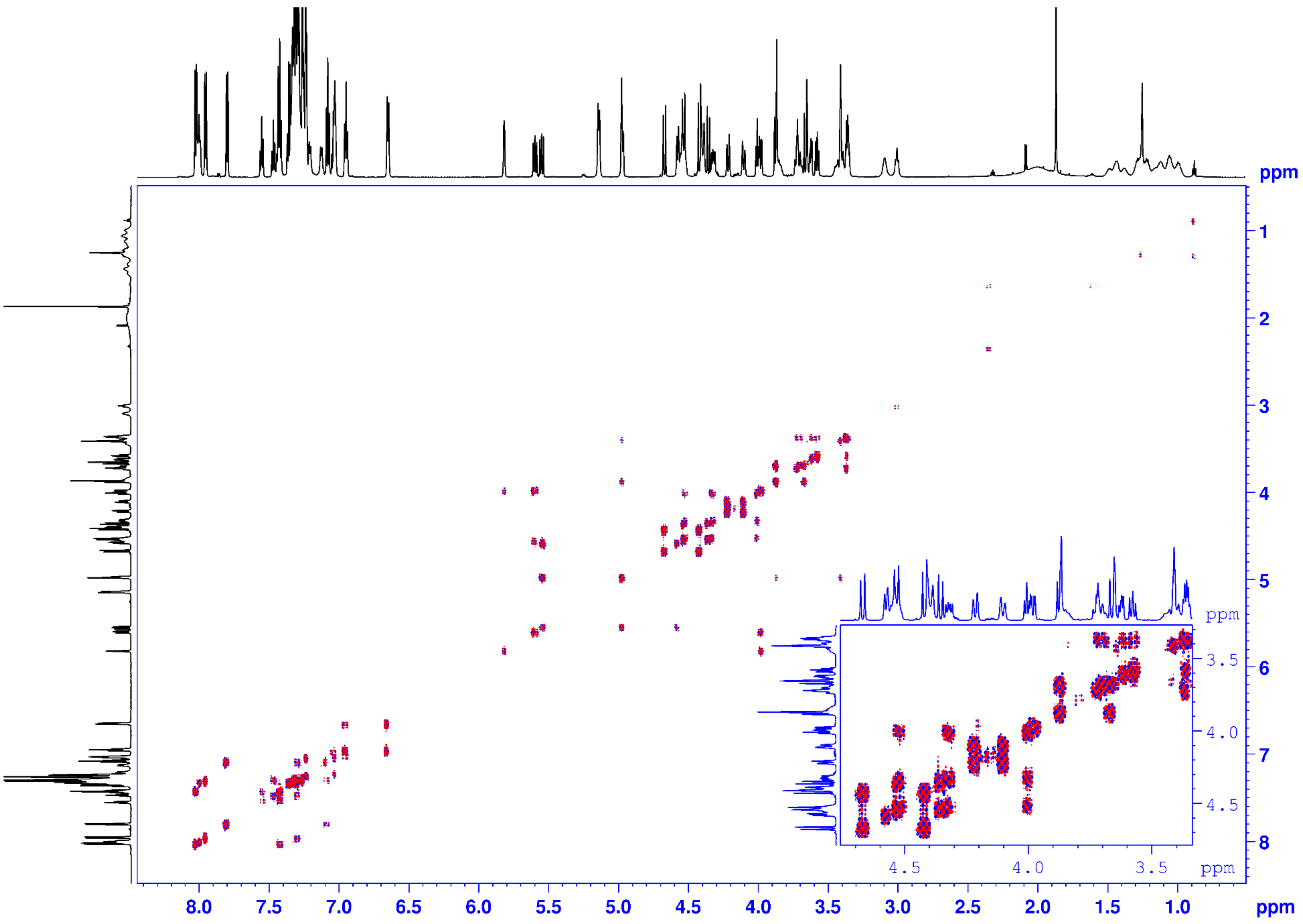
ppm
8.0
7.5
7.0
6.5
6.0
5.5
5.0
4.5
4.0
3.5
3.0
2.5
2.0
1.5
1.0
ppm
1
2
3
4
5
6
7
8
ppm
4.5
4.0
3.5
ppm
3.5
4.0
4.5
ppm

^{1}H-^{13}C HSQC

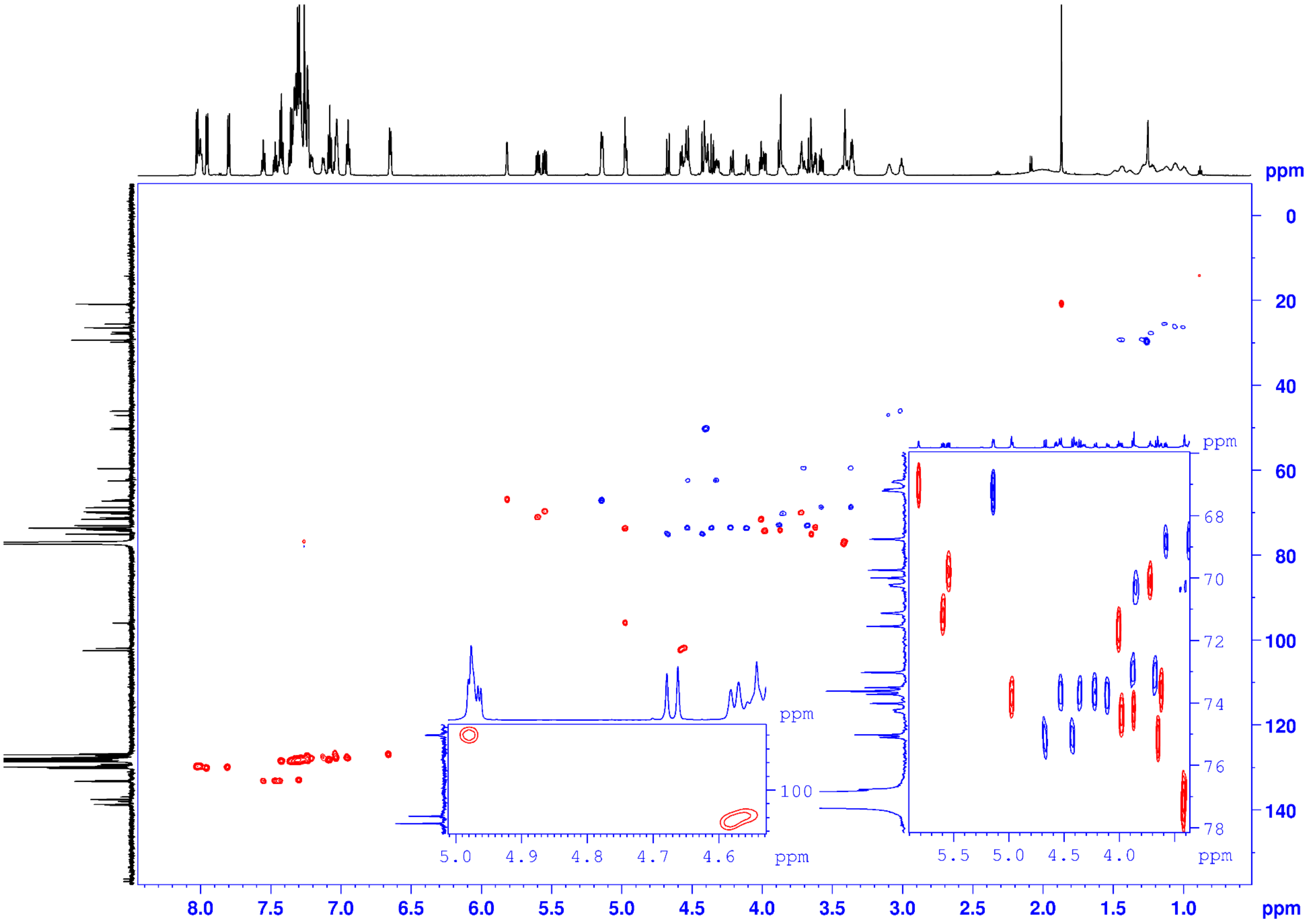

ppm
0
20
40
60
80
100
120
140
8.0
7.5
7.0
6.5
6.0
5.5
5.0
4.5
4.0
3.5
3.0
2.5
2.0
1.5
1.0
ppm
5.0
4.9
4.8
4.7
4.6
ppm
100
ppm
5.5
5.0
4.5
4.0
ppm
68
70
72
74
76
78
ppm

^{1}H-^{13}C non-decoupled HSQC

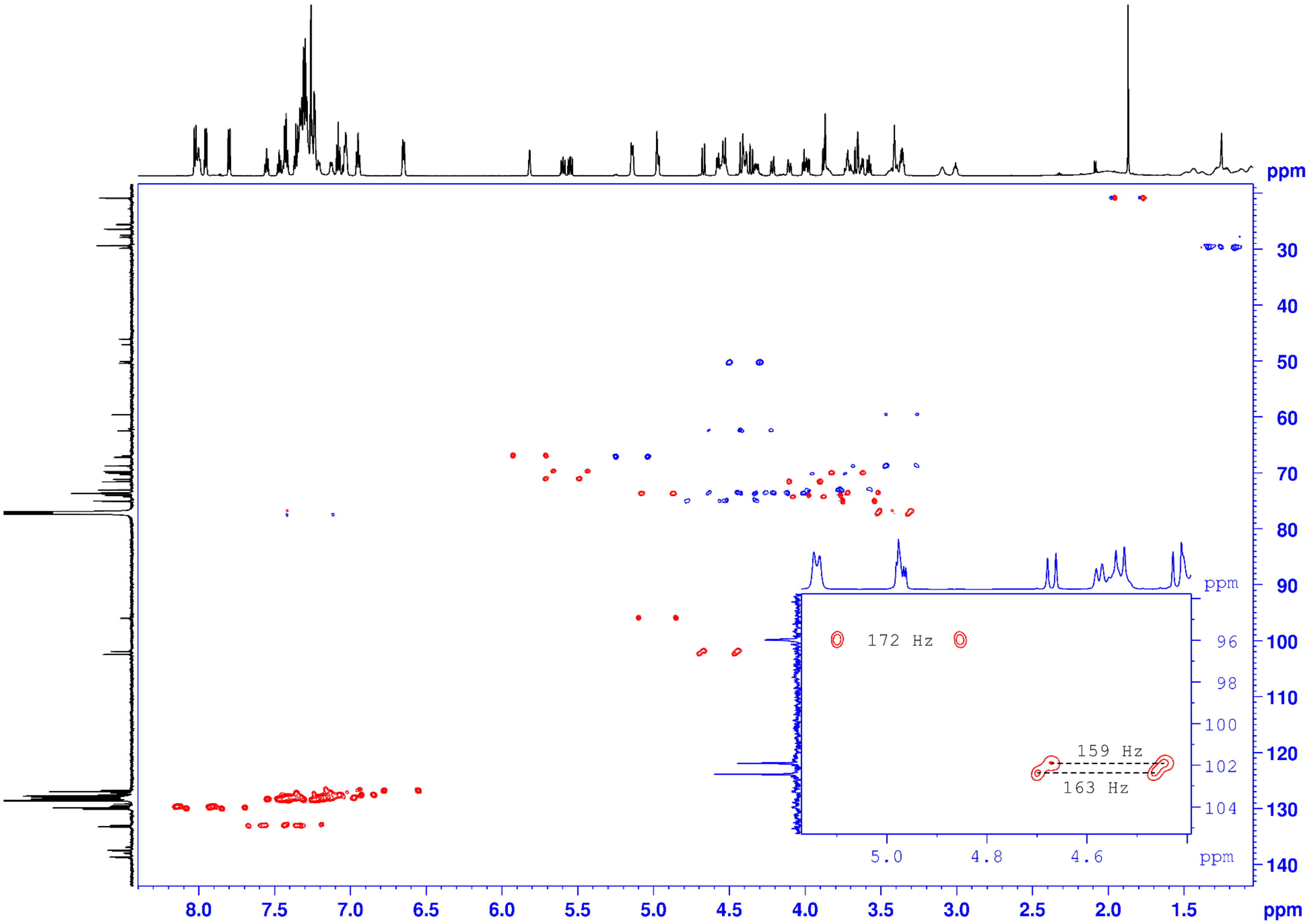

^{1}H-^{13}C HMBC

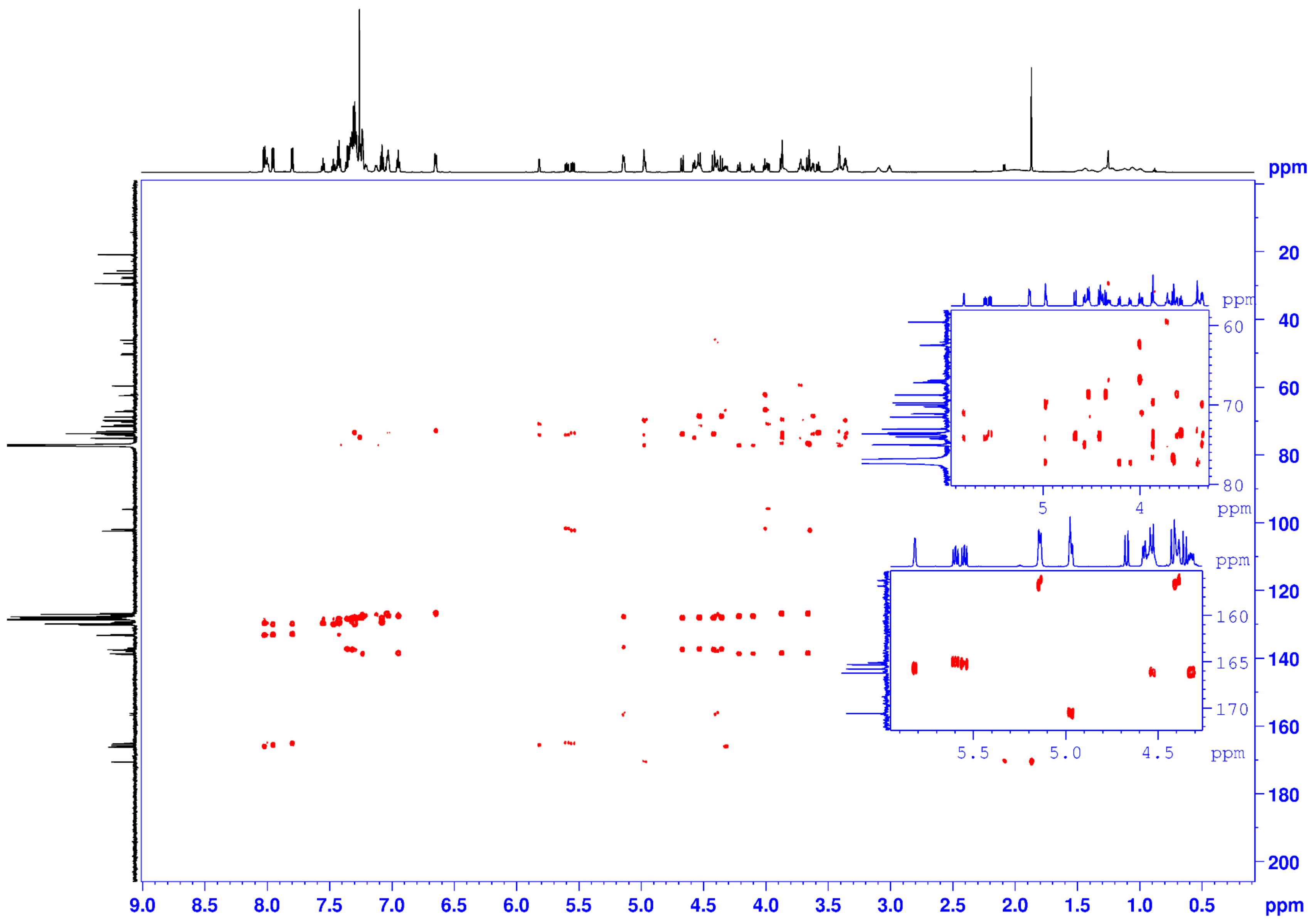

ppm
20
40
60
80
100
120
140
160
180
200
9.0
8.5
8.0
7.5
7.0
6.5
6.0
5.5
5.0
4.5
4.0
3.5
3.0
2.5
2.0
1.5
1.0
0.5
ppm

$^{13}C\{^{1}H\}$ NMR

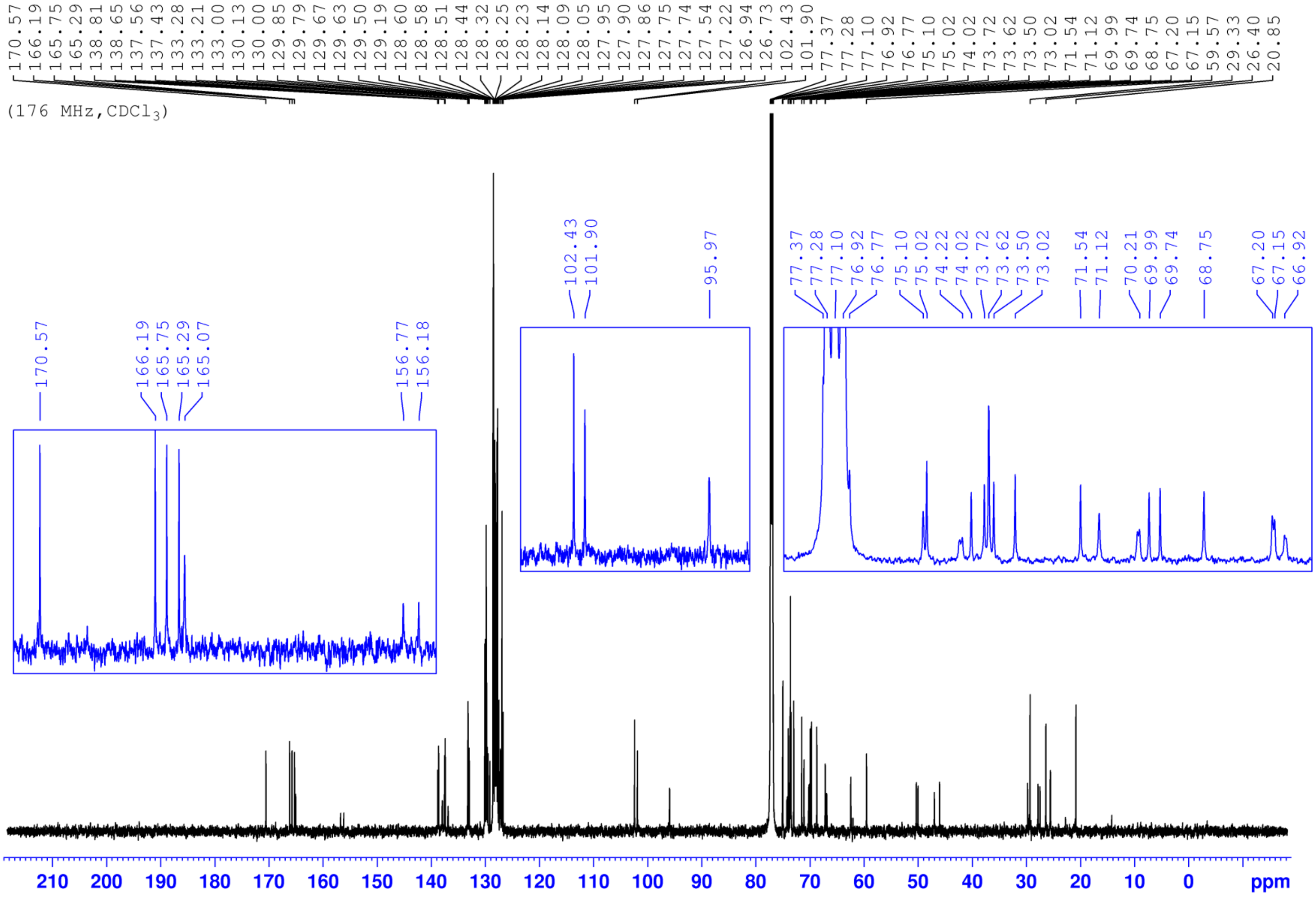

170.57
166.19
165.75
165.29
138.81
138.65
137.56
137.43
133.28
133.21
133.00
130.13
130.00
129.85
129.79
129.67
129.63
129.50
129.19
128.60
128.58
128.51
128.44
128.32
128.25
128.23
128.14
128.09
128.05
127.95
127.90
127.86
127.75
127.74
127.54
127.22
126.94
126.73
102.43
101.90
77.37
77.28
77.10
76.92
76.77
75.10
75.02
74.02
73.72
73.62
73.50
73.02
71.54
71.12
69.99
69.74
68.75
67.20
67.15
59.57
29.33
26.40
20.85
(176 MHz,CDCl3)
170.57
166.19
165.75
165.29
165.07
156.77
156.18
102.43
101.90
95.97
77.37
77.28
77.10
76.92
76.77
75.10
75.02
74.22
74.02
73.72
73.62
73.50
73.02
71.54
71.12
70.21
69.99
69.74
68.75
67.20
67.15
66.92
210
200
190
180
170
160
150
140
130
120
110
100
90
80
70
60
50
40
30
20
10
0
ppm

Compound **36**

^{1}H-NMR

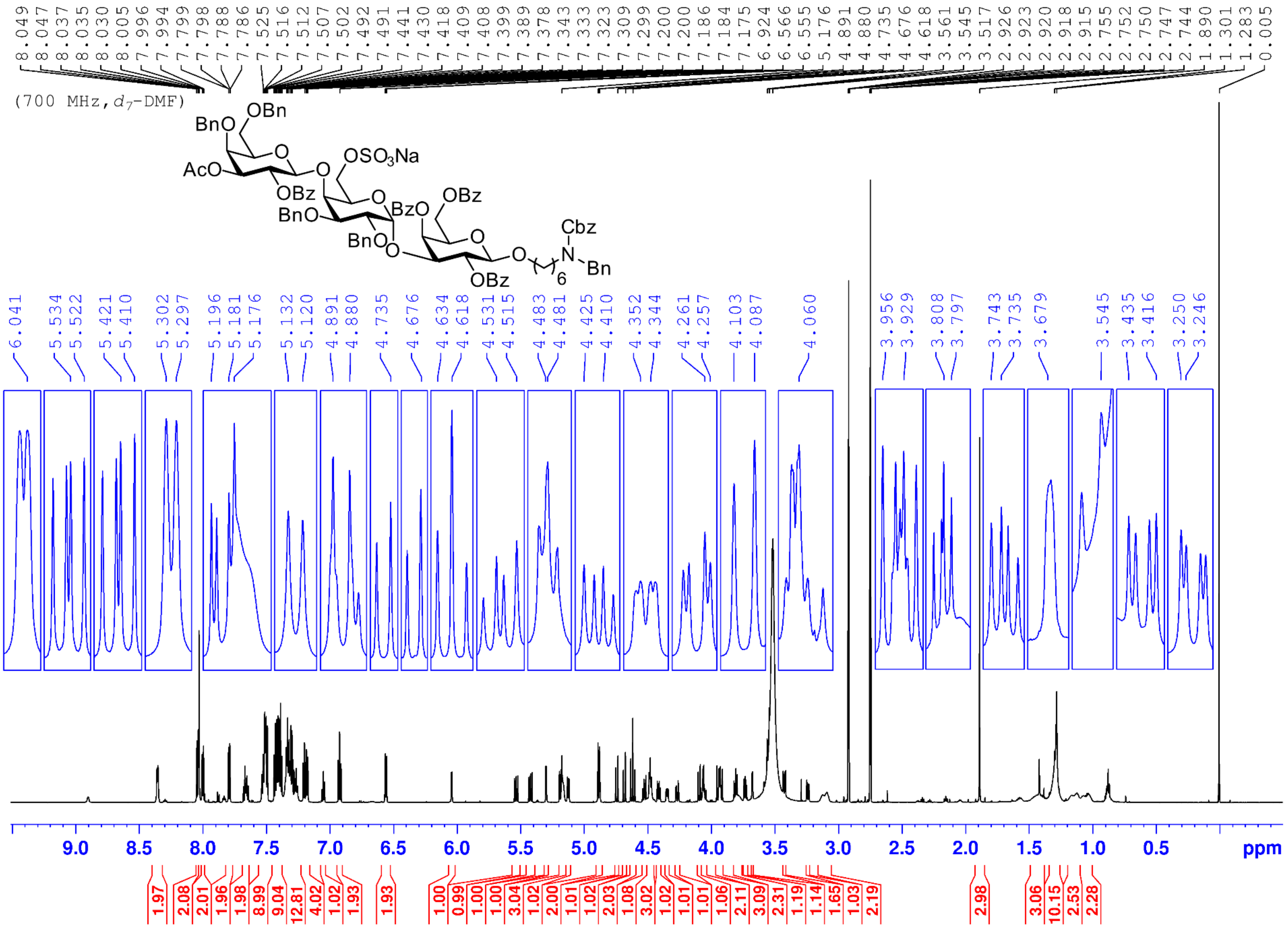

^{1}H-^{1}H COSY

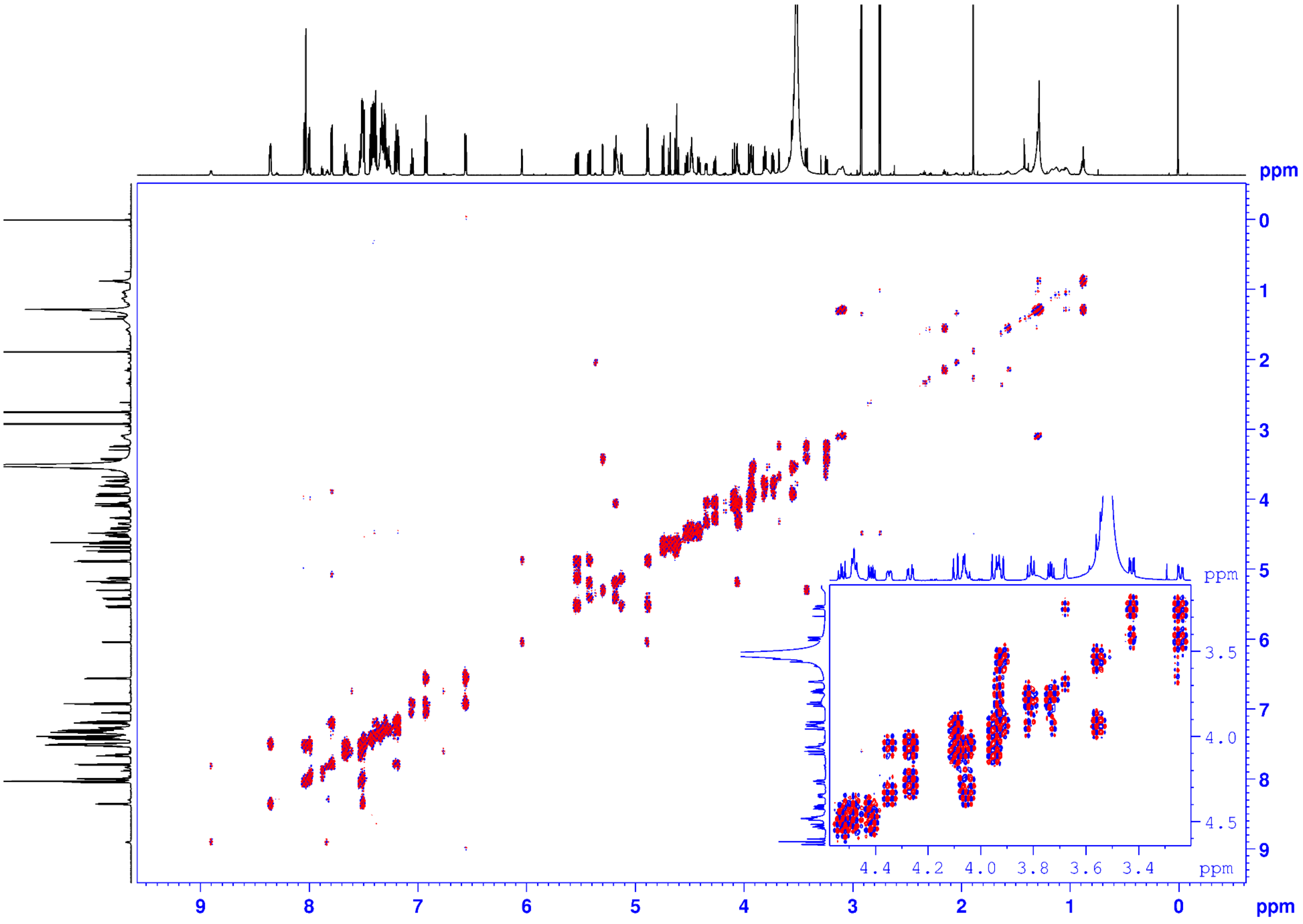

$^{1}H-^{13}C$ HSQC

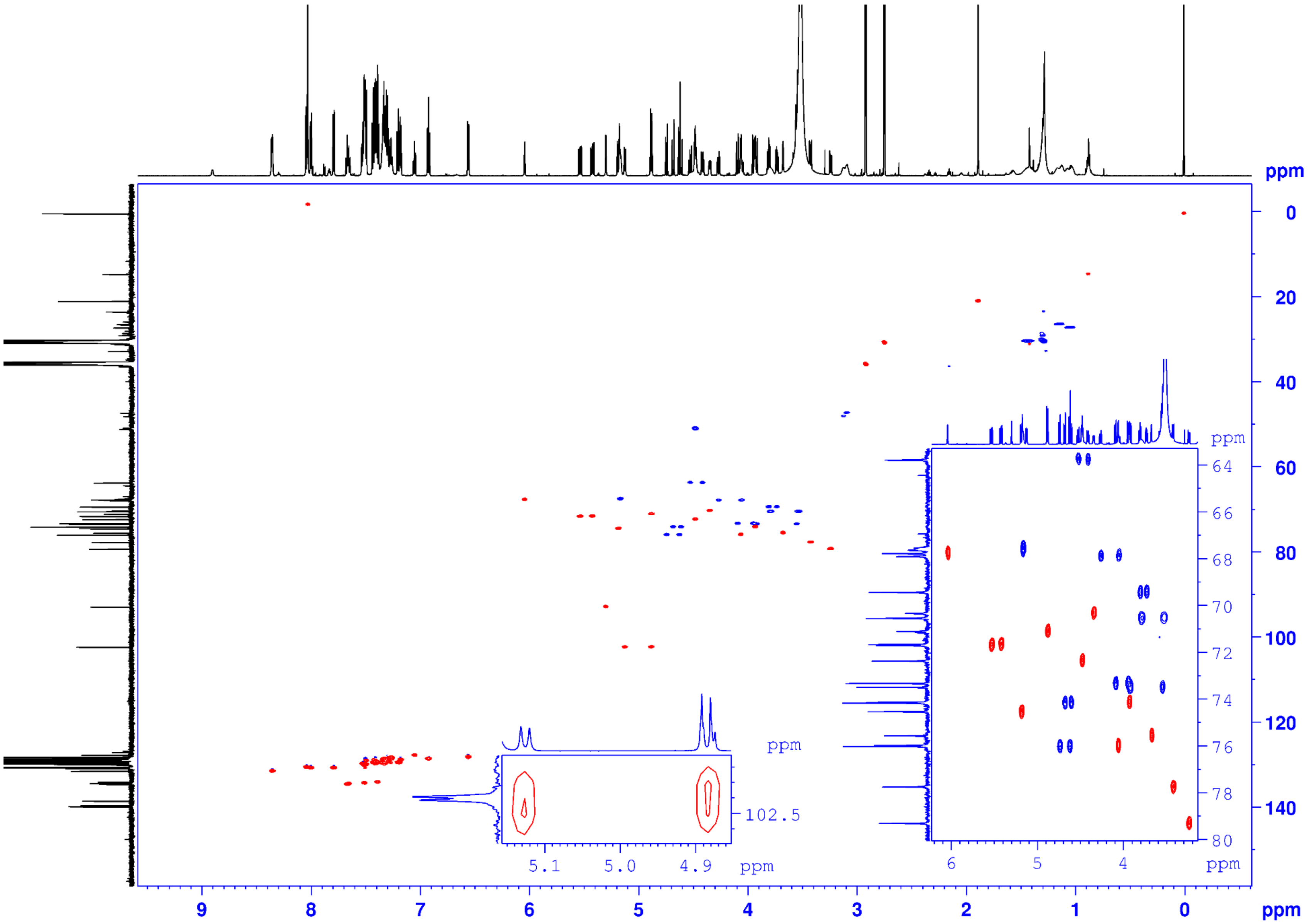

ppm
0
20
40
60
80
100
120
140
9
8
7
6
5
4
3
2
1
0
ppm
5.1
5.0
4.9
ppm
102.5
ppm
64
66
68
70
72
74
76
78
80
6
5
4
ppm

^{1}H-^{13}C non-decoupled HSQC

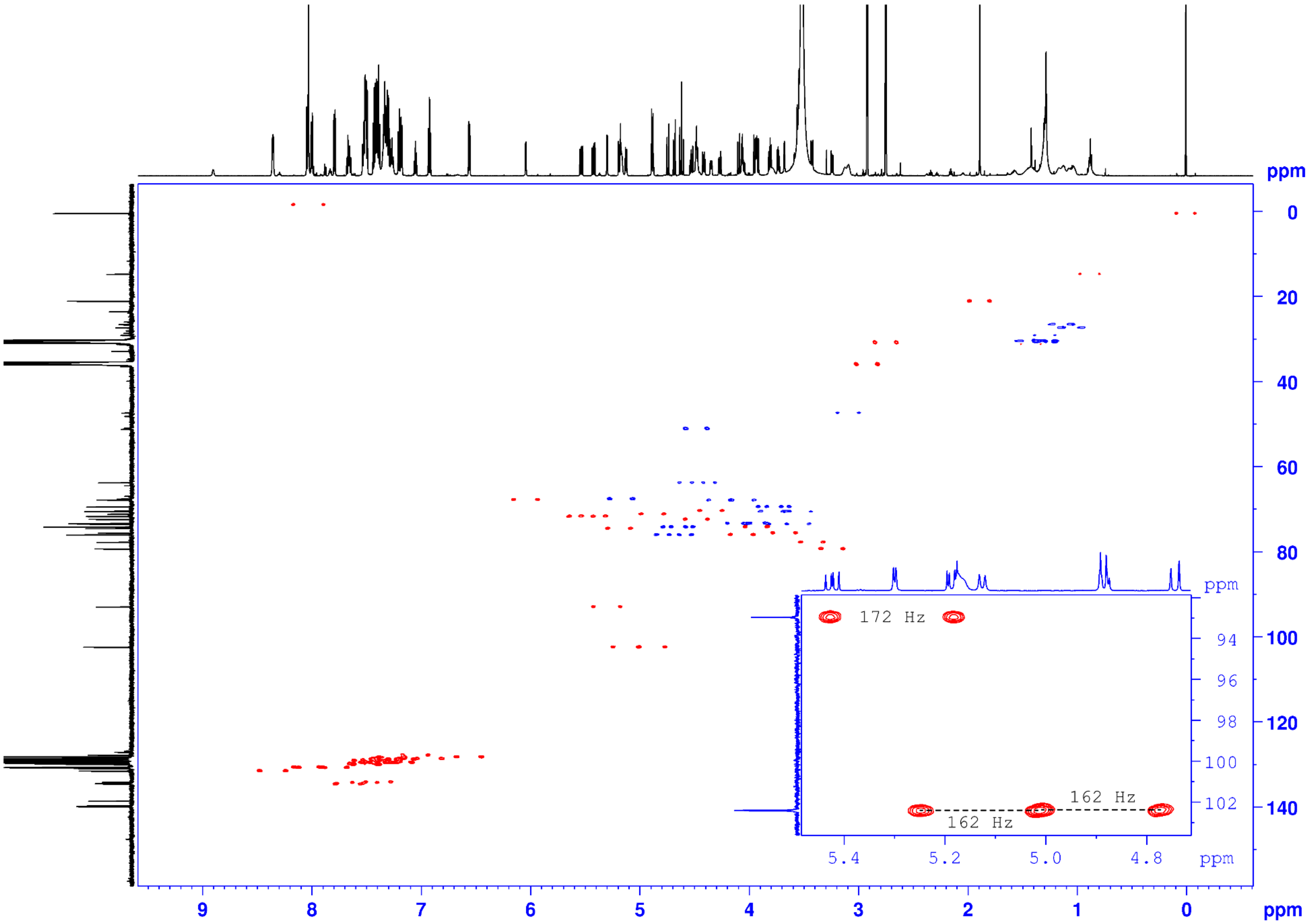

^{1}H-^{13}C HMBC

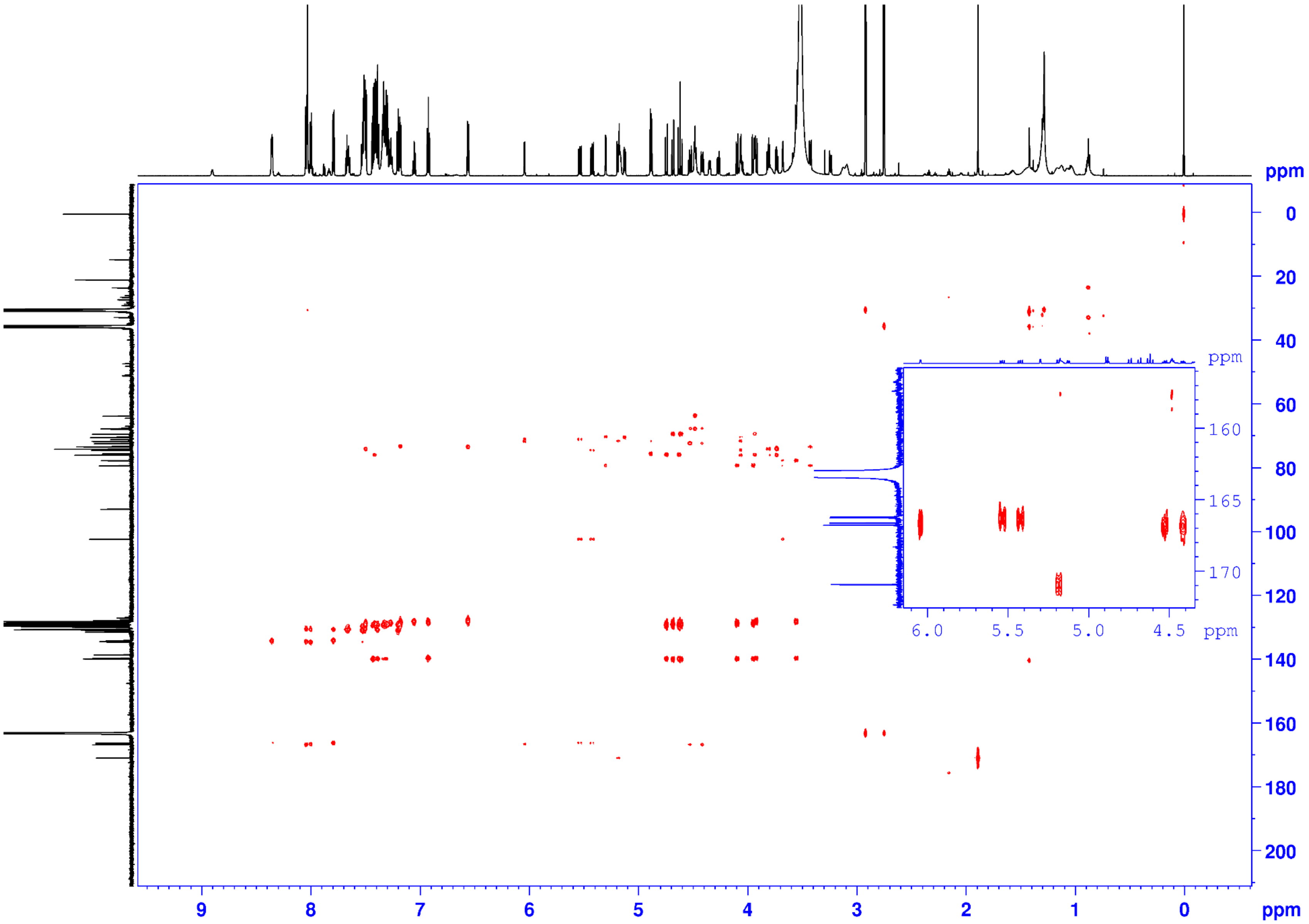

ppm
0
20
40
60
80
100
120
140
160
180
200
9
8
7
6
5
4
3
2
1
0
ppm
160
165
170
6.0
5.5
5.0
4.5

$^{13}C\{^{1}H\}$ NMR

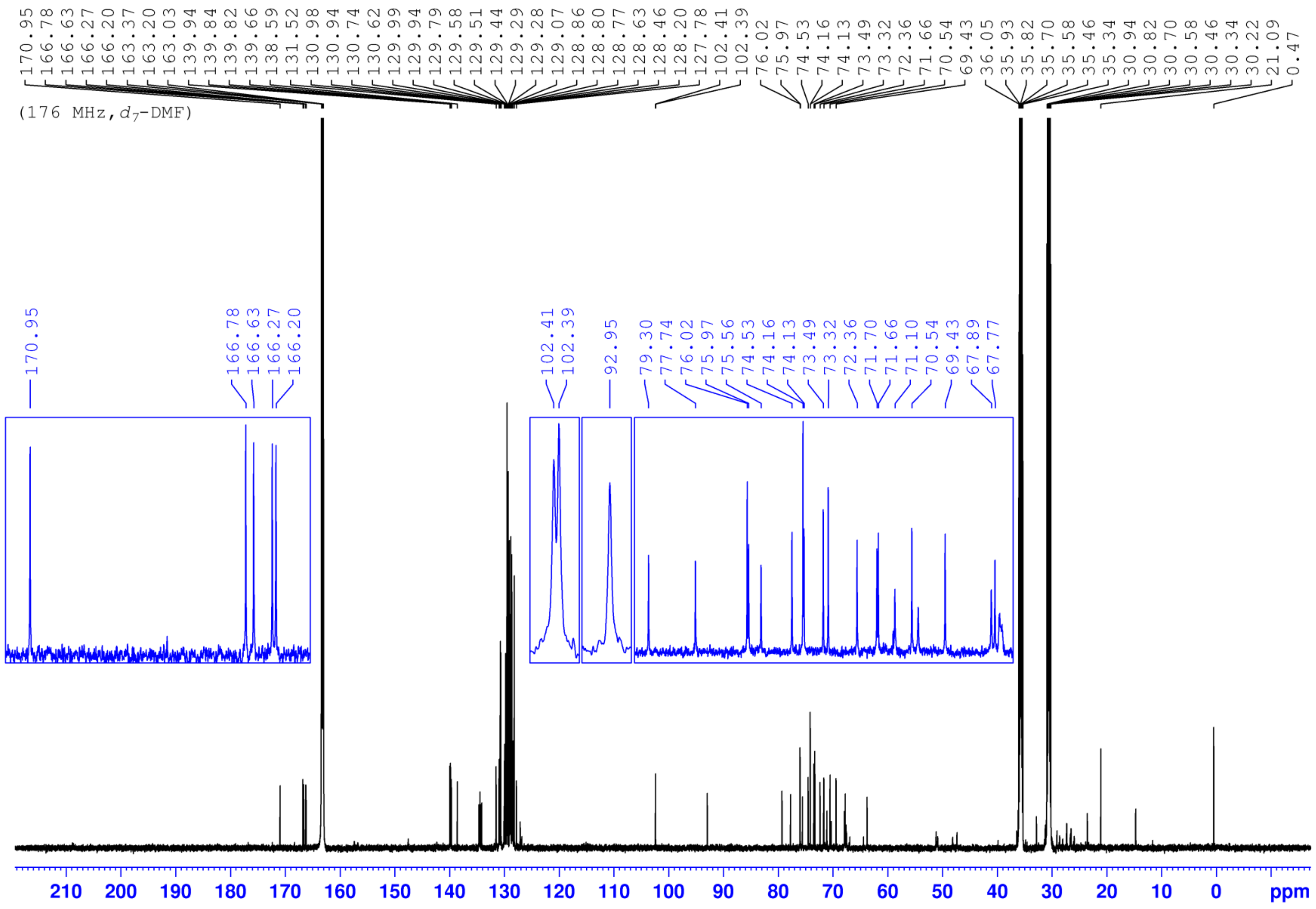

170.95
166.78
166.63
166.27
166.20
163.37
163.20
163.03
139.94
139.84
139.82
139.66
138.59
131.52
130.98
130.94
130.74
130.62
129.99
129.94
129.79
129.58
129.51
129.44
129.29
129.28
129.07
128.86
128.80
128.77
128.63
128.46
128.20
127.78
102.41
102.39
76.02
75.97
74.53
74.16
74.13
73.49
73.32
72.36
71.66
70.54
69.43
36.05
35.93
35.82
35.70
35.58
35.46
35.34
30.94
30.82
30.70
30.58
30.46
30.34
30.22
21.09
0.47
(176 MHz, d_7-DMF)
170.95
166.78
166.63
166.27
166.20
102.41
102.39
92.95
79.30
77.74
76.02
75.97
75.56
74.53
74.16
74.13
73.49
73.32
72.36
71.70
71.66
71.10
70.54
69.43
67.89
67.77
210
200
190
180
170
160
150
140
130
120
110
100
90
80
70
60
50
40
30
20
10
0
ppm

Compound **37**

^{1}H-NMR

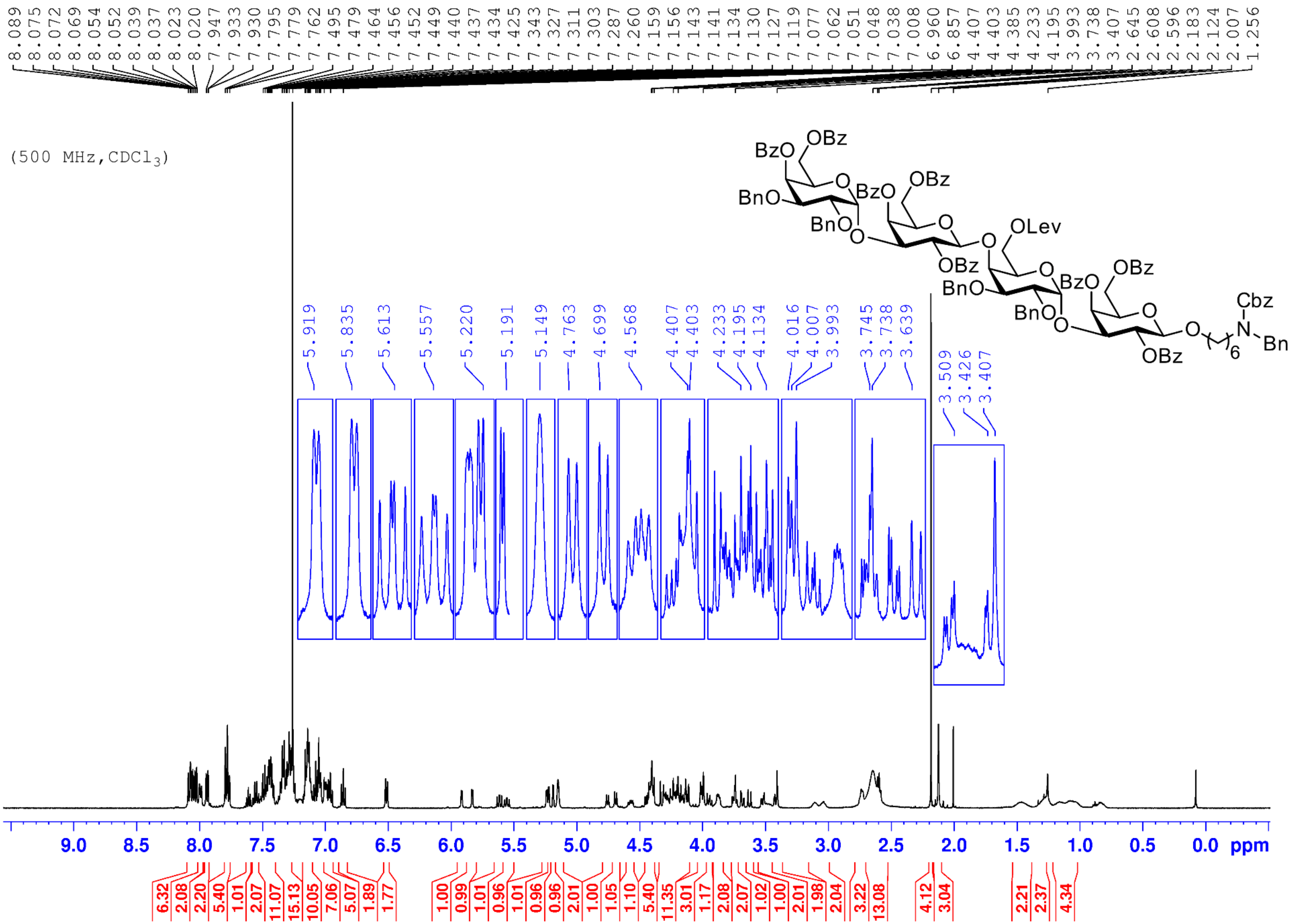

^{1}H-^{1}H COSY

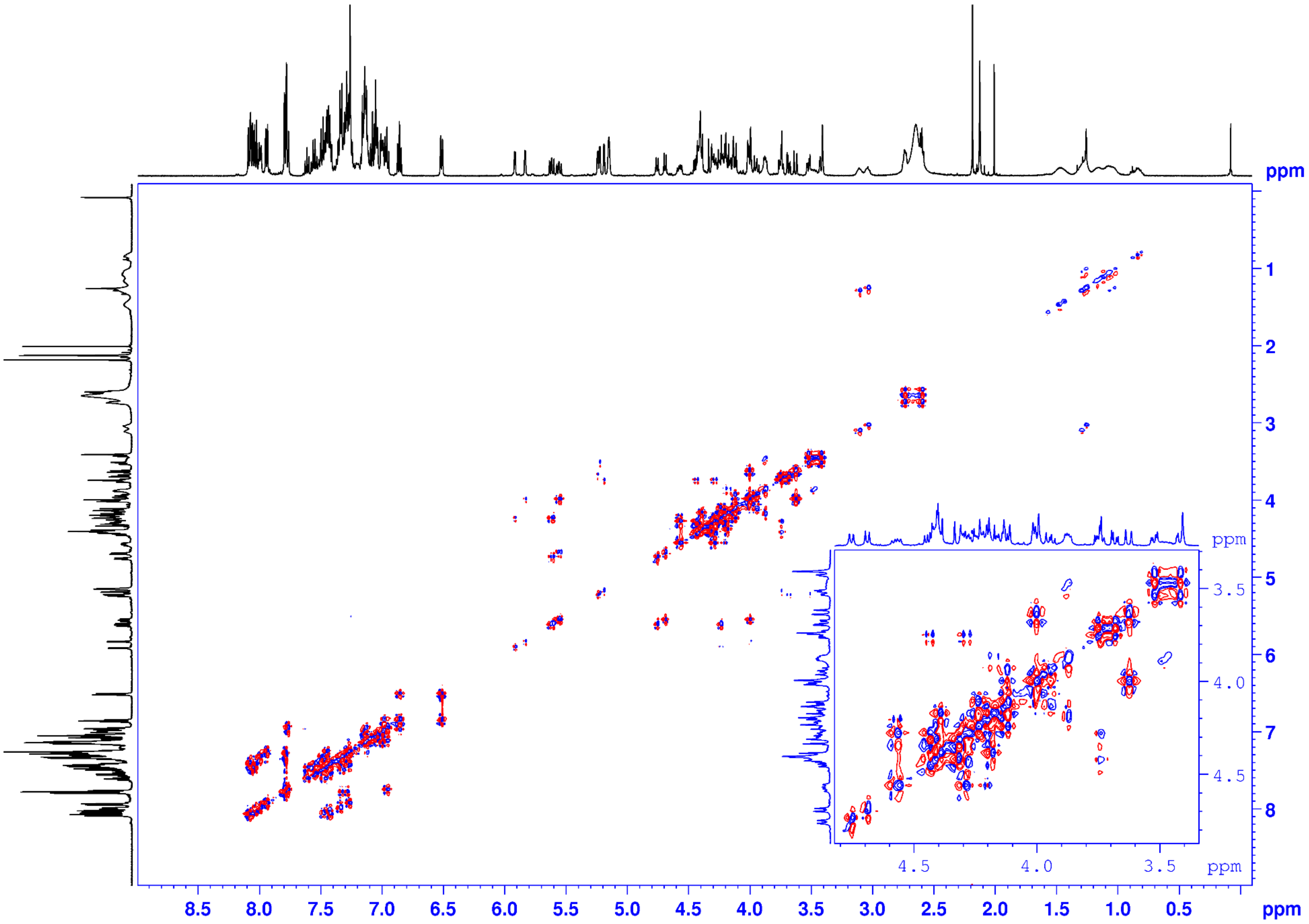
ppm
1
2
3
4
5
6
7
8
8.5
8.0
7.5
7.0
6.5
6.0
5.5
5.0
4.5
4.0
3.5
3.0
2.5
2.0
1.5
1.0
0.5
ppm
3.5
4.0
4.5
ppm
4.5
4.0
3.5
ppm

$^{1}H-^{13}C$ HSQC

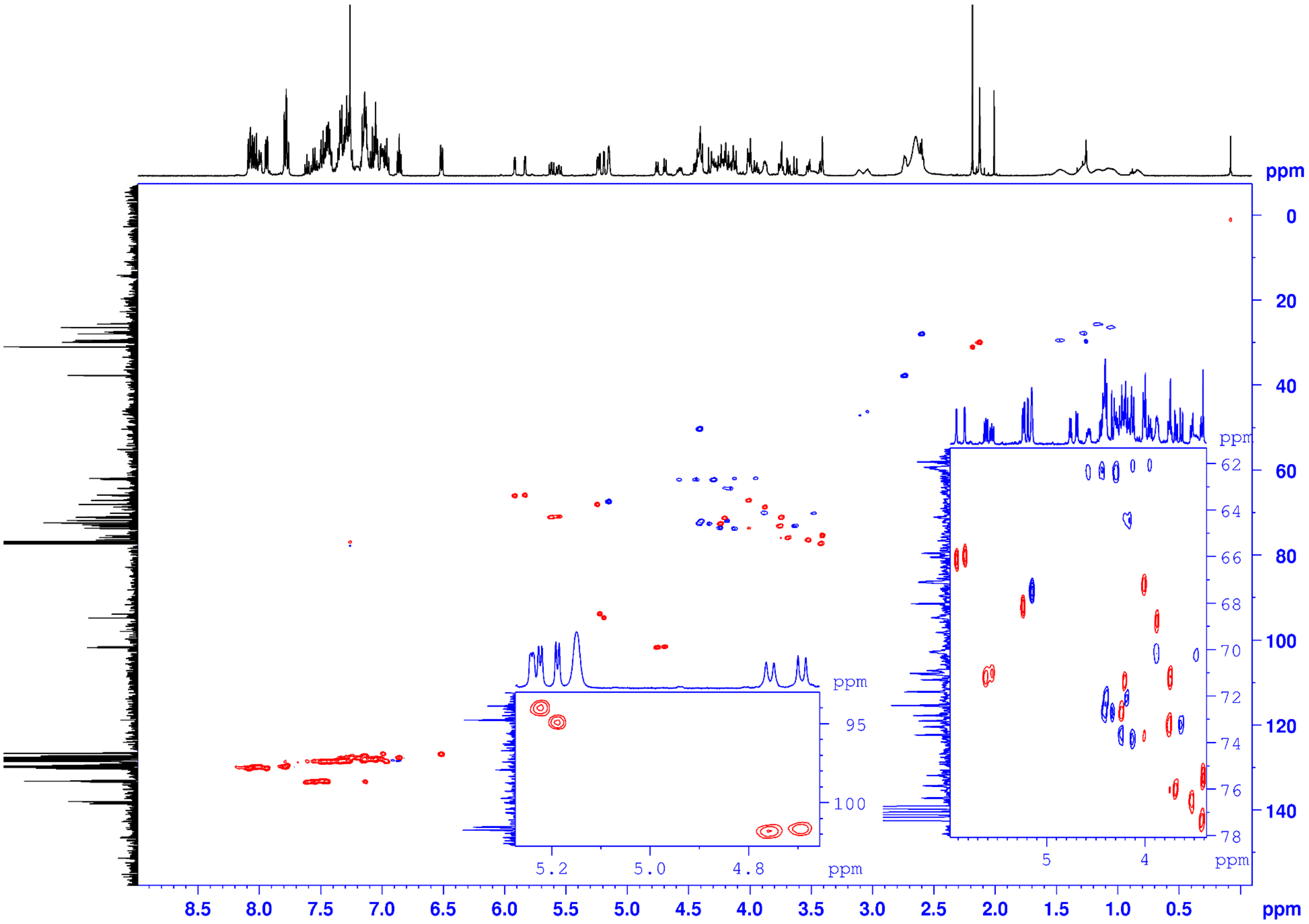

ppm
0
20
40
60
80
100
120
140
8.5
8.0
7.5
7.0
6.5
6.0
5.5
5.0
4.5
4.0
3.5
3.0
2.5
2.0
1.5
1.0
0.5
ppm
95
100
5.2
5.0
4.8
ppm
62
64
66
68
70
72
74
76
78
5
4
ppm

^{1}H-^{13}C non-decoupled HSQC

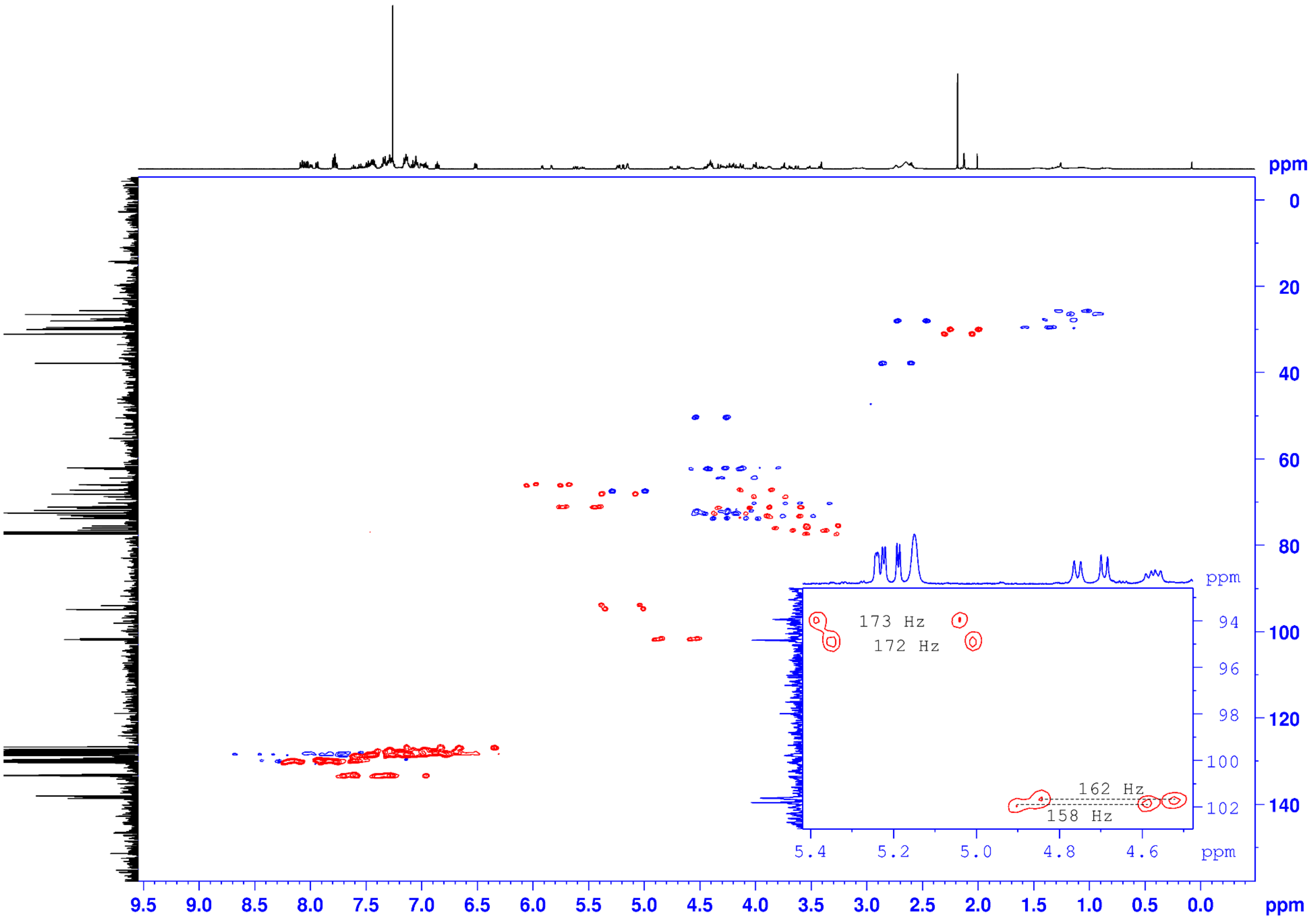

$^{13}C\{^1H\}$ NMR

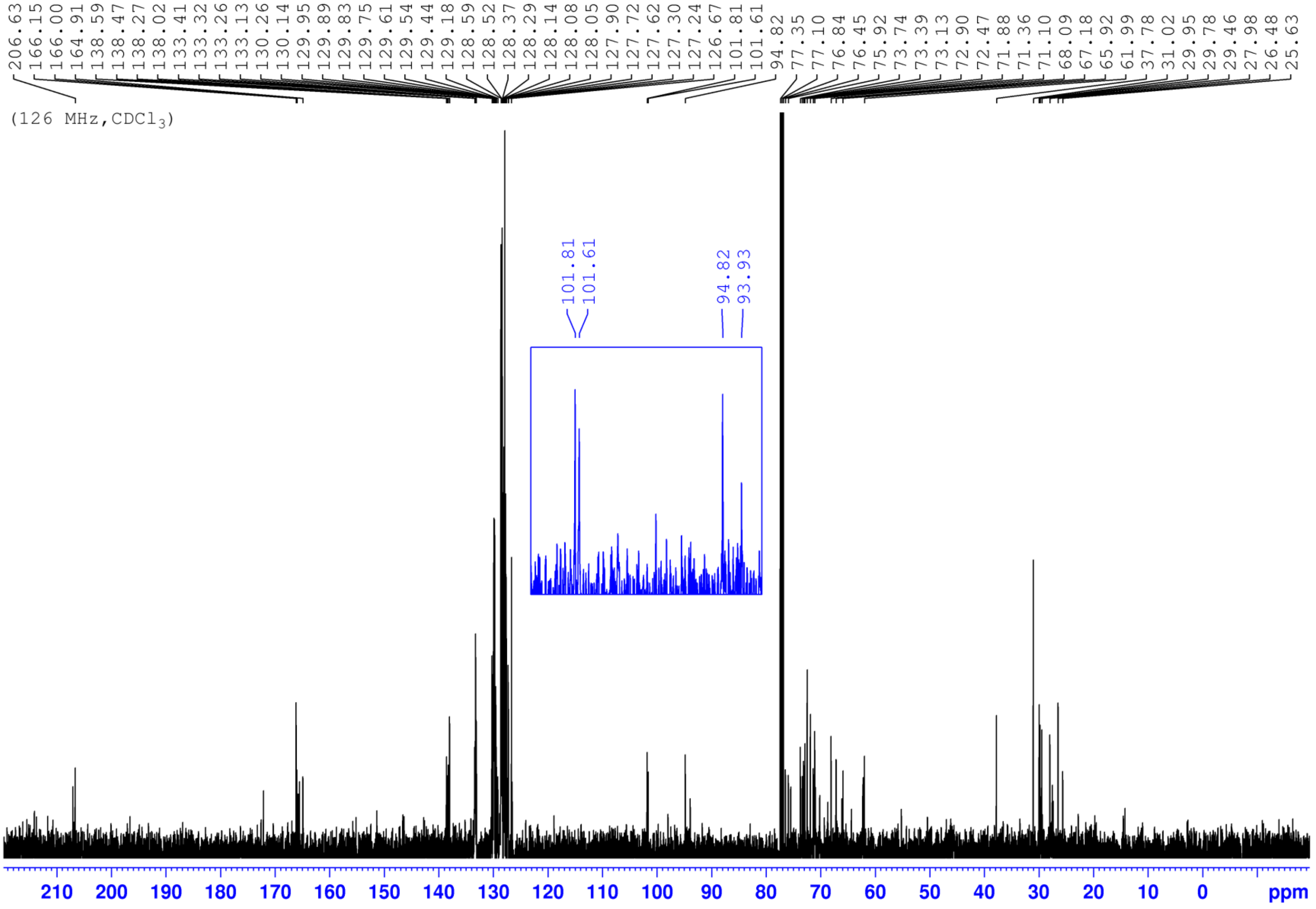
206.63
166.15
166.00
164.91
138.59
138.47
138.27
138.02
133.41
133.32
133.26
133.13
130.26
130.14
129.95
129.89
129.83
129.75
129.61
129.54
129.44
129.18
128.59
128.52
128.37
128.29
128.14
128.08
128.05
127.90
127.72
127.62
127.30
127.24
126.67
101.81
101.61
94.82
77.35
77.10
76.84
76.45
75.92
73.74
73.39
73.13
72.90
72.47
71.88
71.36
71.10
68.09
67.18
65.92
61.99
37.78
31.02
29.95
29.78
29.46
27.98
26.48
25.63
(126 MHz, CDCl3)
101.81
101.61
94.82
93.93
210
200
190
180
170
160
150
140
130
120
110
100
90
80
70
60
50
40
30
20
10
0
ppm

Compound **38**

^{1}H-NMR

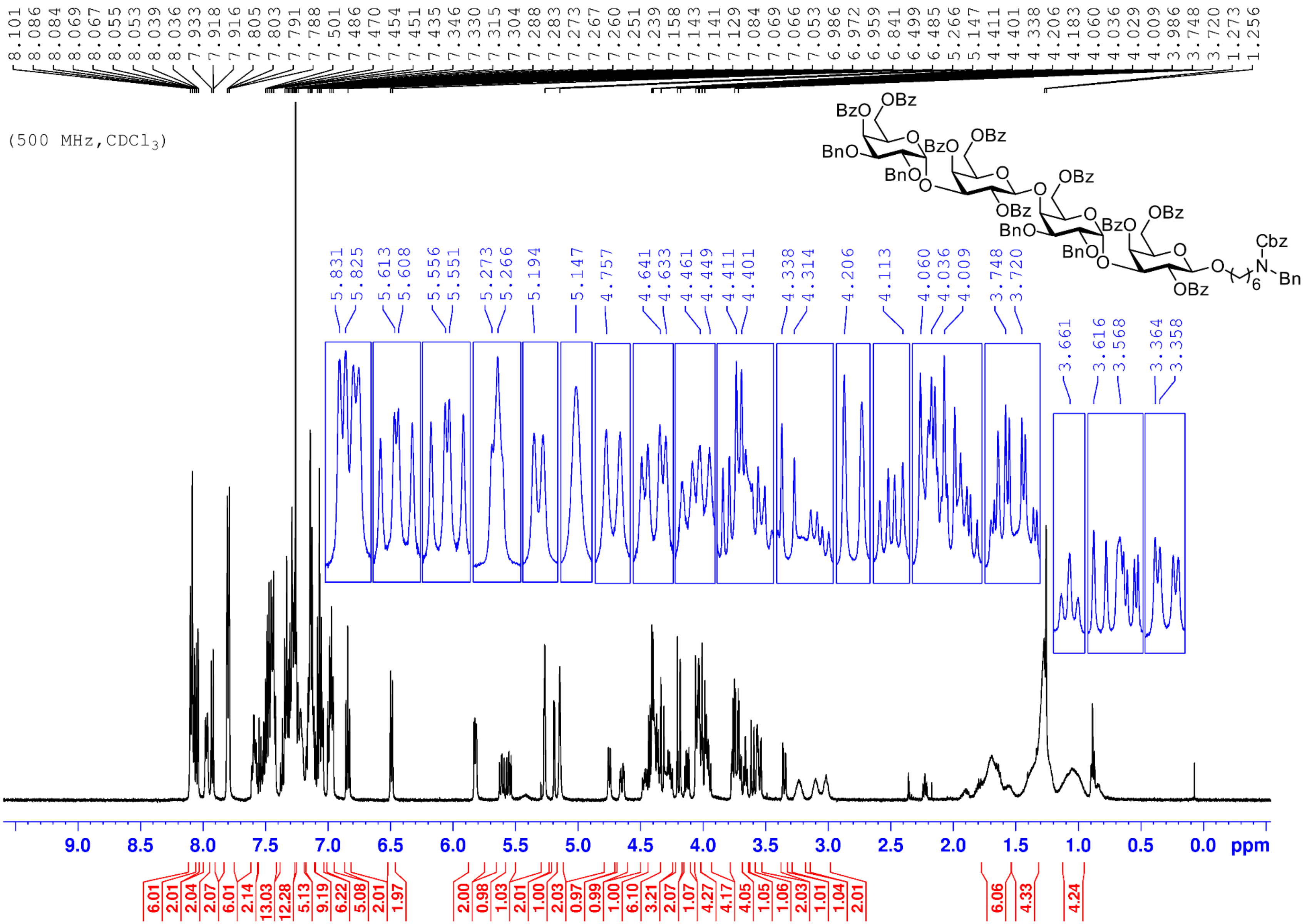

^{1}H-^{1}H COSY

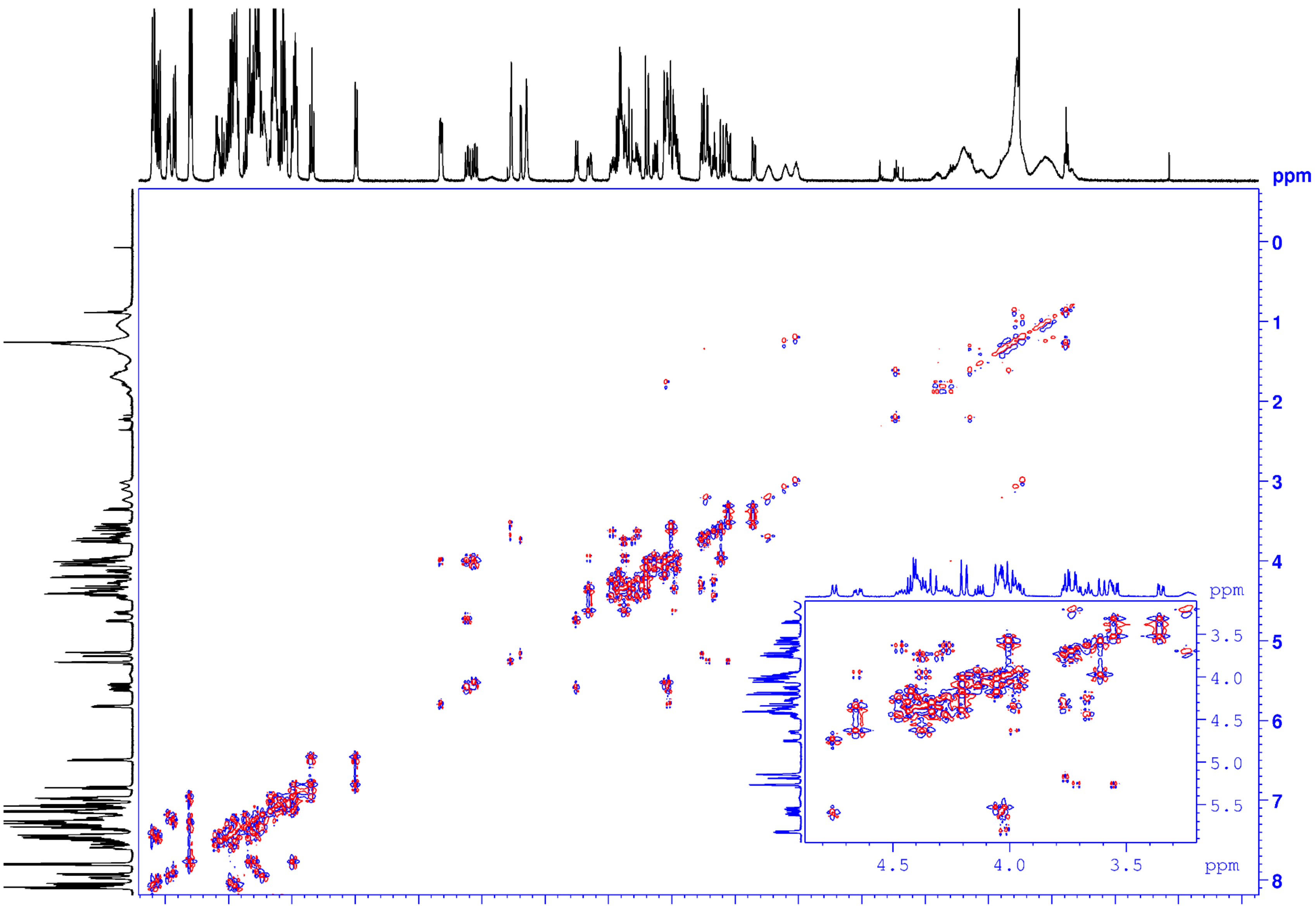
ppm
0
1
2
3
4
5
6
7
8
8.0
7.5
7.0
6.5
6.0
5.5
5.0
4.5
4.0
3.5
3.0
2.5
2.0
1.5
1.0
0.5
0.0
3.5
4.0
4.5
5.0
5.5

^{1}H-^{13}C HSQC

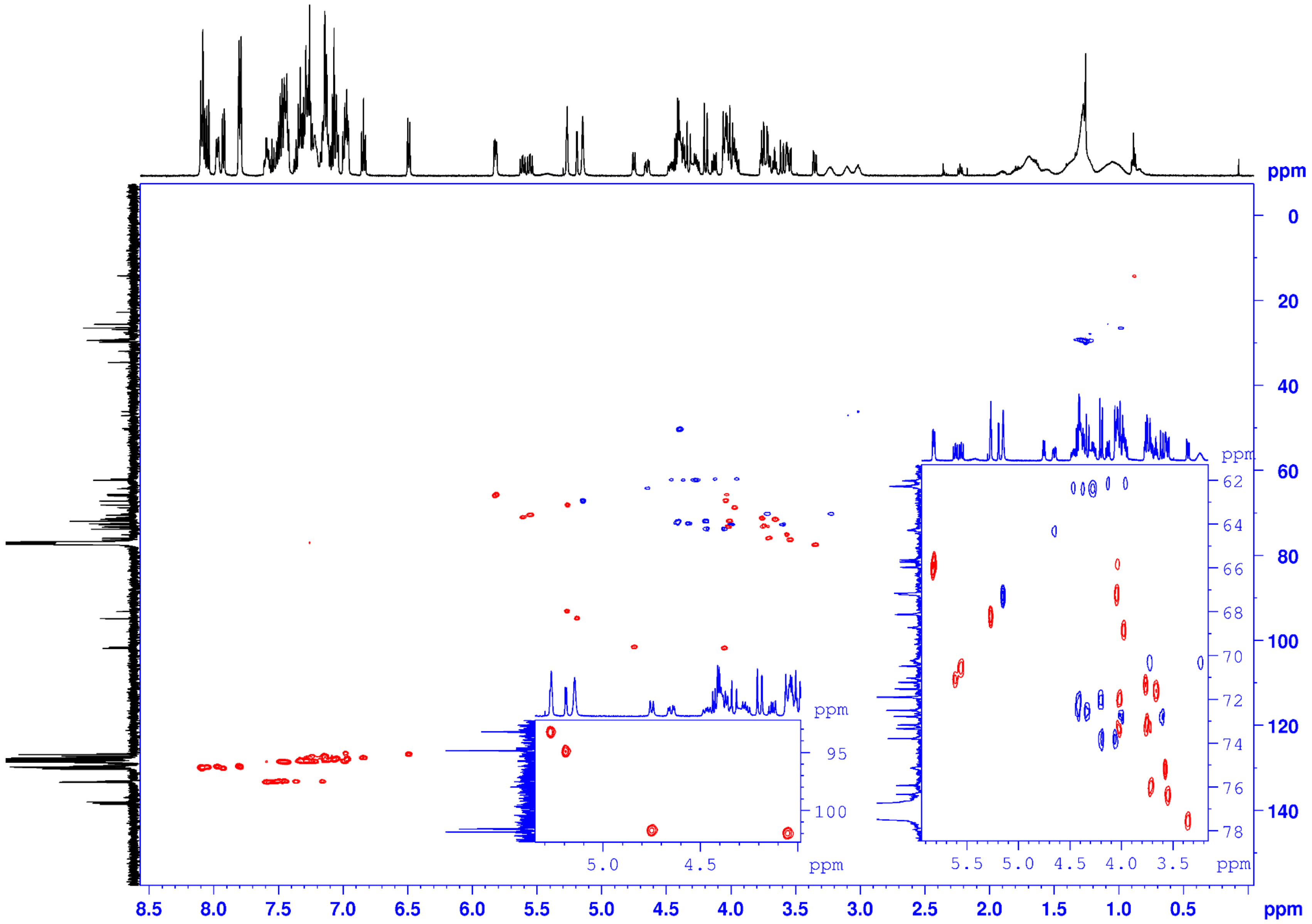

ppm
0
20
40
60
80
100
120
140
8.5
8.0
7.5
7.0
6.5
6.0
5.5
5.0
4.5
4.0
3.5
3.0
2.5
2.0
1.5
1.0
0.5
ppm
95
100
5.0
4.5
ppm
62
64
66
68
70
72
74
76
78
5.5
5.0
4.5
4.0
3.5
ppm

$^{1}H-^{13}C$ non-decoupled HSQC

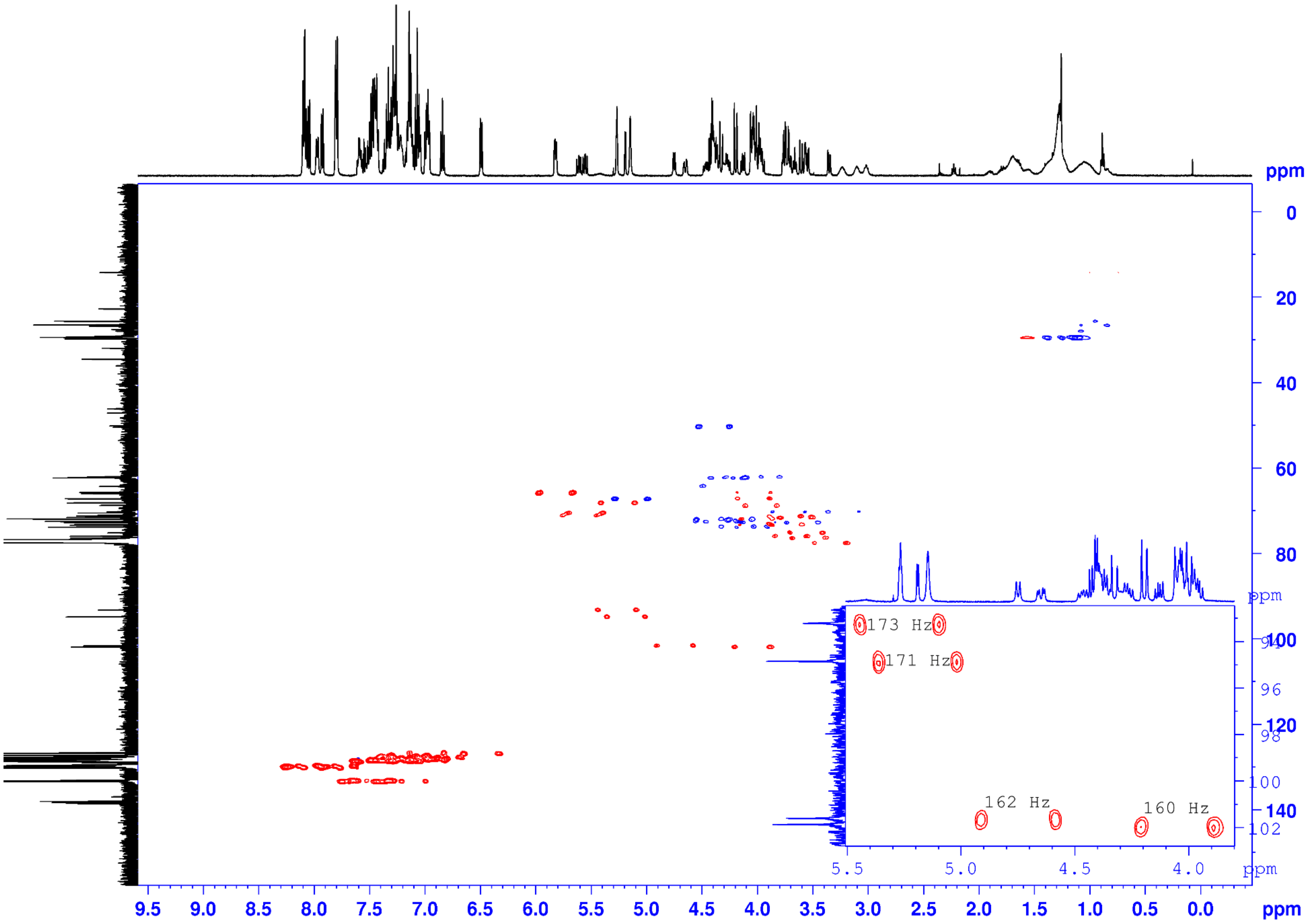

$^{13}C\{^1H\}$ NMR

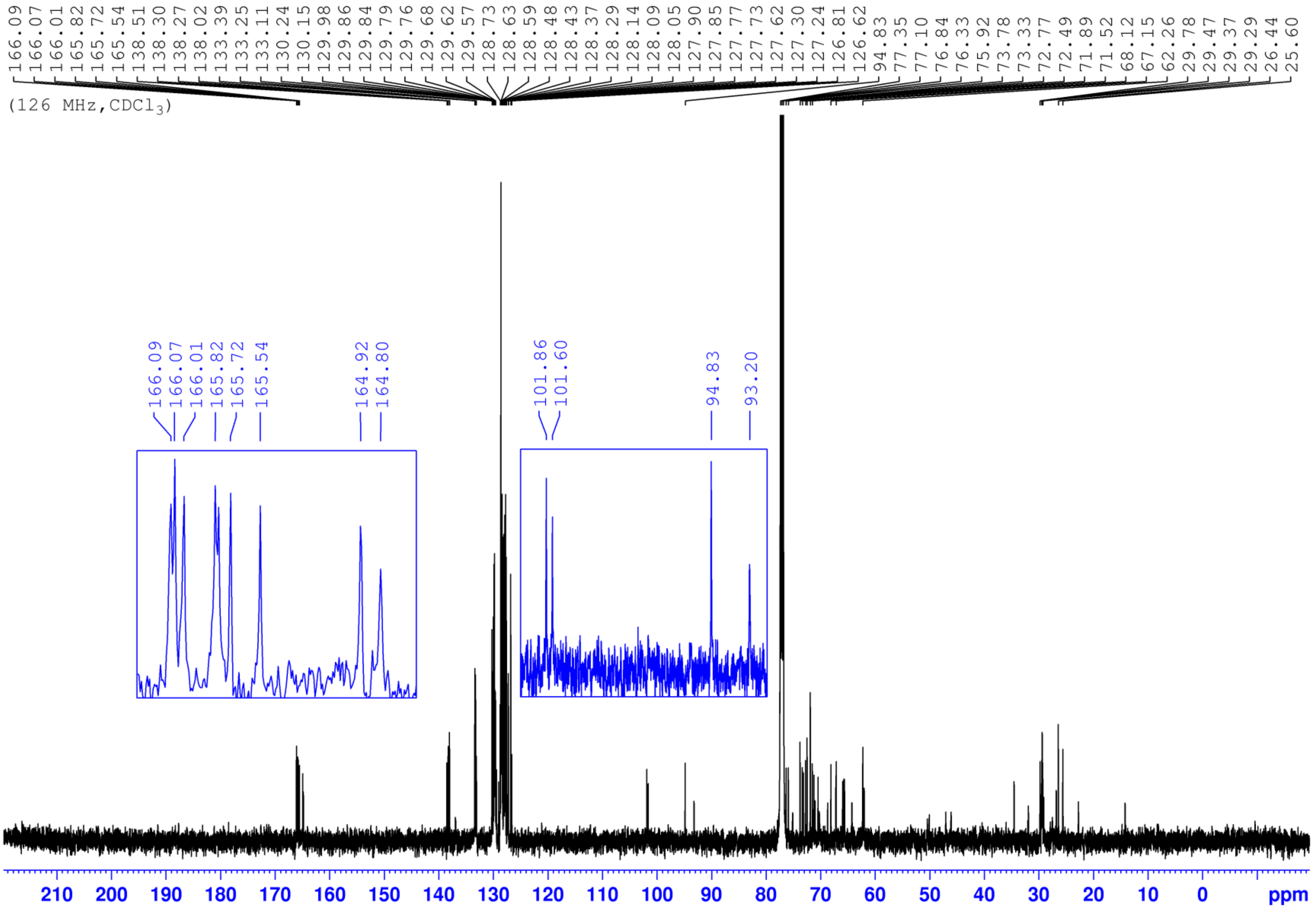
(126 MHz, CDCl3)
166.09
166.07
166.01
165.82
165.72
165.54
138.51
138.30
138.27
138.02
133.39
133.25
133.11
130.24
130.15
129.98
129.86
129.84
129.79
129.76
129.68
129.62
129.57
128.73
128.63
128.59
128.48
128.43
128.37
128.29
128.14
128.09
128.05
127.90
127.85
127.77
127.73
127.62
127.30
127.24
126.81
126.62
94.83
77.35
77.10
76.84
76.33
75.92
73.78
73.33
72.77
72.49
71.89
71.52
68.12
67.15
62.26
29.78
29.47
29.37
29.29
26.44
25.60
164.92
164.80
101.86
101.60
93.20
ppm

Compound **39**

^{1}H-NMR

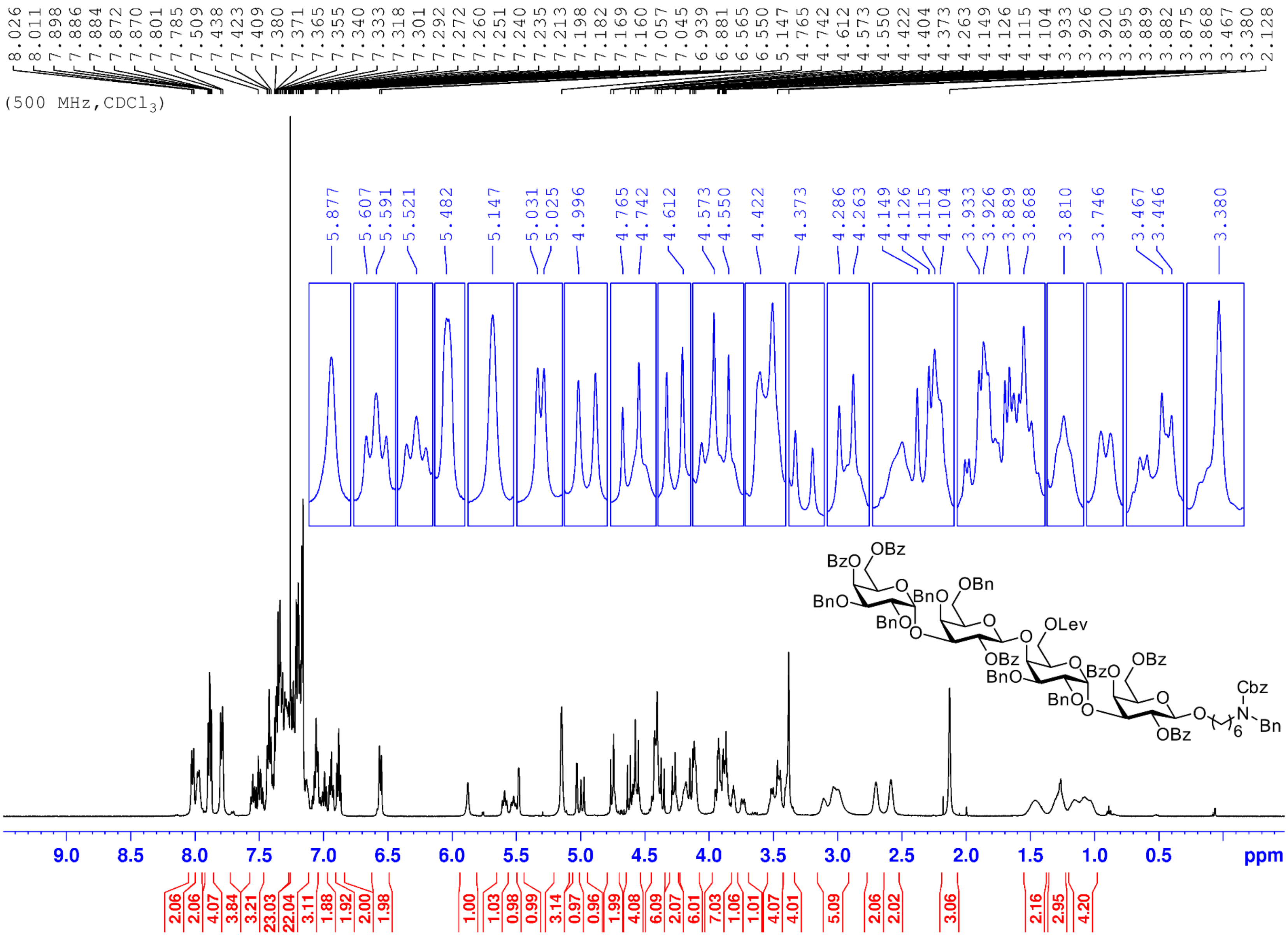

^1H-^1H COSY

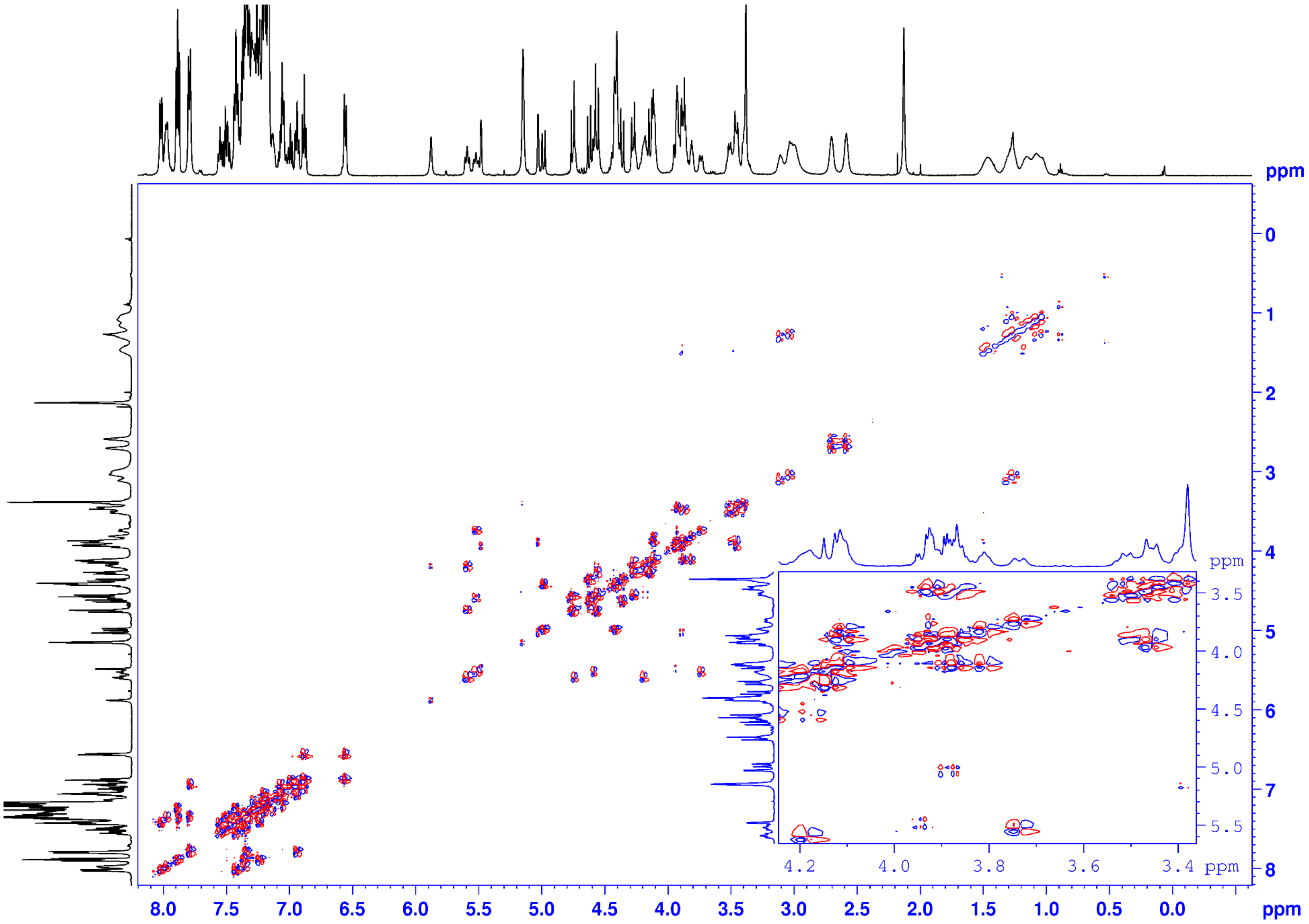

ppm
0
1
2
3
4
5
6
7
8
8.0
7.5
7.0
6.5
6.0
5.5
5.0
4.5
4.0
3.5
3.0
2.5
2.0
1.5
1.0
0.5
0.0
ppm
3.5
4.0
4.5
5.0
5.5
4.2
4.0
3.8
3.6
3.4 ppm

^{1}H-^{13}C HSQC

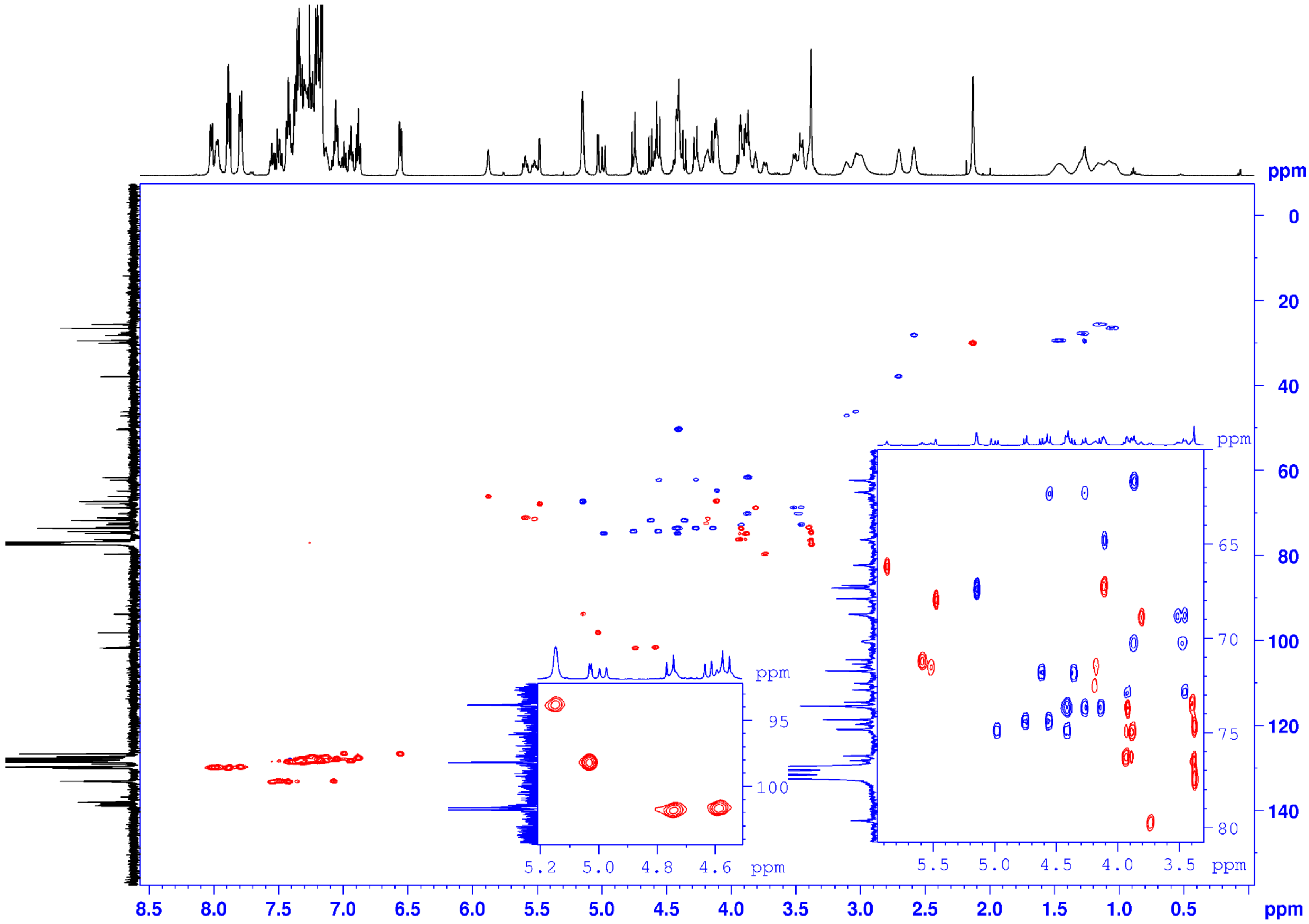
ppm
0
20
40
60
80
100
120
140
8.5
8.0
7.5
7.0
6.5
6.0
5.5
5.0
4.5
4.0
3.5
3.0
2.5
2.0
1.5
1.0
0.5
ppm
95
100
5.2
5.0
4.8
4.6
ppm
65
70
75
80
5.5
5.0
4.5
4.0
3.5
ppm

^{1}H-^{13}C non-decoupled HSQC

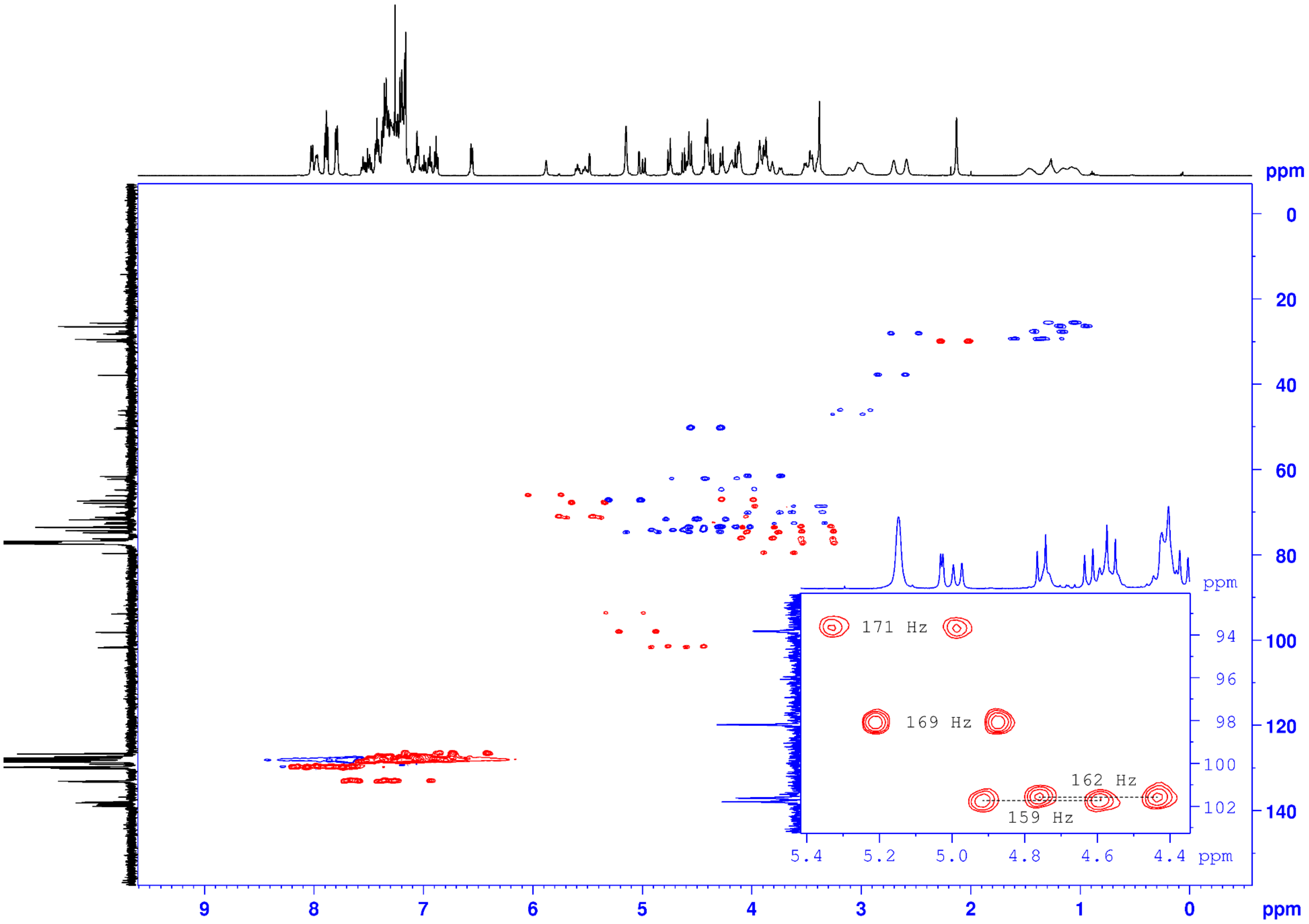

^{1}H-^{13}C HMBC

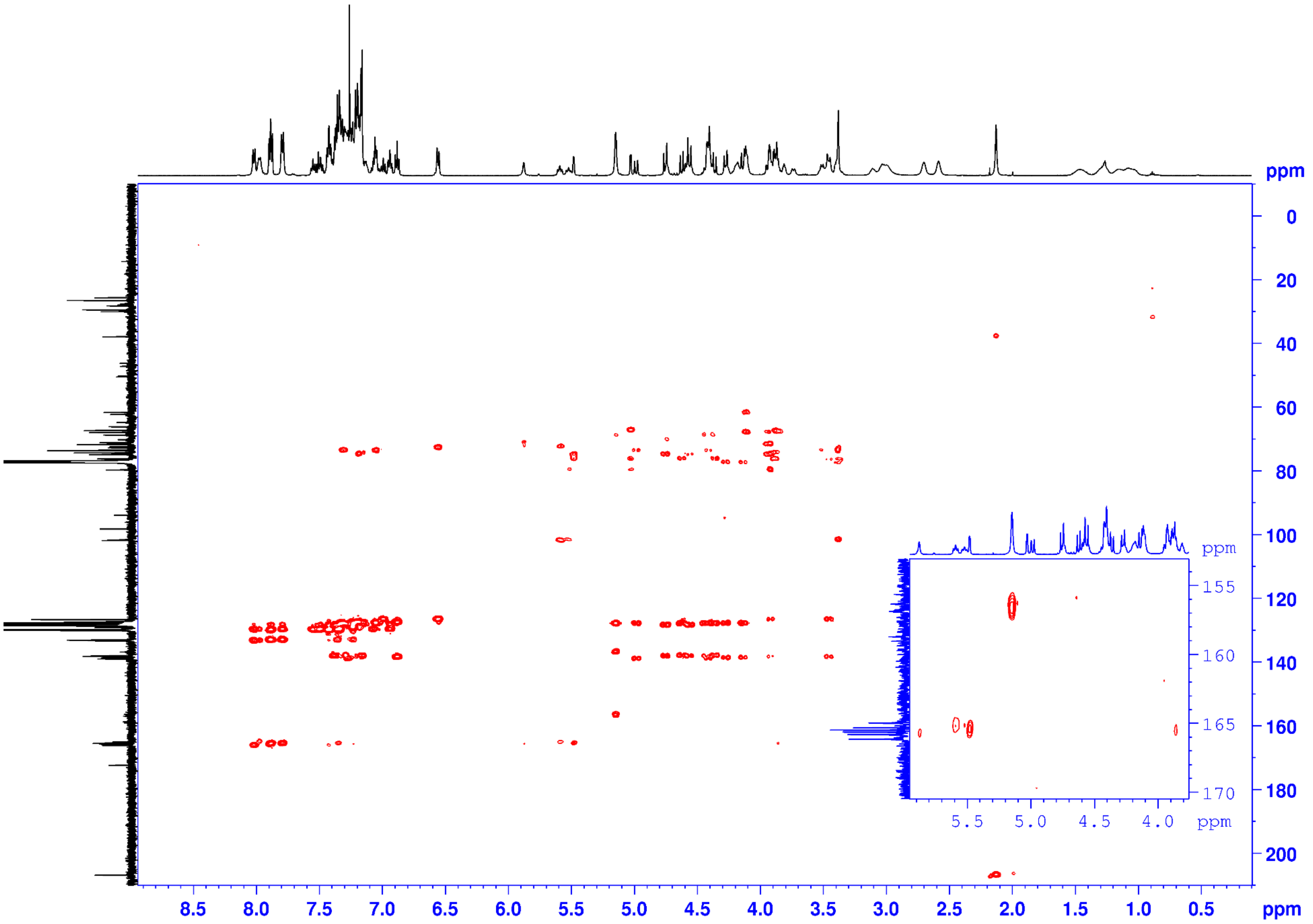
ppm
0
20
40
60
80
100
120
140
160
180
200
8.5
8.0
7.5
7.0
6.5
6.0
5.5
5.0
4.5
4.0
3.5
3.0
2.5
2.0
1.5
1.0
0.5
ppm
155
160
165
170
5.5
5.0
4.5
4.0
ppm

$^{13}C\{^1H\}$ NMR

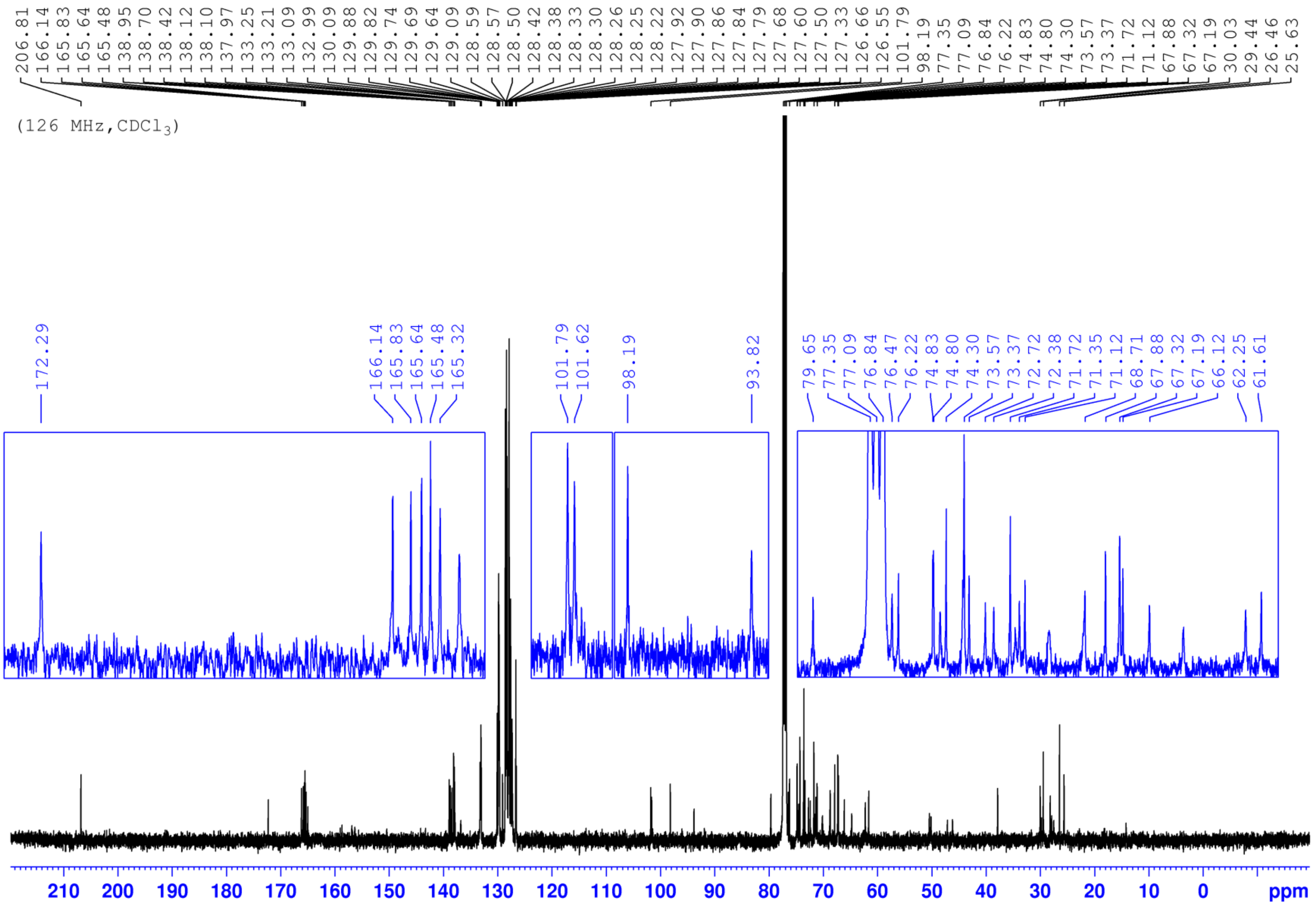

206.81
166.14
165.83
165.64
165.48
138.95
138.70
138.42
138.12
138.10
137.97
133.25
133.21
133.09
132.99
130.09
129.88
129.82
129.74
129.69
129.64
129.09
128.59
128.57
128.50
128.42
128.38
128.33
128.30
128.26
128.25
128.22
127.92
127.90
127.86
127.84
127.79
127.68
127.60
127.50
127.33
126.66
126.55
101.79
98.19
77.35
77.09
76.84
76.22
74.83
74.80
74.30
73.57
73.37
71.72
71.12
67.88
67.32
67.19
30.03
29.44
26.46
25.63
(126 MHz, CDCl3)
172.29
166.14
165.83
165.64
165.48
165.32
101.79
101.62
98.19
93.82
79.65
77.35
77.09
76.84
76.47
76.22
74.83
74.80
74.30
73.57
73.37
72.72
72.38
71.72
71.35
71.12
68.71
67.88
67.32
67.19
66.12
62.25
61.61
210
200
190
180
170
160
150
140
130
120
110
100
90
80
70
60
50
40
30
20
10
0
ppm

Compound **40**

^{1}H-NMR

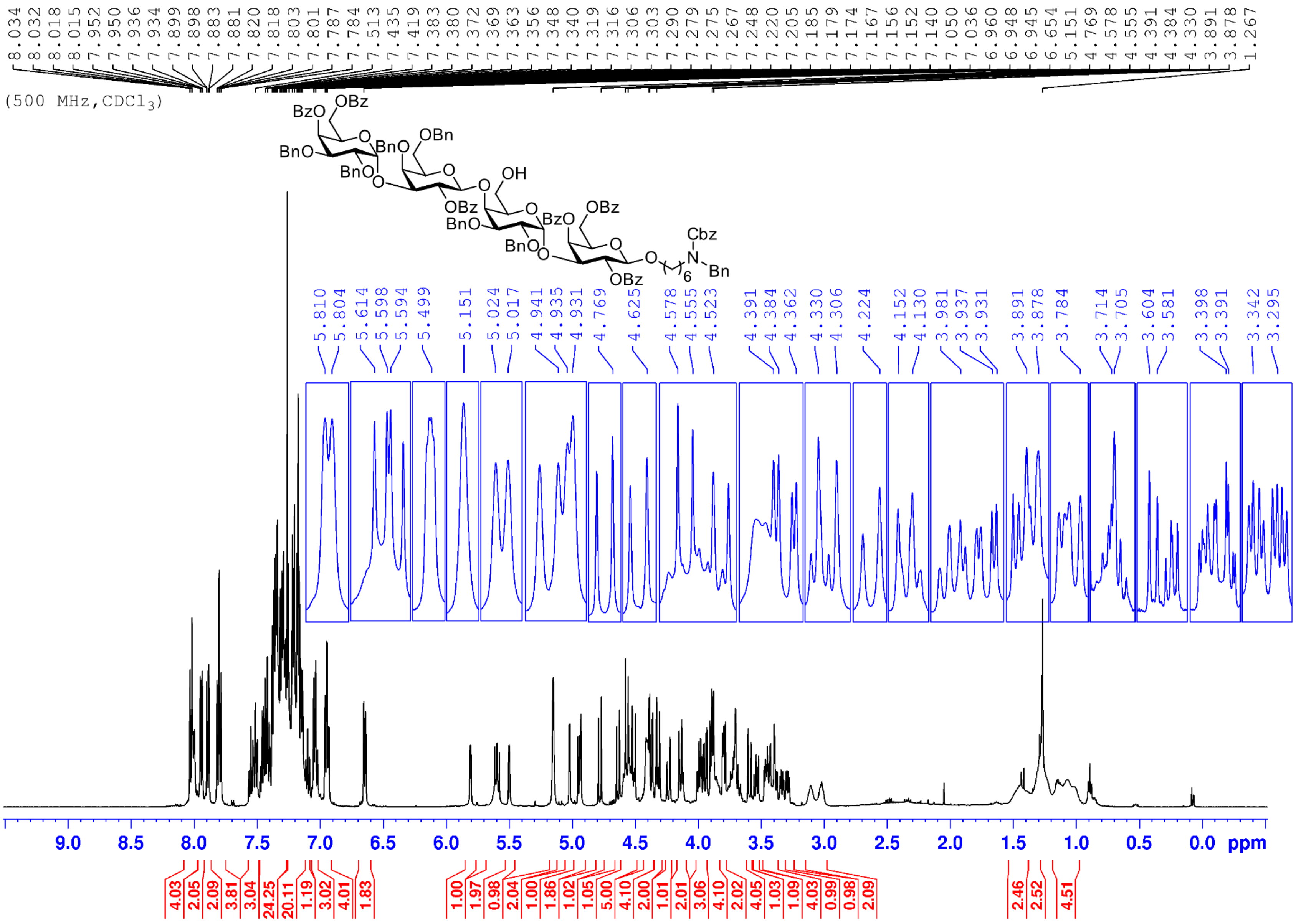

^{1}H-^{1}H COSY

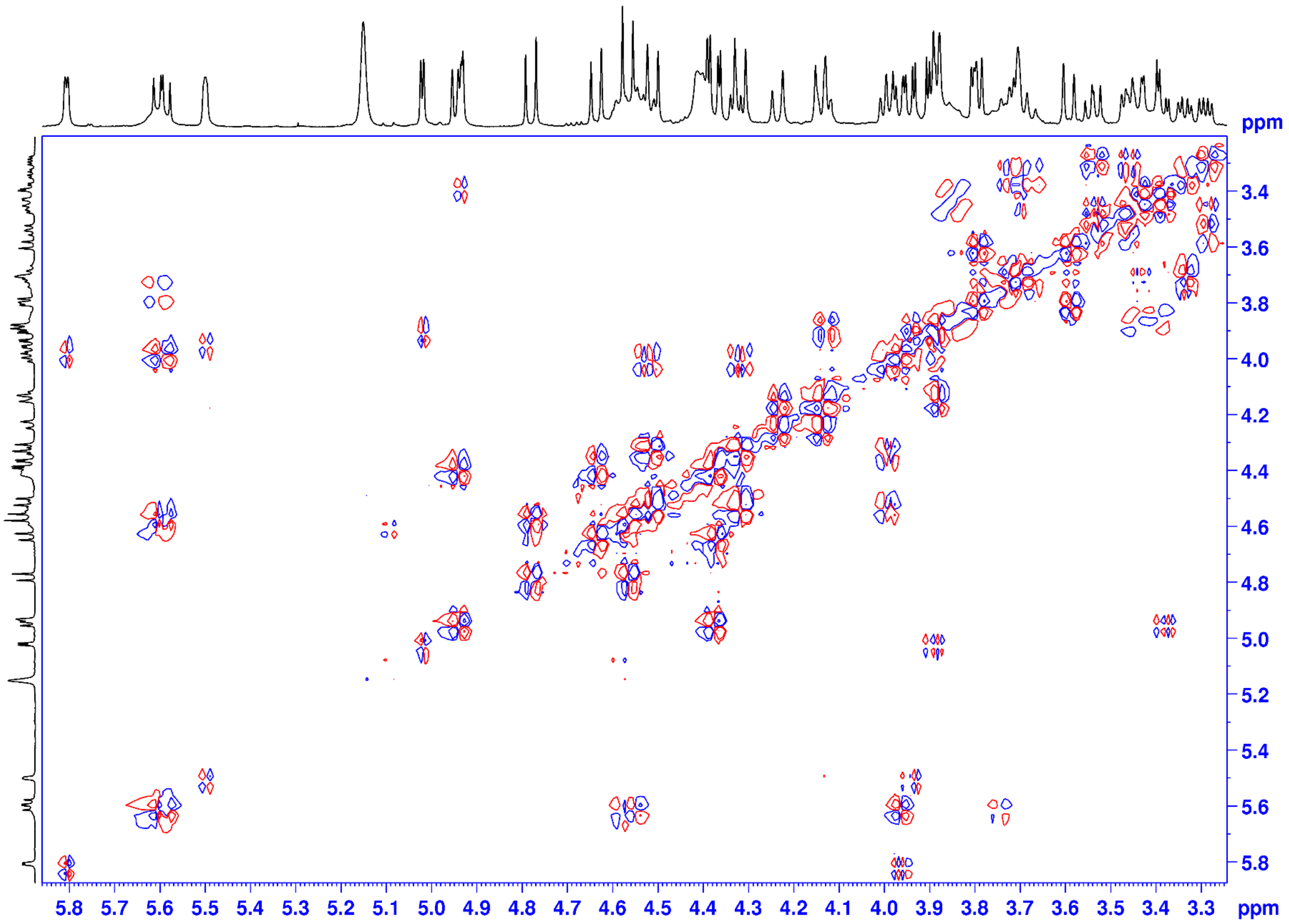

ppm
3.4
3.6
3.8
4.0
4.2
4.4
4.6
4.8
5.0
5.2
5.4
5.6
5.8
5.8 5.7 5.6 5.5 5.4 5.3 5.2 5.1 5.0 4.9 4.8 4.7 4.6 4.5 4.4 4.3 4.2 4.1 4.0 3.9 3.8 3.7 3.6 3.5 3.4 3.3
ppm

^{1}H-^{13}C HSQC

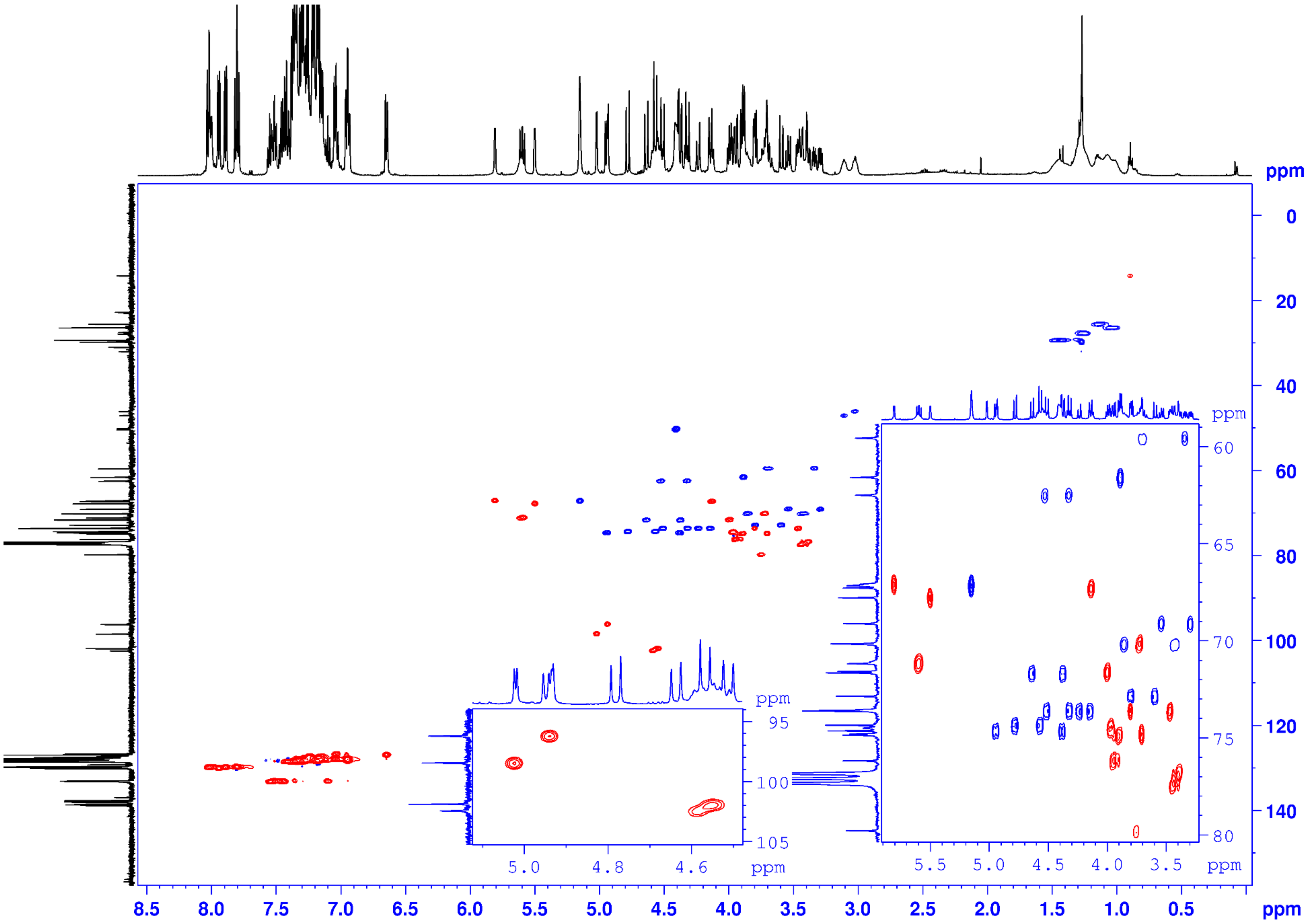

ppm
0
20
40
60
80
100
120
140
8.5
8.0
7.5
7.0
6.5
6.0
5.5
5.0
4.5
4.0
3.5
3.0
2.5
2.0
1.5
1.0
0.5
ppm

^{1}H-^{13}C non-decoupled HSQC

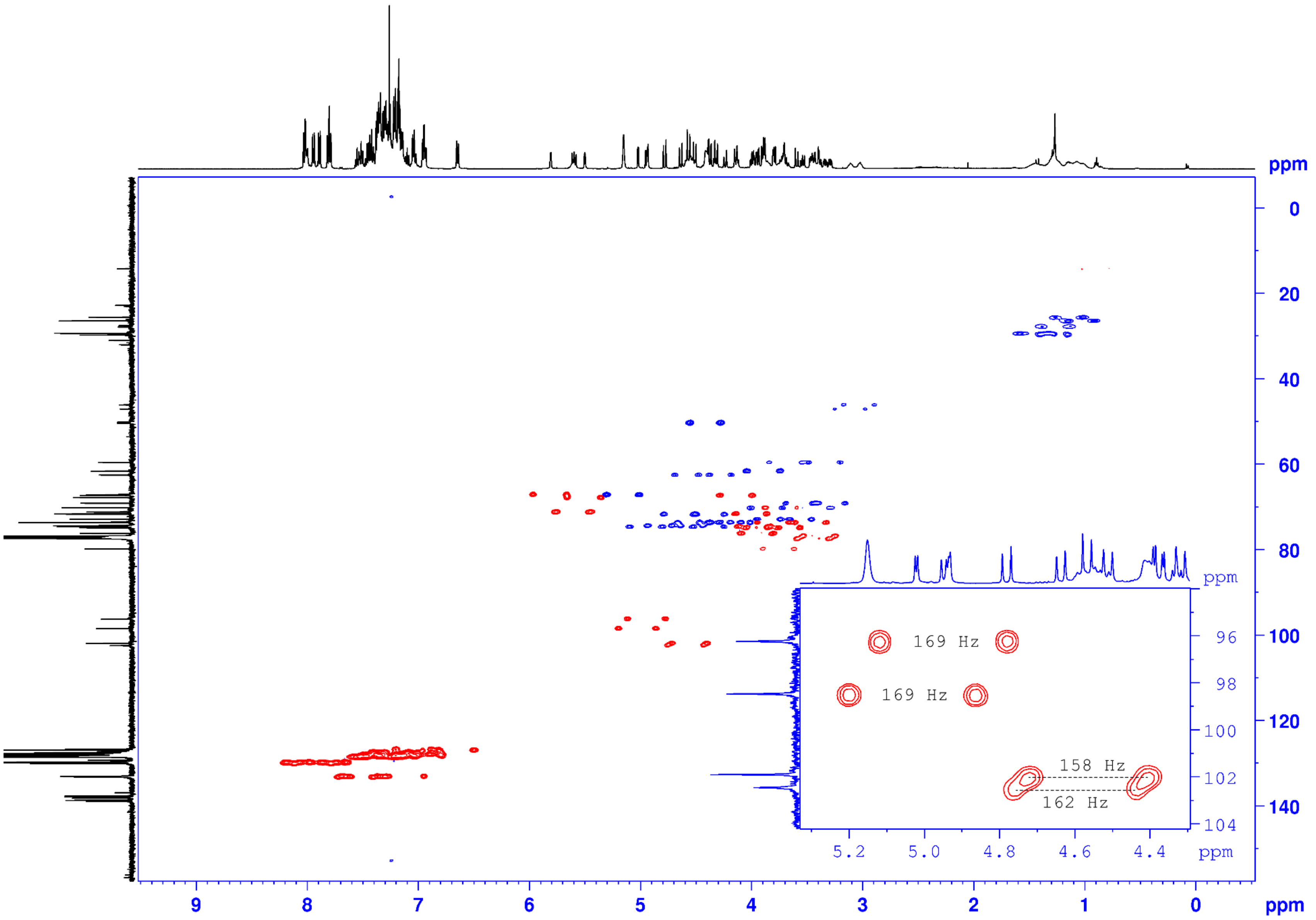

^1H-^{13}C HMBC

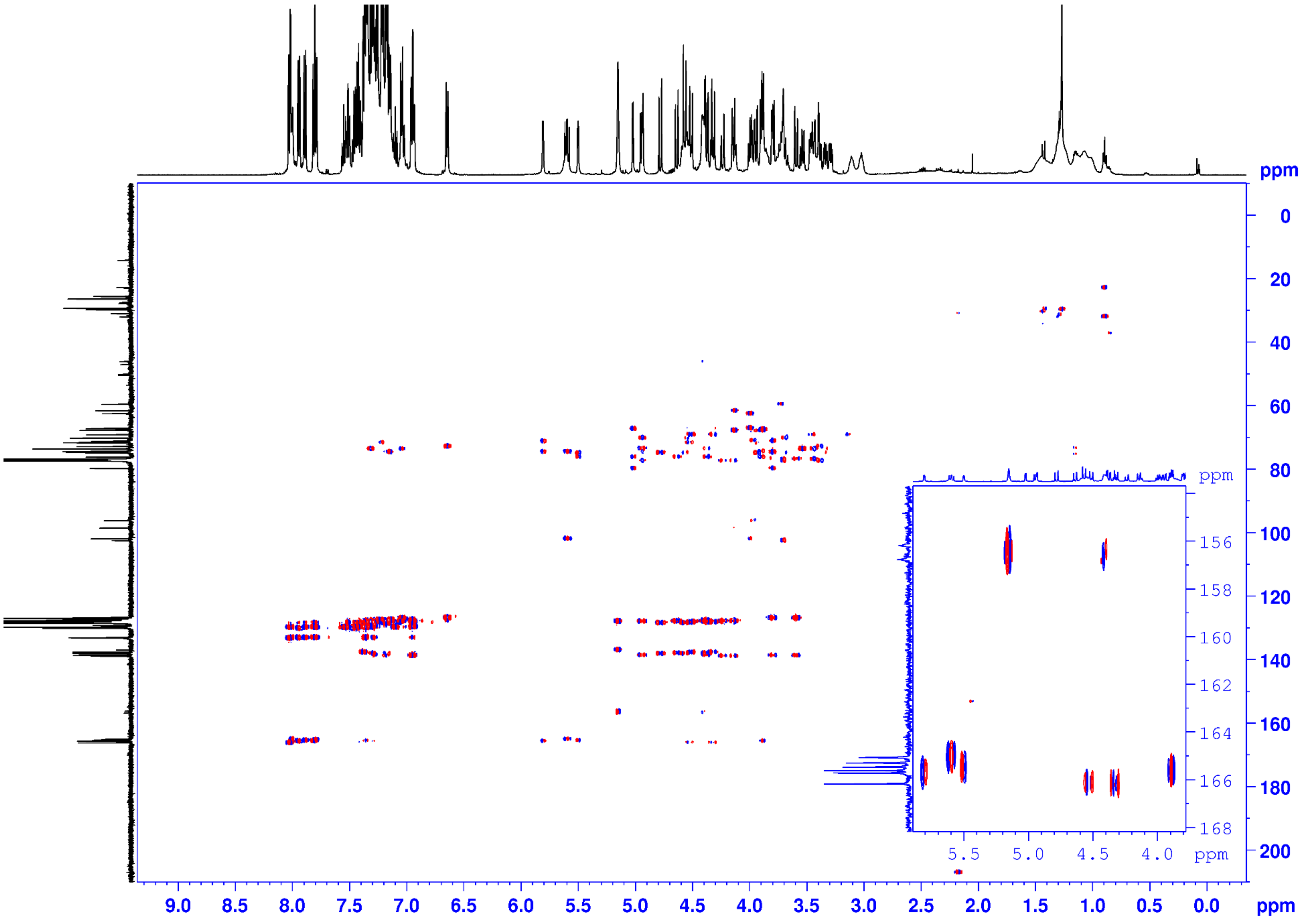
ppm
0
20
40
60
80
100
120
140
160
180
200
9.0
8.5
8.0
7.5
7.0
6.5
6.0
5.5
5.0
4.5
4.0
3.5
3.0
2.5
2.0
1.5
1.0
0.5
0.0
ppm
156
158
160
162
164
166
168
5.5
5.0
4.5
4.0
ppm

$^{13}C\{^{1}H\}$ NMR

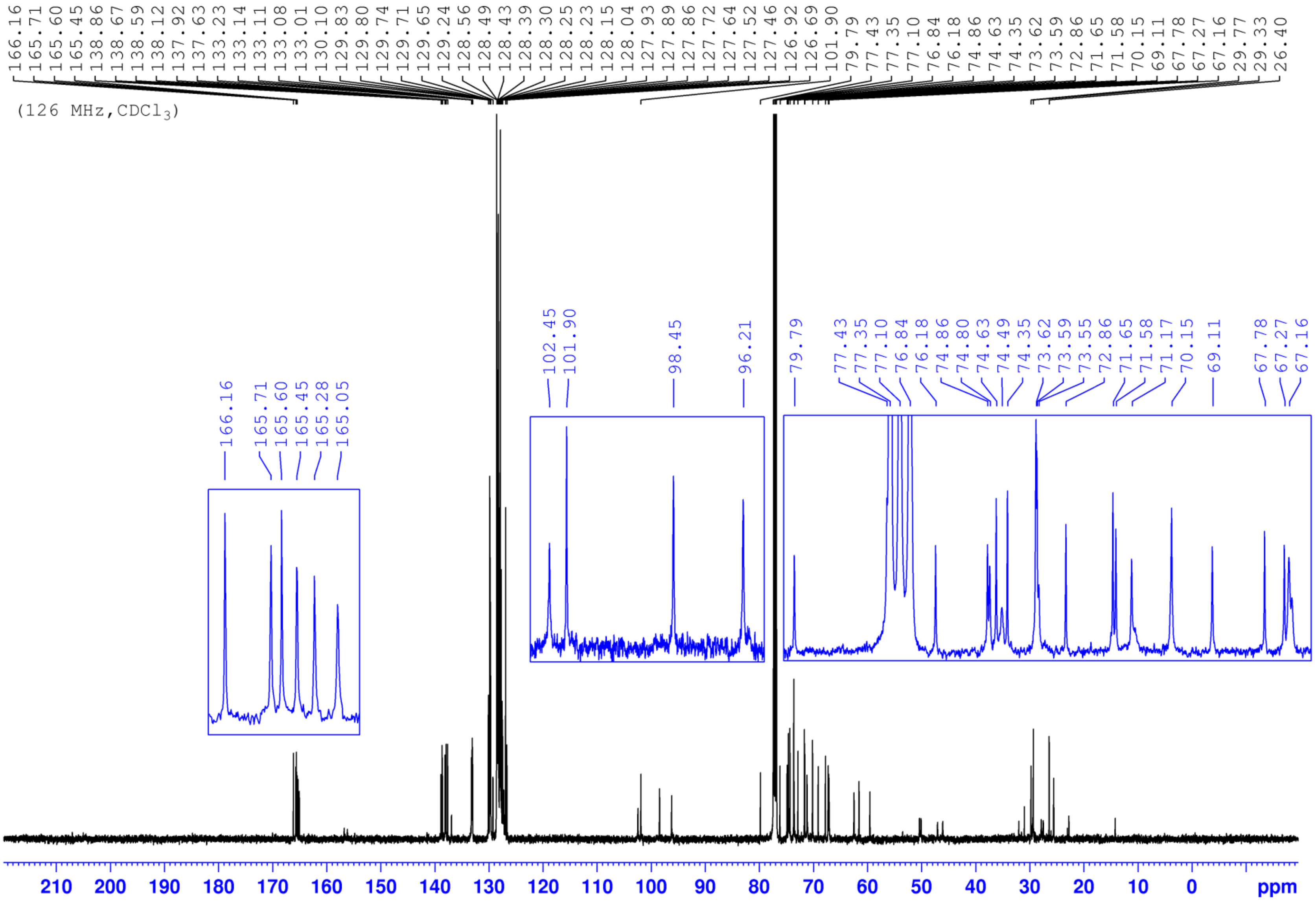

(126 MHz, CDCl3)
166.16 165.71 165.60 165.45 138.86 138.67 138.59 138.12 137.92 137.63 133.23 133.14 133.11 133.08 133.01 130.10 129.83 129.80 129.74 129.71 129.65 129.24 128.56 128.49 128.43 128.39 128.30 128.25 128.23 128.15 128.04 127.93 127.89 127.86 127.72 127.64 127.52 127.46 126.92 126.69 101.90 79.79 77.43 77.35 77.10 76.84 76.18 74.86 74.63 74.35 73.62 73.59 72.86 71.65 71.58 70.15 69.11 67.78 67.27 67.16 29.77 29.33 26.40
166.16 165.71 165.60 165.45 165.28 165.05
102.45 101.90 98.45 96.21
79.79 77.43 77.35 77.10 76.84 76.18 74.86 74.80 74.63 74.49 74.35 73.62 73.59 73.55 72.86 71.65 71.58 71.17 70.15 69.11 67.78 67.27 67.16
210 200 190 180 170 160 150 140 130 120 110 100 90 80 70 60 50 40 30 20 10 0 ppm

Compound **41**

^{1}H-NMR

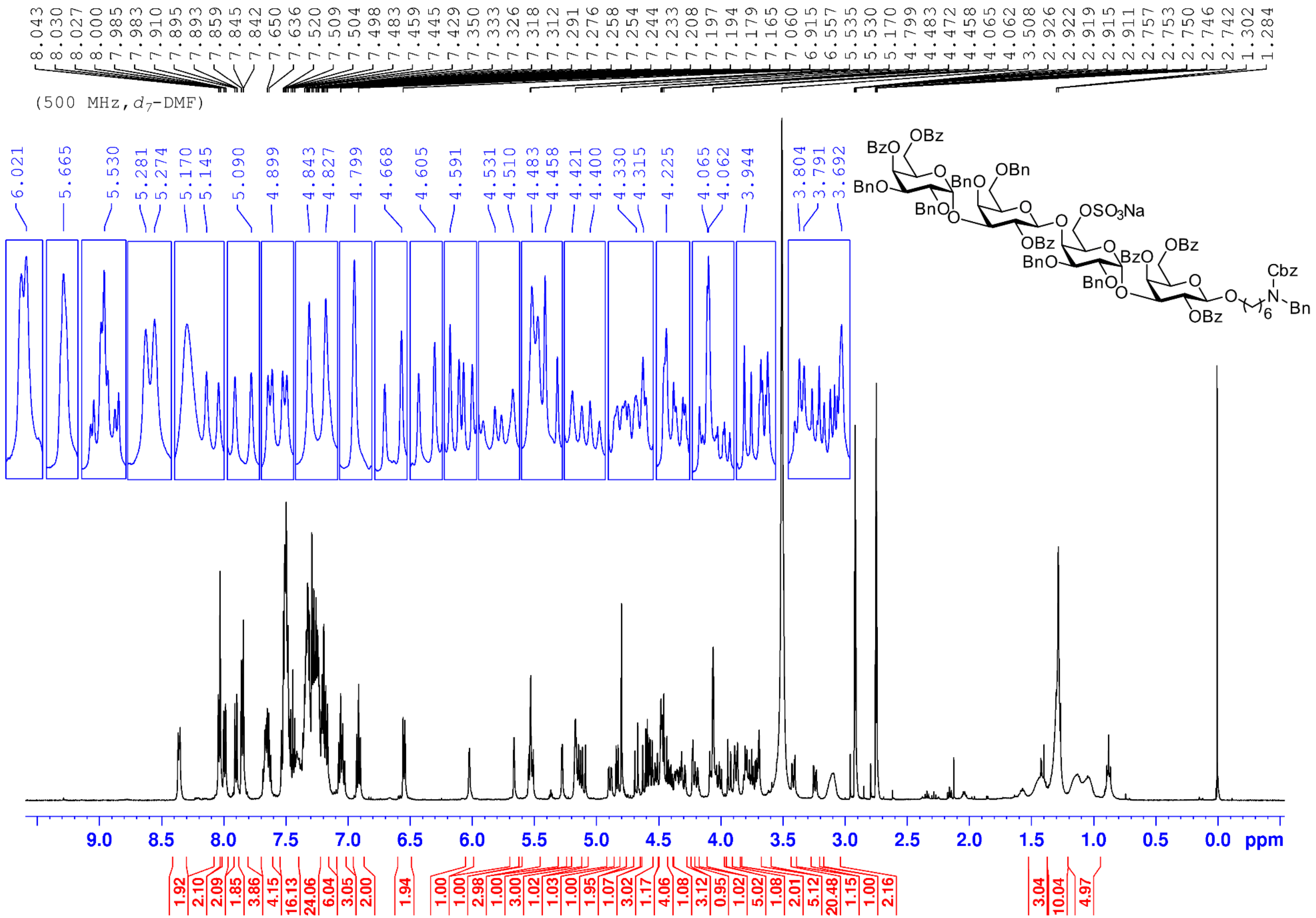

^{1}H-^{1}H COSY

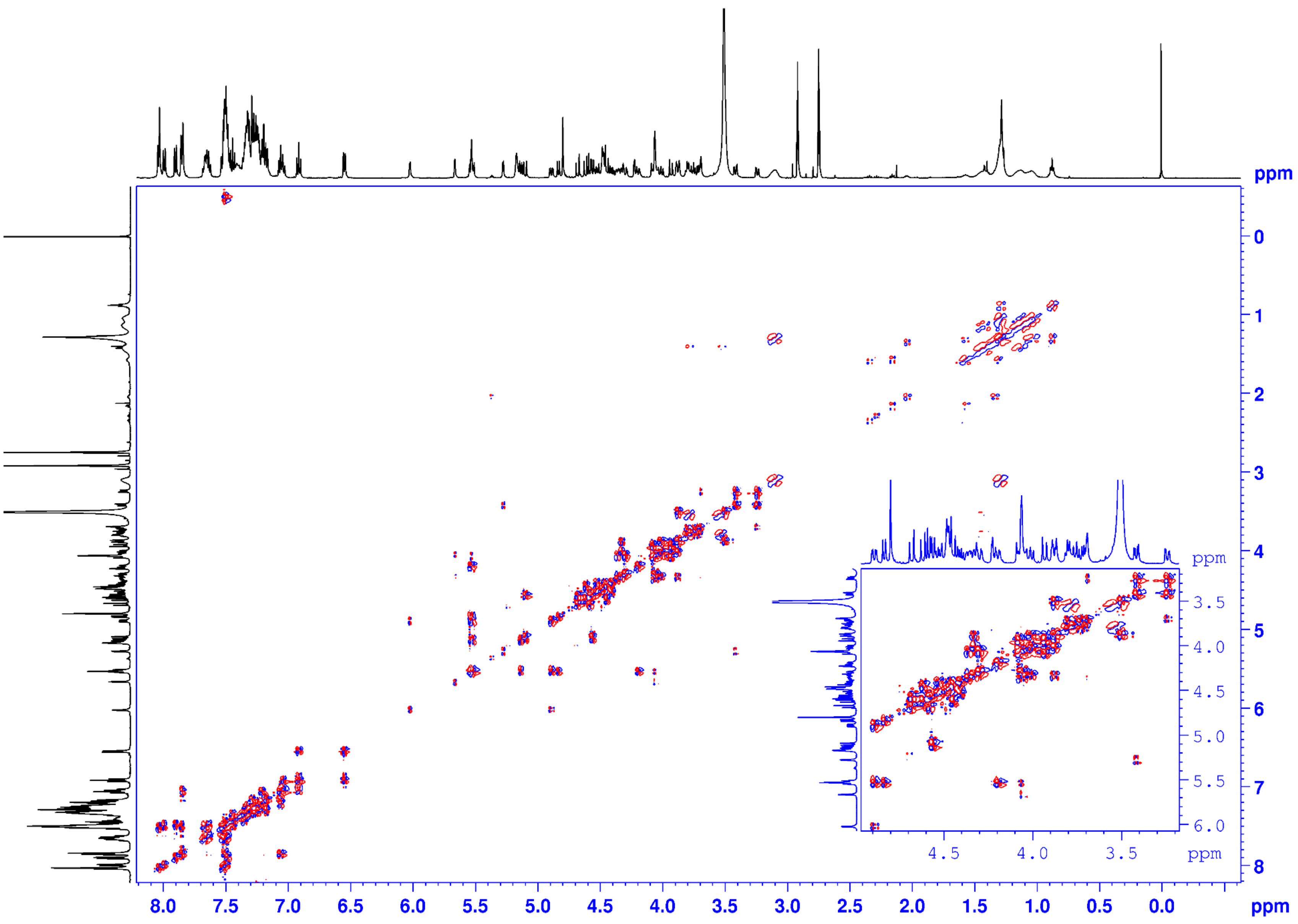

ppm
0
1
2
3
4
5
6
7
8
8.0
7.5
7.0
6.5
6.0
5.5
5.0
4.5
4.0
3.5
3.0
2.5
2.0
1.5
1.0
0.5
0.0
ppm
3.5
4.0
4.5
5.0
5.5
6.0
4.5
4.0
3.5
ppm

^{1}H-^{13}C HSQC

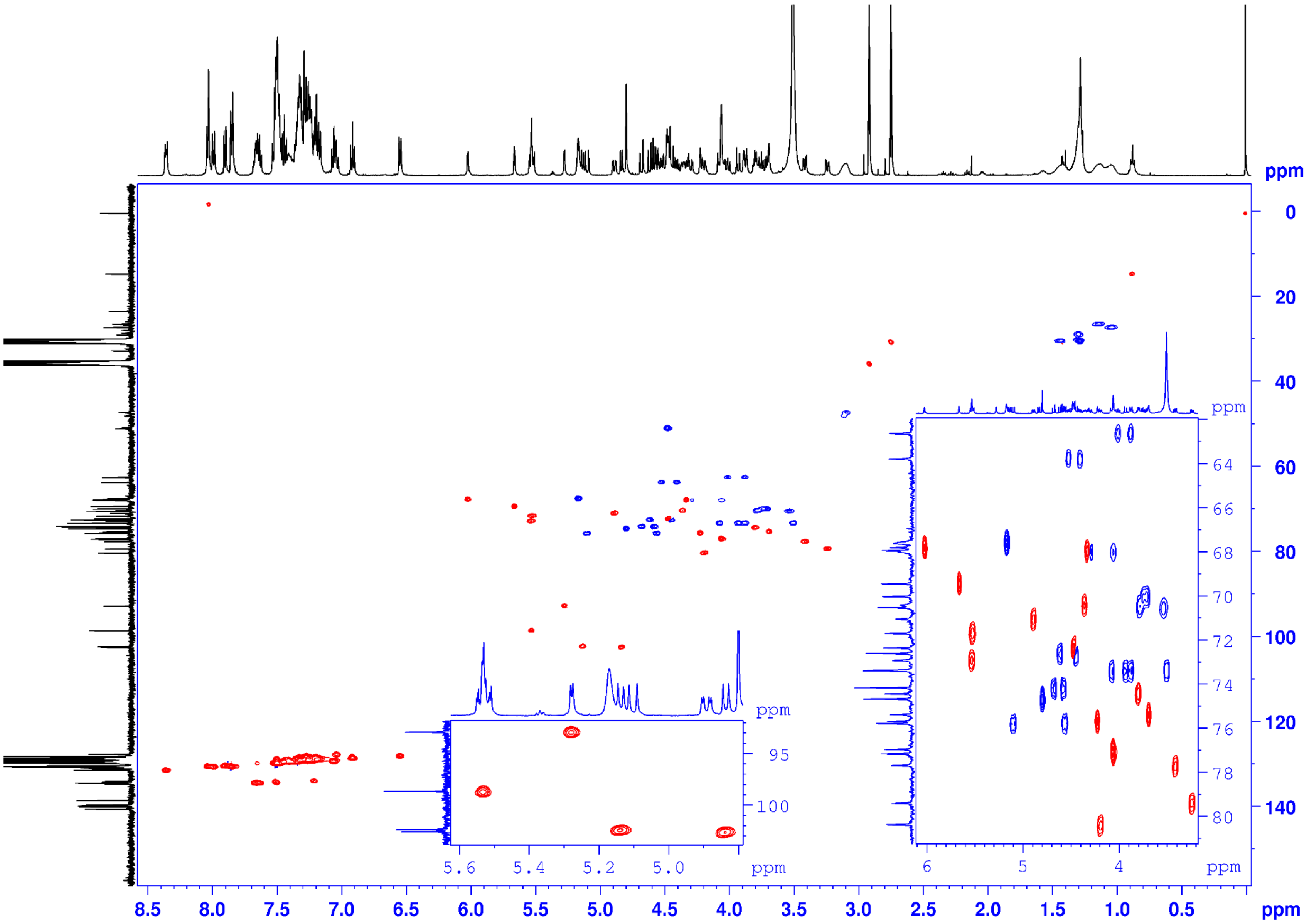

ppm
0
20
40
60
80
100
120
140
8.5
8.0
7.5
7.0
6.5
6.0
5.5
5.0
4.5
4.0
3.5
3.0
2.5
2.0
1.5
1.0
0.5
ppm
5.6
5.4
5.2
5.0
ppm
95
100
6
5
4
64
66
68
70
72
74
76
78
80

^{1}H-^{13}C non-decoupled HSQC

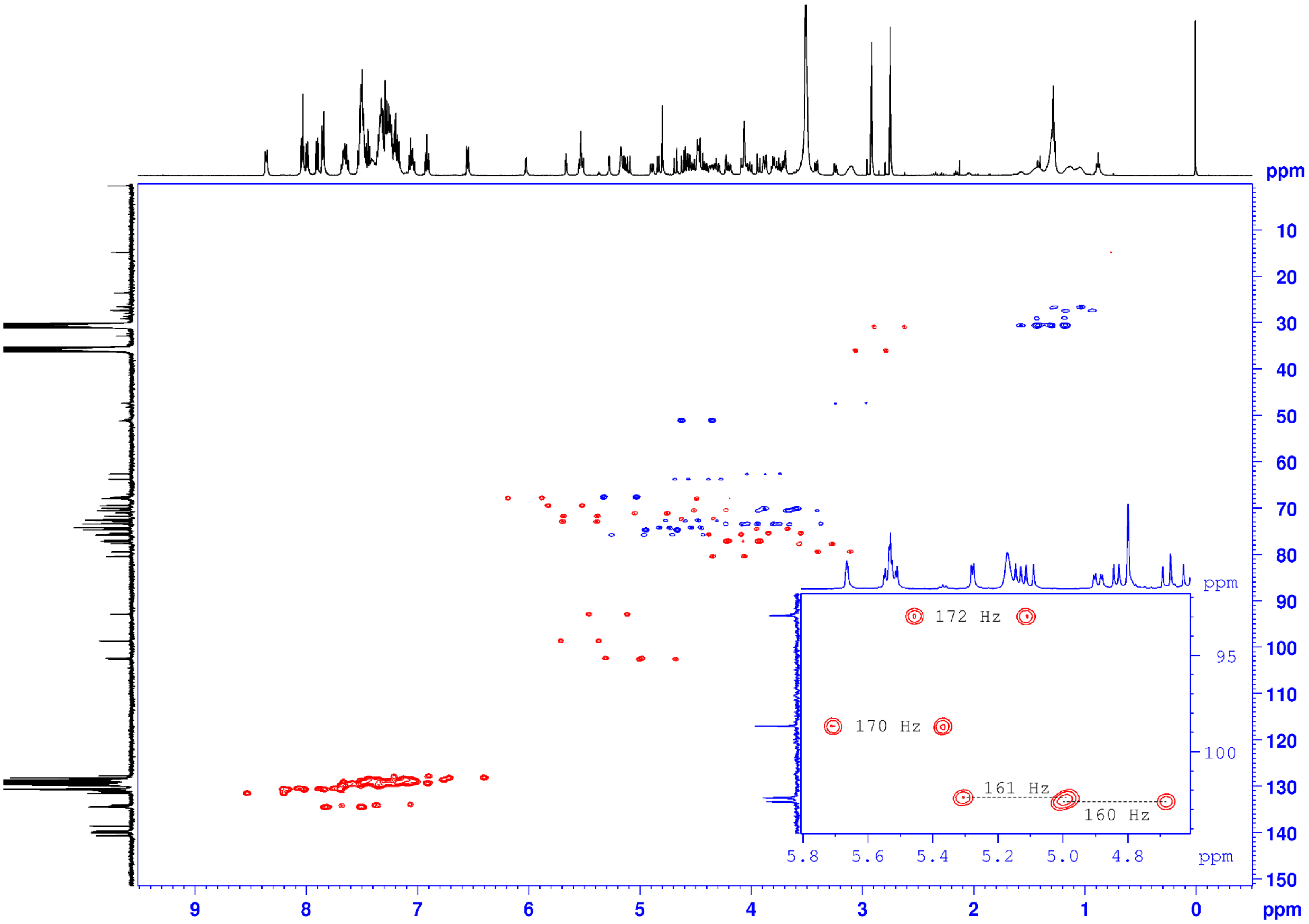

^{1}H-^{13}C HMBC

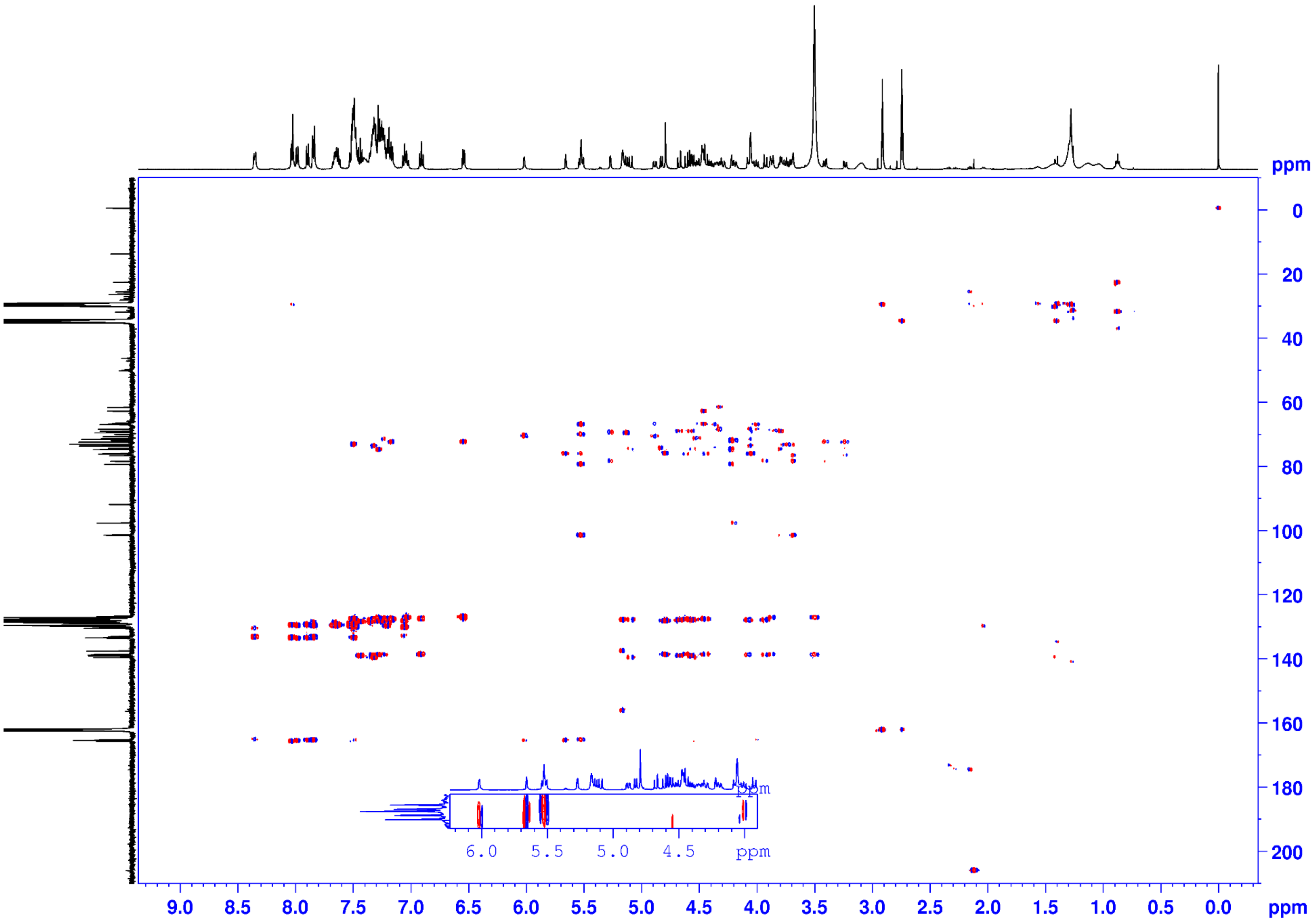

ppm
9.0 8.5 8.0 7.5 7.0 6.5 6.0 5.5 5.0 4.5 4.0 3.5 3.0 2.5 2.0 1.5 1.0 0.5 0.0
0 20 40 60 80 100 120 140 160 180 200
6.0 5.5 5.0 4.5

$^{13}C\{^1H\}$ NMR

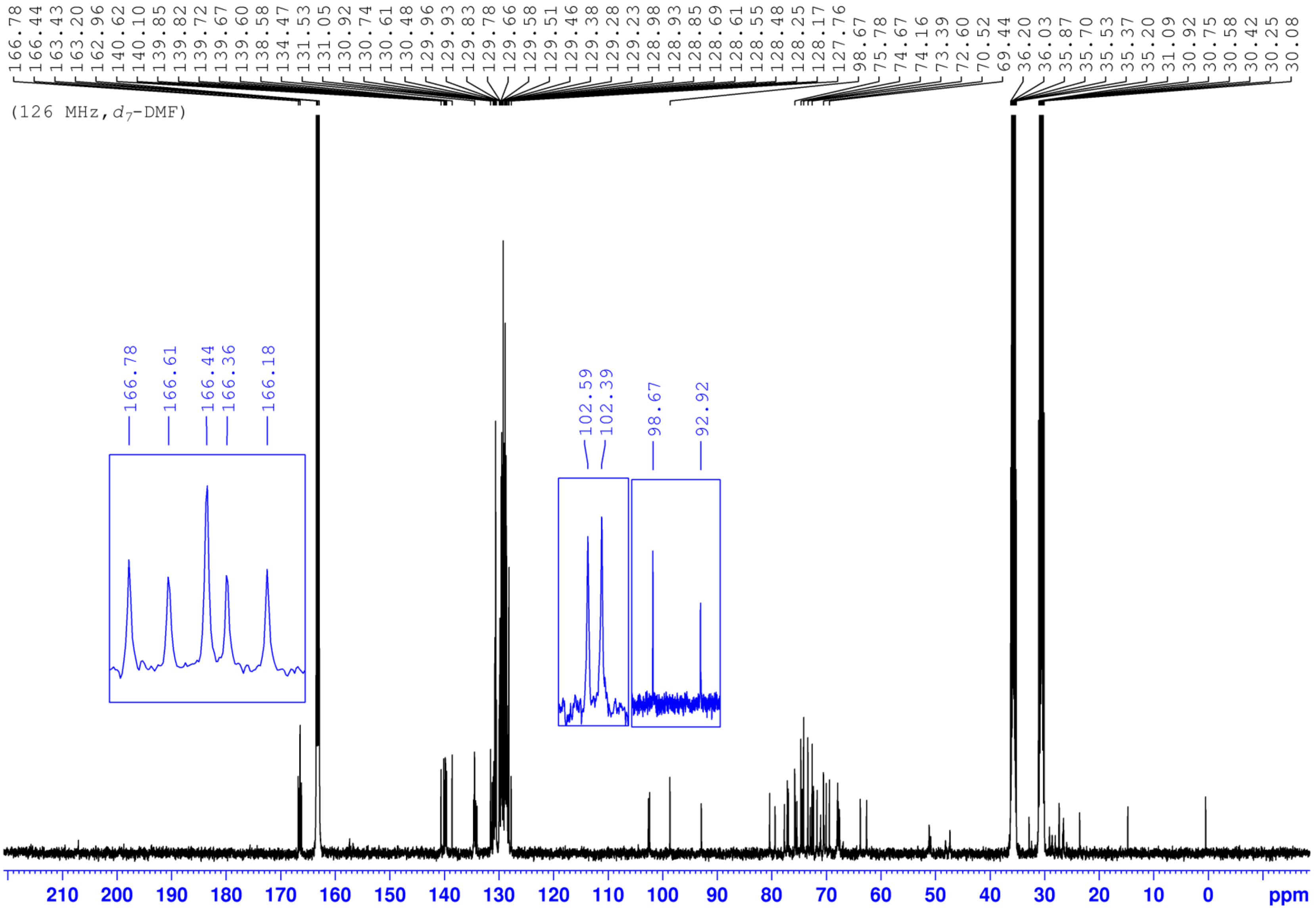

166.78
166.44
163.43
163.20
162.96
140.62
140.10
139.85
139.82
139.72
139.67
139.60
138.58
134.47
131.53
131.05
130.92
130.74
130.61
130.48
129.96
129.93
129.83
129.78
129.66
129.58
129.51
129.46
129.38
129.28
129.23
128.98
128.93
128.85
128.69
128.61
128.55
128.48
128.25
128.17
127.76
98.67
75.78
74.67
74.16
73.39
72.60
70.52
69.44
36.20
36.03
35.87
35.70
35.53
35.37
35.20
31.09
30.92
30.75
30.58
30.42
30.25
30.08
(126 MHz, d_7-DMF)
166.78
166.61
166.44
166.36
166.18
102.59
102.39
98.67
92.92
210 200 190 180 170 160 150 140 130 120 110 100 90 80 70 60 50 40 30 20 10 0 ppm

Compound **S1**

^{1}H-NMR

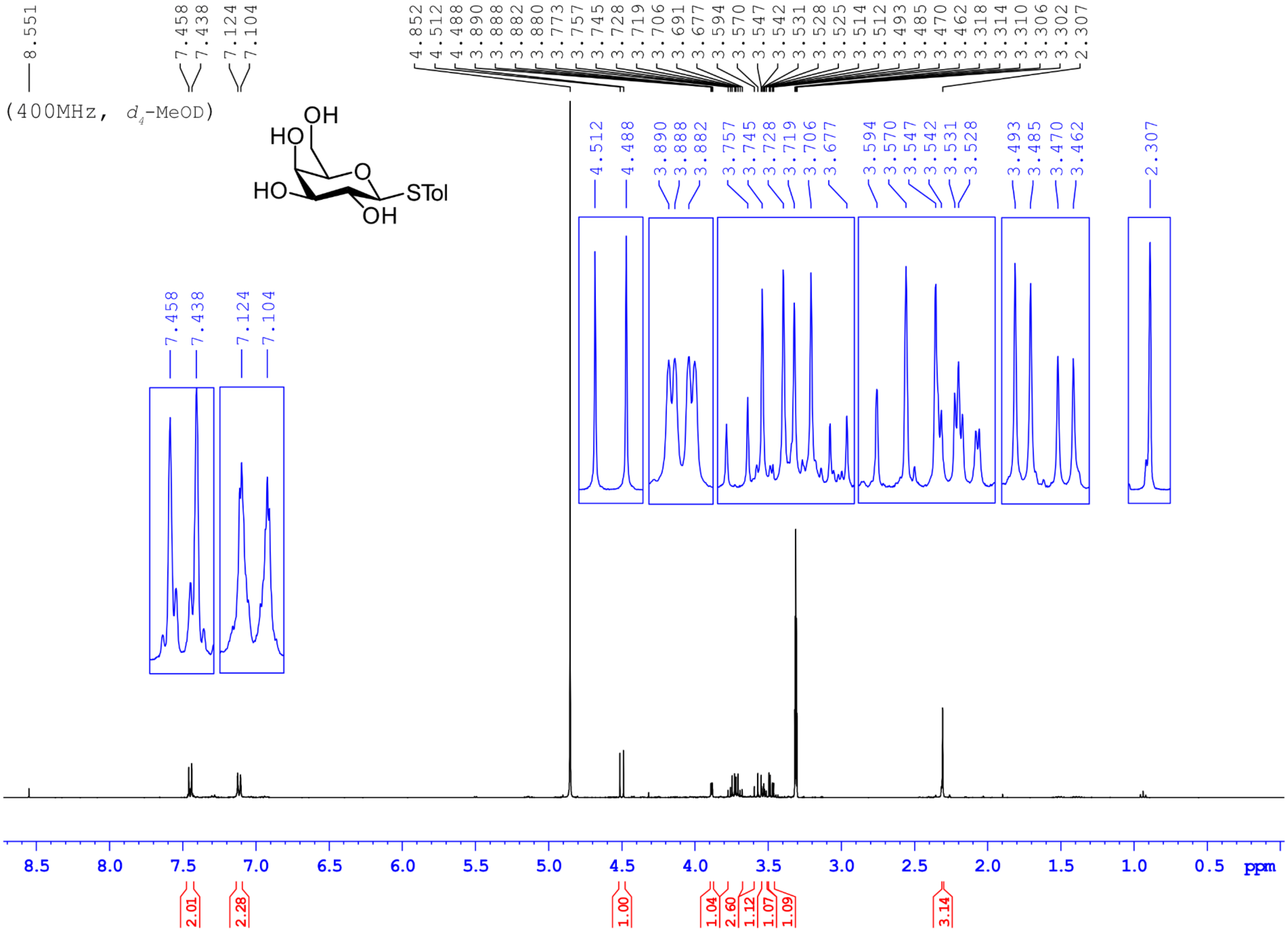

^{1}H-^{1}H COSY

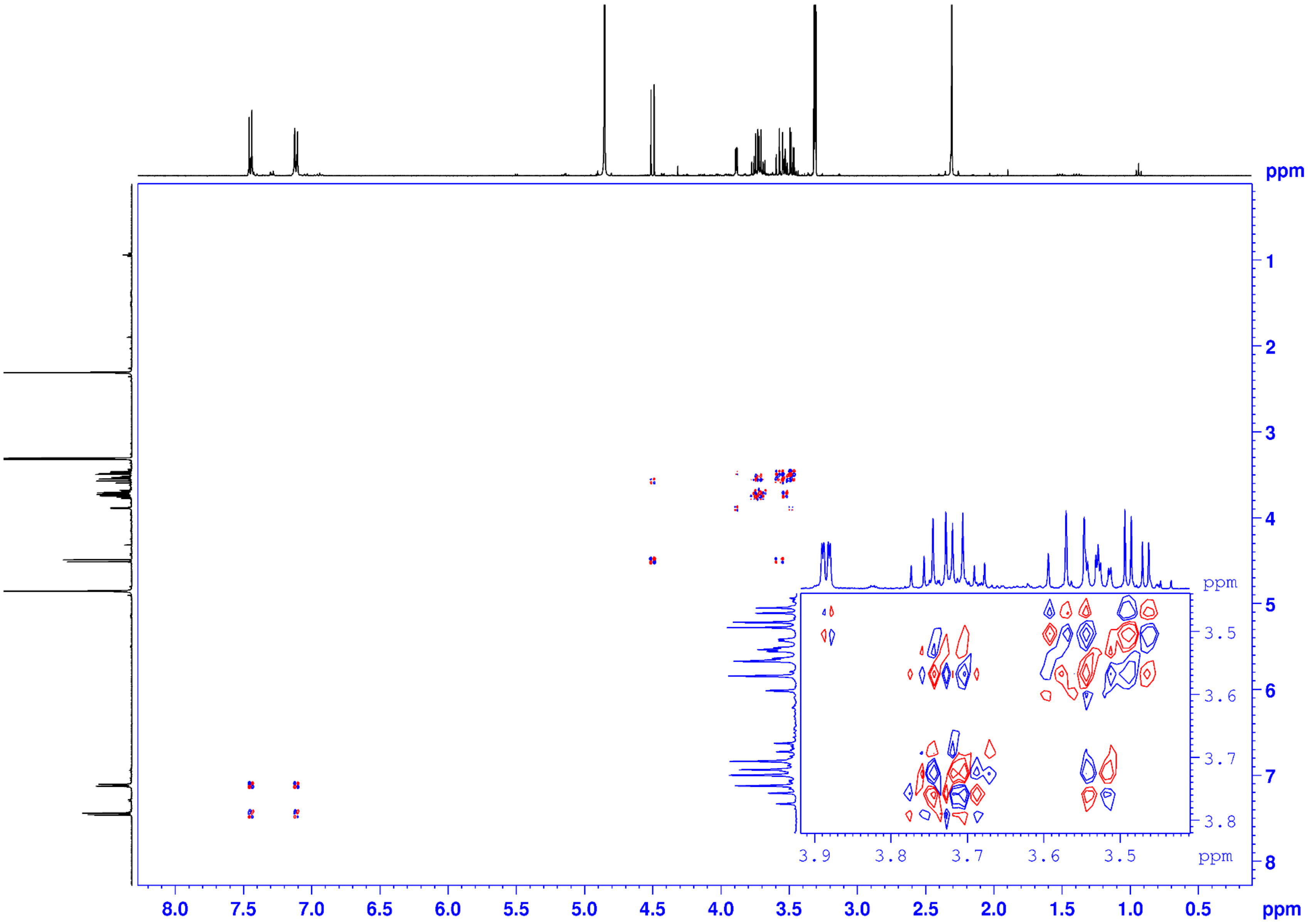
ppm
1
2
3
4
5
6
7
8
8.0 7.5 7.0 6.5 6.0 5.5 5.0 4.5 4.0 3.5 3.0 2.5 2.0 1.5 1.0 0.5 ppm
ppm
3.5
3.6
3.7
3.8
3.9 3.8 3.7 3.6 3.5 ppm

^{1}H-^{13}C HSQC

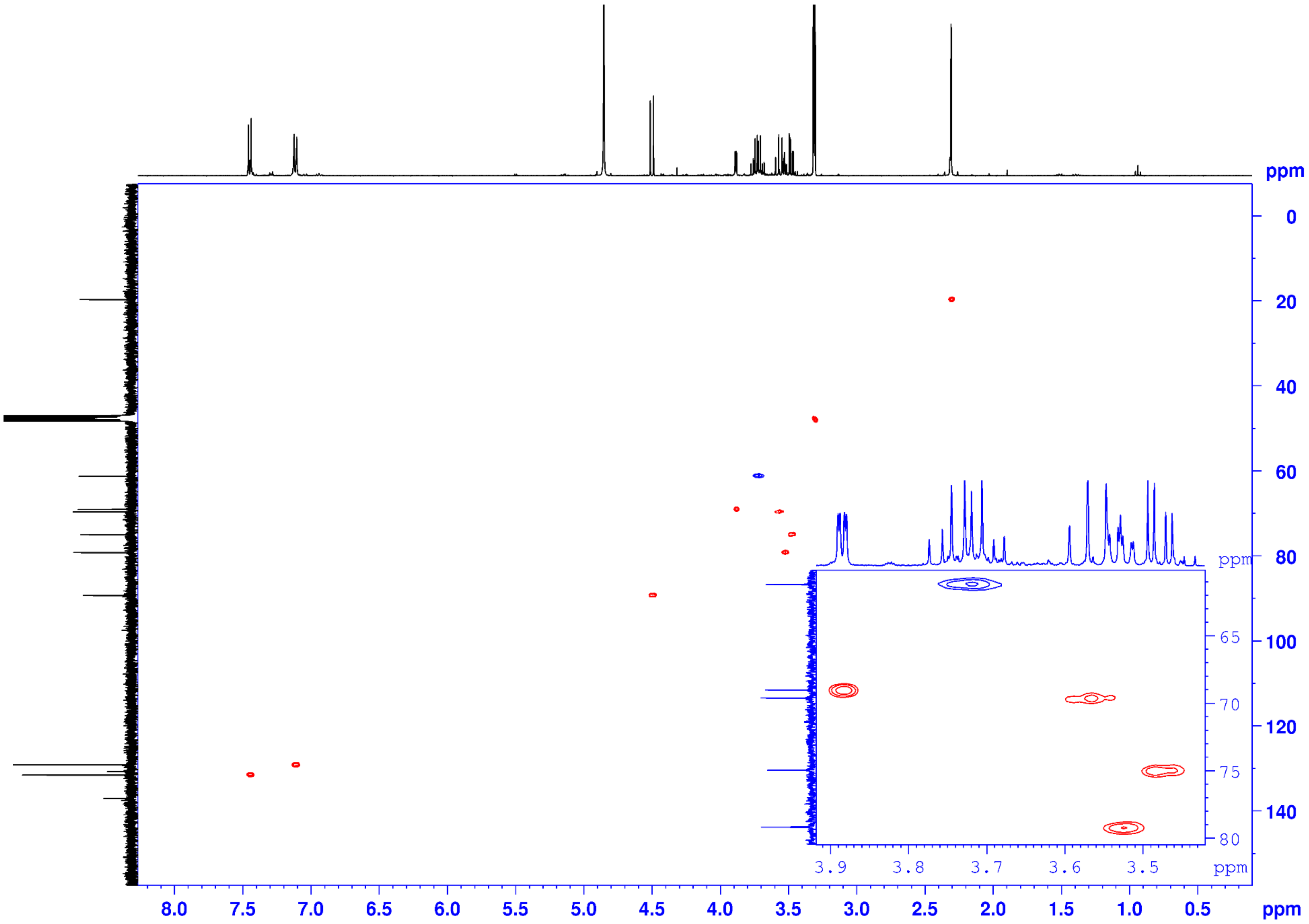

ppm
0
20
40
60
80
100
120
140
8.0
7.5
7.0
6.5
6.0
5.5
5.0
4.5
4.0
3.5
3.0
2.5
2.0
1.5
1.0
0.5
ppm
3.9
3.8
3.7
3.6
3.5
ppm
65
70
75
80
ppm

$^{13}C\{^{1}H\}$ NMR

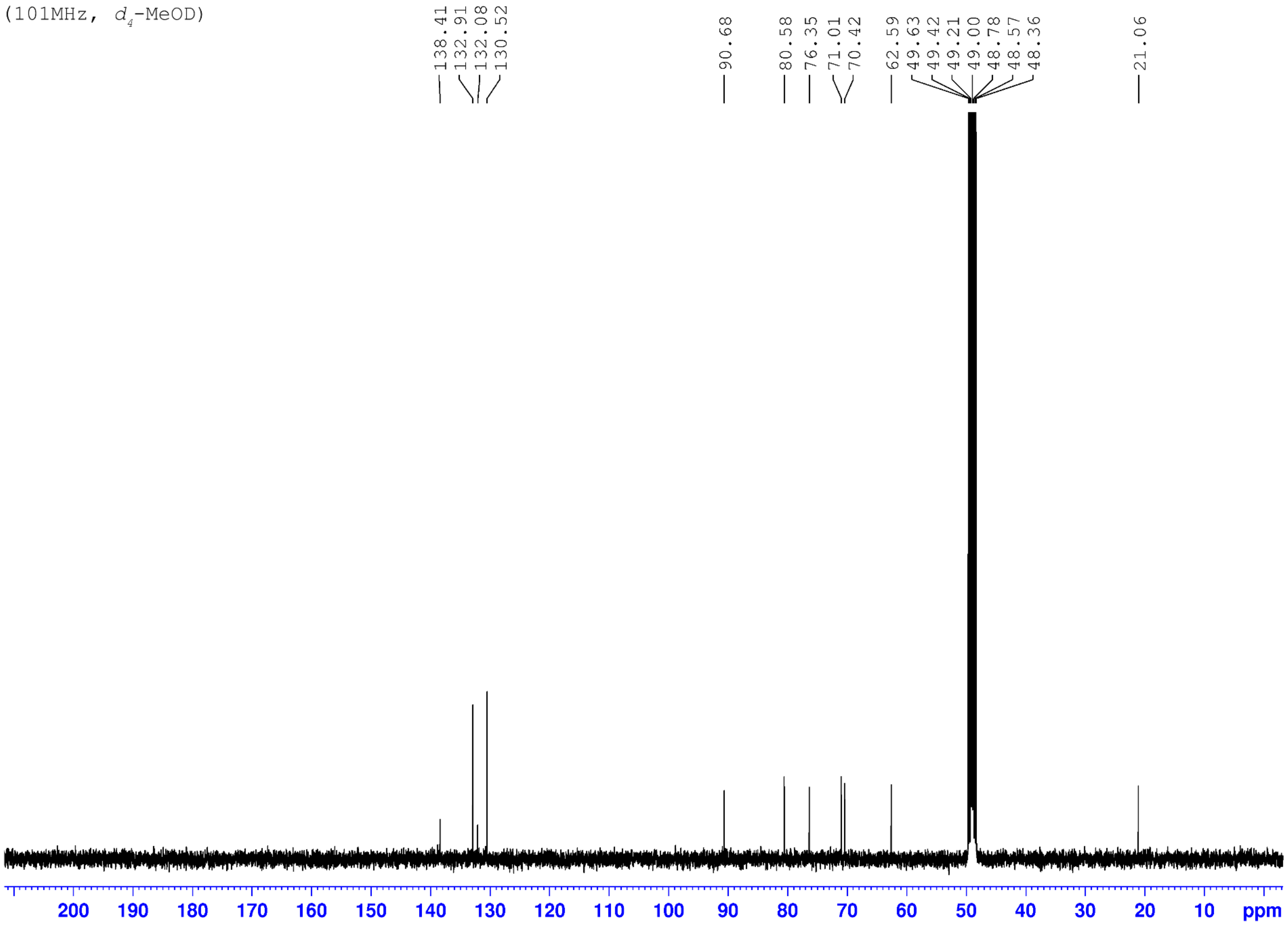
(101MHz, d_4-MeOD)
138.41
132.91
132.08
130.52
90.68
80.58
76.35
71.01
70.42
62.59
49.63
49.42
49.21
49.00
48.78
48.57
48.36
21.06
200 190 180 170 160 150 140 130 120 110 100 90 80 70 60 50 40 30 20 10 ppm

Compound **S2**

^{1}H-NMR

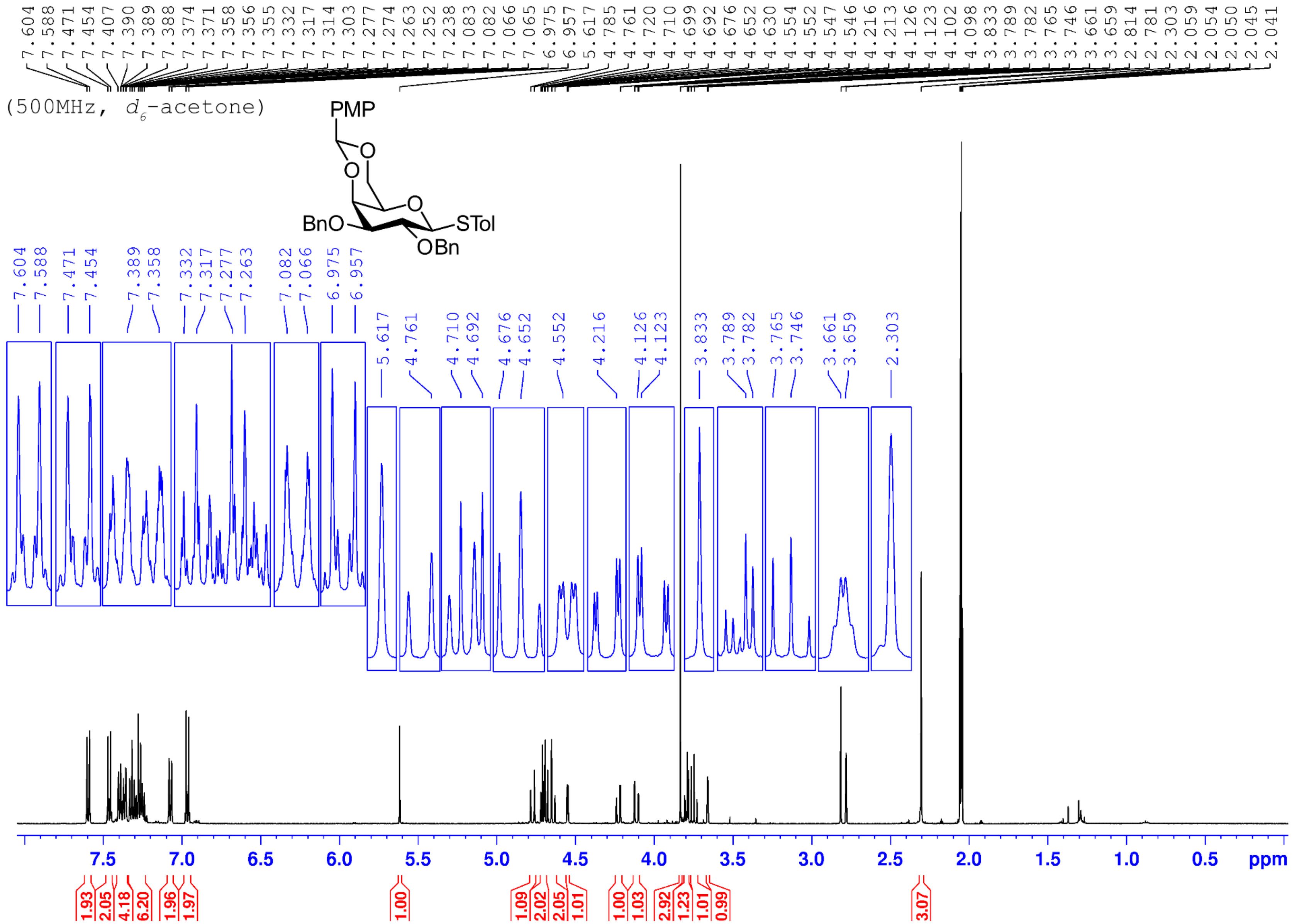

^{1}H-^{1}H COSY

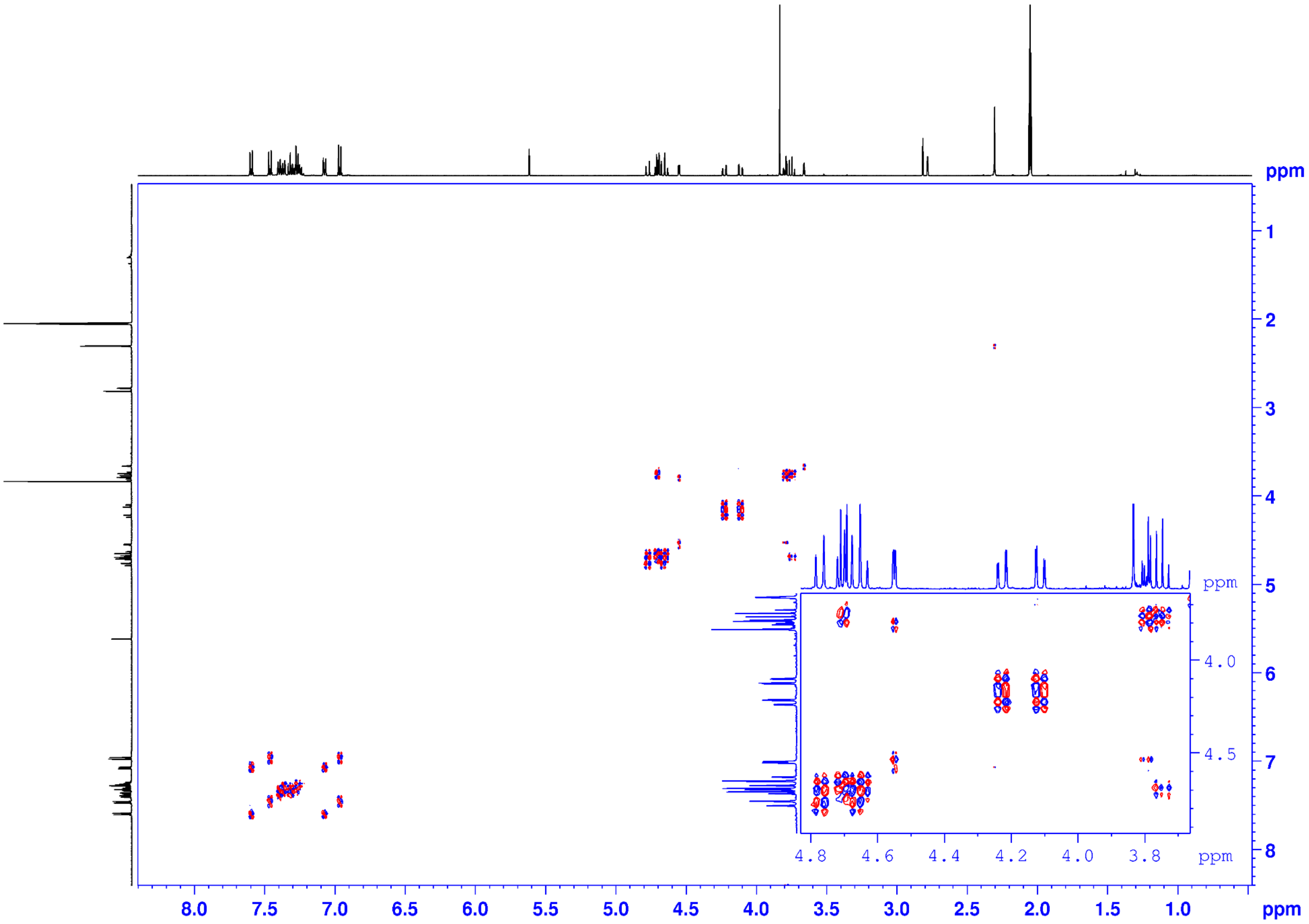
ppm
1
2
3
4
5
6
7
8
8.0
7.5
7.0
6.5
6.0
5.5
5.0
4.5
4.0
3.5
3.0
2.5
2.0
1.5
1.0
ppm
4.0
4.5
4.8
4.6
4.4
4.2
4.0
3.8
ppm

^{1}H-^{13}C HSQC

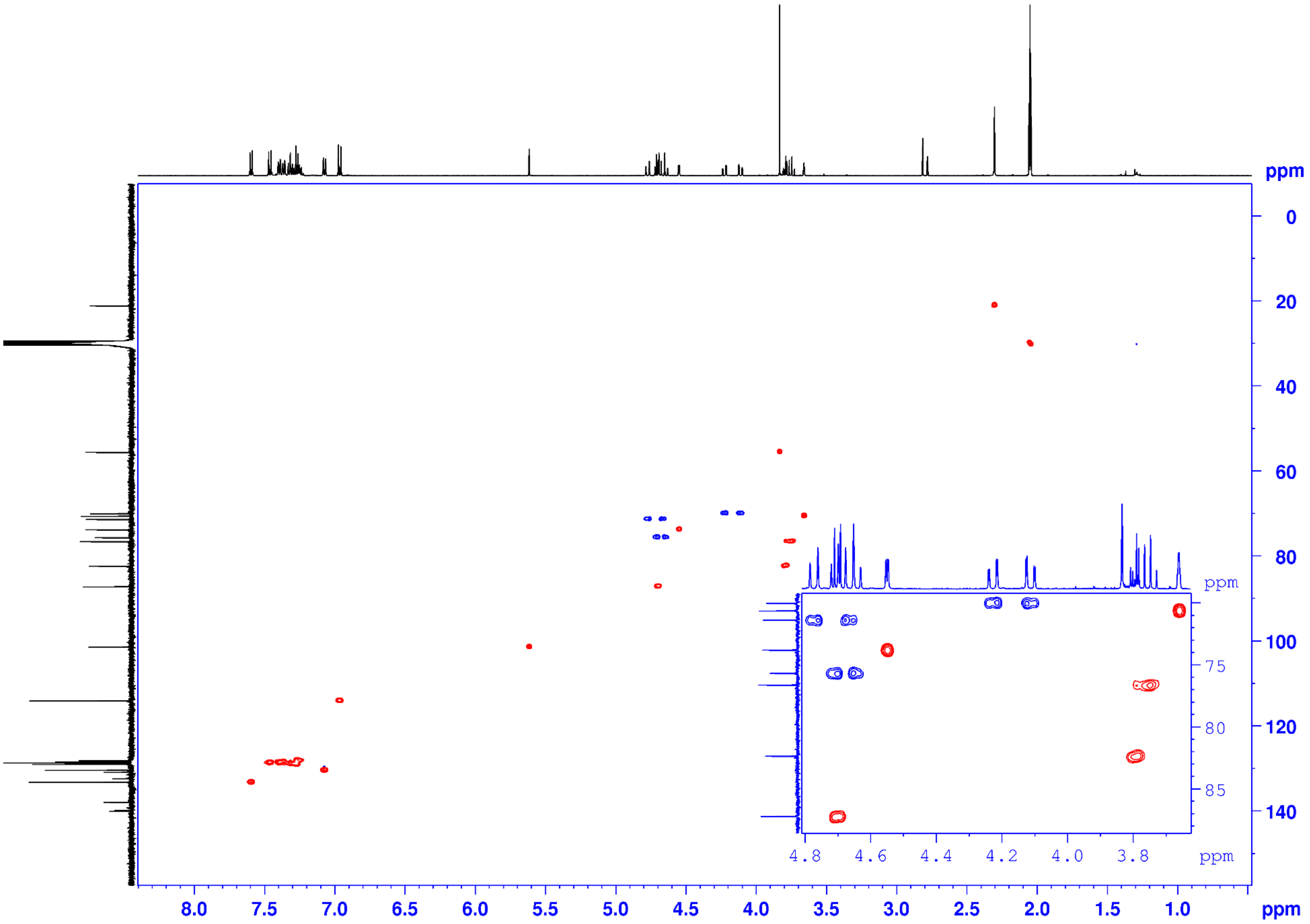

ppm
0
20
40
60
80
100
120
140
8.0
7.5
7.0
6.5
6.0
5.5
5.0
4.5
4.0
3.5
3.0
2.5
2.0
1.5
1.0
ppm
75
80
85
4.8
4.6
4.4
4.2
4.0
3.8
ppm

^{1}H-^{13}C HMBC

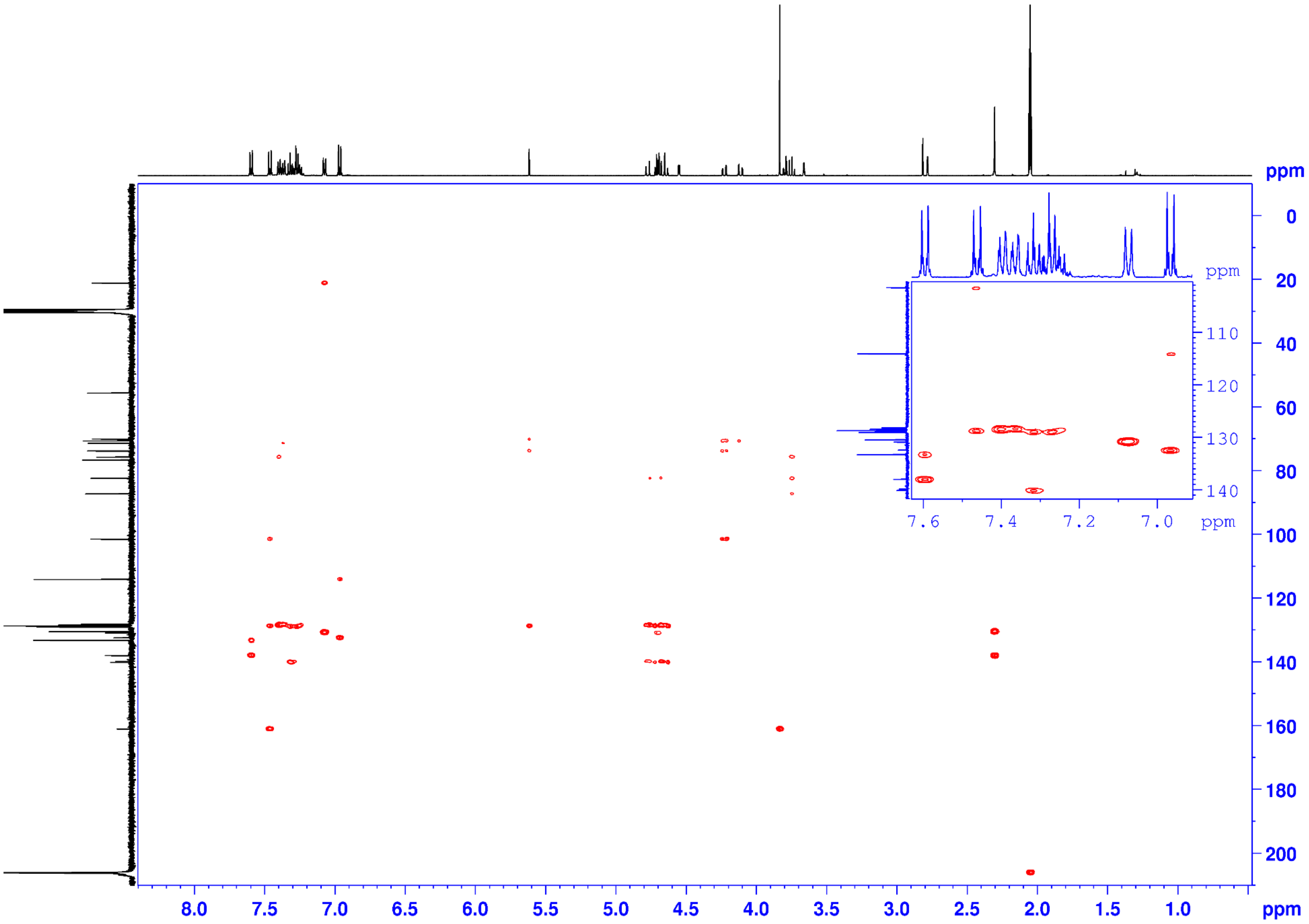

ppm
0
20
40
60
80
100
120
140
160
180
200
8.0
7.5
7.0
6.5
6.0
5.5
5.0
4.5
4.0
3.5
3.0
2.5
2.0
1.5
1.0
ppm
110
120
130
140
7.6
7.4
7.2
7.0

$^{13}C\{^1H\}$ NMR

(126MHz, d_6-acetone)

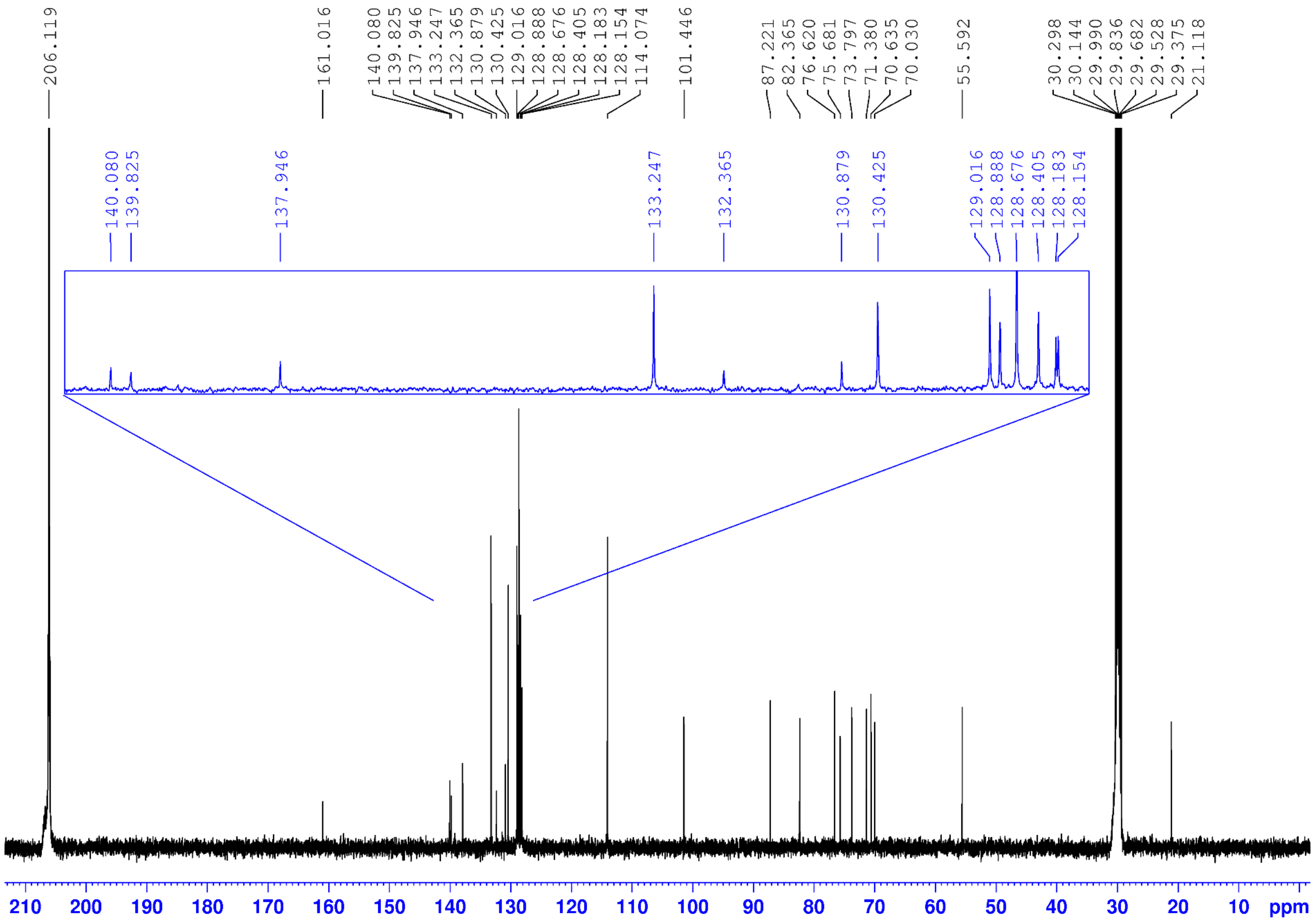

Compound **S3**

^{1}H-NMR

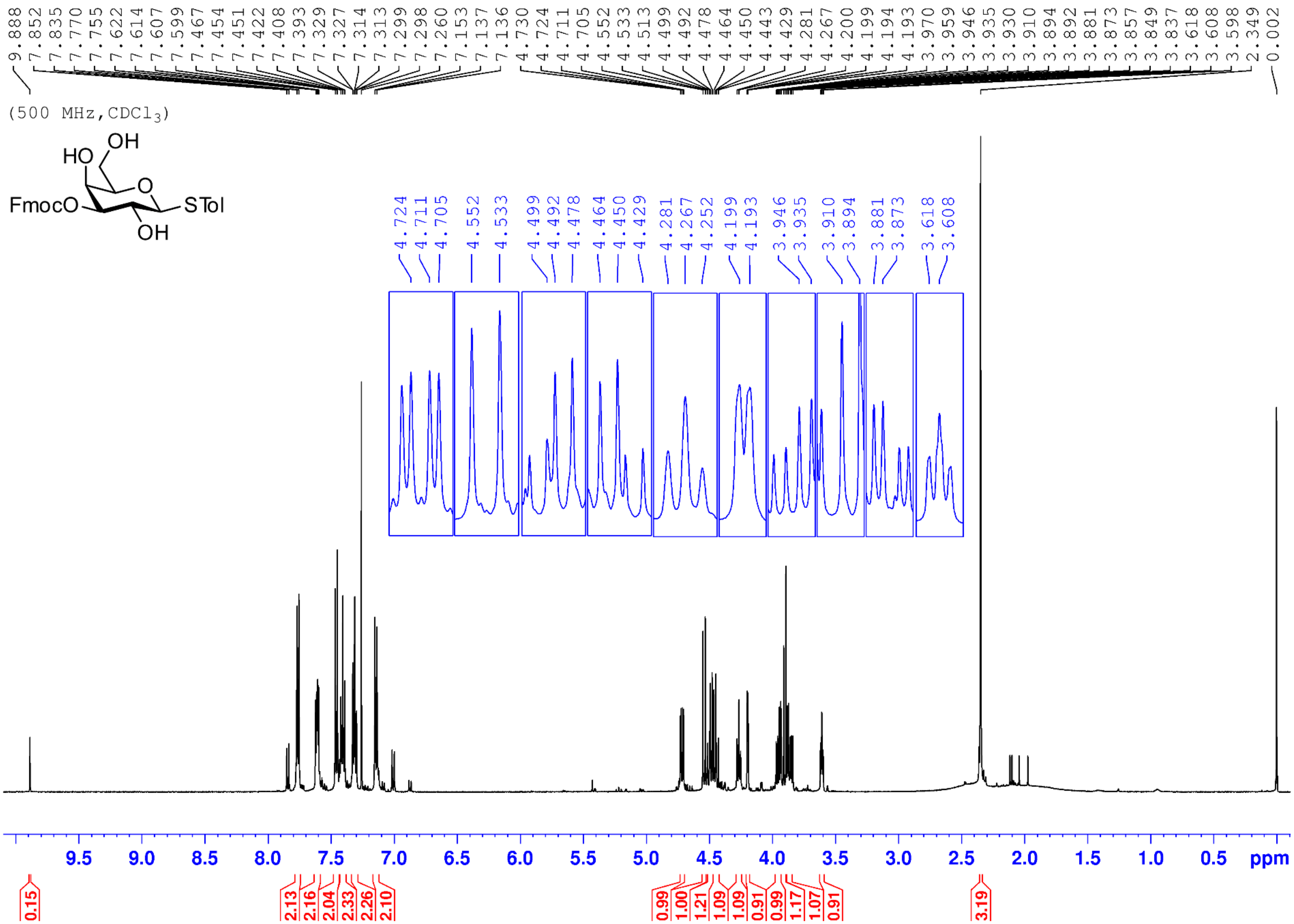

^1H-^1H COSY

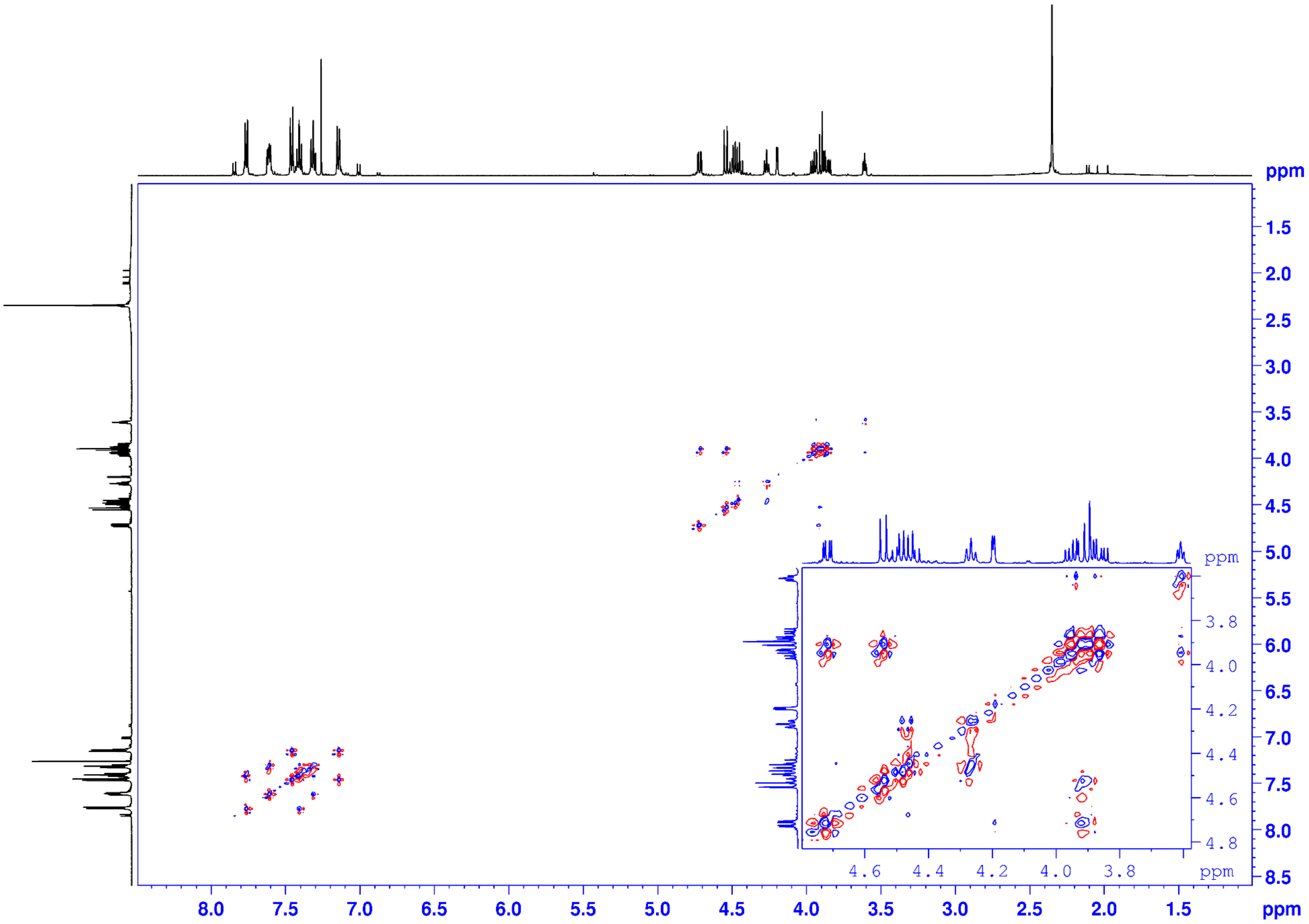
ppm
1.5
2.0
2.5
3.0
3.5
4.0
4.5
5.0
5.5
6.0
6.5
7.0
7.5
8.0
8.5
8.0 7.5 7.0 6.5 6.0 5.5 5.0 4.5 4.0 3.5 3.0 2.5 2.0 1.5 ppm
ppm
3.8
4.0
4.2
4.4
4.6
4.8
4.6 4.4 4.2 4.0 3.8 ppm

^{1}H-^{13}C HSQC

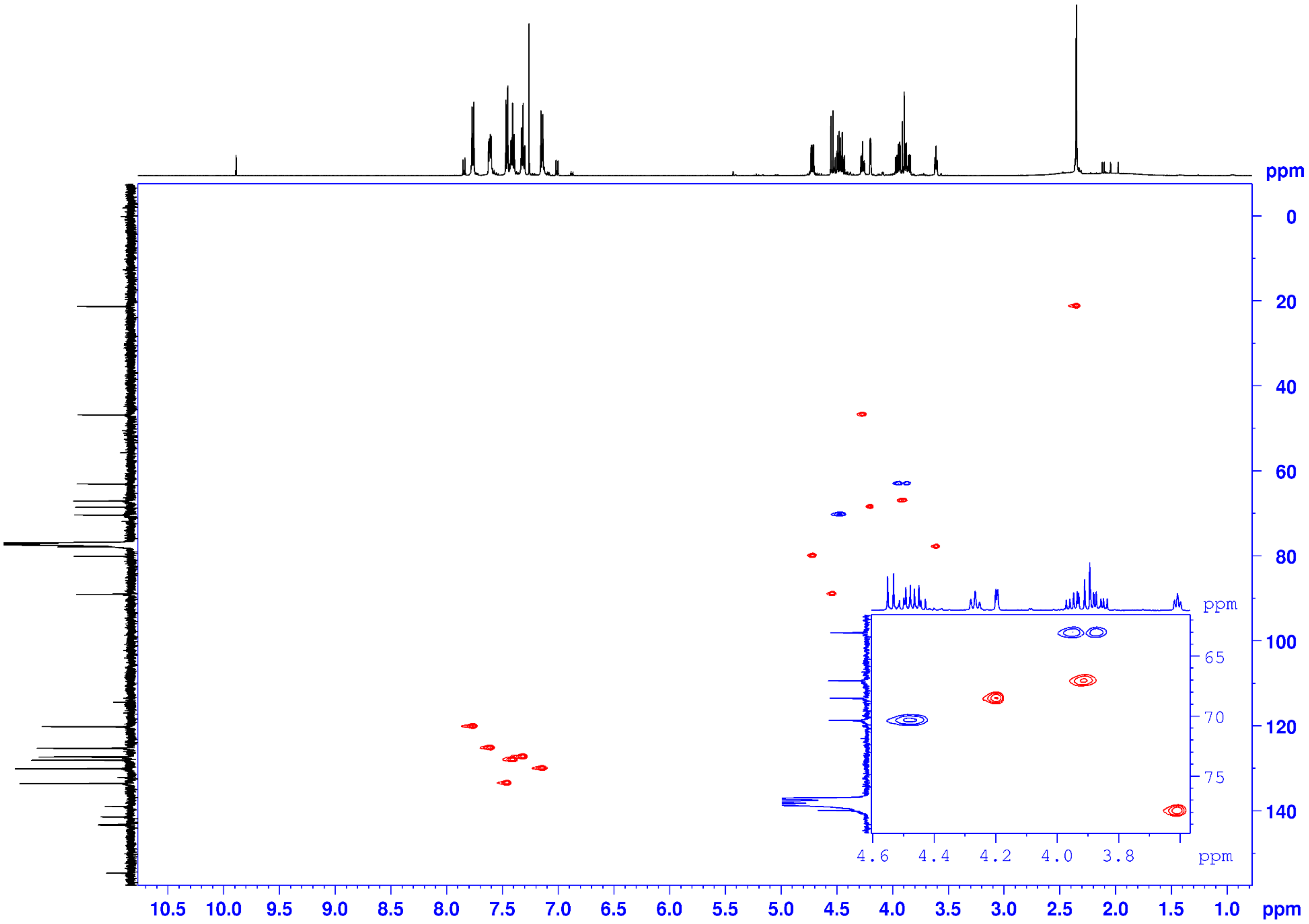

ppm
0
20
40
60
80
100
120
140
10.5 10.0 9.5 9.0 8.5 8.0 7.5 7.0 6.5 6.0 5.5 5.0 4.5 4.0 3.5 3.0 2.5 2.0 1.5 1.0
ppm
65
70
75
4.6 4.4 4.2 4.0 3.8

$^{13}C\{^{1}H\}$ NMR

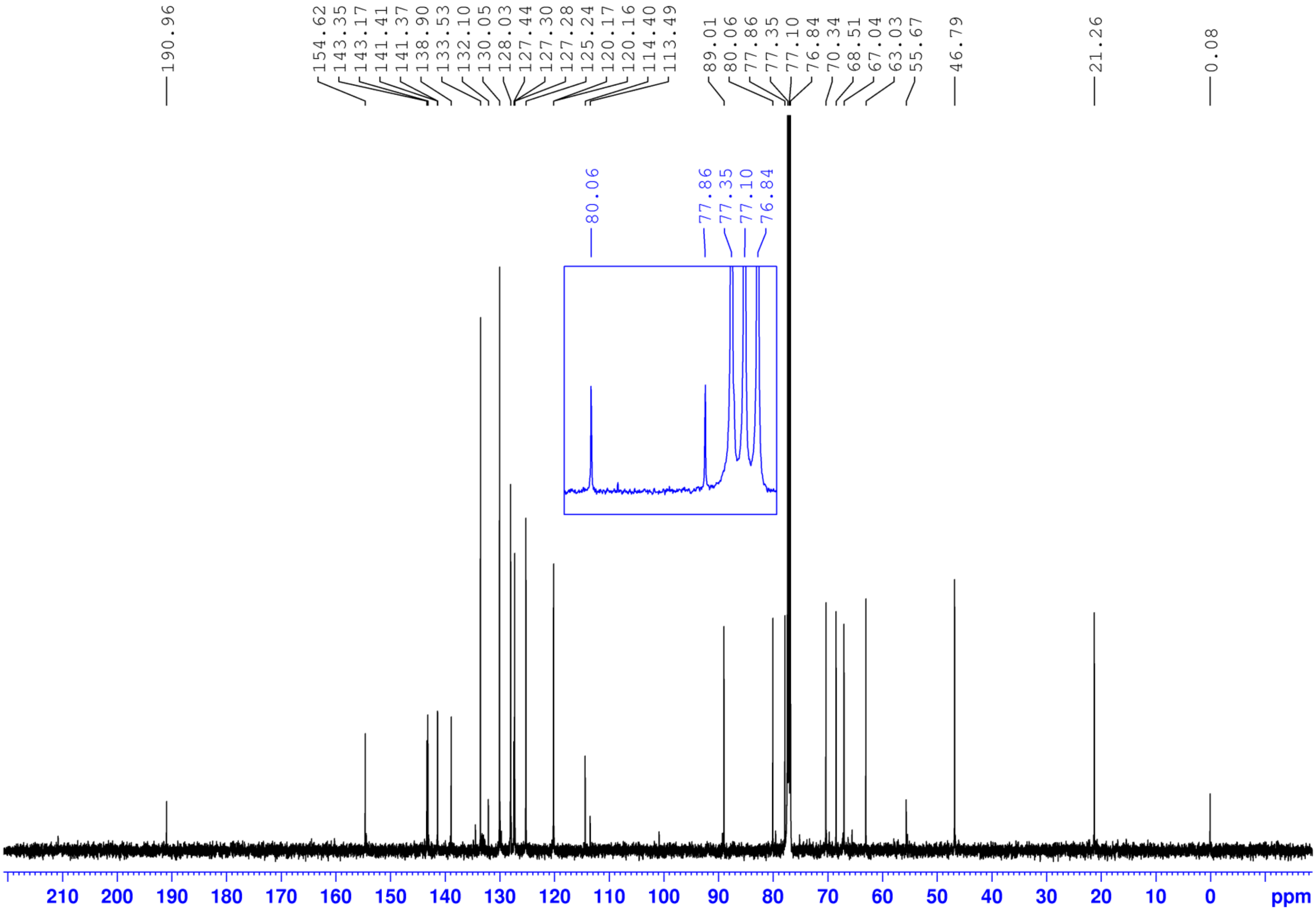

190.96
154.62
143.35
143.17
141.41
141.37
138.90
133.53
132.10
130.05
128.03
127.44
127.30
127.28
125.24
120.17
120.16
114.40
113.49
89.01
80.06
77.86
77.35
77.10
76.84
70.34
68.51
67.04
63.03
55.67
46.79
21.26
0.08
80.06
77.86
77.35
77.10
76.84
210
200
190
180
170
160
150
140
130
120
110
100
90
80
70
60
50
40
30
20
10
0
ppm

Compound **S4a**

^{1}H-NMR

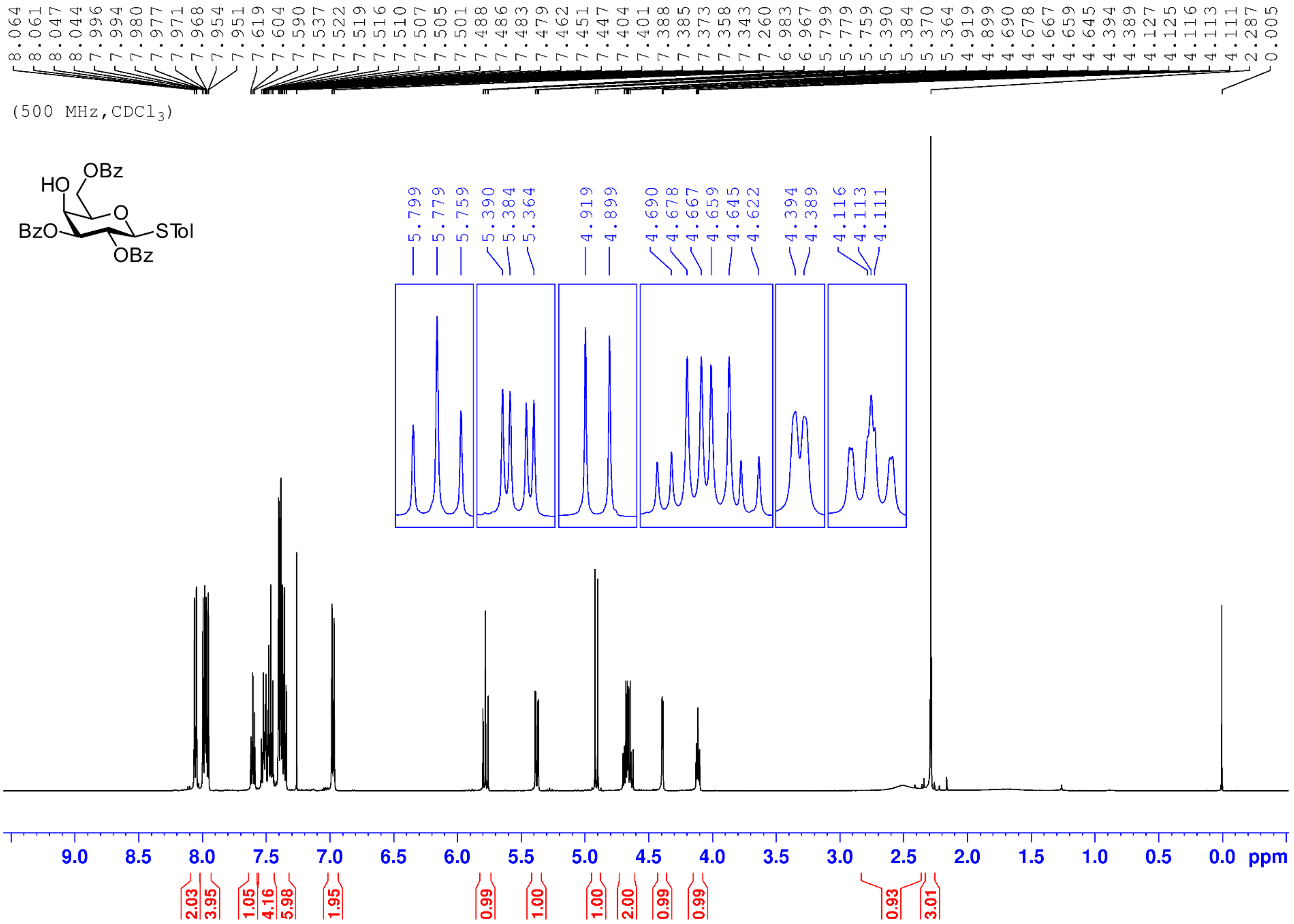

^1H-^1H COSY

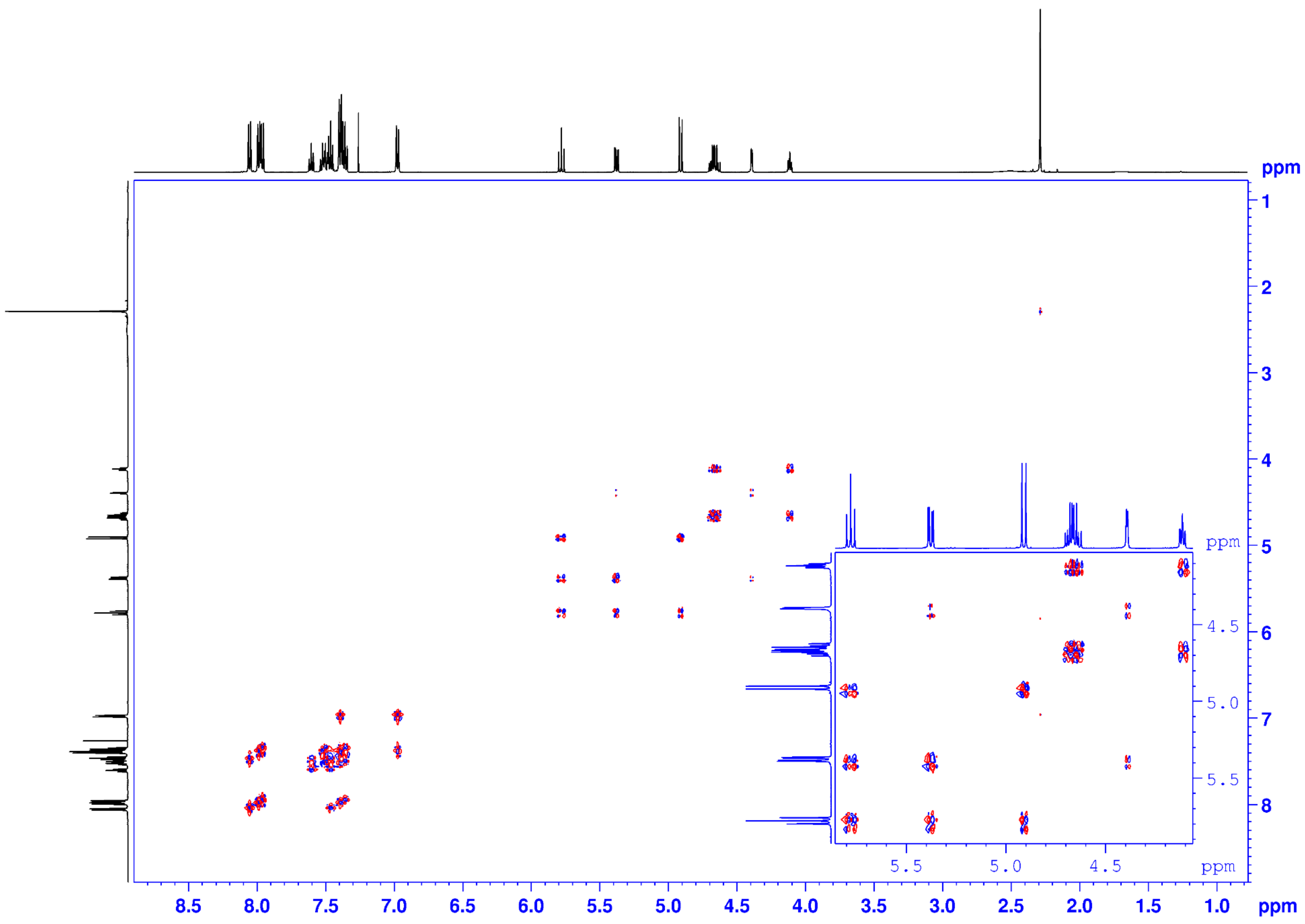
ppm
1
2
3
4
5
6
7
8
8.5
8.0
7.5
7.0
6.5
6.0
5.5
5.0
4.5
4.0
3.5
3.0
2.5
2.0
1.5
1.0
ppm
4.5
5.0
5.5
5.5
5.0
4.5
ppm

^{1}H-^{13}C HSQC

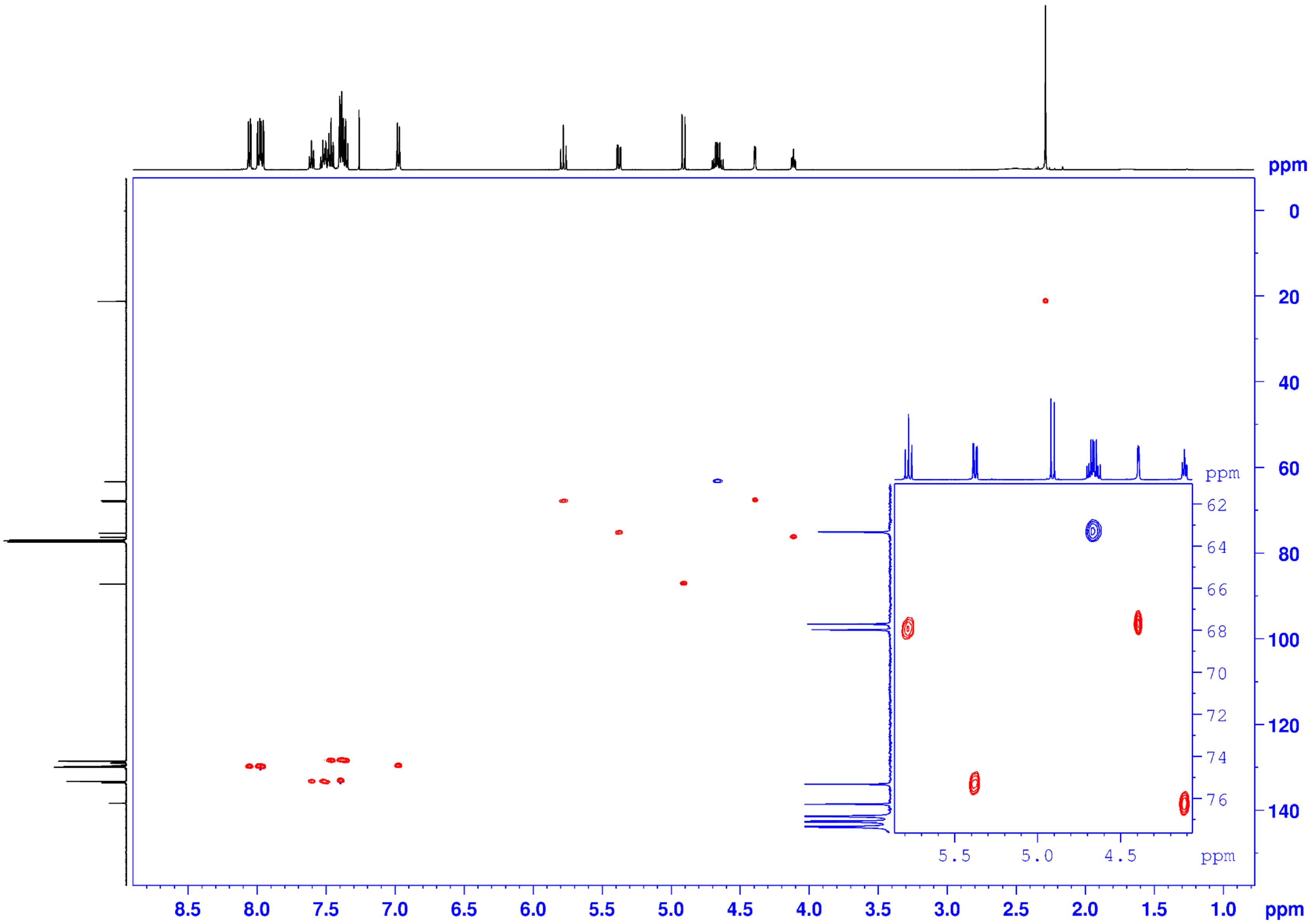
ppm
0
20
40
60
80
100
120
140
8.5
8.0
7.5
7.0
6.5
6.0
5.5
5.0
4.5
4.0
3.5
3.0
2.5
2.0
1.5
1.0
ppm
62
64
66
68
70
72
74
76
5.5
5.0
4.5
ppm

^{1}H-^{13}C HMBC

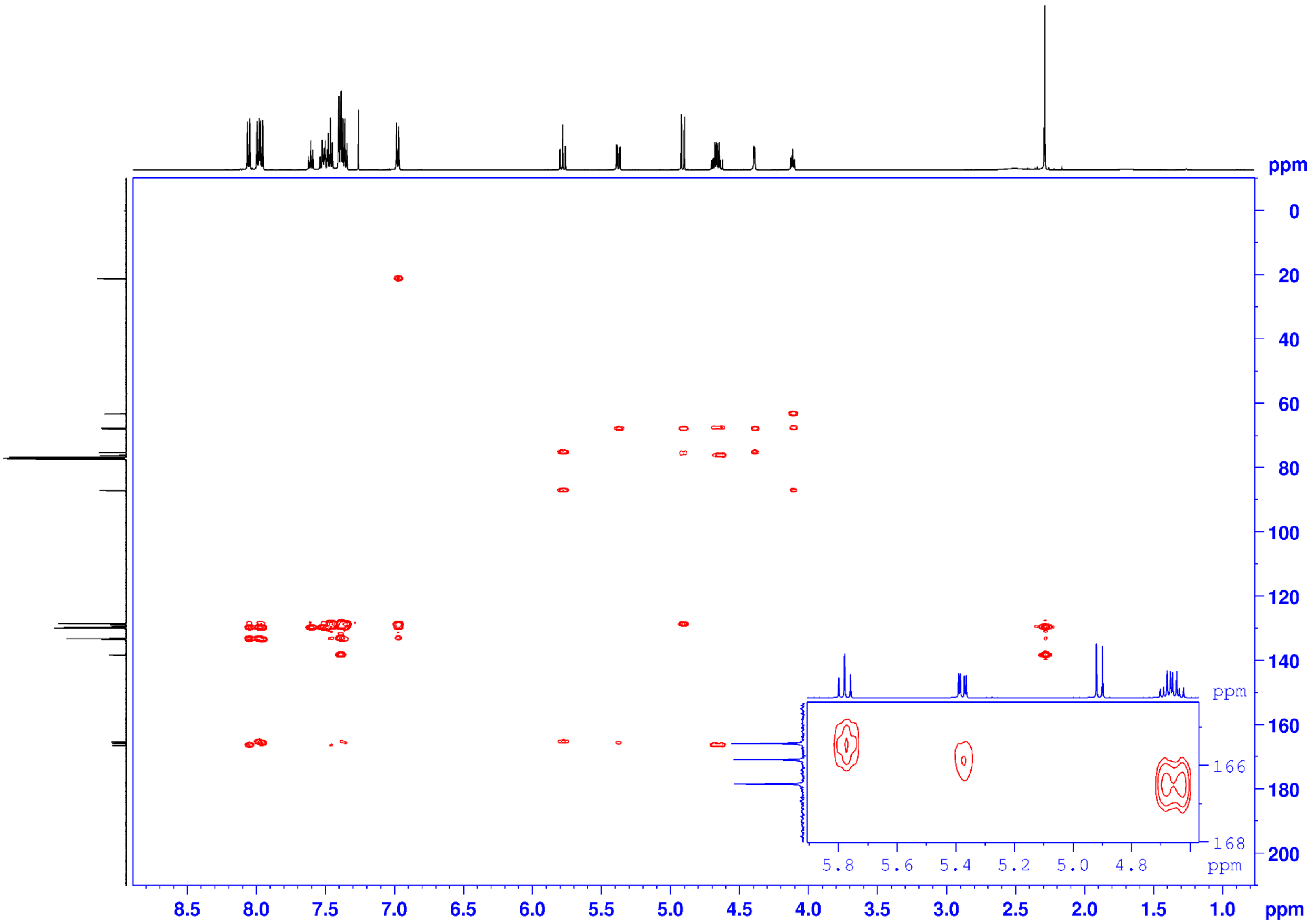
ppm
0
20
40
60
80
100
120
140
160
180
200
8.5
8.0
7.5
7.0
6.5
6.0
5.5
5.0
4.5
4.0
3.5
3.0
2.5
2.0
1.5
1.0
ppm
166
168
5.8
5.6
5.4
5.2
5.0
4.8

$^{13}C\{^1H\}$ NMR

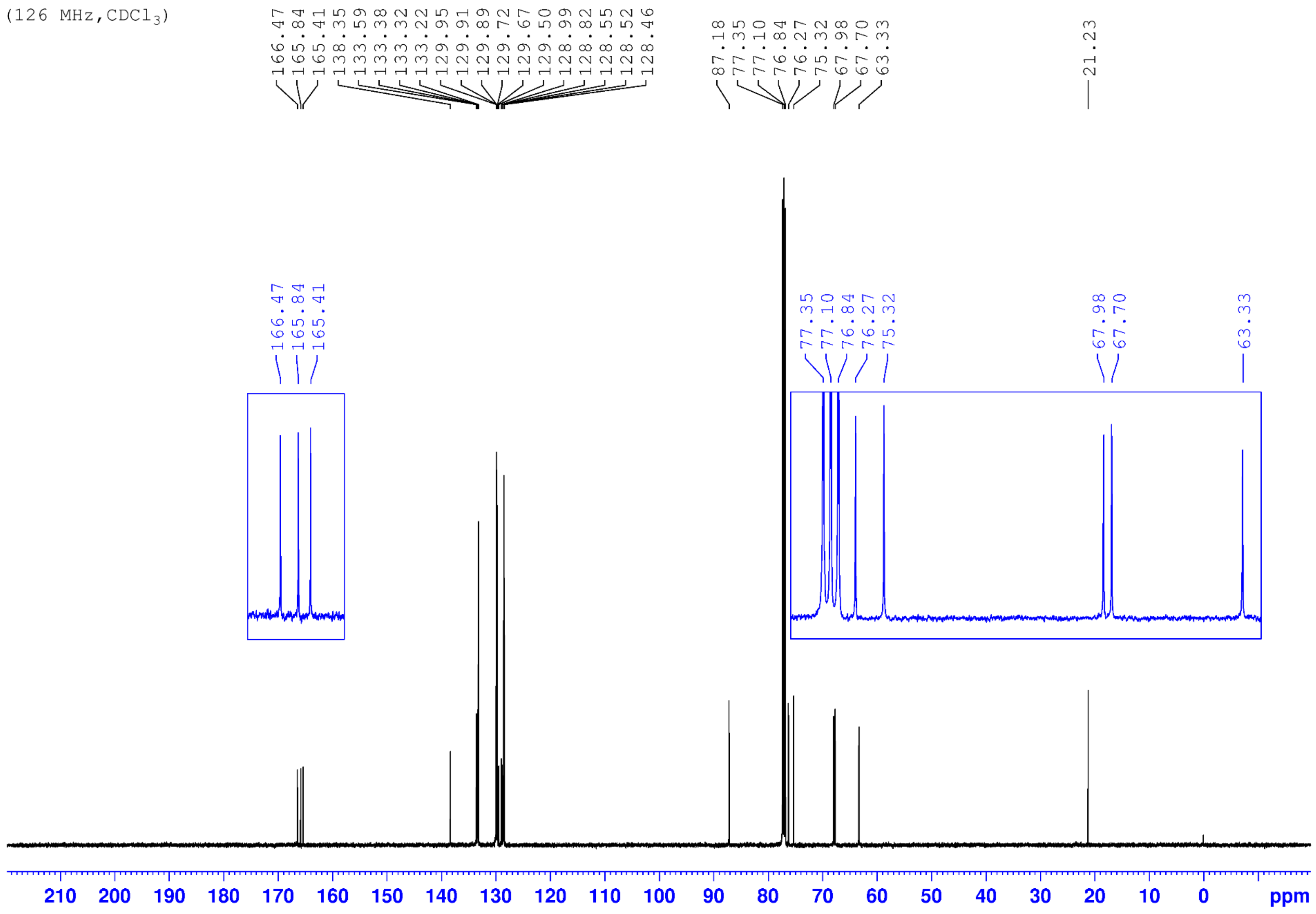
(126 MHz,CDCl3)
166.47
165.84
165.41
138.35
133.59
133.38
133.32
133.22
129.95
129.91
129.89
129.72
129.67
129.50
128.99
128.82
128.55
128.52
128.46
87.18
77.35
77.10
76.84
76.27
75.32
67.98
67.70
63.33
21.23
210 200 190 180 170 160 150 140 130 120 110 100 90 80 70 60 50 40 30 20 10 0 ppm

Compound **S4b**

^{1}H-NMR

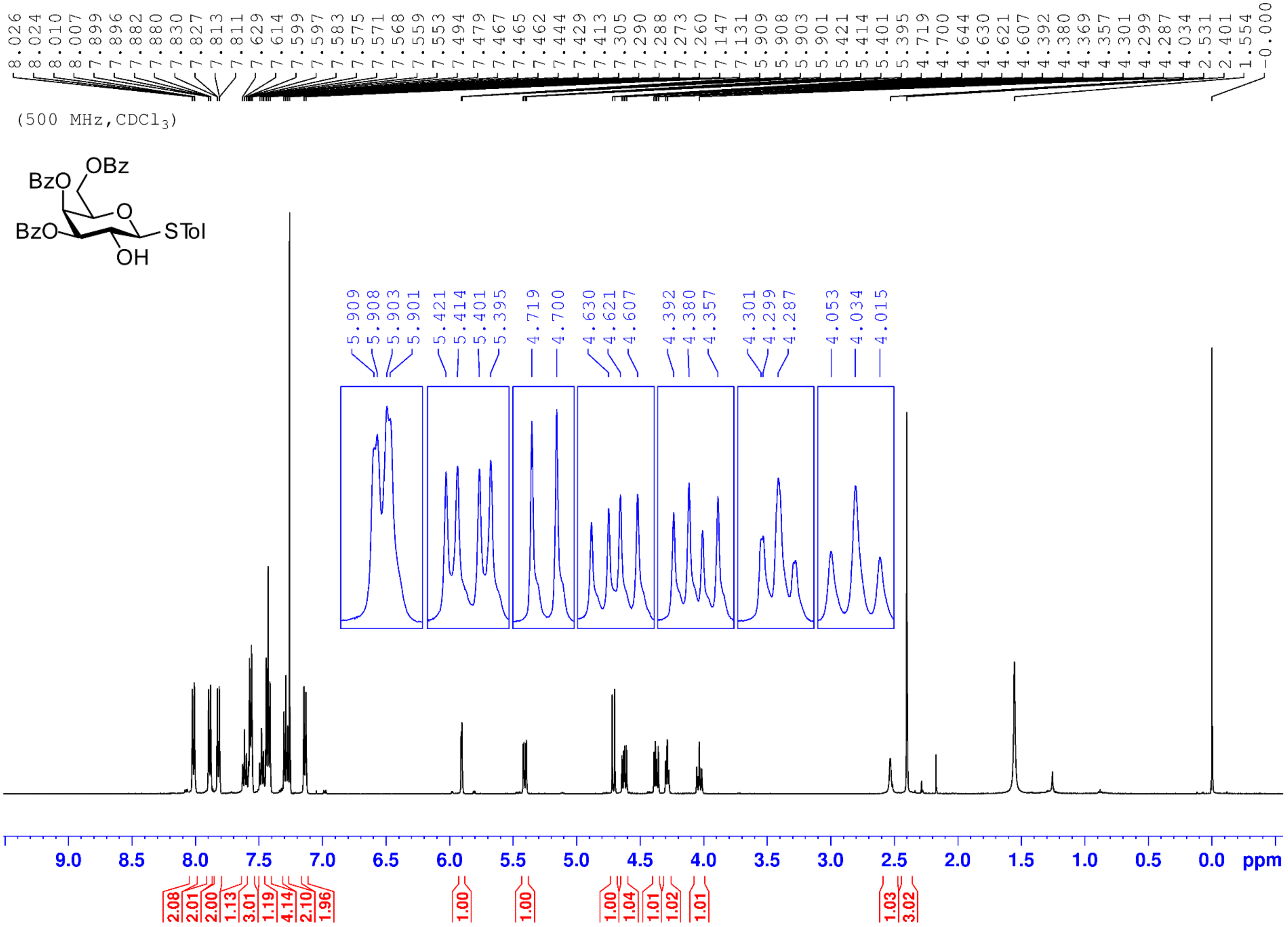

^{1}H-^{1}H COSY

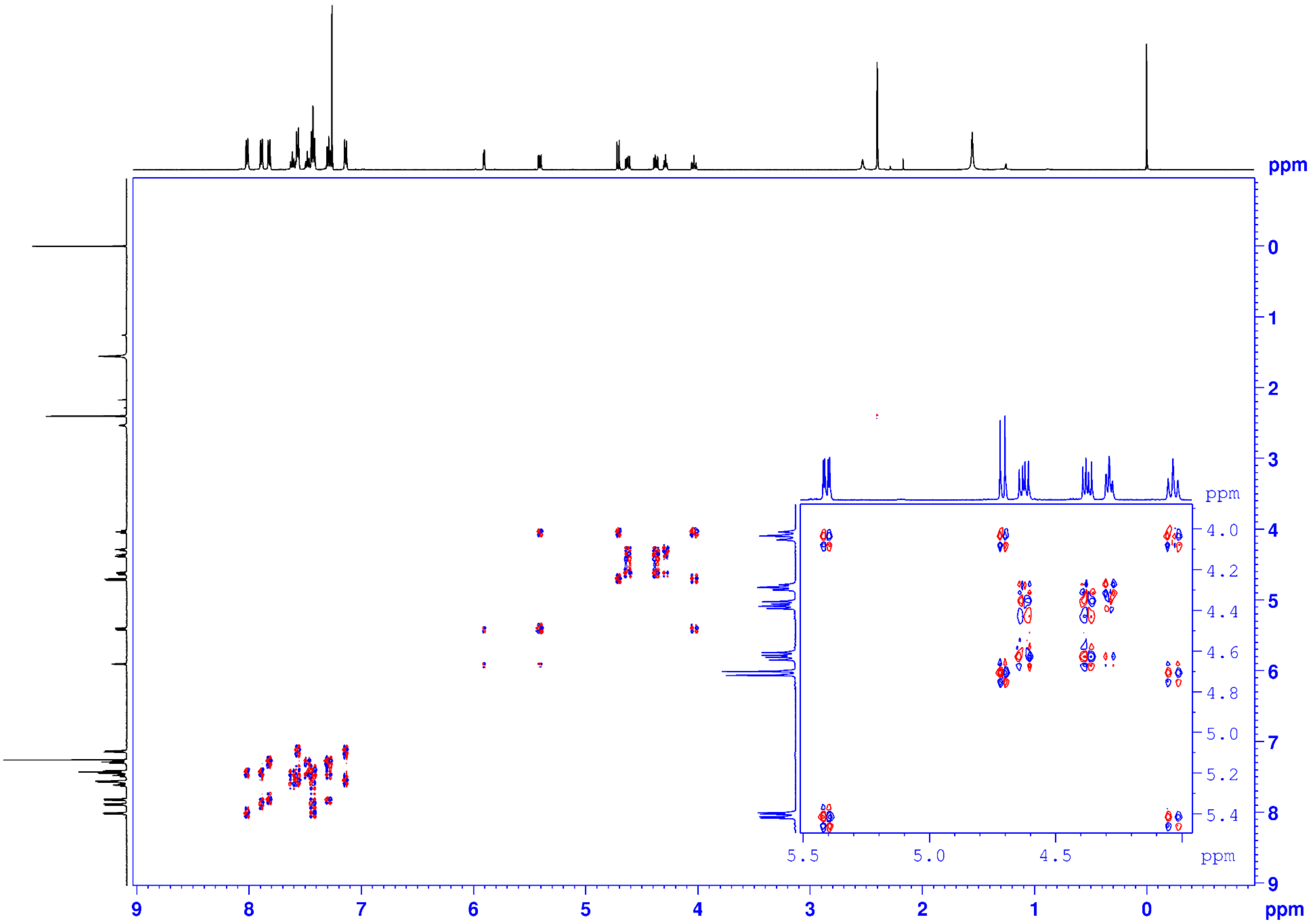

^{1}H-^{13}C HSQC

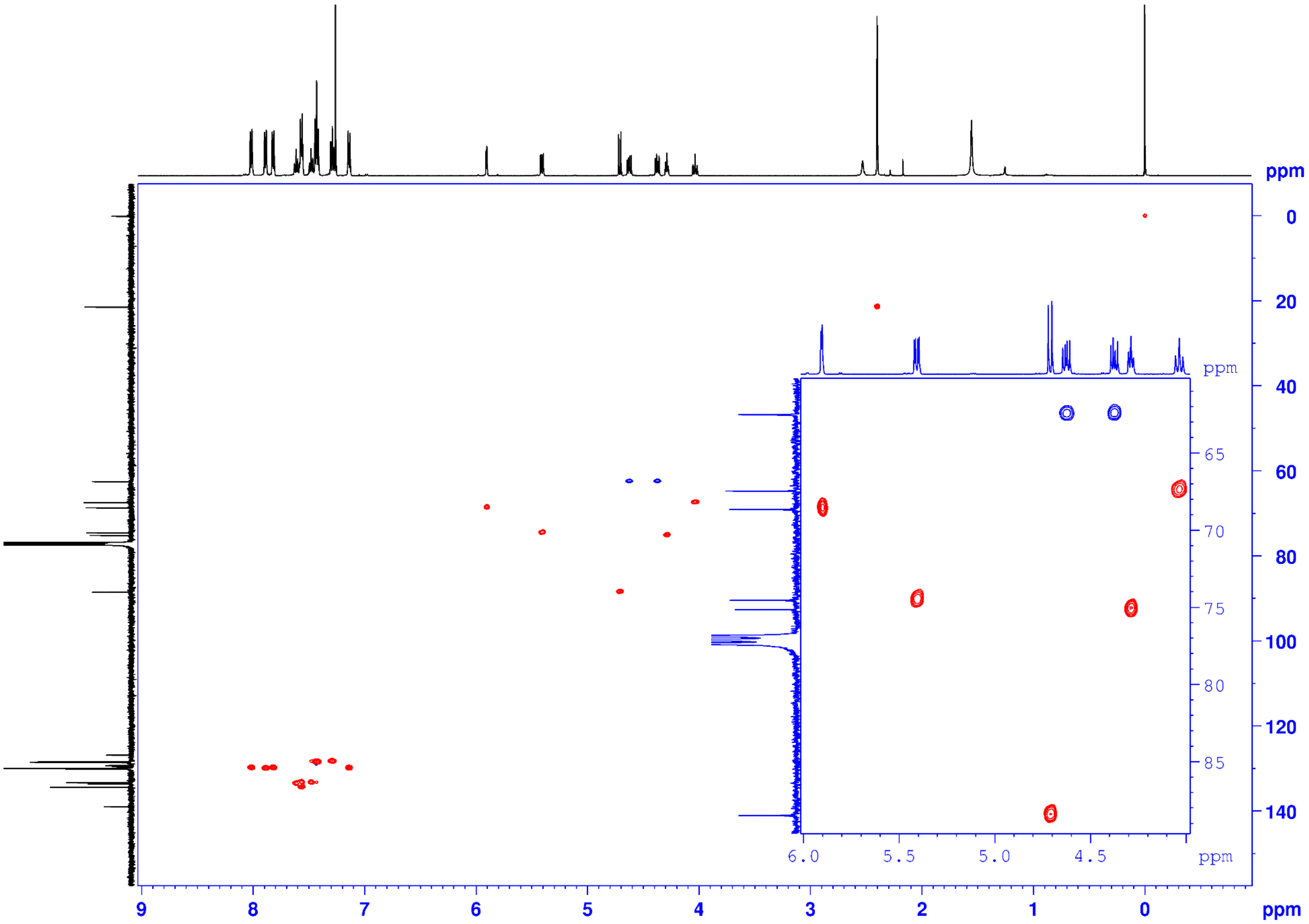

ppm
0
20
40
60
80
100
120
140
9
8
7
6
5
4
3
2
1
0
ppm
65
70
75
80
85
ppm
6.0
5.5
5.0
4.5
ppm

$^{1}H-^{13}C$ HMBC

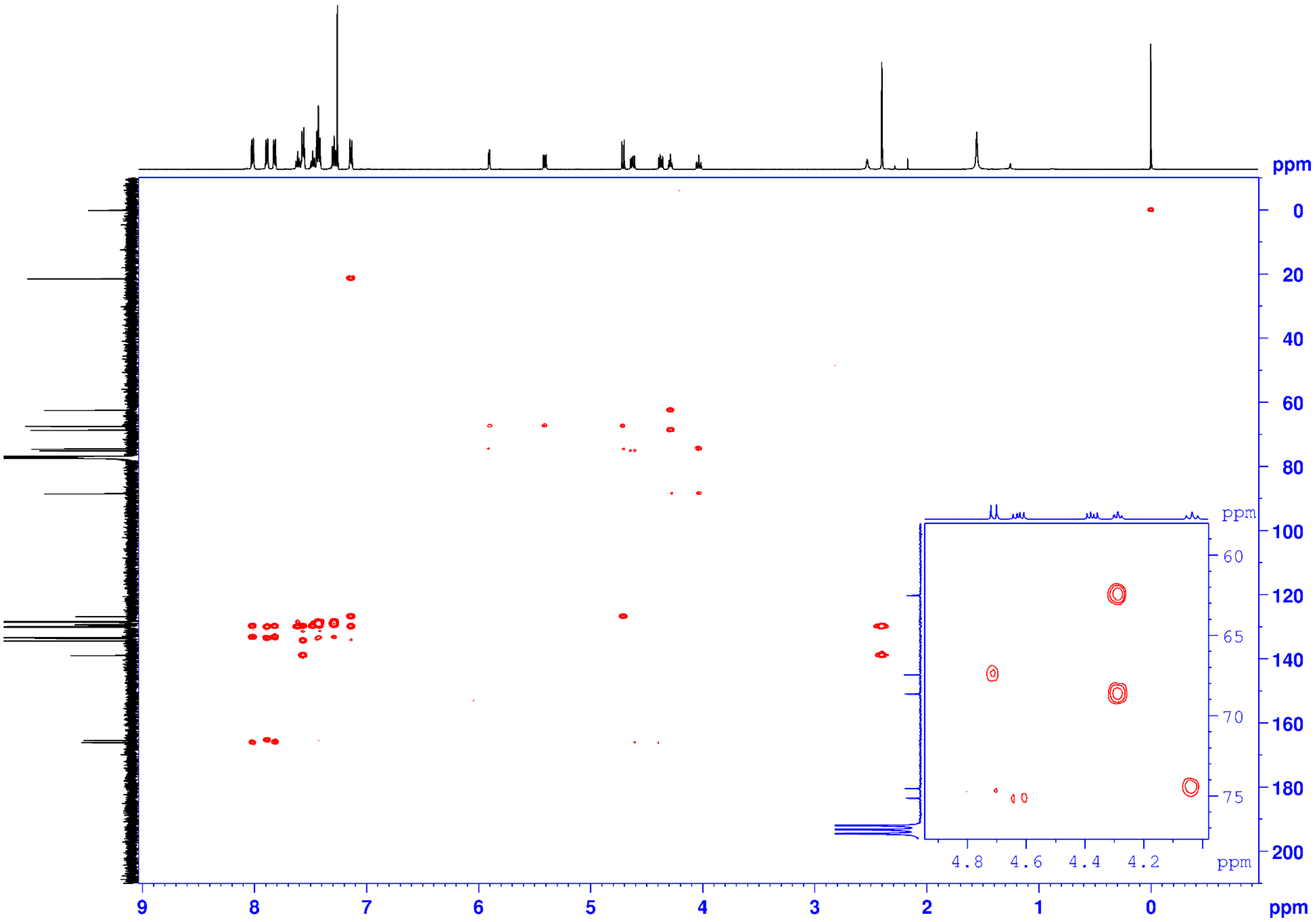

ppm
0
20
40
60
80
100
120
140
160
180
200
9
8
7
6
5
4
3
2
1
0
ppm
60
65
70
75
4.8
4.6
4.4
4.2
ppm

$^{13}C\{^{1}H\}$ NMR

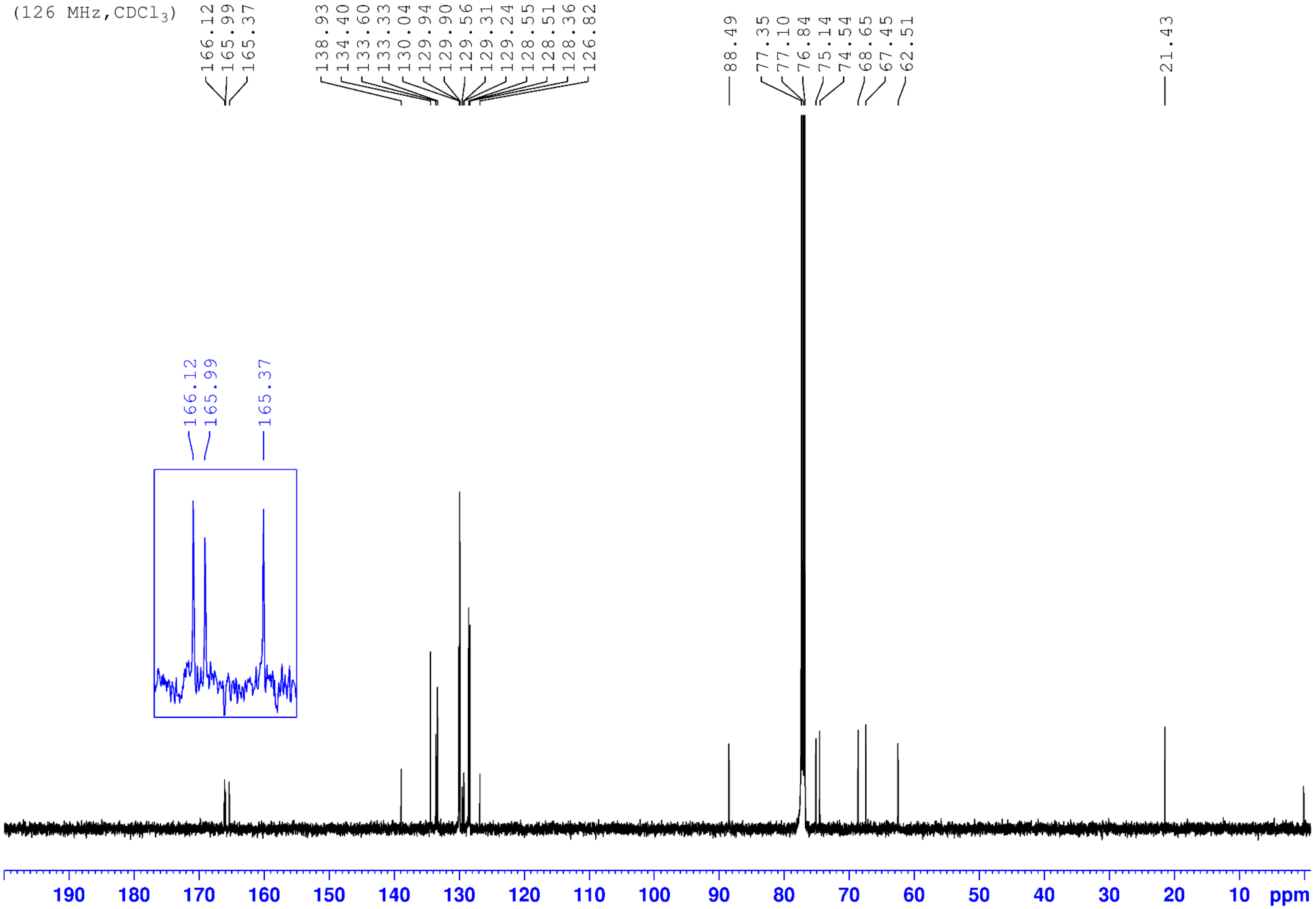

(126 MHz, CDCl3)
166.12
165.99
165.37
138.93
134.40
133.60
133.33
130.04
129.94
129.90
129.56
129.31
129.24
128.55
128.51
128.36
126.82
88.49
77.35
77.10
76.84
75.14
74.54
68.65
67.45
62.51
21.43
166.12
165.99
165.37
190
180
170
160
150
140
130
120
110
100
90
80
70
60
50
40
30
20
10
ppm

Compound **S5**

^{1}H-NMR

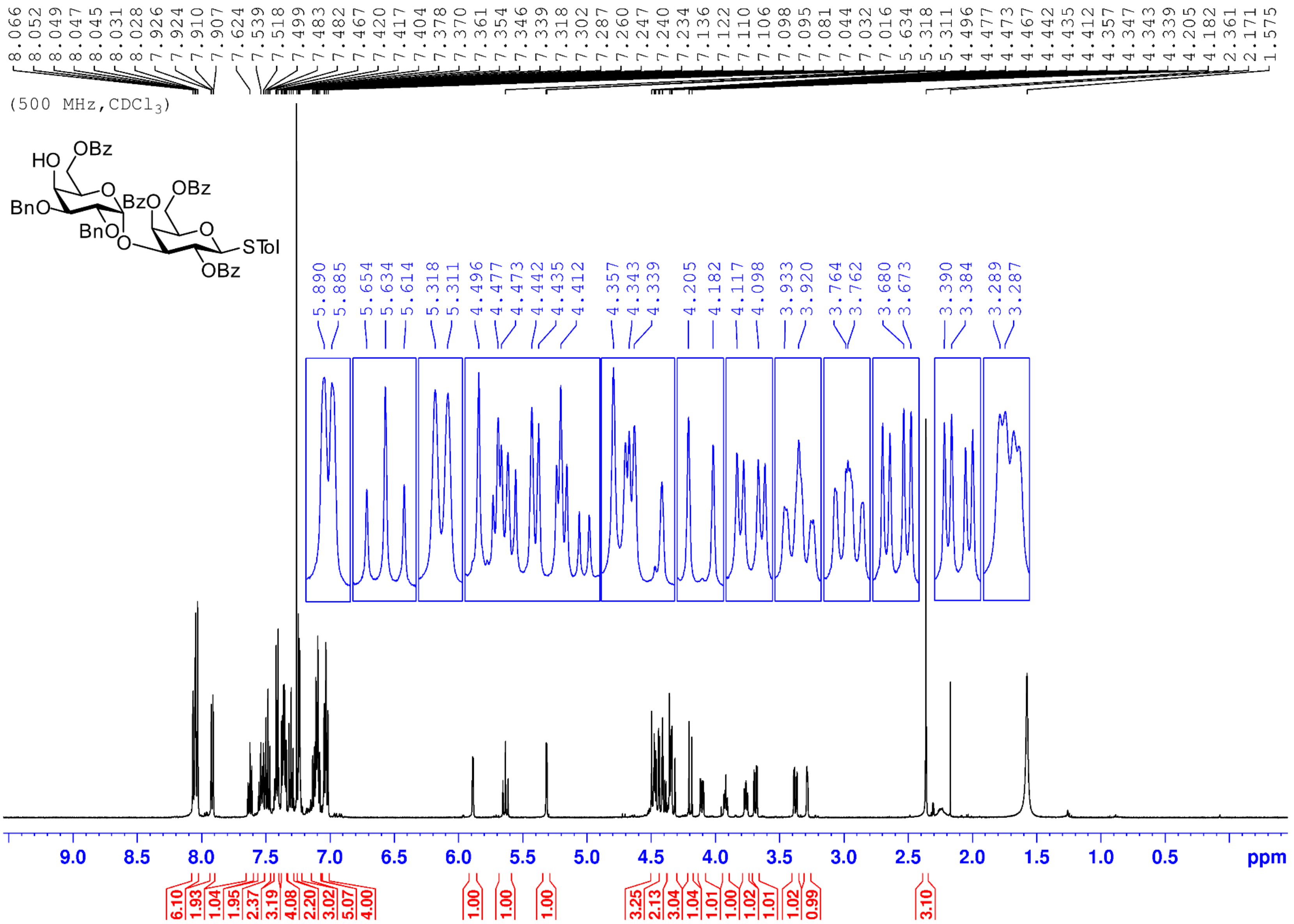

^1H-^1H COSY

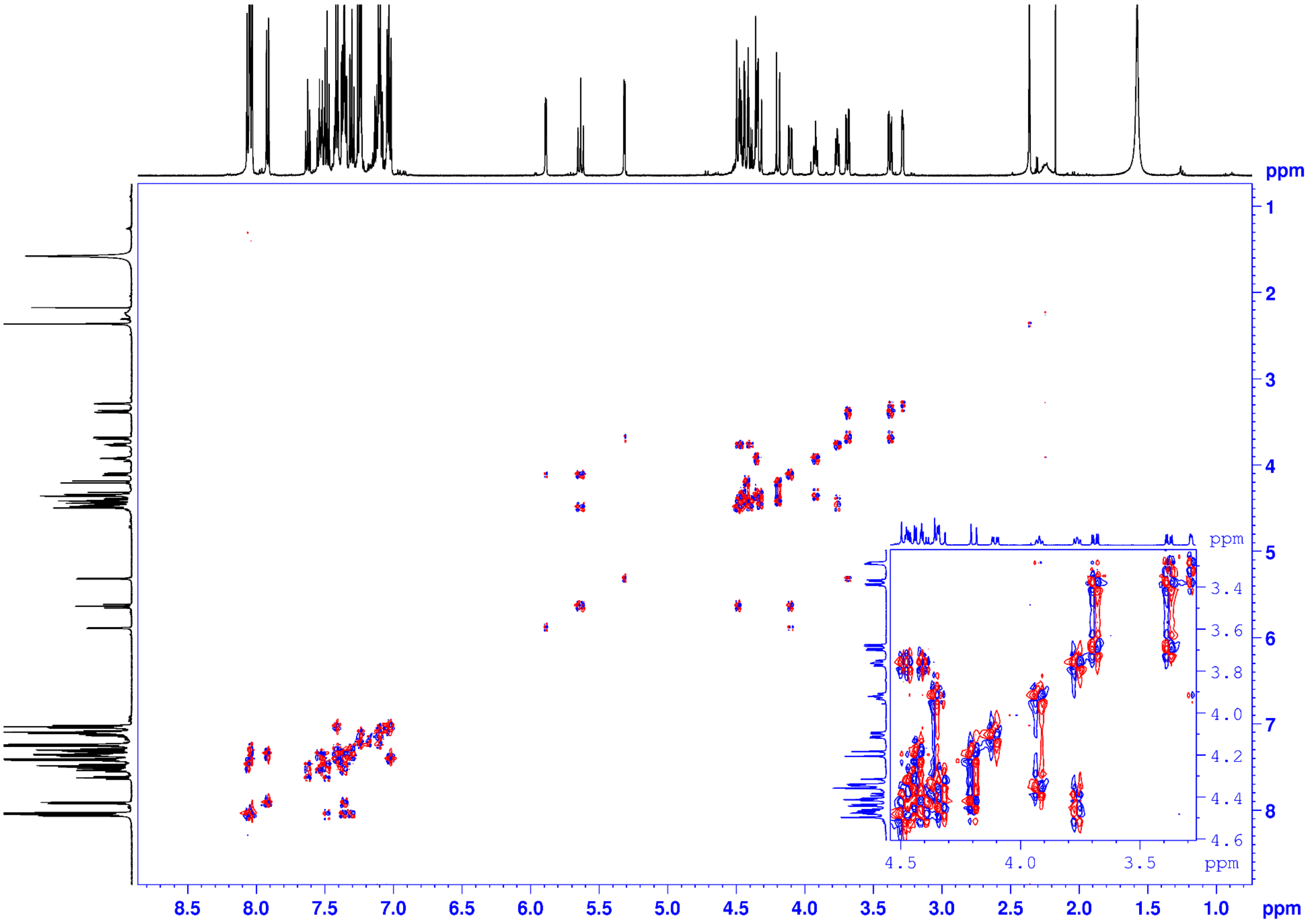
ppm
1
2
3
4
5
6
7
8
8.5
8.0
7.5
7.0
6.5
6.0
5.5
5.0
4.5
4.0
3.5
3.0
2.5
2.0
1.5
1.0
ppm
3.4
3.6
3.8
4.0
4.2
4.4
4.6
4.5
4.0
3.5
ppm

^{1}H-^{13}C HSQC

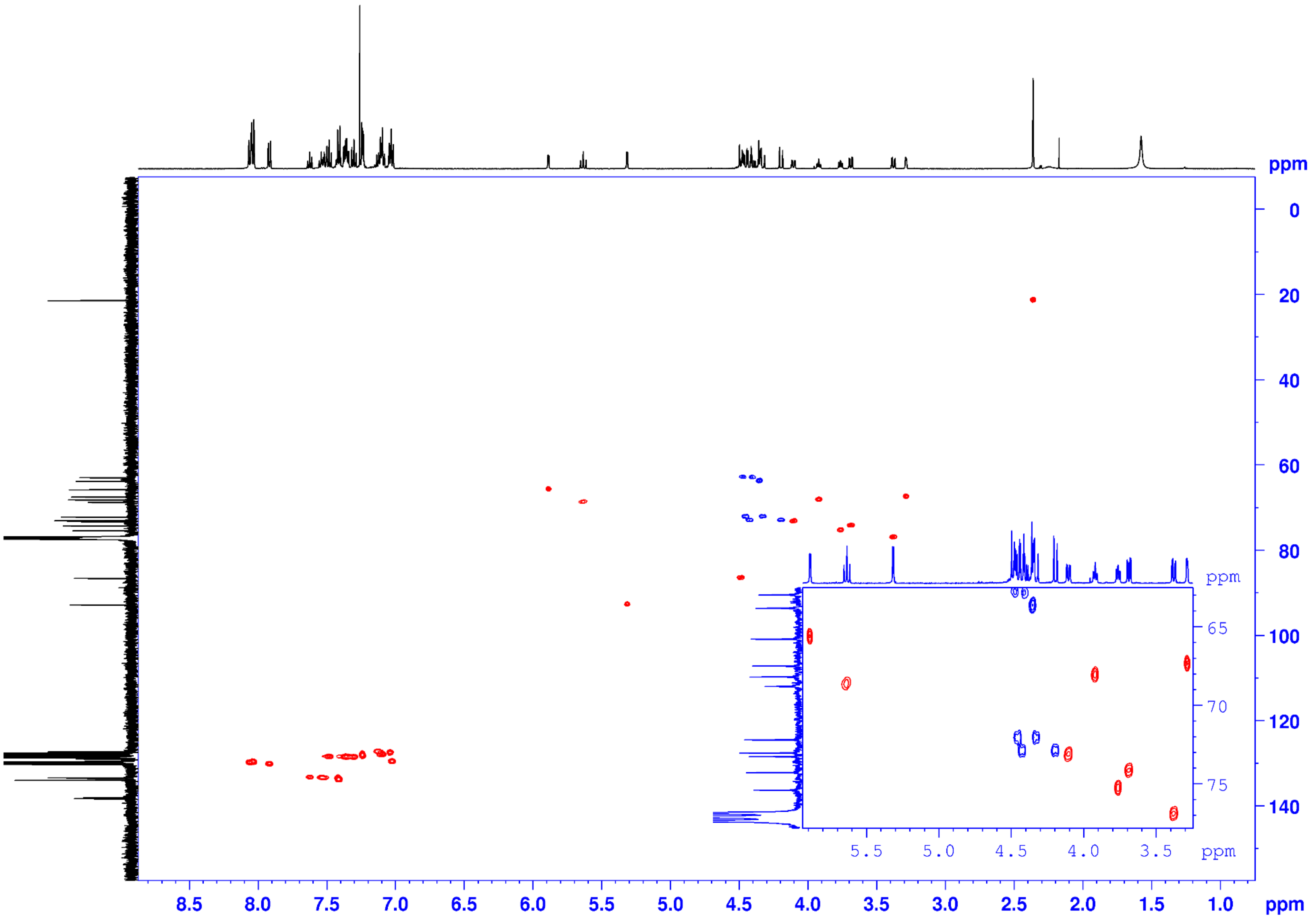

ppm
0
20
40
60
80
100
120
140
8.5
8.0
7.5
7.0
6.5
6.0
5.5
5.0
4.5
4.0
3.5
3.0
2.5
2.0
1.5
1.0
ppm
65
70
75
5.5
5.0
4.5
4.0
3.5

^{1}H-^{13}C HMBC

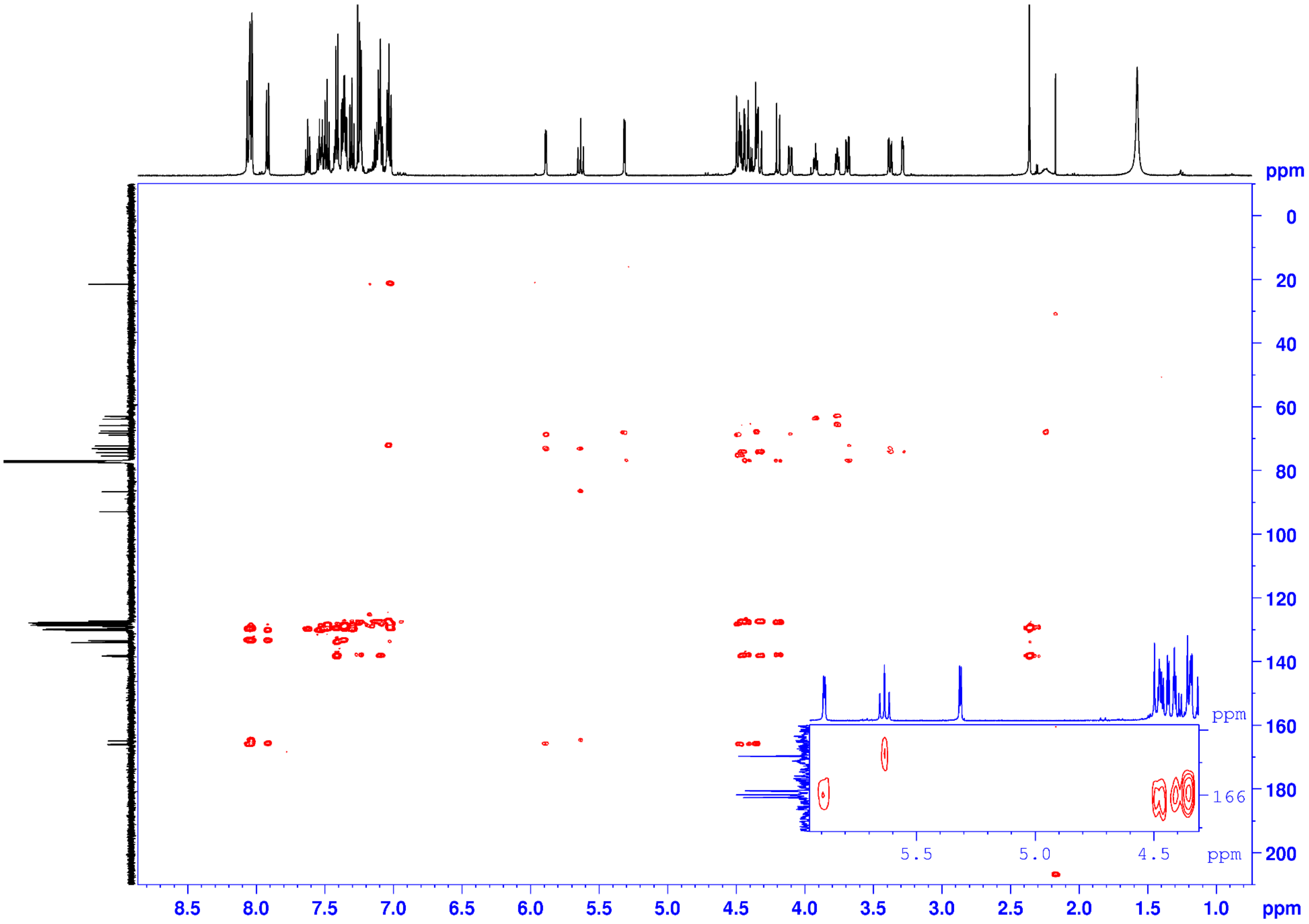

ppm
0
20
40
60
80
100
120
140
160
180
200
8.5
8.0
7.5
7.0
6.5
6.0
5.5
5.0
4.5
4.0
3.5
3.0
2.5
2.0
1.5
1.0
ppm
5.5
5.0
4.5
ppm
166

$^{13}C\{^1H\}$ NMR

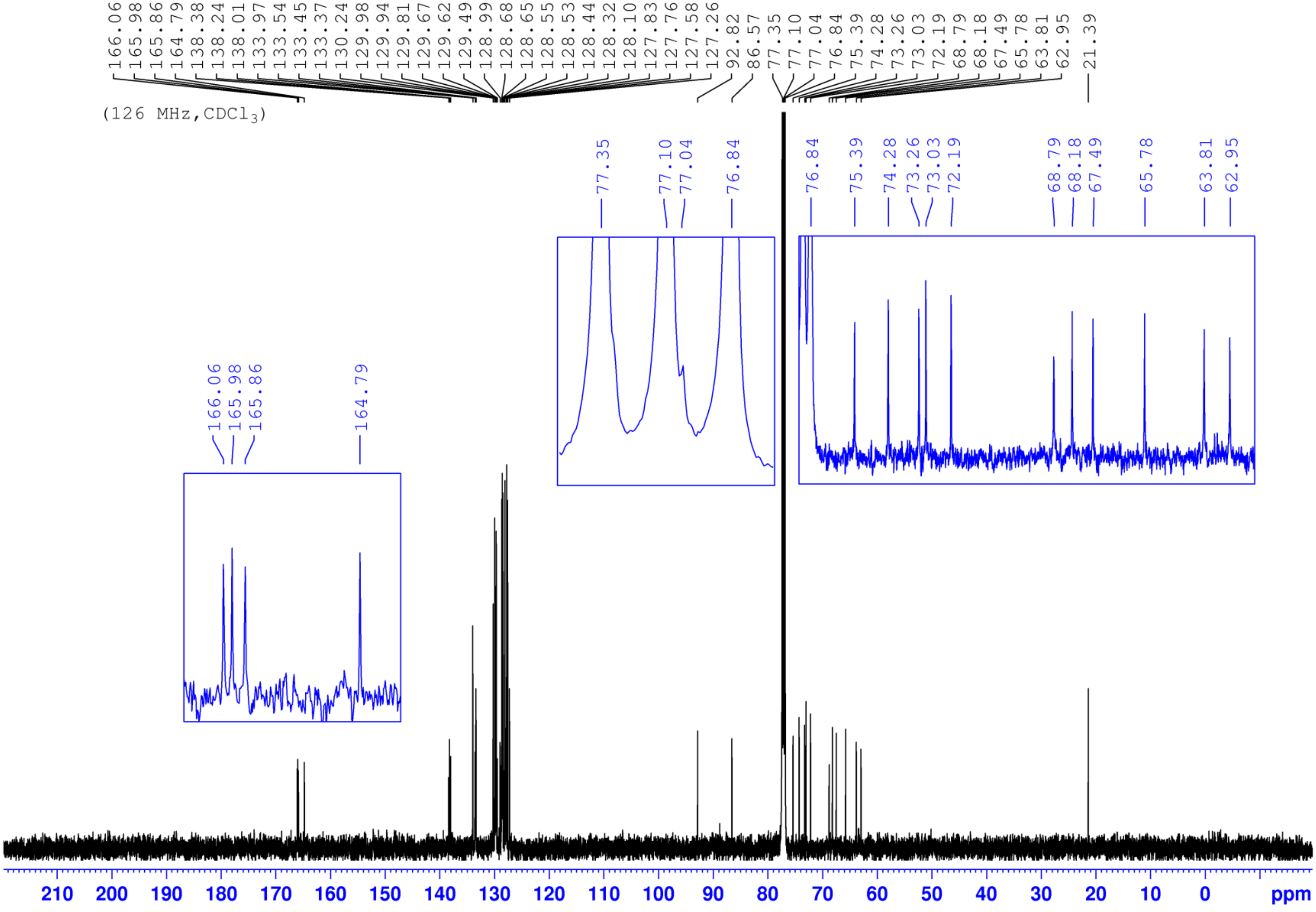
(126 MHz, CDCl3)
166.06
165.98
165.86
164.79
138.38
138.24
138.01
133.97
133.54
133.45
133.37
130.24
129.98
129.94
129.81
129.67
129.62
129.49
128.99
128.68
128.65
128.55
128.53
128.44
128.32
128.10
127.83
127.76
127.58
127.26
92.82
86.57
77.35
77.10
77.04
76.84
75.39
74.28
73.26
73.03
72.19
68.79
68.18
67.49
65.78
63.81
62.95
21.39
ppm

Compound **S6**

^{1}H-NMR

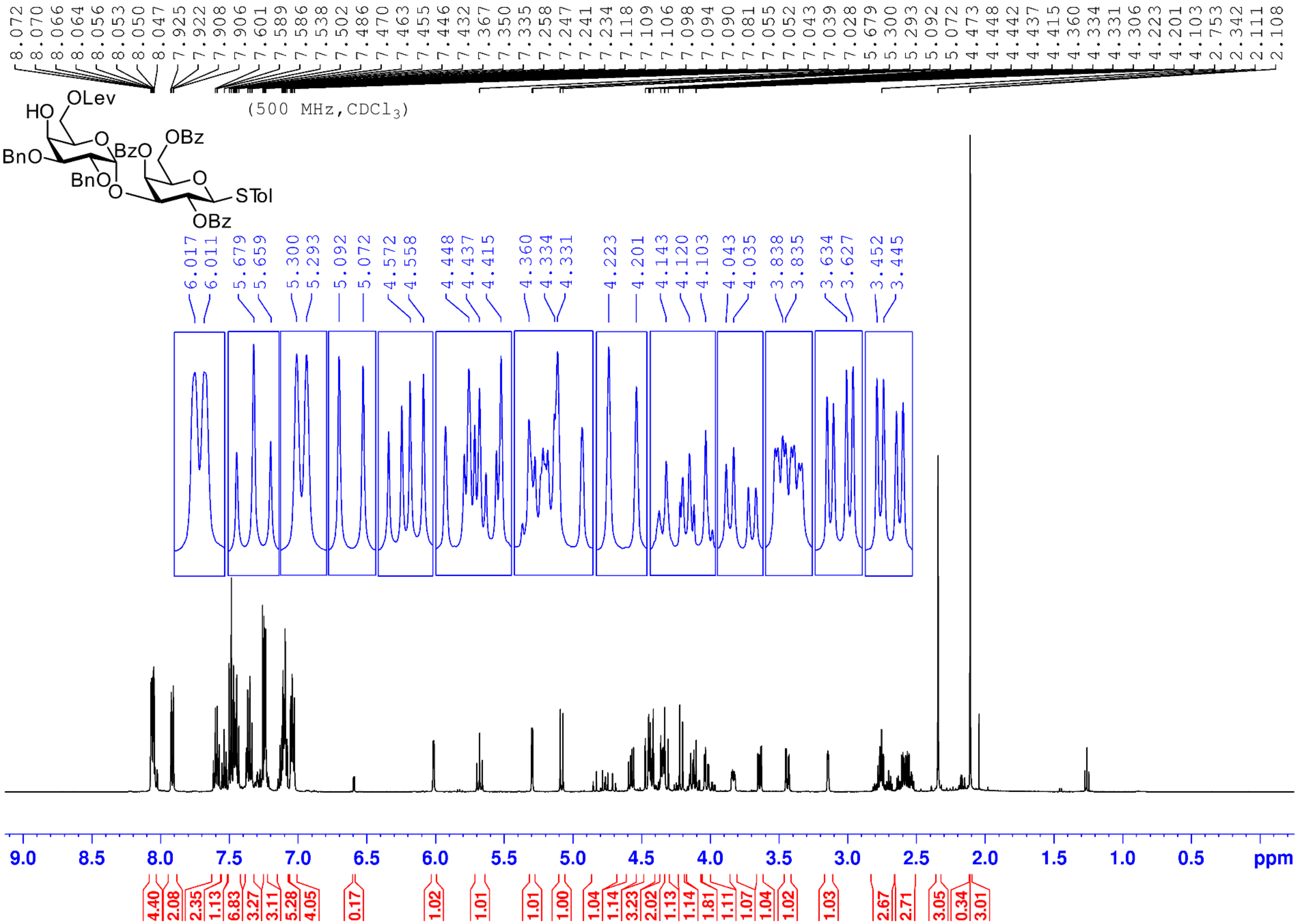

^{1}H-^{1}H COSY

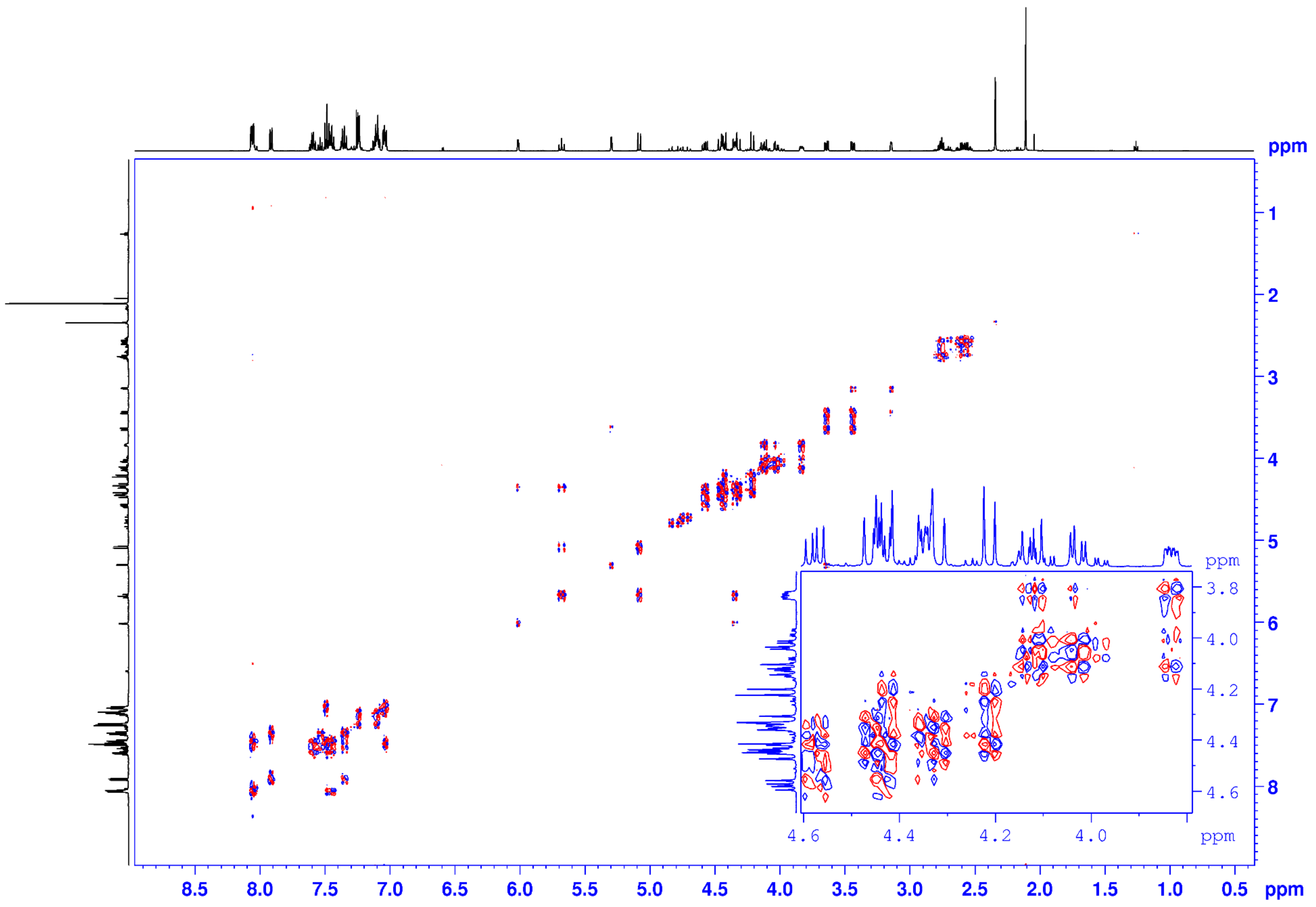
ppm
1
2
3
4
5
6
7
8
8.5
8.0
7.5
7.0
6.5
6.0
5.5
5.0
4.5
4.0
3.5
3.0
2.5
2.0
1.5
1.0
0.5
ppm
3.8
4.0
4.2
4.4
4.6
4.6
4.4
4.2
4.0
ppm

^{1}H-^{13}C HSQC

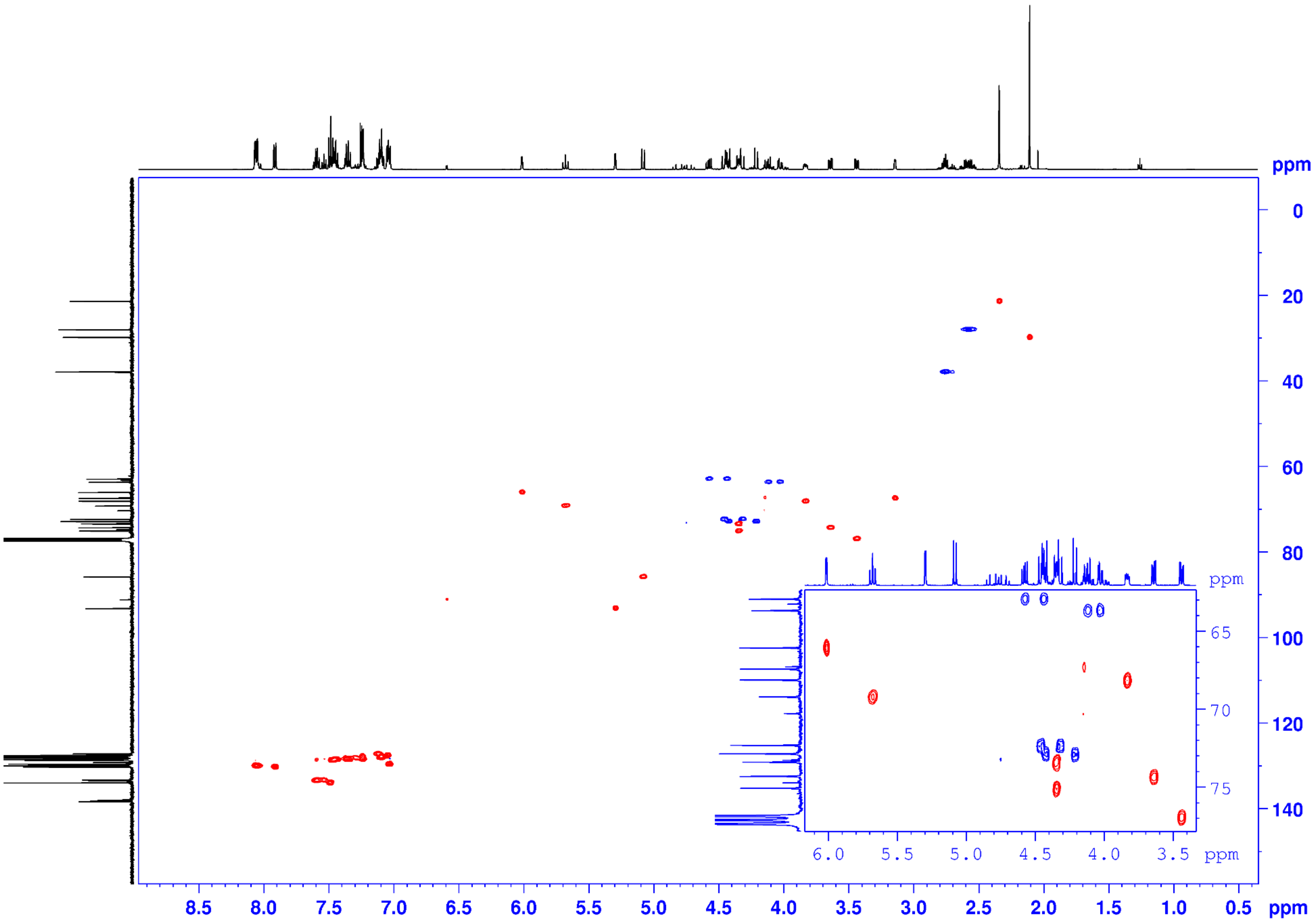
ppm
0
20
40
60
80
100
120
140
8.5
8.0
7.5
7.0
6.5
6.0
5.5
5.0
4.5
4.0
3.5
3.0
2.5
2.0
1.5
1.0
0.5
ppm
65
70
75
6.0
5.5
5.0
4.5
4.0
3.5
ppm

^{1}H-^{13}C HSQC

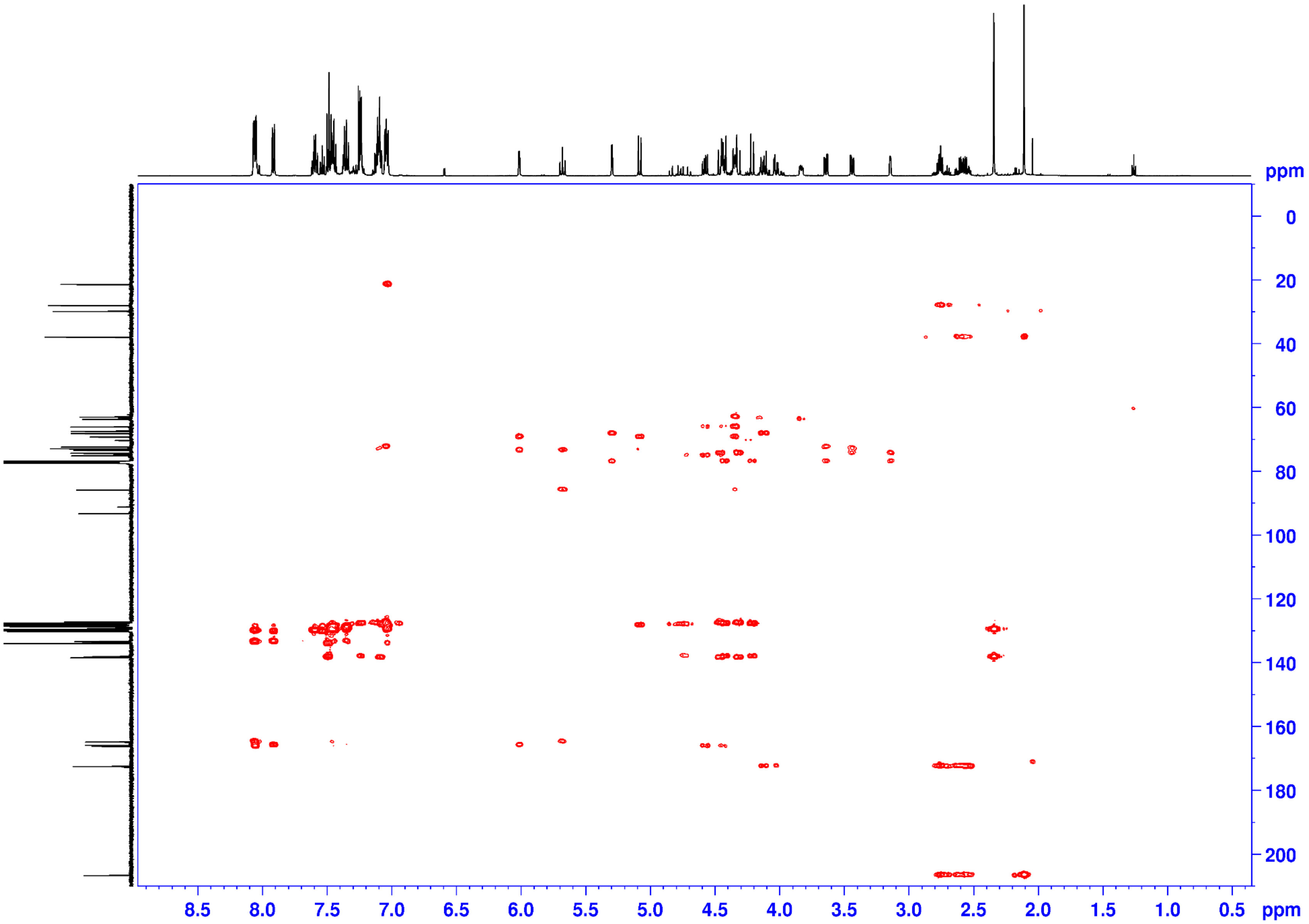

ppm
0
20
40
60
80
100
120
140
160
180
200
8.5
8.0
7.5
7.0
6.5
6.0
5.5
5.0
4.5
4.0
3.5
3.0
2.5
2.0
1.5
1.0
0.5
ppm

$^{13}C\{^{1}H\}$ NMR

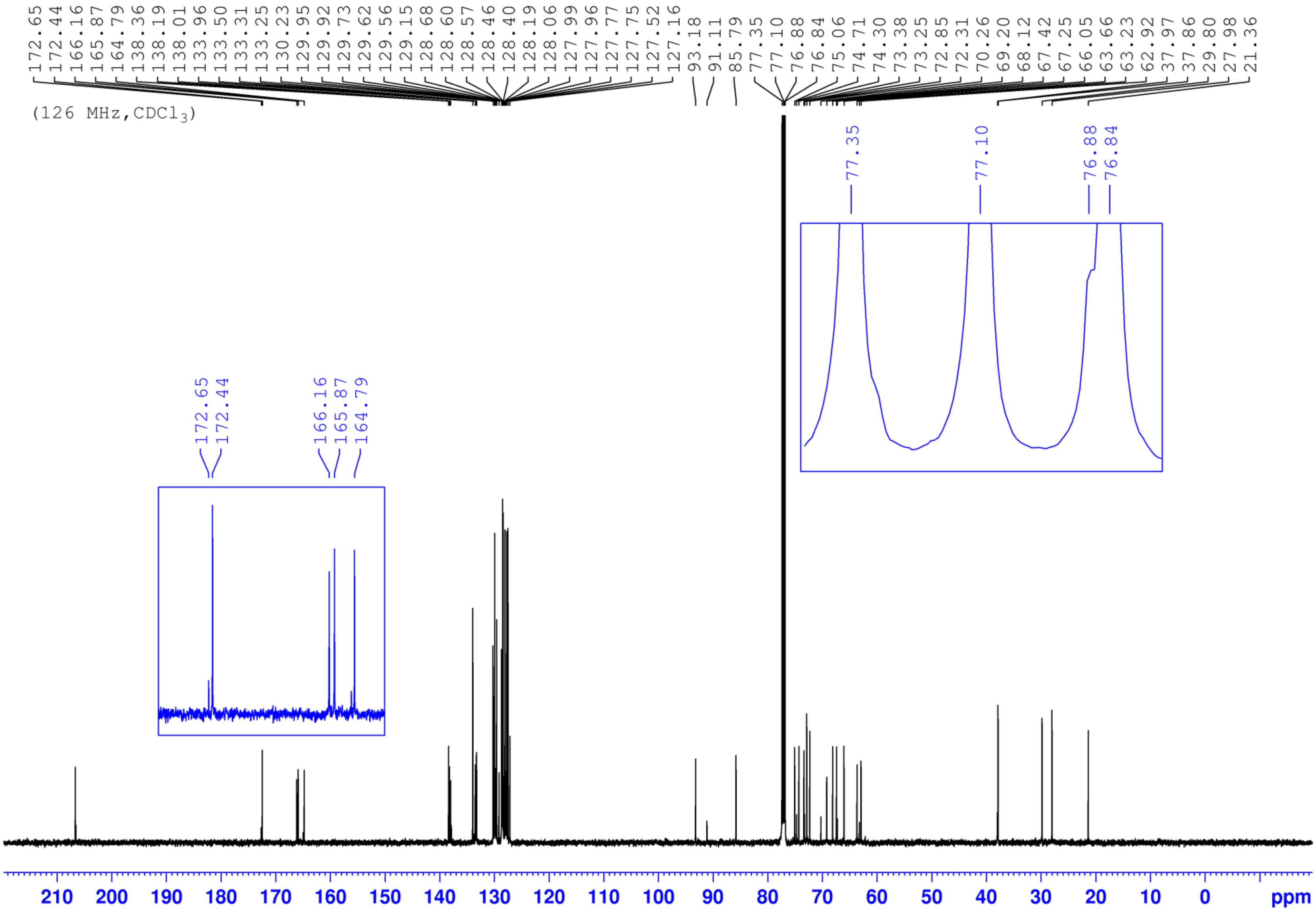
172.65
172.44
166.16
165.87
164.79
138.36
138.19
138.01
133.96
133.50
133.31
133.25
130.23
129.95
129.92
129.73
129.62
129.56
129.15
128.68
128.60
128.57
128.46
128.40
128.19
128.06
127.99
127.96
127.77
127.75
127.52
127.16
93.18
91.11
85.79
77.35
77.10
76.88
76.84
75.06
74.71
74.30
73.38
73.25
72.85
72.31
70.26
69.20
68.12
67.42
67.25
66.05
63.66
63.23
62.92
37.97
37.86
29.80
27.98
21.36
(126 MHz, CDCl3)
77.35
77.10
76.88
76.84
172.65
172.44
166.16
165.87
164.79
210
200
190
180
170
160
150
140
130
120
110
100
90
80
70
60
50
40
30
20
10
0
ppm

Compound **S7**

^{1}H-NMR

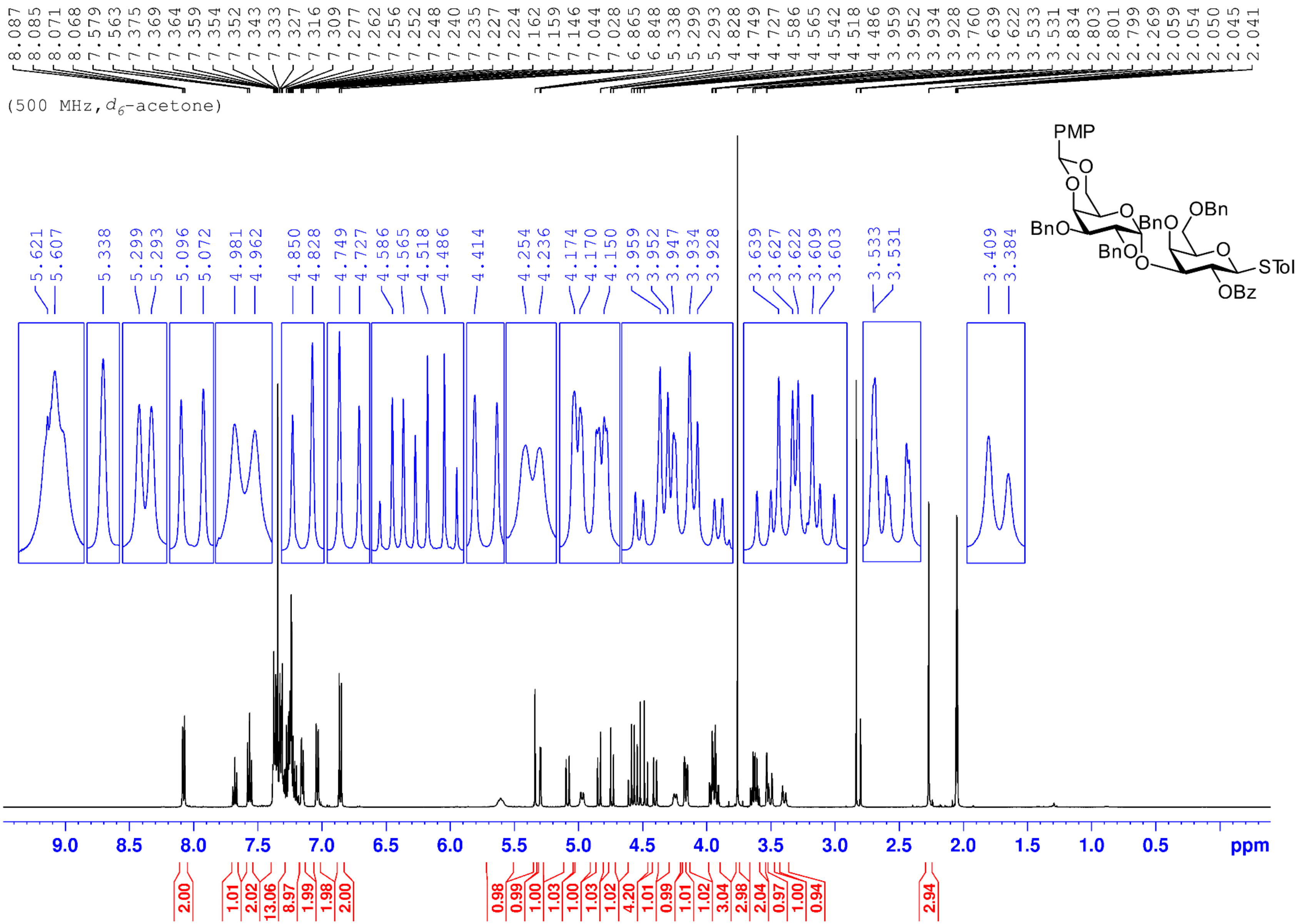

^{1}H-^{1}H COSY

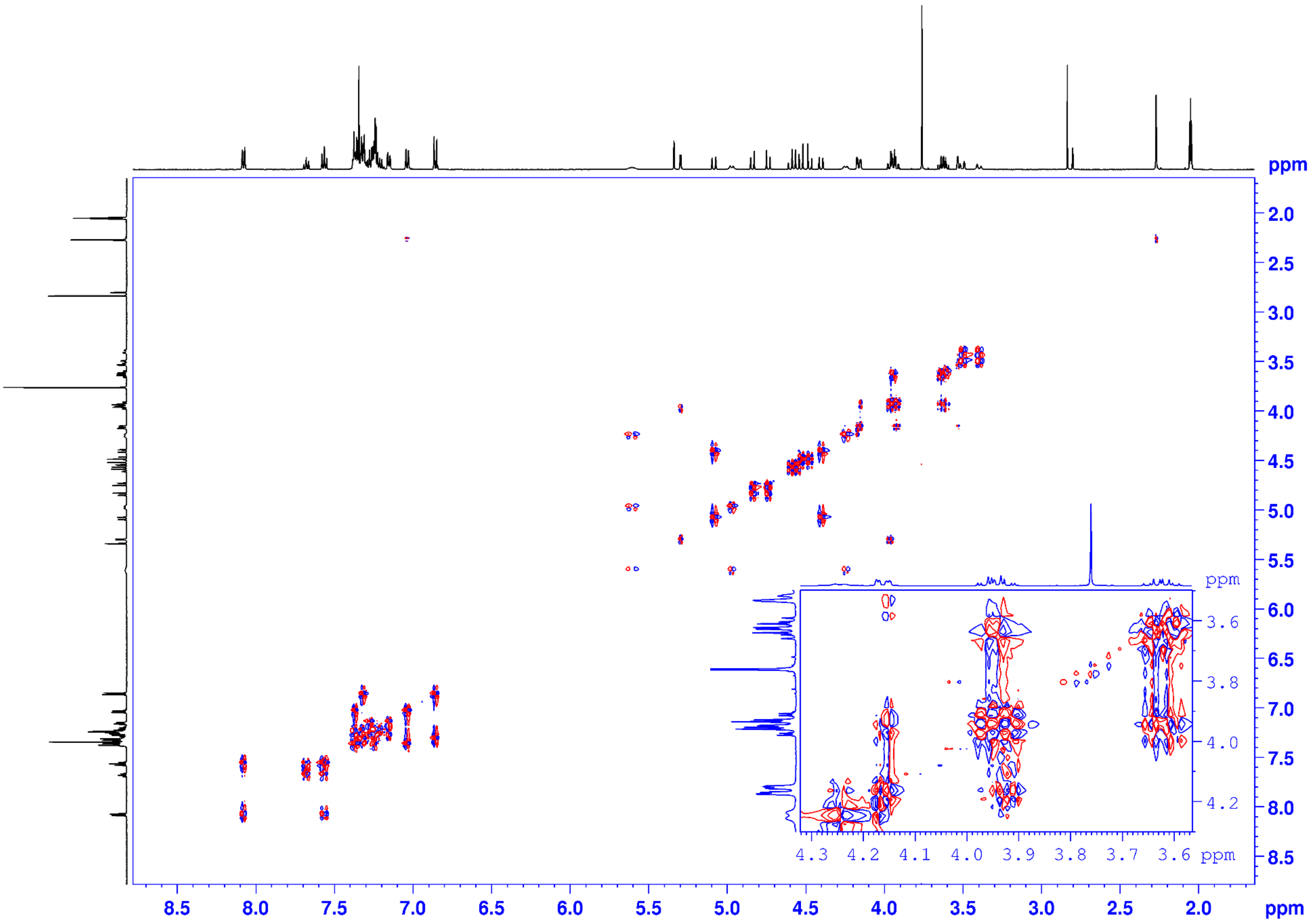
ppm
2.0
2.5
3.0
3.5
4.0
4.5
5.0
5.5
6.0
6.5
7.0
7.5
8.0
8.5
8.5 8.0 7.5 7.0 6.5 6.0 5.5 5.0 4.5 4.0 3.5 3.0 2.5 2.0 ppm
3.6
3.8
4.0
4.2
4.3 4.2 4.1 4.0 3.9 3.8 3.7 3.6 ppm

^{1}H-^{13}C HSQC

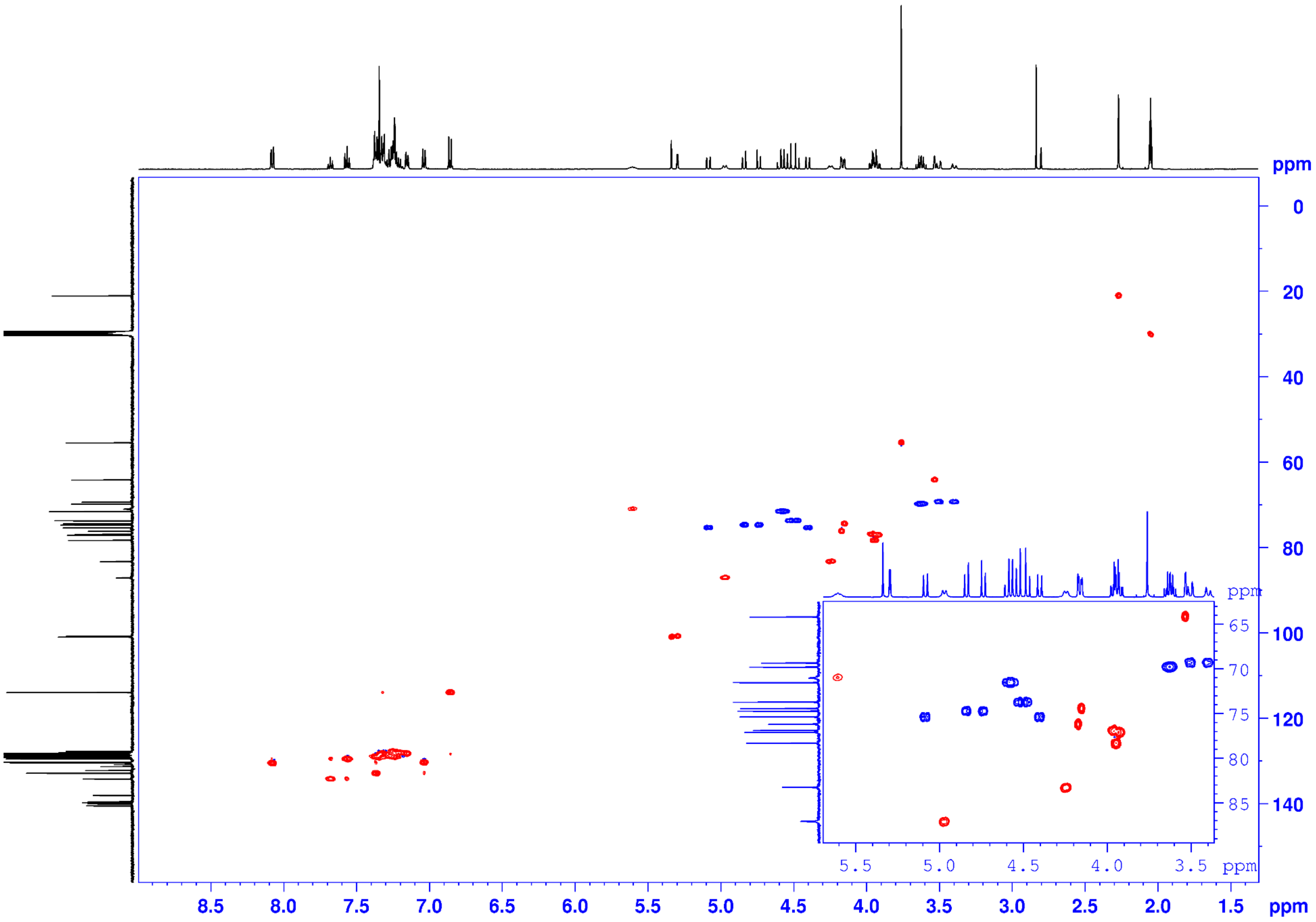

ppm
0
20
40
60
80
100
120
140
8.5
8.0
7.5
7.0
6.5
6.0
5.5
5.0
4.5
4.0
3.5
3.0
2.5
2.0
1.5
ppm
65
70
75
80
85
5.5
5.0
4.5
4.0
3.5
ppm

^{1}H-^{13}C non-decoupled HSQC

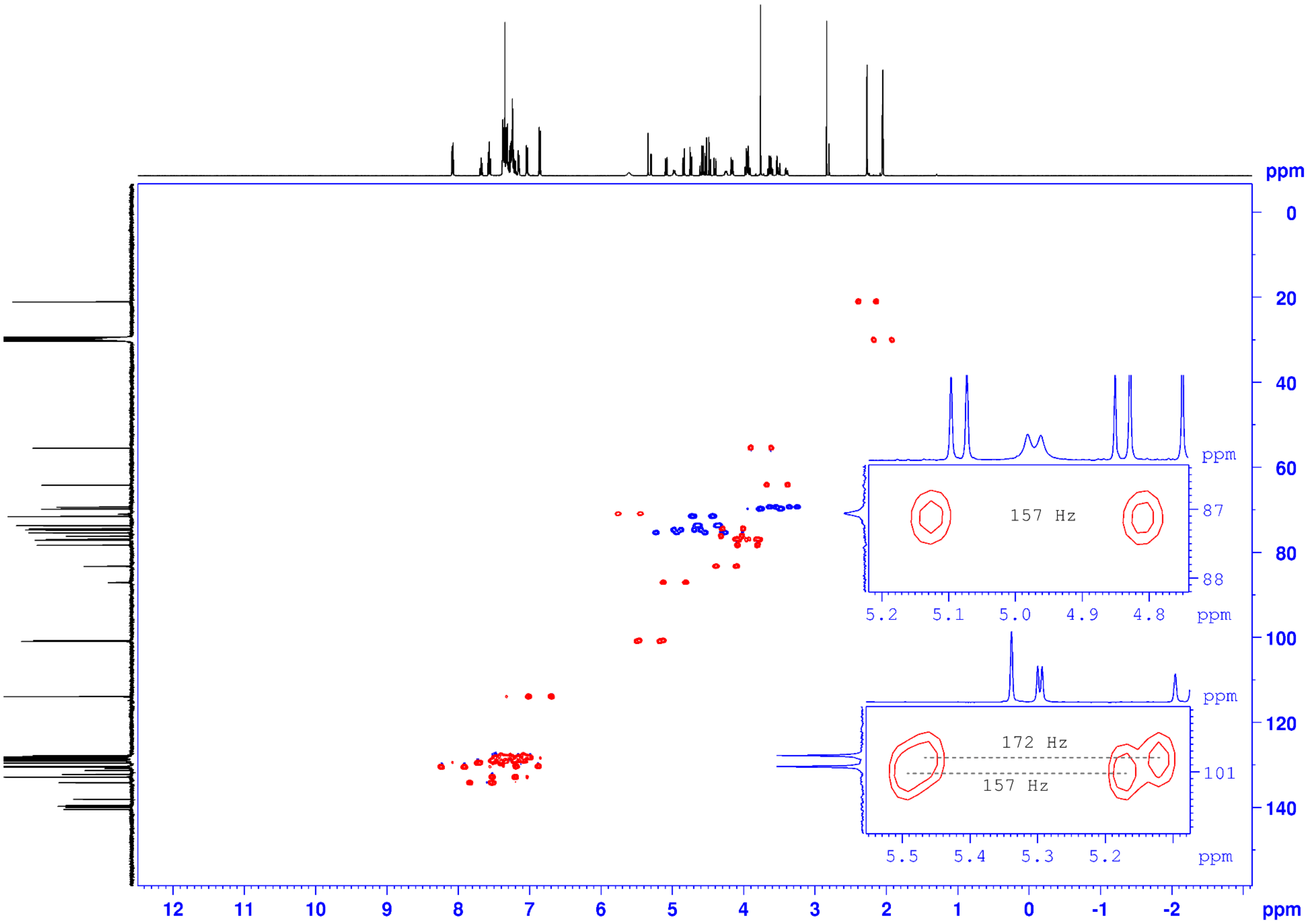

^{1}H-^{13}C HMBC

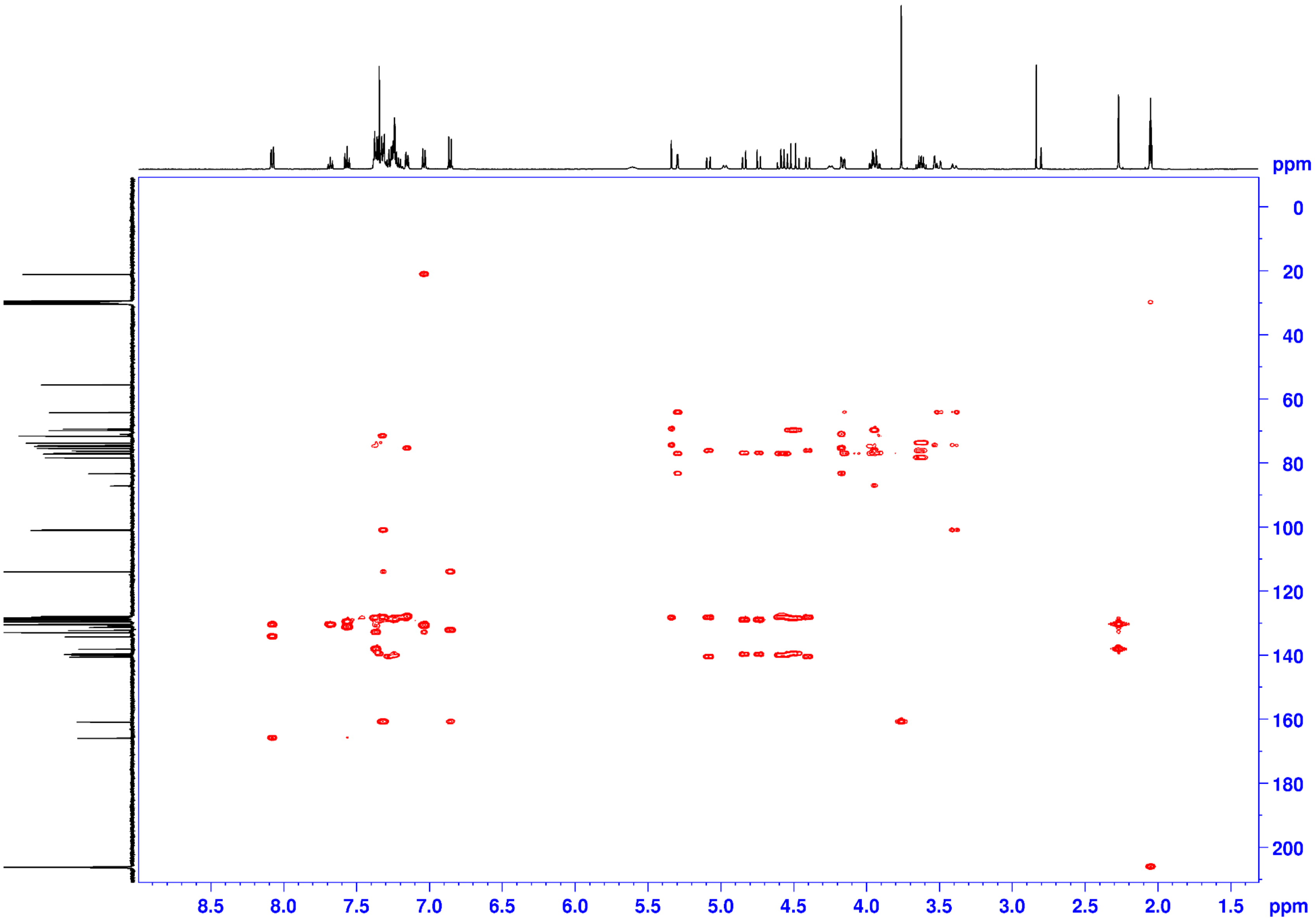

ppm
0
20
40
60
80
100
120
140
160
180
200
8.5
8.0
7.5
7.0
6.5
6.0
5.5
5.0
4.5
4.0
3.5
3.0
2.5
2.0
1.5
ppm

$^{13}C\{^{1}H\}$ NMR

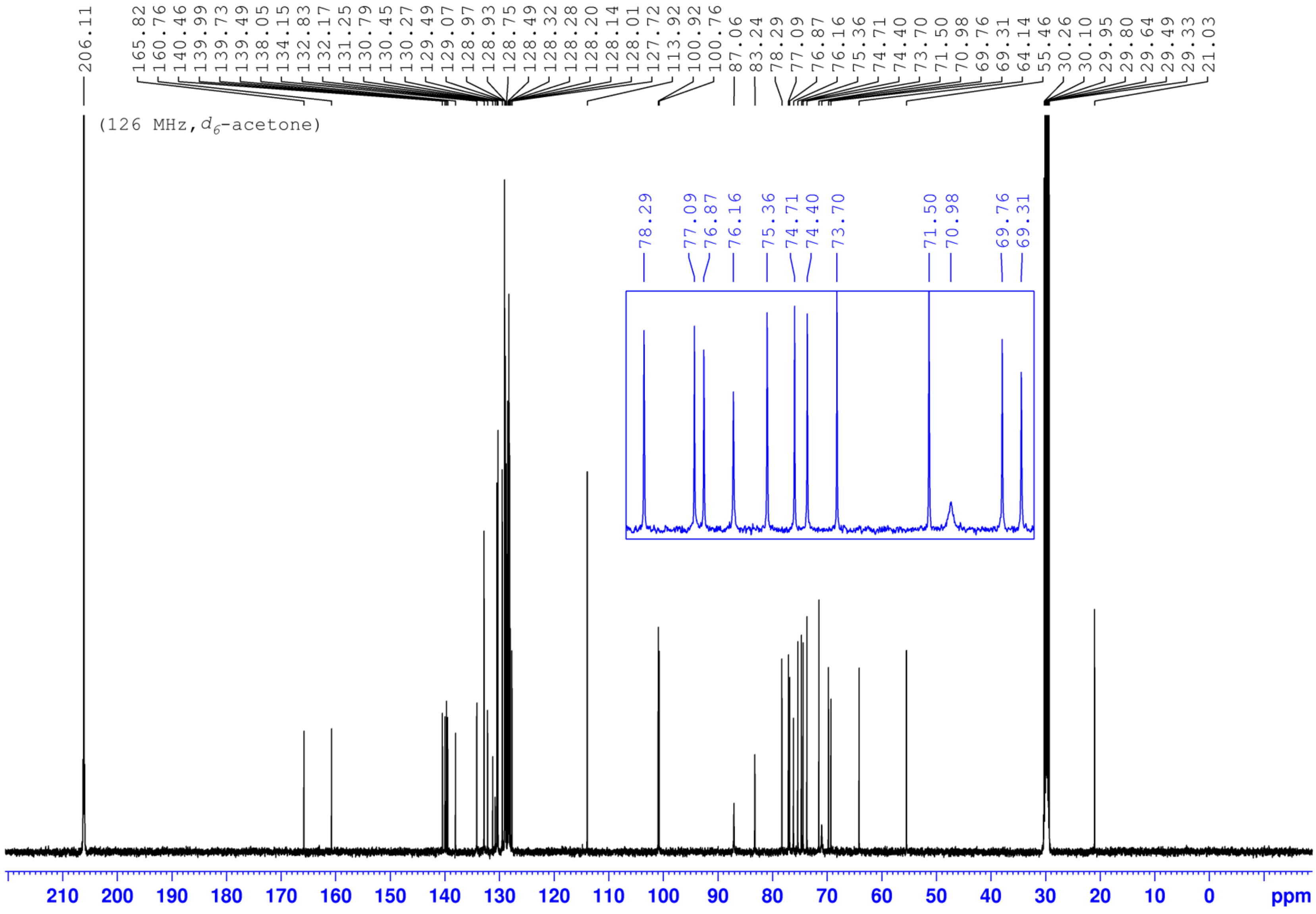
206.11
165.82
160.76
140.46
139.99
139.73
139.49
138.05
134.15
132.83
132.17
131.25
130.79
130.45
130.27
129.49
129.07
128.97
128.93
128.75
128.49
128.32
128.28
128.20
128.14
128.01
127.72
113.92
100.92
100.76
87.06
83.24
78.29
77.09
76.87
76.16
75.36
74.71
74.40
73.70
71.50
70.98
69.76
69.31
64.14
55.46
30.26
30.10
29.95
29.80
29.64
29.49
29.33
21.03
(126 MHz, d_6-acetone)
78.29
77.09
76.87
76.16
75.36
74.71
74.40
73.70
71.50
70.98
69.76
69.31
210
200
190
180
170
160
150
140
130
120
110
100
90
80
70
60
50
40
30
20
10
0
ppm

Compound **S7'**

^{1}H-NMR

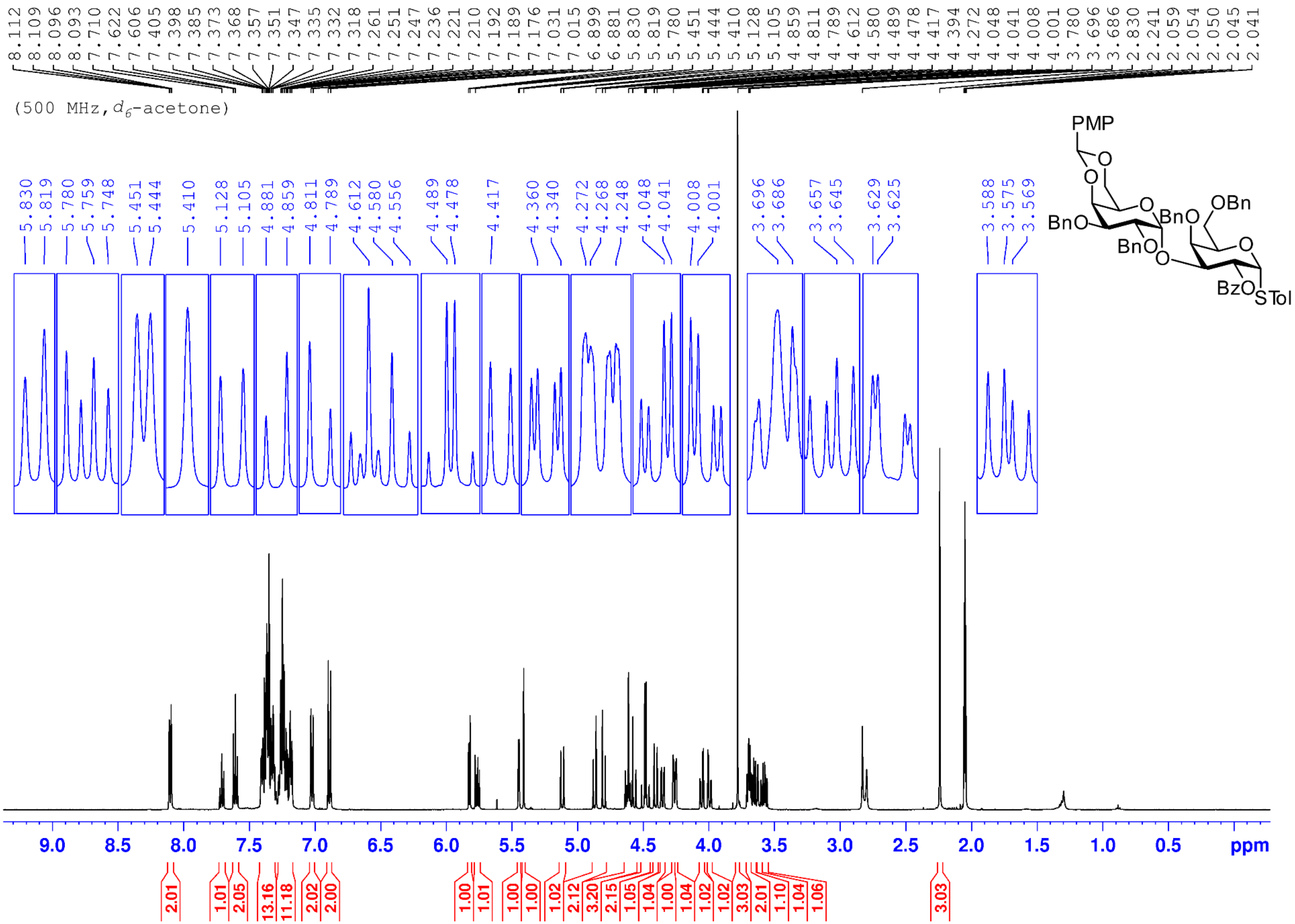

^{1}H-^{1}H COSY

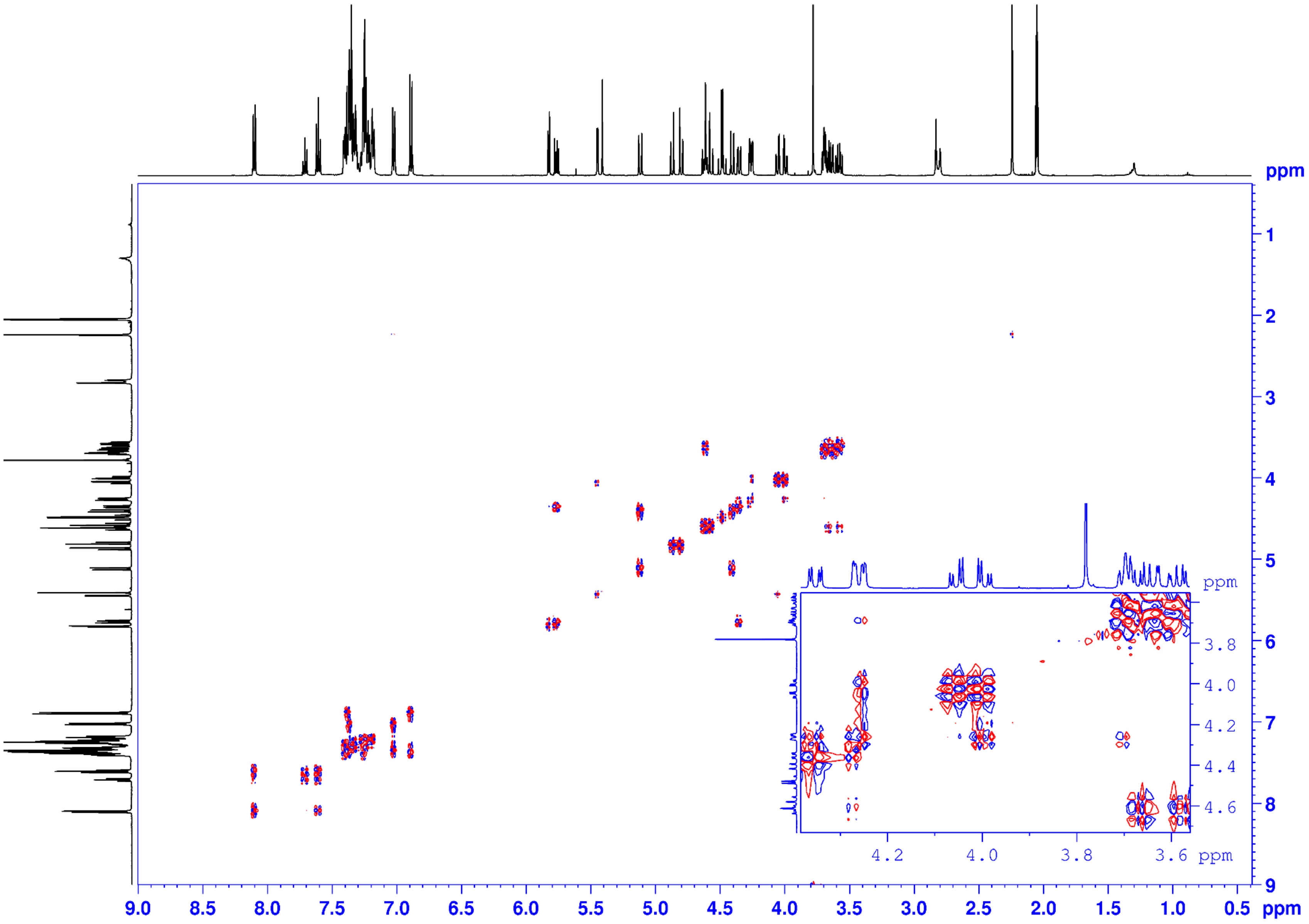

ppm
9.0
8.5
8.0
7.5
7.0
6.5
6.0
5.5
5.0
4.5
4.0
3.5
3.0
2.5
2.0
1.5
1.0
0.5
ppm
1
2
3
4
5
6
7
8
9
4.2
4.0
3.8
3.6
ppm
3.8
4.0
4.2
4.4
4.6

^{1}H-^{13}C HSQC

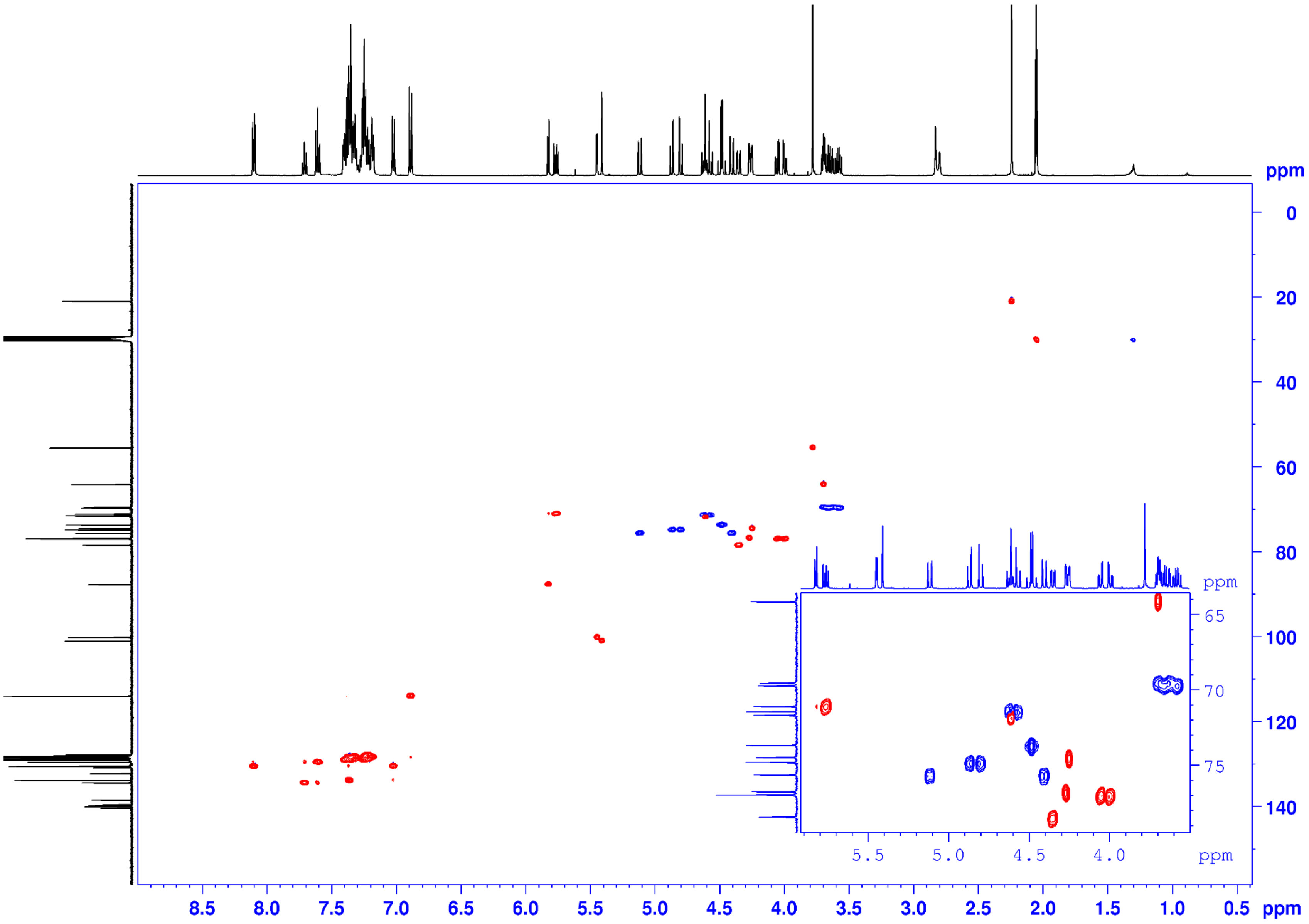

ppm
0
20
40
60
80
100
120
140
8.5
8.0
7.5
7.0
6.5
6.0
5.5
5.0
4.5
4.0
3.5
3.0
2.5
2.0
1.5
1.0
0.5
ppm
65
70
75
5.5
5.0
4.5
4.0

^{1}H-^{13}C non-decoupled HSQC

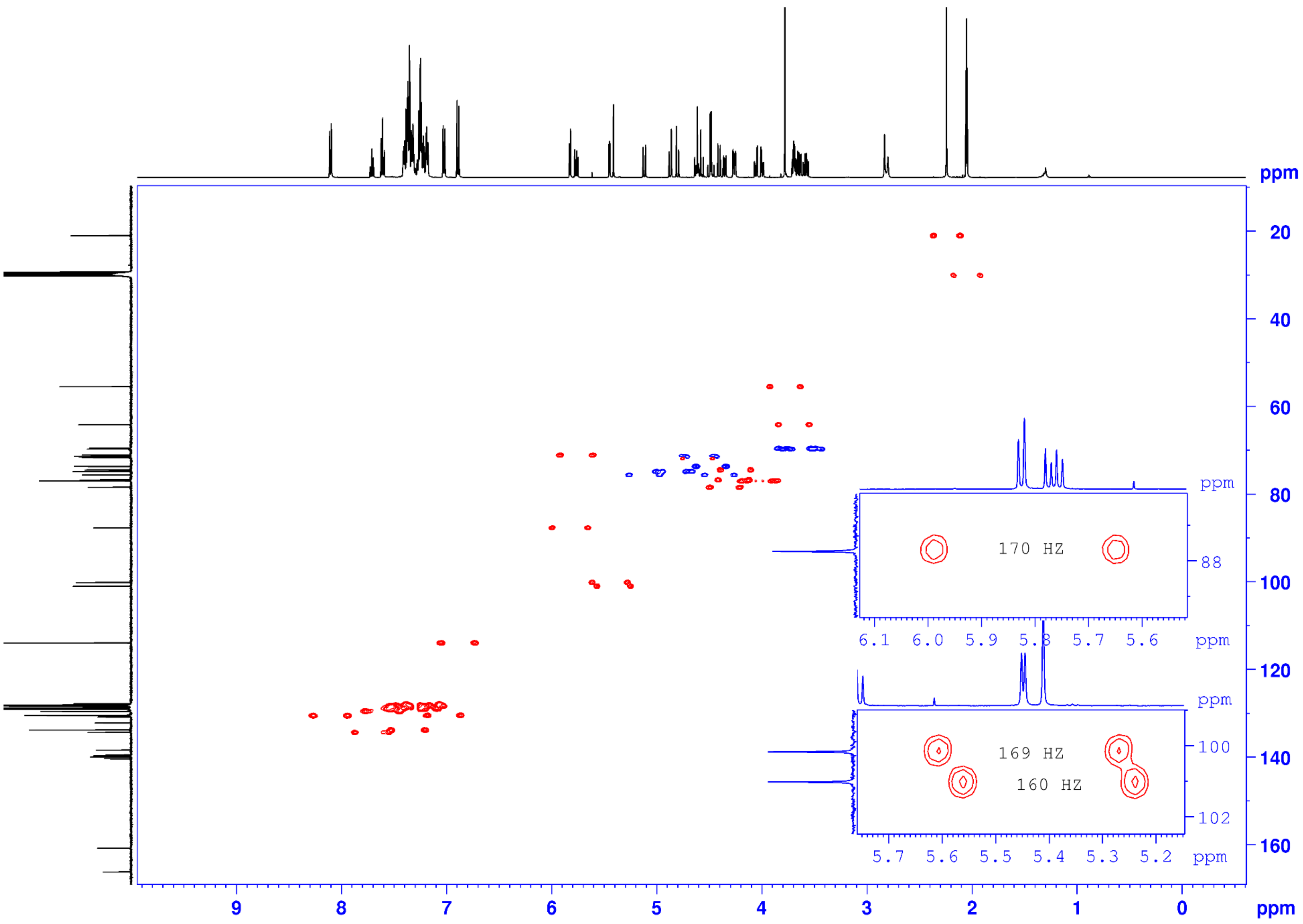

^{1}H-^{13}C HMBC

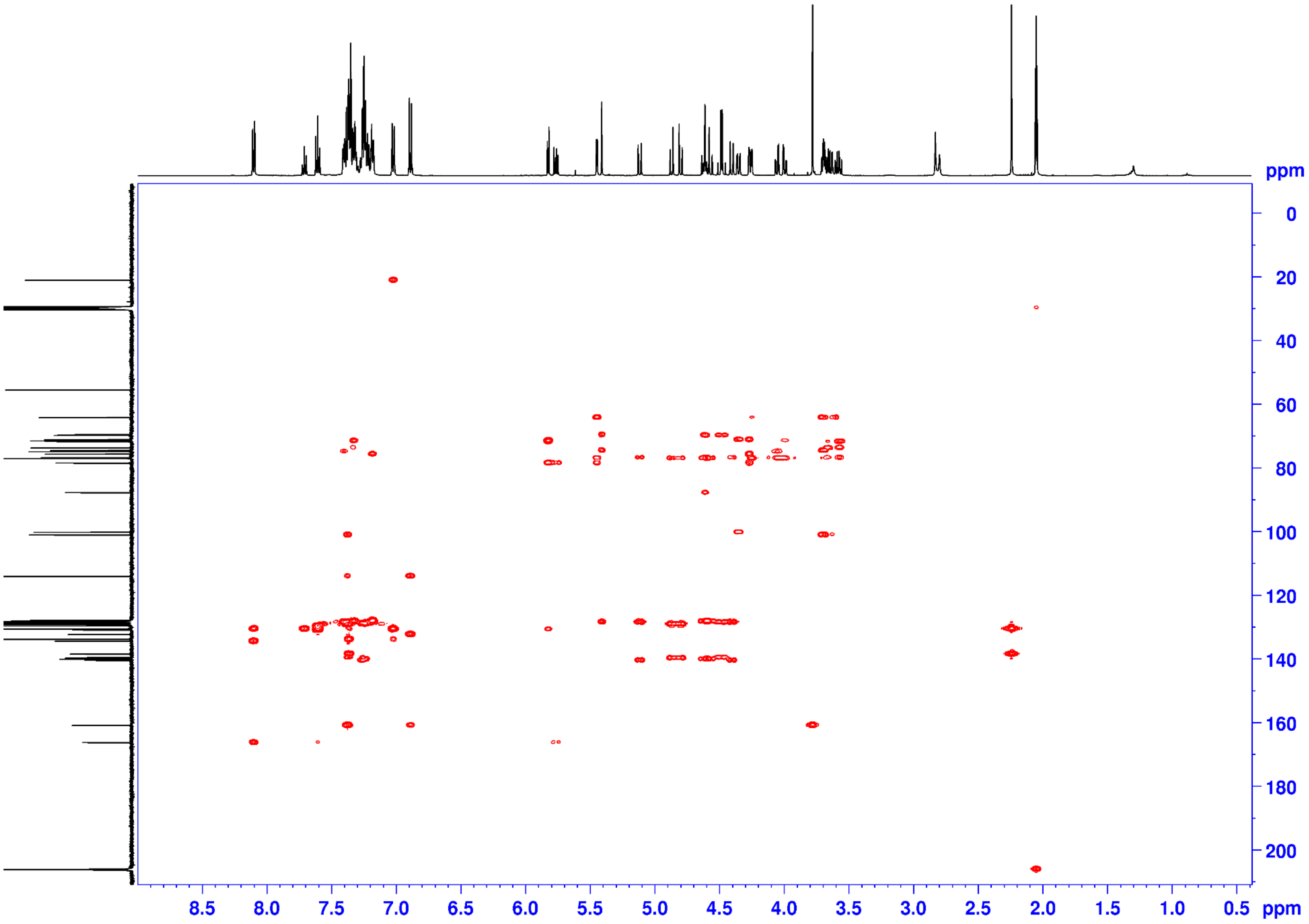
ppm
0
20
40
60
80
100
120
140
160
180
200
8.5
8.0
7.5
7.0
6.5
6.0
5.5
5.0
4.5
4.0
3.5
3.0
2.5
2.0
1.5
1.0
0.5
ppm

$^{13}C\{^{1}H\}$ NMR

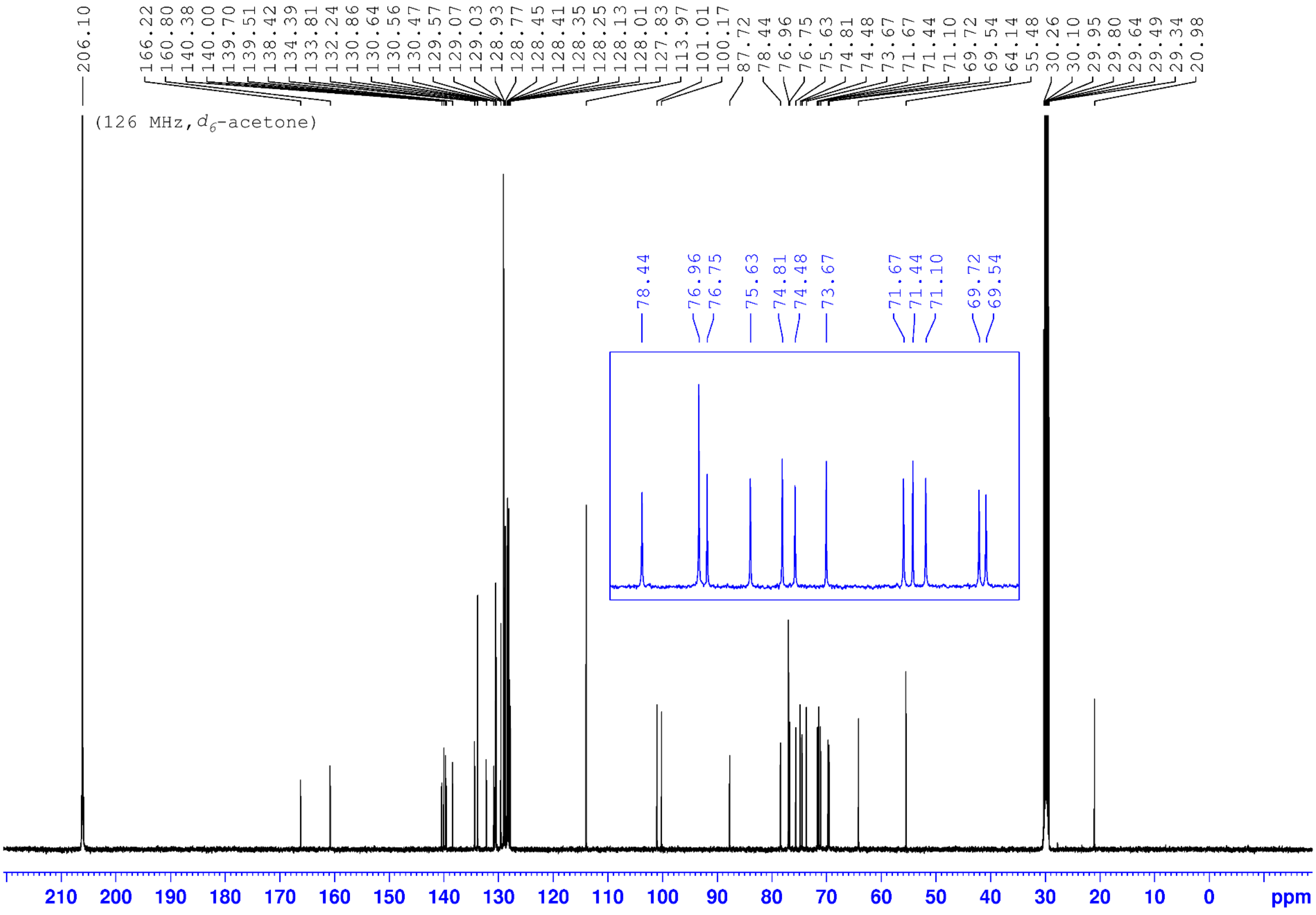

206.10
166.22
160.80
140.38
140.00
139.70
139.51
138.42
134.39
133.81
132.24
130.86
130.64
130.56
130.47
129.57
129.07
129.03
128.93
128.77
128.45
128.41
128.35
128.25
128.13
128.01
127.83
113.97
101.01
100.17
87.72
78.44
76.96
76.75
75.63
74.81
74.48
73.67
71.67
71.44
71.10
69.72
69.54
64.14
55.48
30.26
30.10
29.95
29.80
29.64
29.49
29.34
20.98
(126 MHz, d6-acetone)
78.44
76.96
76.75
75.63
74.81
74.48
73.67
71.67
71.44
71.10
69.72
69.54
210
200
190
180
170
160
150
140
130
120
110
100
90
80
70
60
50
40
30
20
10
0
ppm

Compound **S8**

^{1}H-NMR

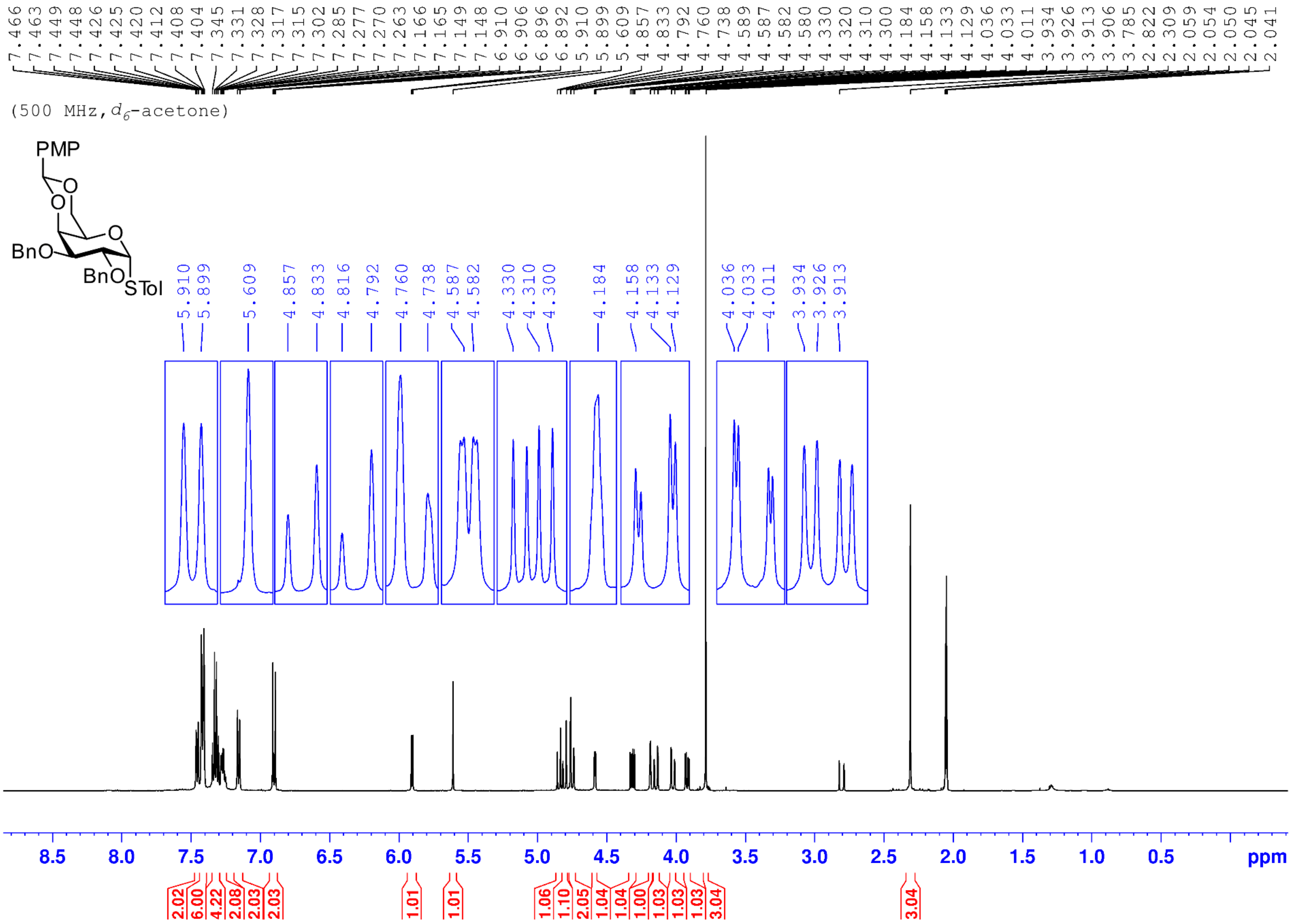

^{1}H-^{1}H COSY

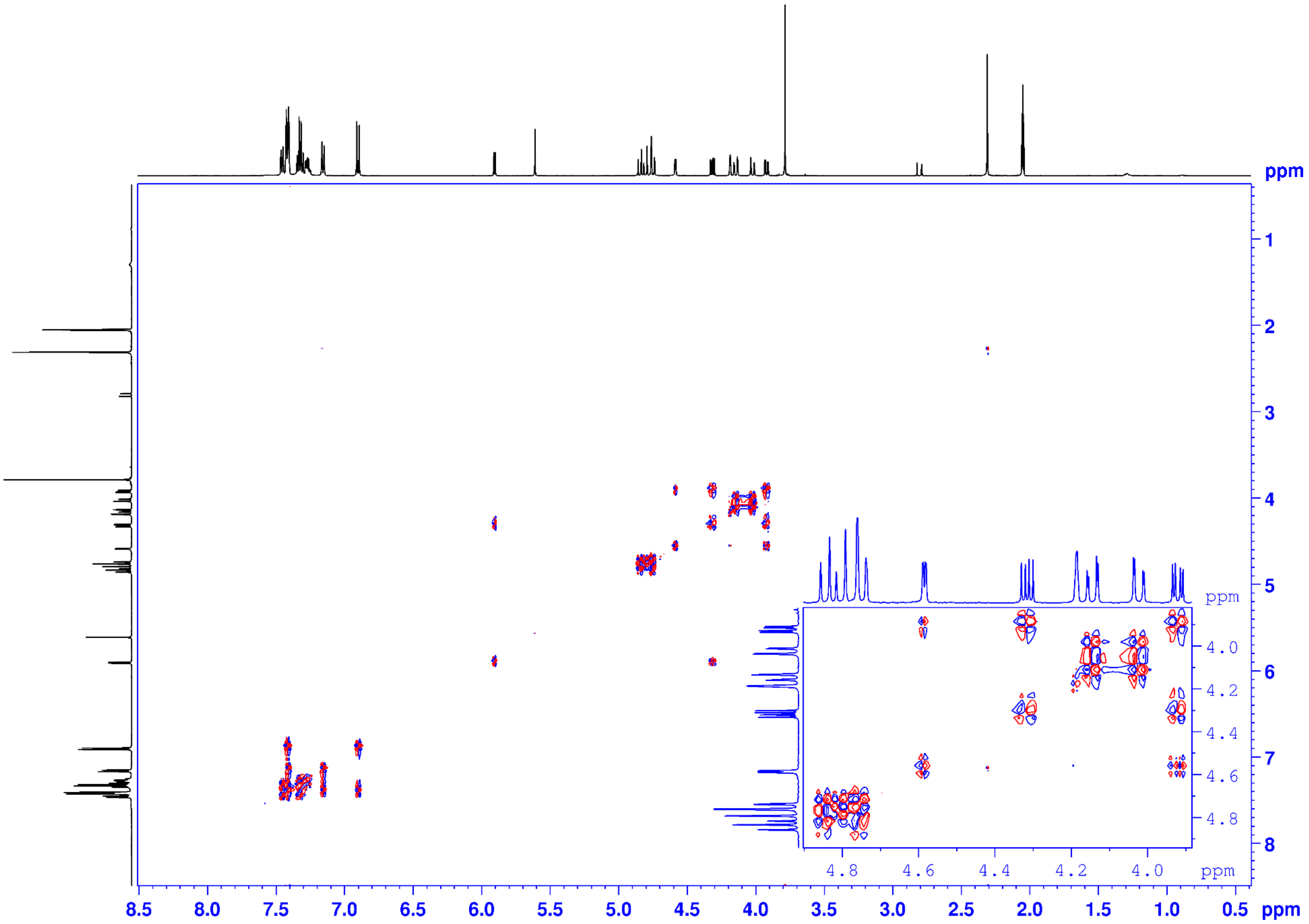

ppm
1
2
3
4
5
6
7
8
8.5
8.0
7.5
7.0
6.5
6.0
5.5
5.0
4.5
4.0
3.5
3.0
2.5
2.0
1.5
1.0
0.5
ppm
4.0
4.2
4.4
4.6
4.8
4.8
4.6
4.4
4.2
4.0
ppm

^{1}H-^{13}C HSQC

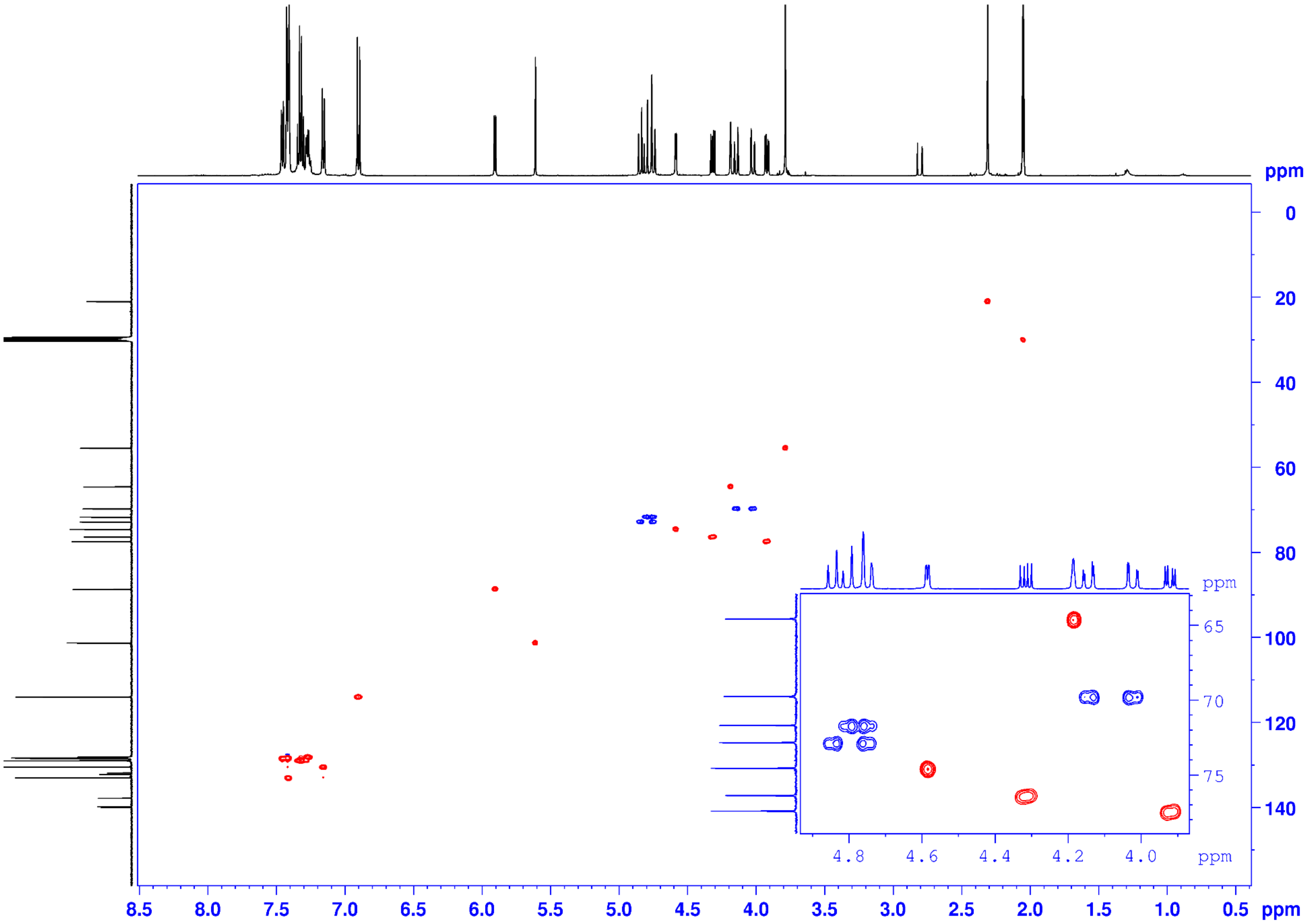
ppm
0
20
40
60
80
100
120
140
65
70
75
4.8
4.6
4.4
4.2
4.0
8.5
8.0
7.5
7.0
6.5
6.0
5.5
5.0
4.5
4.0
3.5
3.0
2.5
2.0
1.5
1.0
0.5
ppm

^{1}H-^{13}C HMBC

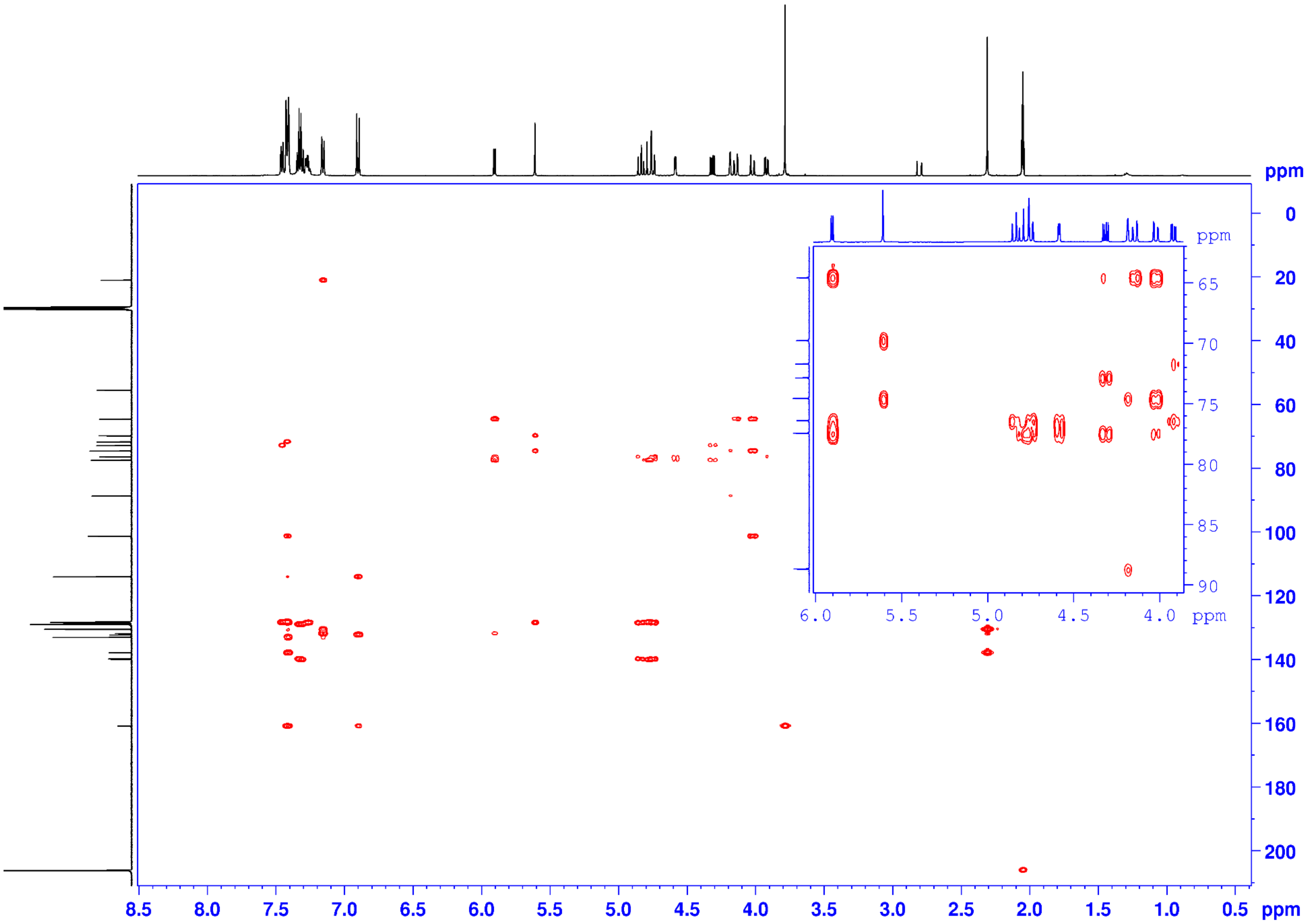

ppm
0
20
40
60
80
100
120
140
160
180
200
8.5
8.0
7.5
7.0
6.5
6.0
5.5
5.0
4.5
4.0
3.5
3.0
2.5
2.0
1.5
1.0
0.5
ppm
65
70
75
80
85
90
6.0
5.5
5.0
4.5
4.0

$^{13}C\{^{1}H\}$ NMR

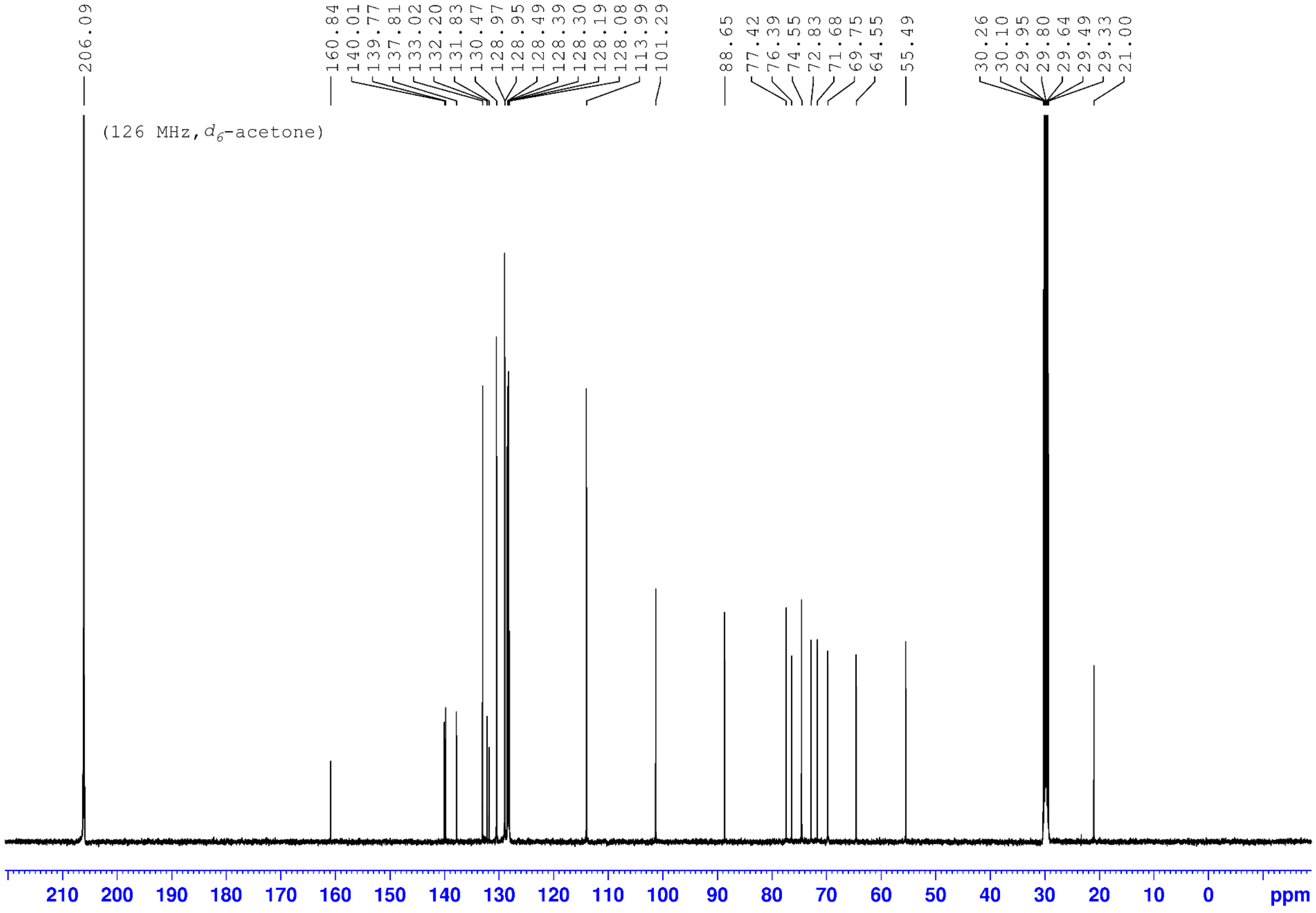

206.09
160.84
140.01
139.77
137.81
133.02
132.20
131.83
130.47
128.97
128.95
128.49
128.39
128.30
128.19
128.08
113.99
101.29
88.65
77.42
76.39
74.55
72.83
71.68
69.75
64.55
55.49
30.26
30.10
29.95
29.80
29.64
29.49
29.33
21.00
(126 MHz, d6-acetone)
210 200 190 180 170 160 150 140 130 120 110 100 90 80 70 60 50 40 30 20 10 0 ppm

Compound **S9**

^{1}H-NMR

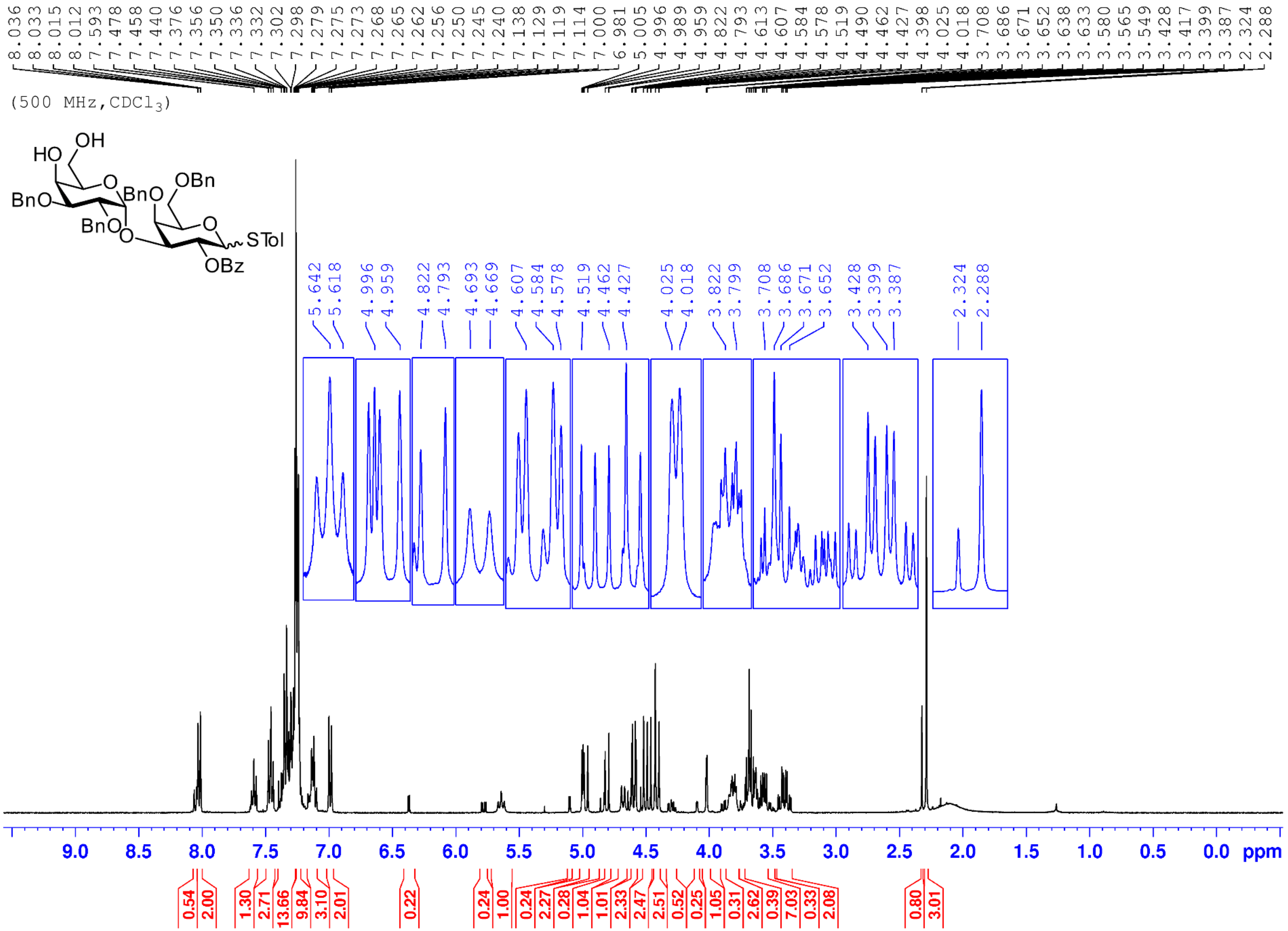

^1H-^1H COSY

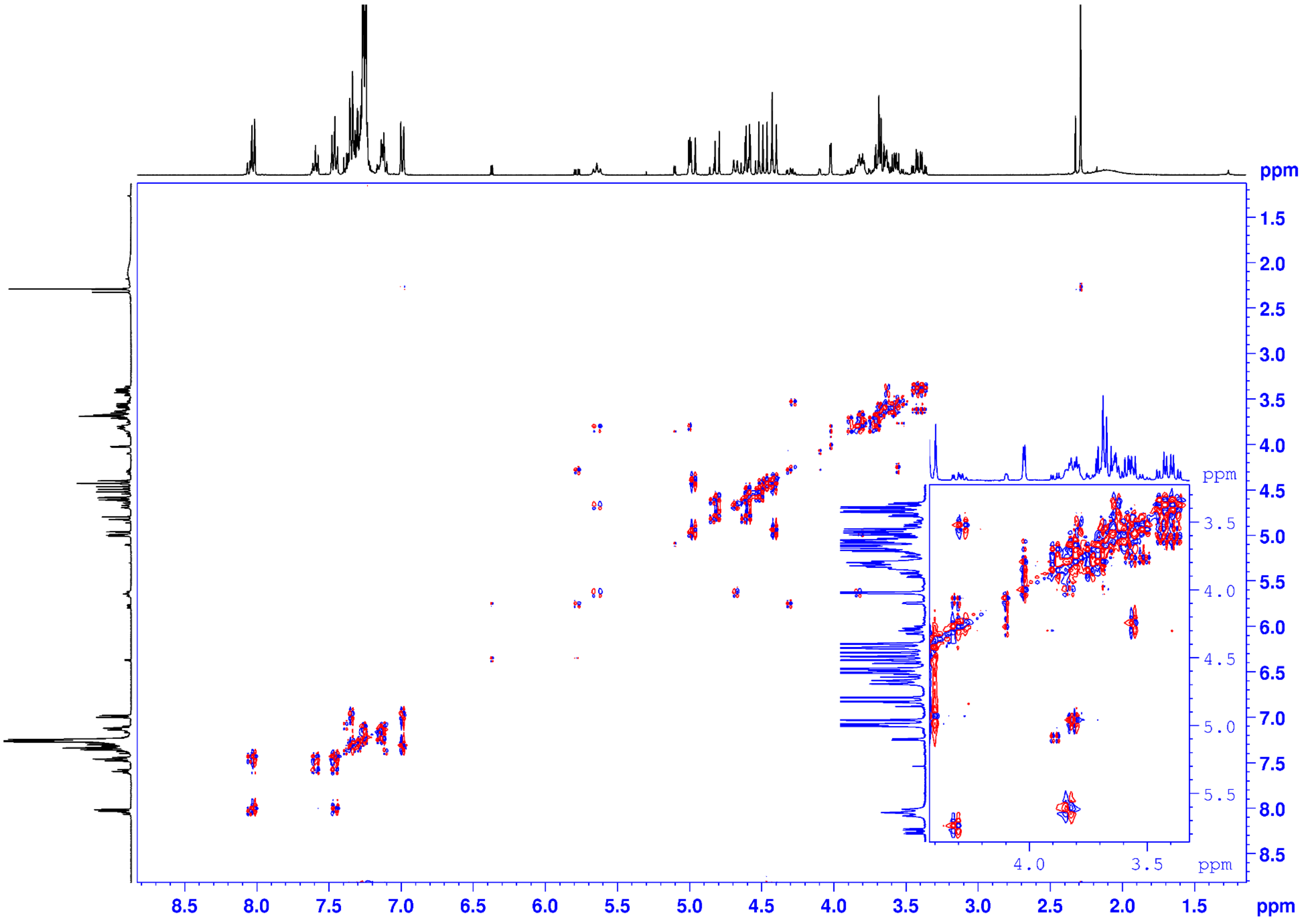

ppm
1.5
2.0
2.5
3.0
3.5
4.0
4.5
5.0
5.5
6.0
6.5
7.0
7.5
8.0
8.5
8.5
8.0
7.5
7.0
6.5
6.0
5.5
5.0
4.5
4.0
3.5
3.0
2.5
2.0
1.5
ppm

^{1}H-^{13}C HSQC

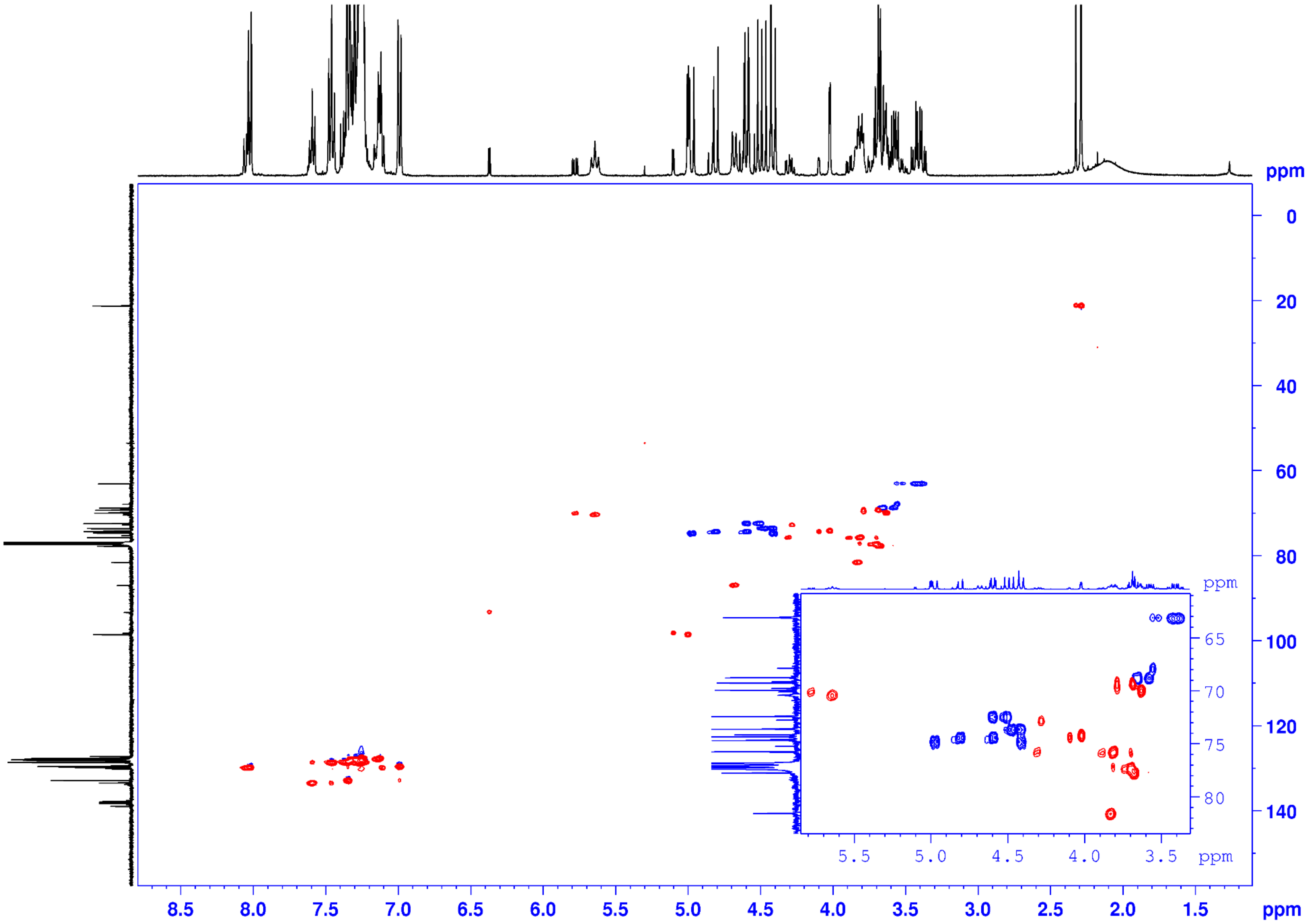
ppm
0
20
40
60
80
100
120
140
8.5
8.0
7.5
7.0
6.5
6.0
5.5
5.0
4.5
4.0
3.5
3.0
2.5
2.0
1.5
ppm
65
70
75
80
5.5
5.0
4.5
4.0
3.5
ppm

^{1}H-^{13}C non-decoupled HSQC

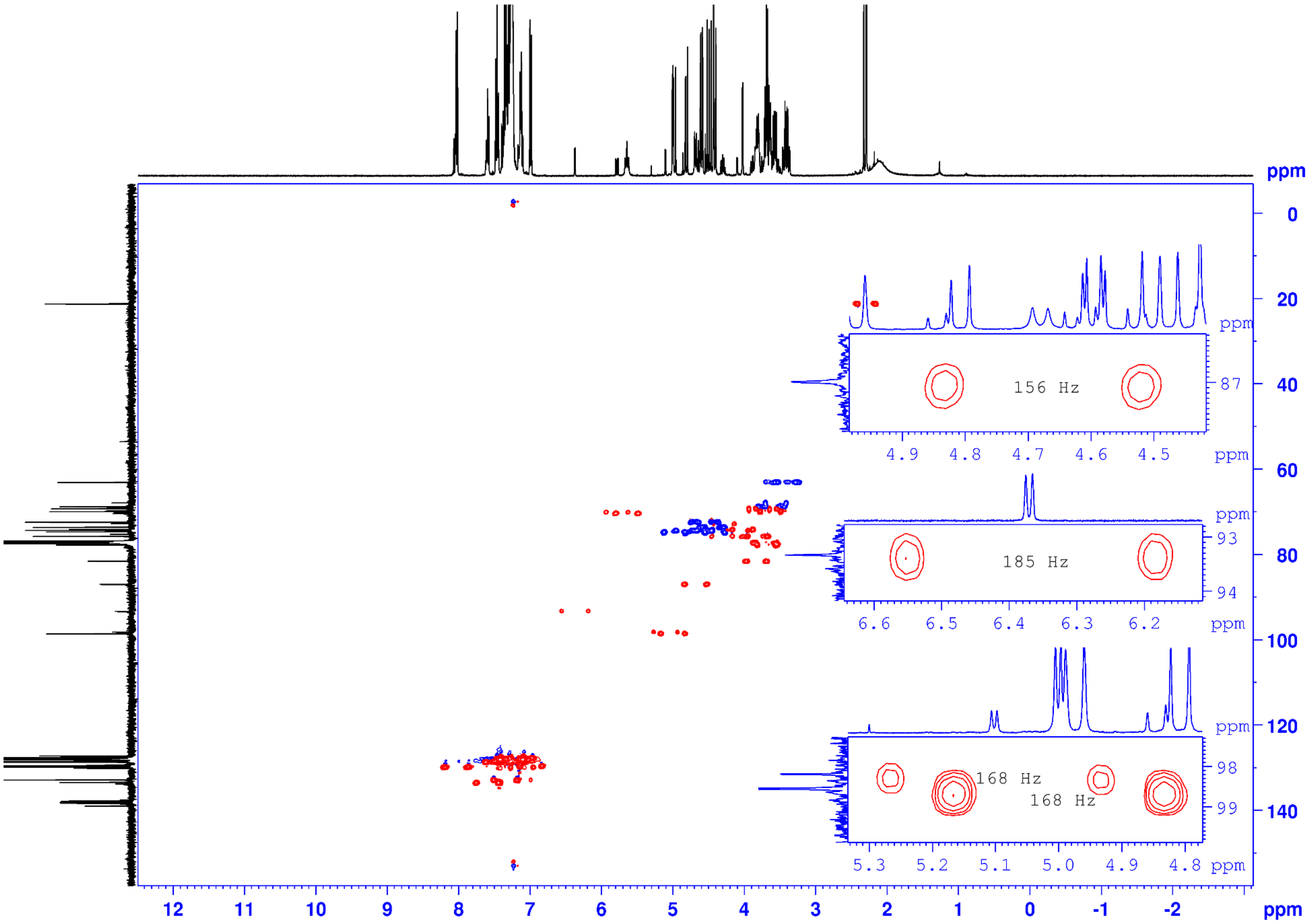

$^{13}C\{^{1}H\}$ NMR

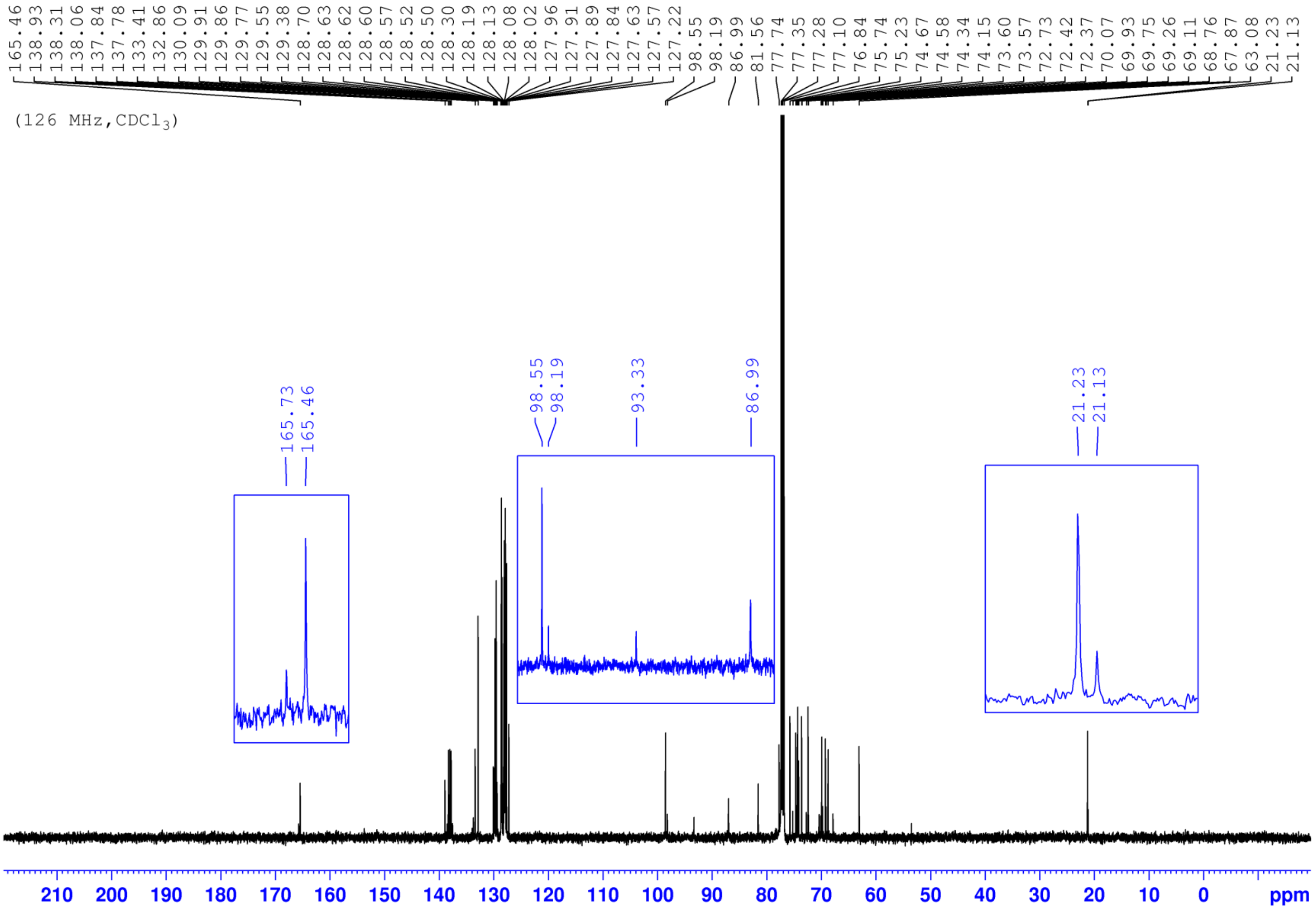
165.46
138.93
138.31
138.06
137.84
137.78
133.41
132.86
130.09
129.91
129.86
129.77
129.55
129.38
128.70
128.63
128.62
128.60
128.57
128.52
128.50
128.30
128.19
128.13
128.08
128.02
127.96
127.91
127.89
127.84
127.63
127.57
127.22
98.55
98.19
86.99
81.56
77.74
77.35
77.28
77.10
76.84
75.74
75.23
74.67
74.58
74.34
74.15
73.60
73.57
72.73
72.42
72.37
70.07
69.93
69.75
69.26
69.11
68.76
67.87
63.08
21.23
21.13
(126 MHz, CDCl3)
165.73
165.46
98.55
98.19
93.33
86.99
21.23
21.13
210
200
190
180
170
160
150
140
130
120
110
100
90
80
70
60
50
40
30
20
10
0
ppm

Compound **S10**

^{1}H-NMR

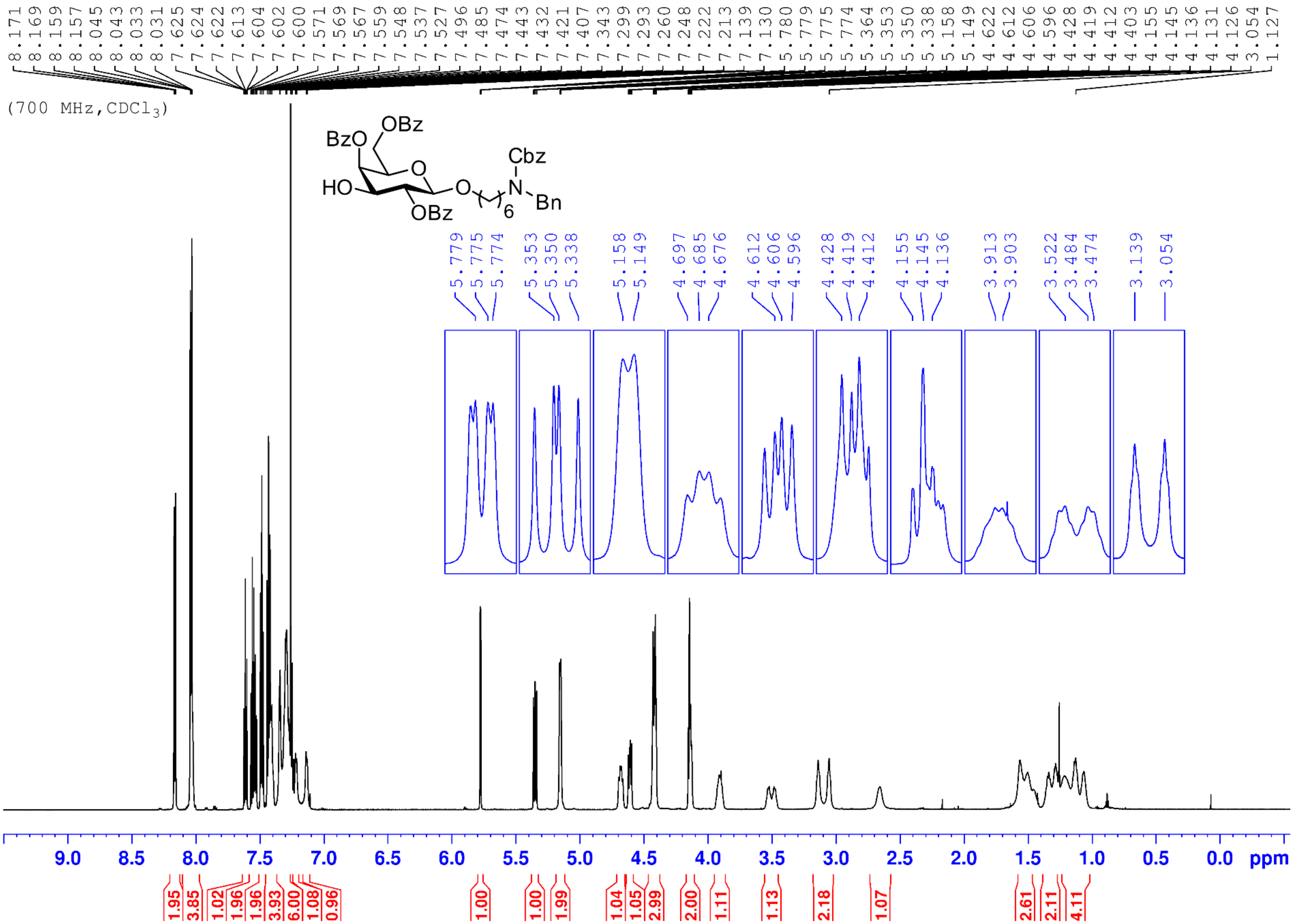

^{1}H-^{1}H COSY

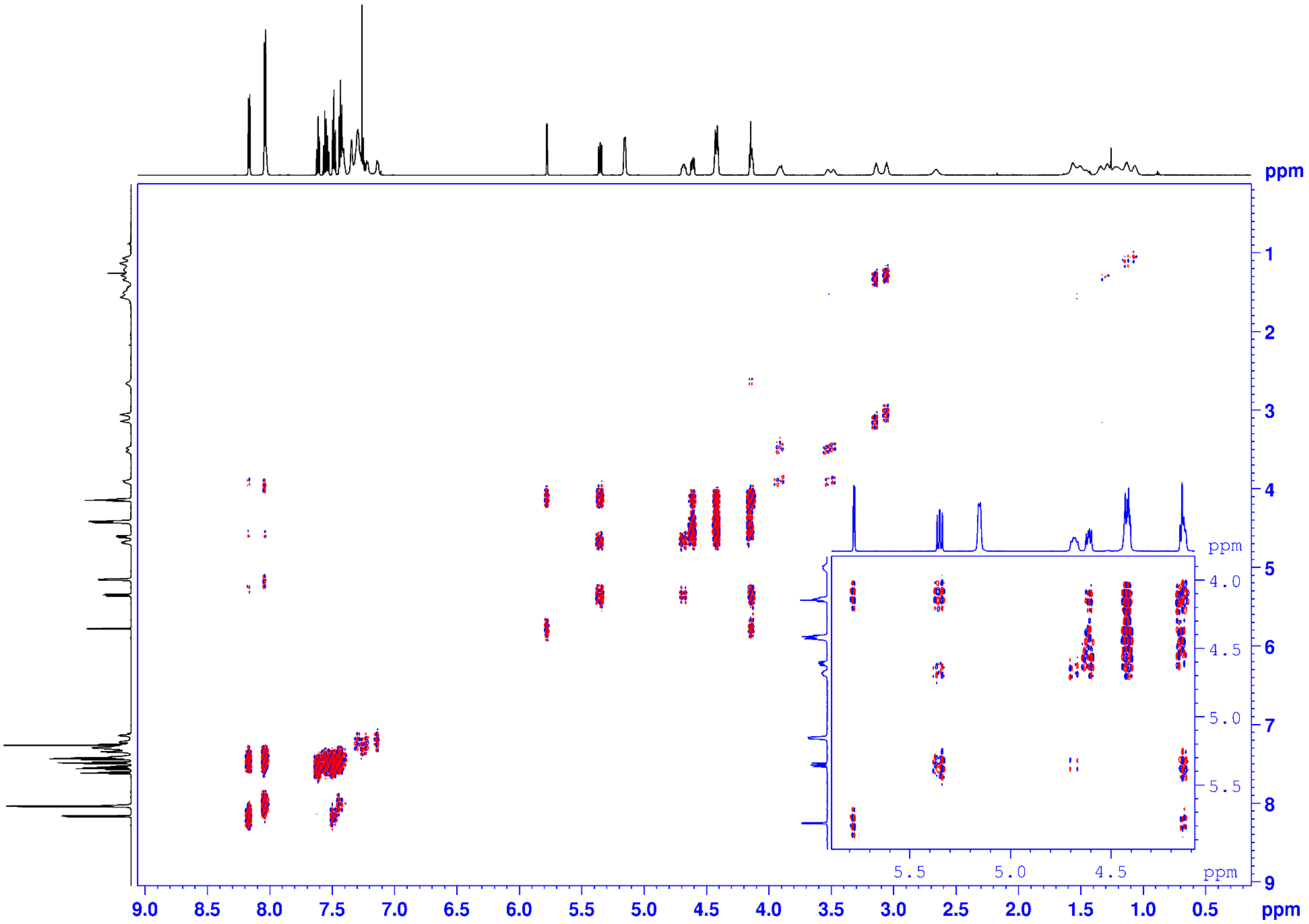

ppm
1
2
3
4
5
6
7
8
9
9.0
8.5
8.0
7.5
7.0
6.5
6.0
5.5
5.0
4.5
4.0
3.5
3.0
2.5
2.0
1.5
1.0
0.5
ppm
4.0
4.5
5.0
5.5
5.5
5.0
4.5
ppm

^{1}H-^{13}C HSQC

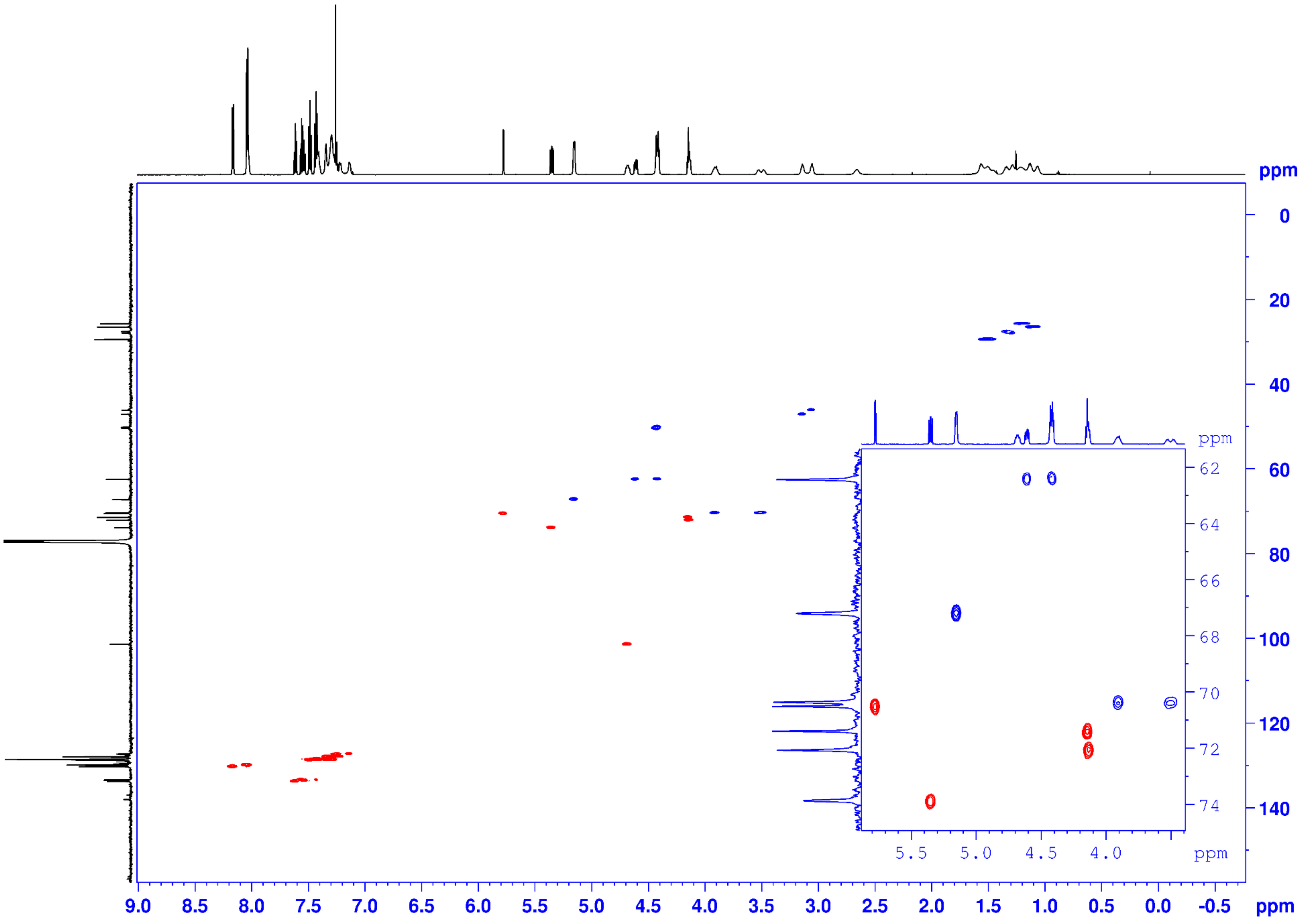

ppm
0
20
40
60
80
100
120
140
9.0
8.5
8.0
7.5
7.0
6.5
6.0
5.5
5.0
4.5
4.0
3.5
3.0
2.5
2.0
1.5
1.0
0.5
0.0
-0.5
ppm
62
64
66
68
70
72
74
5.5
5.0
4.5
4.0
ppm

^{1}H-^{13}C HMBC

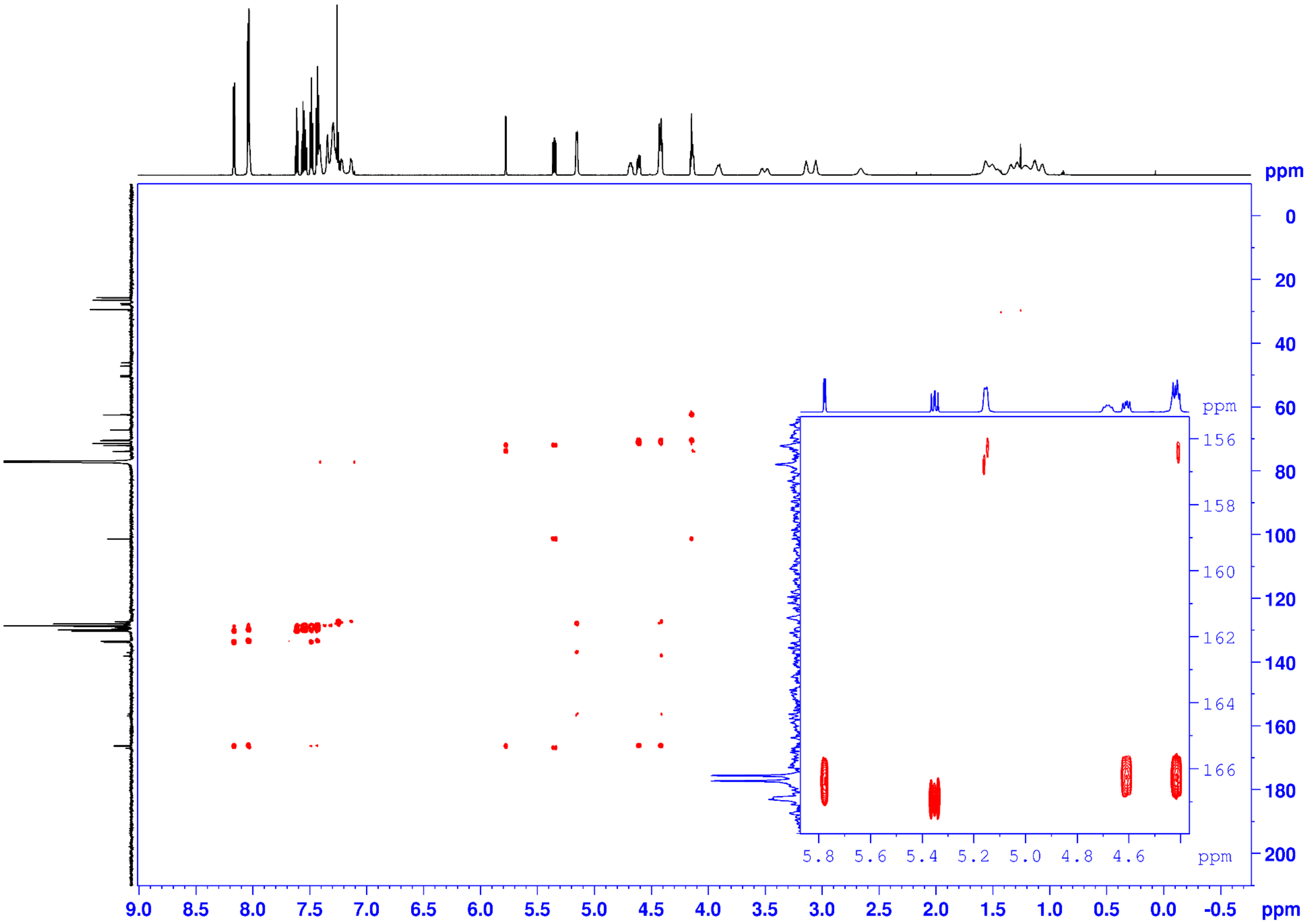
ppm
0
20
40
60
80
100
120
140
160
180
200
9.0
8.5
8.0
7.5
7.0
6.5
6.0
5.5
5.0
4.5
4.0
3.5
3.0
2.5
2.0
1.5
1.0
0.5
0.0
-0.5
ppm
156
158
160
162
164
166
5.8
5.6
5.4
5.2
5.0
4.8
4.6
ppm

$^{13}C\{^{1}H\}$ NMR

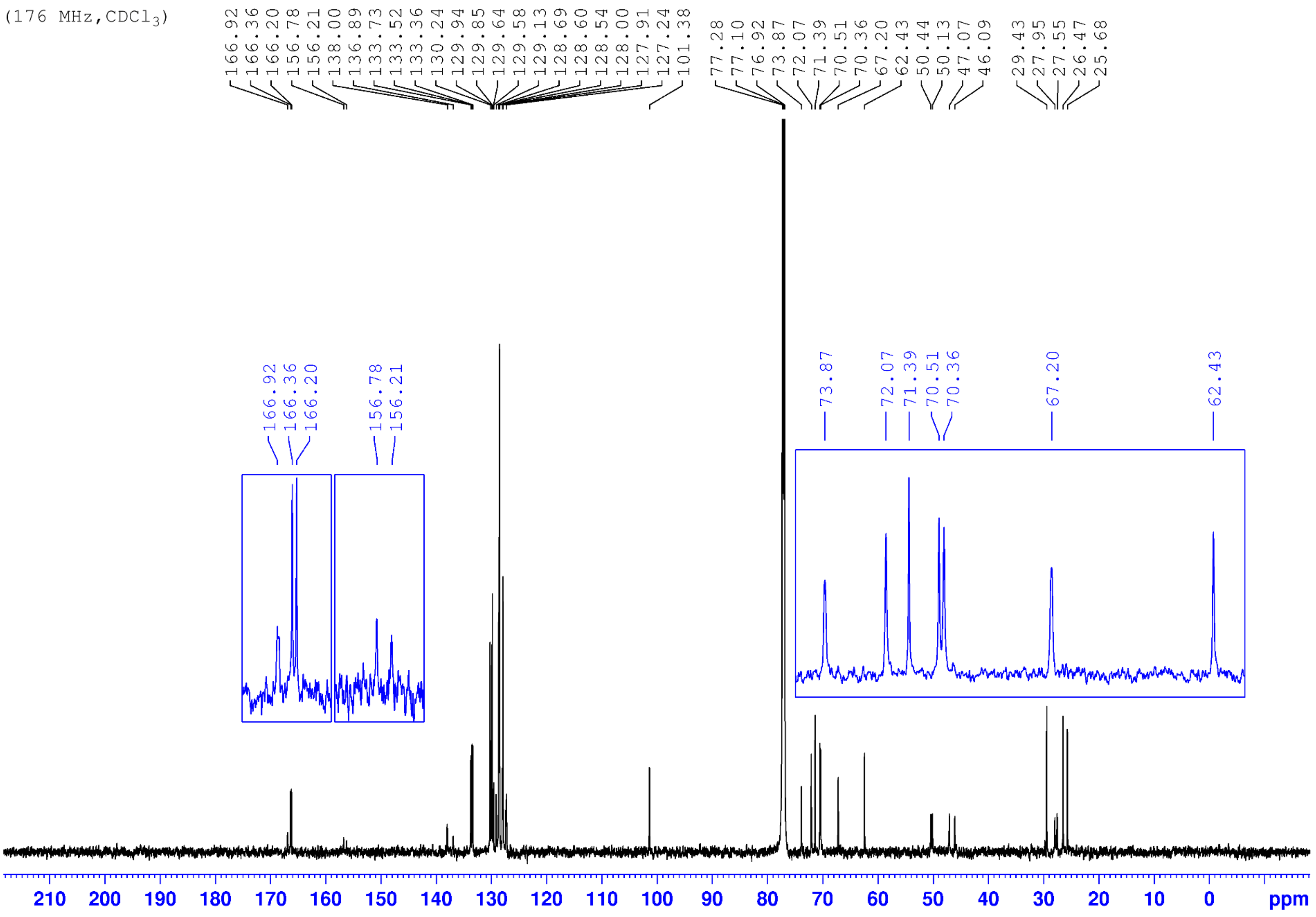

(176 MHz,CDCl3)
166.92
166.36
166.20
156.78
156.21
138.00
136.89
133.73
133.52
133.36
130.24
129.94
129.85
129.64
129.58
129.13
128.69
128.60
128.54
128.00
127.91
127.24
101.38
77.28
77.10
76.92
73.87
72.07
71.39
70.51
70.36
67.20
62.43
50.44
50.13
47.07
46.09
29.43
27.95
27.55
26.47
25.68
210 200 190 180 170 160 150 140 130 120 110 100 90 80 70 60 50 40 30 20 10 0 ppm

Compound **S11**

^{1}H-NMR

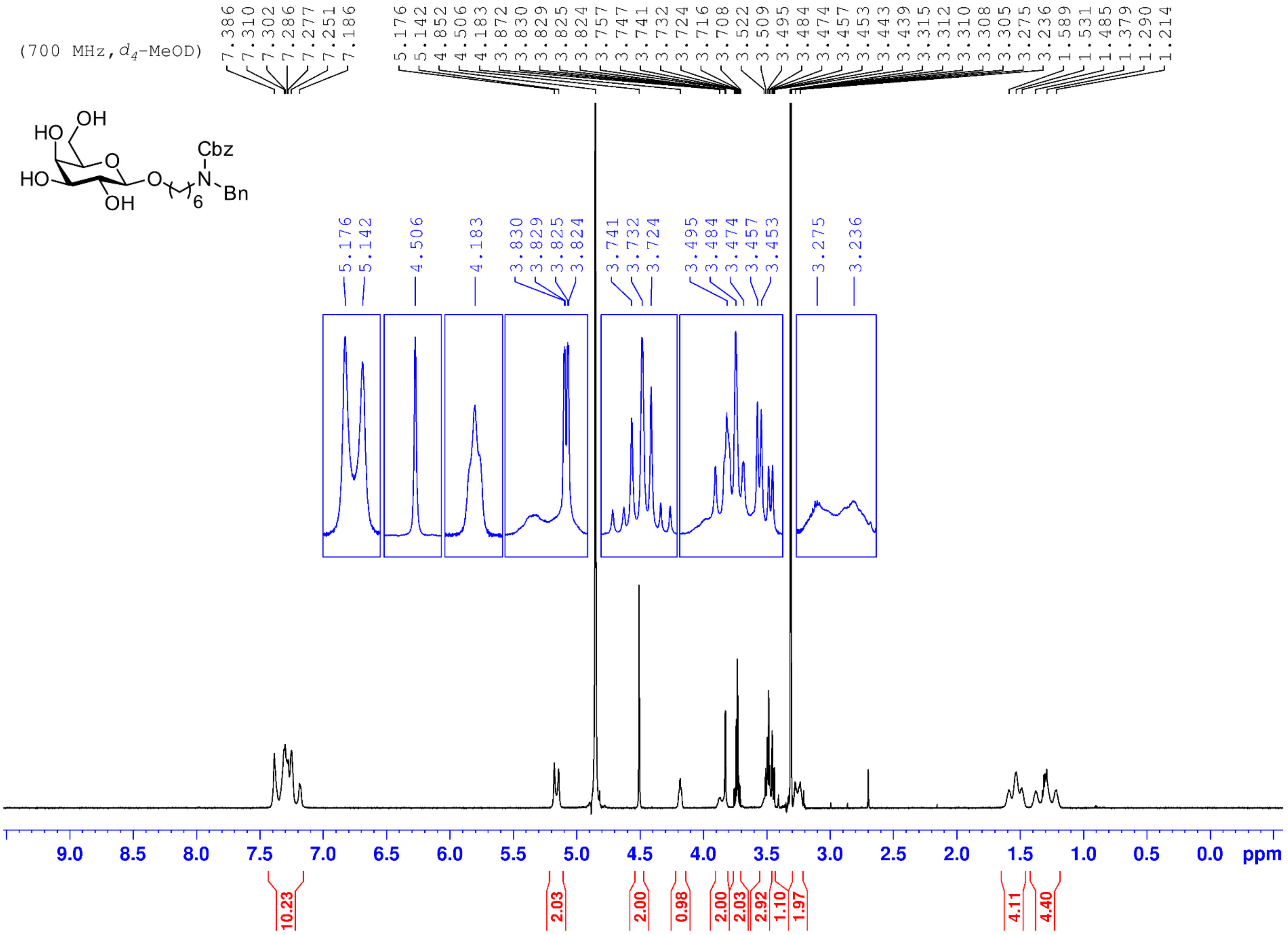

^{1}H-^{1}H COSY

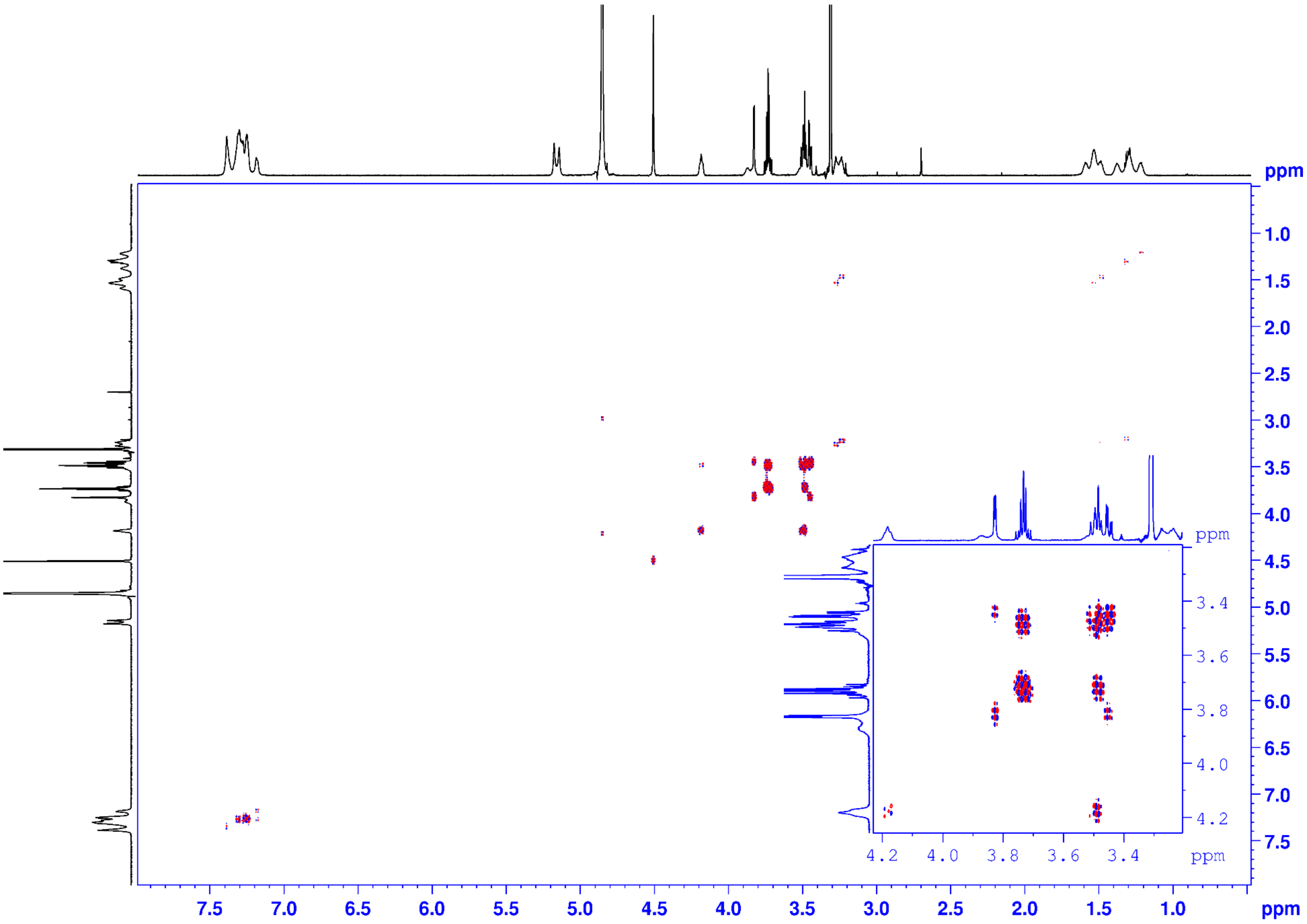

ppm
1.0
1.5
2.0
2.5
3.0
3.5
4.0
4.5
5.0
5.5
6.0
6.5
7.0
7.5
7.5
7.0
6.5
6.0
5.5
5.0
4.5
4.0
3.5
3.0
2.5
2.0
1.5
1.0
ppm
3.4
3.6
3.8
4.0
4.2
4.2
4.0
3.8
3.6
3.4
ppm

^{1}H-^{13}C HSQC

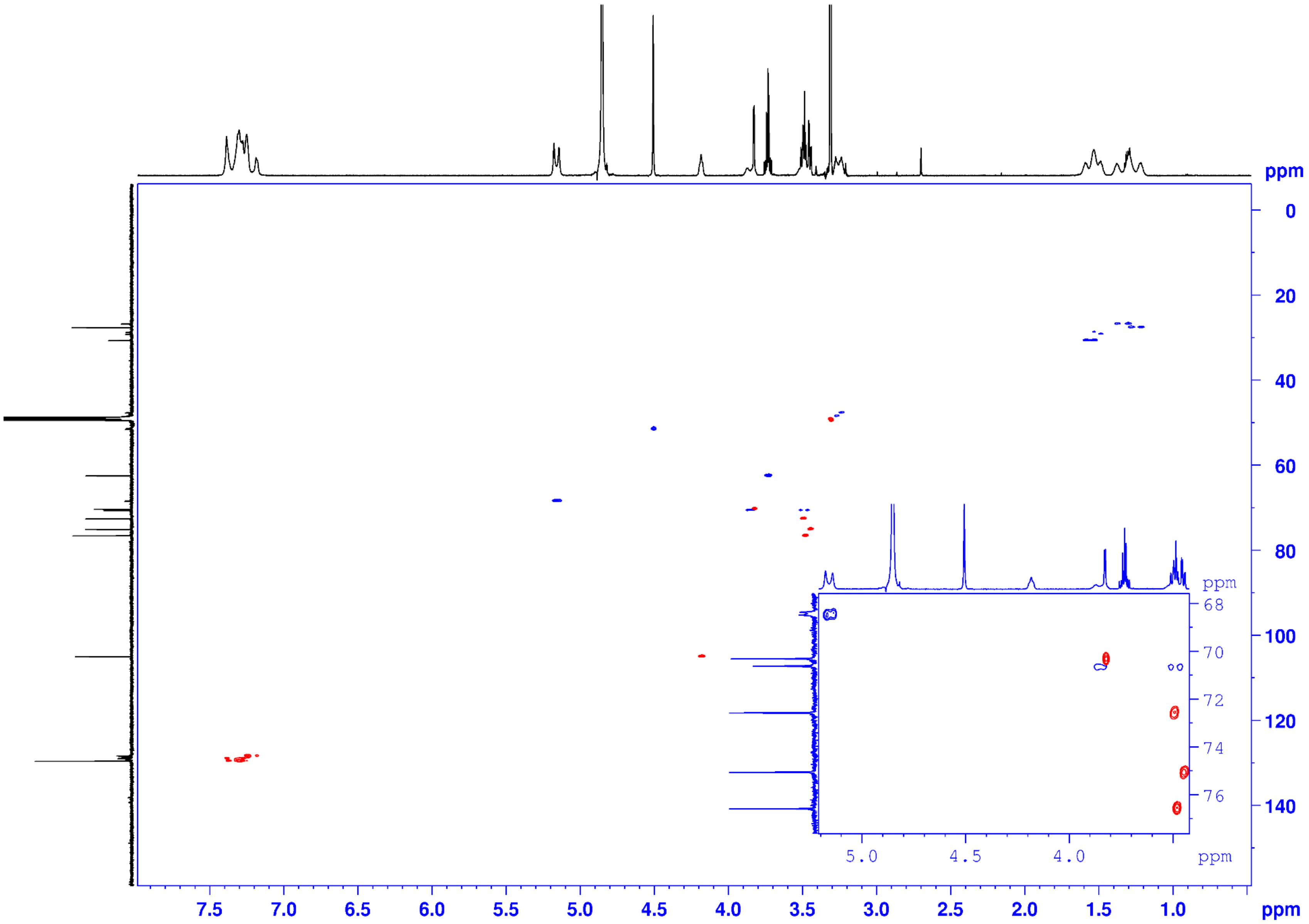
ppm
0
20
40
60
80
100
120
140
7.5
7.0
6.5
6.0
5.5
5.0
4.5
4.0
3.5
3.0
2.5
2.0
1.5
1.0
ppm
68
70
72
74
76
5.0
4.5
4.0
ppm

$^{1}H-^{13}C$ HMBC

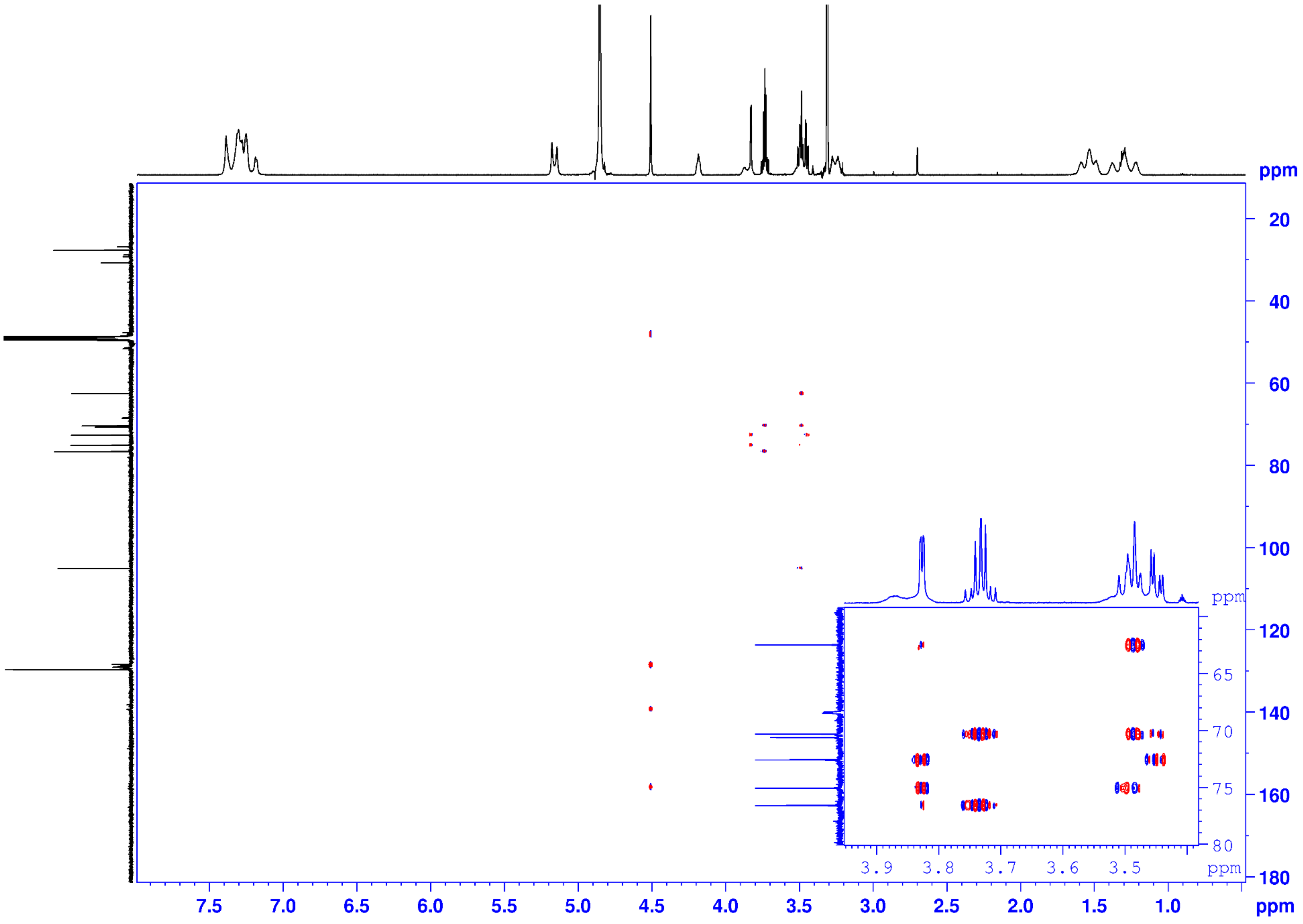
ppm
20
40
60
80
100
120
140
160
180
ppm
7.5
7.0
6.5
6.0
5.5
5.0
4.5
4.0
3.5
3.0
2.5
2.0
1.5
1.0
ppm
65
70
75
80
3.9
3.8
3.7
3.6
3.5
ppm

$^{13}C\{^{1}H\}$ NMR

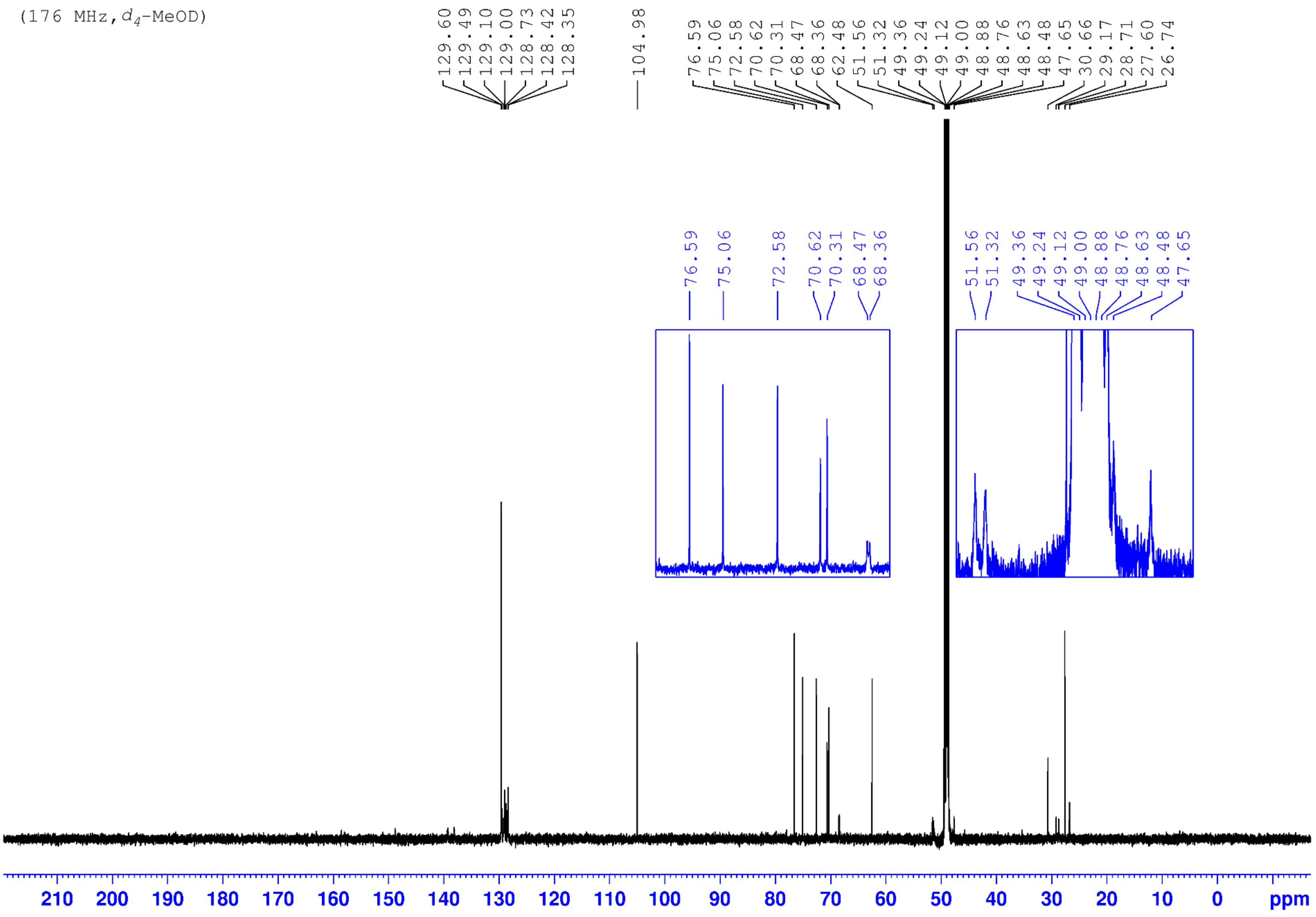
(176 MHz, d_4-MeOD)
129.60
129.49
129.10
129.00
128.73
128.42
128.35
104.98
76.59
75.06
72.58
70.62
70.31
68.47
68.36
62.48
51.56
51.32
49.36
49.24
49.12
49.00
48.88
48.76
48.63
48.48
47.65
30.66
29.17
28.71
27.60
26.74
210
200
190
180
170
160
150
140
130
120
110
100
90
80
70
60
50
40
30
20
10
0
ppm

Compound **S12**

^{1}H-NMR

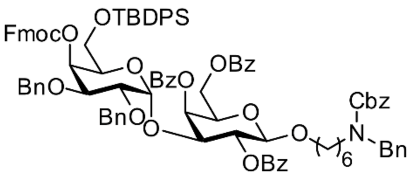

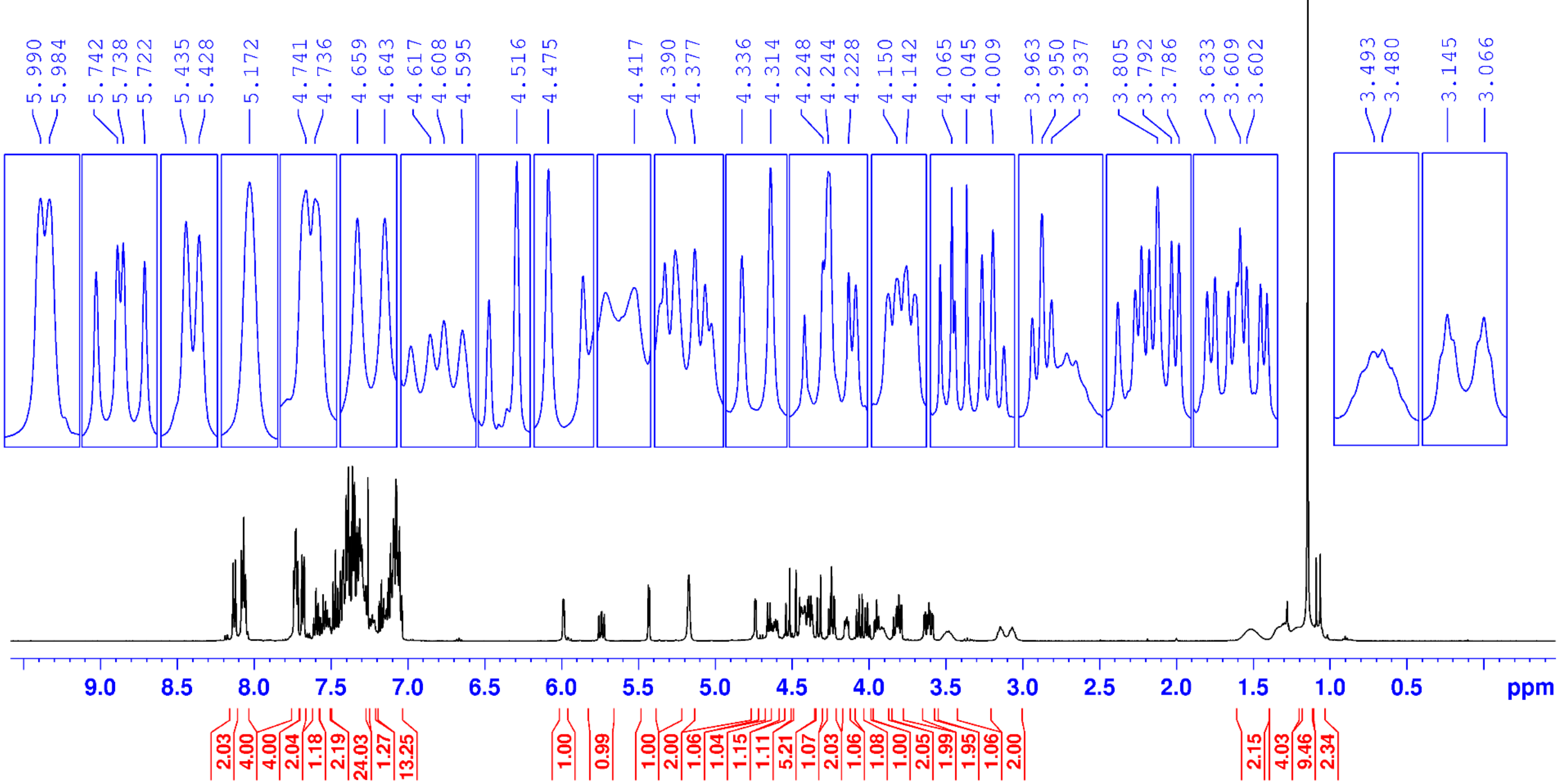

^{1}H-^{1}H COSY

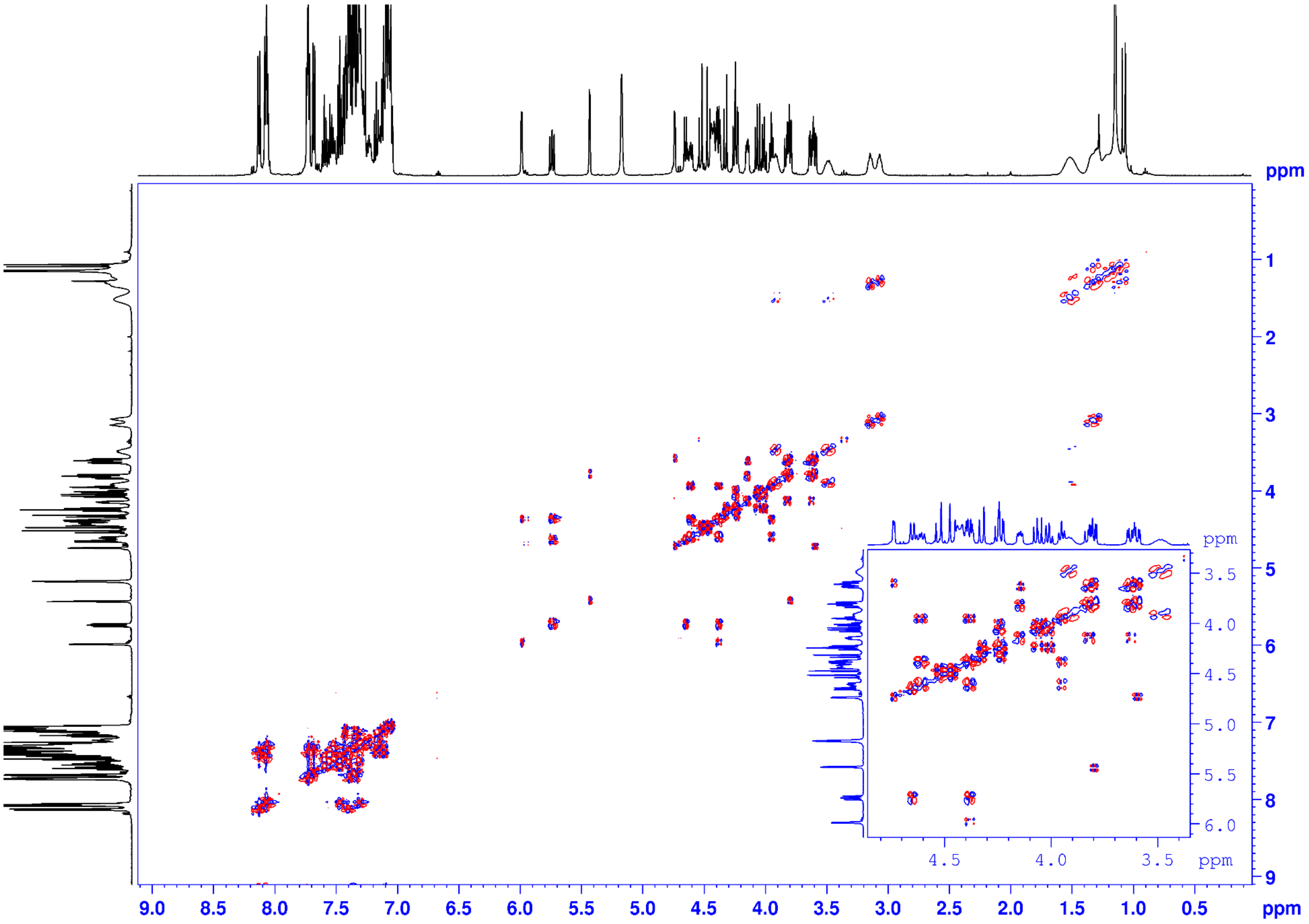

ppm
1
2
3
4
5
6
7
8
9
9.0
8.5
8.0
7.5
7.0
6.5
6.0
5.5
5.0
4.5
4.0
3.5
3.0
2.5
2.0
1.5
1.0
0.5
ppm

^{1}H-^{13}C HSQC

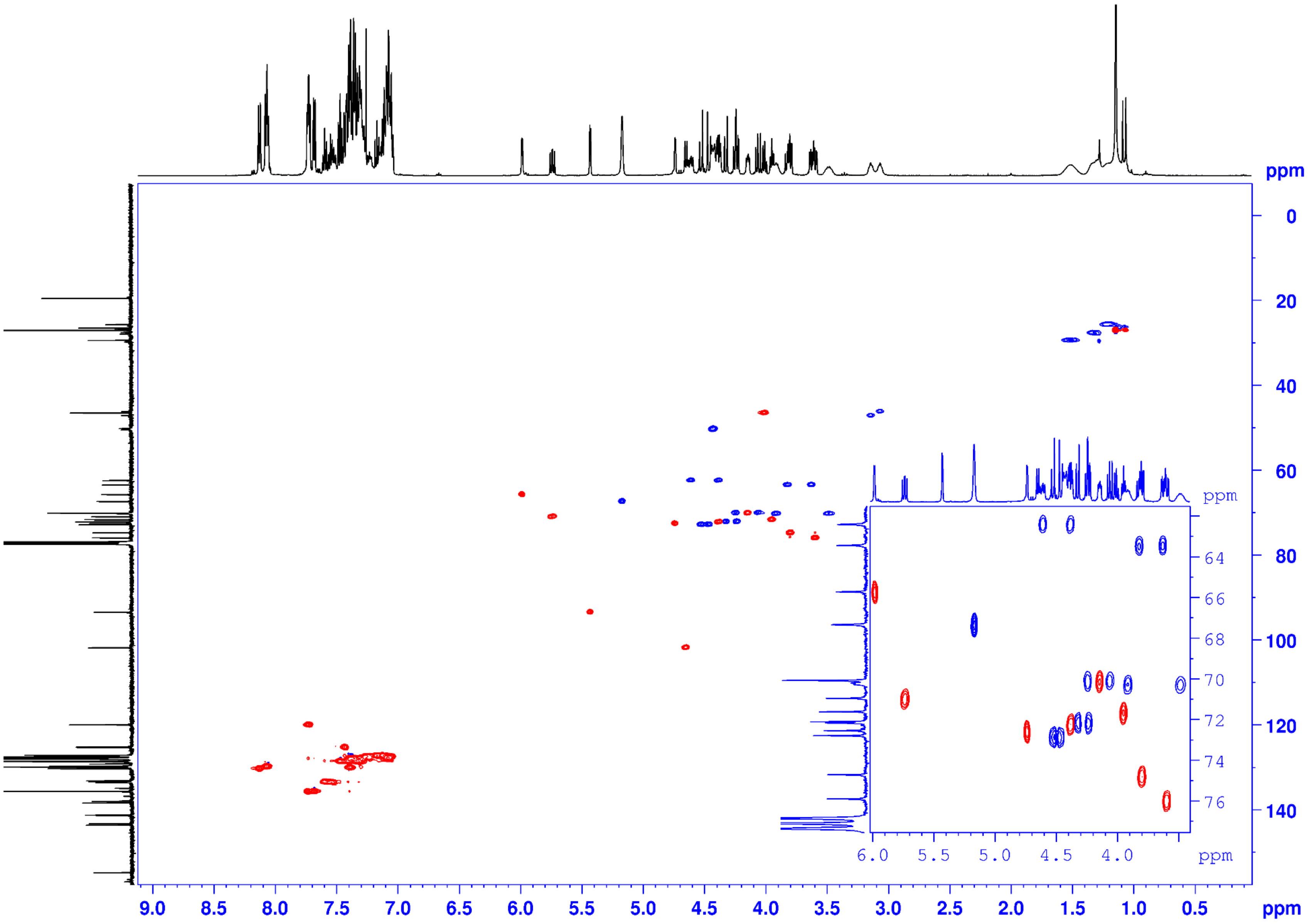

ppm
0
20
40
60
80
100
120
140
9.0
8.5
8.0
7.5
7.0
6.5
6.0
5.5
5.0
4.5
4.0
3.5
3.0
2.5
2.0
1.5
1.0
0.5
ppm
64
66
68
70
72
74
76
6.0
5.5
5.0
4.5
4.0

^1H-^{13}C non-decoupled HSQC

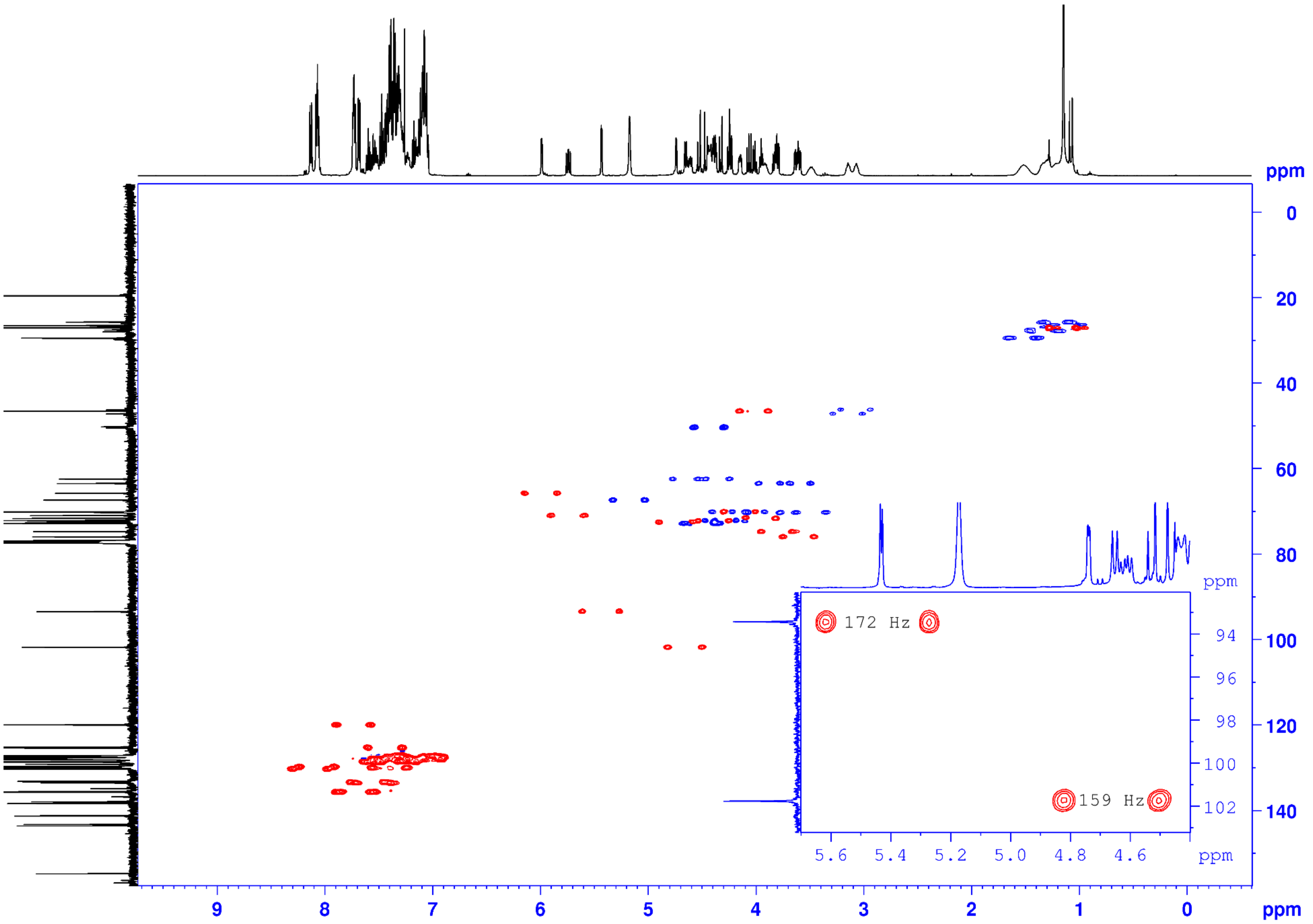

^{1}H-^{13}C HMBC

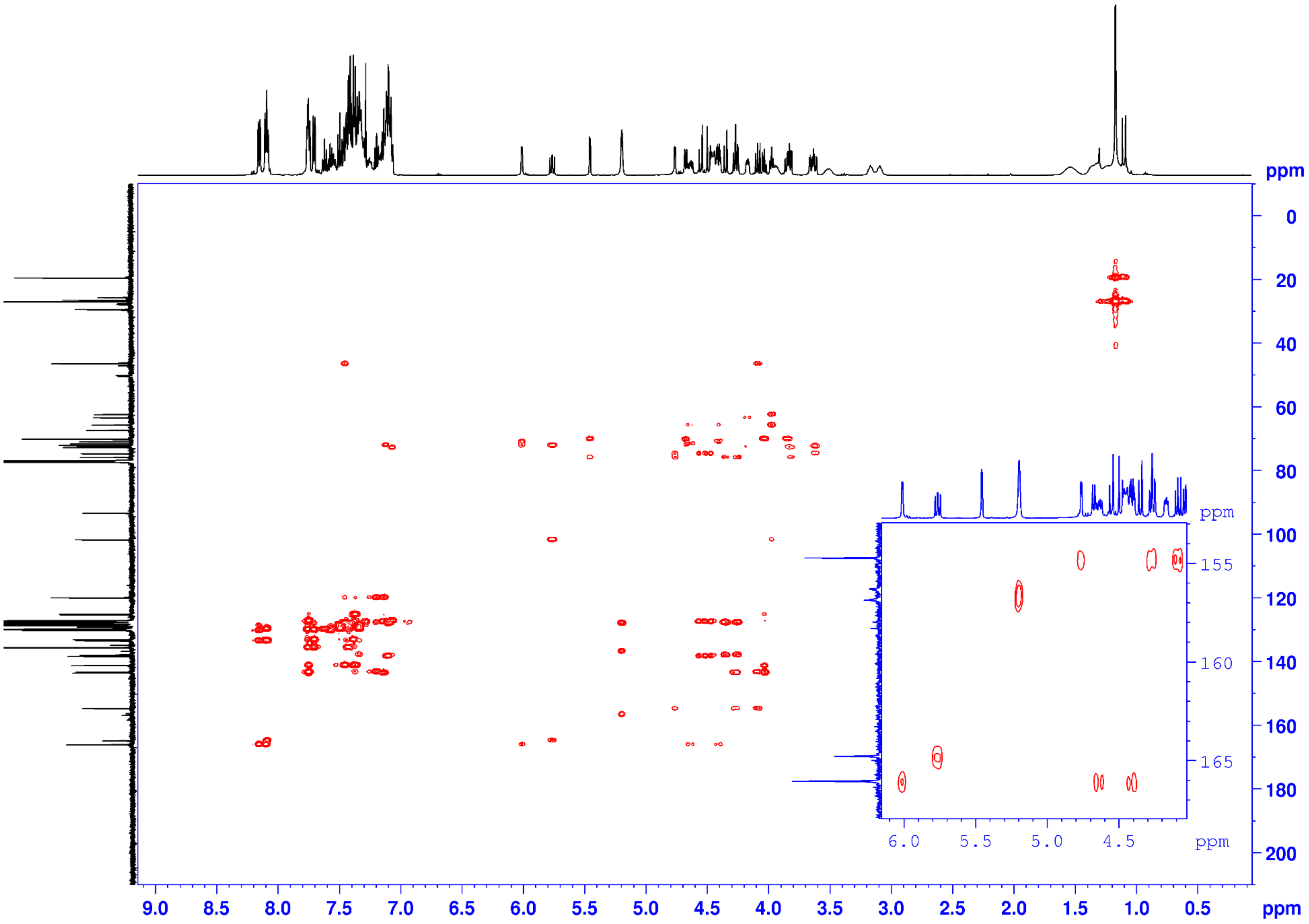

ppm
0
20
40
60
80
100
120
140
160
180
200
9.0
8.5
8.0
7.5
7.0
6.5
6.0
5.5
5.0
4.5
4.0
3.5
3.0
2.5
2.0
1.5
1.0
0.5
ppm
155
160
165
6.0
5.5
5.0
4.5

$^{13}C\{^1H\}$ NMR

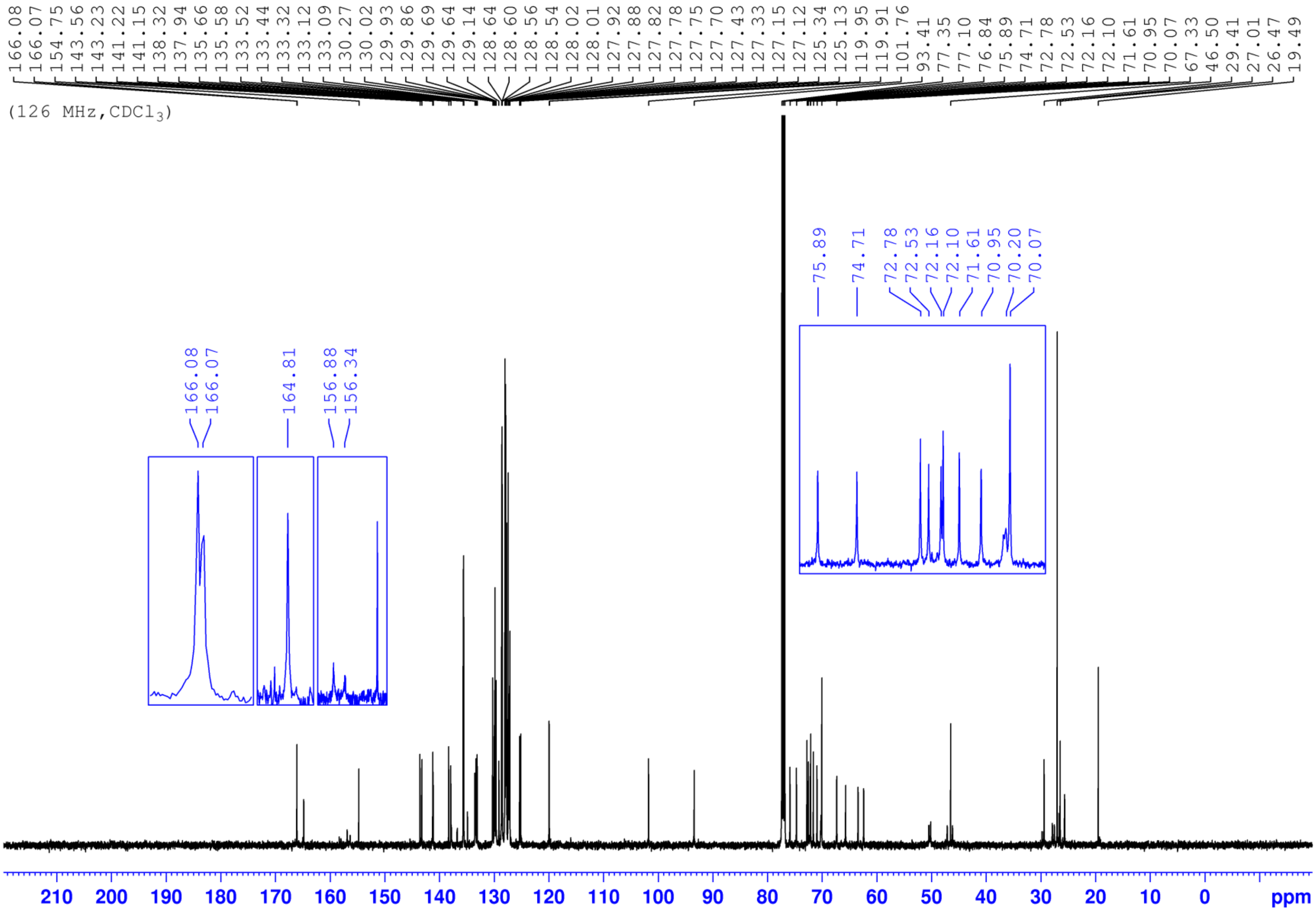

(126 MHz, $CDCl_3$)
166.08
166.07
154.75
143.56
143.23
141.22
141.15
138.32
137.94
135.66
135.58
133.52
133.44
133.32
133.12
133.09
130.27
130.02
129.93
129.86
129.69
129.64
129.14
128.64
128.60
128.56
128.54
128.02
128.01
127.92
127.88
127.82
127.78
127.75
127.70
127.43
127.33
127.15
127.12
125.34
125.13
119.95
119.91
101.76
93.41
77.35
77.10
76.84
75.89
74.71
72.78
72.53
72.16
72.10
71.61
70.95
70.07
67.33
46.50
29.41
27.01
26.47
19.49
166.08
166.07
164.81
156.88
156.34
75.89
74.71
72.78
72.53
72.16
72.10
71.61
70.95
70.20
70.07
210 200 190 180 170 160 150 140 130 120 110 100 90 80 70 60 50 40 30 20 10 0 ppm
